# Dynamic Epigenetic Regulation of Gene Expression during the Life Cycle of Malaria Parasite *Plasmodium falciparum*

**DOI:** 10.1371/journal.ppat.1003170

**Published:** 2013-02-28

**Authors:** Archna P. Gupta, Wai Hoe Chin, Lei Zhu, Sachel Mok, Yen-Hoon Luah, Eng-How Lim, Zbynek Bozdech

**Affiliations:** School of Biological Sciences, Nanyang Technological University, Singapore, Singapore; Weill Medical College of Cornell University, United States of America

## Abstract

Epigenetic mechanisms are emerging as one of the major factors of the dynamics of gene expression in the human malaria parasite, *Plasmodium falciparum*. To elucidate the role of chromatin remodeling in transcriptional regulation associated with the progression of the *P. falciparum* intraerythrocytic development cycle (IDC), we mapped the temporal pattern of chromosomal association with histone H3 and H4 modifications using ChIP-on-chip. Here, we have generated a broad integrative epigenomic map of twelve histone modifications during the *P. falciparum* IDC including H4K5ac, H4K8ac, H4K12ac, H4K16ac, H3K9ac, H3K14ac, H3K56ac, H4K20me1, H4K20me3, H3K4me3, H3K79me3 and H4R3me2. While some modifications were found to be associated with the vast majority of the genome and their occupancy was constant, others showed more specific and highly dynamic distribution. Importantly, eight modifications displaying tight correlations with transcript levels showed differential affinity to distinct genomic regions with H4K8ac occupying predominantly promoter regions while others occurred at the 5′ ends of coding sequences. The promoter occupancy of H4K8ac remained unchanged when ectopically inserted at a different locus, indicating the presence of specific DNA elements that recruit histone modifying enzymes regardless of their broad chromatin environment. In addition, we showed the presence of multivalent domains on the genome carrying more than one histone mark, highlighting the importance of combinatorial effects on transcription. Overall, our work portrays a substantial association between chromosomal locations of various epigenetic markers, transcriptional activity and global stage-specific transitions in the epigenome.

## Author Summary

Malaria is a devastating parasitic disease caused by the protozoan protist *Plasmodium falciparum*. The complex life cycle of *P. falciparum* comprises various morphological and functionally distinct forms and is completed in two different hosts. Various regulatory mechanisms are employed by these parasites to complete their life cycle and survive in human hosts. Epigenetic mechanisms, though not fully explored, have been implicated as one of the key players in gene regulation, morphological differentiation and antigenic variation. Here, we present a comprehensive epigenetic map of 12 histone post-translational modifications during the intraerythrocytic life cycle of *P. falciparum*. We have been able to identify at least eight histone modifications whose dynamic patterns correlate with the transcriptional regulation across the life cycle. In particular, we have shown that a set of euchromatic histone marks work in synergy, creating a dynamic unique histone code that is linked with gene expression during the progression of the *Plasmodium* intraerythrocytic developmental cycle. These findings enhance our knowledge of complex gene regulation and will help to identify novel targets for fighting malaria.

## Introduction

In spite of worldwide efforts, malaria remains one of the most devastating illnesses with an estimated 216 million episodes leading to 655,000 deaths in 2010 [1]. The effectiveness of current treatment strategies is attenuated by increasing resistance of malaria parasites to the available chemotherapeutic drugs. The emergence of artemisinin resistance [2], [3] has motivated researchers to develop alternate control mechanisms by identifying new drug targets. As such, there is a rapid advancement of genomic and epigenomic research to unveil unique molecular mechanisms associated with the growth and development of malaria parasites. *Plasmodium falciparum*, the causative agent of the most severe form of malaria, is also the model organism to study the parasite development due to its ability to be grown *in vitro*. The clinical manifestations of malaria are a result of the parasite development in the red blood cells where it completes its asexual intra-erythrocytic developmental cycle (IDC). Even though transcriptional regulation is important for all developmental stages, the IDC transcriptome revealed a particularly distinct temporal transcriptional regulatory system in *P. falciparum* [4]. Such a broad and dynamic character of transcriptional regulation where each gene is expressed only at a specific time is unprecedented amongst known living organisms and likely represents a unique evolutionary adaptation of the parasite to its host. The presence of plant-like apicomplexan AP2 (Api-AP2) transcription factors [5] and the general paucity of many other types of specific transcription factors [6] further contributes to the unique character of the parasite regulatory machinery. *P. falciparum* also displays several diverse features of its epigenome such as the absence of linker histone H1 [7], the absence of RNA interference machinery [8], the presence of DNA cytosine methyltransferase but apparent absence of DNA methylation [9], [10] and the presence of unusual histone variants with a unique set of modifications [11]. Unlike the majority of higher eukaryotes, *P. falciparum* chromatin is predominantly in a euchromatic state with only a few heterochromatic islands marked by trimethylation of lysine 9 of histone 3 (H3K9me3) [12], [13], [14]. Unlike *Saccharomyces cerevisiae* where K16 acetylation is the dominant modification present at 80% of all H4 molecules [15], K8 and K12 are the favored acetylation sites in *P. falciparum* H4 [11]. Nevertheless, consistent with several studies from yeast and mammalian models showing that regulation of gene expression is mediated by chromatin structure [16], [17], epigenetic states in *P. falciparum* have been shown to affect transcription [18], [19]. In our previous study, we have shown that a potent histone deacetylase inhibitor, apicidin, induces severe alterations in histone modifications as well as gene expression [20]. Recently, it was also shown that epigenetic factors affect clonally variant transcription in *P. falciparum* likely *via* switching between hetero- and euchromatic structures at several genetic loci that mainly encode factors involved in host-parasite interactions [21]. Moreover, there is also evidence suggesting links between the mode of action of artemisinin as well as its resistance mechanism with factors affecting histone modifications [3]. Taken together, these lines of evidence highlight the contribution of the chromatin environment in regulating transcriptional control in *P. falciparum* and stress the need to characterize the overall chromatin landscape as well as its effect on transcriptional regulation during the life cycle.

A total of 44 different post-translational covalent modifications on *P. falciparum* histones including acetylations and methylations have been recently identified [11]. Here, we provide insights into the temporal relationship between twelve of these post-translational modifications and their effect on global transcriptional regulation associated with the complex IDC. The dynamic changes in the transcript pattern during the *P. falciparum* IDC were reflected in the genome wide epigenomic profiles of eight of the studied histone marks. The transcription linked patterns were associated with enrichment of most histone marks predominantly at the start of coding regions while only one modification, acetylation of H4 at lysine 8 (H4K8ac) was found predominantly at the putative promoter regions. Our data also demonstrate co-operative binding of acetylation marks in modulating gene expression across the IDC.

## Results

**Figure 1:**
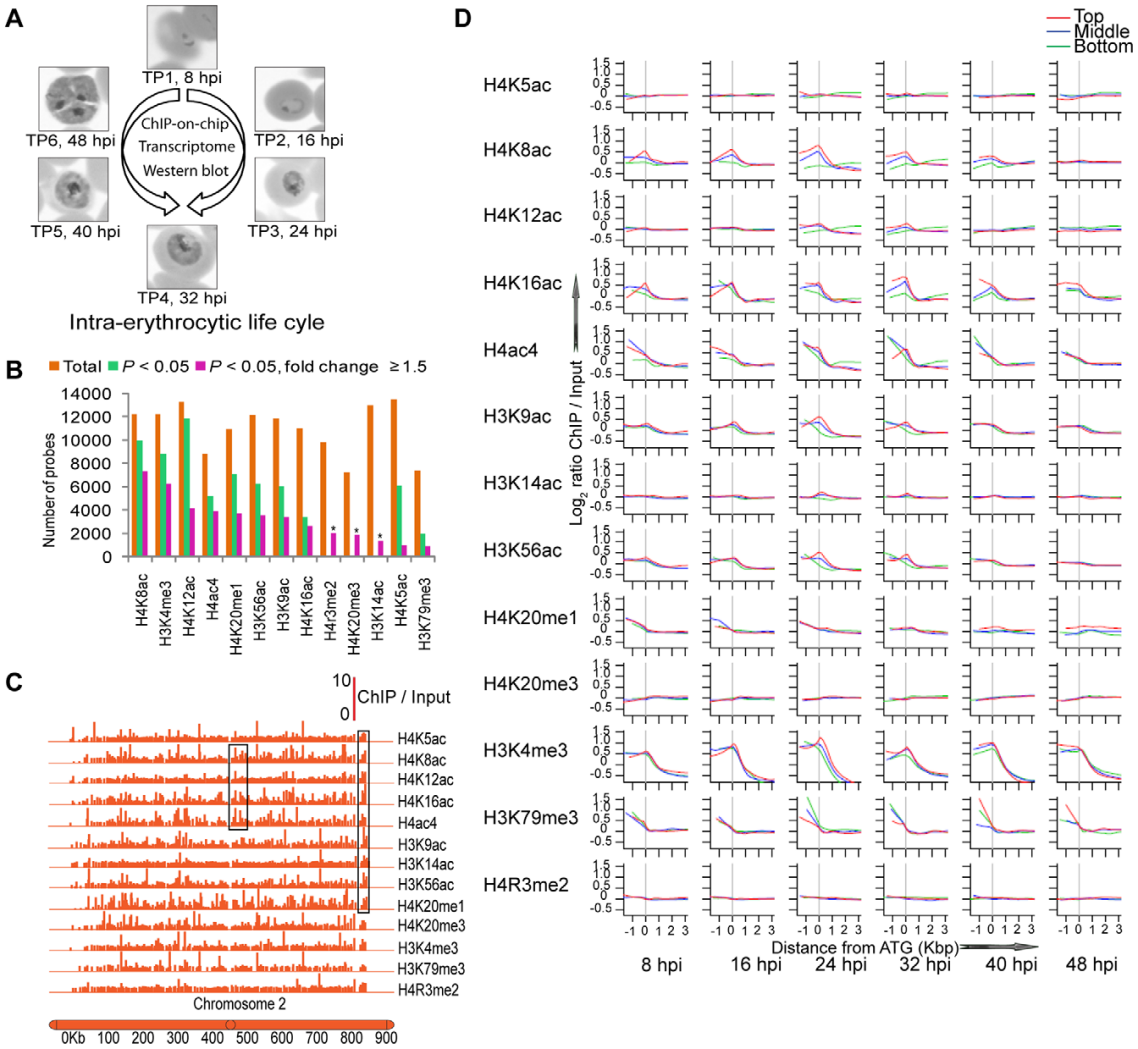
Overview of modified histones occupancy during the *P. falciparum* IDC. (A) *Schematics of the work flow*. *P. falciparum* cells were harvested at 8, 16, 24, 32, 40 and 48 hpi corresponding to time points TP1, TP2, TP3, TP4, TP5 and TP6, respectively, during the 48 h IDC. The same cells were used to carry out all experiments including ChIP-on-chip, transcriptome analysis and western blot assays. (B) *The summary of the occupancy of 13 histone marks in the P. falciparum genome*. The bar graph depicts a total count of microarray probes with positive ChIP signal which corresponds to an association of the genetic loci with the histone marks. For this analysis, only probes with a positive signal for at least 2 consecutive time points (out of 6) were considered. The graph includes the total count of genetic loci (represented by microarray probes) associated with each histone mark (orange bars), only loci with an oscillating occupancy profile (log_2_ ChIP/input) across the IDC defined by *P*<0.05 (green bars), and dynamic occupancy profile across the IDC defined by *P*<0.05 and ≥1.5 fold change (purple bars). The latter are referred to as “dynamic histone marks” and “dynamic occupancy profiles” in the text. Due to the lack of triplicates (scarcity of ChIP material) and consequent inability to calculate *P* values, only the ≥1.5 fold change criteria was applied to the dynamic occupancy profiles of H3K14ac, H4K20me3 and H4R3me2, (marked by an asterisk). The total count of microarray probes is 14,773. (C) *Chromosomal projection of histone occupancy for chromosome 2 at TP2*. Position and height of each vertical line reflect the location on the chromosome and ChIP/input ratio, respectively, of the corresponding probe. Black rectangles highlight apparently similar patterns between different histone marks. (D) *Enrichment of histone modifications along P. falciparum genes*. The graphs represent ChIP signal (see Materials and Methods) plotted along *P falciparum* genes against the probe position relative to translation start codon (ATG) starting −1000 bp (upstream) to +3000 bp (within the ORF) (note that transcriptional start sites remain largely unmapped in *P. falciparum*). For each of the 6 time points across the IDC, the lowess smoothed profiles of the ChIP signal is plotted for three gene groups including 10% of total genes at the top (Top), in the middle (Middle) and at the bottom (Bottom) of the gene list ranked by their mRNA levels at the particular time point. The vertical line in grey indicates the position of the ATG. For these graphs, all histone-mark-occupancy results were used, including both the constitutive and dynamic occupancy profiles.

In order to understand the dynamics of chromatin remodeling and its role in gene expression, we generated an epigenomic map comprising the genome-wide distribution of twelve histone modifications throughout the *P. falciparum* IDC. The main rationale was to recapture the transcriptional cascade of the IDC [4] and to investigate correlations between the occupancy of each histone modification and transcriptional activity along the genome. For this purpose, a large scale culture of highly synchronized cells was grown and samples were collected every 8 h across the 48 h IDC (Figure 1A). It is important to note that the same starting culture was used for the entire set of experiments including chromatin immunoprecipitation combined with microarrays (ChIP-on-chip), transcriptome and western blots to achieve the highest level of comparability within the dataset. For the genome-wide studies, we utilized a *P. falciparum* DNA microarray that contains probes representing both the open reading frames (ORF) and the upstream intergenic regions (IGR) (see Materials and Methods for further details). This microarray was used to carry out ChIP-on-chip to study the chromosomal distributions of thirteen histone marks that included twelve individual histone modifications involving acetylation (ac) of lysine (K) residues on histones H4 and H3, namely H4K5ac, H4K8ac, H4K12ac, H4K16ac, H3K9ac, H3K14ac and H3K56ac; methylation (me) of K or arginine (R) residues, namely H4K20me1, H4K20me3, H3K4me3, H3K79me3 and H4R3me2; and finally one combination of histone modifications H4ac4 (H4 tetra-acetylated at lysines 4, 8, 12 and 16). Apart from H3K9ac and H3K4me3 which have been studied in *P. falciparum* previously [13], [22], other histone marks were selected based on evidence about their roles in transcription from other eukaryotic systems [23], [24], [25], [26], [27], [28], [29]. Using commercially available antibodies directed against modified histones, we confirmed the presence of the thirteen epitopes on *P. falciparum* histones by western blotting directly (Figure S1A) or by peptide competition assays (Figure S1B) and by immuno-fluorescence microscopy (Figure S1C). The designed ChIP-on-chip strategy (Figure 1A) allowed us to generate abundance profiles of histone modification occupancy across the genome and at the same time global mRNA levels during the IDC.

### Distribution of histone modifications across the *P. falciparum* genome

The overview of the occupancy of histone modifications along the *P. falciparum* genome during the IDC revealed that the most abundant histone marks are H4K5ac, H4K12ac, H3K14ac, H4K8ac, H3K4me3, H3K56ac and H3K9ac which associate with >80% of the genome (Figure 1B). This is followed by H4R3me2, H4K20me1 and H4K16ac that associate with 65 to 80%, and finally H4ac4, H4K20me3 and H3K79me3 that associate with less than 60% of the genome (represented by the 14,773 microarray probes). This shows that the individual histone modifications exhibit specific occupancy patterns that reflect their distinct roles in the parasite chromatin structure and function. The visual display of the chromosomal distribution of histone mark occupancy further supports this observation showing distinct patterns of histone marks across the chromosomes but also the existence of some genetic loci marked by more than one modification (Figure 1C, black boxes).

Next we determined the enrichment of histone marks represented as log_2_ ChIP/input ratios with respect to their position within the *Plasmodium* genes. This was done separately for genes with different levels of expression: top, middle and bottom 10% in the rank of their mRNA levels in each IDC time point (Figure 1D). Overall we could divide the studied histone modifications into two groups, (i) those with a biased distribution in the IGRs and/or 5′ termini of the ORFs and (ii) those with no preference in their occupancy within the gene structures. The first group comprises four H4 (K8ac, K16ac, ac4 and K20me1) and four H3 (K9ac, K56ac, K4me3 and K79me3) modifications with higher enrichment at IGRs and gradual decrease towards the 3′ end of the genes. The extreme example is H3K4me3 with sharp IGR occupancy, which is consistent with previous suggestion that the primary role of this modification is to demarcate the non-coding regions in between *P. falciparum* genes [22]. Interestingly, while some modifications such as H3K4me3 and H3K79me3 retained this IGR preference throughout the IDC, others showed stage specific changes in their positional enrichment. These include H4K8ac and H3K56ac that were found at the IGRs predominantly in trophozoites and early schizonts but show essentially no gene position preference in the extremes of the IDC, early rings and late schizonts. For most of the histone marks, there were only small, likely insignificant, differences in their occupancy between genes with high, medium or low levels of expression. The exceptions are, H4K8ac, H4K16ac, H3K9ac and H3K56ac that exhibited somewhat higher IGR enrichment for genes with high mRNA levels (Figure 1D). In addition there was a slight tendency for all H4 acetylations to increase their enrichment towards the 3′ end of genes with low levels of mRNA during 16 to 30 hours post invasion (hpi). In summary the differential occupancy of histone marks within genes suggest their distinct roles as chromatin remodeling factors that may be linked with gene expression during the *P. falciparum* IDC.

### Dynamics of histone modifications across the IDC

The most significant observation made by these studies is the broad and dramatic dynamics of the occupancy of histone modifications across the IDC. Essentially all thirteen histone marks show some degree of variable occupancy at least for a small portion of the genetic loci with which they associate. For the purpose of this study, we define the occupancy variability by two criteria: (1) The statistical significance of the measured change in occupancy across the experimental time points with respect to experimental replicas (*P*<0.05), and (2) in addition to statistical significance (*P*<0.05), a minimum 1.5 fold change in the occupancy profiles across the IDC (Figure 1B, Table S1). Below, we refer to these as “dynamic histone marks” and “dynamic occupancy profiles”, respectively. Quantitative real time PCR was carried out to validate the dynamic occupancy profiles for three modifications in three genes (Figure S2A). We also wished to evaluate the performance of the microarray probes representing the IGRs in comparison to the ORFs. The signal-intensity/signal-ratio distributions between the sets of ORF and IGR probes show essentially identical profiles with no measurement bias towards any ratio/intensity interval (Figure S2B). This supports the fidelity of the applied microarray technology and ensures that the dynamic range and thus ChIP-on-chip measurements of histone occupancy are directly comparable between IGRs and ORFs. The two most dynamic histone modifications were found to be H4K8ac and H3K4me3 that showed significant changes of ChIP-on-chip signal at more than 50% of the loci with which these histone marks associate. Moreover H3K56ac, H3K9ac, H4ac4, H4K16ac, H4K12ac, H4K20me1 and H4K20me3 exhibited dynamic occupancy profiles at more than 25% of their loci. In contrast, H4R3me2, H3K14ac, H3K79me3 and H4K5ac represented the other side of the spectrum with a constitutive pattern of occupancy at the majority of the loci with only 20% or less showing variation across the IDC. The Chi-square test revealed a preference for localization of the dynamic histone marks, with H4K16ac, H4ac4, H4K8ac, H4K12ac, H3K56ac, H4K20me1, H4K20me3 and H3K4me3 showing overrepresentation in the ORFs, whereas H4R3me2 showed overrepresentation in the IGRs (Figure S3). The ORF preference occupancy of the dynamic histone marks is surprising as it is in contrast to their overall (constitutive and dynamic) occupancy in IGRs (Figure 1D). This may suggest that while the constant occupancy of these histone marks at the IGRs may function as general demarcation elements, it is the nucleosomes linked with the 5′ termini of the ORF regions that play a dynamic role in the chromatin remodeling and possibly transcriptional regulation during the IDC (see below).

**Figure 2:**
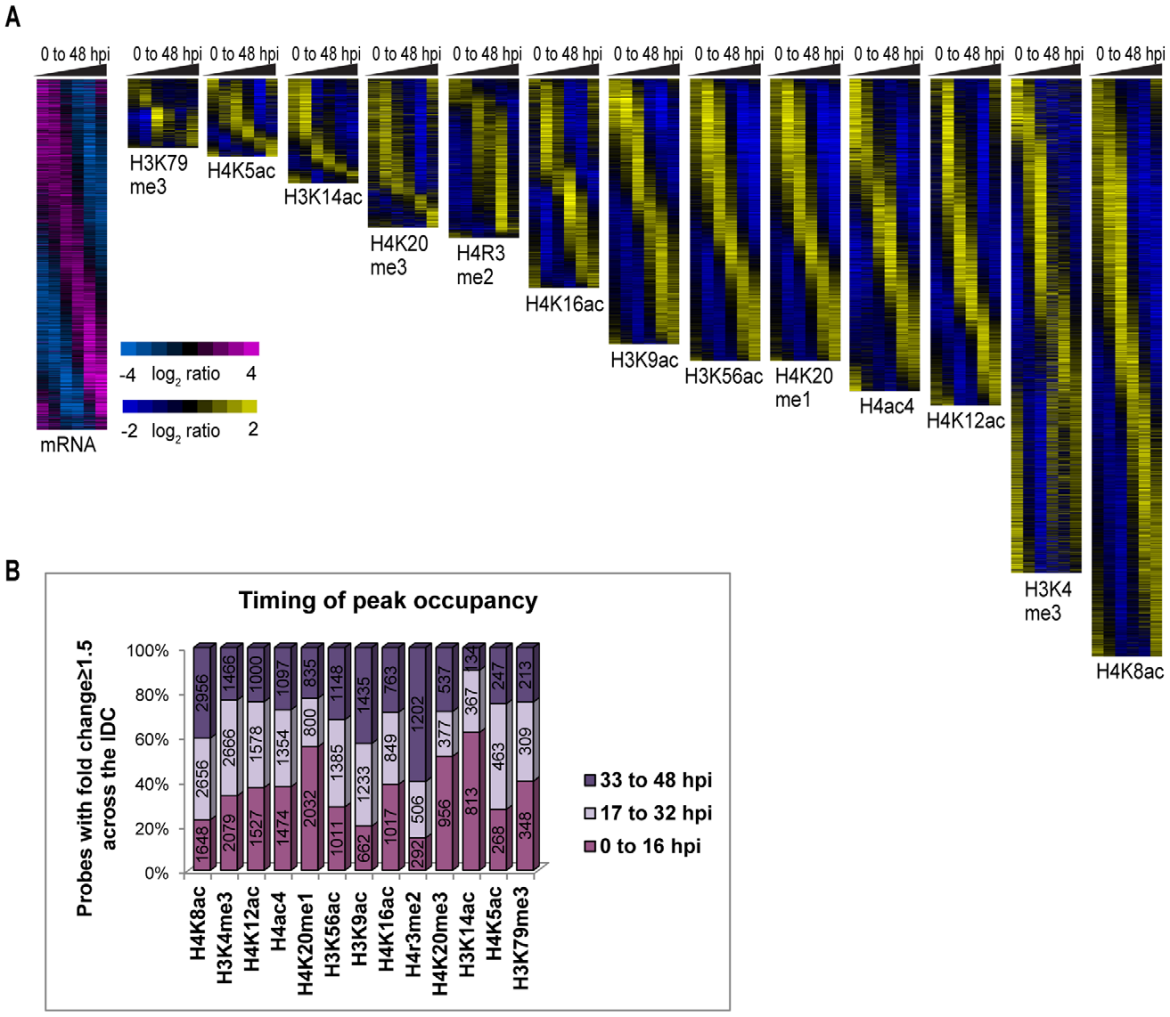
Dynamics of histone occupancy across the *P. falciparum* IDC. (A) *mRNA transcriptome and dynamic occupancy profiles of 13 histone marks across the IDC*. The phaseograms depict the cascades of the dynamic occupancy profiles (*P*<0.05 and fold change ≥1.5 across the IDC, see Figure 1B) for the 13 histone marks. The yellow/blue color scale represents lowess smoothed profiles calculated from centered curves of relative occupancy ratios measured by ChIP/input (log_2_) for the 6 time points (TP1-6 representing 0 to 48 hpi). The mRNA transcriptome presented on the left was assembled similarly. The gene/probe order in each phaseogram was determined independently using Fast Fourier transformation (see Materials and Methods). (B) *Distribution of the peak occupancy of the 13 histone marks along the IDC*. The bar chart shows the maximum occupancy of histone marks at 3 life cycle stages for the dynamic profiles included in the phaseograms (panel A). The percentage represented (by bar size) as well as the probe number (number within the bar) depicts the distribution of the corresponding occupancy profiles that peak at TP1 or TP2 (0–16 hpi), TP3 or TP4 (17–32 hpi) and TP5 or TP6 (33–48 hpi). In our culturing set up, these time points correspond to the three main IDC developmental stages, ring, trophozoite and schizont, respectively.

Similar to mRNA, the occupancy patterns of histone modifications exhibited single peak profiles with each locus being marked once at a specific time during the IDC (Figure 2A). Investigating the time of peak occupancy, we observed no general trends, but instead each histone mark exhibited a distinct pattern (Figure 2B). In particular, more than 50% of genetic loci are associated with the dynamic occupancy of H4K20me1, H4K20me3 and H3K14ac which reach maximum levels between 0 and 16 hpi, whereas 40% of loci associated with the dynamic occupancy of H3K4me3 and H4K5ac peaked between 17 and 32 hpi. In contrast, H4K8ac, H3K9ac and H4R3me2 showed maximum occupancy (more than 40%) at the late schizont stage. Other histone marks were evenly distributed amongst the three stages. This global variation in the occupancy of histone marks again suggests their distinct role in chromatin remodeling with multiple events during the IDC affecting their overall distribution across the IDC.

### Association of histone mark occupancy with transcription during the IDC

**Figure 3:**
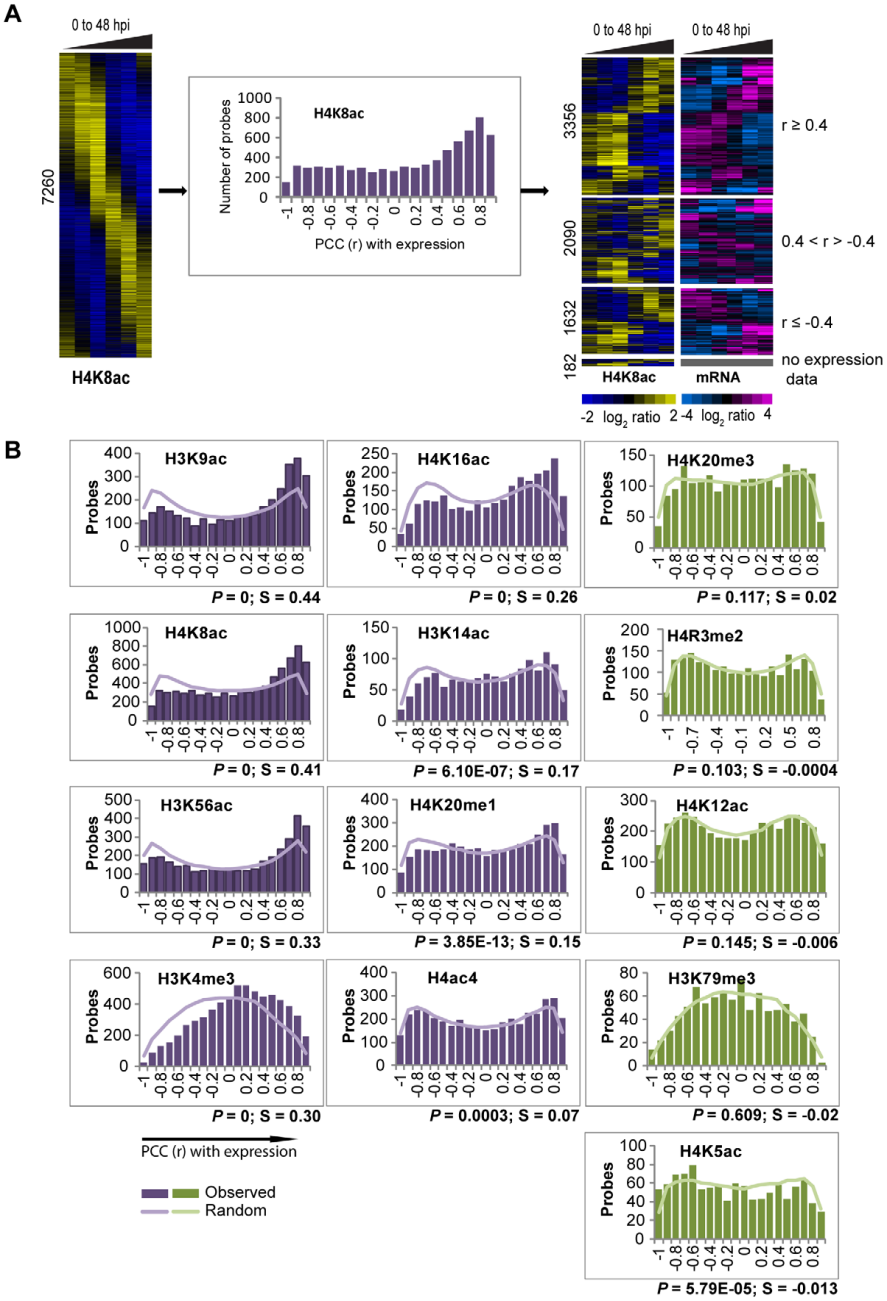
Association of histone mark occupancy with transcription. (A) *Correlation of H4K8ac occupancy with transcription*. The dynamic occupancy profiles for H4K8ac (leftmost panel) was correlated to the corresponding mRNA profiles using Pearson Correlation Coefficient (PCC, also r). The PCC was calculated between each dynamic H4K8ac occupancy profile and mRNA abundance profile of the corresponding gene. The PCC distribution was plotted along bins ranging from −1 to +1 (x-axis) as the number of probes in each bin (bar graph in the middle). A total of 3,356 probes showed r (PCC)≥0.4 which represents a good correlation in the IDC timing (rightmost panels). In addition 2090 probes showed no correlation (r between 0.4 and −0.4) and 1632 probes showed a negative correlation (r≤−0.4) with expression. Out of 7,260 loci linked with the dynamic H4K8ac occupancy profiles, we did not obtain expression data for 182 probes. The scale bar refers to log_2_ ratios of mean-centered profiles across the IDC. (B) *Correlations of the 13 histone-mark-occupancy profiles with transcription*. Similar to H4K8ac (panel A), the distribution of PCC (r) correlations between the dynamic occupancy and steady state mRNA profiles were calculated for all 13 studied histone marks. Similarly, the data were arranged into bins of PCC ranging from −1 to +1 (x-axis) and plotted against the number of probes in each bin for actual distribution (bar graphs) and randomized distribution (line graphs). The statistical significance of the observed distribution (*P*, tested against the randomized distributions) and the degree of skewness (S, positive values towards positive PCCs) of the PCC distribution is presented at the right bottom of each graph (see Materials and Methods). Eight histone marks exhibit statistically significant correlation with transcript levels (purple graphs) while 5 marks show a neutral relationship with transcript levels (green bars).

The dynamic character of the histone modifications and its similarity to the mRNA abundance profiles during the IDC suggests their possible role in transcription. To investigate this, we evaluated the correlations between the occupancy profiles of the dynamic histone marks and steady state mRNA levels of the corresponding genes. Here we hypothesize that a synchrony between the histone marks and mRNA substantiates a link between their deposition at a particular gene and transcriptional activity. Hence we calculated Pearson Correlation Coefficients (PCC or r) between mean-centered profiles of the dynamic histone modification occupancy and the corresponding mRNA. For example, 49% (out of 7260) of the H4K8ac dynamic occupancy profiles showed positive correlations (r≥0.4) with transcription while 22.8 and 22.5% showed no or negative correlation, respectively (Figure 3A). The overall skew of the H4K8ac correlation values to the positive side suggests that this histone mark plays a role in transcriptional induction. Using the Kolmogorov-Smirnov test (KS test) against randomized data, we were able to evaluate the significance of the occupancy profile correlations with mRNA for all histone marks (Figure 3B). In addition, we utilized the degree of skewness (S) to identify all histone marks that are positively correlated with transcription. With *P*<0.0005 and S>0.05, we identified eight histone marks including H4K8ac, H4K16ac, H4ac4, H3K56ac, H3K9ac, H3K14ac, H3K4me3 and H4K20me1 that showed positive correlations with transcription and thus we refer to these as “transcription-linked” histone marks. For these eight histone marks, the percentage of probes that show a positive correlation with expression (r≥0.4) varied from 35 to 48% (Figure S4A). The genes associated with these transcription-linked histone marks show no bias to any particular developmental stage but instead are more or less evenly distributed amongst all stages of the IDC (Figure S4B). Interestingly, the histone marks which followed transcription are also amongst the most dynamic, with at least 25% of the loci changing their occupancy across the IDC (see Figure 1B). There was a statistically significant link between one histone modification (H4K5ac) and transcription that is skewed towards a negative correlation. Although H4K5ac is predominantly a constitutive histone mark, this observation opens the possibility that this otherwise euchromatic mark may play a role in transcriptional repression in a small group of genes. Four histone modifications (H4K20me3, H4R3me2, H4K12ac and H3K79me3) show essentially no association with transcription during the IDC (Figure 3B). One interesting example is H4K20me3 which was shown to be present at both heterochromatic and euchromatic domains of the *P. falciparum* genome [12]. In the future, it will be interesting to study their potential roles in chromatin structure and remodeling which may be distinct from transcription.

**Figure 4:**
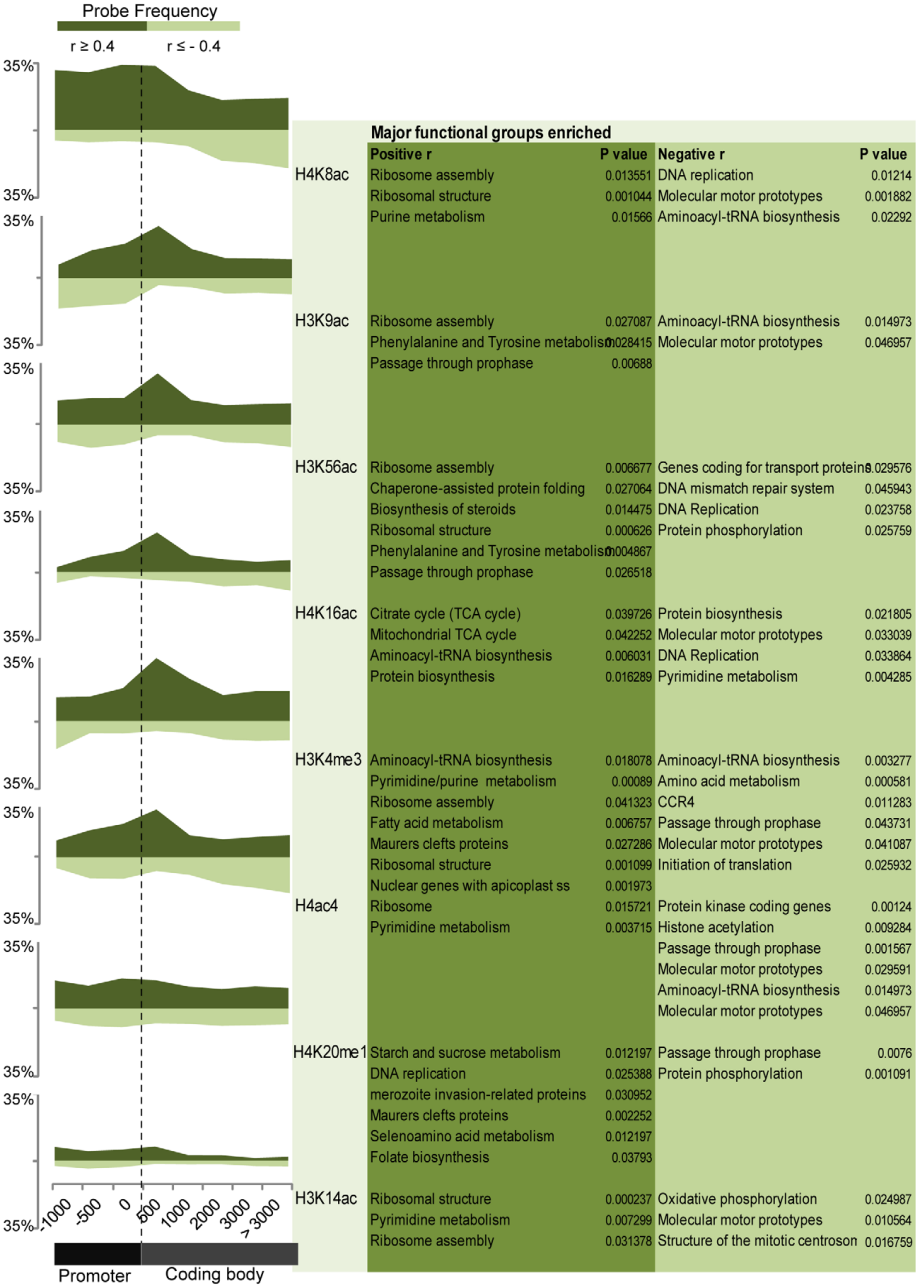
Gene positioning and biological significance of the transcription-linked histone marks. The plots on the left represent the frequency (percentage) of transcription-linked histone marks that are characterized by their dynamic occupancy profiles with positive (r≥0.4) or negative (r≤−0.4) correlation with expression. The frequency of these transcription-linked profiles is plotted along an average gene corresponding to the genetic locus/probe positioning within the gene. In particular, the data was arranged into 500 bp bins ranging from 1500 bp upstream of the ATG start codon (“Promoter”) to +3000 bp into the gene (“Coding body”). The frequency (y-axis) is expressed as the percentage of transcription-linked profiles versus the total number of occupancy profiles (both dynamic and constitutive) for either positive (dark green, above the x-axis) or negative (light green, below the x-axis). The vertical dotted line marks the position of the ATG start codon. Functional enrichment analyses identified over-represented pathways (*P*<0.05) as compared to their respective frequency in the genome for genes associated with each transcription-linked modification (right).

Next, we were interested in the biological significance of the transcription-linked histone modifications. We analyzed the distribution of mRNA correlating histone-marked loci with respect to their position in the gene and subsequently investigated the functional involvement of these genes (Figure 4). Interestingly only one dynamic, transcription-linked histone mark (H4K8ac) showed a strong presence for the IGRs and/or 5′ untranslated regions (5′UTR). This is in good agreement with its overall distribution in the genome (see Figure 1D). All other modifications associated with transcription, including H3K9ac, H3K4me3, H3K56ac, H3K4me3, H4K16ac and H4ac4, appear to accumulate mainly at the 5′ ends of the ORFs. Transcription-linked occupancy of H4K20me1 and H3K14ac showed no positional preference. We did not observe any positional bias for probes negatively correlated with expression (r≤−0.4).

To assess the functional relationship of transcription-linked histone marks, we identified significantly represented functional groups (*P*<0.05) based on Gene Ontology (GO), Kyoto Encyclopedia of Genes and Genomes (KEGG) and Malaria Parasite Metabolic Pathway (MPMP) (Figure 4). The transcription-linked histone marks were mainly enriched in genes associated with growth (ribosomal structure and assembly), metabolism (fatty acid metabolism, nucleotide biosynthesis) and host-parasite interactions (Maurer's cleft, invasion). A small subset of genes associated with H4K8ac, H4K16ac and H4ac4 which negatively correlated with expression belonged to molecular motors or genes coding for kinetochore and centrosome organization. Interestingly, transcription profiles of genes involved in DNA replication correlated positively with H4K20me1 but negatively with H4K8ac and H4K16ac occupancy profiles. This implies that the DNA replication genes may be deacetylated and methylated at the onset of DNA replication. These observations are consistent with previous studies in other eukaryotic systems that have shown both co-existence [30] as well as competition [31] between H4K20me1 and H4K16ac, and imply the presence of a similar histone code in *P. falciparum*. In summary, these results clearly demonstrate the association of transient histone modification states with transcriptional activation where at least eight histone marks either individually or in various combinatorial patterns, have the potential to modulate gene expression during the *P. falciparum* IDC.

### Recruitment of H4K8ac to the promoter regions

**Figure 5:**
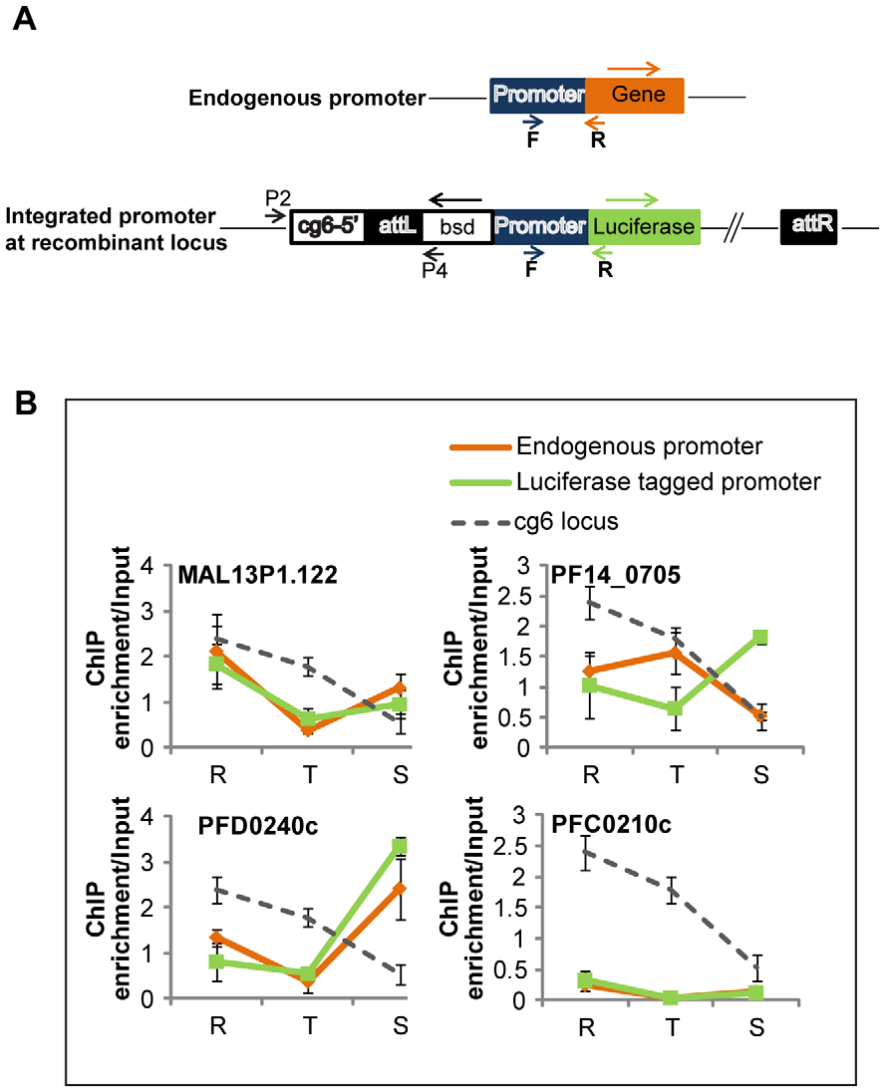
Histone modification patterns on ectopically integrated promoters. (A) *Cloning strategy for ectopic integration of promoter regions*. Four promoter regions (1.5–2 kb upstream of the ATG) corresponding to upstream regions of MAL13P1.122, PF14_0705, PFD0240c and PFC0210c were cloned upstream of the luciferase reporter gene pLN-luc (see Materials and Methods). *P. falciparum* strain Dd2^attB^ was transfected with the above vectors to achieve integration at the *cg6* locus and the transgenic cell lines were selected on blasticidin. Primer pair P2/P4 was used to confirm integration (data not shown). (B) *H4K8ac occupancy at the four ectopically integrated promoter regions*. The graphs represent real time PCR results carried out on H4K8ac-immunoprecipitated DNA from rings (R), trophozoites (T) and schizonts (S). In order to distinguish between the endogenous and luciferase tagged promoter, specific primers were designed to amplify regions spanning the 3′ end of the promoter and either the start of the endogenous gene or the start of the luciferase gene. The positions of forward (F) and reverse (R) primers are shown in panel A. Grey lines refer to the ChIP enrichment of the native *cg6* locus in the untransfected parasites. Orange and green lines represent the ChIP enrichment of native promoters and integrated promoters, respectively, in the transfectants. The error bars give the standard deviation from triplicate experiments.

Presently, very little is known about the mechanisms of chromatin remodeling in *Plasmodium* parasites. Given the highly dynamic character of histone modifications observed by this as well as previous studies [13], [20], [22], these mechanisms are likely to be highly evolutionarily diverse. Transcription factors bound to promoter and upstream regions are known to recruit chromatin modifiers in other species. We therefore investigated the role of promoter regions in the recruitment of H4K8ac that we found mainly in the upstream regions of active genes. In particular, we wanted to assess the presence of any DNA elements which help to establish histone marks in promoter regions. Four promoters (1.5–2 Kb upstream of the ATG) marked by H4K8ac were selected and cloned into luciferase reporter constructs including upstream regions of ring-specific (MAL13P1.122), trophozoite-specific (PF14_0705), schizont-specific (PFD0240c) and sporozoite-specific (PFC0210c) genes. Here, we made use of the strain Dd2^attB^ [32] in which transgenes can be integrated at the *cg6* locus (Figure 5A). We found that the occupancy profile of H4K8ac was recapitulated on three of the four ectopic promoters (Figure 5B). These profiles override an existing profile of the endogenous *cg6* gene (dashed line) that is normally characterized by high levels in rings and gradually declines through trophozoites and schizonts. The luciferase activity profiles were also similar to the acetylation patterns of all transfected promoters (data not shown). For one of the promoters (PF14_0705), there was an incomplete “carry-over” of the H4K8ac occupancy profile that was matched only in the ring stage. This may be due to unknown factors like insufficient promoter length. Overall our data suggest that the promoter regions of *P. falciparum* genes carry DNA regulatory elements that establish H4K8ac independently of their endogenous chromatin environment.

### Combinatorial landscape of co-existing marks

**Figure 6:**
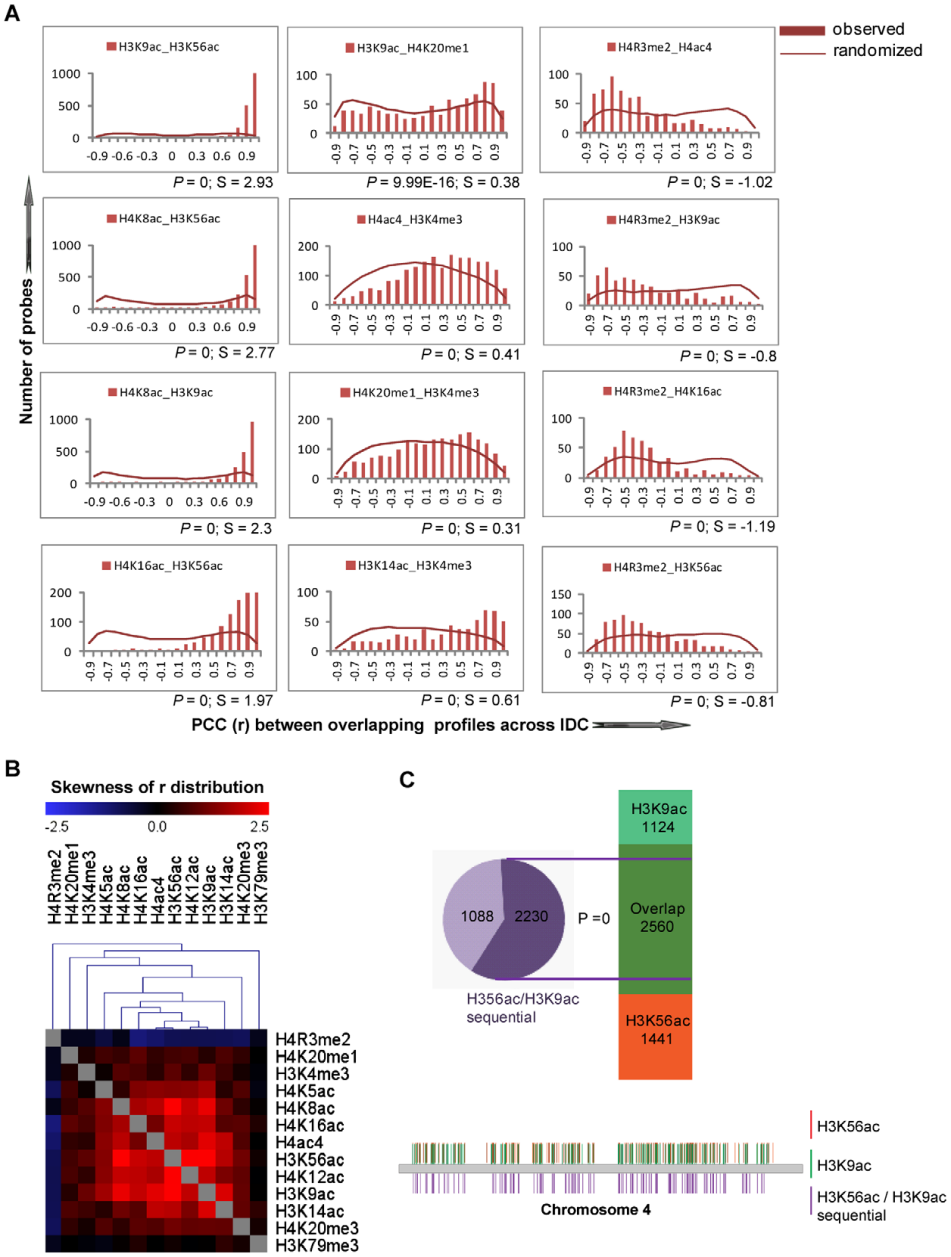
Combinatorial histone modifications. (A) *PCC-based correlation of the dynamic histone occupancy profiles*. The bar charts show examples of pair wise correlation distributions between the dynamic profiles of the individual histone marks calculated analogously to the correlations with steady state mRNA levels (see Figure 3). Line graphs give the randomized distribution of PCC. *P* value (P) and skewness (S) are shown below each graph. (B) *Hierarchical clustering of histone mark associations*. The heat maps represent hierarchical clustering of the skewness (S) values of the PCC distributions for every marked histone pair. High positive skew (red) and low negative skew (blue) indicate positive and negative correlations with the marked histone pairs, respectively. (C) *Sequential ChIP-on-chip for H3K9ac and H3K56ac*. The sequential ChIP-on-chip was carried out with ring stage parasites using anti-H3K56ac antibody followed by anti-H3K9ac antibody. Immunoprecipitation was also done using anti-H3K56ac antibody or anti-H3K9ac antibody separately. Data was averaged from 2 separate experiments and probes with ChIP/input signal ratio above 1 for bead control were subtracted for further analysis. The chart (above) shows the probes overlapping between the sequential ChIP and probes shared in common between both histone marks when used individually (ChIP/input >1). Chromosomal distribution (below) of H3K56ac (orange), H3K9ac (green) and sequential (purple) ChIP enrichment of probes as seen at chromosome 4. Each vertical line depicts the position of a probe on the chromosome.

The abundance of dynamic temporal regulation of individual histone marks suggests the existence of combinatorial patterns forming a putative “dynamic histone code”. To investigate this possibility, we carried out pair-wise analysis of occupancy profiles with all thirteen histone marks. To this end, we evaluated the concordance of the occupancy profiles using PCC distributions and subsequently the skewness and KS-test *P* value as described above (Table S2). Figure 6A shows examples of highly positive (S>1), moderately positive (S between 1 and 0) and highly negative PCC distribution (S<0) between the histone marks. Overall our analysis revealed that most of the overlapping marks (present at the same loci) exhibited a high level of correlation between their occupancy profiles. Here it is important to note that the mean size of ChIP DNA product generated by our protocol is 500 bp which corresponds to approximately three nucleosomes positioned in the vicinity of the genetic locus represented by a microarray probe. Hence the correlations in the occupancy profiles represent either a combinatorial histone modification at the same nucleosome or co-occurrence of these at directly adjacent nucleosomes. High correlations of the occupancy profiles are particularly evident for acetylations that exhibited positive correlations at essentially all overlapping loci (Figure 6B). This suggests that, each nucleosome predominantly undergoes only one set of modifications during the IDC, presumably for one purpose (such as transcriptional regulation). This situation contrasted with the lysine methylation profiles that showed loose or no correlations with each other or with acetylations. Interestingly, H4R3me2 exhibited a strong negative correlation with most of the other marks. This methylation is thought to be mediated by PfPRMT1 and might be playing a similar role to H3R2me2a (asymmetrical histone H3 arginine 2 dimethylation) which has been shown to have a mutually exclusive pattern with H3K4me3 in budding yeast [33].

Acetylation clusters (Figure 6B) comprising H4K8ac, H4K16ac, H4ac4, H4K12ac, H3K9ac and H3K56ac were found to be enriched for specific functional groups which define biologically related genes. Hence, the histones within the nucleosomes associated with the genes within these groups appear to be acetylated at most of the (studied) lysine residues during active transcription. Comparing the associations of all thirteen modifications with mRNA, the most represented gene families associated with ribosome structure, protein biosynthesis, tRNA modifications, Maurer's cleft and invasion displayed positive correlations with the acetylation marks (Figure S5). One of the striking examples involves genes of the early transcribed membrane proteins (ETRAMPs) whose mRNA abundance displayed strong correlations to virtually all acetylated histone marks. Interestingly, genes coding for histones themselves showed an anti-correlation between expression and the histone mark occupancy profiles. However, the majority of the gene groups associated with IDC functionalities was mostly in strong positive correlation with the majority of these euchromatic histone marks. An interesting example involves the group of Api-AP2 transcription factors whose mRNA levels are mostly in positive correlation with all euchromatic marks with the exception of H4K16ac which appears to correlate negatively. Hierarchical clustering of members of each group showed that while the majority of genes in the basic cellular and biochemical pathways expressed during the IDC exhibit good correlation between histone marks and transcription, each of the functional groups contains at least a small subset of genes whose mRNA levels are negatively correlated with at least some histone marks. It will be interesting to investigate the implication of these histone marks on transcriptional regulation and thus the functional involvement of these outlying members.

In order to validate the co-occupancy of more than one histone mark on a genomic region, we carried out sequential ChIP at ring stage parasites to identify bivalent domains having two histone marks: H3K56ac and H3K9ac (Figure 6C, Table S3). From individual ChIP results, a total of 2,560 probes common to H3K56ac and H3K9ac yielded ChIP-to-input ratios above 1. Out of 3,318 probes recognized by sequential ChIP, 2,230 (67%, *P* = 0) overlapped with probes common to the individual ChIPs. As an example, the distribution of ChIP signals on chromosome 4 defined common areas of enrichment between chromatin immunoprecipitated with either H3K56ac or H3K9ac independently, or H3K56ac followed by H3K9ac sequentially (Figure 6C).

## Discussion

The dynamic morphology during the life cycle development is believed to be an evolutionarily unique feature of *Plasmodium* as well as other eukaryotic parasitic organisms and likely reflects their adaptation to a specific host environment. It is clear now that these morphological switches are underlined by broad transcriptional shifts that, in the case of *Plasmodium* species, affect essentially the entire genome [4], [34], [35]. There is mounting evidence that epigenetic mechanisms contribute to the regulation of gene expression across the IDC by maintaining hetero- and euchromatin domains within the *Plasmodium* genome [12], [14], [22], [36], [37]. Although most previous studies focused on heterochromatic domains, here we provide a comprehensive epigenetic atlas of thirteen predominantly euchromatic histone marks and provide evidence suggestive of their unique functions during the parasite's asexual blood stage development. Our data suggests that at least 8 modifications are linked with the transcriptional activity of up to 76% of *P. falciparum* genes.

The dynamic nature of histone modifications and their link with transcription during the *P. falciparum* IDC has been previously suggested. Using ChIP-on-chip, it was shown that H3K9ac and H3K4me3 exhibit highly dynamic patterns of chromosomal distribution between rings and schizonts, and that their occupancy correlates positively with transcription [13]. In the follow-up study by the same group, ChIP-seq results showed that both of these histone modifications associate mainly with promoter regions but only H3K9ac is correlated with transcription while H3K4me3 appears uncoupled from transcription [22]. In agreement with these two reports, our results show a strong accumulation of both H3K9ac and H3K4me3 in the IGRs (Figure 1D). However, for both of these modifications, it is mainly their association with the 5′ end of coding regions that correlates with expression (∼22% and ∼28% of H3K9ac and H3K4me3 modifications associated with the 5′ ends of ORFs are positively correlated with transcript levels, respectively) (Figure 4). On the other hand, only ∼12% and ∼10% of H3K9ac and H3K4me3 within IGRs correlate with transcript levels, respectively. This further highlights previous findings showing that H3K9ac and its association with IGRs plays a greater role in transcription compared to H3K4me3. In addition to these two modifications, we demonstrated that at least six other euchromatic marks of H3 (K14ac and K56ac), and H4 (K8ac, K16ac, K20me1 and ac4) play roles in transcriptional regulation during the *P. falciparum* IDC. With the exception of H4K8ac which shows maximum enrichment at IGRs and/or 5′UTRs, the five others show maximum enrichment at the 5′ ends of ORFs of transcriptionally active genes. This is surprising as in other eukaryotes such as *Toxoplasma gondii*, *Caenorhabditis elegans* and *Saccharomyces cerevisiae*, most acetylations and methylations that are positively correlated with expression typically localize at the promoters and transcriptional start sites [24], [38]. From this perspective, the chromatin structure of *Plasmodium* resembles that of plants where most of the euchromatic histone marks accumulate within the start of ORFs as compared to IGRs [26], [27]. In the future, it will be interesting to study these features of epigenetic regulation, possibly in combination with another plant-like phenomenon in *Plasmodium*, the Api-AP2 transcription factors [5]. On the other hand, the striking shift in the accumulation of H4K8ac towards upstream regions is indicative of a distinct role in transcription that is more related to that of other eukaryotes such as mammals [39].

In eukaryotic organisms, distinct chromatin states are defined by multiple histone modifications acting sequentially and/or in combination, a phenomenon referred to as the histone code. Although a full understanding of the histone code is pending, distinct patterns of modified histones define groups of biologically related genes [29], [39], [40]. Here we show a good concordance of many transcription-linked histone marks suggesting a combinatorial effect in the regulation of gene expression during the *P. falciparum* IDC. Interestingly, these euchromatic marks (individually or in combination) associate with distinct functionalities that could be broadly divided into two main biological categories: (i) growth (e.g. protein biosynthesis, nucleotide metabolism, and DNA replication), and (ii) host parasite interaction (e.g. merozoite invasion, Maurer's cleft proteins) (Figure S5). This may reflect a regulatory link between the two most crucial functions determining parasite virulence during infection: multiplication rate and interaction with the host immune system.

Given the essential role of epigenetic regulation in gene expression, unique factors associated with these processes are presently considered as drug targets for malaria as well as other human parasitic diseases [41], [42]. One such factor is histone deacetylase (HDAC) which plays a pivotal role in chromatin remodeling and thus transcriptional activity. In *P. falciparum*, the HDAC inhibitor apicidin causes a massive hyperacetylation of H3K9 and H4K8 (and demethylation of H3K4) residues leading to global deregulation of the IDC transcriptional cascade [20]. This deregulation can be induced by at least three other HDAC inhibitors including a 2-aminosuberic acid derivative, Trichostatin A and SAHA, the latter being currently approved for cancer therapy [43]. Moreover, these inhibitors are able to effectively inhibit *Plasmodium* HDACs [44] and oral administration of apicidin at 2–20 mg/kg for 3 days cures *P. berghei* infection in mice [45]. This suggests that inhibition of epigenetic mechanisms in *Plasmodium* represents a promising target area for malaria drug development, and more efforts in developing new compounds with higher selectivity as well as bioavailability are ongoing [46]. However, the development of new antimalaria “epi-drugs” may not be restricted to HDACs, but could also target their opposing histone acetyl transferases or other factors such as chromatin remodeling complexes and signaling pathways impinging on these processes. Our results with the histone modification landscape in the most pathogenic malaria parasite, *P. falciparum*, will provide a solid reference for all epi-drug development in malaria as well as other parasitic diseases in the future.

## Materials and Methods

### Parasite culture

Highly synchronized cells of *P. falciparum* strain T996 were cultured at 5% parasitemia and 2% hematocrit under standard conditions [47]. For ChIP, saponin-lysed parasites were cross-linked with 0.5% formaldehyde and harvested at 8, 16, 24, 32, 40 and 48 hpi. Samples were also collected for RNA and protein isolation from the same time points.

### Immunodetection

Equal amounts of total protein lysate obtained from parasite pellets from the 6 time points were separated by 12% SDS PAGE and transferred onto nitrocellulose membrane. Western hybridizations were carried out using antibodies (Millipore, Upstate) directed against the modified histones. Horseradish peroxidase conjugated secondary antibody was purchased from GE Healthcare. We also performed immuno-localization with these antibodies as described [48].

### Chromatin immunoprecipitation

Cross-linked cells were homogenized with 200 strokes of a dounce homogenizer and lysed using 1% SDS. The resulting nuclear extract was sonicated with 8 bursts of 10 sec with 50 sec rest between bursts to shear DNA to a final length of 200 to 1000 bp. The sonicate was then centrifuged for 10 min at 13,000× g, and sheared DNA incubated with the immunoprecipitating antibody overnight at 4°C followed by incubation with salmon sperm DNA/Protein A agarose slurry (Millipore) for 1 h at 4°C. Protein A agarose was gently pelleted followed by extensive washes. The DNA bound to protein of interest was reverse cross-linked using 0.2 M NaCl and incubation overnight at 65°C. Recovered DNA was purified using the QIAEX II kit (QIAGEN). Amplification of immunoprecipitated DNA as well as sonicated genomic DNA (input) was carried out as described [49] with a few modifications [50]. Equal amount of Cy5-labeled amplified ChIP DNA was hybridized to Cy3-labeled amplified input DNA. For sequential ChIP, the eluted complex from the first ChIP was subjected to immunoprecipitation using second antibody as described [51]. During the second round of immunoprecipitation, no antibody control was included.

### RNA extraction

RNA was isolated to carry out transcriptional profiling at the appropriate time points. RNA extraction and cDNA synthesis were carried out as described [52]. Cy5-labeled cDNA was hybridized against a Cy3-labeled reference pool which was made by combining equal amounts of RNA from each time point.

### Microarray hybridizations and data analysis

Equal amounts of Cy5 and Cy3 labeled samples were hybridized to *P. falciparum* microarrays containing 5,402 50-mer intergenic oligonucleotide probes and 10,416 70-mer ORF probes representing 5,343 coding genes [53]. The intergenic regions were represented by one highly specific probe (up to 1.5 kb upstream of the start codon) whose microarray hybridization parameters were matched to the intragenic probe set using the OligoRankPick algorithm [53]. Using PlasmoDB version 8.2, we were able to remap 14,773 probes to the *P. falciparum* genome providing an even coverage with at least one probe per 1.542 kb. *P. falciparum* strain T996 was chosen to carry out these experiments due to an exact IDC length of 48 h and the ease with which it can be synchronized. Since the probes on the array have been designed for 3D7, we excluded *vars*, rifins and stevors from our analysis. The microarray hybridization was carried out at 63.5°C or 65°C in the automated hybridization station (MAUI, USA) for ChIP DNA or cDNA, respectively, as described [4]. The microarrays were scanned using the GenePix scanner 4000B and GenePix pro 6.0 software (Axon Laboratory).

Lowess normalized data was processed to filter out spots with signal intensity less than twice the background intensity for both Cy5 and Cy3 fluorescence. The relative occupancy of histone marks is represented by log_2_ ChIP/input ratios where high and low ratios represent strong and weak enrichment respectively of modified histones. For expression analysis, each gene profile was represented by an average expression value calculated as an average of all probes representing a particular gene. For ChIP-on-chip, all microarrays were done in triplicates. For each time point, probes with data present in at least 2 out of 3 triplicates were included. Data was presented as an average of triplicates after K^th^ nearest neighbor (KNN) imputation. Data was further filtered to include only those probes where signal from ChIP DNA was obtained in at least 2 consecutive time points. To address dynamics across the life cycle, the average ChIP/input ratio of each time point was used to detect the summit and bottom time point of enrichment for every probe. *P* value was assigned based on a student's t-test between the replicates. Significantly oscillated occupancy profiles (relative occupancy defined by log_2_ ChIP/input ratio) between the summit and bottom time points were defined as *P* value<0.05 and fold change ≥1.5 across the IDC referring to all detectable dynamic probes (within the limit of the applied ChIP-on-chip technology) and probes showing the highest level of change in marked histone occupancy, respectively.

To assess overall ChIP enrichment at every time point, probes were divided into 3 groups based on expression of the respective genes at each time point: top, middle and bottom 10% of total probes with highest, intermediate and lowest levels of expression, respectively. For each group, ChIP/input log_2_ ratios were plotted against probe position from −1000 bp to +3000 bp with respect to ATG at every time point.

Phaseograms for expression and ChIP data were generated by fast Fourier transform method where probes/genes were sorted according to phase from −π to π with the mean-centered log_2_ ratios across all the time points.

Pearson's Correlation coefficient (PCC or r) was calculated between the histone mark profiles and corresponding mRNA profiles across the IDC and skewness of correlation distribution was calculated. Negative skew values indicate a long tail on the negative side (higher frequency on positive side). For ease of understanding, skew is represented as the negative of skew throughout the manuscript such that positive skew means a higher frequency on the positive side. To test how statistically significant the histone levels correlate with expression levels at each probe, we randomly generated marked histone and expression profile pairs by randomizing profiles between probes 100 times. The two-sample Kolmogorov-Smirnov (KS) test was used to test whether our observed correlation distributions are different from the random ones (*P* value<0.0005). The same analysis was also performed for correlations between histone modifications. Dice's coefficient was calculated in order to assess the overlap between any two histone modification profiles across the IDC.

ChIP enrichment with respect to position in the gene was calculated using probes with oscillated profiles (*P*<0.05 and fold change ≥1.5 across the IDC). For each histone mark, the number of ChIP-enriched probes showing positive (r≥0.4) and negative (r≤−0.4) correlation with expression profiles of corresponding genes were normalized to total probes in the input. Data were arranged into bins ranging from −1500 bp upstream of ATG to +3000 bp into the gene and plotted against the percentage of probes falling in each bin.

### Quantitative real time PCR (RTQ-PCR)

RTQ-PCR was carried out on immunoprecipitated and input DNA using the SYBR Green PCR Master Mix (Roche) according to manufacturer's instructions. ChIP enrichment was calculated by using the ΔCt method (Ct of immunoprecipitated target gene - Ct of input target gene) where Ct is the threshold cycle. All PCR reactions were done in duplicates or triplicates.

### Transfection

Vector pLN-ENR-GFP and *P. falciparum* strain Dd2^attB^ were provided by D. Fidock. The GFP cassette from the vector pLN-ENR-GFP [32] was replaced by the firefly luciferase gene and *hsp* 86 3′ UTR from the plasmid pPF86 [54] at the *Bam* HI/*Apa* I sites to create the plasmid pLN-Luc. All constructs were confirmed by sequencing. For transfection, promoter regions of various genes were cloned upstream of the luciferase gene at the *Bam* HI/*Sph* 1 sties of pLN-Luc. Transfections of *P. falciparum* strain Dd2^attB^ and subsequent drug selection were carried out as described [32]. Vehicle vector lacking the luciferase gene was used as a negative control and all transfectants were checked for firefly luciferase activity 48 h post infection using a reporter assay from Promega. Plasmid integration at *cg6* locus was confirmed by PCR.

### Primers

All primer sequences used in the current study are listed in Table S4.

### Data access

The microarray data have been submitted to NCBI GEO with accession number GSE39238.

### List of genes/protein names used in the study

1. MAL13P1.122: SET domain protein, putative (SET2)
2. PF14_705: conserved *Plasmodium* protein, unknown function
3. PFD0240c: 6-cysteine protein (P41)
4. PFC0210c: Circumsporozoite protein
5. *cg6*: gene coding for glutaredoxin-like protein (GLP3)
6. *hsp* 86: gene coding for heat shock protein 90 (HSP90)
7. GFP: Green fluorescent protein
8. Luciferase: Luciferase protein

## Supporting Information

Figure S1 **Validation of antibodies used for ChIP-on-chip.** (A) Equal amounts of protein extracted at 8, 16, 24, 32, 40 and 48 hpi (from the same time course used for ChIP-on-chip) were used in 12% SDS PAGE. Western blots were carried out using antibodies against the studied histone epitopes. Actin was used as a loading control. (B) The specificity of the antibodies was further confirmed in peptide competition assays for 7 representative antibodies. The modified peptides (Millipore or Abcam) used correspond to respective modifications on the histone against which the antibodies were raised. Western blots were done with equal amounts (5 µg per lane) of ring stage protein. Antibody was incubated for 2 h with no peptide, 100-fold molar excess of specific peptide, 200-fold molar excess of specific peptide and 200-fold molar excess of a non-specific peptide before reacting with the blot. HDAC1 was used as a loading control in all 4 lanes. (C) Same antibodies were used for immuno-fluorescence analysis (IFA) of histone modifications in *P. falciparum*. Nuclear localization of histone marks was confirmed by IFAs done on formaldehyde fixed ring, trophozoite and schizont stage parasites. Nuclear DNA was stained with DAPI (blue) and all histone marks can be seen in pink.
(TIF)

Figure S2 **Validation of ChIP results.** (A) Using immunoprecipitated DNA, quantitative real time PCR was carried out on representative genes chosen for three histone modifications to validate the ChIP occupancy profiles across the IDC. Primers for real time PCR were designed to amplify 200–300 bp regions around the respective probe on the array. The x-axis represents hours post invasion and Y-axis the smoothed log_2_ ratio of ChIP enrichment over input for both real time PCR and microarray results. Solid red and purple lines refer to real time PCR profiles obtained for IGR and ORF, respectively, of the specified gene, whereas dotted lines refer to the microarray profiles of corresponding genes. Numbers at the bottom right corner of each graph refer to the Spearman's rank coefficient between ChIP-on-chip and RT PCR profile at IGR (red) and ORF (purple) regions. Note that except in two instances, the rest of the profiles from microarray and real time results show positive correlation. (B) Performance of microarray hybridization achieved from amplified DNA at AT rich IGRs was analyzed by comparing the relation between signal intensity and Cy5/Cy3 ratio. MA plots were derived for Cy5 labeled H4K5ac DNA hybridized against Cy3 labeled input DNA (sonicated genomic DNA) at 6 time points (TP1 to TP6) and also for Cy5 labeled input DNA hybridized against Cy3 labeled input DNA. ‘M’ stands for log_2_ ratio of Cy5/Cy3 after lowess normalization (y-axis) and ‘A’ stands for average log_2_ intensity of Cy5 and Cy3 (x-axis). Red line is the lowess smoothed M by A which is used to indicate data trend for each graph. “r” is Pearson's correlation coefficient of M and A (indicated at top of each plot). As shown, Cy5/Cy3 signal ratio is independent of signal intensity at both ORFs (left panel) and IGRs (right panel).
(TIF)

Figure S3 **Gene models of ChIP-on-chip enrichment.** (A) The graph represents the percentage of ChIP-enriched probes for each histone mark with *P*<0.05 and fold change ≥1.5 across the IDC in both intergenic regions (IGRs) and open reading frames (ORFs). Chi-square test was done to identify a positional bias towards IGR or ORF. Dark and light green boxes at top indicate preference at ORF and IGR, respectively. (B) The graph represents the percentage of ChIP-enriched genes (represented by probes for respective genes) for each histone mark with *P*<0.05 and fold change ≥1.5 across the IDC in both IGRs and ORFs. Overlap refers to the genes represented by probes associated with the histone marks in both IGR and ORF.
(TIF)

Figure S4 **Transcription associated histone marks.** (A) PCC was calculated between profiles of ChIP enriched probes (*P*<0.05 and fold change ≥1.5 across the IDC) and expression profiles of corresponding genes. The y-axis represents the percentage of probes showing positive (r≥0.4), no (r between 0.4 and −0.4) or negative (r≤−0.4) PCC for each histone mark. (B) Phaseograms representing histone mark profiles for probes showing maximum correlation with expression across the IDC. The yellow/blue color scale represents lowess smoothed profiles calculated from centered curves of relative occupancy ratios measured by ChIP/input (log_2_) for the 6 time points (TP1-6 representing 0 to 48 hpi). The gene/probe order in each phaseogram was determined independently using Fast Fourier transformation (see Materials and Methods). Vertical numbers indicate probes (p) or genes (g) represented by each histone mark.
(TIF)

Figure S5 **Enrichment of selected gene families.** Heat maps showing the marked histone occupancy of selected gene families. To maximize our dataset, we included all probes showing oscillating profiles (*P*<0.05 without consideration of fold change) across the IDC for every histone mark. Each row represents PCC (r) of ChIP profiles with expression profiles of the respective genes. For enrichment of multiple probes for the same gene, the probe with the maximum correlation with expression was included and hierarchical clustering was carried out. Scale bar indicates PCC between ChIP and expression profiles.
(TIF)

Table S1 **Processed microarray hybridization data.** The table contains log_2_ ratio of cDNA/pooled cDNA or ChIP/input DNA signal for all 6 time points across the IDC. Data was averaged for all probes of the same gene in case of cDNA and each probe of same gene has been assigned the same value in this table. For every histone mark, only those probes were included having ChIP data in at least two time points out of six. All experiments were done in triplicate and averaged across the triplicates after KNN imputing. First and second columns contain the probe id and corresponding gene, respectively, for each row. The next six rows contain RNA expression data. The following columns contain ChIP data, *P* value across the IDC (as described in Materials and Methods), fold change across IDC and Pearson's correlation coefficient with expression profiles for each histone modification. For modifications lacking triplicates, no *P* value was assigned.
(ZIP)

Table S2 **Overlap between histone marks.** This table shows the data for overlap between any two pairs of histone modifications. Probes with fold change ≥1.5 were included in this analysis. Chart 1: Dice's coefficient calculated to assess the degree of overlap between any two marked histone pairs. Chart 2: *P* values based on KS test (as described in Materials and Methods) to test if the r distribution is random or not between overlapping profiles of any two marked histone pairs. Chart 3: Skewness distribution based on r values for overlapping profiles between two marked histone pairs.
(XLSX)

Table S3 **Sequential ChIP.** The table shows the average log_2_ ratios of ChIP/Input microarray signal from duplicate experiments. Sequential ChIP (ring stage parasites) was carried out using anti-H3K56ac antibody followed by either anti-H3K9ac antibody or no antibody as control. Data was compared to immunoprecipitated DNA using antibody against H3K56ac or H3K9ac alone.
(XLSX)

Table S4 **Sequence of primers used for amplifications in the current study.**
(XLSX)
