# Supplementary figures and images for: Dynamic Epigenetic Regulation of Gene Expression during the Life Cycle of Malaria Parasite Plasmodium falciparum
Source: PLoS Pathog. 2013 Feb 28;9(2):e1003170. doi: 10.1371/journal.ppat.1003170 (PMC3585154; doi:10.1371/journal.ppat.1003170)

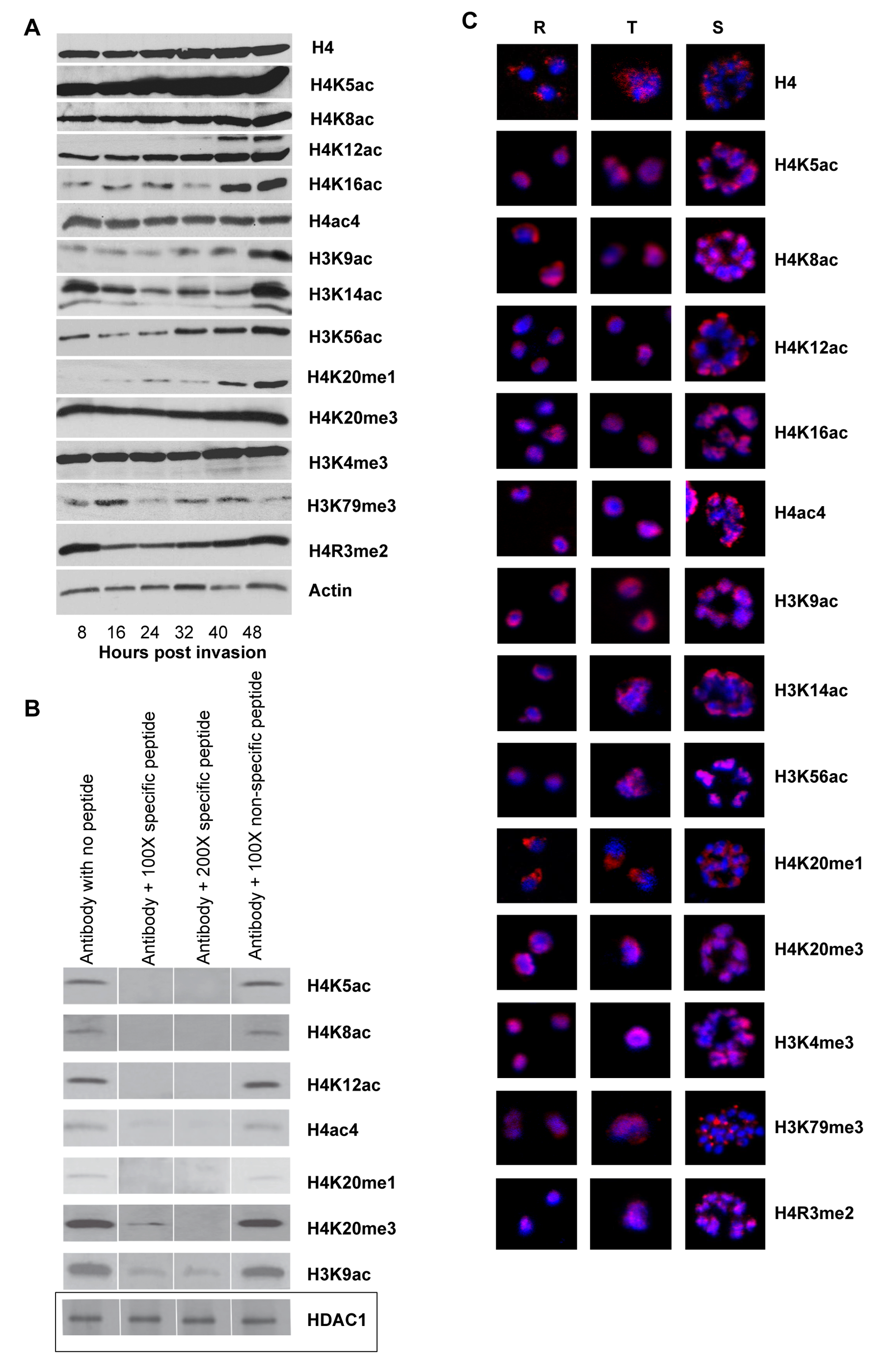

Supplement: Figure S1 — Validation of antibodies used for ChIP-on-chip. (A) Equal amounts of protein extracted at 8, 16, 24, 32, 40 and 48 hpi (from the same time course used for ChIP-on-chip) were used in 12% SDS PAGE. Western blots were carried out using antibodies against the studied histone epitopes. Actin was used as a loading control. (B) The specificity of the antibodies was further confirmed in peptide competition assays for 7 representative antibodies. The modified peptides (Millipore or Abcam) used correspond to respective modifications on the histone against which the antibodies were raised. Western blots were done with equal amounts (5 µg per lane) of ring stage protein. Antibody was incubated for 2 h with no peptide, 100-fold molar excess of specific peptide, 200-fold molar excess of specific peptide and 200-fold molar excess of a non-specific peptide before reacting with the blot. HDAC1 was used as a loading control in all 4 lanes. (C) Same antibodies were used for immuno-fluorescence analysis (IFA) of histone modifications in P. falciparum. Nuclear localization of histone marks was confirmed by IFAs done on formaldehyde fixed ring, trophozoite and schizont stage parasites. Nuclear DNA was stained with DAPI (blue) and all histone marks can be seen in pink. (TIF) [file ppat.1003170.s001.tif]

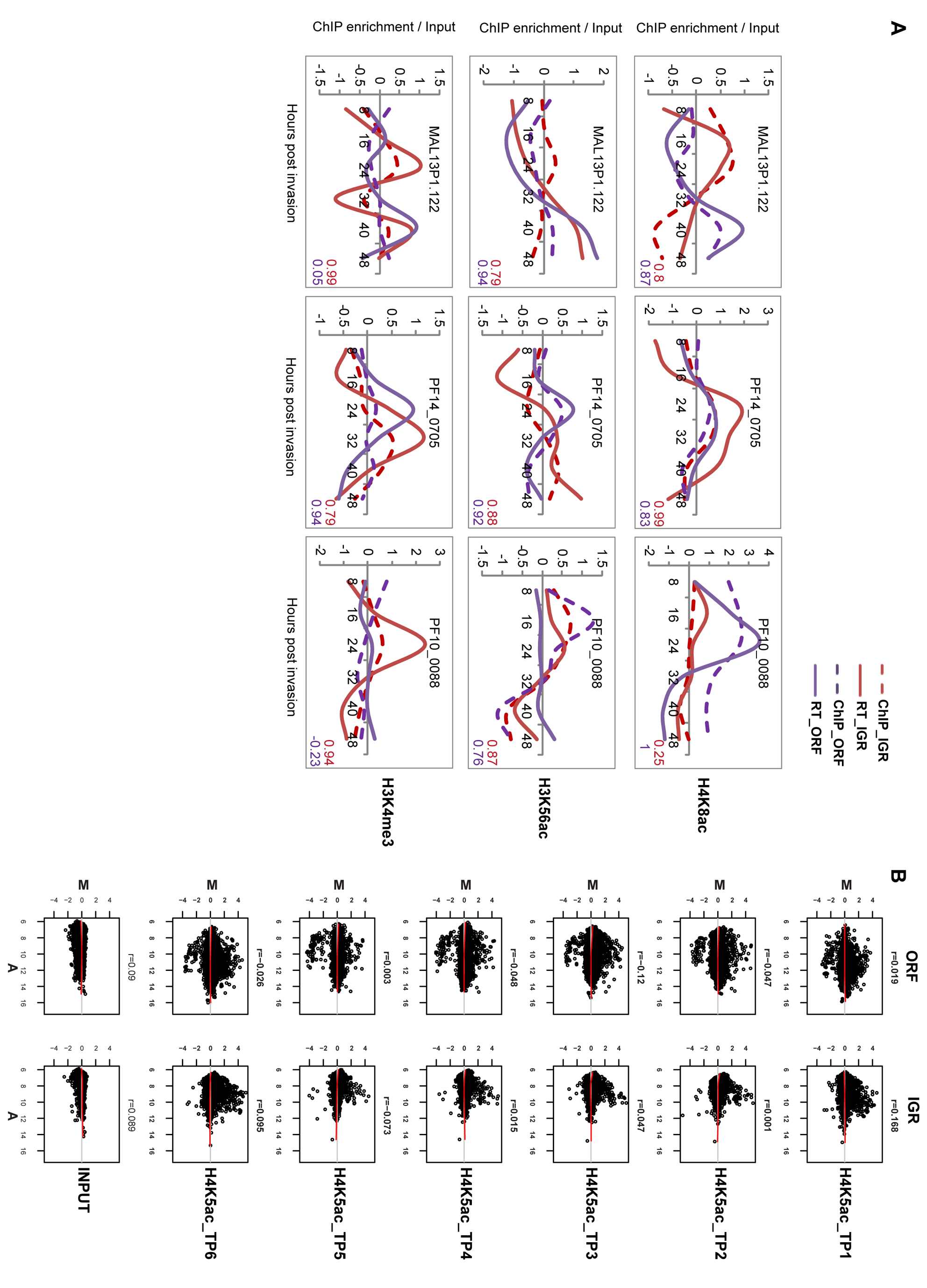

Supplement: Figure S2 — Validation of ChIP results. (A) Using immunoprecipitated DNA, quantitative real time PCR was carried out on representative genes chosen for three histone modifications to validate the ChIP occupancy profiles across the IDC. Primers for real time PCR were designed to amplify 200–300 bp regions around the respective probe on the array. The x-axis represents hours post invasion and Y-axis the smoothed log2 ratio of ChIP enrichment over input for both real time PCR and microarray results. Solid red and purple lines refer to real time PCR profiles obtained for IGR and ORF, respectively, of the specified gene, whereas dotted lines refer to the microarray profiles of corresponding genes. Numbers at the bottom right corner of each graph refer to the Spearman's rank coefficient between ChIP-on-chip and RT PCR profile at IGR (red) and ORF (purple) regions. Note that except in two instances, the rest of the profiles from microarray and real time results show positive correlation. (B) Performance of microarray hybridization achieved from amplified DNA at AT rich IGRs was analyzed by comparing the relation between signal intensity and Cy5/Cy3 ratio. MA plots were derived for Cy5 labeled H4K5ac DNA hybridized against Cy3 labeled input DNA (sonicated genomic DNA) at 6 time points (TP1 to TP6) and also for Cy5 labeled input DNA hybridized against Cy3 labeled input DNA. ‘M’ stands for log2 ratio of Cy5/Cy3 after lowess normalization (y-axis) and ‘A’ stands for average log2 intensity of Cy5 and Cy3 (x-axis). Red line is the lowess smoothed M by A which is used to indicate data trend for each graph. “r” is Pearson's correlation coefficient of M and A (indicated at top of each plot). As shown, Cy5/Cy3 signal ratio is independent of signal intensity at both ORFs (left panel) and IGRs (right panel). (TIF) [file ppat.1003170.s002.tif]

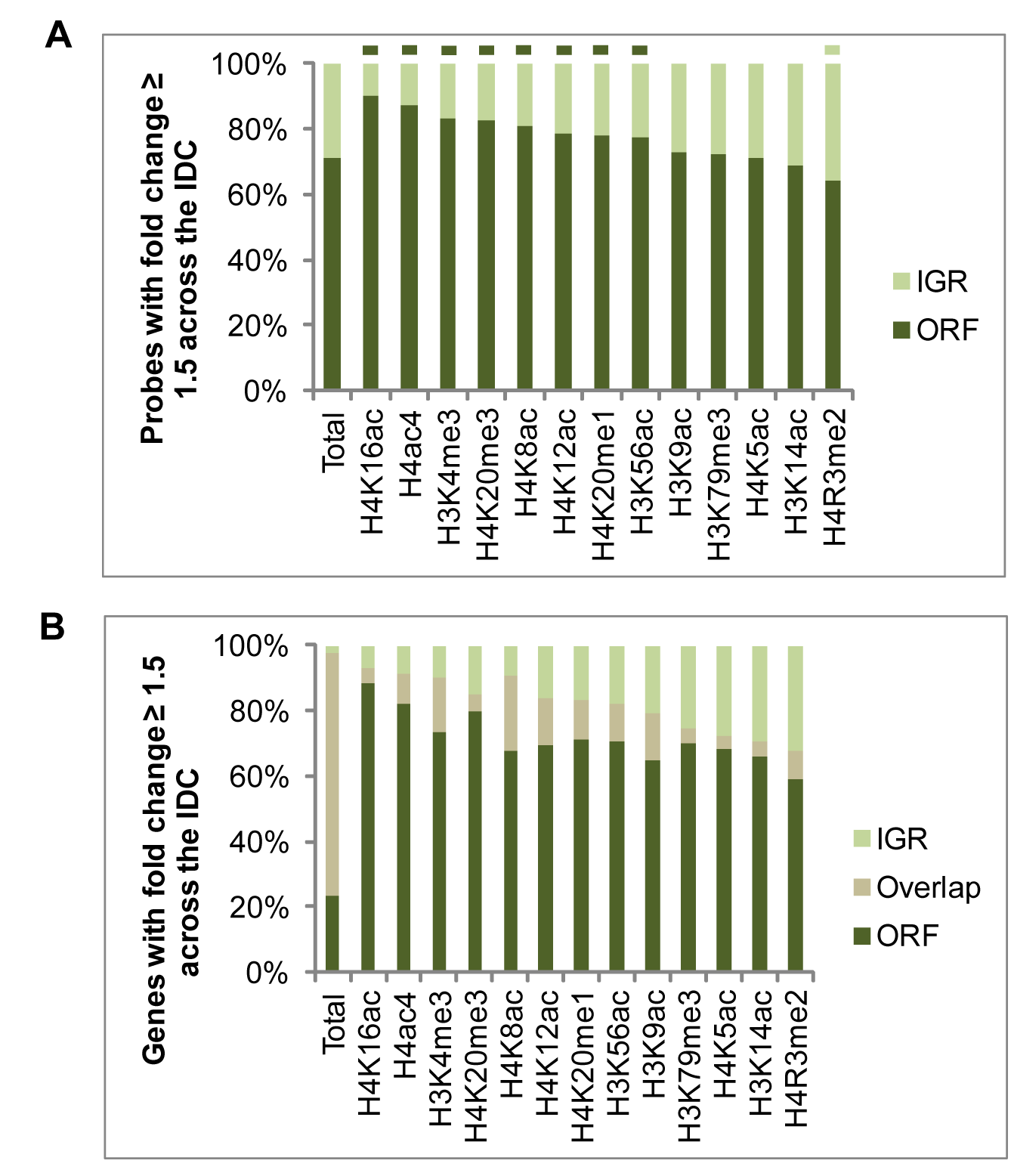

Supplement: Figure S3 — Gene models of ChIP-on-chip enrichment. (A) The graph represents the percentage of ChIP-enriched probes for each histone mark with P<0.05 and fold change ≥1.5 across the IDC in both intergenic regions (IGRs) and open reading frames (ORFs). Chi-square test was done to identify a positional bias towards IGR or ORF. Dark and light green boxes at top indicate preference at ORF and IGR, respectively. (B) The graph represents the percentage of ChIP-enriched genes (represented by probes for respective genes) for each histone mark with P<0.05 and fold change ≥1.5 across the IDC in both IGRs and ORFs. Overlap refers to the genes represented by probes associated with the histone marks in both IGR and ORF. (TIF) [file ppat.1003170.s003.tif]

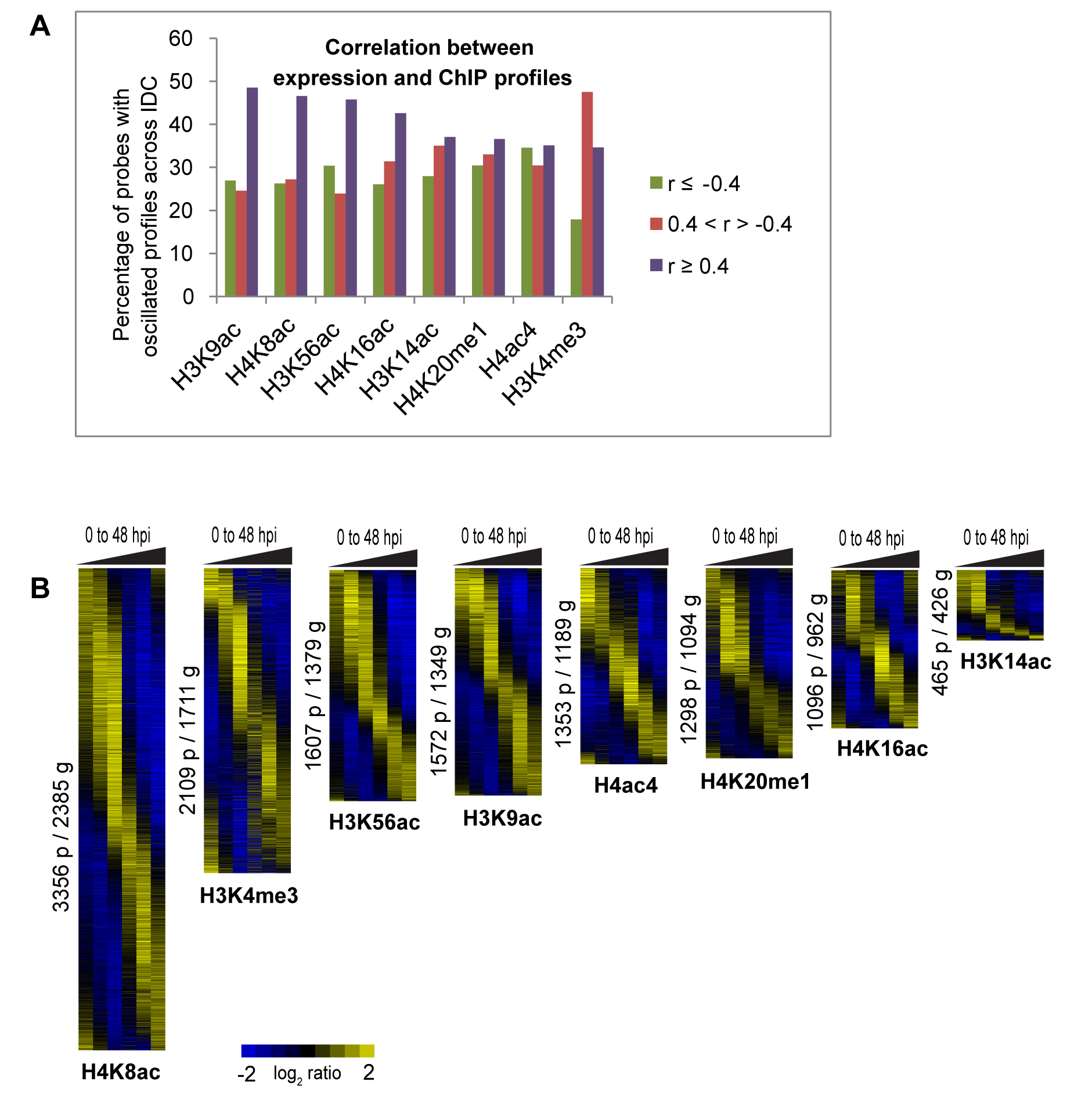

Supplement: Figure S4 — Transcription associated histone marks. (A) PCC was calculated between profiles of ChIP enriched probes (P<0.05 and fold change ≥1.5 across the IDC) and expression profiles of corresponding genes. The y-axis represents the percentage of probes showing positive (r≥0.4), no (r between 0.4 and −0.4) or negative (r≤−0.4) PCC for each histone mark. (B) Phaseograms representing histone mark profiles for probes showing maximum correlation with expression across the IDC. The yellow/blue color scale represents lowess smoothed profiles calculated from centered curves of relative occupancy ratios measured by ChIP/input (log2) for the 6 time points (TP1-6 representing 0 to 48 hpi). The gene/probe order in each phaseogram was determined independently using Fast Fourier transformation (see Materials and Methods). Vertical numbers indicate probes (p) or genes (g) represented by each histone mark. (TIF) [file ppat.1003170.s004.tif]

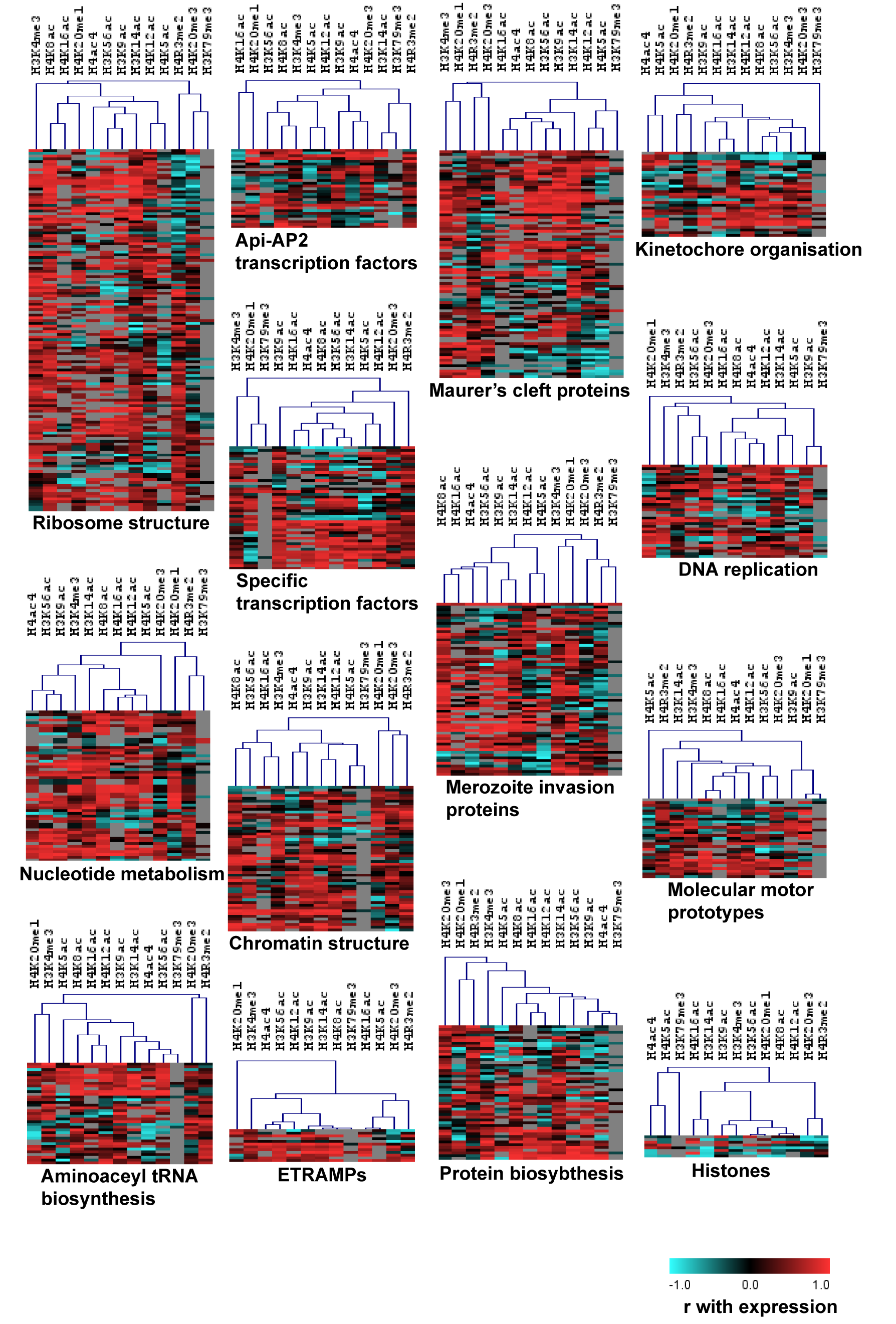

Supplement: Figure S5 — Enrichment of selected gene families. Heat maps showing the marked histone occupancy of selected gene families. To maximize our dataset, we included all probes showing oscillating profiles (P<0.05 without consideration of fold change) across the IDC for every histone mark. Each row represents PCC (r) of ChIP profiles with expression profiles of the respective genes. For enrichment of multiple probes for the same gene, the probe with the maximum correlation with expression was included and hierarchical clustering was carried out. Scale bar indicates PCC between ChIP and expression profiles. (TIF) [file ppat.1003170.s005.tif]
