# Supplementary material for: Dynamic Epigenetic Regulation of Gene Expression during the Life Cycle of Malaria Parasite Plasmodium falciparum
Source: PLoS Pathog. 2013 Feb 28;9(2):e1003170. doi: 10.1371/journal.ppat.1003170 (PMC3585154; doi:10.1371/journal.ppat.1003170)
Supplement: Table S1 — Processed microarray hybridization data. The table contains log2 ratio of cDNA/pooled cDNA or ChIP/input DNA signal for all 6 time points across the IDC. Data was averaged for all probes of the same gene in case of cDNA and each probe of same gene has been assigned the same value in this table. For every histone mark, only those probes were included having ChIP data in at least two time points out of six. All experiments were done in triplicate and averaged across the triplicates after KNN imputing. First and second columns contain the probe id and corresponding gene, respectively, for each row. The next six rows contain RNA expression data. The following columns contain ChIP data, P value across the IDC (as described in Materials and Methods), fold change across IDC and Pearson's correlation coefficient with expression profiles for each histone modification. For modifications lacking triplicates, no P value was assigned. (ZIP) [file Table S1.pdf › Table S1.pdf]

probe_id	target_gene	RNA_TP1	RNA_TP2	RNA_TP3	RNA_TP4	RNA_TP5
achr14-tR-Met-1_0	1396.pre-tR-Met-1	1.008257	0.537727	0.633537	-0.2622	-1.53239
achr11-tR-Gly-1_0	1399.pre-tR-Gly-1	0.679607	0.498947	0.352107	-0.17677	-0.80461
intPF11_0044_2	1399.pre-tR-Leu-1	-0.90407	-0.56851	0.125272	0.635842	0.646452
achr11-tR-Leu-1_0	1399.pre-tR-Leu-1	-0.50834	-0.23607	0.217207	0.412167	0.232107
achr2-tR-Gln-1_0	1400.pre-tR-Gln-1	0.730573	1.304143	0.185213	-0.72655	-0.80348
achr12-tR-Gln-1_0	2277.pre-tR-Glu-1	0.945617	0.375927	0.411147	0.025187	-0.78059
acoxIII_mit_2	mal_mito_3	-1.5054	-2.31448	-0.39912	1.40667	1.80054
acoxIII_mit_1	mal_mito_3	-1.5054	-2.31448	-0.39912	1.40667	1.80054
acoxI_mit_1	mal_mito_3	-1.99495	-2.33677	-0.66446	1.32096	2.30677
acoxI_mit_0	mal_mito_3	-1.99495	-2.33677	-0.66446	1.32096	2.30677
acoI_mit_2	mal_mito_3	-1.58099	-2.24542	-0.37816	1.373163	1.783083
acoI_mit_1	mal_mito_3	-1.58099	-2.24542	-0.37816	1.373163	1.783083
ac1rR28s_2	MAL1_28s	0.637083	1.156103	-0.26271	-1.20263	-0.76394
ac1rR28s_1	MAL1_28s	0.637083	1.156103	-0.26271	-1.20263	-0.76394
ac1rR28s_0	MAL1_28s	0.637083	1.156103	-0.26271	-1.20263	-0.76394
ac1rRITS2_0	MAL1_ITS2	-0.94801	-0.50198	0.134463	0.607203	0.645643
achr13-MALr.8_0	MAL13_tR_Arg1	-0.17077	-0.07858	0.187113	0.191273	-0.0516
achr13-MALr.12_0	MAL13_tR_Arg2	0.600263	1.149583	0.569273	-0.26565	-0.87584
aun-1-tR-Arg-1_0	MAL13_tR_Arg3	1.771133	0.622343	-0.62169	-1.23065	-0.88168
achr13-MALr.16_0	MAL13_tR_Arg3	2.027547	0.735287	-0.33906	-1.2786	-1.48195
achr13-MALr.18_0	MAL13_tR_Cys1	0.957423	0.680463	-0.24513	-0.71501	-0.4886
aun-3-tR-Gly-1_0	MAL13_tR_Gly1	0.504162	0.532262	0.209942	-0.36831	-0.75058
achr13-MALr.20_0	MAL13_tR_Gly1					
achr13-MALr.4_0	MAL13_tR_Met1	0.473757	0.003837	0.343127	0.089407	-0.6503
intMAL13P1.188_2	MAL13_tR_Ser1	0.575107	-0.32352	-2.1282	-1.55196	1.166357
achr13-MALr.2_0	MAL13_tR_Ser1	-0.56508	-0.06475	0.501268	0.411588	-0.15641
achr13-MALr.10_0	MAL13_tR_Thr1	0.264268	0.324308	0.152928	-0.11023	-0.31761
aun-1-tR-Gly-1_0	MAL13_tR_Trp1	1.005023	0.749373	0.279003	-0.40484	-0.95421
achr13-MALr.14_0	MAL13_tR_Trp1	1.32194	1.4493	0.2824	-1.0014	-1.4943
aMAL13P1.100_1	MAL13P1.100	-0.50776	-0.94266	-0.50593	0.458767	1.156497
aMAL13P1.100_0	MAL13P1.100	-0.50776	-0.94266	-0.50593	0.458767	1.156497
intMAL13P1.102_2	MAL13P1.102	1.363833	0.182853	-1.81246	-1.99488	-0.00219
aMAL13P1.102_1	MAL13P1.102	1.363833	0.182853	-1.81246	-1.99488	-0.00219
aMAL13P1.102_0	MAL13P1.102	1.363833	0.182853	-1.81246	-1.99488	-0.00219
intMAL13P1.103_2	MAL13P1.103	-1.03121	-1.69099	-0.75128	0.790943	1.780153
aMAL13P1.103_1	MAL13P1.103	-1.03121	-1.69099	-0.75128	0.790943	1.780153
aMAL13P1.103_0	MAL13P1.103	-1.03121	-1.69099	-0.75128	0.790943	1.780153
intMAL13P1.105_2	MAL13P1.105	-1.14969	-0.77706	-0.15138	0.73688	1.24806
aMAL13P1.105_1	MAL13P1.105	-1.14969	-0.77706	-0.15138	0.73688	1.24806
aMAL13P1.105_0	MAL13P1.105	-1.14969	-0.77706	-0.15138	0.73688	1.24806
intMAL13P1.106_2	MAL13P1.106	-0.13516	0.097797	0.206917	0.182417	0.004537
aMAL13P1.106_0	MAL13P1.106	-0.13516	0.097797	0.206917	0.182417	0.004537
intMAL13P1.107_2	MAL13P1.107	0.122237	0.005797	0.260647	0.213187	-0.19496
aMAL13P1.107_2	MAL13P1.107	0.122237	0.005797	0.260647	0.213187	-0.19496
aMAL13P1.107_1	MAL13P1.107	0.122237	0.005797	0.260647	0.213187	-0.19496
aMAL13P1.107_0	MAL13P1.107	0.122237	0.005797	0.260647	0.213187	-0.19496

intMAL13P1.11_2	MAL13P1.11					
intPF13_0117_2	MAL13P1.111	-0.00414	0.01668	-0.10337	-0.00113	0.20032
aMAL13P1.111_0	MAL13P1.111	0.238847	-0.31122	-0.35704	-0.02862	0.324557
aMAL13P1.112_1	MAL13P1.112	-1.38142	-0.0294	-0.08793	0.212767	0.921237
aMAL13P1.112_0	MAL13P1.112	-1.38142	-0.0294	-0.08793	0.212767	0.921237
intPF13_0118_2	MAL13P1.113	-1.17927	-0.22961	0.509727	0.714607	0.377447
aMAL13P1.113_0	MAL13P1.113	-1.44785	0.45623	0.89505	0.63195	0.09377
intMAL13P1.114_2	MAL13P1.114	0.08456	-0.05965	-2.04528	-1.52238	1.39815
aMAL13P1.114_5	MAL13P1.114	0.08456	-0.05965	-2.04528	-1.52238	1.39815
aMAL13P1.114_4	MAL13P1.114	0.08456	-0.05965	-2.04528	-1.52238	1.39815
aMAL13P1.114_3	MAL13P1.114	0.08456	-0.05965	-2.04528	-1.52238	1.39815
aMAL13P1.114_2	MAL13P1.114	0.08456	-0.05965	-2.04528	-1.52238	1.39815
aMAL13P1.114_1	MAL13P1.114	0.08456	-0.05965	-2.04528	-1.52238	1.39815
aMAL13P1.114_0	MAL13P1.114	0.08456	-0.05965	-2.04528	-1.52238	1.39815
intMAL13P1.115_2	MAL13P1.115	0.314813	0.291913	-0.14883	-0.52051	-0.41825
aMAL13P1.115_0	MAL13P1.115	0.314813	0.291913	-0.14883	-0.52051	-0.41825
aMAL13P1.116_4	MAL13P1.116	0.617038	0.571288	0.224038	-0.37837	-0.80312
aMAL13P1.116_3	MAL13P1.116	0.617038	0.571288	0.224038	-0.37837	-0.80312
aMAL13P1.116_2	MAL13P1.116	0.617038	0.571288	0.224038	-0.37837	-0.80312
aMAL13P1.116_1	MAL13P1.116	0.617038	0.571288	0.224038	-0.37837	-0.80312
aMAL13P1.116_0	MAL13P1.116	0.617038	0.571288	0.224038	-0.37837	-0.80312
intMAL13P1.117_3	MAL13P1.117	0.38465	0.45873	-0.1468	-0.53968	-0.36277
intMAL13P1.116_2	MAL13P1.117	0.617038	0.571288	0.224038	-0.37837	-0.80312
aMAL13P1.117_0	MAL13P1.117	0.38465	0.45873	-0.1468	-0.53968	-0.36277
intMAL13P1.118_2	MAL13P1.118	-0.92243	-1.06633	-1.4826	-0.2329	1.96533
aMAL13P1.118_1	MAL13P1.118	-0.92243	-1.06633	-1.4826	-0.2329	1.96533
aMAL13P1.118_0	MAL13P1.118	-0.92243	-1.06633	-1.4826	-0.2329	1.96533
intMAL13P1.119_2	MAL13P1.119	0.140087	-0.20245	-0.69358	-0.50608	0.330787
aMAL13P1.119_1	MAL13P1.119	0.140087	-0.20245	-0.69358	-0.50608	0.330787
aMAL13P1.119_0	MAL13P1.119	0.140087	-0.20245	-0.69358	-0.50608	0.330787
intMAL13P1.11a_2	MAL13P1.11a					
aMAL13P1.12_0	MAL13P1.12	0.542297	-0.1895	0.048017	0.039317	-0.33616
aMAL13P1.120_1	MAL13P1.120	0.26902	0.79692	0.23941	-0.24641	-0.36692
aMAL13P1.120_0	MAL13P1.120	0.26902	0.79692	0.23941	-0.24641	-0.36692
intMAL13P1.121_2	MAL13P1.121	-0.81813	-1.64384	-0.63308	0.83958	1.59684
aMAL13P1.121_1	MAL13P1.121	-0.81813	-1.64384	-0.63308	0.83958	1.59684
aMAL13P1.121_0	MAL13P1.121	-0.81813	-1.64384	-0.63308	0.83958	1.59684
intMAL13P1.122_2	MAL13P1.122	1.646063	0.803323	0.084683	-1.0058	-1.6771
aMAL13P1.122_3	MAL13P1.122	1.646063	0.803323	0.084683	-1.0058	-1.6771
aMAL13P1.122_2	MAL13P1.122	1.646063	0.803323	0.084683	-1.0058	-1.6771
aMAL13P1.122_1	MAL13P1.122	1.646063	0.803323	0.084683	-1.0058	-1.6771
aMAL13P1.122_0	MAL13P1.122	1.646063	0.803323	0.084683	-1.0058	-1.6771
intMAL13P1.124_2	MAL13P1.124	-1.10481	-1.02364	-0.66822	0.392333	1.499413
aMAL13P1.124_2	MAL13P1.124	-1.10481	-1.02364	-0.66822	0.392333	1.499413
aMAL13P1.124_1	MAL13P1.124	-1.10481	-1.02364	-0.66822	0.392333	1.499413
aMAL13P1.124_0	MAL13P1.124	-1.10481	-1.02364	-0.66822	0.392333	1.499413
aMAL13P1.123_2	MAL13P1.124	-0.86407	-1.31963	-0.67341	0.54141	1.45496

aMAL13P1.123_1	MAL13P1.124	-0.86407	-1.31963	-0.67341	0.54141	1.45496
aMAL13P1.123_0	MAL13P1.124	-0.86407	-1.31963	-0.67341	0.54141	1.45496
intPF13_0126_2	MAL13P1.125	-0.82414	-0.67864	0.216435	0.816455	0.650185
intMAL13P1.125_2	MAL13P1.125	1.36379	1.43907	-0.25864	-1.15736	-0.75707
aMAL13P1.125_0	MAL13P1.125	1.36379	1.43907	-0.25864	-1.15736	-0.75707
intMAL13P1.126_2	MAL13P1.126	-0.80543	-1.17637	-1.31627	-0.04957	1.823033
aMAL13P1.126_1	MAL13P1.126	-0.80543	-1.17637	-1.31627	-0.04957	1.823033
aMAL13P1.126_0	MAL13P1.126	-0.80543	-1.17637	-1.31627	-0.04957	1.823033
intPF13_0127_2	MAL13P1.127	-0.17687	-0.62114	-1.79896	-0.84254	1.67914
intMAL13P1.128_2	MAL13P1.127	-0.97759	-1.36626	0.001923	1.155343	1.144523
aMAL13P1.127_0	MAL13P1.127	-0.40601	-0.80092	0.196837	0.758497	0.378257
intMAL13P1.127_2	MAL13P1.128	-0.40601	-0.80092	0.196837	0.758497	0.378257
aMAL13P1.128_0	MAL13P1.128	-0.97759	-1.36626	0.001923	1.155343	1.144523
intMAL13P1.129_2	MAL13P1.129	-0.63639	-0.53906	0.05811	0.57389	0.60706
aMAL13P1.129_0	MAL13P1.129	-0.63639	-0.53906	0.05811	0.57389	0.60706
intMAL13P1.13_2	MAL13P1.13	1.013633	0.253253	-0.16802	-0.28692	-0.29139
intMAL13P1.12_2	MAL13P1.13	0.542297	-0.1895	0.048017	0.039317	-0.33616
aMAL13P1.13_4	MAL13P1.13	1.013633	0.253253	-0.16802	-0.28692	-0.29139
aMAL13P1.13_3	MAL13P1.13	1.013633	0.253253	-0.16802	-0.28692	-0.29139
aMAL13P1.13_2	MAL13P1.13	1.013633	0.253253	-0.16802	-0.28692	-0.29139
aMAL13P1.13_1	MAL13P1.13	1.013633	0.253253	-0.16802	-0.28692	-0.29139
aMAL13P1.13_0	MAL13P1.13	1.013633	0.253253	-0.16802	-0.28692	-0.29139
intMAL13P1.130_2	MAL13P1.130	-0.68896	-0.76318	-1.84114	-0.88452	1.769517
aMAL13P1.130_0	MAL13P1.130	-0.68896	-0.76318	-1.84114	-0.88452	1.769517
intMAL13P1.131_2	MAL13P1.131	-1.02651	-1.59141	-0.55043	0.867267	1.591247
aMAL13P1.131_1	MAL13P1.131	-1.02651	-1.59141	-0.55043	0.867267	1.591247
aMAL13P1.131_0	MAL13P1.131	-1.02651	-1.59141	-0.55043	0.867267	1.591247
aMAL13P1.132_1	MAL13P1.132	0.654587	0.352067	0.034127	-0.17679	-0.31123
aMAL13P1.132_0	MAL13P1.132	0.654587	0.352067	0.034127	-0.17679	-0.31123
intMAL13P1.133_2	MAL13P1.133	0.61583	-0.09111	-0.06986	-0.10916	-0.30168
intMAL13P1.132_2	MAL13P1.133	0.654587	0.352067	0.034127	-0.17679	-0.31123
aMAL13P1.133_8	MAL13P1.133	0.61583	-0.09111	-0.06986	-0.10916	-0.30168
aMAL13P1.133_7	MAL13P1.133	0.61583	-0.09111	-0.06986	-0.10916	-0.30168
aMAL13P1.133_6	MAL13P1.133	0.61583	-0.09111	-0.06986	-0.10916	-0.30168
aMAL13P1.133_5	MAL13P1.133	0.61583	-0.09111	-0.06986	-0.10916	-0.30168
aMAL13P1.133_4	MAL13P1.133	0.61583	-0.09111	-0.06986	-0.10916	-0.30168
aMAL13P1.133_3	MAL13P1.133	0.61583	-0.09111	-0.06986	-0.10916	-0.30168
aMAL13P1.133_2	MAL13P1.133	0.61583	-0.09111	-0.06986	-0.10916	-0.30168
aMAL13P1.133_1	MAL13P1.133	0.61583	-0.09111	-0.06986	-0.10916	-0.30168
aMAL13P1.133_0	MAL13P1.133	0.61583	-0.09111	-0.06986	-0.10916	-0.30168
intPF13_0139_2	MAL13P1.134	-0.8338	-0.64968	0.006515	0.655265	0.815785
aMAL13P1.134_1	MAL13P1.134	-0.64691	-0.78246	-0.02209	0.698753	0.782623
aMAL13P1.134_0	MAL13P1.134	-0.64691	-0.78246	-0.02209	0.698753	0.782623
intMAL13P1.135_2	MAL13P1.135	0.322863	0.399693	-0.39139	-0.52295	0.091973
aMAL13P1.135_0	MAL13P1.135	0.322863	0.399693	-0.39139	-0.52295	0.091973
intMAL13P1.138_2	MAL13P1.137	-1.75503	-0.93231	0.889377	1.330457	0.328647
intMAL13P1.137_2	MAL13P1.137	-1.02033	-0.99767	-1.42741	-0.15479	2.05974

aMAL13P1.137_5	MAL13P1.137	-1.02033	-0.99767	-1.42741	-0.15479	2.05974
aMAL13P1.137_4	MAL13P1.137	-1.02033	-0.99767	-1.42741	-0.15479	2.05974
aMAL13P1.137_3	MAL13P1.137	-1.02033	-0.99767	-1.42741	-0.15479	2.05974
aMAL13P1.137_2	MAL13P1.137	-1.02033	-0.99767	-1.42741	-0.15479	2.05974
aMAL13P1.137_1	MAL13P1.137	-1.02033	-0.99767	-1.42741	-0.15479	2.05974
aMAL13P1.137_0	MAL13P1.137	-1.02033	-0.99767	-1.42741	-0.15479	2.05974
intMAL13P1.139_2	MAL13P1.138	-0.7578	-1.10802	-0.52603	0.375932	1.042212
aMAL13P1.138_1	MAL13P1.138	-1.75503	-0.93231	0.889377	1.330457	0.328647
aMAL13P1.138_0	MAL13P1.138	-1.75503	-0.93231	0.889377	1.330457	0.328647
aMAL13P1.139_0	MAL13P1.139	-0.7578	-1.10802	-0.52603	0.375932	1.042212
intPF13_0013_2	MAL13P1.14	0.976697	0.150037	0.367927	-0.04809	-0.9722
intMAL13P1.14_3	MAL13P1.14	0.777765	0.771505	0.699495	-0.31005	-1.4924
aMAL13P1.14_2	MAL13P1.14	0.777765	0.771505	0.699495	-0.31005	-1.4924
aMAL13P1.14_1	MAL13P1.14	0.777765	0.771505	0.699495	-0.31005	-1.4924
aMAL13P1.14_0	MAL13P1.14	0.777765	0.771505	0.699495	-0.31005	-1.4924
intMAL13P1.140_2	MAL13P1.140	-1.00588	-0.637	-0.96468	-0.00024	1.678832
aMAL13P1.140_4	MAL13P1.140	-1.00588	-0.637	-0.96468	-0.00024	1.678832
aMAL13P1.140_3	MAL13P1.140	-1.00588	-0.637	-0.96468	-0.00024	1.678832
aMAL13P1.140_2	MAL13P1.140	-1.00588	-0.637	-0.96468	-0.00024	1.678832
aMAL13P1.140_1	MAL13P1.140	-1.00588	-0.637	-0.96468	-0.00024	1.678832
aMAL13P1.140_0	MAL13P1.140	-1.00588	-0.637	-0.96468	-0.00024	1.678832
intMAL13P1.142_2	MAL13P1.141	-0.10225	0.035267	0.373957	0.267377	-0.22693
aMAL13P1.141_0	MAL13P1.141	-1.18334	-1.6433	-0.81645	0.63013	1.74094
intMAL13P1.141_2	MAL13P1.142	-1.18334	-1.6433	-0.81645	0.63013	1.74094
aMAL13P1.142_0	MAL13P1.142	-0.10225	0.035267	0.373957	0.267377	-0.22693
intMAL13P1.144_2	MAL13P1.144	0.381097	0.504007	0.598247	0.160087	-0.64767
aMAL13P1.144_1	MAL13P1.144	0.381097	0.504007	0.598247	0.160087	-0.64767
aMAL13P1.144_0	MAL13P1.144	0.381097	0.504007	0.598247	0.160087	-0.64767
intMAL13P1.145_2	MAL13P1.145	-0.48501	0.005038	-0.12794	0.056358	0.484128
aMAL13P1.145_3	MAL13P1.145	-0.48501	0.005038	-0.12794	0.056358	0.484128
aMAL13P1.145_2	MAL13P1.145	-0.48501	0.005038	-0.12794	0.056358	0.484128
aMAL13P1.145_1	MAL13P1.145	-0.48501	0.005038	-0.12794	0.056358	0.484128
aMAL13P1.145_0	MAL13P1.145	-0.48501	0.005038	-0.12794	0.056358	0.484128
intMAL13P1.146_2	MAL13P1.146	1.259907	0.222447	0.138357	-0.15152	-0.75261
aMAL13P1.146_1	MAL13P1.146	1.259907	0.222447	0.138357	-0.15152	-0.75261
aMAL13P1.146_0	MAL13P1.146	1.259907	0.222447	0.138357	-0.15152	-0.75261
intMAL13P1.147_2	MAL13P1.147	0.372028	-0.45016	-0.7931	-0.21358	0.709528
aMAL13P1.147_0	MAL13P1.147	0.372028	-0.45016	-0.7931	-0.21358	0.709528
intMAL13P1.148_2	MAL13P1.148	-0.96299	-0.99555	0.014988	0.503928	0.385718
aMAL13P1.148_3	MAL13P1.148	-0.96299	-0.99555	0.014988	0.503928	0.385718
aMAL13P1.148_2	MAL13P1.148	-0.96299	-0.99555	0.014988	0.503928	0.385718
aMAL13P1.148_1	MAL13P1.148	-0.96299	-0.99555	0.014988	0.503928	0.385718
aMAL13P1.148_0	MAL13P1.148	-0.96299	-0.99555	0.014988	0.503928	0.385718
intMAL13P1.149_2	MAL13P1.149					
aMAL13P1.149_0	MAL13P1.149					
intPF13_0016_2	MAL13P1.15	-0.82968	-0.4466	0.339717	0.778617	0.523937
aMAL13P1.15_0	MAL13P1.15	-0.76008	-0.7196	0.36721	0.95279	0.5296

intMAL13P1.150_2	MAL13P1.150	0.66864	0.65181	0.76214	-0.02314	-1.22031
aMAL13P1.150_1	MAL13P1.150	0.66864	0.65181	0.76214	-0.02314	-1.22031
aMAL13P1.150_0	MAL13P1.150	0.66864	0.65181	0.76214	-0.02314	-1.22031
intMAL13P1.151_2	MAL13P1.151	-1.00155	-0.89724	-0.38325	0.552837	1.267577
aMAL13P1.151_3	MAL13P1.151	-1.00155	-0.89724	-0.38325	0.552837	1.267577
aMAL13P1.151_2	MAL13P1.151	-1.00155	-0.89724	-0.38325	0.552837	1.267577
aMAL13P1.151_1	MAL13P1.151	-1.00155	-0.89724	-0.38325	0.552837	1.267577
aMAL13P1.151_0	MAL13P1.151	-1.00155	-0.89724	-0.38325	0.552837	1.267577
intPF13_0160_2	MAL13P1.152	-1.05155	-1.76525	-0.95752	0.605183	1.836913
intMAL13P1.152_2	MAL13P1.152	-0.16893	-0.55483	-1.48657	-0.71009	1.346667
aMAL13P1.152_1	MAL13P1.152	-0.16893	-0.55483	-1.48657	-0.71009	1.346667
aMAL13P1.152_0	MAL13P1.152	-0.16893	-0.55483	-1.48657	-0.71009	1.346667
aMAL13P1.153_0	MAL13P1.153	1.096703	0.684813	0.442053	-0.35261	-1.24837
intMAL13P1.155_2	MAL13P1.154	0.422713	0.646933	0.640393	0.049273	-0.80977
intMAL13P1.154_2	MAL13P1.154	0.543493	-0.06748	0.188463	0.137203	-0.36586
intMAL13P1.153_2	MAL13P1.154	1.096703	0.684813	0.442053	-0.35261	-1.24837
aMAL13P1.154_0	MAL13P1.154	0.543493	-0.06748	0.188463	0.137203	-0.36586
aMAL13P1.155_1	MAL13P1.155	0.422713	0.646933	0.640393	0.049273	-0.80977
aMAL13P1.155_0	MAL13P1.155	0.422713	0.646933	0.640393	0.049273	-0.80977
intMAL13P1.156_2	MAL13P1.156	1.117753	0.286753	-1.0675	-1.63584	-0.67809
aMAL13P1.156_1	MAL13P1.156	1.117753	0.286753	-1.0675	-1.63584	-0.67809
intMAL13P1.158_2	MAL13P1.157	0.18199	-0.28146	0.46117	0.36183	-0.55104
intMAL13P1.157_2	MAL13P1.157	0.42361	-0.21407	0.24563	-0.07613	-0.84843
aMAL13P1.157_1	MAL13P1.157	0.42361	-0.21407	0.24563	-0.07613	-0.84843
aMAL13P1.157_0	MAL13P1.157	0.42361	-0.21407	0.24563	-0.07613	-0.84843
intMAL13P1.159_2	MAL13P1.158	0.074873	0.277653	0.129193	-0.05553	-0.15999
aMAL13P1.158_1	MAL13P1.158	0.18199	-0.28146	0.46117	0.36183	-0.55104
aMAL13P1.158_0	MAL13P1.158	0.18199	-0.28146	0.46117	0.36183	-0.55104
intMAL13P1.160_2	MAL13P1.159	-0.53269	-0.72352	-0.44794	0.372277	1.074857
aMAL13P1.159_0	MAL13P1.159	0.074873	0.277653	0.129193	-0.05553	-0.15999
intPF13_0017_2	MAL13P1.16	0.505133	0.572343	0.623813	0.095853	-0.78668
intMAL13P1.16_3	MAL13P1.16	-0.70225	-0.71873	0.20346	0.86254	0.67573
aMAL13P1.16_0	MAL13P1.16	-0.70225	-0.71873	0.20346	0.86254	0.67573
intMAL13P1.161_2	MAL13P1.160	-0.4079	-1.00949	-0.439	0.541337	1.079827
aMAL13P1.160_0	MAL13P1.160	-0.53269	-0.72352	-0.44794	0.372277	1.074857
aMAL13P1.161_0	MAL13P1.161	-0.4079	-1.00949	-0.439	0.541337	1.079827
intMAL13P1.162_2	MAL13P1.162	-0.15935	0.079787	0.379427	0.297907	-0.14245
aMAL13P1.162_0	MAL13P1.162	-0.15935	0.079787	0.379427	0.297907	-0.14245
aMAL13P1.163_0	MAL13P1.163-a	-0.1226	0.099997	-0.57699	-0.24667	0.795337
intMAL13P1.163_2	MAL13P1.163-b	-0.1226	0.099997	-0.57699	-0.24667	0.795337
intMAL13P1.165_2	MAL13P1.164	-0.67392	-0.92489	-0.30423	0.56573	1.01439
aMAL13P1.164_1	MAL13P1.164	-1.36827	-1.03761	0.389977	1.229357	0.893447
aMAL13P1.164_0	MAL13P1.164	-1.36827	-1.03761	0.389977	1.229357	0.893447
intMAL13P1.164_2	MAL13P1.165	-1.36827	-1.03761	0.389977	1.229357	0.893447
aMAL13P1.165_1	MAL13P1.165	-0.67392	-0.92489	-0.30423	0.56573	1.01439
aMAL13P1.165_0	MAL13P1.165	-0.67392	-0.92489	-0.30423	0.56573	1.01439
intPF13_0167_2	MAL13P1.166	0.599077	0.409117	0.270027	-0.21119	-0.73878

intMAL13P1.166_3	MAL13P1.166	0.234627	0.288347	-0.04019	-0.45847	-0.50001
aMAL13P1.166_1	MAL13P1.166	0.234627	0.288347	-0.04019	-0.45847	-0.50001
aMAL13P1.166_0	MAL13P1.166	0.234627	0.288347	-0.04019	-0.45847	-0.50001
intPF13_0168_2	MAL13P1.167	-0.12102	-0.18527	-0.77939	-0.43336	0.695648
intMAL13P1.167_3	MAL13P1.167	-1.12783	-1.40727	-0.36167	0.907875	1.462625
aMAL13P1.167_0	MAL13P1.167	-1.12783	-1.40727	-0.36167	0.907875	1.462625
aMAL13P1.168_0	MAL13P1.168	-0.80793	-2.06236	-1.01279	0.94479	2.16836
intMAL13P1.169_2	MAL13P1.169	0.05399	-0.00794	-0.08512	-0.13388	-0.06806
aMAL13P1.169_0	MAL13P1.169	0.05399	-0.00794	-0.08512	-0.13388	-0.06806
intMAL13P1.17_2	MAL13P1.17	0.079087	0.238567	0.120877	-0.17754	-0.35523
aMAL13P1.17_0	MAL13P1.17	0.079087	0.238567	0.120877	-0.17754	-0.35523
intMAL13P1.170_2	MAL13P1.170	0.277637	0.639297	0.468407	-0.10257	-0.67896
aMAL13P1.170_1	MAL13P1.170	0.277637	0.639297	0.468407	-0.10257	-0.67896
aMAL13P1.170_0	MAL13P1.170	0.277637	0.639297	0.468407	-0.10257	-0.67896
intMAL13P1.172_2	MAL13P1.171	0.568033	0.594823	0.077353	-0.22969	-0.27916
intMAL13P1.171_2	MAL13P1.171	-0.16681	0.19307	0.35337	0.29163	-0.02607
aMAL13P1.171_0	MAL13P1.171	-0.16681	0.19307	0.35337	0.29163	-0.02607
intPF13_0183_2	MAL13P1.172	-0.10419	-0.44453	0.79481	0.89219	-0.38047
aMAL13P1.172_0	MAL13P1.172	0.568033	0.594823	0.077353	-0.22969	-0.27916
intMAL13P1.173_2	MAL13P1.173	-0.74546	-0.44672	-0.89356	-0.10744	1.42772
aMAL13P1.173_0	MAL13P1.173	-0.74546	-0.44672	-0.89356	-0.10744	1.42772
intPF13_0194_1	MAL13P1.174	1.669527	1.153357	-0.07472	-1.21494	-1.47302
intMAL13P1.174_2	MAL13P1.174	-0.28533	-0.60137	-0.9333	0.07764	1.52169
aMAL13P1.174_0	MAL13P1.174	-0.28533	-0.60137	-0.9333	0.07764	1.52169
intMAL13P1.176_2	MAL13P1.176	1.091557	0.332627	-1.795	-2.32016	-0.33149
aMAL13P1.176_4	MAL13P1.176	1.091557	0.332627	-1.795	-2.32016	-0.33149
intMAL13P1.177_2	MAL13P1.177	-0.40213	-0.15739	-0.33823	-0.09211	0.511053
aMAL13P1.177_1	MAL13P1.177	-0.40213	-0.15739	-0.33823	-0.09211	0.511053
aMAL13P1.177_0	MAL13P1.177	-0.40213	-0.15739	-0.33823	-0.09211	0.511053
intMAL13P1.178_2	MAL13P1.178	0.89529	0.98855	-0.06985	-0.57015	-0.36655
aMAL13P1.178_1	MAL13P1.178	0.89529	0.98855	-0.06985	-0.57015	-0.36655
aMAL13P1.178_0	MAL13P1.178	0.89529	0.98855	-0.06985	-0.57015	-0.36655
intPF13_0202_2	MAL13P1.179	-0.99392	-1.08737	-0.23874	0.789077	1.227707
intMAL13P1.179_1	MAL13P1.179	0.505817	0.374407	0.199447	-0.20261	-0.58857
aMAL13P1.179_1	MAL13P1.179	0.505817	0.374407	0.199447	-0.20261	-0.58857
aMAL13P1.179_0	MAL13P1.179	0.505817	0.374407	0.199447	-0.20261	-0.58857
intMAL13P1.18_2	MAL13P1.18	-0.43189	-0.62901	-1.47996	-0.4547	1.755847
aMAL13P1.18_1	MAL13P1.18	-0.43189	-0.62901	-1.47996	-0.4547	1.755847
aMAL13P1.18_0	MAL13P1.18	-0.43189	-0.62901	-1.47996	-0.4547	1.755847
intMAL13P1.181_2	MAL13P1.180	-0.53859	-0.68338	0.002858	0.515938	0.492628
intMAL13P1.180_2	MAL13P1.180	-1.03674	-0.71829	0.137577	0.784757	0.791627
aMAL13P1.180_0	MAL13P1.180	-1.03674	-0.71829	0.137577	0.784757	0.791627
aMAL13P1.181_0	MAL13P1.181	-0.53859	-0.68338	0.002858	0.515938	0.492628
intMAL13P1.183_2	MAL13P1.182	-0.31972	-0.75141	0.193813	0.727853	0.342073
aMAL13P1.182_0	MAL13P1.182	0.43727	-0.38635	-0.5648	-0.1672	0.38135
intMAL13P1.182_2	MAL13P1.183	0.43727	-0.38635	-0.5648	-0.1672	0.38135
aMAL13P1.183_0	MAL13P1.183	-0.31972	-0.75141	0.193813	0.727853	0.342073

intPF13_0205_2	MAL13P1.184	0.247553	0.602733	0.618783	0.120383	-0.63457
intMAL13P1.184_2	MAL13P1.184	-0.71373	0.337113	1.212773	0.669893	-0.75595
aMAL13P1.184_1	MAL13P1.184	-0.71373	0.337113	1.212773	0.669893	-0.75595
aMAL13P1.184_0	MAL13P1.184	-0.71373	0.337113	1.212773	0.669893	-0.75595
intMAL13P1.186_2	MAL13P1.185	0.129527	0.461377	0.278987	0.127347	-0.05604
intMAL13P1.185_2	MAL13P1.185	-1.42083	0.04115	1.49721	1.18779	-0.46215
aMAL13P1.185_0	MAL13P1.185	-1.42083	0.04115	1.49721	1.18779	-0.46215
aMAL13P1.186_1	MAL13P1.186	0.129527	0.461377	0.278987	0.127347	-0.05604
aMAL13P1.186_0	MAL13P1.186	0.129527	0.461377	0.278987	0.127347	-0.05604
aMAL13P1.188_1	MAL13P1.188	0.575107	-0.32352	-2.1282	-1.55196	1.166357
aMAL13P1.188_0	MAL13P1.188	0.575107	-0.32352	-2.1282	-1.55196	1.166357
intMAL13P1.189_2	MAL13P1.189	-0.50106	-0.27521	-0.2491	0.159763	0.679873
aMAL13P1.189_1	MAL13P1.189	-0.50106	-0.27521	-0.2491	0.159763	0.679873
aMAL13P1.189_0	MAL13P1.189	-0.50106	-0.27521	-0.2491	0.159763	0.679873
intMAL13P1.19_2	MAL13P1.19	-1.11481	-1.26356	-0.32163	0.857628	1.405338
aMAL13P1.19_9	MAL13P1.19	-1.11481	-1.26356	-0.32163	0.857628	1.405338
aMAL13P1.19_8	MAL13P1.19	-1.11481	-1.26356	-0.32163	0.857628	1.405338
aMAL13P1.19_7	MAL13P1.19	-1.11481	-1.26356	-0.32163	0.857628	1.405338
aMAL13P1.19_6	MAL13P1.19	-1.11481	-1.26356	-0.32163	0.857628	1.405338
aMAL13P1.19_5	MAL13P1.19	-1.11481	-1.26356	-0.32163	0.857628	1.405338
aMAL13P1.19_4	MAL13P1.19	-1.11481	-1.26356	-0.32163	0.857628	1.405338
aMAL13P1.19_3	MAL13P1.19	-1.11481	-1.26356	-0.32163	0.857628	1.405338
aMAL13P1.19_2	MAL13P1.19	-1.11481	-1.26356	-0.32163	0.857628	1.405338
aMAL13P1.19_13	MAL13P1.19	-1.11481	-1.26356	-0.32163	0.857628	1.405338
aMAL13P1.19_12	MAL13P1.19	-1.11481	-1.26356	-0.32163	0.857628	1.405338
aMAL13P1.19_11	MAL13P1.19	-1.11481	-1.26356	-0.32163	0.857628	1.405338
aMAL13P1.19_10	MAL13P1.19	-1.11481	-1.26356	-0.32163	0.857628	1.405338
aMAL13P1.19_1	MAL13P1.19	-1.11481	-1.26356	-0.32163	0.857628	1.405338
aMAL13P1.19_0	MAL13P1.19	-1.11481	-1.26356	-0.32163	0.857628	1.405338
intMAL13P1.190_3	MAL13P1.190	-0.83636	-0.42918	-0.12888	0.451717	0.906517
aMAL13P1.190_1	MAL13P1.190	-0.83636	-0.42918	-0.12888	0.451717	0.906517
aMAL13P1.190_0	MAL13P1.190	-0.83636	-0.42918	-0.12888	0.451717	0.906517
intMAL13P1.192_2	MAL13P1.191	-0.29395	-0.84675	0.438267	0.900067	0.080087
intMAL13P1.191_2	MAL13P1.191	-0.56304	-0.55181	0.098873	0.502293	0.391973
aMAL13P1.191_1	MAL13P1.191	-0.56304	-0.55181	0.098873	0.502293	0.391973
aMAL13P1.191_0	MAL13P1.191	-0.56304	-0.55181	0.098873	0.502293	0.391973
aMAL13P1.192_1	MAL13P1.192	-0.29395	-0.84675	0.438267	0.900067	0.080087
aMAL13P1.192_0	MAL13P1.192	-0.29395	-0.84675	0.438267	0.900067	0.080087
intMAL13P1.193_2	MAL13P1.193	-0.115	-0.45746	-0.22108	0.085417	0.266797
aMAL13P1.193_1	MAL13P1.193	-0.115	-0.45746	-0.22108	0.085417	0.266797
aMAL13P1.193_0	MAL13P1.193	-0.115	-0.45746	-0.22108	0.085417	0.266797
intMAL13P1.194_2	MAL13P1.194	-0.86841	-1.00896	-0.35734	0.58934	1.15596
aMAL13P1.194_1	MAL13P1.194	-0.86841	-1.00896	-0.35734	0.58934	1.15596
aMAL13P1.194_0	MAL13P1.194	-0.86841	-1.00896	-0.35734	0.58934	1.15596
aMAL13P1.195_1	MAL13P1.195	0.115523	-0.25864	0.346273	0.417393	-0.2197
aMAL13P1.195_0	MAL13P1.195	0.115523	-0.25864	0.346273	0.417393	-0.2197
intMAL13P1.196_2	MAL13P1.196	-0.68976	-0.45667	-0.34966	0.289997	1.001507

intMAL13P1.195_2	MAL13P1.196	0.115523	-0.25864	0.346273	0.417393	-0.2197
aMAL13P1.196_1	MAL13P1.196	-0.68976	-0.45667	-0.34966	0.289997	1.001507
aMAL13P1.196_0	MAL13P1.196	-0.68976	-0.45667	-0.34966	0.289997	1.001507
intMAL13P1.197_2	MAL13P1.197	0.712117	1.139427	-0.34535	-0.99231	-0.33309
aMAL13P1.197_0	MAL13P1.197	0.712117	1.139427	-0.34535	-0.99231	-0.33309
intMAL13P1.20_2	MAL13P1.20	0.257793	0.453013	-2.67E-05	-0.29431	-0.22535
aMAL13P1.20_0	MAL13P1.20	0.257793	0.453013	-2.67E-05	-0.29431	-0.22535
intPF13_0216_2	MAL13P1.200	-0.96972	-1.04737	-0.56076	0.375097	1.215707
intMAL13P1.201_2	MAL13P1.200	-0.44123	0.507153	0.336203	-0.05054	-0.20699
aMAL13P1.200_0	MAL13P1.200	-0.25619	-0.00214	0.763158	0.702218	-0.26183
intMAL13P1.200_2	MAL13P1.201	-0.25619	-0.00214	0.763158	0.702218	-0.26183
aMAL13P1.201_1	MAL13P1.201	-0.44123	0.507153	0.336203	-0.05054	-0.20699
aMAL13P1.201_0	MAL13P1.201	-0.44123	0.507153	0.336203	-0.05054	-0.20699
intMAL13P1.202_2	MAL13P1.202	-0.91566	-0.86572	-1.30382	-0.08769	1.986885
aMAL13P1.202_2	MAL13P1.202	-0.91566	-0.86572	-1.30382	-0.08769	1.986885
aMAL13P1.202_1	MAL13P1.202	-0.91566	-0.86572	-1.30382	-0.08769	1.986885
aMAL13P1.202_0	MAL13P1.202	-0.91566	-0.86572	-1.30382	-0.08769	1.986885
intMAL13P1.203_2	MAL13P1.203	0.867723	0.852903	-0.04205	-0.65079	-0.61974
aMAL13P1.203_1	MAL13P1.203	0.867723	0.852903	-0.04205	-0.65079	-0.61974
aMAL13P1.203_0	MAL13P1.203	0.867723	0.852903	-0.04205	-0.65079	-0.61974
intMAL13P1.204_2	MAL13P1.204	1.170017	0.285917	0.261657	-0.12832	-0.87158
aMAL13P1.204_0	MAL13P1.204	1.170017	0.285917	0.261657	-0.12832	-0.87158
aMAL13P1.205_0	MAL13P1.205	-0.19137	-0.66715	-0.78719	0.083527	1.187487
intMAL13P1.206_2	MAL13P1.206	0.262603	0.538983	0.022533	-0.44787	-0.44132
aMAL13P1.206_1	MAL13P1.206	0.262603	0.538983	0.022533	-0.44787	-0.44132
aMAL13P1.206_0	MAL13P1.206	0.262603	0.538983	0.022533	-0.44787	-0.44132
intMAL13P1.209_3	MAL13P1.209	0.082595	0.486175	0.668325	0.210775	-0.61253
aMAL13P1.209_0	MAL13P1.209	0.082595	0.486175	0.668325	0.210775	-0.61253
intMAL13P1.21_2	MAL13P1.21	-0.72263	-1.15832	-0.65085	0.432187	1.310157
aMAL13P1.21_1	MAL13P1.21	-0.72263	-1.15832	-0.65085	0.432187	1.310157
aMAL13P1.21_0	MAL13P1.21	-0.72263	-1.15832	-0.65085	0.432187	1.310157
intMAL13P1.211_2	MAL13P1.210	0.110193	-0.03598	0.056433	-0.09899	-0.31267
intMAL13P1.210_2	MAL13P1.210	-0.56519	-0.82057	-0.04212	0.660783	0.709733
aMAL13P1.210_1	MAL13P1.210	-0.56519	-0.82057	-0.04212	0.660783	0.709733
aMAL13P1.210_0	MAL13P1.210	-0.56519	-0.82057	-0.04212	0.660783	0.709733
aMAL13P1.211_0	MAL13P1.211	0.110193	-0.03598	0.056433	-0.09899	-0.31267
intMAL13P1.212_2	MAL13P1.212	1.775387	0.644077	-0.87815	-1.70751	-1.11374
aMAL13P1.212_0	MAL13P1.212	1.775387	0.644077	-0.87815	-1.70751	-1.11374
intPF13_0234_2	MAL13P1.213	-0.81986	-0.17621	1.610163	1.213003	-0.97663
intMAL13P1.213_2	MAL13P1.213	0.570483	0.241603	0.670303	0.069363	-1.15394
aMAL13P1.213_0	MAL13P1.213	0.570483	0.241603	0.670303	0.069363	-1.15394
intMAL13P1.214_2	MAL13P1.214	0.2129	-0.19152	-0.84845	-0.15555	1.12352
aMAL13P1.214_0	MAL13P1.214	0.2129	-0.19152	-0.84845	-0.15555	1.12352
intMAL13P1.215_2	MAL13P1.215	-0.27978	-1.24307	-1.10785	-0.08081	1.174407
aMAL13P1.215_0	MAL13P1.215	-0.27978	-1.24307	-1.10785	-0.08081	1.174407
intMAL13P1.216_2	MAL13P1.216	1.01073	0.42369	-0.46821	-0.58301	-0.07058
aMAL13P1.216_2	MAL13P1.216	1.01073	0.42369	-0.46821	-0.58301	-0.07058

aMAL13P1.216_1	MAL13P1.216	1.01073	0.42369	-0.46821	-0.58301	-0.07058
aMAL13P1.216_0	MAL13P1.216	1.01073	0.42369	-0.46821	-0.58301	-0.07058
intMAL13P1.217_2	MAL13P1.217					
aMAL13P1.217_0	MAL13P1.217					
intMAL13P1.218_2	MAL13P1.218	-0.22533	0.11514	0.66623	0.54627	-0.25014
aMAL13P1.218_1	MAL13P1.218	-0.22533	0.11514	0.66623	0.54627	-0.25014
aMAL13P1.218_0	MAL13P1.218	-0.22533	0.11514	0.66623	0.54627	-0.25014
intMAL13P1.220_2	MAL13P1.219	-0.19833	-0.41639	-0.52599	-0.08785	0.621053
aMAL13P1.219_0	MAL13P1.219	-0.19363	-1.06685	-1.22631	-0.03803	1.544513
intMAL13P1.22_2	MAL13P1.22	-0.93919	-1.31705	-0.45441	0.521073	1.049713
aMAL13P1.22_1	MAL13P1.22	-0.93919	-1.31705	-0.45441	0.521073	1.049713
aMAL13P1.22_0	MAL13P1.22	-0.93919	-1.31705	-0.45441	0.521073	1.049713
intMAL13P1.219_2	MAL13P1.220	-0.19363	-1.06685	-1.22631	-0.03803	1.544513
aMAL13P1.220_1	MAL13P1.220	-0.19833	-0.41639	-0.52599	-0.08785	0.621053
aMAL13P1.220_0	MAL13P1.220	-0.19833	-0.41639	-0.52599	-0.08785	0.621053
intPF13_0239_2	MAL13P1.221	0.502037	0.622297	0.019647	-0.34231	-0.30196
intMAL13P1.221_2	MAL13P1.221	-0.29992	0.139573	0.970103	0.670063	-0.58041
aMAL13P1.221_1	MAL13P1.221	-0.29992	0.139573	0.970103	0.670063	-0.58041
aMAL13P1.221_0	MAL13P1.221	-0.29992	0.139573	0.970103	0.670063	-0.58041
intMAL13P1.222_2	MAL13P1.222	0.602245	1.061765	0.707975	-0.41453	-1.41266
aMAL13P1.222_2	MAL13P1.222	0.602245	1.061765	0.707975	-0.41453	-1.41266
aMAL13P1.222_1	MAL13P1.222	0.602245	1.061765	0.707975	-0.41453	-1.41266
aMAL13P1.222_0	MAL13P1.222	0.602245	1.061765	0.707975	-0.41453	-1.41266
intMAL13P1.224_2	MAL13P1.224	-0.25238	0.063907	0.152397	0.187777	0.128997
aMAL13P1.224_0	MAL13P1.224	-0.25238	0.063907	0.152397	0.187777	0.128997
aMAL13P1.223_0	MAL13P1.224	-0.17618	-0.06706	0.039637	0.130697	0.137397
intMAL13P1.225_2	MAL13P1.225	0.381423	0.394893	0.765703	-0.21304	-1.58723
aMAL13P1.225_0	MAL13P1.225	0.381423	0.394893	0.765703	-0.21304	-1.58723
aMAL13P1.226_2	MAL13P1.226	-0.9496	-0.73154	-0.40819	0.381353	1.149203
aMAL13P1.226_1	MAL13P1.226	-0.9496	-0.73154	-0.40819	0.381353	1.149203
aMAL13P1.226_0	MAL13P1.226	-0.9496	-0.73154	-0.40819	0.381353	1.149203
intMAL13P1.227_2	MAL13P1.227	-0.74993	-0.48692	-0.01644	0.491917	0.693397
intMAL13P1.226_2	MAL13P1.227	-0.9496	-0.73154	-0.40819	0.381353	1.149203
aMAL13P1.227_0	MAL13P1.227	-0.74993	-0.48692	-0.01644	0.491917	0.693397
intMAL13P1.228_2	MAL13P1.228	-0.05084	-0.54982	-1.6541	-1.0424	1.11932
aMAL13P1.228_1	MAL13P1.228	-0.05084	-0.54982	-1.6541	-1.0424	1.11932
aMAL13P1.228_0	MAL13P1.228	-0.05084	-0.54982	-1.6541	-1.0424	1.11932
intMAL13P1.229_2	MAL13P1.229	-0.96975	-0.65727	-0.10596	0.62796	1.02127
aMAL13P1.229_0	MAL13P1.229	-0.96975	-0.65727	-0.10596	0.62796	1.02127
intMAL13P1.23_2	MAL13P1.23	0.702597	-0.22399	-0.631	-0.85866	-0.51467
intMAL13P1.230_2	MAL13P1.230	-0.09266	0.301307	0.096897	-0.14206	-0.15897
aMAL13P1.230_1	MAL13P1.230	-0.09266	0.301307	0.096897	-0.14206	-0.15897
aMAL13P1.230_0	MAL13P1.230	-0.09266	0.301307	0.096897	-0.14206	-0.15897
intMAL13P1.231_2	MAL13P1.231	-0.57467	-0.10819	0.221123	0.478543	0.431853
aMAL13P1.231_1	MAL13P1.231	-0.57467	-0.10819	0.221123	0.478543	0.431853
aMAL13P1.231_0	MAL13P1.231	-0.57467	-0.10819	0.221123	0.478543	0.431853
intMAL13P1.232_2	MAL13P1.232	0.725707	0.810447	0.812107	-0.12377	-1.37611

aMAL13P1.232_0	MAL13P1.232	0.725707	0.810447	0.812107	-0.12377	-1.37611
intMAL13P1.233_1	MAL13P1.233	0.49516	0.44419	0.37763	-0.09863	-0.70919
aMAL13P1.233_0	MAL13P1.233	0.49516	0.44419	0.37763	-0.09863	-0.70919
intMAL13P1.234_2	MAL13P1.234	-0.11069	0.029027	-0.54388	-0.33342	0.553227
aMAL13P1.234_8	MAL13P1.234	-0.11069	0.029027	-0.54388	-0.33342	0.553227
aMAL13P1.234_7	MAL13P1.234	-0.11069	0.029027	-0.54388	-0.33342	0.553227
aMAL13P1.234_6	MAL13P1.234	-0.11069	0.029027	-0.54388	-0.33342	0.553227
aMAL13P1.234_5	MAL13P1.234	-0.11069	0.029027	-0.54388	-0.33342	0.553227
aMAL13P1.234_4	MAL13P1.234	-0.11069	0.029027	-0.54388	-0.33342	0.553227
aMAL13P1.234_3	MAL13P1.234	-0.11069	0.029027	-0.54388	-0.33342	0.553227
aMAL13P1.234_2	MAL13P1.234	-0.11069	0.029027	-0.54388	-0.33342	0.553227
aMAL13P1.234_1	MAL13P1.234	-0.11069	0.029027	-0.54388	-0.33342	0.553227
aMAL13P1.234_0	MAL13P1.234	-0.11069	0.029027	-0.54388	-0.33342	0.553227
intMAL13P1.235_3	MAL13P1.235	-0.57479	-0.72405	0.0031	0.7079	0.76505
aMAL13P1.235_0	MAL13P1.235	-0.57479	-0.72405	0.0031	0.7079	0.76505
intMAL13P1.236_2	MAL13P1.236	0.15973	0.57175	0.48895	0.12935	-0.37445
aMAL13P1.236_0	MAL13P1.236	0.15973	0.57175	0.48895	0.12935	-0.37445
aMAL13P1.237_1	MAL13P1.237	0.365167	0.285677	0.411397	0.035437	-0.63234
aMAL13P1.237_0	MAL13P1.237	0.365167	0.285677	0.411397	0.035437	-0.63234
aMAL13P1.238_0	MAL13P1.238	0.87593	-0.17466	-1.06967	-0.95733	0.11666
aMAL13P1.239_2	MAL13P1.239	-0.88477	-1.04332	0.092713	0.938953	0.837813
aMAL13P1.239_1	MAL13P1.239	-0.88477	-1.04332	0.092713	0.938953	0.837813
aMAL13P1.239_0	MAL13P1.239	-0.88477	-1.04332	0.092713	0.938953	0.837813
aMAL13P1.23_1	MAL13P1.23-a	0.702597	-0.22399	-0.631	-0.85866	-0.51467
aMAL13P1.23_0	MAL13P1.23-a	0.702597	-0.22399	-0.631	-0.85866	-0.51467
intMAL13P1.24_2	MAL13P1.24	-0.20053	-0.28486	-0.01118	0.151925	0.125305
aMAL13P1.24_0	MAL13P1.24	-0.20053	-0.28486	-0.01118	0.151925	0.125305
intMAL13P1.241_2	MAL13P1.240	-0.08056	-0.64918	-0.99813	-0.32187	0.94118
intMAL13P1.239_2	MAL13P1.240	-0.88477	-1.04332	0.092713	0.938953	0.837813
aMAL13P1.240_1	MAL13P1.240	-0.428	-0.38801	-0.70348	-0.19736	0.877673
aMAL13P1.240_0	MAL13P1.240	-0.428	-0.38801	-0.70348	-0.19736	0.877673
intMAL13P1.242_2	MAL13P1.241	1.160573	0.241123	-0.05075	-0.30759	-0.59446
intMAL13P1.240_2	MAL13P1.241	-0.428	-0.38801	-0.70348	-0.19736	0.877673
aMAL13P1.241_0	MAL13P1.241	-0.08056	-0.64918	-0.99813	-0.32187	0.94118
aMAL13P1.242_0	MAL13P1.242	1.160573	0.241123	-0.05075	-0.30759	-0.59446
intMAL13P1.243_2	MAL13P1.243	0.822663	1.072663	0.634833	-0.26039	-1.11356
aMAL13P1.243_2	MAL13P1.243	0.822663	1.072663	0.634833	-0.26039	-1.11356
aMAL13P1.243_1	MAL13P1.243	0.822663	1.072663	0.634833	-0.26039	-1.11356
aMAL13P1.243_0	MAL13P1.243	0.822663	1.072663	0.634833	-0.26039	-1.11356
intMAL13P1.244_2	MAL13P1.244	-0.68504	-1.22076	-0.15797	0.813307	0.924097
aMAL13P1.244_0	MAL13P1.244	-0.68504	-1.22076	-0.15797	0.813307	0.924097
aMAL13P1.245_1	MAL13P1.245	0.454833	-0.00417	-0.11017	-0.32717	-0.44317
aMAL13P1.245_0	MAL13P1.245	0.454833	-0.00417	-0.11017	-0.32717	-0.44317
intMAL13P1.246_2	MAL13P1.246	-0.09591	0.056587	0.208337	-0.16	-0.55225
intMAL13P1.245_2	MAL13P1.246	0.454833	-0.00417	-0.11017	-0.32717	-0.44317
aMAL13P1.246_2	MAL13P1.246	-0.09591	0.056587	0.208337	-0.16	-0.55225
aMAL13P1.246_1	MAL13P1.246	-0.09591	0.056587	0.208337	-0.16	-0.55225

aMAL13P1.246_0	MAL13P1.246	-0.09591	0.056587	0.208337	-0.16	-0.55225
intPF13_0256_2	MAL13P1.247	0.851073	0.763643	0.170213	-0.31955	-0.56598
aMAL13P1.247_1	MAL13P1.247	0.581493	-0.07496	-0.07633	-0.03101	-0.15038
aMAL13P1.247_0	MAL13P1.247	0.581493	-0.07496	-0.07633	-0.03101	-0.15038
intMAL13P1.248_2	MAL13P1.248	-0.18669	-0.28483	-0.57068	-0.22688	0.57827
intMAL13P1.247_2	MAL13P1.248	0.581493	-0.07496	-0.07633	-0.03101	-0.15038
aMAL13P1.248_0	MAL13P1.248	-0.18669	-0.28483	-0.57068	-0.22688	0.57827
intMAL13P1.249_3	MAL13P1.249	-0.08655	-0.13759	-0.7544	-0.28832	0.8877
aMAL13P1.249_5	MAL13P1.249	-0.08655	-0.13759	-0.7544	-0.28832	0.8877
aMAL13P1.249_4	MAL13P1.249	-0.08655	-0.13759	-0.7544	-0.28832	0.8877
aMAL13P1.249_3	MAL13P1.249	-0.08655	-0.13759	-0.7544	-0.28832	0.8877
aMAL13P1.249_2	MAL13P1.249	-0.08655	-0.13759	-0.7544	-0.28832	0.8877
aMAL13P1.249_1	MAL13P1.249	-0.08655	-0.13759	-0.7544	-0.28832	0.8877
aMAL13P1.249_0	MAL13P1.249	-0.08655	-0.13759	-0.7544	-0.28832	0.8877
intPF13_0022_2	MAL13P1.25	-0.36217	-0.23815	0.453793	0.569873	-0.01519
intPF13_0021_2	MAL13P1.25	-1.72265	-0.90812	0.583045	1.283855	0.825665
aMAL13P1.25_1	MAL13P1.25	-1.07747	-0.53466	0.687563	0.943103	0.156823
aMAL13P1.25_0	MAL13P1.25	-1.07747	-0.53466	0.687563	0.943103	0.156823
intPF13_0260_2	MAL13P1.250	0.390307	-0.34552	-0.90594	-0.30772	0.838857
intMAL13P1.250_3	MAL13P1.250	-0.80491	-0.88343	-0.35112	0.512457	1.091767
aMAL13P1.250_0	MAL13P1.250	-0.80491	-0.88343	-0.35112	0.512457	1.091767
aMAL13P1.251_0	MAL13P1.251	-0.58896	-0.54516	0.310317	0.759017	0.402497
intMAL13P1.251_2	MAL13P1.252	-0.58896	-0.54516	0.310317	0.759017	0.402497
aMAL13P1.252_0	MAL13P1.252	0.350452	0.287812	-0.34802	-0.40708	0.102472
aMAL13P1.253_0	MAL13P1.253	0.309587	0.117087	0.343837	0.210497	-0.33975
intMAL13P1.254_2	MAL13P1.254	0.019057	0.137727	-0.53044	-0.63622	0.103607
aMAL13P1.254_0	MAL13P1.254	0.019057	0.137727	-0.53044	-0.63622	0.103607
intMAL13P1.255_2	MAL13P1.255	-0.90709	-0.12907	0.958373	0.883293	-0.21577
aMAL13P1.255_1	MAL13P1.255	-0.90709	-0.12907	0.958373	0.883293	-0.21577
aMAL13P1.255_0	MAL13P1.255	-0.90709	-0.12907	0.958373	0.883293	-0.21577
intMAL13P1.256_2	MAL13P1.256	-0.18165	0.627957	0.476757	-0.23598	-0.74884
aMAL13P1.256_2	MAL13P1.256	-0.18165	0.627957	0.476757	-0.23598	-0.74884
aMAL13P1.256_1	MAL13P1.256	-0.18165	0.627957	0.476757	-0.23598	-0.74884
aMAL13P1.256_0	MAL13P1.256	-0.18165	0.627957	0.476757	-0.23598	-0.74884
intMAL13P1.258_2	MAL13P1.257	-0.31651	-0.20043	-0.01714	0.336588	0.508208
intMAL13P1.257_2	MAL13P1.257	1.011463	0.645663	0.189683	-0.17102	-0.511
aMAL13P1.257_0	MAL13P1.257	1.011463	0.645663	0.189683	-0.17102	-0.511
aMAL13P1.258_2	MAL13P1.258	-0.31651	-0.20043	-0.01714	0.336588	0.508208
aMAL13P1.258_1	MAL13P1.258	-0.31651	-0.20043	-0.01714	0.336588	0.508208
aMAL13P1.258_0	MAL13P1.258	-0.31651	-0.20043	-0.01714	0.336588	0.508208
intMAL13P1.259_2	MAL13P1.259	-0.47253	-0.54463	-0.17088	0.22672	0.44777
aMAL13P1.259_1	MAL13P1.259	-0.47253	-0.54463	-0.17088	0.22672	0.44777
aMAL13P1.259_0	MAL13P1.259	-0.47253	-0.54463	-0.17088	0.22672	0.44777
intMAL13P1.26_2	MAL13P1.26	0.517272	0.619152	0.494442	-0.0385	-0.72388
aMAL13P1.26_5	MAL13P1.26	0.517272	0.619152	0.494442	-0.0385	-0.72388
aMAL13P1.26_4	MAL13P1.26	0.517272	0.619152	0.494442	-0.0385	-0.72388
aMAL13P1.26_3	MAL13P1.26	0.517272	0.619152	0.494442	-0.0385	-0.72388

aMAL13P1.26_2	MAL13P1.26	0.517272	0.619152	0.494442	-0.0385	-0.72388
aMAL13P1.26_1	MAL13P1.26	0.517272	0.619152	0.494442	-0.0385	-0.72388
aMAL13P1.26_0	MAL13P1.26	0.517272	0.619152	0.494442	-0.0385	-0.72388
intMAL13P1.260_2	MAL13P1.260	1.106477	0.012107	-1.91646	-2.2017	-0.14977
aMAL13P1.260_1	MAL13P1.260	1.106477	0.012107	-1.91646	-2.2017	-0.14977
aMAL13P1.260_0	MAL13P1.260	1.106477	0.012107	-1.91646	-2.2017	-0.14977
intMAL13P1.262_2	MAL13P1.261	-0.61875	0.226423	0.047483	0.047843	0.341913
aMAL13P1.261_1	MAL13P1.261	0.15793	0.65887	-0.04748	-0.39802	-0.12337
aMAL13P1.261_0	MAL13P1.261	0.15793	0.65887	-0.04748	-0.39802	-0.12337
intMAL13P1.261_2	MAL13P1.262	0.15793	0.65887	-0.04748	-0.39802	-0.12337
aMAL13P1.262_2	MAL13P1.262	-0.61875	0.226423	0.047483	0.047843	0.341913
aMAL13P1.262_1	MAL13P1.262	-0.61875	0.226423	0.047483	0.047843	0.341913
aMAL13P1.262_0	MAL13P1.262	-0.61875	0.226423	0.047483	0.047843	0.341913
intPF13_0271_2	MAL13P1.264	0.456493	0.323033	0.375693	-0.38753	-1.20687
intMAL13P1.264_2	MAL13P1.264	0.797163	0.906453	0.611363	-0.53668	-1.58727
aMAL13P1.264_1	MAL13P1.264	0.797163	0.906453	0.611363	-0.53668	-1.58727
aMAL13P1.264_0	MAL13P1.264	0.797163	0.906453	0.611363	-0.53668	-1.58727
intMAL13P1.265_3	MAL13P1.265	-0.09805	0.023713	0.172813	0.207853	0.043453
aMAL13P1.265_1	MAL13P1.265	-0.09805	0.023713	0.172813	0.207853	0.043453
aMAL13P1.265_0	MAL13P1.265	-0.09805	0.023713	0.172813	0.207853	0.043453
intMAL13P1.266_2	MAL13P1.266	0.444265	0.136995	-0.34658	-0.17304	0.319065
aMAL13P1.266_2	MAL13P1.266	0.444265	0.136995	-0.34658	-0.17304	0.319065
aMAL13P1.266_1	MAL13P1.266	0.444265	0.136995	-0.34658	-0.17304	0.319065
aMAL13P1.266_0	MAL13P1.266	0.444265	0.136995	-0.34658	-0.17304	0.319065
intMAL13P1.267_2	MAL13P1.267	-0.47758	0.428893	0.303703	-0.31504	-0.58423
aMAL13P1.267_0	MAL13P1.267	-0.47758	0.428893	0.303703	-0.31504	-0.58423
intMAL13P1.268_2	MAL13P1.268	2.63004	1.29429	-0.70608	-2.23208	-2.06746
aMAL13P1.268_1	MAL13P1.268	2.63004	1.29429	-0.70608	-2.23208	-2.06746
aMAL13P1.268_0	MAL13P1.268	2.63004	1.29429	-0.70608	-2.23208	-2.06746
intMAL13P1.269_2	MAL13P1.269					
aMAL13P1.269_1	MAL13P1.269					
aMAL13P1.269_0	MAL13P1.269					
intPF13_0282_2	MAL13P1.270	-0.7578	-0.60399	0.258487	0.874847	0.688327
intMAL13P1.270_2	MAL13P1.270	-0.46114	-0.47828	0.249067	0.774267	0.539617
aMAL13P1.270_0	MAL13P1.270	-0.46114	-0.47828	0.249067	0.774267	0.539617
intMAL13P1.271_2	MAL13P1.271	0.00461	0.11994	0.33461	0.01539	-0.51194
aMAL13P1.271_0	MAL13P1.271	0.00461	0.11994	0.33461	0.01539	-0.51194
intMAL13P1.272_2	MAL13P1.272	1.04334	0.87831	0.74387	-0.26087	-1.51731
aMAL13P1.272_0	MAL13P1.272	1.04334	0.87831	0.74387	-0.26087	-1.51731
intMAL13P1.274_2	MAL13P1.274	0.15384	0.9453	0.52289	-0.15039	-0.6053
aMAL13P1.274_1	MAL13P1.274	0.15384	0.9453	0.52289	-0.15039	-0.6053
aMAL13P1.274_0	MAL13P1.274	0.15384	0.9453	0.52289	-0.15039	-0.6053
intMAL13P1.275_2	MAL13P1.275	0.183263	0.247673	-0.01484	-0.3245	-0.35551
aMAL13P1.275_1	MAL13P1.275	0.183263	0.247673	-0.01484	-0.3245	-0.35551
aMAL13P1.275_0	MAL13P1.275	0.183263	0.247673	-0.01484	-0.3245	-0.35551
intMAL13P1.276_2	MAL13P1.276					
aMAL13P1.276_0	MAL13P1.276					

intMAL13P1.277_2	MAL13P1.277	-0.10587	0.595387	0.705227	-0.01289	-0.88005
aMAL13P1.277_0	MAL13P1.277	-0.10587	0.595387	0.705227	-0.01289	-0.88005
intMAL13P1.278_2	MAL13P1.278	0.576935	-0.13531	-1.9443	-1.7775	0.615795
aMAL13P1.278_6	MAL13P1.278	0.576935	-0.13531	-1.9443	-1.7775	0.615795
aMAL13P1.278_5	MAL13P1.278	0.576935	-0.13531	-1.9443	-1.7775	0.615795
aMAL13P1.278_4	MAL13P1.278	0.576935	-0.13531	-1.9443	-1.7775	0.615795
aMAL13P1.278_3	MAL13P1.278	0.576935	-0.13531	-1.9443	-1.7775	0.615795
aMAL13P1.278_2	MAL13P1.278	0.576935	-0.13531	-1.9443	-1.7775	0.615795
aMAL13P1.278_1	MAL13P1.278	0.576935	-0.13531	-1.9443	-1.7775	0.615795
aMAL13P1.278_0	MAL13P1.278	0.576935	-0.13531	-1.9443	-1.7775	0.615795
intMAL13P1.279_2	MAL13P1.279	-0.15961	-0.34297	0.117433	0.452233	0.287633
aMAL13P1.279_0	MAL13P1.279	-0.15961	-0.34297	0.117433	0.452233	0.287633
aMAL13P1.28_1	MAL13P1.28	0.357748	0.646018	0.513088	-0.02392	-0.6651
aMAL13P1.28_0	MAL13P1.28	0.357748	0.646018	0.513088	-0.02392	-0.6651
intPF13_0306_2	MAL13P1.281					
intMAL13P1.281_2	MAL13P1.281	-0.86264	-0.50576	0.104037	0.635297	0.712597
aMAL13P1.281_1	MAL13P1.281	-0.86264	-0.50576	0.104037	0.635297	0.712597
aMAL13P1.281_0	MAL13P1.281	-0.86264	-0.50576	0.104037	0.635297	0.712597
aMAL13P1.282_0	MAL13P1.282	-0.103	0.11998	0.03954	0.09746	0.18802
intMAL13P1.283_2	MAL13P1.283	-0.24568	0.355873	1.143253	0.654413	-0.79521
aMAL13P1.283_1	MAL13P1.283	-0.24568	0.355873	1.143253	0.654413	-0.79521
aMAL13P1.283_0	MAL13P1.283	-0.24568	0.355873	1.143253	0.654413	-0.79521
intMAL13P1.284_3	MAL13P1.284	-1.11023	0.189167	1.770157	1.236177	-0.89483
aMAL13P1.284_0	MAL13P1.284	-1.11023	0.189167	1.770157	1.236177	-0.89483
intMAL13P1.285_2	MAL13P1.285	-0.7708	-1.24802	-0.82213	0.511462	1.699352
aMAL13P1.285_2	MAL13P1.285	-0.7708	-1.24802	-0.82213	0.511462	1.699352
aMAL13P1.285_1	MAL13P1.285	-0.7708	-1.24802	-0.82213	0.511462	1.699352
aMAL13P1.285_0	MAL13P1.285	-0.7708	-1.24802	-0.82213	0.511462	1.699352
intMAL13P1.286_2	MAL13P1.286	0.695247	0.261107	-0.03755	-0.209	-0.30233
aMAL13P1.286_2	MAL13P1.286	0.695247	0.261107	-0.03755	-0.209	-0.30233
aMAL13P1.286_1	MAL13P1.286	0.695247	0.261107	-0.03755	-0.209	-0.30233
aMAL13P1.286_0	MAL13P1.286	0.695247	0.261107	-0.03755	-0.209	-0.30233
intPF13_0313_2	MAL13P1.287	0.51996	1.26118	1.16014	-0.03814	-1.51668
aMAL13P1.287_0	MAL13P1.287	-0.06454	-0.36034	-1.23832	-0.71702	0.976003
intMAL13P1.288_2	MAL13P1.288	0.404743	0.059293	-0.78158	-0.49276	0.598373
aMAL13P1.288_0	MAL13P1.288	0.404743	0.059293	-0.78158	-0.49276	0.598373
intMAL13P1.290_2	MAL13P1.289	-0.72568	-0.48956	0.455137	0.887197	0.411897
intMAL13P1.289_2	MAL13P1.289	0.680148	0.625418	0.404028	-0.17779	-0.81718
aMAL13P1.289_1	MAL13P1.289	0.680148	0.625418	0.404028	-0.17779	-0.81718
aMAL13P1.289_0	MAL13P1.289	0.680148	0.625418	0.404028	-0.17779	-0.81718
intPF13_0031_2	MAL13P1.29	-1.02527	-0.17316	0.666073	0.604093	-0.08868
intMAL13P1.29_2	MAL13P1.29	-0.49378	-0.5841	-1.90661	-0.85837	1.983077
aMAL13P1.29_1	MAL13P1.29	-0.49378	-0.5841	-1.90661	-0.85837	1.983077
aMAL13P1.29_0	MAL13P1.29	-0.49378	-0.5841	-1.90661	-0.85837	1.983077
intMAL13P1.292_2	MAL13P1.290	0.7469	0.47098	0.3183	-0.3113	-0.98548
aMAL13P1.291_0	MAL13P1.290	-0.75853	0.61966	0.38268	0.12932	0.15834
aMAL13P1.290_0	MAL13P1.290	-0.72568	-0.48956	0.455137	0.887197	0.411897

aMAL13P1.292_1	MAL13P1.292	0.7469	0.47098	0.3183	-0.3113	-0.98548
aMAL13P1.292_0	MAL13P1.292	0.7469	0.47098	0.3183	-0.3113	-0.98548
aMAL13P1.293_4	MAL13P1.293	0.928543	0.638973	0.592613	-0.26055	-1.36081
aMAL13P1.293_3	MAL13P1.293	0.928543	0.638973	0.592613	-0.26055	-1.36081
aMAL13P1.293_2	MAL13P1.293	0.928543	0.638973	0.592613	-0.26055	-1.36081
aMAL13P1.293_1	MAL13P1.293	0.928543	0.638973	0.592613	-0.26055	-1.36081
aMAL13P1.293_0	MAL13P1.293	0.928543	0.638973	0.592613	-0.26055	-1.36081
intMAL13P1.294_2	MAL13P1.294	0.65371	0.56195	0.6686	0.1574	-0.83295
aMAL13P1.294_1	MAL13P1.294	0.65371	0.56195	0.6686	0.1574	-0.83295
aMAL13P1.294_0	MAL13P1.294	0.65371	0.56195	0.6686	0.1574	-0.83295
intMAL13P1.295_2	MAL13P1.295	-1.84156	-1.80795	0.382772	1.606312	1.116282
aMAL13P1.295_3	MAL13P1.295	-1.84156	-1.80795	0.382772	1.606312	1.116282
aMAL13P1.295_2	MAL13P1.295	-1.84156	-1.80795	0.382772	1.606312	1.116282
aMAL13P1.295_1	MAL13P1.295	-1.84156	-1.80795	0.382772	1.606312	1.116282
aMAL13P1.295_0	MAL13P1.295	-1.84156	-1.80795	0.382772	1.606312	1.116282
intMAL13P1.296_2	MAL13P1.296	0.575007	-0.04208	-1.88157	-1.42468	1.092797
aMAL13P1.296_5	MAL13P1.296	0.575007	-0.04208	-1.88157	-1.42468	1.092797
aMAL13P1.296_4	MAL13P1.296	0.575007	-0.04208	-1.88157	-1.42468	1.092797
aMAL13P1.296_3	MAL13P1.296	0.575007	-0.04208	-1.88157	-1.42468	1.092797
aMAL13P1.296_2	MAL13P1.296	0.575007	-0.04208	-1.88157	-1.42468	1.092797
aMAL13P1.296_1	MAL13P1.296	0.575007	-0.04208	-1.88157	-1.42468	1.092797
aMAL13P1.296_0	MAL13P1.296	0.575007	-0.04208	-1.88157	-1.42468	1.092797
intMAL13P1.297_2	MAL13P1.297	0.099433	0.894813	-0.86714	-1.2722	0.244853
aMAL13P1.297_0	MAL13P1.297	0.099433	0.894813	-0.86714	-1.2722	0.244853
intMAL13P1.298_3	MAL13P1.298	0.592207	0.538447	-0.50964	-0.76852	-0.05686
aMAL13P1.298_3	MAL13P1.298	0.592207	0.538447	-0.50964	-0.76852	-0.05686
aMAL13P1.298_2	MAL13P1.298	0.592207	0.538447	-0.50964	-0.76852	-0.05686
aMAL13P1.298_1	MAL13P1.298	0.592207	0.538447	-0.50964	-0.76852	-0.05686
aMAL13P1.298_0	MAL13P1.298	0.592207	0.538447	-0.50964	-0.76852	-0.05686
intPF13_0318_2	MAL13P1.299	0.512017	0.601897	0.160697	-0.20236	-0.36756
intMAL13P1.299_3	MAL13P1.299	0.359877	0.367637	0.402727	0.070607	-0.5073
aMAL13P1.299_0	MAL13P1.299	0.359877	0.367637	0.402727	0.070607	-0.5073
intMAL13P1.30_2	MAL13P1.30	-0.82515	-1.24769	-0.46508	0.605443	1.204053
aMAL13P1.30_0	MAL13P1.30	-0.82515	-1.24769	-0.46508	0.605443	1.204053
intPF13_0319_2	MAL13P1.300	0.084117	0.542817	0.879067	0.308067	-0.81568
aMAL13P1.300_1	MAL13P1.300	0.084893	0.231513	0.648713	0.436953	-0.40785
aMAL13P1.300_0	MAL13P1.300	0.084893	0.231513	0.648713	0.436953	-0.40785
intMAL13P1.301_2	MAL13P1.301	0.267365	0.088195	0.359445	0.254925	-0.30694
aMAL13P1.301_4	MAL13P1.301	0.267365	0.088195	0.359445	0.254925	-0.30694
aMAL13P1.301_3	MAL13P1.301	0.267365	0.088195	0.359445	0.254925	-0.30694
aMAL13P1.301_2	MAL13P1.301	0.267365	0.088195	0.359445	0.254925	-0.30694
aMAL13P1.301_1	MAL13P1.301	0.267365	0.088195	0.359445	0.254925	-0.30694
aMAL13P1.301_0	MAL13P1.301	0.267365	0.088195	0.359445	0.254925	-0.30694
intMAL13P1.302_3	MAL13P1.302	0.100177	-0.03537	0.002497	-0.03216	-0.10979
aMAL13P1.302_1	MAL13P1.302	0.100177	-0.03537	0.002497	-0.03216	-0.10979
aMAL13P1.302_0	MAL13P1.302	0.100177	-0.03537	0.002497	-0.03216	-0.10979
aMAL13P1.303_1	MAL13P1.303-a	-1.64796	-2.17372	-0.20581	1.63881	1.93572

aMAL13P1.303_0	MAL13P1.303-a	-1.64796	-2.17372	-0.20581	1.63881	1.93572
intMAL13P1.303_2	MAL13P1.303-b	-1.64796	-2.17372	-0.20581	1.63881	1.93572
intMAL13P1.305_2	MAL13P1.304	-0.28554	-0.68727	-0.1346	0.385843	0.462513
intMAL13P1.304_3	MAL13P1.304	0.11187	0.25532	-0.06614	-0.26397	-0.14177
aMAL13P1.304_2	MAL13P1.304	0.11187	0.25532	-0.06614	-0.26397	-0.14177
aMAL13P1.304_1	MAL13P1.304	0.11187	0.25532	-0.06614	-0.26397	-0.14177
aMAL13P1.304_0	MAL13P1.304	0.11187	0.25532	-0.06614	-0.26397	-0.14177
aMAL13P1.305_0	MAL13P1.305	-0.28554	-0.68727	-0.1346	0.385843	0.462513
intPF13_0326_2	MAL13P1.306-a	0.28558	-0.4979	-1.30321	-1.02279	0.4279
aMAL13P1.306_1	MAL13P1.306-a	0.67495	-0.44727	-1.99896	-1.59154	0.75427
aMAL13P1.306_0	MAL13P1.306-a	0.67495	-0.44727	-1.99896	-1.59154	0.75427
intMAL13P1.306_2	MAL13P1.306-b	0.67495	-0.44727	-1.99896	-1.59154	0.75427
aMAL13P1.307_0	MAL13P1.307	-0.23662	-1.0219	-0.24969	0.62569	0.7939
intMAL13P1.308_3	MAL13P1.308	0.169373	-0.93721	-2.34809	-1.24958	1.749203
aMAL13P1.308_3	MAL13P1.308	0.169373	-0.93721	-2.34809	-1.24958	1.749203
aMAL13P1.308_2	MAL13P1.308	0.169373	-0.93721	-2.34809	-1.24958	1.749203
aMAL13P1.308_1	MAL13P1.308	0.169373	-0.93721	-2.34809	-1.24958	1.749203
aMAL13P1.308_0	MAL13P1.308	0.169373	-0.93721	-2.34809	-1.24958	1.749203
aMAL13P1.309_0	MAL13P1.309	0.348307	0.138457	-0.2309	-0.49176	-0.33812
intMAL13P1.31_2	MAL13P1.31	-1.22939	-0.40658	0.765903	0.984763	0.211743
aMAL13P1.31_1	MAL13P1.31	-1.22939	-0.40658	0.765903	0.984763	0.211743
aMAL13P1.31_0	MAL13P1.31	-1.22939	-0.40658	0.765903	0.984763	0.211743
intMAL13P1.310_2	MAL13P1.310	0.52834	0.77448	-0.23604	-0.74708	-0.34767
aMAL13P1.310_3	MAL13P1.310	0.52834	0.77448	-0.23604	-0.74708	-0.34767
aMAL13P1.310_2	MAL13P1.310	0.52834	0.77448	-0.23604	-0.74708	-0.34767
aMAL13P1.310_1	MAL13P1.310	0.52834	0.77448	-0.23604	-0.74708	-0.34767
aMAL13P1.310_0	MAL13P1.310	0.52834	0.77448	-0.23604	-0.74708	-0.34767
intMAL13P1.312_2	MAL13P1.311	-0.66028	-0.88559	-0.44031	0.340647	0.947927
intMAL13P1.311_2	MAL13P1.311	-0.50697	-0.26418	-0.0699	0.239563	0.480343
aMAL13P1.311_1	MAL13P1.311	-0.50697	-0.26418	-0.0699	0.239563	0.480343
aMAL13P1.311_0	MAL13P1.311	-0.50697	-0.26418	-0.0699	0.239563	0.480343
aMAL13P1.312_0	MAL13P1.312	-0.66028	-0.88559	-0.44031	0.340647	0.947927
intMAL13P1.313_2	MAL13P1.313	-0.49942	-0.28091	-0.4847	-0.12053	0.687013
aMAL13P1.313_2	MAL13P1.313	-0.49942	-0.28091	-0.4847	-0.12053	0.687013
aMAL13P1.313_1	MAL13P1.313	-0.49942	-0.28091	-0.4847	-0.12053	0.687013
aMAL13P1.313_0	MAL13P1.313	-0.49942	-0.28091	-0.4847	-0.12053	0.687013
intMAL13P1.315_2	MAL13P1.315	-0.50756	-0.4047	-1.15087	-0.41097	1.392863
aMAL13P1.315_2	MAL13P1.315	-0.50756	-0.4047	-1.15087	-0.41097	1.392863
aMAL13P1.315_1	MAL13P1.315	-0.50756	-0.4047	-1.15087	-0.41097	1.392863
aMAL13P1.315_0	MAL13P1.315	-0.50756	-0.4047	-1.15087	-0.41097	1.392863
aMAL13P1.314_1	MAL13P1.315	-0.222	-0.45697	-1.39431	-0.62285	1.391307
aMAL13P1.314_0	MAL13P1.315	-0.222	-0.45697	-1.39431	-0.62285	1.391307
intMAL13P1.317_2	MAL13P1.316	1.743727	1.203357	0.430287	-0.78545	-1.74602
intMAL13P1.316_2	MAL13P1.316	0.51204	0.70181	-0.10762	-0.60688	-0.41581
aMAL13P1.316_2	MAL13P1.316	0.51204	0.70181	-0.10762	-0.60688	-0.41581
aMAL13P1.316_1	MAL13P1.316	0.51204	0.70181	-0.10762	-0.60688	-0.41581
aMAL13P1.316_0	MAL13P1.316	0.51204	0.70181	-0.10762	-0.60688	-0.41581

intPF13_0333_2	MAL13P1.317	0.23671	0.00345	-0.5294	-0.2216	0.56155
aMAL13P1.317_1	MAL13P1.317	1.743727	1.203357	0.430287	-0.78545	-1.74602
aMAL13P1.317_0	MAL13P1.317	1.743727	1.203357	0.430287	-0.78545	-1.74602
intPF13_0334_2	MAL13P1.318	0.736107	0.416977	0.551547	0.263287	-0.58364
intMAL13P1.318_2	MAL13P1.318	0.524493	0.629543	0.571423	0.069243	-0.69888
aMAL13P1.318_0	MAL13P1.318	0.524493	0.629543	0.571423	0.069243	-0.69888
intPF13_0337_2	MAL13P1.319	-0.776	-0.44902	0.530442	0.803802	0.192532
aMAL13P1.319_1	MAL13P1.319	-1.01152	-0.99594	0.171633	0.959033	0.797103
aMAL13P1.319_0	MAL13P1.319	-1.01152	-0.99594	0.171633	0.959033	0.797103
intMAL13P1.32_2	MAL13P1.32	-0.49525	-0.18786	-0.01296	0.096822	0.208832
aMAL13P1.32_6	MAL13P1.32	-0.49525	-0.18786	-0.01296	0.096822	0.208832
aMAL13P1.32_5	MAL13P1.32	-0.49525	-0.18786	-0.01296	0.096822	0.208832
aMAL13P1.32_4	MAL13P1.32	-0.49525	-0.18786	-0.01296	0.096822	0.208832
aMAL13P1.32_3	MAL13P1.32	-0.49525	-0.18786	-0.01296	0.096822	0.208832
aMAL13P1.32_2	MAL13P1.32	-0.49525	-0.18786	-0.01296	0.096822	0.208832
aMAL13P1.32_1	MAL13P1.32	-0.49525	-0.18786	-0.01296	0.096822	0.208832
aMAL13P1.32_0	MAL13P1.32	-0.49525	-0.18786	-0.01296	0.096822	0.208832
intMAL13P1.320_2	MAL13P1.320	-0.82077	0.32787	0.42676	0.42224	0.34313
aMAL13P1.320_1	MAL13P1.320	-0.82077	0.32787	0.42676	0.42224	0.34313
aMAL13P1.320_0	MAL13P1.320	-0.82077	0.32787	0.42676	0.42224	0.34313
intMAL13P1.321_2	MAL13P1.321	0.21114	0.43329	-0.44032	-0.50568	0.26721
aMAL13P1.321_1	MAL13P1.321	0.21114	0.43329	-0.44032	-0.50568	0.26721
aMAL13P1.321_0	MAL13P1.321	0.21114	0.43329	-0.44032	-0.50568	0.26721
intMAL13P1.322_2	MAL13P1.322	0.700083	0.616563	0.220373	-0.34471	-0.7434
aMAL13P1.322_1	MAL13P1.322	0.700083	0.616563	0.220373	-0.34471	-0.7434
aMAL13P1.322_0	MAL13P1.322	0.700083	0.616563	0.220373	-0.34471	-0.7434
intMAL13P1.323_2	MAL13P1.323	0.569437	0.495027	0.227037	-0.2047	-0.58309
aMAL13P1.323_4	MAL13P1.323	0.569437	0.495027	0.227037	-0.2047	-0.58309
aMAL13P1.323_3	MAL13P1.323	0.569437	0.495027	0.227037	-0.2047	-0.58309
aMAL13P1.323_2	MAL13P1.323	0.569437	0.495027	0.227037	-0.2047	-0.58309
aMAL13P1.323_1	MAL13P1.323	0.569437	0.495027	0.227037	-0.2047	-0.58309
aMAL13P1.323_0	MAL13P1.323	0.569437	0.495027	0.227037	-0.2047	-0.58309
intMAL13P1.325_2	MAL13P1.324	-0.70634	-0.64829	0.035587	0.615747	0.661627
intMAL13P1.324_2	MAL13P1.324	-0.80831	-0.79197	-0.61483	0.363993	1.414633
aMAL13P1.324_1	MAL13P1.324	-0.80831	-0.79197	-0.61483	0.363993	1.414633
aMAL13P1.324_0	MAL13P1.324	-0.80831	-0.79197	-0.61483	0.363993	1.414633
aMAL13P1.325_0	MAL13P1.325	-0.70634	-0.64829	0.035587	0.615747	0.661627
intPF13_0342_2	MAL13P1.326	0.908897	0.828517	0.270467	-0.31313	-0.71618
aMAL13P1.326_0	MAL13P1.326	1.325087	1.168587	0.384337	-0.51	-1.13225
aMAL13P1.327_0	MAL13P1.327	-0.1315	0.724483	1.084543	0.387123	-0.88082
aMAL13P1.328_1	MAL13P1.328	-0.05448	-0.41013	-1.2295	-0.76434	0.852293
aMAL13P1.328_0	MAL13P1.328	-0.05448	-0.41013	-1.2295	-0.76434	0.852293
intMAL13P1.329_2	MAL13P1.329	-0.31162	0.61212	-0.32323	-0.64577	0.13588
aMAL13P1.329_1	MAL13P1.329	-0.31162	0.61212	-0.32323	-0.64577	0.13588
aMAL13P1.329_0	MAL13P1.329	-0.31162	0.61212	-0.32323	-0.64577	0.13588
intMAL13P1.33_2	MAL13P1.33	-0.55554	-0.70032	0.248643	0.825023	0.499983
aMAL13P1.33_0	MAL13P1.33	-0.55554	-0.70032	0.248643	0.825023	0.499983

intMAL13P1.330_2	MAL13P1.330	0.240813	-0.15905	-0.22291	-0.23443	-0.12929
aMAL13P1.330_0	MAL13P1.330	0.240813	-0.15905	-0.22291	-0.23443	-0.12929
intMAL13P1.332_2	MAL13P1.331	-0.27939	-0.95604	0.13807	0.67693	0.21804
intMAL13P1.331_2	MAL13P1.331					
aMAL13P1.331_0	MAL13P1.331					
aMAL13P1.332_0	MAL13P1.332	-0.27939	-0.95604	0.13807	0.67693	0.21804
intMAL13P1.333_2	MAL13P1.333	0.137522	0.413832	0.391302	-0.04467	-0.55435
aMAL13P1.333_5	MAL13P1.333	0.137522	0.413832	0.391302	-0.04467	-0.55435
aMAL13P1.333_4	MAL13P1.333	0.137522	0.413832	0.391302	-0.04467	-0.55435
aMAL13P1.333_3	MAL13P1.333	0.137522	0.413832	0.391302	-0.04467	-0.55435
aMAL13P1.333_2	MAL13P1.333	0.137522	0.413832	0.391302	-0.04467	-0.55435
aMAL13P1.333_1	MAL13P1.333	0.137522	0.413832	0.391302	-0.04467	-0.55435
aMAL13P1.333_0	MAL13P1.333	0.137522	0.413832	0.391302	-0.04467	-0.55435
intMAL13P1.334_2	MAL13P1.334	0.110427	0.426857	0.569037	0.033297	-0.74552
aMAL13P1.334_1	MAL13P1.334	0.110427	0.426857	0.569037	0.033297	-0.74552
aMAL13P1.334_0	MAL13P1.334	0.110427	0.426857	0.569037	0.033297	-0.74552
intMAL13P1.335_2	MAL13P1.335	0.417022	-0.26544	0.801542	0.536902	-0.98012
aMAL13P1.335_0	MAL13P1.335	0.417022	-0.26544	0.801542	0.536902	-0.98012
intMAL13P1.336_3	MAL13P1.336	-0.25367	-0.02763	-0.07847	0.137307	0.415967
aMAL13P1.336_1	MAL13P1.336	-0.25367	-0.02763	-0.07847	0.137307	0.415967
aMAL13P1.336_0	MAL13P1.336	-0.25367	-0.02763	-0.07847	0.137307	0.415967
intMAL13P1.338_2	MAL13P1.337	0.335743	0.465773	0.283713	-0.04805	-0.39361
aMAL13P1.337_0	MAL13P1.337	-0.73804	-0.70676	0.303527	0.957807	0.655097
intMAL13P1.337_2	MAL13P1.338	-0.73804	-0.70676	0.303527	0.957807	0.655097
aMAL13P1.338_1	MAL13P1.338	0.335743	0.465773	0.283713	-0.04805	-0.39361
aMAL13P1.338_0	MAL13P1.338	0.335743	0.465773	0.283713	-0.04805	-0.39361
intMAL13P1.339_2	MAL13P1.339					
aMAL13P1.339_0	MAL13P1.339					
intMAL13P1.35_2	MAL13P1.34	0.597567	0.357657	0.119947	-0.21311	-0.50032
aMAL13P1.34_1	MAL13P1.34	0.42623	0.69436	-0.04922	-0.51428	-0.36036
aMAL13P1.34_0	MAL13P1.34	0.42623	0.69436	-0.04922	-0.51428	-0.36036
intPF13_0352_2	MAL13P1.340	0.96771	0.87895	0.67735	-0.47935	-1.69745
aMAL13P1.340_0	MAL13P1.340	0.849167	0.982147	0.046207	-0.82987	-0.95781
intPF13_0354_2	MAL13P1.341	0.776992	0.611362	0.417112	-0.16823	-0.86847
aMAL13P1.341_0	MAL13P1.341	1.199747	0.744287	0.718417	-0.19408	-1.51195
intMAL13P1.342_2	MAL13P1.342	0.018278	-0.34999	-0.58688	-0.0561	0.752398
aMAL13P1.342_1	MAL13P1.342	0.018278	-0.34999	-0.58688	-0.0561	0.752398
aMAL13P1.342_0	MAL13P1.342	0.018278	-0.34999	-0.58688	-0.0561	0.752398
intMAL13P1.343_2	MAL13P1.343	-0.95678	-0.52307	0.100147	0.613187	0.706407
aMAL13P1.343_0	MAL13P1.343	-0.95678	-0.52307	0.100147	0.613187	0.706407
intMAL13P1.344_2	MAL13P1.344	-0.42549	0.271457	0.742327	0.535007	-0.23562
aMAL13P1.344_1	MAL13P1.344	-0.42549	0.271457	0.742327	0.535007	-0.23562
aMAL13P1.344_0	MAL13P1.344	-0.42549	0.271457	0.742327	0.535007	-0.23562
intMAL13P1.345_3	MAL13P1.345	1.10654	0.38328	0.00194	-0.19894	-0.41528
aMAL13P1.345_0	MAL13P1.345	1.10654	0.38328	0.00194	-0.19894	-0.41528
intMAL13P1.346_2	MAL13P1.346	-0.78587	-0.69951	-0.31941	0.464133	1.077393
aMAL13P1.346_2	MAL13P1.346	-0.78587	-0.69951	-0.31941	0.464133	1.077393

aMAL13P1.346_1	MAL13P1.346	-0.78587	-0.69951	-0.31941	0.464133	1.077393
aMAL13P1.346_0	MAL13P1.346	-0.78587	-0.69951	-0.31941	0.464133	1.077393
intMAL13P1.347_2	MAL13P1.347	-0.05969	0.072943	0.406363	0.335803	-0.17278
aMAL13P1.347_1	MAL13P1.347	-0.05969	0.072943	0.406363	0.335803	-0.17278
aMAL13P1.347_0	MAL13P1.347	-0.05969	0.072943	0.406363	0.335803	-0.17278
intMAL13P1.348_2	MAL13P1.348	0.47776	-0.06031	-0.53213	-0.25887	0.38781
aMAL13P1.348_1	MAL13P1.348	0.47776	-0.06031	-0.53213	-0.25887	0.38781
aMAL13P1.348_0	MAL13P1.348	0.47776	-0.06031	-0.53213	-0.25887	0.38781
intPF13_0360_2	MAL13P1.349	0.80064	0.30081	0.15764	-0.06564	-0.45381
intMAL13P1.349_2	MAL13P1.349	0.705048	0.886768	0.069828	-0.47166	-0.47935
aMAL13P1.349_3	MAL13P1.349	0.705048	0.886768	0.069828	-0.47166	-0.47935
aMAL13P1.349_2	MAL13P1.349	0.705048	0.886768	0.069828	-0.47166	-0.47935
aMAL13P1.349_1	MAL13P1.349	0.705048	0.886768	0.069828	-0.47166	-0.47935
aMAL13P1.349_0	MAL13P1.349	0.705048	0.886768	0.069828	-0.47166	-0.47935
intMAL13P1.34_2	MAL13P1.35	0.42623	0.69436	-0.04922	-0.51428	-0.36036
aMAL13P1.35_1	MAL13P1.35	0.597567	0.357657	0.119947	-0.21311	-0.50032
aMAL13P1.35_0	MAL13P1.35	0.597567	0.357657	0.119947	-0.21311	-0.50032
intMAL13P1.350_2	MAL13P1.350	0.3804	0.42798	-0.51495	-0.70305	0.04802
aMAL13P1.350_1	MAL13P1.350	0.3804	0.42798	-0.51495	-0.70305	0.04802
aMAL13P1.350_0	MAL13P1.350	0.3804	0.42798	-0.51495	-0.70305	0.04802
intMAL13P1.351_2	MAL13P1.351	0.108917	0.283337	-0.07512	-0.02094	0.258897
aMAL13P1.351_4	MAL13P1.351	0.108917	0.283337	-0.07512	-0.02094	0.258897
aMAL13P1.351_3	MAL13P1.351	0.108917	0.283337	-0.07512	-0.02094	0.258897
aMAL13P1.351_2	MAL13P1.351	0.108917	0.283337	-0.07512	-0.02094	0.258897
aMAL13P1.351_1	MAL13P1.351	0.108917	0.283337	-0.07512	-0.02094	0.258897
aMAL13P1.351_0	MAL13P1.351	0.108917	0.283337	-0.07512	-0.02094	0.258897
intMAL13P1.352_3	MAL13P1.352	0.498337	0.437837	0.557337	0.107997	-0.734
aMAL13P1.352_1	MAL13P1.352	0.498337	0.437837	0.557337	0.107997	-0.734
aMAL13P1.352_0	MAL13P1.352	0.498337	0.437837	0.557337	0.107997	-0.734
aMAL13P1.36_0	MAL13P1.36	0.170208	0.214548	0.566068	0.240048	-0.59143
intMAL13P1.360_2	MAL13P1.360	0.72043	0.15583	-0.19415	-0.55035	-0.60583
aMAL13P1.360_1	MAL13P1.360	0.72043	0.15583	-0.19415	-0.55035	-0.60583
aMAL13P1.360_0	MAL13P1.360	0.72043	0.15583	-0.19415	-0.55035	-0.60583
aMAL13P1.365_1	MAL13P1.365	0.447983	0.353583	0.046593	-0.09093	-0.13542
aMAL13P1.365_0	MAL13P1.365	0.447983	0.353583	0.046593	-0.09093	-0.13542
intMAL13P1.37_2	MAL13P1.37	-0.1282	-0.76646	-1.55731	-0.64035	1.426297
aMAL13P1.37_1	MAL13P1.37	-0.1282	-0.76646	-1.55731	-0.64035	1.426297
aMAL13P1.37_0	MAL13P1.37	-0.1282	-0.76646	-1.55731	-0.64035	1.426297
intMAL13P1.370_2	MAL13P1.370	0.314307	0.485967	0.441327	-0.18449	-0.86663
intMAL13P1.365_2	MAL13P1.370	0.447983	0.353583	0.046593	-0.09093	-0.13542
aMAL13P1.370_1	MAL13P1.370	0.314307	0.485967	0.441327	-0.18449	-0.86663
aMAL13P1.370_0	MAL13P1.370	0.314307	0.485967	0.441327	-0.18449	-0.86663
intMAL13P1.38_2	MAL13P1.38	-0.21902	-0.5984	-1.7472	-0.90214	1.524063
aMAL13P1.38_1	MAL13P1.38	-0.21902	-0.5984	-1.7472	-0.90214	1.524063
intMAL13P1.380_2	MAL13P1.380	0.316143	-0.07636	-1.92041	-1.4514	1.187353
aMAL13P1.380_9	MAL13P1.380	0.316143	-0.07636	-1.92041	-1.4514	1.187353
aMAL13P1.380_8	MAL13P1.380	0.316143	-0.07636	-1.92041	-1.4514	1.187353

aMAL13P1.380_7	MAL13P1.380	0.316143	-0.07636	-1.92041	-1.4514	1.187353
aMAL13P1.380_6	MAL13P1.380	0.316143	-0.07636	-1.92041	-1.4514	1.187353
aMAL13P1.380_5	MAL13P1.380	0.316143	-0.07636	-1.92041	-1.4514	1.187353
aMAL13P1.380_4	MAL13P1.380	0.316143	-0.07636	-1.92041	-1.4514	1.187353
aMAL13P1.380_3	MAL13P1.380	0.316143	-0.07636	-1.92041	-1.4514	1.187353
aMAL13P1.380_2	MAL13P1.380	0.316143	-0.07636	-1.92041	-1.4514	1.187353
aMAL13P1.380_11	MAL13P1.380	0.316143	-0.07636	-1.92041	-1.4514	1.187353
aMAL13P1.380_10	MAL13P1.380	0.316143	-0.07636	-1.92041	-1.4514	1.187353
aMAL13P1.380_1	MAL13P1.380	0.316143	-0.07636	-1.92041	-1.4514	1.187353
aMAL13P1.380_0	MAL13P1.380	0.316143	-0.07636	-1.92041	-1.4514	1.187353
intMAL13P1.385_2	MAL13P1.385	0.509887	0.538067	-0.03713	-0.35203	-0.27123
aMAL13P1.385_1	MAL13P1.385	0.509887	0.538067	-0.03713	-0.35203	-0.27123
aMAL13P1.385_0	MAL13P1.385	0.509887	0.538067	-0.03713	-0.35203	-0.27123
intMAL13P1.39_2	MAL13P1.39	-0.42276	-0.31613	-0.25005	0.202135	0.691775
aMAL13P1.39_9	MAL13P1.39	-0.42276	-0.31613	-0.25005	0.202135	0.691775
aMAL13P1.39_8	MAL13P1.39	-0.42276	-0.31613	-0.25005	0.202135	0.691775
aMAL13P1.39_7	MAL13P1.39	-0.42276	-0.31613	-0.25005	0.202135	0.691775
aMAL13P1.39_6	MAL13P1.39	-0.42276	-0.31613	-0.25005	0.202135	0.691775
aMAL13P1.39_5	MAL13P1.39	-0.42276	-0.31613	-0.25005	0.202135	0.691775
aMAL13P1.39_4	MAL13P1.39	-0.42276	-0.31613	-0.25005	0.202135	0.691775
aMAL13P1.39_3	MAL13P1.39	-0.42276	-0.31613	-0.25005	0.202135	0.691775
aMAL13P1.39_2	MAL13P1.39	-0.42276	-0.31613	-0.25005	0.202135	0.691775
aMAL13P1.39_1	MAL13P1.39	-0.42276	-0.31613	-0.25005	0.202135	0.691775
aMAL13P1.39_0	MAL13P1.39	-0.42276	-0.31613	-0.25005	0.202135	0.691775
intMAL13P1.390_2	MAL13P1.390	-0.65012	-0.64587	0.633747	0.931747	0.021877
aMAL13P1.390_1	MAL13P1.390	-0.65012	-0.64587	0.633747	0.931747	0.021877
aMAL13P1.390_0	MAL13P1.390	-0.65012	-0.64587	0.633747	0.931747	0.021877
intMAL13P1.395_2	MAL13P1.395	0.671033	0.805833	0.465583	-0.67142	-1.54717
aMAL13P1.395_1	MAL13P1.395	0.671033	0.805833	0.465583	-0.67142	-1.54717
aMAL13P1.395_0	MAL13P1.395	0.671033	0.805833	0.465583	-0.67142	-1.54717
intMAL13P1.40_3	MAL13P1.40	-0.68547	-0.78363	0.188507	0.683327	0.385467
aMAL13P1.40_1	MAL13P1.40	-0.68547	-0.78363	0.188507	0.683327	0.385467
aMAL13P1.40_0	MAL13P1.40	-0.68547	-0.78363	0.188507	0.683327	0.385467
intMAL13P1.405_2	MAL13P1.405	-0.0373	-0.47849	-1.28051	-1.16481	0.293827
aMAL13P1.405_2	MAL13P1.405	-0.0373	-0.47849	-1.28051	-1.16481	0.293827
aMAL13P1.405_1	MAL13P1.405	-0.0373	-0.47849	-1.28051	-1.16481	0.293827
aMAL13P1.405_0	MAL13P1.405	-0.0373	-0.47849	-1.28051	-1.16481	0.293827
intPF13_0047_2	MAL13P1.41	-1.1794	-1.13247	-0.35329	0.627627	1.227307
intMAL13P1.41_2	MAL13P1.41	-0.66882	-0.39656	0.492273	0.671503	0.063333
aMAL13P1.41_2	MAL13P1.41	-0.66882	-0.39656	0.492273	0.671503	0.063333
aMAL13P1.41_1	MAL13P1.41	-0.66882	-0.39656	0.492273	0.671503	0.063333
aMAL13P1.41_0	MAL13P1.41	-0.66882	-0.39656	0.492273	0.671503	0.063333
aMAL13P1.410_0	MAL13P1.410	0.353267	0.223667	0.072657	-0.12832	-0.29433
intMAL13P1.413_2	MAL13P1.413	1.848563	3.079183	1.163133	-1.55447	-3.02752
aMAL13P1.413_0	MAL13P1.413	1.848563	3.079183	1.163133	-1.55447	-3.02752
intMAL13P1.42_2	MAL13P1.42	-0.99018	-1.19211	-0.49629	0.63029	1.39061
aMAL13P1.42_1	MAL13P1.42	-0.99018	-1.19211	-0.49629	0.63029	1.39061

aMAL13P1.42_0	MAL13P1.42	-0.99018	-1.19211	-0.49629	0.63029	1.39061
intMAL13P1.43_2	MAL13P1.43	0.26553	0.73835	0.6628	-0.0253	-0.84935
aMAL13P1.43_1	MAL13P1.43	0.26553	0.73835	0.6628	-0.0253	-0.84935
aMAL13P1.43_0	MAL13P1.43	0.26553	0.73835	0.6628	-0.0253	-0.84935
intMAL13P1.44_2	MAL13P1.44	-0.32824	0.35719	-0.01913	-0.31987	-0.09719
aMAL13P1.44_1	MAL13P1.44	-0.32824	0.35719	-0.01913	-0.31987	-0.09719
aMAL13P1.44_0	MAL13P1.44	-0.32824	0.35719	-0.01913	-0.31987	-0.09719
intMAL13P1.45_2	MAL13P1.45	0.447477	0.407627	0.007247	-0.28441	-0.31729
aMAL13P1.45_1	MAL13P1.45	0.447477	0.407627	0.007247	-0.28441	-0.31729
aMAL13P1.45_0	MAL13P1.45	0.447477	0.407627	0.007247	-0.28441	-0.31729
intPF13_0054_2	MAL13P1.46	-0.72069	-0.53059	0.879673	1.080993	-0.10475
intMAL13P1.47_2	MAL13P1.46	-1.42183	-1.09585	0.334707	1.099627	0.779187
intMAL13P1.46_2	MAL13P1.46	-0.16427	-0.18765	0.619303	0.671363	-0.19869
aMAL13P1.46_0	MAL13P1.46	-0.16427	-0.18765	0.619303	0.671363	-0.19869
intMAL13P1.465_2	MAL13P1.465	-0.11785	0.43423	1.36305	0.64995	-1.19523
aMAL13P1.465_0	MAL13P1.465	-0.11785	0.43423	1.36305	0.64995	-1.19523
intMAL13P1.48_2	MAL13P1.47	-0.90192	-0.6084	0.61054	0.92446	0.1624
aMAL13P1.47_0	MAL13P1.47	-1.42183	-1.09585	0.334707	1.099627	0.779187
intMAL13P1.470_2	MAL13P1.470	-0.84113	0.69864	1.36021	0.50029	-0.99714
aMAL13P1.470_1	MAL13P1.470	-0.84113	0.69864	1.36021	0.50029	-0.99714
aMAL13P1.470_0	MAL13P1.470	-0.84113	0.69864	1.36021	0.50029	-0.99714
intMAL13P1.475_2	MAL13P1.475	-0.84956	-0.01017	0.310097	0.291737	0.145507
aMAL13P1.475_1	MAL13P1.475	-0.84956	-0.01017	0.310097	0.291737	0.145507
aMAL13P1.475_0	MAL13P1.475	-0.84956	-0.01017	0.310097	0.291737	0.145507
aMAL13P1.48_1	MAL13P1.48	-0.90192	-0.6084	0.61054	0.92446	0.1624
aMAL13P1.48_0	MAL13P1.48	-0.90192	-0.6084	0.61054	0.92446	0.1624
intMAL13P1.480_2	MAL13P1.480					
aMAL13P1.480_0	MAL13P1.480					
intMAL13P1.485_2	MAL13P1.485	-1.17965	-0.77374	-0.33453	0.57503	1.35324
aMAL13P1.485_1	MAL13P1.485	-1.17965	-0.77374	-0.33453	0.57503	1.35324
intMAL13P1.49_2	MAL13P1.49	0.173773	-0.55585	-1.3278	-0.83554	0.752513
aMAL13P1.49_0	MAL13P1.49	0.173773	-0.55585	-1.3278	-0.83554	0.752513
intMAL13P1.50_2	MAL13P1.50	0.294548	0.409268	0.177368	-0.03757	-0.20686
aMAL13P1.50_0	MAL13P1.50	0.294548	0.409268	0.177368	-0.03757	-0.20686
intMAL13P1.51_2	MAL13P1.51	-0.21629	-0.57753	-0.23993	0.017267	0.152867
aMAL13P1.51_0	MAL13P1.51	-0.21629	-0.57753	-0.23993	0.017267	0.152867
intMAL13P1.52_3	MAL13P1.52	0.600163	0.757843	0.634653	-0.18766	-1.13984
aMAL13P1.52_2	MAL13P1.52	0.600163	0.757843	0.634653	-0.18766	-1.13984
aMAL13P1.52_1	MAL13P1.52	0.600163	0.757843	0.634653	-0.18766	-1.13984
aMAL13P1.52_0	MAL13P1.52	0.600163	0.757843	0.634653	-0.18766	-1.13984
intMAL13P1.525_2	MAL13P1.525					
intPF13_0060_2	MAL13P1.53	1.603873	1.405653	0.038693	-1.10503	-1.32599
intMAL13P1.53_2	MAL13P1.53	1.565737	1.127307	0.545877	-0.48854	-1.50397
aMAL13P1.53_0	MAL13P1.53	1.565737	1.127307	0.545877	-0.48854	-1.50397
intPF13_0061_2	MAL13P1.54	-0.19368	-0.35559	0.14217	0.42383	0.20259
aMAL13P1.54_1	MAL13P1.54	-0.15967	0.40033	0.53458	0.21242	-0.34133
aMAL13P1.54_0	MAL13P1.54	-0.15967	0.40033	0.53458	0.21242	-0.34133

intMAL13P1.540_2	MAL13P1.540	-0.26279	0.640467	-0.20093	-0.26373	0.487367
aMAL13P1.540_1	MAL13P1.540	-0.26279	0.640467	-0.20093	-0.26373	0.487367
aMAL13P1.540_0	MAL13P1.540	-0.26279	0.640467	-0.20093	-0.26373	0.487367
intMAL13P1.55_2	MAL13P1.55	0.996017	0.938897	0.967197	0.007137	-1.46856
aMAL13P1.55_0	MAL13P1.55	0.996017	0.938897	0.967197	0.007137	-1.46856
intMAL13P1.550_2	MAL13P1.550					
intMAL13P1.56_3	MAL13P1.56	0.063	0.421	0.85625	0.30075	-0.859
aMAL13P1.56_1	MAL13P1.56	0.063	0.421	0.85625	0.30075	-0.859
aMAL13P1.56_0	MAL13P1.56	0.063	0.421	0.85625	0.30075	-0.859
intPF13_0067_2	MAL13P1.57	-0.66237	-0.96968	-1.20765	-0.07069	1.682343
aMAL13P1.57_0	MAL13P1.57	-0.0329	1.18152	0.33995	-0.38395	-0.40052
intMAL13P1.58_2	MAL13P1.58	-0.3222	0.275993	1.068513	0.548153	-0.84533
aMAL13P1.58_0	MAL13P1.58	-0.3222	0.275993	1.068513	0.548153	-0.84533
intMAL13P1.580_2	MAL13P1.580					
intMAL13P1.327_2	MAL13P1.580	-0.1315	0.724483	1.084543	0.387123	-0.88082
intMAL13P1.59_2	MAL13P1.59	-1.30803	0.432177	1.710147	0.782187	-1.23784
aMAL13P1.59_0	MAL13P1.59	-1.30803	0.432177	1.710147	0.782187	-1.23784
intMAL13P1.60_2	MAL13P1.60	1.19967	0.10663	-1.523	-2.174	-0.79763
aMAL13P1.60_1	MAL13P1.60	1.19967	0.10663	-1.523	-2.174	-0.79763
aMAL13P1.60_0	MAL13P1.60	1.19967	0.10663	-1.523	-2.174	-0.79763
intMAL13P1.61_2	MAL13P1.61	1.815857	2.817957	1.154317	-1.4975	-3.08914
aMAL13P1.61_0	MAL13P1.61	1.815857	2.817957	1.154317	-1.4975	-3.08914
intMAL13P1.62_2	MAL13P1.62	0.935337	0.324317	-0.39662	-0.67504	-0.38098
aMAL13P1.62_0	MAL13P1.62	0.935337	0.324317	-0.39662	-0.67504	-0.38098
intMAL13P1.63_2	MAL13P1.63	-0.81308	-0.01458	0.14767	0.10133	0.14258
aMAL13P1.63_1	MAL13P1.63	-0.81308	-0.01458	0.14767	0.10133	0.14258
aMAL13P1.63_0	MAL13P1.63	-0.81308	-0.01458	0.14767	0.10133	0.14258
intMAL13P1.66_2	MAL13P1.64	1.050583	-0.08171	-0.25819	-0.24475	-0.29843
intMAL13P1.64_2	MAL13P1.64	0.198597	-0.57499	-0.4045	0.190837	0.578327
aMAL13P1.64_0	MAL13P1.64	0.198597	-0.57499	-0.4045	0.190837	0.578327
aMAL13P1.65_0	MAL13P1.65					
intMAL13P1.65_2	MAL13P1.66					
aMAL13P1.66_4	MAL13P1.66	1.050583	-0.08171	-0.25819	-0.24475	-0.29843
aMAL13P1.66_3	MAL13P1.66	1.050583	-0.08171	-0.25819	-0.24475	-0.29843
aMAL13P1.66_2	MAL13P1.66	1.050583	-0.08171	-0.25819	-0.24475	-0.29843
aMAL13P1.66_1	MAL13P1.66	1.050583	-0.08171	-0.25819	-0.24475	-0.29843
aMAL13P1.66_0	MAL13P1.66	1.050583	-0.08171	-0.25819	-0.24475	-0.29843
intMAL13P1.68_2	MAL13P1.67	-0.65286	-0.7247	0.485893	0.827773	0.085363
intMAL13P1.67_2	MAL13P1.67	-0.2846	-0.03596	0.362427	0.451907	0.084297
aMAL13P1.67_1	MAL13P1.67	-0.2846	-0.03596	0.362427	0.451907	0.084297
aMAL13P1.67_0	MAL13P1.67	-0.2846	-0.03596	0.362427	0.451907	0.084297
aMAL13P1.68_0	MAL13P1.68	-0.65286	-0.7247	0.485893	0.827773	0.085363
intMAL13P1.69_2	MAL13P1.69	0.326457	0.206697	0.171107	0.112227	-0.11636
aMAL13P1.69_0	MAL13P1.69	0.326457	0.206697	0.171107	0.112227	-0.11636
intMAL13P1.70_2	MAL13P1.70	0.855335	0.763485	0.172535	-0.74537	-1.21049
aMAL13P1.70_5	MAL13P1.70	0.855335	0.763485	0.172535	-0.74537	-1.21049
aMAL13P1.70_4	MAL13P1.70	0.855335	0.763485	0.172535	-0.74537	-1.21049

aMAL13P1.70_3	MAL13P1.70	0.855335	0.763485	0.172535	-0.74537	-1.21049
aMAL13P1.70_2	MAL13P1.70	0.855335	0.763485	0.172535	-0.74537	-1.21049
aMAL13P1.70_1	MAL13P1.70	0.855335	0.763485	0.172535	-0.74537	-1.21049
aMAL13P1.70_0	MAL13P1.70	0.855335	0.763485	0.172535	-0.74537	-1.21049
aMAL13P1.71_1	MAL13P1.71	0.51672	0.7734	0.46995	-0.01645	-0.5429
aMAL13P1.71_0	MAL13P1.71	0.51672	0.7734	0.46995	-0.01645	-0.5429
intMAL13P1.72_3	MAL13P1.72	-1.21583	-0.78435	0.399933	1.023233	0.684013
intMAL13P1.71_2	MAL13P1.72	0.51672	0.7734	0.46995	-0.01645	-0.5429
aMAL13P1.72_1	MAL13P1.72	-1.21583	-0.78435	0.399933	1.023233	0.684013
aMAL13P1.72_0	MAL13P1.72	-1.21583	-0.78435	0.399933	1.023233	0.684013
intMAL13P1.73_2	MAL13P1.73	0.344953	0.443243	0.823753	0.422413	-0.69608
aMAL13P1.73_1	MAL13P1.73	0.344953	0.443243	0.823753	0.422413	-0.69608
aMAL13P1.73_0	MAL13P1.73	0.344953	0.443243	0.823753	0.422413	-0.69608
aMAL13P1.74_1	MAL13P1.74	-0.95471	-0.42796	0.333413	0.709413	0.485793
aMAL13P1.74_0	MAL13P1.74	-0.95471	-0.42796	0.333413	0.709413	0.485793
intMAL13P1.75_2	MAL13P1.75	-1.17625	-0.36376	0.617523	0.793643	0.207923
aMAL13P1.75_1	MAL13P1.75	-1.17625	-0.36376	0.617523	0.793643	0.207923
aMAL13P1.75_0	MAL13P1.75	-1.17625	-0.36376	0.617523	0.793643	0.207923
intMAL13P1.76_2	MAL13P1.76	0.678443	0.469773	0.504193	0.038473	-0.77961
aMAL13P1.76_1	MAL13P1.76	0.678443	0.469773	0.504193	0.038473	-0.77961
aMAL13P1.76_0	MAL13P1.76	0.678443	0.469773	0.504193	0.038473	-0.77961
intMAL13P1.77_2	MAL13P1.77	0.698403	0.255993	0.243503	-0.31784	-0.98533
aMAL13P1.77_0	MAL13P1.77	0.698403	0.255993	0.243503	-0.31784	-0.98533
aMAL13P1.78_1	MAL13P1.78	-0.88018	0.027363	0.641273	0.381893	-0.2912
aMAL13P1.78_0	MAL13P1.78	-0.88018	0.027363	0.641273	0.381893	-0.2912
intMAL13P1.80_3	MAL13P1.79	0.360877	-0.39536	0.630727	0.508607	-0.7773
intMAL13P1.79_2	MAL13P1.79	-0.16106	-0.04718	0.345367	0.186967	-0.33048
aMAL13P1.80_0	MAL13P1.79	0.360877	-0.39536	0.630727	0.508607	-0.7773
aMAL13P1.79_1	MAL13P1.79	-0.16106	-0.04718	0.345367	0.186967	-0.33048
aMAL13P1.79_0	MAL13P1.79	-0.16106	-0.04718	0.345367	0.186967	-0.33048
intMAL13P1.81_2	MAL13P1.81	-0.41304	-0.25478	0.58006	0.42894	-0.45222
aMAL13P1.81_0	MAL13P1.81	-0.41304	-0.25478	0.58006	0.42894	-0.45222
intPF13_0087_2	MAL13P1.82	0.374263	0.599673	0.747163	0.074503	-0.98301
intMAL13P1.82_2	MAL13P1.82	-0.781	-0.65798	-0.12154	0.423877	0.680317
aMAL13P1.82_0	MAL13P1.82	-0.781	-0.65798	-0.12154	0.423877	0.680317
intMAL13P1.83_3	MAL13P1.83	0.329077	0.512597	0.248567	-0.12673	-0.41376
aMAL13P1.83_1	MAL13P1.83	0.329077	0.512597	0.248567	-0.12673	-0.41376
aMAL13P1.83_0	MAL13P1.83	0.329077	0.512597	0.248567	-0.12673	-0.41376
intMAL13P1.84_2	MAL13P1.84	-0.01311	-0.08057	0.018342	0.075182	0.029802
aMAL13P1.84_0	MAL13P1.84	-0.01311	-0.08057	0.018342	0.075182	0.029802
aMAL13P1.85_0	MAL13P1.85	0.152887	-0.07542	-0.12015	0.114487	0.290757
intMAL13P1.86_2	MAL13P1.86	-1.32867	-0.59215	0.04304	0.33196	0.46715
aMAL13P1.86_1	MAL13P1.86	-1.32867	-0.59215	0.04304	0.33196	0.46715
aMAL13P1.86_0	MAL13P1.86	-1.32867	-0.59215	0.04304	0.33196	0.46715
intMAL13P1.87_2	MAL13P1.87	0.25107	0.28564	-0.07279	-0.05721	0.15436
aMAL13P1.87_0	MAL13P1.87	0.25107	0.28564	-0.07279	-0.05721	0.15436
intMAL13P1.88_2	MAL13P1.88	-0.03224	-0.00481	0.19087	0.13313	-0.14269

aMAL13P1.88_1	MAL13P1.88	-0.03224	-0.00481	0.19087	0.13313	-0.14269
aMAL13P1.88_0	MAL13P1.88	-0.03224	-0.00481	0.19087	0.13313	-0.14269
intMAL13P1.90_2	MAL13P1.89	0.191107	0.582477	-0.0147	-0.21046	0.035857
intMAL13P1.89_2	MAL13P1.89					
aMAL13P1.89_0	MAL13P1.89					
aMAL13P1.90_1	MAL13P1.90	0.191107	0.582477	-0.0147	-0.21046	0.035857
aMAL13P1.90_0	MAL13P1.90	0.191107	0.582477	-0.0147	-0.21046	0.035857
aMAL13P1.91_0	MAL13P1.91	0.707367	0.488177	0.753147	0.008187	-1.27884
intMAL13P1.92_2	MAL13P1.92	0.173477	0.284637	0.690227	0.430107	-0.4883
intMAL13P1.91_2	MAL13P1.92	0.707367	0.488177	0.753147	0.008187	-1.27884
aMAL13P1.92_0	MAL13P1.92	0.173477	0.284637	0.690227	0.430107	-0.4883
intMAL13P1.93_2	MAL13P1.93	0.70797	0.94667	0.68716	-0.43266	-1.51367
aMAL13P1.93_1	MAL13P1.93	0.70797	0.94667	0.68716	-0.43266	-1.51367
aMAL13P1.93_0	MAL13P1.93	0.70797	0.94667	0.68716	-0.43266	-1.51367
intMAL13P1.94_2	MAL13P1.94	-0.01837	0.425015	-1.36056	-1.3908	0.697635
aMAL13P1.94_0	MAL13P1.94	-0.01837	0.425015	-1.36056	-1.3908	0.697635
intPF13_0100_2	MAL13P1.95	-0.547	-0.66767	-0.00019	0.52183	0.52031
intMAL13P1.95_2	MAL13P1.95	-0.63088	-1.06462	0.03073	0.84627	0.71362
aMAL13P1.95_0	MAL13P1.95	-0.63088	-1.06462	0.03073	0.84627	0.71362
intMAL13P1.96_2	MAL13P1.96	-0.7503	-0.81902	-0.80673	0.164393	1.429683
aMAL13P1.96_1	MAL13P1.96	-0.7503	-0.81902	-0.80673	0.164393	1.429683
aMAL13P1.96_0	MAL13P1.96	-0.7503	-0.81902	-0.80673	0.164393	1.429683
intMAL13P1.97_2	MAL13P1.97	-0.14226	-0.22572	-0.13431	-0.17553	-0.12012
intMAL13P1.100_2	MAL13P1.97	-0.50776	-0.94266	-0.50593	0.458767	1.156497
aMAL13P1.97_0	MAL13P1.97	-0.14226	-0.22572	-0.13431	-0.17553	-0.12012
ac14rR.3-5s_0	MAL14_5S_1	1.426353	-0.57379	0.148073	0.214593	-0.80755
ac14rR.2-5s_0	MAL14_5S_1	1.239513	0.145423	0.098603	-0.20304	-0.80156
ac14rR.1-5s_0	MAL14_5S_1	2.190267	-0.75435	-0.1328	0.048137	-0.91731
achr3-tR-Ile-1_0	MAL3_tR_Ile1	1.051183	1.244083	-0.72318	-1.37216	-0.25342
achr4-tR-Ala-1_0	MAL4_tR_Ala1	0.525188	0.759088	0.607328	-0.11293	-0.95182
achr4-tR-Asn-1_0	MAL4_tR_Asn1	1.295717	0.700897	-0.72504	-1.22012	-0.46556
achr4-tR-Ile-1_0	MAL4_tR_Ile1	0.411813	0.316243	0.249653	-0.03689	-0.43948
achr4-tR-Ser-1_0	MAL4_tR_Ser1	0.830462	-0.36305	0.083482	-0.28265	-1.09587
achr5-tR-Arg-1_0	MAL5_tR_Arg1	0.00553	-0.00264	0.25383	0.04427	-0.40256
achr5-tR-Leu-2_0	MAL5_tR_Leu2	0.785367	0.596177	0.606147	-0.06881	-1.07884
achr5-tR-Phe-1_0	MAL5_tR_Phe1	0.54175	0.33927	-0.04354	-0.19846	-0.17227
intPFF1010c_2	MAL6_tR_Ala1	-0.0457	-0.3335	-0.676	-0.3065	0.5725
achr6-MALr.2_0	MAL6_tR_Ala1					
intPFF1015w_2	MAL6_tR_Leu1	-0.37654	0.106217	0.393067	0.332267	-0.02588
achr6-MALr.4_0	MAL6_tR_Leu1					
achr6-MALr.6_0	MAL6_tR_Ser1	0.393977	0.271117	0.182767	-0.08343	-0.41078
intMAL7P1.142_2	MAL7_18S	-0.44544	0.272203	0.662093	0.343573	-0.36854
ac7rRITS1_0	MAL7_ITS1	0.378063	-0.4754	-0.49926	-0.46844	-0.2303
achr7-tR-Ala-1_0	MAL7_tR_Ala1	0.64158	0.67308	-0.20567	-0.63333	-0.33808
achr7-tR-Asp-1_0	MAL7_tR_Asp1	1.935328	0.725118	-0.00363	-0.56388	-1.00086
aun-4-tR-His-1_0	MAL7_tR_His1	1.348303	0.399473	-0.21145	-0.86489	-1.09281
achr7-tR-Ser-1_0	MAL7_tR_Ser1	1.830813	0.539953	0.630203	-0.25134	-1.78269

aun-4-tR-Thr-1_0	MAL7_tR_Thr1	0.731717	-0.15859	0.059687	0.044647	-0.39207
achr7-tR-Thr-1_0	MAL7_tR_Thr2	0.47242	0.6589	0.32721	-0.22121	-0.6499
intPF07_0010_2	MAL7P1.10	0.402022	0.023382	-0.01543	0.078322	0.027342
aMAL7P1.10_0	MAL7P1.10	0.684703	0.071463	0.334563	0.148103	-0.5718
intMAL7P1.99_2	MAL7P1.100	-0.84267	-0.91964	0.046297	0.836037	0.836977
aMAL7P1.100_1	MAL7P1.100	-0.59648	-0.35448	-0.07232	0.316142	0.569302
aMAL7P1.100_0	MAL7P1.100	-0.59648	-0.35448	-0.07232	0.316142	0.569302
intMAL7P1.102_3	MAL7P1.102	-0.52199	-0.37809	-0.92367	-0.17845	1.347645
aMAL7P1.102_2	MAL7P1.102	-0.52199	-0.37809	-0.92367	-0.17845	1.347645
aMAL7P1.102_1	MAL7P1.102	-0.52199	-0.37809	-0.92367	-0.17845	1.347645
aMAL7P1.102_0	MAL7P1.102	-0.52199	-0.37809	-0.92367	-0.17845	1.347645
aMAL7P1.104_0	MAL7P1.104	0.34901	0.26194	0.31762	0.10138	-0.37194
intMAL7P1.105_2	MAL7P1.105	1.485655	0.495315	-0.68983	-1.30442	-0.85695
aMAL7P1.105_0	MAL7P1.105	1.485655	0.495315	-0.68983	-1.30442	-0.85695
intMAL7P1.106_2	MAL7P1.106	-0.23187	-0.16168	0.259853	0.474813	0.205343
aMAL7P1.106_0	MAL7P1.106	-0.23187	-0.16168	0.259853	0.474813	0.205343
intMAL7P1.107_2	MAL7P1.107	0.036363	0.016173	-0.15085	0.043513	0.340493
aMAL7P1.107_0	MAL7P1.107	0.036363	0.016173	-0.15085	0.043513	0.340493
intMAL7P1.109_2	MAL7P1.108	0.698275	0.917935	-0.6182	-1.25337	-0.36749
intMAL7P1.108_2	MAL7P1.108	0.67623	0.83885	-0.6097	-1.1703	-0.30385
aMAL7P1.109_3	MAL7P1.108	0.698275	0.917935	-0.6182	-1.25337	-0.36749
aMAL7P1.109_2	MAL7P1.108	0.698275	0.917935	-0.6182	-1.25337	-0.36749
aMAL7P1.109_1	MAL7P1.108	0.698275	0.917935	-0.6182	-1.25337	-0.36749
aMAL7P1.109_0	MAL7P1.108	0.698275	0.917935	-0.6182	-1.25337	-0.36749
aMAL7P1.108_0	MAL7P1.108	0.67623	0.83885	-0.6097	-1.1703	-0.30385
aMAL7P1.11_0	MAL7P1.11	-0.23425	-0.19075	0.593	0.572	-0.27825
intMAL7P1.111_2	MAL7P1.110	-0.58965	-0.50879	-0.03393	0.446593	0.589453
intMAL7P1.110_2	MAL7P1.110	-1.08692	-0.73541	0.670173	1.046193	0.212673
aMAL7P1.110_0	MAL7P1.110	-1.08692	-0.73541	0.670173	1.046193	0.212673
aMAL7P1.111_0	MAL7P1.111	-0.58965	-0.50879	-0.03393	0.446593	0.589453
aMAL7P1.112_1	MAL7P1.112	0.648817	0.444927	-0.22359	-0.39357	-0.10009
aMAL7P1.112_0	MAL7P1.112	0.648817	0.444927	-0.22359	-0.39357	-0.10009
intMAL7P1.113_2	MAL7P1.113	0.426873	0.779113	0.687023	-0.09436	-1.02395
intMAL7P1.112_2	MAL7P1.113	0.648817	0.444927	-0.22359	-0.39357	-0.10009
aMAL7P1.113_1	MAL7P1.113	0.426873	0.779113	0.687023	-0.09436	-1.02395
aMAL7P1.113_0	MAL7P1.113	0.426873	0.779113	0.687023	-0.09436	-1.02395
intMAL7P1.115_2	MAL7P1.114	-0.87225	-1.14179	-0.41592	0.669583	1.296453
intMAL7P1.114_2	MAL7P1.114	-1.36256	-1.08422	0.49144	1.32056	0.81122
aMAL7P1.114_0	MAL7P1.114	-1.36256	-1.08422	0.49144	1.32056	0.81122
aMAL7P1.115_1	MAL7P1.115	-0.87225	-1.14179	-0.41592	0.669583	1.296453
aMAL7P1.115_0	MAL7P1.115	-0.87225	-1.14179	-0.41592	0.669583	1.296453
intMAL7P1.117_2	MAL7P1.117	-0.66071	-0.34943	0.16286	0.61714	0.61443
aMAL7P1.117_0	MAL7P1.117	-0.66071	-0.34943	0.16286	0.61714	0.61443
intMAL7P1.118_2	MAL7P1.118	0.33878	0.76559	0.23207	-0.32407	-0.51409
aMAL7P1.118_1	MAL7P1.118	0.33878	0.76559	0.23207	-0.32407	-0.51409
aMAL7P1.118_0	MAL7P1.118	0.33878	0.76559	0.23207	-0.32407	-0.51409
intMAL7P1.119_3	MAL7P1.119	-0.31685	-0.88674	-2.64076	-1.50574	2.09724

aMAL7P1.119_1	MAL7P1.119	-0.31685	-0.88674	-2.64076	-1.50574	2.09724
aMAL7P1.119_0	MAL7P1.119	-0.31685	-0.88674	-2.64076	-1.50574	2.09724
intMAL7P1.12_3	MAL7P1.12	-0.16271	0.069282	0.287322	0.226762	-0.0862
aMAL7P1.12_3	MAL7P1.12	-0.16271	0.069282	0.287322	0.226762	-0.0862
aMAL7P1.12_2	MAL7P1.12	-0.16271	0.069282	0.287322	0.226762	-0.0862
aMAL7P1.12_1	MAL7P1.12	-0.16271	0.069282	0.287322	0.226762	-0.0862
aMAL7P1.12_0	MAL7P1.12	-0.16271	0.069282	0.287322	0.226762	-0.0862
intMAL7P1.122_2	MAL7P1.120	0.025527	0.092877	0.645737	0.428597	-0.48754
intMAL7P1.120_2	MAL7P1.120	0.880833	0.730323	0.239603	-0.56244	-1.08966
aMAL7P1.120_1	MAL7P1.120	0.880833	0.730323	0.239603	-0.56244	-1.08966
aMAL7P1.120_0	MAL7P1.120	0.880833	0.730323	0.239603	-0.56244	-1.08966
aMAL7P1.122_1	MAL7P1.122	0.025527	0.092877	0.645737	0.428597	-0.48754
aMAL7P1.122_0	MAL7P1.122	0.025527	0.092877	0.645737	0.428597	-0.48754
intMAL7P1.123_2	MAL7P1.123	0.223083	-0.15044	-0.58463	-0.32271	0.413103
aMAL7P1.123_0	MAL7P1.123	0.223083	-0.15044	-0.58463	-0.32271	0.413103
intPF07_0096_2	MAL7P1.124	-0.05331	-0.44844	-0.43836	-0.3078	0.060777
aMAL7P1.124_1	MAL7P1.124	1.274557	0.255207	-0.42771	-0.77377	-0.63123
aMAL7P1.124_0	MAL7P1.124	1.274557	0.255207	-0.42771	-0.77377	-0.63123
intMAL7P1.125_2	MAL7P1.125	0.346267	-0.33681	-1.10763	-0.73803	0.583647
aMAL7P1.125_1	MAL7P1.125	0.346267	-0.33681	-1.10763	-0.73803	0.583647
aMAL7P1.125_0	MAL7P1.125	0.346267	-0.33681	-1.10763	-0.73803	0.583647
intMAL7P1.127_2	MAL7P1.126	-0.49229	-0.39151	-0.30997	0.231307	0.818347
intMAL7P1.126_2	MAL7P1.126	-0.4126	0.466517	0.442717	0.214617	-0.04118
aMAL7P1.126_1	MAL7P1.126	-0.4126	0.466517	0.442717	0.214617	-0.04118
aMAL7P1.126_0	MAL7P1.126	-0.4126	0.466517	0.442717	0.214617	-0.04118
aMAL7P1.128_0	MAL7P1.127	0.550873	0.312613	-0.60023	-0.57611	0.261053
aMAL7P1.127_2	MAL7P1.127	-0.49229	-0.39151	-0.30997	0.231307	0.818347
aMAL7P1.127_1	MAL7P1.127	-0.49229	-0.39151	-0.30997	0.231307	0.818347
aMAL7P1.127_0	MAL7P1.127	-0.49229	-0.39151	-0.30997	0.231307	0.818347
intMAL7P1.129_2	MAL7P1.129	-0.4141	-0.32154	0.182313	0.510853	0.355703
aMAL7P1.129_1	MAL7P1.129	-0.4141	-0.32154	0.182313	0.510853	0.355703
aMAL7P1.129_0	MAL7P1.129	-0.4141	-0.32154	0.182313	0.510853	0.355703
intMAL7P1.13_2	MAL7P1.13	-0.75794	-1.07729	-0.9614	0.083737	1.422627
aMAL7P1.13_0	MAL7P1.13	-0.75794	-1.07729	-0.9614	0.083737	1.422627
aMAL7P1.130_0	MAL7P1.130	-0.05606	0.317437	0.284587	0.191347	-0.0044
intMAL7P1.131_2	MAL7P1.131	1.75875	0.63127	-0.10204	-0.82196	-1.21127
aMAL7P1.131_0	MAL7P1.131	1.75875	0.63127	-0.10204	-0.82196	-1.21127
intMAL7P1.132_2	MAL7P1.132	-0.33913	-0.38163	-0.68562	-0.02364	1.080125
aMAL7P1.132_3	MAL7P1.132	-0.33913	-0.38163	-0.68562	-0.02364	1.080125
aMAL7P1.132_2	MAL7P1.132	-0.33913	-0.38163	-0.68562	-0.02364	1.080125
aMAL7P1.132_1	MAL7P1.132	-0.33913	-0.38163	-0.68562	-0.02364	1.080125
aMAL7P1.132_0	MAL7P1.132	-0.33913	-0.38163	-0.68562	-0.02364	1.080125
intMAL7P1.134_3	MAL7P1.134	0.22448	0.33981	0.18829	-0.08489	-0.32081
aMAL7P1.134_4	MAL7P1.134	0.22448	0.33981	0.18829	-0.08489	-0.32081
aMAL7P1.134_3	MAL7P1.134	0.22448	0.33981	0.18829	-0.08489	-0.32081
aMAL7P1.134_2	MAL7P1.134	0.22448	0.33981	0.18829	-0.08489	-0.32081
aMAL7P1.134_1	MAL7P1.134	0.22448	0.33981	0.18829	-0.08489	-0.32081

aMAL7P1.134_0	MAL7P1.134	0.22448	0.33981	0.18829	-0.08489	-0.32081
aMAL7P1.133_0	MAL7P1.134	0.294107	0.618487	0.083537	-0.2522	-0.21715
intMAL7P1.137_2	MAL7P1.137	0.54921	-0.42205	-0.6879	-0.4661	0.09755
aMAL7P1.137_1	MAL7P1.137	0.54921	-0.42205	-0.6879	-0.4661	0.09755
aMAL7P1.137_0	MAL7P1.137	0.54921	-0.42205	-0.6879	-0.4661	0.09755
intMAL7P1.139_2	MAL7P1.138	-0.0922	0.211037	0.336937	0.212397	-0.1277
intMAL7P1.138_2	MAL7P1.138	0.465827	0.351887	0.010987	-0.25315	-0.32055
aMAL7P1.138_2	MAL7P1.138	0.465827	0.351887	0.010987	-0.25315	-0.32055
aMAL7P1.138_1	MAL7P1.138	0.465827	0.351887	0.010987	-0.25315	-0.32055
aMAL7P1.138_0	MAL7P1.138	0.465827	0.351887	0.010987	-0.25315	-0.32055
aMAL7P1.139_0	MAL7P1.139	-0.0922	0.211037	0.336937	0.212397	-0.1277
intMAL7P1.14_3	MAL7P1.14	0.562433	0.244813	0.605363	0.069303	-1.03015
aMAL7P1.14_0	MAL7P1.14	0.562433	0.244813	0.605363	0.069303	-1.03015
intMAL7P1.141_2	MAL7P1.141	1.21323	-0.29915	-2.1507	-2.2193	0.01015
aMAL7P1.141_0	MAL7P1.141	1.21323	-0.29915	-2.1507	-2.2193	0.01015
intMAL7P1.144_2	MAL7P1.144	-0.27111	0.900557	1.749887	0.511447	-1.75022
aMAL7P1.144_1	MAL7P1.144	-0.27111	0.900557	1.749887	0.511447	-1.75022
aMAL7P1.144_0	MAL7P1.144	-0.27111	0.900557	1.749887	0.511447	-1.75022
intMAL7P1.145_2	MAL7P1.145	0.847195	0.438695	-0.48257	-0.76305	-0.24981
aMAL7P1.145_1	MAL7P1.145	0.847195	0.438695	-0.48257	-0.76305	-0.24981
aMAL7P1.145_0	MAL7P1.145	0.847195	0.438695	-0.48257	-0.76305	-0.24981
intMAL7P1.146_3	MAL7P1.146	-0.06103	0.180278	-0.28103	-0.13167	0.458248
aMAL7P1.146_7	MAL7P1.146	-0.06103	0.180278	-0.28103	-0.13167	0.458248
aMAL7P1.146_6	MAL7P1.146	-0.06103	0.180278	-0.28103	-0.13167	0.458248
aMAL7P1.146_5	MAL7P1.146	-0.06103	0.180278	-0.28103	-0.13167	0.458248
aMAL7P1.146_4	MAL7P1.146	-0.06103	0.180278	-0.28103	-0.13167	0.458248
aMAL7P1.146_3	MAL7P1.146	-0.06103	0.180278	-0.28103	-0.13167	0.458248
aMAL7P1.146_2	MAL7P1.146	-0.06103	0.180278	-0.28103	-0.13167	0.458248
aMAL7P1.146_1	MAL7P1.146	-0.06103	0.180278	-0.28103	-0.13167	0.458248
aMAL7P1.146_0	MAL7P1.146	-0.06103	0.180278	-0.28103	-0.13167	0.458248
intMAL7P1.147_2	MAL7P1.147	-0.06813	0.008267	-0.17452	0.072057	0.456267
aMAL7P1.147_4	MAL7P1.147	-0.06813	0.008267	-0.17452	0.072057	0.456267
aMAL7P1.147_3	MAL7P1.147	-0.06813	0.008267	-0.17452	0.072057	0.456267
aMAL7P1.147_2	MAL7P1.147	-0.06813	0.008267	-0.17452	0.072057	0.456267
aMAL7P1.147_1	MAL7P1.147	-0.06813	0.008267	-0.17452	0.072057	0.456267
aMAL7P1.147_0	MAL7P1.147	-0.06813	0.008267	-0.17452	0.072057	0.456267
intMAL7P1.149_2	MAL7P1.149	-0.78341	-0.78398	-0.04205	0.653713	0.796143
aMAL7P1.149_1	MAL7P1.149	-0.78341	-0.78398	-0.04205	0.653713	0.796143
aMAL7P1.149_0	MAL7P1.149	-0.78341	-0.78398	-0.04205	0.653713	0.796143
intMAL7P1.15_2	MAL7P1.15	0.756127	0.847647	0.587257	-0.30515	-1.2213
aMAL7P1.15_6	MAL7P1.15	0.756127	0.847647	0.587257	-0.30515	-1.2213
aMAL7P1.15_5	MAL7P1.15	0.756127	0.847647	0.587257	-0.30515	-1.2213
aMAL7P1.15_4	MAL7P1.15	0.756127	0.847647	0.587257	-0.30515	-1.2213
aMAL7P1.15_3	MAL7P1.15	0.756127	0.847647	0.587257	-0.30515	-1.2213
aMAL7P1.15_2	MAL7P1.15	0.756127	0.847647	0.587257	-0.30515	-1.2213
aMAL7P1.15_1	MAL7P1.15	0.756127	0.847647	0.587257	-0.30515	-1.2213
aMAL7P1.15_0	MAL7P1.15	0.756127	0.847647	0.587257	-0.30515	-1.2213

intMAL7P1.150_2	MAL7P1.150	1.584627	1.169377	0.817247	-0.46241	-1.94054
aMAL7P1.150_1	MAL7P1.150	1.584627	1.169377	0.817247	-0.46241	-1.94054
aMAL7P1.150_0	MAL7P1.150	1.584627	1.169377	0.817247	-0.46241	-1.94054
intPF07_0112_2	MAL7P1.151	-0.59102	-0.6576	0.112525	0.850255	0.827705
intMAL7P1.151_2	MAL7P1.151	0.886093	0.651523	0.317953	-0.24329	-0.80886
aMAL7P1.151_1	MAL7P1.151	0.886093	0.651523	0.317953	-0.24329	-0.80886
aMAL7P1.151_0	MAL7P1.151	0.886093	0.651523	0.317953	-0.24329	-0.80886
intPF07_0113_2	MAL7P1.152	-1.03655	-0.55929	0.126813	0.703853	0.795953
intMAL7P1.152_2	MAL7P1.152	-0.33875	0.438243	0.644523	0.282643	-0.35258
aMAL7P1.152_1	MAL7P1.152	-0.33875	0.438243	0.644523	0.282643	-0.35258
aMAL7P1.152_0	MAL7P1.152	-0.33875	0.438243	0.644523	0.282643	-0.35258
intPF07_0118_2	MAL7P1.153	-0.77214	-1.00941	-0.45619	0.555393	1.253873
intMAL7P1.153_2	MAL7P1.153	-0.60627	-0.76463	-0.14274	0.52574	0.74263
aMAL7P1.153_1	MAL7P1.153	-0.60627	-0.76463	-0.14274	0.52574	0.74263
aMAL7P1.153_0	MAL7P1.153	-0.60627	-0.76463	-0.14274	0.52574	0.74263
intPF07_0119_2	MAL7P1.154	-1.27445	-0.98973	0.406477	1.062857	0.614067
aMAL7P1.154_0	MAL7P1.154	-0.70613	-1.08983	0.121925	0.918925	0.663675
intMAL7P1.155_2	MAL7P1.154a	-0.52456	-0.83521	-0.95395	0.027618	1.408378
intMAL7P1.154a_2	MAL7P1.154a	-0.56236	-0.88619	-0.42329	0.523667	1.158197
aMAL7P1.154a_0	MAL7P1.154a	-0.56236	-0.88619	-0.42329	0.523667	1.158197
aMAL7P1.155_3	MAL7P1.155	-0.52456	-0.83521	-0.95395	0.027618	1.408378
aMAL7P1.155_2	MAL7P1.155	-0.52456	-0.83521	-0.95395	0.027618	1.408378
aMAL7P1.155_1	MAL7P1.155	-0.52456	-0.83521	-0.95395	0.027618	1.408378
aMAL7P1.155_0	MAL7P1.155	-0.52456	-0.83521	-0.95395	0.027618	1.408378
intMAL7P1.156_2	MAL7P1.156	-0.85096	-0.64071	-0.24208	0.35758	0.83671
aMAL7P1.156_1	MAL7P1.156	-0.85096	-0.64071	-0.24208	0.35758	0.83671
aMAL7P1.156_0	MAL7P1.156	-0.85096	-0.64071	-0.24208	0.35758	0.83671
intMAL7P1.157_2	MAL7P1.157	-1.13624	-0.50881	0.455123	0.875543	0.518473
aMAL7P1.157_1	MAL7P1.157	-1.13624	-0.50881	0.455123	0.875543	0.518473
aMAL7P1.157_0	MAL7P1.157	-1.13624	-0.50881	0.455123	0.875543	0.518473
aMAL7P1.157a_1	MAL7P1.157a	0.869087	0.216577	0.060357	-0.12452	-0.44274
aMAL7P1.157a_0	MAL7P1.157a	0.869087	0.216577	0.060357	-0.12452	-0.44274
intMAL7P1.158_3	MAL7P1.158	0.463033	0.324843	0.074813	-0.10815	-0.23718
intMAL7P1.157a_2	MAL7P1.158	0.869087	0.216577	0.060357	-0.12452	-0.44274
aMAL7P1.158_0	MAL7P1.158	0.463033	0.324843	0.074813	-0.10815	-0.23718
intMAL7P1.159_2	MAL7P1.159	-0.92978	-0.81308	0.40741	1.01709	0.54658
aMAL7P1.159_0	MAL7P1.159	-0.92978	-0.81308	0.40741	1.01709	0.54658
intMAL7P1.16_2	MAL7P1.16	0.719413	0.657933	0.128033	-0.36517	-0.58367
aMAL7P1.16_4	MAL7P1.16	0.719413	0.657933	0.128033	-0.36517	-0.58367
aMAL7P1.16_3	MAL7P1.16	0.719413	0.657933	0.128033	-0.36517	-0.58367
aMAL7P1.16_2	MAL7P1.16	0.719413	0.657933	0.128033	-0.36517	-0.58367
aMAL7P1.16_1	MAL7P1.16	0.719413	0.657933	0.128033	-0.36517	-0.58367
aMAL7P1.16_0	MAL7P1.16	0.719413	0.657933	0.128033	-0.36517	-0.58367
aMAL7P1.160_0	MAL7P1.160-a	-0.8066	-1.13839	-0.21954	0.720115	0.992595
intMAL7P1.160_2	MAL7P1.160-b	-0.8066	-1.13839	-0.21954	0.720115	0.992595
intPF07_0124_2	MAL7P1.161	0.257367	-0.28581	-0.77062	-0.55654	0.301647
intMAL7P1.161_2	MAL7P1.161	0.054707	-0.41747	-0.83908	-0.73462	0.139827

aMAL7P1.161_0	MAL7P1.161	0.054707	-0.41747	-0.83908	-0.73462	0.139827
intMAL7P1.162_2	MAL7P1.162	-0.32734	-0.38944	-0.83792	-0.27114	0.97897
aMAL7P1.162_7	MAL7P1.162	-0.32734	-0.38944	-0.83792	-0.27114	0.97897
aMAL7P1.162_6	MAL7P1.162	-0.32734	-0.38944	-0.83792	-0.27114	0.97897
aMAL7P1.162_5	MAL7P1.162	-0.32734	-0.38944	-0.83792	-0.27114	0.97897
aMAL7P1.162_4	MAL7P1.162	-0.32734	-0.38944	-0.83792	-0.27114	0.97897
aMAL7P1.162_3	MAL7P1.162	-0.32734	-0.38944	-0.83792	-0.27114	0.97897
aMAL7P1.162_2	MAL7P1.162	-0.32734	-0.38944	-0.83792	-0.27114	0.97897
aMAL7P1.162_1	MAL7P1.162	-0.32734	-0.38944	-0.83792	-0.27114	0.97897
aMAL7P1.162_0	MAL7P1.162	-0.32734	-0.38944	-0.83792	-0.27114	0.97897
intMAL7P1.163_2	MAL7P1.163	-0.11954	0.1447	0.6396	0.3924	-0.4477
aMAL7P1.163_0	MAL7P1.163	-0.11954	0.1447	0.6396	0.3924	-0.4477
intMAL7P1.164_3	MAL7P1.164	0.574867	0.291177	-0.2231	-0.30556	-0.04684
aMAL7P1.164_1	MAL7P1.164	0.574867	0.291177	-0.2231	-0.30556	-0.04684
aMAL7P1.164_0	MAL7P1.164	0.574867	0.291177	-0.2231	-0.30556	-0.04684
intMAL7P1.167_2	MAL7P1.167	-0.55374	-1.21266	-1.15117	-0.03414	1.435405
aMAL7P1.167_4	MAL7P1.167	-0.55374	-1.21266	-1.15117	-0.03414	1.435405
aMAL7P1.167_3	MAL7P1.167	-0.55374	-1.21266	-1.15117	-0.03414	1.435405
aMAL7P1.167_2	MAL7P1.167	-0.55374	-1.21266	-1.15117	-0.03414	1.435405
aMAL7P1.167_1	MAL7P1.167	-0.55374	-1.21266	-1.15117	-0.03414	1.435405
aMAL7P1.167_0	MAL7P1.167	-0.55374	-1.21266	-1.15117	-0.03414	1.435405
intMAL7P1.17_2	MAL7P1.17	-0.55241	-0.99086	-2.013	-0.64252	2.250283
aMAL7P1.17_5	MAL7P1.17	-0.55241	-0.99086	-2.013	-0.64252	2.250283
aMAL7P1.17_4	MAL7P1.17	-0.55241	-0.99086	-2.013	-0.64252	2.250283
aMAL7P1.17_3	MAL7P1.17	-0.55241	-0.99086	-2.013	-0.64252	2.250283
aMAL7P1.17_2	MAL7P1.17	-0.55241	-0.99086	-2.013	-0.64252	2.250283
aMAL7P1.17_1	MAL7P1.17	-0.55241	-0.99086	-2.013	-0.64252	2.250283
aMAL7P1.17_0	MAL7P1.17	-0.55241	-0.99086	-2.013	-0.64252	2.250283
intMAL7P1.170_2	MAL7P1.170	1.699663	1.207703	0.533583	-1.44492	-2.90704
aMAL7P1.170_0	MAL7P1.170	1.699663	1.207703	0.533583	-1.44492	-2.90704
intMAL7P1.171_2	MAL7P1.171	0.071492	0.735792	1.501542	0.071792	-2.17846
aMAL7P1.171_3	MAL7P1.171	0.071492	0.735792	1.501542	0.071792	-2.17846
aMAL7P1.171_2	MAL7P1.171	0.071492	0.735792	1.501542	0.071792	-2.17846
aMAL7P1.171_1	MAL7P1.171	0.071492	0.735792	1.501542	0.071792	-2.17846
aMAL7P1.171_0	MAL7P1.171	0.071492	0.735792	1.501542	0.071792	-2.17846
intMAL7P1.172_2	MAL7P1.172	2.00505	1.69325	-0.12525	-1.87425	-2.12125
aMAL7P1.172_1	MAL7P1.172	2.00505	1.69325	-0.12525	-1.87425	-2.12125
aMAL7P1.172_0	MAL7P1.172	2.00505	1.69325	-0.12525	-1.87425	-2.12125
intMAL7P1.173_3	MAL7P1.173	1.960647	1.066787	-0.64257	-1.89209	-1.60245
aMAL7P1.173_0	MAL7P1.173	1.960647	1.066787	-0.64257	-1.89209	-1.60245
intMAL7P1.174_3	MAL7P1.174	0.408222	-0.04072	1.296752	0.824132	-1.3044
aMAL7P1.174_0	MAL7P1.174	0.408222	-0.04072	1.296752	0.824132	-1.3044
intMAL7P1.176_2	MAL7P1.176	2.362037	0.486837	-1.82243	-2.89423	-1.463
aMAL7P1.176_2	MAL7P1.176	2.362037	0.486837	-1.82243	-2.89423	-1.463
aMAL7P1.176_1	MAL7P1.176	2.362037	0.486837	-1.82243	-2.89423	-1.463
intMAL7P1.177_2	MAL7P1.177	0.130043	-0.20624	-0.16553	0.208193	0.434903
aMAL7P1.177_0	MAL7P1.177	0.130043	-0.20624	-0.16553	0.208193	0.434903

intMAL7P1.178_2	MAL7P1.178	2.33771	1.39695	-0.9504	-2.2096	-1.41995
aMAL7P1.178_1	MAL7P1.178	2.33771	1.39695	-0.9504	-2.2096	-1.41995
aMAL7P1.178_0	MAL7P1.178	2.33771	1.39695	-0.9504	-2.2096	-1.41995
intMAL7P1.18_2	MAL7P1.18	-0.71948	-0.96891	-1.85317	-0.50393	2.235467
aMAL7P1.18_2	MAL7P1.18	-0.71948	-0.96891	-1.85317	-0.50393	2.235467
aMAL7P1.18_1	MAL7P1.18	-0.71948	-0.96891	-1.85317	-0.50393	2.235467
aMAL7P1.18_0	MAL7P1.18	-0.71948	-0.96891	-1.85317	-0.50393	2.235467
intMAL7P1.186_2	MAL7P1.186					
intMAL7P1.19_2	MAL7P1.19	0.035115	0.183955	-0.1714	-0.23599	0.071165
aMAL7P1.19_5	MAL7P1.19	0.035115	0.183955	-0.1714	-0.23599	0.071165
aMAL7P1.19_4	MAL7P1.19	0.035115	0.183955	-0.1714	-0.23599	0.071165
aMAL7P1.19_3	MAL7P1.19	0.035115	0.183955	-0.1714	-0.23599	0.071165
aMAL7P1.19_2	MAL7P1.19	0.035115	0.183955	-0.1714	-0.23599	0.071165
aMAL7P1.19_1	MAL7P1.19	0.035115	0.183955	-0.1714	-0.23599	0.071165
aMAL7P1.19_0	MAL7P1.19	0.035115	0.183955	-0.1714	-0.23599	0.071165
intMAL7P1.20_2	MAL7P1.20	-0.17555	-0.51722	0.129947	0.531387	0.272557
aMAL7P1.20_0	MAL7P1.20	-0.17555	-0.51722	0.129947	0.531387	0.272557
intMAL7P1.201_2	MAL7P1.201	-0.24455	0.303287	0.597427	0.207407	-0.50045
aMAL7P1.201_1	MAL7P1.201	-0.24455	0.303287	0.597427	0.207407	-0.50045
aMAL7P1.201_0	MAL7P1.201	-0.24455	0.303287	0.597427	0.207407	-0.50045
intMAL7P1.202_2	MAL7P1.202	-0.01464	0.103197	-0.05864	-0.03902	0.122637
aMAL7P1.202_1	MAL7P1.202	-0.01464	0.103197	-0.05864	-0.03902	0.122637
aMAL7P1.202_0	MAL7P1.202	-0.01464	0.103197	-0.05864	-0.03902	0.122637
intMAL7P1.203_2	MAL7P1.203	-0.49973	-0.56486	-0.63903	0.01153	0.97886
aMAL7P1.203_1	MAL7P1.203	-0.49973	-0.56486	-0.63903	0.01153	0.97886
aMAL7P1.203_0	MAL7P1.203	-0.49973	-0.56486	-0.63903	0.01153	0.97886
intMAL7P1.204_3	MAL7P1.204	0.279115	0.634415	0.212835	-0.34795	-0.5822
aMAL7P1.204_2	MAL7P1.204	0.279115	0.634415	0.212835	-0.34795	-0.5822
aMAL7P1.204_1	MAL7P1.204	0.279115	0.634415	0.212835	-0.34795	-0.5822
aMAL7P1.204_0	MAL7P1.204	0.279115	0.634415	0.212835	-0.34795	-0.5822
intMAL7P1.206_2	MAL7P1.205	0.61753	-0.07385	0.32111	0.24949	-0.46955
intMAL7P1.205_2	MAL7P1.205	0.248	0.12302	0.56546	0.24154	-0.67502
aPF00_0001_1	MAL7P1.205	-0.8382	-0.06953	1.433803	1.109863	-0.73081
aPF00_0001_0	MAL7P1.205	-0.8382	-0.06953	1.433803	1.109863	-0.73081
aMAL7P1.205_1	MAL7P1.205	0.248	0.12302	0.56546	0.24154	-0.67502
aMAL7P1.205_0	MAL7P1.205	0.248	0.12302	0.56546	0.24154	-0.67502
aPFI0002w_0	MAL7P1.206	-0.45936	-0.2965	-0.06824	0.321425	0.562685
aPF00_0002_0	MAL7P1.206	0.120373	0.358943	0.386453	0.090933	-0.37293
aMAL7P1.206_0	MAL7P1.206	0.61753	-0.07385	0.32111	0.24949	-0.46955
intMAL7P1.207_2	MAL7P1.207	-0.70106	-1.12089	-0.89515	0.094733	1.269633
aPF00_0003_2	MAL7P1.207	-0.89817	-0.838	-0.70409	0.112055	1.199635
aPF00_0003_1	MAL7P1.207	-0.89817	-0.838	-0.70409	0.112055	1.199635
aPF00_0003_0	MAL7P1.207	-0.89817	-0.838	-0.70409	0.112055	1.199635
aMAL7P1.207_2	MAL7P1.207	-0.70106	-1.12089	-0.89515	0.094733	1.269633
aMAL7P1.207_1	MAL7P1.207	-0.70106	-1.12089	-0.89515	0.094733	1.269633
aMAL7P1.207_0	MAL7P1.207	-0.70106	-1.12089	-0.89515	0.094733	1.269633
intMAL7P1.208_2	MAL7P1.208	0.19993	-0.33366	-1.33492	-0.82608	0.91916

aMAL7P1.208_1	MAL7P1.208	0.19993	-0.33366	-1.33492	-0.82608	0.91916
aMAL7P1.208_0	MAL7P1.208	0.19993	-0.33366	-1.33492	-0.82608	0.91916
intMAL7P1.209_2	MAL7P1.209	-0.71989	-0.13853	1.17055	0.94795	-0.57647
aMAL7P1.209_1	MAL7P1.209	-0.71989	-0.13853	1.17055	0.94795	-0.57647
aMAL7P1.209_0	MAL7P1.209	-0.71989	-0.13853	1.17055	0.94795	-0.57647
intMAL7P1.21_2	MAL7P1.21	-1.15416	-1.14372	-0.75207	0.390233	1.586383
aMAL7P1.21_1	MAL7P1.21	-1.15416	-1.14372	-0.75207	0.390233	1.586383
aMAL7P1.21_0	MAL7P1.21	-1.15416	-1.14372	-0.75207	0.390233	1.586383
intMAL7P1.22_3	MAL7P1.22	1.630163	0.438863	-0.41024	-1.4041	-1.60487
aMAL7P1.22_2	MAL7P1.22	1.630163	0.438863	-0.41024	-1.4041	-1.60487
aMAL7P1.22_1	MAL7P1.22	1.630163	0.438863	-0.41024	-1.4041	-1.60487
aMAL7P1.22_0	MAL7P1.22	1.630163	0.438863	-0.41024	-1.4041	-1.60487
aMAL7P1.220_0	MAL7P1.220	0.95449	0.05256	0.01438	-0.37538	-0.87256
intMAL7P1.224_2	MAL7P1.224					
aMAL7P1.224_0	MAL7P1.224					
intMAL7P1.225_3	MAL7P1.225-a	-1.30692	-0.22344	2.018873	1.525793	-1.1259
aMAL7P1.225_0	MAL7P1.225-a	-1.30692	-0.22344	2.018873	1.525793	-1.1259
intMAL7P1.228_2	MAL7P1.228	1.09823	2.57284	0.77182	-1.16932	-1.81984
intMAL7P1.229_2	MAL7P1.229	-0.14365	-0.62676	-2.13666	-1.18034	1.776752
aMAL7P1.229_2	MAL7P1.229	-0.14365	-0.62676	-2.13666	-1.18034	1.776752
aMAL7P1.229_1	MAL7P1.229	-0.14365	-0.62676	-2.13666	-1.18034	1.776752
aMAL7P1.229_0	MAL7P1.229	-0.14365	-0.62676	-2.13666	-1.18034	1.776752
intMAL7P1.23_2	MAL7P1.23	-0.7847	-0.7065	-0.15773	0.527067	0.870337
aMAL7P1.23_1	MAL7P1.23	-0.7847	-0.7065	-0.15773	0.527067	0.870337
aMAL7P1.23_0	MAL7P1.23	-0.7847	-0.7065	-0.15773	0.527067	0.870337
intMAL7P1.24_2	MAL7P1.24	0.520567	0.312187	0.666137	0.125197	-0.99885
aMAL7P1.24_0	MAL7P1.24	0.520567	0.312187	0.666137	0.125197	-0.99885
intMAL7P1.25_2	MAL7P1.25	-0.76373	-0.06886	0.880463	0.766703	-0.25548
aMAL7P1.25_1	MAL7P1.25	-0.76373	-0.06886	0.880463	0.766703	-0.25548
aMAL7P1.25_0	MAL7P1.25	-0.76373	-0.06886	0.880463	0.766703	-0.25548
intPF07_0028_2	MAL7P1.26	1.17999	0.97854	-0.04858	-0.85542	-0.93504
aMAL7P1.26_0	MAL7P1.26					
intMAL7P1.27_2	MAL7P1.27	0.56242	0.66038	0.65375	-0.17675	-1.21088
aMAL7P1.27_1	MAL7P1.27	0.56242	0.66038	0.65375	-0.17675	-1.21088
aMAL7P1.27_0	MAL7P1.27	0.56242	0.66038	0.65375	-0.17675	-1.21088
intMAL7P1.28_2	MAL7P1.28	0.573617	0.702917	0.603667	-0.09183	-0.96708
aMAL7P1.28_1	MAL7P1.28	0.573617	0.702917	0.603667	-0.09183	-0.96708
aMAL7P1.28_0	MAL7P1.28	0.573617	0.702917	0.603667	-0.09183	-0.96708
intMAL7P1.29_2	MAL7P1.29	0.283438	-0.29321	-0.77245	-0.41288	0.506878
aMAL7P1.29_3	MAL7P1.29	0.283438	-0.29321	-0.77245	-0.41288	0.506878
aMAL7P1.29_2	MAL7P1.29	0.283438	-0.29321	-0.77245	-0.41288	0.506878
aMAL7P1.29_1	MAL7P1.29	0.283438	-0.29321	-0.77245	-0.41288	0.506878
aMAL7P1.29_0	MAL7P1.29	0.283438	-0.29321	-0.77245	-0.41288	0.506878
intPF07_0040_2	MAL7P1.29a					
intMAL7P1.29a_2	MAL7P1.29a					
aMALP1.29a_0	MAL7P1.29a	0.378743	-0.14171	-0.31458	-0.17576	0.092373
intMAL7P1.3_2	MAL7P1.3	1.901887	1.738577	0.675847	-1.07351	-2.32424

aMAL7P1.30_4	MAL7P1.30	0.263783	0.165303	-0.55851	-0.33763	0.539763
aMAL7P1.30_3	MAL7P1.30	0.263783	0.165303	-0.55851	-0.33763	0.539763
aMAL7P1.30_2	MAL7P1.30	0.263783	0.165303	-0.55851	-0.33763	0.539763
aMAL7P1.30_1	MAL7P1.30	0.263783	0.165303	-0.55851	-0.33763	0.539763
aMAL7P1.30_0	MAL7P1.30	0.263783	0.165303	-0.55851	-0.33763	0.539763
intMAL7P1.300_3	MAL7P1.300	-0.04505	0.441562	0.739222	0.401002	-0.44434
aMAL7P1.300_0	MAL7P1.300	-0.04505	0.441562	0.739222	0.401002	-0.44434
intMAL7P1.32_2	MAL7P1.31	0.20488	0.82258	0.53885	-0.06185	-0.60058
intMAL7P1.31_2	MAL7P1.31	0.1492	0.21599	0.93802	0.55098	-0.79899
aMAL7P1.31_0	MAL7P1.31	0.1492	0.21599	0.93802	0.55098	-0.79899
aMAL7P1.32_1	MAL7P1.32	0.20488	0.82258	0.53885	-0.06185	-0.60058
aMAL7P1.32_0	MAL7P1.32	0.20488	0.82258	0.53885	-0.06185	-0.60058
intMAL7P1.320_2	MAL7P1.320	0.015847	0.242757	0.679497	0.453837	-0.40842
aMAL7P1.320_0	MAL7P1.320	0.015847	0.242757	0.679497	0.453837	-0.40842
aMAL7P1.33_0	MAL7P1.33	-0.54021	-0.58893	0.63736	0.94264	0.03293
intMAL7P1.34_3	MAL7P1.34	1.045753	0.908233	-0.07996	-0.94938	-1.02057
aMAL7P1.34_0	MAL7P1.34	1.045753	0.908233	-0.07996	-0.94938	-1.02057
intMAL7P1.340_3	MAL7P1.340					
aMAL7P1.340_0	MAL7P1.340					
intMAL7P1.35_2	MAL7P1.35	0.754857	0.392737	-0.16121	-0.48145	-0.4164
aMAL7P1.35_1	MAL7P1.35	0.754857	0.392737	-0.16121	-0.48145	-0.4164
aMAL7P1.35_0	MAL7P1.35	0.754857	0.392737	-0.16121	-0.48145	-0.4164
intMAL7P1.350_2	MAL7P1.350	0.04796	0.1772	1.1311	0.5309	-1.1752
intMAL7P1.36_2	MAL7P1.36	0.024283	-0.07641	0.517313	0.436353	-0.35293
aMAL7P1.36_0	MAL7P1.36	0.024283	-0.07641	0.517313	0.436353	-0.35293
intMAL7P1.37_2	MAL7P1.37	-1.39595	-0.19025	0.505017	0.727317	0.503587
aMAL7P1.37_1	MAL7P1.37	-1.39595	-0.19025	0.505017	0.727317	0.503587
aMAL7P1.37_0	MAL7P1.37	-1.39595	-0.19025	0.505017	0.727317	0.503587
intMAL7P1.38_2	MAL7P1.38	-0.65097	-0.01917	0.62034	0.65616	0.05117
aMAL7P1.38_1	MAL7P1.38	-0.65097	-0.01917	0.62034	0.65616	0.05117
aMAL7P1.38_0	MAL7P1.38	-0.65097	-0.01917	0.62034	0.65616	0.05117
intMAL7P1.4_2	MAL7P1.4	1.713003	1.858983	0.337543	-1.23988	-1.81032
intMAL7P1.5_2	MAL7P1.5	2.010963	0.857093	-0.25424	-1.1948	-1.42503
aMAL7P1.5_0	MAL7P1.5	2.010963	0.857093	-0.25424	-1.1948	-1.42503
intMAL7P1.58_2	MAL7P1.58	-1.53141	0.034047	1.929417	1.412917	-0.88471
aMAL7P1.58_0	MAL7P1.58	-1.53141	0.034047	1.929417	1.412917	-0.88471
intMAL7P1.6_2	MAL7P1.6	0.860067	0.962647	0.522717	-0.38938	-1.18731
aMAL7P1.6_0	MAL7P1.6	0.860067	0.962647	0.522717	-0.38938	-1.18731
intMAL7P1.59_2	MAL7P1.61	0.828333	1.176353	0.487293	-0.47363	-1.09569
aMAL7P1.63_0	MAL7P1.61	1.19884	1.11781	0.04887	-0.70587	-0.80281
intMAL7P1.65_2	MAL7P1.64	1.164297	0.611037	-0.92806	-1.2526	-0.1582
intMAL7P1.64_2	MAL7P1.64	0.338522	0.061382	-0.06653	-0.05897	-0.03839
aMAL7P1.64_0	MAL7P1.64	0.338522	0.061382	-0.06653	-0.05897	-0.03839
intMAL7P1.66_2	MAL7P1.65	0.205707	-0.02554	-0.14391	0.012747	0.197377
aMAL7P1.65_1	MAL7P1.65	1.164297	0.611037	-0.92806	-1.2526	-0.1582
aMAL7P1.65_0	MAL7P1.65	1.164297	0.611037	-0.92806	-1.2526	-0.1582
aMAL7P1.66_1	MAL7P1.66	0.205707	-0.02554	-0.14391	0.012747	0.197377

aMAL7P1.66_0	MAL7P1.66	0.205707	-0.02554	-0.14391	0.012747	0.197377
intMAL7P1.67_3	MAL7P1.67	-0.76941	-0.27398	0.143453	0.539213	0.619643
aMAL7P1.67_0	MAL7P1.67	-0.76941	-0.27398	0.143453	0.539213	0.619643
intMAL7P1.68_3	MAL7P1.68	1.093287	0.317087	-0.18293	-0.46673	-0.51775
aMAL7P1.68_1	MAL7P1.68	1.093287	0.317087	-0.18293	-0.46673	-0.51775
aMAL7P1.68_0	MAL7P1.68	1.093287	0.317087	-0.18293	-0.46673	-0.51775
intPF07_0057_2	MAL7P1.69	-0.10893	-0.16987	0.14323	0.35127	0.18737
intMAL7P1.69_2	MAL7P1.69					
aMAL7P1.69_0	MAL7P1.69					
intMAL7P1.7_3	MAL7P1.7	2.221397	0.415017	-0.56703	-0.91363	-0.79468
aMAL7P1.7_1	MAL7P1.7	2.221397	0.415017	-0.56703	-0.91363	-0.79468
aMAL7P1.7_0	MAL7P1.7	2.221397	0.415017	-0.56703	-0.91363	-0.79468
intMAL7P1.73_2	MAL7P1.73	-0.55394	-1.01826	-0.74746	0.315297	1.349097
aMAL7P1.73_1	MAL7P1.73	-0.55394	-1.01826	-0.74746	0.315297	1.349097
aMAL7P1.73_0	MAL7P1.73	-0.55394	-1.01826	-0.74746	0.315297	1.349097
aMAL7P1.74_0	MAL7P1.74	-0.83358	-0.97808	0.121167	0.877167	0.719417
intPF07_0062_2	MAL7P1.75	-0.9975	-1.02099	0.12723	0.92727	0.80949
intMAL7P1.74_2	MAL7P1.75	-0.83358	-0.97808	0.121167	0.877167	0.719417
aMAL7P1.75_0	MAL7P1.75	-1.25913	-1.35035	0.359693	1.353973	0.891013
aMAL7P1.76_1	MAL7P1.76	1.330037	1.417307	0.619877	-0.85204	-1.91297
aMAL7P1.76_0	MAL7P1.76	1.330037	1.417307	0.619877	-0.85204	-1.91297
intMAL7P1.77_3	MAL7P1.77	-2.06812	-1.7959	-0.00244	1.592777	1.885737
aMAL7P1.77_1	MAL7P1.77	-2.06812	-1.7959	-0.00244	1.592777	1.885737
aMAL7P1.77_0	MAL7P1.77	-2.06812	-1.7959	-0.00244	1.592777	1.885737
intMAL7P1.79_2	MAL7P1.78	-0.15878	-0.50006	-0.05537	0.352207	0.349397
intMAL7P1.78_2	MAL7P1.78	0.142353	-0.33077	-0.71997	-0.01637	1.027433
aMAL7P1.78_0	MAL7P1.78	0.142353	-0.33077	-0.71997	-0.01637	1.027433
intPF07_0068_2	MAL7P1.79	-0.87748	-0.3721	0.44545	0.71255	0.2966
aMAL7P1.79_1	MAL7P1.79	-0.15878	-0.50006	-0.05537	0.352207	0.349397
aMAL7P1.79_0	MAL7P1.79	-0.15878	-0.50006	-0.05537	0.352207	0.349397
intMAL7P1.81_2	MAL7P1.81	0.257277	0.287627	0.477237	0.087097	-0.63829
aMAL7P1.81_0	MAL7P1.81	0.257277	0.287627	0.477237	0.087097	-0.63829
intMAL7P1.82_2	MAL7P1.82	-0.63776	-0.94767	-0.88116	0.221497	1.528507
aMAL7P1.82_1	MAL7P1.82	-0.63776	-0.94767	-0.88116	0.221497	1.528507
aMAL7P1.82_0	MAL7P1.82	-0.63776	-0.94767	-0.88116	0.221497	1.528507
intMAL7P1.83_2	MAL7P1.83	-0.49232	-0.24189	0.15404	0.46496	0.41789
aMAL7P1.83_1	MAL7P1.83	-0.49232	-0.24189	0.15404	0.46496	0.41789
aMAL7P1.83_0	MAL7P1.83	-0.49232	-0.24189	0.15404	0.46496	0.41789
intMAL7P1.86_2	MAL7P1.84	0.08778	0.00061	0.02978	-0.24178	-0.44611
intMAL7P1.84_2	MAL7P1.84	-0.39181	-0.36041	-0.53292	-0.22974	0.522747
aMAL7P1.84_1	MAL7P1.84	-0.39181	-0.36041	-0.53292	-0.22974	0.522747
aMAL7P1.84_0	MAL7P1.84	-0.39181	-0.36041	-0.53292	-0.22974	0.522747
aMAL7P1.86_1	MAL7P1.86	0.08778	0.00061	0.02978	-0.24178	-0.44611
aMAL7P1.86_0	MAL7P1.86	0.08778	0.00061	0.02978	-0.24178	-0.44611
intMAL7P1.87_2	MAL7P1.87	-0.7553	-1.00798	-1.04228	0.016117	1.514817
aMAL7P1.87_1	MAL7P1.87	-0.7553	-1.00798	-1.04228	0.016117	1.514817
aMAL7P1.87_0	MAL7P1.87	-0.7553	-1.00798	-1.04228	0.016117	1.514817

aMAL7P1.88_1	MAL7P1.88	-0.09604	0.201703	0.954603	0.555063	-0.75104
aMAL7P1.88_0	MAL7P1.88	-0.09604	0.201703	0.954603	0.555063	-0.75104
intMAL7P1.88_2	MAL7P1.89	-0.09604	0.201703	0.954603	0.555063	-0.75104
aMAL7P1.89_8	MAL7P1.89	-0.51624	-0.48315	-0.30586	0.204365	0.717255
aMAL7P1.89_7	MAL7P1.89	-0.51624	-0.48315	-0.30586	0.204365	0.717255
aMAL7P1.89_6	MAL7P1.89	-0.51624	-0.48315	-0.30586	0.204365	0.717255
aMAL7P1.89_5	MAL7P1.89	-0.51624	-0.48315	-0.30586	0.204365	0.717255
aMAL7P1.89_4	MAL7P1.89	-0.51624	-0.48315	-0.30586	0.204365	0.717255
aMAL7P1.89_3	MAL7P1.89	-0.51624	-0.48315	-0.30586	0.204365	0.717255
aMAL7P1.89_2	MAL7P1.89	-0.51624	-0.48315	-0.30586	0.204365	0.717255
aMAL7P1.89_1	MAL7P1.89	-0.51624	-0.48315	-0.30586	0.204365	0.717255
aMAL7P1.89_0	MAL7P1.89	-0.51624	-0.48315	-0.30586	0.204365	0.717255
intMAL7P1.91_2	MAL7P1.91	-0.88057	-0.8158	-0.26389	0.562228	1.076798
intMAL7P1.89_2	MAL7P1.91	-0.51624	-0.48315	-0.30586	0.204365	0.717255
aMAL7P1.91_4	MAL7P1.91	-0.88057	-0.8158	-0.26389	0.562228	1.076798
aMAL7P1.91_3	MAL7P1.91	-0.88057	-0.8158	-0.26389	0.562228	1.076798
aMAL7P1.91_2	MAL7P1.91	-0.88057	-0.8158	-0.26389	0.562228	1.076798
aMAL7P1.91_1	MAL7P1.91	-0.88057	-0.8158	-0.26389	0.562228	1.076798
aMAL7P1.91_0	MAL7P1.91	-0.88057	-0.8158	-0.26389	0.562228	1.076798
intMAL7P1.92_2	MAL7P1.92	0.09992	0.31488	-0.3163	-0.74108	-0.35512
aMAL7P1.92_3	MAL7P1.92	0.09992	0.31488	-0.3163	-0.74108	-0.35512
aMAL7P1.92_2	MAL7P1.92	0.09992	0.31488	-0.3163	-0.74108	-0.35512
aMAL7P1.92_1	MAL7P1.92	0.09992	0.31488	-0.3163	-0.74108	-0.35512
aMAL7P1.92_0	MAL7P1.92	0.09992	0.31488	-0.3163	-0.74108	-0.35512
intMAL7P1.93_2	MAL7P1.93	-0.11142	0.076072	0.087742	-0.00216	-0.07624
aMAL7P1.93_0	MAL7P1.93	-0.11142	0.076072	0.087742	-0.00216	-0.07624
intMAL7P1.94_2	MAL7P1.94	-0.59974	-0.58233	0.557163	0.864503	0.086993
aMAL7P1.94_0	MAL7P1.94	-0.59974	-0.58233	0.557163	0.864503	0.086993
intMAL7P1.95_2	MAL7P1.95	-0.35368	-0.6746	-0.33456	0.07106	0.3881
aMAL7P1.95_1	MAL7P1.95	-0.35368	-0.6746	-0.33456	0.07106	0.3881
aMAL7P1.95_0	MAL7P1.95	-0.35368	-0.6746	-0.33456	0.07106	0.3881
aMAL7P1.97_0	MAL7P1.97					
intMAL7P1.98_2	MAL7P1.98	-0.51399	1.17996	0.99358	0.22042	-0.53296
aMAL7P1.98_0	MAL7P1.98	-0.51399	1.17996	0.99358	0.22042	-0.53296
intMAL7P1.100_2	MAL7P1.99	-0.59648	-0.35448	-0.07232	0.316142	0.569302
aMAL7P1.99_0	MAL7P1.99	-0.84267	-0.91964	0.046297	0.836037	0.836977
intMAL8P1.1_2	MAL8P1.1	-0.60612	0.03795	0.04577	-0.06255	0.04961
aMAL8P1.1_2	MAL8P1.1	-0.60612	0.03795	0.04577	-0.06255	0.04961
aMAL8P1.1_0	MAL8P1.1	-0.60612	0.03795	0.04577	-0.06255	0.04961
intPF08_0006_2	MAL8P1.10	-0.43718	-0.32509	1.25817	1.12583	-0.68891
intMAL8P1.10_2	MAL8P1.10	0.730697	0.417527	0.279447	-0.06511	-0.57519
aMAL8P1.10_0	MAL8P1.10	0.730697	0.417527	0.279447	-0.06511	-0.57519
intMAL8P1.100_2	MAL8P1.100	-0.01485	0.099247	0.796507	0.506827	-0.62891
aMAL8P1.100_1	MAL8P1.100	-0.01485	0.099247	0.796507	0.506827	-0.62891
aMAL8P1.100_0	MAL8P1.100	-0.01485	0.099247	0.796507	0.506827	-0.62891
intMAL8P1.102_2	MAL8P1.101	-0.40701	0.120047	0.407897	0.380937	0.039287
aMAL8P1.101_1	MAL8P1.101	-1.0299	-0.94998	-0.43802	0.454857	1.195317

aMAL8P1.101_0	MAL8P1.101	-1.0299	-0.94998	-0.43802	0.454857	1.195317
intMAL8P1.101_2	MAL8P1.102	-1.0299	-0.94998	-0.43802	0.454857	1.195317
aMAL8P1.102_1	MAL8P1.102	-0.40701	0.120047	0.407897	0.380937	0.039287
aMAL8P1.102_0	MAL8P1.102	-0.40701	0.120047	0.407897	0.380937	0.039287
intMAL8P1.103_2	MAL8P1.103	-0.59469	0.36347	0.70456	0.44544	-0.18297
aMAL8P1.103_1	MAL8P1.103	-0.59469	0.36347	0.70456	0.44544	-0.18297
aMAL8P1.103_0	MAL8P1.103	-0.59469	0.36347	0.70456	0.44544	-0.18297
intMAL8P1.104_2	MAL8P1.104	0.547535	0.118675	-0.58085	-0.60205	0.058435
aMAL8P1.104_2	MAL8P1.104	0.547535	0.118675	-0.58085	-0.60205	0.058435
aMAL8P1.104_1	MAL8P1.104	0.547535	0.118675	-0.58085	-0.60205	0.058435
aMAL8P1.104_0	MAL8P1.104	0.547535	0.118675	-0.58085	-0.60205	0.058435
intPF08_0093_2	MAL8P1.105	-0.35685	-0.25525	-0.32499	0.146327	0.764087
aMAL8P1.105_1	MAL8P1.105	0.266113	0.403923	0.211153	-0.03549	-0.25976
aMAL8P1.105_0	MAL8P1.105	0.266113	0.403923	0.211153	-0.03549	-0.25976
intMAL8P1.106_2	MAL8P1.106-a	-0.07995	-0.54517	-0.82814	-0.27684	0.766192
aMAL8P1.106_0	MAL8P1.106-a	-0.07995	-0.54517	-0.82814	-0.27684	0.766192
intPF08_0095_2	MAL8P1.107	-0.55198	-0.0511	1.03495	0.89605	-0.4034
aMAL8P1.107_0	MAL8P1.107	0.223013	0.269943	0.603123	0.366543	-0.45028
intMAL8P1.108_2	MAL8P1.108	0.104923	0.321373	0.386243	0.085423	-0.40071
aMAL8P1.108_0	MAL8P1.108	0.104923	0.321373	0.386243	0.085423	-0.40071
intMAL8P1.109_3	MAL8P1.109	0.048837	-1.33467	-1.73214	-0.65802	1.282507
aMAL8P1.109_1	MAL8P1.109	0.048837	-1.33467	-1.73214	-0.65802	1.282507
aMAL8P1.109_0	MAL8P1.109	0.048837	-1.33467	-1.73214	-0.65802	1.282507
intMAL8P1.11_2	MAL8P1.11	-0.08118	0.451888	-0.07327	-0.30962	-0.00177
aMAL8P1.11_2	MAL8P1.11	-0.08118	0.451888	-0.07327	-0.30962	-0.00177
aMAL8P1.11_1	MAL8P1.11	-0.08118	0.451888	-0.07327	-0.30962	-0.00177
aMAL8P1.11_0	MAL8P1.11	-0.08118	0.451888	-0.07327	-0.30962	-0.00177
intPF08_0098_2	MAL8P1.110	-0.60439	-0.72806	0.50611	0.80989	0.00306
intMAL8P1.110_2	MAL8P1.110	0.140767	0.049167	0.086157	-0.01782	-0.19883
aMAL8P1.110_0	MAL8P1.110	0.140767	0.049167	0.086157	-0.01782	-0.19883
intMAL8P1.111_2	MAL8P1.111	0.74952	0.1909	-0.8243	-0.6887	0.3556
aMAL8P1.111_1	MAL8P1.111	0.74952	0.1909	-0.8243	-0.6887	0.3556
aMAL8P1.111_0	MAL8P1.111	0.74952	0.1909	-0.8243	-0.6887	0.3556
aMAL8P1.112_1	MAL8P1.112	-0.22276	-0.44719	-0.77862	-0.41204	0.585527
aMAL8P1.112_0	MAL8P1.112	-0.22276	-0.44719	-0.77862	-0.41204	0.585527
intMAL8P1.113_2	MAL8P1.113	-1.37487	-0.80852	0.381557	0.999617	0.719177
aMAL8P1.113_8	MAL8P1.113	-1.37487	-0.80852	0.381557	0.999617	0.719177
aMAL8P1.113_7	MAL8P1.113	-1.37487	-0.80852	0.381557	0.999617	0.719177
aMAL8P1.113_6	MAL8P1.113	-1.37487	-0.80852	0.381557	0.999617	0.719177
aMAL8P1.113_5	MAL8P1.113	-1.37487	-0.80852	0.381557	0.999617	0.719177
aMAL8P1.113_4	MAL8P1.113	-1.37487	-0.80852	0.381557	0.999617	0.719177
aMAL8P1.113_3	MAL8P1.113	-1.37487	-0.80852	0.381557	0.999617	0.719177
aMAL8P1.113_2	MAL8P1.113	-1.37487	-0.80852	0.381557	0.999617	0.719177
aMAL8P1.113_1	MAL8P1.113	-1.37487	-0.80852	0.381557	0.999617	0.719177
aMAL8P1.113_0	MAL8P1.113	-1.37487	-0.80852	0.381557	0.999617	0.719177
intMAL8P1.114_2	MAL8P1.114	0.21121	1.23295	0.4416	-0.3866	-0.63795
aMAL8P1.114_0	MAL8P1.114	0.21121	1.23295	0.4416	-0.3866	-0.63795

intMAL8P1.13_2	MAL8P1.12	-0.18352	-0.49644	-1.56012	-0.84622	1.321103
aMAL8P1.12_1	MAL8P1.12	-0.30341	-0.13793	-0.82562	-0.42304	0.888267
aMAL8P1.12_0	MAL8P1.12	-0.30341	-0.13793	-0.82562	-0.42304	0.888267
aMAL8P1.121_0	MAL8P1.121	-0.18686	-0.08174	-0.14254	0.042203	0.332403
intMAL8P1.122_2	MAL8P1.122	0.54573	-0.16839	-0.90807	-0.73917	0.26187
intMAL8P1.121_2	MAL8P1.122	-0.18686	-0.08174	-0.14254	0.042203	0.332403
aMAL8P1.122_0	MAL8P1.122	0.54573	-0.16839	-0.90807	-0.73917	0.26187
intMAL8P1.123_2	MAL8P1.123	-0.56774	-0.11928	0.057068	0.342488	0.518838
aMAL8P1.123_2	MAL8P1.123	-0.56774	-0.11928	0.057068	0.342488	0.518838
aMAL8P1.123_1	MAL8P1.123	-0.56774	-0.11928	0.057068	0.342488	0.518838
aMAL8P1.123_0	MAL8P1.123	-0.56774	-0.11928	0.057068	0.342488	0.518838
aMAL8P1.124_1	MAL8P1.124	0.27422	-0.29658	0.45616	0.45834	-0.43792
aMAL8P1.124_0	MAL8P1.124	0.27422	-0.29658	0.45616	0.45834	-0.43792
intMAL8P1.125_3	MAL8P1.125	-0.45178	-0.10012	0.568152	0.589312	-0.07381
intMAL8P1.124_2	MAL8P1.125	0.27422	-0.29658	0.45616	0.45834	-0.43792
aMAL8P1.125_0	MAL8P1.125	-0.45178	-0.10012	0.568152	0.589312	-0.07381
intMAL8P1.127_1	MAL8P1.126	-0.20979	0.231675	0.508515	0.332985	-0.20843
aMAL8P1.126_1	MAL8P1.126	0.169727	0.456347	-0.5782	-0.85646	0.022987
aMAL8P1.126_0	MAL8P1.126	0.169727	0.456347	-0.5782	-0.85646	0.022987
aMAL8P1.127_1	MAL8P1.127	-0.20979	0.231675	0.508515	0.332985	-0.20843
aMAL8P1.127_0	MAL8P1.127	-0.20979	0.231675	0.508515	0.332985	-0.20843
intMAL8P1.128_3	MAL8P1.128	-0.94742	-0.59994	0.223873	0.874793	0.817603
aMAL8P1.128_0	MAL8P1.128	-0.94742	-0.59994	0.223873	0.874793	0.817603
intMAL8P1.12_2	MAL8P1.13	-0.30341	-0.13793	-0.82562	-0.42304	0.888267
aMAL8P1.13_1	MAL8P1.13	-0.18352	-0.49644	-1.56012	-0.84622	1.321103
aMAL8P1.13_0	MAL8P1.13	-0.18352	-0.49644	-1.56012	-0.84622	1.321103
intPF08_0111_2	MAL8P1.130	0.254667	0.703687	0.008127	-0.42179	-0.26335
aMAL8P1.130_1	MAL8P1.130	0.20608	-0.10942	0.57508	0.57992	-0.32608
aMAL8P1.130_0	MAL8P1.130	0.20608	-0.10942	0.57508	0.57992	-0.32608
intPF08_0112_2	MAL8P1.131	0.55421	0.01596	-0.23967	-0.04083	0.20404
aMAL8P1.131_0	MAL8P1.131	0.44065	0.30177	0.58097	0.26403	-0.61477
intPF08_0114_2	MAL8P1.132	0.42699	0.59137	-0.07841	-0.33225	-0.10271
intMAL8P1.132_2	MAL8P1.132	1.482267	1.418647	-0.2493	-1.20536	-0.89931
aMAL8P1.132_1	MAL8P1.132	1.482267	1.418647	-0.2493	-1.20536	-0.89931
aMAL8P1.132_0	MAL8P1.132	1.482267	1.418647	-0.2493	-1.20536	-0.89931
intMAL8P1.134_2	MAL8P1.133	-0.36245	-0.68609	-1.36867	-0.41511	1.549972
aMAL8P1.133_0	MAL8P1.133	-0.12114	-0.59529	-0.70193	-0.01607	0.90629
intMAL8P1.133_2	MAL8P1.134	-0.12114	-0.59529	-0.70193	-0.01607	0.90629
aMAL8P1.134_2	MAL8P1.134	-0.36245	-0.68609	-1.36867	-0.41511	1.549972
aMAL8P1.134_1	MAL8P1.134	-0.36245	-0.68609	-1.36867	-0.41511	1.549972
aMAL8P1.134_0	MAL8P1.134	-0.36245	-0.68609	-1.36867	-0.41511	1.549972
intMAL8P1.135_2	MAL8P1.135	-0.30149	-0.73205	-1.6939	-0.6176	1.79155
aMAL8P1.135_1	MAL8P1.135	-0.30149	-0.73205	-1.6939	-0.6176	1.79155
aMAL8P1.135_0	MAL8P1.135	-0.30149	-0.73205	-1.6939	-0.6176	1.79155
aMAL8P1.136_0	MAL8P1.136	0.70028	0.13509	0.11557	0.01043	-0.33959
aMAL8P1.136_1	MAL8P1.136a	0.70028	0.13509	0.11557	0.01043	-0.33959
intMAL8P1.137_2	MAL8P1.137	0.484343	0.085283	-1.21507	-1.17677	0.357883

aMAL8P1.137_1	MAL8P1.137	0.484343	0.085283	-1.21507	-1.17677	0.357883
aMAL8P1.137_0	MAL8P1.137	0.484343	0.085283	-1.21507	-1.17677	0.357883
intMAL8P1.138_2	MAL8P1.138	-0.40952	-0.9264	-0.6167	0.350363	1.175063
aMAL8P1.138_0	MAL8P1.138	-0.40952	-0.9264	-0.6167	0.350363	1.175063
intMAL8P1.139_2	MAL8P1.139	-0.69848	-0.45169	-0.18258	0.34339	0.78153
aMAL8P1.139_8	MAL8P1.139	-0.69848	-0.45169	-0.18258	0.34339	0.78153
aMAL8P1.139_7	MAL8P1.139	-0.69848	-0.45169	-0.18258	0.34339	0.78153
aMAL8P1.139_6	MAL8P1.139	-0.69848	-0.45169	-0.18258	0.34339	0.78153
aMAL8P1.139_5	MAL8P1.139	-0.69848	-0.45169	-0.18258	0.34339	0.78153
aMAL8P1.139_4	MAL8P1.139	-0.69848	-0.45169	-0.18258	0.34339	0.78153
aMAL8P1.139_3	MAL8P1.139	-0.69848	-0.45169	-0.18258	0.34339	0.78153
aMAL8P1.139_2	MAL8P1.139	-0.69848	-0.45169	-0.18258	0.34339	0.78153
aMAL8P1.139_1	MAL8P1.139	-0.69848	-0.45169	-0.18258	0.34339	0.78153
aMAL8P1.139_0	MAL8P1.139	-0.69848	-0.45169	-0.18258	0.34339	0.78153
aMAL8P1.14_1	MAL8P1.14	-0.94925	-0.57977	0.076043	0.621623	0.722933
aMAL8P1.14_0	MAL8P1.14	-0.94925	-0.57977	0.076043	0.621623	0.722933
aMAL8P1.140_1	MAL8P1.140	-0.48127	-0.74361	-0.53602	0.325357	1.135447
aMAL8P1.140_0	MAL8P1.140	-0.48127	-0.74361	-0.53602	0.325357	1.135447
intMAL8P1.141_3	MAL8P1.141	1.511493	0.891013	0.270983	-0.88682	-1.73685
intMAL8P1.140_2	MAL8P1.141	-0.48127	-0.74361	-0.53602	0.325357	1.135447
aMAL8P1.141_1	MAL8P1.141	1.511493	0.891013	0.270983	-0.88682	-1.73685
aMAL8P1.141_0	MAL8P1.141	1.511493	0.891013	0.270983	-0.88682	-1.73685
intMAL8P1.142_2	MAL8P1.142	-1.00944	-0.2788	0.1411	0.6099	0.8068
aMAL8P1.142_0	MAL8P1.142	-1.00944	-0.2788	0.1411	0.6099	0.8068
aMAL8P1.143_2	MAL8P1.143-a	0.306412	0.347772	-0.48946	-0.58888	0.143732
aMAL8P1.143_1	MAL8P1.143-a	0.306412	0.347772	-0.48946	-0.58888	0.143732
aMAL8P1.143_0	MAL8P1.143-a	0.306412	0.347772	-0.48946	-0.58888	0.143732
intMAL8P1.143_2	MAL8P1.143-b	0.306412	0.347772	-0.48946	-0.58888	0.143732
intMAL8P1.144_3	MAL8P1.144	0.88368	0.10727	-0.38038	-0.98795	-1.00761
aMAL8P1.144_2	MAL8P1.144	0.88368	0.10727	-0.38038	-0.98795	-1.00761
aMAL8P1.144_1	MAL8P1.144	0.88368	0.10727	-0.38038	-0.98795	-1.00761
aMAL8P1.144_0	MAL8P1.144	0.88368	0.10727	-0.38038	-0.98795	-1.00761
intMAL8P1.145_2	MAL8P1.145	-0.81831	-0.80941	-0.25892	0.326257	0.697247
aMAL8P1.145_1	MAL8P1.145	-0.81831	-0.80941	-0.25892	0.326257	0.697247
aMAL8P1.145_0	MAL8P1.145	-0.81831	-0.80941	-0.25892	0.326257	0.697247
intMAL8P1.146_3	MAL8P1.146	-0.38447	-1.17416	-1.89269	-0.51565	2.041823
aMAL8P1.146_1	MAL8P1.146	-0.38447	-1.17416	-1.89269	-0.51565	2.041823
aMAL8P1.146_0	MAL8P1.146	-0.38447	-1.17416	-1.89269	-0.51565	2.041823
intPF08_0128_2	MAL8P1.148	0.22016	0.14521	-0.06116	-0.09584	-0.00821
aMAL8P1.148_0	MAL8P1.148	0.147907	0.333457	0.703087	0.361247	-0.57412
aMAL8P1.149_1	MAL8P1.149	-0.59906	-0.81873	-0.74656	0.007223	1.033393
aMAL8P1.149_0	MAL8P1.149	-0.59906	-0.81873	-0.74656	0.007223	1.033393
intPF08_0011_2	MAL8P1.15	-0.728	-0.00032	0.57714	0.55286	0.01365
aMAL8P1.15_1	MAL8P1.15	-0.61201	0.12756	0.40863	0.45337	0.21994
aMAL8P1.15_0	MAL8P1.15	-0.61201	0.12756	0.40863	0.45337	0.21994
intMAL8P1.149_2	MAL8P1.150	-0.59906	-0.81873	-0.74656	0.007223	1.033393
aMAL8P1.150_3	MAL8P1.150	0.16773	0.0066	-1.69696	-1.43679	0.9044

aMAL8P1.150_2	MAL8P1.150	0.16773	0.0066	-1.69696	-1.43679	0.9044
aMAL8P1.150_1	MAL8P1.150	0.16773	0.0066	-1.69696	-1.43679	0.9044
aMAL8P1.150_0	MAL8P1.150	0.16773	0.0066	-1.69696	-1.43679	0.9044
intMAL8P1.151_2	MAL8P1.151	0.117077	0.696937	-0.36837	-0.73318	0.001177
intMAL8P1.150_2	MAL8P1.151	0.16773	0.0066	-1.69696	-1.43679	0.9044
aMAL8P1.151_2	MAL8P1.151	0.117077	0.696937	-0.36837	-0.73318	0.001177
aMAL8P1.151_1	MAL8P1.151	0.117077	0.696937	-0.36837	-0.73318	0.001177
aMAL8P1.151_0	MAL8P1.151	0.117077	0.696937	-0.36837	-0.73318	0.001177
intPF08_0130_2	MAL8P1.152	0.783297	0.885037	0.860437	-0.1941	-1.5397
intMAL8P1.152_3	MAL8P1.152	0.425113	0.577443	0.552613	-0.07795	-0.88678
aMAL8P1.152_1	MAL8P1.152	0.425113	0.577443	0.552613	-0.07795	-0.88678
aMAL8P1.152_0	MAL8P1.152	0.425113	0.577443	0.552613	-0.07795	-0.88678
intMAL8P1.153_2	MAL8P1.153	-0.33176	-1.04748	-1.7278	-0.5191	1.801255
aMAL8P1.153_3	MAL8P1.153	-0.33176	-1.04748	-1.7278	-0.5191	1.801255
aMAL8P1.153_2	MAL8P1.153	-0.33176	-1.04748	-1.7278	-0.5191	1.801255
aMAL8P1.153_1	MAL8P1.153	-0.33176	-1.04748	-1.7278	-0.5191	1.801255
aMAL8P1.153_0	MAL8P1.153	-0.33176	-1.04748	-1.7278	-0.5191	1.801255
intMAL8P1.154_2	MAL8P1.154	0.085378	0.616208	0.028618	-0.50126	-0.42201
aPF08_0133_2	MAL8P1.154	0.231568	0.766158	-0.11349	-0.78195	-0.53127
aPF08_0133_1	MAL8P1.154	0.231568	0.766158	-0.11349	-0.78195	-0.53127
aPF08_0133_0	MAL8P1.154	0.231568	0.766158	-0.11349	-0.78195	-0.53127
aMAL8P1.155_0	MAL8P1.154					
aMAL8P1.154_3	MAL8P1.154	0.085378	0.616208	0.028618	-0.50126	-0.42201
aMAL8P1.154_2	MAL8P1.154	0.085378	0.616208	0.028618	-0.50126	-0.42201
aMAL8P1.154_1	MAL8P1.154	0.085378	0.616208	0.028618	-0.50126	-0.42201
aMAL8P1.154_0	MAL8P1.154	0.085378	0.616208	0.028618	-0.50126	-0.42201
intMAL8P1.156_2	MAL8P1.156	0.028577	0.109597	0.313067	0.265767	-0.11176
aMAL8P1.156_1	MAL8P1.156	0.028577	0.109597	0.313067	0.265767	-0.11176
aMAL8P1.156_0	MAL8P1.156	0.028577	0.109597	0.313067	0.265767	-0.11176
intMAL8P1.157_2	MAL8P1.157	0.254578	0.277458	0.317918	0.107748	-0.32112
aMAL8P1.157_2	MAL8P1.157	0.254578	0.277458	0.317918	0.107748	-0.32112
aMAL8P1.157_1	MAL8P1.157	0.254578	0.277458	0.317918	0.107748	-0.32112
aMAL8P1.157_0	MAL8P1.157	0.254578	0.277458	0.317918	0.107748	-0.32112
intMAL8P1.16_2	MAL8P1.16	-0.40301	-0.28692	-0.03966	0.384997	0.593257
aMAL8P1.16_0	MAL8P1.16	-0.40301	-0.28692	-0.03966	0.384997	0.593257
intMAL8P1.160_2	MAL8P1.160	0.199388	0.098478	0.150438	-0.09084	-0.41288
aMAL8P1.160_0	MAL8P1.160	0.199388	0.098478	0.150438	-0.09084	-0.41288
intMAL8P1.161_2	MAL8P1.161	-0.03856	0.423222	0.434012	-0.04705	-0.57126
aMAL8P1.161_0	MAL8P1.161	-0.03856	0.423222	0.434012	-0.04705	-0.57126
intMAL8P1.162_2	MAL8P1.162	-0.21414	0.117207	0.004837	-0.201	-0.16087
aMAL8P1.162_0	MAL8P1.162	-0.21414	0.117207	0.004837	-0.201	-0.16087
intMAL8P1.163_2	MAL8P1.163	-0.2064	-0.46541	-0.8055	-0.14072	1.024192
aMAL8P1.17_1	MAL8P1.17	-1.08386	-0.06974	0.161963	0.600703	0.918903
aMAL8P1.17_0	MAL8P1.17	-1.08386	-0.06974	0.161963	0.600703	0.918903
intMAL8P1.18_2	MAL8P1.18	-1.19409	-0.63452	-0.01328	0.652397	0.977967
aMAL8P1.18_2	MAL8P1.18	-1.19409	-0.63452	-0.01328	0.652397	0.977967
aMAL8P1.18_1	MAL8P1.18	-1.19409	-0.63452	-0.01328	0.652397	0.977967

aMAL8P1.18_0	MAL8P1.18	-1.19409	-0.63452	-0.01328	0.652397	0.977967
intPF08_0018_2	MAL8P1.19	-0.16955	0.104425	0.324315	0.206015	-0.15943
intMAL8P1.19_2	MAL8P1.19	0.648537	0.682317	0.587607	-0.13327	-1.03848
aMAL8P1.19_1	MAL8P1.19	0.648537	0.682317	0.587607	-0.13327	-1.03848
aMAL8P1.19_0	MAL8P1.19	0.648537	0.682317	0.587607	-0.13327	-1.03848
intMAL8P1.2_2	MAL8P1.2	-0.40368	1.439383	1.872483	0.336683	-1.83372
aMAL8P1.2_1	MAL8P1.2	-0.40368	1.439383	1.872483	0.336683	-1.83372
aMAL8P1.2_0	MAL8P1.2	-0.40368	1.439383	1.872483	0.336683	-1.83372
intMAL8P1.20_2	MAL8P1.20	-0.4277	-0.761	-0.46251	0.357673	1.019663
aMAL8P1.20_1	MAL8P1.20	-0.4277	-0.761	-0.46251	0.357673	1.019663
aMAL8P1.20_0	MAL8P1.20	-0.4277	-0.761	-0.46251	0.357673	1.019663
aMAL8P1.200_0	MAL8P1.200	0.639893	0.817513	0.232963	-0.4803	-0.81585
intMAL8P1.201_2	MAL8P1.201	0.709347	0.692777	-0.74404	-1.00962	0.075057
intMAL8P1.200_2	MAL8P1.201	0.639893	0.817513	0.232963	-0.4803	-0.81585
aMAL8P1.201_1	MAL8P1.201	0.709347	0.692777	-0.74404	-1.00962	0.075057
aMAL8P1.201_0	MAL8P1.201	0.709347	0.692777	-0.74404	-1.00962	0.075057
intMAL8P1.202_2	MAL8P1.202	0.199303	-0.27953	0.305553	0.002113	-0.80981
aMAL8P1.202_0	MAL8P1.202	0.199303	-0.27953	0.305553	0.002113	-0.80981
intMAL8P1.203_2	MAL8P1.203	-1.03361	-1.36549	-1.19544	0.255433	2.008993
aMAL8P1.203_2	MAL8P1.203	-1.03361	-1.36549	-1.19544	0.255433	2.008993
aMAL8P1.203_1	MAL8P1.203	-1.03361	-1.36549	-1.19544	0.255433	2.008993
aMAL8P1.203_0	MAL8P1.203	-1.03361	-1.36549	-1.19544	0.255433	2.008993
intMAL8P1.204_3	MAL8P1.204	1.42831	0.69995	-0.8094	-1.1556	-0.25895
aMAL8P1.204_1	MAL8P1.204	1.42831	0.69995	-0.8094	-1.1556	-0.25895
aMAL8P1.204_0	MAL8P1.204	1.42831	0.69995	-0.8094	-1.1556	-0.25895
intMAL8P1.205_2	MAL8P1.205	-0.53767	0.26085	0.32804	0.27396	0.16265
aMAL8P1.205_1	MAL8P1.205	-0.53767	0.26085	0.32804	0.27396	0.16265
aMAL8P1.205_0	MAL8P1.205	-0.53767	0.26085	0.32804	0.27396	0.16265
intMAL8P1.206_3	MAL8P1.206	-2.35902	-1.0129	2.422297	2.416037	-0.70576
aMAL8P1.206_0	MAL8P1.206	-2.35902	-1.0129	2.422297	2.416037	-0.70576
intMAL8P1.209_2	MAL8P1.209	-0.42242	0.195083	-0.38167	-0.28267	0.546583
intMAL8P1.21_2	MAL8P1.21	-0.92207	-0.13367	0.646323	0.601343	-0.06467
aMAL8P1.21_0	MAL8P1.21	-0.92207	-0.13367	0.646323	0.601343	-0.06467
intMAL8P1.210_2	MAL8P1.210	0.908977	0.504187	0.085907	-0.51561	-0.89777
intMAL8P1.211_2	MAL8P1.211					
intMAL8P1.320_2	MAL8P1.212					
intMAL8P1.213_2	MAL8P1.213					
intMAL8P1.215_2	MAL8P1.214					
intMAL8P1.22_2	MAL8P1.22	-0.56507	-1.15285	-0.51118	0.524997	1.144447
aMAL8P1.22_0	MAL8P1.22	-0.56507	-1.15285	-0.51118	0.524997	1.144447
intMAL8P1.23_2	MAL8P1.23	0.016787	0.467587	0.222377	-0.07019	-0.20686
aMAL8P1.23_9	MAL8P1.23	0.016787	0.467587	0.222377	-0.07019	-0.20686
aMAL8P1.23_8	MAL8P1.23	0.016787	0.467587	0.222377	-0.07019	-0.20686
aMAL8P1.23_7	MAL8P1.23	0.016787	0.467587	0.222377	-0.07019	-0.20686
aMAL8P1.23_6	MAL8P1.23	0.016787	0.467587	0.222377	-0.07019	-0.20686
aMAL8P1.23_5	MAL8P1.23	0.016787	0.467587	0.222377	-0.07019	-0.20686
aMAL8P1.23_4	MAL8P1.23	0.016787	0.467587	0.222377	-0.07019	-0.20686

aMAL8P1.23_3	MAL8P1.23	0.016787	0.467587	0.222377	-0.07019	-0.20686
aMAL8P1.23_2	MAL8P1.23	0.016787	0.467587	0.222377	-0.07019	-0.20686
aMAL8P1.23_12	MAL8P1.23	0.016787	0.467587	0.222377	-0.07019	-0.20686
aMAL8P1.23_11	MAL8P1.23	0.016787	0.467587	0.222377	-0.07019	-0.20686
aMAL8P1.23_10	MAL8P1.23	0.016787	0.467587	0.222377	-0.07019	-0.20686
aMAL8P1.23_1	MAL8P1.23	0.016787	0.467587	0.222377	-0.07019	-0.20686
aMAL8P1.23_0	MAL8P1.23	0.016787	0.467587	0.222377	-0.07019	-0.20686
intMAL8P1.24_2	MAL8P1.24	-1.00736	-0.10322	1.107933	0.878733	-0.44612
aMAL8P1.24_1	MAL8P1.24	-1.00736	-0.10322	1.107933	0.878733	-0.44612
aMAL8P1.24_0	MAL8P1.24	-1.00736	-0.10322	1.107933	0.878733	-0.44612
intPF08_0024_2	MAL8P1.25	-0.0732	-0.15819	-0.03498	0.11951	0.16234
aMAL8P1.25_1	MAL8P1.25	-0.10217	-0.34682	-0.29151	0.182068	0.610378
aMAL8P1.25_0	MAL8P1.25	-0.10217	-0.34682	-0.29151	0.182068	0.610378
intMAL8P1.26_2	MAL8P1.25a	-0.39105	0.00426	0.21298	0.32802	0.23474
aMAL8P1.25a_0	MAL8P1.25a	-0.57366	-0.43972	0.309942	0.650282	0.305272
intMAL8P1.25a_2	MAL8P1.26	-0.57366	-0.43972	0.309942	0.650282	0.305272
aMAL8P1.26_0	MAL8P1.26	-0.39105	0.00426	0.21298	0.32802	0.23474
aMAL8P1.27_0	MAL8P1.27	-1.13573	-1.23037	-0.43126	0.75026	1.47437
intMAL8P1.27_2	MAL8P1.28	-1.13573	-1.23037	-0.43126	0.75026	1.47437
aMAL8P1.28_0	MAL8P1.28					
intMAL8P1.29_2	MAL8P1.29	-0.10319	-0.27141	-0.68302	-0.18321	0.830853
intMAL8P1.28_2	MAL8P1.29					
aMAL8P1.29_2	MAL8P1.29	-0.10319	-0.27141	-0.68302	-0.18321	0.830853
aMAL8P1.29_1	MAL8P1.29	-0.10319	-0.27141	-0.68302	-0.18321	0.830853
aMAL8P1.29_0	MAL8P1.29	-0.10319	-0.27141	-0.68302	-0.18321	0.830853
intMAL8P1.3_2	MAL8P1.3	-0.20003	-0.05884	1.634187	1.013147	-1.44882
aMAL8P1.3_0	MAL8P1.3	-0.20003	-0.05884	1.634187	1.013147	-1.44882
intMAL8P1.30_2	MAL8P1.30	-0.73031	-1.10895	-0.5566	0.5556	1.35795
aMAL8P1.30_1	MAL8P1.30	-0.73031	-1.10895	-0.5566	0.5556	1.35795
aMAL8P1.30_0	MAL8P1.30	-0.73031	-1.10895	-0.5566	0.5556	1.35795
intMAL8P1.300_3	MAL8P1.300	-0.26652	-0.65636	-0.24927	0.181607	0.380697
aMAL8P1.300_0	MAL8P1.300	-0.26652	-0.65636	-0.24927	0.181607	0.380697
intMAL8P1.31_2	MAL8P1.31					
aMAL8P1.31_0	MAL8P1.31					
intMAL8P1.310_2	MAL8P1.310					
aMAL8P1.310_0	MAL8P1.310					
intMAL8P1.33_2	MAL8P1.32	-0.85089	-1.04604	-0.32693	0.53793	0.99254
intMAL8P1.32_2	MAL8P1.32	-0.84529	-0.71104	0.65158	1.10592	0.27204
aMAL8P1.32_1	MAL8P1.32	-0.84529	-0.71104	0.65158	1.10592	0.27204
aMAL8P1.32_0	MAL8P1.32	-0.84529	-0.71104	0.65158	1.10592	0.27204
aMAL8P1.33_1	MAL8P1.33	-0.85089	-1.04604	-0.32693	0.53793	0.99254
aMAL8P1.33_0	MAL8P1.33	-0.85089	-1.04604	-0.32693	0.53793	0.99254
intMAL8P1.34_2	MAL8P1.34	-1.18085	-0.88275	0.668257	1.256077	0.464087
aMAL8P1.34_1	MAL8P1.34	-1.18085	-0.88275	0.668257	1.256077	0.464087
aMAL8P1.34_0	MAL8P1.34	-1.18085	-0.88275	0.668257	1.256077	0.464087
intPF08_0030_2	MAL8P1.35	-0.34413	-0.64966	0.10098	0.55272	0.32556
aMAL8P1.35_1	MAL8P1.35	-1.18145	-0.57026	1.13202	1.27798	-0.14474

aMAL8P1.35_0	MAL8P1.35	-1.18145	-0.57026	1.13202	1.27798	-0.14474
intPF08_0032_2	MAL8P1.36	-0.53961	-0.13842	0.210097	0.538237	0.509757
intMAL8P1.37_2	MAL8P1.36	0.618043	1.069763	0.116473	-0.58881	-0.5896
intMAL8P1.36_3	MAL8P1.36	-0.12934	0.26169	1.19213	0.72487	-0.88069
aMAL8P1.36_0	MAL8P1.36	-0.12934	0.26169	1.19213	0.72487	-0.88069
aMAL8P1.37_1	MAL8P1.37	0.618043	1.069763	0.116473	-0.58881	-0.5896
aMAL8P1.37_0	MAL8P1.37	0.618043	1.069763	0.116473	-0.58881	-0.5896
aMAL8P1.38_1	MAL8P1.38	-0.05594	-0.0313	0.163617	0.118217	-0.12486
aMAL8P1.38_0	MAL8P1.38	-0.05594	-0.0313	0.163617	0.118217	-0.12486
intMAL8P1.4_3	MAL8P1.4	1.839277	1.125617	0.191507	-1.39167	-2.30128
aMAL8P1.4_1	MAL8P1.4	1.839277	1.125617	0.191507	-1.39167	-2.30128
aMAL8P1.4_0	MAL8P1.4	1.839277	1.125617	0.191507	-1.39167	-2.30128
intMAL8P1.40_2	MAL8P1.40	-0.23839	0.207947	0.505107	0.330227	-0.21261
aMAL8P1.40_0	MAL8P1.40	-0.23839	0.207947	0.505107	0.330227	-0.21261
intMAL8P1.42_2	MAL8P1.41	0.468647	0.500767	0.067967	-0.21663	-0.27193
intMAL8P1.41_2	MAL8P1.41	0.824543	0.666263	0.739473	-0.02281	-1.2206
aMAL8P1.41_0	MAL8P1.41	0.824543	0.666263	0.739473	-0.02281	-1.2206
aMAL8P1.42_1	MAL8P1.42	0.468647	0.500767	0.067967	-0.21663	-0.27193
aMAL8P1.42_0	MAL8P1.42	0.468647	0.500767	0.067967	-0.21663	-0.27193
aMAL8P1.43_0	MAL8P1.43	0.263142	0.331842	0.440082	0.077102	-0.55766
intMAL8P1.45_2	MAL8P1.45	0.47345	0.47025	0.10475	-0.33825	-0.54375
intMAL8P1.43_2	MAL8P1.45	0.263142	0.331842	0.440082	0.077102	-0.55766
aMAL8P1.45_1	MAL8P1.45	0.47345	0.47025	0.10475	-0.33825	-0.54375
aMAL8P1.45_0	MAL8P1.45	0.47345	0.47025	0.10475	-0.33825	-0.54375
intMAL8P1.46_2	MAL8P1.46					
aMAL8P1.46_0	MAL8P1.46					
intMAL8P1.47_2	MAL8P1.47	0.926927	0.820867	-0.30748	-0.72168	-0.28053
aMAL8P1.47_1	MAL8P1.47	0.926927	0.820867	-0.30748	-0.72168	-0.28053
aMAL8P1.47_0	MAL8P1.47	0.926927	0.820867	-0.30748	-0.72168	-0.28053
intMAL8P1.48_2	MAL8P1.48	0.39682	0.29991	0.57167	0.21833	-0.65291
aMAL8P1.48_0	MAL8P1.48	0.39682	0.29991	0.57167	0.21833	-0.65291
aMAL8P1.49_2	MAL8P1.49	1.299698	1.071158	0.644688	-0.32947	-1.39526
aMAL8P1.49_1	MAL8P1.49	1.299698	1.071158	0.644688	-0.32947	-1.39526
aMAL8P1.49_0	MAL8P1.49	1.299698	1.071158	0.644688	-0.32947	-1.39526
aMAL8P1.50_0	MAL8P1.50	-0.55884	-0.5648	0.429083	0.839583	0.282463
intMAL8P1.51_2	MAL8P1.51	-0.53204	-0.05876	0.252027	0.513307	0.446097
aMAL8P1.51_0	MAL8P1.51	-0.53204	-0.05876	0.252027	0.513307	0.446097
intMAL8P1.52_3	MAL8P1.52	0.57042	-0.42008	-0.98783	-0.66717	0.34008
aMAL8P1.52_0	MAL8P1.52	0.57042	-0.42008	-0.98783	-0.66717	0.34008
intMAL8P1.53_2	MAL8P1.53	-1.39971	-1.90896	-0.48884	1.071003	1.696623
aMAL8P1.53_1	MAL8P1.53	-1.39971	-1.90896	-0.48884	1.071003	1.696623
aMAL8P1.53_0	MAL8P1.53	-1.39971	-1.90896	-0.48884	1.071003	1.696623
intMAL8P1.55_2	MAL8P1.54	-0.71172	-0.7369	0.234793	0.802373	0.519063
intMAL8P1.54_2	MAL8P1.54	0.4341	0.25118	-0.9412	-0.73269	0.64926
aMAL8P1.54_2	MAL8P1.54	0.4341	0.25118	-0.9412	-0.73269	0.64926
aMAL8P1.54_1	MAL8P1.54	0.4341	0.25118	-0.9412	-0.73269	0.64926
aMAL8P1.54_0	MAL8P1.54	0.4341	0.25118	-0.9412	-0.73269	0.64926

aMAL8P1.55_1	MAL8P1.55	-0.71172	-0.7369	0.234793	0.802373	0.519063
aMAL8P1.55_0	MAL8P1.55	-0.71172	-0.7369	0.234793	0.802373	0.519063
intMAL8P1.56_2	MAL8P1.56	-0.40438	-0.77558	-1.42033	-0.18283	1.947417
aMAL8P1.56_1	MAL8P1.56	-0.40438	-0.77558	-1.42033	-0.18283	1.947417
aMAL8P1.56_0	MAL8P1.56	-0.40438	-0.77558	-1.42033	-0.18283	1.947417
intMAL8P1.57_2	MAL8P1.57	0.59941	0.48543	0.04215	-0.46265	-0.64743
aMAL8P1.57_1	MAL8P1.57	0.59941	0.48543	0.04215	-0.46265	-0.64743
aMAL8P1.57_0	MAL8P1.57	0.59941	0.48543	0.04215	-0.46265	-0.64743
intMAL8P1.58_2	MAL8P1.58	-0.29758	0.115417	0.042667	0.036667	0.152917
aMAL8P1.58_1	MAL8P1.58	-0.29758	0.115417	0.042667	0.036667	0.152917
aMAL8P1.58_0	MAL8P1.58	-0.29758	0.115417	0.042667	0.036667	0.152917
intMAL8P1.59_2	MAL8P1.59	-1.28662	-0.29894	0.594373	0.770793	0.296103
aMAL8P1.59_1	MAL8P1.59	-1.28662	-0.29894	0.594373	0.770793	0.296103
aMAL8P1.59_0	MAL8P1.59	-1.28662	-0.29894	0.594373	0.770793	0.296103
intMAL8P1.6_2	MAL8P1.6	-0.29022	-0.86798	-0.84598	0.045717	1.137617
aMAL8P1.6_0	MAL8P1.6	-0.29022	-0.86798	-0.84598	0.045717	1.137617
aMAL8P1.60_2	MAL8P1.60	-0.58177	-0.49451	-0.15274	0.196345	0.438785
aMAL8P1.60_1	MAL8P1.60	-0.58177	-0.49451	-0.15274	0.196345	0.438785
aMAL8P1.60_0	MAL8P1.60	-0.58177	-0.49451	-0.15274	0.196345	0.438785
intMAL8P1.61_2	MAL8P1.61	-0.29068	-0.86008	-0.69057	0.115907	0.963417
aMAL8P1.61_1	MAL8P1.61	-0.29068	-0.86008	-0.69057	0.115907	0.963417
aMAL8P1.61_0	MAL8P1.61	-0.29068	-0.86008	-0.69057	0.115907	0.963417
intMAL8P1.62_2	MAL8P1.62	-1.14298	-0.99812	-0.25276	0.41876	0.80712
aMAL8P1.62_0	MAL8P1.62	-1.14298	-0.99812	-0.25276	0.41876	0.80712
intMAL8P1.63_2	MAL8P1.63	-0.65838	-0.0791	1.59769	1.07731	-1.1464
aMAL8P1.63_1	MAL8P1.63	-0.65838	-0.0791	1.59769	1.07731	-1.1464
aMAL8P1.63_0	MAL8P1.63	-0.65838	-0.0791	1.59769	1.07731	-1.1464
intPF08_0053_2	MAL8P1.64	0.776023	0.736873	0.709723	-0.2405	-1.42933
intMAL8P1.64_3	MAL8P1.64	0.94986	1.07468	0.34388	-0.63838	-1.18818
aMAL8P1.64_1	MAL8P1.64	0.94986	1.07468	0.34388	-0.63838	-1.18818
aMAL8P1.64_0	MAL8P1.64	0.94986	1.07468	0.34388	-0.63838	-1.18818
intMAL8P1.65_3	MAL8P1.65	0.486817	0.208917	-0.39657	-0.34609	0.184917
aMAL8P1.65_1	MAL8P1.65	0.486817	0.208917	-0.39657	-0.34609	0.184917
aMAL8P1.65_0	MAL8P1.65	0.486817	0.208917	-0.39657	-0.34609	0.184917
intMAL8P1.66_2	MAL8P1.66	0.87951	0.12546	-0.69542	-1.05658	-0.51946
aMAL8P1.66_0	MAL8P1.66	0.87951	0.12546	-0.69542	-1.05658	-0.51946
intMAL8P1.67_2	MAL8P1.67	0.322023	0.376903	0.759203	0.080463	-1.12724
aMAL8P1.67_0	MAL8P1.67	0.322023	0.376903	0.759203	0.080463	-1.12724
intMAL8P1.68_2	MAL8P1.68	1.233147	0.679487	-0.58311	-1.12315	-0.57375
aMAL8P1.68_1	MAL8P1.68	1.233147	0.679487	-0.58311	-1.12315	-0.57375
aMAL8P1.68_0	MAL8P1.68	1.233147	0.679487	-0.58311	-1.12315	-0.57375
intMAL8P1.69_2	MAL8P1.69	-0.54422	-0.87189	-0.28772	0.607057	1.038227
aMAL8P1.69_0	MAL8P1.69	-0.54422	-0.87189	-0.28772	0.607057	1.038227
intMAL8P1.7_2	MAL8P1.7	0.195143	-0.47506	-1.32288	-0.59868	1.145503
aMAL8P1.7_3	MAL8P1.7	0.195143	-0.47506	-1.32288	-0.59868	1.145503
aMAL8P1.7_2	MAL8P1.7	0.195143	-0.47506	-1.32288	-0.59868	1.145503
aMAL8P1.7_1	MAL8P1.7	0.195143	-0.47506	-1.32288	-0.59868	1.145503

aMAL8P1.7_0	MAL8P1.7	0.195143	-0.47506	-1.32288	-0.59868	1.145503
intMAL8P1.70_2	MAL8P1.70	1.447277	0.018627	-1.62201	-2.24065	-0.85729
aMAL8P1.70_1	MAL8P1.70	1.447277	0.018627	-1.62201	-2.24065	-0.85729
aMAL8P1.70_0	MAL8P1.70	1.447277	0.018627	-1.62201	-2.24065	-0.85729
intMAL8P1.71_2	MAL8P1.71	0.505763	-0.17084	-0.38107	-0.31477	-0.056
aMAL8P1.71_1	MAL8P1.71	0.505763	-0.17084	-0.38107	-0.31477	-0.056
aMAL8P1.71_0	MAL8P1.71	0.505763	-0.17084	-0.38107	-0.31477	-0.056
intMAL8P1.72_2	MAL8P1.72	0.478483	0.060583	-0.71766	-0.44768	0.525083
aMAL8P1.72_0	MAL8P1.72	0.478483	0.060583	-0.71766	-0.44768	0.525083
aMAL8P1.73_1	MAL8P1.73	-0.65105	-0.98573	-2.36854	-1.10996	2.23723
aMAL8P1.73_0	MAL8P1.73	-0.65105	-0.98573	-2.36854	-1.10996	2.23723
aMAL8P1.74_1	MAL8P1.74	-0.68271	-0.86945	0.229893	0.897773	0.573113
aMAL8P1.74_0	MAL8P1.74	-0.68271	-0.86945	0.229893	0.897773	0.573113
aMAL8P1.75_1	MAL8P1.75a	-0.65752	-0.0249	1.045053	0.818613	-0.48544
aMAL8P1.75_0	MAL8P1.75a	-0.65752	-0.0249	1.045053	0.818613	-0.48544
intPF08_0062_2	MAL8P1.76	-0.38141	0.16614	0.02467	-7.00E-05	0.19256
aMAL8P1.76_0	MAL8P1.76	0.067032	-0.15327	0.107062	0.112732	-0.15171
intMAL8P1.77_2	MAL8P1.77	-0.85098	-0.09208	0.69366	0.75034	0.07608
aMAL8P1.77_0	MAL8P1.77	-0.85098	-0.09208	0.69366	0.75034	0.07608
intMAL8P1.78_2	MAL8P1.78					
aMAL8P1.78_0	MAL8P1.78					
intMAL8P1.80_2	MAL8P1.79	1.024627	1.527867	0.185267	-0.89793	-1.00953
aMAL8P1.79_0	MAL8P1.79	-0.24442	-0.60287	-0.79474	-0.06892	1.033207
intMAL8P1.8_2	MAL8P1.8	-0.53299	-0.94706	-1.37863	-0.26271	1.679723
aMAL8P1.8_1	MAL8P1.8	-0.53299	-0.94706	-1.37863	-0.26271	1.679723
aMAL8P1.8_0	MAL8P1.8	-0.53299	-0.94706	-1.37863	-0.26271	1.679723
intMAL8P1.79_2	MAL8P1.80	-0.24442	-0.60287	-0.79474	-0.06892	1.033207
aMAL8P1.80_1	MAL8P1.80	1.024627	1.527867	0.185267	-0.89793	-1.00953
aMAL8P1.80_0	MAL8P1.80	1.024627	1.527867	0.185267	-0.89793	-1.00953
intMAL8P1.81_1	MAL8P1.81	0.681493	1.138543	-0.88508	-1.40226	0.051123
aMAL8P1.81_0	MAL8P1.81	0.681493	1.138543	-0.88508	-1.40226	0.051123
intMAL8P1.82_2	MAL8P1.82	-1.29662	-1.19889	-0.5039	0.451122	1.233442
aMAL8P1.82_2	MAL8P1.82	-1.29662	-1.19889	-0.5039	0.451122	1.233442
aMAL8P1.82_1	MAL8P1.82	-1.29662	-1.19889	-0.5039	0.451122	1.233442
aMAL8P1.82_0	MAL8P1.82	-1.29662	-1.19889	-0.5039	0.451122	1.233442
intMAL8P1.83_2	MAL8P1.83	0.301917	0.174417	0.410167	0.252167	-0.35808
aMAL8P1.83_0	MAL8P1.83	0.301917	0.174417	0.410167	0.252167	-0.35808
aMAL8P1.84_1	MAL8P1.84	-0.45768	-0.75361	-1.00604	0.05704	1.58011
aMAL8P1.84_0	MAL8P1.84	-0.45768	-0.75361	-1.00604	0.05704	1.58011
intMAL8P1.85_2	MAL8P1.85	-1.05154	-0.5963	0.089103	0.709563	0.853963
intMAL8P1.84_2	MAL8P1.85	-0.45768	-0.75361	-1.00604	0.05704	1.58011
aMAL8P1.85_0	MAL8P1.85	-1.05154	-0.5963	0.089103	0.709563	0.853963
intPF08_0070_2	MAL8P1.86	0.801162	0.217902	0.039342	-0.43383	-0.84466
aMAL8P1.86_0	MAL8P1.86	-0.7214	-0.47498	0.75895	0.97505	-0.00502
intMAL8P1.90_2	MAL8P1.88					
aMAL8P1.88_1	MAL8P1.88	2.047717	1.373427	-0.5086	-2.12406	-2.02059
aMAL8P1.88_0	MAL8P1.88	2.047717	1.373427	-0.5086	-2.12406	-2.02059

intMAL8P1.9_2	MAL8P1.9	-0.40053	0.030643	0.302223	0.357443	0.136023
aMAL8P1.9_0	MAL8P1.9	-0.40053	0.030643	0.302223	0.357443	0.136023
aMAL8P1.90_0	MAL8P1.90					
intMAL8P1.91_2	MAL8P1.91	1.051383	0.347093	-1.48969	-1.29065	0.676073
intMAL8P1.88_2	MAL8P1.91	2.047717	1.373427	-0.5086	-2.12406	-2.02059
aMAL8P1.91_1	MAL8P1.91	1.051383	0.347093	-1.48969	-1.29065	0.676073
aMAL8P1.91_0	MAL8P1.91	1.051383	0.347093	-1.48969	-1.29065	0.676073
intMAL8P1.92_2	MAL8P1.92	1.1234	0.749	0.29951	-0.60151	-1.3265
aMAL8P1.92_1	MAL8P1.92	1.1234	0.749	0.29951	-0.60151	-1.3265
aMAL8P1.92_0	MAL8P1.92	1.1234	0.749	0.29951	-0.60151	-1.3265
intMAL8P1.93_2	MAL8P1.93	0.772687	0.031547	0.059907	-0.13857	-0.55421
aMAL8P1.93_0	MAL8P1.93	0.772687	0.031547	0.059907	-0.13857	-0.55421
intMAL8P1.95_2	MAL8P1.95					
aMAL8P1.95_0	MAL8P1.95					
intMAL8P1.96_2	MAL8P1.96	-0.4286	-0.332	-0.37949	0.01649	0.642
aMAL8P1.96_0	MAL8P1.96	-0.4286	-0.332	-0.37949	0.01649	0.642
intMAL8P1.97_2	MAL8P1.97	2.085413	1.014753	-0.73059	-1.59425	-1.07059
intMAL8P1.380_2	MAL8P1.97					
aMAL8P1.97_2	MAL8P1.97	2.085413	1.014753	-0.73059	-1.59425	-1.07059
aMAL8P1.97_1	MAL8P1.97	2.085413	1.014753	-0.73059	-1.59425	-1.07059
aMAL8P1.97_0	MAL8P1.97	2.085413	1.014753	-0.73059	-1.59425	-1.07059
intMAL8P1.99_2	MAL8P1.99	-0.18623	-0.56085	0.253703	0.664963	0.220513
aMAL8P1.99_0	MAL8P1.99	-0.18623	-0.56085	0.253703	0.664963	0.220513
aMAL8P1.98_1	MAL8P1.99	-0.32743	-0.35632	0.095907	0.502927	0.450157
aMAL8P1.98_0	MAL8P1.99	-0.32743	-0.35632	0.095907	0.502927	0.450157
intPF07_0002_2	PF07_0002	0.09299	0.69304	0.51842	-0.10042	-0.67004
intPF07_0004_2	PF07_0004	0.894333	0.964353	0.161543	-0.57388	-0.81219
aPF07_0004_1	PF07_0004	0.894333	0.964353	0.161543	-0.57388	-0.81219
aPF07_0004_0	PF07_0004	0.894333	0.964353	0.161543	-0.57388	-0.81219
intPF07_0005_2	PF07_0005	0.911163	0.775703	0.096583	-0.37892	-0.53204
aPF07_0005_1	PF07_0005	0.911163	0.775703	0.096583	-0.37892	-0.53204
aPF07_0005_0	PF07_0005	0.911163	0.775703	0.096583	-0.37892	-0.53204
intPF07_0006_2	PF07_0006	1.405247	0.753747	0.166497	-0.44116	-0.93291
aPF07_0006_1	PF07_0006	1.405247	0.753747	0.166497	-0.44116	-0.93291
aPF07_0006_0	PF07_0006	1.405247	0.753747	0.166497	-0.44116	-0.93291
intPF07_0007_2	PF07_0007	0.093637	-0.80119	0.443887	0.640447	-0.41847
aPF07_0007_0	PF07_0007	0.093637	-0.80119	0.443887	0.640447	-0.41847
intPF07_0008_2	PF07_0008	-0.7336	0.983	2.01251	0.82449	-1.553
aPF07_0008_0	PF07_0008	-0.7336	0.983	2.01251	0.82449	-1.553
intPF07_0009_2	PF07_0009	0.919118	0.846808	-0.02912	-0.86788	-0.99028
aPF07_0009_0	PF07_0009	0.919118	0.846808	-0.02912	-0.86788	-0.99028
intPF07_0011_2	PF07_0010	0.841873	0.592633	0.399733	-0.15307	-0.84797
intMAL7P1.10_2	PF07_0010	0.684703	0.071463	0.334563	0.148103	-0.5718
aPF07_0010_2	PF07_0010	0.402022	0.023382	-0.01543	0.078322	0.027342
aPF07_0010_1	PF07_0010	0.402022	0.023382	-0.01543	0.078322	0.027342
aPF07_0010_0	PF07_0010	0.402022	0.023382	-0.01543	0.078322	0.027342
aPF07_0011_0	PF07_0011	0.841873	0.592633	0.399733	-0.15307	-0.84797

intPF07_0012_2	PF07_0012	-0.69198	-1.08815	-0.31188	0.596545	0.971815
intMAL7P1.11_2	PF07_0012	-0.23425	-0.19075	0.593	0.572	-0.27825
aPF07_0012_2	PF07_0012	-0.69198	-1.08815	-0.31188	0.596545	0.971815
aPF07_0012_1	PF07_0012	-0.69198	-1.08815	-0.31188	0.596545	0.971815
aPF07_0012_0	PF07_0012	-0.69198	-1.08815	-0.31188	0.596545	0.971815
intPF07_0013_2	PF07_0013	-0.16677	-0.14914	0.407537	0.403297	-0.18602
aPF07_0013_1	PF07_0013	-0.16677	-0.14914	0.407537	0.403297	-0.18602
aPF07_0013_0	PF07_0013	-0.16677	-0.14914	0.407537	0.403297	-0.18602
intPF07_0014_2	PF07_0014	0.47421	0.07397	-0.55194	-0.37406	0.34203
aPF07_0014_1	PF07_0014	0.47421	0.07397	-0.55194	-0.37406	0.34203
aPF07_0014_0	PF07_0014	0.47421	0.07397	-0.55194	-0.37406	0.34203
intPF07_0015_2	PF07_0015	0.628787	0.941547	0.703897	-0.16956	-1.12671
aPF07_0015_1	PF07_0015	0.628787	0.941547	0.703897	-0.16956	-1.12671
aPF07_0015_0	PF07_0015	0.628787	0.941547	0.703897	-0.16956	-1.12671
intPF07_0016_2	PF07_0016	-1.10675	-1.06363	-1.25459	0.088923	2.093293
aPF07_0016_2	PF07_0016	-1.10675	-1.06363	-1.25459	0.088923	2.093293
aPF07_0016_1	PF07_0016	-1.10675	-1.06363	-1.25459	0.088923	2.093293
aPF07_0016_0	PF07_0016	-1.10675	-1.06363	-1.25459	0.088923	2.093293
intPF07_0017_2	PF07_0017	-0.57148	-0.1316	-0.3118	-0.0052	0.6666
aPF07_0017_1	PF07_0017	-0.57148	-0.1316	-0.3118	-0.0052	0.6666
aPF07_0017_0	PF07_0017	-0.57148	-0.1316	-0.3118	-0.0052	0.6666
intPF07_0018_2	PF07_0018	0.035933	0.210813	-0.15864	-0.2297	0.074853
aPF07_0018_1	PF07_0018	0.035933	0.210813	-0.15864	-0.2297	0.074853
aPF07_0018_0	PF07_0018	0.035933	0.210813	-0.15864	-0.2297	0.074853
intPF07_0019_3	PF07_0019	-0.13613	-0.61085	-0.97506	-0.43794	0.76885
aPF07_0019_1	PF07_0019	-0.13613	-0.61085	-0.97506	-0.43794	0.76885
aPF07_0019_0	PF07_0019	-0.13613	-0.61085	-0.97506	-0.43794	0.76885
intPF07_0020_2	PF07_0020	0.524393	0.907523	0.125203	-0.47604	-0.51336
aPF07_0020_1	PF07_0020	0.524393	0.907523	0.125203	-0.47604	-0.51336
aPF07_0020_0	PF07_0020	0.524393	0.907523	0.125203	-0.47604	-0.51336
intPF07_0022_2	PF07_0021	-1.06231	-0.41578	0.825228	0.808668	-0.223
intPF07_0021_2	PF07_0021	-0.35591	-0.47294	-1.57338	-0.69362	1.64744
aPF07_0021_1	PF07_0021	-0.35591	-0.47294	-1.57338	-0.69362	1.64744
aPF07_0021_0	PF07_0021	-0.35591	-0.47294	-1.57338	-0.69362	1.64744
aPF07_0022_2	PF07_0022	-1.06231	-0.41578	0.825228	0.808668	-0.223
aPF07_0022_1	PF07_0022	-1.06231	-0.41578	0.825228	0.808668	-0.223
aPF07_0022_0	PF07_0022	-1.06231	-0.41578	0.825228	0.808668	-0.223
intPF07_0023_2	PF07_0023	-0.81152	-1.48887	-0.60375	0.780587	1.555707
aPF07_0023_1	PF07_0023	-0.81152	-1.48887	-0.60375	0.780587	1.555707
aPF07_0023_0	PF07_0023	-0.81152	-1.48887	-0.60375	0.780587	1.555707
intPF07_0024_2	PF07_0024	0.690512	0.533652	-0.83789	-1.07383	0.050982
aPF07_0024_4	PF07_0024	0.690512	0.533652	-0.83789	-1.07383	0.050982
aPF07_0024_3	PF07_0024	0.690512	0.533652	-0.83789	-1.07383	0.050982
aPF07_0024_2	PF07_0024	0.690512	0.533652	-0.83789	-1.07383	0.050982
aPF07_0024_1	PF07_0024	0.690512	0.533652	-0.83789	-1.07383	0.050982
aPF07_0024_0	PF07_0024	0.690512	0.533652	-0.83789	-1.07383	0.050982
intPF07_0025_2	PF07_0025	0.578363	0.388543	0.203993	-0.35161	-0.83516

aPF07_0025_0	PF07_0025	0.578363	0.388543	0.203993	-0.35161	-0.83516
intPF07_0026_2	PF07_0026	-0.20366	-0.1052	0.087643	-0.03348	-0.21314
aPF07_0026_1	PF07_0026	-0.20366	-0.1052	0.087643	-0.03348	-0.21314
aPF07_0026_0	PF07_0026	-0.20366	-0.1052	0.087643	-0.03348	-0.21314
intPF07_0027_2	PF07_0027	0.68456	0.4242	0.7381	0.0449	-1.2312
aPF07_0027_0	PF07_0027	0.68456	0.4242	0.7381	0.0449	-1.2312
intMAL7P1.26_2	PF07_0028					
aPF07_0028_1	PF07_0028	1.17999	0.97854	-0.04858	-0.85542	-0.93504
aPF07_0028_0	PF07_0028	1.17999	0.97854	-0.04858	-0.85542	-0.93504
intPF07_0029_2	PF07_0029	-0.11498	0.15242	0.72116	0.48484	-0.45492
aPF07_0029_1	PF07_0029	-0.11498	0.15242	0.72116	0.48484	-0.45492
aPF07_0029_0	PF07_0029	-0.11498	0.15242	0.72116	0.48484	-0.45492
intPF07_0030_2	PF07_0030	0.593167	0.747667	0.202667	-0.31433	-0.54233
aPF07_0030_1	PF07_0030	0.593167	0.747667	0.202667	-0.31433	-0.54233
intPF07_0031_3	PF07_0031	-0.53984	-0.65039	-0.19492	0.24432	0.47019
aPF07_0031_0	PF07_0031	-0.53984	-0.65039	-0.19492	0.24432	0.47019
intPF07_0032_2	PF07_0032	-0.93898	-1.2501	-0.3878	0.723463	1.275763
aPF07_0032_0	PF07_0032	-0.93898	-1.2501	-0.3878	0.723463	1.275763
intPF07_0033_2	PF07_0033	-0.1932	0.279517	0.579727	0.295607	-0.36868
aPF07_0033_1	PF07_0033	-0.1932	0.279517	0.579727	0.295607	-0.36868
aPF07_0033_0	PF07_0033	-0.1932	0.279517	0.579727	0.295607	-0.36868
intPF07_0034_3	PF07_0034	0.67733	0.23857	-0.42803	-0.41157	0.10063
aPF07_0034_0	PF07_0034	0.67733	0.23857	-0.42803	-0.41157	0.10063
intPF07_0035_2	PF07_0035	-0.40358	-1.37563	-0.69401	0.647673	1.461293
aPF07_0035_1	PF07_0035	-0.40358	-1.37563	-0.69401	0.647673	1.461293
aPF07_0035_0	PF07_0035	-0.40358	-1.37563	-0.69401	0.647673	1.461293
aPF07_0036_0	PF07_0036					
intPF07_0037_2	PF07_0037	0.45891	0.73361	0.3024	-0.22442	-0.55493
intPF07_0036_2	PF07_0037					
aPF07_0037_4	PF07_0037	0.45891	0.73361	0.3024	-0.22442	-0.55493
aPF07_0037_3	PF07_0037	0.45891	0.73361	0.3024	-0.22442	-0.55493
aPF07_0037_2	PF07_0037	0.45891	0.73361	0.3024	-0.22442	-0.55493
aPF07_0037_1	PF07_0037	0.45891	0.73361	0.3024	-0.22442	-0.55493
aPF07_0037_0	PF07_0037	0.45891	0.73361	0.3024	-0.22442	-0.55493
aPF07_0038_1	PF07_0038	-1.11304	-0.72834	-0.23582	0.607483	1.229003
aPF07_0038_0	PF07_0038	-1.11304	-0.72834	-0.23582	0.607483	1.229003
intPF07_0039_2	PF07_0039	-0.02838	-0.21514	0.295273	0.451393	0.001803
intPF07_0038_2	PF07_0039	-1.11304	-0.72834	-0.23582	0.607483	1.229003
aPF07_0039_0	PF07_0039	-0.02838	-0.21514	0.295273	0.451393	0.001803
aPF07_0040_0	PF07_0040					
intPF07_0041_2	PF07_0041	0.56326	-0.18179	-0.11392	-0.00208	-0.10321
intMAL7P1.30_2	PF07_0041	0.263783	0.165303	-0.55851	-0.33763	0.539763
aPF07_0041_1	PF07_0041	0.56326	-0.18179	-0.11392	-0.00208	-0.10321
aPF07_0041_0	PF07_0041	0.56326	-0.18179	-0.11392	-0.00208	-0.10321
intPF07_0042_2	PF07_0042	-0.76524	-0.54042	-0.3814	0.262053	0.987113
aPF07_0042_4	PF07_0042	-0.76524	-0.54042	-0.3814	0.262053	0.987113
aPF07_0042_3	PF07_0042	-0.76524	-0.54042	-0.3814	0.262053	0.987113

aPF07_0042_2	PF07_0042	-0.76524	-0.54042	-0.3814	0.262053	0.987113
aPF07_0042_1	PF07_0042	-0.76524	-0.54042	-0.3814	0.262053	0.987113
aPF07_0042_0	PF07_0042	-0.76524	-0.54042	-0.3814	0.262053	0.987113
intPF07_0043_2	PF07_0043	0.700543	0.255283	0.650933	0.378733	-0.68862
intMAL7P1.33_2	PF07_0043	-0.54021	-0.58893	0.63736	0.94264	0.03293
aPF07_0043_0	PF07_0043	0.700543	0.255283	0.650933	0.378733	-0.68862
aPF07_0044_0	PF07_0044	0.958968	1.803518	0.249638	-0.93252	-0.97375
intPF07_0046_2	PF07_0045	0.568617	0.696437	0.186627	-0.18079	-0.3386
intPF07_0044_2	PF07_0045	0.958968	1.803518	0.249638	-0.93252	-0.97375
aPF07_0045_1	PF07_0045	0.819207	0.506467	0.103567	-0.20123	-0.42713
aPF07_0045_0	PF07_0045	0.819207	0.506467	0.103567	-0.20123	-0.42713
intPF07_0045_2	PF07_0046	0.819207	0.506467	0.103567	-0.20123	-0.42713
aPF07_0046_1	PF07_0046	0.568617	0.696437	0.186627	-0.18079	-0.3386
aPF07_0046_0	PF07_0046	0.568617	0.696437	0.186627	-0.18079	-0.3386
intPF07_0047_2	PF07_0047	-0.27699	-0.35051	0.212267	0.349567	-0.00615
aPF07_0047_1	PF07_0047	-0.27699	-0.35051	0.212267	0.349567	-0.00615
aPF07_0047_0	PF07_0047	-0.27699	-0.35051	0.212267	0.349567	-0.00615
intPF07_0052_2	PF07_0052	0.099183	-0.60994	-1.40214	-0.7682	0.978603
aPF07_0052_0	PF07_0052	0.099183	-0.60994	-1.40214	-0.7682	0.978603
intPF07_0053_2	PF07_0053	0.064975	0.488455	0.176665	-0.09878	-0.16807
aPF07_0053_5	PF07_0053	0.064975	0.488455	0.176665	-0.09878	-0.16807
aPF07_0053_4	PF07_0053	0.064975	0.488455	0.176665	-0.09878	-0.16807
aPF07_0053_3	PF07_0053	0.064975	0.488455	0.176665	-0.09878	-0.16807
aPF07_0053_2	PF07_0053	0.064975	0.488455	0.176665	-0.09878	-0.16807
aPF07_0053_1	PF07_0053	0.064975	0.488455	0.176665	-0.09878	-0.16807
aPF07_0053_0	PF07_0053	0.064975	0.488455	0.176665	-0.09878	-0.16807
intPF07_0054_1	PF07_0054	-0.46869	-0.98455	-1.1699	-0.0741	1.53355
aPF07_0054_0	PF07_0054	-0.46869	-0.98455	-1.1699	-0.0741	1.53355
intPF07_0055_2	PF07_0055	-0.10258	-0.1426	0.132957	0.145377	-0.08656
aPF07_0055_1	PF07_0055	-0.10258	-0.1426	0.132957	0.145377	-0.08656
aPF07_0055_0	PF07_0055	-0.10258	-0.1426	0.132957	0.145377	-0.08656
intPF07_0056_2	PF07_0056	0.0692	0.10298	-0.37221	-0.37029	0.17252
aPF07_0056_1	PF07_0056	0.0692	0.10298	-0.37221	-0.37029	0.17252
aPF07_0056_0	PF07_0056	0.0692	0.10298	-0.37221	-0.37029	0.17252
aPF07_0057_1	PF07_0057	-0.10893	-0.16987	0.14323	0.35127	0.18737
aPF07_0057_0	PF07_0057	-0.10893	-0.16987	0.14323	0.35127	0.18737
intPF07_0058_2	PF07_0058	0.44469	0.70105	-0.01585	-0.45415	-0.33305
aPF07_0058_1	PF07_0058	0.44469	0.70105	-0.01585	-0.45415	-0.33305
aPF07_0058_0	PF07_0058	0.44469	0.70105	-0.01585	-0.45415	-0.33305
intPF07_0059_2	PF07_0059	0.356953	0.289253	0.544483	0.237183	-0.56859
aPF07_0059_0	PF07_0059	0.356953	0.289253	0.544483	0.237183	-0.56859
aPF07_0060_0	PF07_0060	-0.25488	-0.58662	-0.57145	-0.02029	0.711092
intPF07_0061_2	PF07_0061	-1.01174	-0.89579	-1.02128	0.067698	1.713958
intPF07_0060_2	PF07_0061	-0.25488	-0.58662	-0.57145	-0.02029	0.711092
aPF07_0061_3	PF07_0061	-1.01174	-0.89579	-1.02128	0.067698	1.713958
aPF07_0061_2	PF07_0061	-1.01174	-0.89579	-1.02128	0.067698	1.713958
aPF07_0061_1	PF07_0061	-1.01174	-0.89579	-1.02128	0.067698	1.713958

aPF07_0061_0	PF07_0061	-1.01174	-0.89579	-1.02128	0.067698	1.713958
intMAL7P1.75_2	PF07_0062	-1.25913	-1.35035	0.359693	1.353973	0.891013
aPF07_0062_1	PF07_0062	-0.9975	-1.02099	0.12723	0.92727	0.80949
aPF07_0062_0	PF07_0062	-0.9975	-1.02099	0.12723	0.92727	0.80949
intPF07_0063_2	PF07_0063	-0.73552	-0.67143	0.168985	0.796205	0.682045
aPF07_0063_0	PF07_0063	-0.73552	-0.67143	0.168985	0.796205	0.682045
intPF07_0064_2	PF07_0064	-0.79992	-0.4344	0.21154	0.67846	0.6069
aPF07_0064_1	PF07_0064	-0.79992	-0.4344	0.21154	0.67846	0.6069
aPF07_0064_0	PF07_0064	-0.79992	-0.4344	0.21154	0.67846	0.6069
intPF07_0065_2	PF07_0065	-1.34502	-1.6859	-0.1262	1.319863	1.535563
aPF07_0065_1	PF07_0065	-1.34502	-1.6859	-0.1262	1.319863	1.535563
aPF07_0065_0	PF07_0065	-1.34502	-1.6859	-0.1262	1.319863	1.535563
intPF07_0066_3	PF07_0066	0.586413	0.486933	0.189123	-0.21846	-0.54527
aPF07_0066_1	PF07_0066	0.586413	0.486933	0.189123	-0.21846	-0.54527
aPF07_0066_0	PF07_0066	0.586413	0.486933	0.189123	-0.21846	-0.54527
intPF07_0067_3	PF07_0067	0.79735	0.78973	0.86953	-0.20453	-1.64023
intMAL7P1.76_2	PF07_0067	1.330037	1.417307	0.619877	-0.85204	-1.91297
aPF07_0067_1	PF07_0067	0.79735	0.78973	0.86953	-0.20453	-1.64023
aPF07_0067_0	PF07_0067	0.79735	0.78973	0.86953	-0.20453	-1.64023
intPF07_0069_2	PF07_0068	-0.60578	-0.55762	0.53199	0.68151	-0.12288
aPF07_0068_1	PF07_0068	-0.87748	-0.3721	0.44545	0.71255	0.2966
aPF07_0068_0	PF07_0068	-0.87748	-0.3721	0.44545	0.71255	0.2966
aPF07_0069_1	PF07_0069	-0.60578	-0.55762	0.53199	0.68151	-0.12288
aPF07_0069_0	PF07_0069	-0.60578	-0.55762	0.53199	0.68151	-0.12288
intPF07_0070_2	PF07_0070					
aPF07_0070_0	PF07_0070					
intPF07_0071_2	PF07_0071	-0.05254	0.6857	0.89035	-0.17735	-1.4237
aPF07_0071_1	PF07_0071	-0.05254	0.6857	0.89035	-0.17735	-1.4237
aPF07_0071_0	PF07_0071	-0.05254	0.6857	0.89035	-0.17735	-1.4237
intPF07_0072_2	PF07_0072	-0.17517	-0.54569	-1.04363	-0.68271	0.583853
aPF07_0072_1	PF07_0072	-0.17517	-0.54569	-1.04363	-0.68271	0.583853
aPF07_0072_0	PF07_0072	-0.17517	-0.54569	-1.04363	-0.68271	0.583853
aPF07_0073_1	PF07_0073	-0.18281	0.301013	0.449463	0.298703	-0.11435
aPF07_0073_0	PF07_0073	-0.18281	0.301013	0.449463	0.298703	-0.11435
intPF07_0074_2	PF07_0074	-0.27537	-0.33463	-0.03733	0.26066	0.32813
intPF07_0073_2	PF07_0074	-0.18281	0.301013	0.449463	0.298703	-0.11435
aPF07_0074_2	PF07_0074	-0.27537	-0.33463	-0.03733	0.26066	0.32813
aPF07_0074_1	PF07_0074	-0.27537	-0.33463	-0.03733	0.26066	0.32813
aPF07_0074_0	PF07_0074	-0.27537	-0.33463	-0.03733	0.26066	0.32813
aPF07_0075_1	PF07_0075	-1.07537	-0.46517	0.97685	1.17315	0.01717
aPF07_0075_0	PF07_0075	-1.07537	-0.46517	0.97685	1.17315	0.01717
intPF07_0076_2	PF07_0076					
intPF07_0075_2	PF07_0076	-1.07537	-0.46517	0.97685	1.17315	0.01717
intMAL7P1.97_2	PF07_0076					
aPF07_0076_0	PF07_0076					
intPF07_0077_2	PF07_0077	0.348053	0.430253	0.458743	0.080423	-0.54959
aPF07_0077_1	PF07_0077	0.348053	0.430253	0.458743	0.080423	-0.54959

aPF07_0077_0	PF07_0077	0.348053	0.430253	0.458743	0.080423	-0.54959
intPF07_0078_2	PF07_0078	-0.72914	-0.89579	-0.50593	0.31593	1.04729
aPF07_0078_1	PF07_0078	-0.72914	-0.89579	-0.50593	0.31593	1.04729
aPF07_0078_0	PF07_0078	-0.72914	-0.89579	-0.50593	0.31593	1.04729
intPF07_0079_3	PF07_0079	-0.15868	0.50441	0.87167	0.49233	-0.47041
aPF07_0079_0	PF07_0079	-0.15868	0.50441	0.87167	0.49233	-0.47041
intPF07_0080_2	PF07_0080	-0.11628	0.55041	0.81318	0.44382	-0.41941
aPF07_0080_0	PF07_0080	-0.11628	0.55041	0.81318	0.44382	-0.41941
intPF07_0082_2	PF07_0081	0.322758	0.077378	-0.31626	-0.15624	0.289458
intPF07_0081_3	PF07_0081	0.592337	-0.22467	-0.45614	-0.10902	0.325507
aPF07_0081_1	PF07_0081	0.592337	-0.22467	-0.45614	-0.10902	0.325507
aPF07_0081_0	PF07_0081	0.592337	-0.22467	-0.45614	-0.10902	0.325507
intMAL7P1.104_2	PF07_0082	0.34901	0.26194	0.31762	0.10138	-0.37194
aPF07_0082_5	PF07_0082	0.322758	0.077378	-0.31626	-0.15624	0.289458
aPF07_0082_4	PF07_0082	0.322758	0.077378	-0.31626	-0.15624	0.289458
aPF07_0082_3	PF07_0082	0.322758	0.077378	-0.31626	-0.15624	0.289458
aPF07_0082_2	PF07_0082	0.322758	0.077378	-0.31626	-0.15624	0.289458
aPF07_0082_1	PF07_0082	0.322758	0.077378	-0.31626	-0.15624	0.289458
aPF07_0082_0	PF07_0082	0.322758	0.077378	-0.31626	-0.15624	0.289458
intPF07_0084_2	PF07_0083	0.298473	0.357643	0.768723	0.268943	-0.86698
aPF07_0083_0	PF07_0083	0.669363	0.336193	0.233113	-0.18545	-0.70453
intPF07_0083_2	PF07_0084	0.669363	0.336193	0.233113	-0.18545	-0.70453
aPF07_0084_0	PF07_0084	0.298473	0.357643	0.768723	0.268943	-0.86698
intPF07_0085_2	PF07_0085	0.21007	0.59716	0.64267	0.18033	-0.57966
aPF07_0085_1	PF07_0085	0.21007	0.59716	0.64267	0.18033	-0.57966
aPF07_0085_0	PF07_0085	0.21007	0.59716	0.64267	0.18033	-0.57966
intPF07_0086_2	PF07_0086	-0.32149	-0.26692	-0.19675	0.170487	0.545657
aPF07_0086_5	PF07_0086	-0.32149	-0.26692	-0.19675	0.170487	0.545657
aPF07_0086_4	PF07_0086	-0.32149	-0.26692	-0.19675	0.170487	0.545657
aPF07_0086_3	PF07_0086	-0.32149	-0.26692	-0.19675	0.170487	0.545657
aPF07_0086_2	PF07_0086	-0.32149	-0.26692	-0.19675	0.170487	0.545657
aPF07_0086_1	PF07_0086	-0.32149	-0.26692	-0.19675	0.170487	0.545657
aPF07_0086_0	PF07_0086	-0.32149	-0.26692	-0.19675	0.170487	0.545657
intPF07_0087_3	PF07_0087	-2.67624	-1.09784	0.632345	1.701505	1.553135
aPF07_0087_0	PF07_0087	-2.67624	-1.09784	0.632345	1.701505	1.553135
intPF07_0088_2	PF07_0088	-0.32232	0.494627	0.906747	0.540587	-0.41429
aPF07_0088_0	PF07_0088	-0.32232	0.494627	0.906747	0.540587	-0.41429
intPF07_0089_2	PF07_0089	-0.22034	0.190683	0.176123	0.091543	0.014983
aPF07_0089_1	PF07_0089	-0.22034	0.190683	0.176123	0.091543	0.014983
aPF07_0089_0	PF07_0089	-0.22034	0.190683	0.176123	0.091543	0.014983
intPF07_0090_3	PF07_0090	0.077517	-0.0236	0.515697	0.184637	-0.71006
aPF07_0090_0	PF07_0090	0.077517	-0.0236	0.515697	0.184637	-0.71006
intPF07_0091_2	PF07_0091	0.263823	-0.09513	0.022753	0.015913	-0.16921
aPF07_0091_0	PF07_0091	0.263823	-0.09513	0.022753	0.015913	-0.16921
intPF07_0092_2	PF07_0092	0.235027	0.262877	0.516737	0.024597	-0.81354
aPF07_0092_1	PF07_0092	0.235027	0.262877	0.516737	0.024597	-0.81354
aPF07_0092_0	PF07_0092	0.235027	0.262877	0.516737	0.024597	-0.81354

intPF07_0093_3	PF07_0093	-0.95946	-0.63472	0.077933	0.563733	0.599383
aPF07_0093_0	PF07_0093	-0.95946	-0.63472	0.077933	0.563733	0.599383
intPF07_0094_2	PF07_0094	-0.24466	0.192377	0.041287	-0.05059	0.058237
aPF07_0094_1	PF07_0094	-0.24466	0.192377	0.041287	-0.05059	0.058237
aPF07_0094_0	PF07_0094	-0.24466	0.192377	0.041287	-0.05059	0.058237
aPF07_0095_0	PF07_0095	1.044287	1.045587	0.477817	-0.45548	-1.21025
intPF07_0095_2	PF07_0096	1.044287	1.045587	0.477817	-0.45548	-1.21025
intMAL7P1.124_2	PF07_0096	1.274557	0.255207	-0.42771	-0.77377	-0.63123
aPF07_0096_1	PF07_0096	-0.05331	-0.44844	-0.43836	-0.3078	0.060777
aPF07_0096_0	PF07_0096	-0.05331	-0.44844	-0.43836	-0.3078	0.060777
intPF07_0097_1	PF07_0097	0.531733	0.699683	0.552803	-0.25792	-1.11113
aPF07_0097_2	PF07_0097	0.531733	0.699683	0.552803	-0.25792	-1.11113
aPF07_0097_1	PF07_0097	0.531733	0.699683	0.552803	-0.25792	-1.11113
aPF07_0097_0	PF07_0097	0.531733	0.699683	0.552803	-0.25792	-1.11113
intPF07_0099_2	PF07_0098	1.236343	0.948293	0.782913	-0.10825	-1.37763
aPF07_0098_0	PF07_0098	0.33519	1.07205	0.6804	-0.1964	-0.93905
intPF07_0100_2	PF07_0099	-0.03895	0.104723	0.317803	0.250863	-0.12356
intPF07_0098_2	PF07_0099	0.33519	1.07205	0.6804	-0.1964	-0.93905
aPF07_0099_0	PF07_0099	1.236343	0.948293	0.782913	-0.10825	-1.37763
aPF07_0100_1	PF07_0100	-0.03895	0.104723	0.317803	0.250863	-0.12356
aPF07_0100_0	PF07_0100	-0.03895	0.104723	0.317803	0.250863	-0.12356
intPF07_0101_2	PF07_0101	-0.82265	-1.32001	-1.14913	0.158375	1.738505
aPF07_0101_3	PF07_0101	-0.82265	-1.32001	-1.14913	0.158375	1.738505
aPF07_0101_2	PF07_0101	-0.82265	-1.32001	-1.14913	0.158375	1.738505
aPF07_0101_1	PF07_0101	-0.82265	-1.32001	-1.14913	0.158375	1.738505
aPF07_0101_0	PF07_0101	-0.82265	-1.32001	-1.14913	0.158375	1.738505
intPF07_0102_2	PF07_0102	0.762863	0.695203	0.472343	-0.02518	-0.69454
intMAL7P1.130_2	PF07_0102	-0.05606	0.317437	0.284587	0.191347	-0.0044
aPF07_0102_1	PF07_0102	0.762863	0.695203	0.472343	-0.02518	-0.69454
aPF07_0102_0	PF07_0102	0.762863	0.695203	0.472343	-0.02518	-0.69454
intPF07_0103_3	PF07_0103	0.638927	0.531357	0.437787	-0.20645	-0.97102
aPF07_0103_0	PF07_0103	0.638927	0.531357	0.437787	-0.20645	-0.97102
intPF07_0104_2	PF07_0104	2.107035	0.794145	-1.54912	-2.47744	-1.04904
aPF07_0104_2	PF07_0104	2.107035	0.794145	-1.54912	-2.47744	-1.04904
aPF07_0104_1	PF07_0104	2.107035	0.794145	-1.54912	-2.47744	-1.04904
aPF07_0104_0	PF07_0104	2.107035	0.794145	-1.54912	-2.47744	-1.04904
intPF07_0105_2	PF07_0105	-1.10622	-0.96541	-0.43418	0.48518	1.24891
aPF07_0105_1	PF07_0105	-1.10622	-0.96541	-0.43418	0.48518	1.24891
aPF07_0105_0	PF07_0105	-1.10622	-0.96541	-0.43418	0.48518	1.24891
intPF07_0106_2	PF07_0106	0.800653	1.044553	0.260733	-0.62802	-0.99054
aPF07_0106_1	PF07_0106	0.800653	1.044553	0.260733	-0.62802	-0.99054
aPF07_0106_0	PF07_0106	0.800653	1.044553	0.260733	-0.62802	-0.99054
intPF07_0107_2	PF07_0107	2.506107	2.109977	-0.15145	-2.26571	-2.54964
aPF07_0107_1	PF07_0107	2.506107	2.109977	-0.15145	-2.26571	-2.54964
aPF07_0107_0	PF07_0107	2.506107	2.109977	-0.15145	-2.26571	-2.54964
intPF07_0108_2	PF07_0108	-0.38551	0.01004	-0.18458	-0.02342	0.43846
aPF07_0108_1	PF07_0108	-0.38551	0.01004	-0.18458	-0.02342	0.43846

aPF07_0108_0	PF07_0108	-0.38551	0.01004	-0.18458	-0.02342	0.43846
aPF07_0109_0	PF07_0109	-0.12614	0.061717	-0.07093	-0.05173	0.135617
intPF07_0110_2	PF07_0109a	-0.53645	0.012267	0.889477	0.700857	-0.39243
intPF07_0109_2	PF07_0109a	-0.12614	0.061717	-0.07093	-0.05173	0.135617
aPF07_0110_1	PF07_0110	-0.53645	0.012267	0.889477	0.700857	-0.39243
aPF07_0110_0	PF07_0110	-0.53645	0.012267	0.889477	0.700857	-0.39243
intPF07_0111_2	PF07_0111	0.37019	1.18705	0.0414	-0.5604	-0.24855
aPF07_0111_1	PF07_0111	0.37019	1.18705	0.0414	-0.5604	-0.24855
aPF07_0111_0	PF07_0111	0.37019	1.18705	0.0414	-0.5604	-0.24855
aPF07_0112_0	PF07_0112	-0.59102	-0.6576	0.112525	0.850255	0.827705
aPF07_0113_1	PF07_0113	-1.03655	-0.55929	0.126813	0.703853	0.795953
aPF07_0113_0	PF07_0113	-1.03655	-0.55929	0.126813	0.703853	0.795953
intPF07_0114_2	PF07_0114	0.1987	0.229	0.04175	-0.03825	-0.0475
aPF07_0114_1	PF07_0114	0.1987	0.229	0.04175	-0.03825	-0.0475
aPF07_0114_0	PF07_0114	0.1987	0.229	0.04175	-0.03825	-0.0475
intPF07_0115_3	PF07_0115	-0.19657	0.134687	0.148457	0.189597	0.167537
aPF07_0115_2	PF07_0115	-0.19657	0.134687	0.148457	0.189597	0.167537
aPF07_0115_1	PF07_0115	-0.19657	0.134687	0.148457	0.189597	0.167537
aPF07_0115_0	PF07_0115	-0.19657	0.134687	0.148457	0.189597	0.167537
intPF07_0116_2	PF07_0116	1.365888	0.907878	-0.25067	-0.87016	-0.69571
aPF07_0116_2	PF07_0116	1.365888	0.907878	-0.25067	-0.87016	-0.69571
aPF07_0116_1	PF07_0116	1.365888	0.907878	-0.25067	-0.87016	-0.69571
aPF07_0116_0	PF07_0116	1.365888	0.907878	-0.25067	-0.87016	-0.69571
intPF07_0117_2	PF07_0117	-0.17491	-0.17944	0.34987	0.56613	0.14644
aPF07_0117_0	PF07_0117	-0.17491	-0.17944	0.34987	0.56613	0.14644
aPF07_0118_8	PF07_0118	-0.77214	-1.00941	-0.45619	0.555393	1.253873
aPF07_0118_7	PF07_0118	-0.77214	-1.00941	-0.45619	0.555393	1.253873
aPF07_0118_6	PF07_0118	-0.77214	-1.00941	-0.45619	0.555393	1.253873
aPF07_0118_5	PF07_0118	-0.77214	-1.00941	-0.45619	0.555393	1.253873
aPF07_0118_4	PF07_0118	-0.77214	-1.00941	-0.45619	0.555393	1.253873
aPF07_0118_3	PF07_0118	-0.77214	-1.00941	-0.45619	0.555393	1.253873
aPF07_0118_2	PF07_0118	-0.77214	-1.00941	-0.45619	0.555393	1.253873
aPF07_0118_1	PF07_0118	-0.77214	-1.00941	-0.45619	0.555393	1.253873
aPF07_0118_0	PF07_0118	-0.77214	-1.00941	-0.45619	0.555393	1.253873
intMAL7P1.154_2	PF07_0119	-0.70613	-1.08983	0.121925	0.918925	0.663675
aPF07_0119_0	PF07_0119	-1.27445	-0.98973	0.406477	1.062857	0.614067
intPF07_0120_2	PF07_0120	-0.77036	-0.30144	-0.33698	0.233148	1.023358
aPF07_0120_3	PF07_0120	-0.77036	-0.30144	-0.33698	0.233148	1.023358
aPF07_0120_2	PF07_0120	-0.77036	-0.30144	-0.33698	0.233148	1.023358
aPF07_0120_1	PF07_0120	-0.77036	-0.30144	-0.33698	0.233148	1.023358
aPF07_0120_0	PF07_0120	-0.77036	-0.30144	-0.33698	0.233148	1.023358
aPF07_0121_1	PF07_0121	0.62642	0.5644	0.75296	-0.06296	-1.3074
aPF07_0121_0	PF07_0121	0.62642	0.5644	0.75296	-0.06296	-1.3074
intPF07_0122_2	PF07_0122	0.361653	0.436753	1.072483	0.338683	-1.28759
intPF07_0121_2	PF07_0122	0.62642	0.5644	0.75296	-0.06296	-1.3074
aPF07_0122_1	PF07_0122	0.361653	0.436753	1.072483	0.338683	-1.28759
aPF07_0122_0	PF07_0122	0.361653	0.436753	1.072483	0.338683	-1.28759

intPF07_0123_2	PF07_0123	0.551217	0.443917	0.433667	-0.19083	-0.96908
aPF07_0123_1	PF07_0123	0.551217	0.443917	0.433667	-0.19083	-0.96908
aPF07_0123_0	PF07_0123	0.551217	0.443917	0.433667	-0.19083	-0.96908
aPF07_0124_1	PF07_0124	0.257367	-0.28581	-0.77062	-0.55654	0.301647
aPF07_0124_0	PF07_0124	0.257367	-0.28581	-0.77062	-0.55654	0.301647
intPF07_0125_2	PF07_0125	-0.37802	0.5761	0.9548	0.4282	-0.5981
aPF07_0125_1	PF07_0125	-0.37802	0.5761	0.9548	0.4282	-0.5981
aPF07_0125_0	PF07_0125	-0.37802	0.5761	0.9548	0.4282	-0.5981
intPF07_0126_3	PF07_0126	1.52847	0.48266	0.05293	-1.03193	-1.83266
aPF07_0126_1	PF07_0126	1.52847	0.48266	0.05293	-1.03193	-1.83266
aPF07_0126_0	PF07_0126	1.52847	0.48266	0.05293	-1.03193	-1.83266
intPF07_0127_2	PF07_0127	0.81155	-0.22874	-1.10752	-1.08248	-0.01626
aPF07_0127_0	PF07_0127	0.81155	-0.22874	-1.10752	-1.08248	-0.01626
intPF07_0129_2	PF07_0129	-1.37113	-0.87439	0.210023	1.050643	1.065053
aPF07_0129_1	PF07_0129	-1.37113	-0.87439	0.210023	1.050643	1.065053
aPF07_0129_0	PF07_0129	-1.37113	-0.87439	0.210023	1.050643	1.065053
intPF08_0001_2	PF08_0001	0.439517	0.411157	-0.02716	-0.57598	-0.68658
aPF08_0001_0	PF08_0001	0.439517	0.411157	-0.02716	-0.57598	-0.68658
intPF08_0002_2	PF08_0002	1.48214	0.50845	-0.46024	-0.75245	-0.43998
aPF08_0002_3	PF08_0002	1.48214	0.50845	-0.46024	-0.75245	-0.43998
aPF08_0002_2	PF08_0002	1.48214	0.50845	-0.46024	-0.75245	-0.43998
aPF08_0002_1	PF08_0002	1.48214	0.50845	-0.46024	-0.75245	-0.43998
aPF08_0002_0	PF08_0002	1.48214	0.50845	-0.46024	-0.75245	-0.43998
intPF08_0003_2	PF08_0003	3.238117	1.604417	-0.93958	-2.98158	-2.75958
aPF08_0003_1	PF08_0003	3.238117	1.604417	-0.93958	-2.98158	-2.75958
aPF08_0003_0	PF08_0003	3.238117	1.604417	-0.93958	-2.98158	-2.75958
intPF08_0004_2	PF08_0004					
aPF08_0004_0	PF08_0004					
intPF08_0005_2	PF08_0005	1.13653	0.28284	-0.37543	-0.57757	-0.36834
aPF08_0005_1	PF08_0005	1.13653	0.28284	-0.37543	-0.57757	-0.36834
aPF08_0005_0	PF08_0005	1.13653	0.28284	-0.37543	-0.57757	-0.36834
aPF08_0006_0	PF08_0006	-0.43718	-0.32509	1.25817	1.12583	-0.68891
intPF08_0007_2	PF08_0007	0.057693	-0.59546	-1.4101	-0.64324	1.204123
aPF08_0007_0	PF08_0007	0.057693	-0.59546	-1.4101	-0.64324	1.204123
intPF08_0008_2	PF08_0008	1.621407	-0.00054	-1.94991	-2.59675	-0.86112
aPF08_0008_1	PF08_0008	1.621407	-0.00054	-1.94991	-2.59675	-0.86112
aPF08_0008_0	PF08_0008	1.621407	-0.00054	-1.94991	-2.59675	-0.86112
intPF08_0009_2	PF08_0009	-0.57686	-1.17671	-0.52257	0.56857	1.21871
intMAL8P1.14_2	PF08_0009	-0.94925	-0.57977	0.076043	0.621623	0.722933
aPF08_0009_0	PF08_0009	-0.57686	-1.17671	-0.52257	0.56857	1.21871
intPF08_0010_2	PF08_0010	-0.1465	-0.05801	-0.02148	0.16598	0.29851
aPF08_0010_1	PF08_0010	-0.1465	-0.05801	-0.02148	0.16598	0.29851
aPF08_0010_0	PF08_0010	-0.1465	-0.05801	-0.02148	0.16598	0.29851
intMAL8P1.15_2	PF08_0011	-0.61201	0.12756	0.40863	0.45337	0.21994
aPF08_0011_2	PF08_0011	-0.728	-0.00032	0.57714	0.55286	0.01365
aPF08_0011_1	PF08_0011	-0.728	-0.00032	0.57714	0.55286	0.01365
aPF08_0011_0	PF08_0011	-0.728	-0.00032	0.57714	0.55286	0.01365

intPF08_0012_2	PF08_0012	-0.06571	0.303078	-0.52665	-0.40857	0.576618
aPF08_0012_3	PF08_0012	-0.06571	0.303078	-0.52665	-0.40857	0.576618
aPF08_0012_2	PF08_0012	-0.06571	0.303078	-0.52665	-0.40857	0.576618
aPF08_0012_1	PF08_0012	-0.06571	0.303078	-0.52665	-0.40857	0.576618
aPF08_0012_0	PF08_0012	-0.06571	0.303078	-0.52665	-0.40857	0.576618
intPF08_0013_2	PF08_0013	1.087313	0.713453	0.024593	-0.53193	-0.72979
aPF08_0013_1	PF08_0013	1.087313	0.713453	0.024593	-0.53193	-0.72979
aPF08_0013_0	PF08_0013	1.087313	0.713453	0.024593	-0.53193	-0.72979
intPF08_0014_2	PF08_0014	-0.78756	-1.35621	-0.4026	0.780263	1.266873
aPF08_0014_0	PF08_0014	-0.78756	-1.35621	-0.4026	0.780263	1.266873
aPF08_0015_0	PF08_0015	0.404472	0.179472	-0.66876	-0.45771	0.524312
intPF08_0016_2	PF08_0016	0.832237	0.567297	0.267897	-0.26406	-0.78646
intPF08_0015_2	PF08_0016	0.404472	0.179472	-0.66876	-0.45771	0.524312
aPF08_0016_1	PF08_0016	0.832237	0.567297	0.267897	-0.26406	-0.78646
aPF08_0016_0	PF08_0016	0.832237	0.567297	0.267897	-0.26406	-0.78646
intPF08_0017_2	PF08_0017	-0.14989	-0.18243	-0.16679	-0.02497	0.19667
aPF08_0017_1	PF08_0017	-0.14989	-0.18243	-0.16679	-0.02497	0.19667
aPF08_0017_0	PF08_0017	-0.14989	-0.18243	-0.16679	-0.02497	0.19667
aPF08_0018_2	PF08_0018	-0.16955	0.104425	0.324315	0.206015	-0.15943
aPF08_0018_1	PF08_0018	-0.16955	0.104425	0.324315	0.206015	-0.15943
aPF08_0018_0	PF08_0018	-0.16955	0.104425	0.324315	0.206015	-0.15943
intPF08_0019_2	PF08_0019	-0.40423	0.66113	1.13074	0.59626	-0.60313
aPF08_0019_0	PF08_0019	-0.40423	0.66113	1.13074	0.59626	-0.60313
intPF08_0020_2	PF08_0020	-0.08108	-0.1296	-0.30554	0.01554	0.5241
aPF08_0020_1	PF08_0020	-0.08108	-0.1296	-0.30554	0.01554	0.5241
aPF08_0020_0	PF08_0020	-0.08108	-0.1296	-0.30554	0.01554	0.5241
intPF08_0021_2	PF08_0021	-1.11111	-1.11398	-4.67E-05	0.925213	1.015643
aPF08_0021_1	PF08_0021	-1.11111	-1.11398	-4.67E-05	0.925213	1.015643
aPF08_0021_0	PF08_0021	-1.11111	-1.11398	-4.67E-05	0.925213	1.015643
intPF08_0022_2	PF08_0022	-0.61178	-0.85908	-0.96658	0.068417	1.505917
aPF08_0022_1	PF08_0022	-0.61178	-0.85908	-0.96658	0.068417	1.505917
aPF08_0022_0	PF08_0022	-0.61178	-0.85908	-0.96658	0.068417	1.505917
intPF08_0023_2	PF08_0023	-1.43917	0.01883	1.40283	1.10917	-0.41583
aPF08_0023_1	PF08_0023	-1.43917	0.01883	1.40283	1.10917	-0.41583
aPF08_0023_0	PF08_0023	-1.43917	0.01883	1.40283	1.10917	-0.41583
intPF08_0025_2	PF08_0024	0.276807	-0.82404	-0.1179	0.559237	0.413377
intMAL8P1.25_2	PF08_0024	-0.10217	-0.34682	-0.29151	0.182068	0.610378
aPF08_0024_0	PF08_0024	-0.0732	-0.15819	-0.03498	0.11951	0.16234
aPF08_0025_0	PF08_0025	0.276807	-0.82404	-0.1179	0.559237	0.413377
intPF08_0026_3	PF08_0026	1.10844	0.81929	0.41542	-0.57592	-1.44879
aPF08_0026_1	PF08_0026	1.10844	0.81929	0.41542	-0.57592	-1.44879
aPF08_0026_0	PF08_0026	1.10844	0.81929	0.41542	-0.57592	-1.44879
intPF08_0027_2	PF08_0027	0.681277	0.808607	0.545027	-0.27869	-1.10527
aPF08_0027_1	PF08_0027	0.681277	0.808607	0.545027	-0.27869	-1.10527
aPF08_0027_0	PF08_0027	0.681277	0.808607	0.545027	-0.27869	-1.10527
intPF08_0028_2	PF08_0028	0.06071	0.16195	0.25085	0.10315	-0.21745
aPF08_0028_1	PF08_0028	0.06071	0.16195	0.25085	0.10315	-0.21745

aPF08_0028_0	PF08_0028	0.06071	0.16195	0.25085	0.10315	-0.21745
intPF08_0029_2	PF08_0029	-0.30355	-0.12329	0.865095	0.736455	-0.46232
aPF08_0029_1	PF08_0029	-0.30355	-0.12329	0.865095	0.736455	-0.46232
aPF08_0029_0	PF08_0029	-0.30355	-0.12329	0.865095	0.736455	-0.46232
intMAL8P1.35_2	PF08_0030	-1.18145	-0.57026	1.13202	1.27798	-0.14474
aPF08_0030_1	PF08_0030	-0.34413	-0.64966	0.10098	0.55272	0.32556
aPF08_0030_0	PF08_0030	-0.34413	-0.64966	0.10098	0.55272	0.32556
intPF08_0031_2	PF08_0031	-0.95073	-0.48133	0.58716	0.94184	0.33233
aPF08_0031_0	PF08_0031	-0.95073	-0.48133	0.58716	0.94184	0.33233
aPF08_0032_1	PF08_0032	-0.53961	-0.13842	0.210097	0.538237	0.509757
aPF08_0032_0	PF08_0032	-0.53961	-0.13842	0.210097	0.538237	0.509757
intPF08_0033_2	PF08_0033	0.077317	0.704437	-0.15436	-0.4703	0.021397
aPF08_0033_1	PF08_0033	0.077317	0.704437	-0.15436	-0.4703	0.021397
aPF08_0033_0	PF08_0033	0.077317	0.704437	-0.15436	-0.4703	0.021397
intPF08_0034_2	PF08_0034	-0.43186	0.013288	0.192598	0.205848	0.108488
intMAL8P1.38_2	PF08_0034	-0.05594	-0.0313	0.163617	0.118217	-0.12486
aPF08_0034_2	PF08_0034	-0.43186	0.013288	0.192598	0.205848	0.108488
aPF08_0034_1	PF08_0034	-0.43186	0.013288	0.192598	0.205848	0.108488
aPF08_0034_0	PF08_0034	-0.43186	0.013288	0.192598	0.205848	0.108488
intPF08_0035_2	PF08_0035	1.140227	-0.16912	-2.46675	-2.49941	0.280457
aPF08_0035_1	PF08_0035	1.140227	-0.16912	-2.46675	-2.49941	0.280457
aPF08_0035_0	PF08_0035	1.140227	-0.16912	-2.46675	-2.49941	0.280457
intPF08_0036_2	PF08_0036	0.221447	0.234747	-0.33124	-0.37292	0.130587
aPF08_0036_1	PF08_0036	0.221447	0.234747	-0.33124	-0.37292	0.130587
aPF08_0036_0	PF08_0036	0.221447	0.234747	-0.33124	-0.37292	0.130587
intPF08_0037_2	PF08_0037	0.131427	0.370877	0.915747	0.410587	-0.85954
intMAL8P1.49_2	PF08_0037	1.299698	1.071158	0.644688	-0.32947	-1.39526
aPF08_0037_0	PF08_0037	0.131427	0.370877	0.915747	0.410587	-0.85954
intPF08_0038_2	PF08_0038	-0.70191	-0.31295	0.378143	0.618023	0.259113
intMAL8P1.50_2	PF08_0038	-0.55884	-0.5648	0.429083	0.839583	0.282463
aPF08_0038_1	PF08_0038	-0.70191	-0.31295	0.378143	0.618023	0.259113
aPF08_0038_0	PF08_0038	-0.70191	-0.31295	0.378143	0.618023	0.259113
intPF08_0039_3	PF08_0039	-0.08071	0.20857	0.85636	0.60364	-0.49857
aPF08_0039_0	PF08_0039	-0.08071	0.20857	0.85636	0.60364	-0.49857
intPF08_0041_2	PF08_0040	2.309057	1.495727	0.666557	-0.80122	-2.19639
intPF08_0040_2	PF08_0040	0.985703	0.598963	0.499813	-0.16315	-1.0903
aPF08_0040_1	PF08_0040	0.985703	0.598963	0.499813	-0.16315	-1.0903
aPF08_0040_0	PF08_0040	0.985703	0.598963	0.499813	-0.16315	-1.0903
aPF08_0041_0	PF08_0041	2.309057	1.495727	0.666557	-0.80122	-2.19639
intPF08_0042_2	PF08_0042	1.09182	0.78788	0.33073	-0.71423	-1.51638
aPF08_0042_1	PF08_0042	1.09182	0.78788	0.33073	-0.71423	-1.51638
aPF08_0042_0	PF08_0042	1.09182	0.78788	0.33073	-0.71423	-1.51638
intPF08_0043_2	PF08_0043					
aPF08_0043_0	PF08_0043					
intPF08_0044_2	PF08_0044	1.335677	0.446617	-0.35453	-0.94373	-0.91308
aPF08_0044_1	PF08_0044	1.335677	0.446617	-0.35453	-0.94373	-0.91308
aPF08_0044_0	PF08_0044	1.335677	0.446617	-0.35453	-0.94373	-0.91308

intPF08_0045_2	PF08_0045	-0.19255	-0.63929	-0.56069	0.225353	1.003953
aPF08_0045_1	PF08_0045	-0.19255	-0.63929	-0.56069	0.225353	1.003953
aPF08_0045_0	PF08_0045	-0.19255	-0.63929	-0.56069	0.225353	1.003953
intPF08_0046_2	PF08_0046	-0.31913	-0.27685	0.404943	0.568723	0.032513
aPF08_0046_1	PF08_0046	-0.31913	-0.27685	0.404943	0.568723	0.032513
aPF08_0046_0	PF08_0046	-0.31913	-0.27685	0.404943	0.568723	0.032513
intPF08_0047_2	PF08_0047	0.63367	0.50967	-0.28508	-0.75492	-0.48117
aPF08_0047_1	PF08_0047	0.63367	0.50967	-0.28508	-0.75492	-0.48117
aPF08_0047_0	PF08_0047	0.63367	0.50967	-0.28508	-0.75492	-0.48117
intPF08_0048_2	PF08_0048	0.43623	0.56151	0.41665	-0.07787	-0.65173
aPF08_0048_3	PF08_0048	0.43623	0.56151	0.41665	-0.07787	-0.65173
aPF08_0048_2	PF08_0048	0.43623	0.56151	0.41665	-0.07787	-0.65173
aPF08_0048_1	PF08_0048	0.43623	0.56151	0.41665	-0.07787	-0.65173
aPF08_0048_0	PF08_0048	0.43623	0.56151	0.41665	-0.07787	-0.65173
intPF08_0049_2	PF08_0049	0.186063	0.059703	0.319593	0.238073	-0.25104
intMAL8P1.60_2	PF08_0049	-0.58177	-0.49451	-0.15274	0.196345	0.438785
aPF08_0049_0	PF08_0049	0.186063	0.059703	0.319593	0.238073	-0.25104
intPF08_0050_2	PF08_0050	0.732818	0.201578	0.145748	-0.12947	-0.5713
aPF08_0050_1	PF08_0050	0.732818	0.201578	0.145748	-0.12947	-0.5713
aPF08_0050_0	PF08_0050	0.732818	0.201578	0.145748	-0.12947	-0.5713
intPF08_0051_2	PF08_0051	-0.11763	-0.01733	0.644917	0.468917	-0.45133
aPF08_0051_1	PF08_0051	-0.11763	-0.01733	0.644917	0.468917	-0.45133
aPF08_0051_0	PF08_0051	-0.11763	-0.01733	0.644917	0.468917	-0.45133
intPF08_0052_2	PF08_0052	0.924647	0.598747	-0.10924	-0.52542	-0.49691
aPF08_0052_1	PF08_0052	0.924647	0.598747	-0.10924	-0.52542	-0.49691
aPF08_0052_0	PF08_0052	0.924647	0.598747	-0.10924	-0.52542	-0.49691
aPF08_0053_0	PF08_0053	0.776023	0.736873	0.709723	-0.2405	-1.42933
intPF08_0054_2	PF08_0054	-0.19877	0.090377	0.717997	0.540837	-0.37854
aPF08_0054_1	PF08_0054	-0.19877	0.090377	0.717997	0.540837	-0.37854
aPF08_0054_0	PF08_0054	-0.19877	0.090377	0.717997	0.540837	-0.37854
intPF08_0055_2	PF08_0055	0.535705	0.437975	0.378305	-0.27042	-0.98576
aPF08_0055_1	PF08_0055	0.535705	0.437975	0.378305	-0.27042	-0.98576
aPF08_0055_0	PF08_0055	0.535705	0.437975	0.378305	-0.27042	-0.98576
intPF08_0056_2	PF08_0056	0.518253	0.127713	-0.34592	-0.66242	-0.44705
aPF08_0056_0	PF08_0056	0.518253	0.127713	-0.34592	-0.66242	-0.44705
intPF08_0057_2	PF08_0057	-0.96669	-1.57555	-1.02641	0.521073	1.935213
intMAL8P1.73_2	PF08_0057	-0.65105	-0.98573	-2.36854	-1.10996	2.23723
aPF08_0057_0	PF08_0057	-0.96669	-1.57555	-1.02641	0.521073	1.935213
intPF08_0058_2	PF08_0058	0.093043	-0.64122	-2.09232	-1.44602	1.208383
aPF08_0058_0	PF08_0058	0.093043	-0.64122	-2.09232	-1.44602	1.208383
intPF08_0059_2	PF08_0059	-0.27849	-0.47553	-0.07658	0.53528	0.71913
intMAL8P1.74_2	PF08_0059	-0.68271	-0.86945	0.229893	0.897773	0.573113
aPF08_0059_0	PF08_0059	-0.27849	-0.47553	-0.07658	0.53528	0.71913
intPF08_0060_2	PF08_0060	-2.13261	-0.98104	1.10838	1.53573	0.3284
aPF08_0060_3	PF08_0060	-2.13261	-0.98104	1.10838	1.53573	0.3284
aPF08_0060_2	PF08_0060	-2.13261	-0.98104	1.10838	1.53573	0.3284
aPF08_0060_1	PF08_0060	-2.13261	-0.98104	1.10838	1.53573	0.3284

aPF08_0060_0	PF08_0060	-2.13261	-0.98104	1.10838	1.53573	0.3284
intPF08_0061_3	PF08_0061	0.991727	0.115357	-0.38321	-0.43395	-0.20202
aPF08_0061_1	PF08_0061	0.991727	0.115357	-0.38321	-0.43395	-0.20202
aPF08_0061_0	PF08_0061	0.991727	0.115357	-0.38321	-0.43395	-0.20202
intMAL8P1.76_2	PF08_0062	0.067032	-0.15327	0.107062	0.112732	-0.15171
intMAL8P1.75_2	PF08_0062	-0.65752	-0.0249	1.045053	0.818613	-0.48544
aPF08_0062_0	PF08_0062	-0.38141	0.16614	0.02467	-7.00E-05	0.19256
intPF08_0063_2	PF08_0063	-0.83995	-0.08276	0.66002	0.62198	-0.05224
aPF08_0063_1	PF08_0063	-0.83995	-0.08276	0.66002	0.62198	-0.05224
aPF08_0063_0	PF08_0063	-0.83995	-0.08276	0.66002	0.62198	-0.05224
intPF08_0064_2	PF08_0064	-0.28483	-0.77883	-1.00783	-0.00283	1.419167
aPF08_0064_0	PF08_0064	-0.28483	-0.77883	-1.00783	-0.00283	1.419167
intPF08_0065_3	PF08_0065	0.80141	0.97447	0.70732	-0.43382	-1.56497
aPF08_0065_1	PF08_0065	0.80141	0.97447	0.70732	-0.43382	-1.56497
aPF08_0065_0	PF08_0065	0.80141	0.97447	0.70732	-0.43382	-1.56497
intPF08_0066_2	PF08_0066	-0.98589	-0.87258	-0.03835	0.694013	0.858243
aPF08_0066_1	PF08_0066	-0.98589	-0.87258	-0.03835	0.694013	0.858243
aPF08_0066_0	PF08_0066	-0.98589	-0.87258	-0.03835	0.694013	0.858243
intPF08_0067_2	PF08_0067	-0.40026	-0.11171	0.222923	0.438743	0.308373
aPF08_0067_0	PF08_0067	-0.40026	-0.11171	0.222923	0.438743	0.308373
intPF08_0068_2	PF08_0068	-0.80272	-0.43139	0.23478	0.50622	0.30889
aPF08_0068_1	PF08_0068	-0.80272	-0.43139	0.23478	0.50622	0.30889
aPF08_0068_0	PF08_0068	-0.80272	-0.43139	0.23478	0.50622	0.30889
intPF08_0069_2	PF08_0069	-0.21913	0.343677	0.560397	0.316937	-0.24984
aPF08_0069_1	PF08_0069	-0.21913	0.343677	0.560397	0.316937	-0.24984
aPF08_0069_0	PF08_0069	-0.21913	0.343677	0.560397	0.316937	-0.24984
intMAL8P1.86_2	PF08_0070	-0.7214	-0.47498	0.75895	0.97505	-0.00502
aPF08_0070_1	PF08_0070	0.801162	0.217902	0.039342	-0.43383	-0.84466
aPF08_0070_0	PF08_0070	0.801162	0.217902	0.039342	-0.43383	-0.84466
intPF08_0071_2	PF08_0071	-0.01689	-0.10497	0.039677	0.154617	0.094827
aPF08_0071_0	PF08_0071	-0.01689	-0.10497	0.039677	0.154617	0.094827
aMAL8P1.87_0	PF08_0071	0.251593	0.559043	0.277413	-0.26775	-0.62138
aPF08_0072_0	PF08_0072	3.074375	1.465125	-0.30659	-1.77857	-2.15383
intPF08_0073_2	PF08_0073	0.82655	0.80423	0.16654	-0.62804	-0.98673
intPF08_0072_2	PF08_0073	3.074375	1.465125	-0.30659	-1.77857	-2.15383
aPF08_0073_1	PF08_0073	0.82655	0.80423	0.16654	-0.62804	-0.98673
aPF08_0073_0	PF08_0073	0.82655	0.80423	0.16654	-0.62804	-0.98673
intPF08_0074_2	PF08_0074	0.266607	0.244077	0.295717	0.047857	-0.3965
aPF08_0074_0	PF08_0074	0.266607	0.244077	0.295717	0.047857	-0.3965
intPF08_0075_2	PF08_0075	0.174353	0.450213	0.757073	0.337593	-0.63955
aPF08_0075_0	PF08_0075	0.174353	0.450213	0.757073	0.337593	-0.63955
intPF08_0076_3	PF08_0076	0.557807	0.413957	0.743597	0.279737	-0.85362
aPF08_0076_0	PF08_0076	0.557807	0.413957	0.743597	0.279737	-0.85362
aPF08_0077_1	PF08_0077	0.75076	0.10219	-0.06688	-0.46712	-0.76019
aPF08_0077_0	PF08_0077	0.75076	0.10219	-0.06688	-0.46712	-0.76019
intPF08_0078_2	PF08_0078	-0.16111	-0.27046	0.274752	0.287032	-0.19977
intPF08_0077_2	PF08_0078	0.75076	0.10219	-0.06688	-0.46712	-0.76019

aPF08_0078_2	PF08_0078	-0.16111	-0.27046	0.274752	0.287032	-0.19977
aPF08_0078_1	PF08_0078	-0.16111	-0.27046	0.274752	0.287032	-0.19977
aPF08_0078_0	PF08_0078	-0.16111	-0.27046	0.274752	0.287032	-0.19977
intPF08_0079_2	PF08_0079	0.231493	0.256523	0.492963	0.272703	-0.40086
aPF08_0079_0	PF08_0079	0.231493	0.256523	0.492963	0.272703	-0.40086
intPF08_0080_2	PF08_0080	-0.88893	-0.90386	0.20771	0.88729	0.64386
aPF08_0080_1	PF08_0080	-0.88893	-0.90386	0.20771	0.88729	0.64386
aPF08_0080_0	PF08_0080	-0.88893	-0.90386	0.20771	0.88729	0.64386
intPF08_0081_2	PF08_0081	0.60999	0.56707	0.47011	-0.29861	-1.13507
aPF08_0081_1	PF08_0081	0.60999	0.56707	0.47011	-0.29861	-1.13507
aPF08_0081_0	PF08_0081	0.60999	0.56707	0.47011	-0.29861	-1.13507
aMAL8P1.94_0	PF08_0081	0.504727	0.064347	0.327797	-0.09046	-0.86201
intPF08_0082_2	PF08_0082	-0.08976	-0.20568	-1.78589	-1.21627	1.342517
aPF08_0082_1	PF08_0082	-0.08976	-0.20568	-1.78589	-1.21627	1.342517
aPF08_0082_0	PF08_0082	-0.08976	-0.20568	-1.78589	-1.21627	1.342517
intPF08_0083_3	PF08_0083	1.05355	0.24575	-0.3865	-0.923	-0.86325
aPF08_0083_1	PF08_0083	1.05355	0.24575	-0.3865	-0.923	-0.86325
aPF08_0083_0	PF08_0083	1.05355	0.24575	-0.3865	-0.923	-0.86325
intPF08_0085_2	PF08_0084	0.235807	-0.26502	0.038057	0.310277	0.140357
intPF08_0084_2	PF08_0084	0.5742	0.10499	0.38852	0.18648	-0.55399
aPF08_0084_0	PF08_0084	0.5742	0.10499	0.38852	0.18648	-0.55399
aPF08_0085_0	PF08_0085	0.235807	-0.26502	0.038057	0.310277	0.140357
intPF08_0086_2	PF08_0086	0.821653	0.578723	0.034043	-0.45138	-0.62756
aPF08_0086_1	PF08_0086	0.821653	0.578723	0.034043	-0.45138	-0.62756
aPF08_0086_0	PF08_0086	0.821653	0.578723	0.034043	-0.45138	-0.62756
intPF08_0087_1	PF08_0087	-0.51943	-0.19788	0.522503	0.714163	0.154543
aPF08_0087_1	PF08_0087	-0.51943	-0.19788	0.522503	0.714163	0.154543
aPF08_0087_0	PF08_0087	-0.51943	-0.19788	0.522503	0.714163	0.154543
intPF08_0088_2	PF08_0088	0.495067	0.637147	-0.38803	-0.77563	-0.19231
aPF08_0088_1	PF08_0088	0.495067	0.637147	-0.38803	-0.77563	-0.19231
aPF08_0088_0	PF08_0088	0.495067	0.637147	-0.38803	-0.77563	-0.19231
intPF08_0089_2	PF08_0089	0.082237	0.054317	-0.19863	-0.03728	0.317017
aPF08_0089_3	PF08_0089	0.082237	0.054317	-0.19863	-0.03728	0.317017
aPF08_0089_2	PF08_0089	0.082237	0.054317	-0.19863	-0.03728	0.317017
aPF08_0089_1	PF08_0089	0.082237	0.054317	-0.19863	-0.03728	0.317017
aPF08_0089_0	PF08_0089	0.082237	0.054317	-0.19863	-0.03728	0.317017
intPF08_0090_2	PF08_0090	-0.10114	-0.4793	-1.45166	-0.81584	1.1518
aPF08_0090_1	PF08_0090	-0.10114	-0.4793	-1.45166	-0.81584	1.1518
aPF08_0090_0	PF08_0090	-0.10114	-0.4793	-1.45166	-0.81584	1.1518
intPF08_0091_3	PF08_0091	-1.12426	-1.31673	-1.34879	0.056453	2.054393
aPF08_0091_1	PF08_0091	-1.12426	-1.31673	-1.34879	0.056453	2.054393
aPF08_0091_0	PF08_0091	-1.12426	-1.31673	-1.34879	0.056453	2.054393
intPF08_0092_2	PF08_0092	1.010387	0.929067	0.512117	-0.44028	-1.31673
aPF08_0092_1	PF08_0092	1.010387	0.929067	0.512117	-0.44028	-1.31673
aPF08_0092_0	PF08_0092	1.010387	0.929067	0.512117	-0.44028	-1.31673
intMAL8P1.105_2	PF08_0093	0.266113	0.403923	0.211153	-0.03549	-0.25976
aPF08_0093_1	PF08_0093	-0.35685	-0.25525	-0.32499	0.146327	0.764087

aPF08_0093_0	PF08_0093	-0.35685	-0.25525	-0.32499	0.146327	0.764087
intPF08_0094_2	PF08_0094	-0.74926	-0.91273	-0.91879	0.008453	1.338393
aPF08_0094_1	PF08_0094	-0.74926	-0.91273	-0.91879	0.008453	1.338393
aPF08_0094_0	PF08_0094	-0.74926	-0.91273	-0.91879	0.008453	1.338393
intMAL8P1.107_2	PF08_0095	0.223013	0.269943	0.603123	0.366543	-0.45028
aPF08_0095_1	PF08_0095	-0.55198	-0.0511	1.03495	0.89605	-0.4034
aPF08_0095_0	PF08_0095	-0.55198	-0.0511	1.03495	0.89605	-0.4034
intPF08_0096_3	PF08_0096	0.190327	0.137367	0.720277	0.361557	-0.75003
aPF08_0096_1	PF08_0096	0.190327	0.137367	0.720277	0.361557	-0.75003
aPF08_0096_0	PF08_0096	0.190327	0.137367	0.720277	0.361557	-0.75003
intPF08_0097_2	PF08_0097	-0.72797	-0.03913	0.483237	0.462437	0.024287
aPF08_0097_1	PF08_0097	-0.72797	-0.03913	0.483237	0.462437	0.024287
aPF08_0097_0	PF08_0097	-0.72797	-0.03913	0.483237	0.462437	0.024287
aPF08_0098_1	PF08_0098	-0.60439	-0.72806	0.50611	0.80989	0.00306
aPF08_0098_0	PF08_0098	-0.60439	-0.72806	0.50611	0.80989	0.00306
intPF08_0099_2	PF08_0099	0.864833	0.140813	-0.01313	0.024793	-0.13315
aPF08_0099_0	PF08_0099	0.864833	0.140813	-0.01313	0.024793	-0.13315
intPF08_0100_2	PF08_0100	-0.66476	-0.41923	-0.63204	-0.1093	0.936893
aPF08_0100_1	PF08_0100	-0.66476	-0.41923	-0.63204	-0.1093	0.936893
aPF08_0100_0	PF08_0100	-0.66476	-0.41923	-0.63204	-0.1093	0.936893
intPF08_0101_2	PF08_0101	-0.57012	-0.2489	0.051543	0.178623	0.197563
aPF08_0101_1	PF08_0101	-0.57012	-0.2489	0.051543	0.178623	0.197563
aPF08_0101_0	PF08_0101	-0.57012	-0.2489	0.051543	0.178623	0.197563
intPF08_0102_2	PF08_0102	-0.25056	0.39182	0.32012	0.05288	-0.16282
aPF08_0102_1	PF08_0102	-0.25056	0.39182	0.32012	0.05288	-0.16282
aPF08_0102_0	PF08_0102	-0.25056	0.39182	0.32012	0.05288	-0.16282
intPF08_0108_2	PF08_0108	1.46425	0.64777	-1.45529	-2.47671	-1.10327
aPF08_0108_1	PF08_0108	1.46425	0.64777	-1.45529	-2.47671	-1.10327
aPF08_0108_0	PF08_0108	1.46425	0.64777	-1.45529	-2.47671	-1.10327
intPF08_0109_2	PF08_0109	-0.56873	-0.19987	-0.08926	0.22926	0.56387
intMAL8P1.126_2	PF08_0109	0.169727	0.456347	-0.5782	-0.85646	0.022987
aPF08_0109_1	PF08_0109	-0.56873	-0.19987	-0.08926	0.22926	0.56387
aPF08_0109_0	PF08_0109	-0.56873	-0.19987	-0.08926	0.22926	0.56387
intPF08_0110_3	PF08_0110	-0.05169	-0.11659	0.198673	0.339993	0.085253
aPF08_0110_0	PF08_0110	-0.05169	-0.11659	0.198673	0.339993	0.085253
intMAL8P1.130_2	PF08_0111	0.20608	-0.10942	0.57508	0.57992	-0.32608
aPF08_0111_1	PF08_0111	0.254667	0.703687	0.008127	-0.42179	-0.26335
aPF08_0111_0	PF08_0111	0.254667	0.703687	0.008127	-0.42179	-0.26335
intMAL8P1.131_2	PF08_0112	0.44065	0.30177	0.58097	0.26403	-0.61477
aPF08_0112_1	PF08_0112	0.55421	0.01596	-0.23967	-0.04083	0.20404
aPF08_0112_0	PF08_0112	0.55421	0.01596	-0.23967	-0.04083	0.20404
intPF08_0113_3	PF08_0113	0.28415	0.00575	-0.37549	-0.57351	-0.26275
aPF08_0113_1	PF08_0113	0.28415	0.00575	-0.37549	-0.57351	-0.26275
aPF08_0113_0	PF08_0113	0.28415	0.00575	-0.37549	-0.57351	-0.26275
aPF08_0114_3	PF08_0114	0.42699	0.59137	-0.07841	-0.33225	-0.10271
aPF08_0114_2	PF08_0114	0.42699	0.59137	-0.07841	-0.33225	-0.10271
aPF08_0114_1	PF08_0114	0.42699	0.59137	-0.07841	-0.33225	-0.10271

aPF08_0114_0	PF08_0114	0.42699	0.59137	-0.07841	-0.33225	-0.10271
aPF08_0115_1	PF08_0115	0.582097	0.167997	-0.16548	-0.48868	-0.51166
aPF08_0115_0	PF08_0115	0.582097	0.167997	-0.16548	-0.48868	-0.51166
intPF08_0117_2	PF08_0116	0.316437	0.707807	0.332137	-0.0963	-0.38697
aPF08_0116_1	PF08_0116	-0.02674	0.307683	0.138393	-0.03823	-0.09702
intPF08_0116_2	PF08_0117	-0.02674	0.307683	0.138393	-0.03823	-0.09702
intMAL8P1.136_2	PF08_0117	0.70028	0.13509	0.11557	0.01043	-0.33959
aPF08_0117_1	PF08_0117	0.316437	0.707807	0.332137	-0.0963	-0.38697
aPF08_0117_0	PF08_0117	0.316437	0.707807	0.332137	-0.0963	-0.38697
intPF08_0118_2	PF08_0118	1.551023	0.739893	-1.02054	-1.9733	-1.11273
aPF08_0118_1	PF08_0118	1.551023	0.739893	-1.02054	-1.9733	-1.11273
aPF08_0118_0	PF08_0118	1.551023	0.739893	-1.02054	-1.9733	-1.11273
intPF08_0119_2	PF08_0119	2.674347	0.595277	-1.49554	-2.66612	-1.75194
aPF08_0119_0	PF08_0119	2.674347	0.595277	-1.49554	-2.66612	-1.75194
intPF08_0120_2	PF08_0120	0.35287	-0.00783	-0.0131	-0.2279	-0.44067
aPF08_0120_1	PF08_0120	0.35287	-0.00783	-0.0131	-0.2279	-0.44067
aPF08_0120_0	PF08_0120	0.35287	-0.00783	-0.0131	-0.2279	-0.44067
intPF08_0122_2	PF08_0121	1.551013	1.062923	-0.30769	-1.12865	-0.93726
aPF08_0121_0	PF08_0121	1.063043	0.749763	0.099473	-0.46281	-0.7311
intPF08_0121_2	PF08_0122	1.063043	0.749763	0.099473	-0.46281	-0.7311
aPF08_0122_2	PF08_0122	1.551013	1.062923	-0.30769	-1.12865	-0.93726
aPF08_0122_1	PF08_0122	1.551013	1.062923	-0.30769	-1.12865	-0.93726
intPF08_0123_2	PF08_0123	0.720763	0.403683	0.287893	-0.06973	-0.60352
aPF08_0123_1	PF08_0123	0.720763	0.403683	0.287893	-0.06973	-0.60352
aPF08_0123_0	PF08_0123	0.720763	0.403683	0.287893	-0.06973	-0.60352
intPF08_0124_2	PF08_0124	0.072807	0.074967	0.461577	0.171257	-0.56363
aPF08_0124_1	PF08_0124	0.072807	0.074967	0.461577	0.171257	-0.56363
aPF08_0124_0	PF08_0124	0.072807	0.074967	0.461577	0.171257	-0.56363
intPF08_0125_2	PF08_0125	-0.55858	-0.86712	0.19575	0.91325	0.61912
aPF08_0125_1	PF08_0125	-0.55858	-0.86712	0.19575	0.91325	0.61912
aPF08_0125_0	PF08_0125	-0.55858	-0.86712	0.19575	0.91325	0.61912
intPF08_0126_2	PF08_0126	-0.81231	-0.76699	-0.56261	0.274233	1.202233
aPF08_0126_1	PF08_0126	-0.81231	-0.76699	-0.56261	0.274233	1.202233
aPF08_0126_0	PF08_0126	-0.81231	-0.76699	-0.56261	0.274233	1.202233
intPF08_0127_2	PF08_0127	0.4882	0.50498	0.42229	-0.19479	-0.89248
aPF08_0127_1	PF08_0127	0.4882	0.50498	0.42229	-0.19479	-0.89248
aPF08_0127_0	PF08_0127	0.4882	0.50498	0.42229	-0.19479	-0.89248
intMAL8P1.148_2	PF08_0128	0.147907	0.333457	0.703087	0.361247	-0.57412
aPF08_0128_1	PF08_0128	0.22016	0.14521	-0.06116	-0.09584	-0.00821
aPF08_0128_0	PF08_0128	0.22016	0.14521	-0.06116	-0.09584	-0.00821
intPF08_0129_2	PF08_0129	0.054677	-0.70489	-2.04371	-1.08095	1.637227
aPF08_0129_1	PF08_0129	0.054677	-0.70489	-2.04371	-1.08095	1.637227
aPF08_0129_0	PF08_0129	0.054677	-0.70489	-2.04371	-1.08095	1.637227
aPF08_0130_1	PF08_0130	0.783297	0.885037	0.860437	-0.1941	-1.5397
aPF08_0130_0	PF08_0130	0.783297	0.885037	0.860437	-0.1941	-1.5397
intPF08_0131_2	PF08_0131	-1.62318	-1.23309	0.482227	1.374207	0.896227
aPF08_0131_0	PF08_0131	-1.62318	-1.23309	0.482227	1.374207	0.896227

intPF08_0132_2	PF08_0132	-2.35613	-0.94934	1.20401	1.70977	0.50978
aPF08_0132_2	PF08_0132	-2.35613	-0.94934	1.20401	1.70977	0.50978
aPF08_0132_1	PF08_0132	-2.35613	-0.94934	1.20401	1.70977	0.50978
aPF08_0132_0	PF08_0132	-2.35613	-0.94934	1.20401	1.70977	0.50978
intPF08_0135_2	PF08_0134	0.750713	0.796443	0.762373	-0.18921	-1.40078
intPF08_0134_2	PF08_0134	-0.23066	-0.86573	0.053953	0.679713	0.420393
aPF08_0134_0	PF08_0134	-0.23066	-0.86573	0.053953	0.679713	0.420393
aPF08_0135_1	PF08_0135	0.750713	0.796443	0.762373	-0.18921	-1.40078
aPF08_0135_0	PF08_0135	0.750713	0.796443	0.762373	-0.18921	-1.40078
intPF08_0136_2	PF08_0136					
aPF08_0136_0	PF08_0136					
aMAL8P1.158_0	PF08_0136	0.65339	0.17804	0.31543	-0.07643	-0.79204
intPF08_0136b_2	PF08_0136b					
aPF08_0136b_0	PF08_0136b					
intPF08_0137_3	PF08_0137	0.637927	0.342847	0.207057	-0.23822	-0.72101
aPF08_0137_1	PF08_0137	0.637927	0.342847	0.207057	-0.23822	-0.72101
aPF08_0137_0	PF08_0137	0.637927	0.342847	0.207057	-0.23822	-0.72101
intPF10_0007_2	PF10_0007	0.28351	0.80046	0.45558	-0.21758	-0.72246
aPF10_0008_0	PF10_0007	0.001643	0.742763	0.210963	-0.2353	-0.2451
aPF10_0007_0	PF10_0007	0.28351	0.80046	0.45558	-0.21758	-0.72246
aPF10_0011_0	PF10_0012					
intPF10_0013_2	PF10_0013	-1.00624	0.326207	1.992077	1.244257	-1.23287
aPF10_0013_0	PF10_0013	-1.00624	0.326207	1.992077	1.244257	-1.23287
intPF10_0015_2	PF10_0015	2.64761	1.28594	-0.70389	-2.36411	-2.28094
aPF10_0015_0	PF10_0015	2.64761	1.28594	-0.70389	-2.36411	-2.28094
aPF10_0014_0	PF10_0015	2.307713	0.765433	-0.59736	-2.01198	-2.18177
intPF10_0016_2	PF10_0016	-1.25627	-1.96362	0.801605	2.062305	0.733525
aPF10_0016_0	PF10_0016	-1.25627	-1.96362	0.801605	2.062305	0.733525
aPF10_0017_0	PF10_0017	-0.057	-0.75704	0.229083	0.833583	0.344703
intPF10_0018_2	PF10_0018	1.906837	2.070297	0.585917	-1.69358	-2.86996
intPF10_0017_2	PF10_0018	-0.057	-0.75704	0.229083	0.833583	0.344703
aPF10_0018_1	PF10_0018	1.906837	2.070297	0.585917	-1.69358	-2.86996
aPF10_0018_0	PF10_0018	1.906837	2.070297	0.585917	-1.69358	-2.86996
intPF10_0019_2	PF10_0019	2.435377	2.403517	0.148667	-1.97653	-2.44678
aPF10_0019_0	PF10_0019	2.435377	2.403517	0.148667	-1.97653	-2.44678
intPF10_0020_2	PF10_0020	-1.18922	-2.00886	-1.51628	0.620117	2.767197
aPF10_0020_1	PF10_0020	-1.18922	-2.00886	-1.51628	0.620117	2.767197
aPF10_0020_0	PF10_0020	-1.18922	-2.00886	-1.51628	0.620117	2.767197
intPF10_0021_2	PF10_0021	1.297543	0.896303	-0.69911	-1.27223	-0.46164
aPF10_0021_0	PF10_0021	1.297543	0.896303	-0.69911	-1.27223	-0.46164
intPF10_0022_2	PF10_0022	2.47021	0.99195	-0.7289	-1.8021	-1.52895
aPF10_0022_0	PF10_0022	2.47021	0.99195	-0.7289	-1.8021	-1.52895
intPF10_0023_2	PF10_0023	2.198553	1.078243	-0.17399	-1.56435	-2.04158
aPF10_0023_0	PF10_0023	2.198553	1.078243	-0.17399	-1.56435	-2.04158
intPF10_0024_2	PF10_0024	3.213463	1.663203	-1.00765	-2.83169	-2.36254
aPF10_0024_1	PF10_0024	3.213463	1.663203	-1.00765	-2.83169	-2.36254
aPF10_0024_0	PF10_0024	3.213463	1.663203	-1.00765	-2.83169	-2.36254

intPF10_0025_2	PF10_0025	2.950453	1.279753	-0.85927	-2.57807	-2.42209
aPF10_0025_1	PF10_0025	2.950453	1.279753	-0.85927	-2.57807	-2.42209
aPF10_0025_0	PF10_0025	2.950453	1.279753	-0.85927	-2.57807	-2.42209
intPF10_0026_2	PF10_0026	0.05783	-0.56617	0.36408	0.53292	-0.26483
aPF10_0026_1	PF10_0026	0.05783	-0.56617	0.36408	0.53292	-0.26483
aPF10_0026_0	PF10_0026	0.05783	-0.56617	0.36408	0.53292	-0.26483
intPF10_0027_2	PF10_0027	1.33334	1.27828	0.81893	-0.33843	-1.60128
aPF10_0027_1	PF10_0027	1.33334	1.27828	0.81893	-0.33843	-1.60128
aPF10_0027_0	PF10_0027	1.33334	1.27828	0.81893	-0.33843	-1.60128
intPF10_0028_1	PF10_0028-a	0.360933	0.049313	-0.08964	-0.1337	-0.12365
aPF10_0028_0	PF10_0028-a	0.360933	0.049313	-0.08964	-0.1337	-0.12365
intPF10_0030_2	PF10_0029	-0.36685	-0.46776	0.14053	0.50697	0.31326
aPF10_0029_0	PF10_0029	0.780117	0.462427	0.235147	-0.18881	-0.66609
intPF10_0029_2	PF10_0030	0.780117	0.462427	0.235147	-0.18881	-0.66609
aPF10_0030_1	PF10_0030	-0.36685	-0.46776	0.14053	0.50697	0.31326
aPF10_0030_0	PF10_0030	-0.36685	-0.46776	0.14053	0.50697	0.31326
intPF10_0031_2	PF10_0031	-0.342	0.392963	-0.75217	-0.50317	1.000203
aPF10_0031_1	PF10_0031	-0.342	0.392963	-0.75217	-0.50317	1.000203
aPF10_0031_0	PF10_0031	-0.342	0.392963	-0.75217	-0.50317	1.000203
intPF10_0032_2	PF10_0032	-0.77258	-0.32826	0.042938	0.489118	0.693648
aPF10_0032_2	PF10_0032	-0.77258	-0.32826	0.042938	0.489118	0.693648
aPF10_0032_1	PF10_0032	-0.77258	-0.32826	0.042938	0.489118	0.693648
aPF10_0032_0	PF10_0032	-0.77258	-0.32826	0.042938	0.489118	0.693648
intPF10_0033_2	PF10_0033	0.03495	-0.55673	-0.46854	0.14332	0.69117
aPF10_0033_0	PF10_0033	0.03495	-0.55673	-0.46854	0.14332	0.69117
intPF10_0034_2	PF10_0034	0.156287	-0.25945	-2.15935	-1.50831	1.471287
aPF10_0034_1	PF10_0034	0.156287	-0.25945	-2.15935	-1.50831	1.471287
aPF10_0034_0	PF10_0034	0.156287	-0.25945	-2.15935	-1.50831	1.471287
intPF10_0035_2	PF10_0035	-0.71564	-0.40478	0.602567	0.672767	-0.12688
aPF10_0035_0	PF10_0035	-0.71564	-0.40478	0.602567	0.672767	-0.12688
intPF10_0036_2	PF10_0036	-0.66325	-0.26775	0.593783	0.355483	-0.51279
aPF10_0036_0	PF10_0036	-0.66325	-0.26775	0.593783	0.355483	-0.51279
intPF10_0037_2	PF10_0037	0.335033	0.121573	-1.08866	-1.15943	0.226093
aPF10_0037_3	PF10_0037	0.335033	0.121573	-1.08866	-1.15943	0.226093
aPF10_0037_2	PF10_0037	0.335033	0.121573	-1.08866	-1.15943	0.226093
aPF10_0037_1	PF10_0037	0.335033	0.121573	-1.08866	-1.15943	0.226093
aPF10_0037_0	PF10_0037	0.335033	0.121573	-1.08866	-1.15943	0.226093
intPF10_0038_2	PF10_0038	0.16737	0.34713	0.84173	0.46627	-0.66813
aPF10_0038_0	PF10_0038	0.16737	0.34713	0.84173	0.46627	-0.66813
intPF10_0039_2	PF10_0039	1.077723	-0.44163	-1.09574	-1.5186	-0.92271
aPF10_0039_0	PF10_0039	1.077723	-0.44163	-1.09574	-1.5186	-0.92271
intPF10_0040_2	PF10_0040	-0.10797	0.089813	-0.08363	-0.08121	0.127353
aPF10_0040_1	PF10_0040	-0.10797	0.089813	-0.08363	-0.08121	0.127353
aPF10_0040_0	PF10_0040	-0.10797	0.089813	-0.08363	-0.08121	0.127353
intPF10_0041_2	PF10_0041	0.499223	0.739363	0.608533	-0.02137	-0.8212
aPF10_0041_1	PF10_0041	0.499223	0.739363	0.608533	-0.02137	-0.8212
aPF10_0041_0	PF10_0041	0.499223	0.739363	0.608533	-0.02137	-0.8212

intPF10_0042_2	PF10_0042	0.676083	0.710583	0.251333	-0.27367	-0.62292
aPF10_0042_0	PF10_0042	0.676083	0.710583	0.251333	-0.27367	-0.62292
intPF10_0043_2	PF10_0043	0.255093	0.467523	0.808953	0.369713	-0.70186
aPF10_0043_0	PF10_0043	0.255093	0.467523	0.808953	0.369713	-0.70186
intPF10_0044_2	PF10_0044	0.596957	0.798707	-0.04166	-0.4385	-0.24787
aPF10_0044_1	PF10_0044	0.596957	0.798707	-0.04166	-0.4385	-0.24787
aPF10_0044_0	PF10_0044	0.596957	0.798707	-0.04166	-0.4385	-0.24787
intPF10_0045_2	PF10_0045	-1.02889	-0.51192	-0.31964	0.351177	1.114457
aPF10_0045_4	PF10_0045	-1.02889	-0.51192	-0.31964	0.351177	1.114457
aPF10_0045_3	PF10_0045	-1.02889	-0.51192	-0.31964	0.351177	1.114457
aPF10_0045_2	PF10_0045	-1.02889	-0.51192	-0.31964	0.351177	1.114457
aPF10_0045_1	PF10_0045	-1.02889	-0.51192	-0.31964	0.351177	1.114457
aPF10_0045_0	PF10_0045	-1.02889	-0.51192	-0.31964	0.351177	1.114457
aPF10_0046_1	PF10_0046	0.29887	0.31667	-0.1906	-0.4074	-0.15017
aPF10_0046_0	PF10_0046	0.29887	0.31667	-0.1906	-0.4074	-0.15017
intPF10_0047_2	PF10_0047	-1.91715	-0.72726	1.094263	1.313903	0.118923
aPF10_0047_1	PF10_0047	-1.91715	-0.72726	1.094263	1.313903	0.118923
aPF10_0047_0	PF10_0047	-1.91715	-0.72726	1.094263	1.313903	0.118923
aPF10_0048_0	PF10_0048	-0.58646	-0.77449	-0.6319	0.146395	1.057235
intPF10_0049_2	PF10_0049	-0.33004	-0.9288	-0.9444	-0.1896	0.9378
intPF10_0048_2	PF10_0049	-0.58646	-0.77449	-0.6319	0.146395	1.057235
aPF10_0049_1	PF10_0049	-0.33004	-0.9288	-0.9444	-0.1896	0.9378
aPF10_0049_0	PF10_0049	-0.33004	-0.9288	-0.9444	-0.1896	0.9378
intPF10_0051_2	PF10_0050	-0.13388	0.211363	0.328273	0.262393	-0.0227
aPF10_0050_0	PF10_0050	-0.95762	-1.0009	-0.02619	0.811527	0.917237
intPF10_0050_2	PF10_0051	-0.95762	-1.0009	-0.02619	0.811527	0.917237
aPF10_0051_0	PF10_0051	-0.13388	0.211363	0.328273	0.262393	-0.0227
intPF10_0052_2	PF10_0052	-1.33539	-1.00307	0.436412	1.205972	0.785302
aPF10_0052_0	PF10_0052	-1.33539	-1.00307	0.436412	1.205972	0.785302
intPF10_0054_2	PF10_0053	0.81638	0.68308	0.6076	-0.3156	-1.40408
intPF10_0053_3	PF10_0053	1.02922	0.36092	0.07116	-0.34766	-0.75442
aPF10_0053_1	PF10_0053	1.02922	0.36092	0.07116	-0.34766	-0.75442
aPF10_0053_0	PF10_0053	1.02922	0.36092	0.07116	-0.34766	-0.75442
aPF10_0054_1	PF10_0054	0.81638	0.68308	0.6076	-0.3156	-1.40408
aPF10_0054_0	PF10_0054	0.81638	0.68308	0.6076	-0.3156	-1.40408
aPF10_0055_0	PF10_0055	0.037572	-0.32516	0.266062	0.447082	-0.04644
intPF10_0056_2	PF10_0056	-0.23789	-0.11145	-0.04221	0.126773	0.272533
intPF10_0055_2	PF10_0056	0.037572	-0.32516	0.266062	0.447082	-0.04644
aPF10_0056_0	PF10_0056	-0.23789	-0.11145	-0.04221	0.126773	0.272533
intPF10_0057_2	PF10_0057	-0.69306	-0.20105	0.086415	0.384475	0.515265
aPF10_0057_2	PF10_0057	-0.69306	-0.20105	0.086415	0.384475	0.515265
aPF10_0057_1	PF10_0057	-0.69306	-0.20105	0.086415	0.384475	0.515265
aPF10_0057_0	PF10_0057	-0.69306	-0.20105	0.086415	0.384475	0.515265
aPF10_0058_2	PF10_0057a	0.037947	0.344297	0.210737	-0.18818	-0.45174
aPF10_0058_1	PF10_0057a	0.037947	0.344297	0.210737	-0.18818	-0.45174
intPF10_0058_2	PF10_0058	0.037947	0.344297	0.210737	-0.18818	-0.45174
aPF10_0058_0	PF10_0058	0.037947	0.344297	0.210737	-0.18818	-0.45174

intPF10_0059_2	PF10_0059	0.319497	-0.2045	-1.3415	-0.94716	0.795837
aPF10_0059_0	PF10_0059	0.319497	-0.2045	-1.3415	-0.94716	0.795837
intPF10_0060_2	PF10_0060	0.20105	-0.24473	-0.90379	-0.29371	0.98623
aPF10_0060_1	PF10_0060	0.20105	-0.24473	-0.90379	-0.29371	0.98623
aPF10_0060_0	PF10_0060	0.20105	-0.24473	-0.90379	-0.29371	0.98623
intPF10_0061_2	PF10_0061	1.27323	0.49783	-0.71766	-0.96534	-0.22483
aPF10_0061_1	PF10_0061	1.27323	0.49783	-0.71766	-0.96534	-0.22483
aPF10_0061_0	PF10_0061	1.27323	0.49783	-0.71766	-0.96534	-0.22483
intPF10_0062_2	PF10_0062	0.803097	0.171507	-0.2345	-0.33516	-0.22617
aPF10_0062_0	PF10_0062	0.803097	0.171507	-0.2345	-0.33516	-0.22617
intPF10_0063_1	PF10_0063	0.21937	-0.206	0.48658	0.52186	-0.31972
aPF10_0063_0	PF10_0063	0.21937	-0.206	0.48658	0.52186	-0.31972
intPF10_0065_2	PF10_0064	-0.99337	-0.58617	0.81185	1.14415	0.16817
aPF10_0064_1	PF10_0064	-1.21638	-0.59257	0.860397	1.154937	0.165407
aPF10_0064_0	PF10_0064	-1.21638	-0.59257	0.860397	1.154937	0.165407
intPF10_0064_2	PF10_0065	-1.21638	-0.59257	0.860397	1.154937	0.165407
aPF10_0065_0	PF10_0065	-0.99337	-0.58617	0.81185	1.14415	0.16817
intPF10_0067_2	PF10_0066	1.019137	0.447307	0.627887	-0.25355	-1.57297
intPF10_0066_3	PF10_0066	-0.21999	-0.31508	0.778663	0.794003	-0.37326
aPF10_0066_0	PF10_0066	-0.21999	-0.31508	0.778663	0.794003	-0.37326
aPF10_0067_0	PF10_0067	1.019137	0.447307	0.627887	-0.25355	-1.57297
intPF10_0068_3	PF10_0068	0.475892	0.230252	0.503142	0.064372	-0.83101
aPF10_0068_0	PF10_0068	0.475892	0.230252	0.503142	0.064372	-0.83101
intPF10_0069_2	PF10_0069	0.20602	-0.1461	0.0232	0.2438	0.1571
aPF10_0069_0	PF10_0069	0.20602	-0.1461	0.0232	0.2438	0.1571
intPF10_0070_2	PF10_0070	-0.5051	0.07948	0.57355	0.52045	-0.04948
aPF10_0070_0	PF10_0070	-0.5051	0.07948	0.57355	0.52045	-0.04948
intPF10_0071_2	PF10_0071	-0.28363	-1.04487	-1.37852	-0.38482	1.348033
aPF10_0071_1	PF10_0071	-0.28363	-1.04487	-1.37852	-0.38482	1.348033
aPF10_0071_0	PF10_0071	-0.28363	-1.04487	-1.37852	-0.38482	1.348033
intPF10_0073_2	PF10_0072	-0.14671	-0.25041	-0.34568	-0.24998	0.140747
intPF10_0072_2	PF10_0072	0.795733	0.138833	-1.92616	-2.00618	0.349333
aPF10_0072_1	PF10_0072	0.795733	0.138833	-1.92616	-2.00618	0.349333
aPF10_0072_0	PF10_0072	0.795733	0.138833	-1.92616	-2.00618	0.349333
aPF10_0073_0	PF10_0073	-0.14671	-0.25041	-0.34568	-0.24998	0.140747
intPF10_0074_2	PF10_0074	-0.05202	-0.04994	0.143383	0.228283	0.063603
aPF10_0074_0	PF10_0074	-0.05202	-0.04994	0.143383	0.228283	0.063603
intPF10_0075_2	PF10_0075	-2.26719	-1.36343	0.871185	1.696035	0.793655
aPF10_0075_2	PF10_0075	-2.26719	-1.36343	0.871185	1.696035	0.793655
aPF10_0075_1	PF10_0075	-2.26719	-1.36343	0.871185	1.696035	0.793655
aPF10_0075_0	PF10_0075	-2.26719	-1.36343	0.871185	1.696035	0.793655
intPF10_0076_2	PF10_0076	0.345137	0.259807	0.266887	-0.09055	-0.56697
aPF10_0076_1	PF10_0076	0.345137	0.259807	0.266887	-0.09055	-0.56697
aPF10_0076_0	PF10_0076	0.345137	0.259807	0.266887	-0.09055	-0.56697
intPF10_0077_2	PF10_0077	-0.14317	0.252813	0.648873	0.365793	-0.42465
aPF10_0077_1	PF10_0077	-0.14317	0.252813	0.648873	0.365793	-0.42465
aPF10_0077_0	PF10_0077	-0.14317	0.252813	0.648873	0.365793	-0.42465

intPF10_0078_2	PF10_0078	-0.06302	0.028072	-0.23428	-0.08614	0.340012
aPF10_0078_3	PF10_0078	-0.06302	0.028072	-0.23428	-0.08614	0.340012
aPF10_0078_2	PF10_0078	-0.06302	0.028072	-0.23428	-0.08614	0.340012
aPF10_0078_1	PF10_0078	-0.06302	0.028072	-0.23428	-0.08614	0.340012
aPF10_0078_0	PF10_0078	-0.06302	0.028072	-0.23428	-0.08614	0.340012
intPF10_0079_2	PF10_0079	-0.08147	0.050013	-0.06112	-0.14167	-0.03996
aPF10_0079_5	PF10_0079	-0.08147	0.050013	-0.06112	-0.14167	-0.03996
aPF10_0079_4	PF10_0079	-0.08147	0.050013	-0.06112	-0.14167	-0.03996
aPF10_0079_3	PF10_0079	-0.08147	0.050013	-0.06112	-0.14167	-0.03996
aPF10_0079_2	PF10_0079	-0.08147	0.050013	-0.06112	-0.14167	-0.03996
aPF10_0079_1	PF10_0079	-0.08147	0.050013	-0.06112	-0.14167	-0.03996
aPF10_0079_0	PF10_0079	-0.08147	0.050013	-0.06112	-0.14167	-0.03996
intPF10_0080_2	PF10_0080	-0.3676	-0.79196	-0.74332	0.077657	1.073797
aPF10_0080_1	PF10_0080	-0.3676	-0.79196	-0.74332	0.077657	1.073797
aPF10_0080_0	PF10_0080	-0.3676	-0.79196	-0.74332	0.077657	1.073797
intPF10_0082_2	PF10_0081	-0.0129	-0.0295	-0.78701	-0.43999	0.7675
intPF10_0081_3	PF10_0081	-0.91435	-0.48426	-0.15605	0.411232	0.884892
aPF10_0081_1	PF10_0081	-0.91435	-0.48426	-0.15605	0.411232	0.884892
aPF10_0081_0	PF10_0081	-0.91435	-0.48426	-0.15605	0.411232	0.884892
aPF10_0082_1	PF10_0082	-0.0129	-0.0295	-0.78701	-0.43999	0.7675
aPF10_0082_0	PF10_0082	-0.0129	-0.0295	-0.78701	-0.43999	0.7675
intPF10_0083_2	PF10_0083	-0.39423	-0.38029	-0.79926	-0.2503	0.967735
aPF10_0083_0	PF10_0083	-0.39423	-0.38029	-0.79926	-0.2503	0.967735
intPF10_0084_2	PF10_0084	-2.06277	-2.23462	-0.0925	1.707337	1.928457
aPF10_0084_1	PF10_0084	-2.06277	-2.23462	-0.0925	1.707337	1.928457
aPF10_0084_0	PF10_0084	-2.06277	-2.23462	-0.0925	1.707337	1.928457
intPF10_0085_1	PF10_0085	0.57668	0.8216	0.88331	-0.25281	-1.6431
aPF10_0085_1	PF10_0085	0.57668	0.8216	0.88331	-0.25281	-1.6431
aPF10_0085_0	PF10_0085	0.57668	0.8216	0.88331	-0.25281	-1.6431
aPF10_0086_0	PF10_0086	-0.94677	-0.17717	0.91134	1.02766	0.06817
intPF10_0088_2	PF10_0087	0.361877	0.613617	-0.09473	-0.36493	-0.08528
intPF10_0087_3	PF10_0087	0.859883	0.826573	0.798853	-0.17919	-1.46891
intPF10_0086_2	PF10_0087	-0.94677	-0.17717	0.91134	1.02766	0.06817
aPF10_0087_0	PF10_0087	0.859883	0.826573	0.798853	-0.17919	-1.46891
aPF10_0088_1	PF10_0088	0.361877	0.613617	-0.09473	-0.36493	-0.08528
aPF10_0088_0	PF10_0088	0.361877	0.613617	-0.09473	-0.36493	-0.08528
intPF10_0089_2	PF10_0089	1.362557	1.122227	0.529307	-0.53397	-1.47739
aPF10_0089_1	PF10_0089	1.362557	1.122227	0.529307	-0.53397	-1.47739
aPF10_0089_0	PF10_0089	1.362557	1.122227	0.529307	-0.53397	-1.47739
intPF10_0091_2	PF10_0090	0.234487	0.190657	0.423137	0.182797	-0.45262
intPF10_0090_2	PF10_0090	0.79178	0.27858	-0.29141	-0.28609	0.02692
aPF10_0090_1	PF10_0090	0.79178	0.27858	-0.29141	-0.28609	0.02692
aPF10_0090_0	PF10_0090	0.79178	0.27858	-0.29141	-0.28609	0.02692
aPF10_0091_0	PF10_0091	0.234487	0.190657	0.423137	0.182797	-0.45262
intPF10_0092_2	PF10_0092	-0.36906	-0.14673	0.307443	0.481223	0.183393
aPF10_0092_0	PF10_0092	-0.36906	-0.14673	0.307443	0.481223	0.183393
intPF10_0093_2	PF10_0093					

intPF10_0094_2	PF10_0094	0.132067	-0.39533	-2.12557	-1.46609	1.390167
aPF10_0094_1	PF10_0094	0.132067	-0.39533	-2.12557	-1.46609	1.390167
aPF10_0094_0	PF10_0094	0.132067	-0.39533	-2.12557	-1.46609	1.390167
aPF10_0095_1	PF10_0095	0.89368	0.93261	0.33179	-0.54979	-1.10911
aPF10_0095_0	PF10_0095	0.89368	0.93261	0.33179	-0.54979	-1.10911
intPF10_0096_2	PF10_0096	0.96696	0.85221	0.45983	-0.35733	-1.13321
intPF10_0095_2	PF10_0096	0.89368	0.93261	0.33179	-0.54979	-1.10911
aPF10_0096_1	PF10_0096	0.96696	0.85221	0.45983	-0.35733	-1.13321
aPF10_0096_0	PF10_0096	0.96696	0.85221	0.45983	-0.35733	-1.13321
intPF10_0098_2	PF10_0097	0.651227	0.188887	-0.20477	-0.43389	-0.36655
aPF10_0097_0	PF10_0097	0.06665	0.17075	-0.01099	-0.05901	0.02325
intPF10_0097_2	PF10_0098	0.06665	0.17075	-0.01099	-0.05901	0.02325
aPF10_0098_0	PF10_0098	0.651227	0.188887	-0.20477	-0.43389	-0.36655
intPF10_0099_2	PF10_0099	0.39334	0.62665	0.76837	-0.03115	-1.16876
aPF10_0099_2	PF10_0099	0.39334	0.62665	0.76837	-0.03115	-1.16876
aPF10_0099_1	PF10_0099	0.39334	0.62665	0.76837	-0.03115	-1.16876
aPF10_0099_0	PF10_0099	0.39334	0.62665	0.76837	-0.03115	-1.16876
intPF10_0100_3	PF10_0100	1.600633	0.020423	-1.33515	-1.46759	-0.27356
aPF10_0100_0	PF10_0100	1.600633	0.020423	-1.33515	-1.46759	-0.27356
intPF10_0102_2	PF10_0101	1.68067	1.60263	-0.65375	-1.67625	-0.80763
aPF10_0101_0	PF10_0101					
intPF10_0101_2	PF10_0102					
aPF10_0102_0	PF10_0102	1.68067	1.60263	-0.65375	-1.67625	-0.80763
intPF10_0103_2	PF10_0103	0.275037	0.544837	0.489317	0.116017	-0.4515
aPF10_0103_0	PF10_0103	0.275037	0.544837	0.489317	0.116017	-0.4515
intPF10_0104_2	PF10_0104	-0.23257	0.319853	0.433783	0.228883	-0.16119
aPF10_0104_0	PF10_0104	-0.23257	0.319853	0.433783	0.228883	-0.16119
intPF10_0106_2	PF10_0105	0.577117	0.859407	0.594187	-0.14285	-0.92307
aPF10_0105_0	PF10_0105	0.100003	-0.10604	0.385583	0.485083	-0.0833
intPF10_0105_2	PF10_0106	0.100003	-0.10604	0.385583	0.485083	-0.0833
aPF10_0106_0	PF10_0106	0.577117	0.859407	0.594187	-0.14285	-0.92307
intPF10_0107_2	PF10_0107	-0.89366	-1.12771	-0.08809	0.72609	0.79671
aPF10_0107_0	PF10_0107	-0.89366	-1.12771	-0.08809	0.72609	0.79671
intPF10_0109_2	PF10_0108	0.221147	0.165287	0.208927	0.046407	-0.27695
aPF10_0108_1	PF10_0108	0.43853	0.46886	0.22178	-0.10278	-0.39436
aPF10_0108_0	PF10_0108	0.43853	0.46886	0.22178	-0.10278	-0.39436
intPF10_0110_2	PF10_0109	-0.71188	-0.55011	-0.12479	0.491953	0.837273
intPF10_0108_2	PF10_0109	0.43853	0.46886	0.22178	-0.10278	-0.39436
aPF10_0109_0	PF10_0109	0.221147	0.165287	0.208927	0.046407	-0.27695
aPF10_0110_1	PF10_0110	-0.71188	-0.55011	-0.12479	0.491953	0.837273
aPF10_0110_0	PF10_0110	-0.71188	-0.55011	-0.12479	0.491953	0.837273
intPF10_0111_2	PF10_0111	-0.58906	-0.47572	0.15494	0.64306	0.55972
aPF10_0111_0	PF10_0111	-0.58906	-0.47572	0.15494	0.64306	0.55972
intPF10_0112_2	PF10_0112	0.720463	-0.0613	0.152103	-0.33344	-1.06004
aPF10_0112_0	PF10_0112	0.720463	-0.0613	0.152103	-0.33344	-1.06004
intPF10_0113_2	PF10_0113	0.26521	0.45695	0.6431	0.1399	-0.75295
aPF10_0113_1	PF10_0113	0.26521	0.45695	0.6431	0.1399	-0.75295

aPF10_0113_0	PF10_0113	0.26521	0.45695	0.6431	0.1399	-0.75295
intPF10_0114_2	PF10_0114	-0.4438	0.21351	0.52598	0.36302	-0.12951
aPF10_0114_1	PF10_0114	-0.4438	0.21351	0.52598	0.36302	-0.12951
aPF10_0114_0	PF10_0114	-0.4438	0.21351	0.52598	0.36302	-0.12951
intPF10_0115_2	PF10_0115	0.11636	-0.03789	0.14373	0.16057	-0.08671
aPF10_0115_1	PF10_0115	0.11636	-0.03789	0.14373	0.16057	-0.08671
aPF10_0115_0	PF10_0115	0.11636	-0.03789	0.14373	0.16057	-0.08671
intPF10_0117_2	PF10_0116	-0.52954	-0.0103	-0.18614	-0.08752	0.379637
intPF10_0116_2	PF10_0116	-0.85607	-1.02567	-0.37118	0.482843	1.006333
aPF10_0116_0	PF10_0116	-0.85607	-1.02567	-0.37118	0.482843	1.006333
aPF10_0117_1	PF10_0117	-0.52954	-0.0103	-0.18614	-0.08752	0.379637
aPF10_0117_0	PF10_0117	-0.52954	-0.0103	-0.18614	-0.08752	0.379637
intPF10_0118_2	PF10_0118	-0.8053	-0.96398	0.50746	0.78304	-0.13002
aPF10_0118_1	PF10_0118	-0.8053	-0.96398	0.50746	0.78304	-0.13002
aPF10_0118_0	PF10_0118	-0.8053	-0.96398	0.50746	0.78304	-0.13002
intPF10_0119_2	PF10_0119	-0.01719	-0.37901	-2.10231	-1.45761	1.420867
aPF10_0119_0	PF10_0119	-0.01719	-0.37901	-2.10231	-1.45761	1.420867
intPF10_0120_2	PF10_0120	-1.30281	-0.82493	-0.54463	0.376967	1.448267
aPF10_0120_0	PF10_0120	-1.30281	-0.82493	-0.54463	0.376967	1.448267
intPF10_0121_2	PF10_0121	-0.54637	-0.19154	1.25362	1.21808	-0.41676
aPF10_0121_0	PF10_0121	-0.54637	-0.19154	1.25362	1.21808	-0.41676
intPF10_0122_2	PF10_0122	0.27961	0.24197	0.42655	0.19595	-0.41997
aPF10_0122_1	PF10_0122	0.27961	0.24197	0.42655	0.19595	-0.41997
aPF10_0122_0	PF10_0122	0.27961	0.24197	0.42655	0.19595	-0.41997
intPF10_0123_2	PF10_0123	0.82069	0.70354	0.77692	-0.07042	-1.33454
aPF10_0123_1	PF10_0123	0.82069	0.70354	0.77692	-0.07042	-1.33454
aPF10_0123_0	PF10_0123	0.82069	0.70354	0.77692	-0.07042	-1.33454
intPF10_0125_2	PF10_0124	-0.89784	-0.86093	0.600293	1.095833	0.268553
intPF10_0124_2	PF10_0124	0.146797	-0.10931	-0.7262	-0.26824	0.807207
aPF10_0124_2	PF10_0124	0.146797	-0.10931	-0.7262	-0.26824	0.807207
aPF10_0124_1	PF10_0124	0.146797	-0.10931	-0.7262	-0.26824	0.807207
aPF10_0124_0	PF10_0124	0.146797	-0.10931	-0.7262	-0.26824	0.807207
aPF10_0125_0	PF10_0125	-0.89784	-0.86093	0.600293	1.095833	0.268553
intPF10_0126_2	PF10_0126	0.00832	0.13492	0.3964	0.0781	-0.52242
aPF10_0126_1	PF10_0126	0.00832	0.13492	0.3964	0.0781	-0.52242
aPF10_0126_0	PF10_0126	0.00832	0.13492	0.3964	0.0781	-0.52242
intPF10_0127_2	PF10_0127	-0.45319	-0.03251	0.050787	-0.01295	0.016847
aPF10_0127_1	PF10_0127	-0.45319	-0.03251	0.050787	-0.01295	0.016847
aPF10_0127_0	PF10_0127	-0.45319	-0.03251	0.050787	-0.01295	0.016847
intPF10_0128_2	PF10_0128	0.410827	0.644887	0.446487	-0.18065	-0.79655
aPF10_0128_1	PF10_0128	0.410827	0.644887	0.446487	-0.18065	-0.79655
aPF10_0128_0	PF10_0128	0.410827	0.644887	0.446487	-0.18065	-0.79655
intPF10_0129_2	PF10_0129	-0.87721	-0.48395	0.136647	0.247687	0.090787
aPF10_0129_1	PF10_0129	-0.87721	-0.48395	0.136647	0.247687	0.090787
aPF10_0129_0	PF10_0129	-0.87721	-0.48395	0.136647	0.247687	0.090787
intPF10_0130_2	PF10_0130	-0.37665	0.069243	0.435013	0.446153	0.043423
aPF10_0130_1	PF10_0130	-0.37665	0.069243	0.435013	0.446153	0.043423

aPF10_0130_0	PF10_0130	-0.37665	0.069243	0.435013	0.446153	0.043423
intPF10_0131_2	PF10_0131	0.984943	-0.08871	-0.5941	-0.80424	-0.50463
aPF10_0131_1	PF10_0131	0.984943	-0.08871	-0.5941	-0.80424	-0.50463
aPF10_0131_0	PF10_0131	0.984943	-0.08871	-0.5941	-0.80424	-0.50463
intPF10_0132_2	PF10_0132	-0.06798	-0.04657	-0.04036	0.006197	0.074907
aPF10_0132_2	PF10_0132	-0.06798	-0.04657	-0.04036	0.006197	0.074907
aPF10_0132_1	PF10_0132	-0.06798	-0.04657	-0.04036	0.006197	0.074907
aPF10_0132_0	PF10_0132	-0.06798	-0.04657	-0.04036	0.006197	0.074907
intPF10_0134_2	PF10_0133	-0.12793	0.032687	0.308637	0.332697	-0.00235
intPF10_0133_2	PF10_0133	-1.03264	-1.09976	-0.28716	0.705213	1.195313
aPF10_0133_3	PF10_0133	-1.03264	-1.09976	-0.28716	0.705213	1.195313
aPF10_0133_2	PF10_0133	-1.03264	-1.09976	-0.28716	0.705213	1.195313
aPF10_0133_1	PF10_0133	-1.03264	-1.09976	-0.28716	0.705213	1.195313
aPF10_0133_0	PF10_0133	-1.03264	-1.09976	-0.28716	0.705213	1.195313
aPF10_0134_0	PF10_0134	-0.12793	0.032687	0.308637	0.332697	-0.00235
intPF10_0135_2	PF10_0135	0.043063	-0.38934	-1.63633	-1.13601	1.022003
aPF10_0135_1	PF10_0135	0.043063	-0.38934	-1.63633	-1.13601	1.022003
aPF10_0135_0	PF10_0135	0.043063	-0.38934	-1.63633	-1.13601	1.022003
intPF10_0136_2	PF10_0136	1.138723	0.866403	-0.29805	-0.86279	-0.54974
aPF10_0136_1	PF10_0136	1.138723	0.866403	-0.29805	-0.86279	-0.54974
aPF10_0136_0	PF10_0136	1.138723	0.866403	-0.29805	-0.86279	-0.54974
intPF10_0137_2	PF10_0137					
aPF10_0137_0	PF10_0137					
intPF10_0138_2	PF10_0138	0.915682	-0.29826	-2.18107	-2.16843	0.241922
aPF10_0138_2	PF10_0138	0.915682	-0.29826	-2.18107	-2.16843	0.241922
aPF10_0138_1	PF10_0138	0.915682	-0.29826	-2.18107	-2.16843	0.241922
aPF10_0138_0	PF10_0138	0.915682	-0.29826	-2.18107	-2.16843	0.241922
intPF10_0139_2	PF10_0139	1.05892	0.58842	-0.20483	-0.51817	-0.36242
aPF10_0139_1	PF10_0139	1.05892	0.58842	-0.20483	-0.51817	-0.36242
aPF10_0139_0	PF10_0139	1.05892	0.58842	-0.20483	-0.51817	-0.36242
intPF10_0140_2	PF10_0140	-1.17779	-0.6567	-0.33592	0.406927	1.181037
aPF10_0140_3	PF10_0140	-1.17779	-0.6567	-0.33592	0.406927	1.181037
aPF10_0140_2	PF10_0140	-1.17779	-0.6567	-0.33592	0.406927	1.181037
aPF10_0140_1	PF10_0140	-1.17779	-0.6567	-0.33592	0.406927	1.181037
aPF10_0140_0	PF10_0140	-1.17779	-0.6567	-0.33592	0.406927	1.181037
intPF10_0141_2	PF10_0141	0.94405	0.50674	0.37152	-0.09052	-0.79374
aPF10_0141_0	PF10_0141	0.94405	0.50674	0.37152	-0.09052	-0.79374
intPF10_0142_3	PF10_0142	1.39615	0.79524	0.12128	-0.58278	-1.03174
aPF10_0142_1	PF10_0142	1.39615	0.79524	0.12128	-0.58278	-1.03174
aPF10_0142_0	PF10_0142	1.39615	0.79524	0.12128	-0.58278	-1.03174
intPF10_0143_3	PF10_0143	-0.21457	-0.96888	-0.18723	0.464067	0.464967
aPF10_0143_3	PF10_0143	-0.21457	-0.96888	-0.18723	0.464067	0.464967
aPF10_0143_2	PF10_0143	-0.21457	-0.96888	-0.18723	0.464067	0.464967
aPF10_0143_1	PF10_0143	-0.21457	-0.96888	-0.18723	0.464067	0.464967
aPF10_0143_0	PF10_0143	-0.21457	-0.96888	-0.18723	0.464067	0.464967
intPF10_0144_2	PF10_0144	-0.83062	0.138073	1.622353	1.133313	-0.90541
aPF10_0144_0	PF10_0144	-0.83062	0.138073	1.622353	1.133313	-0.90541

aPF10_0145_1	PF10_0145	-0.12539	-0.04703	-0.14068	-0.02598	0.229367
aPF10_0145_0	PF10_0145	-0.12539	-0.04703	-0.14068	-0.02598	0.229367
intPF10_0146_2	PF10_0146	-0.29074	-0.5733	-1.87265	-1.03051	1.602137
intPF10_0145_2	PF10_0146	-0.12539	-0.04703	-0.14068	-0.02598	0.229367
aPF10_0146_3	PF10_0146	-0.29074	-0.5733	-1.87265	-1.03051	1.602137
aPF10_0146_2	PF10_0146	-0.29074	-0.5733	-1.87265	-1.03051	1.602137
aPF10_0146_1	PF10_0146	-0.29074	-0.5733	-1.87265	-1.03051	1.602137
aPF10_0146_0	PF10_0146	-0.29074	-0.5733	-1.87265	-1.03051	1.602137
intPF10_0147_2	PF10_0147	-0.15305	0.255253	-0.11302	-0.20532	0.120413
aPF10_0147_0	PF10_0147	-0.15305	0.255253	-0.11302	-0.20532	0.120413
intPF10_0148_2	PF10_0148	0.928037	-0.00316	-0.31018	-0.26248	-0.1365
aPF10_0148_0	PF10_0148	0.928037	-0.00316	-0.31018	-0.26248	-0.1365
intPF10_0150_2	PF10_0149	0.568003	1.085483	0.734293	-0.34363	-1.32732
intPF10_0149_2	PF10_0149	1.16532	0.62841	0.78792	0.00208	-1.41091
aPF10_0149_1	PF10_0149	1.16532	0.62841	0.78792	0.00208	-1.41091
aPF10_0149_0	PF10_0149	1.16532	0.62841	0.78792	0.00208	-1.41091
aPF10_0150_1	PF10_0150	0.568003	1.085483	0.734293	-0.34363	-1.32732
aPF10_0150_0	PF10_0150	0.568003	1.085483	0.734293	-0.34363	-1.32732
intPF10_0151_2	PF10_0151	-1.05313	-0.73133	-0.1486	0.5586	0.97383
aPF10_0151_1	PF10_0151	-1.05313	-0.73133	-0.1486	0.5586	0.97383
aPF10_0151_0	PF10_0151	-1.05313	-0.73133	-0.1486	0.5586	0.97383
intPF10_0152_3	PF10_0152	-1.37302	-0.63543	1.074363	1.185803	-0.15691
aPF10_0152_1	PF10_0152	-1.37302	-0.63543	1.074363	1.185803	-0.15691
aPF10_0152_0	PF10_0152	-1.37302	-0.63543	1.074363	1.185803	-0.15691
aPF10_0153_1	PF10_0153	-1.86293	-0.47485	1.707193	1.686973	-0.29499
aPF10_0153_0	PF10_0153	-1.86293	-0.47485	1.707193	1.686973	-0.29499
intPF10_0153_2	PF10_0153a-a	-1.86293	-0.47485	1.707193	1.686973	-0.29499
intPF10_0154_2	PF10_0154	-1.36367	-1.78769	-0.23313	1.332793	1.689353
aPF10_0154_0	PF10_0154	-1.36367	-1.78769	-0.23313	1.332793	1.689353
intPF10_0155_2	PF10_0155	0.693087	0.063087	0.140337	0.219997	-0.11625
aPF10_0155_1	PF10_0155	0.693087	0.063087	0.140337	0.219997	-0.11625
aPF10_0155_0	PF10_0155	0.693087	0.063087	0.140337	0.219997	-0.11625
intPF10_0156_2	PF10_0156	0.934877	-0.01239	-1.20746	-1.4777	-0.32277
aPF10_0156_1	PF10_0156	0.934877	-0.01239	-1.20746	-1.4777	-0.32277
aPF10_0156_0	PF10_0156	0.934877	-0.01239	-1.20746	-1.4777	-0.32277
intPF10_0157_2	PF10_0157					
aPF10_0157_0	PF10_0157					
intPF10_0158_2	PF10_0158	0.64725	0.49225	0.38525	-0.22225	-0.92725
aPF10_0158_1	PF10_0158	0.64725	0.49225	0.38525	-0.22225	-0.92725
aPF10_0158_0	PF10_0158	0.64725	0.49225	0.38525	-0.22225	-0.92725
intPF10_0159_2	PF10_0159	-0.97653	0.03867	0.19266	0.59084	0.88433
aPF10_0159_0	PF10_0159	-0.97653	0.03867	0.19266	0.59084	0.88433
intPF10_0160_2	PF10_0160	0.15204	0.23878	1.04994	0.51506	-1.04378
aPF10_0160_1	PF10_0160	0.15204	0.23878	1.04994	0.51506	-1.04378
aPF10_0160_0	PF10_0160	0.15204	0.23878	1.04994	0.51506	-1.04378
aPF10_0161_1	PF10_0161	0.563387	0.936067	-0.13088	-0.68728	-0.35473
aPF10_0161_0	PF10_0161	0.563387	0.936067	-0.13088	-0.68728	-0.35473

intPF10_0161_2	PF10_0161a	0.563387	0.936067	-0.13088	-0.68728	-0.35473
intPF10_0162_2	PF10_0162	1.807647	1.311777	0.297947	-1.45511	-2.45694
aPF10_0162_1	PF10_0162	1.807647	1.311777	0.297947	-1.45511	-2.45694
aPF10_0162_0	PF10_0162	1.807647	1.311777	0.297947	-1.45511	-2.45694
intPF10_0163_2	PF10_0163	2.259102	0.921512	-0.95614	-2.42328	-2.02068
aPF10_0163_0	PF10_0163	2.259102	0.921512	-0.95614	-2.42328	-2.02068
intPF10_0164_2	PF10_0164	-0.82295	0.249738	-0.25051	-0.03937	0.841028
aPF10_0164_0	PF10_0164	-0.82295	0.249738	-0.25051	-0.03937	0.841028
intPF10_0165_2	PF10_0165	-0.65831	-0.81944	0.180637	0.836197	0.596277
aPF10_0165_1	PF10_0165	-0.65831	-0.81944	0.180637	0.836197	0.596277
aPF10_0165_0	PF10_0165	-0.65831	-0.81944	0.180637	0.836197	0.596277
intPF10_0166_2	PF10_0166	-0.39371	-1.25041	-1.63068	-0.45698	1.601747
aPF10_0166_0	PF10_0166	-0.39371	-1.25041	-1.63068	-0.45698	1.601747
intPF10_0167_2	PF10_0167	-0.73513	-1.10585	-0.24904	0.505877	0.723187
aPF10_0167_1	PF10_0167	-0.73513	-1.10585	-0.24904	0.505877	0.723187
aPF10_0167_0	PF10_0167	-0.73513	-1.10585	-0.24904	0.505877	0.723187
aPF10_0168_1	PF10_0168-a	-0.19209	-0.31405	0.153607	-0.00077	-0.42811
aPF10_0168_0	PF10_0168-a	-0.19209	-0.31405	0.153607	-0.00077	-0.42811
intPF10_0168_2	PF10_0168-b	-0.19209	-0.31405	0.153607	-0.00077	-0.42811
intPF10_0169_2	PF10_0169	0.438503	1.351503	0.950003	-0.58134	-1.85884
aPF10_0169_0	PF10_0169	0.438503	1.351503	0.950003	-0.58134	-1.85884
intPF10_0170_3	PF10_0170	2.27912	0.20838	-1.20327	-2.17173	-1.67238
aPF10_0170_0	PF10_0170	2.27912	0.20838	-1.20327	-2.17173	-1.67238
intPF10_0171_2	PF10_0171	0.937433	0.440823	0.142593	-0.14943	-0.50416
aPF10_0171_1	PF10_0171	0.937433	0.440823	0.142593	-0.14943	-0.50416
aPF10_0171_0	PF10_0171	0.937433	0.440823	0.142593	-0.14943	-0.50416
intPF10_0172_2	PF10_0172	0.867797	0.371517	-0.63402	-0.80664	-0.08918
aPF10_0172_1	PF10_0172	0.867797	0.371517	-0.63402	-0.80664	-0.08918
intPF10_0174_2	PF10_0173	-0.66475	0.031753	-0.029	0.210663	0.611913
intPF10_0173_3	PF10_0173	0.1417	0.36751	0.26648	-0.07148	-0.39801
aPF10_0173_1	PF10_0173	0.1417	0.36751	0.26648	-0.07148	-0.39801
aPF10_0173_0	PF10_0173	0.1417	0.36751	0.26648	-0.07148	-0.39801
aPF10_0174_1	PF10_0174	-0.66475	0.031753	-0.029	0.210663	0.611913
aPF10_0174_0	PF10_0174	-0.66475	0.031753	-0.029	0.210663	0.611913
intPF10_0176_2	PF10_0175	0.30182	-0.31811	-0.20529	0.06229	0.15711
intPF10_0175_3	PF10_0175	0.740247	0.319787	0.133917	-0.01358	-0.29945
aPF10_0175_1	PF10_0175	0.740247	0.319787	0.133917	-0.01358	-0.29945
aPF10_0175_0	PF10_0175	0.740247	0.319787	0.133917	-0.01358	-0.29945
aPF10_0176_0	PF10_0176	0.30182	-0.31811	-0.20529	0.06229	0.15711
aPF10_0177_4	PF10_0177a	0.179357	-0.22639	-1.11592	-0.77854	0.667327
aPF10_0177_3	PF10_0177a	0.179357	-0.22639	-1.11592	-0.77854	0.667327
aPF10_0177_2	PF10_0177a	0.179357	-0.22639	-1.11592	-0.77854	0.667327
intPF10_0177_3	PF10_0177b	0.179357	-0.22639	-1.11592	-0.77854	0.667327
aPF10_0177_1	PF10_0177b	0.179357	-0.22639	-1.11592	-0.77854	0.667327
aPF10_0177_0	PF10_0177b	0.179357	-0.22639	-1.11592	-0.77854	0.667327
intPF10_0178_2	PF10_0178	1.231463	0.288703	-0.0069	-0.57644	-1.07004
aPF10_0178_0	PF10_0178	1.231463	0.288703	-0.0069	-0.57644	-1.07004

intPF10_0179_2	PF10_0179	0.923112	0.441452	0.135102	-0.59588	-1.1549
aPF10_0179_2	PF10_0179	0.923112	0.441452	0.135102	-0.59588	-1.1549
aPF10_0179_1	PF10_0179	0.923112	0.441452	0.135102	-0.59588	-1.1549
aPF10_0179_0	PF10_0179	0.923112	0.441452	0.135102	-0.59588	-1.1549
intPF10_0180_3	PF10_0180	0.332297	0.327997	0.533267	-0.03193	-0.91766
aPF10_0180_1	PF10_0180	0.332297	0.327997	0.533267	-0.03193	-0.91766
aPF10_0180_0	PF10_0180	0.332297	0.327997	0.533267	-0.03193	-0.91766
intPF10_0181_2	PF10_0181	0.52243	0.39433	0.19585	-0.25235	-0.64883
aPF10_0181_1	PF10_0181	0.52243	0.39433	0.19585	-0.25235	-0.64883
aPF10_0181_0	PF10_0181	0.52243	0.39433	0.19585	-0.25235	-0.64883
intPF10_0182_2	PF10_0182	0.090253	-0.14127	0.212043	0.090623	-0.37707
aPF10_0182_1	PF10_0182	0.090253	-0.14127	0.212043	0.090623	-0.37707
aPF10_0182_0	PF10_0182	0.090253	-0.14127	0.212043	0.090623	-0.37707
intPF10_0183_2	PF10_0183	-0.15949	0.110203	-0.13416	-0.17543	0.109463
aPF10_0183_3	PF10_0183	-0.15949	0.110203	-0.13416	-0.17543	0.109463
aPF10_0183_2	PF10_0183	-0.15949	0.110203	-0.13416	-0.17543	0.109463
aPF10_0183_1	PF10_0183	-0.15949	0.110203	-0.13416	-0.17543	0.109463
aPF10_0183_0	PF10_0183	-0.15949	0.110203	-0.13416	-0.17543	0.109463
intPF10_0185_2	PF10_0184	0.2292	-0.06261	-0.16408	-0.20952	-0.13159
aPF10_0184_2	PF10_0184	-0.23339	-0.39372	-1.85939	-1.07306	1.614612
aPF10_0184_1	PF10_0184	-0.23339	-0.39372	-1.85939	-1.07306	1.614612
aPF10_0184_0	PF10_0184	-0.23339	-0.39372	-1.85939	-1.07306	1.614612
intPF10_0184_2	PF10_0185	-0.23339	-0.39372	-1.85939	-1.07306	1.614612
aPF10_0185_0	PF10_0185	0.2292	-0.06261	-0.16408	-0.20952	-0.13159
intPF10_0186_2	PF10_0186	-0.30718	-0.12212	-0.91661	-0.42251	1.067665
aPF10_0186_2	PF10_0186	-0.30718	-0.12212	-0.91661	-0.42251	1.067665
aPF10_0186_1	PF10_0186	-0.30718	-0.12212	-0.91661	-0.42251	1.067665
aPF10_0186_0	PF10_0186	-0.30718	-0.12212	-0.91661	-0.42251	1.067665
intPF10_0187_2	PF10_0187	0.180157	0.178207	0.847087	0.548247	-0.67187
aPF10_0187_0	PF10_0187	0.180157	0.178207	0.847087	0.548247	-0.67187
intPF10_0188_2	PF10_0188	-0.77002	-1.0904	-0.6157	0.411363	1.275563
aPF10_0188_1	PF10_0188	-0.77002	-1.0904	-0.6157	0.411363	1.275563
aPF10_0188_0	PF10_0188	-0.77002	-1.0904	-0.6157	0.411363	1.275563
intPF10_0189_2	PF10_0189	-0.52209	-0.65101	-1.57322	-0.60094	1.722847
aPF10_0189_1	PF10_0189	-0.52209	-0.65101	-1.57322	-0.60094	1.722847
aPF10_0189_0	PF10_0189	-0.52209	-0.65101	-1.57322	-0.60094	1.722847
aPF10_0190_1	PF10_0190	-0.35232	-0.74538	-1.04898	-0.11852	1.36588
aPF10_0190_0	PF10_0190	-0.35232	-0.74538	-1.04898	-0.11852	1.36588
intPF10_0192_2	PF10_0191					
intPF10_0191_2	PF10_0191	-0.97562	-1.14588	-0.24423	0.55323	0.83888
intPF10_0190_2	PF10_0191	-0.35232	-0.74538	-1.04898	-0.11852	1.36588
aPF10_0191_1	PF10_0191	-0.97562	-1.14588	-0.24423	0.55323	0.83888
aPF10_0191_0	PF10_0191	-0.97562	-1.14588	-0.24423	0.55323	0.83888
intPF10_0193_2	PF10_0193	-0.58331	-1.22141	-0.56567	-0.14499	0.199747
aPF10_0193_0	PF10_0193	-0.58331	-1.22141	-0.56567	-0.14499	0.199747
intPF10_0194_2	PF10_0194	-0.04561	-0.19897	0.084433	0.051233	-0.19537
aPF10_0194_0	PF10_0194	-0.04561	-0.19897	0.084433	0.051233	-0.19537

intPF10_0196_2	PF10_0195	-0.32378	-0.53462	-0.60076	-0.17708	0.587283
aPF10_0195_1	PF10_0195	0.284393	0.207033	-0.50907	-0.63127	0.029633
aPF10_0195_0	PF10_0195	0.284393	0.207033	-0.50907	-0.63127	0.029633
intPF10_0195_2	PF10_0196	0.284393	0.207033	-0.50907	-0.63127	0.029633
aPF10_0196_1	PF10_0196	-0.32378	-0.53462	-0.60076	-0.17708	0.587283
aPF10_0196_0	PF10_0196	-0.32378	-0.53462	-0.60076	-0.17708	0.587283
intPF10_0198_2	PF10_0197	0.586787	0.254587	0.042567	-0.08623	-0.23325
aPF10_0197_1	PF10_0197	1.31016	1.14372	0.56257	-0.49207	-1.44372
aPF10_0197_0	PF10_0197	1.31016	1.14372	0.56257	-0.49207	-1.44372
intPF10_0199_2	PF10_0198	0.307242	0.370562	0.115752	-0.05842	-0.15523
intPF10_0197_2	PF10_0198	1.31016	1.14372	0.56257	-0.49207	-1.44372
aPF10_0198_1	PF10_0198	0.586787	0.254587	0.042567	-0.08623	-0.23325
aPF10_0198_0	PF10_0198	0.586787	0.254587	0.042567	-0.08623	-0.23325
aPF10_0199_3	PF10_0199	0.307242	0.370562	0.115752	-0.05842	-0.15523
aPF10_0199_2	PF10_0199	0.307242	0.370562	0.115752	-0.05842	-0.15523
aPF10_0199_1	PF10_0199	0.307242	0.370562	0.115752	-0.05842	-0.15523
aPF10_0199_0	PF10_0199	0.307242	0.370562	0.115752	-0.05842	-0.15523
intPF10_0407_2	PF10_0200	0.692517	0.146887	0.134457	-0.18896	-0.66287
intPF10_0200_2	PF10_0200	0.648443	0.621293	0.862403	-0.04574	-1.45163
aPF10_0200_1	PF10_0200	0.648443	0.621293	0.862403	-0.04574	-1.45163
aPF10_0200_0	PF10_0200	0.648443	0.621293	0.862403	-0.04574	-1.45163
intPF10_0203_3	PF10_0203	-1.285	-0.608	-0.1135	0.580837	1.102337
aPF10_0203_0	PF10_0203	-1.285	-0.608	-0.1135	0.580837	1.102337
intPF10_0204_2	PF10_0204	-0.43592	-0.68965	0.076167	0.608407	0.458507
aPF10_0205_2	PF10_0204	0.383323	0.016043	-0.21692	-0.13442	0.078963
aPF10_0204_0	PF10_0204	-0.43592	-0.68965	0.076167	0.608407	0.458507
intPF10_0205_3	PF10_0205	0.383323	0.016043	-0.21692	-0.13442	0.078963
aPF10_0205_1	PF10_0205	0.383323	0.016043	-0.21692	-0.13442	0.078963
aPF10_0205_0	PF10_0205	0.383323	0.016043	-0.21692	-0.13442	0.078963
intPF10_0206_2	PF10_0206	-0.90233	-1.00013	-0.43162	0.480357	1.145867
aPF10_0206_1	PF10_0206	-0.90233	-1.00013	-0.43162	0.480357	1.145867
aPF10_0206_0	PF10_0206	-0.90233	-1.00013	-0.43162	0.480357	1.145867
aPF10_0207_0	PF10_0207	-0.4995	-1.14052	-0.76046	0.47346	1.51052
intPF10_0208_2	PF10_0208	-0.29603	0.09466	0.10393	0.26107	0.36284
intPF10_0207_2	PF10_0208	-0.4995	-1.14052	-0.76046	0.47346	1.51052
aPF10_0208_1	PF10_0208	-0.29603	0.09466	0.10393	0.26107	0.36284
aPF10_0208_0	PF10_0208	-0.29603	0.09466	0.10393	0.26107	0.36284
aPF10_0209_1	PF10_0209	0.680347	0.629257	0.421997	-0.17566	-0.84442
aPF10_0209_0	PF10_0209	0.680347	0.629257	0.421997	-0.17566	-0.84442
intPF10_0210_2	PF10_0210	-0.26124	-0.09741	0.40077	0.51553	0.06071
intPF10_0209_2	PF10_0210	0.680347	0.629257	0.421997	-0.17566	-0.84442
aPF10_0210_0	PF10_0210	-0.26124	-0.09741	0.40077	0.51553	0.06071
intPF10_0211_2	PF10_0211	-0.58731	-0.79304	0.063112	-0.01161	-0.46604
aPF10_0212_6	PF10_0211	-0.81482	-1.15232	-0.81095	-0.01708	0.96452
aPF10_0212_5	PF10_0211	-0.81482	-1.15232	-0.81095	-0.01708	0.96452
aPF10_0212_4	PF10_0211	-0.81482	-1.15232	-0.81095	-0.01708	0.96452
aPF10_0211_6	PF10_0211	-0.58731	-0.79304	0.063112	-0.01161	-0.46604

aPF10_0211_5	PF10_0211	-0.58731	-0.79304	0.063112	-0.01161	-0.46604
aPF10_0211_4	PF10_0211	-0.58731	-0.79304	0.063112	-0.01161	-0.46604
aPF10_0211_3	PF10_0211	-0.58731	-0.79304	0.063112	-0.01161	-0.46604
aPF10_0211_2	PF10_0211	-0.58731	-0.79304	0.063112	-0.01161	-0.46604
aPF10_0211_1	PF10_0211	-0.58731	-0.79304	0.063112	-0.01161	-0.46604
aPF10_0211_0	PF10_0211	-0.58731	-0.79304	0.063112	-0.01161	-0.46604
aPF10_0212_3	PF10_0212a	-0.81482	-1.15232	-0.81095	-0.01708	0.96452
aPF10_0212_2	PF10_0212a	-0.81482	-1.15232	-0.81095	-0.01708	0.96452
aPF10_0212_1	PF10_0212a	-0.81482	-1.15232	-0.81095	-0.01708	0.96452
aPF10_0212_0	PF10_0212a	-0.81482	-1.15232	-0.81095	-0.01708	0.96452
intPF10_0213_2	PF10_0213	-1.9894	-1.83819	-0.22446	1.309123	1.812853
aPF10_0213_3	PF10_0213	-1.9894	-1.83819	-0.22446	1.309123	1.812853
aPF10_0213_2	PF10_0213	-1.9894	-1.83819	-0.22446	1.309123	1.812853
aPF10_0213_1	PF10_0213	-1.9894	-1.83819	-0.22446	1.309123	1.812853
aPF10_0213_0	PF10_0213	-1.9894	-1.83819	-0.22446	1.309123	1.812853
intPF10_0214_2	PF10_0214	-0.40451	-0.04291	0.661567	0.596597	-0.21175
aPF10_0214_2	PF10_0214	-0.40451	-0.04291	0.661567	0.596597	-0.21175
aPF10_0214_1	PF10_0214	-0.40451	-0.04291	0.661567	0.596597	-0.21175
aPF10_0214_0	PF10_0214	-0.40451	-0.04291	0.661567	0.596597	-0.21175
intPF10_0215_2	PF10_0215	0.40685	0.33676	0.37572	-0.03722	-0.65576
aPF10_0215_0	PF10_0215	0.40685	0.33676	0.37572	-0.03722	-0.65576
aPF10_0215_1	PF10_0215a	0.40685	0.33676	0.37572	-0.03722	-0.65576
intPF10_0216_2	PF10_0216	0.778567	0.396147	0.128217	-0.25888	-0.61881
aPF10_0216_0	PF10_0216	0.778567	0.396147	0.128217	-0.25888	-0.61881
intPF10_0217_2	PF10_0217	-0.60722	0.1211	0.6453	0.5602	-0.0596
aPF10_0217_0	PF10_0217	-0.60722	0.1211	0.6453	0.5602	-0.0596
intPF10_0218_2	PF10_0218	0.325497	-0.35899	-1.08927	-0.72389	0.563327
aPF10_0218_1	PF10_0218	0.325497	-0.35899	-1.08927	-0.72389	0.563327
aPF10_0218_0	PF10_0218	0.325497	-0.35899	-1.08927	-0.72389	0.563327
intPF10_0219_2	PF10_0219	0.7524	1.03199	0.53503	-0.59653	-1.43849
aPF10_0219_1	PF10_0219	0.7524	1.03199	0.53503	-0.59653	-1.43849
aPF10_0219_0	PF10_0219	0.7524	1.03199	0.53503	-0.59653	-1.43849
intPF10_0220_2	PF10_0220	0.101495	-0.25417	-0.88639	-0.84605	0.152695
aPF10_0220_0	PF10_0220	0.101495	-0.25417	-0.88639	-0.84605	0.152695
intPF10_0221_2	PF10_0221	-0.48172	-1.07092	-0.46042	0.488583	1.023583
aPF10_0221_1	PF10_0221	-0.48172	-1.07092	-0.46042	0.488583	1.023583
aPF10_0221_0	PF10_0221	-0.48172	-1.07092	-0.46042	0.488583	1.023583
intPF10_0222_2	PF10_0222	-0.71766	-1.17069	-0.18463	0.74763	0.91869
aPF10_0222_0	PF10_0222	-0.71766	-1.17069	-0.18463	0.74763	0.91869
intPF10_0223_2	PF10_0223	0.939537	0.241337	-0.77168	-1.20098	-0.539
aPF10_0223_0	PF10_0223	0.939537	0.241337	-0.77168	-1.20098	-0.539
intPF10_0225_2	PF10_0224	0.236603	0.594963	0.836573	0.231093	-0.8693
intPF10_0224_2	PF10_0224	-0.28649	-0.32938	0.037517	0.263257	0.202017
aPF10_0224_8	PF10_0224	-0.28649	-0.32938	0.037517	0.263257	0.202017
aPF10_0224_7	PF10_0224	-0.28649	-0.32938	0.037517	0.263257	0.202017
aPF10_0224_6	PF10_0224	-0.28649	-0.32938	0.037517	0.263257	0.202017
aPF10_0224_5	PF10_0224	-0.28649	-0.32938	0.037517	0.263257	0.202017

aPF10_0224_4	PF10_0224	-0.28649	-0.32938	0.037517	0.263257	0.202017
aPF10_0224_3	PF10_0224	-0.28649	-0.32938	0.037517	0.263257	0.202017
aPF10_0224_2	PF10_0224	-0.28649	-0.32938	0.037517	0.263257	0.202017
aPF10_0224_1	PF10_0224	-0.28649	-0.32938	0.037517	0.263257	0.202017
aPF10_0224_0	PF10_0224	-0.28649	-0.32938	0.037517	0.263257	0.202017
aPF10_0225_0	PF10_0225	0.236603	0.594963	0.836573	0.231093	-0.8693
intPF10_0227_2	PF10_0226	-0.83421	-0.80995	-0.0031	0.6881	0.77595
aPF10_0226_0	PF10_0226	-0.36342	-0.71254	0.324177	0.925877	0.440597
intPF10_0226_2	PF10_0227	-0.36342	-0.71254	0.324177	0.925877	0.440597
aPF10_0227_0	PF10_0227	-0.83421	-0.80995	-0.0031	0.6881	0.77595
intPF10_0230_2	PF10_0229	0.286167	0.048187	0.027627	0.036707	-0.05885
intPF10_0229_2	PF10_0229	0.57857	0.90516	0.44167	-0.26467	-0.79616
intPF10_0228_2	PF10_0229	-0.70873	-1.01033	-0.29827	0.64981	1.08425
aPF10_0229_0	PF10_0229	0.57857	0.90516	0.44167	-0.26467	-0.79616
aPF10_0228_0	PF10_0229	-0.70873	-1.01033	-0.29827	0.64981	1.08425
aPF10_0230_1	PF10_0230	0.286167	0.048187	0.027627	0.036707	-0.05885
aPF10_0230_0	PF10_0230	0.286167	0.048187	0.027627	0.036707	-0.05885
intPF10_0231_2	PF10_0231	0.911127	-0.17711	-0.80527	-0.79539	-0.13855
aPF10_0231_1	PF10_0231	0.911127	-0.17711	-0.80527	-0.79539	-0.13855
aPF10_0231_0	PF10_0231	0.911127	-0.17711	-0.80527	-0.79539	-0.13855
intPF10_0232_2	PF10_0232	0.14256	-0.47931	-0.50744	-0.20416	0.25701
aPF10_0232_4	PF10_0232	0.14256	-0.47931	-0.50744	-0.20416	0.25701
aPF10_0232_3	PF10_0232	0.14256	-0.47931	-0.50744	-0.20416	0.25701
aPF10_0232_2	PF10_0232	0.14256	-0.47931	-0.50744	-0.20416	0.25701
aPF10_0232_1	PF10_0232	0.14256	-0.47931	-0.50744	-0.20416	0.25701
aPF10_0232_0	PF10_0232	0.14256	-0.47931	-0.50744	-0.20416	0.25701
intPF10_0233_2	PF10_0233	-0.03705	-0.72775	0.194	0.4695	-0.12425
aPF10_0233_1	PF10_0233	-0.03705	-0.72775	0.194	0.4695	-0.12425
aPF10_0233_0	PF10_0233	-0.03705	-0.72775	0.194	0.4695	-0.12425
aPF10_0234_1	PF10_0234	-0.84921	-0.72296	0.39792	0.77058	0.22746
aPF10_0234_0	PF10_0234	-0.84921	-0.72296	0.39792	0.77058	0.22746
intPF10_0236_2	PF10_0235	-0.94958	-1.22206	0.249127	1.172207	0.803397
intPF10_0234_2	PF10_0235	-0.84921	-0.72296	0.39792	0.77058	0.22746
aPF10_0235_0	PF10_0235	-1.05689	-0.66255	0.652107	1.053227	0.294887
intPF10_0235_2	PF10_0236	-1.05689	-0.66255	0.652107	1.053227	0.294887
aPF10_0236_0	PF10_0236	-0.94958	-1.22206	0.249127	1.172207	0.803397
aPF10_0237_1	PF10_0237	-0.13828	-0.07706	-0.64887	-0.31729	0.708397
aPF10_0237_0	PF10_0237	-0.13828	-0.07706	-0.64887	-0.31729	0.708397
intPF10_0238_2	PF10_0238	-0.35531	-0.87043	-0.17513	0.59613	0.75343
intPF10_0237_2	PF10_0238	-0.13828	-0.07706	-0.64887	-0.31729	0.708397
aPF10_0238_0	PF10_0238	-0.35531	-0.87043	-0.17513	0.59613	0.75343
intPF10_0241_2	PF10_0240	-0.42984	-0.88831	0.037603	0.757563	0.618473
aPF10_0240_0	PF10_0240	-0.13657	-0.26814	0.31129	0.49071	0.03214
aPF10_0239_0	PF10_0240	-0.12767	-0.43367	0.259833	0.624833	0.214333
intPF10_0240_2	PF10_0241	-0.13657	-0.26814	0.31129	0.49071	0.03214
aPF10_0241_1	PF10_0241	-0.42984	-0.88831	0.037603	0.757563	0.618473
aPF10_0241_0	PF10_0241	-0.42984	-0.88831	0.037603	0.757563	0.618473

aPF10_0242_2	PF10_0242	1.44955	1.07874	-0.0144	-1.05804	-1.32469
aPF10_0242_1	PF10_0242	1.44955	1.07874	-0.0144	-1.05804	-1.32469
aPF10_0242_0	PF10_0242	1.44955	1.07874	-0.0144	-1.05804	-1.32469
intPF10_0243_2	PF10_0243	-1.05066	-0.81869	0.521147	1.040687	0.410027
aPF10_0243_2	PF10_0243	-1.05066	-0.81869	0.521147	1.040687	0.410027
aPF10_0243_1	PF10_0243	-1.05066	-0.81869	0.521147	1.040687	0.410027
aPF10_0243_0	PF10_0243	-1.05066	-0.81869	0.521147	1.040687	0.410027
intPF10_0244_2	PF10_0244	0.86476	0.37721	-0.59192	-0.98308	-0.42621
aPF10_0244_1	PF10_0244	0.86476	0.37721	-0.59192	-0.98308	-0.42621
aPF10_0244_0	PF10_0244	0.86476	0.37721	-0.59192	-0.98308	-0.42621
intPF10_0245_2	PF10_0245	0.669537	0.356827	-0.63691	-0.72625	0.093007
aPF10_0245_1	PF10_0245	0.669537	0.356827	-0.63691	-0.72625	0.093007
aPF10_0245_0	PF10_0245	0.669537	0.356827	-0.63691	-0.72625	0.093007
aPF10_0246_1	PF10_0246	-0.83155	-0.42422	0.436447	0.786887	0.374557
aPF10_0246_0	PF10_0246	-0.83155	-0.42422	0.436447	0.786887	0.374557
intPF10_0248_2	PF10_0248	0.09582	-0.4321	-1.6305	-1.0869	1.0389
intPF10_0247_2	PF10_0248	0.11223	-0.41764	-1.62322	-1.10928	0.99614
intPF10_0246_2	PF10_0248	-0.83155	-0.42422	0.436447	0.786887	0.374557
aPF10_0248_0	PF10_0248	0.09582	-0.4321	-1.6305	-1.0869	1.0389
aPF10_0247_1	PF10_0248	0.11223	-0.41764	-1.62322	-1.10928	0.99614
aPF10_0247_0	PF10_0248	0.11223	-0.41764	-1.62322	-1.10928	0.99614
intPF10_0252_2	PF10_0250	-0.94839	-0.81204	0.35557	1.00543	0.63104
intPF10_0250_2	PF10_0250	-0.44649	-0.58552	-0.12596	0.430297	0.634857
aPF10_0251_6	PF10_0250	-0.71144	-0.62121	-0.09159	0.411027	0.607457
aPF10_0251_5	PF10_0250	-0.71144	-0.62121	-0.09159	0.411027	0.607457
aPF10_0251_4	PF10_0250	-0.71144	-0.62121	-0.09159	0.411027	0.607457
aPF10_0251_3	PF10_0250	-0.71144	-0.62121	-0.09159	0.411027	0.607457
aPF10_0251_2	PF10_0250	-0.71144	-0.62121	-0.09159	0.411027	0.607457
aPF10_0251_1	PF10_0250	-0.71144	-0.62121	-0.09159	0.411027	0.607457
aPF10_0251_0	PF10_0250	-0.71144	-0.62121	-0.09159	0.411027	0.607457
aPF10_0250_1	PF10_0250	-0.44649	-0.58552	-0.12596	0.430297	0.634857
aPF10_0250_0	PF10_0250	-0.44649	-0.58552	-0.12596	0.430297	0.634857
aPF10_0249_0	PF10_0250	-0.49436	-0.75616	-0.22908	0.421617	0.713097
intPF10_0253_2	PF10_0252	-1.1584	-1.06099	0.407997	1.178337	0.698327
aPF10_0252_0	PF10_0252	-0.94839	-0.81204	0.35557	1.00543	0.63104
aPF10_0253_0	PF10_0253	-1.1584	-1.06099	0.407997	1.178337	0.698327
intPF10_0254_2	PF10_0254	-0.1017	-0.5427	-1.6545	-0.8187	1.4773
aPF10_0256_1	PF10_0254	-0.94675	-1.05029	-2.00267	-0.58367	2.411453
aPF10_0256_0	PF10_0254	-0.94675	-1.05029	-2.00267	-0.58367	2.411453
aPF10_0255_0	PF10_0254	-0.76035	-0.70125	-1.74249	-0.68151	1.95825
aPF10_0254_0	PF10_0254	-0.1017	-0.5427	-1.6545	-0.8187	1.4773
intPF10_0258_2	PF10_0257	0.099693	0.413543	0.916903	0.365763	-0.88988
intPF10_0257_2	PF10_0257	-0.82086	-0.29869	0.88239	0.91861	-0.16531
aPF10_0257_0	PF10_0257	-0.82086	-0.29869	0.88239	0.91861	-0.16531
aPF10_0258_0	PF10_0258	0.099693	0.413543	0.916903	0.365763	-0.88988
intPF10_0260_2	PF10_0259	-1.19751	-0.47341	0.784817	1.031017	0.191247
aPF10_0259_1	PF10_0259	-0.09523	-0.51883	0.562407	0.715927	-0.27133

aPF10_0259_0	PF10_0259	-0.09523	-0.51883	0.562407	0.715927	-0.27133
intPF10_0259_2	PF10_0260	-0.09523	-0.51883	0.562407	0.715927	-0.27133
aPF10_0260_1	PF10_0260	-1.19751	-0.47341	0.784817	1.031017	0.191247
aPF10_0260_0	PF10_0260	-1.19751	-0.47341	0.784817	1.031017	0.191247
intPF10_0261_2	PF10_0261	-0.53206	-0.61132	-0.55255	0.066932	0.883202
aPF10_0261_1	PF10_0261	-0.53206	-0.61132	-0.55255	0.066932	0.883202
aPF10_0261_0	PF10_0261	-0.53206	-0.61132	-0.55255	0.066932	0.883202
intPF10_0262_2	PF10_0262	1.858085	0.525885	-1.75461	-2.75296	-1.18144
aPF10_0262_2	PF10_0262	1.858085	0.525885	-1.75461	-2.75296	-1.18144
aPF10_0262_1	PF10_0262	1.858085	0.525885	-1.75461	-2.75296	-1.18144
aPF10_0262_0	PF10_0262	1.858085	0.525885	-1.75461	-2.75296	-1.18144
intPF10_0264_2	PF10_0264	0.010567	0.440187	0.982137	0.541197	-0.69885
aPF10_0264_0	PF10_0264	0.010567	0.440187	0.982137	0.541197	-0.69885
aPF10_0263_0	PF10_0264	0.77297	0.59636	0.35512	-0.40722	-1.12196
intPF10_0265_2	PF10_0265	-0.29535	-0.07729	0.062565	0.179885	0.202055
aPF10_0265_2	PF10_0265	-0.29535	-0.07729	0.062565	0.179885	0.202055
aPF10_0265_1	PF10_0265	-0.29535	-0.07729	0.062565	0.179885	0.202055
aPF10_0265_0	PF10_0265	-0.29535	-0.07729	0.062565	0.179885	0.202055
intPF10_0266_2	PF10_0266	0.672107	0.803467	0.471817	-0.49948	-1.30263
aPF10_0266_1	PF10_0266	0.672107	0.803467	0.471817	-0.49948	-1.30263
aPF10_0266_0	PF10_0266	0.672107	0.803467	0.471817	-0.49948	-1.30263
aPF10_0267_1	PF10_0267	0.55907	0.27413	0.66098	0.15652	-0.98063
aPF10_0267_0	PF10_0267	0.55907	0.27413	0.66098	0.15652	-0.98063
intPF10_0268_3	PF10_0268	0.722073	-0.36036	-2.22779	-1.31155	1.636023
aPF10_0268_0	PF10_0268	0.722073	-0.36036	-2.22779	-1.31155	1.636023
intPF10_0270_2	PF10_0269	0.837163	0.639203	0.635583	-0.09492	-1.16054
aPF10_0269_0	PF10_0269	0.263692	0.601552	0.899592	0.221252	-1.00424
intPF10_0269_2	PF10_0270	0.263692	0.601552	0.899592	0.221252	-1.00424
aPF10_0270_0	PF10_0270	0.837163	0.639203	0.635583	-0.09492	-1.16054
intPF10_0412_2	PF10_0271					
intPF10_0271_2	PF10_0271	-0.8062	-1.528	-0.71498	0.808317	1.773337
aPF10_0271_0	PF10_0271	-0.8062	-1.528	-0.71498	0.808317	1.773337
intPF10_0272_2	PF10_0272	0.022693	0.397063	0.763143	0.513163	-0.37222
aPF10_0272_1	PF10_0272	0.022693	0.397063	0.763143	0.513163	-0.37222
aPF10_0272_0	PF10_0272	0.022693	0.397063	0.763143	0.513163	-0.37222
intPF10_0273_2	PF10_0273	0.39534	0.38231	0.18537	-0.01237	-0.23531
aPF10_0273_0	PF10_0273	0.39534	0.38231	0.18537	-0.01237	-0.23531
intPF10_0275_2	PF10_0274	0.698627	0.612847	0.329307	-0.10197	-0.58101
intPF10_0274_3	PF10_0274	0.42104	1.13429	0.99717	-0.05267	-1.29129
aPF10_0274_1	PF10_0274	0.42104	1.13429	0.99717	-0.05267	-1.29129
aPF10_0274_0	PF10_0274	0.42104	1.13429	0.99717	-0.05267	-1.29129
aPF10_0275_1	PF10_0275	0.698627	0.612847	0.329307	-0.10197	-0.58101
aPF10_0275_0	PF10_0275	0.698627	0.612847	0.329307	-0.10197	-0.58101
intPF10_0276_2	PF10_0276	-0.33177	-1.08217	-0.64066	0.37366	1.12417
aPF10_0276_0	PF10_0276	-0.33177	-1.08217	-0.64066	0.37366	1.12417
intPF10_0278_2	PF10_0277	0.96648	0.36261	0.68353	-3.00E-05	-1.33361
aPF10_0277_0	PF10_0277	0.792267	0.713687	0.761117	-0.06978	-1.28835

intPF10_0277_2	PF10_0278	0.792267	0.713687	0.761117	-0.06978	-1.28835
aPF10_0278_1	PF10_0278	0.96648	0.36261	0.68353	-3.00E-05	-1.33361
aPF10_0278_0	PF10_0278	0.96648	0.36261	0.68353	-3.00E-05	-1.33361
intPF10_0279_2	PF10_0279	0.7368	0.45002	-0.05004	-0.45846	-0.54152
aPF10_0279_1	PF10_0279	0.7368	0.45002	-0.05004	-0.45846	-0.54152
aPF10_0279_0	PF10_0279	0.7368	0.45002	-0.05004	-0.45846	-0.54152
intPF10_0280_2	PF10_0280	-0.07581	-1.05293	-0.88663	0.038967	1.007267
aPF10_0280_0	PF10_0280	-0.07581	-1.05293	-0.88663	0.038967	1.007267
intPF10_0281_2	PF10_0281	0.903443	-0.91774	-1.99729	-1.40955	0.634403
aPF10_0281_0	PF10_0281	0.903443	-0.91774	-1.99729	-1.40955	0.634403
aPF10_0282_0	PF10_0282	-0.23963	-1.30784	-0.64131	0.385647	0.962177
intPF10_0283_2	PF10_0283	0.711805	-0.10368	-0.55634	-0.98159	-0.75882
intPF10_0282_2	PF10_0283	-0.23963	-1.30784	-0.64131	0.385647	0.962177
aPF10_0283_0	PF10_0283	0.711805	-0.10368	-0.55634	-0.98159	-0.75882
intPF10_0284_2	PF10_0284	0.27832	-0.09759	0.50617	0.39983	-0.48608
aPF10_0284_2	PF10_0284	0.27832	-0.09759	0.50617	0.39983	-0.48608
aPF10_0284_1	PF10_0284	0.27832	-0.09759	0.50617	0.39983	-0.48608
aPF10_0284_0	PF10_0284	0.27832	-0.09759	0.50617	0.39983	-0.48608
intPF10_0285_2	PF10_0285	1.383833	1.333353	0.637293	-0.72663	-1.83069
aPF10_0285_1	PF10_0285	1.383833	1.333353	0.637293	-0.72663	-1.83069
aPF10_0285_0	PF10_0285	1.383833	1.333353	0.637293	-0.72663	-1.83069
aPF10_0286_1	PF10_0286	-0.6261	-0.40949	0.624487	0.804347	-0.00267
aPF10_0286_0	PF10_0286	-0.6261	-0.40949	0.624487	0.804347	-0.00267
intPF10_0287_2	PF10_0287	0.058623	0.162903	0.212193	0.046473	-0.23024
intPF10_0286_2	PF10_0287	-0.6261	-0.40949	0.624487	0.804347	-0.00267
aPF10_0287_1	PF10_0287	0.058623	0.162903	0.212193	0.046473	-0.23024
aPF10_0287_0	PF10_0287	0.058623	0.162903	0.212193	0.046473	-0.23024
intPF10_0289_2	PF10_0288	-0.3738	-0.36448	0.561467	0.875367	0.165317
intPF10_0288_2	PF10_0288	1.592045	1.236325	0.085135	-0.88929	-1.19648
aPF10_0288_0	PF10_0288	1.592045	1.236325	0.085135	-0.88929	-1.19648
aPF10_0289_1	PF10_0289	-0.3738	-0.36448	0.561467	0.875367	0.165317
aPF10_0289_0	PF10_0289	-0.3738	-0.36448	0.561467	0.875367	0.165317
intPF10_0290_2	PF10_0290					
aPF10_0290_1	PF10_0290					
aPF10_0290_0	PF10_0290					
intPF10_0291_2	PF10_0291	-0.88804	-0.46229	-0.12991	0.334747	0.728127
aPF10_0291_1	PF10_0291	-0.88804	-0.46229	-0.12991	0.334747	0.728127
aPF10_0291_0	PF10_0291	-0.88804	-0.46229	-0.12991	0.334747	0.728127
intPF10_0293_2	PF10_0292	0.145547	0.857267	0.181477	-0.42414	-0.44593
intPF10_0292_2	PF10_0292	-1.02373	-1.23201	-2.06447	-0.44775	2.633128
aPF10_0292_2	PF10_0292	-1.02373	-1.23201	-2.06447	-0.44775	2.633128
aPF10_0292_1	PF10_0292	-1.02373	-1.23201	-2.06447	-0.44775	2.633128
aPF10_0292_0	PF10_0292	-1.02373	-1.23201	-2.06447	-0.44775	2.633128
aPF10_0293_0	PF10_0293	0.145547	0.857267	0.181477	-0.42414	-0.44593
intPF10_0294_2	PF10_0294	0.375143	0.371283	0.234933	-0.09127	-0.44512
aPF10_0294_1	PF10_0294	0.375143	0.371283	0.234933	-0.09127	-0.44512
aPF10_0294_0	PF10_0294	0.375143	0.371283	0.234933	-0.09127	-0.44512

intPF10_0295_2	PF10_0295	0.629413	0.276943	-1.62515	-1.98269	-0.01978
aPF10_0295_1	PF10_0295	0.629413	0.276943	-1.62515	-1.98269	-0.01978
aPF10_0295_0	PF10_0295	0.629413	0.276943	-1.62515	-1.98269	-0.01978
intPF10_0296_2	PF10_0296	1.468053	-0.17126	-1.15574	-1.1916	-0.27208
aPF10_0296_1	PF10_0296	1.468053	-0.17126	-1.15574	-1.1916	-0.27208
aPF10_0296_0	PF10_0296	1.468053	-0.17126	-1.15574	-1.1916	-0.27208
intPF10_0298_2	PF10_0297	-0.09414	-0.03879	0.12607	0.26293	0.16879
aPF10_0297_0	PF10_0297	0.39799	0.61504	0.39492	-0.27092	-0.85304
intPF10_0297_2	PF10_0298	0.39799	0.61504	0.39492	-0.27092	-0.85304
aPF10_0298_1	PF10_0298	-0.09414	-0.03879	0.12607	0.26293	0.16879
aPF10_0298_0	PF10_0298	-0.09414	-0.03879	0.12607	0.26293	0.16879
intPF10_0300_2	PF10_0299	0.985557	1.130057	0.643297	-0.45684	-1.43978
aPF10_0299_1	PF10_0299	0.998417	0.718907	0.333687	-0.29785	-0.91207
aPF10_0299_0	PF10_0299	0.998417	0.718907	0.333687	-0.29785	-0.91207
intPF10_0301_2	PF10_0300	-0.10078	-0.54111	-1.36078	-0.69256	1.128773
intPF10_0299_2	PF10_0300	0.998417	0.718907	0.333687	-0.29785	-0.91207
aPF10_0300_0	PF10_0300	0.985557	1.130057	0.643297	-0.45684	-1.43978
aPF10_0301_0	PF10_0301	-0.10078	-0.54111	-1.36078	-0.69256	1.128773
intPF10_0302_2	PF10_0302					
aPF10_0302_0	PF10_0302					
intPF10_0303_2	PF10_0303	0.205838	0.345468	0.451888	0.075678	-0.55325
aPF10_0303_0	PF10_0303	0.205838	0.345468	0.451888	0.075678	-0.55325
intPF10_0305_2	PF10_0304	0.004287	1.312587	0.508317	-0.35198	-0.58625
intPF10_0304_2	PF10_0304	-0.2827	0.034497	0.317517	0.069817	-0.36016
aPF10_0305_0	PF10_0304	0.004287	1.312587	0.508317	-0.35198	-0.58625
aPF10_0304_0	PF10_0304	-0.2827	0.034497	0.317517	0.069817	-0.36016
intPF10_0306_2	PF10_0306	0.13731	-0.42554	-1.88017	-1.40233	1.01404
aPF10_0306_1	PF10_0306	0.13731	-0.42554	-1.88017	-1.40233	1.01404
aPF10_0306_0	PF10_0306	0.13731	-0.42554	-1.88017	-1.40233	1.01404
intPF10_0307_2	PF10_0307	-0.69421	-0.17993	0.222357	0.218977	0.032267
aPF10_0307_1	PF10_0307	-0.69421	-0.17993	0.222357	0.218977	0.032267
aPF10_0307_0	PF10_0307	-0.69421	-0.17993	0.222357	0.218977	0.032267
intPF10_0308_2	PF10_0308	0.213727	-0.49664	-1.31071	-0.87445	0.688977
aPF10_0308_1	PF10_0308	0.213727	-0.49664	-1.31071	-0.87445	0.688977
aPF10_0308_0	PF10_0308	0.213727	-0.49664	-1.31071	-0.87445	0.688977
intPF10_0309_2	PF10_0309	0.153457	0.090727	-0.2232	-0.11196	0.250607
aPF10_0309_1	PF10_0309	0.153457	0.090727	-0.2232	-0.11196	0.250607
aPF10_0309_0	PF10_0309	0.153457	0.090727	-0.2232	-0.11196	0.250607
intPF10_0310_2	PF10_0310	-0.70478	-0.20757	0.262397	0.436937	0.270907
aPF10_0310_1	PF10_0310	-0.70478	-0.20757	0.262397	0.436937	0.270907
aPF10_0310_0	PF10_0310	-0.70478	-0.20757	0.262397	0.436937	0.270907
intPF10_0311_3	PF10_0311	-0.52469	-0.92554	-0.2649	0.502237	0.796877
aPF10_0311_0	PF10_0311	-0.52469	-0.92554	-0.2649	0.502237	0.796877
aPF10_0312_1	PF10_0312	-0.3847	-0.85896	-1.18056	-0.2321	1.371797
aPF10_0312_0	PF10_0312	-0.3847	-0.85896	-1.18056	-0.2321	1.371797
intPF10_0313_2	PF10_0313	-0.12748	-0.28213	0.051253	0.313913	0.231293
aPF10_0313_0	PF10_0313	-0.12748	-0.28213	0.051253	0.313913	0.231293

intPF10_0312_2	PF10_0313a	-0.3847	-0.85896	-1.18056	-0.2321	1.371797
aPF10_0313_1	PF10_0313a	-0.12748	-0.28213	0.051253	0.313913	0.231293
intPF10_0314_3	PF10_0314	0.677063	0.648203	0.018593	-0.59093	-0.71404
aPF10_0314_1	PF10_0314	0.677063	0.648203	0.018593	-0.59093	-0.71404
aPF10_0314_0	PF10_0314	0.677063	0.648203	0.018593	-0.59093	-0.71404
intPF10_0315_2	PF10_0315	0.391187	0.048057	0.710307	0.286267	-0.9712
aPF10_0315_1	PF10_0315	0.391187	0.048057	0.710307	0.286267	-0.9712
aPF10_0315_0	PF10_0315	0.391187	0.048057	0.710307	0.286267	-0.9712
intPF10_0316_2	PF10_0316	-0.62465	-1.13875	-0.29201	0.702673	1.038413
aPF10_0316_1	PF10_0316	-0.62465	-1.13875	-0.29201	0.702673	1.038413
aPF10_0316_0	PF10_0316	-0.62465	-1.13875	-0.29201	0.702673	1.038413
intPF10_0318_2	PF10_0317	-0.25488	0.54438	0.26273	0.00227	-0.03038
aPF10_0317_0	PF10_0317	-1.31321	-0.38391	0.358817	0.819517	0.753247
intPF10_0317_2	PF10_0318	-1.31321	-0.38391	0.358817	0.819517	0.753247
aPF10_0318_0	PF10_0318	-0.25488	0.54438	0.26273	0.00227	-0.03038
aPF10_0319_1	PF10_0319	0.060787	-0.05444	-0.02687	0.024707	0.029777
aPF10_0319_0	PF10_0319	0.060787	-0.05444	-0.02687	0.024707	0.029777
intPF10_0321_2	PF10_0320	1.234537	0.866307	0.034127	-0.50229	-0.64997
aPF10_0320_2	PF10_0320	0.773418	0.329728	0.327578	-0.07288	-0.74787
aPF10_0320_1	PF10_0320	0.773418	0.329728	0.327578	-0.07288	-0.74787
aPF10_0320_0	PF10_0320	0.773418	0.329728	0.327578	-0.07288	-0.74787
aPF10_0320_3	PF10_0320a	0.773418	0.329728	0.327578	-0.07288	-0.74787
intPF10_0320_2	PF10_0321	0.773418	0.329728	0.327578	-0.07288	-0.74787
aPF10_0321_1	PF10_0321	1.234537	0.866307	0.034127	-0.50229	-0.64997
aPF10_0321_0	PF10_0321	1.234537	0.866307	0.034127	-0.50229	-0.64997
intPF10_0322_2	PF10_0322	0.053203	0.078003	0.036483	0.013513	-0.01234
aPF10_0322_2	PF10_0322	0.053203	0.078003	0.036483	0.013513	-0.01234
aPF10_0322_1	PF10_0322	0.053203	0.078003	0.036483	0.013513	-0.01234
aPF10_0322_0	PF10_0322	0.053203	0.078003	0.036483	0.013513	-0.01234
intPF10_0323_2	PF10_0323	-1.51798	-0.95608	0.61366	1.23234	0.59208
aPF10_0323_0	PF10_0323	-1.51798	-0.95608	0.61366	1.23234	0.59208
intPF10_0324_2	PF10_0324	-0.42294	-0.16991	0.993138	0.894948	-0.45063
aPF10_0324_0	PF10_0324	-0.42294	-0.16991	0.993138	0.894948	-0.45063
intPF10_0325_2	PF10_0325	-0.23694	0.17068	1.30813	0.85987	-0.91568
aPF10_0325_0	PF10_0325	-0.23694	0.17068	1.30813	0.85987	-0.91568
intPF10_0326_2	PF10_0326	0.796437	0.426827	0.045097	-0.29626	-0.50799
aPF10_0326_1	PF10_0326	0.796437	0.426827	0.045097	-0.29626	-0.50799
aPF10_0326_0	PF10_0326	0.796437	0.426827	0.045097	-0.29626	-0.50799
intPF10_0327_3	PF10_0327	0.49357	0.39167	0.1959	-0.1284	-0.45517
aPF10_0327_1	PF10_0327	0.49357	0.39167	0.1959	-0.1284	-0.45517
aPF10_0327_0	PF10_0327	0.49357	0.39167	0.1959	-0.1284	-0.45517
intPF10_0329_2	PF10_0328					
intPF10_0328_3	PF10_0328	0.10626	0.18819	0.54037	0.31063	-0.43469
aPF10_0328_1	PF10_0328	0.10626	0.18819	0.54037	0.31063	-0.43469
aPF10_0328_0	PF10_0328	0.10626	0.18819	0.54037	0.31063	-0.43469
aPF10_0329_1	PF10_0329					
aPF10_0329_0	PF10_0329					

intPF10_0330_2	PF10_0330	-0.62559	-0.91386	-1.47444	-0.158	2.065423
aPF10_0330_0	PF10_0330	-0.62559	-0.91386	-1.47444	-0.158	2.065423
aPF10_0331_1	PF10_0331	0.151887	0.154077	-0.3704	-0.24526	0.363257
aPF10_0331_0	PF10_0331	0.151887	0.154077	-0.3704	-0.24526	0.363257
intPF10_0332_2	PF10_0331a					
intPF10_0331_2	PF10_0332	0.151887	0.154077	-0.3704	-0.24526	0.363257
aPF10_0332_0	PF10_0332					
intPF10_0333_2	PF10_0333	0.410337	0.034897	0.073937	0.041357	-0.18704
aPF10_0333_1	PF10_0333	0.410337	0.034897	0.073937	0.041357	-0.18704
aPF10_0333_0	PF10_0333	0.410337	0.034897	0.073937	0.041357	-0.18704
intPF10_0335_2	PF10_0334	-0.62223	-0.27271	-0.21493	0.218595	0.747535
aPF10_0334_1	PF10_0334	-1.0647	-1.03696	0.236187	0.989147	0.714297
aPF10_0334_0	PF10_0334	-1.0647	-1.03696	0.236187	0.989147	0.714297
intPF10_0334_2	PF10_0335	-1.0647	-1.03696	0.236187	0.989147	0.714297
aPF10_0335_2	PF10_0335	-0.62223	-0.27271	-0.21493	0.218595	0.747535
aPF10_0335_1	PF10_0335	-0.62223	-0.27271	-0.21493	0.218595	0.747535
aPF10_0335_0	PF10_0335	-0.62223	-0.27271	-0.21493	0.218595	0.747535
intPF10_0337_2	PF10_0336	-0.31352	-1.2224	-1.2152	-0.02246	1.483737
aPF10_0336_0	PF10_0336	-0.4758	-1.37497	-0.54805	0.532387	1.045307
intPF10_0336_2	PF10_0337	-0.4758	-1.37497	-0.54805	0.532387	1.045307
aPF10_0337_0	PF10_0337	-0.31352	-1.2224	-1.2152	-0.02246	1.483737
intPF10_0339_2	PF10_0338	-0.85191	-0.56241	0.416337	0.701997	0.198747
intPF10_0338_2	PF10_0338	-1.016	-0.615	0.5135	0.7805	0.158
aPF10_0339_0	PF10_0338	-0.85191	-0.56241	0.416337	0.701997	0.198747
aPF10_0338_0	PF10_0338	-1.016	-0.615	0.5135	0.7805	0.158
intPF10_0340_2	PF10_0340	-0.03829	-0.36457	-0.28936	0.245023	0.667733
aPF10_0340_1	PF10_0340	-0.03829	-0.36457	-0.28936	0.245023	0.667733
aPF10_0340_0	PF10_0340	-0.03829	-0.36457	-0.28936	0.245023	0.667733
intPF10_0341_2	PF10_0341	1.141947	1.070757	0.400987	-0.64165	-1.36442
aPF10_0341_1	PF10_0341	1.141947	1.070757	0.400987	-0.64165	-1.36442
aPF10_0341_0	PF10_0341	1.141947	1.070757	0.400987	-0.64165	-1.36442
intPF10_0342_2	PF10_0342	-0.27669	-0.50809	-1.71083	-0.87451	1.578253
aPF10_0342_1	PF10_0342	-0.27669	-0.50809	-1.71083	-0.87451	1.578253
aPF10_0342_0	PF10_0342	-0.27669	-0.50809	-1.71083	-0.87451	1.578253
intPF10_0343_2	PF10_0343	-0.05138	-0.7221	-0.94331	-0.27869	0.8471
aPF10_0343_1	PF10_0343	-0.05138	-0.7221	-0.94331	-0.27869	0.8471
aPF10_0343_0	PF10_0343	-0.05138	-0.7221	-0.94331	-0.27869	0.8471
intPF10_0344_2	PF10_0344	1.046723	-0.09814	-2.02047	-1.97137	0.332803
aPF10_0344_1	PF10_0344	1.046723	-0.09814	-2.02047	-1.97137	0.332803
aPF10_0344_0	PF10_0344	1.046723	-0.09814	-2.02047	-1.97137	0.332803
intPF10_0345_2	PF10_0345	0.718883	0.747593	-1.36869	-1.58065	0.397073
aPF10_0345_0	PF10_0345	0.718883	0.747593	-1.36869	-1.58065	0.397073
intPF10_0346_2	PF10_0346	0.502993	-0.60496	-1.42683	-1.53051	-0.25088
aPF10_0346_1	PF10_0346	0.502993	-0.60496	-1.42683	-1.53051	-0.25088
aPF10_0346_0	PF10_0346	0.502993	-0.60496	-1.42683	-1.53051	-0.25088
intPF10_0347_2	PF10_0347	0.203113	-0.36556	-1.94539	-1.20395	1.449223
aPF10_0347_1	PF10_0347	0.203113	-0.36556	-1.94539	-1.20395	1.449223

aPF10_0347_0	PF10_0347	0.203113	-0.36556	-1.94539	-1.20395	1.449223
intPF10_0348_2	PF10_0348	0.08885	-0.85426	-2.27974	-1.44576	1.41176
aPF10_0348_1	PF10_0348	0.08885	-0.85426	-2.27974	-1.44576	1.41176
aPF10_0348_0	PF10_0348	0.08885	-0.85426	-2.27974	-1.44576	1.41176
intPF10_0350_3	PF10_0350	-0.96332	1.645573	2.400843	0.446323	-2.31391
aPF10_0350_1	PF10_0350	-0.96332	1.645573	2.400843	0.446323	-2.31391
aPF10_0350_0	PF10_0350	-0.96332	1.645573	2.400843	0.446323	-2.31391
aPF10_0349_0	PF10_0350	-0.0776	-0.00298	-0.91503	-0.84497	0.43398
intPF10_0351_2	PF10_0351	1.16766	-0.22081	-2.40787	-2.14513	0.66031
aPF10_0351_1	PF10_0351	1.16766	-0.22081	-2.40787	-2.14513	0.66031
aPF10_0351_0	PF10_0351	1.16766	-0.22081	-2.40787	-2.14513	0.66031
intPF10_0352_2	PF10_0352	0.617467	-0.13034	-1.67906	-1.6726	0.276677
aPF10_0352_1	PF10_0352	0.617467	-0.13034	-1.67906	-1.6726	0.276677
aPF10_0352_0	PF10_0352	0.617467	-0.13034	-1.67906	-1.6726	0.276677
intPF10_0355_2	PF10_0355	0.544063	0.105693	-0.79056	-0.7943	0.147013
aPF10_0355_1	PF10_0355	0.544063	0.105693	-0.79056	-0.7943	0.147013
aPF10_0355_0	PF10_0355	0.544063	0.105693	-0.79056	-0.7943	0.147013
aPF10_0353_0	PF10_0355					
intPF10_0356_2	PF10_0356	0.960187	0.195047	-0.00609	-0.16257	-0.42271
aPF10_0356_1	PF10_0356	0.960187	0.195047	-0.00609	-0.16257	-0.42271
aPF10_0356_0	PF10_0356	0.960187	0.195047	-0.00609	-0.16257	-0.42271
intPF10_0358_2	PF10_0357	-0.39156	-0.82023	0.275943	0.740723	0.188893
intPF10_0357_2	PF10_0357	1.23919	-0.04043	-1.78513	-2.18387	-0.44307
aPF10_0357_1	PF10_0357	1.23919	-0.04043	-1.78513	-2.18387	-0.44307
aPF10_0357_0	PF10_0357	1.23919	-0.04043	-1.78513	-2.18387	-0.44307
aPF10_0358_0	PF10_0358	-0.39156	-0.82023	0.275943	0.740723	0.188893
intPF10_0359_2	PF10_0359	0.08548	0.42758	0.96784	0.38016	-0.94758
aPF10_0359_0	PF10_0359	0.08548	0.42758	0.96784	0.38016	-0.94758
intPF10_0360_3	PF10_0360	-0.38419	-0.68304	-0.85415	0.105487	1.396877
aPF10_0360_1	PF10_0360	-0.38419	-0.68304	-0.85415	0.105487	1.396877
aPF10_0360_0	PF10_0360	-0.38419	-0.68304	-0.85415	0.105487	1.396877
intPF10_0361_2	PF10_0361	0.610852	0.242432	-0.16687	-0.46047	-0.42677
aPF10_0361_2	PF10_0361	0.610852	0.242432	-0.16687	-0.46047	-0.42677
aPF10_0361_1	PF10_0361	0.610852	0.242432	-0.16687	-0.46047	-0.42677
aPF10_0361_0	PF10_0361	0.610852	0.242432	-0.16687	-0.46047	-0.42677
intPF10_0362_2	PF10_0362	0.01677	-0.45625	-0.34095	0.20081	0.61653
aPF10_0362_3	PF10_0362	0.01677	-0.45625	-0.34095	0.20081	0.61653
aPF10_0362_2	PF10_0362	0.01677	-0.45625	-0.34095	0.20081	0.61653
aPF10_0362_1	PF10_0362	0.01677	-0.45625	-0.34095	0.20081	0.61653
aPF10_0362_0	PF10_0362	0.01677	-0.45625	-0.34095	0.20081	0.61653
aPF10_0363_1	PF10_0363	-1.04764	0.144173	1.113403	0.781263	-0.42401
aPF10_0363_0	PF10_0363	-1.04764	0.144173	1.113403	0.781263	-0.42401
intPF10_0365_2	PF10_0365	-0.17347	-0.48763	-0.19924	0.01774	0.12463
intPF10_0363_2	PF10_0365	-1.04764	0.144173	1.113403	0.781263	-0.42401
aPF10_0365_1	PF10_0365	-0.17347	-0.48763	-0.19924	0.01774	0.12463
aPF10_0365_0	PF10_0365	-0.17347	-0.48763	-0.19924	0.01774	0.12463
aPF10_0364_0	PF10_0365	-0.33845	-0.84997	-0.40771	0.132652	0.492632

intPF10_0366_3	PF10_0366	-0.33814	0.355693	1.272113	0.741553	-0.87003
aPF10_0366_0	PF10_0366	-0.33814	0.355693	1.272113	0.741553	-0.87003
intPF10_0367_2	PF10_0367	-0.30597	-0.49014	0.303527	0.136807	-0.57602
aPF10_0367_1	PF10_0367	-0.30597	-0.49014	0.303527	0.136807	-0.57602
aPF10_0367_0	PF10_0367	-0.30597	-0.49014	0.303527	0.136807	-0.57602
intPF10_0368_2	PF10_0368	0.603197	-0.40498	-1.20903	-1.05213	0.167317
aPF10_0368_1	PF10_0368	0.603197	-0.40498	-1.20903	-1.05213	0.167317
aPF10_0368_0	PF10_0368	0.603197	-0.40498	-1.20903	-1.05213	0.167317
intPF10_0369_2	PF10_0369	0.476133	0.181343	0.067313	-0.13565	-0.36468
aPF10_0369_1	PF10_0369	0.476133	0.181343	0.067313	-0.13565	-0.36468
aPF10_0369_0	PF10_0369	0.476133	0.181343	0.067313	-0.13565	-0.36468
intPF10_0370_2	PF10_0370	1.181107	1.389467	0.660067	-0.33373	-1.17813
aPF10_0370_0	PF10_0370	1.181107	1.389467	0.660067	-0.33373	-1.17813
aPF10_0371_1	PF10_0371	-0.09226	0.202837	-0.28943	-0.37873	0.128497
aPF10_0371_0	PF10_0371	-0.09226	0.202837	-0.28943	-0.37873	0.128497
aPF10_0372_0	PF10_0372-a	-0.49604	-0.4208	-0.5079	0.1239	1.0018
intPF10_0372_3	PF10_0372-b	-0.49604	-0.4208	-0.5079	0.1239	1.0018
aPF10_0373_1	PF10_0373	0.09338	0.4156	0.54631	0.16269	-0.5116
aPF10_0373_0	PF10_0373	0.09338	0.4156	0.54631	0.16269	-0.5116
intPF10_0373_2	PF10_0373a	0.09338	0.4156	0.54631	0.16269	-0.5116
intPF10_0374_2	PF10_0374	0.488617	0.138967	-0.46044	-0.50007	0.023807
aPF10_0374_9	PF10_0374	0.488617	0.138967	-0.46044	-0.50007	0.023807
aPF10_0374_8	PF10_0374	0.488617	0.138967	-0.46044	-0.50007	0.023807
aPF10_0374_7	PF10_0374	0.488617	0.138967	-0.46044	-0.50007	0.023807
aPF10_0374_5	PF10_0374	0.488617	0.138967	-0.46044	-0.50007	0.023807
aPF10_0374_13	PF10_0374	0.488617	0.138967	-0.46044	-0.50007	0.023807
aPF10_0374_1	PF10_0374	0.488617	0.138967	-0.46044	-0.50007	0.023807
aPF10_0374_0	PF10_0374	0.488617	0.138967	-0.46044	-0.50007	0.023807
intPF10_0375_2	PF10_0375	-0.98422	-0.53492	0.19284	0.45566	0.30142
aPF10_0375_1	PF10_0375	-0.98422	-0.53492	0.19284	0.45566	0.30142
aPF10_0375_0	PF10_0375	-0.98422	-0.53492	0.19284	0.45566	0.30142
intPF10_0376_2	PF10_0376	-0.12956	1.807827	0.082847	-0.84701	-0.17399
aPF10_0376_1	PF10_0376	-0.12956	1.807827	0.082847	-0.84701	-0.17399
aPF10_0376_0	PF10_0376	-0.12956	1.807827	0.082847	-0.84701	-0.17399
intPF10_0377_2	PF10_0377	1.27638	1.2251	0.00731	-0.89531	-0.9651
aPF10_0377_0	PF10_0377	1.27638	1.2251	0.00731	-0.89531	-0.9651
intPF10_0378_2	PF10_0378	1.93559	2.18107	0.12035	-1.85785	-2.19857
aPF10_0378_1	PF10_0378	1.93559	2.18107	0.12035	-1.85785	-2.19857
aPF10_0378_0	PF10_0378	1.93559	2.18107	0.12035	-1.85785	-2.19857
intPF10_0379_2	PF10_0379	-0.97432	-0.91739	0.606287	1.150047	0.326727
aPF10_0379_1	PF10_0379	-0.97432	-0.91739	0.606287	1.150047	0.326727
aPF10_0379_0	PF10_0379	-0.97432	-0.91739	0.606287	1.150047	0.326727
intPF10_0380_2	PF10_0380	0.12527	0.83214	1.33722	0.16128	-1.69664
aPF10_0380_1	PF10_0380	0.12527	0.83214	1.33722	0.16128	-1.69664
aPF10_0380_0	PF10_0380	0.12527	0.83214	1.33722	0.16128	-1.69664
intPF10_0381_2	PF10_0381	1.385417	0.502937	-0.21387	-0.82479	-0.9716
intPF10_0382_2	PF10_0382	0.886242	0.540402	0.164542	-0.51524	-1.00966

intPF10_0383_2	PF10_0383	1.40196	0.84718	-0.36386	-1.00914	-0.74918
intPF10_0384_2	PF10_0384					
intPF10_0388_2	PF10_0388	0.742427	0.657867	0.283407	-0.25777	-0.71513
intPF10_0390_2	PF10_0390					
aPF10_0390_0	PF10_0390					
intPF10_0391_2	PF10_0391	1.88325	1.76773	0.43454	-1.16654	-1.99573
intPF10_0392_2	PF10_0392	1.973187	1.754067	0.525867	-1.13553	-2.15573
aPF10_0407_1	PF10_0407	0.692517	0.146887	0.134457	-0.18896	-0.66287
aPF10_0407_0	PF10_0407	0.692517	0.146887	0.134457	-0.18896	-0.66287
intPF10_0409_2	PF10_0409					
intPF10_0371_2	PF10_0415	-0.09226	0.202837	-0.28943	-0.37873	0.128497
intPF10_0420_2	PF10_0420					
intPF11_0012_2	PF11_0012					
intPF11_0014_2	PF11_0014					
aPF11_0014_0	PF11_0014					
intPF11_0015_2	PF11_0015					
intPF11_0016_2	PF11_0016	2.062937	0.717297	-0.03324	-1.07694	-1.76392
intPF11_0023_2	PF11_0023	2.08033	1.63333	0.63833	-1.06233	-2.36833
intPF11_0024_2	PF11_0024	1.79188	1.94362	0.41477	-1.30777	-2.02362
intPF11_0025_2	PF11_0025					
intPF11_0026_2	PF11_0026					
intPF11_0033_2	PF11_0033	1.24109	0.93807	-0.35865	-1.04385	-0.69657
intPF11_0034_2	PF11_0034	0.726413	0.315913	0.102663	-0.191	-0.50625
intPF11_0035_2	PF11_0035	1.567077	1.474627	0.591757	-0.82742	-1.86529
aPF11_0035_0	PF11_0035	1.567077	1.474627	0.591757	-0.82742	-1.86529
intPF11_0036_2	PF11_0036	-0.09978	-0.10863	0.540013	0.296653	-0.58421
aPF11_0036_1	PF11_0036	-0.09978	-0.10863	0.540013	0.296653	-0.58421
aPF11_0036_0	PF11_0036	-0.09978	-0.10863	0.540013	0.296653	-0.58421
intPF11_0037_2	PF11_0037	0.676067	0.491167	-0.28532	-0.45134	-0.05183
aPF11_0037_1	PF11_0037	0.676067	0.491167	-0.28532	-0.45134	-0.05183
aPF11_0037_0	PF11_0037	0.676067	0.491167	-0.28532	-0.45134	-0.05183
intPF11_0038_2	PF11_0038	1.402207	1.017967	0.254067	-0.89873	-1.60263
aPF11_0038_0	PF11_0038	1.402207	1.017967	0.254067	-0.89873	-1.60263
intPF11_0039_3	PF11_0039	3.318377	1.931137	-0.45177	-2.84489	-3.2578
aPF11_0039_0	PF11_0039	3.318377	1.931137	-0.45177	-2.84489	-3.2578
intPF11_0040_2	PF11_0040	3.11706	1.5887	-0.9089	-2.8841	-2.6397
aPF11_0040_0	PF11_0040	3.11706	1.5887	-0.9089	-2.8841	-2.6397
intPF11_0041_2	PF11_0041	1.929053	1.218743	-0.39449	-1.83985	-1.86708
aPF11_0041_0	PF11_0041	1.929053	1.218743	-0.39449	-1.83985	-1.86708
intPF11_0042_2	PF11_0042	1.658483	0.765583	0.059843	-0.88518	-1.47992
aPF11_0042_0	PF11_0042	1.658483	0.765583	0.059843	-0.88518	-1.47992
intPF11_0043_2	PF11_0043	0.375107	0.504467	0.686067	0.276267	-0.63213
aPF11_0043_0	PF11_0043	0.375107	0.504467	0.686067	0.276267	-0.63213
aPF11_0044_2	PF11_0044	-0.90407	-0.56851	0.125272	0.635842	0.646452
aPF11_0044_1	PF11_0044	-0.90407	-0.56851	0.125272	0.635842	0.646452
aPF11_0044_0	PF11_0044	-0.90407	-0.56851	0.125272	0.635842	0.646452
intPF11_0046_2	PF11_0045	-0.17492	-0.13084	0.084593	0.221733	0.148583

intPF11_0045_2	PF11_0045	-0.59293	-0.4473	-0.02341	0.327072	0.432962
aPF11_0046_0	PF11_0045	-0.17492	-0.13084	0.084593	0.221733	0.148583
aPF11_0045_0	PF11_0045	-0.59293	-0.4473	-0.02341	0.327072	0.432962
intPF11_0047_2	PF11_0047	0.93377	0.60466	0.51593	-0.06443	-0.95066
aPF11_0047_1	PF11_0047	0.93377	0.60466	0.51593	-0.06443	-0.95066
aPF11_0047_0	PF11_0047	0.93377	0.60466	0.51593	-0.06443	-0.95066
intPF11_0048_3	PF11_0048	-0.31947	-0.39664	0.31678	0.67022	0.26664
aPF11_0048_0	PF11_0048	-0.31947	-0.39664	0.31678	0.67022	0.26664
intPF11_0049_2	PF11_0049	-0.65547	-1.19132	-0.25173	0.625712	0.824342
aPF11_0049_5	PF11_0049	-0.65547	-1.19132	-0.25173	0.625712	0.824342
aPF11_0049_4	PF11_0049	-0.65547	-1.19132	-0.25173	0.625712	0.824342
aPF11_0049_3	PF11_0049	-0.65547	-1.19132	-0.25173	0.625712	0.824342
aPF11_0049_2	PF11_0049	-0.65547	-1.19132	-0.25173	0.625712	0.824342
aPF11_0049_1	PF11_0049	-0.65547	-1.19132	-0.25173	0.625712	0.824342
aPF11_0049_0	PF11_0049	-0.65547	-1.19132	-0.25173	0.625712	0.824342
intPF11_0050_2	PF11_0050	-0.186	-0.375	-0.838	-0.70466	0.291337
aPF11_0050_0	PF11_0050	-0.186	-0.375	-0.838	-0.70466	0.291337
intPF11_0051_2	PF11_0051	0.310433	0.220343	0.431803	0.215363	-0.42518
aPF11_0051_1	PF11_0051	0.310433	0.220343	0.431803	0.215363	-0.42518
aPF11_0051_0	PF11_0051	0.310433	0.220343	0.431803	0.215363	-0.42518
intPF11_0052_3	PF11_0052	0.311627	0.032847	0.142807	-0.03847	-0.40151
aPF11_0052_0	PF11_0052	0.311627	0.032847	0.142807	-0.03847	-0.40151
intPF11_0053_2	PF11_0053	0.250127	0.318367	-0.06556	-0.19799	-0.04792
aPF11_0053_2	PF11_0053	0.250127	0.318367	-0.06556	-0.19799	-0.04792
aPF11_0053_1	PF11_0053	0.250127	0.318367	-0.06556	-0.19799	-0.04792
intPF11_0054_2	PF11_0054	-0.59294	-0.70934	-0.24683	0.491493	0.914503
aPF11_0054_1	PF11_0054	-0.59294	-0.70934	-0.24683	0.491493	0.914503
aPF11_0054_0	PF11_0054	-0.59294	-0.70934	-0.24683	0.491493	0.914503
intPF11_0055_2	PF11_0055	-0.72902	0.092137	0.222977	0.503357	0.650697
aPF11_0055_1	PF11_0055	-0.72902	0.092137	0.222977	0.503357	0.650697
aPF11_0055_0	PF11_0055	-0.72902	0.092137	0.222977	0.503357	0.650697
intPF11_0057_2	PF11_0056	-0.81227	-0.37661	-0.21577	0.227112	0.755922
intPF11_0056_2	PF11_0056					
aPF11_0056_1	PF11_0056					
aPF11_0056_0	PF11_0056					
aPF11_0057_1	PF11_0057	-0.81227	-0.37661	-0.21577	0.227112	0.755922
aPF11_0057_0	PF11_0057	-0.81227	-0.37661	-0.21577	0.227112	0.755922
aPF11_0058_0	PF11_0058-a					
intPF11_0059_2	PF11_0059	-1.24378	-0.3436	1.107453	1.180713	-0.07374
intPF11_0058_2	PF11_0059					
aPF11_0059_1	PF11_0059	-1.24378	-0.3436	1.107453	1.180713	-0.07374
aPF11_0059_0	PF11_0059	-1.24378	-0.3436	1.107453	1.180713	-0.07374
intPF11_0060_2	PF11_0060	0.723198	-0.17632	-1.85751	-1.85804	0.259768
aPF11_0060_2	PF11_0060	0.723198	-0.17632	-1.85751	-1.85804	0.259768
aPF11_0060_1	PF11_0060	0.723198	-0.17632	-1.85751	-1.85804	0.259768
aPF11_0060_0	PF11_0060	0.723198	-0.17632	-1.85751	-1.85804	0.259768
intPF11_0061_2	PF11_0061	-0.82423	-2.48787	-1.37526	0.79826	2.33487

aPF11_0061_0	PF11_0061	-0.82423	-2.48787	-1.37526	0.79826	2.33487
intPF11_0062_2	PF11_0062	-1.87958	-2.0401	-0.99779	0.929127	2.489437
aPF11_0062_0	PF11_0062	-1.87958	-2.0401	-0.99779	0.929127	2.489437
intPF11_0063_2	PF11_0063	0.083338	0.320468	0.184488	-0.11892	-0.33075
aPF11_0063_0	PF11_0063	0.083338	0.320468	0.184488	-0.11892	-0.33075
intPF11_0064_2	PF11_0064	-0.45161	-1.01993	-1.00563	-0.00487	1.30693
aPF11_0064_1	PF11_0064	-0.45161	-1.01993	-1.00563	-0.00487	1.30693
aPF11_0064_0	PF11_0064	-0.45161	-1.01993	-1.00563	-0.00487	1.30693
intPF11_0065_2	PF11_0065	0.067333	0.473353	0.799293	0.458373	-0.48469
aPF11_0065_0	PF11_0065	0.067333	0.473353	0.799293	0.458373	-0.48469
intPF11_0066_2	PF11_0066	-0.28222	-0.58489	-0.22922	0.378557	0.692227
aPF11_0066_0	PF11_0066	-0.28222	-0.58489	-0.22922	0.378557	0.692227
intPF11_0067_3	PF11_0067	0.798983	0.615563	0.611383	-0.61272	-1.8959
aPF11_0067_1	PF11_0067	0.798983	0.615563	0.611383	-0.61272	-1.8959
aPF11_0067_0	PF11_0067	0.798983	0.615563	0.611383	-0.61272	-1.8959
intPF11_0068_2	PF11_0068	0.69742	0.93291	0.29869	-0.55119	-0.98491
aPF11_0068_1	PF11_0068	0.69742	0.93291	0.29869	-0.55119	-0.98491
aPF11_0068_0	PF11_0068	0.69742	0.93291	0.29869	-0.55119	-0.98491
intPF11_0069_2	PF11_0069	-0.74684	-0.7948	0.677583	0.875083	-0.21054
aPF11_0069_0	PF11_0069	-0.74684	-0.7948	0.677583	0.875083	-0.21054
aPF11_0070_0	PF11_0070	0.586937	0.682317	0.282367	-0.22303	-0.59298
intPF11_0071_2	PF11_0071	-0.00329	0.299443	0.777373	0.371793	-0.66678
intPF11_0070_2	PF11_0071	0.586937	0.682317	0.282367	-0.22303	-0.59298
aPF11_0071_1	PF11_0071	-0.00329	0.299443	0.777373	0.371793	-0.66678
aPF11_0071_0	PF11_0071	-0.00329	0.299443	0.777373	0.371793	-0.66678
intPF11_0072_2	PF11_0072	-0.20944	-0.1938	-0.21491	0.158573	0.572463
aPF11_0072_0	PF11_0072	-0.20944	-0.1938	-0.21491	0.158573	0.572463
intPF11_0073_2	PF11_0073	-1.10806	-0.84019	0.279887	0.958947	0.734527
aPF11_0073_1	PF11_0073	-1.10806	-0.84019	0.279887	0.958947	0.734527
aPF11_0073_0	PF11_0073	-1.10806	-0.84019	0.279887	0.958947	0.734527
aPF11_0074_1	PF11_0074	-1.25544	-0.37828	0.39781	0.66819	0.43928
aPF11_0074_0	PF11_0074	-1.25544	-0.37828	0.39781	0.66819	0.43928
intPF11_0075_2	PF11_0075	-0.90192	-1.02242	0.26433	0.87467	0.44642
intPF11_0074_2	PF11_0075	-1.25544	-0.37828	0.39781	0.66819	0.43928
aPF11_0075_0	PF11_0075	-0.90192	-1.02242	0.26433	0.87467	0.44642
intPF11_0077_2	PF11_0076	-2.12516	0.77629	-0.44634	-0.28966	1.60971
aPF11_0076_2	PF11_0076	0.09524	0.49146	0.51009	0.16047	-0.39857
aPF11_0076_1	PF11_0076	0.09524	0.49146	0.51009	0.16047	-0.39857
aPF11_0076_0	PF11_0076	0.09524	0.49146	0.51009	0.16047	-0.39857
intPF11_0076_2	PF11_0077	0.09524	0.49146	0.51009	0.16047	-0.39857
aPF11_0077_1	PF11_0077	-2.12516	0.77629	-0.44634	-0.28966	1.60971
aPF11_0077_0	PF11_0077	-2.12516	0.77629	-0.44634	-0.28966	1.60971
intPF11_0079_2	PF11_0078	0.535567	0.199487	0.112687	-0.05535	-0.33515
aPF11_0078_1	PF11_0078	0.170533	0.478333	0.384583	0.010583	-0.42717
aPF11_0078_0	PF11_0078	0.170533	0.478333	0.384583	0.010583	-0.42717
intPF11_0080_2	PF11_0079	0.272032	0.321412	0.643452	0.221072	-0.72446
intPF11_0078_2	PF11_0079	0.170533	0.478333	0.384583	0.010583	-0.42717

aPF11_0079_2	PF11_0079	0.535567	0.199487	0.112687	-0.05535	-0.33515
aPF11_0079_1	PF11_0079	0.535567	0.199487	0.112687	-0.05535	-0.33515
aPF11_0079_0	PF11_0079	0.535567	0.199487	0.112687	-0.05535	-0.33515
aPF11_0080_0	PF11_0080	0.272032	0.321412	0.643452	0.221072	-0.72446
intPF11_0084_2	PF11_0083	-0.00746	-0.21168	0.38286	0.53614	-0.03632
intPF11_0083_2	PF11_0083	-0.66819	-1.15253	-1.25869	0.01169	1.77953
aPF11_0083_1	PF11_0083	-0.66819	-1.15253	-1.25869	0.01169	1.77953
aPF11_0083_0	PF11_0083	-0.66819	-1.15253	-1.25869	0.01169	1.77953
intPF11_0085_2	PF11_0084	-0.38111	-0.21346	0.441413	0.606753	0.085623
aPF11_0085_1	PF11_0084	-0.38111	-0.21346	0.441413	0.606753	0.085623
aPF11_0085_0	PF11_0084	-0.38111	-0.21346	0.441413	0.606753	0.085623
aPF11_0084_0	PF11_0084	-0.00746	-0.21168	0.38286	0.53614	-0.03632
intPF11_0086_2	PF11_0086	0.423873	0.076813	-0.25783	-0.37571	-0.18095
aPF11_0086_4	PF11_0086	0.423873	0.076813	-0.25783	-0.37571	-0.18095
aPF11_0086_3	PF11_0086	0.423873	0.076813	-0.25783	-0.37571	-0.18095
aPF11_0086_2	PF11_0086	0.423873	0.076813	-0.25783	-0.37571	-0.18095
aPF11_0086_1	PF11_0086	0.423873	0.076813	-0.25783	-0.37571	-0.18095
aPF11_0086_0	PF11_0086	0.423873	0.076813	-0.25783	-0.37571	-0.18095
intPF11_0087_2	PF11_0087	0.10898	-0.54039	0.07628	0.50322	0.21839
aPF11_0087_1	PF11_0087	0.10898	-0.54039	0.07628	0.50322	0.21839
aPF11_0087_0	PF11_0087	0.10898	-0.54039	0.07628	0.50322	0.21839
intPF11_0088_2	PF11_0088	0.511617	0.872927	0.643647	0.004687	-0.76159
aPF11_0088_0	PF11_0088	0.511617	0.872927	0.643647	0.004687	-0.76159
intPF11_0090_2	PF11_0089	0.36444	0.49678	0.70544	-0.05544	-1.16678
intPF11_0089_2	PF11_0089	0.524282	0.069562	0.038982	-0.14271	-0.4139
aPF11_0089_1	PF11_0089	0.524282	0.069562	0.038982	-0.14271	-0.4139
aPF11_0089_0	PF11_0089	0.524282	0.069562	0.038982	-0.14271	-0.4139
aPF11_0090_1	PF11_0090	0.36444	0.49678	0.70544	-0.05544	-1.16678
aPF11_0090_0	PF11_0090	0.36444	0.49678	0.70544	-0.05544	-1.16678
intPF11_0091_2	PF11_0091	-1.33452	-0.64674	0.2779	0.1526	-0.26692
aPF11_0091_2	PF11_0091	-1.33452	-0.64674	0.2779	0.1526	-0.26692
aPF11_0091_1	PF11_0091	-1.33452	-0.64674	0.2779	0.1526	-0.26692
aPF11_0091_0	PF11_0091	-1.33452	-0.64674	0.2779	0.1526	-0.26692
intPF11_0092_2	PF11_0092	-0.17861	-0.28898	-0.63888	-0.31413	0.566975
aPF11_0092_2	PF11_0092	-0.17861	-0.28898	-0.63888	-0.31413	0.566975
aPF11_0092_1	PF11_0092	-0.17861	-0.28898	-0.63888	-0.31413	0.566975
aPF11_0092_0	PF11_0092	-0.17861	-0.28898	-0.63888	-0.31413	0.566975
intPF11_0093_3	PF11_0093	0.298693	0.594033	0.284423	-0.28626	-0.65437
aPF11_0093_1	PF11_0093	0.298693	0.594033	0.284423	-0.28626	-0.65437
aPF11_0093_0	PF11_0093	0.298693	0.594033	0.284423	-0.28626	-0.65437
intPF11_0094_2	PF11_0094	0.550083	0.314563	-1.16513	-1.53221	-0.1359
aPF11_0094_1	PF11_0094	0.550083	0.314563	-1.16513	-1.53221	-0.1359
aPF11_0094_0	PF11_0094	0.550083	0.314563	-1.16513	-1.53221	-0.1359
intPF11_0095_2	PF11_0095	0.777663	0.276683	0.244123	-0.28646	-0.95202
aPF11_0095_0	PF11_0095	0.777663	0.276683	0.244123	-0.28646	-0.95202
intPF11_0096_3	PF11_0096	-0.2614	-0.30397	0.236457	0.560877	0.292307
aPF11_0096_0	PF11_0096	-0.2614	-0.30397	0.236457	0.560877	0.292307

intPF11_0097_2	PF11_0097	-0.85895	-1.34677	-0.65296	0.65046	1.56127
aPF11_0097_1	PF11_0097	-0.85895	-1.34677	-0.65296	0.65046	1.56127
aPF11_0097_0	PF11_0097	-0.85895	-1.34677	-0.65296	0.65046	1.56127
intPF11_0098_2	PF11_0098	-1.54313	-0.59034	-0.23531	0.667647	1.553677
aPF11_0098_0	PF11_0098	-1.54313	-0.59034	-0.23531	0.667647	1.553677
intPF11_0099_3	PF11_0099	-0.92141	0.056087	0.106587	0.378747	0.717247
aPF11_0099_1	PF11_0099	-0.92141	0.056087	0.106587	0.378747	0.717247
aPF11_0099_0	PF11_0099	-0.92141	0.056087	0.106587	0.378747	0.717247
intPF11_0101_2	PF11_0100	0.575563	1.264683	1.066883	-0.02622	-1.34402
intPF11_0100_2	PF11_0100	0.258437	0.367837	0.052827	-0.02049	0.031497
aPF11_0100_0	PF11_0100	0.258437	0.367837	0.052827	-0.02049	0.031497
aPF11_0101_1	PF11_0101	0.575563	1.264683	1.066883	-0.02622	-1.34402
aPF11_0101_0	PF11_0101	0.575563	1.264683	1.066883	-0.02622	-1.34402
intPF11_0105_1	PF11_0105	0.46642	0.50389	0.71448	-0.00998	-1.14439
aPF11_0105_1	PF11_0105	0.46642	0.50389	0.71448	-0.00998	-1.14439
aPF11_0105_0	PF11_0105	0.46642	0.50389	0.71448	-0.00998	-1.14439
aPF11_0106_0	PF11_0106	-0.07105	0.21824	0.81402	0.48998	-0.58824
intPF11_0107_2	PF11_0107	-0.46261	0.554547	-0.47284	-0.73532	0.287787
aPF11_0107_1	PF11_0107	-0.46261	0.554547	-0.47284	-0.73532	0.287787
aPF11_0107_0	PF11_0107	-0.46261	0.554547	-0.47284	-0.73532	0.287787
intPF11_0108_2	PF11_0108	0.55599	0.36154	0.03742	-0.34842	-0.53554
aPF11_0108_1	PF11_0108	0.55599	0.36154	0.03742	-0.34842	-0.53554
aPF11_0108_0	PF11_0108	0.55599	0.36154	0.03742	-0.34842	-0.53554
intPF11_0109_2	PF11_0109	0.777547	-0.13871	0.485937	0.096397	-1.09795
aPF11_0110_0	PF11_0109	0.501183	0.017083	0.549823	0.098843	-1.01542
aPF11_0109_0	PF11_0109	0.777547	-0.13871	0.485937	0.096397	-1.09795
intPF11_0111_2	PF11_0111	0.493113	0.432923	0.077903	-0.2779	-0.43543
aPF11_0111_2	PF11_0111	0.493113	0.432923	0.077903	-0.2779	-0.43543
aPF11_0111_1	PF11_0111	0.493113	0.432923	0.077903	-0.2779	-0.43543
aPF11_0111_0	PF11_0111	0.493113	0.432923	0.077903	-0.2779	-0.43543
intPF11_0112_2	PF11_0112	-0.70687	-0.23613	-0.01373	0.347067	0.623967
aPF11_0112_1	PF11_0112	-0.70687	-0.23613	-0.01373	0.347067	0.623967
aPF11_0112_0	PF11_0112	-0.70687	-0.23613	-0.01373	0.347067	0.623967
aPF11_0113_0	PF11_0113	-0.52374	-0.95631	-0.42488	0.45088	0.99231
intPF11_0114_2	PF11_0114	-0.29451	-0.75742	-0.67066	0.035997	0.876757
intPF11_0113_2	PF11_0114	-0.52374	-0.95631	-0.42488	0.45088	0.99231
aPF11_0114_1	PF11_0114	-0.29451	-0.75742	-0.67066	0.035997	0.876757
aPF11_0114_0	PF11_0114	-0.29451	-0.75742	-0.67066	0.035997	0.876757
intPF11_0116_2	PF11_0115	0.994193	0.847523	0.625943	-0.30828	-1.37636
intPF11_0115_3	PF11_0115	0.60657	0.44466	0.04167	-0.29567	-0.42566
aPF11_0115_1	PF11_0115	0.60657	0.44466	0.04167	-0.29567	-0.42566
aPF11_0115_0	PF11_0115	0.60657	0.44466	0.04167	-0.29567	-0.42566
aPF11_0116_1	PF11_0116	0.994193	0.847523	0.625943	-0.30828	-1.37636
aPF11_0116_0	PF11_0116	0.994193	0.847523	0.625943	-0.30828	-1.37636
intPF11_0117_2	PF11_0117	-1.43466	-1.5487	0.313417	1.501917	1.124037
aPF11_0117_0	PF11_0117	-1.43466	-1.5487	0.313417	1.501917	1.124037
intPF11_0118_2	PF11_0118	0.640813	0.689933	0.575133	-0.01647	-0.83277

aPF11_0118_1	PF11_0118	0.640813	0.689933	0.575133	-0.01647	-0.83277
aPF11_0118_0	PF11_0118	0.640813	0.689933	0.575133	-0.01647	-0.83277
intPF11_0119_3	PF11_0119	0.132803	0.638993	0.222013	-0.06135	-0.11733
aPF11_0119_0	PF11_0119	0.132803	0.638993	0.222013	-0.06135	-0.11733
aPF11_0120_0	PF11_0120	-0.55898	-0.58408	0.29516	0.81084	0.47208
intPF11_0120_2	PF11_0121	-0.55898	-0.58408	0.29516	0.81084	0.47208
intPF11_0122_2	PF11_0122	-0.31629	-0.74553	-0.40744	0.41744	0.98153
aPF11_0122_1	PF11_0122	-0.31629	-0.74553	-0.40744	0.41744	0.98153
aPF11_0122_0	PF11_0122	-0.31629	-0.74553	-0.40744	0.41744	0.98153
intPF11_0124_2	PF11_0123	-0.08907	-0.2985	-0.04938	0.13331	0.12119
aPF11_0123_1	PF11_0123	0.581153	0.833733	0.274023	-0.29286	-0.57957
aPF11_0123_0	PF11_0123	0.581153	0.833733	0.274023	-0.29286	-0.57957
intPF11_0123_2	PF11_0124	0.581153	0.833733	0.274023	-0.29286	-0.57957
aPF11_0124_0	PF11_0124	-0.08907	-0.2985	-0.04938	0.13331	0.12119
intPF11_0126_2	PF11_0125	-0.02878	-0.21513	0.031763	0.031903	-0.14421
intPF11_0125_2	PF11_0125	-1.0523	-1.12072	0.17034	1.04492	0.85872
aPF11_0125_0	PF11_0125	-1.0523	-1.12072	0.17034	1.04492	0.85872
aPF11_0126_0	PF11_0126	-0.02878	-0.21513	0.031763	0.031903	-0.14421
intPF11_0128_2	PF11_0127					
intPF11_0127_3	PF11_0127	-0.42704	-1.05031	-0.98799	-0.08162	1.131038
aPF11_0127_3	PF11_0127	-0.42704	-1.05031	-0.98799	-0.08162	1.131038
aPF11_0127_2	PF11_0127	-0.42704	-1.05031	-0.98799	-0.08162	1.131038
aPF11_0127_1	PF11_0127	-0.42704	-1.05031	-0.98799	-0.08162	1.131038
aPF11_0127_0	PF11_0127	-0.42704	-1.05031	-0.98799	-0.08162	1.131038
aPF11_0128_0	PF11_0128					
intPF11_0130_2	PF11_0129	-0.70709	-1.26952	0.229047	1.112287	0.637857
aPF11_0129_1	PF11_0129	-0.79148	-0.93264	0.527773	0.965893	0.123303
aPF11_0129_0	PF11_0129	-0.79148	-0.93264	0.527773	0.965893	0.123303
intPF11_0129_2	PF11_0130	-0.79148	-0.93264	0.527773	0.965893	0.123303
aPF11_0130_0	PF11_0130	-0.70709	-1.26952	0.229047	1.112287	0.637857
intPF11_0527_2	PF11_0131	0.472963	-0.05684	-0.06257	-0.06277	-0.175
intPF11_0131_2	PF11_0131	-0.70948	-0.92214	-0.07248	0.684143	0.780803
aPF11_0131_1	PF11_0131	-0.70948	-0.92214	-0.07248	0.684143	0.780803
aPF11_0131_0	PF11_0131	-0.70948	-0.92214	-0.07248	0.684143	0.780803
aPF11_0134_1	PF11_0134	0.236583	0.747083	0.320583	-0.21292	-0.48792
aPF11_0134_0	PF11_0134	0.236583	0.747083	0.320583	-0.21292	-0.48792
intPF11_0136_2	PF11_0135	-0.12952	-0.37742	-1.82816	-1.11984	1.46342
intPF11_0135_2	PF11_0135	0.946332	1.943732	0.485822	-1.17443	-1.67193
aPF11_0136_0	PF11_0135	-0.12952	-0.37742	-1.82816	-1.11984	1.46342
aPF11_0135_0	PF11_0135	0.946332	1.943732	0.485822	-1.17443	-1.67193
intPF11_0139_2	PF11_0139	-0.03218	-0.09408	-0.12282	-0.25784	-0.21358
aPF11_0139_0	PF11_0139	-0.03218	-0.09408	-0.12282	-0.25784	-0.21358
intPF11_0140_3	PF11_0140	0.245977	-0.1779	0.108797	-0.00246	-0.40376
aPF11_0140_0	PF11_0140	0.245977	-0.1779	0.108797	-0.00246	-0.40376
intPF11_0141_2	PF11_0141	-1.37726	-1.52273	-0.67454	0.917203	2.056393
aPF11_0141_0	PF11_0141	-1.37726	-1.52273	-0.67454	0.917203	2.056393
aPF11_0142_1	PF11_0142	0.095447	-0.27624	0.075737	0.220597	-0.02392

aPF11_0142_0	PF11_0142	0.095447	-0.27624	0.075737	0.220597	-0.02392
intPF11_0143_2	PF11_0143	0.250593	0.488023	0.281453	-0.23979	-0.63386
intPF11_0142_2	PF11_0143	0.095447	-0.27624	0.075737	0.220597	-0.02392
aPF11_0143_1	PF11_0143	0.250593	0.488023	0.281453	-0.23979	-0.63386
aPF11_0143_0	PF11_0143	0.250593	0.488023	0.281453	-0.23979	-0.63386
intPF11_0145_2	PF11_0144	0.535083	0.541563	0.442373	0.146293	-0.4089
intPF11_0144_2	PF11_0144	0.51477	1.08017	0.23166	-0.59566	-0.76967
aPF11_0144_1	PF11_0144	0.51477	1.08017	0.23166	-0.59566	-0.76967
aPF11_0144_0	PF11_0144	0.51477	1.08017	0.23166	-0.59566	-0.76967
aPF11_0145_0	PF11_0145	0.535083	0.541563	0.442373	0.146293	-0.4089
intPF11_0146_2	PF11_0146	-0.82505	-0.21325	-0.46925	0.06075	1.08425
aPF11_0146_1	PF11_0146	-0.82505	-0.21325	-0.46925	0.06075	1.08425
aPF11_0146_0	PF11_0146	-0.82505	-0.21325	-0.46925	0.06075	1.08425
intPF11_0148_2	PF11_0147	-0.41465	-1.04273	0.170843	0.863423	0.424993
aPF11_0147_1	PF11_0147	0.9227	0.3155	-0.21075	-0.48475	-0.438
aPF11_0147_0	PF11_0147	0.9227	0.3155	-0.21075	-0.48475	-0.438
intPF11_0147_2	PF11_0148	0.9227	0.3155	-0.21075	-0.48475	-0.438
aPF11_0148_0	PF11_0148	-0.41465	-1.04273	0.170843	0.863423	0.424993
aPF11_0150_0	PF11_0149-a	0.167573	-0.35688	-0.23425	-0.26009	-0.25446
aPF11_0149_0	PF11_0149-a	0.255387	-0.34794	-0.21311	-0.25155	-0.30372
intPF11_0149_2	PF11_0149-b	0.255387	-0.34794	-0.21311	-0.25155	-0.30372
intPF11_0150_2	PF11_0150	0.167573	-0.35688	-0.23425	-0.26009	-0.25446
intPF11_0152_2	PF11_0152					
aPF11_0152_0	PF11_0152					
aPF11_0151_0	PF11_0152	1.363483	0.616563	0.191383	-0.99772	-1.8909
intPF11_0154_2	PF11_0153	-0.5792	-0.45353	0.248553	0.547113	0.255693
aPF11_0153_1	PF11_0153	-1.22192	-0.69438	0.7065	1.0545	0.23088
aPF11_0153_0	PF11_0153	-1.22192	-0.69438	0.7065	1.0545	0.23088
intPF11_0153_2	PF11_0154	-1.22192	-0.69438	0.7065	1.0545	0.23088
aPF11_0154_1	PF11_0154	-0.5792	-0.45353	0.248553	0.547113	0.255693
aPF11_0154_0	PF11_0154	-0.5792	-0.45353	0.248553	0.547113	0.255693
intPF11_0155_2	PF11_0155	-0.16608	-0.33356	-0.56187	-0.02479	0.825897
aPF11_0155_0	PF11_0155	-0.16608	-0.33356	-0.56187	-0.02479	0.825897
intPF11_0156_2	PF11_0156	0.136977	0.040137	-0.11852	-0.10414	0.042197
aPF11_0156_1	PF11_0156	0.136977	0.040137	-0.11852	-0.10414	0.042197
aPF11_0156_0	PF11_0156	0.136977	0.040137	-0.11852	-0.10414	0.042197
intPF11_0158_2	PF11_0157	-0.10016	-0.31829	-2.028	-1.13845	1.831512
aPF11_0157_1	PF11_0157	0.214843	0.137273	-0.56655	-0.52979	0.263893
aPF11_0157_0	PF11_0157	0.214843	0.137273	-0.56655	-0.52979	0.263893
intPF11_0159_2	PF11_0158	-0.84344	-1.21477	0.274557	1.084777	0.595107
intPF11_0157_2	PF11_0158	0.214843	0.137273	-0.56655	-0.52979	0.263893
aPF11_0158_3	PF11_0158	-0.10016	-0.31829	-2.028	-1.13845	1.831512
aPF11_0158_2	PF11_0158	-0.10016	-0.31829	-2.028	-1.13845	1.831512
aPF11_0158_1	PF11_0158	-0.10016	-0.31829	-2.028	-1.13845	1.831512
aPF11_0158_0	PF11_0158	-0.10016	-0.31829	-2.028	-1.13845	1.831512
aPF11_0159_0	PF11_0159	-0.84344	-1.21477	0.274557	1.084777	0.595107
intPF11_0160_2	PF11_0160	-0.07445	0.46873	-0.43671	-0.55179	0.31027

aPF11_0160_1	PF11_0160	-0.07445	0.46873	-0.43671	-0.55179	0.31027
aPF11_0160_0	PF11_0160	-0.07445	0.46873	-0.43671	-0.55179	0.31027
intPF11_0161_2	PF11_0161	0.70574	0.73131	1.08288	0.07512	-1.62031
aPF11_0161_0	PF11_0161	0.70574	0.73131	1.08288	0.07512	-1.62031
intPF11_0162_3	PF11_0162	-0.08685	-0.14525	-0.31952	-0.01532	0.494913
aPF11_0162_1	PF11_0162	-0.08685	-0.14525	-0.31952	-0.01532	0.494913
aPF11_0162_0	PF11_0162	-0.08685	-0.14525	-0.31952	-0.01532	0.494913
intPF11_0163_2	PF11_0163	0.551357	-0.7538	-0.38939	0.189727	0.312137
aPF11_0163_0	PF11_0163	0.551357	-0.7538	-0.38939	0.189727	0.312137
intPF11_0164_2	PF11_0164	-0.51021	-0.31095	-0.0686	0.4416	0.75095
aPF11_0164_0	PF11_0164	-0.51021	-0.31095	-0.0686	0.4416	0.75095
intPF11_0165_2	PF11_0165	-0.72814	-0.57226	1.794037	1.404297	-1.3214
aPF11_0165_0	PF11_0165	-0.72814	-0.57226	1.794037	1.404297	-1.3214
intPF11_0166_2	PF11_0166	2.24416	0.84721	-0.89791	-2.19809	-1.83421
aPF11_0166_0	PF11_0166	2.24416	0.84721	-0.89791	-2.19809	-1.83421
intPF11_0167_3	PF11_0167	1.49213	0.22237	-1.33673	-1.54727	-0.21937
aPF11_0167_0	PF11_0167	1.49213	0.22237	-1.33673	-1.54727	-0.21937
aPF11_0168_4	PF11_0168	-0.23668	-1.10522	-1.75887	-0.68587	1.537883
aPF11_0168_3	PF11_0168	-0.23668	-1.10522	-1.75887	-0.68587	1.537883
intPF11_0168_2	PF11_0168a	-0.23668	-1.10522	-1.75887	-0.68587	1.537883
aPF11_0168_2	PF11_0168a	-0.23668	-1.10522	-1.75887	-0.68587	1.537883
aPF11_0168_1	PF11_0168a	-0.23668	-1.10522	-1.75887	-0.68587	1.537883
aPF11_0168_0	PF11_0168a	-0.23668	-1.10522	-1.75887	-0.68587	1.537883
intPF11_0170_2	PF11_0169	0.399077	0.569627	0.369757	-0.15142	-0.65829
intPF11_0169_3	PF11_0169	0.539227	0.976887	0.601227	-0.36789	-1.18255
aPF11_0169_0	PF11_0169	0.539227	0.976887	0.601227	-0.36789	-1.18255
aPF11_0170_0	PF11_0170	0.399077	0.569627	0.369757	-0.15142	-0.65829
intPF11_0171_2	PF11_0171	0.105367	0.115187	0.075377	-0.06654	-0.19635
aPF11_0171_1	PF11_0171	0.105367	0.115187	0.075377	-0.06654	-0.19635
aPF11_0171_0	PF11_0171	0.105367	0.115187	0.075377	-0.06654	-0.19635
intPF11_0172_2	PF11_0172	-1.12182	-1.34337	-0.1735	0.737837	0.903207
aPF11_0172_1	PF11_0172	-1.12182	-1.34337	-0.1735	0.737837	0.903207
aPF11_0172_0	PF11_0172	-1.12182	-1.34337	-0.1735	0.737837	0.903207
intPF11_0173_2	PF11_0173	-0.51081	-0.67695	0.1149	0.6551	0.49095
aPF11_0173_1	PF11_0173	-0.51081	-0.67695	0.1149	0.6551	0.49095
aPF11_0173_0	PF11_0173	-0.51081	-0.67695	0.1149	0.6551	0.49095
intPF11_0174_2	PF11_0174	-0.1183	0.07199	0.98352	0.37248	-1.15699
aPF11_0174_1	PF11_0174	-0.1183	0.07199	0.98352	0.37248	-1.15699
aPF11_0174_0	PF11_0174	-0.1183	0.07199	0.98352	0.37248	-1.15699
intPF11_0175_2	PF11_0175	1.14805	1.10674	0.51252	-1.17752	-2.35074
aPF11_0175_1	PF11_0175	1.14805	1.10674	0.51252	-1.17752	-2.35074
aPF11_0175_0	PF11_0175	1.14805	1.10674	0.51252	-1.17752	-2.35074
intPF11_0177_2	PF11_0176	-0.75985	-1.72823	-0.47728	0.983617	1.451567
intPF11_0176_2	PF11_0176	-1.19218	-0.9256	-0.09556	0.68906	0.9891
aPF11_0176_1	PF11_0176	-1.19218	-0.9256	-0.09556	0.68906	0.9891
aPF11_0176_0	PF11_0176	-1.19218	-0.9256	-0.09556	0.68906	0.9891
intPF11_0178_2	PF11_0177	-0.73553	-0.54672	-0.46707	0.185005	1.014495

aPF11_0177_1	PF11_0177	-0.75985	-1.72823	-0.47728	0.983617	1.451567
aPF11_0177_0	PF11_0177	-0.75985	-1.72823	-0.47728	0.983617	1.451567
aPF11_0178_2	PF11_0178	-0.73553	-0.54672	-0.46707	0.185005	1.014495
aPF11_0178_1	PF11_0178	-0.73553	-0.54672	-0.46707	0.185005	1.014495
aPF11_0178_0	PF11_0178	-0.73553	-0.54672	-0.46707	0.185005	1.014495
aPF11_0179_0	PF11_0179	0.638383	0.113093	-0.75719	-0.67415	0.239573
intPF11_0180_2	PF11_0180	0.636833	-0.05815	-1.31846	-1.12888	0.472813
aPF11_0180_1	PF11_0180	0.636833	-0.05815	-1.31846	-1.12888	0.472813
aPF11_0180_0	PF11_0180	0.636833	-0.05815	-1.31846	-1.12888	0.472813
intPF11_0182_2	PF11_0181	-0.57592	-0.50544	0.560373	0.860293	0.118103
aPF11_0181_1	PF11_0181	-0.55977	-0.37918	0.466113	0.630053	0.024343
aPF11_0181_0	PF11_0181	-0.55977	-0.37918	0.466113	0.630053	0.024343
intPF11_0181_2	PF11_0182	-0.55977	-0.37918	0.466113	0.630053	0.024343
aPF11_0182_0	PF11_0182	-0.57592	-0.50544	0.560373	0.860293	0.118103
intPF11_0183_3	PF11_0183	-0.5973	-0.20349	0.072487	0.474847	0.642827
aPF11_0183_0	PF11_0183	-0.5973	-0.20349	0.072487	0.474847	0.642827
aPF11_0184_1	PF11_0184	0.369587	0.688557	-0.16535	-0.52781	-0.15272
aPF11_0184_0	PF11_0184	0.369587	0.688557	-0.16535	-0.52781	-0.15272
intPF11_0185_2	PF11_0185	-0.20961	0.11038	-0.7536	-0.4994	0.77595
intPF11_0184_2	PF11_0185	0.369587	0.688557	-0.16535	-0.52781	-0.15272
aPF11_0185_2	PF11_0185	-0.20961	0.11038	-0.7536	-0.4994	0.77595
aPF11_0185_1	PF11_0185	-0.20961	0.11038	-0.7536	-0.4994	0.77595
aPF11_0185_0	PF11_0185	-0.20961	0.11038	-0.7536	-0.4994	0.77595
intPF11_0186_2	PF11_0186	0.380387	0.681077	0.127347	-0.21901	-0.23474
aPF11_0186_0	PF11_0186	0.380387	0.681077	0.127347	-0.21901	-0.23474
intPF11_0187_2	PF11_0187	-0.41535	0.01977	0.17097	0.32403	0.32423
aPF11_0187_0	PF11_0187	-0.41535	0.01977	0.17097	0.32403	0.32423
intPF11_0188_2	PF11_0188	-0.99669	-0.14857	1.446133	1.195533	-0.62477
aPF11_0188_1	PF11_0188	-0.99669	-0.14857	1.446133	1.195533	-0.62477
aPF11_0188_0	PF11_0188	-0.99669	-0.14857	1.446133	1.195533	-0.62477
intPF11_0189_2	PF11_0189	0.464277	0.887287	-0.06064	-0.58641	-0.33167
aPF11_0189_2	PF11_0189	0.464277	0.887287	-0.06064	-0.58641	-0.33167
aPF11_0189_1	PF11_0189	0.464277	0.887287	-0.06064	-0.58641	-0.33167
aPF11_0189_0	PF11_0189	0.464277	0.887287	-0.06064	-0.58641	-0.33167
intPF11_0190_2	PF11_0190	-0.15958	0.085893	-0.9463	-0.71104	0.778773
aPF11_0190_0	PF11_0190	-0.15958	0.085893	-0.9463	-0.71104	0.778773
intPF11_0191_2	PF11_0191	0.404813	0.457423	0.470403	-0.13224	-0.89076
aPF11_0191_1	PF11_0191	0.404813	0.457423	0.470403	-0.13224	-0.89076
aPF11_0191_0	PF11_0191	0.404813	0.457423	0.470403	-0.13224	-0.89076
intPF11_0192_2	PF11_0192	0.8657	0.19848	0.25529	-0.25579	-1.00798
aPF11_0192_1	PF11_0192	0.8657	0.19848	0.25529	-0.25579	-1.00798
aPF11_0192_0	PF11_0192	0.8657	0.19848	0.25529	-0.25579	-1.00798
intPF11_0193_2	PF11_0193	0.0931	-0.3605	-1.93301	-1.23799	1.4155
aPF11_0194_0	PF11_0193	0.185523	-0.2396	-2.3278	-1.67454	1.534263
aPF11_0193_0	PF11_0193	0.0931	-0.3605	-1.93301	-1.23799	1.4155
intPF11_0196_2	PF11_0195	0.19924	-0.03021	-0.15458	-0.27242	-0.21179
aPF11_0195_1	PF11_0195	0.57231	0.38547	-0.01344	-0.17956	-0.17847

aPF11_0195_0	PF11_0195	0.57231	0.38547	-0.01344	-0.17956	-0.17847
intPF11_0195_2	PF11_0196	0.57231	0.38547	-0.01344	-0.17956	-0.17847
aPF11_0196_0	PF11_0196	0.19924	-0.03021	-0.15458	-0.27242	-0.21179
intPF11_0198_2	PF11_0197	-0.06846	0.40328	0.88844	0.45656	-0.65228
intPF11_0197_3	PF11_0197	-0.96366	-0.1437	0.211143	0.493023	0.577863
aPF11_0197_1	PF11_0197	-0.96366	-0.1437	0.211143	0.493023	0.577863
aPF11_0197_0	PF11_0197	-0.96366	-0.1437	0.211143	0.493023	0.577863
aPF11_0198_0	PF11_0198	-0.06846	0.40328	0.88844	0.45656	-0.65228
intPF11_0199_3	PF11_0199	0.969923	1.768393	0.645703	-0.81904	-1.55673
aPF11_0199_0	PF11_0199	0.969923	1.768393	0.645703	-0.81904	-1.55673
intPF11_0200_2	PF11_0200	0.489643	-0.07422	0.064933	0.011733	-0.31412
aPF11_0200_0	PF11_0200	0.489643	-0.07422	0.064933	0.011733	-0.31412
intPF11_0201_2	PF11_0201	0.407972	0.180482	-0.27914	-0.25789	0.109242
aPF11_0201_3	PF11_0201	0.407972	0.180482	-0.27914	-0.25789	0.109242
aPF11_0201_2	PF11_0201	0.407972	0.180482	-0.27914	-0.25789	0.109242
aPF11_0201_1	PF11_0201	0.407972	0.180482	-0.27914	-0.25789	0.109242
aPF11_0201_0	PF11_0201	0.407972	0.180482	-0.27914	-0.25789	0.109242
intPF11_0202_2	PF11_0202	-0.34184	0.091663	0.635413	0.457253	-0.304
aPF11_0202_1	PF11_0202	-0.34184	0.091663	0.635413	0.457253	-0.304
aPF11_0202_0	PF11_0202	-0.34184	0.091663	0.635413	0.457253	-0.304
intPF11_0203_3	PF11_0203	0.338407	0.607957	0.488587	0.024747	-0.56612
aPF11_0203_1	PF11_0203	0.338407	0.607957	0.488587	0.024747	-0.56612
aPF11_0203_0	PF11_0203	0.338407	0.607957	0.488587	0.024747	-0.56612
intPF11_0204_2	PF11_0204	0.792427	-0.29014	-0.96271	-0.88595	-0.02152
aPF11_0204_0	PF11_0204	0.792427	-0.29014	-0.96271	-0.88595	-0.02152
intPF11_0205_3	PF11_0205	0.500843	0.226793	0.225413	-0.09075	-0.56513
aPF11_0205_0	PF11_0205	0.500843	0.226793	0.225413	-0.09075	-0.56513
intPF11_0206_3	PF11_0206	0.753975	0.569775	-0.31323	-0.63489	-0.24956
aPF11_0206_2	PF11_0206	0.753975	0.569775	-0.31323	-0.63489	-0.24956
aPF11_0206_1	PF11_0206	0.753975	0.569775	-0.31323	-0.63489	-0.24956
aPF11_0206_0	PF11_0206	0.753975	0.569775	-0.31323	-0.63489	-0.24956
intPF11_0207_2	PF11_0207	0.685113	0.486413	0.185673	-0.30051	-0.69525
aPF11_0207_1	PF11_0207	0.685113	0.486413	0.185673	-0.30051	-0.69525
aPF11_0207_0	PF11_0207	0.685113	0.486413	0.185673	-0.30051	-0.69525
intPF11_0208_2	PF11_0208	-0.47652	0.847617	0.871267	0.414067	-0.25228
aPF11_0208_0	PF11_0208	-0.47652	0.847617	0.871267	0.414067	-0.25228
intPF11_0209_2	PF11_0209	-0.7976	-0.45496	0.016427	0.395907	0.526297
aPF11_0209_0	PF11_0209	-0.7976	-0.45496	0.016427	0.395907	0.526297
intPF11_0210_2	PF11_0210	-0.34724	-0.45028	-0.10544	0.22194	0.34178
aPF11_0210_1	PF11_0210	-0.34724	-0.45028	-0.10544	0.22194	0.34178
aPF11_0210_0	PF11_0210	-0.34724	-0.45028	-0.10544	0.22194	0.34178
intPF11_0211_2	PF11_0211	0.727873	0.688123	0.381253	-0.50209	-1.23596
aPF11_0211_1	PF11_0211	0.727873	0.688123	0.381253	-0.50209	-1.23596
aPF11_0211_0	PF11_0211	0.727873	0.688123	0.381253	-0.50209	-1.23596
intPF11_0212_2	PF11_0212	0.340547	0.323287	0.299687	-0.05035	-0.52295
aPF11_0212_1	PF11_0212	0.340547	0.323287	0.299687	-0.05035	-0.52295
aPF11_0212_0	PF11_0212	0.340547	0.323287	0.299687	-0.05035	-0.52295

intPF11_0213_2	PF11_0213	-1.03878	-0.86834	0.387062	1.041272	0.619672
aPF11_0213_3	PF11_0213	-1.03878	-0.86834	0.387062	1.041272	0.619672
aPF11_0213_2	PF11_0213	-1.03878	-0.86834	0.387062	1.041272	0.619672
aPF11_0213_1	PF11_0213	-1.03878	-0.86834	0.387062	1.041272	0.619672
aPF11_0213_0	PF11_0213	-1.03878	-0.86834	0.387062	1.041272	0.619672
intPF11_0215_2	PF11_0214	-1.27147	-1.36864	0.526777	1.455557	0.728977
intPF11_0214_2	PF11_0214	0.269407	0.205947	-0.05014	-0.10802	-0.02261
aPF11_0214_1	PF11_0214	0.269407	0.205947	-0.05014	-0.10802	-0.02261
aPF11_0214_0	PF11_0214	0.269407	0.205947	-0.05014	-0.10802	-0.02261
intPF11_0216_3	PF11_0215	-1.01655	-1.03525	0.494483	1.179183	0.510913
aPF11_0215_0	PF11_0215	-1.27147	-1.36864	0.526777	1.455557	0.728977
aPF11_0216_0	PF11_0216	-1.01655	-1.03525	0.494483	1.179183	0.510913
intPF11_0217_2	PF11_0217	0.09821	-0.72507	-0.75936	0.00136	0.87807
aPF11_0217_0	PF11_0217	0.09821	-0.72507	-0.75936	0.00136	0.87807
intPF11_0218_2	PF11_0218	0.713053	0.167043	-1.05276	-0.85836	0.516183
aPF11_0218_2	PF11_0218	0.713053	0.167043	-1.05276	-0.85836	0.516183
aPF11_0218_1	PF11_0218	0.713053	0.167043	-1.05276	-0.85836	0.516183
aPF11_0218_0	PF11_0218	0.713053	0.167043	-1.05276	-0.85836	0.516183
intPF11_0219_2	PF11_0219	-0.9942	-0.735	-0.08429	0.654468	0.977648
aPF11_0219_0	PF11_0219	-0.9942	-0.735	-0.08429	0.654468	0.977648
intPF11_0220_2	PF11_0220	-0.51154	-0.71182	-1.45136	-0.29664	1.91182
aPF11_0220_2	PF11_0220	-0.51154	-0.71182	-1.45136	-0.29664	1.91182
aPF11_0220_1	PF11_0220	-0.51154	-0.71182	-1.45136	-0.29664	1.91182
aPF11_0220_0	PF11_0220	-0.51154	-0.71182	-1.45136	-0.29664	1.91182
intPF11_0222_2	PF11_0222					
intPF11_0221_2	PF11_0222	-0.27007	-0.20682	-0.48564	-0.33661	0.337558
aPF11_0222_1	PF11_0222					
aPF11_0222_0	PF11_0222					
aPF11_0221_0	PF11_0222	-0.27007	-0.20682	-0.48564	-0.33661	0.337558
intPF11_0223_2	PF11_0223	0.250987	-0.12041	-0.18343	0.065267	0.270247
aPF11_0223_1	PF11_0223	0.250987	-0.12041	-0.18343	0.065267	0.270247
aPF11_0223_0	PF11_0223	0.250987	-0.12041	-0.18343	0.065267	0.270247
intPF11_0224_3	PF11_0224	0.054233	-0.48315	1.008303	0.688363	-1.15619
aPF11_0224_0	PF11_0224	0.054233	-0.48315	1.008303	0.688363	-1.15619
intPF11_0225_2	PF11_0225	0.432447	0.344287	0.444177	-0.09634	-0.87345
aPF11_0225_1	PF11_0225	0.432447	0.344287	0.444177	-0.09634	-0.87345
aPF11_0225_0	PF11_0225	0.432447	0.344287	0.444177	-0.09634	-0.87345
intPF11_0226_2	PF11_0226	1.179183	0.826073	-0.83166	-1.23618	-0.17191
aPF11_0226_3	PF11_0226	1.179183	0.826073	-0.83166	-1.23618	-0.17191
aPF11_0226_2	PF11_0226	1.179183	0.826073	-0.83166	-1.23618	-0.17191
aPF11_0226_1	PF11_0226	1.179183	0.826073	-0.83166	-1.23618	-0.17191
aPF11_0226_0	PF11_0226	1.179183	0.826073	-0.83166	-1.23618	-0.17191
aPF11_0227_1	PF11_0227	-0.40517	-1.29068	-0.9069	0.312063	1.405343
aPF11_0227_0	PF11_0227	-0.40517	-1.29068	-0.9069	0.312063	1.405343
intPF11_0227_2	PF11_0228	-0.40517	-1.29068	-0.9069	0.312063	1.405343
aPF11_0228_1	PF11_0228	0.259327	0.438367	0.415527	0.044307	-0.48953
aPF11_0228_0	PF11_0228	0.259327	0.438367	0.415527	0.044307	-0.48953

intPF11_0228_2	PF11_0229	0.259327	0.438367	0.415527	0.044307	-0.48953
aPF11_0229_1	PF11_0229	-0.89789	-1.09555	-0.29866	0.747823	1.238213
aPF11_0229_0	PF11_0229	-0.89789	-1.09555	-0.29866	0.747823	1.238213
intPF11_0230_2	PF11_0230	1.152567	1.514147	0.218717	-1.07538	-1.38881
intPF11_0229_2	PF11_0230	-0.89789	-1.09555	-0.29866	0.747823	1.238213
aPF11_0230_0	PF11_0230	1.152567	1.514147	0.218717	-1.07538	-1.38881
intPF11_0231_2	PF11_0231	1.7275	1.15648	-0.69746	-1.56554	-0.87448
aPF11_0231_0	PF11_0231	1.7275	1.15648	-0.69746	-1.56554	-0.87448
intPF11_0233_2	PF11_0232	-0.96236	-1.0729	-0.5572	0.509973	1.392013
intPF11_0232_2	PF11_0232	-0.01732	0.064093	0.421553	0.346613	-0.20443
aPF11_0232_1	PF11_0232	-0.01732	0.064093	0.421553	0.346613	-0.20443
aPF11_0232_0	PF11_0232	-0.01732	0.064093	0.421553	0.346613	-0.20443
aPF11_0233_2	PF11_0233	-0.96236	-1.0729	-0.5572	0.509973	1.392013
aPF11_0233_1	PF11_0233	-0.96236	-1.0729	-0.5572	0.509973	1.392013
aPF11_0233_0	PF11_0233	-0.96236	-1.0729	-0.5572	0.509973	1.392013
intPF11_0236_2	PF11_0234	-0.58646	-0.3712	-0.7946	-0.1874	1.1237
intPF11_0234_2	PF11_0234	-0.3187	-0.13797	-0.70179	-0.32037	0.820307
aPF11_0237_0	PF11_0234	-0.33928	0.074382	-0.66797	-0.30621	0.927972
aPF11_0236_1	PF11_0234	-0.58646	-0.3712	-0.7946	-0.1874	1.1237
aPF11_0236_0	PF11_0234	-0.58646	-0.3712	-0.7946	-0.1874	1.1237
aPF11_0235_0	PF11_0234	1.746743	-1.03673	-1.04904	-0.2523	0.271393
aPF11_0234_1	PF11_0234	-0.3187	-0.13797	-0.70179	-0.32037	0.820307
aPF11_0234_0	PF11_0234	-0.3187	-0.13797	-0.70179	-0.32037	0.820307
intPF11_0238_2	PF11_0238	-0.4967	-0.93351	-1.43699	-0.19235	1.889173
aPF11_0238_0	PF11_0238	-0.4967	-0.93351	-1.43699	-0.19235	1.889173
intPF11_0239_2	PF11_0239	0.894062	1.308032	-0.30391	-1.30087	-0.82015
aPF11_0239_2	PF11_0239	0.894062	1.308032	-0.30391	-1.30087	-0.82015
aPF11_0239_1	PF11_0239	0.894062	1.308032	-0.30391	-1.30087	-0.82015
aPF11_0239_0	PF11_0239	0.894062	1.308032	-0.30391	-1.30087	-0.82015
intPF11_0240_2	PF11_0240	0.328302	-0.30716	0.211652	0.281452	-0.28006
aPF11_0240_7	PF11_0240	0.328302	-0.30716	0.211652	0.281452	-0.28006
aPF11_0240_6	PF11_0240	0.328302	-0.30716	0.211652	0.281452	-0.28006
aPF11_0240_5	PF11_0240	0.328302	-0.30716	0.211652	0.281452	-0.28006
aPF11_0240_4	PF11_0240	0.328302	-0.30716	0.211652	0.281452	-0.28006
aPF11_0240_3	PF11_0240	0.328302	-0.30716	0.211652	0.281452	-0.28006
aPF11_0240_2	PF11_0240	0.328302	-0.30716	0.211652	0.281452	-0.28006
aPF11_0240_1	PF11_0240	0.328302	-0.30716	0.211652	0.281452	-0.28006
aPF11_0240_0	PF11_0240	0.328302	-0.30716	0.211652	0.281452	-0.28006
intPF11_0241_1	PF11_0241	0.337192	0.112062	-0.187	-0.39592	-0.28873
aPF11_0241_3	PF11_0241	0.337192	0.112062	-0.187	-0.39592	-0.28873
aPF11_0241_2	PF11_0241	0.337192	0.112062	-0.187	-0.39592	-0.28873
aPF11_0241_1	PF11_0241	0.337192	0.112062	-0.187	-0.39592	-0.28873
aPF11_0241_0	PF11_0241	0.337192	0.112062	-0.187	-0.39592	-0.28873
intPF11_0242_2	PF11_0242	0.452845	0.534545	-0.68672	-0.94929	0.040455
aPF11_0242_3	PF11_0242	0.452845	0.534545	-0.68672	-0.94929	0.040455
aPF11_0242_2	PF11_0242	0.452845	0.534545	-0.68672	-0.94929	0.040455
aPF11_0242_1	PF11_0242	0.452845	0.534545	-0.68672	-0.94929	0.040455

aPF11_0242_0	PF11_0242	0.452845	0.534545	-0.68672	-0.94929	0.040455
intPF11_0244_2	PF11_0243	-0.24958	-0.1276	-0.05904	0.067377	0.207437
intPF11_0243_2	PF11_0243	0.49028	-0.70139	-1.25322	-0.54378	0.85089
aPF11_0243_1	PF11_0243	0.49028	-0.70139	-1.25322	-0.54378	0.85089
aPF11_0243_0	PF11_0243	0.49028	-0.70139	-1.25322	-0.54378	0.85089
aPF11_0244_1	PF11_0244	-0.24958	-0.1276	-0.05904	0.067377	0.207437
aPF11_0244_0	PF11_0244	-0.24958	-0.1276	-0.05904	0.067377	0.207437
intPF11_0245_2	PF11_0245	-0.10281	0.30104	0.84442	0.49308	-0.57354
aPF11_0245_1	PF11_0245	-0.10281	0.30104	0.84442	0.49308	-0.57354
aPF11_0245_0	PF11_0245	-0.10281	0.30104	0.84442	0.49308	-0.57354
intPF11_0246_2	PF11_0246	0.287787	0.662087	0.183817	-0.35448	-0.52325
aPF11_0246_1	PF11_0246	0.287787	0.662087	0.183817	-0.35448	-0.52325
aPF11_0246_0	PF11_0246	0.287787	0.662087	0.183817	-0.35448	-0.52325
intPF11_0248_2	PF11_0247	-0.76577	-1.05219	0.363883	1.142783	0.600853
intPF11_0247_2	PF11_0247	0.821877	0.710137	0.458477	-0.29014	-1.0763
aPF11_0247_1	PF11_0247	0.821877	0.710137	0.458477	-0.29014	-1.0763
aPF11_0247_0	PF11_0247	0.821877	0.710137	0.458477	-0.29014	-1.0763
aPF11_0248_0	PF11_0248	-0.76577	-1.05219	0.363883	1.142783	0.600853
intPF11_0249_2	PF11_0249	-0.726	-0.93202	-0.58246	0.367123	1.239183
aPF11_0249_1	PF11_0249	-0.726	-0.93202	-0.58246	0.367123	1.239183
aPF11_0249_0	PF11_0249	-0.726	-0.93202	-0.58246	0.367123	1.239183
intPF11_0250_3	PF11_0250	0.290083	0.514603	1.076793	0.311873	-1.25994
aPF11_0250_0	PF11_0250	0.290083	0.514603	1.076793	0.311873	-1.25994
intPF11_0251_2	PF11_0251	0.199287	0.501057	-0.34062	-0.39954	0.292777
aPF11_0251_1	PF11_0251	0.199287	0.501057	-0.34062	-0.39954	0.292777
aPF11_0251_0	PF11_0251	0.199287	0.501057	-0.34062	-0.39954	0.292777
intPF11_0252_2	PF11_0252	0.324345	0.419265	-0.12303	-0.25776	0.010295
aPF11_0252_2	PF11_0252	0.324345	0.419265	-0.12303	-0.25776	0.010295
aPF11_0252_1	PF11_0252	0.324345	0.419265	-0.12303	-0.25776	0.010295
aPF11_0252_0	PF11_0252	0.324345	0.419265	-0.12303	-0.25776	0.010295
intPF11_0253_2	PF11_0253	-0.94075	-0.49721	-0.22633	0.179667	0.666547
aPF11_0253_1	PF11_0253	-0.94075	-0.49721	-0.22633	0.179667	0.666547
aPF11_0253_0	PF11_0253	-0.94075	-0.49721	-0.22633	0.179667	0.666547
intPF11_0254_2	PF11_0254	0.128213	0.004423	0.329913	0.241253	-0.28276
aPF11_0254_1	PF11_0254	0.128213	0.004423	0.329913	0.241253	-0.28276
aPF11_0254_0	PF11_0254	0.128213	0.004423	0.329913	0.241253	-0.28276
aPF11_0255_0	PF11_0255	0.3706	0.501	0.17999	-0.16899	-0.373
intPF11_0256_2	PF11_0256	-0.31127	0.02635	-0.0502	0.0922	0.35165
intPF11_0255_2	PF11_0256	0.3706	0.501	0.17999	-0.16899	-0.373
aPF11_0256_1	PF11_0256	-0.31127	0.02635	-0.0502	0.0922	0.35165
aPF11_0256_0	PF11_0256	-0.31127	0.02635	-0.0502	0.0922	0.35165
intPF11_0257_2	PF11_0257	-1.2348	-1.74099	-0.65777	0.83377	1.70749
aPF11_0257_1	PF11_0257	-1.2348	-1.74099	-0.65777	0.83377	1.70749
aPF11_0257_0	PF11_0257	-1.2348	-1.74099	-0.65777	0.83377	1.70749
intPF11_0258_2	PF11_0258	-0.26925	-0.30675	1.185497	0.942837	-0.87391
aPF11_0258_0	PF11_0258	-0.26925	-0.30675	1.185497	0.942837	-0.87391
intPF11_0259_3	PF11_0259	0.48328	0.40861	0.72178	0.12622	-1.02261

aPF11_0259_0	PF11_0259	0.48328	0.40861	0.72178	0.12622	-1.02261
intPF11_0260_2	PF11_0260	0.22254	0.44132	0.61736	0.32764	-0.42032
aPF11_0260_0	PF11_0260	0.22254	0.44132	0.61736	0.32764	-0.42032
intPF11_0262_2	PF11_0261	-1.11433	-0.79538	-0.18401	0.670673	1.184543
aPF11_0261_0	PF11_0261					
intPF11_0261_2	PF11_0262					
aPF11_0262_1	PF11_0262	-1.11433	-0.79538	-0.18401	0.670673	1.184543
aPF11_0262_0	PF11_0262	-1.11433	-0.79538	-0.18401	0.670673	1.184543
intPF11_0264_2	PF11_0263	-0.11076	0.311817	0.898357	0.420477	-0.77148
aPF11_0263_0	PF11_0263	0.446885	0.744385	0.711255	0.081095	-0.83504
intPF11_0263_2	PF11_0264	0.446885	0.744385	0.711255	0.081095	-0.83504
aPF11_0264_2	PF11_0264	-0.11076	0.311817	0.898357	0.420477	-0.77148
aPF11_0264_1	PF11_0264	-0.11076	0.311817	0.898357	0.420477	-0.77148
aPF11_0264_0	PF11_0264	-0.11076	0.311817	0.898357	0.420477	-0.77148
intPF11_0265_2	PF11_0265	0.035247	0.303787	1.290667	0.641667	-1.21295
aPF11_0265_1	PF11_0265	0.035247	0.303787	1.290667	0.641667	-1.21295
aPF11_0265_0	PF11_0265	0.035247	0.303787	1.290667	0.641667	-1.21295
intPF11_0266_2	PF11_0266	0.415413	0.534953	0.641083	0.124583	-0.77029
aPF11_0266_0	PF11_0266	0.415413	0.534953	0.641083	0.124583	-0.77029
intPF11_0267_3	PF11_0267	1.386037	0.484827	-1.63141	-2.31775	-0.62449
aPF11_0267_1	PF11_0267	1.386037	0.484827	-1.63141	-2.31775	-0.62449
aPF11_0267_0	PF11_0267	1.386037	0.484827	-1.63141	-2.31775	-0.62449
intPF11_0268_2	PF11_0268	1.217173	0.031653	-1.64457	-2.23927	-0.72849
aPF11_0268_1	PF11_0268	1.217173	0.031653	-1.64457	-2.23927	-0.72849
aPF11_0268_0	PF11_0268	1.217173	0.031653	-1.64457	-2.23927	-0.72849
intPF11_0269_2	PF11_0269	0.408507	0.373457	0.492577	-0.19324	-1.08012
aPF11_0269_0	PF11_0269	0.408507	0.373457	0.492577	-0.19324	-1.08012
intPF11_0270_2	PF11_0270	0.217397	0.457017	0.562467	0.162867	-0.55468
aPF11_0270_1	PF11_0270	0.217397	0.457017	0.562467	0.162867	-0.55468
aPF11_0270_0	PF11_0270	0.217397	0.457017	0.562467	0.162867	-0.55468
intPF11_0271_2	PF11_0271	-0.99256	-0.40519	0.144887	0.621947	0.728027
aPF11_0271_1	PF11_0271	-0.99256	-0.40519	0.144887	0.621947	0.728027
aPF11_0271_0	PF11_0271	-0.99256	-0.40519	0.144887	0.621947	0.728027
intPF11_0272_2	PF11_0272	0.389897	0.455517	0.649467	0.339867	-0.50718
aPF11_0272_0	PF11_0272	0.389897	0.455517	0.649467	0.339867	-0.50718
intPF11_0273_3	PF11_0273-a	0.011813	-0.11458	-0.1261	0.018263	0.178243
aPF11_0273_1	PF11_0273-a	0.011813	-0.11458	-0.1261	0.018263	0.178243
aPF11_0273_0	PF11_0273-a	0.011813	-0.11458	-0.1261	0.018263	0.178243
intPF11_0274_2	PF11_0274	0.379893	0.385513	0.830463	0.292203	-0.95385
aPF11_0274_0	PF11_0274	0.379893	0.385513	0.830463	0.292203	-0.95385
intPF11_0275_2	PF11_0275	0.472713	0.564423	0.558153	-0.13738	-1.01065
aPF11_0275_2	PF11_0275	0.472713	0.564423	0.558153	-0.13738	-1.01065
aPF11_0275_1	PF11_0275	0.472713	0.564423	0.558153	-0.13738	-1.01065
aPF11_0275_0	PF11_0275	0.472713	0.564423	0.558153	-0.13738	-1.01065
intPF11_0276_2	PF11_0276	0.145623	0.294883	0.808733	0.030933	-1.28822
aPF11_0276_1	PF11_0276	0.145623	0.294883	0.808733	0.030933	-1.28822
aPF11_0276_0	PF11_0276	0.145623	0.294883	0.808733	0.030933	-1.28822

intPF11_0278_2	PF11_0277	0.65261	-0.22255	-2.20941	-2.11209	0.51655
intPF11_0277_2	PF11_0277	0.85093	-0.48064	-2.30121	-2.05479	0.53264
aPF11_0278_1	PF11_0277	0.65261	-0.22255	-2.20941	-2.11209	0.51655
aPF11_0278_0	PF11_0277	0.65261	-0.22255	-2.20941	-2.11209	0.51655
aPF11_0277_0	PF11_0277	0.85093	-0.48064	-2.30121	-2.05479	0.53264
intPF11_0279_2	PF11_0279	0.949317	1.877407	0.570447	-1.19961	-1.91007
aPF11_0279_1	PF11_0279	0.949317	1.877407	0.570447	-1.19961	-1.91007
aPF11_0279_0	PF11_0279	0.949317	1.877407	0.570447	-1.19961	-1.91007
intPF11_0280_2	PF11_0280	-0.08128	0.013662	0.435052	0.385532	-0.18308
aPF11_0280_0	PF11_0280	-0.08128	0.013662	0.435052	0.385532	-0.18308
intPF11_0281_2	PF11_0281	0.586003	0.753003	0.324003	-0.35934	-0.82634
aPF11_0281_0	PF11_0281	0.586003	0.753003	0.324003	-0.35934	-0.82634
intPF11_0282_2	PF11_0282	-1.38439	-2.02543	0.04016	1.61204	1.45563
aPF11_0282_0	PF11_0282	-1.38439	-2.02543	0.04016	1.61204	1.45563
intPF11_0283_2	PF11_0283					
aPF11_0283_0	PF11_0283					
intPF11_0285_2	PF11_0284	-0.29182	0.04809	0.62833	0.52767	-0.23109
aPF11_0284_0	PF11_0284	0.35845	0.93676	0.75248	-0.10348	-1.02976
intPF11_0286_2	PF11_0285	-0.41473	-0.63886	-0.60353	0.08603	0.94786
intPF11_0284_2	PF11_0285	0.35845	0.93676	0.75248	-0.10348	-1.02976
aPF11_0285_0	PF11_0285	-0.29182	0.04809	0.62833	0.52767	-0.23109
aPF11_0286_1	PF11_0286	-0.41473	-0.63886	-0.60353	0.08603	0.94786
aPF11_0286_0	PF11_0286	-0.41473	-0.63886	-0.60353	0.08603	0.94786
intPF11_0287_3	PF11_0287	-0.54744	-1.22682	-0.04387	0.36387	-0.00918
aPF11_0287_0	PF11_0287	-0.54744	-1.22682	-0.04387	0.36387	-0.00918
intPF11_0288_2	PF11_0288	0.316763	-0.0408	-0.1134	-0.04844	0.002463
aPF11_0288_1	PF11_0288	0.316763	-0.0408	-0.1134	-0.04844	0.002463
aPF11_0288_0	PF11_0288	0.316763	-0.0408	-0.1134	-0.04844	0.002463
intPF11_0289_2	PF11_0289	0.100088	0.342568	0.526188	0.131918	-0.57179
aPF11_0289_2	PF11_0289	0.100088	0.342568	0.526188	0.131918	-0.57179
aPF11_0289_1	PF11_0289	0.100088	0.342568	0.526188	0.131918	-0.57179
aPF11_0289_0	PF11_0289	0.100088	0.342568	0.526188	0.131918	-0.57179
intPF11_0290_2	PF11_0290	0.768017	0.329907	-0.30832	-1.03784	-1.02757
aPF11_0290_1	PF11_0290	0.768017	0.329907	-0.30832	-1.03784	-1.02757
aPF11_0290_0	PF11_0290	0.768017	0.329907	-0.30832	-1.03784	-1.02757
intPF11_0291_3	PF11_0291	0.508908	0.249798	-0.15076	-0.37702	-0.29224
aPF11_0291_2	PF11_0291	0.508908	0.249798	-0.15076	-0.37702	-0.29224
aPF11_0291_1	PF11_0291	0.508908	0.249798	-0.15076	-0.37702	-0.29224
aPF11_0291_0	PF11_0291	0.508908	0.249798	-0.15076	-0.37702	-0.29224
intPF11_0293_2	PF11_0292	-0.24507	0.609333	0.195323	-0.36566	-0.41867
intPF11_0292_2	PF11_0292	-0.84576	-0.41117	0.642837	0.963497	0.274507
aPF11_0292_0	PF11_0292	-0.84576	-0.41117	0.642837	0.963497	0.274507
aPF11_0293_0	PF11_0293	-0.24507	0.609333	0.195323	-0.36566	-0.41867
aPF11_0294_2	PF11_0294	0.29008	0.92462	0.58225	-0.24525	-0.91562
aPF11_0294_1	PF11_0294	0.29008	0.92462	0.58225	-0.24525	-0.91562
aPF11_0294_0	PF11_0294	0.29008	0.92462	0.58225	-0.24525	-0.91562
intPF11_0296_2	PF11_0295	0.702743	1.498783	0.411693	-0.67253	-0.99862

intPF11_0295_2	PF11_0295	-0.02643	-0.57086	0.41621	0.76479	0.01236
intPF11_0294_2	PF11_0295	0.29008	0.92462	0.58225	-0.24525	-0.91562
aPF11_0295_0	PF11_0295	-0.02643	-0.57086	0.41621	0.76479	0.01236
aPF11_0296_1	PF11_0296	0.702743	1.498783	0.411693	-0.67253	-0.99862
aPF11_0296_0	PF11_0296	0.702743	1.498783	0.411693	-0.67253	-0.99862
intPF11_0297_2	PF11_0297	-0.4201	-0.61451	-0.1093	0.469818	0.635478
aPF11_0297_1	PF11_0297	-0.4201	-0.61451	-0.1093	0.469818	0.635478
aPF11_0297_0	PF11_0297	-0.4201	-0.61451	-0.1093	0.469818	0.635478
intPF11_0298_2	PF11_0298	-0.99334	-0.85376	-0.52872	0.354557	1.263097
aPF11_0298_1	PF11_0298	-0.99334	-0.85376	-0.52872	0.354557	1.263097
aPF11_0298_0	PF11_0298	-0.99334	-0.85376	-0.52872	0.354557	1.263097
intPF11_0300_2	PF11_0300	0.05661	-0.44356	-1.73464	-0.95336	1.43556
aPF11_0300_1	PF11_0300	0.05661	-0.44356	-1.73464	-0.95336	1.43556
aPF11_0300_0	PF11_0300	0.05661	-0.44356	-1.73464	-0.95336	1.43556
aPF11_0299_0	PF11_0300					
intPF11_0301_2	PF11_0301	-1.1193	-0.6385	1.054757	1.307077	-0.02566
aPF11_0301_0	PF11_0301	-1.1193	-0.6385	1.054757	1.307077	-0.02566
intPF11_0302_3	PF11_0302	0.087453	0.013243	0.775503	0.164663	-1.19508
aPF11_0302_1	PF11_0302	0.087453	0.013243	0.775503	0.164663	-1.19508
aPF11_0302_0	PF11_0302	0.087453	0.013243	0.775503	0.164663	-1.19508
intPF11_0303_2	PF11_0303	-0.94591	-0.48645	0.058393	0.592773	0.773113
aPF11_0303_1	PF11_0303	-0.94591	-0.48645	0.058393	0.592773	0.773113
aPF11_0303_0	PF11_0303	-0.94591	-0.48645	0.058393	0.592773	0.773113
intPF11_0304_2	PF11_0304	0.016267	-0.14597	-1.93471	-1.40662	1.334307
aPF11_0304_2	PF11_0304	0.016267	-0.14597	-1.93471	-1.40662	1.334307
aPF11_0304_1	PF11_0304	0.016267	-0.14597	-1.93471	-1.40662	1.334307
aPF11_0304_0	PF11_0304	0.016267	-0.14597	-1.93471	-1.40662	1.334307
aPF11_0305_1	PF11_0305	0.80002	0.9234	0.9202	-0.0292	-1.3819
aPF11_0305_0	PF11_0305	0.80002	0.9234	0.9202	-0.0292	-1.3819
intPF11_0307_2	PF11_0306	0.95893	-0.36964	-0.87171	-0.59029	0.14664
intPF11_0306_3	PF11_0306	-0.56732	-0.38388	0.371017	0.695817	0.296717
intPF11_0305_2	PF11_0306	0.80002	0.9234	0.9202	-0.0292	-1.3819
aPF11_0306_1	PF11_0306	-0.56732	-0.38388	0.371017	0.695817	0.296717
aPF11_0306_0	PF11_0306	-0.56732	-0.38388	0.371017	0.695817	0.296717
aPF11_0307_1	PF11_0307	0.95893	-0.36964	-0.87171	-0.59029	0.14664
aPF11_0307_0	PF11_0307	0.95893	-0.36964	-0.87171	-0.59029	0.14664
intPF11_0308_2	PF11_0308	-0.21154	0.263737	-0.46597	-0.34269	0.586597
aPF11_0308_0	PF11_0308	-0.21154	0.263737	-0.46597	-0.34269	0.586597
intPF11_0309_2	PF11_0309	0.398493	0.431523	0.221463	-0.0458	-0.31986
aPF11_0309_0	PF11_0309	0.398493	0.431523	0.221463	-0.0458	-0.31986
intPF11_0310_2	PF11_0310	-1.15088	-0.09312	1.43998	1.04302	-0.75388
aPF11_0310_1	PF11_0310	-1.15088	-0.09312	1.43998	1.04302	-0.75388
aPF11_0310_0	PF11_0310	-1.15088	-0.09312	1.43998	1.04302	-0.75388
aPF11_0311_1	PF11_0311	0.078847	-0.05325	0.058017	0.154817	0.064087
aPF11_0311_0	PF11_0311	0.078847	-0.05325	0.058017	0.154817	0.064087
intPF11_0311_2	PF11_0312	0.078847	-0.05325	0.058017	0.154817	0.064087
aPF11_0312_0	PF11_0312	0.39157	0.72479	0.19374	-0.18582	-0.28125

intPF11_0313_2	PF11_0313	-0.17255	0.584713	0.782063	0.423603	-0.35105
intPF11_0312_2	PF11_0313	0.39157	0.72479	0.19374	-0.18582	-0.28125
aPF11_0313_0	PF11_0313	-0.17255	0.584713	0.782063	0.423603	-0.35105
intPF11_0314_2	PF11_0314	-0.64889	-0.25505	0.09359	0.37391	0.43555
aPF11_0314_1	PF11_0314	-0.64889	-0.25505	0.09359	0.37391	0.43555
aPF11_0314_0	PF11_0314	-0.64889	-0.25505	0.09359	0.37391	0.43555
intPF11_0315_2	PF11_0315	-0.69041	-0.88494	-0.42985	0.429687	1.072777
aPF11_0315_1	PF11_0315	-0.69041	-0.88494	-0.42985	0.429687	1.072777
aPF11_0315_0	PF11_0315	-0.69041	-0.88494	-0.42985	0.429687	1.072777
intPF11_0316_2	PF11_0316	0.657403	0.834003	0.756733	-0.05357	-1.13084
aPF11_0316_1	PF11_0316	0.657403	0.834003	0.756733	-0.05357	-1.13084
aPF11_0316_0	PF11_0316	0.657403	0.834003	0.756733	-0.05357	-1.13084
intPF11_0317_2	PF11_0317	-0.9528	-0.95167	0.016512	0.855932	0.936782
aPF11_0317_2	PF11_0317	-0.9528	-0.95167	0.016512	0.855932	0.936782
aPF11_0317_1	PF11_0317	-0.9528	-0.95167	0.016512	0.855932	0.936782
aPF11_0317_0	PF11_0317	-0.9528	-0.95167	0.016512	0.855932	0.936782
intPF11_0319_2	PF11_0318	-0.96191	-0.44697	1.05718	1.08832	-0.28303
intPF11_0318_2	PF11_0318	-0.40043	-0.12193	0.159918	0.329488	0.253228
aPF11_0318_1	PF11_0318	-0.40043	-0.12193	0.159918	0.329488	0.253228
aPF11_0318_0	PF11_0318	-0.40043	-0.12193	0.159918	0.329488	0.253228
aPF11_0319_1	PF11_0319	-0.96191	-0.44697	1.05718	1.08832	-0.28303
aPF11_0319_0	PF11_0319	-0.96191	-0.44697	1.05718	1.08832	-0.28303
intPF11_0320_2	PF11_0320	0.306623	-1.1251	0.196193	0.858473	0.075763
aPF11_0320_0	PF11_0320	0.306623	-1.1251	0.196193	0.858473	0.075763
intPF11_0322_2	PF11_0321	0.39319	0.47705	0.0384	-0.3784	-0.45105
aPF11_0321_1	PF11_0321	-1.23437	0.53233	-0.1924	-0.2376	0.76267
aPF11_0321_0	PF11_0321	-1.23437	0.53233	-0.1924	-0.2376	0.76267
intPF11_0321_2	PF11_0322	-1.23437	0.53233	-0.1924	-0.2376	0.76267
aPF11_0322_0	PF11_0322	0.39319	0.47705	0.0384	-0.3784	-0.45105
aPF11_0323_1	PF11_0323	-0.86093	-1.03236	0.25871	0.96929	0.57836
aPF11_0323_0	PF11_0323	-0.86093	-1.03236	0.25871	0.96929	0.57836
intPF11_0324_2	PF11_0324	-1.38437	-0.50867	1.005582	1.220642	0.109722
intPF11_0323_2	PF11_0324	-0.86093	-1.03236	0.25871	0.96929	0.57836
aPF11_0324_2	PF11_0324	-1.38437	-0.50867	1.005582	1.220642	0.109722
aPF11_0324_1	PF11_0324	-1.38437	-0.50867	1.005582	1.220642	0.109722
aPF11_0324_0	PF11_0324	-1.38437	-0.50867	1.005582	1.220642	0.109722
intPF11_0325_2	PF11_0325	-0.79606	0.811297	1.127907	0.208427	-0.94896
aPF11_0325_0	PF11_0325	-0.79606	0.811297	1.127907	0.208427	-0.94896
intPF11_0326_2	PF11_0326	0.275158	0.350868	-0.10515	-0.23548	-0.01825
aPF11_0326_4	PF11_0326	0.275158	0.350868	-0.10515	-0.23548	-0.01825
aPF11_0326_3	PF11_0326	0.275158	0.350868	-0.10515	-0.23548	-0.01825
aPF11_0326_2	PF11_0326	0.275158	0.350868	-0.10515	-0.23548	-0.01825
aPF11_0326_1	PF11_0326	0.275158	0.350868	-0.10515	-0.23548	-0.01825
aPF11_0326_0	PF11_0326	0.275158	0.350868	-0.10515	-0.23548	-0.01825
intPF11_0327_2	PF11_0327	-0.96988	-0.65058	0.686328	0.661228	-0.37686
aPF11_0327_2	PF11_0327	-0.96988	-0.65058	0.686328	0.661228	-0.37686
aPF11_0327_1	PF11_0327	-0.96988	-0.65058	0.686328	0.661228	-0.37686

aPF11_0327_0	PF11_0327	-0.96988	-0.65058	0.686328	0.661228	-0.37686
intPF11_0328_2	PF11_0328	-0.79982	-0.89189	0.20179	0.68921	0.33589
aPF11_0328_1	PF11_0328	-0.79982	-0.89189	0.20179	0.68921	0.33589
aPF11_0328_0	PF11_0328	-0.79982	-0.89189	0.20179	0.68921	0.33589
intPF11_0329_1	PF11_0329	0.221447	-0.15725	-0.21524	-0.12692	0.025587
aPF11_0329_1	PF11_0329	0.221447	-0.15725	-0.21524	-0.12692	0.025587
aPF11_0329_0	PF11_0329	0.221447	-0.15725	-0.21524	-0.12692	0.025587
intPF11_0330_2	PF11_0330	-0.37281	0.128832	0.586442	0.338712	-0.35691
aPF11_0330_1	PF11_0330	-0.37281	0.128832	0.586442	0.338712	-0.35691
aPF11_0330_0	PF11_0330	-0.37281	0.128832	0.586442	0.338712	-0.35691
intPF11_0331_3	PF11_0331	0.233873	0.350633	0.987983	0.475683	-0.94197
aPF11_0331_1	PF11_0331	0.233873	0.350633	0.987983	0.475683	-0.94197
aPF11_0331_0	PF11_0331	0.233873	0.350633	0.987983	0.475683	-0.94197
intPF11_0332_2	PF11_0332	-1.19364	-1.11779	0.16857	1.11943	1.02279
aPF11_0332_0	PF11_0332	-1.19364	-1.11779	0.16857	1.11943	1.02279
aPF11_0333_2	PF11_0333	-1.12675	-0.25824	0.682972	0.687142	-0.01799
aPF11_0333_1	PF11_0333	-1.12675	-0.25824	0.682972	0.687142	-0.01799
aPF11_0333_0	PF11_0333	-1.12675	-0.25824	0.682972	0.687142	-0.01799
intPF11_0334_2	PF11_0334	-0.15663	-0.36685	-0.13931	-0.17003	-0.19199
intPF11_0333_2	PF11_0334	-1.12675	-0.25824	0.682972	0.687142	-0.01799
aPF11_0335_0	PF11_0334	-0.12377	-0.09715	-1.1227	-0.94664	0.614813
aPF11_0334_1	PF11_0334	-0.15663	-0.36685	-0.13931	-0.17003	-0.19199
aPF11_0334_0	PF11_0334	-0.15663	-0.36685	-0.13931	-0.17003	-0.19199
intPF11_0337_2	PF11_0336	0.29628	0.18561	0.08028	-0.03028	-0.16761
aPF11_0336_1	PF11_0336	0.024877	0.501637	-0.04877	-0.27489	0.003197
aPF11_0336_0	PF11_0336	0.024877	0.501637	-0.04877	-0.27489	0.003197
intPF11_0336_2	PF11_0337	0.024877	0.501637	-0.04877	-0.27489	0.003197
aPF11_0337_0	PF11_0337	0.29628	0.18561	0.08028	-0.03028	-0.16761
intPF11_0339_2	PF11_0338					
intPF11_0338_2	PF11_0338	-1.64574	-1.11032	-0.73664	0.395922	1.752752
aPF11_0338_0	PF11_0338	-1.64574	-1.11032	-0.73664	0.395922	1.752752
aPF11_0339_0	PF11_0339					
intPF11_0340_2	PF11_0340	0.137037	-0.5592	-1.3206	-0.70206	0.949537
intPF11_0341_2	PF11_0341	0.816717	0.780397	0.633707	-0.34187	-1.42656
aPF11_0341_1	PF11_0341	0.816717	0.780397	0.633707	-0.34187	-1.42656
aPF11_0341_0	PF11_0341	0.816717	0.780397	0.633707	-0.34187	-1.42656
intPF11_0342_2	PF11_0342	-1.17777	-1.43642	-0.79142	0.63817	1.84317
aPF11_0342_3	PF11_0342	-1.17777	-1.43642	-0.79142	0.63817	1.84317
aPF11_0342_2	PF11_0342	-1.17777	-1.43642	-0.79142	0.63817	1.84317
aPF11_0342_1	PF11_0342	-1.17777	-1.43642	-0.79142	0.63817	1.84317
aPF11_0342_0	PF11_0342	-1.17777	-1.43642	-0.79142	0.63817	1.84317
intPF11_0343_2	PF11_0343	-0.4757	-0.34153	-1.54495	-0.79739	1.567193
aPF11_0343_0	PF11_0343	-0.4757	-0.34153	-1.54495	-0.79739	1.567193
intPF11_0344_2	PF11_0344	0.861103	-0.32052	-1.85622	-1.82412	0.166183
aPF11_0344_1	PF11_0344	0.861103	-0.32052	-1.85622	-1.82412	0.166183
aPF11_0344_0	PF11_0344	0.861103	-0.32052	-1.85622	-1.82412	0.166183
intPF11_0345_2	PF11_0345	-0.22739	-0.66003	-0.02568	0.281017	0.104367

aPF11_0345_1	PF11_0345	-0.22739	-0.66003	-0.02568	0.281017	0.104367
aPF11_0345_0	PF11_0345	-0.22739	-0.66003	-0.02568	0.281017	0.104367
intPF11_0347_2	PF11_0346	1.7866	2.25448	0.08728	-1.84628	-2.02198
intPF11_0346_2	PF11_0346	0.53607	0.07667	0.4939	0.1926	-0.74417
aPF11_0346_1	PF11_0346	0.53607	0.07667	0.4939	0.1926	-0.74417
aPF11_0346_0	PF11_0346	0.53607	0.07667	0.4939	0.1926	-0.74417
aPF11_0347_1	PF11_0347	1.7866	2.25448	0.08728	-1.84628	-2.02198
aPF11_0347_0	PF11_0347	1.7866	2.25448	0.08728	-1.84628	-2.02198
intPF11_0348_3	PF11_0348	-0.35668	-0.30757	-0.23484	0.143677	0.559907
aPF11_0348_1	PF11_0348	-0.35668	-0.30757	-0.23484	0.143677	0.559907
aPF11_0348_0	PF11_0348	-0.35668	-0.30757	-0.23484	0.143677	0.559907
intPF11_0350_2	PF11_0349	0.533787	0.748077	-0.39366	-0.6405	0.081757
intPF11_0349_2	PF11_0349	0.922627	0.538867	-0.46823	-0.67643	-0.10453
aPF11_0350_1	PF11_0349	0.533787	0.748077	-0.39366	-0.6405	0.081757
aPF11_0350_0	PF11_0349	0.533787	0.748077	-0.39366	-0.6405	0.081757
aPF11_0349_0	PF11_0349	0.922627	0.538867	-0.46823	-0.67643	-0.10453
intPF11_0351_2	PF11_0351	-0.83531	-0.54846	1.489403	1.490263	-0.58238
aPF11_0351_1	PF11_0351	-0.83531	-0.54846	1.489403	1.490263	-0.58238
aPF11_0351_0	PF11_0351	-0.83531	-0.54846	1.489403	1.490263	-0.58238
intPF11_0352_2	PF11_0352	-0.78002	-0.95142	-0.16616	0.804823	1.137583
aPF11_0352_1	PF11_0352	-0.78002	-0.95142	-0.16616	0.804823	1.137583
aPF11_0352_0	PF11_0352	-0.78002	-0.95142	-0.16616	0.804823	1.137583
intPF11_0353_2	PF11_0353	0.71432	0.83205	0.65423	-0.25467	-1.26483
aPF11_0353_2	PF11_0353	0.71432	0.83205	0.65423	-0.25467	-1.26483
aPF11_0353_1	PF11_0353	0.71432	0.83205	0.65423	-0.25467	-1.26483
aPF11_0353_0	PF11_0353	0.71432	0.83205	0.65423	-0.25467	-1.26483
intPF11_0354_2	PF11_0354	-0.51841	-0.81443	-1.43276	-0.30482	1.799348
aPF11_0354_3	PF11_0354	-0.51841	-0.81443	-1.43276	-0.30482	1.799348
aPF11_0354_2	PF11_0354	-0.51841	-0.81443	-1.43276	-0.30482	1.799348
aPF11_0354_1	PF11_0354	-0.51841	-0.81443	-1.43276	-0.30482	1.799348
aPF11_0354_0	PF11_0354	-0.51841	-0.81443	-1.43276	-0.30482	1.799348
intPF11_0355_2	PF11_0355	-0.13796	-0.81716	0.321817	0.881517	0.233497
aPF11_0355_0	PF11_0355	-0.13796	-0.81716	0.321817	0.881517	0.233497
intPF11_0356_2	PF11_0356	-0.29963	0.07831	0.09897	0.0717	0.07819
aPF11_0356_2	PF11_0356	-0.29963	0.07831	0.09897	0.0717	0.07819
aPF11_0356_1	PF11_0356	-0.29963	0.07831	0.09897	0.0717	0.07819
aPF11_0356_0	PF11_0356	-0.29963	0.07831	0.09897	0.0717	0.07819
intPF11_0357_2	PF11_0357	0.31562	0.72026	0.10606	-0.41012	-0.43465
aPF11_0357_2	PF11_0357	0.31562	0.72026	0.10606	-0.41012	-0.43465
aPF11_0357_1	PF11_0357	0.31562	0.72026	0.10606	-0.41012	-0.43465
aPF11_0357_0	PF11_0357	0.31562	0.72026	0.10606	-0.41012	-0.43465
intPF11_0358_2	PF11_0358	0.802712	0.937762	0.753142	-0.40692	-1.63354
aPF11_0358_2	PF11_0358	0.802712	0.937762	0.753142	-0.40692	-1.63354
aPF11_0358_1	PF11_0358	0.802712	0.937762	0.753142	-0.40692	-1.63354
aPF11_0358_0	PF11_0358	0.802712	0.937762	0.753142	-0.40692	-1.63354
intPF11_0360_2	PF11_0359	-0.27432	-0.22892	-1.36418	-0.62216	1.506583
intPF11_0359_2	PF11_0359	0.590568	0.459218	-0.04047	-0.31824	-0.29386

aPF11_0359_1	PF11_0359	0.590568	0.459218	-0.04047	-0.31824	-0.29386
aPF11_0359_0	PF11_0359	0.590568	0.459218	-0.04047	-0.31824	-0.29386
intPF11_0361_2	PF11_0360	-0.53258	-0.76212	0.12475	0.76425	0.58712
aPF11_0360_1	PF11_0360	-0.27432	-0.22892	-1.36418	-0.62216	1.506583
aPF11_0360_0	PF11_0360	-0.27432	-0.22892	-1.36418	-0.62216	1.506583
aPF11_0361_0	PF11_0361-a	-0.53258	-0.76212	0.12475	0.76425	0.58712
intPF11_0362_1	PF11_0362	0.50014	1.21629	-0.28007	-1.12093	-0.52379
aPF11_0362_1	PF11_0362	0.50014	1.21629	-0.28007	-1.12093	-0.52379
aPF11_0362_0	PF11_0362	0.50014	1.21629	-0.28007	-1.12093	-0.52379
intPF11_0363_3	PF11_0363	0.22574	0.26432	0.14011	-0.01961	-0.18532
aPF11_0363_1	PF11_0363	0.22574	0.26432	0.14011	-0.01961	-0.18532
aPF11_0363_0	PF11_0363	0.22574	0.26432	0.14011	-0.01961	-0.18532
intPF11_0364_2	PF11_0364	0.1838	0.33299	0.32996	0.30894	0.01921
aPF11_0364_0	PF11_0364	0.1838	0.33299	0.32996	0.30894	0.01921
aPF11_0365_0	PF11_0365	-1.08097	-1.21204	0.087037	1.096897	1.038077
intPF11_0368_2	PF11_0368	-0.49253	-0.73984	0.17343	0.73457	0.45484
intPF11_0365_2	PF11_0368	-1.08097	-1.21204	0.087037	1.096897	1.038077
aPF11_0368_1	PF11_0368	-0.49253	-0.73984	0.17343	0.73457	0.45484
aPF11_0368_0	PF11_0368	-0.49253	-0.73984	0.17343	0.73457	0.45484
aPF11_0367_0	PF11_0368	-0.42504	-0.36878	0.26506	0.56994	0.26478
aPF11_0366_0	PF11_0368	0.308907	-0.53352	0.086547	0.340787	-0.10614
intPF11_0369_2	PF11_0369	0.083933	0.131313	0.702363	0.368303	-0.67565
aPF11_0369_0	PF11_0369	0.083933	0.131313	0.702363	0.368303	-0.67565
intPF11_0370_3	PF11_0370	-0.78523	-0.66381	-0.67438	0.227717	1.397147
aPF11_0370_0	PF11_0370	-0.78523	-0.66381	-0.67438	0.227717	1.397147
intPF11_0372_2	PF11_0371					
intPF11_0371_2	PF11_0371	-0.60535	-0.93763	-1.16012	0.025338	1.741598
aPF11_0371_5	PF11_0371	-0.60535	-0.93763	-1.16012	0.025338	1.741598
aPF11_0371_4	PF11_0371	-0.60535	-0.93763	-1.16012	0.025338	1.741598
aPF11_0371_3	PF11_0371	-0.60535	-0.93763	-1.16012	0.025338	1.741598
aPF11_0371_2	PF11_0371	-0.60535	-0.93763	-1.16012	0.025338	1.741598
aPF11_0371_1	PF11_0371	-0.60535	-0.93763	-1.16012	0.025338	1.741598
aPF11_0371_0	PF11_0371	-0.60535	-0.93763	-1.16012	0.025338	1.741598
aPF11_0372_1	PF11_0372					
aPF11_0372_0	PF11_0372					
intPF11_0373_2	PF11_0373	-0.23705	-0.21027	-1.76496	-1.25654	1.28977
aPF11_0373_1	PF11_0373	-0.23705	-0.21027	-1.76496	-1.25654	1.28977
aPF11_0373_0	PF11_0373	-0.23705	-0.21027	-1.76496	-1.25654	1.28977
intPF11_0374_2	PF11_0374	0.287167	0.756677	-0.21035	-0.42381	0.158657
aPF11_0374_1	PF11_0374	0.287167	0.756677	-0.21035	-0.42381	0.158657
aPF11_0374_0	PF11_0374	0.287167	0.756677	-0.21035	-0.42381	0.158657
intPF11_0375_2	PF11_0375	0.366537	0.439327	-0.07216	-0.461	-0.38849
aPF11_0375_1	PF11_0375	0.366537	0.439327	-0.07216	-0.461	-0.38849
aPF11_0375_0	PF11_0375	0.366537	0.439327	-0.07216	-0.461	-0.38849
intPF11_0377_2	PF11_0377-a	-0.28502	-0.44293	-0.41837	-0.03459	0.514833
aPF11_0377_0	PF11_0377-a	-0.28502	-0.44293	-0.41837	-0.03459	0.514833
intPF11_0378_2	PF11_0378	0.680543	0.692783	0.564183	-0.42752	-1.44062

aPF11_0378_1	PF11_0378	0.680543	0.692783	0.564183	-0.42752	-1.44062
aPF11_0378_0	PF11_0378	0.680543	0.692783	0.564183	-0.42752	-1.44062
intPF11_0379_2	PF11_0379	-0.56665	-0.19829	-1.07393	-0.79441	0.833953
aPF11_0379_1	PF11_0379	-0.56665	-0.19829	-1.07393	-0.79441	0.833953
aPF11_0379_0	PF11_0379	-0.56665	-0.19829	-1.07393	-0.79441	0.833953
intPF11_0380_3	PF11_0380	0.391387	0.171077	0.021847	-0.10151	-0.20874
aPF11_0380_0	PF11_0380	0.391387	0.171077	0.021847	-0.10151	-0.20874
intPF11_0381_2	PF11_0381	2.6837	1.10351	-1.37802	-2.52798	-1.42451
aPF11_0381_1	PF11_0381	2.6837	1.10351	-1.37802	-2.52798	-1.42451
aPF11_0381_0	PF11_0381	2.6837	1.10351	-1.37802	-2.52798	-1.42451
intPF11_0382_2	PF11_0381a	1.276397	0.783997	0.302507	-0.32217	-0.94066
aPF11_0382_0	PF11_0382	1.276397	0.783997	0.302507	-0.32217	-0.94066
intPF11_0383_2	PF11_0383	0.337893	-0.39295	-1.83711	-1.23723	1.137613
aPF11_0383_1	PF11_0383	0.337893	-0.39295	-1.83711	-1.23723	1.137613
aPF11_0383_0	PF11_0383	0.337893	-0.39295	-1.83711	-1.23723	1.137613
intPF11_0384_2	PF11_0384	0.34687	0.38565	0.61594	0.10506	-0.83015
aPF11_0384_1	PF11_0384	0.34687	0.38565	0.61594	0.10506	-0.83015
aPF11_0384_0	PF11_0384	0.34687	0.38565	0.61594	0.10506	-0.83015
intPF11_0385_2	PF11_0385	-0.82028	0.071883	0.946743	0.762423	-0.27072
aPF11_0385_1	PF11_0385	-0.82028	0.071883	0.946743	0.762423	-0.27072
aPF11_0385_0	PF11_0385	-0.82028	0.071883	0.946743	0.762423	-0.27072
aPF11_0386_0	PF11_0386	-1.03694	-1.1403	-0.49141	0.639073	1.444963
intPF11_0389_2	PF11_0388	-0.30719	-0.07156	0.04287	0.11363	0.14656
intPF11_0386_2	PF11_0388	-1.03694	-1.1403	-0.49141	0.639073	1.444963
aPF11_0388_0	PF11_0388	-0.21564	-0.31999	0.060347	0.257747	0.134527
aPF11_0387_1	PF11_0388					
aPF11_0387_0	PF11_0388					
intPF11_0388_2	PF11_0389	-0.21564	-0.31999	0.060347	0.257747	0.134527
aPF11_0389_1	PF11_0389	-0.30719	-0.07156	0.04287	0.11363	0.14656
aPF11_0389_0	PF11_0389	-0.30719	-0.07156	0.04287	0.11363	0.14656
intPF11_0390_2	PF11_0390	0.380297	0.176497	0.439517	0.201817	-0.51216
aPF11_0391_0	PF11_0390	0.4056	0.18989	-0.15583	-0.36777	-0.27459
aPF11_0390_1	PF11_0390	0.380297	0.176497	0.439517	0.201817	-0.51216
aPF11_0390_0	PF11_0390	0.380297	0.176497	0.439517	0.201817	-0.51216
intPF11_0392_2	PF11_0392	-0.45503	-0.63707	-2.06736	-0.88804	2.18507
aPF11_0392_4	PF11_0392	-0.45503	-0.63707	-2.06736	-0.88804	2.18507
aPF11_0392_3	PF11_0392	-0.45503	-0.63707	-2.06736	-0.88804	2.18507
aPF11_0392_2	PF11_0392	-0.45503	-0.63707	-2.06736	-0.88804	2.18507
aPF11_0392_1	PF11_0392	-0.45503	-0.63707	-2.06736	-0.88804	2.18507
aPF11_0392_0	PF11_0392	-0.45503	-0.63707	-2.06736	-0.88804	2.18507
intPF11_0394_2	PF11_0393	0.21219	-0.23895	-1.02265	-0.40045	1.04415
intPF11_0393_2	PF11_0393	1.199248	0.858788	0.323398	-0.47443	-1.13177
aPF11_0393_0	PF11_0393	1.199248	0.858788	0.323398	-0.47443	-1.13177
aPF11_0394_0	PF11_0394	0.21219	-0.23895	-1.02265	-0.40045	1.04415
intPF11_0530_2	PF11_0395					
intPF11_0395_2	PF11_0395	-0.49032	-0.62509	-1.55495	-0.51111	1.830797
aPF11_0395_6	PF11_0395	-0.49032	-0.62509	-1.55495	-0.51111	1.830797

aPF11_0395_5	PF11_0395	-0.49032	-0.62509	-1.55495	-0.51111	1.830797
aPF11_0395_4	PF11_0395	-0.49032	-0.62509	-1.55495	-0.51111	1.830797
aPF11_0395_3	PF11_0395	-0.49032	-0.62509	-1.55495	-0.51111	1.830797
aPF11_0395_2	PF11_0395	-0.49032	-0.62509	-1.55495	-0.51111	1.830797
aPF11_0395_1	PF11_0395	-0.49032	-0.62509	-1.55495	-0.51111	1.830797
aPF11_0395_0	PF11_0395	-0.49032	-0.62509	-1.55495	-0.51111	1.830797
intPF11_0396_3	PF11_0396	0.311317	0.613897	-0.39478	-0.63888	0.070937
aPF11_0396_1	PF11_0396	0.311317	0.613897	-0.39478	-0.63888	0.070937
aPF11_0396_0	PF11_0396	0.311317	0.613897	-0.39478	-0.63888	0.070937
intPF11_0397_2	PF11_0397	0.792893	0.612023	0.108703	-0.28654	-0.48536
aPF11_0397_1	PF11_0397	0.792893	0.612023	0.108703	-0.28654	-0.48536
aPF11_0397_0	PF11_0397	0.792893	0.612023	0.108703	-0.28654	-0.48536
aPF11_0398_2	PF11_0398	1.703913	0.912083	-0.07234	-1.43556	-1.98064
aPF11_0398_1	PF11_0398	1.703913	0.912083	-0.07234	-1.43556	-1.98064
aPF11_0398_0	PF11_0398	1.703913	0.912083	-0.07234	-1.43556	-1.98064
intPF11_0400_2	PF11_0400	0.871187	0.501087	-0.11717	-0.46149	-0.43375
aPF11_0401_2	PF11_0400	0.25275	0.52426	-0.06753	-0.38703	-0.19148
aPF11_0401_1	PF11_0400	0.25275	0.52426	-0.06753	-0.38703	-0.19148
aPF11_0401_0	PF11_0400	0.25275	0.52426	-0.06753	-0.38703	-0.19148
aPF11_0400_0	PF11_0400	0.871187	0.501087	-0.11717	-0.46149	-0.43375
aPF11_0399_0	PF11_0400	0.23979	0.48607	0.03936	-0.28136	-0.25007
intPF11_0401_2	PF11_0401	0.25275	0.52426	-0.06753	-0.38703	-0.19148
intPF11_0402_2	PF11_0402	0.43891	0.71777	0.32829	-0.15496	-0.5021
aPF11_0402_2	PF11_0402	0.43891	0.71777	0.32829	-0.15496	-0.5021
aPF11_0402_1	PF11_0402	0.43891	0.71777	0.32829	-0.15496	-0.5021
aPF11_0402_0	PF11_0402	0.43891	0.71777	0.32829	-0.15496	-0.5021
intPF11_0403_2	PF11_0403	0.86158	0.4331	0.25054	-0.09754	-0.6041
aPF11_0403_1	PF11_0403	0.86158	0.4331	0.25054	-0.09754	-0.6041
aPF11_0403_0	PF11_0403	0.86158	0.4331	0.25054	-0.09754	-0.6041
intPF11_0404_2	PF11_0404	-0.67127	0.771573	1.167353	0.359063	-0.86341
aPF11_0404_3	PF11_0404	-0.67127	0.771573	1.167353	0.359063	-0.86341
aPF11_0404_2	PF11_0404	-0.67127	0.771573	1.167353	0.359063	-0.86341
aPF11_0404_1	PF11_0404	-0.67127	0.771573	1.167353	0.359063	-0.86341
aPF11_0404_0	PF11_0404	-0.67127	0.771573	1.167353	0.359063	-0.86341
intPF11_0405_2	PF11_0405-a	0.347668	0.491478	-0.25995	-0.50721	-0.07248
aPF11_0405_1	PF11_0405-a	0.347668	0.491478	-0.25995	-0.50721	-0.07248
aPF11_0405_0	PF11_0405-a	0.347668	0.491478	-0.25995	-0.50721	-0.07248
intPF11_0406_2	PF11_0406	1.566327	0.323357	0.210297	-0.19396	-0.98302
aPF11_0406_1	PF11_0406	1.566327	0.323357	0.210297	-0.19396	-0.98302
aPF11_0406_0	PF11_0406	1.566327	0.323357	0.210297	-0.19396	-0.98302
intPF11_0407_2	PF11_0407	0.465527	-0.33364	-0.25322	-0.09444	-0.05502
aPF11_0407_1	PF11_0407	0.465527	-0.33364	-0.25322	-0.09444	-0.05502
aPF11_0407_0	PF11_0407	0.465527	-0.33364	-0.25322	-0.09444	-0.05502
intPF11_0408_2	PF11_0408	0.320758	0.128868	-0.25748	-0.14646	0.231358
aPF11_0408_3	PF11_0408	0.320758	0.128868	-0.25748	-0.14646	0.231358
aPF11_0408_2	PF11_0408	0.320758	0.128868	-0.25748	-0.14646	0.231358
aPF11_0408_1	PF11_0408	0.320758	0.128868	-0.25748	-0.14646	0.231358

aPF11_0408_0	PF11_0408	0.320758	0.128868	-0.25748	-0.14646	0.231358
intPF11_0409_2	PF11_0409	0.00315	0.13227	0.62397	0.48303	-0.33227
aPF11_0409_0	PF11_0409	0.00315	0.13227	0.62397	0.48303	-0.33227
intPF11_0411_2	PF11_0411	0.552757	0.439207	0.111577	-0.23124	-0.44287
aPF11_0411_0	PF11_0411	0.552757	0.439207	0.111577	-0.23124	-0.44287
aPF11_0410_1	PF11_0411	0.814248	0.703068	0.165528	-0.54208	-0.91929
aPF11_0410_0	PF11_0411	0.814248	0.703068	0.165528	-0.54208	-0.91929
intPF11_0412_2	PF11_0412	-0.58407	-0.05762	0.456247	0.510087	0.084957
aPF11_0412_0	PF11_0412	-0.58407	-0.05762	0.456247	0.510087	0.084957
intPF11_0413_2	PF11_0413	-1.35141	-0.76695	0.121917	0.740417	0.826287
aPF11_0413_0	PF11_0413	-1.35141	-0.76695	0.121917	0.740417	0.826287
intPF11_0414_2	PF11_0414	-1.0579	-0.419	0.505267	0.653567	0.127337
aPF11_0414_1	PF11_0414	-1.0579	-0.419	0.505267	0.653567	0.127337
aPF11_0414_0	PF11_0414	-1.0579	-0.419	0.505267	0.653567	0.127337
intPF11_0415_2	PF11_0415	-0.70992	-1.46039	-2.2007	-0.69196	2.259727
aPF11_0415_0	PF11_0415	-0.70992	-1.46039	-2.2007	-0.69196	2.259727
intPF11_0416_2	PF11_0416	-0.67681	-0.54197	-0.42381	0.037092	0.696912
aPF11_0416_2	PF11_0416	-0.67681	-0.54197	-0.42381	0.037092	0.696912
aPF11_0416_1	PF11_0416	-0.67681	-0.54197	-0.42381	0.037092	0.696912
aPF11_0416_0	PF11_0416	-0.67681	-0.54197	-0.42381	0.037092	0.696912
intPF11_0417_2	PF11_0417	0.398205	0.646985	-1.60606	-1.57945	0.873845
aPF11_0417_2	PF11_0417	0.398205	0.646985	-1.60606	-1.57945	0.873845
aPF11_0417_1	PF11_0417	0.398205	0.646985	-1.60606	-1.57945	0.873845
aPF11_0417_0	PF11_0417	0.398205	0.646985	-1.60606	-1.57945	0.873845
intPF11_0418_2	PF11_0418	0.690643	0.658873	0.751553	-0.14903	-1.39235
aPF11_0418_3	PF11_0418	0.690643	0.658873	0.751553	-0.14903	-1.39235
aPF11_0418_2	PF11_0418	0.690643	0.658873	0.751553	-0.14903	-1.39235
aPF11_0418_1	PF11_0418	0.690643	0.658873	0.751553	-0.14903	-1.39235
aPF11_0418_0	PF11_0418	0.690643	0.658873	0.751553	-0.14903	-1.39235
intPF11_0419_2	PF11_0419	0.763327	0.452847	0.243067	-0.29423	-0.83951
aPF11_0419_1	PF11_0419	0.763327	0.452847	0.243067	-0.29423	-0.83951
aPF11_0419_0	PF11_0419	0.763327	0.452847	0.243067	-0.29423	-0.83951
aPF11_0420_2	PF11_0420	-0.79446	-0.78871	-0.864	0.155052	1.555602
aPF11_0420_1	PF11_0420	-0.79446	-0.78871	-0.864	0.155052	1.555602
aPF11_0420_0	PF11_0420	-0.79446	-0.78871	-0.864	0.155052	1.555602
intPF11_0421_2	PF11_0421	0.185383	0.243093	-0.22419	-0.19715	0.215573
intPF11_0420_2	PF11_0421	-0.79446	-0.78871	-0.864	0.155052	1.555602
aPF11_0421_1	PF11_0421	0.185383	0.243093	-0.22419	-0.19715	0.215573
aPF11_0421_0	PF11_0421	0.185383	0.243093	-0.22419	-0.19715	0.215573
intPF11_0422_2	PF11_0422	0.788517	1.656427	0.609927	-0.61315	-1.19648
aPF11_0422_3	PF11_0422	0.788517	1.656427	0.609927	-0.61315	-1.19648
aPF11_0422_2	PF11_0422	0.788517	1.656427	0.609927	-0.61315	-1.19648
aPF11_0422_1	PF11_0422	0.788517	1.656427	0.609927	-0.61315	-1.19648
aPF11_0422_0	PF11_0422	0.788517	1.656427	0.609927	-0.61315	-1.19648
intPF11_0423_2	PF11_0423	-1.1296	-0.48302	-0.00813	0.52339	0.8545
aPF11_0423_0	PF11_0423	-1.1296	-0.48302	-0.00813	0.52339	0.8545
intPF11_0425_2	PF11_0424	-1.41998	-1.80608	-0.58584	1.09384	1.98408

aPF11_0424_1	PF11_0424	-0.35553	-0.57734	-0.72256	0.036897	1.113677
aPF11_0424_0	PF11_0424	-0.35553	-0.57734	-0.72256	0.036897	1.113677
intPF11_0426_2	PF11_0425	0.021863	-0.03333	-0.13335	-0.02799	0.172993
intPF11_0424_2	PF11_0425	-0.35553	-0.57734	-0.72256	0.036897	1.113677
aPF11_0425_0	PF11_0425	-1.41998	-1.80608	-0.58584	1.09384	1.98408
aPF11_0426_0	PF11_0426	0.021863	-0.03333	-0.13335	-0.02799	0.172993
aPF11_0427_0	PF11_0427	-0.61278	-0.69304	0.069612	0.612072	0.532722
intPF11_0428_2	PF11_0428	0.232503	0.448483	0.491043	0.212623	-0.35982
intPF11_0427_2	PF11_0428	-0.61278	-0.69304	0.069612	0.612072	0.532722
aPF11_0428_0	PF11_0428	0.232503	0.448483	0.491043	0.212623	-0.35982
intPF11_0429_2	PF11_0429	-0.6172	-1.12898	-1.99252	-0.49514	2.363317
aPF11_0429_0	PF11_0429	-0.6172	-1.12898	-1.99252	-0.49514	2.363317
intPF11_0432_2	PF11_0431	-0.81582	-0.63092	0.893323	1.045343	-0.21842
aPF11_0431_1	PF11_0431					
aPF11_0431_0	PF11_0431					
intPF11_0431_2	PF11_0432					
aPF11_0432_1	PF11_0432	-0.81582	-0.63092	0.893323	1.045343	-0.21842
aPF11_0432_0	PF11_0432	-0.81582	-0.63092	0.893323	1.045343	-0.21842
intPF11_0433_2	PF11_0433	0.895008	0.502188	-0.774	-0.88283	0.131228
aPF11_0433_3	PF11_0433	0.895008	0.502188	-0.774	-0.88283	0.131228
aPF11_0433_2	PF11_0433	0.895008	0.502188	-0.774	-0.88283	0.131228
aPF11_0433_1	PF11_0433	0.895008	0.502188	-0.774	-0.88283	0.131228
aPF11_0433_0	PF11_0433	0.895008	0.502188	-0.774	-0.88283	0.131228
intPF11_0435_2	PF11_0434	-0.82077	-0.53613	-0.32623	0.187237	0.795127
intPF11_0434_2	PF11_0434	-1.10429	0.29645	0.8591	0.5999	-0.10945
aPF11_0434_0	PF11_0434	-1.10429	0.29645	0.8591	0.5999	-0.10945
aPF11_0435_2	PF11_0435	-0.82077	-0.53613	-0.32623	0.187237	0.795127
aPF11_0435_1	PF11_0435	-0.82077	-0.53613	-0.32623	0.187237	0.795127
aPF11_0435_0	PF11_0435	-0.82077	-0.53613	-0.32623	0.187237	0.795127
intPF11_0436_3	PF11_0436	0.109437	0.198317	0.159367	-0.00903	-0.20998
aPF11_0436_1	PF11_0436	0.109437	0.198317	0.159367	-0.00903	-0.20998
aPF11_0436_0	PF11_0436	0.109437	0.198317	0.159367	-0.00903	-0.20998
intPF11_0437_2	PF11_0437	-0.00205	0.717277	1.007947	0.385387	-0.79094
aPF11_0437_0	PF11_0437	-0.00205	0.717277	1.007947	0.385387	-0.79094
intPF11_0438_3	PF11_0438	0.290507	0.379057	0.641437	0.288937	-0.58669
aPF11_0438_0	PF11_0438	0.290507	0.379057	0.641437	0.288937	-0.58669
intPF11_0440_2	PF11_0439	0.318098	0.114498	-0.02532	0.063838	0.112468
aPF11_0439_1	PF11_0439	0.553893	0.868013	0.823463	-0.0808	-1.23685
aPF11_0439_0	PF11_0439	0.553893	0.868013	0.823463	-0.0808	-1.23685
intPF11_0439_2	PF11_0440	0.553893	0.868013	0.823463	-0.0808	-1.23685
aPF11_0440_0	PF11_0440	0.318098	0.114498	-0.02532	0.063838	0.112468
intPF11_0441_2	PF11_0441	-0.30886	-0.75472	-0.8333	-0.0892	0.99372
aPF11_0441_0	PF11_0441	-0.30886	-0.75472	-0.8333	-0.0892	0.99372
aPF11_0441_1	PF11_0441a	-0.30886	-0.75472	-0.8333	-0.0892	0.99372
intPF11_0442_2	PF11_0442	1.186392	0.419052	-1.72911	-2.34889	-0.46939
aPF11_0442_2	PF11_0442	1.186392	0.419052	-1.72911	-2.34889	-0.46939
aPF11_0442_1	PF11_0442	1.186392	0.419052	-1.72911	-2.34889	-0.46939

aPF11_0442_0	PF11_0442	1.186392	0.419052	-1.72911	-2.34889	-0.46939
intPF11_0443_2	PF11_0443	-1.26964	-1.93983	-1.60335	0.454013	2.750493
aPF11_0443_0	PF11_0443	-1.26964	-1.93983	-1.60335	0.454013	2.750493
intPF11_0445_2	PF11_0445	0.34008	0.4496	0.56279	0.09121	-0.7086
aPF11_0445_0	PF11_0445	0.34008	0.4496	0.56279	0.09121	-0.7086
intPF11_0447_3	PF11_0447	-0.37593	0.111667	0.648177	0.508157	-0.22633
aPF11_0447_0	PF11_0447	-0.37593	0.111667	0.648177	0.508157	-0.22633
intPF11_0448_2	PF11_0448	-0.97644	-1.13678	-0.41119	0.61919	1.25028
aPF11_0448_1	PF11_0448	-0.97644	-1.13678	-0.41119	0.61919	1.25028
aPF11_0448_0	PF11_0448	-0.97644	-1.13678	-0.41119	0.61919	1.25028
aPF11_0449_0	PF11_0449					
intPF11_0451_2	PF11_0451	-0.72758	-0.97806	-0.18237	0.703707	0.980397
intPF11_0449_2	PF11_0451					
aPF11_0451_1	PF11_0451	-0.72758	-0.97806	-0.18237	0.703707	0.980397
aPF11_0451_0	PF11_0451	-0.72758	-0.97806	-0.18237	0.703707	0.980397
aPF11_0450_0	PF11_0451	0.620113	0.148423	-0.31635	-0.39799	-0.12476
intPF11_0452_3	PF11_0452	0.761873	0.304123	0.643503	-0.36634	-1.78046
aPF11_0452_1	PF11_0452	0.761873	0.304123	0.643503	-0.36634	-1.78046
aPF11_0452_0	PF11_0452	0.761873	0.304123	0.643503	-0.36634	-1.78046
intPF11_0454_1	PF11_0453	0.525197	0.780007	0.165487	-0.31815	-0.43567
intPF11_0453_2	PF11_0453	-0.29256	-0.31622	0.672923	0.704243	-0.29062
aPF11_0453_1	PF11_0453	-0.29256	-0.31622	0.672923	0.704243	-0.29062
aPF11_0453_0	PF11_0453	-0.29256	-0.31622	0.672923	0.704243	-0.29062
aPF11_0454_0	PF11_0454	0.525197	0.780007	0.165487	-0.31815	-0.43567
intPF11_0560_2	PF11_0455					
intPF11_0455_3	PF11_0455	-0.2387	0.187493	0.467513	0.343153	-0.13783
aPF11_0455_0	PF11_0455	-0.2387	0.187493	0.467513	0.343153	-0.13783
intPF11_0456_2	PF11_0456					
aPF11_0456_2	PF11_0456					
aPF11_0456_1	PF11_0456					
aPF11_0456_0	PF11_0456					
intPF11_0457_2	PF11_0457	0.714797	0.126017	0.451477	0.324857	-0.49468
aPF11_0457_0	PF11_0457	0.714797	0.126017	0.451477	0.324857	-0.49468
intPF11_0458_2	PF11_0458	0.841127	0.047347	0.185307	0.065027	-0.49101
aPF11_0458_0	PF11_0458	0.841127	0.047347	0.185307	0.065027	-0.49101
intPF11_0460_2	PF11_0459	-0.62065	-0.69326	-0.30024	0.44274	0.95926
aPF11_0459_0	PF11_0459	-0.80841	-0.9254	-0.22443	0.701948	1.116918
intPF11_0459_2	PF11_0460	-0.80841	-0.9254	-0.22443	0.701948	1.116918
aPF11_0460_1	PF11_0460	-0.62065	-0.69326	-0.30024	0.44274	0.95926
aPF11_0460_0	PF11_0460	-0.62065	-0.69326	-0.30024	0.44274	0.95926
intPF11_0461_2	PF11_0461	-0.69291	-0.44541	-0.41766	0.209997	1.014747
aPF11_0461_0	PF11_0461	-0.69291	-0.44541	-0.41766	0.209997	1.014747
intPF11_0462_2	PF11_0462	0.645737	0.367337	-0.00468	-0.43648	-0.6165
aPF11_0462_1	PF11_0462	0.645737	0.367337	-0.00468	-0.43648	-0.6165
aPF11_0462_0	PF11_0462	0.645737	0.367337	-0.00468	-0.43648	-0.6165
intPF11_0463_2	PF11_0463	0.145563	0.616703	-0.19991	-0.39443	0.137463
aPF11_0463_1	PF11_0463	0.145563	0.616703	-0.19991	-0.39443	0.137463

aPF11_0463_0	PF11_0463	0.145563	0.616703	-0.19991	-0.39443	0.137463
intPF11_0464_2	PF11_0464	-0.49355	-0.81104	-2.34731	-1.32395	1.941285
aPF11_0464_3	PF11_0464	-0.49355	-0.81104	-2.34731	-1.32395	1.941285
aPF11_0464_2	PF11_0464	-0.49355	-0.81104	-2.34731	-1.32395	1.941285
aPF11_0464_1	PF11_0464	-0.49355	-0.81104	-2.34731	-1.32395	1.941285
aPF11_0464_0	PF11_0464	-0.49355	-0.81104	-2.34731	-1.32395	1.941285
intPF11_0465_3	PF11_0465	-0.5006	-0.02251	-0.68725	-0.28709	0.981173
aPF11_0465_1	PF11_0465	-0.5006	-0.02251	-0.68725	-0.28709	0.981173
aPF11_0465_0	PF11_0465	-0.5006	-0.02251	-0.68725	-0.28709	0.981173
intPF11_0466_2	PF11_0466	-0.96108	-0.5581	0.633707	0.965627	0.234937
aPF11_0466_1	PF11_0466	-0.96108	-0.5581	0.633707	0.965627	0.234937
aPF11_0466_0	PF11_0466	-0.96108	-0.5581	0.633707	0.965627	0.234937
intPF11_0468_2	PF11_0467	-0.66473	-0.77286	-0.70904	0.203203	1.311023
intPF11_0467_2	PF11_0467					
aPF11_0467_0	PF11_0467					
aPF11_0468_1	PF11_0468	-0.66473	-0.77286	-0.70904	0.203203	1.311023
aPF11_0468_0	PF11_0468	-0.66473	-0.77286	-0.70904	0.203203	1.311023
intPF11_0469_2	PF11_0469	0.52607	0.55514	0.61196	-0.04996	-1.00214
aPF11_0469_1	PF11_0469	0.52607	0.55514	0.61196	-0.04996	-1.00214
aPF11_0469_0	PF11_0469	0.52607	0.55514	0.61196	-0.04996	-1.00214
intPF11_0470_2	PF11_0470	-0.11119	0.382933	-0.81687	-0.84947	0.515733
aPF11_0470_0	PF11_0470	-0.11119	0.382933	-0.81687	-0.84947	0.515733
intPF11_0471_2	PF11_0471	-0.52495	1.15427	0.73321	-0.23671	-0.75477
aPF11_0471_1	PF11_0471	-0.52495	1.15427	0.73321	-0.23671	-0.75477
aPF11_0471_0	PF11_0471	-0.52495	1.15427	0.73321	-0.23671	-0.75477
intPF11_0472_2	PF11_0472	-0.10454	-0.31509	0.369652	0.441332	-0.19093
aPF11_0472_0	PF11_0472	-0.10454	-0.31509	0.369652	0.441332	-0.19093
intPF11_0473_2	PF11_0473	0.69491	0.54046	0.43609	-0.53109	-1.46746
aPF11_0473_0	PF11_0473	0.69491	0.54046	0.43609	-0.53109	-1.46746
aPF11_0475_0	PF11_0474					
aPF11_0474_0	PF11_0474	0.544682	-0.18241	-1.00756	-0.78436	0.367492
intPF11_0476_2	PF11_0476	-0.28431	-0.34388	-0.23018	0.069882	0.392332
intPF11_0474_2	PF11_0476	0.544682	-0.18241	-1.00756	-0.78436	0.367492
aPF11_0476_0	PF11_0476	-0.28431	-0.34388	-0.23018	0.069882	0.392332
intPF11_0477_2	PF11_0477	0.119723	-0.45713	-1.36349	-0.85485	0.872793
aPF11_0477_1	PF11_0477	0.119723	-0.45713	-1.36349	-0.85485	0.872793
aPF11_0477_0	PF11_0477	0.119723	-0.45713	-1.36349	-0.85485	0.872793
intPF11_0478_2	PF11_0478	-0.08609	-0.05082	-0.57114	-0.33011	0.546743
aPF11_0478_1	PF11_0478	-0.08609	-0.05082	-0.57114	-0.33011	0.546743
aPF11_0478_0	PF11_0478	-0.08609	-0.05082	-0.57114	-0.33011	0.546743
intPF11_0479_3	PF11_0479	0.269307	0.267687	-0.07711	-0.29225	-0.2083
aPF11_0479_4	PF11_0479	0.269307	0.267687	-0.07711	-0.29225	-0.2083
aPF11_0479_3	PF11_0479	0.269307	0.267687	-0.07711	-0.29225	-0.2083
aPF11_0479_2	PF11_0479	0.269307	0.267687	-0.07711	-0.29225	-0.2083
aPF11_0479_1	PF11_0479	0.269307	0.267687	-0.07711	-0.29225	-0.2083
aPF11_0479_0	PF11_0479	0.269307	0.267687	-0.07711	-0.29225	-0.2083
intPF11_0480_2	PF11_0480	-0.31486	-0.344	-1.07241	-0.43853	1.183897

aPF11_0480_4	PF11_0480	-0.31486	-0.344	-1.07241	-0.43853	1.183897
aPF11_0480_3	PF11_0480	-0.31486	-0.344	-1.07241	-0.43853	1.183897
aPF11_0480_2	PF11_0480	-0.31486	-0.344	-1.07241	-0.43853	1.183897
aPF11_0480_1	PF11_0480	-0.31486	-0.344	-1.07241	-0.43853	1.183897
aPF11_0480_0	PF11_0480	-0.31486	-0.344	-1.07241	-0.43853	1.183897
intPF11_0481_2	PF11_0481	0.1362	0.09542	-0.82568	-0.92498	0.14449
aPF11_0481_4	PF11_0481	0.1362	0.09542	-0.82568	-0.92498	0.14449
aPF11_0481_3	PF11_0481	0.1362	0.09542	-0.82568	-0.92498	0.14449
aPF11_0481_2	PF11_0481	0.1362	0.09542	-0.82568	-0.92498	0.14449
aPF11_0481_1	PF11_0481	0.1362	0.09542	-0.82568	-0.92498	0.14449
aPF11_0481_0	PF11_0481	0.1362	0.09542	-0.82568	-0.92498	0.14449
intPF11_0482_2	PF11_0482	-0.9945	-0.88753	0.143063	0.927103	0.868193
aPF11_0482_2	PF11_0482	-0.9945	-0.88753	0.143063	0.927103	0.868193
aPF11_0482_1	PF11_0482	-0.9945	-0.88753	0.143063	0.927103	0.868193
aPF11_0482_0	PF11_0482	-0.9945	-0.88753	0.143063	0.927103	0.868193
intPF11_0483_2	PF11_0483	-0.02663	-0.26185	0.126693	0.353973	0.133013
aPF11_0483_0	PF11_0483	-0.02663	-0.26185	0.126693	0.353973	0.133013
aPF11_0483_1	PF11_0483a	-0.02663	-0.26185	0.126693	0.353973	0.133013
aPF11_0484_1	PF11_0484	-0.59205	-1.53323	-0.27729	0.95979	1.12323
aPF11_0484_0	PF11_0484	-0.59205	-1.53323	-0.27729	0.95979	1.12323
intPF11_0485_2	PF11_0485	-1.18331	-1.11443	0.000917	0.918517	1.027567
intPF11_0484_2	PF11_0485	-0.59205	-1.53323	-0.27729	0.95979	1.12323
aPF11_0485_0	PF11_0485	-1.18331	-1.11443	0.000917	0.918517	1.027567
intPF11_0486_2	PF11_0486	0.282647	-0.05888	-0.17739	-0.01316	0.171997
aPF11_0486_3	PF11_0486	0.282647	-0.05888	-0.17739	-0.01316	0.171997
aPF11_0486_2	PF11_0486	0.282647	-0.05888	-0.17739	-0.01316	0.171997
aPF11_0486_1	PF11_0486	0.282647	-0.05888	-0.17739	-0.01316	0.171997
aPF11_0486_0	PF11_0486	0.282647	-0.05888	-0.17739	-0.01316	0.171997
intPF11_0487_2	PF11_0487					
aPF11_0487_0	PF11_0487					
intPF11_0488_2	PF11_0488	-1.04294	-0.78127	-0.11803	0.669427	1.047327
aPF11_0488_2	PF11_0488	-1.04294	-0.78127	-0.11803	0.669427	1.047327
aPF11_0488_1	PF11_0488	-1.04294	-0.78127	-0.11803	0.669427	1.047327
aPF11_0488_0	PF11_0488	-1.04294	-0.78127	-0.11803	0.669427	1.047327
intPF11_0503_2	PF11_0503	0.09665	2.60573	1.65605	-0.72105	-2.41273
aPF11_0503_0	PF11_0503	0.09665	2.60573	1.65605	-0.72105	-2.41273
intPF11_0504_2	PF11_0504	3.176713	1.144433	-0.71286	-2.27048	-2.39477
aPF11_0504_0	PF11_0504	3.176713	1.144433	-0.71286	-2.27048	-2.39477
intPF11_0505_2	PF11_0505	1.7928	2.00601	0.57398	-1.71698	-2.88801
aPF11_0505_0	PF11_0505	1.7928	2.00601	0.57398	-1.71698	-2.88801
intPF11_0507_2	PF11_0506	-1.61843	-0.97496	1.251342	1.605812	0.004092
intPF11_0506_2	PF11_0506	-1.46974	-1.15026	1.211767	1.640067	-0.0384
aPF11_0507_8	PF11_0506	-1.61843	-0.97496	1.251342	1.605812	0.004092
aPF11_0507_7	PF11_0506	-1.61843	-0.97496	1.251342	1.605812	0.004092
aPF11_0507_6	PF11_0506	-1.61843	-0.97496	1.251342	1.605812	0.004092
aPF11_0507_5	PF11_0506	-1.61843	-0.97496	1.251342	1.605812	0.004092
aPF11_0507_4	PF11_0506	-1.61843	-0.97496	1.251342	1.605812	0.004092

aPF11_0507_3	PF11_0506	-1.61843	-0.97496	1.251342	1.605812	0.004092
aPF11_0507_2	PF11_0506	-1.61843	-0.97496	1.251342	1.605812	0.004092
aPF11_0507_1	PF11_0506	-1.61843	-0.97496	1.251342	1.605812	0.004092
aPF11_0507_0	PF11_0506	-1.61843	-0.97496	1.251342	1.605812	0.004092
aPF11_0506_1	PF11_0506	-1.46974	-1.15026	1.211767	1.640067	-0.0384
aPF11_0506_0	PF11_0506	-1.46974	-1.15026	1.211767	1.640067	-0.0384
intPF11_0508_2	PF11_0508	-0.64971	0.723547	0.999647	0.405687	-0.52521
aPF11_0508_1	PF11_0508	-0.64971	0.723547	0.999647	0.405687	-0.52521
aPF11_0508_0	PF11_0508	-0.64971	0.723547	0.999647	0.405687	-0.52521
intPF11_0509_2	PF11_0509	2.948873	0.887113	-1.44348	-2.97986	-2.21495
aPF11_0509_1	PF11_0509	2.948873	0.887113	-1.44348	-2.97986	-2.21495
aPF11_0509_0	PF11_0509	2.948873	0.887113	-1.44348	-2.97986	-2.21495
intPF11_0510_2	PF11_0510	1.707497	1.118537	0.146917	-1.23558	-1.9462
aPF11_0510_1	PF11_0510	1.707497	1.118537	0.146917	-1.23558	-1.9462
aPF11_0510_0	PF11_0510	1.707497	1.118537	0.146917	-1.23558	-1.9462
intPF11_0511_2	PF11_0511	-2.58266	-1.32336	2.168902	2.299882	-0.54786
aPF11_0511_0	PF11_0511	-2.58266	-1.32336	2.168902	2.299882	-0.54786
intPF11_0512_2	PF11_0512	1.02505	1.02274	0.04527	-0.71827	-0.82024
aPF11_0512_1	PF11_0512	1.02505	1.02274	0.04527	-0.71827	-0.82024
aPF11_0512_0	PF11_0512	1.02505	1.02274	0.04527	-0.71827	-0.82024
intPF11_0513_3	PF11_0513	1.650897	1.507027	0.103447	-1.94661	-2.65519
aPF11_0513_1	PF11_0513	1.650897	1.507027	0.103447	-1.94661	-2.65519
aPF11_0513_0	PF11_0513	1.650897	1.507027	0.103447	-1.94661	-2.65519
intPF11_0514_2	PF11_0514	0.56797	0.57037	1.01286	-0.13466	-1.86797
aPF11_0518_0	PF11_0518					
intPF11_0524_2	PF11_0524	-0.20641	0.30006	0.15837	0.03963	0.03794
aPF11_0524_0	PF11_0524	-0.20641	0.30006	0.15837	0.03963	0.03794
intPF11_0526_2	PF11_0526	1.04609	0.59654	0.50791	-0.06491	-0.97954
aPF11_0526_0	PF11_0526	1.04609	0.59654	0.50791	-0.06491	-0.97954
intPF11_0134_2	PF11_0527	0.236583	0.747083	0.320583	-0.21292	-0.48792
aPF11_0527_1	PF11_0527	0.472963	-0.05684	-0.06257	-0.06277	-0.175
aPF11_0527_0	PF11_0527	0.472963	-0.05684	-0.06257	-0.06277	-0.175
intPF11_0528_2	PF11_0528	-0.06025	-0.19613	-1.37618	-0.82776	1.170695
aPF11_0528_8	PF11_0528	-0.06025	-0.19613	-1.37618	-0.82776	1.170695
aPF11_0528_7	PF11_0528	-0.06025	-0.19613	-1.37618	-0.82776	1.170695
aPF11_0528_6	PF11_0528	-0.06025	-0.19613	-1.37618	-0.82776	1.170695
aPF11_0528_5	PF11_0528	-0.06025	-0.19613	-1.37618	-0.82776	1.170695
aPF11_0528_4	PF11_0528	-0.06025	-0.19613	-1.37618	-0.82776	1.170695
aPF11_0528_3	PF11_0528	-0.06025	-0.19613	-1.37618	-0.82776	1.170695
aPF11_0528_2	PF11_0528	-0.06025	-0.19613	-1.37618	-0.82776	1.170695
aPF11_0528_1	PF11_0528	-0.06025	-0.19613	-1.37618	-0.82776	1.170695
aPF11_0528_0	PF11_0528	-0.06025	-0.19613	-1.37618	-0.82776	1.170695
intPF11_0535_2	PF11_0535					
intPF11_0540_2	PF11_0540					
aPF11_0525_0	PF11_0540					
aPF11_0525_2	PF11_0540a					
aPF11_0525_1	PF11_0540a					

intPF11_0545_2	PF11_0545					
intPF11_0550_3	PF11_0550					
intPF13_0010_2	PF13_0010	1.03745	0.86027	0.23571	-0.33621	-0.70877
aPF13_0010_0	PF13_0010	1.03745	0.86027	0.23571	-0.33621	-0.70877
intPF13_0011_2	PF13_0011	-0.65866	-0.57774	-0.04653	0.336898	0.425058
aPF13_0011_0	PF13_0011	-0.65866	-0.57774	-0.04653	0.336898	0.425058
intPF13_0012_2	PF13_0012	2.102037	0.620817	-0.83364	-1.74802	-1.38048
aPF13_0012_0	PF13_0012	2.102037	0.620817	-0.83364	-1.74802	-1.38048
aPF13_0013_1	PF13_0013	0.976697	0.150037	0.367927	-0.04809	-0.9722
aPF13_0013_0	PF13_0013	0.976697	0.150037	0.367927	-0.04809	-0.9722
intPF13_0014_1	PF13_0014	0.402067	0.402647	0.681217	0.362117	-0.57131
aPF13_0014_0	PF13_0014	0.402067	0.402647	0.681217	0.362117	-0.57131
intPF13_0015_2	PF13_0015	-0.47586	0.528793	1.199413	0.558753	-0.84963
intMAL13P1.15_2	PF13_0015	-0.76008	-0.7196	0.36721	0.95279	0.5296
aPF13_0015_1	PF13_0015	-0.47586	0.528793	1.199413	0.558753	-0.84963
aPF13_0015_0	PF13_0015	-0.47586	0.528793	1.199413	0.558753	-0.84963
aPF13_0016_0	PF13_0016	-0.82968	-0.4466	0.339717	0.778617	0.523937
aPF13_0017_0	PF13_0017	0.505133	0.572343	0.623813	0.095853	-0.78668
intPF13_0018_1	PF13_0018	0.302943	0.229803	0.134883	-0.21072	-0.51114
aPF13_0018_1	PF13_0018	0.302943	0.229803	0.134883	-0.21072	-0.51114
aPF13_0018_0	PF13_0018	0.302943	0.229803	0.134883	-0.21072	-0.51114
intPF13_0019_3	PF13_0019	0.35925	0.12539	-1.0272	-0.96363	0.40161
aPF13_0019_2	PF13_0019	0.35925	0.12539	-1.0272	-0.96363	0.40161
aPF13_0019_1	PF13_0019	0.35925	0.12539	-1.0272	-0.96363	0.40161
aPF13_0019_0	PF13_0019	0.35925	0.12539	-1.0272	-0.96363	0.40161
intPF13_0020_2	PF13_0020	0.51504	0.37928	-0.36406	-0.39694	0.15322
aPF13_0020_1	PF13_0020	0.51504	0.37928	-0.36406	-0.39694	0.15322
aPF13_0020_0	PF13_0020	0.51504	0.37928	-0.36406	-0.39694	0.15322
intMAL13P1.25_2	PF13_0021	-1.07747	-0.53466	0.687563	0.943103	0.156823
aPF13_0021_0	PF13_0021	-1.72265	-0.90812	0.583045	1.283855	0.825665
aPF13_0022_0	PF13_0022	-0.36217	-0.23815	0.453793	0.569873	-0.01519
intPF13_0023_3	PF13_0023	0.145723	0.112373	0.617263	0.378403	-0.53771
aPF13_0023_0	PF13_0023	0.145723	0.112373	0.617263	0.378403	-0.53771
intPF13_0024_2	PF13_0024	-1.21733	-1.52469	-0.19228	1.0145	1.26358
aPF13_0024_2	PF13_0024	-1.21733	-1.52469	-0.19228	1.0145	1.26358
aPF13_0024_1	PF13_0024	-1.21733	-1.52469	-0.19228	1.0145	1.26358
aPF13_0024_0	PF13_0024	-1.21733	-1.52469	-0.19228	1.0145	1.26358
intPF13_0026_2	PF13_0025	-0.29003	-0.03484	0.174687	0.300647	0.204177
intPF13_0025_2	PF13_0025	-0.61592	-0.74943	-0.4724	0.322563	1.055593
aPF13_0025_1	PF13_0025	-0.61592	-0.74943	-0.4724	0.322563	1.055593
aPF13_0025_0	PF13_0025	-0.61592	-0.74943	-0.4724	0.322563	1.055593
aPF13_0026_0	PF13_0026	-0.29003	-0.03484	0.174687	0.300647	0.204177
intPF13_0027_2	PF13_0027	0.786953	0.582233	0.587523	0.063143	-0.85657
intMAL13P1.28_2	PF13_0027	0.357748	0.646018	0.513088	-0.02392	-0.6651
aPF13_0027_1	PF13_0027	0.786953	0.582233	0.587523	0.063143	-0.85657
aPF13_0027_0	PF13_0027	0.786953	0.582233	0.587523	0.063143	-0.85657
intPF13_0028_2	PF13_0028	-0.70755	-0.73627	-0.17998	0.604643	0.981933

aPF13_0028_0	PF13_0028	-0.70755	-0.73627	-0.17998	0.604643	0.981933
intPF13_0030_2	PF13_0029	-0.40646	-0.10302	0.140372	0.209852	0.124242
intPF13_0029_2	PF13_0029	0.256973	0.810143	0.581223	-0.03156	-0.65848
aPF13_0029_0	PF13_0029	0.256973	0.810143	0.581223	-0.03156	-0.65848
aPF13_0030_0	PF13_0030	-0.40646	-0.10302	0.140372	0.209852	0.124242
aPF13_0031_1	PF13_0031	-1.02527	-0.17316	0.666073	0.604093	-0.08868
aPF13_0031_0	PF13_0031	-1.02527	-0.17316	0.666073	0.604093	-0.08868
intPF13_0032_2	PF13_0032	-1.17241	-1.34245	-0.49486	0.837023	1.658613
aPF13_0032_1	PF13_0032	-1.17241	-1.34245	-0.49486	0.837023	1.658613
aPF13_0032_0	PF13_0032	-1.17241	-1.34245	-0.49486	0.837023	1.658613
intPF13_0033_2	PF13_0033	-1.115	-0.5275	-0.175	0.519337	1.119837
aPF13_0033_1	PF13_0033	-1.115	-0.5275	-0.175	0.519337	1.119837
aPF13_0033_0	PF13_0033	-1.115	-0.5275	-0.175	0.519337	1.119837
intPF13_0034_3	PF13_0034	-0.34071	0.13707	0.44986	0.34214	-0.10657
aPF13_0034_1	PF13_0034	-0.34071	0.13707	0.44986	0.34214	-0.10657
aPF13_0034_0	PF13_0034	-0.34071	0.13707	0.44986	0.34214	-0.10657
intPF13_0035_2	PF13_0035	0.468217	0.441897	0.086207	-0.50537	-0.77756
intMAL13P1.36_2	PF13_0035	0.170208	0.214548	0.566068	0.240048	-0.59143
aPF13_0035_1	PF13_0035	0.468217	0.441897	0.086207	-0.50537	-0.77756
aPF13_0035_0	PF13_0035	0.468217	0.441897	0.086207	-0.50537	-0.77756
intPF13_0037_2	PF13_0036	0.70121	1.12494	0.63237	-0.17587	-0.90694
aPF13_0036_0	PF13_0036	0.0785	0.2605	0.132	0.064	0.0015
intPF13_0036_2	PF13_0037	0.0785	0.2605	0.132	0.064	0.0015
aPF13_0037_1	PF13_0037	0.70121	1.12494	0.63237	-0.17587	-0.90694
aPF13_0037_0	PF13_0037	0.70121	1.12494	0.63237	-0.17587	-0.90694
aPF13_0038_1	PF13_0038	0.513883	0.028073	-0.2159	-0.21544	-0.07991
aPF13_0038_0	PF13_0038	0.513883	0.028073	-0.2159	-0.21544	-0.07991
intPF13_0039_2	PF13_0039	0.690647	-0.26823	-1.78953	-1.77813	0.204567
intPF13_0038_2	PF13_0039	0.513883	0.028073	-0.2159	-0.21544	-0.07991
aPF13_0039_0	PF13_0039	0.690647	-0.26823	-1.78953	-1.77813	0.204567
intPF13_0040_2	PF13_0040	-0.7282	-0.66199	-0.2555	0.299337	0.718827
aPF13_0040_1	PF13_0040	-0.7282	-0.66199	-0.2555	0.299337	0.718827
aPF13_0040_0	PF13_0040	-0.7282	-0.66199	-0.2555	0.299337	0.718827
intPF13_0041_3	PF13_0041	-0.03429	0.153423	-0.19134	-0.4035	-0.14076
aPF13_0041_1	PF13_0041	-0.03429	0.153423	-0.19134	-0.4035	-0.14076
aPF13_0041_0	PF13_0041	-0.03429	0.153423	-0.19134	-0.4035	-0.14076
intPF13_0042_2	PF13_0042	0.11482	0.24188	0.56223	0.30327	-0.45288
aPF13_0042_1	PF13_0042	0.11482	0.24188	0.56223	0.30327	-0.45288
aPF13_0042_0	PF13_0042	0.11482	0.24188	0.56223	0.30327	-0.45288
intPF13_0043_2	PF13_0043	0.784073	0.008623	0.249253	0.178413	-0.44496
aPF13_0043_0	PF13_0043	0.784073	0.008623	0.249253	0.178413	-0.44496
intPF13_0044_2	PF13_0044	0.115798	0.378488	0.780278	0.248498	-0.84404
aPF13_0044_3	PF13_0044	0.115798	0.378488	0.780278	0.248498	-0.84404
aPF13_0044_2	PF13_0044	0.115798	0.378488	0.780278	0.248498	-0.84404
aPF13_0044_1	PF13_0044	0.115798	0.378488	0.780278	0.248498	-0.84404
aPF13_0044_0	PF13_0044	0.115798	0.378488	0.780278	0.248498	-0.84404
intPF13_0045_2	PF13_0045	0.00865	0.24173	0.61155	0.46145	-0.27073

aPF13_0045_0	PF13_0045	0.00865	0.24173	0.61155	0.46145	-0.27073
aPF13_0047_1	PF13_0047	-1.1794	-1.13247	-0.35329	0.627627	1.227307
aPF13_0047_0	PF13_0047	-1.1794	-1.13247	-0.35329	0.627627	1.227307
intPF13_0048_3	PF13_0048	-0.11993	0.503177	0.540157	0.125677	-0.42634
aPF13_0048_1	PF13_0048	-0.11993	0.503177	0.540157	0.125677	-0.42634
aPF13_0048_0	PF13_0048	-0.11993	0.503177	0.540157	0.125677	-0.42634
intPF13_0049_2	PF13_0049	0.012985	0.314335	0.629455	0.307295	-0.48784
aPF13_0049_0	PF13_0049	0.012985	0.314335	0.629455	0.307295	-0.48784
intPF13_0050_2	PF13_0050	-0.60159	-0.35801	-0.31147	0.129307	0.726847
aPF13_0050_1	PF13_0050	-0.60159	-0.35801	-0.31147	0.129307	0.726847
aPF13_0050_0	PF13_0050	-0.60159	-0.35801	-0.31147	0.129307	0.726847
intPF13_0052_2	PF13_0051	0.092073	1.055643	0.263713	-0.48505	-0.53798
aPF13_0051_0	PF13_0051	0.714353	0.553253	0.708993	0.005673	-1.16059
intPF13_0053_2	PF13_0052	0.986988	0.801068	0.002368	-0.53748	-0.60551
intPF13_0051_2	PF13_0052	0.714353	0.553253	0.708993	0.005673	-1.16059
aPF13_0052_0	PF13_0052	0.092073	1.055643	0.263713	-0.48505	-0.53798
aPF13_0053_2	PF13_0053	0.986988	0.801068	0.002368	-0.53748	-0.60551
aPF13_0053_1	PF13_0053	0.986988	0.801068	0.002368	-0.53748	-0.60551
aPF13_0053_0	PF13_0053	0.986988	0.801068	0.002368	-0.53748	-0.60551
aPF13_0054_0	PF13_0054	-0.72069	-0.53059	0.879673	1.080993	-0.10475
intPF13_0055_2	PF13_0055					
aPF13_0055_0	PF13_0055					
intPF13_0056_2	PF13_0056	0.70014	0.62731	0.45289	-0.38989	-1.23031
aPF13_0056_1	PF13_0056	0.70014	0.62731	0.45289	-0.38989	-1.23031
aPF13_0056_0	PF13_0056	0.70014	0.62731	0.45289	-0.38989	-1.23031
intPF13_0058_2	PF13_0058	1.3264	-0.1	-2.36649	-2.57051	-0.026
aPF13_0058_0	PF13_0058	1.3264	-0.1	-2.36649	-2.57051	-0.026
intPF13_0059_3	PF13_0059	-0.21244	0.06318	0.65763	0.53837	-0.28518
aPF13_0059_0	PF13_0059	-0.21244	0.06318	0.65763	0.53837	-0.28518
aPF13_0060_1	PF13_0060	1.603873	1.405653	0.038693	-1.10503	-1.32599
aPF13_0060_0	PF13_0060	1.603873	1.405653	0.038693	-1.10503	-1.32599
intMAL13P1.54_2	PF13_0061	-0.15967	0.40033	0.53458	0.21242	-0.34133
aPF13_0061_0	PF13_0061	-0.19368	-0.35559	0.14217	0.42383	0.20259
intPF13_0062_2	PF13_0062	-0.70911	-0.11795	0.20116	0.42534	0.42695
aPF13_0062_1	PF13_0062	-0.70911	-0.11795	0.20116	0.42534	0.42695
aPF13_0062_0	PF13_0062	-0.70911	-0.11795	0.20116	0.42534	0.42695
intPF13_0063_2	PF13_0063	-1.0446	-0.47903	-0.13796	0.432623	0.930693
aPF13_0063_1	PF13_0063	-1.0446	-0.47903	-0.13796	0.432623	0.930693
aPF13_0063_0	PF13_0063	-1.0446	-0.47903	-0.13796	0.432623	0.930693
aPF13_0064_1	PF13_0064	0.510183	0.497583	-0.20818	-0.67816	-0.47392
aPF13_0064_0	PF13_0064	0.510183	0.497583	-0.20818	-0.67816	-0.47392
intPF13_0065_2	PF13_0065	-0.58508	0.025408	0.398218	0.445498	0.150158
aPF13_0065_1	PF13_0065	-0.58508	0.025408	0.398218	0.445498	0.150158
aPF13_0065_0	PF13_0065	-0.58508	0.025408	0.398218	0.445498	0.150158
intPF13_0066_2	PF13_0066	-0.65876	-0.41127	-0.28201	0.217648	0.788908
aPF13_0066_1	PF13_0066	-0.65876	-0.41127	-0.28201	0.217648	0.788908
aPF13_0066_0	PF13_0066	-0.65876	-0.41127	-0.28201	0.217648	0.788908

intMAL13P1.57_2	PF13_0067	-0.0329	1.18152	0.33995	-0.38395	-0.40052
aPF13_0067_1	PF13_0067	-0.66237	-0.96968	-1.20765	-0.07069	1.682343
aPF13_0067_0	PF13_0067	-0.66237	-0.96968	-1.20765	-0.07069	1.682343
intPF13_0069_2	PF13_0068	0.405653	0.115233	0.312083	0.148183	-0.40827
aPF13_0068_0	PF13_0068					
aPF13_0069_1	PF13_0069	0.405653	0.115233	0.312083	0.148183	-0.40827
aPF13_0069_0	PF13_0069	0.405653	0.115233	0.312083	0.148183	-0.40827
intPF13_0070_2	PF13_0070	-0.16299	-0.23204	-0.88642	-0.41624	0.900377
aPF13_0070_1	PF13_0070	-0.16299	-0.23204	-0.88642	-0.41624	0.900377
aPF13_0070_0	PF13_0070	-0.16299	-0.23204	-0.88642	-0.41624	0.900377
intPF13_0071_2	PF13_0071	-1.33129	-0.86805	1.2396	1.4324	-0.25895
aPF13_0071_1	PF13_0071	-1.33129	-0.86805	1.2396	1.4324	-0.25895
aPF13_0071_0	PF13_0071	-1.33129	-0.86805	1.2396	1.4324	-0.25895
intPF13_0072_2	PF13_0072	-0.43097	-0.79162	-1.217	-0.48841	1.114457
aPF13_0072_3	PF13_0072	-0.43097	-0.79162	-1.217	-0.48841	1.114457
aPF13_0072_2	PF13_0072	-0.43097	-0.79162	-1.217	-0.48841	1.114457
aPF13_0072_1	PF13_0072	-0.43097	-0.79162	-1.217	-0.48841	1.114457
aPF13_0072_0	PF13_0072	-0.43097	-0.79162	-1.217	-0.48841	1.114457
intPF13_0073_3	PF13_0073	-1.01398	1.952863	2.364773	0.394893	-2.1032
aPF13_0073_0	PF13_0073	-1.01398	1.952863	2.364773	0.394893	-2.1032
intPF13_0075_2	PF13_0075					
intPF13_0074_2	PF13_0075	0.8892	0.77533	-0.27699	-0.8039	-0.47755
aPF13_0074_2	PF13_0075	0.8892	0.77533	-0.27699	-0.8039	-0.47755
aPF13_0074_1	PF13_0075	0.8892	0.77533	-0.27699	-0.8039	-0.47755
aPF13_0074_0	PF13_0075	0.8892	0.77533	-0.27699	-0.8039	-0.47755
intPF13_0076_2	PF13_0076	1.23349	1.23856	0.63438	-0.89738	-2.09456
aPF13_0076_0	PF13_0076	1.23349	1.23856	0.63438	-0.89738	-2.09456
aPF13_0077_1	PF13_0077	-0.83465	0.048747	0.240267	0.129567	0.064587
aPF13_0077_0	PF13_0077	-0.83465	0.048747	0.240267	0.129567	0.064587
intPF13_0078_2	PF13_0078	0.319382	-0.50256	-1.92372	-1.27762	1.168892
intPF13_0077_2	PF13_0078	-0.83465	0.048747	0.240267	0.129567	0.064587
aPF13_0078_2	PF13_0078	0.319382	-0.50256	-1.92372	-1.27762	1.168892
aPF13_0078_1	PF13_0078	0.319382	-0.50256	-1.92372	-1.27762	1.168892
aPF13_0078_0	PF13_0078	0.319382	-0.50256	-1.92372	-1.27762	1.168892
intPF13_0079_2	PF13_0079	-0.17564	-0.33579	-1.88767	-1.17627	1.531008
aPF13_0079_3	PF13_0079	-0.17564	-0.33579	-1.88767	-1.17627	1.531008
aPF13_0079_2	PF13_0079	-0.17564	-0.33579	-1.88767	-1.17627	1.531008
aPF13_0079_1	PF13_0079	-0.17564	-0.33579	-1.88767	-1.17627	1.531008
aPF13_0079_0	PF13_0079	-0.17564	-0.33579	-1.88767	-1.17627	1.531008
intPF13_0080_2	PF13_0080	-1.3562	-0.84751	0.15752	0.9337	0.99882
aPF13_0080_3	PF13_0080	-1.3562	-0.84751	0.15752	0.9337	0.99882
aPF13_0080_2	PF13_0080	-1.3562	-0.84751	0.15752	0.9337	0.99882
aPF13_0080_1	PF13_0080	-1.3562	-0.84751	0.15752	0.9337	0.99882
aPF13_0080_0	PF13_0080	-1.3562	-0.84751	0.15752	0.9337	0.99882
intMAL13P1.74_2	PF13_0081	-0.95471	-0.42796	0.333413	0.709413	0.485793
aPF13_0081_0	PF13_0081	-0.38514	-0.2763	0.203107	0.483227	0.296637
intPF13_0083_2	PF13_0082	0.724503	0.744993	0.792523	0.136643	-0.99283

intPF13_0081_2	PF13_0082	-0.38514	-0.2763	0.203107	0.483227	0.296637
aPF13_0082_0	PF13_0082	-0.56777	0.377833	0.387843	0.263823	0.125833
intPF13_0082_2	PF13_0083	-0.56777	0.377833	0.387843	0.263823	0.125833
aPF13_0083_1	PF13_0083	0.724503	0.744993	0.792523	0.136643	-0.99283
aPF13_0083_0	PF13_0083	0.724503	0.744993	0.792523	0.136643	-0.99283
intPF13_0084_2	PF13_0084	1.022457	0.724697	0.161607	-0.50227	-0.90736
aPF13_0084_0	PF13_0084	1.022457	0.724697	0.161607	-0.50227	-0.90736
intPF13_0085_2	PF13_0085	0.85199	0.18304	-0.25408	-0.55492	-0.52854
aPF13_0085_1	PF13_0085	0.85199	0.18304	-0.25408	-0.55492	-0.52854
aPF13_0085_0	PF13_0085	0.85199	0.18304	-0.25408	-0.55492	-0.52854
intPF13_0086_2	PF13_0086	-0.4277	-0.83597	-1.25529	-0.25187	1.508807
intMAL13P1.78_2	PF13_0086	-0.88018	0.027363	0.641273	0.381893	-0.2912
aPF13_0086_1	PF13_0086	-0.4277	-0.83597	-1.25529	-0.25187	1.508807
aPF13_0086_0	PF13_0086	-0.4277	-0.83597	-1.25529	-0.25187	1.508807
aPF13_0087_0	PF13_0087	0.374263	0.599673	0.747163	0.074503	-0.98301
intPF13_0088_2	PF13_0088	0.41362	0.1589	0.09944	-0.04544	-0.2824
aPF13_0088_1	PF13_0088	0.41362	0.1589	0.09944	-0.04544	-0.2824
aPF13_0088_0	PF13_0088	0.41362	0.1589	0.09944	-0.04544	-0.2824
intPF13_0089_2	PF13_0089	0.058942	0.130962	-0.43726	-0.21174	0.551702
aPF13_0089_2	PF13_0089	0.058942	0.130962	-0.43726	-0.21174	0.551702
aPF13_0089_1	PF13_0089	0.058942	0.130962	-0.43726	-0.21174	0.551702
aPF13_0089_0	PF13_0089	0.058942	0.130962	-0.43726	-0.21174	0.551702
intPF13_0090_2	PF13_0090	0.687917	-0.00358	-0.23133	-0.85233	-1.08608
aPF13_0090_0	PF13_0090	0.687917	-0.00358	-0.23133	-0.85233	-1.08608
intMAL13P1.85_2	PF13_0090a	0.152887	-0.07542	-0.12015	0.114487	0.290757
intPF13_0091_2	PF13_0091	0.650597	0.391507	0.545497	0.056837	-0.87067
aPF13_0091_1	PF13_0091	0.650597	0.391507	0.545497	0.056837	-0.87067
aPF13_0091_0	PF13_0091	0.650597	0.391507	0.545497	0.056837	-0.87067
intPF13_0093_2	PF13_0093	-0.18711	0.49006	0.48664	0.19636	-0.20856
aPF13_0093_1	PF13_0093	-0.18711	0.49006	0.48664	0.19636	-0.20856
aPF13_0093_0	PF13_0093	-0.18711	0.49006	0.48664	0.19636	-0.20856
intPF13_0095_2	PF13_0095	-1.59638	-1.6781	-0.56231	1.012973	1.963763
aPF13_0095_1	PF13_0095	-1.59638	-1.6781	-0.56231	1.012973	1.963763
aPF13_0095_0	PF13_0095	-1.59638	-1.6781	-0.56231	1.012973	1.963763
intPF13_0096_2	PF13_0096	0.380437	0.361317	0.046367	-0.20003	-0.27098
aPF13_0096_1	PF13_0096	0.380437	0.361317	0.046367	-0.20003	-0.27098
aPF13_0096_0	PF13_0096	0.380437	0.361317	0.046367	-0.20003	-0.27098
intPF13_0097_2	PF13_0097	-1.19825	-0.30174	1.250977	1.116857	-0.41992
aPF13_0097_3	PF13_0097	-1.19825	-0.30174	1.250977	1.116857	-0.41992
aPF13_0097_2	PF13_0097	-1.19825	-0.30174	1.250977	1.116857	-0.41992
aPF13_0097_1	PF13_0097	-1.19825	-0.30174	1.250977	1.116857	-0.41992
aPF13_0097_0	PF13_0097	-1.19825	-0.30174	1.250977	1.116857	-0.41992
aPF13_0098_0	PF13_0098	-1.03639	-0.19406	0.597113	0.823553	0.356723
intPF13_0099_2	PF13_0099	-1.09325	-0.24477	1.01504	1.05696	-0.07423
intPF13_0098_2	PF13_0099	-1.03639	-0.19406	0.597113	0.823553	0.356723
aPF13_0099_0	PF13_0099	-1.09325	-0.24477	1.01504	1.05696	-0.07423
aPF13_0100_1	PF13_0100	-0.547	-0.66767	-0.00019	0.52183	0.52031

aPF13_0100_0	PF13_0100	-0.547	-0.66767	-0.00019	0.52183	0.52031
aPF13_0101_2	PF13_0101	-1.00993	0.40298	0.71821	0.38734	-0.13042
aPF13_0101_1	PF13_0101	-1.00993	0.40298	0.71821	0.38734	-0.13042
aPF13_0101_0	PF13_0101	-1.00993	0.40298	0.71821	0.38734	-0.13042
intPF13_0102_2	PF13_0102	0.110563	0.086663	0.049923	-0.15726	-0.3065
aPF13_0102_1	PF13_0102	0.110563	0.086663	0.049923	-0.15726	-0.3065
aPF13_0102_0	PF13_0102	0.110563	0.086663	0.049923	-0.15726	-0.3065
intPF13_0103_2	PF13_0103	0.977433	0.380353	-0.54722	-0.93712	-0.47469
aPF13_0103_1	PF13_0103	0.977433	0.380353	-0.54722	-0.93712	-0.47469
aPF13_0103_0	PF13_0103	0.977433	0.380353	-0.54722	-0.93712	-0.47469
intPF13_0104_2	PF13_0104	-0.4347	-0.74099	-0.10802	0.52802	0.64099
aPF13_0104_1	PF13_0104	-0.4347	-0.74099	-0.10802	0.52802	0.64099
aPF13_0104_0	PF13_0104	-0.4347	-0.74099	-0.10802	0.52802	0.64099
intPF13_0106_3	PF13_0106	1.205783	0.563073	-0.64865	-1.15269	-0.56641
aPF13_0106_1	PF13_0106	1.205783	0.563073	-0.64865	-1.15269	-0.56641
aPF13_0106_0	PF13_0106	1.205783	0.563073	-0.64865	-1.15269	-0.56641
intPF13_0107_2	PF13_0107	0.40877	0.42063	0.03474	-0.22074	-0.25063
aPF13_0107_1	PF13_0107	0.40877	0.42063	0.03474	-0.22074	-0.25063
aPF13_0107_0	PF13_0107	0.40877	0.42063	0.03474	-0.22074	-0.25063
intPF13_0109_2	PF13_0109	0.529597	0.688007	0.604747	-0.23041	-1.17217
aPF13_0109_1	PF13_0109	0.529597	0.688007	0.604747	-0.23041	-1.17217
aPF13_0109_0	PF13_0109	0.529597	0.688007	0.604747	-0.23041	-1.17217
intPF13_0112_2	PF13_0112	-0.51931	-0.25047	0.61744	0.71756	-0.04953
aPF13_0112_0	PF13_0112	-0.51931	-0.25047	0.61744	0.71756	-0.04953
intPF13_0114_2	PF13_0114	-0.86968	-0.37509	-0.87614	-0.20696	1.335647
aPF13_0114_2	PF13_0114	-0.86968	-0.37509	-0.87614	-0.20696	1.335647
aPF13_0114_1	PF13_0114	-0.86968	-0.37509	-0.87614	-0.20696	1.335647
aPF13_0114_0	PF13_0114	-0.86968	-0.37509	-0.87614	-0.20696	1.335647
intPF13_0116_3	PF13_0116	0.413577	2.129127	0.040757	-1.39442	-0.88479
aPF13_0116_1	PF13_0116	0.413577	2.129127	0.040757	-1.39442	-0.88479
aPF13_0116_0	PF13_0116	0.413577	2.129127	0.040757	-1.39442	-0.88479
intMAL13P1.111_2	PF13_0117	0.238847	-0.31122	-0.35704	-0.02862	0.324557
aPF13_0117_1	PF13_0117	-0.00414	0.01668	-0.10337	-0.00113	0.20032
aPF13_0117_0	PF13_0117	-0.00414	0.01668	-0.10337	-0.00113	0.20032
intMAL13P1.113_2	PF13_0118	-1.44785	0.45623	0.89505	0.63195	0.09377
intMAL13P1.112_2	PF13_0118	-1.38142	-0.0294	-0.08793	0.212767	0.921237
aPF13_0118_1	PF13_0118	-1.17927	-0.22961	0.509727	0.714607	0.377447
aPF13_0118_0	PF13_0118	-1.17927	-0.22961	0.509727	0.714607	0.377447
intPF13_0119_2	PF13_0119	0.13837	0.09613	0.21723	-0.09323	-0.52013
aPF13_0119_0	PF13_0119	0.13837	0.09613	0.21723	-0.09323	-0.52013
intPF13_0120_2	PF13_0120	-0.39501	-0.54927	-1.68312	-0.7401	1.741048
aPF13_0120_2	PF13_0120	-0.39501	-0.54927	-1.68312	-0.7401	1.741048
aPF13_0120_1	PF13_0120	-0.39501	-0.54927	-1.68312	-0.7401	1.741048
aPF13_0120_0	PF13_0120	-0.39501	-0.54927	-1.68312	-0.7401	1.741048
intPF13_0121_2	PF13_0121	-0.34339	-1.04005	0.22284	0.86366	0.30805
aPF13_0121_1	PF13_0121	-0.34339	-1.04005	0.22284	0.86366	0.30805
aPF13_0121_0	PF13_0121	-0.34339	-1.04005	0.22284	0.86366	0.30805

aPF13_0122_0	PF13_0122	1.269393	1.097053	0.227893	-0.49123	-0.84639
intPF13_0123_2	PF13_0123	0.12742	0.45447	0.48177	0.10319	-0.46795
intPF13_0122_2	PF13_0123	1.269393	1.097053	0.227893	-0.49123	-0.84639
aPF13_0123_1	PF13_0123	0.12742	0.45447	0.48177	0.10319	-0.46795
aPF13_0123_0	PF13_0123	0.12742	0.45447	0.48177	0.10319	-0.46795
intPF13_0124_3	PF13_0124	0.219888	0.756808	0.429268	-0.3131	-0.82539
aPF13_0124_0	PF13_0124	0.219888	0.756808	0.429268	-0.3131	-0.82539
intPF13_0125_2	PF13_0125	0.5044	0.02498	0.18405	-0.45405	-1.16998
aPF13_0125_0	PF13_0125	0.5044	0.02498	0.18405	-0.45405	-1.16998
aPF13_0126_2	PF13_0126	-0.82414	-0.67864	0.216435	0.816455	0.650185
aPF13_0126_1	PF13_0126	-0.82414	-0.67864	0.216435	0.816455	0.650185
aPF13_0126_0	PF13_0126	-0.82414	-0.67864	0.216435	0.816455	0.650185
aPF13_0127_1	PF13_0127	-0.17687	-0.62114	-1.79896	-0.84254	1.67914
aPF13_0127_0	PF13_0127	-0.17687	-0.62114	-1.79896	-0.84254	1.67914
aPF13_0128_0	PF13_0128	0.20835	0.72427	0.21245	-0.29945	-0.42527
intPF13_0129_2	PF13_0129	-0.05069	0.37943	0.76913	0.44387	-0.47443
intPF13_0128_2	PF13_0129	0.20835	0.72427	0.21245	-0.29945	-0.42527
aPF13_0129_0	PF13_0129	-0.05069	0.37943	0.76913	0.44387	-0.47443
intPF13_0131_2	PF13_0130	-0.73071	-0.78045	-0.2756	0.5146	1.00045
intPF13_0130_2	PF13_0130	-0.30017	0.01885	0.24179	0.36721	0.22015
aPF13_0130_0	PF13_0130	-0.30017	0.01885	0.24179	0.36721	0.22015
aPF13_0131_0	PF13_0131	-0.73071	-0.78045	-0.2756	0.5146	1.00045
intPF13_0132_2	PF13_0132	0.143877	0.437597	0.655807	0.323527	-0.47326
aPF13_0132_0	PF13_0132	0.143877	0.437597	0.655807	0.323527	-0.47326
intPF13_0133_2	PF13_0133	-0.98947	-0.11267	0.33684	0.55866	0.47517
aPF13_0133_1	PF13_0133	-0.98947	-0.11267	0.33684	0.55866	0.47517
aPF13_0133_0	PF13_0133	-0.98947	-0.11267	0.33684	0.55866	0.47517
intPF13_0134_2	PF13_0134	-0.38354	-0.8343	0.25735	0.78065	0.2708
aPF13_0134_1	PF13_0134	-0.38354	-0.8343	0.25735	0.78065	0.2708
aPF13_0134_0	PF13_0134	-0.38354	-0.8343	0.25735	0.78065	0.2708
intPF13_0135_3	PF13_0135	0.29813	0.71987	0.03327	-0.30827	-0.14287
aPF13_0135_1	PF13_0135	0.29813	0.71987	0.03327	-0.30827	-0.14287
aPF13_0135_0	PF13_0135	0.29813	0.71987	0.03327	-0.30827	-0.14287
intPF13_0136_2	PF13_0136	0.325823	0.643873	0.286003	-0.17934	-0.47271
aPF13_0136_1	PF13_0136	0.325823	0.643873	0.286003	-0.17934	-0.47271
aPF13_0136_0	PF13_0136	0.325823	0.643873	0.286003	-0.17934	-0.47271
intPF13_0137_2	PF13_0137	0.978	1.32568	0.46397	-0.66175	-1.28546
aPF13_0137_2	PF13_0137	0.978	1.32568	0.46397	-0.66175	-1.28546
aPF13_0137_1	PF13_0137	0.978	1.32568	0.46397	-0.66175	-1.28546
aPF13_0137_0	PF13_0137	0.978	1.32568	0.46397	-0.66175	-1.28546
intPF13_0138_3	PF13_0138	1.054877	0.811597	0.063807	-0.71447	-0.99926
aPF13_0138_0	PF13_0138	1.054877	0.811597	0.063807	-0.71447	-0.99926
intMAL13P1.134_2	PF13_0139	-0.64691	-0.78246	-0.02209	0.698753	0.782623
aPF13_0139_2	PF13_0139	-0.8338	-0.64968	0.006515	0.655265	0.815785
aPF13_0139_1	PF13_0139	-0.8338	-0.64968	0.006515	0.655265	0.815785
aPF13_0139_0	PF13_0139	-0.8338	-0.64968	0.006515	0.655265	0.815785
intPF13_0140_2	PF13_0140	-0.21407	-0.58217	-0.15993	0.460593	0.667333

aPF13_0140_1	PF13_0140	-0.21407	-0.58217	-0.15993	0.460593	0.667333
aPF13_0140_0	PF13_0140	-0.21407	-0.58217	-0.15993	0.460593	0.667333
intPF13_0141_2	PF13_0141	-0.74067	0.22633	0.96783	0.87617	-0.06233
aPF13_0141_0	PF13_0141	-0.74067	0.22633	0.96783	0.87617	-0.06233
intPF13_0142_2	PF13_0142	0.590263	0.425673	0.399163	0.020503	-0.61401
aPF13_0142_0	PF13_0142	0.590263	0.425673	0.399163	0.020503	-0.61401
intPF13_0143_3	PF13_0143	0.234103	0.178993	0.567263	0.263903	-0.60283
aPF13_0143_1	PF13_0143	0.234103	0.178993	0.567263	0.263903	-0.60283
aPF13_0143_0	PF13_0143	0.234103	0.178993	0.567263	0.263903	-0.60283
intPF13_0144_2	PF13_0144	-0.09582	0.190127	0.050247	-0.10691	-0.09379
aPF13_0144_0	PF13_0144	-0.09582	0.190127	0.050247	-0.10691	-0.09379
intPF13_0146_2	PF13_0146	-0.75137	0.630823	1.364603	0.557063	-0.99516
aPF13_0146_2	PF13_0146	-0.75137	0.630823	1.364603	0.557063	-0.99516
aPF13_0146_1	PF13_0146	-0.75137	0.630823	1.364603	0.557063	-0.99516
aPF13_0146_0	PF13_0146	-0.75137	0.630823	1.364603	0.557063	-0.99516
intPF13_0147_2	PF13_0147	0.01303	0.03736	0.31453	0.28447	-0.12936
aPF13_0147_1	PF13_0147	0.01303	0.03736	0.31453	0.28447	-0.12936
aPF13_0147_0	PF13_0147	0.01303	0.03736	0.31453	0.28447	-0.12936
intPF13_0148_2	PF13_0148	-1.10644	-1.04855	-0.18554	0.73856	1.11778
aPF13_0148_7	PF13_0148	-1.10644	-1.04855	-0.18554	0.73856	1.11778
aPF13_0148_6	PF13_0148	-1.10644	-1.04855	-0.18554	0.73856	1.11778
aPF13_0148_5	PF13_0148	-1.10644	-1.04855	-0.18554	0.73856	1.11778
aPF13_0148_4	PF13_0148	-1.10644	-1.04855	-0.18554	0.73856	1.11778
aPF13_0148_3	PF13_0148	-1.10644	-1.04855	-0.18554	0.73856	1.11778
aPF13_0148_2	PF13_0148	-1.10644	-1.04855	-0.18554	0.73856	1.11778
aPF13_0148_1	PF13_0148	-1.10644	-1.04855	-0.18554	0.73856	1.11778
aPF13_0148_0	PF13_0148	-1.10644	-1.04855	-0.18554	0.73856	1.11778
intPF13_0149_2	PF13_0149	0.11168	-0.77391	-0.65717	0.21317	0.97141
aPF13_0149_1	PF13_0149	0.11168	-0.77391	-0.65717	0.21317	0.97141
aPF13_0149_0	PF13_0149	0.11168	-0.77391	-0.65717	0.21317	0.97141
intPF13_0151_2	PF13_0150	-0.06656	-0.01168	-0.08013	0.039467	0.220517
intPF13_0150_2	PF13_0150	0.820998	0.665168	0.495458	-0.29376	-1.17922
aPF13_0150_3	PF13_0150	0.820998	0.665168	0.495458	-0.29376	-1.17922
aPF13_0150_1	PF13_0150	0.820998	0.665168	0.495458	-0.29376	-1.17922
aPF13_0150_0	PF13_0150	0.820998	0.665168	0.495458	-0.29376	-1.17922
aPF13_0151_1	PF13_0151	-0.06656	-0.01168	-0.08013	0.039467	0.220517
aPF13_0151_0	PF13_0151	-0.06656	-0.01168	-0.08013	0.039467	0.220517
intPF13_0152_2	PF13_0152	0.38675	0.32927	-0.51304	-0.67296	0.02173
aPF13_0152_0	PF13_0152	0.38675	0.32927	-0.51304	-0.67296	0.02173
intPF13_0153_3	PF13_0153	-0.43319	-0.22208	0.220153	-0.00999	-0.42226
aPF13_0153_1	PF13_0153	-0.43319	-0.22208	0.220153	-0.00999	-0.42226
aPF13_0153_0	PF13_0153	-0.43319	-0.22208	0.220153	-0.00999	-0.42226
intPF13_0154_2	PF13_0154	-1.17265	-1.08021	-0.19131	0.669942	1.026392
aPF13_0154_0	PF13_0154	-1.17265	-1.08021	-0.19131	0.669942	1.026392
intPF13_0155_2	PF13_0155	-0.63145	-0.71226	-0.99187	-0.04889	1.480755
aPF13_0155_3	PF13_0155	-0.63145	-0.71226	-0.99187	-0.04889	1.480755
aPF13_0155_2	PF13_0155	-0.63145	-0.71226	-0.99187	-0.04889	1.480755

aPF13_0155_1	PF13_0155	-0.63145	-0.71226	-0.99187	-0.04889	1.480755
aPF13_0155_0	PF13_0155	-0.63145	-0.71226	-0.99187	-0.04889	1.480755
intPF13_0156_2	PF13_0156	-0.8481	-0.52051	0.067503	0.688163	0.844173
aPF13_0156_0	PF13_0156	-0.8481	-0.52051	0.067503	0.688163	0.844173
intPF13_0158_2	PF13_0157					
intPF13_0157_2	PF13_0157	0.505233	0.488343	0.628823	0.104343	-0.83918
aPF13_0157_1	PF13_0157	0.505233	0.488343	0.628823	0.104343	-0.83918
aPF13_0157_0	PF13_0157	0.505233	0.488343	0.628823	0.104343	-0.83918
aPF13_0158_0	PF13_0158					
intPF13_0159_2	PF13_0159	0.471187	-0.10693	0.249867	-0.26953	-1.09073
aPF13_0159_0	PF13_0159	0.471187	-0.10693	0.249867	-0.26953	-1.09073
aPF13_0160_0	PF13_0160	-1.05155	-1.76525	-0.95752	0.605183	1.836913
intPF13_0161_2	PF13_0161	1.269273	0.472963	-1.1361	-2.05824	-1.1123
aPF13_0161_2	PF13_0161	1.269273	0.472963	-1.1361	-2.05824	-1.1123
aPF13_0161_1	PF13_0161	1.269273	0.472963	-1.1361	-2.05824	-1.1123
aPF13_0161_0	PF13_0161	1.269273	0.472963	-1.1361	-2.05824	-1.1123
intPF13_0162_2	PF13_0162	1.92989	1.04554	-0.24783	-1.54123	-1.80376
aPF13_0162_2	PF13_0162	1.92989	1.04554	-0.24783	-1.54123	-1.80376
aPF13_0162_1	PF13_0162	1.92989	1.04554	-0.24783	-1.54123	-1.80376
aPF13_0162_0	PF13_0162	1.92989	1.04554	-0.24783	-1.54123	-1.80376
intPF13_0163_2	PF13_0163	0.954373	0.204113	0.039273	-0.50961	-1.01845
aPF13_0163_0	PF13_0163	0.954373	0.204113	0.039273	-0.50961	-1.01845
intPF13_0164_2	PF13_0164	-0.44552	-0.57886	-0.84827	-0.12239	1.134197
aPF13_0164_1	PF13_0164	-0.44552	-0.57886	-0.84827	-0.12239	1.134197
aPF13_0164_0	PF13_0164	-0.44552	-0.57886	-0.84827	-0.12239	1.134197
intPF13_0165_2	PF13_0165	0.310923	0.184373	0.794993	0.143673	-1.22271
aPF13_0165_1	PF13_0165	0.310923	0.184373	0.794993	0.143673	-1.22271
aPF13_0165_0	PF13_0165	0.310923	0.184373	0.794993	0.143673	-1.22271
intPF13_0166_2	PF13_0166	0.796847	-0.02324	-0.77	-1.02066	-0.40042
aPF13_0166_1	PF13_0166	0.796847	-0.02324	-0.77	-1.02066	-0.40042
aPF13_0166_0	PF13_0166	0.796847	-0.02324	-0.77	-1.02066	-0.40042
aPF13_0167_1	PF13_0167	0.599077	0.409117	0.270027	-0.21119	-0.73878
aPF13_0167_0	PF13_0167	0.599077	0.409117	0.270027	-0.21119	-0.73878
aPF13_0168_1	PF13_0168	-0.12102	-0.18527	-0.77939	-0.43336	0.695648
aPF13_0168_0	PF13_0168	-0.12102	-0.18527	-0.77939	-0.43336	0.695648
intPF13_0169_2	PF13_0169	0.62673	-0.14316	-0.51518	-0.86732	-0.66084
aPF13_0169_1	PF13_0169	0.62673	-0.14316	-0.51518	-0.86732	-0.66084
aPF13_0169_0	PF13_0169	0.62673	-0.14316	-0.51518	-0.86732	-0.66084
intPF13_0170_3	PF13_0170	-0.13759	0.486913	0.519663	0.155003	-0.34975
intMAL13P1.168_2	PF13_0170	-0.80793	-2.06236	-1.01279	0.94479	2.16836
aPF13_0170_1	PF13_0170	-0.13759	0.486913	0.519663	0.155003	-0.34975
aPF13_0170_0	PF13_0170	-0.13759	0.486913	0.519663	0.155003	-0.34975
intPF13_0171_2	PF13_0171	0.1192	0.44799	0.88902	0.47198	-0.66299
aPF13_0171_0	PF13_0171	0.1192	0.44799	0.88902	0.47198	-0.66299
intPF13_0172_2	PF13_0172	0.26698	0.26612	0.06076	-0.31076	-0.48912
aPF13_0172_1	PF13_0172	0.26698	0.26612	0.06076	-0.31076	-0.48912
aPF13_0172_0	PF13_0172	0.26698	0.26612	0.06076	-0.31076	-0.48912

intPF13_0174_2	PF13_0173	-0.35272	-0.11341	0.21032	0.05268	-0.26459
intPF13_0173_2	PF13_0173	0.458667	0.001167	-1.86533	-1.91233	0.399167
aPF13_0173_1	PF13_0173	0.458667	0.001167	-1.86533	-1.91233	0.399167
aPF13_0173_0	PF13_0173	0.458667	0.001167	-1.86533	-1.91233	0.399167
aPF13_0174_0	PF13_0174	-0.35272	-0.11341	0.21032	0.05268	-0.26459
intPF13_0175_3	PF13_0175	0.727923	0.836603	0.307643	-0.46408	-0.94504
aPF13_0175_0	PF13_0175	0.727923	0.836603	0.307643	-0.46408	-0.94504
intPF13_0176_2	PF13_0176	-0.3637	-0.24101	-0.28648	-0.06452	0.38901
aPF13_0176_1	PF13_0176	-0.3637	-0.24101	-0.28648	-0.06452	0.38901
aPF13_0176_0	PF13_0176	-0.3637	-0.24101	-0.28648	-0.06452	0.38901
intPF13_0177_2	PF13_0177	0.527133	0.679843	0.861063	-0.0124	-1.31968
aPF13_0177_1	PF13_0177	0.527133	0.679843	0.861063	-0.0124	-1.31968
aPF13_0177_0	PF13_0177	0.527133	0.679843	0.861063	-0.0124	-1.31968
intPF13_0178_2	PF13_0178	0.673863	0.671693	0.934113	0.111553	-1.32203
aPF13_0178_0	PF13_0178	0.673863	0.671693	0.934113	0.111553	-1.32203
intPF13_0180_2	PF13_0179	-0.83201	-0.04194	1.000377	0.923957	-0.19872
intPF13_0179_3	PF13_0179	-0.06555	0.505733	0.714023	0.365643	-0.40157
aPF13_0179_1	PF13_0179	-0.06555	0.505733	0.714023	0.365643	-0.40157
aPF13_0179_0	PF13_0179	-0.06555	0.505733	0.714023	0.365643	-0.40157
aPF13_0180_0	PF13_0180	-0.83201	-0.04194	1.000377	0.923957	-0.19872
intPF13_0181_2	PF13_0181	0.514113	0.192433	0.050383	-0.12422	-0.32177
aPF13_0181_1	PF13_0181	0.514113	0.192433	0.050383	-0.12422	-0.32177
aPF13_0181_0	PF13_0181	0.514113	0.192433	0.050383	-0.12422	-0.32177
intPF13_0182_2	PF13_0182	-0.72777	-0.12113	-0.03557	0.276348	0.641578
aPF13_0182_2	PF13_0182	-0.72777	-0.12113	-0.03557	0.276348	0.641578
aPF13_0182_1	PF13_0182	-0.72777	-0.12113	-0.03557	0.276348	0.641578
aPF13_0182_0	PF13_0182	-0.72777	-0.12113	-0.03557	0.276348	0.641578
aPF13_0183_1	PF13_0183	-0.10419	-0.44453	0.79481	0.89219	-0.38047
aPF13_0183_0	PF13_0183	-0.10419	-0.44453	0.79481	0.89219	-0.38047
intPF13_0184_2	PF13_0184	0.8413	0.77249	0.82502	-0.24902	-1.65149
aPF13_0184_1	PF13_0184	0.8413	0.77249	0.82502	-0.24902	-1.65149
aPF13_0184_0	PF13_0184	0.8413	0.77249	0.82502	-0.24902	-1.65149
intPF13_0185_2	PF13_0185	-0.12251	-1.17742	-0.61616	0.123497	0.570757
aPF13_0185_0	PF13_0185	-0.12251	-1.17742	-0.61616	0.123497	0.570757
intPF13_0186_2	PF13_0186	0.754337	-0.0517	0.002417	-0.12308	-0.47496
aPF13_0186_0	PF13_0186	0.754337	-0.0517	0.002417	-0.12308	-0.47496
intPF13_0187_2	PF13_0187	-0.8852	-0.86497	-0.01579	0.747627	0.868807
aPF13_0187_1	PF13_0187	-0.8852	-0.86497	-0.01579	0.747627	0.868807
aPF13_0187_0	PF13_0187	-0.8852	-0.86497	-0.01579	0.747627	0.868807
intPF13_0188_2	PF13_0188	-0.53031	-1.18345	-0.6461	0.4621	1.26545
aPF13_0188_1	PF13_0188	-0.53031	-1.18345	-0.6461	0.4621	1.26545
aPF13_0188_0	PF13_0188	-0.53031	-1.18345	-0.6461	0.4621	1.26545
intPF13_0189_2	PF13_0189	-0.8226	-0.73148	-0.19303	0.56303	0.98498
aPF13_0189_1	PF13_0189	-0.8226	-0.73148	-0.19303	0.56303	0.98498
aPF13_0189_0	PF13_0189	-0.8226	-0.73148	-0.19303	0.56303	0.98498
intPF13_0190_2	PF13_0190	0.276927	0.380357	0.997287	0.478047	-0.95002
aPF13_0190_1	PF13_0190	0.276927	0.380357	0.997287	0.478047	-0.95002

aPF13_0190_0	PF13_0190	0.276927	0.380357	0.997287	0.478047	-0.95002
intPF13_0191_2	PF13_0191	-0.91676	-0.00072	0.90694	0.75956	-0.21828
aPF13_0191_1	PF13_0191	-0.91676	-0.00072	0.90694	0.75956	-0.21828
aPF13_0191_0	PF13_0191	-0.91676	-0.00072	0.90694	0.75956	-0.21828
intPF13_0192_2	PF13_0192	-1.10599	-1.00003	0.89931	1.47519	0.26603
aPF13_0193_1	PF13_0192	0.913768	0.569638	-1.73227	-1.41317	1.131248
aPF13_0192_1	PF13_0192	-1.10599	-1.00003	0.89931	1.47519	0.26603
aPF13_0192_0	PF13_0192	-1.10599	-1.00003	0.89931	1.47519	0.26603
intPF13_0193_2	PF13_0193	0.913768	0.569638	-1.73227	-1.41317	1.131248
aPF13_0193_0	PF13_0193	0.913768	0.569638	-1.73227	-1.41317	1.131248
aPF13_0194_0	PF13_0194	1.669527	1.153357	-0.07472	-1.21494	-1.47302
intPF13_0196_2	PF13_0196	0.930815	0.671645	-0.11556	-0.6796	-0.67005
aPF13_0196_1	PF13_0196	0.930815	0.671645	-0.11556	-0.6796	-0.67005
aPF13_0196_0	PF13_0196	0.930815	0.671645	-0.11556	-0.6796	-0.67005
intPF13_0197_2	PF13_0197	-0.2305	-1.4335	-2.8485	-1.21684	2.518163
aPF13_0197_0	PF13_0197	-0.2305	-1.4335	-2.8485	-1.21684	2.518163
intPF13_0198_2	PF13_0198	1.14435	0.08507	-1.84189	-2.26921	-0.35177
aPF13_0198_4	PF13_0198	1.14435	0.08507	-1.84189	-2.26921	-0.35177
intPF13_0200_2	PF13_0199	-0.97727	-0.72667	0.026093	0.668573	0.796333
intPF13_0199_2	PF13_0199	-0.68434	-0.53899	-0.18886	0.412862	0.834322
aPF13_0199_2	PF13_0199	-0.68434	-0.53899	-0.18886	0.412862	0.834322
aPF13_0199_1	PF13_0199	-0.68434	-0.53899	-0.18886	0.412862	0.834322
aPF13_0199_0	PF13_0199	-0.68434	-0.53899	-0.18886	0.412862	0.834322
aPF13_0200_1	PF13_0200	-0.97727	-0.72667	0.026093	0.668573	0.796333
aPF13_0200_0	PF13_0200	-0.97727	-0.72667	0.026093	0.668573	0.796333
intPF13_0201_2	PF13_0201	-0.2221	-0.68446	-0.46082	0.271157	0.869297
aPF13_0201_1	PF13_0201	-0.2221	-0.68446	-0.46082	0.271157	0.869297
aPF13_0201_0	PF13_0201	-0.2221	-0.68446	-0.46082	0.271157	0.869297
aPF13_0202_0	PF13_0202	-0.99392	-1.08737	-0.23874	0.789077	1.227707
intPF13_0203_2	PF13_0203	0.473573	0.017143	0.422713	-0.03405	-0.97248
aPF13_0203_0	PF13_0203	0.473573	0.017143	0.422713	-0.03405	-0.97248
intPF13_0204_2	PF13_0204	-0.15412	0.318573	0.963353	0.528313	-0.70991
aPF13_0204_1	PF13_0204	-0.15412	0.318573	0.963353	0.528313	-0.70991
aPF13_0204_0	PF13_0204	-0.15412	0.318573	0.963353	0.528313	-0.70991
aPF13_0205_1	PF13_0205	0.247553	0.602733	0.618783	0.120383	-0.63457
aPF13_0205_0	PF13_0205	0.247553	0.602733	0.618783	0.120383	-0.63457
intPF13_0208_2	PF13_0208	0.218683	0.659093	0.705803	0.050363	-0.85193
aPF13_0208_1	PF13_0208	0.218683	0.659093	0.705803	0.050363	-0.85193
aPF13_0208_0	PF13_0208	0.218683	0.659093	0.705803	0.050363	-0.85193
intPF13_0209_2	PF13_0209	0.884617	0.808397	0.048707	-0.54987	-0.67006
aPF13_0209_1	PF13_0209	0.884617	0.808397	0.048707	-0.54987	-0.67006
aPF13_0209_0	PF13_0209	0.884617	0.808397	0.048707	-0.54987	-0.67006
intPF13_0210_2	PF13_0210	0.51664	0.46162	-0.40903	-0.52217	0.10218
aPF13_0210_4	PF13_0210	0.51664	0.46162	-0.40903	-0.52217	0.10218
aPF13_0210_3	PF13_0210	0.51664	0.46162	-0.40903	-0.52217	0.10218
aPF13_0210_2	PF13_0210	0.51664	0.46162	-0.40903	-0.52217	0.10218
aPF13_0210_1	PF13_0210	0.51664	0.46162	-0.40903	-0.52217	0.10218

aPF13_0210_0	PF13_0210	0.51664	0.46162	-0.40903	-0.52217	0.10218
intPF13_0211_2	PF13_0211	1.880923	0.593883	-1.13853	-2.33531	-1.64672
aPF13_0211_1	PF13_0211	1.880923	0.593883	-1.13853	-2.33531	-1.64672
aPF13_0211_0	PF13_0211	1.880923	0.593883	-1.13853	-2.33531	-1.64672
intPF13_0212_2	PF13_0212	0.372227	0.014847	-0.0632	-0.14546	-0.21651
aPF13_0212_0	PF13_0212	0.372227	0.014847	-0.0632	-0.14546	-0.21651
intPF13_0213_1	PF13_0213	0.125783	0.347073	0.656353	0.262313	-0.62041
aPF13_0213_0	PF13_0213	0.125783	0.347073	0.656353	0.262313	-0.62041
intPF13_0214_3	PF13_0214	-0.42854	0.196207	0.627837	0.508997	-0.11387
aPF13_0214_1	PF13_0214	-0.42854	0.196207	0.627837	0.508997	-0.11387
aPF13_0214_0	PF13_0214	-0.42854	0.196207	0.627837	0.508997	-0.11387
intPF13_0215_2	PF13_0215	-0.05479	0.184922	0.607282	0.003802	-0.96609
aPF13_0215_0	PF13_0215	-0.05479	0.184922	0.607282	0.003802	-0.96609
aPF13_0216_0	PF13_0216	-0.96972	-1.04737	-0.56076	0.375097	1.215707
intPF13_0218_2	PF13_0217	-0.62915	-0.46778	0.070553	0.503613	0.523943
aPF13_0217_0	PF13_0217	-1.06814	-0.65628	0.161067	0.754267	0.754617
intPF13_0217_2	PF13_0218	-1.06814	-0.65628	0.161067	0.754267	0.754617
aPF13_0218_1	PF13_0218	-0.62915	-0.46778	0.070553	0.503613	0.523943
aPF13_0218_0	PF13_0218	-0.62915	-0.46778	0.070553	0.503613	0.523943
intPF13_0219_2	PF13_0218a	0.820767	1.243677	0.807637	-0.4888	-1.65834
aPF13_0219_1	PF13_0219	0.820767	1.243677	0.807637	-0.4888	-1.65834
aPF13_0219_0	PF13_0219	0.820767	1.243677	0.807637	-0.4888	-1.65834
intPF13_0220_2	PF13_0220	0.226853	-0.09589	0.185713	0.025253	-0.44215
aPF13_0220_1	PF13_0220	0.226853	-0.09589	0.185713	0.025253	-0.44215
aPF13_0220_0	PF13_0220	0.226853	-0.09589	0.185713	0.025253	-0.44215
intPF13_0221_2	PF13_0221	0.20295	0.63774	0.26752	-0.28452	-0.55974
aPF13_0221_1	PF13_0221	0.20295	0.63774	0.26752	-0.28452	-0.55974
aPF13_0221_0	PF13_0221	0.20295	0.63774	0.26752	-0.28452	-0.55974
intPF13_0222_2	PF13_0222	-0.89228	0.166363	0.982283	0.719883	-0.3127
aPF13_0222_1	PF13_0222	-0.89228	0.166363	0.982283	0.719883	-0.3127
aPF13_0222_0	PF13_0222	-0.89228	0.166363	0.982283	0.719883	-0.3127
intPF13_0223_2	PF13_0223	-0.02631	-0.52943	-0.70063	0.020967	0.971767
intMAL13P1.205_2	PF13_0223	-0.19137	-0.66715	-0.78719	0.083527	1.187487
aPF13_0223_0	PF13_0223	-0.02631	-0.52943	-0.70063	0.020967	0.971767
intPF13_0224_2	PF13_0224	0.411843	0.491793	0.856413	0.340253	-0.86913
aPF13_0224_0	PF13_0224	0.411843	0.491793	0.856413	0.340253	-0.86913
intPF13_0225_2	PF13_0225	0.44499	0.08355	-1.61485	-1.45565	0.68445
aPF13_0225_1	PF13_0225	0.44499	0.08355	-1.61485	-1.45565	0.68445
aPF13_0225_0	PF13_0225	0.44499	0.08355	-1.61485	-1.45565	0.68445
intPF13_0226_2	PF13_0226	-0.14556	-0.17767	-1.3784	-1.02776	0.915507
aPF13_0226_1	PF13_0226	-0.14556	-0.17767	-1.3784	-1.02776	0.915507
aPF13_0226_0	PF13_0226	-0.14556	-0.17767	-1.3784	-1.02776	0.915507
intPF13_0227_2	PF13_0227	-0.38723	-0.04087	0.485652	0.534032	0.012502
aPF13_0227_0	PF13_0227	-0.38723	-0.04087	0.485652	0.534032	0.012502
intPF13_0228_3	PF13_0228	0.37505	0.73074	0.08502	-0.36602	-0.34274
aPF13_0228_0	PF13_0228	0.37505	0.73074	0.08502	-0.36602	-0.34274
intPF13_0229_2	PF13_0229	-0.57851	-0.34494	-0.90287	-0.31013	1.15294

aPF13_0229_1	PF13_0229	-0.57851	-0.34494	-0.90287	-0.31013	1.15294
aPF13_0229_0	PF13_0229	-0.57851	-0.34494	-0.90287	-0.31013	1.15294
intPF13_0231_2	PF13_0230	0.1055	-0.58552	-0.21346	0.32246	0.44952
aPF13_0230_1	PF13_0230	-0.32482	-0.32642	0.8061	0.7464	-0.46758
aPF13_0230_0	PF13_0230	-0.32482	-0.32642	0.8061	0.7464	-0.46758
intPF13_0230_2	PF13_0231	-0.32482	-0.32642	0.8061	0.7464	-0.46758
aPF13_0231_0	PF13_0231	0.1055	-0.58552	-0.21346	0.32246	0.44952
intPF13_0232_2	PF13_0232	-0.47702	-0.13591	0.55807	0.67943	0.06441
aPF13_0232_1	PF13_0232	-0.47702	-0.13591	0.55807	0.67943	0.06441
aPF13_0232_0	PF13_0232	-0.47702	-0.13591	0.55807	0.67943	0.06441
intPF13_0233_3	PF13_0233	1.26441	-0.08506	-1.20712	-1.92488	-1.15244
aPF13_0233_1	PF13_0233	1.26441	-0.08506	-1.20712	-1.92488	-1.15244
aPF13_0233_0	PF13_0233	1.26441	-0.08506	-1.20712	-1.92488	-1.15244
aPF13_0234_1	PF13_0234	-0.81986	-0.17621	1.610163	1.213003	-0.97663
aPF13_0234_0	PF13_0234	-0.81986	-0.17621	1.610163	1.213003	-0.97663
intPF13_0235_2	PF13_0235	0.47947	0.74765	0.57851	-0.20071	-1.02305
aPF13_0235_5	PF13_0235	0.47947	0.74765	0.57851	-0.20071	-1.02305
aPF13_0235_4	PF13_0235	0.47947	0.74765	0.57851	-0.20071	-1.02305
aPF13_0235_3	PF13_0235	0.47947	0.74765	0.57851	-0.20071	-1.02305
aPF13_0235_2	PF13_0235	0.47947	0.74765	0.57851	-0.20071	-1.02305
aPF13_0235_1	PF13_0235	0.47947	0.74765	0.57851	-0.20071	-1.02305
aPF13_0235_0	PF13_0235	0.47947	0.74765	0.57851	-0.20071	-1.02305
intPF13_0236_2	PF13_0236	-0.28973	0.240133	0.307233	0.016433	-0.28197
aPF13_0236_1	PF13_0236	-0.28973	0.240133	0.307233	0.016433	-0.28197
aPF13_0236_0	PF13_0236	-0.28973	0.240133	0.307233	0.016433	-0.28197
intPF13_0237_2	PF13_0237	-1.5415	-0.3895	0.09125	0.60475	0.994
aPF13_0237_1	PF13_0237	-1.5415	-0.3895	0.09125	0.60475	0.994
aPF13_0237_0	PF13_0237	-1.5415	-0.3895	0.09125	0.60475	0.994
intPF13_0238_3	PF13_0238	1.329147	0.806257	-0.24851	-1.18765	-1.23142
aPF13_0238_1	PF13_0238	1.329147	0.806257	-0.24851	-1.18765	-1.23142
aPF13_0238_0	PF13_0238	1.329147	0.806257	-0.24851	-1.18765	-1.23142
aPF13_0239_1	PF13_0239	0.502037	0.622297	0.019647	-0.34231	-0.30196
aPF13_0239_0	PF13_0239	0.502037	0.622297	0.019647	-0.34231	-0.30196
intPF13_0241_2	PF13_0241	-0.78664	-0.40676	0.250787	0.594547	0.423097
aPF13_0241_1	PF13_0241	-0.78664	-0.40676	0.250787	0.594547	0.423097
aPF13_0241_0	PF13_0241	-0.78664	-0.40676	0.250787	0.594547	0.423097
intPF13_0242_2	PF13_0242	-0.7743	-0.5385	0.167233	0.652433	0.571163
aPF13_0242_1	PF13_0242	-0.7743	-0.5385	0.167233	0.652433	0.571163
aPF13_0242_0	PF13_0242	-0.7743	-0.5385	0.167233	0.652433	0.571163
intPF13_0243_3	PF13_0243	0.115457	0.416207	0.477087	0.139087	-0.42704
aPF13_0243_1	PF13_0243	0.115457	0.416207	0.477087	0.139087	-0.42704
aPF13_0243_0	PF13_0243	0.115457	0.416207	0.477087	0.139087	-0.42704
intPF13_0245_2	PF13_0245	1.02947	0.74413	0.37374	-0.31924	-1.01113
aPF13_0245_1	PF13_0245	1.02947	0.74413	0.37374	-0.31924	-1.01113
aPF13_0245_0	PF13_0245	1.02947	0.74413	0.37374	-0.31924	-1.01113
intPF13_0246_2	PF13_0246	-0.62959	-0.83528	-0.25435	0.474813	0.831543
aPF13_0246_0	PF13_0246	-0.62959	-0.83528	-0.25435	0.474813	0.831543

intPF13_0247_2	PF13_0247	-0.02651	-0.25247	0.37194	0.41956	-0.21203
aPF13_0247_1	PF13_0247	-0.02651	-0.25247	0.37194	0.41956	-0.21203
aPF13_0247_0	PF13_0247	-0.02651	-0.25247	0.37194	0.41956	-0.21203
intPF13_0249_2	PF13_0248	-0.39126	-1.24569	-0.93212	0.281457	1.431027
intPF13_0248_2	PF13_0248	0.085247	-0.15925	-0.2645	-0.16516	0.102587
aPF13_0248_1	PF13_0248	0.085247	-0.15925	-0.2645	-0.16516	0.102587
aPF13_0248_0	PF13_0248	0.085247	-0.15925	-0.2645	-0.16516	0.102587
aPF13_0249_0	PF13_0249	-0.39126	-1.24569	-0.93212	0.281457	1.431027
intPF13_0251_2	PF13_0250	-1.01695	-0.95324	-0.36877	0.51177	1.14724
intPF13_0250_2	PF13_0250	-0.27353	0.971633	-0.29801	-0.69383	0.244533
aPF13_0250_1	PF13_0250	-0.27353	0.971633	-0.29801	-0.69383	0.244533
aPF13_0250_0	PF13_0250	-0.27353	0.971633	-0.29801	-0.69383	0.244533
aPF13_0251_1	PF13_0251	-1.01695	-0.95324	-0.36877	0.51177	1.14724
aPF13_0251_0	PF13_0251	-1.01695	-0.95324	-0.36877	0.51177	1.14724
intPF13_0252_2	PF13_0252	-0.6427	-0.31198	1.220977	1.157357	-0.49618
aPF13_0252_1	PF13_0252	-0.6427	-0.31198	1.220977	1.157357	-0.49618
aPF13_0252_0	PF13_0252	-0.6427	-0.31198	1.220977	1.157357	-0.49618
intPF13_0253_2	PF13_0253	1.12349	0.30705	-0.3996	-0.9809	-0.90855
intMAL13P1.238_2	PF13_0253	0.87593	-0.17466	-1.06967	-0.95733	0.11666
aPF13_0253_1	PF13_0253	1.12349	0.30705	-0.3996	-0.9809	-0.90855
aPF13_0253_0	PF13_0253	1.12349	0.30705	-0.3996	-0.9809	-0.90855
intPF13_0254_2	PF13_0254	-0.93402	-0.55513	0.053953	0.598713	0.740393
aPF13_0254_5	PF13_0254	-0.93402	-0.55513	0.053953	0.598713	0.740393
aPF13_0254_4	PF13_0254	-0.93402	-0.55513	0.053953	0.598713	0.740393
aPF13_0254_3	PF13_0254	-0.93402	-0.55513	0.053953	0.598713	0.740393
aPF13_0254_2	PF13_0254	-0.93402	-0.55513	0.053953	0.598713	0.740393
aPF13_0254_1	PF13_0254	-0.93402	-0.55513	0.053953	0.598713	0.740393
aPF13_0254_0	PF13_0254	-0.93402	-0.55513	0.053953	0.598713	0.740393
intPF13_0255_2	PF13_0255	0.00432	-0.35611	-1.50029	-1.07771	0.89511
aPF13_0255_1	PF13_0255	0.00432	-0.35611	-1.50029	-1.07771	0.89511
aPF13_0255_0	PF13_0255	0.00432	-0.35611	-1.50029	-1.07771	0.89511
aPF13_0256_0	PF13_0256	0.851073	0.763643	0.170213	-0.31955	-0.56598
intPF13_0257_2	PF13_0257	0.5483	0.39998	0.23254	-0.09004	-0.47748
aPF13_0257_1	PF13_0257	0.5483	0.39998	0.23254	-0.09004	-0.47748
aPF13_0257_0	PF13_0257	0.5483	0.39998	0.23254	-0.09004	-0.47748
intPF13_0258_2	PF13_0258	0.846348	-0.0264	-1.16903	-0.7403	0.733068
aPF13_0258_2	PF13_0258	0.846348	-0.0264	-1.16903	-0.7403	0.733068
aPF13_0258_1	PF13_0258	0.846348	-0.0264	-1.16903	-0.7403	0.733068
aPF13_0258_0	PF13_0258	0.846348	-0.0264	-1.16903	-0.7403	0.733068
intPF13_0259_2	PF13_0259	0.734107	0.530447	0.365107	-0.13777	-0.76711
aPF13_0259_0	PF13_0259	0.734107	0.530447	0.365107	-0.13777	-0.76711
aPF13_0260_0	PF13_0260	0.390307	-0.34552	-0.90594	-0.30772	0.838857
intPF13_0261_2	PF13_0261	0.888537	0.795817	0.567857	-0.34852	-1.32948
aPF13_0261_1	PF13_0261	0.888537	0.795817	0.567857	-0.34852	-1.32948
aPF13_0261_0	PF13_0261	0.888537	0.795817	0.567857	-0.34852	-1.32948
intMAL13P1.252_2	PF13_0262	0.350452	0.287812	-0.34802	-0.40708	0.102472
aPF13_0262_1	PF13_0262	-0.41374	0.112697	0.766097	0.726737	-0.10136

aPF13_0262_0	PF13_0262	-0.41374	0.112697	0.766097	0.726737	-0.10136
intPF13_0264_2	PF13_0263	-0.14922	-0.3064	-0.3372	0.025037	0.501237
intPF13_0263_2	PF13_0263	0.445613	0.374943	0.470613	0.028053	-0.71928
intMAL13P1.253_2	PF13_0263	0.309587	0.117087	0.343837	0.210497	-0.33975
aPF13_0263_0	PF13_0263	0.445613	0.374943	0.470613	0.028053	-0.71928
aPF13_0264_1	PF13_0264	-0.14922	-0.3064	-0.3372	0.025037	0.501237
aPF13_0264_0	PF13_0264	-0.14922	-0.3064	-0.3372	0.025037	0.501237
intPF13_0265_3	PF13_0265	0.342083	0.500063	0.121123	-0.27846	-0.4204
aPF13_0265_1	PF13_0265	0.342083	0.500063	0.121123	-0.27846	-0.4204
aPF13_0265_0	PF13_0265	0.342083	0.500063	0.121123	-0.27846	-0.4204
intPF13_0266_2	PF13_0266	-0.2862	-0.80396	-0.52406	0.133397	0.720297
aPF13_0266_0	PF13_0266	-0.2862	-0.80396	-0.52406	0.133397	0.720297
intPF13_0267_2	PF13_0267	0.16785	1.08875	0.23449	-0.40849	-0.37275
aPF13_0267_1	PF13_0267	0.16785	1.08875	0.23449	-0.40849	-0.37275
aPF13_0267_0	PF13_0267	0.16785	1.08875	0.23449	-0.40849	-0.37275
intPF13_0268_2	PF13_0268	0.154738	0.343268	0.867938	0.491378	-0.6769
aPF13_0268_0	PF13_0268	0.154738	0.343268	0.867938	0.491378	-0.6769
intPF13_0269_2	PF13_0269	-0.08149	0.391443	0.423123	-0.13146	-0.68478
aPF13_0269_1	PF13_0269	-0.08149	0.391443	0.423123	-0.13146	-0.68478
aPF13_0269_0	PF13_0269	-0.08149	0.391443	0.423123	-0.13146	-0.68478
intPF13_0270_2	PF13_0270	0.886663	0.901663	0.625663	-0.531	-1.638
aPF13_0270_0	PF13_0270	0.886663	0.901663	0.625663	-0.531	-1.638
aPF13_0271_1	PF13_0271	0.456493	0.323033	0.375693	-0.38753	-1.20687
aPF13_0271_0	PF13_0271	0.456493	0.323033	0.375693	-0.38753	-1.20687
intPF13_0272_1	PF13_0272	-1.68772	-0.39737	-0.23326	0.587597	1.606707
aPF13_0272_0	PF13_0272	-1.68772	-0.39737	-0.23326	0.587597	1.606707
intPF13_0273_2	PF13_0273	0.29154	0.51404	0.37717	0.00017	-0.44571
aPF13_0273_3	PF13_0273	0.29154	0.51404	0.37717	0.00017	-0.44571
aPF13_0273_2	PF13_0273	0.29154	0.51404	0.37717	0.00017	-0.44571
aPF13_0273_1	PF13_0273	0.29154	0.51404	0.37717	0.00017	-0.44571
aPF13_0273_0	PF13_0273	0.29154	0.51404	0.37717	0.00017	-0.44571
intPF13_0274_2	PF13_0274	-0.30779	-0.36004	0.47408	0.61342	-0.08646
aPF13_0274_1	PF13_0274	-0.30779	-0.36004	0.47408	0.61342	-0.08646
aPF13_0274_0	PF13_0274	-0.30779	-0.36004	0.47408	0.61342	-0.08646
aPF13_0275_0	PF13_0275	1.291537	2.383337	1.523817	-0.99248	-3.124
intPF13_0276_2	PF13_0276	2.144863	2.446613	0.784743	-1.62871	-2.96564
aPF13_0276_0	PF13_0276	2.144863	2.446613	0.784743	-1.62871	-2.96564
aPF13_0277_3	PF13_0277	-0.68208	-0.21267	0.10814	0.36483	0.43456
aPF13_0277_2	PF13_0277	-0.68208	-0.21267	0.10814	0.36483	0.43456
aPF13_0277_1	PF13_0277	-0.68208	-0.21267	0.10814	0.36483	0.43456
aPF13_0277_0	PF13_0277	-0.68208	-0.21267	0.10814	0.36483	0.43456
intPF13_0279_2	PF13_0278	0.182247	0.359787	0.462917	0.153417	-0.43945
intPF13_0278_2	PF13_0278	0.68321	0.98244	0.31662	-0.39612	-0.74744
intPF13_0277_2	PF13_0278	-0.68208	-0.21267	0.10814	0.36483	0.43456
aPF13_0278_1	PF13_0278	0.68321	0.98244	0.31662	-0.39612	-0.74744
aPF13_0278_0	PF13_0278	0.68321	0.98244	0.31662	-0.39612	-0.74744
aPF13_0279_0	PF13_0279	0.182247	0.359787	0.462917	0.153417	-0.43945

intPF13_0280_3	PF13_0280	-0.69943	-0.40184	-0.10083	0.34383	0.66584
aPF13_0280_0	PF13_0280	-0.69943	-0.40184	-0.10083	0.34383	0.66584
intPF13_0281_2	PF13_0281	0.723643	1.314763	0.989963	-0.0143	-1.2011
aPF13_0281_0	PF13_0281	0.723643	1.314763	0.989963	-0.0143	-1.2011
aPF13_0282_0	PF13_0282	-0.7578	-0.60399	0.258487	0.874847	0.688327
aPF13_0283_1	PF13_0283	-0.39179	0.098477	-0.06745	-0.11171	0.152357
aPF13_0283_0	PF13_0283	-0.39179	0.098477	-0.06745	-0.11171	0.152357
intPF13_0284_2	PF13_0284	-0.55181	0.50507	0.77287	0.12813	-0.70407
intPF13_0283_2	PF13_0284	-0.39179	0.098477	-0.06745	-0.11171	0.152357
aPF13_0284_0	PF13_0284	-0.55181	0.50507	0.77287	0.12813	-0.70407
intPF13_0285_2	PF13_0285	-0.23384	-0.0448	-0.16617	-0.29417	-0.08854
aPF13_0285_1	PF13_0285	-0.23384	-0.0448	-0.16617	-0.29417	-0.08854
aPF13_0285_0	PF13_0285	-0.23384	-0.0448	-0.16617	-0.29417	-0.08854
intPF13_0286_2	PF13_0286	0.622177	0.878597	0.827807	-0.26097	-1.53076
aPF13_0286_1	PF13_0286	0.622177	0.878597	0.827807	-0.26097	-1.53076
aPF13_0286_0	PF13_0286	0.622177	0.878597	0.827807	-0.26097	-1.53076
intPF13_0287_2	PF13_0287	-0.0993	0.373112	1.036452	0.623332	-0.68333
aPF13_0287_1	PF13_0287	-0.0993	0.373112	1.036452	0.623332	-0.68333
intPF13_0288_2	PF13_0288	0.241268	-0.32808	-0.04523	0.076128	-0.10202
aPF13_0288_1	PF13_0288	0.241268	-0.32808	-0.04523	0.076128	-0.10202
aPF13_0288_0	PF13_0288	0.241268	-0.32808	-0.04523	0.076128	-0.10202
intPF13_0289_2	PF13_0289	0.262667	0.876147	0.257957	-0.37762	-0.54281
aPF13_0289_1	PF13_0289	0.262667	0.876147	0.257957	-0.37762	-0.54281
aPF13_0289_0	PF13_0289	0.262667	0.876147	0.257957	-0.37762	-0.54281
intPF13_0290_2	PF13_0290	0.064727	0.415877	-0.01725	-0.22891	-0.05604
aPF13_0290_1	PF13_0290	0.064727	0.415877	-0.01725	-0.22891	-0.05604
aPF13_0290_0	PF13_0290	0.064727	0.415877	-0.01725	-0.22891	-0.05604
intPF13_0291_2	PF13_0291	-1.13801	-1.44395	-0.3391	0.962937	1.482787
aPF13_0291_1	PF13_0291	-1.13801	-1.44395	-0.3391	0.962937	1.482787
aPF13_0291_0	PF13_0291	-1.13801	-1.44395	-0.3391	0.962937	1.482787
aMAL13P1.273_0	PF13_0291	-1.38822	-0.80488	-0.23141	0.701228	1.402248
intPF13_0293_2	PF13_0292	0.904563	0.804203	0.448093	-0.48243	-1.31054
intPF13_0292_2	PF13_0292	1.684753	0.880713	0.253083	-0.73642	-1.54305
aPF13_0292_1	PF13_0292	1.684753	0.880713	0.253083	-0.73642	-1.54305
aPF13_0292_0	PF13_0292	1.684753	0.880713	0.253083	-0.73642	-1.54305
aPF13_0293_1	PF13_0293	0.904563	0.804203	0.448093	-0.48243	-1.31054
aPF13_0293_0	PF13_0293	0.904563	0.804203	0.448093	-0.48243	-1.31054
intPF13_0295_2	PF13_0295	0.248617	0.116427	0.471397	0.242937	-0.50509
aPF13_0295_1	PF13_0295	0.248617	0.116427	0.471397	0.242937	-0.50509
aPF13_0295_0	PF13_0295	0.248617	0.116427	0.471397	0.242937	-0.50509
intPF13_0296_2	PF13_0296	0.204123	-0.11962	0.369733	0.225933	-0.48672
aPF13_0296_0	PF13_0296	0.204123	-0.11962	0.369733	0.225933	-0.48672
intPF13_0297_2	PF13_0297	0.473573	0.573123	0.309503	-0.07384	-0.45396
aPF13_0297_1	PF13_0297	0.473573	0.573123	0.309503	-0.07384	-0.45396
aPF13_0297_0	PF13_0297	0.473573	0.573123	0.309503	-0.07384	-0.45396
intPF13_0298_2	PF13_0298	0.281143	-0.21765	0.276503	0.192303	-0.4573
aPF13_0298_2	PF13_0298	0.281143	-0.21765	0.276503	0.192303	-0.4573

aPF13_0298_1	PF13_0298	0.281143	-0.21765	0.276503	0.192303	-0.4573
aPF13_0298_0	PF13_0298	0.281143	-0.21765	0.276503	0.192303	-0.4573
intPF13_0299_2	PF13_0299	-1.09601	-1.40345	-1.3416	0.1261	2.07845
aPF13_0299_1	PF13_0299	-1.09601	-1.40345	-1.3416	0.1261	2.07845
aPF13_0299_0	PF13_0299	-1.09601	-1.40345	-1.3416	0.1261	2.07845
intPF13_0300_2	PF13_0300	-0.51375	0.000787	1.084417	0.703917	-0.76045
aPF13_0300_0	PF13_0300	-0.51375	0.000787	1.084417	0.703917	-0.76045
intPF13_0301_2	PF13_0301					
aPF13_0301_0	PF13_0301					
intPF13_0302_2	PF13_0302	-0.06473	-0.35486	0.08772	0.34678	0.14436
aPF13_0302_1	PF13_0302	-0.06473	-0.35486	0.08772	0.34678	0.14436
aPF13_0302_0	PF13_0302	-0.06473	-0.35486	0.08772	0.34678	0.14436
intPF13_0303_2	PF13_0303	0.10434	0.4593	0.10889	-0.27839	-0.3458
aPF13_0303_1	PF13_0303	0.10434	0.4593	0.10889	-0.27839	-0.3458
aPF13_0303_0	PF13_0303	0.10434	0.4593	0.10889	-0.27839	-0.3458
intPF13_0305_2	PF13_0304	-0.28332	0.40411	0.81404	0.64296	-0.16411
intPF13_0304_2	PF13_0304	-0.15766	0.381297	0.850417	0.626917	-0.31196
aPF13_0305_0	PF13_0304	-0.28332	0.40411	0.81404	0.64296	-0.16411
aPF13_0304_0	PF13_0304	-0.15766	0.381297	0.850417	0.626917	-0.31196
aPF13_0305_1	PF13_0305	-0.28332	0.40411	0.81404	0.64296	-0.16411
aPF13_0304_1	PF13_0305	-0.15766	0.381297	0.850417	0.626917	-0.31196
aPF13_0306_0	PF13_0306					
intPF13_0307_2	PF13_0307	-0.98438	-0.37213	0.273003	0.512163	0.347793
aPF13_0307_1	PF13_0307	-0.98438	-0.37213	0.273003	0.512163	0.347793
aPF13_0307_0	PF13_0307	-0.98438	-0.37213	0.273003	0.512163	0.347793
intPF13_0308_2	PF13_0308	-0.3418	-0.3625	-0.48252	-0.04282	0.692663
intMAL13P1.282_2	PF13_0308	-0.103	0.11998	0.03954	0.09746	0.18802
aPF13_0308_1	PF13_0308	-0.3418	-0.3625	-0.48252	-0.04282	0.692663
aPF13_0308_0	PF13_0308	-0.3418	-0.3625	-0.48252	-0.04282	0.692663
intPF13_0309_2	PF13_0309	0.68461	1.00547	0.8878	-0.2073	-1.49647
aPF13_0309_1	PF13_0309	0.68461	1.00547	0.8878	-0.2073	-1.49647
aPF13_0309_0	PF13_0309	0.68461	1.00547	0.8878	-0.2073	-1.49647
intPF13_0310_3	PF13_0310	0.421683	0.575083	0.669823	-0.05816	-1.07242
aPF13_0310_1	PF13_0310	0.421683	0.575083	0.669823	-0.05816	-1.07242
aPF13_0310_0	PF13_0310	0.421683	0.575083	0.669823	-0.05816	-1.07242
intPF13_0312_2	PF13_0312	-0.62403	-1.25084	-0.34132	0.67732	1.01584
intMAL13P1.287_2	PF13_0312	-0.06454	-0.36034	-1.23832	-0.71702	0.976003
aPF13_0312_0	PF13_0312	-0.62403	-1.25084	-0.34132	0.67732	1.01584
aPF13_0313_1	PF13_0313	0.51996	1.26118	1.16014	-0.03814	-1.51668
aPF13_0313_0	PF13_0313	0.51996	1.26118	1.16014	-0.03814	-1.51668
intPF13_0314_2	PF13_0314	0.22288	0.4976	-0.79919	-1.16881	-0.0306
aPF13_0314_1	PF13_0314	0.22288	0.4976	-0.79919	-1.16881	-0.0306
aPF13_0314_0	PF13_0314	0.22288	0.4976	-0.79919	-1.16881	-0.0306
intPF13_0315_1	PF13_0315	-0.00942	0.2751	0.51004	0.25696	-0.3631
intMAL13P1.410_2	PF13_0315	0.353267	0.223667	0.072657	-0.12832	-0.29433
aPF13_0315_1	PF13_0315	-0.00942	0.2751	0.51004	0.25696	-0.3631
aPF13_0315_0	PF13_0315	-0.00942	0.2751	0.51004	0.25696	-0.3631

intPF13_0316_2	PF13_0316	0.550333	0.241353	0.759793	0.485873	-0.68669
aPF13_0316_0	PF13_0316	0.550333	0.241353	0.759793	0.485873	-0.68669
intPF13_0317_2	PF13_0317	-0.15312	-0.51677	-0.19164	0.263872	0.453652
aPF13_0317_0	PF13_0317	-0.15312	-0.51677	-0.19164	0.263872	0.453652
aPF13_0318_0	PF13_0318	0.512017	0.601897	0.160697	-0.20236	-0.36756
intMAL13P1.300_2	PF13_0319	0.084893	0.231513	0.648713	0.436953	-0.40785
aPF13_0319_0	PF13_0319	0.084117	0.542817	0.879067	0.308067	-0.81568
intPF13_0321_2	PF13_0321	-0.22917	-0.71614	-0.24973	0.35223	0.58514
aPF13_0321_1	PF13_0321	-0.22917	-0.71614	-0.24973	0.35223	0.58514
aPF13_0321_0	PF13_0321	-0.22917	-0.71614	-0.24973	0.35223	0.58514
intPF13_0322_2	PF13_0322	-0.44839	0.132957	0.155837	0.251997	0.330377
aPF13_0322_1	PF13_0322	-0.44839	0.132957	0.155837	0.251997	0.330377
aPF13_0322_0	PF13_0322	-0.44839	0.132957	0.155837	0.251997	0.330377
intPF13_0323_2	PF13_0323	0.54138	0.64108	0.55035	-0.26635	-1.16158
aPF13_0323_1	PF13_0323	0.54138	0.64108	0.55035	-0.26635	-1.16158
aPF13_0323_0	PF13_0323	0.54138	0.64108	0.55035	-0.26635	-1.16158
aPF13_0324_1	PF13_0324	0.280087	0.263547	-0.59608	-0.52658	0.385287
aPF13_0324_0	PF13_0324	0.280087	0.263547	-0.59608	-0.52658	0.385287
aPF13_0326_0	PF13_0326	0.28558	-0.4979	-1.30321	-1.02279	0.4279
intPF13_0327_2	PF13_0327	-0.49974	-0.89193	-0.53491	0.21278	0.8718
intMAL13P1.307_2	PF13_0327	-0.23662	-1.0219	-0.24969	0.62569	0.7939
aPF13_0327_0	PF13_0327	-0.49974	-0.89193	-0.53491	0.21278	0.8718
intPF13_0328_2	PF13_0328	-2.0211	-2.11052	-0.07045	1.73845	2.00352
aPF13_0328_0	PF13_0328	-2.0211	-2.11052	-0.07045	1.73845	2.00352
intPF13_0329_2	PF13_0329	-0.89991	-1.06844	-0.44063	0.43063	1.04144
aPF13_0329_0	PF13_0329	-0.89991	-1.06844	-0.44063	0.43063	1.04144
intPF13_0331_2	PF13_0330	-1.27942	-1.15992	-0.50967	0.603333	1.492583
intMAL13P1.309_2	PF13_0330	0.348307	0.138457	-0.2309	-0.49176	-0.33812
aPF13_0330_1	PF13_0330	0.4555	0.87252	0.85271	0.06129	-1.04152
aPF13_0330_0	PF13_0330	0.4555	0.87252	0.85271	0.06129	-1.04152
intPF13_0330_2	PF13_0331	0.4555	0.87252	0.85271	0.06129	-1.04152
aPF13_0331_0	PF13_0331	-1.27942	-1.15992	-0.50967	0.603333	1.492583
aPF13_0333_1	PF13_0333	0.23671	0.00345	-0.5294	-0.2216	0.56155
aPF13_0333_0	PF13_0333	0.23671	0.00345	-0.5294	-0.2216	0.56155
aPF13_0334_1	PF13_0334	0.736107	0.416977	0.551547	0.263287	-0.58364
aPF13_0334_0	PF13_0334	0.736107	0.416977	0.551547	0.263287	-0.58364
intPF13_0336_2	PF13_0335	-0.70002	-0.69391	-0.39693	0.270093	0.903573
intPF13_0335_3	PF13_0335	-0.06491	0.190513	0.054223	0.092943	0.188653
aPF13_0335_1	PF13_0335	-0.06491	0.190513	0.054223	0.092943	0.188653
aPF13_0335_0	PF13_0335	-0.06491	0.190513	0.054223	0.092943	0.188653
aPF13_0336_1	PF13_0336	-0.70002	-0.69391	-0.39693	0.270093	0.903573
aPF13_0336_0	PF13_0336	-0.70002	-0.69391	-0.39693	0.270093	0.903573
intMAL13P1.319_2	PF13_0337	-1.01152	-0.99594	0.171633	0.959033	0.797103
aPF13_0337_0	PF13_0337	-0.776	-0.44902	0.530442	0.803802	0.192532
intPF13_0338_2	PF13_0338	-1.19529	-1.95751	-1.41522	0.641557	2.650347
aPF13_0338_1	PF13_0338	-1.19529	-1.95751	-1.41522	0.641557	2.650347
aPF13_0338_0	PF13_0338	-1.19529	-1.95751	-1.41522	0.641557	2.650347

intPF13_0339_3	PF13_0339	0.76143	0.12321	-0.17834	-0.41138	-0.46177
aPF13_0339_2	PF13_0339	0.76143	0.12321	-0.17834	-0.41138	-0.46177
aPF13_0339_1	PF13_0339	0.76143	0.12321	-0.17834	-0.41138	-0.46177
aPF13_0339_0	PF13_0339	0.76143	0.12321	-0.17834	-0.41138	-0.46177
intPF13_0340_2	PF13_0340	0.616803	0.876973	0.269803	-0.34814	-0.63781
aPF13_0340_1	PF13_0340	0.616803	0.876973	0.269803	-0.34814	-0.63781
aPF13_0340_0	PF13_0340	0.616803	0.876973	0.269803	-0.34814	-0.63781
intPF13_0341_2	PF13_0341	0.011973	2.056123	0.863763	-0.8011	-1.41846
aPF13_0341_0	PF13_0341	0.011973	2.056123	0.863763	-0.8011	-1.41846
intMAL13P1.326_2	PF13_0342	1.325087	1.168587	0.384337	-0.51	-1.13225
aPF13_0342_0	PF13_0342	0.908897	0.828517	0.270467	-0.31313	-0.71618
intPF13_0343_2	PF13_0343	0.759417	0.508927	0.105147	-0.34331	-0.62159
aPF13_0343_1	PF13_0343	0.759417	0.508927	0.105147	-0.34331	-0.62159
aPF13_0343_0	PF13_0343	0.759417	0.508927	0.105147	-0.34331	-0.62159
aPF13_0344_1	PF13_0344	0.280073	0.855133	0.838483	0.054683	-0.97847
aPF13_0344_0	PF13_0344	0.280073	0.855133	0.838483	0.054683	-0.97847
intPF13_0345_2	PF13_0345	-0.46628	-0.4001	0.622957	0.805877	-0.04456
intPF13_0344_2	PF13_0345	0.280073	0.855133	0.838483	0.054683	-0.97847
intMAL13P1.328_2	PF13_0345	-0.05448	-0.41013	-1.2295	-0.76434	0.852293
aPF13_0345_1	PF13_0345	-0.46628	-0.4001	0.622957	0.805877	-0.04456
aPF13_0345_0	PF13_0345	-0.46628	-0.4001	0.622957	0.805877	-0.04456
intPF13_0346_2	PF13_0346	0.196187	0.173087	0.405827	0.225507	-0.35575
aPF13_0346_0	PF13_0346	0.196187	0.173087	0.405827	0.225507	-0.35575
intPF13_0347_2	PF13_0347	0.468707	0.489487	0.569027	-0.18069	-1.14415
aPF13_0347_0	PF13_0347	0.468707	0.489487	0.569027	-0.18069	-1.14415
intPF13_0348_2	PF13_0348	-0.26724	-0.30678	0.16531	0.45819	0.26878
aPF13_0348_1	PF13_0348	-0.26724	-0.30678	0.16531	0.45819	0.26878
aPF13_0348_0	PF13_0348	-0.26724	-0.30678	0.16531	0.45819	0.26878
intPF13_0349_2	PF13_0349	-1.12562	-1.57088	0.215767	1.460567	1.123217
aPF13_0349_0	PF13_0349	-1.12562	-1.57088	0.215767	1.460567	1.123217
intPF13_0351_2	PF13_0350	0.597833	0.260353	-0.00721	-0.47513	-0.72519
intPF13_0350_3	PF13_0350	-0.35162	-0.18991	-0.01094	0.001603	0.013073
aPF13_0350_1	PF13_0350	-0.35162	-0.18991	-0.01094	0.001603	0.013073
aPF13_0350_0	PF13_0350	-0.35162	-0.18991	-0.01094	0.001603	0.013073
aPF13_0351_1	PF13_0351	0.597833	0.260353	-0.00721	-0.47513	-0.72519
aPF13_0351_0	PF13_0351	0.597833	0.260353	-0.00721	-0.47513	-0.72519
intMAL13P1.340_2	PF13_0352	0.849167	0.982147	0.046207	-0.82987	-0.95781
aPF13_0352_1	PF13_0352	0.96771	0.87895	0.67735	-0.47935	-1.69745
aPF13_0352_0	PF13_0352	0.96771	0.87895	0.67735	-0.47935	-1.69745
intPF13_0353_2	PF13_0353	-0.58862	-0.59342	-0.58942	0.173583	1.141583
aPF13_0353_1	PF13_0353	-0.58862	-0.59342	-0.58942	0.173583	1.141583
aPF13_0353_0	PF13_0353	-0.58862	-0.59342	-0.58942	0.173583	1.141583
intMAL13P1.341_2	PF13_0354	1.199747	0.744287	0.718417	-0.19408	-1.51195
aPF13_0354_2	PF13_0354	0.776992	0.611362	0.417112	-0.16823	-0.86847
aPF13_0354_1	PF13_0354	0.776992	0.611362	0.417112	-0.16823	-0.86847
aPF13_0354_0	PF13_0354	0.776992	0.611362	0.417112	-0.16823	-0.86847
intPF13_0355_2	PF13_0355	-0.19208	-0.00212	-0.09356	0.004857	0.241417

aPF13_0355_1	PF13_0355	-0.19208	-0.00212	-0.09356	0.004857	0.241417
aPF13_0355_0	PF13_0355	-0.19208	-0.00212	-0.09356	0.004857	0.241417
aPF13_0356_0	PF13_0356	-0.13684	0.221163	-0.13334	-0.122	0.254503
intPF13_0357_3	PF13_0357	0.392087	0.346047	0.353167	0.014167	-0.52621
intPF13_0356_2	PF13_0357	-0.13684	0.221163	-0.13334	-0.122	0.254503
aPF13_0357_1	PF13_0357	0.392087	0.346047	0.353167	0.014167	-0.52621
aPF13_0357_0	PF13_0357	0.392087	0.346047	0.353167	0.014167	-0.52621
intPF13_0358_2	PF13_0358	-0.26534	0.14671	1.20959	0.81841	-0.80371
aPF13_0358_0	PF13_0358	-0.26534	0.14671	1.20959	0.81841	-0.80371
intPF13_0359_2	PF13_0359	-0.94881	-0.55495	0.10765	0.64035	0.70945
aPF13_0359_1	PF13_0359	-0.94881	-0.55495	0.10765	0.64035	0.70945
aPF13_0359_0	PF13_0359	-0.94881	-0.55495	0.10765	0.64035	0.70945
aPF13_0360_1	PF13_0360	0.80064	0.30081	0.15764	-0.06564	-0.45381
aPF13_0360_0	PF13_0360	0.80064	0.30081	0.15764	-0.06564	-0.45381
intPF13_0361_2	PF13_0361	-0.47146	-0.52421	-0.03559	0.464413	0.569543
aPF13_0361_1	PF13_0361	-0.47146	-0.52421	-0.03559	0.464413	0.569543
aPF13_0361_0	PF13_0361	-0.47146	-0.52421	-0.03559	0.464413	0.569543
intPF14_0009_2	PF14_0009	0.590842	0.720202	0.363172	-0.16479	-0.62982
intPF14_0010_2	PF14_0010	0.563583	1.134063	0.716873	-0.22021	-1.0764
aPF14_0010_0	PF14_0010	0.563583	1.134063	0.716873	-0.22021	-1.0764
intPF14_0013_2	PF14_0013	3.277483	1.404603	-0.60095	-2.55839	-2.89194
aPF14_0013_1	PF14_0013	3.277483	1.404603	-0.60095	-2.55839	-2.89194
aPF14_0013_0	PF14_0013	3.277483	1.404603	-0.60095	-2.55839	-2.89194
intPF14_0014_2	PF14_0014	-1.80486	-1.56672	1.088933	1.985733	0.539383
aPF14_0014_0	PF14_0014	-1.80486	-1.56672	1.088933	1.985733	0.539383
intPF14_0015_2	PF14_0015	0.333103	0.419963	0.580573	0.015093	-0.8728
aPF14_0015_1	PF14_0015	0.333103	0.419963	0.580573	0.015093	-0.8728
aPF14_0015_0	PF14_0015	0.333103	0.419963	0.580573	0.015093	-0.8728
intPF14_0016_1	PF14_0016	2.469258	1.996258	-0.77675	-2.67473	-2.08029
aPF14_0016_0	PF14_0016	2.469258	1.996258	-0.77675	-2.67473	-2.08029
intPF14_0017_2	PF14_0017	0.94332	0.88241	1.19767	-0.09367	-2.06241
aPF14_0017_1	PF14_0017	0.94332	0.88241	1.19767	-0.09367	-2.06241
aPF14_0017_0	PF14_0017	0.94332	0.88241	1.19767	-0.09367	-2.06241
intPF14_0018_2	PF14_0018	0.100047	1.957767	-1.00702	-2.22064	-0.21293
aPF14_0018_1	PF14_0018	0.100047	1.957767	-1.00702	-2.22064	-0.21293
aPF14_0018_0	PF14_0018	0.100047	1.957767	-1.00702	-2.22064	-0.21293
intPF14_0019_2	PF14_0019	2.190547	1.841287	-0.45156	-1.7781	-1.34195
aPF14_0019_0	PF14_0019	2.190547	1.841287	-0.45156	-1.7781	-1.34195
intPF14_0020_2	PF14_0020	-1.34964	-1.16279	-0.58218	0.36318	1.28679
aPF14_0020_1	PF14_0020	-1.34964	-1.16279	-0.58218	0.36318	1.28679
aPF14_0020_0	PF14_0020	-1.34964	-1.16279	-0.58218	0.36318	1.28679
aPF14_0021_1	PF14_0021	0.722763	-0.20932	-0.74961	-0.74823	-0.12852
aPF14_0021_0	PF14_0021	0.722763	-0.20932	-0.74961	-0.74823	-0.12852
intPF14_0023_2	PF14_0022	0.896363	1.132203	0.658593	-0.25643	-1.13604
intPF14_0021_2	PF14_0022	0.722763	-0.20932	-0.74961	-0.74823	-0.12852
aPF14_0022_1	PF14_0022	0.213423	0.787373	0.867743	0.240923	-0.77571
aPF14_0022_0	PF14_0022	0.213423	0.787373	0.867743	0.240923	-0.77571

intPF14_0022_2	PF14_0023	0.213423	0.787373	0.867743	0.240923	-0.77571
aPF14_0023_1	PF14_0023	0.896363	1.132203	0.658593	-0.25643	-1.13604
aPF14_0023_0	PF14_0023	0.896363	1.132203	0.658593	-0.25643	-1.13604
intPF14_0024_2	PF14_0024	-0.7988	-1.225	-0.85052	0.181183	1.280663
aPF14_0024_0	PF14_0024	-0.7988	-1.225	-0.85052	0.181183	1.280663
intPF14_0025_2	PF14_0025	-0.55906	-0.24822	-0.04781	0.32281	0.59272
aPF14_0025_1	PF14_0025	-0.55906	-0.24822	-0.04781	0.32281	0.59272
aPF14_0025_0	PF14_0025	-0.55906	-0.24822	-0.04781	0.32281	0.59272
intPF14_0026_2	PF14_0026	0.100263	0.021173	0.023913	0.143753	0.152493
aPF14_0026_1	PF14_0026	0.100263	0.021173	0.023913	0.143753	0.152493
aPF14_0026_0	PF14_0026	0.100263	0.021173	0.023913	0.143753	0.152493
intPF14_0027_2	PF14_0027	0.44409	0.30654	0.71291	0.45409	-0.56954
aPF14_0027_0	PF14_0027	0.44409	0.30654	0.71291	0.45409	-0.56954
intPF14_0028_2	PF14_0028	0.250423	1.204363	0.552263	-0.2741	-0.7042
aPF14_0028_1	PF14_0028	0.250423	1.204363	0.552263	-0.2741	-0.7042
aPF14_0028_0	PF14_0028	0.250423	1.204363	0.552263	-0.2741	-0.7042
intPF14_0029_2	PF14_0029	-0.45369	-0.03378	-0.39506	-0.30252	0.399198
aPF14_0029_1	PF14_0029	-0.45369	-0.03378	-0.39506	-0.30252	0.399198
aPF14_0029_0	PF14_0029	-0.45369	-0.03378	-0.39506	-0.30252	0.399198
intPF14_0030_2	PF14_0029a	0.359857	0.140557	-0.10221	-0.18295	-0.11328
aPF14_0029_3	PF14_0029a	-0.45369	-0.03378	-0.39506	-0.30252	0.399198
aPF14_0029_2	PF14_0029a	-0.45369	-0.03378	-0.39506	-0.30252	0.399198
aPF14_0030_1	PF14_0030	0.359857	0.140557	-0.10221	-0.18295	-0.11328
aPF14_0030_0	PF14_0030	0.359857	0.140557	-0.10221	-0.18295	-0.11328
intPF14_0032_2	PF14_0031	-0.84977	-1.06719	-0.29162	0.758283	1.243853
aPF14_0031_1	PF14_0031	-1.05701	-0.88472	-0.3312	0.546445	1.189365
aPF14_0031_0	PF14_0031	-1.05701	-0.88472	-0.3312	0.546445	1.189365
aPF14_0031_5	PF14_0031a	-1.05701	-0.88472	-0.3312	0.546445	1.189365
aPF14_0031_4	PF14_0031a	-1.05701	-0.88472	-0.3312	0.546445	1.189365
aPF14_0031_3	PF14_0031a	-1.05701	-0.88472	-0.3312	0.546445	1.189365
aPF14_0031_2	PF14_0031a	-1.05701	-0.88472	-0.3312	0.546445	1.189365
intPF14_0031_2	PF14_0032	-1.05701	-0.88472	-0.3312	0.546445	1.189365
aPF14_0032_1	PF14_0032	-0.84977	-1.06719	-0.29162	0.758283	1.243853
aPF14_0032_0	PF14_0032	-0.84977	-1.06719	-0.29162	0.758283	1.243853
aPF14_0033_0	PF14_0033	-0.17068	-0.95961	-0.64529	0.13229	0.79861
intPF14_0034_2	PF14_0034					
aPF14_0034_0	PF14_0034					
intPF14_0035_2	PF14_0035	-0.01284	0.348172	-0.30835	-0.3791	0.233052
aPF14_0035_2	PF14_0035	-0.01284	0.348172	-0.30835	-0.3791	0.233052
aPF14_0035_1	PF14_0035	-0.01284	0.348172	-0.30835	-0.3791	0.233052
aPF14_0035_0	PF14_0035	-0.01284	0.348172	-0.30835	-0.3791	0.233052
intPF14_0037_2	PF14_0036	-0.07285	0.169267	0.055967	-0.02363	-0.00093
intPF14_0036_2	PF14_0036	-0.79037	-0.08815	1.24681	1.04819	-0.50885
aPF14_0036_0	PF14_0036	-0.79037	-0.08815	1.24681	1.04819	-0.50885
aPF14_0037_0	PF14_0037	-0.07285	0.169267	0.055967	-0.02363	-0.00093
intPF14_0038_2	PF14_0038	-0.64075	-0.17425	1.118497	1.039837	-0.39341
aPF14_0038_0	PF14_0038	-0.64075	-0.17425	1.118497	1.039837	-0.39341

intPF14_0039_2	PF14_0039	-0.53076	-0.31772	-0.57154	0.054143	1.094133
aPF14_0039_0	PF14_0039	-0.53076	-0.31772	-0.57154	0.054143	1.094133
intPF14_0040_2	PF14_0040	0.325312	0.220462	-0.27046	-0.25598	0.150412
aPF14_0040_0	PF14_0040	0.325312	0.220462	-0.27046	-0.25598	0.150412
intPF14_0042_2	PF14_0041	0.45511	0.48396	0.36582	-0.15582	-0.73346
intPF14_0041_2	PF14_0041	0.12408	0.4366	0.01929	-0.16929	-0.0396
aPF14_0041_0	PF14_0041	0.12408	0.4366	0.01929	-0.16929	-0.0396
aPF14_0042_1	PF14_0042	0.45511	0.48396	0.36582	-0.15582	-0.73346
aPF14_0042_0	PF14_0042	0.45511	0.48396	0.36582	-0.15582	-0.73346
intPF14_0044_3	PF14_0044	2.216843	0.957773	-0.99655	-2.41779	-1.90011
aPF14_0044_0	PF14_0044	2.216843	0.957773	-0.99655	-2.41779	-1.90011
intPF14_0045_2	PF14_0045	1.974337	1.297337	0.332337	-1.66	-2.8925
aPF14_0045_1	PF14_0045	1.974337	1.297337	0.332337	-1.66	-2.8925
aPF14_0045_0	PF14_0045	1.974337	1.297337	0.332337	-1.66	-2.8925
intPF14_0046_2	PF14_0046	0.541223	-0.28711	-0.12078	-0.22856	-0.49323
aPF14_0046_0	PF14_0046	0.541223	-0.28711	-0.12078	-0.22856	-0.49323
intPF14_0047_2	PF14_0047	0.515847	0.473247	-0.00998	-0.35618	-0.37241
aPF14_0047_1	PF14_0047	0.515847	0.473247	-0.00998	-0.35618	-0.37241
aPF14_0047_0	PF14_0047	0.515847	0.473247	-0.00998	-0.35618	-0.37241
aPF14_0048_1	PF14_0048	-0.10481	0.173547	0.278657	-0.05932	-0.44921
aPF14_0048_0	PF14_0048	-0.10481	0.173547	0.278657	-0.05932	-0.44921
intPF14_0050_2	PF14_0049	0.066943	-0.11024	-0.26954	-0.0213	0.366403
intPF14_0049_3	PF14_0049	0.929143	-0.13674	-0.41904	-0.1753	0.104403
intPF14_0048_2	PF14_0049	-0.10481	0.173547	0.278657	-0.05932	-0.44921
aPF14_0049_0	PF14_0049	0.929143	-0.13674	-0.41904	-0.1753	0.104403
aPF14_0050_1	PF14_0050	0.066943	-0.11024	-0.26954	-0.0213	0.366403
aPF14_0050_0	PF14_0050	0.066943	-0.11024	-0.26954	-0.0213	0.366403
intPF14_0052_2	PF14_0051	0.516463	0.776193	0.721373	-0.00921	-0.99103
intPF14_0051_2	PF14_0051	-0.51938	-0.00847	0.641515	0.618875	-0.08032
aPF14_0051_2	PF14_0051	-0.51938	-0.00847	0.641515	0.618875	-0.08032
aPF14_0051_1	PF14_0051	-0.51938	-0.00847	0.641515	0.618875	-0.08032
aPF14_0051_0	PF14_0051	-0.51938	-0.00847	0.641515	0.618875	-0.08032
aPF14_0052_1	PF14_0052	0.516463	0.776193	0.721373	-0.00921	-0.99103
aPF14_0052_0	PF14_0052	0.516463	0.776193	0.721373	-0.00921	-0.99103
intPF14_0053_2	PF14_0053	-0.83663	-1.59683	-0.05385	1.24185	1.17583
aPF14_0053_0	PF14_0053	-0.83663	-1.59683	-0.05385	1.24185	1.17583
intPF14_0054_2	PF14_0054	0.044077	-0.04887	0.023257	-0.41392	-0.71079
aPF14_0054_1	PF14_0054	0.044077	-0.04887	0.023257	-0.41392	-0.71079
aPF14_0054_0	PF14_0054	0.044077	-0.04887	0.023257	-0.41392	-0.71079
aPF14_0055_1	PF14_0055	0.949927	0.844357	0.577287	-0.47495	-1.52452
aPF14_0055_0	PF14_0055	0.949927	0.844357	0.577287	-0.47495	-1.52452
intPF14_0055_2	PF14_0056	0.949927	0.844357	0.577287	-0.47495	-1.52452
aPF14_0056_1	PF14_0056	0.76948	1.23912	0.26301	-0.76401	-1.05862
aPF14_0056_0	PF14_0056	0.76948	1.23912	0.26301	-0.76401	-1.05862
intPF14_0057_2	PF14_0057	0.331967	0.443157	0.571687	0.253647	-0.48282
aPF14_0057_0	PF14_0057	0.331967	0.443157	0.571687	0.253647	-0.48282
intPF14_0058_2	PF14_0058	0.312917	0.703097	0.023307	-0.38697	-0.25876

aPF14_0058_2	PF14_0058	0.312917	0.703097	0.023307	-0.38697	-0.25876
aPF14_0058_1	PF14_0058	0.312917	0.703097	0.023307	-0.38697	-0.25876
aPF14_0058_0	PF14_0058	0.312917	0.703097	0.023307	-0.38697	-0.25876
intPF14_0059_2	PF14_0059	0.343627	0.549167	0.220307	-0.20827	-0.46473
aPF14_0059_4	PF14_0059	0.343627	0.549167	0.220307	-0.20827	-0.46473
aPF14_0059_3	PF14_0059	0.343627	0.549167	0.220307	-0.20827	-0.46473
aPF14_0059_2	PF14_0059	0.343627	0.549167	0.220307	-0.20827	-0.46473
aPF14_0059_1	PF14_0059	0.343627	0.549167	0.220307	-0.20827	-0.46473
aPF14_0059_0	PF14_0059	0.343627	0.549167	0.220307	-0.20827	-0.46473
intPF14_0060_2	PF14_0060	0.013723	-0.30362	-0.35026	-0.09858	0.287283
aPF14_0060_1	PF14_0060	0.013723	-0.30362	-0.35026	-0.09858	0.287283
aPF14_0060_0	PF14_0060	0.013723	-0.30362	-0.35026	-0.09858	0.287283
intPF14_0061_2	PF14_0061	-1.19034	-1.09683	0.157143	0.998023	0.874493
aPF14_0061_1	PF14_0061	-1.19034	-1.09683	0.157143	0.998023	0.874493
aPF14_0061_0	PF14_0061	-1.19034	-1.09683	0.157143	0.998023	0.874493
intPF14_0062_2	PF14_0062	-0.98991	-0.63471	0.791365	1.004005	-0.03798
aPF14_0062_2	PF14_0062	-0.98991	-0.63471	0.791365	1.004005	-0.03798
aPF14_0062_1	PF14_0062	-0.98991	-0.63471	0.791365	1.004005	-0.03798
aPF14_0062_0	PF14_0062	-0.98991	-0.63471	0.791365	1.004005	-0.03798
intPF14_0063_2	PF14_0063	-1.16782	-1.19691	-0.40217	0.75817	1.46591
aPF14_0063_1	PF14_0063	-1.16782	-1.19691	-0.40217	0.75817	1.46591
aPF14_0063_0	PF14_0063	-1.16782	-1.19691	-0.40217	0.75817	1.46591
intPF14_0064_2	PF14_0064	-1.17062	-0.89488	0.129267	0.853067	0.836217
aPF14_0064_0	PF14_0064	-1.17062	-0.89488	0.129267	0.853067	0.836217
intPF14_0065_2	PF14_0065	-0.35956	-0.7262	-1.4151	-0.5379	1.4232
aPF14_0065_0	PF14_0065	-0.35956	-0.7262	-1.4151	-0.5379	1.4232
intPF14_0067_2	PF14_0066	0.53193	1.60936	-0.49171	-1.54629	-0.52236
intPF14_0066_2	PF14_0066	-1.25129	-0.60451	0.749027	0.719307	-0.28015
aPF14_0066_0	PF14_0066	-1.25129	-0.60451	0.749027	0.719307	-0.28015
aPF14_0067_1	PF14_0067	0.53193	1.60936	-0.49171	-1.54629	-0.52236
aPF14_0067_0	PF14_0067	0.53193	1.60936	-0.49171	-1.54629	-0.52236
intPF14_0068_2	PF14_0068	0.451293	0.720513	0.555973	-0.22831	-1.03185
aPF14_0068_0	PF14_0068	0.451293	0.720513	0.555973	-0.22831	-1.03185
intPF14_0069_3	PF14_0069	0.823437	0.576837	0.163827	-0.69749	-1.2365
aPF14_0069_0	PF14_0069	0.823437	0.576837	0.163827	-0.69749	-1.2365
intPF14_0070_2	PF14_0070	0.435983	0.035083	0.119843	-0.18718	-0.62242
aPF14_0070_1	PF14_0070	0.435983	0.035083	0.119843	-0.18718	-0.62242
aPF14_0070_0	PF14_0070	0.435983	0.035083	0.119843	-0.18718	-0.62242
aPF14_0071_0	PF14_0071	0.13304	-0.14822	0.34594	0.28906	-0.34378
intPF14_0072_2	PF14_0072	0.445207	0.327947	0.877607	0.274727	-1.12661
intPF14_0071_2	PF14_0072	0.13304	-0.14822	0.34594	0.28906	-0.34378
aPF14_0072_0	PF14_0072	0.445207	0.327947	0.877607	0.274727	-1.12661
intPF14_0073_2	PF14_0073	1.48813	1.05583	-0.4639	-1.1221	-0.62483
aPF14_0073_3	PF14_0073	1.48813	1.05583	-0.4639	-1.1221	-0.62483
aPF14_0073_2	PF14_0073	1.48813	1.05583	-0.4639	-1.1221	-0.62483
aPF14_0073_1	PF14_0073	1.48813	1.05583	-0.4639	-1.1221	-0.62483
aPF14_0073_0	PF14_0073	1.48813	1.05583	-0.4639	-1.1221	-0.62483

intPF14_0074_2	PF14_0074	0.739168	0.171608	0.139008	-0.11864	-0.56479
aPF14_0074_1	PF14_0074	0.739168	0.171608	0.139008	-0.11864	-0.56479
aPF14_0074_0	PF14_0074	0.739168	0.171608	0.139008	-0.11864	-0.56479
intPF14_0075_1	PF14_0075	1.061643	0.444803	0.088893	-0.70723	-1.28114
aPF14_0075_1	PF14_0075	1.061643	0.444803	0.088893	-0.70723	-1.28114
aPF14_0075_0	PF14_0075	1.061643	0.444803	0.088893	-0.70723	-1.28114
intPF14_0076_2	PF14_0076	1.80969	1.01357	0.16337	-1.31637	-2.20157
aPF14_0076_1	PF14_0076	1.80969	1.01357	0.16337	-1.31637	-2.20157
aPF14_0076_0	PF14_0076	1.80969	1.01357	0.16337	-1.31637	-2.20157
intPF14_0077_3	PF14_0077	-0.51668	-0.45061	0.862213	0.904453	-0.35223
aPF14_0077_1	PF14_0077	-0.51668	-0.45061	0.862213	0.904453	-0.35223
aPF14_0077_0	PF14_0077	-0.51668	-0.45061	0.862213	0.904453	-0.35223
intPF14_0078_2	PF14_0078	-0.16835	0.61527	0.93272	0.39228	-0.65527
aPF14_0078_1	PF14_0078	-0.16835	0.61527	0.93272	0.39228	-0.65527
aPF14_0078_0	PF14_0078	-0.16835	0.61527	0.93272	0.39228	-0.65527
intPF14_0079_2	PF14_0079	0.747633	0.982493	0.695513	-0.36263	-1.41327
aPF14_0079_2	PF14_0079	0.747633	0.982493	0.695513	-0.36263	-1.41327
aPF14_0079_1	PF14_0079	0.747633	0.982493	0.695513	-0.36263	-1.41327
aPF14_0079_0	PF14_0079	0.747633	0.982493	0.695513	-0.36263	-1.41327
intPF14_0080_2	PF14_0080	-0.28638	-0.51473	-0.21397	0.116352	0.319112
aPF14_0080_0	PF14_0080	-0.28638	-0.51473	-0.21397	0.116352	0.319112
intPF14_0081_2	PF14_0081	-0.38184	-0.35032	0.121123	0.352543	0.200483
aPF14_0081_1	PF14_0081	-0.38184	-0.35032	0.121123	0.352543	0.200483
aPF14_0081_0	PF14_0081	-0.38184	-0.35032	0.121123	0.352543	0.200483
intPF14_0082_2	PF14_0082	0.417837	0.451837	0.718337	0.038997	-1.0965
aPF14_0082_0	PF14_0082	0.417837	0.451837	0.718337	0.038997	-1.0965
intPF14_0083_3	PF14_0083	-0.25715	0.33573	0.87353	0.66747	-0.29073
aPF14_0083_0	PF14_0083	-0.25715	0.33573	0.87353	0.66747	-0.29073
intPF14_0084_3	PF14_0084	0.339977	0.438077	-0.58271	-0.56127	0.405127
aPF14_0084_9	PF14_0084	0.339977	0.438077	-0.58271	-0.56127	0.405127
aPF14_0084_8	PF14_0084	0.339977	0.438077	-0.58271	-0.56127	0.405127
aPF14_0084_7	PF14_0084	0.339977	0.438077	-0.58271	-0.56127	0.405127
aPF14_0084_6	PF14_0084	0.339977	0.438077	-0.58271	-0.56127	0.405127
aPF14_0084_5	PF14_0084	0.339977	0.438077	-0.58271	-0.56127	0.405127
aPF14_0084_4	PF14_0084	0.339977	0.438077	-0.58271	-0.56127	0.405127
aPF14_0084_3	PF14_0084	0.339977	0.438077	-0.58271	-0.56127	0.405127
aPF14_0084_2	PF14_0084	0.339977	0.438077	-0.58271	-0.56127	0.405127
aPF14_0084_10	PF14_0084	0.339977	0.438077	-0.58271	-0.56127	0.405127
aPF14_0084_1	PF14_0084	0.339977	0.438077	-0.58271	-0.56127	0.405127
aPF14_0084_0	PF14_0084	0.339977	0.438077	-0.58271	-0.56127	0.405127
intPF14_0085_2	PF14_0085	0.170622	0.034462	-0.28265	-0.18972	0.199722
aPF14_0085_0	PF14_0085	0.170622	0.034462	-0.28265	-0.18972	0.199722
intPF14_0086_2	PF14_0086	1.172143	0.968283	1.047433	-0.08877	-1.79862
aPF14_0086_0	PF14_0086	1.172143	0.968283	1.047433	-0.08877	-1.79862
aPF14_0087_1	PF14_0087	0.654817	0.764397	0.698217	-0.01288	-1.00806
aPF14_0087_0	PF14_0087	0.654817	0.764397	0.698217	-0.01288	-1.00806
intPF14_0088_2	PF14_0088	-0.51146	-0.44868	-0.38014	0.270477	0.974017

intPF14_0087_2	PF14_0088	0.654817	0.764397	0.698217	-0.01288	-1.00806
aPF14_0088_1	PF14_0088	-0.51146	-0.44868	-0.38014	0.270477	0.974017
aPF14_0088_0	PF14_0088	-0.51146	-0.44868	-0.38014	0.270477	0.974017
aPF14_0089_0	PF14_0089-a	-0.9056	-0.751	-0.64549	0.137827	1.191337
intPF14_0090_2	PF14_0090	-1.27541	-1.24248	-0.57205	0.535713	1.449143
intPF14_0089_2	PF14_0090	-0.9056	-0.751	-0.64549	0.137827	1.191337
aPF14_0090_1	PF14_0090	-1.27541	-1.24248	-0.57205	0.535713	1.449143
aPF14_0090_0	PF14_0090	-1.27541	-1.24248	-0.57205	0.535713	1.449143
intPF14_0092_2	PF14_0092	-0.63558	-0.81312	-1.36675	-0.52425	1.38912
intPF14_0091_2	PF14_0092	-0.79967	-1.54213	-1.31301	-0.05001	1.570633
aPF14_0092_0	PF14_0092	-0.63558	-0.81312	-1.36675	-0.52425	1.38912
aPF14_0091_0	PF14_0092	-0.79967	-1.54213	-1.31301	-0.05001	1.570633
intPF14_0093_2	PF14_0093	0.424937	0.116317	-0.50313	-0.90553	-0.49998
aPF14_0093_1	PF14_0093	0.424937	0.116317	-0.50313	-0.90553	-0.49998
aPF14_0093_0	PF14_0093	0.424937	0.116317	-0.50313	-0.90553	-0.49998
intPF14_0094_2	PF14_0094	0.17878	-0.02088	-0.19174	-0.10076	0.12688
aPF14_0094_1	PF14_0094	0.17878	-0.02088	-0.19174	-0.10076	0.12688
aPF14_0094_0	PF14_0094	0.17878	-0.02088	-0.19174	-0.10076	0.12688
intPF14_0095_2	PF14_0095	-0.35071	-0.46347	0.47444	0.72256	0.02197
aPF14_0095_1	PF14_0095	-0.35071	-0.46347	0.47444	0.72256	0.02197
aPF14_0095_0	PF14_0095	-0.35071	-0.46347	0.47444	0.72256	0.02197
intPF14_0096_2	PF14_0096	-1.00014	-0.37229	1.00632	1.10368	-0.10421
aPF14_0096_1	PF14_0096	-1.00014	-0.37229	1.00632	1.10368	-0.10421
aPF14_0096_0	PF14_0096	-1.00014	-0.37229	1.00632	1.10368	-0.10421
intPF14_0097_2	PF14_0097	-0.91406	-0.48219	0.61313	0.94637	0.27869
aPF14_0097_1	PF14_0097	-0.91406	-0.48219	0.61313	0.94637	0.27869
aPF14_0097_0	PF14_0097	-0.91406	-0.48219	0.61313	0.94637	0.27869
intPF14_0098_2	PF14_0098	0.821203	0.485983	0.237023	-0.18136	-0.65632
aPF14_0098_0	PF14_0098	0.821203	0.485983	0.237023	-0.18136	-0.65632
intPF14_0099_2	PF14_0099	0.376022	0.538882	-0.18049	-0.50735	-0.19621
aPF14_0099_0	PF14_0099	0.376022	0.538882	-0.18049	-0.50735	-0.19621
intPF14_0100_2	PF14_0100	0.434033	0.828323	1.182353	0.057313	-1.67416
aPF14_0100_1	PF14_0100	0.434033	0.828323	1.182353	0.057313	-1.67416
aPF14_0100_0	PF14_0100	0.434033	0.828323	1.182353	0.057313	-1.67416
intPF14_0101_2	PF14_0101	0.872073	0.514433	-0.20262	-0.45824	-0.26363
aPF14_0101_6	PF14_0101	0.872073	0.514433	-0.20262	-0.45824	-0.26363
aPF14_0101_5	PF14_0101	0.872073	0.514433	-0.20262	-0.45824	-0.26363
aPF14_0101_4	PF14_0101	0.872073	0.514433	-0.20262	-0.45824	-0.26363
aPF14_0101_3	PF14_0101	0.872073	0.514433	-0.20262	-0.45824	-0.26363
aPF14_0101_2	PF14_0101	0.872073	0.514433	-0.20262	-0.45824	-0.26363
aPF14_0101_1	PF14_0101	0.872073	0.514433	-0.20262	-0.45824	-0.26363
aPF14_0101_0	PF14_0101	0.872073	0.514433	-0.20262	-0.45824	-0.26363
intPF14_0102_2	PF14_0102	-0.70169	-1.35458	-2.73335	-1.12949	2.647743
aPF14_0102_1	PF14_0102	-0.70169	-1.35458	-2.73335	-1.12949	2.647743
aPF14_0102_0	PF14_0102	-0.70169	-1.35458	-2.73335	-1.12949	2.647743
intPF14_0103_2	PF14_0103	-0.13108	-0.6516	0.14871	0.63529	0.2896
aPF14_0103_0	PF14_0103	-0.13108	-0.6516	0.14871	0.63529	0.2896

intPF14_0104_2	PF14_0104	-0.10045	0.15773	0.53029	0.38821	-0.25123
aPF14_0104_1	PF14_0104	-0.10045	0.15773	0.53029	0.38821	-0.25123
aPF14_0104_0	PF14_0104	-0.10045	0.15773	0.53029	0.38821	-0.25123
intPF14_0105_3	PF14_0105	-1.46224	-2.16677	-0.96396	1.075297	2.423107
aPF14_0105_0	PF14_0105	-1.46224	-2.16677	-0.96396	1.075297	2.423107
intPF14_0106_2	PF14_0106	0.129572	-0.42705	-1.44306	-0.87411	1.006552
aPF14_0106_0	PF14_0106	0.129572	-0.42705	-1.44306	-0.87411	1.006552
intPF14_0107_2	PF14_0107	0.275497	0.008027	-0.13381	-0.29335	-0.28119
aPF14_0107_1	PF14_0107	0.275497	0.008027	-0.13381	-0.29335	-0.28119
aPF14_0107_0	PF14_0107	0.275497	0.008027	-0.13381	-0.29335	-0.28119
intPF14_0108_3	PF14_0108-a	1.681057	0.460057	-1.60777	-2.53622	-1.11039
aPF14_0108_2	PF14_0108-a	1.681057	0.460057	-1.60777	-2.53622	-1.11039
aPF14_0108_1	PF14_0108-a	1.681057	0.460057	-1.60777	-2.53622	-1.11039
aPF14_0108_0	PF14_0108-a	1.681057	0.460057	-1.60777	-2.53622	-1.11039
intPF14_0110_3	PF14_0110	0.37266	0.25321	-0.05016	-0.66084	-0.85471
aPF14_0110_1	PF14_0110	0.37266	0.25321	-0.05016	-0.66084	-0.85471
aPF14_0110_0	PF14_0110	0.37266	0.25321	-0.05016	-0.66084	-0.85471
intPF14_0111_2	PF14_0111	-0.03926	0.18328	0.13494	-0.11544	-0.28528
aPF14_0111_1	PF14_0111	-0.03926	0.18328	0.13494	-0.11544	-0.28528
aPF14_0111_0	PF14_0111	-0.03926	0.18328	0.13494	-0.11544	-0.28528
aPF14_0112_2	PF14_0112	-0.70608	-0.84763	0.034415	0.774365	0.768735
aPF14_0112_1	PF14_0112	-0.70608	-0.84763	0.034415	0.774365	0.768735
aPF14_0112_0	PF14_0112	-0.70608	-0.84763	0.034415	0.774365	0.768735
intPF14_0113_3	PF14_0113	1.162867	0.944667	0.567667	-0.45383	-1.47933
intPF14_0112_2	PF14_0113	-0.70608	-0.84763	0.034415	0.774365	0.768735
aPF14_0113_1	PF14_0113	1.162867	0.944667	0.567667	-0.45383	-1.47933
aPF14_0113_0	PF14_0113	1.162867	0.944667	0.567667	-0.45383	-1.47933
intPF14_0115_2	PF14_0114	0.649047	0.635777	-0.01929	-0.34187	-0.26994
intPF14_0114_2	PF14_0114	-0.57274	-0.4278	0.195603	0.545563	0.365463
aPF14_0114_1	PF14_0114	-0.57274	-0.4278	0.195603	0.545563	0.365463
aPF14_0114_0	PF14_0114	-0.57274	-0.4278	0.195603	0.545563	0.365463
aPF14_0115_1	PF14_0115	0.649047	0.635777	-0.01929	-0.34187	-0.26994
aPF14_0115_0	PF14_0115	0.649047	0.635777	-0.01929	-0.34187	-0.26994
intPF14_0116_3	PF14_0116	1.210617	0.430437	0.297377	-0.20454	-0.9686
aPF14_0116_1	PF14_0116	1.210617	0.430437	0.297377	-0.20454	-0.9686
aPF14_0116_0	PF14_0116	1.210617	0.430437	0.297377	-0.20454	-0.9686
intPF14_0117_2	PF14_0117	1.300173	1.044123	0.848743	-0.65208	-2.27146
aPF14_0117_0	PF14_0117	1.300173	1.044123	0.848743	-0.65208	-2.27146
intPF14_0119_2	PF14_0119	0.482023	-0.0931	-1.9923	-1.57204	1.071763
aPF14_0119_0	PF14_0119	0.482023	-0.0931	-1.9923	-1.57204	1.071763
aPF14_0118_0	PF14_0119	0.52111	1.23946	0.03257	-0.95557	-0.84046
intPF14_0120_2	PF14_0120	0.004933	-1.28419	-0.44239	0.389053	0.536853
aPF14_0120_1	PF14_0120	0.004933	-1.28419	-0.44239	0.389053	0.536853
aPF14_0120_0	PF14_0120	0.004933	-1.28419	-0.44239	0.389053	0.536853
intPF14_0121_2	PF14_0121	0.84874	0.73478	-0.21931	-0.64819	-0.36328
aPF14_0121_1	PF14_0121	0.84874	0.73478	-0.21931	-0.64819	-0.36328
aPF14_0121_0	PF14_0121	0.84874	0.73478	-0.21931	-0.64819	-0.36328

intPF14_0122_2	PF14_0122	0.04721	-0.38707	-0.06236	0.46436	0.53707
aPF14_0122_0	PF14_0122	0.04721	-0.38707	-0.06236	0.46436	0.53707
intPF14_0123_2	PF14_0123	0.35864	0.48841	-0.31531	-0.37809	0.21699
aPF14_0123_4	PF14_0123	0.35864	0.48841	-0.31531	-0.37809	0.21699
aPF14_0123_3	PF14_0123	0.35864	0.48841	-0.31531	-0.37809	0.21699
aPF14_0123_2	PF14_0123	0.35864	0.48841	-0.31531	-0.37809	0.21699
aPF14_0123_1	PF14_0123	0.35864	0.48841	-0.31531	-0.37809	0.21699
aPF14_0123_0	PF14_0123	0.35864	0.48841	-0.31531	-0.37809	0.21699
intPF14_0124_1	PF14_0124	0.161653	0.877743	1.483003	0.391663	-1.60008
aPF14_0124_1	PF14_0124	0.161653	0.877743	1.483003	0.391663	-1.60008
aPF14_0124_0	PF14_0124	0.161653	0.877743	1.483003	0.391663	-1.60008
intPF14_0126_2	PF14_0125	-0.27856	0.11878	0.68394	0.51606	-0.30778
aPF14_0125_1	PF14_0125	0.94262	0.74538	0.89148	0.02552	-1.41338
aPF14_0125_0	PF14_0125	0.94262	0.74538	0.89148	0.02552	-1.41338
intPF14_0125_2	PF14_0126	0.94262	0.74538	0.89148	0.02552	-1.41338
aPF14_0126_1	PF14_0126	-0.27856	0.11878	0.68394	0.51606	-0.30778
aPF14_0126_0	PF14_0126	-0.27856	0.11878	0.68394	0.51606	-0.30778
intPF14_0127_2	PF14_0127	0.772873	0.862623	-0.164	-0.6045	-0.28863
aPF14_0127_1	PF14_0127	0.772873	0.862623	-0.164	-0.6045	-0.28863
aPF14_0127_0	PF14_0127	0.772873	0.862623	-0.164	-0.6045	-0.28863
intPF14_0128_2	PF14_0128-a	0.453607	0.664947	-0.03789	-0.34277	-0.15261
aPF14_0128_0	PF14_0128-a	0.453607	0.664947	-0.03789	-0.34277	-0.15261
intPF14_0129_2	PF14_0129	1.012152	0.768922	0.368982	-0.39476	-1.09337
aPF14_0129_1	PF14_0129	1.012152	0.768922	0.368982	-0.39476	-1.09337
aPF14_0129_0	PF14_0129	1.012152	0.768922	0.368982	-0.39476	-1.09337
intPF14_0130_2	PF14_0130	0.053177	0.182897	-0.03139	-0.09847	0.014037
aPF14_0130_1	PF14_0130	0.053177	0.182897	-0.03139	-0.09847	0.014037
aPF14_0130_0	PF14_0130	0.053177	0.182897	-0.03139	-0.09847	0.014037
intPF14_0131_2	PF14_0131	1.298212	0.644952	-0.86753	-0.98589	0.108882
aPF14_0131_0	PF14_0131	1.298212	0.644952	-0.86753	-0.98589	0.108882
intPF14_0132_2	PF14_0132	-0.95608	-0.8891	-0.26879	0.636127	1.172937
aPF14_0132_1	PF14_0132	-0.95608	-0.8891	-0.26879	0.636127	1.172937
aPF14_0132_0	PF14_0132	-0.95608	-0.8891	-0.26879	0.636127	1.172937
intPF14_0134_2	PF14_0133					
aPF14_0133_0	PF14_0133	-0.69011	-0.97845	-0.25811	0.674773	1.063113
intPF14_0133_2	PF14_0134	-0.69011	-0.97845	-0.25811	0.674773	1.063113
aPF14_0134_0	PF14_0134					
intPF14_0135_2	PF14_0135	0.813973	-0.06589	-1.32047	-1.44737	-0.06545
aPF14_0135_1	PF14_0135	0.813973	-0.06589	-1.32047	-1.44737	-0.06545
aPF14_0135_0	PF14_0135	0.813973	-0.06589	-1.32047	-1.44737	-0.06545
intPF14_0136_2	PF14_0136	1.388293	1.110053	0.578893	-0.82323	-2.01889
aPF14_0136_1	PF14_0136	1.388293	1.110053	0.578893	-0.82323	-2.01889
aPF14_0136_0	PF14_0136	1.388293	1.110053	0.578893	-0.82323	-2.01889
intPF14_0137_2	PF14_0137	0.092193	0.109033	0.201923	0.188243	-0.04587
aPF14_0137_1	PF14_0137	0.092193	0.109033	0.201923	0.188243	-0.04587
aPF14_0137_0	PF14_0137	0.092193	0.109033	0.201923	0.188243	-0.04587
intPF14_0138_2	PF14_0138	-0.97454	-1.06228	-0.34993	0.692267	1.296617

aPF14_0138_0	PF14_0138	-0.97454	-1.06228	-0.34993	0.692267	1.296617
intPF14_0139_2	PF14_0139	0.695753	0.151243	-0.16973	-0.52911	-0.61358
aPF14_0139_1	PF14_0139	0.695753	0.151243	-0.16973	-0.52911	-0.61358
intPF14_0140_2	PF14_0140	0.122707	-0.24051	-0.17897	-0.22869	-0.21615
aPF14_0140_1	PF14_0140	0.122707	-0.24051	-0.17897	-0.22869	-0.21615
aPF14_0140_0	PF14_0140	0.122707	-0.24051	-0.17897	-0.22869	-0.21615
intPF14_0141_3	PF14_0141	-0.43261	0.310708	0.739258	0.534308	-0.20249
aPF14_0141_0	PF14_0141	-0.43261	0.310708	0.739258	0.534308	-0.20249
intPF14_0142_2	PF14_0142	-1.10491	-1.6652	-0.74421	0.653378	1.602618
aPF14_0142_0	PF14_0142	-1.10491	-1.6652	-0.74421	0.653378	1.602618
intPF14_0143_2	PF14_0143	0.202238	0.431538	-0.64169	-0.90339	0.041628
aPF14_0143_4	PF14_0143	0.202238	0.431538	-0.64169	-0.90339	0.041628
aPF14_0143_3	PF14_0143	0.202238	0.431538	-0.64169	-0.90339	0.041628
aPF14_0143_2	PF14_0143	0.202238	0.431538	-0.64169	-0.90339	0.041628
aPF14_0143_1	PF14_0143	0.202238	0.431538	-0.64169	-0.90339	0.041628
aPF14_0143_0	PF14_0143	0.202238	0.431538	-0.64169	-0.90339	0.041628
intPF14_0144_2	PF14_0144	0.32039	0.88254	0.01218	-0.71718	-0.61654
aPF14_0144_1	PF14_0144	0.32039	0.88254	0.01218	-0.71718	-0.61654
aPF14_0144_0	PF14_0144	0.32039	0.88254	0.01218	-0.71718	-0.61654
intPF14_0145_3	PF14_0145	0.950562	0.854192	0.360272	-0.71005	-1.47297
aPF14_0145_2	PF14_0145	0.950562	0.854192	0.360272	-0.71005	-1.47297
aPF14_0145_1	PF14_0145	0.950562	0.854192	0.360272	-0.71005	-1.47297
aPF14_0145_0	PF14_0145	0.950562	0.854192	0.360272	-0.71005	-1.47297
intPF14_0146_2	PF14_0146	-0.08698	0.64488	0.58974	0.18626	-0.34288
aPF14_0146_0	PF14_0146	-0.08698	0.64488	0.58974	0.18626	-0.34288
aPF14_0147_1	PF14_0147	-0.26869	0.415453	0.717093	0.221573	-0.61579
aPF14_0147_0	PF14_0147	-0.26869	0.415453	0.717093	0.221573	-0.61579
intPF14_0149_2	PF14_0148	0.030253	-0.81079	-0.61292	-0.21242	0.254453
intPF14_0148_2	PF14_0148	-0.5395	-1.17048	-0.27804	0.712377	0.977817
intPF14_0147_2	PF14_0148	-0.26869	0.415453	0.717093	0.221573	-0.61579
aPF14_0148_0	PF14_0148	-0.5395	-1.17048	-0.27804	0.712377	0.977817
intPF14_0150_2	PF14_0149	0.944017	0.711897	0.763197	-0.29686	-1.68456
aPF14_0149_1	PF14_0149	0.030253	-0.81079	-0.61292	-0.21242	0.254453
aPF14_0149_0	PF14_0149	0.030253	-0.81079	-0.61292	-0.21242	0.254453
aPF14_0150_0	PF14_0150	0.944017	0.711897	0.763197	-0.29686	-1.68456
intPF14_0151_2	PF14_0151	-0.85637	-0.81013	0.415767	0.721567	0.065467
aPF14_0151_0	PF14_0151	-0.85637	-0.81013	0.415767	0.721567	0.065467
intPF14_0152_2	PF14_0152	-1.02021	-0.43728	0.56473	0.8836	0.33862
aPF14_0152_2	PF14_0152	-1.02021	-0.43728	0.56473	0.8836	0.33862
aPF14_0152_1	PF14_0152	-1.02021	-0.43728	0.56473	0.8836	0.33862
aPF14_0152_0	PF14_0152	-1.02021	-0.43728	0.56473	0.8836	0.33862
intPF14_0153_2	PF14_0153	-1.10721	-0.98749	0.029723	0.898943	1.004653
aPF14_0153_1	PF14_0153	-1.10721	-0.98749	0.029723	0.898943	1.004653
aPF14_0153_0	PF14_0153	-1.10721	-0.98749	0.029723	0.898943	1.004653
intPF14_0154_2	PF14_0154	-0.85044	0.35221	0.29333	0.24017	0.34079
aPF14_0154_1	PF14_0154	-0.85044	0.35221	0.29333	0.24017	0.34079
aPF14_0154_0	PF14_0154	-0.85044	0.35221	0.29333	0.24017	0.34079

intPF14_0155_2	PF14_0155	-0.92931	-0.53645	-0.30574	0.215218	0.835498
aPF14_0155_1	PF14_0155	-0.92931	-0.53645	-0.30574	0.215218	0.835498
aPF14_0155_0	PF14_0155	-0.92931	-0.53645	-0.30574	0.215218	0.835498
intPF14_0156_2	PF14_0156	0.561367	0.452147	0.872207	0.168627	-1.23181
aPF14_0156_1	PF14_0156	0.561367	0.452147	0.872207	0.168627	-1.23181
aPF14_0156_0	PF14_0156	0.561367	0.452147	0.872207	0.168627	-1.23181
intPF14_0158_2	PF14_0157					
intPF14_0157_2	PF14_0157	0.35643	0.52286	0.42529	-0.06129	-0.64186
aPF14_0157_0	PF14_0157	0.35643	0.52286	0.42529	-0.06129	-0.64186
aPF14_0158_0	PF14_0158					
intPF14_0159_2	PF14_0159	0.835333	0.451313	-0.82888	-0.93046	0.146353
aPF14_0159_1	PF14_0159	0.835333	0.451313	-0.82888	-0.93046	0.146353
aPF14_0159_0	PF14_0159	0.835333	0.451313	-0.82888	-0.93046	0.146353
intPF14_0160_2	PF14_0160	0.089257	-0.36851	-1.08822	-0.54519	0.901847
aPF14_0160_3	PF14_0160	0.089257	-0.36851	-1.08822	-0.54519	0.901847
aPF14_0160_2	PF14_0160	0.089257	-0.36851	-1.08822	-0.54519	0.901847
aPF14_0160_1	PF14_0160	0.089257	-0.36851	-1.08822	-0.54519	0.901847
aPF14_0160_0	PF14_0160	0.089257	-0.36851	-1.08822	-0.54519	0.901847
intPF14_0161_2	PF14_0161	-0.7815	-0.346	-0.0145	0.293163	0.496163
aPF14_0161_1	PF14_0161	-0.7815	-0.346	-0.0145	0.293163	0.496163
aPF14_0161_0	PF14_0161	-0.7815	-0.346	-0.0145	0.293163	0.496163
intPF14_0162_2	PF14_0162					
aPF14_0163_0	PF14_0162	2.019893	0.702513	-0.40354	-1.3198	-1.44485
aPF14_0162_0	PF14_0162					
intPF14_0164_2	PF14_0164	1.756247	0.098747	-0.89525	-1.42541	-1.01641
aPF14_0164_1	PF14_0164	1.756247	0.098747	-0.89525	-1.42541	-1.01641
aPF14_0164_0	PF14_0164	1.756247	0.098747	-0.89525	-1.42541	-1.01641
intPF14_0165_2	PF14_0165	-0.24418	-0.14154	-0.39289	-0.35475	0.175203
aPF14_0165_4	PF14_0165	-0.24418	-0.14154	-0.39289	-0.35475	0.175203
aPF14_0165_3	PF14_0165	-0.24418	-0.14154	-0.39289	-0.35475	0.175203
aPF14_0165_2	PF14_0165	-0.24418	-0.14154	-0.39289	-0.35475	0.175203
aPF14_0165_1	PF14_0165	-0.24418	-0.14154	-0.39289	-0.35475	0.175203
aPF14_0165_0	PF14_0165	-0.24418	-0.14154	-0.39289	-0.35475	0.175203
aPF14_0166_1	PF14_0166	-1.40071	0.053547	0.809647	0.714687	0.090287
aPF14_0166_0	PF14_0166	-1.40071	0.053547	0.809647	0.714687	0.090287
intPF14_0167_2	PF14_0167	-0.55977	-0.32113	0.92576	1.02124	-0.19287
aPF14_0167_0	PF14_0167	-0.55977	-0.32113	0.92576	1.02124	-0.19287
intPF14_0169_2	PF14_0168	-0.43686	-0.2462	-0.06234	0.207177	0.406537
intPF14_0168_2	PF14_0168					
aPF14_0168_1	PF14_0168					
aPF14_0168_0	PF14_0168					
aPF14_0169_0	PF14_0169	-0.43686	-0.2462	-0.06234	0.207177	0.406537
intPF14_0170_1	PF14_0170	0.564085	0.302415	-0.12912	-0.41965	-0.37918
aPF14_0170_6	PF14_0170	0.564085	0.302415	-0.12912	-0.41965	-0.37918
aPF14_0170_5	PF14_0170	0.564085	0.302415	-0.12912	-0.41965	-0.37918
aPF14_0170_4	PF14_0170	0.564085	0.302415	-0.12912	-0.41965	-0.37918
aPF14_0170_3	PF14_0170	0.564085	0.302415	-0.12912	-0.41965	-0.37918

aPF14_0170_2	PF14_0170	0.564085	0.302415	-0.12912	-0.41965	-0.37918
aPF14_0170_1	PF14_0170	0.564085	0.302415	-0.12912	-0.41965	-0.37918
aPF14_0170_0	PF14_0170	0.564085	0.302415	-0.12912	-0.41965	-0.37918
intPF14_0171_2	PF14_0171	0.145043	-0.40823	-1.64255	-0.92229	1.307393
aPF14_0171_1	PF14_0171	0.145043	-0.40823	-1.64255	-0.92229	1.307393
aPF14_0171_0	PF14_0171	0.145043	-0.40823	-1.64255	-0.92229	1.307393
intPF14_0173_2	PF14_0173	0.693907	-0.13801	-1.71372	-1.52544	0.530347
intPF14_0172_2	PF14_0173	0.498602	0.345342	-0.99468	-1.27522	-0.02523
aPF14_0173_1	PF14_0173	0.693907	-0.13801	-1.71372	-1.52544	0.530347
aPF14_0173_0	PF14_0173	0.693907	-0.13801	-1.71372	-1.52544	0.530347
aPF14_0172_3	PF14_0173	0.498602	0.345342	-0.99468	-1.27522	-0.02523
aPF14_0172_2	PF14_0173	0.498602	0.345342	-0.99468	-1.27522	-0.02523
aPF14_0172_1	PF14_0173	0.498602	0.345342	-0.99468	-1.27522	-0.02523
aPF14_0172_0	PF14_0173	0.498602	0.345342	-0.99468	-1.27522	-0.02523
intPF14_0174_3	PF14_0174	0.46049	0.72603	0.72669	-0.06019	-1.09203
aPF14_0174_1	PF14_0174	0.46049	0.72603	0.72669	-0.06019	-1.09203
aPF14_0174_0	PF14_0174	0.46049	0.72603	0.72669	-0.06019	-1.09203
intPF14_0175_2	PF14_0175	0.411062	0.459832	-0.13624	-0.54162	-0.39312
aPF14_0175_6	PF14_0175	0.411062	0.459832	-0.13624	-0.54162	-0.39312
aPF14_0175_5	PF14_0175	0.411062	0.459832	-0.13624	-0.54162	-0.39312
aPF14_0175_4	PF14_0175	0.411062	0.459832	-0.13624	-0.54162	-0.39312
aPF14_0175_3	PF14_0175	0.411062	0.459832	-0.13624	-0.54162	-0.39312
aPF14_0175_2	PF14_0175	0.411062	0.459832	-0.13624	-0.54162	-0.39312
aPF14_0175_1	PF14_0175	0.411062	0.459832	-0.13624	-0.54162	-0.39312
aPF14_0175_0	PF14_0175	0.411062	0.459832	-0.13624	-0.54162	-0.39312
intPF14_0176_2	PF14_0176	-0.19044	-0.1078	0.137593	0.366073	0.288463
aPF14_0176_0	PF14_0176	-0.19044	-0.1078	0.137593	0.366073	0.288463
intPF14_0177_2	PF14_0177	-1.13305	-1.51273	-0.64128	0.866117	1.844067
aPF14_0177_1	PF14_0177	-1.13305	-1.51273	-0.64128	0.866117	1.844067
aPF14_0177_0	PF14_0177	-1.13305	-1.51273	-0.64128	0.866117	1.844067
intPF14_0178_2	PF14_0178	-0.38288	0.26238	0.17059	0.11933	0.16878
aPF14_0178_0	PF14_0178	-0.38288	0.26238	0.17059	0.11933	0.16878
intPF14_0179_2	PF14_0179	-0.70721	-0.52502	-0.08191	0.361453	0.578063
aPF14_0179_5	PF14_0179	-0.70721	-0.52502	-0.08191	0.361453	0.578063
aPF14_0179_4	PF14_0179	-0.70721	-0.52502	-0.08191	0.361453	0.578063
aPF14_0179_3	PF14_0179	-0.70721	-0.52502	-0.08191	0.361453	0.578063
aPF14_0179_2	PF14_0179	-0.70721	-0.52502	-0.08191	0.361453	0.578063
aPF14_0179_1	PF14_0179	-0.70721	-0.52502	-0.08191	0.361453	0.578063
aPF14_0179_0	PF14_0179	-0.70721	-0.52502	-0.08191	0.361453	0.578063
intPF14_0181_2	PF14_0180	-0.53039	0.454943	0.792113	0.394553	-0.38028
intPF14_0180_2	PF14_0180	-1.37633	0.386647	0.828707	0.585627	0.075687
aPF14_0180_0	PF14_0180	-1.37633	0.386647	0.828707	0.585627	0.075687
aPF14_0181_0	PF14_0181	-0.53039	0.454943	0.792113	0.394553	-0.38028
intPF14_0183_3	PF14_0183b	1.032633	0.704323	0.536103	-0.39944	-1.45666
aPF14_0183_1	PF14_0183b	1.032633	0.704323	0.536103	-0.39944	-1.45666
aPF14_0183_0	PF14_0183b	1.032633	0.704323	0.536103	-0.39944	-1.45666
intPF14_0184_2	PF14_0184	-0.77536	-0.94169	-0.76586	0.270197	1.440027

aPF14_0184_1	PF14_0184	-0.77536	-0.94169	-0.76586	0.270197	1.440027
aPF14_0184_0	PF14_0184	-0.77536	-0.94169	-0.76586	0.270197	1.440027
intPF14_0185_2	PF14_0185	0.659407	0.729477	0.464297	-0.31696	-1.06014
aPF14_0185_1	PF14_0185	0.659407	0.729477	0.464297	-0.31696	-1.06014
aPF14_0185_0	PF14_0185	0.659407	0.729477	0.464297	-0.31696	-1.06014
intPF14_0186_2	PF14_0186	-1.42126	-0.8552	0.900153	1.408013	0.365363
aPF14_0186_1	PF14_0186	-1.42126	-0.8552	0.900153	1.408013	0.365363
aPF14_0186_0	PF14_0186	-1.42126	-0.8552	0.900153	1.408013	0.365363
intPF14_0187_2	PF14_0187	0.267943	0.606293	0.930903	0.328763	-0.89863
aPF14_0187_0	PF14_0187	0.267943	0.606293	0.930903	0.328763	-0.89863
intPF14_0188_3	PF14_0188	0.389223	0.581213	0.271903	-0.32913	-0.73444
aPF14_0188_3	PF14_0188	0.389223	0.581213	0.271903	-0.32913	-0.73444
aPF14_0188_2	PF14_0188	0.389223	0.581213	0.271903	-0.32913	-0.73444
aPF14_0188_1	PF14_0188	0.389223	0.581213	0.271903	-0.32913	-0.73444
aPF14_0188_0	PF14_0188	0.389223	0.581213	0.271903	-0.32913	-0.73444
intPF14_0190_2	PF14_0190	0.41443	0.51486	-0.34846	-0.61454	-0.07786
intPF14_0189_2	PF14_0190	-0.33322	-0.75389	-0.67722	0.12522	1.03789
aPF14_0190_1	PF14_0190	0.41443	0.51486	-0.34846	-0.61454	-0.07786
aPF14_0190_0	PF14_0190	0.41443	0.51486	-0.34846	-0.61454	-0.07786
intPF14_0191_3	PF14_0191	0.265667	0.313187	0.443877	0.084457	-0.56685
aPF14_0191_1	PF14_0191	0.265667	0.313187	0.443877	0.084457	-0.56685
aPF14_0191_0	PF14_0191	0.265667	0.313187	0.443877	0.084457	-0.56685
intPF14_0192_2	PF14_0192	-0.77347	-0.91568	0.470853	1.116813	0.459343
aPF14_0192_1	PF14_0192	-0.77347	-0.91568	0.470853	1.116813	0.459343
aPF14_0192_0	PF14_0192	-0.77347	-0.91568	0.470853	1.116813	0.459343
intPF14_0193_2	PF14_0193	1.269683	0.883573	0.200843	-0.67068	-1.20841
aPF14_0193_1	PF14_0193	1.269683	0.883573	0.200843	-0.67068	-1.20841
aPF14_0193_0	PF14_0193	1.269683	0.883573	0.200843	-0.67068	-1.20841
intPF14_0194_2	PF14_0194	0.167017	0.559417	0.523907	0.078427	-0.52558
aPF14_0194_1	PF14_0194	0.167017	0.559417	0.523907	0.078427	-0.52558
aPF14_0194_0	PF14_0194	0.167017	0.559417	0.523907	0.078427	-0.52558
intPF14_0195_2	PF14_0195	-0.52528	-0.43862	-0.54176	-0.06708	0.775283
aPF14_0195_2	PF14_0195	-0.52528	-0.43862	-0.54176	-0.06708	0.775283
aPF14_0195_1	PF14_0195	-0.52528	-0.43862	-0.54176	-0.06708	0.775283
aPF14_0195_0	PF14_0195	-0.52528	-0.43862	-0.54176	-0.06708	0.775283
intPF14_0197_2	PF14_0196	0.94296	0.7402	0.3991	-0.7071	-1.6132
aPF14_0196_1	PF14_0196	-0.10818	0.00791	-0.68183	-0.60117	0.38959
aPF14_0196_0	PF14_0196	-0.10818	0.00791	-0.68183	-0.60117	0.38959
intPF14_0196_2	PF14_0197	-0.10818	0.00791	-0.68183	-0.60117	0.38959
aPF14_0197_0	PF14_0197	0.94296	0.7402	0.3991	-0.7071	-1.6132
intPF14_0198_2	PF14_0198	0.185833	0.537853	0.640293	0.159373	-0.63819
aPF14_0198_1	PF14_0198	0.185833	0.537853	0.640293	0.159373	-0.63819
aPF14_0198_0	PF14_0198	0.185833	0.537853	0.640293	0.159373	-0.63819
intPF14_0199_2	PF14_0199	0.38086	-0.11131	-0.12539	-0.11861	-0.14919
aPF14_0199_1	PF14_0199	0.38086	-0.11131	-0.12539	-0.11861	-0.14919
aPF14_0199_0	PF14_0199	0.38086	-0.11131	-0.12539	-0.11861	-0.14919
intPF14_0200_2	PF14_0200	-0.82938	-0.39457	0.584397	0.898937	0.290407

aPF14_0200_1	PF14_0200	-0.82938	-0.39457	0.584397	0.898937	0.290407
aPF14_0200_0	PF14_0200	-0.82938	-0.39457	0.584397	0.898937	0.290407
intPF14_0201_1	PF14_0201	0.766123	0.382373	0.295253	-0.42275	-1.17588
aPF14_0201_1	PF14_0201	0.766123	0.382373	0.295253	-0.42275	-1.17588
aPF14_0201_0	PF14_0201	0.766123	0.382373	0.295253	-0.42275	-1.17588
intPF14_0204_2	PF14_0204	0.928833	0.357343	-0.37619	-0.59865	-0.27968
aPF14_0204_1	PF14_0204	0.928833	0.357343	-0.37619	-0.59865	-0.27968
aPF14_0204_0	PF14_0204	0.928833	0.357343	-0.37619	-0.59865	-0.27968
intPF14_0205_3	PF14_0205	1.131597	0.541027	0.674457	0.106877	-1.09269
aPF14_0205_0	PF14_0205	1.131597	0.541027	0.674457	0.106877	-1.09269
intPF14_0206_2	PF14_0206	-0.16068	-0.88612	-0.98227	-0.04557	1.195283
aPF14_0206_1	PF14_0206	-0.16068	-0.88612	-0.98227	-0.04557	1.195283
aPF14_0206_0	PF14_0206	-0.16068	-0.88612	-0.98227	-0.04557	1.195283
intPF14_0208_2	PF14_0207	-0.05806	0.254337	0.679327	0.389007	-0.473
aPF14_0207_0	PF14_0207	0.816127	0.681367	0.769267	0.048067	-1.15603
intPF14_0209_2	PF14_0208	0.685013	0.186943	0.588623	0.286043	-0.75378
intPF14_0207_2	PF14_0208	0.816127	0.681367	0.769267	0.048067	-1.15603
aPF14_0208_0	PF14_0208	-0.05806	0.254337	0.679327	0.389007	-0.473
aPF14_0209_1	PF14_0209	0.685013	0.186943	0.588623	0.286043	-0.75378
aPF14_0209_0	PF14_0209	0.685013	0.186943	0.588623	0.286043	-0.75378
intPF14_0210_2	PF14_0210	-0.70473	-0.48538	0.472503	0.583663	-0.07946
aPF14_0210_1	PF14_0210	-0.70473	-0.48538	0.472503	0.583663	-0.07946
aPF14_0210_0	PF14_0210	-0.70473	-0.48538	0.472503	0.583663	-0.07946
aPF14_0211_0	PF14_0211	-0.38585	-0.75877	-0.44145	0.27845	0.84977
intPF14_0787_2	PF14_0212					
intPF14_0212_2	PF14_0212	-0.19451	-1.02995	-0.6526	0.159437	0.813787
aPF14_0212_1	PF14_0212	-0.19451	-1.02995	-0.6526	0.159437	0.813787
aPF14_0212_0	PF14_0212	-0.19451	-1.02995	-0.6526	0.159437	0.813787
intPF14_0214_2	PF14_0213a	0.615957	0.806217	0.580987	-0.31915	-1.21171
aPF14_0213_0	PF14_0213a	0.28249	-0.17897	-0.90381	-0.53869	0.63547
intPF14_0213_2	PF14_0214	0.28249	-0.17897	-0.90381	-0.53869	0.63547
aPF14_0214_2	PF14_0214	0.615957	0.806217	0.580987	-0.31915	-1.21171
aPF14_0214_1	PF14_0214	0.615957	0.806217	0.580987	-0.31915	-1.21171
aPF14_0214_0	PF14_0214	0.615957	0.806217	0.580987	-0.31915	-1.21171
intPF14_0215_2	PF14_0215	-1.0449	-1.513	-0.81601	0.621673	1.768163
aPF14_0215_1	PF14_0215	-1.0449	-1.513	-0.81601	0.621673	1.768163
aPF14_0215_0	PF14_0215	-1.0449	-1.513	-0.81601	0.621673	1.768163
intPF14_0216_2	PF14_0216	-0.5015	-0.37001	-0.26998	0.205143	0.723173
aPF14_0216_1	PF14_0216	-0.5015	-0.37001	-0.26998	0.205143	0.723173
aPF14_0216_0	PF14_0216	-0.5015	-0.37001	-0.26998	0.205143	0.723173
intPF14_0217_2	PF14_0217	-0.0083	0.285527	0.441697	0.189637	-0.33219
aPF14_0217_1	PF14_0217	-0.0083	0.285527	0.441697	0.189637	-0.33219
aPF14_0217_0	PF14_0217	-0.0083	0.285527	0.441697	0.189637	-0.33219
intPF14_0218_2	PF14_0218	0.26657	0.86466	0.48967	-0.09467	-0.55216
aPF14_0218_1	PF14_0218	0.26657	0.86466	0.48967	-0.09467	-0.55216
aPF14_0218_0	PF14_0218	0.26657	0.86466	0.48967	-0.09467	-0.55216
intPF14_0220_2	PF14_0220	0.800357	0.673207	-0.13616	-0.556	-0.40237

aPF14_0220_1	PF14_0220	0.800357	0.673207	-0.13616	-0.556	-0.40237
aPF14_0220_0	PF14_0220	0.800357	0.673207	-0.13616	-0.556	-0.40237
aPF14_0219_0	PF14_0220	0.831327	0.198067	-0.47981	-0.54097	-0.07685
intPF14_0221_2	PF14_0221	0.70239	0.62256	0.63989	-0.11789	-1.16906
aPF14_0221_1	PF14_0221	0.70239	0.62256	0.63989	-0.11789	-1.16906
aPF14_0221_0	PF14_0221	0.70239	0.62256	0.63989	-0.11789	-1.16906
intPF14_0222_2	PF14_0222	0.534067	-0.17685	-1.37403	-1.28763	0.291687
aPF14_0222_1	PF14_0222	0.534067	-0.17685	-1.37403	-1.28763	0.291687
aPF14_0222_0	PF14_0222	0.534067	-0.17685	-1.37403	-1.28763	0.291687
intPF14_0223_2	PF14_0223	0.287427	0.831847	0.441307	-0.11347	-0.52051
aPF14_0223_1	PF14_0223	0.287427	0.831847	0.441307	-0.11347	-0.52051
aPF14_0223_0	PF14_0223	0.287427	0.831847	0.441307	-0.11347	-0.52051
intPF14_0225_2	PF14_0224	-0.16699	-0.35206	-0.72638	-0.71396	0.081723
intPF14_0224_2	PF14_0224	0.5692	-0.34799	-1.85752	-1.64148	0.52149
aPF14_0224_1	PF14_0224	0.5692	-0.34799	-1.85752	-1.64148	0.52149
aPF14_0224_0	PF14_0224	0.5692	-0.34799	-1.85752	-1.64148	0.52149
aPF14_0225_2	PF14_0225	-0.16699	-0.35206	-0.72638	-0.71396	0.081723
aPF14_0225_1	PF14_0225	-0.16699	-0.35206	-0.72638	-0.71396	0.081723
aPF14_0225_0	PF14_0225	-0.16699	-0.35206	-0.72638	-0.71396	0.081723
intPF14_0226_2	PF14_0226	-0.42126	0.435292	-1.01693	-0.96574	0.846372
aPF14_0226_2	PF14_0226	-0.42126	0.435292	-1.01693	-0.96574	0.846372
aPF14_0226_1	PF14_0226	-0.42126	0.435292	-1.01693	-0.96574	0.846372
aPF14_0226_0	PF14_0226	-0.42126	0.435292	-1.01693	-0.96574	0.846372
intPF14_0227_2	PF14_0227	-1.12822	-0.14387	1.058237	0.884097	-0.33379
aPF14_0227_0	PF14_0227	-1.12822	-0.14387	1.058237	0.884097	-0.33379
intPF14_0228_2	PF14_0228	0.020405	0.145985	0.470025	0.245085	-0.40355
aPF14_0228_2	PF14_0228	0.020405	0.145985	0.470025	0.245085	-0.40355
aPF14_0228_1	PF14_0228	0.020405	0.145985	0.470025	0.245085	-0.40355
aPF14_0228_0	PF14_0228	0.020405	0.145985	0.470025	0.245085	-0.40355
intPF14_0229_2	PF14_0229	0.721647	0.448777	0.116197	-0.23436	-0.50594
aPF14_0229_1	PF14_0229	0.721647	0.448777	0.116197	-0.23436	-0.50594
aPF14_0229_0	PF14_0229	0.721647	0.448777	0.116197	-0.23436	-0.50594
intPF14_0230_3	PF14_0230	-0.04075	0.376787	0.766977	0.488457	-0.40865
aPF14_0230_0	PF14_0230	-0.04075	0.376787	0.766977	0.488457	-0.40865
intPF14_0231_2	PF14_0231	0.06803	0.58084	0.78882	0.37118	-0.52484
aPF14_0231_0	PF14_0231	0.06803	0.58084	0.78882	0.37118	-0.52484
intPF14_0232_2	PF14_0232	-0.64701	-0.09996	0.70142	0.74858	-0.01404
aPF14_0232_1	PF14_0232	-0.64701	-0.09996	0.70142	0.74858	-0.01404
aPF14_0232_0	PF14_0232	-0.64701	-0.09996	0.70142	0.74858	-0.01404
intPF14_0234_2	PF14_0233	0.163067	-0.38885	-0.27278	-0.36788	-0.36531
intPF14_0233_2	PF14_0233	-0.3825	-1.02612	-0.83782	0.017783	1.006083
aPF14_0233_0	PF14_0233	-0.3825	-1.02612	-0.83782	0.017783	1.006083
intPF14_0235_2	PF14_0234					
aPF14_0234_1	PF14_0234	0.163067	-0.38885	-0.27278	-0.36788	-0.36531
aPF14_0234_0	PF14_0234	0.163067	-0.38885	-0.27278	-0.36788	-0.36531
aPF14_0235_0	PF14_0235					
intPF14_0236_2	PF14_0236	-0.06248	-0.14294	0.023543	0.092233	0.020713

aPF14_0236_2	PF14_0236	-0.06248	-0.14294	0.023543	0.092233	0.020713
aPF14_0236_1	PF14_0236	-0.06248	-0.14294	0.023543	0.092233	0.020713
aPF14_0236_0	PF14_0236	-0.06248	-0.14294	0.023543	0.092233	0.020713
intPF14_0237_2	PF14_0237	0.234933	0.764833	-0.06143	-0.40691	-0.06617
aPF14_0237_1	PF14_0237	0.234933	0.764833	-0.06143	-0.40691	-0.06617
aPF14_0237_0	PF14_0237	0.234933	0.764833	-0.06143	-0.40691	-0.06617
intPF14_0238_2	PF14_0238	-0.65296	-0.42072	-0.81382	-0.07352	1.318883
aPF14_0238_1	PF14_0238	-0.65296	-0.42072	-0.81382	-0.07352	1.318883
aPF14_0238_0	PF14_0238	-0.65296	-0.42072	-0.81382	-0.07352	1.318883
intPF14_0239_2	PF14_0239	0.380078	0.678608	1.134158	0.172758	-1.49444
aPF14_0239_0	PF14_0239	0.380078	0.678608	1.134158	0.172758	-1.49444
intPF14_0240_2	PF14_0240	0.301417	0.344917	0.633167	0.391167	-0.44458
aPF14_0240_0	PF14_0240	0.301417	0.344917	0.633167	0.391167	-0.44458
intPF14_0241_3	PF14_0241	0.549408	0.820908	0.822648	0.046068	-1.07494
aPF14_0241_0	PF14_0241	0.549408	0.820908	0.822648	0.046068	-1.07494
intPF14_0242_2	PF14_0242	-0.52379	0.39545	0.93335	0.56565	-0.42445
aPF14_0242_1	PF14_0242	-0.52379	0.39545	0.93335	0.56565	-0.42445
aPF14_0242_0	PF14_0242	-0.52379	0.39545	0.93335	0.56565	-0.42445
aPF14_0243_1	PF14_0243a	-0.0858	-0.28649	-0.64827	-0.17573	0.76249
aPF14_0243_0	PF14_0243b	-0.0858	-0.28649	-0.64827	-0.17573	0.76249
intPF14_0244_2	PF14_0244	0.303017	0.457917	0.103157	-0.17782	-0.25158
intPF14_0243_2	PF14_0244	-0.0858	-0.28649	-0.64827	-0.17573	0.76249
aPF14_0244_1	PF14_0244	0.303017	0.457917	0.103157	-0.17782	-0.25158
aPF14_0244_0	PF14_0244	0.303017	0.457917	0.103157	-0.17782	-0.25158
aPF14_0245_2	PF14_0245	-0.28135	-0.39227	-0.51398	0.076085	0.888705
aPF14_0245_1	PF14_0245	-0.28135	-0.39227	-0.51398	0.076085	0.888705
aPF14_0245_0	PF14_0245	-0.28135	-0.39227	-0.51398	0.076085	0.888705
intPF14_0246_2	PF14_0246	0.01003	0.03435	-0.8482	-0.5943	0.68315
intPF14_0245_2	PF14_0246	-0.28135	-0.39227	-0.51398	0.076085	0.888705
aPF14_0246_1	PF14_0246	0.01003	0.03435	-0.8482	-0.5943	0.68315
aPF14_0246_0	PF14_0246	0.01003	0.03435	-0.8482	-0.5943	0.68315
intPF14_0247_3	PF14_0247	0.763502	0.679232	0.139412	-0.49908	-0.8059
aPF14_0247_3	PF14_0247	0.763502	0.679232	0.139412	-0.49908	-0.8059
aPF14_0247_2	PF14_0247	0.763502	0.679232	0.139412	-0.49908	-0.8059
aPF14_0247_1	PF14_0247	0.763502	0.679232	0.139412	-0.49908	-0.8059
aPF14_0247_0	PF14_0247	0.763502	0.679232	0.139412	-0.49908	-0.8059
intPF14_0249_2	PF14_0248	-0.63905	-0.63773	-0.29404	0.30754	0.78823
aPF14_0248_0	PF14_0248	-0.95759	-1.58009	-0.45434	0.858003	1.385753
intPF14_0248_2	PF14_0249	-0.95759	-1.58009	-0.45434	0.858003	1.385753
aPF14_0249_1	PF14_0249	-0.63905	-0.63773	-0.29404	0.30754	0.78823
aPF14_0249_0	PF14_0249	-0.63905	-0.63773	-0.29404	0.30754	0.78823
intPF14_0250_2	PF14_0250	1.608633	1.354813	0.236373	-1.55421	-2.39765
aPF14_0250_1	PF14_0250	1.608633	1.354813	0.236373	-1.55421	-2.39765
aPF14_0250_0	PF14_0250	1.608633	1.354813	0.236373	-1.55421	-2.39765
intPF14_0251_2	PF14_0251	1.224793	0.004013	-1.53503	-1.07531	0.795653
aPF14_0251_0	PF14_0251	1.224793	0.004013	-1.53503	-1.07531	0.795653
intPF14_0252_2	PF14_0252	-0.84221	-0.78382	-0.27963	0.533685	1.071365

aPF14_0252_2	PF14_0252	-0.84221	-0.78382	-0.27963	0.533685	1.071365
aPF14_0252_1	PF14_0252	-0.84221	-0.78382	-0.27963	0.533685	1.071365
aPF14_0252_0	PF14_0252	-0.84221	-0.78382	-0.27963	0.533685	1.071365
intPF14_0253_2	PF14_0253-a	-0.99729	-0.27208	0.253663	0.590503	0.571743
aPF14_0253_1	PF14_0253-a	-0.99729	-0.27208	0.253663	0.590503	0.571743
aPF14_0253_0	PF14_0253-a	-0.99729	-0.27208	0.253663	0.590503	0.571743
intPF14_0254_2	PF14_0254	-1.14629	-1.15455	-0.00839	1.060727	1.218887
aPF14_0254_1	PF14_0254	-1.14629	-1.15455	-0.00839	1.060727	1.218887
aPF14_0254_0	PF14_0254	-1.14629	-1.15455	-0.00839	1.060727	1.218887
intPF14_0255_2	PF14_0255	-1.02215	-0.78024	0.27272	0.90378	0.67624
aPF14_0255_1	PF14_0255	-1.02215	-0.78024	0.27272	0.90378	0.67624
aPF14_0255_0	PF14_0255	-1.02215	-0.78024	0.27272	0.90378	0.67624
intPF14_0256_2	PF14_0256	0.490547	0.547247	0.845017	0.231317	-1.00091
aPF14_0256_0	PF14_0256	0.490547	0.547247	0.845017	0.231317	-1.00091
intPF14_0257_2	PF14_0257	-1.57411	-0.72844	0.22339	1.11861	1.30744
aPF14_0257_0	PF14_0257	-1.57411	-0.72844	0.22339	1.11861	1.30744
intPF14_0258_2	PF14_0258	-0.17574	-0.30979	-0.47842	-0.07058	0.62329
aPF14_0258_1	PF14_0258	-0.17574	-0.30979	-0.47842	-0.07058	0.62329
aPF14_0258_0	PF14_0258	-0.17574	-0.30979	-0.47842	-0.07058	0.62329
intPF14_0259_2	PF14_0259	0.316047	0.077787	-0.03556	-0.3561	-0.52245
aPF14_0259_1	PF14_0259	0.316047	0.077787	-0.03556	-0.3561	-0.52245
aPF14_0259_0	PF14_0259	0.316047	0.077787	-0.03556	-0.3561	-0.52245
intPF14_0260_2	PF14_0260	0.324383	0.691103	-0.28296	-0.86488	-0.42594
aPF14_0260_1	PF14_0260	0.324383	0.691103	-0.28296	-0.86488	-0.42594
aPF14_0260_0	PF14_0260	0.324383	0.691103	-0.28296	-0.86488	-0.42594
intPF14_0261_2	PF14_0261	0.223363	0.586663	0.729423	0.221743	-0.688
aPF14_0261_1	PF14_0261	0.223363	0.586663	0.729423	0.221743	-0.688
aPF14_0261_0	PF14_0261	0.223363	0.586663	0.729423	0.221743	-0.688
intPF14_0262_2	PF14_0262	0.091193	0.407023	0.154443	-0.11478	-0.21436
aPF14_0263_2	PF14_0262	-1.30246	-0.48373	-0.07455	0.435713	0.901893
aPF14_0263_1	PF14_0262	-1.30246	-0.48373	-0.07455	0.435713	0.901893
aPF14_0263_0	PF14_0262	-1.30246	-0.48373	-0.07455	0.435713	0.901893
aPF14_0262_0	PF14_0262	0.091193	0.407023	0.154443	-0.11478	-0.21436
intPF14_0265_2	PF14_0264	-1.20677	-0.92617	0.39484	1.04016	0.62117
intPF14_0264_2	PF14_0264	1.654925	1.247055	-0.80427	-1.6151	-0.66842
aPF14_0264_3	PF14_0264	1.654925	1.247055	-0.80427	-1.6151	-0.66842
aPF14_0264_2	PF14_0264	1.654925	1.247055	-0.80427	-1.6151	-0.66842
aPF14_0264_1	PF14_0264	1.654925	1.247055	-0.80427	-1.6151	-0.66842
aPF14_0264_0	PF14_0264	1.654925	1.247055	-0.80427	-1.6151	-0.66842
aPF14_0265_0	PF14_0265	-1.20677	-0.92617	0.39484	1.04016	0.62117
intPF14_0266_2	PF14_0266	-0.57975	-0.00521	0.704917	0.633417	-0.15245
aPF14_0266_1	PF14_0266	-0.57975	-0.00521	0.704917	0.633417	-0.15245
aPF14_0266_0	PF14_0266	-0.57975	-0.00521	0.704917	0.633417	-0.15245
intPF14_0267_3	PF14_0267	0.230997	0.070527	0.239687	0.176147	-0.20519
aPF14_0267_1	PF14_0267	0.230997	0.070527	0.239687	0.176147	-0.20519
aPF14_0267_0	PF14_0267	0.230997	0.070527	0.239687	0.176147	-0.20519
intPF14_0268_2	PF14_0268	-1.01119	-0.9737	-0.22551	0.69267	1.14037

aPF14_0268_2	PF14_0268	-1.01119	-0.9737	-0.22551	0.69267	1.14037
aPF14_0268_1	PF14_0268	-1.01119	-0.9737	-0.22551	0.69267	1.14037
aPF14_0268_0	PF14_0268	-1.01119	-0.9737	-0.22551	0.69267	1.14037
intPF14_0270_2	PF14_0269	-1.20522	-0.5999	-0.00871	0.679873	1.037563
aPF14_0269_0	PF14_0269	0.841063	0.912683	0.405633	-0.22897	-0.75902
intPF14_0269_2	PF14_0270	0.841063	0.912683	0.405633	-0.22897	-0.75902
aPF14_0270_1	PF14_0270	-1.20522	-0.5999	-0.00871	0.679873	1.037563
aPF14_0270_0	PF14_0270	-1.20522	-0.5999	-0.00871	0.679873	1.037563
intPF14_0271_2	PF14_0271	0.30734	0.3033	0.08664	-0.08114	-0.1808
aPF14_0271_1	PF14_0271	0.30734	0.3033	0.08664	-0.08114	-0.1808
aPF14_0271_0	PF14_0271	0.30734	0.3033	0.08664	-0.08114	-0.1808
intPF14_0272_2	PF14_0272	0.734883	0.468593	0.274313	-0.12765	-0.62693
aPF14_0272_0	PF14_0272	0.734883	0.468593	0.274313	-0.12765	-0.62693
intPF14_0274_2	PF14_0273	1.362477	1.283117	0.729017	-0.58968	-1.81978
intPF14_0273_2	PF14_0273	0.41795	1.21594	0.85337	-0.11657	-1.06804
aPF14_0273_4	PF14_0273	0.41795	1.21594	0.85337	-0.11657	-1.06804
aPF14_0273_3	PF14_0273	0.41795	1.21594	0.85337	-0.11657	-1.06804
aPF14_0273_2	PF14_0273	0.41795	1.21594	0.85337	-0.11657	-1.06804
aPF14_0273_1	PF14_0273	0.41795	1.21594	0.85337	-0.11657	-1.06804
aPF14_0273_0	PF14_0273	0.41795	1.21594	0.85337	-0.11657	-1.06804
aPF14_0274_1	PF14_0274	1.362477	1.283117	0.729017	-0.58968	-1.81978
aPF14_0274_0	PF14_0274	1.362477	1.283117	0.729017	-0.58968	-1.81978
intPF14_0275_2	PF14_0275	-0.18291	-0.27041	-0.12016	0.256497	0.485747
aPF14_0275_0	PF14_0275	-0.18291	-0.27041	-0.12016	0.256497	0.485747
intPF14_0276_2	PF14_0276	-0.04453	-0.01687	0.375993	0.399173	-0.08697
aPF14_0276_1	PF14_0276	-0.04453	-0.01687	0.375993	0.399173	-0.08697
aPF14_0276_0	PF14_0276	-0.04453	-0.01687	0.375993	0.399173	-0.08697
intPF14_0277_3	PF14_0277	0.114367	0.309177	-0.3176	-0.31606	0.276157
aPF14_0277_2	PF14_0277	0.114367	0.309177	-0.3176	-0.31606	0.276157
aPF14_0277_1	PF14_0277	0.114367	0.309177	-0.3176	-0.31606	0.276157
aPF14_0277_0	PF14_0277	0.114367	0.309177	-0.3176	-0.31606	0.276157
intPF14_0278_2	PF14_0278	-0.02402	0.181742	-0.17849	-0.21529	0.133482
aPF14_0278_2	PF14_0278	-0.02402	0.181742	-0.17849	-0.21529	0.133482
aPF14_0278_1	PF14_0278	-0.02402	0.181742	-0.17849	-0.21529	0.133482
aPF14_0278_0	PF14_0278	-0.02402	0.181742	-0.17849	-0.21529	0.133482
intPF14_0279_2	PF14_0279	-0.16716	0.200773	0.254453	-0.00879	-0.29011
aPF14_0279_0	PF14_0279	-0.16716	0.200773	0.254453	-0.00879	-0.29011
intPF14_0280_2	PF14_0280	-1.36602	-0.38489	0.862952	0.905232	-0.02548
aPF14_0280_0	PF14_0280	-1.36602	-0.38489	0.862952	0.905232	-0.02548
intPF14_0281_2	PF14_0281	0.191467	-0.22784	-1.63781	-1.20185	0.984177
aPF14_0281_1	PF14_0281	0.191467	-0.22784	-1.63781	-1.20185	0.984177
aPF14_0281_0	PF14_0281	0.191467	-0.22784	-1.63781	-1.20185	0.984177
intPF14_0282_2	PF14_0282	0.961102	0.665812	0.140542	-0.33021	-0.62948
aPF14_0282_3	PF14_0282	0.961102	0.665812	0.140542	-0.33021	-0.62948
aPF14_0282_2	PF14_0282	0.961102	0.665812	0.140542	-0.33021	-0.62948
aPF14_0282_1	PF14_0282	0.961102	0.665812	0.140542	-0.33021	-0.62948
aPF14_0282_0	PF14_0282	0.961102	0.665812	0.140542	-0.33021	-0.62948

intPF14_0284_2	PF14_0283	-0.49523	-0.24217	-0.49832	-0.10212	0.764025
intPF14_0283_2	PF14_0283	0.47779	0.53105	0.2014	-0.0874	-0.30555
aPF14_0283_1	PF14_0283	0.47779	0.53105	0.2014	-0.0874	-0.30555
aPF14_0283_0	PF14_0283	0.47779	0.53105	0.2014	-0.0874	-0.30555
aPF14_0284_1	PF14_0284	-0.49523	-0.24217	-0.49832	-0.10212	0.764025
aPF14_0284_0	PF14_0284	-0.49523	-0.24217	-0.49832	-0.10212	0.764025
intPF14_0285_2	PF14_0285	-0.0662	-1.28948	0.039477	0.792857	0.279317
aPF14_0285_1	PF14_0285	-0.0662	-1.28948	0.039477	0.792857	0.279317
aPF14_0285_0	PF14_0285	-0.0662	-1.28948	0.039477	0.792857	0.279317
intPF14_0286_2	PF14_0286	-1.08785	0.441277	0.274947	0.215887	0.476557
aPF14_0286_1	PF14_0286	-1.08785	0.441277	0.274947	0.215887	0.476557
aPF14_0286_0	PF14_0286	-1.08785	0.441277	0.274947	0.215887	0.476557
intPF14_0287_2	PF14_0287	-0.6453	0.119527	-0.6093	-0.42764	0.770367
aPF14_0287_0	PF14_0287	-0.6453	0.119527	-0.6093	-0.42764	0.770367
intPF14_0288_2	PF14_0288	-0.33686	-0.87374	-0.43804	0.319703	0.812403
aPF14_0288_0	PF14_0288	-0.33686	-0.87374	-0.43804	0.319703	0.812403
intPF14_0289_2	PF14_0289	-1.10317	-0.96712	0.462757	1.120577	0.555457
aPF14_0289_0	PF14_0289	-1.10317	-0.96712	0.462757	1.120577	0.555457
aPF14_0290_0	PF14_0290	0.182877	0.092607	-0.18228	-0.36914	-0.21875
intPF14_0291_3	PF14_0291	1.483707	0.900457	0.199837	-0.972	-1.71862
aPF14_0291_1	PF14_0291	1.483707	0.900457	0.199837	-0.972	-1.71862
aPF14_0291_0	PF14_0291	1.483707	0.900457	0.199837	-0.972	-1.71862
intPF14_0292_2	PF14_0292	0.716653	0.771213	0.544813	-0.44365	-1.38905
aPF14_0292_1	PF14_0292	0.716653	0.771213	0.544813	-0.44365	-1.38905
aPF14_0292_0	PF14_0292	0.716653	0.771213	0.544813	-0.44365	-1.38905
intPF14_0293_2	PF14_0293	-0.69129	-0.52551	-2.06797	-1.26969	1.766847
aPF14_0293_1	PF14_0293	-0.69129	-0.52551	-2.06797	-1.26969	1.766847
aPF14_0293_0	PF14_0293	-0.69129	-0.52551	-2.06797	-1.26969	1.766847
intPF14_0294_2	PF14_0294	-0.8788	-0.56999	0.17548	0.51652	0.36599
aPF14_0294_1	PF14_0294	-0.8788	-0.56999	0.17548	0.51652	0.36599
aPF14_0294_0	PF14_0294	-0.8788	-0.56999	0.17548	0.51652	0.36599
intPF14_0295_2	PF14_0295	-0.72166	-0.9482	-0.41961	0.540773	1.188863
aPF14_0295_1	PF14_0295	-0.72166	-0.9482	-0.41961	0.540773	1.188863
aPF14_0295_0	PF14_0295	-0.72166	-0.9482	-0.41961	0.540773	1.188863
intPF14_0296_1	PF14_0296	0.10363	0.31185	0.76881	0.51419	-0.46485
aPF14_0296_0	PF14_0296	0.10363	0.31185	0.76881	0.51419	-0.46485
intPF14_0297_2	PF14_0297	-0.73396	-0.20868	0.775357	0.799977	-0.11598
aPF14_0297_1	PF14_0297	-0.73396	-0.20868	0.775357	0.799977	-0.11598
aPF14_0297_0	PF14_0297	-0.73396	-0.20868	0.775357	0.799977	-0.11598
intPF14_0298_2	PF14_0298	-0.21115	-0.61027	-1.25547	-0.58287	1.090933
aPF14_0299_0	PF14_0298					
aPF14_0298_0	PF14_0298	-0.21115	-0.61027	-1.25547	-0.58287	1.090933
intPF14_0300_3	PF14_0300	-0.67727	-0.45064	0.168037	0.615797	0.540977
aPF14_0300_1	PF14_0300	-0.67727	-0.45064	0.168037	0.615797	0.540977
aPF14_0300_0	PF14_0300	-0.67727	-0.45064	0.168037	0.615797	0.540977
intPF14_0301_2	PF14_0301	-0.31696	-0.4262	0.413893	0.662773	0.056863
aPF14_0301_0	PF14_0301	-0.31696	-0.4262	0.413893	0.662773	0.056863

intPF14_0303_2	PF14_0303	-0.17851	-0.34691	-1.62368	-0.88248	1.481247
intPF14_0302_3	PF14_0303	-0.2964	-0.30604	-1.89043	-1.11591	1.686703
aPF14_0303_1	PF14_0303	-0.17851	-0.34691	-1.62368	-0.88248	1.481247
aPF14_0303_0	PF14_0303	-0.17851	-0.34691	-1.62368	-0.88248	1.481247
aPF14_0302_1	PF14_0303	-0.2964	-0.30604	-1.89043	-1.11591	1.686703
aPF14_0302_0	PF14_0303	-0.2964	-0.30604	-1.89043	-1.11591	1.686703
intPF14_0304_2	PF14_0304	0.17389	0.05107	-0.53238	-0.45512	0.26943
aPF14_0304_2	PF14_0304	0.17389	0.05107	-0.53238	-0.45512	0.26943
aPF14_0304_1	PF14_0304	0.17389	0.05107	-0.53238	-0.45512	0.26943
aPF14_0304_0	PF14_0304	0.17389	0.05107	-0.53238	-0.45512	0.26943
intPF14_0305_2	PF14_0305	-0.68505	-0.44822	-0.02655	0.441887	0.641057
aPF14_0306_0	PF14_0305	-0.507	-0.604	-0.3215	0.4455	1.024
aPF14_0305_1	PF14_0305	-0.68505	-0.44822	-0.02655	0.441887	0.641057
aPF14_0305_0	PF14_0305	-0.68505	-0.44822	-0.02655	0.441887	0.641057
intPF14_0307_2	PF14_0307	0.89771	0.97194	0.55737	-0.36087	-1.21444
aPF14_0307_1	PF14_0307	0.89771	0.97194	0.55737	-0.36087	-1.21444
aPF14_0307_0	PF14_0307	0.89771	0.97194	0.55737	-0.36087	-1.21444
intPF14_0308_2	PF14_0308	0.641333	0.318843	0.088563	-0.0824	-0.28718
aPF14_0308_1	PF14_0308	0.641333	0.318843	0.088563	-0.0824	-0.28718
aPF14_0308_0	PF14_0308	0.641333	0.318843	0.088563	-0.0824	-0.28718
intPF14_0309_2	PF14_0309	-1.37323	-0.59883	-0.07834	0.618677	1.130167
aPF14_0309_0	PF14_0309	-1.37323	-0.59883	-0.07834	0.618677	1.130167
intPF14_0310_2	PF14_0310	-1.33493	-0.89734	0.19392	0.90708	0.85184
aPF14_0310_1	PF14_0310	-1.33493	-0.89734	0.19392	0.90708	0.85184
aPF14_0310_0	PF14_0310	-1.33493	-0.89734	0.19392	0.90708	0.85184
intPF14_0311_2	PF14_0311	-0.02548	-0.54956	-0.93938	-0.32828	0.871897
aPF14_0311_0	PF14_0311	-0.02548	-0.54956	-0.93938	-0.32828	0.871897
aPF14_0312_1	PF14_0312	0.11202	-0.25758	0.06041	0.31059	0.14608
aPF14_0312_0	PF14_0312	0.11202	-0.25758	0.06041	0.31059	0.14608
intPF14_0313_2	PF14_0313	-0.59859	-0.32256	0.154353	0.264813	0.098723
aPF14_0313_1	PF14_0313	-0.59859	-0.32256	0.154353	0.264813	0.098723
aPF14_0313_0	PF14_0313	-0.59859	-0.32256	0.154353	0.264813	0.098723
intPF14_0314_2	PF14_0314	-0.46031	-0.73445	-0.31937	0.200533	0.550113
aPF14_0314_1	PF14_0314	-0.46031	-0.73445	-0.31937	0.200533	0.550113
aPF14_0314_0	PF14_0314	-0.46031	-0.73445	-0.31937	0.200533	0.550113
intPF14_0315_3	PF14_0315	-1.29908	-0.92445	-0.20419	0.660482	1.181802
aPF14_0315_8	PF14_0315	-1.29908	-0.92445	-0.20419	0.660482	1.181802
aPF14_0315_7	PF14_0315	-1.29908	-0.92445	-0.20419	0.660482	1.181802
aPF14_0315_6	PF14_0315	-1.29908	-0.92445	-0.20419	0.660482	1.181802
aPF14_0315_5	PF14_0315	-1.29908	-0.92445	-0.20419	0.660482	1.181802
aPF14_0315_4	PF14_0315	-1.29908	-0.92445	-0.20419	0.660482	1.181802
aPF14_0315_3	PF14_0315	-1.29908	-0.92445	-0.20419	0.660482	1.181802
aPF14_0315_2	PF14_0315	-1.29908	-0.92445	-0.20419	0.660482	1.181802
aPF14_0315_1	PF14_0315	-1.29908	-0.92445	-0.20419	0.660482	1.181802
aPF14_0315_0	PF14_0315	-1.29908	-0.92445	-0.20419	0.660482	1.181802
intPF14_0316_2	PF14_0316	1.016403	0.564303	-0.58394	-1.14973	-0.61664
aPF14_0316_2	PF14_0316	1.016403	0.564303	-0.58394	-1.14973	-0.61664

aPF14_0316_1	PF14_0316	1.016403	0.564303	-0.58394	-1.14973	-0.61664
aPF14_0316_0	PF14_0316	1.016403	0.564303	-0.58394	-1.14973	-0.61664
intPF14_0317_2	PF14_0317	-1.01017	-1.47517	0.158833	1.269833	0.966833
aPF14_0317_0	PF14_0317	-1.01017	-1.47517	0.158833	1.269833	0.966833
intPF14_0318_2	PF14_0318	-0.85605	-0.54172	0.154697	0.621637	0.573057
aPF14_0318_2	PF14_0318	-0.85605	-0.54172	0.154697	0.621637	0.573057
aPF14_0318_1	PF14_0318	-0.85605	-0.54172	0.154697	0.621637	0.573057
aPF14_0318_0	PF14_0318	-0.85605	-0.54172	0.154697	0.621637	0.573057
aPF14_0319_4	PF14_0319	-0.99839	-0.52318	-0.52658	0.188367	1.231967
aPF14_0319_3	PF14_0319	-0.99839	-0.52318	-0.52658	0.188367	1.231967
aPF14_0319_2	PF14_0319	-0.99839	-0.52318	-0.52658	0.188367	1.231967
aPF14_0319_1	PF14_0319	-0.99839	-0.52318	-0.52658	0.188367	1.231967
aPF14_0319_0	PF14_0319	-0.99839	-0.52318	-0.52658	0.188367	1.231967
intPF14_0320_2	PF14_0320	-0.30213	-0.19721	-0.28684	0.010172	0.510372
intPF14_0319_2	PF14_0320	-0.99839	-0.52318	-0.52658	0.188367	1.231967
aPF14_0320_2	PF14_0320	-0.30213	-0.19721	-0.28684	0.010172	0.510372
aPF14_0320_1	PF14_0320	-0.30213	-0.19721	-0.28684	0.010172	0.510372
aPF14_0320_0	PF14_0320	-0.30213	-0.19721	-0.28684	0.010172	0.510372
intPF14_0322_2	PF14_0321	0.64359	0.84105	0.97739	0.01511	-1.42305
aPF14_0321_0	PF14_0321	0.587977	0.690117	0.670767	0.058567	-0.87778
intPF14_0321_2	PF14_0322	0.587977	0.690117	0.670767	0.058567	-0.87778
aPF14_0322_1	PF14_0322	0.64359	0.84105	0.97739	0.01511	-1.42305
aPF14_0322_0	PF14_0322	0.64359	0.84105	0.97739	0.01511	-1.42305
intPF14_0323_2	PF14_0323	-1.0835	-0.6325	-0.115	0.624337	1.086837
aPF14_0323_0	PF14_0323	-1.0835	-0.6325	-0.115	0.624337	1.086837
intPF14_0324_2	PF14_0324	-0.37392	0.31808	0.90833	0.52467	-0.54158
aPF14_0324_1	PF14_0324	-0.37392	0.31808	0.90833	0.52467	-0.54158
aPF14_0324_0	PF14_0324	-0.37392	0.31808	0.90833	0.52467	-0.54158
aPF14_0325_0	PF14_0325	0.417123	0.054363	-0.59823	-0.76911	-0.1597
intPF14_0325_2	PF14_0326	0.417123	0.054363	-0.59823	-0.76911	-0.1597
aPF14_0326_9	PF14_0326	0.550178	0.509788	0.679078	-0.08131	-1.21048
aPF14_0326_8	PF14_0326	0.550178	0.509788	0.679078	-0.08131	-1.21048
aPF14_0326_7	PF14_0326	0.550178	0.509788	0.679078	-0.08131	-1.21048
aPF14_0326_6	PF14_0326	0.550178	0.509788	0.679078	-0.08131	-1.21048
aPF14_0326_5	PF14_0326	0.550178	0.509788	0.679078	-0.08131	-1.21048
aPF14_0326_4	PF14_0326	0.550178	0.509788	0.679078	-0.08131	-1.21048
aPF14_0326_3	PF14_0326	0.550178	0.509788	0.679078	-0.08131	-1.21048
aPF14_0326_2	PF14_0326	0.550178	0.509788	0.679078	-0.08131	-1.21048
aPF14_0326_12	PF14_0326	0.550178	0.509788	0.679078	-0.08131	-1.21048
aPF14_0326_11	PF14_0326	0.550178	0.509788	0.679078	-0.08131	-1.21048
aPF14_0326_10	PF14_0326	0.550178	0.509788	0.679078	-0.08131	-1.21048
aPF14_0326_1	PF14_0326	0.550178	0.509788	0.679078	-0.08131	-1.21048
aPF14_0326_0	PF14_0326	0.550178	0.509788	0.679078	-0.08131	-1.21048
intPF14_0327_2	PF14_0327	0.587057	0.335227	0.597057	0.037277	-1.01089
intPF14_0326_2	PF14_0327	0.550178	0.509788	0.679078	-0.08131	-1.21048
aPF14_0327_1	PF14_0327	0.587057	0.335227	0.597057	0.037277	-1.01089
aPF14_0327_0	PF14_0327	0.587057	0.335227	0.597057	0.037277	-1.01089

aPF14_0328_0	PF14_0328	-1.01099	-0.26502	1.576037	1.308297	-0.76664
intPF14_0330_2	PF14_0329	-1.0183	-0.82854	0.342063	0.871283	0.466863
intPF14_0329_2	PF14_0329	-1.36128	-1.15561	1.53172	1.95028	-0.19939
aPF14_0329_0	PF14_0329	-1.36128	-1.15561	1.53172	1.95028	-0.19939
aPF14_0330_0	PF14_0330	-1.0183	-0.82854	0.342063	0.871283	0.466863
intPF14_0331_2	PF14_0331	-0.97896	-0.99221	0.226413	0.922753	0.633873
aPF14_0331_1	PF14_0331	-0.97896	-0.99221	0.226413	0.922753	0.633873
aPF14_0331_0	PF14_0331	-0.97896	-0.99221	0.226413	0.922753	0.633873
intPF14_0332_2	PF14_0332	-0.86928	-0.99859	0.22468	0.94432	0.62859
aPF14_0332_0	PF14_0332	-0.86928	-0.99859	0.22468	0.94432	0.62859
intPF14_0333_2	PF14_0333	-0.12136	-0.14417	-0.37715	-0.39051	0.050007
aPF14_0333_1	PF14_0333	-0.12136	-0.14417	-0.37715	-0.39051	0.050007
aPF14_0333_0	PF14_0333	-0.12136	-0.14417	-0.37715	-0.39051	0.050007
intPF14_0334_2	PF14_0334	-0.01551	-0.26362	-1.21222	-0.77816	0.884588
aPF14_0334_4	PF14_0334	-0.01551	-0.26362	-1.21222	-0.77816	0.884588
aPF14_0334_3	PF14_0334	-0.01551	-0.26362	-1.21222	-0.77816	0.884588
aPF14_0334_2	PF14_0334	-0.01551	-0.26362	-1.21222	-0.77816	0.884588
aPF14_0334_1	PF14_0334	-0.01551	-0.26362	-1.21222	-0.77816	0.884588
aPF14_0334_0	PF14_0334	-0.01551	-0.26362	-1.21222	-0.77816	0.884588
intPF14_0335_3	PF14_0335	-0.17356	-0.10918	0.53937	0.59263	-0.11482
aPF14_0335_0	PF14_0335	-0.17356	-0.10918	0.53937	0.59263	-0.11482
intPF14_0336_2	PF14_0336	-0.15962	-0.08991	0.015813	0.210853	0.280573
aPF14_0336_0	PF14_0336	-0.15962	-0.08991	0.015813	0.210853	0.280573
intPF14_0337_2	PF14_0337	0.538965	-0.14715	-2.16389	-1.87157	0.882035
aPF14_0337_2	PF14_0337	0.538965	-0.14715	-2.16389	-1.87157	0.882035
aPF14_0337_1	PF14_0337	0.538965	-0.14715	-2.16389	-1.87157	0.882035
aPF14_0337_0	PF14_0337	0.538965	-0.14715	-2.16389	-1.87157	0.882035
aPF14_0338_1	PF14_0338-a	-1.47446	-0.8032	0.5314	1.0316	0.5292
aPF14_0338_0	PF14_0338-a	-1.47446	-0.8032	0.5314	1.0316	0.5292
intPF14_0338_2	PF14_0338-b	-1.47446	-0.8032	0.5314	1.0316	0.5292
intPF14_0339_2	PF14_0339	-1.08054	-0.54682	0.80139	1.01861	0.05432
aPF14_0339_1	PF14_0339	-1.08054	-0.54682	0.80139	1.01861	0.05432
aPF14_0339_0	PF14_0339	-1.08054	-0.54682	0.80139	1.01861	0.05432
intPF14_0340_2	PF14_0340	-0.84532	-0.69637	0.432247	0.929087	0.418707
aPF14_0340_0	PF14_0340	-0.84532	-0.69637	0.432247	0.929087	0.418707
intPF14_0341_2	PF14_0341	-0.84703	0.479143	1.622473	0.965193	-0.92498
aPF14_0341_1	PF14_0341	-0.84703	0.479143	1.622473	0.965193	-0.92498
aPF14_0341_0	PF14_0341	-0.84703	0.479143	1.622473	0.965193	-0.92498
intPF14_0342_3	PF14_0342	0.36276	0.38853	-0.12806	-0.27494	-0.03953
aPF14_0342_2	PF14_0342	0.36276	0.38853	-0.12806	-0.27494	-0.03953
aPF14_0342_1	PF14_0342	0.36276	0.38853	-0.12806	-0.27494	-0.03953
aPF14_0342_0	PF14_0342	0.36276	0.38853	-0.12806	-0.27494	-0.03953
intPF14_0343_2	PF14_0343	0.311723	-0.4776	-1.30364	-0.83214	0.719483
aPF14_0343_3	PF14_0343	0.311723	-0.4776	-1.30364	-0.83214	0.719483
aPF14_0343_2	PF14_0343	0.311723	-0.4776	-1.30364	-0.83214	0.719483
aPF14_0343_1	PF14_0343	0.311723	-0.4776	-1.30364	-0.83214	0.719483
aPF14_0343_0	PF14_0343	0.311723	-0.4776	-1.30364	-0.83214	0.719483

intPF14_0344_2	PF14_0344	1.041897	1.208497	0.554257	-1.28392	-2.48366
aPF14_0344_1	PF14_0344	1.041897	1.208497	0.554257	-1.28392	-2.48366
aPF14_0344_0	PF14_0344	1.041897	1.208497	0.554257	-1.28392	-2.48366
intPF14_0345_2	PF14_0345a	0.404007	0.127777	0.404867	0.164637	-0.54477
aPF14_0345_1	PF14_0345a	0.404007	0.127777	0.404867	0.164637	-0.54477
aPF14_0345_0	PF14_0345a	0.404007	0.127777	0.404867	0.164637	-0.54477
aPF14_0345_2	PF14_0345b	0.404007	0.127777	0.404867	0.164637	-0.54477
intPF14_0346_2	PF14_0346	0.062273	-0.34385	-1.69005	-1.08829	1.216013
aPF14_0346_1	PF14_0346	0.062273	-0.34385	-1.69005	-1.08829	1.216013
aPF14_0346_0	PF14_0346	0.062273	-0.34385	-1.69005	-1.08829	1.216013
aPF14_0347_1	PF14_0347	-0.65818	-0.58461	-0.01354	0.527203	0.645273
aPF14_0347_0	PF14_0347	-0.65818	-0.58461	-0.01354	0.527203	0.645273
intPF14_0347_2	PF14_0348	-0.65818	-0.58461	-0.01354	0.527203	0.645273
aPF14_0348_0	PF14_0348	-0.59521	-0.65999	-0.03653	0.592193	0.713653
intPF14_0349_2	PF14_0349	0.863537	0.526297	0.245397	-0.30406	-0.84296
intPF14_0348_2	PF14_0349	-0.59521	-0.65999	-0.03653	0.592193	0.713653
aPF14_0349_0	PF14_0349	0.863537	0.526297	0.245397	-0.30406	-0.84296
intPF14_0350_3	PF14_0350	0.037633	0.118823	-0.0404	-0.08094	0.019343
aPF14_0350_1	PF14_0350	0.037633	0.118823	-0.0404	-0.08094	0.019343
aPF14_0350_0	PF14_0350	0.037633	0.118823	-0.0404	-0.08094	0.019343
intPF14_0351_2	PF14_0351	-0.65501	-0.96596	-0.52608	0.53408	1.33996
aPF14_0351_0	PF14_0351	-0.65501	-0.96596	-0.52608	0.53408	1.33996
intPF14_0352_2	PF14_0352	-1.59842	-1.50841	-0.54119	0.88269	1.84341
aPF14_0352_1	PF14_0352	-1.59842	-1.50841	-0.54119	0.88269	1.84341
aPF14_0352_0	PF14_0352	-1.59842	-1.50841	-0.54119	0.88269	1.84341
intPF14_0353_2	PF14_0353	-0.34654	-0.72031	-1.60175	-0.75973	1.432262
aPF14_0353_0	PF14_0353	-0.34654	-0.72031	-1.60175	-0.75973	1.432262
intPF14_0354_2	PF14_0354	0.023173	0.304123	0.291743	0.150923	-0.11346
aPF14_0354_1	PF14_0354	0.023173	0.304123	0.291743	0.150923	-0.11346
aPF14_0354_0	PF14_0354	0.023173	0.304123	0.291743	0.150923	-0.11346
intPF14_0355_3	PF14_0355	0.433142	0.183382	-0.3328	-0.30825	0.125642
aPF14_0355_0	PF14_0355	0.433142	0.183382	-0.3328	-0.30825	0.125642
intPF14_0356_2	PF14_0356	0.20536	-0.01079	0.61307	0.43893	-0.54121
aPF14_0356_1	PF14_0356	0.20536	-0.01079	0.61307	0.43893	-0.54121
aPF14_0356_0	PF14_0356	0.20536	-0.01079	0.61307	0.43893	-0.54121
intPF14_0357_2	PF14_0357	-0.48267	-0.64264	-0.8382	0.033537	1.319477
aPF14_0357_1	PF14_0357	-0.48267	-0.64264	-0.8382	0.033537	1.319477
aPF14_0357_0	PF14_0357	-0.48267	-0.64264	-0.8382	0.033537	1.319477
intPF14_0358_2	PF14_0358	-0.44187	-0.37599	0.2331	0.43802	0.12633
aPF14_0358_0	PF14_0358	-0.44187	-0.37599	0.2331	0.43802	0.12633
intPF14_0359_3	PF14_0359					
aPF14_0359_0	PF14_0359					
intPF14_0360_2	PF14_0360	0.32593	0.55833	0.3761	-0.1146	-0.59783
aPF14_0360_1	PF14_0360	0.32593	0.55833	0.3761	-0.1146	-0.59783
aPF14_0360_0	PF14_0360	0.32593	0.55833	0.3761	-0.1146	-0.59783
intPF14_0361_2	PF14_0361	0.071393	0.136053	-0.37311	-0.29623	0.306613
aPF14_0361_0	PF14_0361	0.071393	0.136053	-0.37311	-0.29623	0.306613

intPF14_0363_2	PF14_0363	-1.20077	-0.56318	-0.08432	0.116983	0.354843
intPF14_0362_2	PF14_0363	-0.77596	-0.05418	-0.07264	-0.24036	-0.00482
aPF14_0363_2	PF14_0363	-1.20077	-0.56318	-0.08432	0.116983	0.354843
aPF14_0363_1	PF14_0363	-1.20077	-0.56318	-0.08432	0.116983	0.354843
aPF14_0363_0	PF14_0363	-1.20077	-0.56318	-0.08432	0.116983	0.354843
aPF14_0362_0	PF14_0363	-0.77596	-0.05418	-0.07264	-0.24036	-0.00482
intPF14_0364_2	PF14_0364	1.013417	0.761907	0.431687	-0.32785	-1.11307
aPF14_0364_1	PF14_0364	1.013417	0.761907	0.431687	-0.32785	-1.11307
aPF14_0364_0	PF14_0364	1.013417	0.761907	0.431687	-0.32785	-1.11307
intPF14_0365_2	PF14_0365	0.525047	0.320787	0.238437	-0.0741	-0.50945
aPF14_0365_0	PF14_0365	0.525047	0.320787	0.238437	-0.0741	-0.50945
intPF14_0366_2	PF14_0366	-0.65101	-0.79544	0.06413	0.76387	0.71494
aPF14_0366_1	PF14_0366	-0.65101	-0.79544	0.06413	0.76387	0.71494
aPF14_0366_0	PF14_0366	-0.65101	-0.79544	0.06413	0.76387	0.71494
intPF14_0367_2	PF14_0367	0.142842	0.057072	-0.03193	-0.07593	-0.06493
aPF14_0367_1	PF14_0367	0.142842	0.057072	-0.03193	-0.07593	-0.06493
aPF14_0367_0	PF14_0367	0.142842	0.057072	-0.03193	-0.07593	-0.06493
intPF14_0368_2	PF14_0368	-0.69318	0.17189	0.89071	0.78029	-0.11689
aPF14_0368_0	PF14_0368	-0.69318	0.17189	0.89071	0.78029	-0.11689
intPF14_0369_2	PF14_0369	-0.51131	-1.24147	-1.17906	0.20406	1.81047
aPF14_0369_0	PF14_0369	-0.51131	-1.24147	-1.17906	0.20406	1.81047
intPF14_0371_2	PF14_0370	0.486847	-0.27922	-0.06104	0.196377	0.058057
intPF14_0370_2	PF14_0370	0.771243	0.772923	0.282063	-0.27723	-0.67476
aPF14_0370_3	PF14_0370	0.771243	0.772923	0.282063	-0.27723	-0.67476
aPF14_0370_2	PF14_0370	0.771243	0.772923	0.282063	-0.27723	-0.67476
aPF14_0370_1	PF14_0370	0.771243	0.772923	0.282063	-0.27723	-0.67476
aPF14_0370_0	PF14_0370	0.771243	0.772923	0.282063	-0.27723	-0.67476
aPF14_0371_1	PF14_0371	0.486847	-0.27922	-0.06104	0.196377	0.058057
aPF14_0371_0	PF14_0371	0.486847	-0.27922	-0.06104	0.196377	0.058057
aPF14_0372_1	PF14_0372	0.151667	0.076687	-0.33887	-0.32279	0.137647
aPF14_0372_0	PF14_0372	0.151667	0.076687	-0.33887	-0.32279	0.137647
intPF14_0373_2	PF14_0373	-0.59487	-0.95161	-0.98027	0.122607	1.544947
aPF14_0373_0	PF14_0373	-0.59487	-0.95161	-0.98027	0.122607	1.544947
intPF14_0374_2	PF14_0374	1.392387	-0.42145	-2.01934	-1.65282	0.477787
aPF14_0374_1	PF14_0374	1.392387	-0.42145	-2.01934	-1.65282	0.477787
aPF14_0374_0	PF14_0374	1.392387	-0.42145	-2.01934	-1.65282	0.477787
aPF14_0375_0	PF14_0375	-0.75257	-1.25814	-0.56371	0.54971	1.27014
intPF14_0376_2	PF14_0376	-0.97903	-1.04182	-0.09535	0.825687	1.045157
intPF14_0375_2	PF14_0376	-0.75257	-1.25814	-0.56371	0.54971	1.27014
aPF14_0376_0	PF14_0376	-0.97903	-1.04182	-0.09535	0.825687	1.045157
intPF14_0377_3	PF14_0377	-0.89291	-0.18144	0.01387	0.43013	0.79644
aPF14_0377_0	PF14_0377	-0.89291	-0.18144	0.01387	0.43013	0.79644
intPF14_0378_2	PF14_0378	-0.5551	0.018497	0.709507	0.844827	0.163837
aPF14_0378_0	PF14_0378	-0.5551	0.018497	0.709507	0.844827	0.163837
intPF14_0379_2	PF14_0379	-0.67806	-1.03988	-0.95468	0.226535	1.622815
aPF14_0379_3	PF14_0379	-0.67806	-1.03988	-0.95468	0.226535	1.622815
aPF14_0379_2	PF14_0379	-0.67806	-1.03988	-0.95468	0.226535	1.622815

aPF14_0379_1	PF14_0379	-0.67806	-1.03988	-0.95468	0.226535	1.622815
aPF14_0379_0	PF14_0379	-0.67806	-1.03988	-0.95468	0.226535	1.622815
intPF14_0380_2	PF14_0380	-0.93785	-1.43876	-1.06622	0.35672	1.84326
aPF14_0380_1	PF14_0380	-0.93785	-1.43876	-1.06622	0.35672	1.84326
aPF14_0380_0	PF14_0380	-0.93785	-1.43876	-1.06622	0.35672	1.84326
aPF14_0381_0	PF14_0381	-0.79237	0.178847	0.829307	0.667027	-0.13651
intPF14_0382_2	PF14_0382	-0.38334	0.019037	0.213437	0.207787	0.060857
intPF14_0381_2	PF14_0382	-0.79237	0.178847	0.829307	0.667027	-0.13651
aPF14_0381_1	PF14_0382	-0.79237	0.178847	0.829307	0.667027	-0.13651
intPF14_0383_2	PF14_0383	-0.18893	-0.62933	-1.75507	-0.77159	1.703667
aPF14_0383_1	PF14_0383	-0.18893	-0.62933	-1.75507	-0.77159	1.703667
aPF14_0383_0	PF14_0383	-0.18893	-0.62933	-1.75507	-0.77159	1.703667
intPF14_0385_2	PF14_0384	-0.14689	0.145477	0.345547	0.203287	-0.18264
intPF14_0384_1	PF14_0384	-0.44673	1.12816	1.16893	0.13257	-1.04316
aPF14_0384_1	PF14_0384	-0.44673	1.12816	1.16893	0.13257	-1.04316
aPF14_0384_0	PF14_0384	-0.44673	1.12816	1.16893	0.13257	-1.04316
aPF14_0385_1	PF14_0385	-0.14689	0.145477	0.345547	0.203287	-0.18264
aPF14_0385_0	PF14_0385	-0.14689	0.145477	0.345547	0.203287	-0.18264
intPF14_0386_2	PF14_0386	-0.77127	0.31387	0.40326	0.22774	0.06863
aPF14_0386_1	PF14_0386	-0.77127	0.31387	0.40326	0.22774	0.06863
aPF14_0386_0	PF14_0386	-0.77127	0.31387	0.40326	0.22774	0.06863
intPF14_0387_2	PF14_0387	-1.47505	-0.90825	0.98825	1.39925	0.17325
aPF14_0387_1	PF14_0387	-1.47505	-0.90825	0.98825	1.39925	0.17325
aPF14_0387_0	PF14_0387	-1.47505	-0.90825	0.98825	1.39925	0.17325
intPF14_0388_3	PF14_0388	0.143267	-0.23531	-1.11188	-0.94078	0.422647
aPF14_0388_0	PF14_0388	0.143267	-0.23531	-1.11188	-0.94078	0.422647
intPF14_0389_2	PF14_0389	1.410977	1.252117	0.733267	-0.44593	-1.64878
aPF14_0389_1	PF14_0389	1.410977	1.252117	0.733267	-0.44593	-1.64878
aPF14_0389_0	PF14_0389	1.410977	1.252117	0.733267	-0.44593	-1.64878
intPF14_0390_2	PF14_0390	0.339857	0.099697	-0.04439	-0.20327	-0.27036
aPF14_0390_0	PF14_0390	0.339857	0.099697	-0.04439	-0.20327	-0.27036
intPF14_0391_2	PF14_0391	0.059457	0.538737	0.821027	0.364307	-0.6194
aPF14_0391_0	PF14_0391	0.059457	0.538737	0.821027	0.364307	-0.6194
intPF14_0392_2	PF14_0392	0.023887	-0.20477	-1.63017	-0.97213	1.385967
aPF14_0392_3	PF14_0392	0.023887	-0.20477	-1.63017	-0.97213	1.385967
aPF14_0392_2	PF14_0392	0.023887	-0.20477	-1.63017	-0.97213	1.385967
aPF14_0392_1	PF14_0392	0.023887	-0.20477	-1.63017	-0.97213	1.385967
aPF14_0392_0	PF14_0392	0.023887	-0.20477	-1.63017	-0.97213	1.385967
intPF14_0393_2	PF14_0393	-0.42943	-0.17435	0.576967	0.684367	-0.00431
aPF14_0393_1	PF14_0393	-0.42943	-0.17435	0.576967	0.684367	-0.00431
aPF14_0393_0	PF14_0393	-0.42943	-0.17435	0.576967	0.684367	-0.00431
intPF14_0394_2	PF14_0394	-0.25798	-0.20314	-0.09173	0.227393	0.459803
aPF14_0394_1	PF14_0394	-0.25798	-0.20314	-0.09173	0.227393	0.459803
aPF14_0394_0	PF14_0394	-0.25798	-0.20314	-0.09173	0.227393	0.459803
intPF14_0395_2	PF14_0395	-0.73952	-0.26737	0.190747	0.259087	0.107207
aPF14_0395_1	PF14_0395	-0.73952	-0.26737	0.190747	0.259087	0.107207
aPF14_0395_0	PF14_0395	-0.73952	-0.26737	0.190747	0.259087	0.107207

intPF14_0396_2	PF14_0396	-0.53951	-0.58345	0.222397	0.592437	0.271787
aPF14_0396_1	PF14_0396	-0.53951	-0.58345	0.222397	0.592437	0.271787
aPF14_0396_0	PF14_0396	-0.53951	-0.58345	0.222397	0.592437	0.271787
intPF14_0398_2	PF14_0397	0.754348	0.423428	0.251068	-0.0795	-0.54871
intPF14_0397_2	PF14_0397	-0.06162	-0.21993	-0.06215	0.187813	0.269593
aPF14_0397_0	PF14_0397	-0.06162	-0.21993	-0.06215	0.187813	0.269593
aPF14_0398_0	PF14_0398	0.754348	0.423428	0.251068	-0.0795	-0.54871
aPF14_0399_0	PF14_0399	-0.20612	0.365597	1.095807	0.605527	-0.78826
intPF14_0400_2	PF14_0400	-0.38551	-0.30891	0.318317	0.468517	0.041747
aPF14_0400_1	PF14_0400	-0.38551	-0.30891	0.318317	0.468517	0.041747
aPF14_0400_0	PF14_0400	-0.38551	-0.30891	0.318317	0.468517	0.041747
intPF14_0401_2	PF14_0401	0.333907	0.201967	0.474817	0.165017	-0.59963
aPF14_0401_1	PF14_0401	0.333907	0.201967	0.474817	0.165017	-0.59963
aPF14_0401_0	PF14_0401	0.333907	0.201967	0.474817	0.165017	-0.59963
intPF14_0402_2	PF14_0402	-1.18295	-0.4016	0.402773	0.503113	0.142823
aPF14_0402_3	PF14_0402	-1.18295	-0.4016	0.402773	0.503113	0.142823
aPF14_0402_2	PF14_0402	-1.18295	-0.4016	0.402773	0.503113	0.142823
aPF14_0402_1	PF14_0402	-1.18295	-0.4016	0.402773	0.503113	0.142823
aPF14_0402_0	PF14_0402	-1.18295	-0.4016	0.402773	0.503113	0.142823
intPF14_0403_2	PF14_0403	-0.27189	0.371913	0.876923	0.529243	-0.47525
aPF14_0403_1	PF14_0403	-0.27189	0.371913	0.876923	0.529243	-0.47525
aPF14_0403_0	PF14_0403	-0.27189	0.371913	0.876923	0.529243	-0.47525
intPF14_0404_2	PF14_0404	1.600517	1.166327	-0.45078	-1.02999	-0.47452
aPF14_0404_5	PF14_0404	1.600517	1.166327	-0.45078	-1.02999	-0.47452
aPF14_0404_4	PF14_0404	1.600517	1.166327	-0.45078	-1.02999	-0.47452
aPF14_0404_3	PF14_0404	1.600517	1.166327	-0.45078	-1.02999	-0.47452
aPF14_0404_2	PF14_0404	1.600517	1.166327	-0.45078	-1.02999	-0.47452
aPF14_0404_1	PF14_0404	1.600517	1.166327	-0.45078	-1.02999	-0.47452
aPF14_0404_0	PF14_0404	1.600517	1.166327	-0.45078	-1.02999	-0.47452
intPF14_0405_2	PF14_0405	-0.87591	-1.16979	-0.97421	0.29735	1.74418
aPF14_0405_3	PF14_0405	-0.87591	-1.16979	-0.97421	0.29735	1.74418
aPF14_0405_2	PF14_0405	-0.87591	-1.16979	-0.97421	0.29735	1.74418
aPF14_0405_1	PF14_0405	-0.87591	-1.16979	-0.97421	0.29735	1.74418
aPF14_0405_0	PF14_0405	-0.87591	-1.16979	-0.97421	0.29735	1.74418
intPF14_0406_2	PF14_0406	1.391603	1.216503	-0.10801	-0.86833	-0.75734
aPF14_0406_1	PF14_0406	1.391603	1.216503	-0.10801	-0.86833	-0.75734
aPF14_0406_0	PF14_0406	1.391603	1.216503	-0.10801	-0.86833	-0.75734
intPF14_0407_3	PF14_0407	0.577487	0.613087	-0.5575	-0.74649	0.118577
aPF14_0407_5	PF14_0407	0.577487	0.613087	-0.5575	-0.74649	0.118577
aPF14_0407_4	PF14_0407	0.577487	0.613087	-0.5575	-0.74649	0.118577
aPF14_0407_3	PF14_0407	0.577487	0.613087	-0.5575	-0.74649	0.118577
aPF14_0407_2	PF14_0407	0.577487	0.613087	-0.5575	-0.74649	0.118577
aPF14_0407_1	PF14_0407	0.577487	0.613087	-0.5575	-0.74649	0.118577
aPF14_0407_0	PF14_0407	0.577487	0.613087	-0.5575	-0.74649	0.118577
intPF14_0408_2	PF14_0408	-0.35325	-0.02877	-0.70046	-0.40888	0.769433
aPF14_0408_1	PF14_0408	-0.35325	-0.02877	-0.70046	-0.40888	0.769433
aPF14_0408_0	PF14_0408	-0.35325	-0.02877	-0.70046	-0.40888	0.769433

intPF14_0409_2	PF14_0409	-1.00881	-0.28793	-0.37263	0.10763	0.98893
aPF14_0409_1	PF14_0409	-1.00881	-0.28793	-0.37263	0.10763	0.98893
aPF14_0409_0	PF14_0409	-1.00881	-0.28793	-0.37263	0.10763	0.98893
intPF14_0411_2	PF14_0410	0.198343	0.576273	0.779453	0.272213	-0.70261
intPF14_0410_2	PF14_0410	-0.37291	0.18206	0.72187	0.44713	-0.40706
aPF14_0410_1	PF14_0410	-0.37291	0.18206	0.72187	0.44713	-0.40706
aPF14_0410_0	PF14_0410	-0.37291	0.18206	0.72187	0.44713	-0.40706
aPF14_0411_0	PF14_0411	0.198343	0.576273	0.779453	0.272213	-0.70261
intPF14_0412_2	PF14_0412	-0.833	-0.388	0.08025	0.52275	0.656
aPF14_0412_1	PF14_0412	-0.833	-0.388	0.08025	0.52275	0.656
aPF14_0412_0	PF14_0412	-0.833	-0.388	0.08025	0.52275	0.656
intPF14_0413_2	PF14_0413	-1.49209	-0.40405	1.09536	1.09514	-0.13745
aPF14_0413_1	PF14_0413	-1.49209	-0.40405	1.09536	1.09514	-0.13745
aPF14_0413_0	PF14_0413	-1.49209	-0.40405	1.09536	1.09514	-0.13745
intPF14_0414_2	PF14_0414	-1.15439	-1.04808	-0.05135	0.844013	1.046243
aPF14_0414_2	PF14_0414	-1.15439	-1.04808	-0.05135	0.844013	1.046243
aPF14_0414_1	PF14_0414	-1.15439	-1.04808	-0.05135	0.844013	1.046243
aPF14_0414_0	PF14_0414	-1.15439	-1.04808	-0.05135	0.844013	1.046243
intPF14_0415_2	PF14_0415					
aPF14_0415_0	PF14_0415					
intPF14_0417_2	PF14_0416	-1.36877	-0.18867	0.506593	0.758073	0.538833
intPF14_0416_3	PF14_0416	0.200987	0.258577	0.119587	0.034747	-0.06174
aPF14_0416_1	PF14_0416	0.200987	0.258577	0.119587	0.034747	-0.06174
aPF14_0416_0	PF14_0416	0.200987	0.258577	0.119587	0.034747	-0.06174
intPF14_0418_2	PF14_0417	-0.24655	-0.04126	0.03402	0.18398	0.26826
aPF14_0417_1	PF14_0417	-1.36877	-0.18867	0.506593	0.758073	0.538833
aPF14_0417_0	PF14_0417	-1.36877	-0.18867	0.506593	0.758073	0.538833
aPF14_0418_0	PF14_0418	-0.24655	-0.04126	0.03402	0.18398	0.26826
intPF14_0420_2	PF14_0419	-0.11085	-0.54321	-0.64588	-0.14073	0.647835
aPF14_0419_9	PF14_0419	-0.30158	-0.08167	-0.46988	-0.2994	0.458438
aPF14_0419_8	PF14_0419	-0.30158	-0.08167	-0.46988	-0.2994	0.458438
aPF14_0419_7	PF14_0419	-0.30158	-0.08167	-0.46988	-0.2994	0.458438
aPF14_0419_6	PF14_0419	-0.30158	-0.08167	-0.46988	-0.2994	0.458438
aPF14_0419_5	PF14_0419	-0.30158	-0.08167	-0.46988	-0.2994	0.458438
aPF14_0419_4	PF14_0419	-0.30158	-0.08167	-0.46988	-0.2994	0.458438
aPF14_0419_3	PF14_0419	-0.30158	-0.08167	-0.46988	-0.2994	0.458438
aPF14_0419_2	PF14_0419	-0.30158	-0.08167	-0.46988	-0.2994	0.458438
aPF14_0419_10	PF14_0419	-0.30158	-0.08167	-0.46988	-0.2994	0.458438
aPF14_0419_1	PF14_0419	-0.30158	-0.08167	-0.46988	-0.2994	0.458438
aPF14_0419_0	PF14_0419	-0.30158	-0.08167	-0.46988	-0.2994	0.458438
intPF14_0419_2	PF14_0420	-0.30158	-0.08167	-0.46988	-0.2994	0.458438
aPF14_0420_0	PF14_0420	-0.11085	-0.54321	-0.64588	-0.14073	0.647835
intPF14_0421_2	PF14_0421	-0.39311	-0.66446	-0.20757	0.32857	0.56146
aPF14_0421_0	PF14_0421	-0.39311	-0.66446	-0.20757	0.32857	0.56146
intPF14_0422_2	PF14_0422	1.106753	1.180753	0.457003	-0.66534	-1.41759
aPF14_0422_1	PF14_0422	1.106753	1.180753	0.457003	-0.66534	-1.41759
aPF14_0422_0	PF14_0422	1.106753	1.180753	0.457003	-0.66534	-1.41759

intPF14_0423_3	PF14_0423	1.454302	1.015142	-0.63479	-1.33943	-0.65337
aPF14_0423_2	PF14_0423	1.454302	1.015142	-0.63479	-1.33943	-0.65337
aPF14_0423_1	PF14_0423	1.454302	1.015142	-0.63479	-1.33943	-0.65337
aPF14_0423_0	PF14_0423	1.454302	1.015142	-0.63479	-1.33943	-0.65337
aPF14_0424_0	PF14_0424	-0.583	-0.86398	0.135457	0.836877	0.625317
intPF14_0425_2	PF14_0425	-0.70549	-0.66758	0.463163	0.673503	-0.04876
aPF14_0425_1	PF14_0425	-0.70549	-0.66758	0.463163	0.673503	-0.04876
aPF14_0425_0	PF14_0425	-0.70549	-0.66758	0.463163	0.673503	-0.04876
intPF14_0426_2	PF14_0426	-0.4778	-0.57299	1.043987	0.965347	-0.68867
aPF14_0426_0	PF14_0426	-0.4778	-0.57299	1.043987	0.965347	-0.68867
intPF14_0427_2	PF14_0427	-0.17147	-0.79516	-0.55644	0.218103	0.874323
aPF14_0427_1	PF14_0427	-0.17147	-0.79516	-0.55644	0.218103	0.874323
aPF14_0427_0	PF14_0427	-0.17147	-0.79516	-0.55644	0.218103	0.874323
intPF14_0429_2	PF14_0428	0.41997	0.73964	0.77347	-0.06847	-1.16764
intPF14_0428_2	PF14_0428	0.143187	0.850067	0.709867	-0.01353	-0.80273
aPF14_0428_1	PF14_0428	0.143187	0.850067	0.709867	-0.01353	-0.80273
aPF14_0428_0	PF14_0428	0.143187	0.850067	0.709867	-0.01353	-0.80273
aPF14_0429_1	PF14_0429	0.41997	0.73964	0.77347	-0.06847	-1.16764
aPF14_0429_0	PF14_0429	0.41997	0.73964	0.77347	-0.06847	-1.16764
intPF14_0430_2	PF14_0430	-0.52179	-0.43705	0.415107	0.738227	0.228887
aPF14_0430_1	PF14_0430	-0.52179	-0.43705	0.415107	0.738227	0.228887
aPF14_0430_0	PF14_0430	-0.52179	-0.43705	0.415107	0.738227	0.228887
intPF14_0431_2	PF14_0431	0.04653	0.48084	1.16007	0.14293	-1.60734
aPF14_0431_1	PF14_0431	0.04653	0.48084	1.16007	0.14293	-1.60734
aPF14_0431_0	PF14_0431	0.04653	0.48084	1.16007	0.14293	-1.60734
intPF14_0432_2	PF14_0432	0.407587	0.468047	0.864417	-0.10008	-1.55871
aPF14_0432_0	PF14_0432	0.407587	0.468047	0.864417	-0.10008	-1.55871
intPF14_0433_2	PF14_0433	0.56333	-0.01166	-0.00119	-0.28631	-0.62284
aPF14_0433_1	PF14_0433	0.56333	-0.01166	-0.00119	-0.28631	-0.62284
aPF14_0433_0	PF14_0433	0.56333	-0.01166	-0.00119	-0.28631	-0.62284
intPF14_0434_3	PF14_0434	0.226047	0.106747	0.674017	0.388317	-0.65741
aPF14_0434_0	PF14_0434	0.226047	0.106747	0.674017	0.388317	-0.65741
intPF14_0435_2	PF14_0435	0.26234	-0.2252	-0.63511	-0.18139	0.6547
aPF14_0435_1	PF14_0435	0.26234	-0.2252	-0.63511	-0.18139	0.6547
aPF14_0435_0	PF14_0435	0.26234	-0.2252	-0.63511	-0.18139	0.6547
intPF14_0437_2	PF14_0437	0.176167	0.370167	0.716667	0.066667	-1.02583
aPF14_0437_0	PF14_0437	0.176167	0.370167	0.716667	0.066667	-1.02583
aPF14_0436_0	PF14_0437	0.346763	0.386173	0.688163	0.015503	-1.09651
intPF14_0438_2	PF14_0438	0.467577	0.618627	0.031007	-0.40767	-0.41179
aPF14_0438_1	PF14_0438	0.467577	0.618627	0.031007	-0.40767	-0.41179
aPF14_0438_0	PF14_0438	0.467577	0.618627	0.031007	-0.40767	-0.41179
intPF14_0439_2	PF14_0439	-0.62644	0.602217	1.196827	0.696007	-0.53988
aPF14_0439_1	PF14_0439	-0.62644	0.602217	1.196827	0.696007	-0.53988
aPF14_0439_0	PF14_0439	-0.62644	0.602217	1.196827	0.696007	-0.53988
intPF14_0440_2	PF14_0440	0.524587	0.271087	0.127087	-0.60675	-1.13725
aPF14_0440_1	PF14_0440	0.524587	0.271087	0.127087	-0.60675	-1.13725
aPF14_0440_0	PF14_0440	0.524587	0.271087	0.127087	-0.60675	-1.13725

intPF14_0441_2	PF14_0441	0.566713	0.366413	-0.10182	-0.28852	-0.19075
aPF14_0441_1	PF14_0441	0.566713	0.366413	-0.10182	-0.28852	-0.19075
aPF14_0441_0	PF14_0441	0.566713	0.366413	-0.10182	-0.28852	-0.19075
intPF14_0442_1	PF14_0442	-0.05651	0.233587	0.324037	0.204297	-0.11305
aPF14_0442_4	PF14_0442	-0.05651	0.233587	0.324037	0.204297	-0.11305
aPF14_0442_3	PF14_0442	-0.05651	0.233587	0.324037	0.204297	-0.11305
aPF14_0442_2	PF14_0442	-0.05651	0.233587	0.324037	0.204297	-0.11305
aPF14_0442_1	PF14_0442	-0.05651	0.233587	0.324037	0.204297	-0.11305
aPF14_0442_0	PF14_0442	-0.05651	0.233587	0.324037	0.204297	-0.11305
intPF14_0443_2	PF14_0443	-1.06825	-0.75073	-0.25173	0.536887	1.122427
aPF14_0443_0	PF14_0443	-1.06825	-0.75073	-0.25173	0.536887	1.122427
intPF14_0444_2	PF14_0444	-0.3608	-0.193	-0.17813	0.087167	0.448937
aPF14_0445_0	PF14_0444	-0.68932	-0.81938	-0.34471	0.36237	0.85906
aPF14_0444_0	PF14_0444	-0.3608	-0.193	-0.17813	0.087167	0.448937
intPF14_0446_2	PF14_0446	-0.59102	-0.7049	0.315303	0.808363	0.361563
aPF14_0446_0	PF14_0446	-0.59102	-0.7049	0.315303	0.808363	0.361563
intPF14_0447_2	PF14_0447	-0.56685	-0.70373	-0.14653	0.53153	0.78573
aPF14_0447_1	PF14_0447	-0.56685	-0.70373	-0.14653	0.53153	0.78573
aPF14_0447_0	PF14_0447	-0.56685	-0.70373	-0.14653	0.53153	0.78573
aPF14_0448_0	PF14_0448	-0.34943	0.29213	0.73454	0.50406	-0.27933
intPF14_0450_2	PF14_0449	0.333623	0.373383	0.170983	-0.08932	-0.30972
aPF14_0449_0	PF14_0449	0.51865	0.34475	0.34851	0.04149	-0.51975
intPF14_0449_2	PF14_0450	0.51865	0.34475	0.34851	0.04149	-0.51975
aPF14_0450_1	PF14_0450	0.333623	0.373383	0.170983	-0.08932	-0.30972
aPF14_0450_0	PF14_0450	0.333623	0.373383	0.170983	-0.08932	-0.30972
intPF14_0451_2	PF14_0451	-0.48128	-0.04161	0.665223	0.671443	-0.07973
aPF14_0451_0	PF14_0451	-0.48128	-0.04161	0.665223	0.671443	-0.07973
intPF14_0452_2	PF14_0452	0.97771	0.62447	-0.15244	-0.71356	-0.70047
aPF14_0452_0	PF14_0452	0.97771	0.62447	-0.15244	-0.71356	-0.70047
intPF14_0453_2	PF14_0453	-0.15245	0.156257	-0.06475	0.076587	0.388577
aPF14_0453_0	PF14_0453	-0.15245	0.156257	-0.06475	0.076587	0.388577
intPF14_0454_2	PF14_0454	0.18247	0.53833	0.01267	-0.275	-0.13767
aPF14_0454_2	PF14_0454	0.18247	0.53833	0.01267	-0.275	-0.13767
aPF14_0454_1	PF14_0454	0.18247	0.53833	0.01267	-0.275	-0.13767
aPF14_0454_0	PF14_0454	0.18247	0.53833	0.01267	-0.275	-0.13767
intPF14_0455_2	PF14_0455	-1.54566	-0.88367	1.523357	1.652477	-0.38799
aPF14_0455_1	PF14_0455	-1.54566	-0.88367	1.523357	1.652477	-0.38799
aPF14_0455_0	PF14_0455	-1.54566	-0.88367	1.523357	1.652477	-0.38799
intPF14_0457_2	PF14_0456	0.938307	1.187457	0.687597	-0.28626	-1.21112
intPF14_0456_2	PF14_0456	0.8882	1.26848	0.82104	-0.41654	-1.58048
aPF14_0456_1	PF14_0456	0.8882	1.26848	0.82104	-0.41654	-1.58048
aPF14_0456_0	PF14_0456	0.8882	1.26848	0.82104	-0.41654	-1.58048
aPF14_0457_0	PF14_0457	0.938307	1.187457	0.687597	-0.28626	-1.21112
intPF14_0458_2	PF14_0458	0.347543	0.082303	-0.42161	-0.46573	0.013363
aPF14_0460_0	PF14_0458					
aPF14_0459_0	PF14_0458					
aPF14_0458_1	PF14_0458	0.347543	0.082303	-0.42161	-0.46573	0.013363

aPF14_0458_0	PF14_0458	0.347543	0.082303	-0.42161	-0.46573	0.013363
intPF14_0461_2	PF14_0461	0.248228	-0.21847	-0.5373	-0.13831	0.549198
aPF14_0461_2	PF14_0461	0.248228	-0.21847	-0.5373	-0.13831	0.549198
aPF14_0461_1	PF14_0461	0.248228	-0.21847	-0.5373	-0.13831	0.549198
aPF14_0461_0	PF14_0461	0.248228	-0.21847	-0.5373	-0.13831	0.549198
intPF14_0462_2	PF14_0462	-0.69254	-0.93882	-0.11111	0.68911	0.84232
aPF14_0462_1	PF14_0462	-0.69254	-0.93882	-0.11111	0.68911	0.84232
aPF14_0462_0	PF14_0462	-0.69254	-0.93882	-0.11111	0.68911	0.84232
intPF14_0463_2	PF14_0463	-1.07627	-1.00105	-0.63059	0.39279	1.43665
aPF14_0463_5	PF14_0463	-1.07627	-1.00105	-0.63059	0.39279	1.43665
aPF14_0463_4	PF14_0463	-1.07627	-1.00105	-0.63059	0.39279	1.43665
aPF14_0463_3	PF14_0463	-1.07627	-1.00105	-0.63059	0.39279	1.43665
aPF14_0463_2	PF14_0463	-1.07627	-1.00105	-0.63059	0.39279	1.43665
aPF14_0463_1	PF14_0463	-1.07627	-1.00105	-0.63059	0.39279	1.43665
aPF14_0463_0	PF14_0463	-1.07627	-1.00105	-0.63059	0.39279	1.43665
aPF14_0464_0	PF14_0464	0.622017	0.355917	0.078157	-0.24682	-0.48358
intPF14_0465_2	PF14_0465	0.702827	0.580877	0.392257	-0.29492	-1.00854
intPF14_0464_2	PF14_0465	0.622017	0.355917	0.078157	-0.24682	-0.48358
aPF14_0465_0	PF14_0465	0.702827	0.580877	0.392257	-0.29492	-1.00854
aPF14_0466_0	PF14_0466	0.847967	0.161177	0.230147	0.148187	-0.37484
intPF14_0467_2	PF14_0467	1.22211	0.55146	-1.29893	-1.60607	-0.06746
aPF14_0467_0	PF14_0467	1.22211	0.55146	-1.29893	-1.60607	-0.06746
intPF14_0468_2	PF14_0468	-0.34367	-0.68965	-1.32421	-0.47813	1.365313
aPF14_0468_1	PF14_0468	-0.34367	-0.68965	-1.32421	-0.47813	1.365313
aPF14_0468_0	PF14_0468	-0.34367	-0.68965	-1.32421	-0.47813	1.365313
intPF14_0469_2	PF14_0469-a	0.71633	0.70535	-0.13146	-0.71354	-0.59785
aPF14_0469_1	PF14_0469-a	0.71633	0.70535	-0.13146	-0.71354	-0.59785
aPF14_0469_0	PF14_0469-a	0.71633	0.70535	-0.13146	-0.71354	-0.59785
intPF14_0470_2	PF14_0470	-0.49615	-1.01127	-0.38872	0.362383	0.747433
aPF14_0470_1	PF14_0470	-0.49615	-1.01127	-0.38872	0.362383	0.747433
aPF14_0470_0	PF14_0470	-0.49615	-1.01127	-0.38872	0.362383	0.747433
intPF14_0471_2	PF14_0471	-1.0587	-0.49197	0.080207	0.498627	0.625807
aPF14_0471_1	PF14_0471	-1.0587	-0.49197	0.080207	0.498627	0.625807
aPF14_0471_0	PF14_0471	-1.0587	-0.49197	0.080207	0.498627	0.625807
intPF14_0472_2	PF14_0472	0.170827	-0.18714	-1.1237	-0.78196	0.705477
aPF14_0472_1	PF14_0472	0.170827	-0.18714	-1.1237	-0.78196	0.705477
aPF14_0472_0	PF14_0472	0.170827	-0.18714	-1.1237	-0.78196	0.705477
aPF14_0473_1	PF14_0473	0.463077	0.559637	0.291987	-0.15915	-0.5553
aPF14_0473_0	PF14_0473	0.463077	0.559637	0.291987	-0.15915	-0.5553
intPF14_0474_2	PF14_0474					
intPF14_0473_2	PF14_0474	0.463077	0.559637	0.291987	-0.15915	-0.5553
aPF14_0474_0	PF14_0474					
intPF14_0475_2	PF14_0475	-0.15738	-0.19712	0.175733	0.181933	-0.12822
aPF14_0475_0	PF14_0475	-0.15738	-0.19712	0.175733	0.181933	-0.12822
intPF14_0476_2	PF14_0476	0.497167	0.387187	0.212627	-0.50229	-1.05085
aPF14_0476_1	PF14_0476	0.497167	0.387187	0.212627	-0.50229	-1.05085
aPF14_0476_0	PF14_0476	0.497167	0.387187	0.212627	-0.50229	-1.05085

intPF14_0477_2	PF14_0477	0.615923	0.419393	0.032703	-0.38804	-0.56773
aPF14_0477_1	PF14_0477	0.615923	0.419393	0.032703	-0.38804	-0.56773
aPF14_0477_0	PF14_0477	0.615923	0.419393	0.032703	-0.38804	-0.56773
intPF14_0478_2	PF14_0477a	0.764887	0.665087	0.375577	-0.38274	-1.07425
aPF14_0478_1	PF14_0478	0.764887	0.665087	0.375577	-0.38274	-1.07425
aPF14_0478_0	PF14_0478	0.764887	0.665087	0.375577	-0.38274	-1.07425
intPF14_0479_3	PF14_0479	0.388645	0.617115	0.048765	-0.38366	-0.38301
aPF14_0479_2	PF14_0479	0.388645	0.617115	0.048765	-0.38366	-0.38301
aPF14_0479_1	PF14_0479	0.388645	0.617115	0.048765	-0.38366	-0.38301
aPF14_0479_0	PF14_0479	0.388645	0.617115	0.048765	-0.38366	-0.38301
intPF14_0480_2	PF14_0480	-0.20549	-0.06525	-1.06227	-0.70002	0.922465
aPF14_0480_2	PF14_0480	-0.20549	-0.06525	-1.06227	-0.70002	0.922465
aPF14_0480_1	PF14_0480	-0.20549	-0.06525	-1.06227	-0.70002	0.922465
aPF14_0480_0	PF14_0480	-0.20549	-0.06525	-1.06227	-0.70002	0.922465
intPF14_0482_2	PF14_0481	0.341803	0.255493	0.020763	-0.1221	-0.16483
aPF14_0481_1	PF14_0481	0.58251	0.70544	0.70037	-0.01537	-1.03094
aPF14_0481_0	PF14_0481	0.58251	0.70544	0.70037	-0.01537	-1.03094
intPF14_0481_2	PF14_0482	0.58251	0.70544	0.70037	-0.01537	-1.03094
aPF14_0482_1	PF14_0482	0.341803	0.255493	0.020763	-0.1221	-0.16483
aPF14_0482_0	PF14_0482	0.341803	0.255493	0.020763	-0.1221	-0.16483
aPF14_0483_1	PF14_0483	-0.32276	-0.20922	0.294443	0.518723	0.206383
aPF14_0483_0	PF14_0483	-0.32276	-0.20922	0.294443	0.518723	0.206383
intPF14_0484_2	PF14_0484	0.299947	-0.92524	0.428737	0.902597	-0.14892
intPF14_0483_2	PF14_0484	-0.32276	-0.20922	0.294443	0.518723	0.206383
aPF14_0484_1	PF14_0484	0.299947	-0.92524	0.428737	0.902597	-0.14892
aPF14_0484_0	PF14_0484	0.299947	-0.92524	0.428737	0.902597	-0.14892
intPF14_0485_2	PF14_0485	-0.15109	0.077913	0.347413	0.293253	-0.09475
aPF14_0485_1	PF14_0485	-0.15109	0.077913	0.347413	0.293253	-0.09475
aPF14_0485_0	PF14_0485	-0.15109	0.077913	0.347413	0.293253	-0.09475
intPF14_0486_2	PF14_0486	-0.25585	0.645213	0.921813	0.503353	-0.41955
aPF14_0486_1	PF14_0486	-0.25585	0.645213	0.921813	0.503353	-0.41955
aPF14_0486_0	PF14_0486	-0.25585	0.645213	0.921813	0.503353	-0.41955
intPF14_0487_2	PF14_0487	1.116448	0.066758	-0.46835	-0.72287	-0.55331
aPF14_0487_2	PF14_0487	1.116448	0.066758	-0.46835	-0.72287	-0.55331
aPF14_0487_1	PF14_0487	1.116448	0.066758	-0.46835	-0.72287	-0.55331
aPF14_0487_0	PF14_0487	1.116448	0.066758	-0.46835	-0.72287	-0.55331
intPF14_0488_2	PF14_0488	-0.54115	-1.17927	-0.09397	0.771633	0.723933
aPF14_0488_0	PF14_0488	-0.54115	-1.17927	-0.09397	0.771633	0.723933
intPF14_0489_2	PF14_0489	0.671153	0.670243	0.534503	-0.02284	-0.79108
aPF14_0489_1	PF14_0489	0.671153	0.670243	0.534503	-0.02284	-0.79108
aPF14_0489_0	PF14_0489	0.671153	0.670243	0.534503	-0.02284	-0.79108
aPF14_0490_0	PF14_0490	1.008572	0.448372	0.358882	-0.06805	-0.79729
aPF14_0491_1	PF14_0491	-0.36402	-0.22503	-0.28085	-0.20109	0.184587
aPF14_0491_0	PF14_0491	-0.36402	-0.22503	-0.28085	-0.20109	0.184587
intPF14_0492_2	PF14_0492	-0.78292	-1.06022	-1.07296	0.10202	1.67428
intPF14_0491_2	PF14_0492	-0.36402	-0.22503	-0.28085	-0.20109	0.184587
aPF14_0492_0	PF14_0492	-0.78292	-1.06022	-1.07296	0.10202	1.67428

intPF14_0493_2	PF14_0493	-1.32008	-0.86906	0.172877	0.845457	0.811897
aPF14_0493_1	PF14_0493	-1.32008	-0.86906	0.172877	0.845457	0.811897
aPF14_0493_0	PF14_0493	-1.32008	-0.86906	0.172877	0.845457	0.811897
intPF14_0494_2	PF14_0494	0.43806	0.43218	0.36713	-0.16313	-0.77568
aPF14_0494_1	PF14_0494	0.43806	0.43218	0.36713	-0.16313	-0.77568
aPF14_0494_0	PF14_0494	0.43806	0.43218	0.36713	-0.16313	-0.77568
intPF14_0495_2	PF14_0495	-0.13474	-0.90357	-2.47085	-1.3264	1.98282
aPF14_0495_3	PF14_0495	-0.13474	-0.90357	-2.47085	-1.3264	1.98282
aPF14_0495_2	PF14_0495	-0.13474	-0.90357	-2.47085	-1.3264	1.98282
aPF14_0495_1	PF14_0495	-0.13474	-0.90357	-2.47085	-1.3264	1.98282
aPF14_0495_0	PF14_0495	-0.13474	-0.90357	-2.47085	-1.3264	1.98282
intPF14_0497_2	PF14_0496	-0.83323	-0.35984	0.839677	0.924157	-0.11532
intPF14_0496_2	PF14_0496	0.899727	1.020387	0.414727	-0.51939	-1.15905
aPF14_0496_0	PF14_0496	0.899727	1.020387	0.414727	-0.51939	-1.15905
aPF14_0497_1	PF14_0497	-0.83323	-0.35984	0.839677	0.924157	-0.11532
aPF14_0497_0	PF14_0497	-0.83323	-0.35984	0.839677	0.924157	-0.11532
intPF14_0498_2	PF14_0498	-0.80029	-0.75853	-0.04694	0.67694	0.86253
aPF14_0498_0	PF14_0498	-0.80029	-0.75853	-0.04694	0.67694	0.86253
intPF14_0499_2	PF14_0499	0.072297	0.928537	0.246187	-0.29785	-0.3032
aPF14_0499_1	PF14_0499	0.072297	0.928537	0.246187	-0.29785	-0.3032
aPF14_0499_0	PF14_0499	0.072297	0.928537	0.246187	-0.29785	-0.3032
intPF14_0500_2	PF14_0500	-0.84946	-0.89668	-0.57364	0.285977	1.166017
aPF14_0500_0	PF14_0500	-0.84946	-0.89668	-0.57364	0.285977	1.166017
intPF14_0501_2	PF14_0501	0.364415	0.529515	-0.43132	-0.62954	0.077985
aPF14_0501_4	PF14_0501	0.364415	0.529515	-0.43132	-0.62954	0.077985
aPF14_0501_3	PF14_0501	0.364415	0.529515	-0.43132	-0.62954	0.077985
aPF14_0501_2	PF14_0501	0.364415	0.529515	-0.43132	-0.62954	0.077985
aPF14_0501_1	PF14_0501	0.364415	0.529515	-0.43132	-0.62954	0.077985
aPF14_0501_0	PF14_0501	0.364415	0.529515	-0.43132	-0.62954	0.077985
intPF14_0503_2	PF14_0502	-0.11156	-0.52718	0.03287	0.43713	0.28118
intPF14_0502_2	PF14_0502	-1.15987	-0.84761	0.770727	1.253607	0.288947
aPF14_0502_0	PF14_0502	-1.15987	-0.84761	0.770727	1.253607	0.288947
aPF14_0503_0	PF14_0503	-0.11156	-0.52718	0.03287	0.43713	0.28118
aPF14_0504_1	PF14_0504	0.51698	0.48159	0.01757	-0.21007	-0.19859
aPF14_0504_0	PF14_0504	0.51698	0.48159	0.01757	-0.21007	-0.19859
intPF14_0504_2	PF14_0505	0.51698	0.48159	0.01757	-0.21007	-0.19859
aPF14_0505_1	PF14_0505	-0.31784	-1.01328	-1.10443	-0.04173	1.392617
aPF14_0505_0	PF14_0505	-0.31784	-1.01328	-1.10443	-0.04173	1.392617
intPF14_0506_2	PF14_0506	-0.1537	-0.78348	-1.45527	-0.38239	1.623317
intPF14_0505_2	PF14_0506	-0.31784	-1.01328	-1.10443	-0.04173	1.392617
aPF14_0506_1	PF14_0506	-0.1537	-0.78348	-1.45527	-0.38239	1.623317
aPF14_0506_0	PF14_0506	-0.1537	-0.78348	-1.45527	-0.38239	1.623317
intPF14_0507_2	PF14_0506a	0.750932	0.388992	-0.21332	-0.37013	-0.1551
aPF14_0507_2	PF14_0507	0.750932	0.388992	-0.21332	-0.37013	-0.1551
aPF14_0507_1	PF14_0507	0.750932	0.388992	-0.21332	-0.37013	-0.1551
aPF14_0507_0	PF14_0507	0.750932	0.388992	-0.21332	-0.37013	-0.1551
intPF14_0508_2	PF14_0508	0.042518	0.094008	-0.01483	-0.08905	-0.05789

aPF14_0508_1	PF14_0508	0.042518	0.094008	-0.01483	-0.08905	-0.05789
aPF14_0508_0	PF14_0508	0.042518	0.094008	-0.01483	-0.08905	-0.05789
intPF14_0510_2	PF14_0509	-0.41134	-0.42229	0.80259	0.98141	-0.14371
intPF14_0509_2	PF14_0509	0.109085	0.724575	1.038845	0.282265	-1.03447
aPF14_0509_2	PF14_0509	0.109085	0.724575	1.038845	0.282265	-1.03447
aPF14_0509_1	PF14_0509	0.109085	0.724575	1.038845	0.282265	-1.03447
aPF14_0509_0	PF14_0509	0.109085	0.724575	1.038845	0.282265	-1.03447
aPF14_0510_0	PF14_0510	-0.41134	-0.42229	0.80259	0.98141	-0.14371
intPF14_0511_2	PF14_0511	0.502057	0.267717	-0.01867	-0.25449	-0.33638
aPF14_0511_1	PF14_0511	0.502057	0.267717	-0.01867	-0.25449	-0.33638
aPF14_0511_0	PF14_0511	0.502057	0.267717	-0.01867	-0.25449	-0.33638
intPF14_0512_2	PF14_0512	0.97304	0.99728	0.16869	-0.79569	-1.16228
aPF14_0512_1	PF14_0512	0.97304	0.99728	0.16869	-0.79569	-1.16228
aPF14_0512_0	PF14_0512	0.97304	0.99728	0.16869	-0.79569	-1.16228
intPF14_0514_2	PF14_0513	-0.94062	-0.98393	0.167853	0.991813	0.837593
intPF14_0513_2	PF14_0513	0.772013	0.779423	0.544663	-0.114	-0.90726
aPF14_0513_1	PF14_0513	0.772013	0.779423	0.544663	-0.114	-0.90726
aPF14_0513_0	PF14_0513	0.772013	0.779423	0.544663	-0.114	-0.90726
aPF14_0514_0	PF14_0514	-0.94062	-0.98393	0.167853	0.991813	0.837593
intPF14_0515_2	PF14_0515	0.69982	0.6549	0.72779	-0.19996	-1.4309
aPF14_0515_2	PF14_0515	0.69982	0.6549	0.72779	-0.19996	-1.4309
aPF14_0515_1	PF14_0515	0.69982	0.6549	0.72779	-0.19996	-1.4309
aPF14_0515_0	PF14_0515	0.69982	0.6549	0.72779	-0.19996	-1.4309
intPF14_0516_2	PF14_0516	0.792177	0.676127	-0.36825	-0.64941	-0.11229
aPF14_0516_1	PF14_0516	0.792177	0.676127	-0.36825	-0.64941	-0.11229
aPF14_0516_0	PF14_0516	0.792177	0.676127	-0.36825	-0.64941	-0.11229
intPF14_0517_2	PF14_0517	-0.38606	0.01379	0.91842	0.69108	-0.50929
aPF14_0517_1	PF14_0517	-0.38606	0.01379	0.91842	0.69108	-0.50929
aPF14_0517_0	PF14_0517	-0.38606	0.01379	0.91842	0.69108	-0.50929
intPF14_0518_2	PF14_0518	0.662643	0.480803	0.228393	-0.39473	-0.91114
aPF14_0518_0	PF14_0518	0.662643	0.480803	0.228393	-0.39473	-0.91114
aPF14_0519_0	PF14_0519	0.50675	0.55623	0.29854	-0.12654	-0.53523
intPF14_0520_3	PF14_0520	-0.00653	0.22217	0.16266	0.22484	0.18933
intPF14_0519_2	PF14_0520	0.50675	0.55623	0.29854	-0.12654	-0.53523
aPF14_0520_1	PF14_0520	-0.00653	0.22217	0.16266	0.22484	0.18933
aPF14_0520_0	PF14_0520	-0.00653	0.22217	0.16266	0.22484	0.18933
intPF14_0521_2	PF14_0521					
aPF14_0521_0	PF14_0521					
intPF14_0522_2	PF14_0522	0.243947	0.236257	0.232987	0.079347	-0.23192
aPF14_0522_0	PF14_0522	0.243947	0.236257	0.232987	0.079347	-0.23192
intPF14_0523_2	PF14_0523	0.03571	0.21545	-0.5059	-0.4251	0.42155
aPF14_0523_0	PF14_0523	0.03571	0.21545	-0.5059	-0.4251	0.42155
intPF14_0524_2	PF14_0524	0.192837	0.025237	0.695817	0.070237	-1.21776
aPF14_0525_0	PF14_0524	0.141357	0.518217	0.057067	-0.37773	-0.37288
aPF14_0524_0	PF14_0524	0.192837	0.025237	0.695817	0.070237	-1.21776
aPF14_0526_0	PF14_0526-a	-0.02071	0.420533	-0.20457	-0.32077	0.181133
intPF14_0526_2	PF14_0526-b	-0.02071	0.420533	-0.20457	-0.32077	0.181133

intPF14_0527_2	PF14_0527	0.849787	-0.32093	-1.65164	-1.90502	-0.32673
aPF14_0527_1	PF14_0527	0.849787	-0.32093	-1.65164	-1.90502	-0.32673
aPF14_0527_0	PF14_0527	0.849787	-0.32093	-1.65164	-1.90502	-0.32673
intPF14_0528_2	PF14_0528	1.037947	0.215277	0.064947	-0.22161	-0.65394
aPF14_0528_0	PF14_0528	1.037947	0.215277	0.064947	-0.22161	-0.65394
intPF14_0529_2	PF14_0529	-0.02305	0.040777	-0.0203	0.082637	0.196057
aPF14_0529_1	PF14_0529	-0.02305	0.040777	-0.0203	0.082637	0.196057
aPF14_0529_0	PF14_0529	-0.02305	0.040777	-0.0203	0.082637	0.196057
intPF14_0530_2	PF14_0530	-0.3489	-0.37152	-1.28414	-0.70898	1.159405
aPF14_0530_2	PF14_0530	-0.3489	-0.37152	-1.28414	-0.70898	1.159405
aPF14_0530_1	PF14_0530	-0.3489	-0.37152	-1.28414	-0.70898	1.159405
aPF14_0530_0	PF14_0530	-0.3489	-0.37152	-1.28414	-0.70898	1.159405
intPF14_0531_2	PF14_0531	-0.95079	-0.74255	-0.56489	0.314227	1.328887
aPF14_0531_1	PF14_0531	-0.95079	-0.74255	-0.56489	0.314227	1.328887
aPF14_0531_0	PF14_0531	-0.95079	-0.74255	-0.56489	0.314227	1.328887
aPF14_0532_2	PF14_0532	-0.24917	0.137857	0.130797	0.115537	0.108477
aPF14_0532_1	PF14_0532	-0.24917	0.137857	0.130797	0.115537	0.108477
aPF14_0532_0	PF14_0532	-0.24917	0.137857	0.130797	0.115537	0.108477
intPF14_0533_2	PF14_0533	-0.89567	-1.24931	-0.61354	0.58121	1.46231
aPF14_0533_2	PF14_0533	-0.89567	-1.24931	-0.61354	0.58121	1.46231
aPF14_0533_1	PF14_0533	-0.89567	-1.24931	-0.61354	0.58121	1.46231
aPF14_0533_0	PF14_0533	-0.89567	-1.24931	-0.61354	0.58121	1.46231
intPF14_0535_2	PF14_0534	-0.46946	-0.10769	0.551377	0.452457	-0.24747
aPF14_0534_1	PF14_0534	-1.41676	-0.3317	1.372897	1.279937	-0.34596
aPF14_0534_0	PF14_0534	-1.41676	-0.3317	1.372897	1.279937	-0.34596
intPF14_0534_2	PF14_0535	-1.41676	-0.3317	1.372897	1.279937	-0.34596
aPF14_0535_1	PF14_0535	-0.46946	-0.10769	0.551377	0.452457	-0.24747
aPF14_0535_0	PF14_0535	-0.46946	-0.10769	0.551377	0.452457	-0.24747
intPF14_0536_2	PF14_0536	0.805283	0.811663	0.685613	-0.08435	-1.1108
aPF14_0536_1	PF14_0536	0.805283	0.811663	0.685613	-0.08435	-1.1108
aPF14_0536_0	PF14_0536	0.805283	0.811663	0.685613	-0.08435	-1.1108
intPF14_0537_2	PF14_0537	-0.40781	0.389667	-0.11463	-0.08311	0.481207
aPF14_0537_1	PF14_0537	-0.40781	0.389667	-0.11463	-0.08311	0.481207
aPF14_0537_0	PF14_0537	-0.40781	0.389667	-0.11463	-0.08311	0.481207
intPF14_0538_2	PF14_0538	-0.90463	-1.0751	-0.30379	0.718627	1.219687
aPF14_0538_3	PF14_0538	-0.90463	-1.0751	-0.30379	0.718627	1.219687
aPF14_0538_2	PF14_0538	-0.90463	-1.0751	-0.30379	0.718627	1.219687
aPF14_0538_1	PF14_0538	-0.90463	-1.0751	-0.30379	0.718627	1.219687
aPF14_0538_0	PF14_0538	-0.90463	-1.0751	-0.30379	0.718627	1.219687
intPF14_0539_2	PF14_0539	-0.27102	-0.1727	-0.09201	0.229907	0.488757
aPF14_0539_0	PF14_0539	-0.27102	-0.1727	-0.09201	0.229907	0.488757
intPF14_0540_2	PF14_0540	-0.09064	-0.15076	-0.00146	0.283797	0.358097
aPF14_0540_1	PF14_0540	-0.09064	-0.15076	-0.00146	0.283797	0.358097
aPF14_0540_0	PF14_0540	-0.09064	-0.15076	-0.00146	0.283797	0.358097
intPF14_0541_1	PF14_0541	0.7875	0.635	0.40975	-0.52075	-1.3715
aPF14_0541_1	PF14_0541	0.7875	0.635	0.40975	-0.52075	-1.3715
aPF14_0541_0	PF14_0541	0.7875	0.635	0.40975	-0.52075	-1.3715

intPF14_0542_2	PF14_0542	0.184313	0.565413	0.296173	-0.13351	-0.42775
aPF14_0542_0	PF14_0542	0.184313	0.565413	0.296173	-0.13351	-0.42775
intPF14_0543_2	PF14_0543	-0.94893	0.03963	0.31623	0.44227	0.42637
aPF14_0543_1	PF14_0543	-0.94893	0.03963	0.31623	0.44227	0.42637
aPF14_0543_0	PF14_0543	-0.94893	0.03963	0.31623	0.44227	0.42637
intPF14_0544_2	PF14_0544	-0.37791	0.496533	0.468183	0.015983	-0.37737
aPF14_0544_1	PF14_0544	-0.37791	0.496533	0.468183	0.015983	-0.37737
aPF14_0544_0	PF14_0544	-0.37791	0.496533	0.468183	0.015983	-0.37737
intPF14_0545_2	PF14_0545	-0.8516	-0.112	0.02901	0.50399	0.912
aPF14_0545_0	PF14_0545	-0.8516	-0.112	0.02901	0.50399	0.912
intPF14_0546_2	PF14_0546	0.150173	0.231133	0.573973	0.276193	-0.53397
aPF14_0546_1	PF14_0546	0.150173	0.231133	0.573973	0.276193	-0.53397
aPF14_0546_0	PF14_0546	0.150173	0.231133	0.573973	0.276193	-0.53397
intPF14_0547_2	PF14_0547	0.703788	0.239228	-0.75899	-0.61389	0.395538
aPF14_0547_4	PF14_0547	0.703788	0.239228	-0.75899	-0.61389	0.395538
aPF14_0547_3	PF14_0547	0.703788	0.239228	-0.75899	-0.61389	0.395538
aPF14_0547_2	PF14_0547	0.703788	0.239228	-0.75899	-0.61389	0.395538
aPF14_0547_1	PF14_0547	0.703788	0.239228	-0.75899	-0.61389	0.395538
aPF14_0547_0	PF14_0547	0.703788	0.239228	-0.75899	-0.61389	0.395538
intPF14_0549_2	PF14_0548	0.756557	0.199737	0.220537	-0.1147	-0.6954
intPF14_0548_2	PF14_0548	-0.50541	-0.69895	-0.15761	0.212773	0.310613
aPF14_0548_1	PF14_0548	-0.50541	-0.69895	-0.15761	0.212773	0.310613
aPF14_0548_0	PF14_0548	-0.50541	-0.69895	-0.15761	0.212773	0.310613
aPF14_0549_1	PF14_0549	0.756557	0.199737	0.220537	-0.1147	-0.6954
aPF14_0549_0	PF14_0549	0.756557	0.199737	0.220537	-0.1147	-0.6954
intPF14_0550_2	PF14_0550	0.628267	0.859007	0.887827	-0.26882	-1.66767
aPF14_0550_2	PF14_0550	0.628267	0.859007	0.887827	-0.26882	-1.66767
aPF14_0550_1	PF14_0550	0.628267	0.859007	0.887827	-0.26882	-1.66767
aPF14_0550_0	PF14_0550	0.628267	0.859007	0.887827	-0.26882	-1.66767
intPF14_0552_2	PF14_0551	0.058438	0.250968	-0.80986	-0.53847	0.824868
intPF14_0551_2	PF14_0551	-0.34134	-0.57428	-0.32868	0.22718	0.67428
aPF14_0551_1	PF14_0551	-0.34134	-0.57428	-0.32868	0.22718	0.67428
aPF14_0551_0	PF14_0551	-0.34134	-0.57428	-0.32868	0.22718	0.67428
aPF14_0552_5	PF14_0552	0.058438	0.250968	-0.80986	-0.53847	0.824868
aPF14_0552_4	PF14_0552	0.058438	0.250968	-0.80986	-0.53847	0.824868
aPF14_0552_3	PF14_0552	0.058438	0.250968	-0.80986	-0.53847	0.824868
aPF14_0552_2	PF14_0552	0.058438	0.250968	-0.80986	-0.53847	0.824868
aPF14_0552_1	PF14_0552	0.058438	0.250968	-0.80986	-0.53847	0.824868
aPF14_0552_0	PF14_0552	0.058438	0.250968	-0.80986	-0.53847	0.824868
intPF14_0553_3	PF14_0553	0.689397	0.728017	0.339967	-0.52963	-1.16218
aPF14_0553_1	PF14_0553	0.689397	0.728017	0.339967	-0.52963	-1.16218
aPF14_0553_0	PF14_0553	0.689397	0.728017	0.339967	-0.52963	-1.16218
intPF14_0554_2	PF14_0554	0.50128	-0.16941	-0.76318	-0.28782	0.68741
aPF14_0554_0	PF14_0554	0.50128	-0.16941	-0.76318	-0.28782	0.68741
aPF14_0555_0	PF14_0555	0.894827	0.116877	-0.68874	-0.48392	0.316457
intPF14_0556_2	PF14_0556	-0.64849	-0.99352	-1.32271	-0.17795	1.711857
aPF14_0556_1	PF14_0556	-0.64849	-0.99352	-1.32271	-0.17795	1.711857

aPF14_0556_0	PF14_0556	-0.64849	-0.99352	-1.32271	-0.17795	1.711857
intPF14_0558_2	PF14_0557	-0.65901	-0.63193	-0.78064	0.085477	1.357767
intPF14_0557_2	PF14_0557	0.383897	0.660537	0.840927	0.269407	-0.8252
aPF14_0557_0	PF14_0557	0.383897	0.660537	0.840927	0.269407	-0.8252
aPF14_0558_2	PF14_0558	-0.65901	-0.63193	-0.78064	0.085477	1.357767
aPF14_0558_1	PF14_0558	-0.65901	-0.63193	-0.78064	0.085477	1.357767
aPF14_0558_0	PF14_0558	-0.65901	-0.63193	-0.78064	0.085477	1.357767
intPF14_0559_2	PF14_0559	0.46153	0.59083	0.03334	-0.38584	-0.39983
aPF14_0559_1	PF14_0559	0.46153	0.59083	0.03334	-0.38584	-0.39983
aPF14_0559_0	PF14_0559	0.46153	0.59083	0.03334	-0.38584	-0.39983
intPF14_0560_2	PF14_0560	-0.50568	-0.82792	-0.50975	0.216028	0.875198
aPF14_0560_0	PF14_0560	-0.50568	-0.82792	-0.50975	0.216028	0.875198
intPF14_0562_2	PF14_0561	0.736657	1.104727	-0.11795	-0.71971	-0.37239
intPF14_0561_2	PF14_0561	0.815217	1.053897	0.510957	-0.42712	-1.14656
aPF14_0561_1	PF14_0561	0.815217	1.053897	0.510957	-0.42712	-1.14656
aPF14_0561_0	PF14_0561	0.815217	1.053897	0.510957	-0.42712	-1.14656
aPF14_0562_0	PF14_0562	0.736657	1.104727	-0.11795	-0.71971	-0.37239
intPF14_0564_2	PF14_0563	0.339473	0.155143	0.695473	0.339193	-0.77598
intPF14_0563_2	PF14_0563	0.68418	0.51111	-0.14946	-0.54954	-0.43761
aPF14_0563_1	PF14_0563	0.68418	0.51111	-0.14946	-0.54954	-0.43761
aPF14_0563_0	PF14_0563	0.68418	0.51111	-0.14946	-0.54954	-0.43761
aPF14_0564_1	PF14_0564	0.339473	0.155143	0.695473	0.339193	-0.77598
aPF14_0564_0	PF14_0564	0.339473	0.155143	0.695473	0.339193	-0.77598
intPF14_0565_2	PF14_0565	1.259293	0.583053	-0.28236	-0.90598	-0.87239
aPF14_0565_1	PF14_0565	1.259293	0.583053	-0.28236	-0.90598	-0.87239
aPF14_0565_0	PF14_0565	1.259293	0.583053	-0.28236	-0.90598	-0.87239
intPF14_0566_2	PF14_0566	0.440793	-0.04695	-0.81461	-0.49573	0.571613
aPF14_0566_0	PF14_0566	0.440793	-0.04695	-0.81461	-0.49573	0.571613
intPF14_0567_3	PF14_0567	0.28735	0.83025	-0.67801	-0.75199	0.57275
aPF14_0567_0	PF14_0567	0.28735	0.83025	-0.67801	-0.75199	0.57275
intPF14_0568_2	PF14_0568	-1.25332	-0.17041	1.25108	1.14092	-0.27809
aPF14_0568_1	PF14_0568	-1.25332	-0.17041	1.25108	1.14092	-0.27809
aPF14_0568_0	PF14_0568	-1.25332	-0.17041	1.25108	1.14092	-0.27809
intPF14_0569_3	PF14_0569	-0.16415	0.02323	-0.05147	0.04947	0.23877
aPF14_0569_1	PF14_0569	-0.16415	0.02323	-0.05147	0.04947	0.23877
aPF14_0569_0	PF14_0569	-0.16415	0.02323	-0.05147	0.04947	0.23877
intPF14_0570_2	PF14_0570	-1.23429	-0.95153	0.580567	1.272767	0.621867
aPF14_0570_0	PF14_0570	-1.23429	-0.95153	0.580567	1.272767	0.621867
intPF14_0571_2	PF14_0571	-1.01487	-0.57263	0.20127	0.80073	0.78863
aPF14_0571_0	PF14_0571	-1.01487	-0.57263	0.20127	0.80073	0.78863
aPF14_0572_0	PF14_0572	0.548837	-0.0082	-1.69406	-1.47172	0.709777
intPF14_0573_2	PF14_0573	0.058237	0.044107	-0.08061	-0.24299	-0.20671
intPF14_0572_2	PF14_0573	0.548837	-0.0082	-1.69406	-1.47172	0.709777
aPF14_0573_0	PF14_0573	0.058237	0.044107	-0.08061	-0.24299	-0.20671
intPF14_0574_2	PF14_0574	0.92	0.587	0.41	-0.688	-1.699
aPF14_0574_0	PF14_0574	0.92	0.587	0.41	-0.688	-1.699
intPF14_0575_2	PF14_0575	-0.34398	-0.2891	0.649703	0.687963	-0.23724

aPF14_0575_0	PF14_0575	-0.34398	-0.2891	0.649703	0.687963	-0.23724
intPF14_0576_2	PF14_0575a	0.482203	-0.07004	0.591563	0.485103	-0.5643
aPF14_0576_0	PF14_0576	0.482203	-0.07004	0.591563	0.485103	-0.5643
intPF14_0577_2	PF14_0577	-0.94078	-0.90074	-0.41425	0.462197	1.165857
aPF14_0577_2	PF14_0577	-0.94078	-0.90074	-0.41425	0.462197	1.165857
aPF14_0577_1	PF14_0577	-0.94078	-0.90074	-0.41425	0.462197	1.165857
aPF14_0577_0	PF14_0577	-0.94078	-0.90074	-0.41425	0.462197	1.165857
intPF14_0578_2	PF14_0578	0.297893	-0.66645	-0.96711	-0.73423	0.128113
aPF14_0578_0	PF14_0578	0.297893	-0.66645	-0.96711	-0.73423	0.128113
intPF14_0579_2	PF14_0579	1.129967	0.654177	0.706147	0.108187	-1.07284
aPF14_0579_0	PF14_0579	1.129967	0.654177	0.706147	0.108187	-1.07284
intPF14_0581_2	PF14_0580	-0.7579	-1.18548	-0.72605	0.42805	1.43548
aPF14_0580_1	PF14_0580	-0.5828	-0.80998	-0.14954	0.55904	0.76698
aPF14_0580_0	PF14_0580	-0.5828	-0.80998	-0.14954	0.55904	0.76698
intPF14_0582_2	PF14_0581-a	-0.43992	-0.98314	0.412	1.02048	0.26662
intPF14_0580_2	PF14_0581-a	-0.5828	-0.80998	-0.14954	0.55904	0.76698
aPF14_0581_0	PF14_0581-a	-0.7579	-1.18548	-0.72605	0.42805	1.43548
aPF14_0582_1	PF14_0582	-0.43992	-0.98314	0.412	1.02048	0.26662
aPF14_0582_0	PF14_0582	-0.43992	-0.98314	0.412	1.02048	0.26662
intPF14_0583_2	PF14_0583	-0.57353	-0.65385	-0.5283	0.007463	0.735013
aPF14_0583_1	PF14_0583	-0.57353	-0.65385	-0.5283	0.007463	0.735013
aPF14_0583_0	PF14_0583	-0.57353	-0.65385	-0.5283	0.007463	0.735013
intPF14_0584_3	PF14_0584	0.88048	1.1216	0.7318	-0.4048	-1.4946
aPF14_0584_0	PF14_0584	0.88048	1.1216	0.7318	-0.4048	-1.4946
intPF14_0585_2	PF14_0585	-0.18565	0.09525	0.52699	0.39701	-0.24525
aPF14_0585_0	PF14_0585	-0.18565	0.09525	0.52699	0.39701	-0.24525
intPF14_0586_2	PF14_0586	0.94818	-0.5638	-1.75145	-1.41327	0.39916
aPF14_0586_0	PF14_0586	0.94818	-0.5638	-1.75145	-1.41327	0.39916
intPF14_0587_2	PF14_0587	0.444063	0.512703	0.288843	-0.13518	-0.53854
aPF14_0587_1	PF14_0587	0.444063	0.512703	0.288843	-0.13518	-0.53854
aPF14_0587_0	PF14_0587	0.444063	0.512703	0.288843	-0.13518	-0.53854
intPF14_0792_2	PF14_0588					
intPF14_0588_2	PF14_0588	0.201988	0.521178	-0.40628	-0.71474	-0.04713
aPF14_0588_4	PF14_0588	0.201988	0.521178	-0.40628	-0.71474	-0.04713
aPF14_0588_3	PF14_0588	0.201988	0.521178	-0.40628	-0.71474	-0.04713
aPF14_0588_2	PF14_0588	0.201988	0.521178	-0.40628	-0.71474	-0.04713
aPF14_0588_1	PF14_0588	0.201988	0.521178	-0.40628	-0.71474	-0.04713
aPF14_0588_0	PF14_0588	0.201988	0.521178	-0.40628	-0.71474	-0.04713
intPF14_0589_2	PF14_0589	0.357807	0.655447	0.892367	0.215467	-0.99511
aPF14_0589_1	PF14_0589	0.357807	0.655447	0.892367	0.215467	-0.99511
aPF14_0589_0	PF14_0589	0.357807	0.655447	0.892367	0.215467	-0.99511
aPF14_0590_0	PF14_0590	0.01994	0.04928	-0.05506	0.01706	0.15272
intPF14_0592_2	PF14_0591	-0.91711	-0.94491	0.090033	0.821593	0.743093
intPF14_0591_2	PF14_0591	-0.65179	-1.08353	-0.28144	0.67744	1.02703
intPF14_0590_2	PF14_0591	0.01994	0.04928	-0.05506	0.01706	0.15272
aPF14_0591_1	PF14_0591	-0.65179	-1.08353	-0.28144	0.67744	1.02703
aPF14_0591_0	PF14_0591	-0.65179	-1.08353	-0.28144	0.67744	1.02703

aPF14_0592_0	PF14_0592	-0.91711	-0.94491	0.090033	0.821593	0.743093
intPF14_0593_3	PF14_0593	0.165372	0.240822	0.001022	-0.00669	0.093512
aPF14_0593_2	PF14_0593	0.165372	0.240822	0.001022	-0.00669	0.093512
aPF14_0593_1	PF14_0593	0.165372	0.240822	0.001022	-0.00669	0.093512
aPF14_0593_0	PF14_0593	0.165372	0.240822	0.001022	-0.00669	0.093512
intPF14_0594_2	PF14_0594	1.400243	1.163773	0.093243	-1.26618	-1.76111
aPF14_0594_4	PF14_0594	1.400243	1.163773	0.093243	-1.26618	-1.76111
aPF14_0594_3	PF14_0594	1.400243	1.163773	0.093243	-1.26618	-1.76111
aPF14_0594_2	PF14_0594	1.400243	1.163773	0.093243	-1.26618	-1.76111
aPF14_0594_1	PF14_0594	1.400243	1.163773	0.093243	-1.26618	-1.76111
aPF14_0594_0	PF14_0594	1.400243	1.163773	0.093243	-1.26618	-1.76111
aPF14_0595_0	PF14_0595	0.919005	-0.23156	0.303345	0.180355	-0.7463
intPF14_0596_2	PF14_0596					
intPF14_0595_2	PF14_0596	0.919005	-0.23156	0.303345	0.180355	-0.7463
aPF14_0596_1	PF14_0596					
aPF14_0596_0	PF14_0596					
intPF14_0597_2	PF14_0597	-0.73182	-1.42741	-0.4182	0.851363	1.336073
aPF14_0597_1	PF14_0597	-0.73182	-1.42741	-0.4182	0.851363	1.336073
aPF14_0597_0	PF14_0597	-0.73182	-1.42741	-0.4182	0.851363	1.336073
intPF14_0598_2	PF14_0598	-0.55577	0.20483	0.77734	0.80766	0.10817
aPF14_0598_0	PF14_0598	-0.55577	0.20483	0.77734	0.80766	0.10817
intPF14_0599_2	PF14_0599	0.757137	0.999807	0.539887	-0.21055	-0.89147
aPF14_0599_1	PF14_0599	0.757137	0.999807	0.539887	-0.21055	-0.89147
aPF14_0599_0	PF14_0599	0.757137	0.999807	0.539887	-0.21055	-0.89147
intPF14_0600_2	PF14_0600	0.11908	-0.19042	0.15133	0.25567	-0.06058
aPF14_0600_0	PF14_0600	0.11908	-0.19042	0.15133	0.25567	-0.06058
intPF14_0601_3	PF14_0601	-0.65571	-0.80943	0.148857	0.850477	0.681767
aPF14_0601_0	PF14_0601	-0.65571	-0.80943	0.148857	0.850477	0.681767
intPF14_0603_2	PF14_0602	0.60193	2.17536	0.80579	-0.92579	-1.61636
intPF14_0602_2	PF14_0602	-1.63676	-1.07424	0.321983	1.167183	0.989903
aPF14_0602_1	PF14_0602	-1.63676	-1.07424	0.321983	1.167183	0.989903
aPF14_0602_0	PF14_0602	-1.63676	-1.07424	0.321983	1.167183	0.989903
aPF14_0603_0	PF14_0603	0.60193	2.17536	0.80579	-0.92579	-1.61636
intPF14_0604_2	PF14_0604	1.756675	0.768175	-0.4806	-1.2379	-1.0492
aPF14_0604_0	PF14_0604	1.756675	0.768175	-0.4806	-1.2379	-1.0492
intPF14_0605_2	PF14_0604a	0.465763	0.206183	0.471723	0.201143	-0.58092
aPF14_0605_0	PF14_0605	0.465763	0.206183	0.471723	0.201143	-0.58092
intPF14_0606_2	PF14_0606	-0.34946	-0.56066	0.198817	0.444517	0.044997
aPF14_0606_0	PF14_0606	-0.34946	-0.56066	0.198817	0.444517	0.044997
intPF14_0607_2	PF14_0607	-0.35275	-0.61326	-2.24773	-1.34711	1.808923
aPF14_0607_1	PF14_0607	-0.35275	-0.61326	-2.24773	-1.34711	1.808923
aPF14_0607_0	PF14_0607	-0.35275	-0.61326	-2.24773	-1.34711	1.808923
aPF14_0608_1	PF14_0608	-0.14954	0.79418	0.13064	-0.44964	-0.33468
aPF14_0608_0	PF14_0608	-0.14954	0.79418	0.13064	-0.44964	-0.33468
intPF14_0609_2	PF14_0609	0.040267	0.291657	0.531177	0.190657	-0.50682
intPF14_0608_2	PF14_0609	-0.14954	0.79418	0.13064	-0.44964	-0.33468
aPF14_0609_1	PF14_0609	0.040267	0.291657	0.531177	0.190657	-0.50682

aPF14_0609_0	PF14_0609	0.040267	0.291657	0.531177	0.190657	-0.50682
intPF14_0611_2	PF14_0610	0.647953	0.306213	0.056313	-0.13865	-0.32305
intPF14_0610_2	PF14_0610	1.974017	1.086897	0.291197	-0.81486	-1.68956
aPF14_0610_0	PF14_0610	1.974017	1.086897	0.291197	-0.81486	-1.68956
aPF14_0611_1	PF14_0611	0.647953	0.306213	0.056313	-0.13865	-0.32305
aPF14_0611_0	PF14_0611	0.647953	0.306213	0.056313	-0.13865	-0.32305
intPF14_0612_2	PF14_0612	-0.24239	0.28394	0.62161	0.26639	-0.46994
intPF14_0613_2	PF14_0613	-1.08254	-0.6506	-0.12654	0.491835	0.896845
aPF14_0613_4	PF14_0613	-1.08254	-0.6506	-0.12654	0.491835	0.896845
aPF14_0613_3	PF14_0613	-1.08254	-0.6506	-0.12654	0.491835	0.896845
aPF14_0613_2	PF14_0613	-1.08254	-0.6506	-0.12654	0.491835	0.896845
aPF14_0613_1	PF14_0613	-1.08254	-0.6506	-0.12654	0.491835	0.896845
aPF14_0613_0	PF14_0613	-1.08254	-0.6506	-0.12654	0.491835	0.896845
intPF14_0614_2	PF14_0614	0.106947	0.208937	0.454457	-0.27923	-1.14838
aPF14_0614_2	PF14_0614	0.106947	0.208937	0.454457	-0.27923	-1.14838
aPF14_0614_1	PF14_0614	0.106947	0.208937	0.454457	-0.27923	-1.14838
aPF14_0614_0	PF14_0614	0.106947	0.208937	0.454457	-0.27923	-1.14838
intPF14_0615_2	PF14_0615	0.145327	-0.06611	0.149487	-0.05965	-0.45605
aPF14_0615_1	PF14_0615	0.145327	-0.06611	0.149487	-0.05965	-0.45605
aPF14_0615_0	PF14_0615	0.145327	-0.06611	0.149487	-0.05965	-0.45605
intPF14_0616_2	PF14_0616	0.232173	0.420113	0.462263	-0.1101	-0.80995
aPF14_0616_1	PF14_0616	0.232173	0.420113	0.462263	-0.1101	-0.80995
aPF14_0616_0	PF14_0616	0.232173	0.420113	0.462263	-0.1101	-0.80995
intPF14_0617_2	PF14_0617	-0.76344	-1.0748	-0.57541	0.381073	1.164463
aPF14_0617_0	PF14_0617	-0.76344	-1.0748	-0.57541	0.381073	1.164463
intPF14_0618_2	PF14_0618	-0.88497	-0.98615	-0.55595	0.477787	1.373487
aPF14_0618_1	PF14_0618	-0.88497	-0.98615	-0.55595	0.477787	1.373487
aPF14_0618_0	PF14_0618	-0.88497	-0.98615	-0.55595	0.477787	1.373487
intPF14_0620_2	PF14_0619	1.291697	1.127507	0.477987	-0.50265	-1.30917
aPF14_0619_1	PF14_0619	0.779287	0.430047	0.623147	0.105187	-0.95771
aPF14_0619_0	PF14_0619	0.779287	0.430047	0.623147	0.105187	-0.95771
intPF14_0619_2	PF14_0620	0.779287	0.430047	0.623147	0.105187	-0.95771
aPF14_0620_1	PF14_0620	1.291697	1.127507	0.477987	-0.50265	-1.30917
aPF14_0620_0	PF14_0620	1.291697	1.127507	0.477987	-0.50265	-1.30917
intPF14_0621_2	PF14_0621	1.355693	1.025523	0.347443	-1.01778	-1.93186
aPF14_0621_0	PF14_0621	1.355693	1.025523	0.347443	-1.01778	-1.93186
intPF14_0622_2	PF14_0622	1.644302	0.390352	-0.50896	-1.02986	-0.89956
aPF14_0622_2	PF14_0622	1.644302	0.390352	-0.50896	-1.02986	-0.89956
aPF14_0622_1	PF14_0622	1.644302	0.390352	-0.50896	-1.02986	-0.89956
aPF14_0622_0	PF14_0622	1.644302	0.390352	-0.50896	-1.02986	-0.89956
aPF14_0624_0	PF14_0624	2.01698	0.99912	-0.00574	-0.43426	-0.64712
aPF14_0623_0	PF14_0624	1.317007	0.919977	-0.06196	-0.6737	-0.72264
intPF14_0626_2	PF14_0625	0.022498	-0.55727	-0.10556	0.384138	0.390718
intPF14_0624_2	PF14_0625	2.01698	0.99912	-0.00574	-0.43426	-0.64712
aPF14_0625_0	PF14_0625					
intPF14_0625_2	PF14_0626					
aPF14_0626_9	PF14_0626	0.022498	-0.55727	-0.10556	0.384138	0.390718

aPF14_0626_8	PF14_0626	0.022498	-0.55727	-0.10556	0.384138	0.390718
aPF14_0626_7	PF14_0626	0.022498	-0.55727	-0.10556	0.384138	0.390718
aPF14_0626_6	PF14_0626	0.022498	-0.55727	-0.10556	0.384138	0.390718
aPF14_0626_5	PF14_0626	0.022498	-0.55727	-0.10556	0.384138	0.390718
aPF14_0626_4	PF14_0626	0.022498	-0.55727	-0.10556	0.384138	0.390718
aPF14_0626_3	PF14_0626	0.022498	-0.55727	-0.10556	0.384138	0.390718
aPF14_0626_2	PF14_0626	0.022498	-0.55727	-0.10556	0.384138	0.390718
aPF14_0626_1	PF14_0626	0.022498	-0.55727	-0.10556	0.384138	0.390718
aPF14_0626_0	PF14_0626	0.022498	-0.55727	-0.10556	0.384138	0.390718
intPF14_0627_1	PF14_0627	0.103697	0.303507	0.718487	0.444847	-0.48217
aPF14_0627_0	PF14_0627	0.103697	0.303507	0.718487	0.444847	-0.48217
aPF14_0629_0	PF14_0629	0.149183	0.424063	0.059363	-0.2077	-0.1874
aPF14_0628_0	PF14_0629	-0.49055	-0.47524	-0.19202	0.17502	0.46124
intPF14_0630_2	PF14_0630	-0.81357	-1.24166	-0.33592	0.70892	1.12266
intPF14_0629_2	PF14_0630	0.149183	0.424063	0.059363	-0.2077	-0.1874
aPF14_0630_1	PF14_0630	-0.81357	-1.24166	-0.33592	0.70892	1.12266
aPF14_0630_0	PF14_0630	-0.81357	-1.24166	-0.33592	0.70892	1.12266
intPF14_0631_2	PF14_0631	0.426838	0.508078	-0.35416	-0.60178	-0.05694
aPF14_0631_4	PF14_0631	0.426838	0.508078	-0.35416	-0.60178	-0.05694
aPF14_0631_3	PF14_0631	0.426838	0.508078	-0.35416	-0.60178	-0.05694
aPF14_0631_2	PF14_0631	0.426838	0.508078	-0.35416	-0.60178	-0.05694
aPF14_0631_1	PF14_0631	0.426838	0.508078	-0.35416	-0.60178	-0.05694
aPF14_0631_0	PF14_0631	0.426838	0.508078	-0.35416	-0.60178	-0.05694
intPF14_0632_1	PF14_0632	-0.6568	0.049993	-0.056	0.112163	0.523173
aPF14_0632_1	PF14_0632	-0.6568	0.049993	-0.056	0.112163	0.523173
aPF14_0632_0	PF14_0632	-0.6568	0.049993	-0.056	0.112163	0.523173
intPF14_0633_2	PF14_0633	1.603547	0.473767	-0.27202	-0.81764	-0.94643
aPF14_0633_1	PF14_0633	1.603547	0.473767	-0.27202	-0.81764	-0.94643
aPF14_0633_0	PF14_0633	1.603547	0.473767	-0.27202	-0.81764	-0.94643
intPF14_0635_2	PF14_0634	1.31762	1.10492	0.81865	-0.37965	-1.77292
intPF14_0634_2	PF14_0634	1.474763	0.677193	0.166623	-0.51796	-1.12253
aPF14_0634_0	PF14_0634	1.474763	0.677193	0.166623	-0.51796	-1.12253
aPF14_0635_0	PF14_0635	1.31762	1.10492	0.81865	-0.37965	-1.77292
intPF14_0636_2	PF14_0636	0.972377	0.736617	0.646267	-0.21893	-1.34628
aPF14_0636_1	PF14_0636	0.972377	0.736617	0.646267	-0.21893	-1.34628
aPF14_0636_0	PF14_0636	0.972377	0.736617	0.646267	-0.21893	-1.34628
intPF14_0637_2	PF14_0637	0.142045	0.285425	0.299645	-0.01009	-0.42154
aPF14_0637_2	PF14_0637	0.142045	0.285425	0.299645	-0.01009	-0.42154
aPF14_0637_1	PF14_0637	0.142045	0.285425	0.299645	-0.01009	-0.42154
aPF14_0637_0	PF14_0637	0.142045	0.285425	0.299645	-0.01009	-0.42154
intPF14_0638_2	PF14_0638					
aPF14_0638_1	PF14_0638					
aPF14_0638_0	PF14_0638					
intPF14_0640_2	PF14_0639	1.50952	1.41441	0.52768	-0.69718	-1.57341
aPF14_0639_1	PF14_0639	0.954527	0.988367	0.370507	-0.29667	-0.78353
aPF14_0639_0	PF14_0639	0.954527	0.988367	0.370507	-0.29667	-0.78353
intPF14_0639_2	PF14_0640	0.954527	0.988367	0.370507	-0.29667	-0.78353

aPF14_0640_1	PF14_0640	1.50952	1.41441	0.52768	-0.69718	-1.57341
aPF14_0640_0	PF14_0640	1.50952	1.41441	0.52768	-0.69718	-1.57341
intPF14_0641_2	PF14_0641	-0.58153	-0.69836	-0.09278	0.639443	0.857523
aPF14_0641_1	PF14_0641	-0.58153	-0.69836	-0.09278	0.639443	0.857523
aPF14_0641_0	PF14_0641	-0.58153	-0.69836	-0.09278	0.639443	0.857523
intPF14_0643_2	PF14_0642	0.511573	0.391133	0.116233	-0.11107	-0.28947
aPF14_0642_0	PF14_0642	-0.26839	-0.64901	-0.15346	0.544797	0.755347
intPF14_0642_2	PF14_0643	-0.26839	-0.64901	-0.15346	0.544797	0.755347
aPF14_0643_1	PF14_0643	0.511573	0.391133	0.116233	-0.11107	-0.28947
aPF14_0643_0	PF14_0643	0.511573	0.391133	0.116233	-0.11107	-0.28947
intPF14_0644_2	PF14_0644	0.772648	1.913998	-0.04686	-1.18161	-0.66805
aPF14_0644_3	PF14_0644	0.772648	1.913998	-0.04686	-1.18161	-0.66805
aPF14_0644_2	PF14_0644	0.772648	1.913998	-0.04686	-1.18161	-0.66805
aPF14_0644_1	PF14_0644	0.772648	1.913998	-0.04686	-1.18161	-0.66805
aPF14_0644_0	PF14_0644	0.772648	1.913998	-0.04686	-1.18161	-0.66805
intPF14_0645_2	PF14_0645					
aPF14_0645_0	PF14_0645					
intPF14_0646_2	PF14_0646	-0.26356	-0.9362	-0.1251	0.7061	0.7522
aPF14_0646_0	PF14_0646	-0.26356	-0.9362	-0.1251	0.7061	0.7522
intPF14_0647_2	PF14_0647	-0.3313	-0.97684	-0.8999	-0.23315	0.759288
aPF14_0647_2	PF14_0647	-0.3313	-0.97684	-0.8999	-0.23315	0.759288
aPF14_0647_1	PF14_0647	-0.3313	-0.97684	-0.8999	-0.23315	0.759288
aPF14_0647_0	PF14_0647	-0.3313	-0.97684	-0.8999	-0.23315	0.759288
intPF14_0648_2	PF14_0648	0.07779	-0.07295	-0.0591	-0.1599	-0.20605
aPF14_0648_2	PF14_0648	0.07779	-0.07295	-0.0591	-0.1599	-0.20605
aPF14_0648_1	PF14_0648	0.07779	-0.07295	-0.0591	-0.1599	-0.20605
aPF14_0648_0	PF14_0648	0.07779	-0.07295	-0.0591	-0.1599	-0.20605
intPF14_0649_2	PF14_0649	-0.3287	0.051	-0.16475	-0.05703	0.36006
aPF14_0649_3	PF14_0649	-0.3287	0.051	-0.16475	-0.05703	0.36006
aPF14_0649_2	PF14_0649	-0.3287	0.051	-0.16475	-0.05703	0.36006
aPF14_0649_1	PF14_0649	-0.3287	0.051	-0.16475	-0.05703	0.36006
aPF14_0649_0	PF14_0649	-0.3287	0.051	-0.16475	-0.05703	0.36006
intPF14_0651_2	PF14_0650	0.44715	0.84023	0.19255	-0.42155	-0.57423
intPF14_0650_2	PF14_0650	-0.30606	-0.16466	-0.13567	0.054007	0.321997
aPF14_0650_0	PF14_0650	-0.30606	-0.16466	-0.13567	0.054007	0.321997
aPF14_0651_0	PF14_0651	0.44715	0.84023	0.19255	-0.42155	-0.57423
intPF14_0652_2	PF14_0652	1.264163	-0.10981	-1.6024	-1.80444	-0.26353
aPF14_0652_1	PF14_0652	1.264163	-0.10981	-1.6024	-1.80444	-0.26353
aPF14_0652_0	PF14_0652	1.264163	-0.10981	-1.6024	-1.80444	-0.26353
intPF14_0653_2	PF14_0653-a	-0.69474	-0.14027	-0.52096	-0.0917	0.955607
aPF14_0653_0	PF14_0653-a	-0.69474	-0.14027	-0.52096	-0.0917	0.955607
intPF14_0654_2	PF14_0654	-0.93371	-0.63612	0.026402	0.485212	0.566562
aPF14_0654_2	PF14_0654	-0.93371	-0.63612	0.026402	0.485212	0.566562
aPF14_0654_1	PF14_0654	-0.93371	-0.63612	0.026402	0.485212	0.566562
aPF14_0654_0	PF14_0654	-0.93371	-0.63612	0.026402	0.485212	0.566562
intPF14_0655_2	PF14_0655	-0.07523	0.27515	0.74495	0.42405	-0.52115
aPF14_0655_1	PF14_0655	-0.07523	0.27515	0.74495	0.42405	-0.52115

aPF14_0655_0	PF14_0655	-0.07523	0.27515	0.74495	0.42405	-0.52115
intPF14_0656_2	PF14_0656	0.005727	0.367387	0.311727	0.081607	-0.20605
aPF14_0656_1	PF14_0656	0.005727	0.367387	0.311727	0.081607	-0.20605
aPF14_0656_0	PF14_0656	0.005727	0.367387	0.311727	0.081607	-0.20605
intPF14_0657_2	PF14_0657	0.256933	0.934353	0.579283	0.017383	-0.49869
aPF14_0657_1	PF14_0657	0.256933	0.934353	0.579283	0.017383	-0.49869
aPF14_0657_0	PF14_0657	0.256933	0.934353	0.579283	0.017383	-0.49869
aPF14_0658_0	PF14_0658	-0.9574	-0.48658	0.191372	0.628112	0.586062
intPF14_0659_3	PF14_0659	0.194877	0.067597	0.278807	0.218527	-0.20326
intPF14_0658_2	PF14_0659	-0.9574	-0.48658	0.191372	0.628112	0.586062
aPF14_0659_0	PF14_0659	0.194877	0.067597	0.278807	0.218527	-0.20326
intPF14_0660_2	PF14_0660	0.354513	-0.31005	-1.1764	-1.05244	0.253213
aPF14_0660_1	PF14_0660	0.354513	-0.31005	-1.1764	-1.05244	0.253213
aPF14_0660_0	PF14_0660	0.354513	-0.31005	-1.1764	-1.05244	0.253213
intPF14_0661_2	PF14_0661	0.36117	0.29517	0.95417	0.31183	-1.20517
aPF14_0661_0	PF14_0661	0.36117	0.29517	0.95417	0.31183	-1.20517
intPF14_0662_2	PF14_0662	-0.09943	-0.00884	0.995947	0.609887	-0.88382
aPF14_0662_1	PF14_0662	-0.09943	-0.00884	0.995947	0.609887	-0.88382
aPF14_0662_0	PF14_0662	-0.09943	-0.00884	0.995947	0.609887	-0.88382
intPF14_0663_2	PF14_0663	1.247973	1.416613	0.547533	-0.71337	-1.54545
aPF14_0663_1	PF14_0663	1.247973	1.416613	0.547533	-0.71337	-1.54545
aPF14_0663_0	PF14_0663	1.247973	1.416613	0.547533	-0.71337	-1.54545
intPF14_0664_2	PF14_0664	0.018692	-0.04782	-0.43273	-0.15079	0.529052
aPF14_0664_4	PF14_0664	0.018692	-0.04782	-0.43273	-0.15079	0.529052
aPF14_0664_3	PF14_0664	0.018692	-0.04782	-0.43273	-0.15079	0.529052
aPF14_0664_2	PF14_0664	0.018692	-0.04782	-0.43273	-0.15079	0.529052
aPF14_0664_1	PF14_0664	0.018692	-0.04782	-0.43273	-0.15079	0.529052
aPF14_0664_0	PF14_0664	0.018692	-0.04782	-0.43273	-0.15079	0.529052
intPF14_0665_2	PF14_0665	-0.16835	0.046992	0.183142	0.262942	0.146092
aPF14_0665_0	PF14_0665	-0.16835	0.046992	0.183142	0.262942	0.146092
intPF14_0667_2	PF14_0666	-0.11235	-0.10828	-0.36246	-0.13788	0.422943
aPF14_0666_0	PF14_0666	-0.84474	-0.91929	0.182577	0.849757	0.608627
intPF14_0666_2	PF14_0667	-0.84474	-0.91929	0.182577	0.849757	0.608627
aPF14_0667_1	PF14_0667	-0.11235	-0.10828	-0.36246	-0.13788	0.422943
aPF14_0667_0	PF14_0667	-0.11235	-0.10828	-0.36246	-0.13788	0.422943
intPF14_0668_2	PF14_0667a	0.14999	0.37257	0.54149	0.23787	-0.43754
aPF14_0668_3	PF14_0668	0.14999	0.37257	0.54149	0.23787	-0.43754
aPF14_0668_2	PF14_0668	0.14999	0.37257	0.54149	0.23787	-0.43754
aPF14_0668_1	PF14_0668	0.14999	0.37257	0.54149	0.23787	-0.43754
aPF14_0668_0	PF14_0668	0.14999	0.37257	0.54149	0.23787	-0.43754
intPF14_0670_2	PF14_0669	1.123603	0.457963	0.394073	-0.27241	-1.2003
aPF14_0669_0	PF14_0669	1.21456	0.76718	0.77213	-0.26413	-1.70518
intPF14_0669_2	PF14_0670	1.21456	0.76718	0.77213	-0.26413	-1.70518
aPF14_0670_0	PF14_0670	1.123603	0.457963	0.394073	-0.27241	-1.2003
aPF14_0671_0	PF14_0671	-0.0598	-0.42222	-0.36908	0.080878	0.536408
intPF14_0672_2	PF14_0672	-0.28379	-0.46558	-0.39309	0.075253	0.617743
intPF14_0671_2	PF14_0672	-0.0598	-0.42222	-0.36908	0.080878	0.536408

aPF14_0672_1	PF14_0672	-0.28379	-0.46558	-0.39309	0.075253	0.617743
aPF14_0672_0	PF14_0672	-0.28379	-0.46558	-0.39309	0.075253	0.617743
aPF14_0673_1	PF14_0673	-0.09956	0.296303	0.870633	0.464533	-0.66864
aPF14_0673_0	PF14_0673	-0.09956	0.296303	0.870633	0.464533	-0.66864
intPF14_0674_3	PF14_0674	-0.22788	0.287893	0.797213	0.468953	-0.49023
intPF14_0673_2	PF14_0674	-0.09956	0.296303	0.870633	0.464533	-0.66864
aPF14_0674_1	PF14_0674	-0.22788	0.287893	0.797213	0.468953	-0.49023
aPF14_0674_0	PF14_0674	-0.22788	0.287893	0.797213	0.468953	-0.49023
intPF14_0676_2	PF14_0675	-0.53425	-0.32271	0.055917	0.538417	0.668047
intPF14_0675_2	PF14_0675	-0.84257	-1.04915	-0.50369	0.59219	1.39315
aPF14_0675_1	PF14_0675	-0.84257	-1.04915	-0.50369	0.59219	1.39315
aPF14_0675_0	PF14_0675	-0.84257	-1.04915	-0.50369	0.59219	1.39315
aPF14_0676_0	PF14_0676	-0.53425	-0.32271	0.055917	0.538417	0.668047
intPF14_0677_3	PF14_0677	0.909647	0.887287	0.891677	-0.12534	-1.53445
aPF14_0677_1	PF14_0677	0.909647	0.887287	0.891677	-0.12534	-1.53445
aPF14_0677_0	PF14_0677	0.909647	0.887287	0.891677	-0.12534	-1.53445
intPF14_0678_2	PF14_0678	0.899067	-0.78785	0.956887	0.793907	-1.38835
aPF14_0678_0	PF14_0678	0.899067	-0.78785	0.956887	0.793907	-1.38835
intPF14_0679_2	PF14_0679	-1.12806	-0.63866	0.014327	0.720007	1.003997
aPF14_0679_1	PF14_0679	-1.12806	-0.63866	0.014327	0.720007	1.003997
aPF14_0679_0	PF14_0679	-1.12806	-0.63866	0.014327	0.720007	1.003997
intPF14_0680_2	PF14_0680	-0.92041	-0.72198	0.092193	0.617753	0.583083
aPF14_0680_1	PF14_0680	-0.92041	-0.72198	0.092193	0.617753	0.583083
aPF14_0680_0	PF14_0680	-0.92041	-0.72198	0.092193	0.617753	0.583083
intPF14_0681_2	PF14_0681	-0.99449	-1.17803	-0.67894	0.38844	1.37353
aPF14_0681_1	PF14_0681	-0.99449	-1.17803	-0.67894	0.38844	1.37353
aPF14_0681_0	PF14_0681	-0.99449	-1.17803	-0.67894	0.38844	1.37353
intPF14_0682_3	PF14_0682	-0.27877	-0.69713	-0.81374	-0.13526	0.91713
aPF14_0682_0	PF14_0682	-0.27877	-0.69713	-0.81374	-0.13526	0.91713
intPF14_0684_2	PF14_0683	-0.71747	-0.40163	-0.34524	0.083077	0.728967
intPF14_0683_2	PF14_0683					
aPF14_0683_1	PF14_0683					
aPF14_0683_0	PF14_0683					
intPF14_0685_2	PF14_0684	-0.4926	-0.39256	0.647568	0.785728	-0.10614
aPF14_0684_1	PF14_0684	-0.71747	-0.40163	-0.34524	0.083077	0.728967
aPF14_0684_0	PF14_0684	-0.71747	-0.40163	-0.34524	0.083077	0.728967
aPF14_0685_0	PF14_0685	-0.4926	-0.39256	0.647568	0.785728	-0.10614
intPF14_0688_2	PF14_0686	0.781917	0.856417	0.347667	-0.43733	-0.98308
intPF14_0686_2	PF14_0686	1.341377	0.828137	0.139727	-0.47139	-0.8583
aPF14_0687_1	PF14_0686	1.208483	0.880083	0.193593	-0.47993	-0.89092
aPF14_0687_0	PF14_0686	1.208483	0.880083	0.193593	-0.47993	-0.89092
aPF14_0686_1	PF14_0686	1.341377	0.828137	0.139727	-0.47139	-0.8583
aPF14_0686_0	PF14_0686	1.341377	0.828137	0.139727	-0.47139	-0.8583
aPF14_0688_0	PF14_0688	0.781917	0.856417	0.347667	-0.43733	-0.98308
intPF14_0689_2	PF14_0689	0.21325	-0.04425	0.128	0.077	-0.21975
aPF14_0689_0	PF14_0689	0.21325	-0.04425	0.128	0.077	-0.21975
intPF14_0690_2	PF14_0690	0.913502	0.538502	-0.121	-0.58431	-0.60014

aPF14_0690_3	PF14_0690	0.913502	0.538502	-0.121	-0.58431	-0.60014
aPF14_0690_2	PF14_0690	0.913502	0.538502	-0.121	-0.58431	-0.60014
aPF14_0690_1	PF14_0690	0.913502	0.538502	-0.121	-0.58431	-0.60014
aPF14_0690_0	PF14_0690	0.913502	0.538502	-0.121	-0.58431	-0.60014
intPF14_0692_2	PF14_0691	0.140753	0.156733	0.089293	-0.13463	-0.30807
intPF14_0691_1	PF14_0691	-0.02604	-0.38934	-0.25482	-0.04352	0.151003
aPF14_0691_1	PF14_0691	-0.02604	-0.38934	-0.25482	-0.04352	0.151003
aPF14_0691_0	PF14_0691	-0.02604	-0.38934	-0.25482	-0.04352	0.151003
aPF14_0692_1	PF14_0692	0.140753	0.156733	0.089293	-0.13463	-0.30807
aPF14_0692_0	PF14_0692	0.140753	0.156733	0.089293	-0.13463	-0.30807
aPF14_0693_0	PF14_0693	-0.00267	-0.06465	-0.74418	-0.9166	-0.05278
intPF14_0694_2	PF14_0694	0.224777	-0.86588	-0.99949	-0.45567	0.496717
intPF14_0693_2	PF14_0694	-0.00267	-0.06465	-0.74418	-0.9166	-0.05278
aPF14_0694_1	PF14_0694	0.224777	-0.86588	-0.99949	-0.45567	0.496717
aPF14_0694_0	PF14_0694	0.224777	-0.86588	-0.99949	-0.45567	0.496717
intPF14_0695_2	PF14_0695	-0.50584	-1.1228	-0.40089	0.334727	0.657137
aPF14_0695_1	PF14_0695	-0.50584	-1.1228	-0.40089	0.334727	0.657137
aPF14_0695_0	PF14_0695	-0.50584	-1.1228	-0.40089	0.334727	0.657137
intPF14_0697_2	PF14_0696	0.72459	0.32705	0.60339	0.07111	-1.02305
aPF14_0696_1	PF14_0696	-0.09536	-0.21419	-0.26536	-0.0673	0.274527
aPF14_0696_0	PF14_0696	-0.09536	-0.21419	-0.26536	-0.0673	0.274527
intPF14_0696_2	PF14_0697	-0.09536	-0.21419	-0.26536	-0.0673	0.274527
aPF14_0697_1	PF14_0697	0.72459	0.32705	0.60339	0.07111	-1.02305
aPF14_0697_0	PF14_0697	0.72459	0.32705	0.60339	0.07111	-1.02305
aPF14_0698_0	PF14_0698	1.780837	0.265797	0.120917	-0.32758	-1.12946
intPF14_0699_2	PF14_0699	0.4122	0.21349	0.07452	0.04248	-0.06799
intPF14_0698_2	PF14_0699	1.780837	0.265797	0.120917	-0.32758	-1.12946
aPF14_0699_1	PF14_0699	0.4122	0.21349	0.07452	0.04248	-0.06799
aPF14_0699_0	PF14_0699	0.4122	0.21349	0.07452	0.04248	-0.06799
intPF14_0700_1	PF14_0700	-1.38667	-0.48469	0.502123	0.972543	0.677353
aPF14_0700_1	PF14_0700	-1.38667	-0.48469	0.502123	0.972543	0.677353
aPF14_0700_0	PF14_0700	-1.38667	-0.48469	0.502123	0.972543	0.677353
intPF14_0701_2	PF14_0701	-0.459	-0.62499	-0.44052	0.34902	1.06749
aPF14_0702_1	PF14_0701	-0.80765	-0.33028	-0.0137	0.425863	0.712943
aPF14_0702_0	PF14_0701	-0.80765	-0.33028	-0.0137	0.425863	0.712943
aPF14_0701_1	PF14_0701	-0.459	-0.62499	-0.44052	0.34902	1.06749
aPF14_0701_0	PF14_0701	-0.459	-0.62499	-0.44052	0.34902	1.06749
intPF14_0703_2	PF14_0703	1.33921	0.39894	-0.94238	-1.44412	-0.61894
aPF14_0703_1	PF14_0703	1.33921	0.39894	-0.94238	-1.44412	-0.61894
aPF14_0703_0	PF14_0703	1.33921	0.39894	-0.94238	-1.44412	-0.61894
intPF14_0704_3	PF14_0704	0.91086	0.17269	0.49911	0.12589	-0.91469
aPF14_0704_1	PF14_0704	0.91086	0.17269	0.49911	0.12589	-0.91469
aPF14_0704_0	PF14_0704	0.91086	0.17269	0.49911	0.12589	-0.91469
aPF14_0705_0	PF14_0705	-0.20135	-0.73225	1.05201	1.22199	-0.51675
intPF14_0706_2	PF14_0706	0.204827	0.867387	0.222237	-0.5774	-0.76355
intPF14_0705_2	PF14_0706	-0.20135	-0.73225	1.05201	1.22199	-0.51675
aPF14_0706_1	PF14_0706	0.204827	0.867387	0.222237	-0.5774	-0.76355

aPF14_0706_0	PF14_0706	0.204827	0.867387	0.222237	-0.5774	-0.76355
intPF14_0707_2	PF14_0707	0.992973	1.094633	0.505493	-0.68333	-1.55297
aPF14_0707_1	PF14_0707	0.992973	1.094633	0.505493	-0.68333	-1.55297
aPF14_0707_0	PF14_0707	0.992973	1.094633	0.505493	-0.68333	-1.55297
intPF14_0708_2	PF14_0708	-0.0309	0.405668	-0.02124	-0.3547	-0.21236
aPF14_0708_3	PF14_0708	-0.0309	0.405668	-0.02124	-0.3547	-0.21236
aPF14_0708_2	PF14_0708	-0.0309	0.405668	-0.02124	-0.3547	-0.21236
aPF14_0708_1	PF14_0708	-0.0309	0.405668	-0.02124	-0.3547	-0.21236
aPF14_0708_0	PF14_0708	-0.0309	0.405668	-0.02124	-0.3547	-0.21236
intPF14_0709_2	PF14_0709	0.806087	0.429587	0.152837	-0.1165	-0.43725
aPF14_0709_0	PF14_0709	0.806087	0.429587	0.152837	-0.1165	-0.43725
intPF14_0710_2	PF14_0710	0.456008	-0.25354	-0.37024	-0.04231	0.294278
aPF14_0710_6	PF14_0710	0.456008	-0.25354	-0.37024	-0.04231	0.294278
aPF14_0710_5	PF14_0710	0.456008	-0.25354	-0.37024	-0.04231	0.294278
aPF14_0710_4	PF14_0710	0.456008	-0.25354	-0.37024	-0.04231	0.294278
aPF14_0710_3	PF14_0710	0.456008	-0.25354	-0.37024	-0.04231	0.294278
aPF14_0710_2	PF14_0710	0.456008	-0.25354	-0.37024	-0.04231	0.294278
aPF14_0710_1	PF14_0710	0.456008	-0.25354	-0.37024	-0.04231	0.294278
aPF14_0710_0	PF14_0710	0.456008	-0.25354	-0.37024	-0.04231	0.294278
intPF14_0712_2	PF14_0711	-0.9034	-0.36014	-0.30442	0.244287	0.985587
intPF14_0711_2	PF14_0711	-0.62291	-0.30245	0.437893	0.713273	0.273113
aPF14_0711_1	PF14_0711	-0.62291	-0.30245	0.437893	0.713273	0.273113
aPF14_0711_0	PF14_0711	-0.62291	-0.30245	0.437893	0.713273	0.273113
aPF14_0712_4	PF14_0712	-0.9034	-0.36014	-0.30442	0.244287	0.985587
aPF14_0712_3	PF14_0712	-0.9034	-0.36014	-0.30442	0.244287	0.985587
aPF14_0712_2	PF14_0712	-0.9034	-0.36014	-0.30442	0.244287	0.985587
aPF14_0712_1	PF14_0712	-0.9034	-0.36014	-0.30442	0.244287	0.985587
aPF14_0712_0	PF14_0712	-0.9034	-0.36014	-0.30442	0.244287	0.985587
intPF14_0713_2	PF14_0713	0.04526	0.0892	0.19735	0.06215	-0.2242
aPF14_0713_1	PF14_0713	0.04526	0.0892	0.19735	0.06215	-0.2242
aPF14_0713_0	PF14_0713	0.04526	0.0892	0.19735	0.06215	-0.2242
intPF14_0714_2	PF14_0714	0.116493	-0.01096	0.068923	-0.09526	-0.31538
aPF14_0714_1	PF14_0714	0.116493	-0.01096	0.068923	-0.09526	-0.31538
aPF14_0714_0	PF14_0714	0.116493	-0.01096	0.068923	-0.09526	-0.31538
intPF14_0716_2	PF14_0715	-0.10719	-0.54907	0.115633	0.678033	0.474733
intPF14_0715_2	PF14_0715	0.802727	0.486847	0.280047	-0.15871	-0.69451
aPF14_0715_1	PF14_0715	0.802727	0.486847	0.280047	-0.15871	-0.69451
aPF14_0715_0	PF14_0715	0.802727	0.486847	0.280047	-0.15871	-0.69451
aPF14_0716_0	PF14_0716	-0.10719	-0.54907	0.115633	0.678033	0.474733
intPF14_0717_3	PF14_0717	-0.69675	-1.05229	-0.09142	0.833083	0.947953
aPF14_0717_0	PF14_0717	-0.69675	-1.05229	-0.09142	0.833083	0.947953
intPF14_0718_2	PF14_0718	0.227323	1.043393	0.373213	-0.36155	-0.60673
aPF14_0718_0	PF14_0718	0.227323	1.043393	0.373213	-0.36155	-0.60673
intPF14_0720_2	PF14_0719	-0.78204	-0.64179	0.07133	0.57117	0.55879
intPF14_0719_2	PF14_0719	-0.08166	0.720638	0.212218	-0.19266	-0.17042
aPF14_0719_2	PF14_0719	-0.08166	0.720638	0.212218	-0.19266	-0.17042
aPF14_0719_1	PF14_0719	-0.08166	0.720638	0.212218	-0.19266	-0.17042

aPF14_0719_0	PF14_0719	-0.08166	0.720638	0.212218	-0.19266	-0.17042
intPF14_0721_2	PF14_0720	-1.16115	-0.38527	0.47503	0.83397	0.51027
aPF14_0720_1	PF14_0720	-0.78204	-0.64179	0.07133	0.57117	0.55879
aPF14_0720_0	PF14_0720	-0.78204	-0.64179	0.07133	0.57117	0.55879
aPF14_0721_0	PF14_0721	-1.16115	-0.38527	0.47503	0.83397	0.51027
intPF14_0722_2	PF14_0722	0.062123	0.233863	0.155523	0.007143	-0.1392
aPF14_0722_8	PF14_0722	0.062123	0.233863	0.155523	0.007143	-0.1392
aPF14_0722_7	PF14_0722	0.062123	0.233863	0.155523	0.007143	-0.1392
aPF14_0722_6	PF14_0722	0.062123	0.233863	0.155523	0.007143	-0.1392
aPF14_0722_5	PF14_0722	0.062123	0.233863	0.155523	0.007143	-0.1392
aPF14_0722_4	PF14_0722	0.062123	0.233863	0.155523	0.007143	-0.1392
aPF14_0722_3	PF14_0722	0.062123	0.233863	0.155523	0.007143	-0.1392
aPF14_0722_2	PF14_0722	0.062123	0.233863	0.155523	0.007143	-0.1392
aPF14_0722_1	PF14_0722	0.062123	0.233863	0.155523	0.007143	-0.1392
aPF14_0722_0	PF14_0722	0.062123	0.233863	0.155523	0.007143	-0.1392
intPF14_0723_2	PF14_0723					
aPF14_0723_2	PF14_0723					
aPF14_0723_1	PF14_0723					
aPF14_0723_0	PF14_0723					
intPF14_0724_2	PF14_0724	0.820153	0.783213	0.214813	-0.62765	-1.08655
aPF14_0724_1	PF14_0724	0.820153	0.783213	0.214813	-0.62765	-1.08655
aPF14_0724_0	PF14_0724	0.820153	0.783213	0.214813	-0.62765	-1.08655
intPF14_0725_2	PF14_0725	0.1513	0.50548	-0.05421	-0.41729	-0.23998
aPF14_0725_1	PF14_0725	0.1513	0.50548	-0.05421	-0.41729	-0.23998
aPF14_0725_0	PF14_0725	0.1513	0.50548	-0.05421	-0.41729	-0.23998
intPF14_0726_2	PF14_0726	0.243562	-0.2261	0.192042	0.022862	-0.54971
aPF14_0726_0	PF14_0726	0.243562	-0.2261	0.192042	0.022862	-0.54971
intPF14_0727_2	PF14_0727	0.815353	0.407253	-0.17601	-0.39933	-0.27659
aPF14_0727_1	PF14_0727	0.815353	0.407253	-0.17601	-0.39933	-0.27659
aPF14_0727_0	PF14_0727	0.815353	0.407253	-0.17601	-0.39933	-0.27659
intPF14_0728_2	PF14_0728	1.996648	1.792608	0.931848	-1.00808	-2.69099
aPF14_0728_0	PF14_0728	1.996648	1.792608	0.931848	-1.00808	-2.69099
intPF14_0729_2	PF14_0729					
aPF14_0729_0	PF14_0729					
intPF14_0731_2	PF14_0730	-0.5004	0.215807	2.082287	1.116647	-1.83187
intPF14_0730_2	PF14_0730	-0.85782	0.52609	1.99133	0.96467	-1.56709
aPF14_0731_0	PF14_0730	-0.5004	0.215807	2.082287	1.116647	-1.83187
aPF14_0730_0	PF14_0730	-0.85782	0.52609	1.99133	0.96467	-1.56709
intPF14_0732_2	PF14_0732	-0.01261	0.212077	-0.30515	-0.52051	-0.07574
aPF14_0732_1	PF14_0732	-0.01261	0.212077	-0.30515	-0.52051	-0.07574
aPF14_0732_0	PF14_0732	-0.01261	0.212077	-0.30515	-0.52051	-0.07574
intPF14_0734_2	PF14_0733	1.875088	0.738858	0.223988	-1.29751	-2.48938
intPF14_0733_2	PF14_0733	2.158403	0.568973	0.046543	-1.35988	-2.46531
aPF14_0733_0	PF14_0733	2.158403	0.568973	0.046543	-1.35988	-2.46531
aPF14_0734_0	PF14_0734	1.875088	0.738858	0.223988	-1.29751	-2.48938
intPF14_0735_2	PF14_0735	2.13499	1.59806	-0.53212	-1.62688	-1.11106
aPF14_0735_0	PF14_0735	2.13499	1.59806	-0.53212	-1.62688	-1.11106

intPF14_0736_3	PF14_0736	1.724983	1.013563	-0.13812	-0.86422	-0.9424
aPF14_0736_1	PF14_0736	1.724983	1.013563	-0.13812	-0.86422	-0.9424
aPF14_0736_0	PF14_0736	1.724983	1.013563	-0.13812	-0.86422	-0.9424
intPF14_0737_2	PF14_0737	0.476437	-0.11318	0.604867	0.394467	-0.75148
aPF14_0737_0	PF14_0737	0.476437	-0.11318	0.604867	0.394467	-0.75148
intPF14_0738_2	PF14_0738	1.078617	1.194907	-0.16481	-1.11385	-0.93157
aPF14_0738_0	PF14_0738	1.078617	1.194907	-0.16481	-1.11385	-0.93157
aPF14_0739_0	PF14_0739a	1.70132	0.96239	0.19271	-0.70771	-1.34039
intPF14_0741_2	PF14_0740	-0.63752	0.4831	2.0798	0.9572	-1.8426
intPF14_0740_2	PF14_0740	1.821483	0.001083	0.172343	-0.19068	-1.20842
aPF14_0740_1	PF14_0740	1.821483	0.001083	0.172343	-0.19068	-1.20842
aPF14_0740_0	PF14_0740	1.821483	0.001083	0.172343	-0.19068	-1.20842
intPF14_0742_2	PF14_0742	-0.43962	-0.13391	1.593813	1.018853	-1.33643
aPF14_0742_0	PF14_0742	-0.43962	-0.13391	1.593813	1.018853	-1.33643
intPF14_0743_2	PF14_0743	-0.90615	-0.0553	1.552755	1.082095	-0.95841
aPF14_0743_0	PF14_0743	-0.90615	-0.0553	1.552755	1.082095	-0.95841
intPF14_0744_2	PF14_0744	-0.08573	0.68763	0.43568	-0.01378	-0.33243
aPF14_0744_0	PF14_0744	-0.08573	0.68763	0.43568	-0.01378	-0.33243
intPF14_0745_2	PF14_0745	0.27413	0.41935	0.37231	-0.05331	-0.57435
aPF14_0745_0	PF14_0745	0.27413	0.41935	0.37231	-0.05331	-0.57435
intPF14_0746_2	PF14_0746	-0.21549	1.33346	1.77083	0.19517	-1.99296
aPF14_0746_1	PF14_0746	-0.21549	1.33346	1.77083	0.19517	-1.99296
aPF14_0746_0	PF14_0746	-0.21549	1.33346	1.77083	0.19517	-1.99296
aPF14_0747_2	PF14_0747	-0.28158	0.040533	0.076753	0.058323	0.067643
aPF14_0747_0	PF14_0747	-0.28158	0.040533	0.076753	0.058323	0.067643
intPF14_0748_2	PF14_0748	0.131137	0.337327	0.217847	-0.01451	-0.23999
aPF14_0748_0	PF14_0748	0.131137	0.337327	0.217847	-0.01451	-0.23999
intPF14_0749_2	PF14_0749	2.247347	1.143257	-0.5805	-2.30416	-2.37892
aPF14_0749_0	PF14_0749	2.247347	1.143257	-0.5805	-2.30416	-2.37892
intPF14_0751_2	PF14_0751	1.630027	0.667877	-0.12226	-0.8384	-1.13154
aPF14_0751_1	PF14_0751	1.630027	0.667877	-0.12226	-0.8384	-1.13154
aPF14_0751_0	PF14_0751	1.630027	0.667877	-0.12226	-0.8384	-1.13154
intPF14_0752_2	PF14_0752	2.439373	2.275113	0.077273	-2.03961	-2.49745
aPF14_0752_0	PF14_0752	2.439373	2.275113	0.077273	-2.03961	-2.49745
intPF14_0753_3	PF14_0753	1.79981	1.38995	-1.1449	-2.0931	-0.71395
aPF14_0753_0	PF14_0753	1.79981	1.38995	-1.1449	-2.0931	-0.71395
intPF14_0755_2	PF14_0754	-0.14529	0.43043	0.71364	0.07036	-0.86743
aPF14_0754_0	PF14_0754	-0.59242	-0.30992	-0.12617	0.32317	0.70692
intPF14_0754_2	PF14_0755	-0.59242	-0.30992	-0.12617	0.32317	0.70692
aPF14_0755_0	PF14_0755	-0.14529	0.43043	0.71364	0.07036	-0.86743
intPF14_0756_2	PF14_0756	0.099072	0.332392	0.494632	0.206972	-0.40779
aPF14_0756_1	PF14_0756	0.099072	0.332392	0.494632	0.206972	-0.40779
aPF14_0756_0	PF14_0756	0.099072	0.332392	0.494632	0.206972	-0.40779
intPF14_0757_2	PF14_0757	1.171268	0.453288	0.154748	-0.30253	-0.82572
aPF14_0757_0	PF14_0757	1.171268	0.453288	0.154748	-0.30253	-0.82572
aPF14_0758_1	PF14_0758	-0.83914	0.476237	1.805287	0.908047	-1.3504
aPF14_0758_0	PF14_0758	-0.83914	0.476237	1.805287	0.908047	-1.3504

intPF14_0759_2	PF14_0759	-1.14713	0.1939	1.70974	0.84336	-1.35785
aPF14_0759_0	PF14_0759	-1.14713	0.1939	1.70974	0.84336	-1.35785
intPF14_0760_2	PF14_0760	1.124963	1.017203	-0.0004	-1.22894	-1.53954
aPF14_0760_0	PF14_0760	1.124963	1.017203	-0.0004	-1.22894	-1.53954
intPF14_0761_2	PF14_0761	2.90626	1.49622	-0.81444	-2.37106	-2.03472
aPF14_0761_1	PF14_0761	2.90626	1.49622	-0.81444	-2.37106	-2.03472
aPF14_0761_0	PF14_0761	2.90626	1.49622	-0.81444	-2.37106	-2.03472
intPF14_0762_2	PF14_0762	-0.69592	-0.61168	0.419133	0.690053	0.090863
intPF14_0763_2	PF14_0763					
aPF14_0763_0	PF14_0763					
intPF14_0764_2	PF14_0764					
intPF14_0774_2	PF14_0774	-0.29025	-0.53972	-0.37331	0.083147	0.546057
intPF14_0202_2	PF14_0774	-0.17218	0.663893	0.442713	-0.05905	-0.40023
aPF14_0774_1	PF14_0774	-0.29025	-0.53972	-0.37331	0.083147	0.546057
aPF14_0774_0	PF14_0774	-0.29025	-0.53972	-0.37331	0.083147	0.546057
intPF14_0775_2	PF14_0775	0.426023	0.165903	0.110203	-0.05654	-0.31824
aPF14_0775_0	PF14_0775	0.426023	0.165903	0.110203	-0.05654	-0.31824
intPF14_0778_2	PF14_0778-a					
intPF14_0109_2	PF14_0778-a	0.30296	-0.27778	-0.55544	-0.63556	-0.22122
aPF14_0109_0	PF14_0778-a	0.30296	-0.27778	-0.55544	-0.63556	-0.22122
intPF14_0781_2	PF14_0781					
intPF14_0424_2	PF14_0781	-0.583	-0.86398	0.135457	0.836877	0.625317
intPF14_0783_2	PF14_0783					
intPF14_0399_2	PF14_0783	-0.20612	0.365597	1.095807	0.605527	-0.78826
intPF14_0784_2	PF14_0784					
intPF14_0312_2	PF14_0784	0.11202	-0.25758	0.06041	0.31059	0.14608
intPF14_0785_2	PF14_0785					
intPF14_0211_2	PF14_0787	-0.38585	-0.75877	-0.44145	0.27845	0.84977
intPF14_0043_2	PF14_0788-a	1.19012	0.91388	-0.20477	-0.82823	-0.65438
aPF14_0043_1	PF14_0788-a	1.19012	0.91388	-0.20477	-0.82823	-0.65438
aPF14_0043_0	PF14_0788-a	1.19012	0.91388	-0.20477	-0.82823	-0.65438
intPF14_0788_2	PF14_0788-c					
intPF14_0790_2	PF14_0790					
intPF14_0791_2	PF14_0791					
intPF14_0805_2	PF14_0805					
intPF14_0166_2	PF14_0805	-1.40071	0.053547	0.809647	0.714687	0.090287
intPF14_0810_2	PF14_0810					
intPF14_0033_2	PF14_0810	-0.17068	-0.95961	-0.64529	0.13229	0.79861
intPF14_0815_2	PF14_0815					
intPF14_0466_2	PF14_0815	0.847967	0.161177	0.230147	0.148187	-0.37484
intPFA0015c_2	PFA0015c	1.601813	0.756933	0.276133	-0.34647	-1.05527
aPFA0015c_1	PFA0015c	1.601813	0.756933	0.276133	-0.34647	-1.05527
aPFA0015c_0	PFA0015c	1.601813	0.756933	0.276133	-0.34647	-1.05527
intPFA0035c_2	PFA0035c	0.269672	0.855772	0.914512	0.061192	-1.10418
intPFA0055c_2	PFA0055c	2.24892	1.62338	0.60075	-1.11175	-2.43638
intPFA0060w_2	PFA0060w	1.933208	2.033488	0.598248	-1.36628	-2.43497
intPFA0065w_2	PFA0065w					

aPFA0065w_0	PFA0065w					
aPFA0100c_0	PFA0100c	1.84333	1.95035	0.92029	-1.00729	-2.51235
intPFA0100c_2	PFA0105w	1.84333	1.95035	0.92029	-1.00729	-2.51235
intPFA0110w_2	PFA0110w	3.307903	0.803473	-1.55946	-2.96588	-2.15681
aPFA0110w_1	PFA0110w	3.307903	0.803473	-1.55946	-2.96588	-2.15681
aPFA0110w_0	PFA0110w	3.307903	0.803473	-1.55946	-2.96588	-2.15681
intPFA0115w_2	PFA0115w	-0.98979	-1.31407	0.37464	1.43536	0.92007
aPFA0115w_0	PFA0115w	-0.98979	-1.31407	0.37464	1.43536	0.92007
intPFA0120c_2	PFA0120c	0.477003	-0.523	-0.412	-0.06334	0.152663
aPFA0120c_0	PFA0120c	0.477003	-0.523	-0.412	-0.06334	0.152663
intPFA0125c_2	PFA0125c	1.538698	0.062498	-2.15867	-2.65433	-0.49516
aPFA0125c_2	PFA0125c	1.538698	0.062498	-2.15867	-2.65433	-0.49516
aPFA0125c_1	PFA0125c	1.538698	0.062498	-2.15867	-2.65433	-0.49516
aPFA0125c_0	PFA0125c	1.538698	0.062498	-2.15867	-2.65433	-0.49516
intPFA0130c_2	PFA0130c	-0.07523	0.744153	1.593203	0.374463	-1.83799
aPFA0130c_1	PFA0130c	-0.07523	0.744153	1.593203	0.374463	-1.83799
aPFA0130c_0	PFA0130c	-0.07523	0.744153	1.593203	0.374463	-1.83799
intPFA0135w_2	PFA0135w	1.850307	0.549457	-0.3739	-1.56476	-1.91212
aPFA0135w_0	PFA0135w	1.850307	0.549457	-0.3739	-1.56476	-1.91212
aPFA0140c_1	PFA0140c	-0.55415	-0.36622	0.184207	0.485127	0.330557
aPFA0140c_0	PFA0140c	-0.55415	-0.36622	0.184207	0.485127	0.330557
intPFA0145c_2	PFA0145c	-0.25395	0.13625	0.8955	0.676	-0.45225
intPFA0140c_2	PFA0145c	-0.55415	-0.36622	0.184207	0.485127	0.330557
aPFA0145c_1	PFA0145c	-0.25395	0.13625	0.8955	0.676	-0.45225
aPFA0145c_0	PFA0145c	-0.25395	0.13625	0.8955	0.676	-0.45225
intPFA0150c_2	PFA0150c	-1.00839	-0.96304	-0.63643	0.45743	1.54704
aPFA0150c_0	PFA0150c	-1.00839	-0.96304	-0.63643	0.45743	1.54704
intPFA0155c_2	PFA0155c	-0.67021	-0.45094	-0.55846	-0.01832	0.91916
aPFA0155c_2	PFA0155c	-0.67021	-0.45094	-0.55846	-0.01832	0.91916
aPFA0155c_1	PFA0155c	-0.67021	-0.45094	-0.55846	-0.01832	0.91916
aPFA0155c_0	PFA0155c	-0.67021	-0.45094	-0.55846	-0.01832	0.91916
intPFA0160c_2	PFA0160c	-1.14744	-0.19129	1.075597	1.149737	0.007627
aPFA0160c_1	PFA0160c	-1.14744	-0.19129	1.075597	1.149737	0.007627
aPFA0160c_0	PFA0160c	-1.14744	-0.19129	1.075597	1.149737	0.007627
intPFA0165c_2	PFA0165c	0.66884	0.62981	-0.05963	-0.54437	-0.51031
aPFA0165c_1	PFA0165c	0.66884	0.62981	-0.05963	-0.54437	-0.51031
aPFA0165c_0	PFA0165c	0.66884	0.62981	-0.05963	-0.54437	-0.51031
intPFA0175w_2	PFA0170c	-0.49567	-0.47282	0.011223	0.313553	0.299763
intPFA0170c_2	PFA0170c	-0.14754	-0.1353	0.038602	-0.11861	-0.2897
aPFA0170c_2	PFA0170c	-0.14754	-0.1353	0.038602	-0.11861	-0.2897
aPFA0170c_1	PFA0170c	-0.14754	-0.1353	0.038602	-0.11861	-0.2897
aPFA0170c_0	PFA0170c	-0.14754	-0.1353	0.038602	-0.11861	-0.2897
intPFA0180w_2	PFA0175w	-0.7386	-0.09064	0.419278	0.456278	0.101528
aPFA0175w_2	PFA0175w	-0.49567	-0.47282	0.011223	0.313553	0.299763
aPFA0175w_1	PFA0175w	-0.49567	-0.47282	0.011223	0.313553	0.299763
aPFA0175w_0	PFA0175w	-0.49567	-0.47282	0.011223	0.313553	0.299763
intPFA0185w_2	PFA0180w	0.77885	0.73176	0.27397	-0.30147	-0.72626

aPFA0180w_2	PFA0180w	-0.7386	-0.09064	0.419278	0.456278	0.101528
aPFA0180w_1	PFA0180w	-0.7386	-0.09064	0.419278	0.456278	0.101528
aPFA0180w_0	PFA0180w	-0.7386	-0.09064	0.419278	0.456278	0.101528
aPFA0185w_1	PFA0185w	0.77885	0.73176	0.27397	-0.30147	-0.72626
aPFA0185w_0	PFA0185w	0.77885	0.73176	0.27397	-0.30147	-0.72626
aPFA0190c_1	PFA0190c	-0.08855	-0.14774	-0.93901	-0.54065	0.841577
aPFA0190c_0	PFA0190c	-0.08855	-0.14774	-0.93901	-0.54065	0.841577
intPFA0195w_2	PFA0195w	-0.91251	-0.42741	0.246817	0.729017	0.660247
intPFA0190c_2	PFA0195w	-0.08855	-0.14774	-0.93901	-0.54065	0.841577
aPFA0195w_1	PFA0195w	-0.91251	-0.42741	0.246817	0.729017	0.660247
aPFA0195w_0	PFA0195w	-0.91251	-0.42741	0.246817	0.729017	0.660247
intPFA0200w_2	PFA0200w	-0.4461	-0.53428	-0.68317	-0.03951	0.985723
aPFA0200w_0	PFA0200w	-0.4461	-0.53428	-0.68317	-0.03951	0.985723
intPFA0205w_2	PFA0205w	0.394617	-0.13858	-1.50783	-0.97533	1.077417
aPFA0205w_1	PFA0205w	0.394617	-0.13858	-1.50783	-0.97533	1.077417
aPFA0205w_0	PFA0205w	0.394617	-0.13858	-1.50783	-0.97533	1.077417
intPFA0210c_2	PFA0210c	-0.89903	-1.35681	0.142877	0.551957	-0.03885
aPFA0210c_1	PFA0210c	-0.89903	-1.35681	0.142877	0.551957	-0.03885
aPFA0210c_0	PFA0210c	-0.89903	-1.35681	0.142877	0.551957	-0.03885
intPFA0220w_2	PFA0220w	1.076113	0.381433	-0.31512	-1.21272	-1.34577
intPFA0215w_3	PFA0220w	1.162362	0.128862	-0.48976	-1.2006	-1.20456
aPFA0220w_1	PFA0220w	1.076113	0.381433	-0.31512	-1.21272	-1.34577
aPFA0220w_0	PFA0220w	1.076113	0.381433	-0.31512	-1.21272	-1.34577
aPFA0215w_3	PFA0220w	1.162362	0.128862	-0.48976	-1.2006	-1.20456
aPFA0215w_2	PFA0220w	1.162362	0.128862	-0.48976	-1.2006	-1.20456
aPFA0215w_1	PFA0220w	1.162362	0.128862	-0.48976	-1.2006	-1.20456
aPFA0215w_0	PFA0220w	1.162362	0.128862	-0.48976	-1.2006	-1.20456
intPFA0225w_2	PFA0225w	-0.20842	-0.9529	0.00129	0.58371	0.3074
aPFA0225w_1	PFA0225w	-0.20842	-0.9529	0.00129	0.58371	0.3074
aPFA0225w_0	PFA0225w	-0.20842	-0.9529	0.00129	0.58371	0.3074
intPFA0235w_2	PFA0230c	0.830833	0.852823	0.578863	-0.14931	-0.9936
aPFA0230c_0	PFA0230c	0.250597	0.420007	1.033997	0.506337	-0.93967
intPFA0240w_2	PFA0235w					
intPFA0230c_2	PFA0235w	0.250597	0.420007	1.033997	0.506337	-0.93967
aPFA0235w_2	PFA0235w	0.830833	0.852823	0.578863	-0.14931	-0.9936
aPFA0235w_1	PFA0235w	0.830833	0.852823	0.578863	-0.14931	-0.9936
aPFA0235w_0	PFA0235w	0.830833	0.852823	0.578863	-0.14931	-0.9936
aPFA0240w_1	PFA0240w					
aPFA0240w_0	PFA0240w					
intPFA0245w_2	PFA0245w	-0.52857	-1.20311	-0.45902	0.473857	0.926447
aPFA0245w_1	PFA0245w	-0.52857	-1.20311	-0.45902	0.473857	0.926447
aPFA0245w_0	PFA0245w	-0.52857	-1.20311	-0.45902	0.473857	0.926447
intPFA0250w_2	PFA0250w	-0.8243	-1.80342	0.236933	1.456473	0.822823
aPFA0250w_0	PFA0250w	-0.8243	-1.80342	0.236933	1.456473	0.822823
intPFA0255c_2	PFA0255c	-0.86067	-0.93615	0.50929	0.87171	0.03665
aPFA0255c_1	PFA0255c	-0.86067	-0.93615	0.50929	0.87171	0.03665
aPFA0255c_0	PFA0255c	-0.86067	-0.93615	0.50929	0.87171	0.03665

intPFA0260c_2	PFA0260c	-0.67412	-1.63136	-1.79027	-0.23039	2.073697
aPFA0260c_0	PFA0260c	-0.67412	-1.63136	-1.79027	-0.23039	2.073697
intPFA0265c_2	PFA0265c	0.0541	-0.35048	-1.65155	-1.14645	1.05648
aPFA0265c_0	PFA0265c	0.0541	-0.35048	-1.65155	-1.14645	1.05648
aPFA0270c_1	PFA0270c	-1.2098	-0.76204	0.663833	1.139333	0.387213
aPFA0270c_0	PFA0270c	-1.2098	-0.76204	0.663833	1.139333	0.387213
intPFA0275c_2	PFA0275c	0.526207	0.374967	0.282067	-0.05273	-0.52163
intPFA0270c_2	PFA0275c	-1.2098	-0.76204	0.663833	1.139333	0.387213
aPFA0275c_1	PFA0275c	0.526207	0.374967	0.282067	-0.05273	-0.52163
aPFA0275c_0	PFA0275c	0.526207	0.374967	0.282067	-0.05273	-0.52163
intPFA0280w_2	PFA0280w	-0.73039	-0.68126	-0.20828	0.36568	0.71966
aPFA0280w_4	PFA0280w	-0.73039	-0.68126	-0.20828	0.36568	0.71966
aPFA0280w_3	PFA0280w	-0.73039	-0.68126	-0.20828	0.36568	0.71966
aPFA0280w_2	PFA0280w	-0.73039	-0.68126	-0.20828	0.36568	0.71966
aPFA0280w_1	PFA0280w	-0.73039	-0.68126	-0.20828	0.36568	0.71966
aPFA0280w_0	PFA0280w	-0.73039	-0.68126	-0.20828	0.36568	0.71966
intPFA0290w_2	PFA0285c	-0.87624	-1.18078	-0.17119	0.65969	0.80828
aPFA0285c_1	PFA0285c	-1.01743	-1.14736	-0.67054	0.39154	1.39086
aPFA0285c_0	PFA0285c	-1.01743	-1.14736	-0.67054	0.39154	1.39086
intPFA0285c_2	PFA0290w	-1.01743	-1.14736	-0.67054	0.39154	1.39086
aPFA0290w_1	PFA0290w	-0.87624	-1.18078	-0.17119	0.65969	0.80828
aPFA0290w_0	PFA0290w	-0.87624	-1.18078	-0.17119	0.65969	0.80828
intPFA0295c_2	PFA0295c	-0.61564	-0.88231	-0.96264	-0.17436	1.12031
aPFA0295c_1	PFA0295c	-0.61564	-0.88231	-0.96264	-0.17436	1.12031
aPFA0295c_0	PFA0295c	-0.61564	-0.88231	-0.96264	-0.17436	1.12031
intPFA0300c_2	PFA0300c	-0.48173	-0.27236	0.34047	0.51203	0.12286
aPFA0300c_1	PFA0300c	-0.48173	-0.27236	0.34047	0.51203	0.12286
aPFA0300c_0	PFA0300c	-0.48173	-0.27236	0.34047	0.51203	0.12286
intPFA0305c_3	PFA0305c	-0.20724	-0.63683	-1.04305	-0.26025	1.166405
aPFA0305c_0	PFA0305c	-0.20724	-0.63683	-1.04305	-0.26025	1.166405
intPFA0310c_2	PFA0310c	-0.09662	-0.11494	-0.27038	0.063543	0.546603
aPFA0310c_1	PFA0310c	-0.09662	-0.11494	-0.27038	0.063543	0.546603
aPFA0310c_0	PFA0310c	-0.09662	-0.11494	-0.27038	0.063543	0.546603
intPFA0320w_2	PFA0315w	-0.7859	-0.53851	-0.60848	0.214368	1.340068
intPFA0315w_2	PFA0315w	0.67424	0.61179	1.03542	0.12758	-1.52379
aPFA0315w_0	PFA0315w	0.67424	0.61179	1.03542	0.12758	-1.52379
intPFA0325w_2	PFA0320w	-0.22026	-0.45069	-0.30562	-0.02838	0.29069
aPFA0320w_2	PFA0320w	-0.7859	-0.53851	-0.60848	0.214368	1.340068
aPFA0320w_1	PFA0320w	-0.7859	-0.53851	-0.60848	0.214368	1.340068
aPFA0320w_0	PFA0320w	-0.7859	-0.53851	-0.60848	0.214368	1.340068
aPFA0325w_0	PFA0325w	-0.22026	-0.45069	-0.30562	-0.02838	0.29069
intPFA0330w_2	PFA0330w	0.454805	0.601635	0.659405	-0.1303	-1.15486
aPFA0330w_2	PFA0330w	0.454805	0.601635	0.659405	-0.1303	-1.15486
aPFA0330w_1	PFA0330w	0.454805	0.601635	0.659405	-0.1303	-1.15486
aPFA0330w_0	PFA0330w	0.454805	0.601635	0.659405	-0.1303	-1.15486
intPFA0335w_3	PFA0335w					
aPFA0335w_0	PFA0335w					

intPFA0340w_3	PFA0340w	-1.38081	-1.35743	-1.49763	0.14063	2.51193
aPFA0340w_1	PFA0340w	-1.38081	-1.35743	-1.49763	0.14063	2.51193
aPFA0340w_0	PFA0340w	-1.38081	-1.35743	-1.49763	0.14063	2.51193
aPFA0345w_0	PFA0345w-a	-1.12448	-1.92898	-0.6031	1.178718	1.962598
intPFA0345w_2	PFA0345w-b	-1.12448	-1.92898	-0.6031	1.178718	1.962598
aPFA0350c_1	PFA0350c	-0.37867	-0.61661	0.590237	0.900597	-0.01605
aPFA0350c_0	PFA0350c	-0.37867	-0.61661	0.590237	0.900597	-0.01605
intPFA0350c_2	PFA0355w	-0.37867	-0.61661	0.590237	0.900597	-0.01605
aPFA0350w_1	PFA0355w	-1.0853	-0.44553	0.950043	1.134123	0.024193
aPFA0350w_0	PFA0355w	-1.0853	-0.44553	0.950043	1.134123	0.024193
aPFA0360c_1	PFA0360c	-0.00698	-0.09108	-0.09884	0.03384	0.17358
aPFA0360c_0	PFA0360c	-0.00698	-0.09108	-0.09884	0.03384	0.17358
intPFA0365c_2	PFA0365c					
intPFA0360c_2	PFA0365c	-0.00698	-0.09108	-0.09884	0.03384	0.17358
aPFA0365c_0	PFA0365c					
intPFA0370w_2	PFA0370w	-0.54354	-0.74832	-0.09336	0.619023	0.781983
aPFA0370w_0	PFA0370w	-0.54354	-0.74832	-0.09336	0.619023	0.781983
intPFA0375c_3	PFA0375c	0.76174	0.48463	0.27941	-0.60896	-1.35652
aPFA0375c_2	PFA0375c	0.76174	0.48463	0.27941	-0.60896	-1.35652
aPFA0375c_1	PFA0375c	0.76174	0.48463	0.27941	-0.60896	-1.35652
aPFA0375c_0	PFA0375c	0.76174	0.48463	0.27941	-0.60896	-1.35652
intPFA0385w_2	PFA0380w	-0.14471	-0.34498	0.344013	0.565253	0.035443
intPFA0380w_2	PFA0380w	0.799293	0.065553	-0.67361	-0.62273	0.078113
aPFA0380w_2	PFA0380w	0.799293	0.065553	-0.67361	-0.62273	0.078113
aPFA0380w_1	PFA0380w	0.799293	0.065553	-0.67361	-0.62273	0.078113
aPFA0380w_0	PFA0380w	0.799293	0.065553	-0.67361	-0.62273	0.078113
aPFA0385w_1	PFA0385w	-0.14471	-0.34498	0.344013	0.565253	0.035443
aPFA0385w_0	PFA0385w	-0.14471	-0.34498	0.344013	0.565253	0.035443
intPFA0390w_2	PFA0390w	-1.00581	-0.39941	0.237077	0.655257	0.617247
aPFA0390w_1	PFA0390w	-1.00581	-0.39941	0.237077	0.655257	0.617247
aPFA0390w_0	PFA0390w	-1.00581	-0.39941	0.237077	0.655257	0.617247
intPFA0395c_2	PFA0395w	-0.71625	-0.14473	0.83596	0.83804	-0.13327
aPFA0395c_0	PFA0395w	-0.71625	-0.14473	0.83596	0.83804	-0.13327
intPFA0400c_2	PFA0400c	-0.67283	-0.43864	0.10737	0.63203	0.68304
aPFA0400c_0	PFA0400c	-0.67283	-0.43864	0.10737	0.63203	0.68304
intPFA0405w_3	PFA0405w	-0.21028	-0.2881	-0.11329	0.226127	0.424937
aPFA0405w_1	PFA0405w	-0.21028	-0.2881	-0.11329	0.226127	0.424937
aPFA0405w_0	PFA0405w	-0.21028	-0.2881	-0.11329	0.226127	0.424937
intPFA0410w_2	PFA0410w	0.333378	0.123088	0.207988	-0.13343	-0.61053
aPFA0410w_3	PFA0410w	0.333378	0.123088	0.207988	-0.13343	-0.61053
aPFA0410w_2	PFA0410w	0.333378	0.123088	0.207988	-0.13343	-0.61053
aPFA0410w_1	PFA0410w	0.333378	0.123088	0.207988	-0.13343	-0.61053
aPFA0410w_0	PFA0410w	0.333378	0.123088	0.207988	-0.13343	-0.61053
intPFA0420w_1	PFA0415c	2.645163	1.143183	-1.14738	-2.62096	-1.94752
intPFA0415c_2	PFA0415c	0.90059	0.60004	0.29891	-0.41991	-1.07804
aPFA0415c_0	PFA0415c	0.90059	0.60004	0.29891	-0.41991	-1.07804
aPFA0420w_0	PFA0420w	2.645163	1.143183	-1.14738	-2.62096	-1.94752

aPFA0425c_0	PFA0425c	0.09382	-0.06232	-0.25534	-0.10864	0.23234
intPFA0430c_2	PFA0430c	-0.4516	-0.05951	0.134003	0.299663	0.314673
intPFA0425c_2	PFA0430c	0.09382	-0.06232	-0.25534	-0.10864	0.23234
aPFA0430c_1	PFA0430c	-0.4516	-0.05951	0.134003	0.299663	0.314673
aPFA0430c_0	PFA0430c	-0.4516	-0.05951	0.134003	0.299663	0.314673
intPFA0435w_2	PFA0435w	-0.38512	-0.24786	0.371227	0.490107	0.017697
aPFA0435w_1	PFA0435w	-0.38512	-0.24786	0.371227	0.490107	0.017697
aPFA0435w_0	PFA0435w	-0.38512	-0.24786	0.371227	0.490107	0.017697
intPFA0440w_2	PFA0440w	1.21125	-0.18823	-2.06504	-2.33296	-0.24277
aPFA0440w_0	PFA0440w	1.21125	-0.18823	-2.06504	-2.33296	-0.24277
intPFA0445w_2	PFA0445w	1.36564	0.50481	-1.80836	-2.27564	-0.21681
aPFA0445w_1	PFA0445w	1.36564	0.50481	-1.80836	-2.27564	-0.21681
aPFA0445w_0	PFA0445w	1.36564	0.50481	-1.80836	-2.27564	-0.21681
intPFA0450c_2	PFA0450c	-0.3698	-0.19102	0.438523	0.617143	0.117683
aPFA0450c_0	PFA0450c	-0.3698	-0.19102	0.438523	0.617143	0.117683
intPFA0455c_2	PFA0455c	-1.09019	-0.61505	0.3946	0.8894	0.56405
aPFA0455c_0	PFA0455c	-1.09019	-0.61505	0.3946	0.8894	0.56405
intPFA0460c_2	PFA0460c	-0.79882	-1.1759	0.577283	1.248383	0.296563
aPFA0460c_0	PFA0460c	-0.79882	-1.1759	0.577283	1.248383	0.296563
aPFA0465c_0	PFA0465c	-0.45493	0.294647	0.723717	0.493617	-0.23831
intPFA0465c_2	PFA0470c	-0.45493	0.294647	0.723717	0.493617	-0.23831
aPFA0470c_0	PFA0470c	0.377462	-0.08671	0.299612	0.253662	-0.35243
intPFA0470c_2	PFA0475c	0.377462	-0.08671	0.299612	0.253662	-0.35243
aPFA0475c_0	PFA0475c	1.079387	0.666077	0.248847	-0.41451	-0.99374
intPFA0480w_3	PFA0480w	0.430933	0.447323	0.304343	-0.03818	-0.46066
intPFA0475c_2	PFA0480w	1.079387	0.666077	0.248847	-0.41451	-0.99374
aPFA0480w_1	PFA0480w	0.430933	0.447323	0.304343	-0.03818	-0.46066
aPFA0480w_0	PFA0480w	0.430933	0.447323	0.304343	-0.03818	-0.46066
intPFA0485w_2	PFA0485w	-0.48342	-1.08339	-1.19695	0.067287	1.734227
intPFA0490w_2	PFA0485w-a	0.233507	-0.54754	-0.21142	0.244757	0.311877
aPFA0485w_1	PFA0485w-a	-0.48342	-1.08339	-1.19695	0.067287	1.734227
aPFA0485w_0	PFA0485w-a	-0.48342	-1.08339	-1.19695	0.067287	1.734227
aPFA0490w_0	PFA0490w	0.233507	-0.54754	-0.21142	0.244757	0.311877
intPFA0500w_2	PFA0495c	0.036785	0.617175	0.311715	-0.01835	-0.19981
aPFA0495c_1	PFA0495c	0.63378	1.06661	0.19603	-0.43803	-0.51661
aPFA0495c_0	PFA0495c	0.63378	1.06661	0.19603	-0.43803	-0.51661
intPFA0495c_2	PFA0500w	0.63378	1.06661	0.19603	-0.43803	-0.51661
aPFA0500w_0	PFA0500w	0.036785	0.617175	0.311715	-0.01835	-0.19981
aPFA0505c_0	PFA0505c	0.60523	0.24287	0.51526	0.19174	-0.69687
intPFA0510w_2	PFA0510w	-0.24357	-0.12818	0.398242	0.351092	-0.20774
intPFA0505c_2	PFA0510w	0.60523	0.24287	0.51526	0.19174	-0.69687
aPFA0510w_3	PFA0510w	-0.24357	-0.12818	0.398242	0.351092	-0.20774
aPFA0510w_2	PFA0510w	-0.24357	-0.12818	0.398242	0.351092	-0.20774
aPFA0510w_1	PFA0510w	-0.24357	-0.12818	0.398242	0.351092	-0.20774
aPFA0510w_0	PFA0510w	-0.24357	-0.12818	0.398242	0.351092	-0.20774
intPFA0515w_3	PFA0515w	0.456942	0.358612	0.124612	-0.33528	-0.64461
aPFA0515w_2	PFA0515w	0.456942	0.358612	0.124612	-0.33528	-0.64461

aPFA0515w_1	PFA0515w	0.456942	0.358612	0.124612	-0.33528	-0.64461
aPFA0515w_0	PFA0515w	0.456942	0.358612	0.124612	-0.33528	-0.64461
intPFA0520c_2	PFA0520c	-0.92396	-1.38017	0.646837	1.440997	0.363507
aPFA0520c_1	PFA0520c	-0.92396	-1.38017	0.646837	1.440997	0.363507
aPFA0520c_0	PFA0520c	-0.92396	-1.38017	0.646837	1.440997	0.363507
intPFA0525w_1	PFA0525w	-0.02199	0.138473	0.252323	0.082523	-0.23917
aPFA0525w_1	PFA0525w	-0.02199	0.138473	0.252323	0.082523	-0.23917
aPFA0525w_0	PFA0525w	-0.02199	0.138473	0.252323	0.082523	-0.23917
aPFA0530c_0	PFA0530c	0.426053	0.477723	0.877053	0.265613	-1.03306
intPFA0535c_3	PFA0535c	-0.52556	0.169133	-0.45436	-0.25315	0.741203
intPFA0530c_2	PFA0535c	0.426053	0.477723	0.877053	0.265613	-1.03306
aPFA0535c_2	PFA0535c	-0.52556	0.169133	-0.45436	-0.25315	0.741203
aPFA0535c_1	PFA0535c	-0.52556	0.169133	-0.45436	-0.25315	0.741203
aPFA0535c_0	PFA0535c	-0.52556	0.169133	-0.45436	-0.25315	0.741203
aPFA0540c_0	PFA0540c	-0.08528	-1.13959	-0.12981	0.750147	0.631927
intPFA0550w_2	PFA0545c	-1.17175	-1.09275	-0.113	0.894	1.21025
intPFA0545c_2	PFA0545c	-0.58752	-1.21641	-0.29618	0.77268	1.08691
intPFA0540c_2	PFA0545c	-0.08528	-1.13959	-0.12981	0.750147	0.631927
aPFA0545c_1	PFA0545c	-0.58752	-1.21641	-0.29618	0.77268	1.08691
aPFA0545c_0	PFA0545c	-0.58752	-1.21641	-0.29618	0.77268	1.08691
aPFA0550w_1	PFA0550w	-1.17175	-1.09275	-0.113	0.894	1.21025
aPFA0550w_0	PFA0550w	-1.17175	-1.09275	-0.113	0.894	1.21025
intPFA0555c_2	PFA0555c	-1.34027	-1.86869	-0.19512	1.085783	1.187353
aPFA0555c_1	PFA0555c	-1.34027	-1.86869	-0.19512	1.085783	1.187353
aPFA0555c_0	PFA0555c	-1.34027	-1.86869	-0.19512	1.085783	1.187353
aPFA0560c_0	PFA0560c	-0.17263	0.32807	0.60712	0.48772	-0.10523
intPFA0565c_2	PFA0565c	-0.45071	0.097547	0.836397	0.687937	-0.28621
intPFA0560c_2	PFA0565c	-0.17263	0.32807	0.60712	0.48772	-0.10523
aPFA0565c_0	PFA0565c	-0.45071	0.097547	0.836397	0.687937	-0.28621
aPFA0570w_1	PFA0570w	-1.15686	-0.70121	0.70843	1.12757	0.31071
aPFA0570w_0	PFA0570w	-1.15686	-0.70121	0.70843	1.12757	0.31071
intPFA0575c_2	PFA0575c	0.128057	-0.14729	0.186597	0.377737	0.083627
aPFA0575c_0	PFA0575c	0.128057	-0.14729	0.186597	0.377737	0.083627
intPFA0580c_2	PFA0580c	0.529303	0.530503	0.389493	0.060673	-0.44584
aPFA0580c_1	PFA0580c	0.529303	0.530503	0.389493	0.060673	-0.44584
aPFA0580c_0	PFA0580c	0.529303	0.530503	0.389493	0.060673	-0.44584
intPFA0585w_2	PFA0585w	2.022312	1.084622	0.229282	-0.812	-1.58939
aPFA0585w_0	PFA0585w	2.022312	1.084622	0.229282	-0.812	-1.58939
intPFA0590w_2	PFA0590w	-1.6227	-0.69519	0.937212	1.323682	0.344742
aPFA0590w_2	PFA0590w	-1.6227	-0.69519	0.937212	1.323682	0.344742
aPFA0590w_1	PFA0590w	-1.6227	-0.69519	0.937212	1.323682	0.344742
aPFA0590w_0	PFA0590w	-1.6227	-0.69519	0.937212	1.323682	0.344742
intPFA0610c_2	PFA0610c	2.706683	0.466563	-1.18164	-1.5147	-0.6969
aPFA0610c_0	PFA0610c	2.706683	0.466563	-1.18164	-1.5147	-0.6969
intPFA0615w_2	PFA0615w	-2.33481	-1.48691	1.229327	1.860007	0.323247
aPFA0615w_0	PFA0615w	-2.33481	-1.48691	1.229327	1.860007	0.323247
intPFA0620c_2	PFA0620c	-0.54835	1.29577	1.62297	0.34603	-1.40977

aPFA0620c_1	PFA0620c	-0.54835	1.29577	1.62297	0.34603	-1.40977
aPFA0620c_0	PFA0620c	-0.54835	1.29577	1.62297	0.34603	-1.40977
intPFA0625w_2	PFA0625w	-0.36211	-0.18464	-0.26955	-0.06706	0.3912
aPFA0625w_2	PFA0625w	-0.36211	-0.18464	-0.26955	-0.06706	0.3912
aPFA0625w_1	PFA0625w	-0.36211	-0.18464	-0.26955	-0.06706	0.3912
aPFA0625w_0	PFA0625w	-0.36211	-0.18464	-0.26955	-0.06706	0.3912
intPFA0630c_2	PFA0630c	-0.95245	-0.30174	0.749247	0.741587	-0.14492
aPFA0630c_1	PFA0630c	-0.95245	-0.30174	0.749247	0.741587	-0.14492
aPFA0630c_0	PFA0630c	-0.95245	-0.30174	0.749247	0.741587	-0.14492
intPFA0635c_2	PFA0635c	2.021213	1.726423	0.218163	-1.708	-2.48476
aPFA0635c_1	PFA0635c	2.021213	1.726423	0.218163	-1.708	-2.48476
aPFA0635c_0	PFA0635c	2.021213	1.726423	0.218163	-1.708	-2.48476
aPFA0640c_1	PFA0640c	0.165667	0.633187	0.352877	-0.25354	-0.66035
aPFA0640c_0	PFA0640c	0.165667	0.633187	0.352877	-0.25354	-0.66035
intPFA0650w_2	PFA0645c	1.822352	0.631142	-0.52272	-0.90321	-0.58318
intPFA0640c_2	PFA0645c	0.165667	0.633187	0.352877	-0.25354	-0.66035
aPFA0645c_0	PFA0645c					
intPFA0645c_2	PFA0650w					
intPFA0660w_2	PFA0660w	1.606333	1.637353	0.463293	-1.31563	-2.26669
aPFA0660w_1	PFA0660w	1.606333	1.637353	0.463293	-1.31563	-2.26669
aPFA0660w_0	PFA0660w	1.606333	1.637353	0.463293	-1.31563	-2.26669
intPFA0665w_2	PFA0665w	1.172272	0.206292	-0.27774	-0.56132	-0.58601
aPFA0665w_4	PFA0665w	1.172272	0.206292	-0.27774	-0.56132	-0.58601
aPFA0665w_3	PFA0665w	1.172272	0.206292	-0.27774	-0.56132	-0.58601
aPFA0665w_2	PFA0665w	1.172272	0.206292	-0.27774	-0.56132	-0.58601
aPFA0665w_1	PFA0665w	1.172272	0.206292	-0.27774	-0.56132	-0.58601
aPFA0665w_0	PFA0665w	1.172272	0.206292	-0.27774	-0.56132	-0.58601
intPFA0670c_2	PFA0670c	1.507578	1.208098	0.059318	-0.9911	-1.29253
aPFA0670c_0	PFA0670c	1.507578	1.208098	0.059318	-0.9911	-1.29253
intPFA0675w_2	PFA0675w	0.323633	0.630853	-0.02921	-0.40863	-0.24669
aPFA0675w_2	PFA0675w	0.323633	0.630853	-0.02921	-0.40863	-0.24669
aPFA0675w_1	PFA0675w	0.323633	0.630853	-0.02921	-0.40863	-0.24669
intPFA0680c_2	PFA0680c	1.087623	1.138883	0.122233	-0.69657	-0.87222
aPFA0685c_0	PFA0685c					
intPFA0685c_2	PFA0690w					
aPFA0690w_1	PFA0690w	-0.17894	0.02788	0.45818	0.40492	-0.15438
aPFA0690w_0	PFA0690w	-0.17894	0.02788	0.45818	0.40492	-0.15438
intPFA0700c_2	PFA0700c	-0.82556	0.599817	2.309867	1.157467	-1.82348
aPFA0700c_0	PFA0700c	-0.82556	0.599817	2.309867	1.157467	-1.82348
intPFA0715c_2	PFA0715c	0.390198	0.751388	0.702018	0.044388	-0.84959
aPFA0715c_0	PFA0715c	0.390198	0.751388	0.702018	0.044388	-0.84959
intPFA0720w_2	PFA0720w	0.686707	0.513467	0.628067	-0.03673	-1.09313
aPFA0720w_0	PFA0720w	0.686707	0.513467	0.628067	-0.03673	-1.09313
intPFA0725w_2	PFA0725w	-0.27351	0.145485	-0.10854	-0.14902	0.163615
aPFA0725w_0	PFA0725w	-0.27351	0.145485	-0.10854	-0.14902	0.163615
intPFA0735w_2	PFA0735w					
intPFB0056c_2	PFB0056c					

intPFB0070w_2	PFB0070w					
aPFB0070w_0	PFB0070w					
intPFB0075c_2	PFB0075c	-0.40297	0.039843	0.774313	0.512353	-0.49018
aPFB0075c_0	PFB0075c	-0.40297	0.039843	0.774313	0.512353	-0.49018
intPFB0080c_2	PFB0080c	0.316073	1.643623	1.626253	0.001413	-1.98896
aPFB0080c_1	PFB0080c	0.316073	1.643623	1.626253	0.001413	-1.98896
aPFB0080c_0	PFB0080c	0.316073	1.643623	1.626253	0.001413	-1.98896
intPFB0085c_2	PFB0085c	2.435173	2.274143	-0.19055	-2.63879	-2.90448
aPFB0085c_1	PFB0085c	2.435173	2.274143	-0.19055	-2.63879	-2.90448
aPFB0085c_0	PFB0085c	2.435173	2.274143	-0.19055	-2.63879	-2.90448
intPFB0090c_2	PFB0090c	0.976243	1.809303	1.310903	-0.53674	-2.32764
aPFB0090c_1	PFB0090c	0.976243	1.809303	1.310903	-0.53674	-2.32764
aPFB0090c_0	PFB0090c	0.976243	1.809303	1.310903	-0.53674	-2.32764
intPFB0100c_2	PFB0100c	0.23674	2.12529	1.42567	-0.32267	-1.75979
aPFB0100c_1	PFB0100c	0.23674	2.12529	1.42567	-0.32267	-1.75979
aPFB0100c_0	PFB0100c	0.23674	2.12529	1.42567	-0.32267	-1.75979
intPFB0105c_2	PFB0105c	1.516983	0.664063	-0.53762	-1.68872	-1.6104
aPFB0105c_0	PFB0105c	1.516983	0.664063	-0.53762	-1.68872	-1.6104
intPFB0106c_2	PFB0106c	2.284277	1.584607	-0.21772	-2.26894	-2.70927
aPFB0106c_0	PFB0106c	2.284277	1.584607	-0.21772	-2.26894	-2.70927
intPFB0110w_2	PFB0110w	0.908	0.28298	1.17104	0.25596	-1.87698
aPFB0110w_0	PFB0110w	0.908	0.28298	1.17104	0.25596	-1.87698
intPFB0115w_2	PFB0115w	-1.36066	-0.35674	1.012973	0.982193	-0.1681
aPFB0115w_1	PFB0115w	-1.36066	-0.35674	1.012973	0.982193	-0.1681
aPFB0115w_0	PFB0115w	-1.36066	-0.35674	1.012973	0.982193	-0.1681
intPFB0120w_2	PFB0120w	2.282853	1.788753	-0.50051	-2.61883	-2.57909
aPFB0120w_0	PFB0120w	2.282853	1.788753	-0.50051	-2.61883	-2.57909
intPFB0125c_2	PFB0125c	-0.15714	0.37022	0.13055	-0.04405	-0.00622
aPFB0125c_1	PFB0125c	-0.15714	0.37022	0.13055	-0.04405	-0.00622
aPFB0125c_0	PFB0125c	-0.15714	0.37022	0.13055	-0.04405	-0.00622
intPFB0130w_2	PFB0130w	-0.44215	-0.22171	0.131187	0.397647	0.355547
aPFB0130w_1	PFB0130w	-0.44215	-0.22171	0.131187	0.397647	0.355547
aPFB0130w_0	PFB0130w	-0.44215	-0.22171	0.131187	0.397647	0.355547
intPFB0140w_2	PFB0140w	-0.43747	-0.84063	-0.66956	0.20452	1.11971
aPFB0140w_0	PFB0140w	-0.43747	-0.84063	-0.66956	0.20452	1.11971
intPFB0145c_2	PFB0145c	0.22245	-0.65125	-1.33	-0.7245	0.84325
aPFB0145c_2	PFB0145c	0.22245	-0.65125	-1.33	-0.7245	0.84325
aPFB0145c_1	PFB0145c	0.22245	-0.65125	-1.33	-0.7245	0.84325
aPFB0145c_0	PFB0145c	0.22245	-0.65125	-1.33	-0.7245	0.84325
aPFB0150c_4	PFB0150c	0.393537	-0.00751	-1.00108	-1.26298	-0.19535
aPFB0150c_3	PFB0150c	0.393537	-0.00751	-1.00108	-1.26298	-0.19535
aPFB0150c_2	PFB0150c	0.393537	-0.00751	-1.00108	-1.26298	-0.19535
aPFB0150c_1	PFB0150c	0.393537	-0.00751	-1.00108	-1.26298	-0.19535
aPFB0150c_0	PFB0150c	0.393537	-0.00751	-1.00108	-1.26298	-0.19535
intPFB0160w_2	PFB0151c	0.02452	-0.03962	-0.05526	0.13326	0.26662
intPFB0150c_2	PFB0160w	0.393537	-0.00751	-1.00108	-1.26298	-0.19535
aPFB0160w_0	PFB0160w	0.02452	-0.03962	-0.05526	0.13326	0.26662

aPFB0161c_0	PFB0161c	-0.82119	-2.19709	-0.49433	1.354993	1.747753
intPFB0170w_2	PFB0170w	-0.3349	-0.9605	-0.59351	0.376173	1.123663
intPFB0161c_1	PFB0170w	-0.82119	-2.19709	-0.49433	1.354993	1.747753
aPFB0170w_1	PFB0170w	-0.3349	-0.9605	-0.59351	0.376173	1.123663
aPFB0170w_0	PFB0170w	-0.3349	-0.9605	-0.59351	0.376173	1.123663
intPFB0175c_2	PFB0175c	0.913157	0.946217	0.635567	-0.56473	-1.68588
aPFB0175c_1	PFB0175c	0.913157	0.946217	0.635567	-0.56473	-1.68588
aPFB0175c_0	PFB0175c	0.913157	0.946217	0.635567	-0.56473	-1.68588
aPFB0177c_0	PFB0177c	-0.95143	-0.45393	0.371907	0.778147	0.499907
aPFB0176c_0	PFB0177c	-0.82365	-0.74973	0.18795	0.84105	0.69173
intPFB0185w_2	PFB0180w	-0.68978	-0.13706	0.311377	0.486457	0.297397
intPFB0180w_2	PFB0180w	-1.02997	-0.25818	0.594103	0.820563	0.312843
intPFB0177c_2	PFB0180w	-0.95143	-0.45393	0.371907	0.778147	0.499907
aPFB0180w_1	PFB0180w	-1.02997	-0.25818	0.594103	0.820563	0.312843
aPFB0180w_0	PFB0180w	-1.02997	-0.25818	0.594103	0.820563	0.312843
aPFB0185w_1	PFB0185w	-0.68978	-0.13706	0.311377	0.486457	0.297397
aPFB0185w_0	PFB0185w	-0.68978	-0.13706	0.311377	0.486457	0.297397
intPFB0190c_2	PFB0190c	-0.76822	-0.84013	-1.50149	-0.35067	1.922717
aPFB0190c_3	PFB0190c	-0.76822	-0.84013	-1.50149	-0.35067	1.922717
aPFB0190c_2	PFB0190c	-0.76822	-0.84013	-1.50149	-0.35067	1.922717
aPFB0190c_1	PFB0190c	-0.76822	-0.84013	-1.50149	-0.35067	1.922717
aPFB0190c_0	PFB0190c	-0.76822	-0.84013	-1.50149	-0.35067	1.922717
intPFB0194w_2	PFB0194w	0.056643	-0.1932	0.078893	-0.19923	-0.59114
aPFB0194w_0	PFB0194w	0.056643	-0.1932	0.078893	-0.19923	-0.59114
aPFB0195c_1	PFB0195c	0.44892	0.4439	0.18246	-0.12346	-0.3734
aPFB0195c_0	PFB0195c	0.44892	0.4439	0.18246	-0.12346	-0.3734
intPFB0195c_2	PFB0196c	0.44892	0.4439	0.18246	-0.12346	-0.3734
aPFB0200c_1	PFB0200c	0.472597	0.325497	0.282267	0.031567	-0.41066
aPFB0200c_0	PFB0200c	0.472597	0.325497	0.282267	0.031567	-0.41066
intPFB0205c_2	PFB0205c	-0.17707	0.42537	0.52852	0.16898	-0.37287
intPFB0200c_2	PFB0205c	0.472597	0.325497	0.282267	0.031567	-0.41066
aPFB0205c_1	PFB0205c	-0.17707	0.42537	0.52852	0.16898	-0.37287
aPFB0205c_0	PFB0205c	-0.17707	0.42537	0.52852	0.16898	-0.37287
intPFB0210c_3	PFB0210c	0.147563	-0.04882	0.316133	0.232533	-0.31252
aPFB0210c_1	PFB0210c	0.147563	-0.04882	0.316133	0.232533	-0.31252
aPFB0210c_0	PFB0210c	0.147563	-0.04882	0.316133	0.232533	-0.31252
intPFB0220w_2	PFB0215c	-0.63375	-0.73825	0.042503	0.691163	0.677913
aPFB0215c_1	PFB0215c	-0.49469	-0.77604	0.15983	0.77067	0.51054
aPFB0215c_0	PFB0215c	-0.49469	-0.77604	0.15983	0.77067	0.51054
intPFB0215c_2	PFB0220w	-0.49469	-0.77604	0.15983	0.77067	0.51054
aPFB0220w_0	PFB0220w	-0.63375	-0.73825	0.042503	0.691163	0.677913
aPFB0225c_1	PFB0225c	-0.97784	-0.85829	0.387337	0.966997	0.494127
aPFB0225c_0	PFB0225c	-0.97784	-0.85829	0.387337	0.966997	0.494127
intPFB0225c_2	PFB0227c	-0.97784	-0.85829	0.387337	0.966997	0.494127
aPFB0230c_1	PFB0227c	-1.20283	-0.68484	0.687437	1.075397	0.297177
aPFB0230c_0	PFB0227c	-1.20283	-0.68484	0.687437	1.075397	0.297177
intPFB0235w_2	PFB0230c	0.161903	-0.09202	0.342523	0.248643	-0.37032

intPFB0230c_2	PFB0230c	-1.20283	-0.68484	0.687437	1.075397	0.297177
intPFB0240w_1	PFB0235w	0.60411	0.41795	0.51959	0.07591	-0.76145
aPFB0235w_1	PFB0235w	0.161903	-0.09202	0.342523	0.248643	-0.37032
aPFB0235w_0	PFB0235w	0.161903	-0.09202	0.342523	0.248643	-0.37032
aPFB0240w_1	PFB0240w	0.60411	0.41795	0.51959	0.07591	-0.76145
aPFB0240w_0	PFB0240w	0.60411	0.41795	0.51959	0.07591	-0.76145
intPFB0250w_2	PFB0245c	-0.18365	-0.61921	0.033187	0.531647	0.385047
intPFB0245c_2	PFB0245c	0.546287	0.368587	0.463317	0.095017	-0.64325
aPFB0245c_0	PFB0245c	0.546287	0.368587	0.463317	0.095017	-0.64325
aPFB0250w_1	PFB0250w	-0.18365	-0.61921	0.033187	0.531647	0.385047
aPFB0250w_0	PFB0250w	-0.18365	-0.61921	0.033187	0.531647	0.385047
intPFB0255w_3	PFB0255w-a	1.200833	0.865853	-0.23021	-0.87213	-0.70919
aPFB0256w_0	PFB0255w-a	1.652313	1.372453	-0.12391	-1.02143	-0.94079
aPFB0255w_0	PFB0255w-a	1.200833	0.865853	-0.23021	-0.87213	-0.70919
intPFB0257c_3	PFB0257c					
intPFB0260w_2	PFB0260w	-1.03621	-0.37793	0.022107	0.526227	0.842267
aPFB0260w_1	PFB0260w	-1.03621	-0.37793	0.022107	0.526227	0.842267
aPFB0260w_0	PFB0260w	-1.03621	-0.37793	0.022107	0.526227	0.842267
intPFB0270w_2	PFB0265c	0.77854	0.2203	0.2084	-0.3104	-0.9603
intPFB0265c_2	PFB0265c	0.522027	0.535547	-0.43302	-0.78192	-0.19599
aPFB0265c_2	PFB0265c	0.522027	0.535547	-0.43302	-0.78192	-0.19599
aPFB0265c_1	PFB0265c	0.522027	0.535547	-0.43302	-0.78192	-0.19599
aPFB0265c_0	PFB0265c	0.522027	0.535547	-0.43302	-0.78192	-0.19599
aPFB0270w_0	PFB0270w	0.77854	0.2203	0.2084	-0.3104	-0.9603
intPFB0275w_2	PFB0275w	1.042067	0.420177	-1.13284	-1.76532	-0.63834
aPFB0275w_1	PFB0275w	1.042067	0.420177	-1.13284	-1.76532	-0.63834
aPFB0275w_0	PFB0275w	1.042067	0.420177	-1.13284	-1.76532	-0.63834
intPFB0280w_2	PFB0279w	0.308458	0.333708	0.368948	-0.04353	-0.62204
intPFB0279w_2	PFB0279w	0.474453	0.219243	0.251503	-0.07434	-0.58458
aPFB0279w_1	PFB0279w	0.474453	0.219243	0.251503	-0.07434	-0.58458
aPFB0279w_0	PFB0279w	0.474453	0.219243	0.251503	-0.07434	-0.58458
aPFB0280w_3	PFB0280w	0.308458	0.333708	0.368948	-0.04353	-0.62204
aPFB0280w_2	PFB0280w	0.308458	0.333708	0.368948	-0.04353	-0.62204
aPFB0280w_1	PFB0280w	0.308458	0.333708	0.368948	-0.04353	-0.62204
aPFB0280w_0	PFB0280w	0.308458	0.333708	0.368948	-0.04353	-0.62204
intPFB0285c_2	PFB0285c	0.907755	0.046905	-0.13222	-0.5294	-0.82302
aPFB0285c_2	PFB0285c	0.907755	0.046905	-0.13222	-0.5294	-0.82302
aPFB0285c_1	PFB0285c	0.907755	0.046905	-0.13222	-0.5294	-0.82302
aPFB0285c_0	PFB0285c	0.907755	0.046905	-0.13222	-0.5294	-0.82302
aPFB0290c_0	PFB0290c	1.559693	0.587523	0.396943	-0.45928	-1.54486
intPFB0295w_2	PFB0295w	0.62224	-0.11121	-0.10958	-0.41042	-0.69629
intPFB0290c_2	PFB0295w	1.559693	0.587523	0.396943	-0.45928	-1.54486
aPFB0295w_1	PFB0295w	0.62224	-0.11121	-0.10958	-0.41042	-0.69629
aPFB0295w_0	PFB0295w	0.62224	-0.11121	-0.10958	-0.41042	-0.69629
intPFB0300c_2	PFB0300c	0.20972	-0.69259	-1.54432	-0.92868	0.90659
aPFB0300c_0	PFB0300c	0.20972	-0.69259	-1.54432	-0.92868	0.90659
intPFB0305c_3	PFB0305c-a	0.43011	-0.46354	-0.45043	-0.68857	-0.65946

aPFB0305c_0	PFB0305c-a	0.43011	-0.46354	-0.45043	-0.68857	-0.65946
intPFB0310c_2	PFB0310c	0.13636	-0.53028	0.10313	0.27491	-0.17568
aPFB0310c_0	PFB0310c	0.13636	-0.53028	0.10313	0.27491	-0.17568
intPFB0315w_2	PFB0315w	0.399198	-0.67501	-1.36239	-0.96833	0.462128
aPFB0315w_2	PFB0315w	0.399198	-0.67501	-1.36239	-0.96833	0.462128
aPFB0315w_1	PFB0315w	0.399198	-0.67501	-1.36239	-0.96833	0.462128
aPFB0315w_0	PFB0315w	0.399198	-0.67501	-1.36239	-0.96833	0.462128
intPFB0320c_2	PFB0320c	0.0645	-0.89052	-0.11646	0.08346	-0.27648
aPFB0320c_0	PFB0320c	0.0645	-0.89052	-0.11646	0.08346	-0.27648
intPFB0325c_2	PFB0325c					
aPFB0325c_1	PFB0325c					
aPFB0325c_0	PFB0325c					
intPFB0330c_2	PFB0330c	-0.87944	-1.2913	-1.63413	0.020967	2.459637
aPFB0330c_1	PFB0330c	-0.87944	-1.2913	-1.63413	0.020967	2.459637
aPFB0330c_0	PFB0330c	-0.87944	-1.2913	-1.63413	0.020967	2.459637
intPFB0335c_2	PFB0335c	-0.76899	-1.36709	-1.53782	0.146983	2.384753
aPFB0335c_1	PFB0335c	-0.76899	-1.36709	-1.53782	0.146983	2.384753
aPFB0335c_0	PFB0335c	-0.76899	-1.36709	-1.53782	0.146983	2.384753
intPFB0340c_2	PFB0340c	-1.76643	-2.00389	-2.24323	0.160393	3.606053
aPFB0340c_1	PFB0340c	-1.76643	-2.00389	-2.24323	0.160393	3.606053
aPFB0340c_0	PFB0340c	-1.76643	-2.00389	-2.24323	0.160393	3.606053
intPFB0345c_2	PFB0345c	-1.00549	-1.84204	-1.74292	0.356257	2.836877
aPFB0345c_1	PFB0345c	-1.00549	-1.84204	-1.74292	0.356257	2.836877
aPFB0345c_0	PFB0345c	-1.00549	-1.84204	-1.74292	0.356257	2.836877
aPFB0350c_1	PFB0350c	-0.5689	-0.58351	-1.76824	-0.74626	1.92301
aPFB0350c_0	PFB0350c	-0.5689	-0.58351	-1.76824	-0.74626	1.92301
intPFB0355c_2	PFB0355c	-0.30784	-0.56978	-0.88443	-0.06373	1.248617
intPFB0350c_2	PFB0355c	-0.5689	-0.58351	-1.76824	-0.74626	1.92301
aPFB0355c_1	PFB0355c	-0.30784	-0.56978	-0.88443	-0.06373	1.248617
aPFB0355c_0	PFB0355c	-0.30784	-0.56978	-0.88443	-0.06373	1.248617
intPFB0360c_2	PFB0360c	-0.4263	-0.67554	-1.53569	-0.51165	1.739703
aPFB0360c_1	PFB0360c	-0.4263	-0.67554	-1.53569	-0.51165	1.739703
aPFB0360c_0	PFB0360c	-0.4263	-0.67554	-1.53569	-0.51165	1.739703
intPFB0365w_2	PFB0365w	-1.08414	-1.33497	-0.89407	0.549402	1.934632
aPFB0365w_2	PFB0365w	-1.08414	-1.33497	-0.89407	0.549402	1.934632
aPFB0365w_1	PFB0365w	-1.08414	-1.33497	-0.89407	0.549402	1.934632
aPFB0365w_0	PFB0365w	-1.08414	-1.33497	-0.89407	0.549402	1.934632
intPFB0375w_2	PFB0370c	1.334893	0.981033	0.430933	-0.59327	-1.47087
aPFB0370c_0	PFB0370c	0.80134	0.5723	0.57464	-0.20614	-1.2483
intPFB0370c_2	PFB0375w	0.80134	0.5723	0.57464	-0.20614	-1.2483
aPFB0375w_2	PFB0375w	1.334893	0.981033	0.430933	-0.59327	-1.47087
aPFB0375w_1	PFB0375w	1.334893	0.981033	0.430933	-0.59327	-1.47087
aPFB0375w_0	PFB0375w	1.334893	0.981033	0.430933	-0.59327	-1.47087
intPFB0380c_2	PFB0380c	-0.10919	0.180102	-0.4463	-0.28648	0.544952
aPFB0380c_2	PFB0380c	-0.10919	0.180102	-0.4463	-0.28648	0.544952
aPFB0380c_1	PFB0380c	-0.10919	0.180102	-0.4463	-0.28648	0.544952
aPFB0380c_0	PFB0380c	-0.10919	0.180102	-0.4463	-0.28648	0.544952

intPFB0385w_2	PFB0385w	-1.13134	-0.87427	0.006047	0.734287	0.884607
aPFB0385w_0	PFB0385w	-1.13134	-0.87427	0.006047	0.734287	0.884607
intPFB0390w_2	PFB0390w	-0.88515	-1.81728	-0.21245	1.129613	1.167443
aPFB0390w_1	PFB0390w	-0.88515	-1.81728	-0.21245	1.129613	1.167443
aPFB0390w_0	PFB0390w	-0.88515	-1.81728	-0.21245	1.129613	1.167443
aPFB0391c_0	PFB0391c-a	-0.07267	0.268353	0.775293	0.500373	-0.46769
intPFB0395w_2	PFB0391c-b	-0.11417	0.21485	0.38629	0.33871	-0.01885
intPFB0391c_2	PFB0395w	-0.07267	0.268353	0.775293	0.500373	-0.46769
aPFB0395w_0	PFB0395w	-0.11417	0.21485	0.38629	0.33871	-0.01885
intPFB0400w_2	PFB0400w	-0.01422	0.077745	-0.29666	-0.13902	0.391925
aPFB0400w_3	PFB0400w	-0.01422	0.077745	-0.29666	-0.13902	0.391925
aPFB0400w_2	PFB0400w	-0.01422	0.077745	-0.29666	-0.13902	0.391925
aPFB0400w_1	PFB0400w	-0.01422	0.077745	-0.29666	-0.13902	0.391925
aPFB0400w_0	PFB0400w	-0.01422	0.077745	-0.29666	-0.13902	0.391925
intPFB0405w_2	PFB0405w	0.045963	0.532223	0.132953	-0.23284	-0.25351
aPFB0405w_4	PFB0405w	0.045963	0.532223	0.132953	-0.23284	-0.25351
aPFB0405w_3	PFB0405w	0.045963	0.532223	0.132953	-0.23284	-0.25351
aPFB0405w_2	PFB0405w	0.045963	0.532223	0.132953	-0.23284	-0.25351
aPFB0405w_1	PFB0405w	0.045963	0.532223	0.132953	-0.23284	-0.25351
aPFB0405w_0	PFB0405w	0.045963	0.532223	0.132953	-0.23284	-0.25351
intPFB0410c_2	PFB0410c	0.612427	0.402857	-0.15421	-0.35645	-0.18752
aPFB0410c_1	PFB0410c	0.612427	0.402857	-0.15421	-0.35645	-0.18752
aPFB0410c_0	PFB0410c	0.612427	0.402857	-0.15421	-0.35645	-0.18752
intPFB0420w_2	PFB0415c	-0.40412	-0.32536	0.572727	0.764607	0.014697
aPFB0415c_0	PFB0415c	0.544397	0.669037	0.460427	-0.03609	-0.6337
intPFB0415c_2	PFB0420w	0.544397	0.669037	0.460427	-0.03609	-0.6337
aPFB0420w_0	PFB0420w	-0.40412	-0.32536	0.572727	0.764607	0.014697
aPFB0425c_1	PFB0423c	1.329103	0.604993	-0.73474	-1.1191	-0.37133
intPFB0425c_2	PFB0425c	1.329103	0.604993	-0.73474	-1.1191	-0.37133
aPFB0425c_0	PFB0425c	1.329103	0.604993	-0.73474	-1.1191	-0.37133
intPFB0435c_2	PFB0435c	-0.04895	-0.25776	-1.66649	-0.88585	1.570923
aPFB0435c_1	PFB0435c	-0.04895	-0.25776	-1.66649	-0.88585	1.570923
aPFB0435c_0	PFB0435c	-0.04895	-0.25776	-1.66649	-0.88585	1.570923
intPFB0440c_2	PFB0440c	1.276497	1.212537	0.149417	-1.07308	-1.5007
aPFB0440c_1	PFB0440c	1.276497	1.212537	0.149417	-1.07308	-1.5007
aPFB0440c_0	PFB0440c	1.276497	1.212537	0.149417	-1.07308	-1.5007
intPFB0445c_3	PFB0445c	-0.10418	0.242937	0.568137	0.370197	-0.2896
aPFB0445c_1	PFB0445c	-0.10418	0.242937	0.568137	0.370197	-0.2896
aPFB0445c_0	PFB0445c	-0.10418	0.242937	0.568137	0.370197	-0.2896
intPFB0450w_2	PFB0450w	-0.61992	0.095603	0.175293	0.317373	0.425063
aPFB0450w_0	PFB0450w	-0.61992	0.095603	0.175293	0.317373	0.425063
intPFB0455w_1	PFB0455w-a	0.576563	0.644703	0.466843	-0.06018	-0.70854
aPFB0455w_0	PFB0455w-a	0.576563	0.644703	0.466843	-0.06018	-0.70854
intPFB0460c_2	PFB0460c	-0.83099	-0.97271	-0.55225	0.48359	1.36637
aPFB0460c_3	PFB0460c	-0.83099	-0.97271	-0.55225	0.48359	1.36637
aPFB0460c_2	PFB0460c	-0.83099	-0.97271	-0.55225	0.48359	1.36637
aPFB0460c_1	PFB0460c	-0.83099	-0.97271	-0.55225	0.48359	1.36637

aPFB0460c_0	PFB0460c	-0.83099	-0.97271	-0.55225	0.48359	1.36637
intPFB0467w_2	PFB0465c					
aPFB0465c_1	PFB0465c	-0.88787	-0.80116	0.18133	0.88317	0.75416
aPFB0465c_0	PFB0465c	-0.88787	-0.80116	0.18133	0.88317	0.75416
intPFB0465c_2	PFB0467w	-0.88787	-0.80116	0.18133	0.88317	0.75416
intPFB0470w_2	PFB0470w	0.03339	0.35957	0.72387	0.13263	-0.89957
aPFB0470w_1	PFB0470w	0.03339	0.35957	0.72387	0.13263	-0.89957
aPFB0470w_0	PFB0470w	0.03339	0.35957	0.72387	0.13263	-0.89957
intPFB0475c_2	PFB0475c	1.062167	-0.43035	-1.64479	-1.80087	-0.32681
aPFB0475c_1	PFB0475c	1.062167	-0.43035	-1.64479	-1.80087	-0.32681
aPFB0475c_0	PFB0475c	1.062167	-0.43035	-1.64479	-1.80087	-0.32681
intPFB0480w_2	PFB0480w	1.18699	-0.05896	-0.71458	-1.16542	-0.87304
aPFB0480w_0	PFB0480w	1.18699	-0.05896	-0.71458	-1.16542	-0.87304
intPFB0485c_2	PFB0485c	1.685383	0.707563	-0.02563	-1.22221	-1.8764
aPFB0485c_1	PFB0485c	1.685383	0.707563	-0.02563	-1.22221	-1.8764
aPFB0485c_0	PFB0485c	1.685383	0.707563	-0.02563	-1.22221	-1.8764
intPFB0490c_2	PFB0490c	0.01921	0.06897	0.31306	0.23494	-0.18197
aPFB0490c_0	PFB0490c	0.01921	0.06897	0.31306	0.23494	-0.18197
intPFB0495w_2	PFB0495w	0.117177	0.629127	0.904247	0.153087	-1.04779
aPFB0495w_1	PFB0495w	0.117177	0.629127	0.904247	0.153087	-1.04779
aPFB0495w_0	PFB0495w	0.117177	0.629127	0.904247	0.153087	-1.04779
intPFB0500c_2	PFB0500c	-1.4127	-0.95502	0.155268	0.968548	1.002388
aPFB0500c_0	PFB0500c	-1.4127	-0.95502	0.155268	0.968548	1.002388
aPFB0501c_0	PFB0501c	-0.67127	-0.87668	0.043335	0.704325	0.616345
intPFB0501c_2	PFB0505c	-0.67127	-0.87668	0.043335	0.704325	0.616345
aPFB0505c_1	PFB0505c	-0.24546	-0.22374	0.047123	0.280063	0.266363
aPFB0505c_0	PFB0505c	-0.24546	-0.22374	0.047123	0.280063	0.266363
intPFB0510w_2	PFB0510w	-0.69456	-0.67769	-1.02886	-0.0783	1.548527
intPFB0505c_2	PFB0510w	-0.24546	-0.22374	0.047123	0.280063	0.266363
aPFB0510w_1	PFB0510w	-0.69456	-0.67769	-1.02886	-0.0783	1.548527
aPFB0510w_0	PFB0510w	-0.69456	-0.67769	-1.02886	-0.0783	1.548527
intPFB0515w_2	PFB0515w	-0.65709	-1.08154	-1.17991	-0.03709	1.60554
aPFB0515w_0	PFB0515w	-0.65709	-1.08154	-1.17991	-0.03709	1.60554
intPFB0520w_2	PFB0520w	-0.96184	-0.73482	0.223873	0.719793	0.499983
aPFB0520w_1	PFB0520w	-0.96184	-0.73482	0.223873	0.719793	0.499983
aPFB0520w_0	PFB0520w	-0.96184	-0.73482	0.223873	0.719793	0.499983
intPFB0525w_3	PFB0525w	-0.6069	0.210037	0.654687	0.606147	0.051297
aPFB0525w_1	PFB0525w	-0.6069	0.210037	0.654687	0.606147	0.051297
aPFB0525w_0	PFB0525w	-0.6069	0.210037	0.654687	0.606147	0.051297
intPFB0535w_2	PFB0530c	-1.03004	-1.1418	-0.4394	0.6794	1.4068
aPFB0530c_1	PFB0530c	-0.83122	-1.32438	-0.35399	0.83449	1.29488
aPFB0530c_0	PFB0530c	-0.83122	-1.32438	-0.35399	0.83449	1.29488
intPFB0530c_2	PFB0535w	-0.83122	-1.32438	-0.35399	0.83449	1.29488
aPFB0535w_0	PFB0535w	-1.03004	-1.1418	-0.4394	0.6794	1.4068
intPFB0540w_3	PFB0540w	-0.44972	-0.14542	-0.33699	0.08199	0.79376
aPFB0540w_2	PFB0540w	-0.44972	-0.14542	-0.33699	0.08199	0.79376
aPFB0540w_1	PFB0540w	-0.44972	-0.14542	-0.33699	0.08199	0.79376

aPFB0540w_0	PFB0540w	-0.44972	-0.14542	-0.33699	0.08199	0.79376
intPFB0545c_3	PFB0545c	0.00065	-0.31723	0.75347	0.66953	-0.58877
aPFB0545c_0	PFB0545c	0.00065	-0.31723	0.75347	0.66953	-0.58877
intPFB0550w_2	PFB0550w	-0.04154	0.107663	0.769683	0.470983	-0.619
aPFB0550w_1	PFB0550w	-0.04154	0.107663	0.769683	0.470983	-0.619
aPFB0550w_0	PFB0550w	-0.04154	0.107663	0.769683	0.470983	-0.619
intPFB0555c_2	PFB0555c	0.248692	0.185482	0.058212	0.002282	-0.06256
aPFB0555c_6	PFB0555c	0.248692	0.185482	0.058212	0.002282	-0.06256
aPFB0555c_5	PFB0555c	0.248692	0.185482	0.058212	0.002282	-0.06256
aPFB0555c_4	PFB0555c	0.248692	0.185482	0.058212	0.002282	-0.06256
aPFB0555c_3	PFB0555c	0.248692	0.185482	0.058212	0.002282	-0.06256
aPFB0555c_2	PFB0555c	0.248692	0.185482	0.058212	0.002282	-0.06256
aPFB0555c_1	PFB0555c	0.248692	0.185482	0.058212	0.002282	-0.06256
aPFB0555c_0	PFB0555c	0.248692	0.185482	0.058212	0.002282	-0.06256
intPFB0560w_2	PFB0560w	0.010845	-0.01273	0.019445	0.111165	0.119005
aPFB0560w_5	PFB0560w	0.010845	-0.01273	0.019445	0.111165	0.119005
aPFB0560w_4	PFB0560w	0.010845	-0.01273	0.019445	0.111165	0.119005
aPFB0560w_3	PFB0560w	0.010845	-0.01273	0.019445	0.111165	0.119005
aPFB0560w_2	PFB0560w	0.010845	-0.01273	0.019445	0.111165	0.119005
aPFB0560w_1	PFB0560w	0.010845	-0.01273	0.019445	0.111165	0.119005
aPFB0560w_0	PFB0560w	0.010845	-0.01273	0.019445	0.111165	0.119005
aPFB0570w_0	PFB0570w	1.330863	-0.36931	-1.44939	-1.76695	-0.68303
intPFB0575c_2	PFB0575c	0.337967	0.485187	0.472627	0.068207	-0.55335
aPFB0575c_1	PFB0575c	0.337967	0.485187	0.472627	0.068207	-0.55335
aPFB0575c_0	PFB0575c	0.337967	0.485187	0.472627	0.068207	-0.55335
intPFB0580w_2	PFB0580w	0.10348	0.20758	0.00509	-0.02409	0.05842
aPFB0580w_1	PFB0580w	0.10348	0.20758	0.00509	-0.02409	0.05842
aPFB0580w_0	PFB0580w	0.10348	0.20758	0.00509	-0.02409	0.05842
intPFB0585w_2	PFB0585w	-0.92555	-1.46671	-0.68132	0.505157	1.337547
aPFB0585w_1	PFB0585w	-0.92555	-1.46671	-0.68132	0.505157	1.337547
aPFB0585w_0	PFB0585w	-0.92555	-1.46671	-0.68132	0.505157	1.337547
intPFB0590w_2	PFB0590w	-0.90053	-1.26934	0.07218	0.95582	0.75534
aPFB0590w_0	PFB0590w	-0.90053	-1.26934	0.07218	0.95582	0.75534
intPFB0595w_2	PFB0595w	-0.43276	-0.18521	0.92942	0.94158	-0.27079
aPFB0595w_0	PFB0595w	-0.43276	-0.18521	0.92942	0.94158	-0.27079
aPFB0600c_0	PFB0600c	-0.80344	-0.73879	0.478097	1.006237	0.408127
intPFB0605w_2	PFB0601c	-0.90998	-0.5091	0.6772	0.9098	0.0871
intPFB0600c_2	PFB0601c	-0.80344	-0.73879	0.478097	1.006237	0.408127
aPFB0605w_0	PFB0605w	-0.90998	-0.5091	0.6772	0.9098	0.0871
intPFB0610c_2	PFB0610c	0.35706	0.4502	0.4156	0.0144	-0.5592
aPFB0610c_1	PFB0610c	0.35706	0.4502	0.4156	0.0144	-0.5592
aPFB0610c_0	PFB0610c	0.35706	0.4502	0.4156	0.0144	-0.5592
intPFB0615c_2	PFB0615c	-0.35116	-0.50217	0.186517	0.540707	0.251397
aPFB0615c_3	PFB0615c	-0.35116	-0.50217	0.186517	0.540707	0.251397
aPFB0615c_2	PFB0615c	-0.35116	-0.50217	0.186517	0.540707	0.251397
aPFB0615c_1	PFB0615c	-0.35116	-0.50217	0.186517	0.540707	0.251397
aPFB0615c_0	PFB0615c	-0.35116	-0.50217	0.186517	0.540707	0.251397

intPFB0625w_2	PFB0620w	-0.01361	0.791557	-0.00736	-0.3453	0.027777
intPFB0620w_3	PFB0620w	-0.65061	0.513053	1.567893	0.848773	-1.04239
aPFB0620w_0	PFB0620w	-0.65061	0.513053	1.567893	0.848773	-1.04239
aPFB0625w_1	PFB0625w	-0.01361	0.791557	-0.00736	-0.3453	0.027777
aPFB0625w_0	PFB0625w	-0.01361	0.791557	-0.00736	-0.3453	0.027777
aPFB0630c_1	PFB0630c	0.552843	0.537783	0.794433	-0.04727	-1.35312
aPFB0630c_0	PFB0630c	0.552843	0.537783	0.794433	-0.04727	-1.35312
intPFB0635w_2	PFB0635w	0.294163	-0.00131	0.996103	0.656063	-0.94103
intPFB0630c_2	PFB0635w	0.552843	0.537783	0.794433	-0.04727	-1.35312
aPFB0635w_1	PFB0635w	0.294163	-0.00131	0.996103	0.656063	-0.94103
aPFB0635w_0	PFB0635w	0.294163	-0.00131	0.996103	0.656063	-0.94103
intPFB0640c_1	PFB0640c	0.375307	0.357477	-0.30544	-0.43689	0.017857
aPFB0640c_2	PFB0640c	0.375307	0.357477	-0.30544	-0.43689	0.017857
aPFB0640c_1	PFB0640c	0.375307	0.357477	-0.30544	-0.43689	0.017857
aPFB0640c_0	PFB0640c	0.375307	0.357477	-0.30544	-0.43689	0.017857
intPFB0645c_2	PFB0645c	-0.44266	-0.8607	0.092917	0.746417	0.523037
aPFB0645c_0	PFB0645c	-0.44266	-0.8607	0.092917	0.746417	0.523037
intPFB0650w_3	PFB0650w	1.327158	0.474208	-0.70154	-1.24679	-0.71029
aPFB0650w_3	PFB0650w	1.327158	0.474208	-0.70154	-1.24679	-0.71029
aPFB0650w_2	PFB0650w	1.327158	0.474208	-0.70154	-1.24679	-0.71029
aPFB0650w_1	PFB0650w	1.327158	0.474208	-0.70154	-1.24679	-0.71029
aPFB0650w_0	PFB0650w	1.327158	0.474208	-0.70154	-1.24679	-0.71029
intPFB0660w_2	PFB0655c	1.069567	0.622147	0.512717	-0.17738	-1.14781
aPFB0655c_1	PFB0655c	0.99676	0.73618	0.60314	-0.37864	-1.51518
aPFB0655c_0	PFB0655c	0.99676	0.73618	0.60314	-0.37864	-1.51518
intPFB0655c_2	PFB0660w	0.99676	0.73618	0.60314	-0.37864	-1.51518
aPFB0660w_0	PFB0660w	1.069567	0.622147	0.512717	-0.17738	-1.14781
intPFB0665w_2	PFB0665w	1.754737	0.877997	-1.98916	-2.93006	-0.74788
aPFB0665w_2	PFB0665w	1.754737	0.877997	-1.98916	-2.93006	-0.74788
aPFB0665w_1	PFB0665w	1.754737	0.877997	-1.98916	-2.93006	-0.74788
aPFB0665w_0	PFB0665w	1.754737	0.877997	-1.98916	-2.93006	-0.74788
intPFB0670c_2	PFB0670c	1.414963	0.804203	-1.17145	-1.92699	-0.67834
aPFB0670c_0	PFB0670c	1.414963	0.804203	-1.17145	-1.92699	-0.67834
intPFB0675w_2	PFB0675w	0.650338	0.660318	0.266768	-0.72282	-1.34988
aPFB0675w_2	PFB0675w	0.650338	0.660318	0.266768	-0.72282	-1.34988
aPFB0675w_1	PFB0675w	0.650338	0.660318	0.266768	-0.72282	-1.34988
aPFB0675w_0	PFB0675w	0.650338	0.660318	0.266768	-0.72282	-1.34988
intPFB0680w_2	PFB0680w	-0.10445	-0.40924	-2.11075	-1.44441	1.465077
aPFB0680w_1	PFB0680w	-0.10445	-0.40924	-2.11075	-1.44441	1.465077
aPFB0680w_0	PFB0680w	-0.10445	-0.40924	-2.11075	-1.44441	1.465077
intPFB0685c_2	PFB0685c	-0.63437	-0.31769	-0.05938	0.345543	0.626853
aPFB0685c_1	PFB0685c	-0.63437	-0.31769	-0.05938	0.345543	0.626853
aPFB0685c_0	PFB0685c	-0.63437	-0.31769	-0.05938	0.345543	0.626853
intPFB0687c_2	PFB0687c	-0.24302	-0.27248	-0.03277	0.173473	0.219623
aPFB0687c_0	PFB0687c	-0.24302	-0.27248	-0.03277	0.173473	0.219623
intPFB0690w_2	PFB0690w	0.379897	0.153477	-0.11572	-0.26296	-0.2066
aPFB0690w_0	PFB0690w	0.379897	0.153477	-0.11572	-0.26296	-0.2066

intPFB0695c_2	PFB0695c	2.01793	1.81483	-0.0014	-1.9371	-2.36583
aPFB0695c_1	PFB0695c	2.01793	1.81483	-0.0014	-1.9371	-2.36583
aPFB0695c_0	PFB0695c	2.01793	1.81483	-0.0014	-1.9371	-2.36583
intPFB0700c_2	PFB0700c	0.380437	0.105307	0.232387	-0.13255	-0.68147
aPFB0700c_1	PFB0700c	0.380437	0.105307	0.232387	-0.13255	-0.68147
aPFB0700c_0	PFB0700c	0.380437	0.105307	0.232387	-0.13255	-0.68147
intPFB0705w_3	PFB0705w	0.46939	0.47904	0.19693	-0.34193	-0.71104
aPFB0705w_1	PFB0705w	0.46939	0.47904	0.19693	-0.34193	-0.71104
aPFB0705w_0	PFB0705w	0.46939	0.47904	0.19693	-0.34193	-0.71104
intPFB0710c_3	PFB0710c	0.493043	0.548273	0.303453	-0.07029	-0.46061
aPFB0710c_1	PFB0710c	0.493043	0.548273	0.303453	-0.07029	-0.46061
intPFB0715w_2	PFB0715w	0.114417	0.402577	0.698347	0.266317	-0.65057
aPFB0715w_2	PFB0715w	0.114417	0.402577	0.698347	0.266317	-0.65057
aPFB0715w_1	PFB0715w	0.114417	0.402577	0.698347	0.266317	-0.65057
aPFB0715w_0	PFB0715w	0.114417	0.402577	0.698347	0.266317	-0.65057
intPFB0720c_2	PFB0720c	-0.93354	-0.8313	0.026103	0.794563	0.900963
aPFB0720c_1	PFB0720c	-0.93354	-0.8313	0.026103	0.794563	0.900963
aPFB0720c_0	PFB0720c	-0.93354	-0.8313	0.026103	0.794563	0.900963
intPFB0725c_2	PFB0725c	-0.19006	-1.08268	-0.98163	0.14763	1.36268
aPFB0725c_0	PFB0725c	-0.19006	-1.08268	-0.98163	0.14763	1.36268
intPFB0730w_2	PFB0730w	0.30886	-0.75311	-0.84387	-0.11363	0.77161
aPFB0730w_2	PFB0730w	0.30886	-0.75311	-0.84387	-0.11363	0.77161
aPFB0730w_1	PFB0730w	0.30886	-0.75311	-0.84387	-0.11363	0.77161
aPFB0730w_0	PFB0730w	0.30886	-0.75311	-0.84387	-0.11363	0.77161
aPFB0735c_2	PFB0732c	0.364383	0.107233	0.321443	0.282003	-0.21629
aPFB0735c_1	PFB0732c	0.364383	0.107233	0.321443	0.282003	-0.21629
intPFB0745w_2	PFB0735c					
aPFB0735c_0	PFB0735c	0.364383	0.107233	0.321443	0.282003	-0.21629
intPFB0750w_2	PFB0745w	-0.87407	-0.95415	-0.35594	0.609777	1.222487
intPFB0735c_2	PFB0745w	0.364383	0.107233	0.321443	0.282003	-0.21629
aPFB0745w_1	PFB0745w					
aPFB0745w_0	PFB0745w					
intPFB0755w_2	PFB0750w	-0.54644	-0.35981	-0.38655	0.081992	0.773922
aPFB0750w_1	PFB0750w	-0.87407	-0.95415	-0.35594	0.609777	1.222487
aPFB0750w_0	PFB0750w	-0.87407	-0.95415	-0.35594	0.609777	1.222487
aPFB0755w_2	PFB0755w	-0.54644	-0.35981	-0.38655	0.081992	0.773922
aPFB0755w_1	PFB0755w	-0.54644	-0.35981	-0.38655	0.081992	0.773922
aPFB0755w_0	PFB0755w	-0.54644	-0.35981	-0.38655	0.081992	0.773922
intPFB0760w_3	PFB0760w	0.33841	0.56595	-0.40614	-0.79836	-0.18845
aPFB0760w_1	PFB0760w	0.33841	0.56595	-0.40614	-0.79836	-0.18845
aPFB0760w_0	PFB0760w	0.33841	0.56595	-0.40614	-0.79836	-0.18845
intPFB0765w_2	PFB0765w	0.78173	-0.47098	-1.24638	-1.1244	0.02387
aPFB0765w_2	PFB0765w	0.78173	-0.47098	-1.24638	-1.1244	0.02387
aPFB0765w_1	PFB0765w	0.78173	-0.47098	-1.24638	-1.1244	0.02387
aPFB0765w_0	PFB0765w	0.78173	-0.47098	-1.24638	-1.1244	0.02387
intPFB0775w_2	PFB0770c	-0.98252	-0.29044	0.079133	0.306533	0.448603
intPFB0770c_2	PFB0770c	-0.17074	-0.59579	-1.60317	-0.59349	1.708627

aPFB0770c_1	PFB0770c	-0.17074	-0.59579	-1.60317	-0.59349	1.708627
aPFB0770c_0	PFB0770c	-0.17074	-0.59579	-1.60317	-0.59349	1.708627
aPFB0775w_1	PFB0775w	-0.98252	-0.29044	0.079133	0.306533	0.448603
aPFB0775w_0	PFB0775w	-0.98252	-0.29044	0.079133	0.306533	0.448603
intPFB0795w_2	PFB0790c	-0.92923	-1.18433	-0.29734	0.81334	1.28533
intPFB0790c_2	PFB0790c	-0.58455	-0.88876	-0.51898	0.36498	1.10126
aPFB0790c_1	PFB0790c	-0.58455	-0.88876	-0.51898	0.36498	1.10126
aPFB0790c_0	PFB0790c	-0.58455	-0.88876	-0.51898	0.36498	1.10126
aPFB0795w_1	PFB0795w	-0.92923	-1.18433	-0.29734	0.81334	1.28533
aPFB0795w_0	PFB0795w	-0.92923	-1.18433	-0.29734	0.81334	1.28533
aPFB0800c_2	PFB0800c	-0.26977	-0.28346	0.190337	0.275277	-0.03509
aPFB0800c_1	PFB0800c	-0.26977	-0.28346	0.190337	0.275277	-0.03509
aPFB0800c_0	PFB0800c	-0.26977	-0.28346	0.190337	0.275277	-0.03509
intPFB0810w_2	PFB0805c	-0.44936	0.053763	0.170463	0.239203	0.231903
intPFB0800c_2	PFB0805c	-0.26977	-0.28346	0.190337	0.275277	-0.03509
aPFB0805c_0	PFB0805c	0.66399	0.72604	-0.27308	-0.81892	-0.46504
intPFB0805c_2	PFB0810w	0.66399	0.72604	-0.27308	-0.81892	-0.46504
aPFB0810w_0	PFB0810w	-0.44936	0.053763	0.170463	0.239203	0.231903
intPFB0815w_3	PFB0815w	0.099693	-0.77298	-1.71531	-1.07203	0.988143
aPFB0815w_1	PFB0815w	0.099693	-0.77298	-1.71531	-1.07203	0.988143
aPFB0815w_0	PFB0815w	0.099693	-0.77298	-1.71531	-1.07203	0.988143
aPFB0814c_0	PFB0815w	0.008828	-0.06631	-0.24633	-0.08497	0.277008
intPFB0820c_2	PFB0820c	-0.28424	0.248185	0.240965	0.109365	-0.01752
aPFB0820c_2	PFB0820c	-0.28424	0.248185	0.240965	0.109365	-0.01752
aPFB0820c_1	PFB0820c	-0.28424	0.248185	0.240965	0.109365	-0.01752
aPFB0820c_0	PFB0820c	-0.28424	0.248185	0.240965	0.109365	-0.01752
intPFB0826c_2	PFB0826c	0.68229	1.04655	1.0564	-0.0294	-1.51055
aPFB0826c_0	PFB0826c	0.68229	1.04655	1.0564	-0.0294	-1.51055
intPFB0830w_2	PFB0830w	-0.03733	0.178623	0.768743	0.595923	-0.38396
aPFB0830w_0	PFB0830w	-0.03733	0.178623	0.768743	0.595923	-0.38396
aPFB0835c_1	PFB0835c	-0.87903	-1.10483	-0.24559	0.82309	1.24133
aPFB0835c_0	PFB0835c	-0.87903	-1.10483	-0.24559	0.82309	1.24133
intPFB0840w_2	PFB0840w	-0.3873	-1.05347	-0.07605	0.782387	0.739807
intPFB0835c_2	PFB0840w	-0.87903	-1.10483	-0.24559	0.82309	1.24133
aPFB0840w_0	PFB0840w	-0.3873	-1.05347	-0.07605	0.782387	0.739807
intPFB0845w_2	PFB0845w	-0.37666	-0.81468	-1.16912	-0.19854	1.428017
aPFB0845w_0	PFB0845w	-0.37666	-0.81468	-1.16912	-0.19854	1.428017
aPFB0855c_1	PFB0850c	-0.06505	-0.37278	0.199043	0.432123	0.056443
aPFB0855c_0	PFB0855c	-0.06505	-0.37278	0.199043	0.432123	0.056443
intPFB0855c_2	PFB0860c	-0.06505	-0.37278	0.199043	0.432123	0.056443
aPFB0860c_1	PFB0860c	0.522117	0.680907	0.714437	-0.1421	-1.24307
aPFB0860c_0	PFB0860c	0.522117	0.680907	0.714437	-0.1421	-1.24307
intPFB0865w_2	PFB0865w	0.279525	-0.04087	0.532605	0.306645	-0.63689
intPFB0860c_2	PFB0865w	0.522117	0.680907	0.714437	-0.1421	-1.24307
aPFB0865w_0	PFB0865w	0.279525	-0.04087	0.532605	0.306645	-0.63689
intPFB0870w_2	PFB0870w	-0.55059	-0.04154	0.715948	0.431468	-0.50954
aPFB0870w_3	PFB0870w	-0.55059	-0.04154	0.715948	0.431468	-0.50954

aPFB0870w_2	PFB0870w	-0.55059	-0.04154	0.715948	0.431468	-0.50954
aPFB0870w_1	PFB0870w	-0.55059	-0.04154	0.715948	0.431468	-0.50954
aPFB0870w_0	PFB0870w	-0.55059	-0.04154	0.715948	0.431468	-0.50954
intPFB0875c_2	PFB0875c	0.622817	0.407927	0.158157	-0.33732	-0.73159
aPFB0875c_1	PFB0875c	0.622817	0.407927	0.158157	-0.33732	-0.73159
aPFB0875c_0	PFB0875c	0.622817	0.407927	0.158157	-0.33732	-0.73159
intPFB0877c_2	PFB0877c					
intPFB0880w_2	PFB0880w	0.284387	0.024077	0.630597	0.369737	-0.68024
aPFB0880w_1	PFB0880w	0.284387	0.024077	0.630597	0.369737	-0.68024
aPFB0880w_0	PFB0880w	0.284387	0.024077	0.630597	0.369737	-0.68024
intPFB0885w_2	PFB0885w	-0.3718	-0.04001	0.64252	0.67648	-0.06599
aPFB0885w_0	PFB0885w	-0.3718	-0.04001	0.64252	0.67648	-0.06599
intPFB0888w_1	PFB0886c	-0.88698	-0.54914	-0.04073	0.594393	0.895803
aPFB0888w_0	PFB0888w-a	-0.88698	-0.54914	-0.04073	0.594393	0.895803
intPFB0886c_2	PFB0888w-b	-0.0489	0.440463	0.322573	-0.00091	-0.2828
intPFB0890c_2	PFB0890c	-0.83685	-1.30073	-0.06203	0.946367	0.945067
aPFB0890c_0	PFB0890c	-0.83685	-1.30073	-0.06203	0.946367	0.945067
intPFB0895c_2	PFB0895c	-0.73651	-1.20242	-0.31166	0.781997	1.188257
aPFB0895c_1	PFB0895c	-0.73651	-1.20242	-0.31166	0.781997	1.188257
aPFB0895c_0	PFB0895c	-0.73651	-1.20242	-0.31166	0.781997	1.188257
intPFB0900c_2	PFB0900c	2.224667	1.048687	-0.57887	-2.00279	-1.98535
aPFB0900c_0	PFB0900c	2.224667	1.048687	-0.57887	-2.00279	-1.98535
intPFB0905c_2	PFB0905c	0.92462	0.06092	-0.94835	-1.11465	-0.20092
aPFB0905c_0	PFB0905c	0.92462	0.06092	-0.94835	-1.11465	-0.20092
intPFB0910w_2	PFB0910w	2.139488	1.400248	-0.08434	-1.78977	-2.30969
aPFB0910w_0	PFB0910w	2.139488	1.400248	-0.08434	-1.78977	-2.30969
intPFB0915w_2	PFB0915w	1.602502	0.291482	-1.1748	-1.55376	-0.5167
aPFB0915w_2	PFB0915w	1.602502	0.291482	-1.1748	-1.55376	-0.5167
aPFB0915w_1	PFB0915w	1.602502	0.291482	-1.1748	-1.55376	-0.5167
aPFB0915w_0	PFB0915w	1.602502	0.291482	-1.1748	-1.55376	-0.5167
intPFB0920w_2	PFB0920w	1.154647	1.216247	0.385007	-1.05367	-1.86041
aPFB0920w_1	PFB0920w	1.154647	1.216247	0.385007	-1.05367	-1.86041
aPFB0920w_0	PFB0920w	1.154647	1.216247	0.385007	-1.05367	-1.86041
intPFB0921c_2	PFB0921c	-0.61199	-0.52606	1.257623	0.998043	-0.95528
aPFB0921c_0	PFB0921c	-0.61199	-0.52606	1.257623	0.998043	-0.95528
intPFB0923c_3	PFB0923c	-0.94902	0.5731	1.5828	0.6162	-1.2791
aPFB0923c_0	PFB0923c	-0.94902	0.5731	1.5828	0.6162	-1.2791
intPFB0925w_2	PFB0925w	2.29482	1.23041	-0.46658	-2.06842	-2.19191
aPFB0925w_1	PFB0925w	2.29482	1.23041	-0.46658	-2.06842	-2.19191
aPFB0925w_0	PFB0925w	2.29482	1.23041	-0.46658	-2.06842	-2.19191
intPFB0926c_2	PFB0926c	0.062887	0.295557	0.714387	-0.46505	-1.83122
intPFB0930w_2	PFB0930w	0.417488	0.917538	0.937838	-0.07182	-1.35457
aPFB0930w_0	PFB0930w	0.417488	0.917538	0.937838	-0.07182	-1.35457
intPFB0932w_2	PFB0932w	2.103157	1.380207	0.153587	-1.47625	-2.27687
aPFB0932w_0	PFB0932w	2.103157	1.380207	0.153587	-1.47625	-2.27687
intPFB0935w_2	PFB0935w	0.061923	-0.54612	-1.69703	-1.00981	1.211783
aPFB0935w_2	PFB0935w	0.061923	-0.54612	-1.69703	-1.00981	1.211783

aPFB0935w_1	PFB0935w	0.061923	-0.54612	-1.69703	-1.00981	1.211783
aPFB0935w_0	PFB0935w	0.061923	-0.54612	-1.69703	-1.00981	1.211783
intPFB0950w_2	PFB0946c					
intPFB0946c_2	PFB0946c					
aPFB0946c_0	PFB0946c					
aPFB0950w_0	PFB0950w					
intPFB0953w_2	PFB0953w	-0.31934	0.944707	0.037087	-0.42975	0.023627
aPFB0953w_0	PFB0953w	-0.31934	0.944707	0.037087	-0.42975	0.023627
intPFB0960c_3	PFB0960c	0.166052	0.447482	0.453632	0.107492	-0.42747
aPFB0960c_0	PFB0960c	0.166052	0.447482	0.453632	0.107492	-0.42747
intPFB0965c_2	PFB0965c					
intPFB0970c_2	PFB0970c	0.07465	0.77173	0.6073	-0.1233	-0.80873
aPFB0970c_0	PFB0970c	0.07465	0.77173	0.6073	-0.1233	-0.80873
intPFB0972w_2	PFB0972w	0.23114	0.46629	0.17943	-0.07543	-0.20829
aPFB0972w_0	PFB0972w	0.23114	0.46629	0.17943	-0.07543	-0.20829
intPFB0976w_2	PFB0973c	0.83282	0.89191	0.10767	-0.54767	-0.70191
intPFB0973c_2	PFB0973c					
aPFB0973c_0	PFB0973c					
intPFB0980w_2	PFB0980w	0.79767	1.09367	0.28017	-0.48017	-0.77067
intPFB0985c_2	PFB0985c	-0.91795	0.107555	0.130095	0.186365	0.418715
aPFB0985c_0	PFB0985c	-0.91795	0.107555	0.130095	0.186365	0.418715
intPFB0990c_2	PFB0990c	1.649307	1.912457	0.408097	-1.24676	-1.89312
intPFB0995w_2	PFB0995w	1.173698	1.290498	0.611478	-0.85127	-1.92884
intPFB1030w_2	PFB1030w	0.35246	0.29872	-0.31911	-0.43863	0.00876
intPFC0050c_2	PFC0050c	1.79844	0.11434	0.12969	-0.20074	-1.06213
aPFC0050c_1	PFC0050c	1.79844	0.11434	0.12969	-0.20074	-1.06213
aPFC0050c_0	PFC0050c	1.79844	0.11434	0.12969	-0.20074	-1.06213
intPFC0055w_2	PFC0055w					
aPFC0055w_0	PFC0055w					
intPFC0060c_3	PFC0060c	2.540583	1.385073	-1.16665	-2.49319	-1.52441
aPFC0060c_1	PFC0060c	2.540583	1.385073	-1.16665	-2.49319	-1.52441
aPFC0060c_0	PFC0060c	2.540583	1.385073	-1.16665	-2.49319	-1.52441
intPFC0065c_2	PFC0065c	1.52887	0.96966	0.71992	-0.74342	-2.29816
aPFC0065c_1	PFC0065c	1.52887	0.96966	0.71992	-0.74342	-2.29816
aPFC0065c_0	PFC0065c	1.52887	0.96966	0.71992	-0.74342	-2.29816
intPFC0070c_2	PFC0070c	-0.84266	0.187293	1.210913	0.877753	-0.49763
aPFC0070c_0	PFC0070c	-0.84266	0.187293	1.210913	0.877753	-0.49763
intPFC0075c_3	PFC0075c	1.264947	1.534277	0.219947	-0.92861	-1.19494
aPFC0075c_0	PFC0075c	1.264947	1.534277	0.219947	-0.92861	-1.19494
intPFC0080c_2	PFC0080c	1.347143	1.064793	0.818163	-0.818	-2.46613
aPFC0080c_1	PFC0080c	1.347143	1.064793	0.818163	-0.818	-2.46613
aPFC0080c_0	PFC0080c	1.347143	1.064793	0.818163	-0.818	-2.46613
intPFC0085c_2	PFC0085c	-1.61559	-0.91039	1.687102	1.823132	-0.42673
aPFC0085c_0	PFC0085c	-1.61559	-0.91039	1.687102	1.823132	-0.42673
intPFC0090w_2	PFC0090w	1.000023	0.386903	-0.4433	-0.95904	-0.70124
aPFC0090w_0	PFC0090w	1.000023	0.386903	-0.4433	-0.95904	-0.70124
intPFC0095c_2	PFC0095c	-0.04953	-0.65485	0.352957	0.650877	-0.09081

aPFC0095c_1	PFC0095c	-0.04953	-0.65485	0.352957	0.650877	-0.09081
aPFC0095c_0	PFC0095c	-0.04953	-0.65485	0.352957	0.650877	-0.09081
intPFC0100c_2	PFC0100c	0.912417	0.792437	0.322877	-0.38854	-0.9506
aPFC0100c_1	PFC0100c	0.912417	0.792437	0.322877	-0.38854	-0.9506
aPFC0100c_0	PFC0100c	0.912417	0.792437	0.322877	-0.38854	-0.9506
intPFC0105w_2	PFC0105w	0.011257	0.530707	0.858577	0.305757	-0.76537
aPFC0105w_1	PFC0105w	0.011257	0.530707	0.858577	0.305757	-0.76537
aPFC0105w_0	PFC0105w	0.011257	0.530707	0.858577	0.305757	-0.76537
intPFC0110w_2	PFC0110w	-0.65455	-0.85025	-2.57849	-1.30767	2.417087
aPFC0110w_2	PFC0110w	-0.65455	-0.85025	-2.57849	-1.30767	2.417087
intPFC0120w_2	PFC0120w	-0.64233	-1.35333	-2.67358	-1.11142	2.54633
aPFC0120w_2	PFC0120w	-0.64233	-1.35333	-2.67358	-1.11142	2.54633
aPFC0120w_0	PFC0120w	-0.64233	-1.35333	-2.67358	-1.11142	2.54633
intPFC0125w_2	PFC0125w	-0.74281	-0.86775	0.465258	0.991458	0.303308
aPFC0125w_2	PFC0125w	-0.74281	-0.86775	0.465258	0.991458	0.303308
aPFC0125w_1	PFC0125w	-0.74281	-0.86775	0.465258	0.991458	0.303308
aPFC0125w_0	PFC0125w	-0.74281	-0.86775	0.465258	0.991458	0.303308
intPFC0126c_2	PFC0126c	-0.71643	-0.46284	0.14367	0.50133	0.41884
aPFC0126c_0	PFC0126c	-0.71643	-0.46284	0.14367	0.50133	0.41884
intPFC0130c_2	PFC0130c	0.716613	0.544433	0.239883	-0.48372	-1.04127
aPFC0130c_1	PFC0130c	0.716613	0.544433	0.239883	-0.48372	-1.04127
aPFC0130c_0	PFC0130c	0.716613	0.544433	0.239883	-0.48372	-1.04127
intPFC0135c_2	PFC0135c	-0.02853	-0.01586	-0.05403	0.181693	0.370523
aPFC0135c_1	PFC0135c	-0.02853	-0.01586	-0.05403	0.181693	0.370523
aPFC0135c_0	PFC0135c	-0.02853	-0.01586	-0.05403	0.181693	0.370523
intPFC0140c_2	PFC0140c	-0.18773	0.509147	-0.08555	-0.28511	0.131187
aPFC0140c_1	PFC0140c	-0.18773	0.509147	-0.08555	-0.28511	0.131187
aPFC0140c_0	PFC0140c	-0.18773	0.509147	-0.08555	-0.28511	0.131187
intPFC0145c_2	PFC0145c	-0.20516	0.179255	-0.26743	-0.39936	0.079135
aPFC0145c_2	PFC0145c	-0.20516	0.179255	-0.26743	-0.39936	0.079135
aPFC0145c_1	PFC0145c	-0.20516	0.179255	-0.26743	-0.39936	0.079135
aPFC0145c_0	PFC0145c	-0.20516	0.179255	-0.26743	-0.39936	0.079135
intPFC0150w_3	PFC0150w	-0.67246	-0.78466	-1.03993	-0.27273	1.198497
aPFC0150w_1	PFC0150w	-0.67246	-0.78466	-1.03993	-0.27273	1.198497
aPFC0150w_0	PFC0150w	-0.67246	-0.78466	-1.03993	-0.27273	1.198497
intPFC0155c_2	PFC0155c	0.245857	0.260737	0.620037	0.260297	-0.6544
aPFC0155c_0	PFC0155c	0.245857	0.260737	0.620037	0.260297	-0.6544
intPFC0160w_2	PFC0160w	-0.33411	-0.52443	-1.26638	-0.38578	1.504767
aPFC0160w_1	PFC0160w	-0.33411	-0.52443	-1.26638	-0.38578	1.504767
aPFC0160w_0	PFC0160w	-0.33411	-0.52443	-1.26638	-0.38578	1.504767
intPFC0165w_2	PFC0165w	-1.00749	-1.06022	-0.45956	0.597562	1.367882
aPFC0165w_3	PFC0165w	-1.00749	-1.06022	-0.45956	0.597562	1.367882
aPFC0165w_2	PFC0165w	-1.00749	-1.06022	-0.45956	0.597562	1.367882
aPFC0165w_1	PFC0165w	-1.00749	-1.06022	-0.45956	0.597562	1.367882
aPFC0165w_0	PFC0165w	-1.00749	-1.06022	-0.45956	0.597562	1.367882
intPFC0166w_2	PFC0166w	-0.46787	0.12937	1.24277	0.88323	-0.71137
aPFC0166w_0	PFC0166w	-0.46787	0.12937	1.24277	0.88323	-0.71137

aPFC0170c_1	PFC0170c	-0.54863	-0.88434	0.078187	0.694147	0.491177
aPFC0170c_0	PFC0170c	-0.54863	-0.88434	0.078187	0.694147	0.491177
intPFC0175w_2	PFC0175w	-1.11805	-0.59777	0.56479	0.83671	0.19377
intPFC0170c_2	PFC0175w	-0.54863	-0.88434	0.078187	0.694147	0.491177
aPFC0175w_1	PFC0175w	-1.11805	-0.59777	0.56479	0.83671	0.19377
aPFC0175w_0	PFC0175w	-1.11805	-0.59777	0.56479	0.83671	0.19377
intPFC0176c_2	PFC0176c	-0.75529	-0.69456	-0.76912	0.077723	1.315343
aPFC0176c_0	PFC0176c	-0.75529	-0.69456	-0.76912	0.077723	1.315343
intPFC0180c_2	PFC0180c	0.316873	0.134653	-0.59381	-0.58453	0.196013
aPFC0180c_1	PFC0180c	0.316873	0.134653	-0.59381	-0.58453	0.196013
aPFC0180c_0	PFC0180c	0.316873	0.134653	-0.59381	-0.58453	0.196013
intPFC0185w_2	PFC0185w	0.95608	0.26608	-2.19217	-2.40783	0.26592
aPFC0185w_1	PFC0185w	0.95608	0.26608	-2.19217	-2.40783	0.26592
aPFC0185w_0	PFC0185w	0.95608	0.26608	-2.19217	-2.40783	0.26592
intPFC0190c_2	PFC0190c	0.691213	0.593943	0.128873	-0.64271	-1.03478
aPFC0190c_1	PFC0190c	0.691213	0.593943	0.128873	-0.64271	-1.03478
aPFC0190c_0	PFC0190c	0.691213	0.593943	0.128873	-0.64271	-1.03478
intPFC0195w_2	PFC0191c	-0.71674	-0.48532	-0.15786	0.41536	0.82782
aPFC0191c_0	PFC0191c	-0.37672	-0.83948	-0.08929	0.57713	0.59532
intPFC0191c_2	PFC0195w	-0.37672	-0.83948	-0.08929	0.57713	0.59532
aPFC0195w_1	PFC0195w	-0.71674	-0.48532	-0.15786	0.41536	0.82782
aPFC0195w_0	PFC0195w	-0.71674	-0.48532	-0.15786	0.41536	0.82782
intPFC0200w_2	PFC0200w-a	0.015233	0.361853	0.754303	0.440363	-0.48619
aPFC0200w_0	PFC0200w-a	0.015233	0.361853	0.754303	0.440363	-0.48619
intPFC0205c_2	PFC0205c	-0.90945	-0.72778	0.974553	1.308113	-0.00656
aPFC0205c_0	PFC0205c	-0.90945	-0.72778	0.974553	1.308113	-0.00656
intPFC0210c_2	PFC0210c	0.124187	-0.14741	-0.29517	0.085507	0.529747
aPFC0210c_1	PFC0210c	0.124187	-0.14741	-0.29517	0.085507	0.529747
aPFC0210c_0	PFC0210c	0.124187	-0.14741	-0.29517	0.085507	0.529747
intPFC0220w_2	PFC0215c	-0.98565	-1.13973	-0.38055	0.68855	1.29923
aPFC0215c_1	PFC0215c	-0.55197	-0.38513	-0.30974	0.183577	0.770467
aPFC0215c_0	PFC0215c	-0.55197	-0.38513	-0.30974	0.183577	0.770467
intPFC0215c_2	PFC0220w	-0.55197	-0.38513	-0.30974	0.183577	0.770467
aPFC0220w_1	PFC0220w	-0.98565	-1.13973	-0.38055	0.68855	1.29923
aPFC0220w_0	PFC0220w	-0.98565	-1.13973	-0.38055	0.68855	1.29923
intPFC0221c_2	PFC0221c	0.471823	0.251873	0.600253	0.134413	-0.88821
aPFC0221c_0	PFC0221c	0.471823	0.251873	0.600253	0.134413	-0.88821
intPFC0225c_2	PFC0225c	-0.12548	0.068863	-0.02973	0.055393	0.225303
aPFC0225c_1	PFC0225c	-0.12548	0.068863	-0.02973	0.055393	0.225303
aPFC0225c_0	PFC0225c	-0.12548	0.068863	-0.02973	0.055393	0.225303
intPFC0230c_2	PFC0230c	-1.06553	-0.47603	0.22406	0.67744	0.6432
aPFC0230c_5	PFC0230c	-1.06553	-0.47603	0.22406	0.67744	0.6432
aPFC0230c_4	PFC0230c	-1.06553	-0.47603	0.22406	0.67744	0.6432
aPFC0230c_3	PFC0230c	-1.06553	-0.47603	0.22406	0.67744	0.6432
aPFC0230c_2	PFC0230c	-1.06553	-0.47603	0.22406	0.67744	0.6432
aPFC0230c_1	PFC0230c	-1.06553	-0.47603	0.22406	0.67744	0.6432
aPFC0230c_0	PFC0230c	-1.06553	-0.47603	0.22406	0.67744	0.6432

aPFC0235w_1	PFC0235w	0.211793	0.310043	-0.27009	-0.57075	-0.22488
aPFC0235w_0	PFC0235w	0.211793	0.310043	-0.27009	-0.57075	-0.22488
aPFC0240c_1	PFC0240c	1.375713	0.893923	-0.17734	-1.1195	-1.21676
aPFC0240c_0	PFC0240c	1.375713	0.893923	-0.17734	-1.1195	-1.21676
intPFC0241w_2	PFC0241w	0.433082	0.165102	-0.11714	-0.17147	-0.07674
aPFC0241w_0	PFC0241w	0.433082	0.165102	-0.11714	-0.17147	-0.07674
intPFC0245c_2	PFC0245c	-0.26665	0.104687	0.274077	-0.06094	-0.43522
aPFC0245c_5	PFC0245c	-0.26665	0.104687	0.274077	-0.06094	-0.43522
aPFC0245c_4	PFC0245c	-0.26665	0.104687	0.274077	-0.06094	-0.43522
aPFC0245c_3	PFC0245c	-0.26665	0.104687	0.274077	-0.06094	-0.43522
aPFC0245c_2	PFC0245c	-0.26665	0.104687	0.274077	-0.06094	-0.43522
aPFC0245c_1	PFC0245c	-0.26665	0.104687	0.274077	-0.06094	-0.43522
aPFC0245c_0	PFC0245c	-0.26665	0.104687	0.274077	-0.06094	-0.43522
intPFC0250c_2	PFC0250c	-0.66068	-1.50312	-0.87127	0.57577	1.67912
aPFC0250c_1	PFC0250c	-0.66068	-1.50312	-0.87127	0.57577	1.67912
aPFC0250c_0	PFC0250c	-0.66068	-1.50312	-0.87127	0.57577	1.67912
aPFC0255c_0	PFC0255c	-0.31388	0.023427	-0.05585	0.054187	0.303907
intPFC0260w_2	PFC0260w	-0.55077	-0.20961	-0.24152	-0.08914	0.352947
intPFC0255c_1	PFC0260w	-0.31388	0.023427	-0.05585	0.054187	0.303907
aPFC0260w_1	PFC0260w	-0.55077	-0.20961	-0.24152	-0.08914	0.352947
aPFC0260w_0	PFC0260w	-0.55077	-0.20961	-0.24152	-0.08914	0.352947
aPFC0261c_0	PFC0261c	-0.45112	-0.7614	-0.34919	0.256527	0.667737
intPFC0262c_2	PFC0262c	-0.64121	-1.16995	-0.33911	0.612773	0.974613
intPFC0261c_2	PFC0262c	-0.45112	-0.7614	-0.34919	0.256527	0.667737
aPFC0262c_0	PFC0262c	-0.64121	-1.16995	-0.33911	0.612773	0.974613
intPFC0265c_2	PFC0265c	0.219607	0.225467	0.091567	-0.01823	-0.11813
aPFC0265c_1	PFC0265c	0.219607	0.225467	0.091567	-0.01823	-0.11813
aPFC0265c_0	PFC0265c	0.219607	0.225467	0.091567	-0.01823	-0.11813
intPFC0270w_2	PFC0270w	-0.18343	0.125713	0.829373	0.672493	-0.36685
aPFC0270w_0	PFC0270w	-0.18343	0.125713	0.829373	0.672493	-0.36685
intPFC0271c_2	PFC0271c	0.030027	0.090877	0.703237	0.608097	-0.32654
aPFC0271c_0	PFC0271c	0.030027	0.090877	0.703237	0.608097	-0.32654
intPFC0275w_2	PFC0275w	-0.97128	-0.96611	-0.82003	0.35203	1.71111
aPFC0275w_1	PFC0275w	-0.97128	-0.96611	-0.82003	0.35203	1.71111
aPFC0275w_0	PFC0275w	-0.97128	-0.96611	-0.82003	0.35203	1.71111
intPFC0281w_2	PFC0280c	-0.7509	-1.15288	-0.18155	0.774798	0.976758
intPFC0280c_2	PFC0280c	-0.89658	-1.2441	-0.65179	0.54179	1.4771
aPFC0280c_0	PFC0280c	-0.89658	-1.2441	-0.65179	0.54179	1.4771
aPFC0281w_0	PFC0281w	-0.7509	-1.15288	-0.18155	0.774798	0.976758
intPFC0282w_2	PFC0282w	0.301663	0.446683	0.443623	0.130043	-0.42102
aPFC0282w_0	PFC0282w	0.301663	0.446683	0.443623	0.130043	-0.42102
intPFC0285c_2	PFC0285c	0.03683	0.48035	0.95679	0.40221	-0.84285
aPFC0285c_1	PFC0285c	0.03683	0.48035	0.95679	0.40221	-0.84285
aPFC0285c_0	PFC0285c	0.03683	0.48035	0.95679	0.40221	-0.84285
intPFC0290w_2	PFC0290w	0.09579	0.30105	0.6439	0.4551	-0.32905
aPFC0290w_0	PFC0290w	0.09579	0.30105	0.6439	0.4551	-0.32905
intPFC0295c_2	PFC0295c	0.562203	0.066003	0.524983	0.413683	-0.48534

aPFC0295c_0	PFC0295c	0.562203	0.066003	0.524983	0.413683	-0.48534
intPFC0300c_2	PFC0300c	0.30294	0.37228	0.70694	0.32306	-0.66428
aPFC0300c_0	PFC0300c	0.30294	0.37228	0.70694	0.32306	-0.66428
intPFC0305w_2	PFC0305w	0.05618	-0.06138	-0.29423	-0.33927	-0.02912
aPFC0305w_1	PFC0305w	0.05618	-0.06138	-0.29423	-0.33927	-0.02912
aPFC0305w_0	PFC0305w	0.05618	-0.06138	-0.29423	-0.33927	-0.02912
intPFC0310c_2	PFC0310c	-0.95114	-1.38131	-0.29514	0.874803	1.249473
aPFC0310c_1	PFC0310c	-0.95114	-1.38131	-0.29514	0.874803	1.249473
aPFC0310c_0	PFC0310c	-0.95114	-1.38131	-0.29514	0.874803	1.249473
intPFC0320w_2	PFC0315c	-1.10777	-1.35467	-0.29491	0.88591	1.33567
aPFC0315c_1	PFC0315c	-1.20489	-1.28005	-0.17641	0.94491	1.28905
aPFC0315c_0	PFC0315c	-1.20489	-1.28005	-0.17641	0.94491	1.28905
intPFC0315c_2	PFC0320w	-1.20489	-1.28005	-0.17641	0.94491	1.28905
aPFC0320w_1	PFC0320w	-1.10777	-1.35467	-0.29491	0.88591	1.33567
aPFC0320w_0	PFC0320w	-1.10777	-1.35467	-0.29491	0.88591	1.33567
intPFC0325c_3	PFC0325c	-1.50945	-1.48872	-0.29923	0.915118	1.431938
aPFC0325c_2	PFC0325c	-1.50945	-1.48872	-0.29923	0.915118	1.431938
aPFC0325c_1	PFC0325c	-1.50945	-1.48872	-0.29923	0.915118	1.431938
aPFC0325c_0	PFC0325c	-1.50945	-1.48872	-0.29923	0.915118	1.431938
intPFC0330w_2	PFC0330w	0.248637	0.342337	0.256077	-0.06224	-0.4175
aPFC0330w_1	PFC0330w	0.248637	0.342337	0.256077	-0.06224	-0.4175
aPFC0330w_0	PFC0330w	0.248637	0.342337	0.256077	-0.06224	-0.4175
intPFC0335c_3	PFC0335c	-0.81588	-0.18432	0.541852	0.692582	0.194552
aPFC0335c_5	PFC0335c	-0.81588	-0.18432	0.541852	0.692582	0.194552
aPFC0335c_4	PFC0335c	-0.81588	-0.18432	0.541852	0.692582	0.194552
aPFC0335c_3	PFC0335c	-0.81588	-0.18432	0.541852	0.692582	0.194552
aPFC0335c_2	PFC0335c	-0.81588	-0.18432	0.541852	0.692582	0.194552
aPFC0335c_1	PFC0335c	-0.81588	-0.18432	0.541852	0.692582	0.194552
aPFC0335c_0	PFC0335c	-0.81588	-0.18432	0.541852	0.692582	0.194552
intPFC0340w_2	PFC0340w	-1.30274	-0.77734	0.410183	1.012983	0.683503
aPFC0340w_1	PFC0340w	-1.30274	-0.77734	0.410183	1.012983	0.683503
aPFC0340w_0	PFC0340w	-1.30274	-0.77734	0.410183	1.012983	0.683503
intPFC0345w_2	PFC0345w	-0.41103	-0.31119	-1.67096	-0.89882	1.644742
aPFC0345w_2	PFC0345w	-0.41103	-0.31119	-1.67096	-0.89882	1.644742
aPFC0345w_1	PFC0345w	-0.41103	-0.31119	-1.67096	-0.89882	1.644742
aPFC0345w_0	PFC0345w	-0.41103	-0.31119	-1.67096	-0.89882	1.644742
intPFC0350c_2	PFC0350c	-0.17111	0.095057	0.836137	0.408197	-0.80022
aPFC0350c_1	PFC0350c	-0.17111	0.095057	0.836137	0.408197	-0.80022
aPFC0350c_0	PFC0350c	-0.17111	0.095057	0.836137	0.408197	-0.80022
intPFC0355c_2	PFC0355c	1.375447	0.212757	-1.35801	-1.89165	-0.66442
aPFC0355c_0	PFC0355c	1.375447	0.212757	-1.35801	-1.89165	-0.66442
intPFC0360w_2	PFC0360w	1.362053	-0.02926	0.403513	0.126153	-1.02408
aPFC0360w_0	PFC0360w	1.362053	-0.02926	0.403513	0.126153	-1.02408
intPFC0365w_3	PFC0365w	1.013207	0.211957	0.314587	0.012587	-0.75429
aPFC0365w_1	PFC0365w	1.013207	0.211957	0.314587	0.012587	-0.75429
aPFC0365w_0	PFC0365w	1.013207	0.211957	0.314587	0.012587	-0.75429
intPFC0370w_2	PFC0370w	0.649637	0.368827	-1.36265	-1.67001	0.022507

aPFC0370w_1	PFC0370w	0.649637	0.368827	-1.36265	-1.67001	0.022507
aPFC0370w_0	PFC0370w	0.649637	0.368827	-1.36265	-1.67001	0.022507
intPFC0371w_2	PFC0371w	0.141277	-0.44737	-0.39826	-0.1694	0.130707
aPFC0371w_0	PFC0371w	0.141277	-0.44737	-0.39826	-0.1694	0.130707
intPFC0380w_1	PFC0375c	0.399723	0.469363	0.233283	-0.07512	-0.3607
aPFC0375c_2	PFC0375c	0.617295	0.620505	0.290825	-0.18816	-0.60751
aPFC0375c_1	PFC0375c	0.617295	0.620505	0.290825	-0.18816	-0.60751
aPFC0375c_0	PFC0375c	0.617295	0.620505	0.290825	-0.18816	-0.60751
intPFC0375c_1	PFC0380w	0.617295	0.620505	0.290825	-0.18816	-0.60751
aPFC0380w_1	PFC0380w	0.399723	0.469363	0.233283	-0.07512	-0.3607
aPFC0380w_0	PFC0380w	0.399723	0.469363	0.233283	-0.07512	-0.3607
intPFC0381c_2	PFC0381c	-0.32798	-0.5191	-0.7813	-0.0917	1.0581
aPFC0381c_0	PFC0381c	-0.32798	-0.5191	-0.7813	-0.0917	1.0581
intPFC0385c_2	PFC0385c	-0.46431	0.122523	-0.42538	-0.21974	0.686703
aPFC0385c_2	PFC0385c	-0.46431	0.122523	-0.42538	-0.21974	0.686703
aPFC0385c_1	PFC0385c	-0.46431	0.122523	-0.42538	-0.21974	0.686703
aPFC0385c_0	PFC0385c	-0.46431	0.122523	-0.42538	-0.21974	0.686703
intPFC0390w_2	PFC0390w	0.332047	0.896777	0.306707	-0.38887	-0.65844
aPFC0390w_1	PFC0390w	0.332047	0.896777	0.306707	-0.38887	-0.65844
aPFC0390w_0	PFC0390w	0.332047	0.896777	0.306707	-0.38887	-0.65844
intPFC0395w_2	PFC0395w	0.022333	0.044313	0.432373	0.261293	-0.37865
aPFC0395w_1	PFC0395w	0.022333	0.044313	0.432373	0.261293	-0.37865
aPFC0395w_0	PFC0395w	0.022333	0.044313	0.432373	0.261293	-0.37865
intPFC0400w_2	PFC0400w	0.063463	0.262683	0.606143	0.391523	-0.37002
aPFC0400w_0	PFC0400w	0.063463	0.262683	0.606143	0.391523	-0.37002
aPFC0405c_1	PFC0405c	1.00088	0.6261	-0.19894	-0.71306	-0.6211
aPFC0405c_0	PFC0405c	1.00088	0.6261	-0.19894	-0.71306	-0.6211
intPFC0410w_2	PFC0410w	0.934903	0.431493	0.275503	-0.41984	-1.15883
intPFC0405c_2	PFC0410w	1.00088	0.6261	-0.19894	-0.71306	-0.6211
aPFC0410w_0	PFC0410w	0.934903	0.431493	0.275503	-0.41984	-1.15883
intPFC0416w_2	PFC0415c	-0.55957	-1.02016	-0.07717	0.77017	0.80316
aPFC0415c_2	PFC0415c	-0.54028	-0.47927	0.040128	0.496148	0.531218
aPFC0415c_1	PFC0415c	-0.54028	-0.47927	0.040128	0.496148	0.531218
aPFC0415c_0	PFC0415c	-0.54028	-0.47927	0.040128	0.496148	0.531218
intPFC0415c_2	PFC0416w	-0.54028	-0.47927	0.040128	0.496148	0.531218
aPFC0416w_0	PFC0416w	-0.55957	-1.02016	-0.07717	0.77017	0.80316
intPFC0420w_2	PFC0420w	-0.12357	0.08485	-0.46219	-0.19531	0.65215
aPFC0420w_1	PFC0420w	-0.12357	0.08485	-0.46219	-0.19531	0.65215
aPFC0420w_0	PFC0420w	-0.12357	0.08485	-0.46219	-0.19531	0.65215
intPFC0425w_2	PFC0425w	0.507183	0.014603	0.092033	-0.27737	-0.74411
aPFC0425w_6	PFC0425w	0.507183	0.014603	0.092033	-0.27737	-0.74411
aPFC0425w_5	PFC0425w	0.507183	0.014603	0.092033	-0.27737	-0.74411
aPFC0425w_4	PFC0425w	0.507183	0.014603	0.092033	-0.27737	-0.74411
aPFC0425w_3	PFC0425w	0.507183	0.014603	0.092033	-0.27737	-0.74411
aPFC0425w_2	PFC0425w	0.507183	0.014603	0.092033	-0.27737	-0.74411
aPFC0425w_1	PFC0425w	0.507183	0.014603	0.092033	-0.27737	-0.74411
aPFC0425w_0	PFC0425w	0.507183	0.014603	0.092033	-0.27737	-0.74411

intPFC0430w_2	PFC0430w	0.38842	0.75342	0.65467	-0.04867	-0.90042
aPFC0430w_1	PFC0430w	0.38842	0.75342	0.65467	-0.04867	-0.90042
aPFC0430w_0	PFC0430w	0.38842	0.75342	0.65467	-0.04867	-0.90042
intPFC0435w_2	PFC0435w	-1.31975	-1.47475	-1.29	0.447	2.49225
aPFC0435w_1	PFC0435w	-1.31975	-1.47475	-1.29	0.447	2.49225
aPFC0435w_0	PFC0435w	-1.31975	-1.47475	-1.29	0.447	2.49225
intPFC0440c_2	PFC0440c	0.042177	0.526637	0.386987	0.119097	-0.1938
aPFC0440c_3	PFC0440c	0.042177	0.526637	0.386987	0.119097	-0.1938
aPFC0440c_2	PFC0440c	0.042177	0.526637	0.386987	0.119097	-0.1938
aPFC0440c_1	PFC0440c	0.042177	0.526637	0.386987	0.119097	-0.1938
aPFC0440c_0	PFC0440c	0.042177	0.526637	0.386987	0.119097	-0.1938
aPFC0441c_0	PFC0441c-a	0.45562	0.0909	0.53419	0.25381	-0.6899
intPFC0450w_2	PFC0445w	-0.20636	0.021783	0.342433	0.226233	-0.20512
intPFC0445w_3	PFC0445w	-0.15937	-0.11418	0.379853	0.328813	-0.22616
intPFC0441c_2	PFC0445w	0.45562	0.0909	0.53419	0.25381	-0.6899
aPFC0445w_0	PFC0445w	-0.15937	-0.11418	0.379853	0.328813	-0.22616
aPFC0450w_0	PFC0450w	-0.20636	0.021783	0.342433	0.226233	-0.20512
intPFC0460w_2	PFC0455w	-0.03337	0.012823	-0.44915	-0.35819	0.305843
intPFC0455w_2	PFC0455w	-0.55223	-0.44683	-0.44884	0.10384	0.86483
aPFC0455w_1	PFC0455w	-0.55223	-0.44683	-0.44884	0.10384	0.86483
aPFC0455w_0	PFC0455w	-0.55223	-0.44683	-0.44884	0.10384	0.86483
aPFC0460w_0	PFC0460w	-0.03337	0.012823	-0.44915	-0.35819	0.305843
aPFC0465c_1	PFC0465c	0.371597	0.222507	0.072497	-0.05016	-0.18367
aPFC0465c_0	PFC0465c	0.371597	0.222507	0.072497	-0.05016	-0.18367
intPFC0470w_2	PFC0470w	0.331285	0.503265	0.427135	0.034415	-0.50638
intPFC0465c_2	PFC0470w	0.371597	0.222507	0.072497	-0.05016	-0.18367
aPFC0470w_2	PFC0470w	0.331285	0.503265	0.427135	0.034415	-0.50638
aPFC0470w_1	PFC0470w	0.331285	0.503265	0.427135	0.034415	-0.50638
aPFC0470w_0	PFC0470w	0.331285	0.503265	0.427135	0.034415	-0.50638
intPFC0475c_2	PFC0475c	-0.9466	-1.076	-0.92724	0.16924	1.552
aPFC0475c_1	PFC0475c	-0.9466	-1.076	-0.92724	0.16924	1.552
aPFC0475c_0	PFC0475c	-0.9466	-1.076	-0.92724	0.16924	1.552
intPFC0485w_2	PFC0485w	-0.11978	-0.2783	-0.77065	-0.43552	0.613963
aPFC0485w_3	PFC0485w	-0.11978	-0.2783	-0.77065	-0.43552	0.613963
aPFC0485w_2	PFC0485w	-0.11978	-0.2783	-0.77065	-0.43552	0.613963
aPFC0485w_1	PFC0485w	-0.11978	-0.2783	-0.77065	-0.43552	0.613963
aPFC0485w_0	PFC0485w	-0.11978	-0.2783	-0.77065	-0.43552	0.613963
intPFC0486c_2	PFC0486c	0.895373	0.733153	0.209193	-0.67753	-1.20949
aPFC0486c_0	PFC0486c	0.895373	0.733153	0.209193	-0.67753	-1.20949
intPFC0495w_2	PFC0490w	-0.0742	-0.13903	-0.1862	-0.08814	0.141193
intPFC0490w_2	PFC0490w	0.25309	-0.12243	0.6601	0.3894	-0.79207
aPFC0490w_1	PFC0490w	0.25309	-0.12243	0.6601	0.3894	-0.79207
aPFC0490w_0	PFC0490w	0.25309	-0.12243	0.6601	0.3894	-0.79207
intPFC0500w_2	PFC0495w	-0.80503	-1.40781	-0.29687	0.873707	1.184647
aPFC0495w_1	PFC0495w	-0.0742	-0.13903	-0.1862	-0.08814	0.141193
aPFC0495w_0	PFC0495w	-0.0742	-0.13903	-0.1862	-0.08814	0.141193
aPFC0500w_1	PFC0500w	-0.80503	-1.40781	-0.29687	0.873707	1.184647

aPFC0500w_0	PFC0500w	-0.80503	-1.40781	-0.29687	0.873707	1.184647
intPFC0506w_2	PFC0505c	-1.10613	-1.03134	0.557687	1.336647	0.663677
aPFC0505c_1	PFC0505c	-0.94817	-0.65362	0.448007	0.865327	0.356957
aPFC0505c_0	PFC0505c	-0.94817	-0.65362	0.448007	0.865327	0.356957
intPFC0510w_2	PFC0506w	-0.22851	-0.75146	-0.66358	0.216243	1.116123
intPFC0505c_2	PFC0506w	-0.94817	-0.65362	0.448007	0.865327	0.356957
aPFC0506w_0	PFC0506w	-1.10613	-1.03134	0.557687	1.336647	0.663677
aPFC0510w_1	PFC0510w	-0.22851	-0.75146	-0.66358	0.216243	1.116123
aPFC0510w_0	PFC0510w	-0.22851	-0.75146	-0.66358	0.216243	1.116123
intPFC0520w_2	PFC0515c	-0.61813	-0.21539	0.228273	0.497393	0.390053
intPFC0515c_2	PFC0515c	-0.13495	-0.26073	-0.51054	-0.15896	0.56873
aPFC0515c_1	PFC0515c	-0.13495	-0.26073	-0.51054	-0.15896	0.56873
aPFC0515c_0	PFC0515c	-0.13495	-0.26073	-0.51054	-0.15896	0.56873
aPFC0520w_0	PFC0520w	-0.61813	-0.21539	0.228273	0.497393	0.390053
intPFC0525c_3	PFC0525c	-1.4604	-1.57754	-0.21243	1.099093	1.473203
aPFC0525c_1	PFC0525c	-1.4604	-1.57754	-0.21243	1.099093	1.473203
aPFC0525c_0	PFC0525c	-1.4604	-1.57754	-0.21243	1.099093	1.473203
intPFC0530w_2	PFC0530w	0.561123	0.708363	0.685773	0.064893	-0.8747
aPFC0530w_1	PFC0530w	0.561123	0.708363	0.685773	0.064893	-0.8747
aPFC0530w_0	PFC0530w	0.561123	0.708363	0.685773	0.064893	-0.8747
intPFC0540w_2	PFC0535w	0.441017	1.053937	0.130117	-0.60178	-0.5856
intPFC0535w_2	PFC0535w	0.979543	0.452283	0.480933	0.078733	-0.78862
aPFC0535w_0	PFC0535w	0.979543	0.452283	0.480933	0.078733	-0.78862
aPFC0540w_0	PFC0540w	0.441017	1.053937	0.130117	-0.60178	-0.5856
intPFC0550w_2	PFC0545c	-0.80482	-0.48338	0.700517	0.941317	0.073717
aPFC0545c_0	PFC0545c	0.731673	0.622643	-0.0418	-0.38454	-0.32898
intPFC0545c_2	PFC0550w	0.731673	0.622643	-0.0418	-0.38454	-0.32898
aPFC0550w_1	PFC0550w	-0.80482	-0.48338	0.700517	0.941317	0.073717
aPFC0550w_0	PFC0550w	-0.80482	-0.48338	0.700517	0.941317	0.073717
aPFC0555c_0	PFC0555c	-0.21494	-0.67129	-0.0189	0.623237	0.593627
intPFC0556c_2	PFC0556c	-0.20668	-0.25263	-0.10867	0.108652	0.262662
intPFC0555c_2	PFC0556c	-0.21494	-0.67129	-0.0189	0.623237	0.593627
aPFC0556c_0	PFC0556c	-0.20668	-0.25263	-0.10867	0.108652	0.262662
intPFC0565w_2	PFC0560c	0.42584	0.8528	0.77839	0.10011	-0.8503
aPFC0560c_1	PFC0560c	0.871957	1.083727	0.494547	-0.29571	-0.91839
aPFC0560c_0	PFC0560c	0.871957	1.083727	0.494547	-0.29571	-0.91839
intPFC0560c_2	PFC0565w	0.871957	1.083727	0.494547	-0.29571	-0.91839
aPFC0565w_1	PFC0565w	0.42584	0.8528	0.77839	0.10011	-0.8503
aPFC0565w_0	PFC0565w	0.42584	0.8528	0.77839	0.10011	-0.8503
aPFC0570c_1	PFC0570c	-0.95115	-0.75121	0.012437	0.634397	0.745047
aPFC0570c_0	PFC0570c	-0.95115	-0.75121	0.012437	0.634397	0.745047
intPFC0571c_2	PFC0571c	-0.60989	-0.70204	0.112837	0.700997	0.579877
intPFC0575w_2	PFC0571c-a	-0.90299	-0.52902	0.257537	0.751797	0.603857
intPFC0570c_2	PFC0571c-a	-0.95115	-0.75121	0.012437	0.634397	0.745047
aPFC0571c_1	PFC0571c-a	-0.60989	-0.70204	0.112837	0.700997	0.579877
aPFC0571c_0	PFC0571c-a	-0.60989	-0.70204	0.112837	0.700997	0.579877
aPFC0575w_1	PFC0575w	-0.90299	-0.52902	0.257537	0.751797	0.603857

aPFC0575w_0	PFC0575w	-0.90299	-0.52902	0.257537	0.751797	0.603857
intPFC0581w_2	PFC0580c	-0.60922	-0.33793	-0.44515	0.043813	0.859593
intPFC0580c_2	PFC0580c	-0.3445	-0.13553	0.053563	0.207603	0.237693
aPFC0580c_1	PFC0580c	-0.3445	-0.13553	0.053563	0.207603	0.237693
aPFC0580c_0	PFC0580c	-0.3445	-0.13553	0.053563	0.207603	0.237693
aPFC0581w_0	PFC0581w	-0.60922	-0.33793	-0.44515	0.043813	0.859593
intPFC0582c_2	PFC0582c-a	0.948332	0.360792	-0.12082	-0.88615	-1.18331
aPFC0582c_0	PFC0582c-a	0.948332	0.360792	-0.12082	-0.88615	-1.18331
intPFC0590c_2	PFC0590c	-1.25391	-1.16899	0.040723	1.024443	1.100653
aPFC0590c_2	PFC0590c	-1.25391	-1.16899	0.040723	1.024443	1.100653
aPFC0590c_1	PFC0590c	-1.25391	-1.16899	0.040723	1.024443	1.100653
aPFC0590c_0	PFC0590c	-1.25391	-1.16899	0.040723	1.024443	1.100653
intPFC0595c_3	PFC0595c	0.105513	0.501453	0.970293	0.346773	-0.95959
aPFC0595c_0	PFC0595c	0.105513	0.501453	0.970293	0.346773	-0.95959
intPFC0600w_2	PFC0600w	0.443753	0.596213	0.577083	-0.06542	-0.90655
aPFC0600w_0	PFC0600w	0.443753	0.596213	0.577083	-0.06542	-0.90655
intPFC0605w_2	PFC0605w	-0.10714	0.150545	-0.06366	-0.06572	0.154195
aPFC0605w_0	PFC0605w	-0.10714	0.150545	-0.06366	-0.06572	0.154195
intPFC0610c_2	PFC0610c	0.029477	0.198627	0.163247	0.004587	-0.16979
aPFC0610c_1	PFC0610c	0.029477	0.198627	0.163247	0.004587	-0.16979
aPFC0610c_0	PFC0610c	0.029477	0.198627	0.163247	0.004587	-0.16979
intPFC0615w_2	PFC0615w	-0.42495	-0.61724	-1.79075	-0.74241	1.899577
aPFC0615w_1	PFC0615w	-0.42495	-0.61724	-1.79075	-0.74241	1.899577
aPFC0615w_0	PFC0615w	-0.42495	-0.61724	-1.79075	-0.74241	1.899577
intPFC0630w_2	PFC0625w	-0.94305	-0.92175	0.148483	0.895183	0.770413
intPFC0625w_2	PFC0625w	-0.49648	-1.05709	-1.04207	0.03357	1.42159
aPFC0625w_1	PFC0625w	-0.49648	-1.05709	-1.04207	0.03357	1.42159
aPFC0625w_0	PFC0625w	-0.49648	-1.05709	-1.04207	0.03357	1.42159
aPFC0630w_0	PFC0630w	-0.94305	-0.92175	0.148483	0.895183	0.770413
intPFC0635c_2	PFC0635c	0.79207	0.30166	0.44017	-0.07517	-0.98266
aPFC0635c_0	PFC0635c	0.79207	0.30166	0.44017	-0.07517	-0.98266
intPFC0645w_2	PFC0640w					
intPFC0640w_2	PFC0640w	1.563845	1.341115	-0.54173	-1.52028	-0.91445
aPFC0640w_3	PFC0640w	1.563845	1.341115	-0.54173	-1.52028	-0.91445
aPFC0640w_2	PFC0640w	1.563845	1.341115	-0.54173	-1.52028	-0.91445
aPFC0640w_1	PFC0640w	1.563845	1.341115	-0.54173	-1.52028	-0.91445
aPFC0640w_0	PFC0640w	1.563845	1.341115	-0.54173	-1.52028	-0.91445
aPFC0645w_0	PFC0645w					
intPFC0650w_2	PFC0650w	-0.96056	-0.65374	0.216213	0.641953	0.450903
aPFC0650w_1	PFC0650w	-0.96056	-0.65374	0.216213	0.641953	0.450903
aPFC0650w_0	PFC0650w	-0.96056	-0.65374	0.216213	0.641953	0.450903
intPFC0670c_2	PFC0670c	-1.78978	-1.34009	0.11718	1.34382	1.50409
aPFC0670c_0	PFC0670c	-1.78978	-1.34009	0.11718	1.34382	1.50409
intPFC0675c_2	PFC0675c	-0.82313	-0.77034	0.294187	0.910147	0.586677
aPFC0675c_0	PFC0675c	-0.82313	-0.77034	0.294187	0.910147	0.586677
intPFC0685w_2	PFC0680w	1.025157	-0.19729	-0.28241	-0.15125	-0.18237
intPFC0680w_2	PFC0680w	-0.01047	-0.37469	-0.42463	-0.00521	0.524353

aPFC0680w_1	PFC0680w	-0.01047	-0.37469	-0.42463	-0.00521	0.524353
aPFC0680w_0	PFC0680w	-0.01047	-0.37469	-0.42463	-0.00521	0.524353
aPFC0685w_1	PFC0685w	1.025157	-0.19729	-0.28241	-0.15125	-0.18237
aPFC0685w_0	PFC0685w	1.025157	-0.19729	-0.28241	-0.15125	-0.18237
aPFC0690c_1	PFC0690c	0.585503	0.602463	-0.18292	-0.62142	-0.3903
aPFC0690c_0	PFC0690c	0.585503	0.602463	-0.18292	-0.62142	-0.3903
intPFC0695w_2	PFC0695w	0.25353	0.09136	0.02978	-0.01878	-0.10636
intPFC0690c_2	PFC0695w	0.585503	0.602463	-0.18292	-0.62142	-0.3903
aPFC0695w_0	PFC0695w	0.25353	0.09136	0.02978	-0.01878	-0.10636
intPFC0700c_3	PFC0700c	0.933833	-0.29215	-0.81971	-1.20663	-0.81319
aPFC0700c_0	PFC0700c	0.933833	-0.29215	-0.81971	-1.20663	-0.81319
intPFC0701w_2	PFC0701w	0.773973	-0.71086	-0.52678	-0.42356	-0.40148
aPFC0701w_0	PFC0701w	0.773973	-0.71086	-0.52678	-0.42356	-0.40148
aPFC0705c_3	PFC0705c	-0.90418	-1.11308	-0.44847	0.570138	1.236748
aPFC0705c_2	PFC0705c	-0.90418	-1.11308	-0.44847	0.570138	1.236748
aPFC0705c_1	PFC0705c	-0.90418	-1.11308	-0.44847	0.570138	1.236748
aPFC0705c_0	PFC0705c	-0.90418	-1.11308	-0.44847	0.570138	1.236748
intPFC0710w_2	PFC0710w-a	-1.20128	-1.84707	-0.65185	0.978187	1.831407
intPFC0705c_2	PFC0710w-a	-0.90418	-1.11308	-0.44847	0.570138	1.236748
aPFC0710w_1	PFC0710w-a	-1.20128	-1.84707	-0.65185	0.978187	1.831407
aPFC0710w_0	PFC0710w-a	-1.20128	-1.84707	-0.65185	0.978187	1.831407
intPFC0715c_2	PFC0715c	-0.67335	-0.85476	0.089753	0.714913	0.562423
aPFC0715c_1	PFC0715c	-0.67335	-0.85476	0.089753	0.714913	0.562423
aPFC0715c_0	PFC0715c	-0.67335	-0.85476	0.089753	0.714913	0.562423
intPFC0720w_2	PFC0720w	-0.82769	-1.02059	-0.45083	0.429493	1.066253
aPFC0720w_1	PFC0720w	-0.82769	-1.02059	-0.45083	0.429493	1.066253
aPFC0720w_0	PFC0720w	-0.82769	-1.02059	-0.45083	0.429493	1.066253
intPFC0725c_2	PFC0725c	-1.18813	0.50563	1.29323	0.85877	-0.34963
aPFC0725c_0	PFC0725c	-1.18813	0.50563	1.29323	0.85877	-0.34963
intPFC0730w_2	PFC0730w	-1.30325	-0.60475	-0.0265	0.642837	1.044087
aPFC0730w_0	PFC0730w	-1.30325	-0.60475	-0.0265	0.642837	1.044087
intPFC0735w_2	PFC0735w	0.106082	0.098202	0.718242	0.509252	-0.5228
aPFC0735w_0	PFC0735w	0.106082	0.098202	0.718242	0.509252	-0.5228
intPFC0740c_2	PFC0740c	0.164977	0.189637	0.007727	-0.39939	-0.5418
aPFC0740c_1	PFC0740c	0.164977	0.189637	0.007727	-0.39939	-0.5418
aPFC0740c_0	PFC0740c	0.164977	0.189637	0.007727	-0.39939	-0.5418
intPFC0745c_2	PFC0745c	-1.23055	-0.60224	0.22948	0.93152	0.98524
aPFC0745c_0	PFC0745c	-1.23055	-0.60224	0.22948	0.93152	0.98524
intPFC0750w_2	PFC0750w	0.043007	0.140487	0.056017	-0.06718	-0.12415
aPFC0750w_1	PFC0750w	0.043007	0.140487	0.056017	-0.06718	-0.12415
aPFC0750w_0	PFC0750w	0.043007	0.140487	0.056017	-0.06718	-0.12415
intPFC0755c_2	PFC0755c	-1.39309	-0.58522	-0.85566	0.091933	1.780823
aPFC0757w_0	PFC0755c					
aPFC0755c_2	PFC0755c	-1.39309	-0.58522	-0.85566	0.091933	1.780823
aPFC0755c_1	PFC0755c	-1.39309	-0.58522	-0.85566	0.091933	1.780823
aPFC0755c_0	PFC0755c	-1.39309	-0.58522	-0.85566	0.091933	1.780823
intPFC0760c_1	PFC0760c	-0.11429	0.552805	-0.24693	-0.41003	0.244295

aPFC0760c_4	PFC0760c	-0.11429	0.552805	-0.24693	-0.41003	0.244295
aPFC0760c_3	PFC0760c	-0.11429	0.552805	-0.24693	-0.41003	0.244295
aPFC0760c_2	PFC0760c	-0.11429	0.552805	-0.24693	-0.41003	0.244295
aPFC0760c_1	PFC0760c	-0.11429	0.552805	-0.24693	-0.41003	0.244295
aPFC0760c_0	PFC0760c	-0.11429	0.552805	-0.24693	-0.41003	0.244295
intPFC0765c_2	PFC0765c	-0.05333	-0.98834	-0.19356	0.53506	0.51634
aPFC0765c_1	PFC0765c	-0.05333	-0.98834	-0.19356	0.53506	0.51634
aPFC0765c_0	PFC0765c	-0.05333	-0.98834	-0.19356	0.53506	0.51634
intPFC0770c_2	PFC0770c	-0.24425	-0.42972	-0.38122	0.107997	0.655837
aPFC0770c_2	PFC0770c	-0.24425	-0.42972	-0.38122	0.107997	0.655837
aPFC0770c_1	PFC0770c	-0.24425	-0.42972	-0.38122	0.107997	0.655837
aPFC0770c_0	PFC0770c	-0.24425	-0.42972	-0.38122	0.107997	0.655837
intPFC0775w_2	PFC0775w	0.880415	1.608905	0.458825	-0.55408	-0.89316
aPFC0775w_0	PFC0775w	0.880415	1.608905	0.458825	-0.55408	-0.89316
intPFC0780w_2	PFC0780w	0.439603	0.512183	0.013543	-0.18348	-0.10512
aPFC0780w_4	PFC0780w	0.439603	0.512183	0.013543	-0.18348	-0.10512
aPFC0780w_3	PFC0780w	0.439603	0.512183	0.013543	-0.18348	-0.10512
aPFC0780w_2	PFC0780w	0.439603	0.512183	0.013543	-0.18348	-0.10512
aPFC0780w_1	PFC0780w	0.439603	0.512183	0.013543	-0.18348	-0.10512
aPFC0780w_0	PFC0780w	0.439603	0.512183	0.013543	-0.18348	-0.10512
intPFC0790w_2	PFC0785c	0.669937	0.707797	0.335407	-0.13507	-0.56896
aPFC0785c_0	PFC0785c	-0.0303	0.03749	0.27602	0.37098	0.08551
intPFC0795w_2	PFC0790w					
intPFC0785c_2	PFC0790w	-0.0303	0.03749	0.27602	0.37098	0.08551
aPFC0790w_1	PFC0790w	0.669937	0.707797	0.335407	-0.13507	-0.56896
aPFC0790w_0	PFC0790w	0.669937	0.707797	0.335407	-0.13507	-0.56896
aPFC0795w_0	PFC0795w					
intPFC0800w_2	PFC0800w	-0.4278	0.033537	1.068427	0.834407	-0.5422
aPFC0800w_1	PFC0800w	-0.4278	0.033537	1.068427	0.834407	-0.5422
aPFC0800w_0	PFC0800w	-0.4278	0.033537	1.068427	0.834407	-0.5422
intPFC0805w_3	PFC0805w	0.195142	0.423302	0.337642	-0.00792	-0.41375
aPFC0805w_3	PFC0805w	0.195142	0.423302	0.337642	-0.00792	-0.41375
aPFC0805w_2	PFC0805w	0.195142	0.423302	0.337642	-0.00792	-0.41375
aPFC0805w_1	PFC0805w	0.195142	0.423302	0.337642	-0.00792	-0.41375
aPFC0805w_0	PFC0805w	0.195142	0.423302	0.337642	-0.00792	-0.41375
intPFC0810c_2	PFC0810c	0.5303	0.5405	-0.23675	-0.43475	-0.0345
aPFC0810c_1	PFC0810c	0.5303	0.5405	-0.23675	-0.43475	-0.0345
aPFC0810c_0	PFC0810c	0.5303	0.5405	-0.23675	-0.43475	-0.0345
intPFC0815c_2	PFC0815c	0.43734	0.07065	-0.3945	-0.47716	-0.09117
aPFC0815c_1	PFC0815c	0.43734	0.07065	-0.3945	-0.47716	-0.09117
aPFC0815c_0	PFC0815c	0.43734	0.07065	-0.3945	-0.47716	-0.09117
intPFC0820w_2	PFC0820w	0.40974	-0.28216	-1.67463	-1.36159	0.70308
aPFC0820w_7	PFC0820w	0.40974	-0.28216	-1.67463	-1.36159	0.70308
aPFC0820w_6	PFC0820w	0.40974	-0.28216	-1.67463	-1.36159	0.70308
aPFC0820w_5	PFC0820w	0.40974	-0.28216	-1.67463	-1.36159	0.70308
aPFC0820w_4	PFC0820w	0.40974	-0.28216	-1.67463	-1.36159	0.70308
aPFC0820w_3	PFC0820w	0.40974	-0.28216	-1.67463	-1.36159	0.70308

aPFC0820w_2	PFC0820w	0.40974	-0.28216	-1.67463	-1.36159	0.70308
aPFC0820w_1	PFC0820w	0.40974	-0.28216	-1.67463	-1.36159	0.70308
intPFC0825c_2	PFC0825c	0.860327	0.795347	0.338317	-0.74348	-1.49201
aPFC0825c_1	PFC0825c	0.860327	0.795347	0.338317	-0.74348	-1.49201
aPFC0825c_0	PFC0825c	0.860327	0.795347	0.338317	-0.74348	-1.49201
intPFC0830w_2	PFC0830w	1.624257	0.765727	-1.49746	-2.5892	-1.16939
aPFC0830w_0	PFC0830w	1.624257	0.765727	-1.49746	-2.5892	-1.16939
intPFC0831w_2	PFC0831w	0.553943	0.165773	-0.29581	-0.72653	-0.62161
aPFC0831w_1	PFC0831w	0.553943	0.165773	-0.29581	-0.72653	-0.62161
aPFC0831w_0	PFC0831w	0.553943	0.165773	-0.29581	-0.72653	-0.62161
intPFC0835c_2	PFC0835c	-0.62212	0.01259	0.32583	0.39167	0.20591
aPFC0835c_1	PFC0835c	-0.62212	0.01259	0.32583	0.39167	0.20591
aPFC0835c_0	PFC0835c	-0.62212	0.01259	0.32583	0.39167	0.20591
intPFC0840w_2	PFC0840w	-0.70801	-0.72863	-0.52582	0.211762	1.031912
aPFC0840w_2	PFC0840w	-0.70801	-0.72863	-0.52582	0.211762	1.031912
aPFC0840w_1	PFC0840w	-0.70801	-0.72863	-0.52582	0.211762	1.031912
aPFC0840w_0	PFC0840w	-0.70801	-0.72863	-0.52582	0.211762	1.031912
intPFC0845c_3	PFC0845c	-0.23545	-0.17775	0.130017	0.326317	0.211087
aPFC0845c_0	PFC0845c	-0.23545	-0.17775	0.130017	0.326317	0.211087
intPFC0855w_2	PFC0850c	0.359233	0.237853	0.540303	0.264363	-0.55519
aPFC0850c_1	PFC0850c	0.562427	0.428887	0.347477	-0.03764	-0.59505
aPFC0850c_0	PFC0850c	0.562427	0.428887	0.347477	-0.03764	-0.59505
intPFC0850c_2	PFC0855w	0.562427	0.428887	0.347477	-0.03764	-0.59505
aPFC0855w_0	PFC0855w	0.359233	0.237853	0.540303	0.264363	-0.55519
intPFC0860w_2	PFC0860w	-0.86418	-1.2851	-0.72678	0.571117	1.620437
aPFC0860w_1	PFC0860w	-0.86418	-1.2851	-0.72678	0.571117	1.620437
aPFC0860w_0	PFC0860w	-0.86418	-1.2851	-0.72678	0.571117	1.620437
intPFC0865w_2	PFC0865w	0.735027	0.569877	0.258737	-0.1274	-0.53054
aPFC0865w_0	PFC0865w	0.735027	0.569877	0.258737	-0.1274	-0.53054
intPFC0870w_2	PFC0870w	-0.08756	0.25478	0.81444	0.67956	-0.27478
aPFC0870w_0	PFC0870w	-0.08756	0.25478	0.81444	0.67956	-0.27478
intPFC0875w_2	PFC0875w	0.720093	0.906933	0.625593	-0.69343	-1.82247
aPFC0875w_4	PFC0875w	0.720093	0.906933	0.625593	-0.69343	-1.82247
aPFC0875w_3	PFC0875w	0.720093	0.906933	0.625593	-0.69343	-1.82247
aPFC0875w_2	PFC0875w	0.720093	0.906933	0.625593	-0.69343	-1.82247
aPFC0875w_1	PFC0875w	0.720093	0.906933	0.625593	-0.69343	-1.82247
aPFC0875w_0	PFC0875w	0.720093	0.906933	0.625593	-0.69343	-1.82247
intPFC0880c_2	PFC0880c	-0.29143	-0.10389	-0.31873	-0.18611	0.333053
aPFC0880c_1	PFC0880c	-0.29143	-0.10389	-0.31873	-0.18611	0.333053
aPFC0880c_0	PFC0880c	-0.29143	-0.10389	-0.31873	-0.18611	0.333053
intPFC0885c_2	PFC0885c	0.545627	0.780887	0.058727	-0.39439	-0.36055
aPFC0885c_0	PFC0885c	0.545627	0.780887	0.058727	-0.39439	-0.36055
intPFC0886w_2	PFC0886w	-0.86781	-1.76091	-0.07917	1.282507	1.184247
aPFC0886w_0	PFC0886w	-0.86781	-1.76091	-0.07917	1.282507	1.184247
intPFC0895w_2	PFC0890w	-0.32795	-0.33673	0.09821	0.28229	0.12823
intPFC0890w_1	PFC0890w	-0.13339	-0.04505	-0.05639	0.169727	0.372387
aPFC0890w_0	PFC0890w	-0.13339	-0.04505	-0.05639	0.169727	0.372387

aPFC0895w_1	PFC0895w	-0.32795	-0.33673	0.09821	0.28229	0.12823
aPFC0895w_0	PFC0895w	-0.32795	-0.33673	0.09821	0.28229	0.12823
intPFC0900w_2	PFC0900w	-0.41302	0.115627	1.093747	0.826087	-0.55129
aPFC0900w_1	PFC0900w	-0.41302	0.115627	1.093747	0.826087	-0.55129
aPFC0900w_0	PFC0900w	-0.41302	0.115627	1.093747	0.826087	-0.55129
intPFC0905c_2	PFC0905c	0.643255	0.201295	-0.30397	-0.40912	-0.13663
aPFC0905c_4	PFC0905c	0.643255	0.201295	-0.30397	-0.40912	-0.13663
aPFC0905c_3	PFC0905c	0.643255	0.201295	-0.30397	-0.40912	-0.13663
aPFC0905c_2	PFC0905c	0.643255	0.201295	-0.30397	-0.40912	-0.13663
aPFC0905c_1	PFC0905c	0.643255	0.201295	-0.30397	-0.40912	-0.13663
aPFC0905c_0	PFC0905c	0.643255	0.201295	-0.30397	-0.40912	-0.13663
intPFC0910w_2	PFC0910w	-1.10846	-1.03318	0.830607	0.977727	-0.37548
aPFC0910w_1	PFC0910w	-1.10846	-1.03318	0.830607	0.977727	-0.37548
aPFC0910w_0	PFC0910w	-1.10846	-1.03318	0.830607	0.977727	-0.37548
intPFC0912w_2	PFC0911c	-1.15972	-1.83943	-0.26965	1.122313	1.338093
aPFC0911c_0	PFC0911c	-0.65612	-1.1784	-0.06319	0.800527	0.749737
intPFC0911c_2	PFC0912w	-0.65612	-1.1784	-0.06319	0.800527	0.749737
aPFC0912w_0	PFC0912w	-1.15972	-1.83943	-0.26965	1.122313	1.338093
intPFC0915w_2	PFC0915w	-1.30534	-0.90077	0.544047	1.053287	0.417107
aPFC0915w_1	PFC0915w	-1.30534	-0.90077	0.544047	1.053287	0.417107
aPFC0915w_0	PFC0915w	-1.30534	-0.90077	0.544047	1.053287	0.417107
intPFC0920w_2	PFC0920w	-0.18787	-0.78066	-0.84469	0.004353	1.097323
aPFC0920w_0	PFC0920w	-0.18787	-0.78066	-0.84469	0.004353	1.097323
intPFC0925w_2	PFC0925w	0.133953	0.175223	0.250043	0.062123	-0.29306
aPFC0925w_1	PFC0925w	0.133953	0.175223	0.250043	0.062123	-0.29306
aPFC0925w_0	PFC0925w	0.133953	0.175223	0.250043	0.062123	-0.29306
intPFC0930c_2	PFC0930c	-0.18749	-0.40686	-1.72778	-0.90489	1.60153
aPFC0930c_2	PFC0930c	-0.18749	-0.40686	-1.72778	-0.90489	1.60153
aPFC0930c_1	PFC0930c	-0.18749	-0.40686	-1.72778	-0.90489	1.60153
aPFC0930c_0	PFC0930c	-0.18749	-0.40686	-1.72778	-0.90489	1.60153
intPFC0935c_2	PFC0935c	-1.07668	-0.61311	0.638213	0.862453	0.073773
aPFC0935c_1	PFC0935c	-1.07668	-0.61311	0.638213	0.862453	0.073773
aPFC0935c_0	PFC0935c	-1.07668	-0.61311	0.638213	0.862453	0.073773
intPFC0940c_2	PFC0940c	-0.58298	-0.59664	-0.72548	-0.25286	0.747303
aPFC0940c_1	PFC0940c	-0.58298	-0.59664	-0.72548	-0.25286	0.747303
aPFC0940c_0	PFC0940c	-0.58298	-0.59664	-0.72548	-0.25286	0.747303
intPFC0945w_2	PFC0945w	0.256017	-0.09406	-1.373	-1.2303	0.523677
aPFC0945w_0	PFC0945w	0.256017	-0.09406	-1.373	-1.2303	0.523677
intPFC0955w_2	PFC0950c	-1.16158	-0.62762	0.19175	0.68325	0.64212
aPFC0950c_1	PFC0950c	-1.28866	-1.05771	0.269413	0.997253	0.726373
aPFC0950c_0	PFC0950c	-1.28866	-1.05771	0.269413	0.997253	0.726373
intPFC0950c_2	PFC0955w	-1.28866	-1.05771	0.269413	0.997253	0.726373
aPFC0955w_1	PFC0955w	-1.16158	-0.62762	0.19175	0.68325	0.64212
aPFC0955w_0	PFC0955w	-1.16158	-0.62762	0.19175	0.68325	0.64212
intPFC0960c_2	PFC0960c	-0.61302	-0.99372	-1.17563	0.001137	1.698887
aPFC0960c_2	PFC0960c	-0.61302	-0.99372	-1.17563	0.001137	1.698887
aPFC0960c_1	PFC0960c	-0.61302	-0.99372	-1.17563	0.001137	1.698887

aPFC0960c_0	PFC0960c	-0.61302	-0.99372	-1.17563	0.001137	1.698887
intPFC0965w_1	PFC0965w	0.377327	0.278347	-0.02349	-0.42457	-0.53401
aPFC0965w_4	PFC0965w	0.377327	0.278347	-0.02349	-0.42457	-0.53401
aPFC0965w_3	PFC0965w	0.377327	0.278347	-0.02349	-0.42457	-0.53401
aPFC0965w_2	PFC0965w	0.377327	0.278347	-0.02349	-0.42457	-0.53401
aPFC0965w_1	PFC0965w	0.377327	0.278347	-0.02349	-0.42457	-0.53401
aPFC0965w_0	PFC0965w	0.377327	0.278347	-0.02349	-0.42457	-0.53401
intPFC0970w_2	PFC0970w	0.434923	0.584393	-0.2138	-0.47654	-0.07823
aPFC0970w_1	PFC0970w	0.434923	0.584393	-0.2138	-0.47654	-0.07823
aPFC0970w_0	PFC0970w	0.434923	0.584393	-0.2138	-0.47654	-0.07823
intPFC0975c_2	PFC0975c	-0.30462	-0.02253	0.870907	0.869867	-0.20535
aPFC0975c_0	PFC0975c	-0.30462	-0.02253	0.870907	0.869867	-0.20535
aPFC0980c_1	PFC0980c	0.550203	0.527993	0.226253	-0.11409	-0.41733
aPFC0980c_0	PFC0980c	0.550203	0.527993	0.226253	-0.11409	-0.41733
intPFC0990c_2	PFC0990c	-0.14274	-0.33531	-0.48388	-0.11146	0.543973
intPFC0980c_2	PFC0990c	0.550203	0.527993	0.226253	-0.11409	-0.41733
aPFC0990c_1	PFC0990c	-0.14274	-0.33531	-0.48388	-0.11146	0.543973
aPFC0990c_0	PFC0990c	-0.14274	-0.33531	-0.48388	-0.11146	0.543973
intPFC0995c_2	PFC0995c	-1.21991	-1.20892	-0.51489	0.730727	1.640757
aPFC0995c_1	PFC0995c	-1.21991	-1.20892	-0.51489	0.730727	1.640757
aPFC0995c_0	PFC0995c	-1.21991	-1.20892	-0.51489	0.730727	1.640757
atR_R-Ile_0	PFC10_API0001:tR	0.63362	0.6475	0.30504	-0.14534	-0.5568
aSSU_rR_1	PFC10_API0002:rR	0.558043	-0.55021	-1.46884	-0.77734	0.974043
aSSU_rR_0	PFC10_API0002:rR	0.558043	-0.55021	-1.46884	-0.77734	0.974043
atR_R-Ala_0	PFC10_API0003:tR	-0.54685	-0.79073	-0.49453	0.416867	1.187067
atR_R-Asn_0	PFC10_API0004:tR	0.73546	-0.20532	-0.66086	-0.30814	0.36732
atR_R-Leu_0	PFC10_API0005:tR	-0.13313	-0.61937	-0.42677	0.029433	0.458033
atR_R-Arg2_0	PFC10_API0006:tR	0.809993	0.326043	-0.23858	-0.50276	-0.36938
atR_R-Val_0	PFC10_API0007:tR					
atR_R-Arg1_0	PFC10_API0008:tR	0.231412	-0.15591	-0.47626	-0.13651	0.487292
atR_R-Met_0	PFC10_API0009:tR	-1.81138	-2.22412	-1.04227	1.023933	2.567783
aLSU_rR_1	PFC10_API0010:rR	0.62517	-0.09083	-1.27808	-0.97592	0.61033
aLSU_rR_0	PFC10_API0010:rR	0.62517	-0.09083	-1.27808	-0.97592	0.61033
aORF470_1	PFC10_API0012	-0.66791	-0.61796	-0.47034	0.16034	0.91346
aORF470_0	PFC10_API0012	-0.66791	-0.61796	-0.47034	0.16034	0.91346
arpoB_1	PFC10_API0015	-0.88988	-0.99906	-0.68312	0.329957	1.377897
arpoB_0	PFC10_API0015	-0.88988	-0.99906	-0.68312	0.329957	1.377897
arpoC_1	PFC10_API0016	-0.29442	-0.48591	-0.40969	0.113853	0.696073
arpoC_0	PFC10_API0016	-0.29442	-0.48591	-0.40969	0.113853	0.696073
arps2_0	PFC10_API0018	0.158323	-0.26763	-0.61464	-0.1525	0.666893
atR_R-Gly1_0	PFC10_API0021:tR	-1.09999	-1.24604	-0.24942	0.654757	0.975377
atR_R-Trp_0	PFC10_API0023:tR	-0.37082	-1.0639	-0.69622	0.203883	0.996563
atR_R-Gly2_0	PFC10_API0024:tR	0.320213	0.576953	-0.2809	-0.65144	-0.18429
atR_R-Gln_0	PFC10_API0025:tR	-0.4111	-0.80253	-0.42696	0.167623	0.636193
atufA_1	PFC10_API0028	-0.51268	-0.53911	-1.09129	-0.33871	1.30411
atufA_0	PFC10_API0028	-0.51268	-0.53911	-1.09129	-0.33871	1.30411
arps7_0	PFC10_API0029	-0.74302	-0.09294	-0.96162	-0.53672	1.143603

arps12_0	PFC10_API0030	-0.44618	-1.10509	-1.11333	-0.07267	1.34409
arps5_0	PFC10_API0033	-0.94996	-0.8092	-0.73611	0.088773	1.259863
arpl6_0	PFC10_API0034	-0.9241	-0.42753	-0.84546	-0.20388	1.267193
arps8_0	PFC10_API0035	-0.74565	-0.84879	-0.35393	0.410593	0.947453
arpl14_0	PFC10_API0036	-0.49671	-0.36368	-0.79512	-0.27854	0.963025
arps17_0	PFC10_API0037	-0.45891	-0.59526	-0.627	0.052118	0.983828
arpl16_0	PFC10_API0038	-0.62774	-0.71429	-0.81742	-0.05158	1.15429
arps3_0	PFC10_API0039	-0.89746	-1.05568	-0.71314	0.305477	1.361017
arps19_0	PFC10_API0040	-0.18961	-0.24287	-0.20334	0.077277	0.390007
arpl2_0	PFC10_API0041	-0.92501	-1.15716	-0.69329	0.589887	1.692757
arpl4_0	PFC10_API0043	-0.93183	-1.08886	-0.5413	0.476963	1.292523
atRR-Pro_0	PFC10_API0044:tR	-0.80966	-1.2707	-0.49136	0.621523	1.255863
atRR-Glu_0	PFC10_API0045:tR	0.147278	0.057618	-0.44765	-0.34581	0.291308
atRR-Lys_0	PFC10_API0046:tR					
atRR-Asp_0	PFC10_API0047:tR	1.74167	-0.08035	-0.28079	-0.31021	-0.58465
atRR-Ser_0	PFC10_API0048:tR					
atRR-Tyr_0	PFC10_API0049:tR	0.612813	0.124913	0.096673	-0.16801	-0.55025
atRR-Met_0	PFC10_API0050:tR					
atRR-Cys_0	PFC10_API0053:tR	0.11075	-0.70975	-0.349	0.194	0.42075
atRR-His_0	PFC10_API0054:tR					
arps4_0	PFC10_API0055					
arps11_0	PFC10_API0059	-0.81982	-0.5069	-0.94672	-0.22362	1.335563
aClp_1	PFC10_API0060	-0.87183	-1.14835	-0.76582	0.257983	1.316013
aClp_0	PFC10_API0060	-0.87183	-1.14835	-0.76582	0.257983	1.316013
intPFC1000w_2	PFC1000w	0.599307	1.102477	0.634307	-0.25397	-1.00864
aPFC1000w_1	PFC1000w	0.599307	1.102477	0.634307	-0.25397	-1.00864
aPFC1000w_0	PFC1000w	0.599307	1.102477	0.634307	-0.25397	-1.00864
aPFC1005c_1	PFC1005c	-0.38621	0.605023	-0.2248	-0.46104	0.252643
aPFC1005c_0	PFC1005c	-0.38621	0.605023	-0.2248	-0.46104	0.252643
intPFC1010w_2	PFC1010w	0.098703	-0.34701	-0.4595	-0.08734	0.447173
intPFC1005c_2	PFC1010w	-0.38621	0.605023	-0.2248	-0.46104	0.252643
aPFC1010w_1	PFC1010w	0.098703	-0.34701	-0.4595	-0.08734	0.447173
aPFC1010w_0	PFC1010w	0.098703	-0.34701	-0.4595	-0.08734	0.447173
aPFC1011c_1	PFC1011c	0.198207	0.306447	0.032357	-0.08502	-0.04861
aPFC1011c_0	PFC1011c	0.198207	0.306447	0.032357	-0.08502	-0.04861
intPFC1016w_2	PFC1015c					
intPFC1015c_2	PFC1015c	0.507782	0.487352	0.003162	-0.23308	-0.19977
intPFC1011c_2	PFC1015c	0.198207	0.306447	0.032357	-0.08502	-0.04861
aPFC1015c_3	PFC1015c	0.507782	0.487352	0.003162	-0.23308	-0.19977
aPFC1015c_2	PFC1015c	0.507782	0.487352	0.003162	-0.23308	-0.19977
aPFC1015c_1	PFC1015c	0.507782	0.487352	0.003162	-0.23308	-0.19977
aPFC1015c_0	PFC1015c	0.507782	0.487352	0.003162	-0.23308	-0.19977
aPFC1016w_0	PFC1016w					
intPFC1020c_2	PFC1020c	-0.14884	0.319163	0.752663	0.418003	-0.4905
aPFC1020c_0	PFC1020c	-0.14884	0.319163	0.752663	0.418003	-0.4905
intPFC1025w_2	PFC1025w	-0.81969	-0.04752	0.655557	0.778777	0.207857
aPFC1025w_0	PFC1025w	-0.81969	-0.04752	0.655557	0.778777	0.207857

intPFC1030w_2	PFC1030w	-0.34539	0.2173	0.48231	0.28819	-0.19197
aPFC1030w_2	PFC1030w	-0.34539	0.2173	0.48231	0.28819	-0.19197
aPFC1030w_1	PFC1030w	-0.34539	0.2173	0.48231	0.28819	-0.19197
aPFC1030w_0	PFC1030w	-0.34539	0.2173	0.48231	0.28819	-0.19197
intPFC1040w_2	PFC1035w	-0.01324	-0.31634	-0.25132	0.126483	0.444003
intPFC1035w_2	PFC1035w	-0.16409	-0.67806	-0.02562	0.54262	0.46356
aPFC1035w_1	PFC1035w	-0.16409	-0.67806	-0.02562	0.54262	0.46356
aPFC1035w_0	PFC1035w	-0.16409	-0.67806	-0.02562	0.54262	0.46356
aPFC1040w_1	PFC1040w	-0.01324	-0.31634	-0.25132	0.126483	0.444003
aPFC1040w_0	PFC1040w	-0.01324	-0.31634	-0.25132	0.126483	0.444003
intPFC1045c_2	PFC1045c	1.39251	0.38244	-1.66688	-2.40812	-0.76544
aPFC1045c_1	PFC1045c	1.39251	0.38244	-1.66688	-2.40812	-0.76544
aPFC1045c_0	PFC1045c	1.39251	0.38244	-1.66688	-2.40812	-0.76544
intPFC1050w_2	PFC1050w	0.48007	0.32266	-0.30383	-0.86517	-0.68566
aPFC1050w_0	PFC1050w	0.48007	0.32266	-0.30383	-0.86517	-0.68566
intPFC1055w_3	PFC1055w	0.209253	0.643233	0.417793	-0.03613	-0.46107
aPFC1055w_1	PFC1055w	0.209253	0.643233	0.417793	-0.03613	-0.46107
aPFC1055w_0	PFC1055w	0.209253	0.643233	0.417793	-0.03613	-0.46107
intPFC1060c_2	PFC1060c	0.403	0.17352	-0.20029	-0.30471	-0.10852
aPFC1060c_1	PFC1060c	0.403	0.17352	-0.20029	-0.30471	-0.10852
aPFC1060c_0	PFC1060c	0.403	0.17352	-0.20029	-0.30471	-0.10852
intPFC1065w_1	PFC1065w	0.53966	0.8637	0.19286	-0.37136	-0.5147
aPFC1065w_1	PFC1065w	0.53966	0.8637	0.19286	-0.37136	-0.5147
aPFC1065w_0	PFC1065w	0.53966	0.8637	0.19286	-0.37136	-0.5147
intPFC1075w_2	PFC1075w	0.40044	0.90478	-0.09406	-0.43994	-0.01778
intPFC1080c_2	PFC1080c	0.215417	0.698917	1.301167	0.419167	-1.36258
intPFC1085c_2	PFC1085c	1.358167	1.883147	0.524707	-1.19237	-1.94781
intPFC1090w_2	PFC1090w	1.871802	1.558032	0.649962	-0.96033	-2.21735
intPFD0075w_2	PFD0075w	0.134857	-0.05979	1.461087	0.927747	-1.37187
aPFD0075w_1	PFD0075w	0.134857	-0.05979	1.461087	0.927747	-1.37187
aPFD0075w_0	PFD0075w	0.134857	-0.05979	1.461087	0.927747	-1.37187
intPFD0080c_2	PFD0080c	-0.44458	-0.00858	1.77117	0.83483	-1.85242
aPFD0080c_1	PFD0080c	-0.44458	-0.00858	1.77117	0.83483	-1.85242
aPFD0080c_0	PFD0080c	-0.44458	-0.00858	1.77117	0.83483	-1.85242
intPFD0085c_2	PFD0085c	1.75972	1.22192	-0.34134	-1.46116	-1.33792
aPFD0085c_1	PFD0085c	1.75972	1.22192	-0.34134	-1.46116	-1.33792
aPFD0085c_0	PFD0085c	1.75972	1.22192	-0.34134	-1.46116	-1.33792
intPFD0090c_2	PFD0090c	-2.47545	-0.99978	2.005553	2.155113	-0.28556
aPFD0090c_0	PFD0090c	-2.47545	-0.99978	2.005553	2.155113	-0.28556
intPFD0095c_3	PFD0095c	1.676897	1.962537	-0.07707	-2.29259	-2.5482
aPFD0095c_1	PFD0095c	1.676897	1.962537	-0.07707	-2.29259	-2.5482
aPFD0095c_0	PFD0095c	1.676897	1.962537	-0.07707	-2.29259	-2.5482
intPFD0105c_2	PFD0100c	-1.14745	1.17423	-1.58346	-2.33104	0.57177
intPFD0100c_2	PFD0100c	1.30462	0.4354	-2.11356	-2.59044	-0.1554
aPFD0105c_1	PFD0100c	-1.14745	1.17423	-1.58346	-2.33104	0.57177
aPFD0105c_0	PFD0100c	-1.14745	1.17423	-1.58346	-2.33104	0.57177
aPFD0100c_1	PFD0100c	1.30462	0.4354	-2.11356	-2.59044	-0.1554

aPFD0100c_0	PFD0100c	1.30462	0.4354	-2.11356	-2.59044	-0.1554
aPFD0110w_4	PFD0110w	1.410703	0.123503	-1.69352	-2.30582	-0.74184
aPFD0110w_3	PFD0110w	1.410703	0.123503	-1.69352	-2.30582	-0.74184
aPFD0110w_2	PFD0110w	1.410703	0.123503	-1.69352	-2.30582	-0.74184
aPFD0110w_1	PFD0110w	1.410703	0.123503	-1.69352	-2.30582	-0.74184
aPFD0110w_0	PFD0110w	1.410703	0.123503	-1.69352	-2.30582	-0.74184
intPFD0115c_3	PFD0115c	-0.01987	-0.05084	0.376952	0.184232	-0.44198
aPFD0115c_0	PFD0115c	-0.01987	-0.05084	0.376952	0.184232	-0.44198
aPFD0135w_2	PFD0140w	-0.05152	0.435603	1.038303	0.437363	-0.93994
aPFD0135w_1	PFD0140w	-0.05152	0.435603	1.038303	0.437363	-0.93994
aPFD0135w_0	PFD0140w	-0.05152	0.435603	1.038303	0.437363	-0.93994
intPFD0144c_3	PFD0144c	2.187407	0.791977	-0.78945	-1.98121	-1.72564
aPFD0144c_0	PFD0144c	2.187407	0.791977	-0.78945	-1.98121	-1.72564
aPFD0145c_1	PFD0145c	-0.20965	0.012022	-0.05152	0.068202	0.274662
aPFD0145c_0	PFD0145c	-0.20965	0.012022	-0.05152	0.068202	0.274662
intPFD0150w_2	PFD0150w	0.850373	0.520133	-0.22927	-0.44007	-0.17647
intPFD0145c_2	PFD0150w	-0.20965	0.012022	-0.05152	0.068202	0.274662
aPFD0150w_0	PFD0150w	0.850373	0.520133	-0.22927	-0.44007	-0.17647
intPFD0155c_3	PFD0155c	0.46141	0.21994	0.14513	-0.02513	-0.31094
aPFD0155c_0	PFD0155c	0.46141	0.21994	0.14513	-0.02513	-0.31094
intPFD0160w_3	PFD0160w	0.752165	0.365005	-0.20118	-0.47222	-0.3469
aPFD0160w_5	PFD0160w	0.752165	0.365005	-0.20118	-0.47222	-0.3469
aPFD0160w_4	PFD0160w	0.752165	0.365005	-0.20118	-0.47222	-0.3469
aPFD0160w_3	PFD0160w	0.752165	0.365005	-0.20118	-0.47222	-0.3469
aPFD0160w_2	PFD0160w	0.752165	0.365005	-0.20118	-0.47222	-0.3469
aPFD0160w_1	PFD0160w	0.752165	0.365005	-0.20118	-0.47222	-0.3469
aPFD0160w_0	PFD0160w	0.752165	0.365005	-0.20118	-0.47222	-0.3469
intPFD0165w_2	PFD0165w	-0.02817	0.182357	0.169047	0.026287	-0.13952
aPFD0165w_1	PFD0165w	-0.02817	0.182357	0.169047	0.026287	-0.13952
aPFD0165w_0	PFD0165w	-0.02817	0.182357	0.169047	0.026287	-0.13952
intPFD0170c_2	PFD0170c	-1.2702	-1.431	-0.38476	0.920923	1.556163
aPFD0175c_0	PFD0170c	-0.83885	-1.05775	-0.23649	0.70649	1.06775
aPFD0170c_1	PFD0170c	-1.2702	-1.431	-0.38476	0.920923	1.556163
aPFD0170c_0	PFD0170c	-1.2702	-1.431	-0.38476	0.920923	1.556163
intPFD0180c_3	PFD0180c	0.434203	0.356483	0.377023	0.006643	-0.58832
aPFD0180c_1	PFD0180c	0.434203	0.356483	0.377023	0.006643	-0.58832
aPFD0180c_0	PFD0180c	0.434203	0.356483	0.377023	0.006643	-0.58832
intPFD0185c_2	PFD0185c	0.935597	-0.00846	-1.34281	-1.53135	-0.1527
aPFD0185c_1	PFD0185c	0.935597	-0.00846	-1.34281	-1.53135	-0.1527
aPFD0185c_0	PFD0185c	0.935597	-0.00846	-1.34281	-1.53135	-0.1527
intPFD0190w_2	PFD0190w	0.406893	-0.68048	-1.5713	-0.77154	1.134143
aPFD0190w_1	PFD0190w	0.406893	-0.68048	-1.5713	-0.77154	1.134143
aPFD0190w_0	PFD0190w	0.406893	-0.68048	-1.5713	-0.77154	1.134143
aPFD0195c_0	PFD0195c	1.22972	0.28041	-1.71932	-2.01168	-0.08841
intPFD0195c_2	PFD0200c	1.22972	0.28041	-1.71932	-2.01168	-0.08841
aPFD0200c_3	PFD0200c	-0.18162	0.029088	-0.52155	-0.27703	0.620578
aPFD0200c_2	PFD0200c	-0.18162	0.029088	-0.52155	-0.27703	0.620578

aPFD0200c_1	PFD0200c	-0.18162	0.029088	-0.52155	-0.27703	0.620578
aPFD0200c_0	PFD0200c	-0.18162	0.029088	-0.52155	-0.27703	0.620578
intPFD0200c_2	PFD0205c	-0.18162	0.029088	-0.52155	-0.27703	0.620578
aPFD0205c_0	PFD0205c					
intPFD0207c_2	PFD0207c	0.632913	0.511423	-0.02611	-0.27773	-0.23876
intPFD0205c_2	PFD0207c					
aPFD0207c_1	PFD0207c	0.632913	0.511423	-0.02611	-0.27773	-0.23876
aPFD0207c_0	PFD0207c	0.632913	0.511423	-0.02611	-0.27773	-0.23876
intPFD0210c_2	PFD0210c	0.09481	0.24992	0.09835	-0.21932	-0.37427
aPFD0210c_0	PFD0210c	0.09481	0.24992	0.09835	-0.21932	-0.37427
intPFD0215c_2	PFD0215c	0.349443	0.627283	0.392423	-0.24826	-0.79012
aPFD0215c_1	PFD0215c	0.349443	0.627283	0.392423	-0.24826	-0.79012
aPFD0215c_0	PFD0215c	0.349443	0.627283	0.392423	-0.24826	-0.79012
intPFD0225w_2	PFD0225w	-0.77122	0.67268	0.85236	0.28067	-0.43614
aPFD0225w_6	PFD0225w	-0.77122	0.67268	0.85236	0.28067	-0.43614
aPFD0225w_5	PFD0225w	-0.77122	0.67268	0.85236	0.28067	-0.43614
aPFD0225w_4	PFD0225w	-0.77122	0.67268	0.85236	0.28067	-0.43614
aPFD0225w_3	PFD0225w	-0.77122	0.67268	0.85236	0.28067	-0.43614
aPFD0225w_2	PFD0225w	-0.77122	0.67268	0.85236	0.28067	-0.43614
aPFD0225w_1	PFD0225w	-0.77122	0.67268	0.85236	0.28067	-0.43614
aPFD0225w_0	PFD0225w	-0.77122	0.67268	0.85236	0.28067	-0.43614
aPFD0230c_1	PFD0230c	-0.08393	-0.56336	-1.96454	-1.33046	1.25836
aPFD0230c_0	PFD0230c	-0.08393	-0.56336	-1.96454	-1.33046	1.25836
intPFD0235c_2	PFD0235c	0.439363	-0.2703	-0.73841	-0.68443	0.000463
intPFD0230c_2	PFD0235c	-0.08393	-0.56336	-1.96454	-1.33046	1.25836
aPFD0235c_1	PFD0235c	0.439363	-0.2703	-0.73841	-0.68443	0.000463
aPFD0235c_0	PFD0235c	0.439363	-0.2703	-0.73841	-0.68443	0.000463
aPFD0240c_1	PFD0240c	-0.55185	-1.10976	-1.48122	-0.27028	1.75426
aPFD0240c_0	PFD0240c	-0.55185	-1.10976	-1.48122	-0.27028	1.75426
intPFD0245c_2	PFD0245c	0.393483	0.456573	0.657113	0.039053	-0.97291
intPFD0240c_2	PFD0245c	-0.55185	-1.10976	-1.48122	-0.27028	1.75426
aPFD0245c_1	PFD0245c	0.393483	0.456573	0.657113	0.039053	-0.97291
aPFD0245c_0	PFD0245c	0.393483	0.456573	0.657113	0.039053	-0.97291
intPFD0250c_3	PFD0250c	-0.59353	-0.01171	-0.27927	-0.01918	0.673255
aPFD0250c_2	PFD0250c	-0.59353	-0.01171	-0.27927	-0.01918	0.673255
aPFD0250c_1	PFD0250c	-0.59353	-0.01171	-0.27927	-0.01918	0.673255
aPFD0250c_0	PFD0250c	-0.59353	-0.01171	-0.27927	-0.01918	0.673255
intPFD0255w_2	PFD0255w	-0.39408	-0.81811	-1.1068	-0.44454	0.948273
aPFD0255w_1	PFD0255w	-0.39408	-0.81811	-1.1068	-0.44454	0.948273
aPFD0255w_0	PFD0255w	-0.39408	-0.81811	-1.1068	-0.44454	0.948273
intPFD0260c_2	PFD0260c	0.27619	0.71407	-0.03163	-0.38737	-0.13907
aPFD0260c_2	PFD0260c	0.27619	0.71407	-0.03163	-0.38737	-0.13907
aPFD0260c_1	PFD0260c	0.27619	0.71407	-0.03163	-0.38737	-0.13907
aPFD0260c_0	PFD0260c	0.27619	0.71407	-0.03163	-0.38737	-0.13907
intPFD0265w_3	PFD0265w	0.053013	0.321343	0.335113	0.094353	-0.27628
aPFD0265w_4	PFD0265w	0.053013	0.321343	0.335113	0.094353	-0.27628
aPFD0265w_3	PFD0265w	0.053013	0.321343	0.335113	0.094353	-0.27628

aPFD0265w_2	PFD0265w	0.053013	0.321343	0.335113	0.094353	-0.27628
aPFD0265w_1	PFD0265w	0.053013	0.321343	0.335113	0.094353	-0.27628
aPFD0265w_0	PFD0265w	0.053013	0.321343	0.335113	0.094353	-0.27628
intPFD0275w_2	PFD0270c	0.14216	0.63021	0.34409	-0.12409	-0.44421
aPFD0270c_0	PFD0270c	0.97678	0.41111	0.39078	0.13122	-0.57111
intPFD0270c_2	PFD0275w	0.97678	0.41111	0.39078	0.13122	-0.57111
aPFD0280w_0	PFD0275w	-0.25036	-0.12817	0.533847	0.635487	-0.02749
aPFD0275w_0	PFD0275w	0.14216	0.63021	0.34409	-0.12409	-0.44421
intPFD0285c_2	PFD0285c	-1.14498	-0.1491	1.21103	0.90153	-0.58568
aPFD0285c_3	PFD0285c	-1.14498	-0.1491	1.21103	0.90153	-0.58568
aPFD0285c_2	PFD0285c	-1.14498	-0.1491	1.21103	0.90153	-0.58568
aPFD0285c_1	PFD0285c	-1.14498	-0.1491	1.21103	0.90153	-0.58568
aPFD0285c_0	PFD0285c	-1.14498	-0.1491	1.21103	0.90153	-0.58568
intPFD0290w_2	PFD0290w	-0.07279	-1.05903	-0.11043	0.855767	0.804367
aPFD0290w_0	PFD0290w	-0.07279	-1.05903	-0.11043	0.855767	0.804367
intPFD0295c_2	PFD0295c	-0.02502	-0.45636	-2.27402	-1.56064	1.532197
aPFD0295c_1	PFD0295c	-0.02502	-0.45636	-2.27402	-1.56064	1.532197
aPFD0295c_0	PFD0295c	-0.02502	-0.45636	-2.27402	-1.56064	1.532197
intPFD0300w_2	PFD0300w	-0.19909	-0.23208	-0.05261	-0.01923	-0.02076
aPFD0300w_1	PFD0300w	-0.19909	-0.23208	-0.05261	-0.01923	-0.02076
aPFD0300w_0	PFD0300w	-0.19909	-0.23208	-0.05261	-0.01923	-0.02076
aPFD0305c_1	PFD0305c	-0.78099	-0.04953	0.585057	0.674777	0.166867
aPFD0305c_0	PFD0305c	-0.78099	-0.04953	0.585057	0.674777	0.166867
intPFD0310w_3	PFD0310w	-0.9029	-0.51152	0.60553	0.83847	0.10752
aPFD0310w_0	PFD0310w	-0.9029	-0.51152	0.60553	0.83847	0.10752
intPFD0311w_2	PFD0311w	-0.18164	0.287173	0.556653	0.160013	-0.52851
aPFD0315c_0	PFD0311w	0.607367	0.845177	0.151147	-0.36381	-0.46184
aPFD0311w_0	PFD0311w	-0.18164	0.287173	0.556653	0.160013	-0.52851
intPFD0320c_2	PFD0320c	0.065335	0.479005	-0.0123	-0.39006	-0.26499
aPFD0320c_5	PFD0320c	0.065335	0.479005	-0.0123	-0.39006	-0.26499
aPFD0320c_4	PFD0320c	0.065335	0.479005	-0.0123	-0.39006	-0.26499
aPFD0320c_3	PFD0320c	0.065335	0.479005	-0.0123	-0.39006	-0.26499
aPFD0320c_2	PFD0320c	0.065335	0.479005	-0.0123	-0.39006	-0.26499
aPFD0320c_1	PFD0320c	0.065335	0.479005	-0.0123	-0.39006	-0.26499
aPFD0320c_0	PFD0320c	0.065335	0.479005	-0.0123	-0.39006	-0.26499
intPFD0330w_2	PFD0325w	0.456037	0.004307	-1.11162	-0.80754	0.677527
intPFD0325w_2	PFD0325w	0.005913	-0.66157	0.193123	0.583543	0.078233
aPFD0325w_0	PFD0325w	0.005913	-0.66157	0.193123	0.583543	0.078233
aPFD0330w_1	PFD0330w	0.456037	0.004307	-1.11162	-0.80754	0.677527
aPFD0330w_0	PFD0330w	0.456037	0.004307	-1.11162	-0.80754	0.677527
intPFD0335c_2	PFD0335c	-1.06684	-1.0318	0.427583	1.181083	0.655463
aPFD0335c_0	PFD0335c	-1.06684	-1.0318	0.427583	1.181083	0.655463
aPFD0340c_2	PFD0340c	-0.09627	-0.28017	-0.2626	0.033642	0.377212
aPFD0340c_1	PFD0340c	-0.09627	-0.28017	-0.2626	0.033642	0.377212
aPFD0340c_0	PFD0340c	-0.09627	-0.28017	-0.2626	0.033642	0.377212
intPFD0350w_2	PFD0345c	0.558203	0.109963	0.046063	-0.0854	-0.3253
intPFD0340c_2	PFD0345c	-0.09627	-0.28017	-0.2626	0.033642	0.377212

aPFD0345c_1	PFD0345c	1.62469	1.31606	0.33663	-0.85313	-1.56106
aPFD0345c_0	PFD0345c	1.62469	1.31606	0.33663	-0.85313	-1.56106
intPFD0345c_2	PFD0350w	1.62469	1.31606	0.33663	-0.85313	-1.56106
aPFD0350w_0	PFD0350w	0.558203	0.109963	0.046063	-0.0854	-0.3253
aPFD0355c_0	PFD0355c	0.709837	0.505817	0.383877	-0.21354	-0.92348
intPFD0360w_2	PFD0360w	1.88844	1.47682	0.71937	-0.71337	-2.03382
intPFD0355c_2	PFD0360w	0.709837	0.505817	0.383877	-0.21354	-0.92348
aPFD0360w_0	PFD0360w	1.88844	1.47682	0.71937	-0.71337	-2.03382
intPFD0365c_2	PFD0365c	0.0616	-0.12298	0.68295	0.65905	-0.36602
aPFD0365c_0	PFD0365c	0.0616	-0.12298	0.68295	0.65905	-0.36602
intPFD0367w_2	PFD0367w	0.088883	0.478573	0.326353	0.009313	-0.29491
aPFD0367w_1	PFD0367w	0.088883	0.478573	0.326353	0.009313	-0.29491
aPFD0367w_0	PFD0367w	0.088883	0.478573	0.326353	0.009313	-0.29491
intPFD0375w_2	PFD0375w	0.352327	0.330377	0.141257	-0.15592	-0.39004
aPFD0375w_1	PFD0375w	0.352327	0.330377	0.141257	-0.15592	-0.39004
aPFD0375w_0	PFD0375w	0.352327	0.330377	0.141257	-0.15592	-0.39004
intPFD0380c_2	PFD0380c	-0.16162	-0.5284	-1.50944	-0.85656	1.18407
aPFD0380c_2	PFD0380c	-0.16162	-0.5284	-1.50944	-0.85656	1.18407
aPFD0380c_1	PFD0380c	-0.16162	-0.5284	-1.50944	-0.85656	1.18407
aPFD0380c_0	PFD0380c	-0.16162	-0.5284	-1.50944	-0.85656	1.18407
intPFD0385w_2	PFD0385w	-0.36385	-0.83408	-1.70417	-0.79527	1.496638
aPFD0385w_2	PFD0385w	-0.36385	-0.83408	-1.70417	-0.79527	1.496638
aPFD0385w_1	PFD0385w	-0.36385	-0.83408	-1.70417	-0.79527	1.496638
aPFD0385w_0	PFD0385w	-0.36385	-0.83408	-1.70417	-0.79527	1.496638
aPFD0385c_1	PFD0390c	0.741487	-0.10742	-0.96091	-1.33475	-0.55924
aPFD0385c_0	PFD0390c	0.741487	-0.10742	-0.96091	-1.33475	-0.55924
intPFD0395c_2	PFD0395c	2.045727	0.599387	-0.98377	-2.00889	-1.49205
aPFD0395c_0	PFD0395c	2.045727	0.599387	-0.98377	-2.00889	-1.49205
intPFD0403w_2	PFD0400w	1.174398	0.478978	-0.74068	-1.09295	-0.35366
intPFD0400w_2	PFD0400w	1.091387	0.696057	-0.77536	-1.2533	-0.35822
aPFD0400w_1	PFD0400w	1.091387	0.696057	-0.77536	-1.2533	-0.35822
aPFD0400w_0	PFD0400w	1.091387	0.696057	-0.77536	-1.2533	-0.35822
aPFD0403w_1	PFD0403w	1.174398	0.478978	-0.74068	-1.09295	-0.35366
aPFD0403w_0	PFD0403w	1.174398	0.478978	-0.74068	-1.09295	-0.35366
intPFD0405c_2	PFD0405c	-0.05015	0.22875	0.66749	0.25251	-0.67575
aPFD0405c_0	PFD0405c	-0.05015	0.22875	0.66749	0.25251	-0.67575
intPFD0415c_2	PFD0415c	-0.31976	-0.08519	0.32538	0.39862	0.05119
aPFD0415c_1	PFD0415c	-0.31976	-0.08519	0.32538	0.39862	0.05119
aPFD0415c_0	PFD0415c	-0.31976	-0.08519	0.32538	0.39862	0.05119
intPFD0420c_2	PFD0420c	-0.91973	-1.21333	-0.58459	0.63359	1.51983
aPFD0420c_1	PFD0420c	-0.91973	-1.21333	-0.58459	0.63359	1.51983
aPFD0420c_0	PFD0420c	-0.91973	-1.21333	-0.58459	0.63359	1.51983
intPFD0425w_2	PFD0425w	0.56283	-0.06063	-0.275	-0.274	-0.13487
aPFD0425w_1	PFD0425w	0.56283	-0.06063	-0.275	-0.274	-0.13487
aPFD0425w_0	PFD0425w	0.56283	-0.06063	-0.275	-0.274	-0.13487
intPFD0430c_2	PFD0430c	0.5725	-0.01448	0.13696	0.13504	-0.24902
aPFD0430c_1	PFD0430c	0.5725	-0.01448	0.13696	0.13504	-0.24902

aPFD0430c_0	PFD0430c	0.5725	-0.01448	0.13696	0.13504	-0.24902
aPFD0435c_0	PFD0435c					
intPFD0440w_2	PFD0440w	-1.02526	-0.77867	0.263597	0.893457	0.679567
intPFD0435c_2	PFD0440w					
aPFD0440w_1	PFD0440w	-1.02526	-0.77867	0.263597	0.893457	0.679567
aPFD0440w_0	PFD0440w	-1.02526	-0.77867	0.263597	0.893457	0.679567
intPFD0445c_2	PFD0445c	-0.73741	-0.4552	-0.85284	0.007647	1.517347
aPFD0445c_3	PFD0445c	-0.73741	-0.4552	-0.85284	0.007647	1.517347
aPFD0445c_2	PFD0445c	-0.73741	-0.4552	-0.85284	0.007647	1.517347
aPFD0445c_1	PFD0445c	-0.73741	-0.4552	-0.85284	0.007647	1.517347
aPFD0445c_0	PFD0445c	-0.73741	-0.4552	-0.85284	0.007647	1.517347
intPFD0455w_2	PFD0450c	0.582227	0.323887	0.536227	0.005107	-0.95355
aPFD0450c_1	PFD0450c	0.269997	0.258517	0.343957	0.009377	-0.53418
aPFD0450c_0	PFD0450c	0.269997	0.258517	0.343957	0.009377	-0.53418
intPFD0450c_2	PFD0455w	0.269997	0.258517	0.343957	0.009377	-0.53418
aPFD0455w_1	PFD0455w	0.582227	0.323887	0.536227	0.005107	-0.95355
aPFD0455w_0	PFD0455w	0.582227	0.323887	0.536227	0.005107	-0.95355
intPFD0460c_3	PFD0460c	0.607117	0.560427	0.696397	-0.04706	-1.17609
aPFD0460c_1	PFD0460c	0.607117	0.560427	0.696397	-0.04706	-1.17609
aPFD0460c_0	PFD0460c	0.607117	0.560427	0.696397	-0.04706	-1.17609
intPFD0462w_2	PFD0462w	0.055877	0.300117	1.049017	0.492317	-1.00328
aPFD0462w_1	PFD0462w	0.055877	0.300117	1.049017	0.492317	-1.00328
aPFD0462w_0	PFD0462w	0.055877	0.300117	1.049017	0.492317	-1.00328
intPFD0465c_2	PFD0465c	-0.58309	-1.99121	-0.34793	1.282373	1.428313
aPFD0466c_0	PFD0465c	-0.78272	-1.39679	-0.17348	1.011098	1.164408
aPFD0465c_0	PFD0465c	-0.58309	-1.99121	-0.34793	1.282373	1.428313
aPFD0470c_1	PFD0470c	-1.29404	-0.67982	0.133393	0.643273	0.698483
aPFD0470c_0	PFD0470c	-1.29404	-0.67982	0.133393	0.643273	0.698483
intPFD0480w_3	PFD0480w	-0.49698	-0.66206	0.189117	0.487217	0.108397
intPFD0470c_2	PFD0480w	-1.29404	-0.67982	0.133393	0.643273	0.698483
aPFD0480w_0	PFD0480w	-0.49698	-0.66206	0.189117	0.487217	0.108397
intPFD0485w_2	PFD0485w	0.038397	0.327497	0.268257	-0.26292	-0.68066
aPFD0485w_1	PFD0485w	0.038397	0.327497	0.268257	-0.26292	-0.68066
aPFD0485w_0	PFD0485w	0.038397	0.327497	0.268257	-0.26292	-0.68066
aPFD0487c_1	PFD0487c	-0.32052	-0.0714	-0.6452	-0.4638	0.5464
aPFD0487c_0	PFD0487c	-0.32052	-0.0714	-0.6452	-0.4638	0.5464
intPFD0495c_2	PFD0495c	0.44759	0.34607	0.26035	-0.81785	-1.62257
intPFD0487c_2	PFD0495c	-0.32052	-0.0714	-0.6452	-0.4638	0.5464
aPFD0495c_1	PFD0495c	0.44759	0.34607	0.26035	-0.81785	-1.62257
aPFD0495c_0	PFD0495c	0.44759	0.34607	0.26035	-0.81785	-1.62257
aPFD0505c_1	PFD0505c	0.35769	0.49156	-0.05887	-0.25163	-0.06106
aPFD0505c_0	PFD0505c	0.35769	0.49156	-0.05887	-0.25163	-0.06106
intPFD0515w_2	PFD0515w	0.663223	0.740873	0.510763	-0.1251	-0.85121
intPFD0505c_2	PFD0515w	0.35769	0.49156	-0.05887	-0.25163	-0.06106
aPFD0515w_0	PFD0515w	0.663223	0.740873	0.510763	-0.1251	-0.85121
intPFD0520c_2	PFD0520c	0.824605	0.146515	-0.58523	-0.80091	-0.30565
aPFD0520c_0	PFD0520c	0.824605	0.146515	-0.58523	-0.80091	-0.30565

intPFD0525w_2	PFD0525w	0.133107	0.199467	0.784567	0.139767	-1.14013
aPFD0525w_0	PFD0525w	0.133107	0.199467	0.784567	0.139767	-1.14013
intPFD0530c_2	PFD0530c	-0.7864	-0.86499	-0.68978	0.00228	0.95349
aPFD0530c_1	PFD0530c	-0.7864	-0.86499	-0.68978	0.00228	0.95349
aPFD0530c_0	PFD0530c	-0.7864	-0.86499	-0.68978	0.00228	0.95349
intPFD0535w_2	PFD0535w	-1.0021	-0.67452	-0.55923	0.117393	1.085683
aPFD0535w_1	PFD0535w	-1.0021	-0.67452	-0.55923	0.117393	1.085683
aPFD0535w_0	PFD0535w	-1.0021	-0.67452	-0.55923	0.117393	1.085683
intPFD0540c_2	PFD0540c	0.136633	0.309343	0.347063	0.156603	-0.24068
aPFD0540c_1	PFD0540c	0.136633	0.309343	0.347063	0.156603	-0.24068
aPFD0540c_0	PFD0540c	0.136633	0.309343	0.347063	0.156603	-0.24068
intPFD0545w_2	PFD0545w	0.519065	1.962685	1.164135	-0.70814	-2.06102
aPFD0545w_2	PFD0545w	0.519065	1.962685	1.164135	-0.70814	-2.06102
aPFD0545w_1	PFD0545w	0.519065	1.962685	1.164135	-0.70814	-2.06102
aPFD0545w_0	PFD0545w	0.519065	1.962685	1.164135	-0.70814	-2.06102
aPFD0550c_0	PFD0550c	-0.08387	-0.15567	0.12885	-0.11885	-0.49033
intPFD0560w_2	PFD0555c	-0.80027	-0.09763	0.59776	0.68724	0.13663
intPFD0555c_2	PFD0555c	-0.55834	0.329707	0.912087	0.498247	-0.51887
intPFD0550c_2	PFD0555c	-0.08387	-0.15567	0.12885	-0.11885	-0.49033
aPFD0555c_1	PFD0555c	-0.55834	0.329707	0.912087	0.498247	-0.51887
aPFD0555c_0	PFD0555c	-0.55834	0.329707	0.912087	0.498247	-0.51887
aPFD0560w_1	PFD0560w	-0.80027	-0.09763	0.59776	0.68724	0.13663
aPFD0560w_0	PFD0560w	-0.80027	-0.09763	0.59776	0.68724	0.13663
intPFD0565c_2	PFD0565c	0.30451	0.75945	0.86085	0.14865	-0.95045
aPFD0565c_1	PFD0565c	0.30451	0.75945	0.86085	0.14865	-0.95045
aPFD0565c_0	PFD0565c	0.30451	0.75945	0.86085	0.14865	-0.95045
aPFD0580c_0	PFD0580c	-0.3432	-0.207	0.021017	0.176317	0.202337
intPFD0585c_2	PFD0585c	-0.95538	-0.74679	-0.50242	0.398642	1.339682
intPFD0580c_2	PFD0585c	-0.3432	-0.207	0.021017	0.176317	0.202337
aPFD0585c_2	PFD0585c	-0.95538	-0.74679	-0.50242	0.398642	1.339682
aPFD0585c_1	PFD0585c	-0.95538	-0.74679	-0.50242	0.398642	1.339682
aPFD0585c_0	PFD0585c	-0.95538	-0.74679	-0.50242	0.398642	1.339682
intPFD0590c_2	PFD0590c	-1.1686	-1.11267	-0.69599	0.52255	1.70756
aPFD0590c_2	PFD0590c	-1.1686	-1.11267	-0.69599	0.52255	1.70756
aPFD0590c_1	PFD0590c	-1.1686	-1.11267	-0.69599	0.52255	1.70756
aPFD0590c_0	PFD0590c	-1.1686	-1.11267	-0.69599	0.52255	1.70756
intPFD0595w_2	PFD0595w	-0.94149	-0.78505	-0.1489	0.6204	0.99405
aPFD0595w_1	PFD0595w	-0.94149	-0.78505	-0.1489	0.6204	0.99405
aPFD0595w_0	PFD0595w	-0.94149	-0.78505	-0.1489	0.6204	0.99405
aPFD0600c_0	PFD0600c	0.16918	-0.17191	0.11133	0.33467	0.12691
intPFD0610w_2	PFD0605c	0.308387	0.379547	0.006657	-0.07982	0.018287
intPFD0600c_2	PFD0605c	0.16918	-0.17191	0.11133	0.33467	0.12691
aPFD0605c_0	PFD0605c	1.388373	0.677133	0.218733	-0.32407	-0.89847
intPFD0605c_2	PFD0610w	1.388373	0.677133	0.218733	-0.32407	-0.89847
aPFD0610w_1	PFD0610w	0.308387	0.379547	0.006657	-0.07982	0.018287
aPFD0610w_0	PFD0610w	0.308387	0.379547	0.006657	-0.07982	0.018287
aPFD0650w_0	PFD0655w					

intPFD0660w_3	PFD0660w	-0.59516	-1.80023	-1.26155	0.497213	2.056893
aPFD0660w_0	PFD0660w	-0.59516	-1.80023	-1.26155	0.497213	2.056893
intPFD0665c_2	PFD0665c	-0.45702	-0.52092	-0.12241	0.429073	0.673083
aPFD0665c_1	PFD0665c	-0.45702	-0.52092	-0.12241	0.429073	0.673083
aPFD0665c_0	PFD0665c	-0.45702	-0.52092	-0.12241	0.429073	0.673083
intPFD0695w_2	PFD0669c					
aPFD0669c_0	PFD0669c	0.102383	0.105093	0.529313	0.415353	-0.31143
intPFD0675w_2	PFD0670c	-0.67895	-0.87621	-0.47756	0.459897	1.207547
intPFD0670c_3	PFD0670c	-1.04988	-1.28364	-0.13473	1.047393	1.312303
aPFD0670c_0	PFD0670c	-1.04988	-1.28364	-0.13473	1.047393	1.312303
aPFD0675w_0	PFD0675w	-0.67895	-0.87621	-0.47756	0.459897	1.207547
aPFD0655c_1	PFD0680c	0.00178	0.14058	-0.71566	-0.46184	0.71242
aPFD0655c_0	PFD0680c	0.00178	0.14058	-0.71566	-0.46184	0.71242
aPFD0685c_1	PFD0685c	-1.2511	-1.37602	-0.08095	1.07695	1.26352
aPFD0685c_0	PFD0685c	-1.2511	-1.37602	-0.08095	1.07695	1.26352
intPFD0690c_2	PFD0690c	-1.30668	-0.59197	1.005765	1.220125	0.027185
intPFD0685c_2	PFD0690c	-1.2511	-1.37602	-0.08095	1.07695	1.26352
aPFD0690c_2	PFD0690c	-1.30668	-0.59197	1.005765	1.220125	0.027185
aPFD0690c_1	PFD0690c	-1.30668	-0.59197	1.005765	1.220125	0.027185
aPFD0690c_0	PFD0690c	-1.30668	-0.59197	1.005765	1.220125	0.027185
intPFD0692c_2	PFD0692c	-0.85535	-1.23325	-0.72799	0.533327	1.597587
aPFD0692c_0	PFD0692c	-0.85535	-1.23325	-0.72799	0.533327	1.597587
intPFD0669c_2	PFD0695w	0.102383	0.105093	0.529313	0.415353	-0.31143
aPFD0695w_0	PFD0695w					
intPFD0700c_2	PFD0700c	0.871353	0.484233	0.115033	-0.40737	-0.78957
aPFD0700c_0	PFD0700c	0.871353	0.484233	0.115033	-0.40737	-0.78957
intPFD0710w_2	PFD0705c	-0.41463	-0.79984	-0.41481	0.092147	0.503677
aPFD0705c_0	PFD0705c	-0.79103	-0.99988	-0.67274	0.187403	1.111543
intPFD0705c_2	PFD0710w	-0.79103	-0.99988	-0.67274	0.187403	1.111543
aPFD0710w_1	PFD0710w	-0.41463	-0.79984	-0.41481	0.092147	0.503677
aPFD0710w_0	PFD0710w	-0.41463	-0.79984	-0.41481	0.092147	0.503677
intPFD0715c_2	PFD0715c	1.901353	0.416213	-1.81193	-2.49341	-0.77455
aPFD0715c_0	PFD0715c	1.901353	0.416213	-1.81193	-2.49341	-0.77455
intPFD0720w_2	PFD0720w	1.171	-0.12002	-1.66346	-1.99154	-0.40798
aPFD0720w_0	PFD0720w	1.171	-0.12002	-1.66346	-1.99154	-0.40798
aPFD0725c_1	PFD0725c	0.1474	0.38049	0.13553	-0.03003	-0.08899
aPFD0725c_0	PFD0725c	0.1474	0.38049	0.13553	-0.03003	-0.08899
intPFD0725c_2	PFD0730w	0.1474	0.38049	0.13553	-0.03003	-0.08899
aPFD0730w_1	PFD0730w	0.720777	0.982627	0.168487	-0.39815	-0.49129
aPFD0730w_0	PFD0730w	0.720777	0.982627	0.168487	-0.39815	-0.49129
intPFD0735c_2	PFD0735c	-0.54808	-0.82663	-0.45058	0.455463	1.140853
aPFD0735c_3	PFD0735c	-0.54808	-0.82663	-0.45058	0.455463	1.140853
aPFD0735c_2	PFD0735c	-0.54808	-0.82663	-0.45058	0.455463	1.140853
aPFD0735c_1	PFD0735c	-0.54808	-0.82663	-0.45058	0.455463	1.140853
aPFD0735c_0	PFD0735c	-0.54808	-0.82663	-0.45058	0.455463	1.140853
aPFD0711c_0	PFD0735c	-0.27001	-0.35698	-0.36454	0.294203	0.961643
intPFD0740w_2	PFD0740w	0.569017	0.429917	0.523657	-0.02132	-0.89508

aPFD0740w_1	PFD0740w	0.569017	0.429917	0.523657	-0.02132	-0.89508
aPFD0740w_0	PFD0740w	0.569017	0.429917	0.523657	-0.02132	-0.89508
intPFD0750w_2	PFD0745c	0.193947	0.174277	0.486447	0.186887	-0.55994
aPFD0745c_0	PFD0745c	-0.10167	0.031357	-0.1802	-0.09746	0.238977
intPFD0745c_2	PFD0750w	-0.10167	0.031357	-0.1802	-0.09746	0.238977
aPFD0750w_0	PFD0750w	0.193947	0.174277	0.486447	0.186887	-0.55994
intPFD0755c_2	PFD0755c	0.86599	0.36206	-0.13612	-0.77888	-0.96606
aPFD0755c_0	PFD0755c	0.86599	0.36206	-0.13612	-0.77888	-0.96606
intPFD0765w_2	PFD0760c	0.982713	0.261413	-0.62507	-1.10027	-0.65775
aPFD0760c_1	PFD0760c	-0.18766	-1.06071	-0.37134	0.35034	0.56171
aPFD0760c_0	PFD0760c	-0.18766	-1.06071	-0.37134	0.35034	0.56171
intPFD0760c_2	PFD0765w	-0.18766	-1.06071	-0.37134	0.35034	0.56171
aPFD0765w_1	PFD0765w	0.982713	0.261413	-0.62507	-1.10027	-0.65775
aPFD0765w_0	PFD0765w	0.982713	0.261413	-0.62507	-1.10027	-0.65775
intPFD0770c_3	PFD0770c	-0.17744	0.48118	0.93813	0.52387	-0.55418
aPFD0770c_0	PFD0770c	-0.17744	0.48118	0.93813	0.52387	-0.55418
intPFD0775c_2	PFD0775c	-0.07021	-0.80391	-0.08618	0.378517	0.213247
aPFD0775c_1	PFD0775c	-0.07021	-0.80391	-0.08618	0.378517	0.213247
aPFD0775c_0	PFD0775c	-0.07021	-0.80391	-0.08618	0.378517	0.213247
intPFD0780w_2	PFD0780w	-0.55551	-0.36296	-0.19408	0.160743	0.540123
aPFD0780w_1	PFD0780w	-0.55551	-0.36296	-0.19408	0.160743	0.540123
aPFD0780w_0	PFD0780w	-0.55551	-0.36296	-0.19408	0.160743	0.540123
intPFD0785c_2	PFD0785c	0.505643	0.739763	-0.09654	-0.6118	-0.4161
aPFD0785c_0	PFD0785c	0.505643	0.739763	-0.09654	-0.6118	-0.4161
intPFD0790c_2	PFD0790c	-0.60868	-0.74274	-0.56161	0.17489	0.99968
aPFD0790c_2	PFD0790c	-0.60868	-0.74274	-0.56161	0.17489	0.99968
aPFD0790c_1	PFD0790c	-0.60868	-0.74274	-0.56161	0.17489	0.99968
aPFD0790c_0	PFD0790c	-0.60868	-0.74274	-0.56161	0.17489	0.99968
intPFD0795w_2	PFD0795w	-1.65148	-0.75909	-0.43707	0.47757	1.56209
aPFD0795w_1	PFD0795w	-1.65148	-0.75909	-0.43707	0.47757	1.56209
aPFD0795w_0	PFD0795w	-1.65148	-0.75909	-0.43707	0.47757	1.56209
intPFD0800c_2	PFD0800c	-0.38434	-0.60279	-0.86216	-0.0935	1.166627
aPFD0800c_1	PFD0800c	-0.38434	-0.60279	-0.86216	-0.0935	1.166627
aPFD0800c_0	PFD0800c	-0.38434	-0.60279	-0.86216	-0.0935	1.166627
intPFD0805w_2	PFD0805w	-0.6535	-0.9025	0.065	0.693	0.5365
aPFD0805w_0	PFD0805w	-0.6535	-0.9025	0.065	0.693	0.5365
intPFD0810w_2	PFD0810w	-1.07244	-0.44978	0.26606	0.80994	0.78478
aPFD0810w_0	PFD0810w	-1.07244	-0.44978	0.26606	0.80994	0.78478
intPFD0815c_2	PFD0815c	0.380435	0.519075	0.163225	-0.15073	-0.30608
aPFD0815c_3	PFD0815c	0.380435	0.519075	0.163225	-0.15073	-0.30608
aPFD0815c_2	PFD0815c	0.380435	0.519075	0.163225	-0.15073	-0.30608
aPFD0815c_1	PFD0815c	0.380435	0.519075	0.163225	-0.15073	-0.30608
aPFD0815c_0	PFD0815c	0.380435	0.519075	0.163225	-0.15073	-0.30608
intPFD0820w_2	PFD0820w	0.424963	0.032163	0.152183	0.016483	-0.3745
aPFD0820w_0	PFD0820w	0.424963	0.032163	0.152183	0.016483	-0.3745
intPFD0825c_2	PFD0825c					
aPFD0825c_1	PFD0825c					

aPFD0825c_0	PFD0825c					
intPFD0830w_2	PFD0830w	-1.27845	-1.05125	0.118993	1.070173	1.112413
aPFD0830w_1	PFD0830w	-1.27845	-1.05125	0.118993	1.070173	1.112413
aPFD0830w_0	PFD0830w	-1.27845	-1.05125	0.118993	1.070173	1.112413
intPFD0835c_2	PFD0835c	-0.04951	0.166543	0.580923	0.313743	-0.46688
aPFD0835c_1	PFD0835c	-0.04951	0.166543	0.580923	0.313743	-0.46688
aPFD0835c_0	PFD0835c	-0.04951	0.166543	0.580923	0.313743	-0.46688
intPFD0840w_2	PFD0840w	0.054863	0.495293	0.047283	-0.21233	-0.09328
aPFD0840w_9	PFD0840w	0.054863	0.495293	0.047283	-0.21233	-0.09328
aPFD0840w_8	PFD0840w	0.054863	0.495293	0.047283	-0.21233	-0.09328
aPFD0840w_7	PFD0840w	0.054863	0.495293	0.047283	-0.21233	-0.09328
aPFD0840w_6	PFD0840w	0.054863	0.495293	0.047283	-0.21233	-0.09328
aPFD0840w_5	PFD0840w	0.054863	0.495293	0.047283	-0.21233	-0.09328
aPFD0840w_4	PFD0840w	0.054863	0.495293	0.047283	-0.21233	-0.09328
aPFD0840w_3	PFD0840w	0.054863	0.495293	0.047283	-0.21233	-0.09328
aPFD0840w_2	PFD0840w	0.054863	0.495293	0.047283	-0.21233	-0.09328
aPFD0840w_10	PFD0840w	0.054863	0.495293	0.047283	-0.21233	-0.09328
aPFD0840w_1	PFD0840w	0.054863	0.495293	0.047283	-0.21233	-0.09328
aPFD0840w_0	PFD0840w	0.054863	0.495293	0.047283	-0.21233	-0.09328
aPFD0850c_0	PFD0850c-a	-1.61906	-0.82673	0.189943	0.980723	1.111393
intPFD0860w_2	PFD0855c	-0.73149	-0.97057	-1.02511	0.09261	1.61507
intPFD0855c_2	PFD0855c	-0.51695	-0.57225	-1.23398	-0.33368	1.552587
intPFD0850c_2	PFD0855c	-1.61906	-0.82673	0.189943	0.980723	1.111393
aPFD0855c_1	PFD0855c	-0.51695	-0.57225	-1.23398	-0.33368	1.552587
aPFD0855c_0	PFD0855c	-0.51695	-0.57225	-1.23398	-0.33368	1.552587
aPFD0860w_1	PFD0860w	-0.73149	-0.97057	-1.02511	0.09261	1.61507
aPFD0860w_0	PFD0860w	-0.73149	-0.97057	-1.02511	0.09261	1.61507
aPFD0865c_1	PFD0865c	-0.9468	-0.38554	0.271313	0.642353	0.524703
aPFD0865c_0	PFD0865c	-0.9468	-0.38554	0.271313	0.642353	0.524703
intPFD0872w_2	PFD0870w	-0.18589	-0.07986	-0.35235	-0.14265	0.44072
intPFD0870w_2	PFD0870w	-0.4688	0.299537	-0.26282	-0.15834	0.600297
intPFD0865c_2	PFD0870w	-0.9468	-0.38554	0.271313	0.642353	0.524703
aPFD0870w_1	PFD0870w	-0.4688	0.299537	-0.26282	-0.15834	0.600297
aPFD0870w_0	PFD0870w	-0.4688	0.299537	-0.26282	-0.15834	0.600297
aPFD0872w_7	PFD0872w	-0.18589	-0.07986	-0.35235	-0.14265	0.44072
aPFD0872w_6	PFD0872w	-0.18589	-0.07986	-0.35235	-0.14265	0.44072
aPFD0872w_5	PFD0872w	-0.18589	-0.07986	-0.35235	-0.14265	0.44072
aPFD0872w_4	PFD0872w	-0.18589	-0.07986	-0.35235	-0.14265	0.44072
aPFD0872w_3	PFD0872w	-0.18589	-0.07986	-0.35235	-0.14265	0.44072
aPFD0872w_2	PFD0872w	-0.18589	-0.07986	-0.35235	-0.14265	0.44072
aPFD0872w_1	PFD0872w	-0.18589	-0.07986	-0.35235	-0.14265	0.44072
aPFD0872w_0	PFD0872w	-0.18589	-0.07986	-0.35235	-0.14265	0.44072
intPFD0875c_2	PFD0875c	0.54323	0.85487	0.59676	-0.21376	-1.02587
aPFD0875c_1	PFD0875c	0.54323	0.85487	0.59676	-0.21376	-1.02587
aPFD0875c_0	PFD0875c	0.54323	0.85487	0.59676	-0.21376	-1.02587
intPFD0880w_3	PFD0880w	0.083333	0.349313	0.679873	0.364793	-0.49415
aPFD0880w_1	PFD0880w	0.083333	0.349313	0.679873	0.364793	-0.49415

aPFD0880w_0	PFD0880w	0.083333	0.349313	0.679873	0.364793	-0.49415
intPFD0885c_2	PFD0885c	0.1929	-0.31498	-0.54578	-0.35022	0.20098
aPFD0885c_1	PFD0885c	0.1929	-0.31498	-0.54578	-0.35022	0.20098
aPFD0885c_0	PFD0885c	0.1929	-0.31498	-0.54578	-0.35022	0.20098
intPFD0890w_2	PFD0890w					
aPFD0890w_0	PFD0890w					
intPFD0895c_2	PFD0895c	-0.07049	-0.25154	0.16958	0.26042	-0.06446
aPFD0895c_0	PFD0895c	-0.07049	-0.25154	0.16958	0.26042	-0.06446
intPFD0905w_2	PFD0900w	0.6768	0.45901	0.26848	-0.67748	-1.42801
intPFD0900w_2	PFD0900w	1.296735	0.147665	-2.014	-2.31901	-0.12
aPFD0900w_2	PFD0900w	1.296735	0.147665	-2.014	-2.31901	-0.12
aPFD0900w_1	PFD0900w	1.296735	0.147665	-2.014	-2.31901	-0.12
aPFD0900w_0	PFD0900w	1.296735	0.147665	-2.014	-2.31901	-0.12
intPFD0910w_2	PFD0905w	0.339083	0.167603	-0.43271	-0.48063	0.071063
aPFD0905w_1	PFD0905w	0.6768	0.45901	0.26848	-0.67748	-1.42801
aPFD0905w_0	PFD0905w	0.6768	0.45901	0.26848	-0.67748	-1.42801
aPFD0910w_0	PFD0910w	0.339083	0.167603	-0.43271	-0.48063	0.071063
intPFD0920w_2	PFD0915w	-0.58963	-0.45189	-0.85823	0.115893	1.642553
intPFD0915w_2	PFD0915w	-0.42169	-0.39956	0.322113	0.644053	0.249723
aPFD0915w_1	PFD0915w	-0.42169	-0.39956	0.322113	0.644053	0.249723
aPFD0915w_0	PFD0915w	-0.42169	-0.39956	0.322113	0.644053	0.249723
intPFD0925w_2	PFD0920w	-0.40921	-0.11393	0.652857	0.638477	-0.17873
aPFD0920w_1	PFD0920w	-0.58963	-0.45189	-0.85823	0.115893	1.642553
aPFD0920w_0	PFD0920w	-0.58963	-0.45189	-0.85823	0.115893	1.642553
aPFD0925w_0	PFD0925w	-0.40921	-0.11393	0.652857	0.638477	-0.17873
intPFD0930w_2	PFD0930w	-0.16759	-0.30005	0.046083	0.171583	0.028713
aPFD0930w_0	PFD0930w	-0.16759	-0.30005	0.046083	0.171583	0.028713
aPFD0935c_0	PFD0935c					
intPFD0940w_2	PFD0940w	-0.65097	-0.81014	-1.79285	-0.48848	2.231058
intPFD0935c_2	PFD0940w					
aPFD0940w_3	PFD0940w	-0.65097	-0.81014	-1.79285	-0.48848	2.231058
aPFD0940w_2	PFD0940w	-0.65097	-0.81014	-1.79285	-0.48848	2.231058
aPFD0940w_1	PFD0940w	-0.65097	-0.81014	-1.79285	-0.48848	2.231058
aPFD0940w_0	PFD0940w	-0.65097	-0.81014	-1.79285	-0.48848	2.231058
intPFD0950w_1	PFD0945c	0.09793	0.15434	-0.17967	-0.06733	0.29866
intPFD0945c_2	PFD0945c	0.910873	0.420623	-0.4915	-0.672	-0.13013
aPFD0945c_1	PFD0945c	0.910873	0.420623	-0.4915	-0.672	-0.13013
aPFD0945c_0	PFD0945c	0.910873	0.420623	-0.4915	-0.672	-0.13013
aPFD0950w_0	PFD0950w	0.09793	0.15434	-0.17967	-0.06733	0.29866
intPFD0955w_2	PFD0955w	0.142187	-0.31895	-1.55709	-1.21757	0.768287
aPFD0955w_0	PFD0955w	0.142187	-0.31895	-1.55709	-1.21757	0.768287
intPFD0960c_2	PFD0960c	0.390187	0.492047	0.563907	-0.08457	-0.96271
aPFD0960c_0	PFD0960c	0.390187	0.492047	0.563907	-0.08457	-0.96271
aPFD0965w_7	PFD0965w	-0.23808	0.543365	-0.16458	-0.36681	0.193115
aPFD0965w_6	PFD0965w	-0.23808	0.543365	-0.16458	-0.36681	0.193115
aPFD0965w_5	PFD0965w	-0.23808	0.543365	-0.16458	-0.36681	0.193115
aPFD0965w_4	PFD0965w	-0.23808	0.543365	-0.16458	-0.36681	0.193115

aPFD0965w_3	PFD0965w	-0.23808	0.543365	-0.16458	-0.36681	0.193115
aPFD0965w_2	PFD0965w	-0.23808	0.543365	-0.16458	-0.36681	0.193115
aPFD0965w_1	PFD0965w	-0.23808	0.543365	-0.16458	-0.36681	0.193115
aPFD0965w_0	PFD0965w	-0.23808	0.543365	-0.16458	-0.36681	0.193115
intPFD0970c_2	PFD0970c	0.405985	0.143325	0.033405	-0.17159	-0.3584
aPFD0970c_5	PFD0970c	0.405985	0.143325	0.033405	-0.17159	-0.3584
aPFD0970c_4	PFD0970c	0.405985	0.143325	0.033405	-0.17159	-0.3584
aPFD0970c_3	PFD0970c	0.405985	0.143325	0.033405	-0.17159	-0.3584
aPFD0970c_2	PFD0970c	0.405985	0.143325	0.033405	-0.17159	-0.3584
aPFD0970c_1	PFD0970c	0.405985	0.143325	0.033405	-0.17159	-0.3584
aPFD0970c_0	PFD0970c	0.405985	0.143325	0.033405	-0.17159	-0.3584
intPFD0975w_3	PFD0975w	0.211617	0.374907	0.241937	-0.1186	-0.44207
aPFD0975w_1	PFD0975w	0.211617	0.374907	0.241937	-0.1186	-0.44207
aPFD0975w_0	PFD0975w	0.211617	0.374907	0.241937	-0.1186	-0.44207
intPFD0980w_2	PFD0980w	-0.52106	-0.4302	0.540407	0.751927	0.024037
aPFD0980w_1	PFD0980w	-0.52106	-0.4302	0.540407	0.751927	0.024037
aPFD0980w_0	PFD0980w	-0.52106	-0.4302	0.540407	0.751927	0.024037
intPFD0985w_2	PFD0985w	0.591237	0.008497	-0.16166	-0.97761	-1.36144
aPFD0985w_5	PFD0985w	0.591237	0.008497	-0.16166	-0.97761	-1.36144
aPFD0985w_4	PFD0985w	0.591237	0.008497	-0.16166	-0.97761	-1.36144
aPFD0985w_3	PFD0985w	0.591237	0.008497	-0.16166	-0.97761	-1.36144
aPFD0985w_2	PFD0985w	0.591237	0.008497	-0.16166	-0.97761	-1.36144
aPFD0985w_1	PFD0985w	0.591237	0.008497	-0.16166	-0.97761	-1.36144
aPFD0985w_0	PFD0985w	0.591237	0.008497	-0.16166	-0.97761	-1.36144
intPFD0990w_2	PFD0990w					
aPFD0990w_0	PFD0990w					
intPFD0993c_2	PFD0993c					
intPFD1006w_2	PFD1006w	0.270137	0.339327	-0.14815	-0.25451	0.026007
aPFD1006w_0	PFD1006w	0.270137	0.339327	-0.14815	-0.25451	0.026007
intPFD1007w_2	PFD1007w	0.19719	0.5268	0.15118	-0.34712	-0.51238
aPFD1007w_0	PFD1007w	0.19719	0.5268	0.15118	-0.34712	-0.51238
intPFD1030c_2	PFD1030c					
aPFD1030c_2	PFD1030c					
aPFD1030c_1	PFD1030c					
aPFD1030c_0	PFD1030c					
aPFD1004c_0	PFD1030c	0.805633	-0.16918	-1.07165	-0.69569	0.539843
intPFD1035w_2	PFD1035w	-0.03793	0.474687	0.409637	0.089697	-0.28735
aPFD1035w_0	PFD1035w	-0.03793	0.474687	0.409637	0.089697	-0.28735
intPFD1015w_2	PFD1037w	-0.38076	0.1093	0.41065	0.50685	0.2072
aPFD1015w_1	PFD1037w	-0.38076	0.1093	0.41065	0.50685	0.2072
aPFD1015w_0	PFD1037w	-0.38076	0.1093	0.41065	0.50685	0.2072
intPFD1045c_2	PFD1045c	0.451812	0.700472	0.274222	-0.38097	-0.75781
aPFD1045c_6	PFD1045c	0.451812	0.700472	0.274222	-0.38097	-0.75781
aPFD1045c_5	PFD1045c	0.451812	0.700472	0.274222	-0.38097	-0.75781
aPFD1045c_4	PFD1045c	0.451812	0.700472	0.274222	-0.38097	-0.75781
aPFD1045c_3	PFD1045c	0.451812	0.700472	0.274222	-0.38097	-0.75781
aPFD1045c_2	PFD1045c	0.451812	0.700472	0.274222	-0.38097	-0.75781

aPFD1045c_1	PFD1045c	0.451812	0.700472	0.274222	-0.38097	-0.75781
aPFD1045c_0	PFD1045c	0.451812	0.700472	0.274222	-0.38097	-0.75781
intPFD1050w_2	PFD1050w	-0.40288	-0.9201	-0.15504	0.574877	0.667437
aPFD1050w_1	PFD1050w	-0.40288	-0.9201	-0.15504	0.574877	0.667437
aPFD1050w_0	PFD1050w	-0.40288	-0.9201	-0.15504	0.574877	0.667437
intPFD1055w_2	PFD1055w	0.74082	0.50191	0.49217	0.02583	-0.77591
aPFD1055w_0	PFD1055w	0.74082	0.50191	0.49217	0.02583	-0.77591
intPFD1060w_2	PFD1060w	0.323633	0.421513	0.200933	-0.09767	-0.34175
aPFD1060w_4	PFD1060w	0.323633	0.421513	0.200933	-0.09767	-0.34175
aPFD1060w_3	PFD1060w	0.323633	0.421513	0.200933	-0.09767	-0.34175
aPFD1060w_2	PFD1060w	0.323633	0.421513	0.200933	-0.09767	-0.34175
aPFD1060w_1	PFD1060w	0.323633	0.421513	0.200933	-0.09767	-0.34175
aPFD1060w_0	PFD1060w	0.323633	0.421513	0.200933	-0.09767	-0.34175
aPFD1065c_0	PFD1065c	1.147863	0.520673	0.321153	-0.21649	-0.94901
intPFD1070w_2	PFD1070w	0.49096	0.34019	0.68612	0.21138	-0.87769
intPFD1065c_2	PFD1070w	1.147863	0.520673	0.321153	-0.21649	-0.94901
intPFD1045w_2	PFD1070w	-0.56795	-0.98276	-0.66024	0.469903	1.449423
aPFD1070w_1	PFD1070w	0.49096	0.34019	0.68612	0.21138	-0.87769
aPFD1070w_0	PFD1070w	0.49096	0.34019	0.68612	0.21138	-0.87769
intPFD1080w_2	PFD1075w	-0.41949	-0.18554	0.372077	0.445257	0.001877
aPFD1045w_1	PFD1075w	-0.56795	-0.98276	-0.66024	0.469903	1.449423
aPFD1045w_0	PFD1075w	-0.56795	-0.98276	-0.66024	0.469903	1.449423
aPFD1080w_0	PFD1080w	-0.41949	-0.18554	0.372077	0.445257	0.001877
intPFD1085w_2	PFD1085w	-0.07272	-0.39575	-0.27381	0.028117	0.304577
aPFD1085w_0	PFD1085w	-0.07272	-0.39575	-0.27381	0.028117	0.304577
intPFD1095w_2	PFD1090c	-0.09048	-0.1006	-0.2498	-0.00854	0.408263
aPFD1090c_0	PFD1090c	0.033387	-0.03894	0.022387	0.176947	0.187277
intPFD1090c_2	PFD1095w	0.033387	-0.03894	0.022387	0.176947	0.187277
aPFD1095w_0	PFD1095w	-0.09048	-0.1006	-0.2498	-0.00854	0.408263
intPFD1100c_2	PFD1100c	0.171433	-0.28515	-2.15072	-1.58762	1.314313
aPFD1100c_1	PFD1100c	0.171433	-0.28515	-2.15072	-1.58762	1.314313
aPFD1100c_0	PFD1100c	0.171433	-0.28515	-2.15072	-1.58762	1.314313
intPFD1105w_2	PFD1105w	0.726787	0.089087	-1.39068	-1.44898	0.193247
aPFD1105w_0	PFD1105w	0.726787	0.089087	-1.39068	-1.44898	0.193247
intPFD1110w_2	PFD1110w	-0.62641	-1.16045	-1.51486	-0.33264	1.71345
aPFD1110w_1	PFD1110w	-0.62641	-1.16045	-1.51486	-0.33264	1.71345
aPFD1110w_0	PFD1110w	-0.62641	-1.16045	-1.51486	-0.33264	1.71345
intPFD1115c_1	PFD1115c	0.072867	0.223317	0.100867	-0.11487	-0.23264
aPFD1115c_1	PFD1115c	0.072867	0.223317	0.100867	-0.11487	-0.23264
aPFD1115c_0	PFD1115c	0.072867	0.223317	0.100867	-0.11487	-0.23264
intPFD1120c_2	PFD1120c					
aPFD1120c_0	PFD1120c					
intPFD1130w_2	PFD1130w	1.301977	0.152627	-1.36925	-1.78391	-0.49779
aPFD1130w_1	PFD1130w	1.301977	0.152627	-1.36925	-1.78391	-0.49779
aPFD1130w_0	PFD1130w	1.301977	0.152627	-1.36925	-1.78391	-0.49779
aPFD1135c_0	PFD1135c	1.56342	0.74892	-0.53833	-1.75467	-1.66692
intPFD1140w_2	PFD1140w					

aPFD1140w_0	PFD1140w					
intPFD1145c_2	PFD1145c	0.951523	0.287873	-1.426	-2.12984	-0.70571
aPFD1145c_1	PFD1145c	0.951523	0.287873	-1.426	-2.12984	-0.70571
aPFD1145c_0	PFD1145c	0.951523	0.287873	-1.426	-2.12984	-0.70571
intPFD1150c_2	PFD1150c	1.356117	0.619767	-1.37087	-2.28279	-0.94977
aPFD1150c_2	PFD1150c	1.356117	0.619767	-1.37087	-2.28279	-0.94977
aPFD1150c_1	PFD1150c	1.356117	0.619767	-1.37087	-2.28279	-0.94977
aPFD1150c_0	PFD1150c	1.356117	0.619767	-1.37087	-2.28279	-0.94977
intPFD1155w_2	PFD1155w	1.95633	0.49485	-1.28171	-2.18029	-1.24285
aPFD1155w_2	PFD1155w	1.95633	0.49485	-1.28171	-2.18029	-1.24285
aPFD1155w_1	PFD1155w	1.95633	0.49485	-1.28171	-2.18029	-1.24285
aPFD1155w_0	PFD1155w	1.95633	0.49485	-1.28171	-2.18029	-1.24285
intPFD1160w_2	PFD1160w	0.974463	0.193663	0.114683	-0.24802	-0.778
aPFD1160w_3	PFD1160w	0.974463	0.193663	0.114683	-0.24802	-0.778
aPFD1160w_2	PFD1160w	0.974463	0.193663	0.114683	-0.24802	-0.778
aPFD1160w_1	PFD1160w	0.974463	0.193663	0.114683	-0.24802	-0.778
aPFD1160w_0	PFD1160w	0.974463	0.193663	0.114683	-0.24802	-0.778
aPFD1165w_1	PFD1165w	2.021447	1.717757	0.335487	-1.89315	-2.98342
aPFD1165w_0	PFD1165w	2.021447	1.717757	0.335487	-1.89315	-2.98342
intPFD1170c_3	PFD1170c	1.427983	0.067063	0.164883	-0.93322	-2.1334
aPFD1170c_0	PFD1170c	1.427983	0.067063	0.164883	-0.93322	-2.1334
intPFD1175w_2	PFD1175w	-0.0495	1.938517	1.295707	-0.62037	-1.99718
aPFD1175w_1	PFD1175w	-0.0495	1.938517	1.295707	-0.62037	-1.99718
aPFD1175w_0	PFD1175w	-0.0495	1.938517	1.295707	-0.62037	-1.99718
intPFD1180w_2	PFD1180w	2.513503	1.601463	-0.26292	-1.82642	-2.0278
aPFD1180w_0	PFD1180w	2.513503	1.601463	-0.26292	-1.82642	-2.0278
intPFD1185w_2	PFD1185w	2.073097	1.266067	-0.05453	-1.17158	-1.50438
aPFD1185w_0	PFD1185w	2.073097	1.266067	-0.05453	-1.17158	-1.50438
intPFD1195c_2	PFD1195c	0.170878	-0.09039	-0.12899	-0.00939	0.105158
aPFD1195c_0	PFD1195c	0.170878	-0.09039	-0.12899	-0.00939	0.105158
aPFD1190c_0	PFD1195c					
aPFD1200c_0	PFD1200c	-0.05204	-0.16287	0.023328	0.139388	0.075088
intPFD1205w_2	PFD1205w					
intPFD1200c_2	PFD1205w	-0.05204	-0.16287	0.023328	0.139388	0.075088
aPFD1205w_0	PFD1205w					
intPFD1210w_2	PFD1210w					
aPFD1210w_0	PFD1210w					
intPFD1215w_2	PFD1215w					
aPFD1215w_0	PFD1215w					
intPFE0040c_2	PFE0040c	-2.14092	-1.69575	0.886503	1.939383	0.866973
aPFE0040c_2	PFE0040c	-2.14092	-1.69575	0.886503	1.939383	0.866973
aPFE0040c_1	PFE0040c	-2.14092	-1.69575	0.886503	1.939383	0.866973
aPFE0040c_0	PFE0040c	-2.14092	-1.69575	0.886503	1.939383	0.866973
intPFE0045c_2	PFE0045c	-0.04198	-0.24914	-0.91648	-0.70786	0.466303
aPFE0045c_1	PFE0045c	-0.04198	-0.24914	-0.91648	-0.70786	0.466303
aPFE0045c_0	PFE0045c	-0.04198	-0.24914	-0.91648	-0.70786	0.466303
intPFE0050w_2	PFE0050w	-0.34503	0.345692	1.654432	1.002772	-1.18349

aPFE0050w_0	PFE0050w	-0.34503	0.345692	1.654432	1.002772	-1.18349
intPFE0055c_2	PFE0055c	1.96985	0.92375	-0.47026	-1.70574	-1.73725
aPFE0055c_1	PFE0055c	1.96985	0.92375	-0.47026	-1.70574	-1.73725
aPFE0055c_0	PFE0055c	1.96985	0.92375	-0.47026	-1.70574	-1.73725
intPFE0060w_2	PFE0060w	-2.08731	-0.84346	1.924903	2.073763	-0.28488
aPFE0060w_1	PFE0060w	-2.08731	-0.84346	1.924903	2.073763	-0.28488
aPFE0060w_0	PFE0060w	-2.08731	-0.84346	1.924903	2.073763	-0.28488
intPFE0065w_3	PFE0065w	2.624653	2.736743	0.305003	-2.41234	-3.22808
aPFE0065w_0	PFE0065w	2.624653	2.736743	0.305003	-2.41234	-3.22808
intPFE0070w_3	PFE0070w	2.670783	1.368723	-1.21586	-2.60242	-1.65234
aPFE0070w_2	PFE0070w	2.670783	1.368723	-1.21586	-2.60242	-1.65234
aPFE0070w_0	PFE0070w	2.670783	1.368723	-1.21586	-2.60242	-1.65234
intPFE0075c_2	PFE0075c	-0.19008	-0.92213	-2.29776	-1.21658	1.836293
aPFE0075c_1	PFE0075c	-0.19008	-0.92213	-2.29776	-1.21658	1.836293
aPFE0075c_0	PFE0075c	-0.19008	-0.92213	-2.29776	-1.21658	1.836293
intPFE0080c_2	PFE0080c	-0.46014	-1.24176	-2.41996	-1.0767	2.147097
aPFE0080c_1	PFE0080c	-0.46014	-1.24176	-2.41996	-1.0767	2.147097
aPFE0080c_0	PFE0080c	-0.46014	-1.24176	-2.41996	-1.0767	2.147097
intPFE0085c_2	PFE0085c	-0.3237	-0.61251	-0.30448	0.08248	0.38151
aPFE0085c_1	PFE0085c	-0.3237	-0.61251	-0.30448	0.08248	0.38151
aPFE0085c_0	PFE0085c	-0.3237	-0.61251	-0.30448	0.08248	0.38151
intPFE0090w_2	PFE0090w	-1.16491	-1.05192	-0.29639	0.734227	1.331757
aPFE0090w_1	PFE0090w	-1.16491	-1.05192	-0.29639	0.734227	1.331757
aPFE0090w_0	PFE0090w	-1.16491	-1.05192	-0.29639	0.734227	1.331757
intPFE0100w_2	PFE0095c	0.62914	0.47628	-0.1223	-0.2982	-0.11528
aPFE0095c_0	PFE0095c	0.424532	0.220782	-0.02906	-0.18694	-0.22148
intPFE0095c_2	PFE0100w	0.424532	0.220782	-0.02906	-0.18694	-0.22148
aPFE0100w_1	PFE0100w	0.62914	0.47628	-0.1223	-0.2982	-0.11528
aPFE0100w_0	PFE0100w	0.62914	0.47628	-0.1223	-0.2982	-0.11528
aPFE0105c_0	PFE0105c	-0.28559	-0.21907	0.069123	0.261543	0.214733
intPFE0110w_2	PFE0110w	-0.10774	0.0812	0.12585	0.08165	-0.0182
intPFE0105c_2	PFE0110w	-0.28559	-0.21907	0.069123	0.261543	0.214733
aPFE0110w_1	PFE0110w	-0.10774	0.0812	0.12585	0.08165	-0.0182
aPFE0110w_0	PFE0110w	-0.10774	0.0812	0.12585	0.08165	-0.0182
intPFE0115c_2	PFE0115c	0.730233	0.241813	-0.16412	-0.30122	-0.23315
aPFE0115c_0	PFE0115c	0.730233	0.241813	-0.16412	-0.30122	-0.23315
intPFE0120c_2	PFE0120c	2.194477	2.134127	0.255997	-2.13316	-2.97779
aPFE0120c_1	PFE0120c	2.194477	2.134127	0.255997	-2.13316	-2.97779
aPFE0120c_0	PFE0120c	2.194477	2.134127	0.255997	-2.13316	-2.97779
intPFE0125w_2	PFE0125w	1.748017	0.421427	-0.33161	-0.99855	-1.19159
aPFE0125w_1	PFE0125w	1.748017	0.421427	-0.33161	-0.99855	-1.19159
aPFE0125w_0	PFE0125w	1.748017	0.421427	-0.33161	-0.99855	-1.19159
aPFE0130c_1	PFE0127c	0.57395	0.24825	0.38975	0.13975	-0.53075
intPFE0135w_2	PFE0130c	0.634667	0.961667	0.802167	-0.12283	-1.22533
intPFE0130c_2	PFE0130c	0.57395	0.24825	0.38975	0.13975	-0.53075
aPFE0130c_0	PFE0130c	0.57395	0.24825	0.38975	0.13975	-0.53075
aPFE0135w_0	PFE0135w	0.634667	0.961667	0.802167	-0.12283	-1.22533

intPFE0145w_2	PFE0140c	-0.41004	-1.5558	-0.3894	0.746063	0.932463
aPFE0140c_0	PFE0140c	-0.14246	-0.37968	0.473357	0.542977	-0.25898
intPFE0140c_2	PFE0145w	-0.14246	-0.37968	0.473357	0.542977	-0.25898
aPFE0145w_0	PFE0145w	-0.41004	-1.5558	-0.3894	0.746063	0.932463
intPFE0150c_2	PFE0150c	-0.86002	0.1011	0.31505	0.39095	0.3629
aPFE0150c_1	PFE0150c	-0.86002	0.1011	0.31505	0.39095	0.3629
aPFE0150c_0	PFE0150c	-0.86002	0.1011	0.31505	0.39095	0.3629
intPFE0155w_2	PFE0155w	-0.87797	-0.84765	-0.2827	0.5232	1.03065
aPFE0155w_1	PFE0155w	-0.87797	-0.84765	-0.2827	0.5232	1.03065
aPFE0155w_0	PFE0155w	-0.87797	-0.84765	-0.2827	0.5232	1.03065
intPFE0160c_2	PFE0160c	0.202603	0.338003	0.346993	0.026673	-0.43834
aPFE0160c_0	PFE0160c	0.202603	0.338003	0.346993	0.026673	-0.43834
intPFE0165w_3	PFE0165w	-0.36215	-1.79327	-1.28297	0.10797	1.43927
aPFE0165w_0	PFE0165w	-0.36215	-1.79327	-1.28297	0.10797	1.43927
intPFE0170c_2	PFE0170c	-0.94417	0.21634	0.70756	0.27494	-0.42584
aPFE0170c_1	PFE0170c	-0.94417	0.21634	0.70756	0.27494	-0.42584
aPFE0170c_0	PFE0170c	-0.94417	0.21634	0.70756	0.27494	-0.42584
intPFE0175c_2	PFE0175c	1.42963	0.45734	-1.37042	-2.05308	-0.73884
aPFE0180w_0	PFE0175c	0.270163	0.565183	0.086623	-0.26296	-0.26652
aPFE0175c_1	PFE0175c	1.42963	0.45734	-1.37042	-2.05308	-0.73884
aPFE0175c_0	PFE0175c	1.42963	0.45734	-1.37042	-2.05308	-0.73884
intPFE0185c_3	PFE0185c	-0.00518	0.30989	0.73521	0.45379	-0.45889
aPFE0185c_0	PFE0185c	-0.00518	0.30989	0.73521	0.45379	-0.45889
intPFE0190c_2	PFE0190c	-0.67764	0.335707	0.763587	0.545247	-0.13237
aPFE0190c_1	PFE0190c	-0.67764	0.335707	0.763587	0.545247	-0.13237
aPFE0190c_0	PFE0190c	-0.67764	0.335707	0.763587	0.545247	-0.13237
intPFE0195w_2	PFE0195w	-0.22932	0.04259	0.00266	0.00356	0.1053
aPFE0195w_3	PFE0195w	-0.22932	0.04259	0.00266	0.00356	0.1053
aPFE0195w_2	PFE0195w	-0.22932	0.04259	0.00266	0.00356	0.1053
aPFE0195w_1	PFE0195w	-0.22932	0.04259	0.00266	0.00356	0.1053
aPFE0195w_0	PFE0195w	-0.22932	0.04259	0.00266	0.00356	0.1053
intPFE0205w_2	PFE0200c	-0.78048	-1.17308	-0.02709	0.626427	0.467417
aPFE0200c_0	PFE0200c	-0.06163	-0.92884	0.079687	0.310647	-0.27882
intPFE0200c_2	PFE0205w	-0.06163	-0.92884	0.079687	0.310647	-0.27882
aPFE0205w_1	PFE0205w	-0.78048	-1.17308	-0.02709	0.626427	0.467417
aPFE0205w_0	PFE0205w	-0.78048	-1.17308	-0.02709	0.626427	0.467417
aPFE0210c_0	PFE0210c	0.176187	0.479087	0.271327	-0.09399	-0.37775
intPFE0220w_2	PFE0215w	-1.4109	-0.77504	0.039825	0.755515	1.013865
intPFE0215w_2	PFE0215w	-1.28018	-1.0691	0.136967	1.051367	1.039937
intPFE0210c_2	PFE0215w	0.176187	0.479087	0.271327	-0.09399	-0.37775
aPFE0215w_1	PFE0215w	-1.28018	-1.0691	0.136967	1.051367	1.039937
aPFE0215w_0	PFE0215w	-1.28018	-1.0691	0.136967	1.051367	1.039937
aPFE0220w_0	PFE0220w	-1.4109	-0.77504	0.039825	0.755515	1.013865
intPFE0225w_2	PFE0225w	-0.21875	-0.47073	-0.82604	-0.23262	0.924567
aPFE0225w_1	PFE0225w	-0.21875	-0.47073	-0.82604	-0.23262	0.924567
aPFE0225w_0	PFE0225w	-0.21875	-0.47073	-0.82604	-0.23262	0.924567
intPFE0230w_2	PFE0230w	0.722598	0.990268	0.201838	-0.43542	-0.60385

aPFE0230w_3	PFE0230w	0.722598	0.990268	0.201838	-0.43542	-0.60385
aPFE0230w_1	PFE0230w	0.722598	0.990268	0.201838	-0.43542	-0.60385
aPFE0230w_0	PFE0230w	0.722598	0.990268	0.201838	-0.43542	-0.60385
aPFE0240c_8	PFE0235c	-0.862	-0.99327	-0.92044	0.259013	1.701133
aPFE0240c_7	PFE0235c	-0.862	-0.99327	-0.92044	0.259013	1.701133
aPFE0240c_6	PFE0235c	-0.862	-0.99327	-0.92044	0.259013	1.701133
aPFE0240c_5	PFE0235c	-0.862	-0.99327	-0.92044	0.259013	1.701133
aPFE0240c_4	PFE0235c	-0.862	-0.99327	-0.92044	0.259013	1.701133
aPFE0240c_3	PFE0235c	-0.862	-0.99327	-0.92044	0.259013	1.701133
aPFE0240c_2	PFE0235c	-0.862	-0.99327	-0.92044	0.259013	1.701133
aPFE0240c_1	PFE0235c	-0.862	-0.99327	-0.92044	0.259013	1.701133
aPFE0240c_0	PFE0235c	-0.862	-0.99327	-0.92044	0.259013	1.701133
intPFE0240w_2	PFE0240w	-0.37987	-1.23763	-0.61323	0.54723	1.24663
aPFE0240w_0	PFE0240w	-0.37987	-1.23763	-0.61323	0.54723	1.24663
intPFE0245c_2	PFE0245c	0.6408	0.87249	0.61303	-0.18778	-1.03749
aPFE0245c_4	PFE0245c	0.6408	0.87249	0.61303	-0.18778	-1.03749
aPFE0245c_3	PFE0245c	0.6408	0.87249	0.61303	-0.18778	-1.03749
aPFE0245c_2	PFE0245c	0.6408	0.87249	0.61303	-0.18778	-1.03749
aPFE0245c_1	PFE0245c	0.6408	0.87249	0.61303	-0.18778	-1.03749
aPFE0245c_0	PFE0245c	0.6408	0.87249	0.61303	-0.18778	-1.03749
intPFE0250w_2	PFE0250w	-0.4327	0.200103	-0.0832	-0.02214	0.396963
aPFE0250w_4	PFE0250w	-0.4327	0.200103	-0.0832	-0.02214	0.396963
aPFE0250w_3	PFE0250w	-0.4327	0.200103	-0.0832	-0.02214	0.396963
aPFE0250w_2	PFE0250w	-0.4327	0.200103	-0.0832	-0.02214	0.396963
aPFE0250w_1	PFE0250w	-0.4327	0.200103	-0.0832	-0.02214	0.396963
aPFE0250w_0	PFE0250w	-0.4327	0.200103	-0.0832	-0.02214	0.396963
intPFE0255w_2	PFE0255w	-0.20721	-0.49346	0.162913	0.190913	-0.27221
aPFE0255w_1	PFE0255w	-0.20721	-0.49346	0.162913	0.190913	-0.27221
aPFE0255w_0	PFE0255w	-0.20721	-0.49346	0.162913	0.190913	-0.27221
intPFE0260w_2	PFE0260w	0.70042	-0.21659	-0.82656	-0.62894	0.19409
aPFE0260w_1	PFE0260w	0.70042	-0.21659	-0.82656	-0.62894	0.19409
aPFE0260w_0	PFE0260w	0.70042	-0.21659	-0.82656	-0.62894	0.19409
intPFE0265c_2	PFE0265c	-0.95088	-0.91261	-0.79604	0.212203	1.486273
aPFE0265c_1	PFE0265c	-0.95088	-0.91261	-0.79604	0.212203	1.486273
aPFE0265c_0	PFE0265c	-0.95088	-0.91261	-0.79604	0.212203	1.486273
intPFE0270c_2	PFE0270c	-0.99361	-1.22341	-0.11817	0.960007	1.172247
aPFE0270c_1	PFE0270c	-0.99361	-1.22341	-0.11817	0.960007	1.172247
aPFE0270c_0	PFE0270c	-0.99361	-1.22341	-0.11817	0.960007	1.172247
intPFE0275w_2	PFE0275w					
aPFE0275w_0	PFE0275w					
intPFE0280c_2	PFE0280c	0.226897	0.354037	-0.09657	-0.16809	0.085297
aPFE0280c_1	PFE0280c	0.226897	0.354037	-0.09657	-0.16809	0.085297
aPFE0280c_0	PFE0280c	0.226897	0.354037	-0.09657	-0.16809	0.085297
intPFE0285c_1	PFE0285c	-1.03176	-0.42919	0.42588	0.94012	0.68719
aPFE0285c_0	PFE0285c	-1.03176	-0.42919	0.42588	0.94012	0.68719
intPFE0295w_2	PFE0290c					
aPFE0290c_0	PFE0290c					

intPFE0290c_2	PFE0295w					
aPFE0295w_0	PFE0295w					
intPFE0305w_2	PFE0300c	1.53891	0.77346	0.03709	-0.78009	-1.23546
aPFE0300c_0	PFE0300c	0.263907	0.213477	0.698547	0.316787	-0.75114
intPFE0300c_2	PFE0305w	0.263907	0.213477	0.698547	0.316787	-0.75114
aPFE0305w_0	PFE0305w	1.53891	0.77346	0.03709	-0.78009	-1.23546
aPFE0310c_1	PFE0310c	-0.76284	-0.76833	0.546393	1.004773	0.247493
aPFE0310c_0	PFE0310c	-0.76284	-0.76833	0.546393	1.004773	0.247493
intPFE0315c_2	PFE0315c	0.015503	-0.292	-0.3965	-0.11916	0.348343
intPFE0310c_2	PFE0315c	-0.76284	-0.76833	0.546393	1.004773	0.247493
aPFE0315c_0	PFE0315c	0.015503	-0.292	-0.3965	-0.11916	0.348343
intPFE0320w_2	PFE0320w	0.11888	0.03012	-0.80698	-0.81502	0.23738
aPFE0325w_4	PFE0320w	-0.419	0.205763	0.147463	0.029603	0.050903
aPFE0325w_3	PFE0320w	-0.419	0.205763	0.147463	0.029603	0.050903
aPFE0325w_2	PFE0320w	-0.419	0.205763	0.147463	0.029603	0.050903
aPFE0325w_1	PFE0320w	-0.419	0.205763	0.147463	0.029603	0.050903
aPFE0325w_0	PFE0320w	-0.419	0.205763	0.147463	0.029603	0.050903
aPFE0320w_1	PFE0320w	0.11888	0.03012	-0.80698	-0.81502	0.23738
aPFE0320w_0	PFE0320w	0.11888	0.03012	-0.80698	-0.81502	0.23738
intPFE0330w_2	PFE0328w	-0.7143	0.517017	0.133717	-0.03388	0.286817
aPFE0330w_1	PFE0330w	-0.7143	0.517017	0.133717	-0.03388	0.286817
aPFE0330w_0	PFE0330w	-0.7143	0.517017	0.133717	-0.03388	0.286817
intPFE0335w_2	PFE0335w	0.050257	0.342237	0.180017	-0.02418	-0.1549
aPFE0335w_1	PFE0335w	0.050257	0.342237	0.180017	-0.02418	-0.1549
aPFE0335w_0	PFE0335w	0.050257	0.342237	0.180017	-0.02418	-0.1549
intPFE0340c_2	PFE0340c	-0.63681	-0.77697	-1.87683	-0.74101	2.023633
aPFE0340c_1	PFE0340c	-0.63681	-0.77697	-1.87683	-0.74101	2.023633
aPFE0340c_0	PFE0340c	-0.63681	-0.77697	-1.87683	-0.74101	2.023633
intPFE0345c_2	PFE0345c	-0.26262	0.025832	0.144712	0.212872	0.158752
aPFE0345c_0	PFE0345c	-0.26262	0.025832	0.144712	0.212872	0.158752
intPFE0350c_2	PFE0350c	0.020917	0.468397	0.794207	0.465127	-0.45306
intPFE0355c_2	PFE0355c	-0.1709	-0.16701	-1.70199	-1.09585	1.422173
aPFE0355c_1	PFE0355c	-0.1709	-0.16701	-1.70199	-1.09585	1.422173
aPFE0355c_0	PFE0355c	-0.1709	-0.16701	-1.70199	-1.09585	1.422173
intPFE0360c_2	PFE0360c	1.32839	1.18454	-1.07882	-2.20918	-0.98904
aPFE0360c_1	PFE0360c	1.32839	1.18454	-1.07882	-2.20918	-0.98904
aPFE0360c_0	PFE0360c	1.32839	1.18454	-1.07882	-2.20918	-0.98904
aPFE0365c_0	PFE0365c	0.271332	0.039532	-0.88064	-1.28881	-0.38281
intPFE0370c_2	PFE0370c	0.805853	-0.09727	-1.94997	-2.17737	-0.02457
intPFE0365c_2	PFE0370c	0.271332	0.039532	-0.88064	-1.28881	-0.38281
aPFE0370c_1	PFE0370c	0.805853	-0.09727	-1.94997	-2.17737	-0.02457
aPFE0370c_0	PFE0370c	0.805853	-0.09727	-1.94997	-2.17737	-0.02457
intPFE0375w_1	PFE0375w	-0.12401	-0.26049	0.084233	0.164933	-0.03935
aPFE0375w_1	PFE0375w	-0.12401	-0.26049	0.084233	0.164933	-0.03935
aPFE0375w_0	PFE0375w	-0.12401	-0.26049	0.084233	0.164933	-0.03935
intPFE0380c_2	PFE0380c	-0.50818	-0.3501	0.18244	0.50906	0.3651
aPFE0380c_1	PFE0380c	-0.50818	-0.3501	0.18244	0.50906	0.3651

aPFE0380c_0	PFE0380c	-0.50818	-0.3501	0.18244	0.50906	0.3651
intPFE0390w_2	PFE0385w	0.854677	0.530607	0.273787	-0.26745	-0.83427
intPFE0385w_2	PFE0385w	0.246607	0.233647	0.210877	-0.29143	-0.7502
aPFE0385w_2	PFE0385w	0.246607	0.233647	0.210877	-0.29143	-0.7502
aPFE0385w_1	PFE0385w	0.246607	0.233647	0.210877	-0.29143	-0.7502
aPFE0385w_0	PFE0385w	0.246607	0.233647	0.210877	-0.29143	-0.7502
aPFE0390w_0	PFE0390w	0.854677	0.530607	0.273787	-0.26745	-0.83427
intPFE0395c_2	PFE0395c	-0.45048	-0.94662	-1.85776	-0.53024	2.12962
aPFE0395c_0	PFE0395c	-0.45048	-0.94662	-1.85776	-0.53024	2.12962
intPFE0400w_2	PFE0400w	-0.43079	-1.1907	-2.09577	-0.5549	2.3597
aPFE0400w_2	PFE0400w	-0.43079	-1.1907	-2.09577	-0.5549	2.3597
aPFE0400w_1	PFE0400w	-0.43079	-1.1907	-2.09577	-0.5549	2.3597
aPFE0400w_0	PFE0400w	-0.43079	-1.1907	-2.09577	-0.5549	2.3597
intPFE0405c_2	PFE0405c	-0.79879	-0.96305	-0.6234	0.327063	1.257713
aPFE0405c_0	PFE0405c	-0.79879	-0.96305	-0.6234	0.327063	1.257713
intPFE0410w_3	PFE0410w	-1.38346	-0.79268	0.89986	1.47314	0.49268
aPFE0410w_0	PFE0410w	-1.38346	-0.79268	0.89986	1.47314	0.49268
intPFE0415w_2	PFE0415w	-0.46125	-0.46122	-1.18981	-0.38235	1.454057
aPFE0415w_1	PFE0415w	-0.46125	-0.46122	-1.18981	-0.38235	1.454057
aPFE0415w_0	PFE0415w	-0.46125	-0.46122	-1.18981	-0.38235	1.454057
intPFE0420c_2	PFE0420c	0.05122	-0.21137	-1.35101	-0.89799	0.97187
aPFE0420c_3	PFE0420c	0.05122	-0.21137	-1.35101	-0.89799	0.97187
aPFE0420c_2	PFE0420c	0.05122	-0.21137	-1.35101	-0.89799	0.97187
aPFE0420c_1	PFE0420c	0.05122	-0.21137	-1.35101	-0.89799	0.97187
aPFE0420c_0	PFE0420c	0.05122	-0.21137	-1.35101	-0.89799	0.97187
intPFE0425w_2	PFE0425w	0.3114	0.47602	-0.18703	-0.52697	-0.23402
intPFE0430w_2	PFE0430w	0.168635	0.211835	-0.02909	-0.11153	-0.02895
aPFE0430w_2	PFE0430w	0.168635	0.211835	-0.02909	-0.11153	-0.02895
aPFE0430w_1	PFE0430w	0.168635	0.211835	-0.02909	-0.11153	-0.02895
aPFE0430w_0	PFE0430w	0.168635	0.211835	-0.02909	-0.11153	-0.02895
intPFE0435c_2	PFE0435c	-0.72763	-0.83487	-0.23927	0.333933	0.625533
aPFE0435c_0	PFE0435c	-0.72763	-0.83487	-0.23927	0.333933	0.625533
intPFE0440w_2	PFE0440w	-0.38137	-0.73495	-2.046	-0.9224	2.00455
aPFE0440w_4	PFE0440w	-0.38137	-0.73495	-2.046	-0.9224	2.00455
aPFE0440w_3	PFE0440w	-0.38137	-0.73495	-2.046	-0.9224	2.00455
aPFE0440w_2	PFE0440w	-0.38137	-0.73495	-2.046	-0.9224	2.00455
aPFE0440w_1	PFE0440w	-0.38137	-0.73495	-2.046	-0.9224	2.00455
aPFE0440w_0	PFE0440w	-0.38137	-0.73495	-2.046	-0.9224	2.00455
intPFE0445c_2	PFE0445c	-0.6821	-0.39346	0.119927	0.522407	0.528797
aPFE0445c_0	PFE0445c	-0.6821	-0.39346	0.119927	0.522407	0.528797
intPFE0455w_2	PFE0450w	-0.80544	-0.68479	0.23738	0.82522	0.61459
intPFE0450w_2	PFE0450w	-1.26489	-1.39457	-0.7147	0.679362	1.821242
aPFE0450w_2	PFE0450w	-1.26489	-1.39457	-0.7147	0.679362	1.821242
aPFE0450w_1	PFE0450w	-1.26489	-1.39457	-0.7147	0.679362	1.821242
aPFE0450w_0	PFE0450w	-1.26489	-1.39457	-0.7147	0.679362	1.821242
aPFE0455w_0	PFE0455w	-0.80544	-0.68479	0.23738	0.82522	0.61459
aPFE0460c_0	PFE0460c	0.609147	-0.21575	0.219007	0.214327	-0.42691

intPFE0465c_2	PFE0465c	0.558112	0.762712	0.740942	-0.23511	-1.38863
intPFE0460c_2	PFE0465c	0.609147	-0.21575	0.219007	0.214327	-0.42691
aPFE0465c_4	PFE0465c	0.558112	0.762712	0.740942	-0.23511	-1.38863
aPFE0465c_3	PFE0465c	0.558112	0.762712	0.740942	-0.23511	-1.38863
aPFE0465c_2	PFE0465c	0.558112	0.762712	0.740942	-0.23511	-1.38863
aPFE0465c_1	PFE0465c	0.558112	0.762712	0.740942	-0.23511	-1.38863
aPFE0465c_0	PFE0465c	0.558112	0.762712	0.740942	-0.23511	-1.38863
intPFE0475w_2	PFE0470w	-0.48194	-0.25934	0.602923	0.721743	-0.035
intPFE0470w_2	PFE0470w	0.029903	-0.12351	-0.369	-0.06534	0.486173
aPFE0470w_1	PFE0470w	0.029903	-0.12351	-0.369	-0.06534	0.486173
aPFE0470w_0	PFE0470w	0.029903	-0.12351	-0.369	-0.06534	0.486173
aPFE0475w_1	PFE0475w	-0.48194	-0.25934	0.602923	0.721743	-0.035
aPFE0475w_0	PFE0475w	-0.48194	-0.25934	0.602923	0.721743	-0.035
intPFE0480c_2	PFE0480c	-0.31406	-0.7067	0.131407	0.543927	0.208537
aPFE0480c_1	PFE0480c	-0.31406	-0.7067	0.131407	0.543927	0.208537
aPFE0480c_0	PFE0480c	-0.31406	-0.7067	0.131407	0.543927	0.208537
intPFE0485w_2	PFE0485w	0.19972	0.59338	0.10999	-0.45849	-0.56038
aPFE0485w_2	PFE0485w	0.19972	0.59338	0.10999	-0.45849	-0.56038
aPFE0485w_1	PFE0485w	0.19972	0.59338	0.10999	-0.45849	-0.56038
aPFE0485w_0	PFE0485w	0.19972	0.59338	0.10999	-0.45849	-0.56038
intPFE0490w_2	PFE0490w	0.15603	0.09365	-0.17839	-0.04701	0.26695
aPFE0490w_0	PFE0490w	0.15603	0.09365	-0.17839	-0.04701	0.26695
intPFE0495w_2	PFE0495w	-0.56501	-0.87192	-0.18366	0.504997	0.701257
aPFE0495w_1	PFE0495w	-0.56501	-0.87192	-0.18366	0.504997	0.701257
aPFE0495w_0	PFE0495w	-0.56501	-0.87192	-0.18366	0.504997	0.701257
intPFE0500c_2	PFE0500c	-1.66333	-1.73867	-0.34274	1.083018	1.648198
aPFE0500c_3	PFE0500c	-1.66333	-1.73867	-0.34274	1.083018	1.648198
aPFE0500c_2	PFE0500c	-1.66333	-1.73867	-0.34274	1.083018	1.648198
aPFE0500c_1	PFE0500c	-1.66333	-1.73867	-0.34274	1.083018	1.648198
aPFE0500c_0	PFE0500c	-1.66333	-1.73867	-0.34274	1.083018	1.648198
intPFE0505w_2	PFE0505w	0.326857	0.210727	0.039797	-0.08096	-0.16289
aPFE0505w_1	PFE0505w	0.326857	0.210727	0.039797	-0.08096	-0.16289
aPFE0505w_0	PFE0505w	0.326857	0.210727	0.039797	-0.08096	-0.16289
intPFE0510c_2	PFE0510c	0.67029	0.39853	0.15744	-0.19744	-0.54253
aPFE0510c_0	PFE0510c	0.67029	0.39853	0.15744	-0.19744	-0.54253
intPFE0515w_2	PFE0515w	1.409603	1.341993	0.648263	-0.5211	-1.54533
aPFE0515w_1	PFE0515w	1.409603	1.341993	0.648263	-0.5211	-1.54533
aPFE0515w_0	PFE0515w	1.409603	1.341993	0.648263	-0.5211	-1.54533
intPFE0520c_2	PFE0520c	-0.84216	-0.28173	-0.19005	0.237713	0.797893
aPFE0520c_1	PFE0520c	-0.84216	-0.28173	-0.19005	0.237713	0.797893
aPFE0520c_0	PFE0520c	-0.84216	-0.28173	-0.19005	0.237713	0.797893
intPFE0535w_2	PFE0530w	0.36179	1.15803	0.78794	-0.06394	-0.88903
intPFE0530w_2	PFE0530w	0.183925	0.203315	0.032045	-0.23502	-0.33705
aPFE0530w_0	PFE0530w	0.183925	0.203315	0.032045	-0.23502	-0.33705
aPFE0535w_0	PFE0535w	0.36179	1.15803	0.78794	-0.06394	-0.88903
intPFE0540w_2	PFE0540w	-0.17191	0.065557	-0.47085	-0.39031	0.378777
aPFE0540w_1	PFE0540w	-0.17191	0.065557	-0.47085	-0.39031	0.378777

aPFE0540w_0	PFE0540w	-0.17191	0.065557	-0.47085	-0.39031	0.378777
aPFE0545c_0	PFE0545c	-0.72173	-0.5011	0.469545	0.942135	0.458885
intPFE0550w_2	PFE0550w	0.31117	0.66397	0.39495	-0.11615	-0.55937
intPFE0545c_2	PFE0550w	-0.72173	-0.5011	0.469545	0.942135	0.458885
aPFE0550w_0	PFE0550w	0.31117	0.66397	0.39495	-0.11615	-0.55937
intPFE0555w_2	PFE0555w	-1.1288	-1.14852	-1.24023	0.170893	2.140683
aPFE0555w_1	PFE0555w	-1.1288	-1.14852	-1.24023	0.170893	2.140683
aPFE0555w_0	PFE0555w	-1.1288	-1.14852	-1.24023	0.170893	2.140683
intPFE0560c_2	PFE0560c	0.228737	0.417667	0.180497	0.025727	-0.09011
aPFE0560c_2	PFE0560c	0.228737	0.417667	0.180497	0.025727	-0.09011
aPFE0560c_1	PFE0560c	0.228737	0.417667	0.180497	0.025727	-0.09011
aPFE0560c_0	PFE0560c	0.228737	0.417667	0.180497	0.025727	-0.09011
intPFE0565w_2	PFE0565w	-0.04454	0.04868	-0.06586	-0.01914	0.13932
aPFE0565w_0	PFE0565w	-0.04454	0.04868	-0.06586	-0.01914	0.13932
intPFE0570w_2	PFE0570w	0.43403	0.23909	-0.12009	-0.34037	-0.27567
aPFE0570w_9	PFE0570w	0.43403	0.23909	-0.12009	-0.34037	-0.27567
aPFE0570w_8	PFE0570w	0.43403	0.23909	-0.12009	-0.34037	-0.27567
aPFE0570w_7	PFE0570w	0.43403	0.23909	-0.12009	-0.34037	-0.27567
aPFE0570w_6	PFE0570w	0.43403	0.23909	-0.12009	-0.34037	-0.27567
aPFE0570w_5	PFE0570w	0.43403	0.23909	-0.12009	-0.34037	-0.27567
aPFE0570w_4	PFE0570w	0.43403	0.23909	-0.12009	-0.34037	-0.27567
aPFE0570w_3	PFE0570w	0.43403	0.23909	-0.12009	-0.34037	-0.27567
aPFE0570w_2	PFE0570w	0.43403	0.23909	-0.12009	-0.34037	-0.27567
aPFE0570w_14	PFE0570w	0.43403	0.23909	-0.12009	-0.34037	-0.27567
aPFE0570w_13	PFE0570w	0.43403	0.23909	-0.12009	-0.34037	-0.27567
aPFE0570w_12	PFE0570w	0.43403	0.23909	-0.12009	-0.34037	-0.27567
aPFE0570w_11	PFE0570w	0.43403	0.23909	-0.12009	-0.34037	-0.27567
aPFE0570w_10	PFE0570w	0.43403	0.23909	-0.12009	-0.34037	-0.27567
aPFE0570w_1	PFE0570w	0.43403	0.23909	-0.12009	-0.34037	-0.27567
aPFE0570w_0	PFE0570w	0.43403	0.23909	-0.12009	-0.34037	-0.27567
intPFE0580w_2	PFE0575c	-0.01457	0.27586	0.43429	0.02571	-0.56886
aPFE0575c_0	PFE0575c	0.49764	0.61681	-0.61361	-1.21739	-0.45581
intPFE0575c_2	PFE0580w	0.49764	0.61681	-0.61361	-1.21739	-0.45581
aPFE0580w_0	PFE0580w	-0.01457	0.27586	0.43429	0.02571	-0.56886
intPFE0585c_3	PFE0585c	-0.71752	0.00162	0.27326	0.47074	0.44538
aPFE0585c_1	PFE0585c	-0.71752	0.00162	0.27326	0.47074	0.44538
aPFE0585c_0	PFE0585c	-0.71752	0.00162	0.27326	0.47074	0.44538
intPFE0595w_2	PFE0590w	-0.66776	0.037773	0.796463	0.744203	-0.09611
intPFE0590w_2	PFE0590w	0.147837	1.148307	0.981897	0.024937	-1.04647
aPFE0590w_1	PFE0590w	0.147837	1.148307	0.981897	0.024937	-1.04647
aPFE0590w_0	PFE0590w	0.147837	1.148307	0.981897	0.024937	-1.04647
aPFE0595w_0	PFE0595w	-0.66776	0.037773	0.796463	0.744203	-0.09611
aPFE0600c_0	PFE0600c	-0.63853	-0.24734	0.501687	0.835647	0.381677
intPFE0605c_2	PFE0605c	-0.41137	0.425323	1.288603	0.710063	-0.87666
intPFE0600c_2	PFE0605c	-0.63853	-0.24734	0.501687	0.835647	0.381677
aPFE0605c_1	PFE0605c	-0.41137	0.425323	1.288603	0.710063	-0.87666
aPFE0605c_0	PFE0605c	-0.41137	0.425323	1.288603	0.710063	-0.87666

intPFE0610c_2	PFE0610c	0.577487	0.507557	0.374877	-0.02654	-0.58122
aPFE0610c_0	PFE0610c	0.577487	0.507557	0.374877	-0.02654	-0.58122
intPFE0615w_2	PFE0615w	1.459503	1.598483	0.446543	-0.79888	-1.43782
aPFE0615w_0	PFE0615w	1.459503	1.598483	0.446543	-0.79888	-1.43782
intPFE0620c_2	PFE0620c	-1.03197	-0.49414	0.261777	0.598557	0.432477
aPFE0620c_1	PFE0620c	-1.03197	-0.49414	0.261777	0.598557	0.432477
aPFE0620c_0	PFE0620c	-1.03197	-0.49414	0.261777	0.598557	0.432477
intPFE0625w_2	PFE0625w	-0.75036	-0.25222	0.035433	0.439233	0.675883
aPFE0625w_0	PFE0625w	-0.75036	-0.25222	0.035433	0.439233	0.675883
intPFE0630c_2	PFE0630c	-0.71528	0.094253	1.226063	0.952363	-0.51797
aPFE0630c_0	PFE0630c	-0.71528	0.094253	1.226063	0.952363	-0.51797
intPFE0640w_2	PFE0635c	-0.1959	0.30548	0.12103	0.08097	0.16852
aPFE0635c_2	PFE0635c	0.114062	0.853692	0.867942	0.101392	-0.90803
aPFE0635c_1	PFE0635c	0.114062	0.853692	0.867942	0.101392	-0.90803
aPFE0635c_0	PFE0635c	0.114062	0.853692	0.867942	0.101392	-0.90803
intPFE0645w_2	PFE0640w	-0.18768	0.22539	0.11121	0.11979	0.18861
intPFE0635c_2	PFE0640w	0.114062	0.853692	0.867942	0.101392	-0.90803
aPFE0640w_0	PFE0640w	-0.1959	0.30548	0.12103	0.08097	0.16852
aPFE0645w_0	PFE0645w	-0.18768	0.22539	0.11121	0.11979	0.18861
aPFE0650c_0	PFE0650c	-0.077	-0.5283	-0.52004	-0.19472	0.334795
intPFE0655w_2	PFE0655w	0.26402	0.46809	0.06022	-0.24422	-0.25269
intPFE0650c_2	PFE0655w	-0.077	-0.5283	-0.52004	-0.19472	0.334795
aPFE0655w_4	PFE0655w	0.26402	0.46809	0.06022	-0.24422	-0.25269
aPFE0655w_3	PFE0655w	0.26402	0.46809	0.06022	-0.24422	-0.25269
aPFE0655w_2	PFE0655w	0.26402	0.46809	0.06022	-0.24422	-0.25269
aPFE0655w_1	PFE0655w	0.26402	0.46809	0.06022	-0.24422	-0.25269
aPFE0655w_0	PFE0655w	0.26402	0.46809	0.06022	-0.24422	-0.25269
intPFE0660c_2	PFE0660c	-0.80582	-0.32487	1.090747	1.138587	-0.23979
aPFE0660c_0	PFE0660c	-0.80582	-0.32487	1.090747	1.138587	-0.23979
intPFE0670w_2	PFE0665c	0.88374	0.91529	0.64542	-0.15842	-1.10529
aPFE0665c_0	PFE0665c	0.228228	0.107218	0.451258	0.225878	-0.49308
intPFE0665c_2	PFE0670w	0.228228	0.107218	0.451258	0.225878	-0.49308
aPFE0670w_0	PFE0670w	0.88374	0.91529	0.64542	-0.15842	-1.10529
intPFE0675c_2	PFE0675c	-1.75009	-1.68354	0.08834	1.43966	1.45854
aPFE0675c_1	PFE0675c	-1.75009	-1.68354	0.08834	1.43966	1.45854
aPFE0675c_0	PFE0675c	-1.75009	-1.68354	0.08834	1.43966	1.45854
intPFE0680w_2	PFE0680w	-0.94774	-0.74027	0.113037	0.704297	0.671607
aPFE0685w_1	PFE0680w	0.05699	-0.46193	-0.00664	0.30714	0.14593
aPFE0685w_0	PFE0680w	0.05699	-0.46193	-0.00664	0.30714	0.14593
aPFE0680w_0	PFE0680w	-0.94774	-0.74027	0.113037	0.704297	0.671607
intPFE0695w_2	PFE0690c	0.76637	-0.37683	-0.26235	0.02435	0.01083
intPFE0690c_2	PFE0690c	-0.2326	-1.38798	-0.04803	0.977367	0.706317
aPFE0690c_0	PFE0690c	-0.2326	-1.38798	-0.04803	0.977367	0.706317
aPFE0695w_0	PFE0695w	0.76637	-0.37683	-0.26235	0.02435	0.01083
intPFE0700c_2	PFE0700c	-0.84145	-0.99474	-0.33702	0.43302	0.88474
aPFE0700c_0	PFE0700c	-0.84145	-0.99474	-0.33702	0.43302	0.88474
intPFE0710w_2	PFE0705c	-0.05365	0.295277	-0.15354	-0.14812	0.274057

aPFE0705c_2	PFE0705c	-1.04737	-1.06012	-0.43676	0.510538	1.208898
aPFE0705c_1	PFE0705c	-1.04737	-1.06012	-0.43676	0.510538	1.208898
intPFE0705c_2	PFE0710w	-1.04737	-1.06012	-0.43676	0.510538	1.208898
aPFE0710w_1	PFE0710w	-0.05365	0.295277	-0.15354	-0.14812	0.274057
aPFE0710w_0	PFE0710w	-0.05365	0.295277	-0.15354	-0.14812	0.274057
intPFE0715w_2	PFE0715w	-1.24789	-0.48156	0.371613	0.742053	0.526723
aPFE0715w_1	PFE0715w	-1.24789	-0.48156	0.371613	0.742053	0.526723
aPFE0715w_0	PFE0715w	-1.24789	-0.48156	0.371613	0.742053	0.526723
aPFE0725c_0	PFE0725c	-0.38656	-0.61922	-0.29106	0.33306	0.74922
intPFE0730c_2	PFE0730c	0.676317	1.063437	0.642637	-0.2063	-1.0041
intPFE0725c_2	PFE0730c	-0.38656	-0.61922	-0.29106	0.33306	0.74922
aPFE0730c_0	PFE0730c	0.676317	1.063437	0.642637	-0.2063	-1.0041
intPFE0735w_2	PFE0735w	0.045903	-0.19353	-0.25546	0.112123	0.492193
aPFE0735w_2	PFE0735w	0.045903	-0.19353	-0.25546	0.112123	0.492193
aPFE0735w_1	PFE0735w	0.045903	-0.19353	-0.25546	0.112123	0.492193
aPFE0735w_0	PFE0735w	0.045903	-0.19353	-0.25546	0.112123	0.492193
intPFE0740c_2	PFE0740c	0.717887	0.704577	-0.00865	-0.36001	-0.29374
aPFE0740c_1	PFE0740c	0.717887	0.704577	-0.00865	-0.36001	-0.29374
aPFE0740c_0	PFE0740c	0.717887	0.704577	-0.00865	-0.36001	-0.29374
intPFE0745w_2	PFE0745w	0.554927	0.616887	0.199227	-0.22039	-0.46955
aPFE0745w_1	PFE0745w	0.554927	0.616887	0.199227	-0.22039	-0.46955
aPFE0745w_0	PFE0745w	0.554927	0.616887	0.199227	-0.22039	-0.46955
intPFE0750c_2	PFE0750c	-0.31819	0.024947	0.510617	0.436217	-0.15911
aPFE0750c_1	PFE0750c	-0.31819	0.024947	0.510617	0.436217	-0.15911
aPFE0750c_0	PFE0750c	-0.31819	0.024947	0.510617	0.436217	-0.15911
intPFE0760w_2	PFE0755c	-0.29474	-0.79729	-0.21492	0.455257	0.643627
aPFE0755c_1	PFE0755c	-0.76954	-0.3853	0.115103	0.555563	0.621963
aPFE0755c_0	PFE0755c	-0.76954	-0.3853	0.115103	0.555563	0.621963
intPFE0755c_2	PFE0760w	-0.76954	-0.3853	0.115103	0.555563	0.621963
aPFE0760w_0	PFE0760w	-0.29474	-0.79729	-0.21492	0.455257	0.643627
intPFE0765w_2	PFE0765w	-0.1142	0.193485	-0.35009	-0.20542	0.500765
aPFE0765w_3	PFE0765w	-0.1142	0.193485	-0.35009	-0.20542	0.500765
aPFE0765w_2	PFE0765w	-0.1142	0.193485	-0.35009	-0.20542	0.500765
aPFE0765w_1	PFE0765w	-0.1142	0.193485	-0.35009	-0.20542	0.500765
aPFE0765w_0	PFE0765w	-0.1142	0.193485	-0.35009	-0.20542	0.500765
intPFE0770w_2	PFE0770w	0.840457	0.115727	-1.16845	-1.34771	-0.08239
aPFE0770w_1	PFE0770w	0.840457	0.115727	-1.16845	-1.34771	-0.08239
aPFE0770w_0	PFE0770w	0.840457	0.115727	-1.16845	-1.34771	-0.08239
intPFE0775c_2	PFE0775c	1.324633	-0.37668	-0.39315	-0.14219	-0.18516
aPFE0775c_1	PFE0775c	1.324633	-0.37668	-0.39315	-0.14219	-0.18516
aPFE0775c_0	PFE0775c	1.324633	-0.37668	-0.39315	-0.14219	-0.18516
intPFE0780w_2	PFE0780w	-0.27699	-0.27709	-0.20082	0.205983	0.584753
aPFE0780w_1	PFE0780w	-0.27699	-0.27709	-0.20082	0.205983	0.584753
aPFE0780w_0	PFE0780w	-0.27699	-0.27709	-0.20082	0.205983	0.584753
intPFE0785c_2	PFE0785c	-0.93589	-1.72304	-1.97291	-0.16525	2.532377
aPFE0785c_1	PFE0785c	-0.93589	-1.72304	-1.97291	-0.16525	2.532377
aPFE0785c_0	PFE0785c	-0.93589	-1.72304	-1.97291	-0.16525	2.532377

intPFE0790c_2	PFE0790c					
aPFE0790c_0	PFE0790c					
aPFE0795c_0	PFE0795c	-1.62221	-1.02393	0.28386	1.00814	0.84993
intPFE0805w_2	PFE0800w	-1.15249	-1.03284	0.276045	0.939705	0.599085
intPFE0800w_2	PFE0800w	-1.39635	-0.08524	0.972237	0.675597	-0.36042
aPFE0800w_1	PFE0800w	-1.39635	-0.08524	0.972237	0.675597	-0.36042
aPFE0800w_0	PFE0800w	-1.39635	-0.08524	0.972237	0.675597	-0.36042
aPFE0805w_3	PFE0805w	-1.15249	-1.03284	0.276045	0.939705	0.599085
aPFE0805w_2	PFE0805w	-1.15249	-1.03284	0.276045	0.939705	0.599085
aPFE0805w_1	PFE0805w	-1.15249	-1.03284	0.276045	0.939705	0.599085
aPFE0805w_0	PFE0805w	-1.15249	-1.03284	0.276045	0.939705	0.599085
intPFE0810c_2	PFE0810c	0.050557	0.555207	0.793097	0.343237	-0.58587
aPFE0810c_0	PFE0810c	0.050557	0.555207	0.793097	0.343237	-0.58587
intPFE0815w_2	PFE0815w	0.203773	0.565633	0.574983	-0.01432	-0.76647
aPFE0815w_1	PFE0815w	0.203773	0.565633	0.574983	-0.01432	-0.76647
aPFE0815w_0	PFE0815w	0.203773	0.565633	0.574983	-0.01432	-0.76647
intPFE0825w_2	PFE0820c	-0.4294	0.97951	0.18297	-0.38147	-0.11151
aPFE0820c_0	PFE0820c	-0.27066	-0.2657	-0.17908	0.084417	0.368037
intPFE0820c_2	PFE0825w	-0.27066	-0.2657	-0.17908	0.084417	0.368037
aPFE0825w_1	PFE0825w	-0.4294	0.97951	0.18297	-0.38147	-0.11151
aPFE0825w_0	PFE0825w	-0.4294	0.97951	0.18297	-0.38147	-0.11151
intPFE0830c_2	PFE0830c	-0.91635	-0.63224	0.06265	0.52291	0.55346
aPFE0830c_2	PFE0830c	-0.91635	-0.63224	0.06265	0.52291	0.55346
aPFE0830c_1	PFE0830c	-0.91635	-0.63224	0.06265	0.52291	0.55346
aPFE0830c_0	PFE0830c	-0.91635	-0.63224	0.06265	0.52291	0.55346
intPFE0835w_3	PFE0835w	0.315943	0.249793	-0.4391	-0.61124	-0.05063
aPFE0835w_1	PFE0835w	0.315943	0.249793	-0.4391	-0.61124	-0.05063
aPFE0835w_0	PFE0835w	0.315943	0.249793	-0.4391	-0.61124	-0.05063
intPFE0840c_2	PFE0840c	-0.47888	-0.60159	-0.86532	-0.00743	1.33384
aPFE0840c_3	PFE0840c	-0.47888	-0.60159	-0.86532	-0.00743	1.33384
aPFE0840c_2	PFE0840c	-0.47888	-0.60159	-0.86532	-0.00743	1.33384
aPFE0840c_1	PFE0840c	-0.47888	-0.60159	-0.86532	-0.00743	1.33384
aPFE0840c_0	PFE0840c	-0.47888	-0.60159	-0.86532	-0.00743	1.33384
intPFE0845c_2	PFE0845c	1.013543	0.859263	0.783473	0.005193	-1.1936
aPFE0845c_0	PFE0845c	1.013543	0.859263	0.783473	0.005193	-1.1936
intPFE0850c_2	PFE0850c	0.050003	0.451963	0.791083	0.451583	-0.4883
aPFE0850c_0	PFE0850c	0.050003	0.451963	0.791083	0.451583	-0.4883
aPFE0855c_1	PFE0855c	0.72815	0.18327	0.20872	-0.26772	-0.90477
aPFE0855c_0	PFE0855c	0.72815	0.18327	0.20872	-0.26772	-0.90477
intPFE0860c_2	PFE0860c	1.63007	0.89965	-0.32331	-1.34519	-1.36865
intPFE0855c_2	PFE0860c	0.72815	0.18327	0.20872	-0.26772	-0.90477
aPFE0860c_1	PFE0860c	1.63007	0.89965	-0.32331	-1.34519	-1.36865
aPFE0860c_0	PFE0860c	1.63007	0.89965	-0.32331	-1.34519	-1.36865
intPFE0865c_3	PFE0865c	0.736877	0.448597	0.269307	-0.39597	-1.03426
aPFE0865c_0	PFE0865c	0.736877	0.448597	0.269307	-0.39597	-1.03426
intPFE0870w_2	PFE0870w	-0.78098	-0.43559	0.273677	0.684157	0.494427
aPFE0870w_1	PFE0870w	-0.78098	-0.43559	0.273677	0.684157	0.494427

aPFE0870w_0	PFE0870w	-0.78098	-0.43559	0.273677	0.684157	0.494427
intPFE0875c_2	PFE0875c	0.921187	0.678067	-0.47213	-0.84253	-0.25323
aPFE0875c_1	PFE0875c	0.921187	0.678067	-0.47213	-0.84253	-0.25323
aPFE0875c_0	PFE0875c	0.921187	0.678067	-0.47213	-0.84253	-0.25323
intPFE0880c_2	PFE0880c					
aPFE0880c_0	PFE0880c					
intPFE0885w_2	PFE0885w	0.064963	0.445193	0.668373	0.244793	-0.58303
aPFE0885w_1	PFE0885w	0.064963	0.445193	0.668373	0.244793	-0.58303
aPFE0885w_0	PFE0885w	0.064963	0.445193	0.668373	0.244793	-0.58303
intPFE0890c_2	PFE0890c	-0.09734	-0.41129	-0.21824	0.138978	0.366818
aPFE0890c_0	PFE0890c	-0.09734	-0.41129	-0.21824	0.138978	0.366818
intPFE0900w_2	PFE0895c	1.0232	0.67899	-0.01348	-0.53352	-0.66399
aPFE0895c_1	PFE0895c	0.113097	-0.52348	-1.19602	-0.51314	1.036317
aPFE0895c_0	PFE0895c	0.113097	-0.52348	-1.19602	-0.51314	1.036317
intPFE0895c_2	PFE0900w	0.113097	-0.52348	-1.19602	-0.51314	1.036317
aPFE0900w_0	PFE0900w	1.0232	0.67899	-0.01348	-0.53352	-0.66399
intPFE0910w_2	PFE0905w	-0.4191	-0.22813	0.63383	0.58084	-0.30315
intPFE0905w_2	PFE0905w	-0.79744	0.121888	0.654718	0.528838	-0.06
aPFE0905w_2	PFE0905w	-0.79744	0.121888	0.654718	0.528838	-0.06
aPFE0905w_1	PFE0905w	-0.79744	0.121888	0.654718	0.528838	-0.06
aPFE0905w_0	PFE0905w	-0.79744	0.121888	0.654718	0.528838	-0.06
aPFE0910w_0	PFE0910w	-0.4191	-0.22813	0.63383	0.58084	-0.30315
intPFE0915c_2	PFE0915c	-1.41537	-0.74524	0.582727	1.105697	0.565187
aPFE0915c_0	PFE0915c	-1.41537	-0.74524	0.582727	1.105697	0.565187
aPFE0920c_0	PFE0920c	0.205893	0.068513	-0.08254	-0.0928	-0.01085
intPFE0925c_2	PFE0925c	0.282113	0.391453	-0.02866	-0.17118	-0.03729
intPFE0920c_2	PFE0925c	0.205893	0.068513	-0.08254	-0.0928	-0.01085
aPFE0925c_1	PFE0925c	0.282113	0.391453	-0.02866	-0.17118	-0.03729
aPFE0925c_0	PFE0925c	0.282113	0.391453	-0.02866	-0.17118	-0.03729
intPFE0930w_2	PFE0930w	-0.68063	-1.00436	-0.69104	0.368203	1.376523
aPFE0930w_1	PFE0930w	-0.68063	-1.00436	-0.69104	0.368203	1.376523
aPFE0930w_0	PFE0930w	-0.68063	-1.00436	-0.69104	0.368203	1.376523
intPFE0935c_2	PFE0935c	0.356708	0.523458	-0.24124	-0.44209	0.009208
aPFE0935c_2	PFE0935c	0.356708	0.523458	-0.24124	-0.44209	0.009208
aPFE0935c_1	PFE0935c	0.356708	0.523458	-0.24124	-0.44209	0.009208
aPFE0935c_0	PFE0935c	0.356708	0.523458	-0.24124	-0.44209	0.009208
intPFE0940c_2	PFE0940c	-0.26081	0.212792	-0.22818	-0.12022	0.466812
aPFE0945c_0	PFE0940c					
aPFE0940c_0	PFE0940c	-0.26081	0.212792	-0.22818	-0.12022	0.466812
intPFE0950c_2	PFE0950c	0.465443	-0.03923	0.157443	-0.02278	-0.50411
aPFE0950c_0	PFE0950c	0.465443	-0.03923	0.157443	-0.02278	-0.50411
intPFE0955w_2	PFE0955w	-1.2673	-0.57952	1.330023	1.297643	-0.45582
aPFE0955w_0	PFE0955w	-1.2673	-0.57952	1.330023	1.297643	-0.45582
intPFE0960w_2	PFE0960w	-0.55657	-0.51119	-0.06964	0.364303	0.518853
aPFE0960w_0	PFE0960w	-0.55657	-0.51119	-0.06964	0.364303	0.518853
intPFE0965c_1	PFE0965c	-0.75008	-0.4266	0.130707	0.470627	0.431937
aPFE0965c_0	PFE0965c	-0.75008	-0.4266	0.130707	0.470627	0.431937

intPFE0970w_2	PFE0970w	-0.53926	-1.02569	0.10988	0.88012	0.61469
aPFE0970w_1	PFE0970w	-0.53926	-1.02569	0.10988	0.88012	0.61469
aPFE0970w_0	PFE0970w	-0.53926	-1.02569	0.10988	0.88012	0.61469
intPFE0975c_1	PFE0975c	0.118933	0.180313	0.654363	0.502303	-0.36565
aPFE0975c_0	PFE0975c	0.118933	0.180313	0.654363	0.502303	-0.36565
intPFE0980c_2	PFE0980c	-0.69061	-0.00294	0.732758	0.606158	-0.20589
aPFE0980c_3	PFE0980c	-0.69061	-0.00294	0.732758	0.606158	-0.20589
aPFE0980c_2	PFE0980c	-0.69061	-0.00294	0.732758	0.606158	-0.20589
aPFE0980c_1	PFE0980c	-0.69061	-0.00294	0.732758	0.606158	-0.20589
aPFE0980c_0	PFE0980c	-0.69061	-0.00294	0.732758	0.606158	-0.20589
intPFE0985w_2	PFE0985w	0.072373	0.373123	0.244503	0.062003	-0.13063
aPFE0985w_1	PFE0985w	0.072373	0.373123	0.244503	0.062003	-0.13063
aPFE0985w_0	PFE0985w	0.072373	0.373123	0.244503	0.062003	-0.13063
intPFE0990w_2	PFE0990w	0.508183	0.338083	0.166323	-0.25566	-0.63242
aPFE0990w_0	PFE0990w	0.508183	0.338083	0.166323	-0.25566	-0.63242
intPFE0995c_2	PFE0995c	0.178057	0.924237	0.665537	-0.0707	-0.7694
aPFE0995c_1	PFE0995c	0.178057	0.924237	0.665537	-0.0707	-0.7694
aPFE0995c_0	PFE0995c	0.178057	0.924237	0.665537	-0.0707	-0.7694
intPFE1005w_2	PFE1000c	0.08593	0.48636	0.66779	0.35621	-0.39436
intPFE1000c_2	PFE1000c	-0.45437	-0.12417	0.24885	0.40515	0.22017
aPFE1000c_1	PFE1000c	-0.45437	-0.12417	0.24885	0.40515	0.22017
aPFE1000c_0	PFE1000c	-0.45437	-0.12417	0.24885	0.40515	0.22017
aPFE1005w_0	PFE1005w	0.08593	0.48636	0.66779	0.35621	-0.39436
intPFE1010w_2	PFE1010w	0.56444	0.75578	0.67844	0.00756	-0.91678
aPFE1010w_1	PFE1010w	0.56444	0.75578	0.67844	0.00756	-0.91678
aPFE1010w_0	PFE1010w	0.56444	0.75578	0.67844	0.00756	-0.91678
aPFE1015c_1	PFE1015c	0.1206	0.13299	0.55626	0.42824	-0.32899
aPFE1015c_0	PFE1015c	0.1206	0.13299	0.55626	0.42824	-0.32899
intPFE1020w_3	PFE1020w	-0.1417	0.01249	0.64052	0.61148	-0.20149
intPFE1015c_2	PFE1020w	0.1206	0.13299	0.55626	0.42824	-0.32899
aPFE1020w_0	PFE1020w	-0.1417	0.01249	0.64052	0.61148	-0.20149
intPFE1025c_2	PFE1025c	0.148677	-0.55539	0.345287	0.519047	-0.27427
aPFE1025c_0	PFE1025c	0.148677	-0.55539	0.345287	0.519047	-0.27427
intPFE1030c_2	PFE1030c	0.10402	0.00902	-0.10899	-0.15009	-0.05398
aPFE1030c_0	PFE1030c	0.10402	0.00902	-0.10899	-0.15009	-0.05398
intPFE1035c_2	PFE1035c	0.492163	0.752183	0.996123	0.087543	-1.35752
aPFE1035c_0	PFE1035c	0.492163	0.752183	0.996123	0.087543	-1.35752
intPFE1040c_2	PFE1040c	0.847247	0.632757	0.439977	-0.09714	-0.81292
aPFE1040c_1	PFE1040c	0.847247	0.632757	0.439977	-0.09714	-0.81292
aPFE1040c_0	PFE1040c	0.847247	0.632757	0.439977	-0.09714	-0.81292
intPFE1050w_2	PFE1045c	-0.66136	-0.1647	-0.00711	0.431773	0.771363
aPFE1045c_3	PFE1045c	-0.65234	-0.02605	0.163343	0.291073	0.337213
aPFE1045c_2	PFE1045c	-0.65234	-0.02605	0.163343	0.291073	0.337213
aPFE1045c_1	PFE1045c	-0.65234	-0.02605	0.163343	0.291073	0.337213
aPFE1045c_0	PFE1045c	-0.65234	-0.02605	0.163343	0.291073	0.337213
intPFE1045c_2	PFE1050w	-0.65234	-0.02605	0.163343	0.291073	0.337213
aPFE1050w_1	PFE1050w	-0.66136	-0.1647	-0.00711	0.431773	0.771363

aPFE1050w_0	PFE1050w	-0.66136	-0.1647	-0.00711	0.431773	0.771363
aPFE1055c_1	PFE1055c	0.320067	0.217677	0.548407	0.283427	-0.54184
aPFE1055c_0	PFE1055c	0.320067	0.217677	0.548407	0.283427	-0.54184
intPFE1055c_2	PFE1060c	0.320067	0.217677	0.548407	0.283427	-0.54184
aPFE1060c_2	PFE1060c	-0.23912	0.436082	-0.05292	-0.04058	0.406582
aPFE1060c_1	PFE1060c	-0.23912	0.436082	-0.05292	-0.04058	0.406582
aPFE1060c_0	PFE1060c	-0.23912	0.436082	-0.05292	-0.04058	0.406582
intPFE1065w_2	PFE1065w	-1.04097	-0.62971	0.521152	0.969332	0.425742
intPFE1060c_2	PFE1065w	-0.23912	0.436082	-0.05292	-0.04058	0.406582
aPFE1065w_0	PFE1065w	-1.04097	-0.62971	0.521152	0.969332	0.425742
intPFE1070c_2	PFE1070c	0.803163	1.174193	0.181603	-0.71444	-0.88953
aPFE1070c_1	PFE1070c	0.803163	1.174193	0.181603	-0.71444	-0.88953
aPFE1070c_0	PFE1070c	0.803163	1.174193	0.181603	-0.71444	-0.88953
intPFE1080w_2	PFE1075c	-0.28964	0.094703	0.587843	0.547323	-0.09704
aPFE1075c_1	PFE1075c	-1.05293	-0.57484	0.54942	0.98758	0.44184
aPFE1075c_0	PFE1075c	-1.05293	-0.57484	0.54942	0.98758	0.44184
intPFE1075c_2	PFE1080w	-1.05293	-0.57484	0.54942	0.98758	0.44184
aPFE1080w_1	PFE1080w	-0.28964	0.094703	0.587843	0.547323	-0.09704
aPFE1080w_0	PFE1080w	-0.28964	0.094703	0.587843	0.547323	-0.09704
intPFE1090w_2	PFE1085w	0.255117	0.233397	-0.58204	-0.54712	0.316937
intPFE1085w_3	PFE1085w	1.110467	0.944157	0.444187	-0.33885	-1.06332
aPFE1085w_1	PFE1085w	1.110467	0.944157	0.444187	-0.33885	-1.06332
aPFE1085w_0	PFE1085w	1.110467	0.944157	0.444187	-0.33885	-1.06332
intPFE1095w_2	PFE1090w	-0.07941	-0.30459	0.41651	0.6506	0.0357
aPFE1090w_1	PFE1090w	0.255117	0.233397	-0.58204	-0.54712	0.316937
aPFE1090w_0	PFE1090w	0.255117	0.233397	-0.58204	-0.54712	0.316937
aPFE1095w_2	PFE1095w	-0.07941	-0.30459	0.41651	0.6506	0.0357
aPFE1095w_1	PFE1095w	-0.07941	-0.30459	0.41651	0.6506	0.0357
aPFE1095w_0	PFE1095w	-0.07941	-0.30459	0.41651	0.6506	0.0357
intPFE1100w_2	PFE1100w	-0.19647	0.266377	0.673237	0.408097	-0.37904
aPFE1100w_0	PFE1100w	-0.19647	0.266377	0.673237	0.408097	-0.37904
intPFE1105c_2	PFE1105c	0.016963	0.518193	-0.62113	-0.94371	0.062973
aPFE1105c_1	PFE1105c	0.016963	0.518193	-0.62113	-0.94371	0.062973
aPFE1105c_0	PFE1105c	0.016963	0.518193	-0.62113	-0.94371	0.062973
intPFE1110w_2	PFE1110w	-0.03458	0.329512	0.342402	0.020882	-0.36523
aPFE1110w_0	PFE1110w	-0.03458	0.329512	0.342402	0.020882	-0.36523
intPFE1115c_2	PFE1115c	0.75648	0.5436	0.6688	-0.1758	-1.3796
aPFE1115c_0	PFE1115c	0.75648	0.5436	0.6688	-0.1758	-1.3796
intPFE1120w_2	PFE1120w	-0.31651	-0.36308	-1.48256	-0.86355	1.286158
aPFE1120w_9	PFE1120w	-0.31651	-0.36308	-1.48256	-0.86355	1.286158
aPFE1120w_8	PFE1120w	-0.31651	-0.36308	-1.48256	-0.86355	1.286158
aPFE1120w_7	PFE1120w	-0.31651	-0.36308	-1.48256	-0.86355	1.286158
aPFE1120w_6	PFE1120w	-0.31651	-0.36308	-1.48256	-0.86355	1.286158
aPFE1120w_5	PFE1120w	-0.31651	-0.36308	-1.48256	-0.86355	1.286158
aPFE1120w_4	PFE1120w	-0.31651	-0.36308	-1.48256	-0.86355	1.286158
aPFE1120w_3	PFE1120w	-0.31651	-0.36308	-1.48256	-0.86355	1.286158
aPFE1120w_2	PFE1120w	-0.31651	-0.36308	-1.48256	-0.86355	1.286158

aPFE1120w_13	PFE1120w	-0.31651	-0.36308	-1.48256	-0.86355	1.286158
aPFE1120w_12	PFE1120w	-0.31651	-0.36308	-1.48256	-0.86355	1.286158
aPFE1120w_11	PFE1120w	-0.31651	-0.36308	-1.48256	-0.86355	1.286158
aPFE1120w_10	PFE1120w	-0.31651	-0.36308	-1.48256	-0.86355	1.286158
aPFE1120w_1	PFE1120w	-0.31651	-0.36308	-1.48256	-0.86355	1.286158
aPFE1120w_0	PFE1120w	-0.31651	-0.36308	-1.48256	-0.86355	1.286158
intPFE1125w_2	PFE1125w	-0.44712	-1.2344	-0.59719	0.512527	1.189737
aPFE1125w_0	PFE1125w	-0.44712	-1.2344	-0.59719	0.512527	1.189737
intPFE1130w_2	PFE1130w	-0.23697	-0.85916	-1.39918	-0.49482	1.32916
aPFE1130w_1	PFE1130w	-0.23697	-0.85916	-1.39918	-0.49482	1.32916
aPFE1130w_0	PFE1130w	-0.23697	-0.85916	-1.39918	-0.49482	1.32916
intPFE1135w_2	PFE1135w	-0.34832	-0.3484	0.137783	0.545883	0.450063
aPFE1135w_0	PFE1135w	-0.34832	-0.3484	0.137783	0.545883	0.450063
intPFE1140c_2	PFE1140c	0.416117	0.323407	0.388187	0.089147	-0.50107
aPFE1140c_0	PFE1140c	0.416117	0.323407	0.388187	0.089147	-0.50107
intPFE1145w_2	PFE1145w	0.284075	0.277805	-0.58786	-0.66348	0.173025
aPFE1145w_2	PFE1145w	0.284075	0.277805	-0.58786	-0.66348	0.173025
aPFE1145w_1	PFE1145w	0.284075	0.277805	-0.58786	-0.66348	0.173025
aPFE1145w_0	PFE1145w	0.284075	0.277805	-0.58786	-0.66348	0.173025
intPFE1150w_2	PFE1150w	0.8379	0.31	0.26126	-0.47326	-1.2615
aPFE1150w_2	PFE1150w	0.8379	0.31	0.26126	-0.47326	-1.2615
aPFE1150w_1	PFE1150w	0.8379	0.31	0.26126	-0.47326	-1.2615
aPFE1150w_0	PFE1150w	0.8379	0.31	0.26126	-0.47326	-1.2615
intPFE1160w_2	PFE1155c	0.527087	-0.09091	0.338087	0.244247	-0.48975
aPFE1155c_1	PFE1155c	-0.01047	-0.21365	0.403297	0.436537	-0.22351
aPFE1155c_0	PFE1155c	-0.01047	-0.21365	0.403297	0.436537	-0.22351
intPFE1155c_2	PFE1160w	-0.01047	-0.21365	0.403297	0.436537	-0.22351
aPFE1160w_1	PFE1160w	0.527087	-0.09091	0.338087	0.244247	-0.48975
aPFE1160w_0	PFE1160w	0.527087	-0.09091	0.338087	0.244247	-0.48975
intPFE1165c_2	PFE1165c	-0.05133	-1.05233	-0.63783	0.299167	0.933667
aPFE1165c_0	PFE1165c	-0.05133	-1.05233	-0.63783	0.299167	0.933667
intPFE1170w_3	PFE1170w	-0.26141	-0.13745	-0.02936	0.018523	0.077113
aPFE1170w_1	PFE1170w	-0.26141	-0.13745	-0.02936	0.018523	0.077113
aPFE1170w_0	PFE1170w	-0.26141	-0.13745	-0.02936	0.018523	0.077113
intPFE1175w_2	PFE1173c	-0.33772	-0.77306	-0.12369	0.474815	0.536185
aPFE1173c_0	PFE1173c	-0.71941	-0.95995	0.453917	1.044417	0.334287
intPFE1173c_2	PFE1175w	-0.71941	-0.95995	0.453917	1.044417	0.334287
aPFE1175w_0	PFE1175w	-0.33772	-0.77306	-0.12369	0.474815	0.536185
intPFE1180c_2	PFE1180c	0.633912	0.225452	0.259932	-0.17927	-0.80645
aPFE1180c_2	PFE1180c	0.633912	0.225452	0.259932	-0.17927	-0.80645
aPFE1180c_1	PFE1180c	0.633912	0.225452	0.259932	-0.17927	-0.80645
aPFE1180c_0	PFE1180c	0.633912	0.225452	0.259932	-0.17927	-0.80645
intPFE1185w_2	PFE1185w	1.535943	1.027763	-0.29954	-0.9813	-0.7496
aPFE1185w_1	PFE1185w	1.535943	1.027763	-0.29954	-0.9813	-0.7496
aPFE1185w_0	PFE1185w	1.535943	1.027763	-0.29954	-0.9813	-0.7496
intPFE1190c_2	PFE1190c	0.997537	0.574297	-0.2846	-0.69406	-0.46896
aPFE1190c_0	PFE1190c	0.997537	0.574297	-0.2846	-0.69406	-0.46896

intPFE1195w_2	PFE1195w	0.313003	0.464483	0.284293	-0.10163	-0.46832
aPFE1195w_1	PFE1195w	0.313003	0.464483	0.284293	-0.10163	-0.46832
aPFE1195w_0	PFE1195w	0.313003	0.464483	0.284293	-0.10163	-0.46832
intPFE1200w_2	PFE1200w	0.948847	0.688247	-0.24698	-0.74418	-0.52091
aPFE1200w_1	PFE1200w	0.948847	0.688247	-0.24698	-0.74418	-0.52091
aPFE1200w_0	PFE1200w	0.948847	0.688247	-0.24698	-0.74418	-0.52091
intPFE1205c_2	PFE1205c	-0.07219	-0.26107	-0.00637	0.142033	0.074733
aPFE1205c_0	PFE1205c	-0.07219	-0.26107	-0.00637	0.142033	0.074733
intPFE1210c_2	PFE1210c	0.422593	0.166023	0.399953	0.264713	-0.36636
aPFE1210c_1	PFE1210c	0.422593	0.166023	0.399953	0.264713	-0.36636
aPFE1210c_0	PFE1210c	0.422593	0.166023	0.399953	0.264713	-0.36636
intPFE1215c_2	PFE1215c	0.494167	0.342167	0.556417	0.100917	-0.80533
aPFE1215c_1	PFE1215c	0.494167	0.342167	0.556417	0.100917	-0.80533
aPFE1215c_0	PFE1215c	0.494167	0.342167	0.556417	0.100917	-0.80533
intPFE1220w_3	PFE1220w	0.142187	-0.05593	0.221867	0.178467	-0.22373
aPFE1220w_0	PFE1220w	0.142187	-0.05593	0.221867	0.178467	-0.22373
intPFE1225w_2	PFE1225w	-1.04817	-0.84517	-0.55267	0.46267	1.49317
aPFE1225w_0	PFE1225w	-1.04817	-0.84517	-0.55267	0.46267	1.49317
intPFE1230c_2	PFE1230c	-0.47172	-0.25137	1.287737	1.114597	-0.69929
aPFE1230c_0	PFE1230c	-0.47172	-0.25137	1.287737	1.114597	-0.69929
intPFE1235c_2	PFE1235c	0.507263	0.418703	0.325603	-0.39644	-1.08154
aPFE1235c_1	PFE1235c	0.507263	0.418703	0.325603	-0.39644	-1.08154
aPFE1235c_0	PFE1235c	0.507263	0.418703	0.325603	-0.39644	-1.08154
intPFE1240w_2	PFE1240w	0.98818	0.8761	0.77581	-0.26531	-1.5656
aPFE1240w_1	PFE1240w	0.98818	0.8761	0.77581	-0.26531	-1.5656
aPFE1240w_0	PFE1240w	0.98818	0.8761	0.77581	-0.26531	-1.5656
intPFE1245w_2	PFE1245w	-1.8316	-0.977	1.802483	1.831683	-0.59784
aPFE1245w_1	PFE1245w	-1.8316	-0.977	1.802483	1.831683	-0.59784
aPFE1245w_0	PFE1245w	-1.8316	-0.977	1.802483	1.831683	-0.59784
intPFE1250w_2	PFE1250w	0.499383	-0.42241	-0.39019	-0.22015	-0.06293
aPFE1250w_1	PFE1250w	0.499383	-0.42241	-0.39019	-0.22015	-0.06293
aPFE1250w_0	PFE1250w	0.499383	-0.42241	-0.39019	-0.22015	-0.06293
intPFE1255w_2	PFE1255w	-0.12691	-1.07295	-0.90158	0.052917	1.059287
aPFE1255w_2	PFE1255w	-0.12691	-1.07295	-0.90158	0.052917	1.059287
aPFE1255w_1	PFE1255w	-0.12691	-1.07295	-0.90158	0.052917	1.059287
aPFE1255w_0	PFE1255w	-0.12691	-1.07295	-0.90158	0.052917	1.059287
intPFE1260c_2	PFE1260c	1.091757	-0.24827	-1.09846	-1.4352	-0.66839
aPFE1260c_1	PFE1260c	1.091757	-0.24827	-1.09846	-1.4352	-0.66839
aPFE1260c_0	PFE1260c	1.091757	-0.24827	-1.09846	-1.4352	-0.66839
intPFE1265w_2	PFE1265w	0.16008	-0.77138	-0.69	-0.062	0.60438
aPFE1265w_1	PFE1265w	0.16008	-0.77138	-0.69	-0.062	0.60438
aPFE1265w_0	PFE1265w	0.16008	-0.77138	-0.69	-0.062	0.60438
intPFE1270c_2	PFE1270c	1.049557	1.328857	0.845957	-0.4544	-1.69646
aPFE1270c_2	PFE1270c	1.049557	1.328857	0.845957	-0.4544	-1.69646
aPFE1270c_1	PFE1270c	1.049557	1.328857	0.845957	-0.4544	-1.69646
aPFE1270c_0	PFE1270c	1.049557	1.328857	0.845957	-0.4544	-1.69646
intPFE1280w_2	PFE1275c	-0.43277	-1.30211	-0.32077	0.598107	0.761447

aPFE1275c_1	PFE1275c	-0.30728	-0.52459	0.19368	0.37032	-0.04691
aPFE1275c_0	PFE1275c	-0.30728	-0.52459	0.19368	0.37032	-0.04691
intPFE1275c_2	PFE1280w	-0.30728	-0.52459	0.19368	0.37032	-0.04691
aPFE1280w_0	PFE1280w	-0.43277	-1.30211	-0.32077	0.598107	0.761447
intPFE1285w_2	PFE1285w	1.231457	-0.3423	-2.04489	-2.23377	-0.24036
aPFE1285w_0	PFE1285w	1.231457	-0.3423	-2.04489	-2.23377	-0.24036
intPFE1290w_2	PFE1290w	0.953783	-0.12032	-0.86308	-0.92466	-0.20282
aPFE1290w_0	PFE1290w	0.953783	-0.12032	-0.86308	-0.92466	-0.20282
intPFE1300w_2	PFE1295c	-0.78329	-0.05356	0.704468	0.556788	-0.23095
intPFE1295c_2	PFE1295c	-0.83416	0.323793	0.878413	0.550253	-0.29113
aPFE1295c_1	PFE1295c	-0.83416	0.323793	0.878413	0.550253	-0.29113
aPFE1295c_0	PFE1295c	-0.83416	0.323793	0.878413	0.550253	-0.29113
aPFE1300w_0	PFE1300w	-0.78329	-0.05356	0.704468	0.556788	-0.23095
intPFE1305c_2	PFE1305c-a	-0.81559	-1.48203	-0.44993	0.687767	1.140367
aPFE1305c_1	PFE1305c-a	-0.81559	-1.48203	-0.44993	0.687767	1.140367
aPFE1305c_0	PFE1305c-a	-0.81559	-1.48203	-0.44993	0.687767	1.140367
intPFE1310c_3	PFE1310c	0.512223	0.660873	0.614263	-0.1966	-1.15121
aPFE1310c_0	PFE1310c	0.512223	0.660873	0.614263	-0.1966	-1.15121
intPFE1315w_2	PFE1315w	0.227943	-0.84771	-0.8706	0.026263	0.994373
aPFE1315w_0	PFE1315w	0.227943	-0.84771	-0.8706	0.026263	0.994373
intPFE1320w_2	PFE1320w	1.38791	0.59846	0.03084	-0.43584	-0.77396
aPFE1320w_2	PFE1320w	1.38791	0.59846	0.03084	-0.43584	-0.77396
aPFE1320w_1	PFE1320w	1.38791	0.59846	0.03084	-0.43584	-0.77396
aPFE1320w_0	PFE1320w	1.38791	0.59846	0.03084	-0.43584	-0.77396
intPFE1325w_2	PFE1325w	-1.08828	-0.52449	0.052755	0.501435	0.668535
aPFE1325w_6	PFE1325w	-1.08828	-0.52449	0.052755	0.501435	0.668535
aPFE1325w_5	PFE1325w	-1.08828	-0.52449	0.052755	0.501435	0.668535
aPFE1325w_4	PFE1325w	-1.08828	-0.52449	0.052755	0.501435	0.668535
aPFE1325w_3	PFE1325w	-1.08828	-0.52449	0.052755	0.501435	0.668535
aPFE1325w_2	PFE1325w	-1.08828	-0.52449	0.052755	0.501435	0.668535
aPFE1325w_1	PFE1325w	-1.08828	-0.52449	0.052755	0.501435	0.668535
aPFE1325w_0	PFE1325w	-1.08828	-0.52449	0.052755	0.501435	0.668535
intPFE1330c_2	PFE1330c	-0.67888	-0.76711	-0.02279	0.68129	0.77861
aPFE1330c_1	PFE1330c	-0.67888	-0.76711	-0.02279	0.68129	0.77861
aPFE1330c_0	PFE1330c	-0.67888	-0.76711	-0.02279	0.68129	0.77861
intPFE1335c_2	PFE1335c	-0.17261	0.532023	0.620203	0.250963	-0.34836
aPFE1335c_1	PFE1335c	-0.17261	0.532023	0.620203	0.250963	-0.34836
aPFE1335c_0	PFE1335c	-0.17261	0.532023	0.620203	0.250963	-0.34836
intPFE1340w_3	PFE1340w	0.04509	0.33954	0.32591	0.13709	-0.18054
aPFE1340w_0	PFE1340w	0.04509	0.33954	0.32591	0.13709	-0.18054
intPFE1345c_2	PFE1345c	-1.65572	-1.50991	-0.62544	0.943103	2.106573
aPFE1345c_1	PFE1345c	-1.65572	-1.50991	-0.62544	0.943103	2.106573
aPFE1345c_0	PFE1345c	-1.65572	-1.50991	-0.62544	0.943103	2.106573
intPFE1350c_2	PFE1350c	-0.19573	-0.18635	0.023197	0.326137	0.387687
aPFE1350c_0	PFE1350c	-0.19573	-0.18635	0.023197	0.326137	0.387687
intPFE1355c_3	PFE1355c	-0.6461	-0.52448	-0.02703	0.464367	0.611817
aPFE1355c_1	PFE1355c	-0.6461	-0.52448	-0.02703	0.464367	0.611817

aPFE1355c_0	PFE1355c	-0.6461	-0.52448	-0.02703	0.464367	0.611817
intPFE1360c_2	PFE1360c	0.425587	0.250087	0.400837	-0.0725	-0.81875
aPFE1360c_0	PFE1360c	0.425587	0.250087	0.400837	-0.0725	-0.81875
intPFE1365w_2	PFE1365w	1.248393	0.539053	0.262393	-0.62973	-1.48239
aPFE1365w_0	PFE1365w	1.248393	0.539053	0.262393	-0.62973	-1.48239
intPFE1370w_2	PFE1370w	-0.33337	-0.15318	0.756353	0.754313	-0.24616
aPFE1370w_1	PFE1370w	-0.33337	-0.15318	0.756353	0.754313	-0.24616
aPFE1370w_0	PFE1370w	-0.33337	-0.15318	0.756353	0.754313	-0.24616
intPFE1375c_2	PFE1375c	-0.74674	0.535707	-0.07667	-0.19299	0.457127
aPFE1375c_1	PFE1375c	-0.74674	0.535707	-0.07667	-0.19299	0.457127
aPFE1375c_0	PFE1375c	-0.74674	0.535707	-0.07667	-0.19299	0.457127
intPFE1390w_2	PFE1390w	0.80391	0.45845	0.24686	-0.17936	-0.68395
aPFE1390w_1	PFE1390w	0.80391	0.45845	0.24686	-0.17936	-0.68395
aPFE1390w_0	PFE1390w	0.80391	0.45845	0.24686	-0.17936	-0.68395
intPFE1395c_2	PFE1395c	-0.28847	-0.59468	-0.12215	0.607813	0.835343
aPFE1395c_0	PFE1395c	-0.28847	-0.59468	-0.12215	0.607813	0.835343
intPFE1400c_1	PFE1400c	-0.36362	0.04512	0.06777	0.06323	0.12188
aPFE1400c_1	PFE1400c	-0.36362	0.04512	0.06777	0.06323	0.12188
aPFE1400c_0	PFE1400c	-0.36362	0.04512	0.06777	0.06323	0.12188
intPFE1405c_3	PFE1405c	0.222947	0.297777	0.493697	0.238637	-0.42294
aPFE1405c_1	PFE1405c	0.222947	0.297777	0.493697	0.238637	-0.42294
aPFE1405c_0	PFE1405c	0.222947	0.297777	0.493697	0.238637	-0.42294
intPFE1410c_2	PFE1410c	0.719923	0.243873	-1.50951	-1.61283	0.270793
aPFE1410c_1	PFE1410c	0.719923	0.243873	-1.50951	-1.61283	0.270793
aPFE1410c_0	PFE1410c	0.719923	0.243873	-1.50951	-1.61283	0.270793
intPFE1415w_2	PFE1415w	0.424837	-0.1962	-1.60858	-1.27308	0.767037
aPFE1415w_1	PFE1415w	0.424837	-0.1962	-1.60858	-1.27308	0.767037
aPFE1415w_0	PFE1415w	0.424837	-0.1962	-1.60858	-1.27308	0.767037
intPFE1420w_2	PFE1420w	-0.46954	-0.84732	-0.56886	0.30786	1.09632
aPFE1420w_0	PFE1420w	-0.46954	-0.84732	-0.56886	0.30786	1.09632
aPFE1425c_0	PFE1425c	0.882543	0.356303	0.330893	-0.19423	-0.95464
intPFE1430c_2	PFE1430c	0.53539	0.34206	0.47089	0.10911	-0.65006
intPFE1425c_1	PFE1430c	0.882543	0.356303	0.330893	-0.19423	-0.95464
aPFE1430c_0	PFE1430c	0.53539	0.34206	0.47089	0.10911	-0.65006
intPFE1435c_2	PFE1435c	0.539627	0.899857	0.807537	-0.2117	-1.37802
aPFE1435c_1	PFE1435c	0.539627	0.899857	0.807537	-0.2117	-1.37802
aPFE1435c_0	PFE1435c	0.539627	0.899857	0.807537	-0.2117	-1.37802
intPFE1440c_2	PFE1440c	0.299452	0.399912	0.129572	-0.19814	-0.36797
aPFE1440c_0	PFE1440c	0.299452	0.399912	0.129572	-0.19814	-0.36797
intPFE1445c_2	PFE1445c	0.07276	0.00021	0.17958	-0.01358	-0.37371
aPFE1445c_1	PFE1445c	0.07276	0.00021	0.17958	-0.01358	-0.37371
aPFE1445c_0	PFE1445c	0.07276	0.00021	0.17958	-0.01358	-0.37371
intPFE1455w_2	PFE1450c	-0.10152	-0.54538	0.171017	0.531317	0.153717
aPFE1450c_0	PFE1450c	-0.75223	-1.08283	0.371157	1.151177	0.575167
intPFE1450c_2	PFE1455w	-0.75223	-1.08283	0.371157	1.151177	0.575167
aPFE1455w_1	PFE1455w	-0.10152	-0.54538	0.171017	0.531317	0.153717
aPFE1455w_0	PFE1455w	-0.10152	-0.54538	0.171017	0.531317	0.153717

intPFE1460w_2	PFE1460w	-0.32452	-0.46067	0.013487	0.591047	0.662907
aPFE1460w_0	PFE1460w	-0.32452	-0.46067	0.013487	0.591047	0.662907
intPFE1465w_3	PFE1465w-a	-1.36372	-1.2914	-0.76703	0.489028	1.733408
aPFE1465w_2	PFE1465w-a	-1.36372	-1.2914	-0.76703	0.489028	1.733408
aPFE1465w_1	PFE1465w-a	-1.36372	-1.2914	-0.76703	0.489028	1.733408
aPFE1465w_0	PFE1465w-a	-1.36372	-1.2914	-0.76703	0.489028	1.733408
intPFE1470w_2	PFE1470w	0.23742	0.59888	0.50175	0.10925	-0.43588
aPFE1470w_0	PFE1470w	0.23742	0.59888	0.50175	0.10925	-0.43588
intPFE1485w_2	PFE1480c	-0.53306	-0.19773	-0.29739	0.081165	0.712835
aPFE1480c_1	PFE1480c	-1.11133	0.081133	0.774223	0.518943	-0.21647
aPFE1480c_0	PFE1480c	-1.11133	0.081133	0.774223	0.518943	-0.21647
intPFE1480c_2	PFE1485w	-1.11133	0.081133	0.774223	0.518943	-0.21647
aPFE1485w_2	PFE1485w	-0.53306	-0.19773	-0.29739	0.081165	0.712835
aPFE1485w_1	PFE1485w	-0.53306	-0.19773	-0.29739	0.081165	0.712835
aPFE1485w_0	PFE1485w	-0.53306	-0.19773	-0.29739	0.081165	0.712835
intPFE1490c_3	PFE1490c	0.04902	0.33688	-0.05426	-0.30774	-0.15388
aPFE1495w_0	PFE1490c					
aPFE1490c_0	PFE1490c	0.04902	0.33688	-0.05426	-0.30774	-0.15388
intPFE1500c_2	PFE1500c	0.175717	-0.04834	-0.04784	-0.00484	-0.01034
aPFE1500c_0	PFE1500c	0.175717	-0.04834	-0.04784	-0.00484	-0.01034
intPFE1505w_2	PFE1505w					
aPFE1505w_0	PFE1505w					
intPFE1510c_2	PFE1510c	-1.15677	-1.35715	-0.26495	0.965287	1.414487
aPFE1510c_1	PFE1510c	-1.15677	-1.35715	-0.26495	0.965287	1.414487
aPFE1510c_0	PFE1510c	-1.15677	-1.35715	-0.26495	0.965287	1.414487
intPFE1515w_2	PFE1515w	-0.75644	-0.7928	-0.92424	-0.06898	1.314578
aPFE1515w_2	PFE1515w	-0.75644	-0.7928	-0.92424	-0.06898	1.314578
aPFE1515w_1	PFE1515w	-0.75644	-0.7928	-0.92424	-0.06898	1.314578
aPFE1515w_0	PFE1515w	-0.75644	-0.7928	-0.92424	-0.06898	1.314578
intPFE1525w_2	PFE1520c	-0.31193	0.608123	0.610003	0.159663	-0.36946
aPFE1520c_2	PFE1520c	-0.88934	-0.03398	1.09596	0.80815	-0.52324
aPFE1520c_1	PFE1520c	-0.88934	-0.03398	1.09596	0.80815	-0.52324
aPFE1520c_0	PFE1520c	-0.88934	-0.03398	1.09596	0.80815	-0.52324
intPFE1520c_2	PFE1525w	-0.88934	-0.03398	1.09596	0.80815	-0.52324
aPFE1525w_1	PFE1525w	-0.31193	0.608123	0.610003	0.159663	-0.36946
aPFE1525w_0	PFE1525w	-0.31193	0.608123	0.610003	0.159663	-0.36946
aPFE1530c_1	PFE1530c	0.71716	0.39321	0.47109	0.02891	-0.79721
aPFE1530c_0	PFE1530c	0.71716	0.39321	0.47109	0.02891	-0.79721
intPFE1535w_2	PFE1535w	0.9443	1.10599	0.55877	-0.33077	-1.09799
intPFE1530c_2	PFE1535w	0.71716	0.39321	0.47109	0.02891	-0.79721
aPFE1535w_1	PFE1535w	0.9443	1.10599	0.55877	-0.33077	-1.09799
aPFE1535w_0	PFE1535w	0.9443	1.10599	0.55877	-0.33077	-1.09799
intPFE1540w_2	PFE1540w	-1.31821	-0.98693	0.22636	0.97964	0.83593
aPFE1540w_0	PFE1540w	-1.31821	-0.98693	0.22636	0.97964	0.83593
aPFE1545c_3	PFE1545c	-0.29259	-0.11963	-1.56321	-0.95816	1.446433
aPFE1545c_2	PFE1545c	-0.29259	-0.11963	-1.56321	-0.95816	1.446433
aPFE1545c_1	PFE1545c	-0.29259	-0.11963	-1.56321	-0.95816	1.446433

aPFE1545c_0	PFE1545c	-0.29259	-0.11963	-1.56321	-0.95816	1.446433
intPFE1550w_2	PFE1550w	0.64016	0.79371	-0.20116	-0.68384	-0.34121
intPFE1545c_2	PFE1550w	-0.29259	-0.11963	-1.56321	-0.95816	1.446433
aPFE1550w_1	PFE1550w	0.64016	0.79371	-0.20116	-0.68384	-0.34121
aPFE1550w_0	PFE1550w	0.64016	0.79371	-0.20116	-0.68384	-0.34121
intPFE1555c_2	PFE1555c	-0.55023	-0.47711	-1.77879	-0.80679	1.916272
aPFE1555c_3	PFE1555c	-0.55023	-0.47711	-1.77879	-0.80679	1.916272
aPFE1555c_2	PFE1555c	-0.55023	-0.47711	-1.77879	-0.80679	1.916272
aPFE1555c_1	PFE1555c	-0.55023	-0.47711	-1.77879	-0.80679	1.916272
aPFE1555c_0	PFE1555c	-0.55023	-0.47711	-1.77879	-0.80679	1.916272
intPFE1565w_2	PFE1560c	1.268773	-1.25585	-0.6113	0.227963	0.202513
aPFE1560c_0	PFE1560c	-1.35729	-0.45857	0.706643	0.932023	0.249233
intPFE1560c_2	PFE1565w	-1.35729	-0.45857	0.706643	0.932023	0.249233
aPFE1565w_0	PFE1565w	1.268773	-1.25585	-0.6113	0.227963	0.202513
intPFE1570c_2	PFE1570c	0.939967	0.331667	0.184917	-0.19308	-0.72083
aPFE1570c_1	PFE1570c	0.939967	0.331667	0.184917	-0.19308	-0.72083
aPFE1570c_0	PFE1570c	0.939967	0.331667	0.184917	-0.19308	-0.72083
intPFE1590w_2	PFE1590w	-0.10298	0.355897	1.065197	0.499137	-0.93256
aPFE1590w_0	PFE1590w	-0.10298	0.355897	1.065197	0.499137	-0.93256
intPFE1595c_2	PFE1595c	0.94784	0.83981	-0.44963	-1.01137	-0.44881
aPFE1595c_0	PFE1595c	0.94784	0.83981	-0.44963	-1.01137	-0.44881
intPFE1600w_2	PFE1600w	-0.27656	1.907293	2.133403	0.215263	-2.22463
aPFE1600w_1	PFE1600w	-0.27656	1.907293	2.133403	0.215263	-2.22463
aPFE1600w_0	PFE1600w	-0.27656	1.907293	2.133403	0.215263	-2.22463
intPFE1605w_2	PFE1605w	-1.57086	0.706773	1.706453	0.985713	-0.65511
aPFE1605w_1	PFE1605w	-1.57086	0.706773	1.706453	0.985713	-0.65511
aPFE1605w_0	PFE1605w	-1.57086	0.706773	1.706453	0.985713	-0.65511
intPFE1610w_2	PFE1610w	-1.25463	-0.48189	0.972273	1.171393	0.071553
aPFE1610w_0	PFE1610w	-1.25463	-0.48189	0.972273	1.171393	0.071553
intPFE1615c_2	PFE1615c	-1.15311	0.297553	2.126893	1.243773	-1.45089
aPFE1615c_0	PFE1615c	-1.15311	0.297553	2.126893	1.243773	-1.45089
intPFF0050c_2	PFF0050c	1.534917	1.599437	0.688127	-0.92279	-2.0911
intPFF0055w_3	PFF0055w	1.25593	1.06435	0.2435	-0.7431	-1.27015
intPFF0060w_2	PFF0060w					
aPFF0060w_0	PFF0060w					
intPFF0065c_2	PFF0065c	0.47882	1.0679	0.20276	-0.43544	-0.47254
intPFF0075c_2	PFF0075c	-0.2554	0.328997	0.175017	-0.10218	-0.16966
intPFF0085w_2	PFF0085w	-0.04493	0.234647	0.237217	-0.00788	-0.27531
intPFF0080c_2	PFF0085w	-0.0196	-0.273	-1.06199	-0.60301	0.867
intPFF0090w_3	PFF0090w	0.275627	0.760867	1.212267	0.203067	-1.50253
aPFF0090w_0	PFF0090w	0.275627	0.760867	1.212267	0.203067	-1.50253
intPFF0095c_2	PFF0095c	-0.63623	-0.62521	-0.11042	0.468195	0.699985
aPFF0095c_2	PFF0095c	-0.63623	-0.62521	-0.11042	0.468195	0.699985
aPFF0095c_1	PFF0095c	-0.63623	-0.62521	-0.11042	0.468195	0.699985
aPFF0095c_0	PFF0095c	-0.63623	-0.62521	-0.11042	0.468195	0.699985
intPFF0100w_3	PFF0100w	1.019577	0.610637	0.583737	-0.1629	-1.2473
aPFF0100w_1	PFF0100w	1.019577	0.610637	0.583737	-0.1629	-1.2473

aPFF0100w_0	PFF0100w	1.019577	0.610637	0.583737	-0.1629	-1.2473
intPFF0105w_2	PFF0105w	-0.20917	0.211813	1.199873	0.753793	-0.85815
aPFF0105w_0	PFF0105w	-0.20917	0.211813	1.199873	0.753793	-0.85815
intPFF0110w_2	PFF0110w	-0.54406	-0.4077	0.184782	0.635242	0.523652
aPFF0110w_0	PFF0110w	-0.54406	-0.4077	0.184782	0.635242	0.523652
intPFF0115c_2	PFF0115c	-0.84419	-0.32505	0.5456	0.7634	0.20955
aPFF0115c_1	PFF0115c	-0.84419	-0.32505	0.5456	0.7634	0.20955
aPFF0115c_0	PFF0115c	-0.84419	-0.32505	0.5456	0.7634	0.20955
intPFF0120w_2	PFF0120w	0.274267	0.632157	0.951177	0.348657	-0.89082
aPFF0120w_1	PFF0120w	0.274267	0.632157	0.951177	0.348657	-0.89082
aPFF0120w_0	PFF0120w	0.274267	0.632157	0.951177	0.348657	-0.89082
intPFF0125c_2	PFF0125c	0.52416	0.54072	0.30532	-0.19882	-0.67222
aPFF0125c_1	PFF0125c	0.52416	0.54072	0.30532	-0.19882	-0.67222
aPFF0125c_0	PFF0125c	0.52416	0.54072	0.30532	-0.19882	-0.67222
intPFF0135w_2	PFF0130c	2.031223	1.356873	0.170763	-1.1331	-1.78521
aPFF0130c_1	PFF0130c	2.229627	0.971357	-0.37496	-1.0812	-1.03002
aPFF0130c_0	PFF0130c	2.229627	0.971357	-0.37496	-1.0812	-1.03002
intPFF0130c_2	PFF0135w	2.229627	0.971357	-0.37496	-1.0812	-1.03002
aPFF0135w_1	PFF0135w	2.031223	1.356873	0.170763	-1.1331	-1.78521
aPFF0135w_0	PFF0135w	2.031223	1.356873	0.170763	-1.1331	-1.78521
intPFF0140c_2	PFF0140c	0.696573	0.399143	-0.86929	-0.97805	0.160523
aPFF0140c_1	PFF0140c	0.696573	0.399143	-0.86929	-0.97805	0.160523
aPFF0140c_0	PFF0140c	0.696573	0.399143	-0.86929	-0.97805	0.160523
intPFF0145w_2	PFF0145w	0.564247	-0.02024	-0.32877	-0.31539	-0.07192
aPFF0145w_1	PFF0145w	0.564247	-0.02024	-0.32877	-0.31539	-0.07192
aPFF0145w_0	PFF0145w	0.564247	-0.02024	-0.32877	-0.31539	-0.07192
intPFF0150c_2	PFF0150c	-0.0398	-0.25203	-0.17721	0.158373	0.407693
aPFF0150c_1	PFF0150c	-0.0398	-0.25203	-0.17721	0.158373	0.407693
aPFF0150c_0	PFF0150c	-0.0398	-0.25203	-0.17721	0.158373	0.407693
intPFF0155w_2	PFF0155w	-1.28391	-1.01496	0.79991	1.37809	0.35196
aPFF0155w_1	PFF0155w	-1.28391	-1.01496	0.79991	1.37809	0.35196
aPFF0155w_0	PFF0155w	-1.28391	-1.01496	0.79991	1.37809	0.35196
intPFF0160c_2	PFF0160c	0.232817	0.556417	1.102177	0.495157	-0.98458
aPFF0160c_1	PFF0160c	0.232817	0.556417	1.102177	0.495157	-0.98458
aPFF0160c_0	PFF0160c	0.232817	0.556417	1.102177	0.495157	-0.98458
intPFF0165c_2	PFF0165c	-0.06128	0.115417	0.380167	0.268667	-0.19658
aPFF0165c_1	PFF0165c	-0.06128	0.115417	0.380167	0.268667	-0.19658
aPFF0165c_0	PFF0165c	-0.06128	0.115417	0.380167	0.268667	-0.19658
intPFF0170w_2	PFF0170w	-0.60011	-0.48496	-0.29632	0.35232	0.94846
aPFF0170w_1	PFF0170w	-0.60011	-0.48496	-0.29632	0.35232	0.94846
aPFF0170w_0	PFF0170w	-0.60011	-0.48496	-0.29632	0.35232	0.94846
intPFF0175c_2	PFF0175c	0.211372	-0.08351	-0.05415	0.143592	0.188532
aPFF0175c_3	PFF0175c	0.211372	-0.08351	-0.05415	0.143592	0.188532
aPFF0175c_2	PFF0175c	0.211372	-0.08351	-0.05415	0.143592	0.188532
aPFF0175c_1	PFF0175c	0.211372	-0.08351	-0.05415	0.143592	0.188532
aPFF0175c_0	PFF0175c	0.211372	-0.08351	-0.05415	0.143592	0.188532
intPFF0180w_2	PFF0180w	-1.0334	-0.99601	0.368263	1.047903	0.577173

aPFF0180w_1	PFF0180w	-1.0334	-0.99601	0.368263	1.047903	0.577173
aPFF0180w_0	PFF0180w	-1.0334	-0.99601	0.368263	1.047903	0.577173
aPFF0185c_2	PFF0185c	0.107733	-0.26632	-1.21654	-0.61147	1.099653
aPFF0185c_1	PFF0185c	0.107733	-0.26632	-1.21654	-0.61147	1.099653
aPFF0185c_0	PFF0185c	0.107733	-0.26632	-1.21654	-0.61147	1.099653
intPFF0190c_2	PFF0190c	-0.1971	-0.54152	-0.42595	0.14595	0.70452
intPFF0185c_2	PFF0190c	0.107733	-0.26632	-1.21654	-0.61147	1.099653
aPFF0190c_1	PFF0190c	-0.1971	-0.54152	-0.42595	0.14595	0.70452
aPFF0190c_0	PFF0190c	-0.1971	-0.54152	-0.42595	0.14595	0.70452
intPFF0195c_2	PFF0195c	-1.09097	-1.19333	0.750483	1.343853	0.207993
aPFF0195c_2	PFF0195c	-1.09097	-1.19333	0.750483	1.343853	0.207993
aPFF0195c_1	PFF0195c	-1.09097	-1.19333	0.750483	1.343853	0.207993
aPFF0195c_0	PFF0195c	-1.09097	-1.19333	0.750483	1.343853	0.207993
intPFF0200c_2	PFF0200c	-1.185	-1.14349	-2.21393	-0.63029	2.746108
aPFF0200c_2	PFF0200c	-1.185	-1.14349	-2.21393	-0.63029	2.746108
aPFF0200c_1	PFF0200c	-1.185	-1.14349	-2.21393	-0.63029	2.746108
aPFF0200c_0	PFF0200c	-1.185	-1.14349	-2.21393	-0.63029	2.746108
intPFF0205w_2	PFF0205w	-1.27964	-0.65479	0.61657	0.96543	0.30779
aPFF0205w_0	PFF0205w	-1.27964	-0.65479	0.61657	0.96543	0.30779
intPFF0215w_2	PFF0210w					
intPFF0210w_2	PFF0210w	0.166433	-0.15118	-0.14516	-0.01268	0.090843
aPFF0210w_1	PFF0210w	0.166433	-0.15118	-0.14516	-0.01268	0.090843
aPFF0210w_0	PFF0210w	0.166433	-0.15118	-0.14516	-0.01268	0.090843
aPFF0215w_0	PFF0215w					
intPFF0225w_2	PFF0220w	0.29298	0.13809	0.29424	0.14104	-0.33348
aPFF0220w_4	PFF0220w	0.4984	0.133	-0.23369	-0.36291	-0.1934
aPFF0220w_3	PFF0220w	0.4984	0.133	-0.23369	-0.36291	-0.1934
aPFF0220w_2	PFF0220w	0.4984	0.133	-0.23369	-0.36291	-0.1934
aPFF0220w_1	PFF0220w	0.4984	0.133	-0.23369	-0.36291	-0.1934
aPFF0220w_0	PFF0220w	0.4984	0.133	-0.23369	-0.36291	-0.1934
aPFF0225w_2	PFF0225w	0.29298	0.13809	0.29424	0.14104	-0.33348
aPFF0225w_1	PFF0225w	0.29298	0.13809	0.29424	0.14104	-0.33348
aPFF0225w_0	PFF0225w	0.29298	0.13809	0.29424	0.14104	-0.33348
aPFF0230c_0	PFF0230c	-0.00541	-0.25191	-0.07916	0.255497	0.362247
intPFF0235c_2	PFF0235c	-1.28926	-0.01988	1.307835	1.168445	-0.22852
intPFF0230c_2	PFF0235c	-0.00541	-0.25191	-0.07916	0.255497	0.362247
aPFF0235c_2	PFF0235c	-1.28926	-0.01988	1.307835	1.168445	-0.22852
aPFF0235c_1	PFF0235c	-1.28926	-0.01988	1.307835	1.168445	-0.22852
aPFF0235c_0	PFF0235c	-1.28926	-0.01988	1.307835	1.168445	-0.22852
intPFF0245w_2	PFF0240c	0.164247	0.799787	0.823917	0.222417	-0.69845
aPFF0240c_0	PFF0240c	-0.38795	-0.62421	0.527437	0.894897	0.088547
intPFF0240c_2	PFF0245w	-0.38795	-0.62421	0.527437	0.894897	0.088547
aPFF0245w_0	PFF0245w	0.164247	0.799787	0.823917	0.222417	-0.69845
intPFF0250w_2	PFF0250w	0.23509	-0.37647	-0.02207	0.07657	-0.17403
aPFF0250w_1	PFF0250w	0.23509	-0.37647	-0.02207	0.07657	-0.17403
aPFF0250w_0	PFF0250w	0.23509	-0.37647	-0.02207	0.07657	-0.17403
intPFF0260w_2	PFF0255c	-0.74597	-0.8839	-0.18687	0.688093	1.034123

aPFF0255c_0	PFF0255c	0.024943	-0.84871	-0.2001	0.251763	0.170373
intPFF0255c_2	PFF0260w	0.024943	-0.84871	-0.2001	0.251763	0.170373
aPFF0260w_0	PFF0260w	-0.74597	-0.8839	-0.18687	0.688093	1.034123
aPFF0265c_0	PFF0265c	-0.72361	-0.54592	-0.60165	0.145987	1.199257
intPFF0265c_2	PFF0270c	-0.72361	-0.54592	-0.60165	0.145987	1.199257
aPFF0270c_1	PFF0270c	-0.64776	-0.75518	-0.65439	0.227227	1.253017
aPFF0270c_0	PFF0270c	-0.64776	-0.75518	-0.65439	0.227227	1.253017
intPFF0270c_2	PFF0275c	-0.64776	-0.75518	-0.65439	0.227227	1.253017
aPFF0275c_2	PFF0275c	0.548527	0.120857	-0.43372	-0.48294	-0.03152
aPFF0275c_1	PFF0275c	0.548527	0.120857	-0.43372	-0.48294	-0.03152
aPFF0275c_0	PFF0275c	0.548527	0.120857	-0.43372	-0.48294	-0.03152
intPFF0280w_2	PFF0280w	0.420357	0.235217	-0.11443	-0.43423	-0.42488
intPFF0275c_2	PFF0280w	0.548527	0.120857	-0.43372	-0.48294	-0.03152
aPFF0280w_0	PFF0280w	0.420357	0.235217	-0.11443	-0.43423	-0.42488
intPFF0285c_2	PFF0285c	-0.10619	-0.49451	-0.35377	0.171543	0.611623
aPFF0285c_3	PFF0285c	-0.10619	-0.49451	-0.35377	0.171543	0.611623
aPFF0285c_2	PFF0285c	-0.10619	-0.49451	-0.35377	0.171543	0.611623
aPFF0285c_1	PFF0285c	-0.10619	-0.49451	-0.35377	0.171543	0.611623
aPFF0285c_0	PFF0285c	-0.10619	-0.49451	-0.35377	0.171543	0.611623
intPFF0290w_2	PFF0290w	1.65009	0.06254	-1.04709	-1.52391	-0.87454
aPFF0290w_0	PFF0290w	1.65009	0.06254	-1.04709	-1.52391	-0.87454
intPFF0295c_3	PFF0295c	1.310863	1.149163	-0.13158	-1.38026	-1.5
aPFF0295c_1	PFF0295c	1.310863	1.149163	-0.13158	-1.38026	-1.5
aPFF0295c_0	PFF0295c	1.310863	1.149163	-0.13158	-1.38026	-1.5
intPFF0300w_2	PFF0300w	0.671157	0.410207	0.310837	-0.2975	-0.96637
aPFF0300w_1	PFF0300w	0.671157	0.410207	0.310837	-0.2975	-0.96637
aPFF0300w_0	PFF0300w	0.671157	0.410207	0.310837	-0.2975	-0.96637
intPFF0310w_2	PFF0305c	0.507543	-0.64974	-1.07603	-0.60731	0.459403
aPFF0305c_0	PFF0305c	-0.16465	-0.22977	-0.31547	-0.24953	0.10577
intPFF0305c_2	PFF0310w	-0.16465	-0.22977	-0.31547	-0.24953	0.10577
aPFF0310w_0	PFF0310w	0.507543	-0.64974	-1.07603	-0.60731	0.459403
intPFF0315c_2	PFF0315c	0.204025	0.036135	-0.05007	-0.13613	-0.15633
aPFF0315c_0	PFF0315c	0.204025	0.036135	-0.05007	-0.13613	-0.15633
intPFF0320c_2	PFF0320c	0.44957	0.64666	0.21342	-0.29042	-0.54566
aPFF0320c_1	PFF0320c	0.44957	0.64666	0.21342	-0.29042	-0.54566
aPFF0320c_0	PFF0320c	0.44957	0.64666	0.21342	-0.29042	-0.54566
intPFF0325c_3	PFF0325c	0.873085	0.528325	0.167475	-0.51248	-1.01458
aPFF0325c_3	PFF0325c	0.873085	0.528325	0.167475	-0.51248	-1.01458
aPFF0325c_2	PFF0325c	0.873085	0.528325	0.167475	-0.51248	-1.01458
aPFF0325c_1	PFF0325c	0.873085	0.528325	0.167475	-0.51248	-1.01458
aPFF0325c_0	PFF0325c	0.873085	0.528325	0.167475	-0.51248	-1.01458
intPFF0330w_2	PFF0330w	-0.11445	0.08358	-0.31616	-0.1165	0.49875
aPFF0330w_2	PFF0330w	-0.11445	0.08358	-0.31616	-0.1165	0.49875
aPFF0330w_1	PFF0330w	-0.11445	0.08358	-0.31616	-0.1165	0.49875
aPFF0330w_0	PFF0330w	-0.11445	0.08358	-0.31616	-0.1165	0.49875
intPFF0335c_2	PFF0335c	-1.02895	-1.40228	-1.22545	0.426113	2.293943
aPFF0335c_0	PFF0335c	-1.02895	-1.40228	-1.22545	0.426113	2.293943

intPFF0340c_3	PFF0340c	-0.00792	0.0956	0.73379	0.48021	-0.5586
aPFF0340c_0	PFF0340c	-0.00792	0.0956	0.73379	0.48021	-0.5586
intPFF0345w_2	PFF0345w	0.202787	0.208567	0.268107	0.113227	-0.25023
aPFF0345w_1	PFF0345w	0.202787	0.208567	0.268107	0.113227	-0.25023
aPFF0345w_0	PFF0345w	0.202787	0.208567	0.268107	0.113227	-0.25023
intPFF0350w_2	PFF0350w	0.126497	0.131027	-0.14531	-0.11085	0.145307
aPFF0350w_1	PFF0350w	0.126497	0.131027	-0.14531	-0.11085	0.145307
aPFF0350w_0	PFF0350w	0.126497	0.131027	-0.14531	-0.11085	0.145307
intPFF0360w_2	PFF0355c	0.432897	-0.11448	0.055967	0.148367	-0.10068
intPFF0355c_2	PFF0355c	-0.51635	-0.56924	-0.11526	0.434097	0.655077
aPFF0355c_1	PFF0355c	-0.51635	-0.56924	-0.11526	0.434097	0.655077
aPFF0355c_0	PFF0355c	-0.51635	-0.56924	-0.11526	0.434097	0.655077
aPFF0360w_1	PFF0360w	0.432897	-0.11448	0.055967	0.148367	-0.10068
aPFF0360w_0	PFF0360w	0.432897	-0.11448	0.055967	0.148367	-0.10068
intPFF0365c_3	PFF0365c	-0.38045	-0.30557	-0.0415	0.395738	0.592808
aPFF0365c_1	PFF0365c	-0.38045	-0.30557	-0.0415	0.395738	0.592808
aPFF0365c_0	PFF0365c	-0.38045	-0.30557	-0.0415	0.395738	0.592808
intPFF0370w_2	PFF0370w	0.163753	0.128713	-0.00517	0.011833	0.058453
aPFF0370w_1	PFF0370w	0.163753	0.128713	-0.00517	0.011833	0.058453
aPFF0370w_0	PFF0370w	0.163753	0.128713	-0.00517	0.011833	0.058453
intPFF0375c_2	PFF0375c	-0.9743	-1.60303	-1.33046	0.334623	2.197193
aPFF0375c_1	PFF0375c	-0.9743	-1.60303	-1.33046	0.334623	2.197193
aPFF0375c_0	PFF0375c	-0.9743	-1.60303	-1.33046	0.334623	2.197193
intPFF0380w_2	PFF0380w	-0.55829	-0.59859	-1.75573	-0.65641	2.021253
aPFF0380w_4	PFF0380w	-0.55829	-0.59859	-1.75573	-0.65641	2.021253
aPFF0380w_3	PFF0380w	-0.55829	-0.59859	-1.75573	-0.65641	2.021253
aPFF0380w_2	PFF0380w	-0.55829	-0.59859	-1.75573	-0.65641	2.021253
aPFF0380w_1	PFF0380w	-0.55829	-0.59859	-1.75573	-0.65641	2.021253
aPFF0380w_0	PFF0380w	-0.55829	-0.59859	-1.75573	-0.65641	2.021253
intPFF0390w_2	PFF0385c	-0.00133	-0.51885	0.39696	0.64104	-0.11165
aPFF0385c_1	PFF0385c	-0.47211	-0.80595	0.564157	0.833677	-0.16371
aPFF0385c_0	PFF0385c	-0.47211	-0.80595	0.564157	0.833677	-0.16371
intPFF0385c_2	PFF0390w	-0.47211	-0.80595	0.564157	0.833677	-0.16371
aPFF0390w_1	PFF0390w	-0.00133	-0.51885	0.39696	0.64104	-0.11165
aPFF0390w_0	PFF0390w	-0.00133	-0.51885	0.39696	0.64104	-0.11165
intPFF0400w_2	PFF0395c	0.030357	0.330707	-0.04516	-0.08	0.173127
aPFF0395c_0	PFF0395c	0.437043	0.661783	0.483933	0.079733	-0.47212
intPFF0395c_2	PFF0400w	0.437043	0.661783	0.483933	0.079733	-0.47212
aPFF0400w_1	PFF0400w	0.030357	0.330707	-0.04516	-0.08	0.173127
aPFF0400w_0	PFF0400w	0.030357	0.330707	-0.04516	-0.08	0.173127
intPFF0410w_2	PFF0405c	-0.2122	0.50199	0.71768	0.31543	-0.43721
aPFF0405c_0	PFF0405c					
intPFF0405c_2	PFF0410w					
aPFF0410w_2	PFF0410w	-0.2122	0.50199	0.71768	0.31543	-0.43721
aPFF0410w_1	PFF0410w	-0.2122	0.50199	0.71768	0.31543	-0.43721
aPFF0410w_0	PFF0410w	-0.2122	0.50199	0.71768	0.31543	-0.43721
intPFF0415c_2	PFF0415c	2.364467	-0.07733	-1.17483	-0.73061	0.218227

aPFF0415c_0	PFF0415c	2.364467	-0.07733	-1.17483	-0.73061	0.218227
intPFF0420c_2	PFF0420c	-0.83117	-0.47419	0.310373	0.835293	0.644853
aPFF0420c_0	PFF0420c	-0.83117	-0.47419	0.310373	0.835293	0.644853
intPFF0425w_2	PFF0425w	-0.1371	-0.3895	0.005007	0.371327	0.333837
aPFF0425w_0	PFF0425w	-0.1371	-0.3895	0.005007	0.371327	0.333837
intPFF0430w_2	PFF0430w	-0.13162	0.14858	1.15185	0.77715	-0.80308
aPFF0430w_1	PFF0430w	-0.13162	0.14858	1.15185	0.77715	-0.80308
aPFF0430w_0	PFF0430w	-0.13162	0.14858	1.15185	0.77715	-0.80308
intPFF0435w_2	PFF0435w	-2.72794	-1.31179	1.39058	2.22392	0.82129
aPFF0435w_1	PFF0435w	-2.72794	-1.31179	1.39058	2.22392	0.82129
aPFF0435w_0	PFF0435w	-2.72794	-1.31179	1.39058	2.22392	0.82129
intPFF0440w_2	PFF0440w	-0.71448	-0.96111	-0.48737	0.375655	1.054395
aPFF0440w_1	PFF0440w	-0.71448	-0.96111	-0.48737	0.375655	1.054395
aPFF0440w_0	PFF0440w	-0.71448	-0.96111	-0.48737	0.375655	1.054395
intPFF0445w_2	PFF0445w	-0.15599	0.028617	0.081947	0.161577	0.163207
aPFF0445w_9	PFF0445w	-0.15599	0.028617	0.081947	0.161577	0.163207
aPFF0445w_8	PFF0445w	-0.15599	0.028617	0.081947	0.161577	0.163207
aPFF0445w_7	PFF0445w	-0.15599	0.028617	0.081947	0.161577	0.163207
aPFF0445w_6	PFF0445w	-0.15599	0.028617	0.081947	0.161577	0.163207
aPFF0445w_5	PFF0445w	-0.15599	0.028617	0.081947	0.161577	0.163207
aPFF0445w_4	PFF0445w	-0.15599	0.028617	0.081947	0.161577	0.163207
aPFF0445w_3	PFF0445w	-0.15599	0.028617	0.081947	0.161577	0.163207
aPFF0445w_2	PFF0445w	-0.15599	0.028617	0.081947	0.161577	0.163207
aPFF0445w_1	PFF0445w	-0.15599	0.028617	0.081947	0.161577	0.163207
aPFF0445w_0	PFF0445w	-0.15599	0.028617	0.081947	0.161577	0.163207
intPFF0450c_2	PFF0450c	0.336483	0.622603	0.172303	-0.41264	-0.63194
aPFF0450c_0	PFF0450c	0.336483	0.622603	0.172303	-0.41264	-0.63194
intPFF0455w_3	PFF0455w	0.712847	0.570257	0.299247	-0.43991	-1.06592
aPFF0455w_1	PFF0455w	0.712847	0.570257	0.299247	-0.43991	-1.06592
aPFF0455w_0	PFF0455w	0.712847	0.570257	0.299247	-0.43991	-1.06592
intPFF0460w_2	PFF0460w	-0.88068	-0.8021	0.152967	0.830367	0.723937
aPFF0460w_1	PFF0460w	-0.88068	-0.8021	0.152967	0.830367	0.723937
aPFF0460w_0	PFF0460w	-0.88068	-0.8021	0.152967	0.830367	0.723937
intPFF0470w_2	PFF0465c	-0.09026	0.63431	0.16688	-0.15088	-0.07931
aPFF0465c_1	PFF0465c	-0.05942	0.81676	0.47365	-0.05477	-0.3862
aPFF0465c_0	PFF0465c	-0.05942	0.81676	0.47365	-0.05477	-0.3862
intPFF0475w_2	PFF0470w	-0.34317	-0.13912	-0.53499	-0.18967	0.717957
intPFF0465c_2	PFF0470w	-0.05942	0.81676	0.47365	-0.05477	-0.3862
aPFF0470w_1	PFF0470w	-0.09026	0.63431	0.16688	-0.15088	-0.07931
aPFF0470w_0	PFF0470w	-0.09026	0.63431	0.16688	-0.15088	-0.07931
aPFF0475w_1	PFF0475w	-0.34317	-0.13912	-0.53499	-0.18967	0.717957
aPFF0475w_0	PFF0475w	-0.34317	-0.13912	-0.53499	-0.18967	0.717957
intPFF0480w_2	PFF0480w	1.350992	1.122692	-0.50022	-1.68289	-1.30914
aPFF0480w_2	PFF0480w	1.350992	1.122692	-0.50022	-1.68289	-1.30914
aPFF0480w_1	PFF0480w	1.350992	1.122692	-0.50022	-1.68289	-1.30914
intPFF0485c_2	PFF0485c	-0.46855	-0.97025	-1.59452	-0.46982	1.727913
aPFF0485c_0	PFF0485c	-0.46855	-0.97025	-1.59452	-0.46982	1.727913

intPFF0490w_2	PFF0490w	1.144532	0.682512	-0.53911	-1.06133	-0.53013
aPFF0490w_2	PFF0490w	1.144532	0.682512	-0.53911	-1.06133	-0.53013
aPFF0490w_1	PFF0490w	1.144532	0.682512	-0.53911	-1.06133	-0.53013
aPFF0490w_0	PFF0490w	1.144532	0.682512	-0.53911	-1.06133	-0.53013
intPFF0495w_2	PFF0495w	-1.14302	-1.12888	0.248767	1.060567	0.763217
aPFF0495w_0	PFF0495w	-1.14302	-1.12888	0.248767	1.060567	0.763217
aPFF0500c_1	PFF0500c	0.455613	0.090443	0.352863	0.124803	-0.55128
aPFF0500c_0	PFF0500c	0.455613	0.090443	0.352863	0.124803	-0.55128
intPFF0505c_2	PFF0505c	0.524067	0.471177	0.282907	-0.14407	-0.59534
intPFF0500c_1	PFF0505c	0.455613	0.090443	0.352863	0.124803	-0.55128
aPFF0505c_1	PFF0505c	0.524067	0.471177	0.282907	-0.14407	-0.59534
aPFF0505c_0	PFF0505c	0.524067	0.471177	0.282907	-0.14407	-0.59534
intPFF0510w_2	PFF0510w	-0.97051	-2.78642	-1.66766	0.960997	2.964757
aPFF0510w_0	PFF0510w	-0.97051	-2.78642	-1.66766	0.960997	2.964757
intPFF0515c_2	PFF0515c	0.59298	0.62732	0.56097	0.01155	-0.79058
aPFF0515c_0	PFF0515c	0.59298	0.62732	0.56097	0.01155	-0.79058
intPFF0520w_3	PFF0520w	-1.00787	-0.54712	0.450247	0.718247	0.223127
aPFF0520w_1	PFF0520w	-1.00787	-0.54712	0.450247	0.718247	0.223127
aPFF0520w_0	PFF0520w	-1.00787	-0.54712	0.450247	0.718247	0.223127
intPFF0530w_2	PFF0525w	0.390843	0.362783	0.744433	0.062733	-1.15912
intPFF0525w_2	PFF0525w	0.453417	0.254937	0.691627	0.083707	-1.1236
aPFF0525w_0	PFF0525w	0.453417	0.254937	0.691627	0.083707	-1.1236
aPFF0530w_1	PFF0530w	0.390843	0.362783	0.744433	0.062733	-1.15912
aPFF0530w_0	PFF0530w	0.390843	0.362783	0.744433	0.062733	-1.15912
intPFF0535c_2	PFF0535c	0.434677	0.404607	0.267787	-0.19845	-0.66377
aPFF0535c_1	PFF0535c	0.434677	0.404607	0.267787	-0.19845	-0.66377
aPFF0535c_0	PFF0535c	0.434677	0.404607	0.267787	-0.19845	-0.66377
intPFF0540c_2	PFF0540c	-0.84543	-1.12188	-0.63425	0.515913	1.470543
aPFF0540c_0	PFF0540c	-0.84543	-1.12188	-0.63425	0.515913	1.470543
intPFF0550w_2	PFF0545c	-1.27165	-1.06874	0.525997	1.207337	0.558077
aPFF0545c_0	PFF0545c	-0.21026	-0.88969	0.431377	0.893957	0.027027
intPFF0545c_2	PFF0550w	-0.21026	-0.88969	0.431377	0.893957	0.027027
aPFF0550w_0	PFF0550w	-1.27165	-1.06874	0.525997	1.207337	0.558077
intPFF0555w_2	PFF0555w	-0.74067	-0.71367	0.368333	0.956333	0.530333
aPFF0555w_0	PFF0555w	-0.74067	-0.71367	0.368333	0.956333	0.530333
intPFF0560c_2	PFF0560c	0.641147	0.339757	-0.04801	-0.19515	-0.19192
aPFF0560c_1	PFF0560c	0.641147	0.339757	-0.04801	-0.19515	-0.19192
aPFF0560c_0	PFF0560c	0.641147	0.339757	-0.04801	-0.19515	-0.19192
intPFF0565c_2	PFF0565c	-0.17605	-0.27272	-0.1988	-0.04986	0.166557
aPFF0565c_1	PFF0565c	-0.17605	-0.27272	-0.1988	-0.04986	0.166557
aPFF0565c_0	PFF0565c	-0.17605	-0.27272	-0.1988	-0.04986	0.166557
intPFF0570c_2	PFF0570c	-0.65572	-0.6844	-1.7402	-0.58696	2.072237
aPFF0570c_1	PFF0570c	-0.65572	-0.6844	-1.7402	-0.58696	2.072237
aPFF0570c_0	PFF0570c	-0.65572	-0.6844	-1.7402	-0.58696	2.072237
intPFF0575c_2	PFF0575c	-0.27686	-0.25159	0.104637	0.117597	-0.09087
aPFF0575c_4	PFF0575c	-0.27686	-0.25159	0.104637	0.117597	-0.09087
aPFF0575c_3	PFF0575c	-0.27686	-0.25159	0.104637	0.117597	-0.09087

aPFF0575c_2	PFF0575c	-0.27686	-0.25159	0.104637	0.117597	-0.09087
aPFF0575c_1	PFF0575c	-0.27686	-0.25159	0.104637	0.117597	-0.09087
aPFF0575c_0	PFF0575c	-0.27686	-0.25159	0.104637	0.117597	-0.09087
intPFF0580w_2	PFF0580w	-0.43807	-0.55585	0.29735	0.68975	0.26495
aPFF0580w_0	PFF0580w	-0.43807	-0.55585	0.29735	0.68975	0.26495
aPFF0585c_0	PFF0585c	-0.72023	-1.06589	-0.33873	0.583393	1.025553
intPFF0590c_1	PFF0590c	-0.04274	0.356717	0.663057	0.356777	-0.42838
intPFF0585c_2	PFF0590c	-0.72023	-1.06589	-0.33873	0.583393	1.025553
aPFF0590c_1	PFF0590c	-0.04274	0.356717	0.663057	0.356777	-0.42838
aPFF0590c_0	PFF0590c	-0.04274	0.356717	0.663057	0.356777	-0.42838
aPFF0595c_2	PFF0595c	-0.38634	-0.98595	-1.70342	-0.39313	2.004728
aPFF0595c_1	PFF0595c	-0.38634	-0.98595	-1.70342	-0.39313	2.004728
aPFF0595c_0	PFF0595c	-0.38634	-0.98595	-1.70342	-0.39313	2.004728
intPFF0600w_3	PFF0600w	-0.89419	-1.21303	-0.32794	0.62494	1.02803
intPFF0595c_2	PFF0600w	-0.38634	-0.98595	-1.70342	-0.39313	2.004728
aPFF0600w_0	PFF0602w	-0.89419	-1.21303	-0.32794	0.62494	1.02803
intPFF0605c_2	PFF0605c	0.176843	0.146773	-0.14055	-0.44979	-0.37811
aPFF0605c_0	PFF0605c	0.176843	0.146773	-0.14055	-0.44979	-0.37811
aPFF0610c_1	PFF0610c	-0.08431	0.043557	-0.66011	-0.68005	0.247277
aPFF0610c_0	PFF0610c	-0.08431	0.043557	-0.66011	-0.68005	0.247277
intPFF0615c_2	PFF0615c	-0.81669	-1.57853	-1.91644	-0.38256	2.15953
intPFF0610c_2	PFF0615c	-0.08431	0.043557	-0.66011	-0.68005	0.247277
aPFF0615c_0	PFF0615c	-0.81669	-1.57853	-1.91644	-0.38256	2.15953
intPFF0620c_2	PFF0620c	0.480153	0.369243	0.434003	-0.02334	-0.74458
aPFF0620c_0	PFF0620c	0.480153	0.369243	0.434003	-0.02334	-0.74458
intPFF0625w_2	PFF0625w	0.50801	0.76093	0.85339	-0.01789	-1.25343
aPFF0625w_1	PFF0625w	0.50801	0.76093	0.85339	-0.01789	-1.25343
aPFF0625w_0	PFF0625w	0.50801	0.76093	0.85339	-0.01789	-1.25343
aPFF0630c_0	PFF0630c-a	-1.41051	-1.60946	0.356423	1.614243	1.165123
intPFF0630c_2	PFF0630c-c	-1.41051	-1.60946	0.356423	1.614243	1.165123
intPFF0640w_2	PFF0635w	-0.33373	-1.15637	-0.18226	0.56326	0.51937
intPFF0635w_3	PFF0635w	0.569873	-0.30939	-0.05273	-0.01161	-0.31695
aPFF0635w_0	PFF0635w	0.569873	-0.30939	-0.05273	-0.01161	-0.31695
aPFF0640w_0	PFF0640w	-0.33373	-1.15637	-0.18226	0.56326	0.51937
intPFF0645c_2	PFF0645c	0.319013	-0.20505	-1.9359	-1.58094	0.934713
aPFF0645c_1	PFF0645c	0.319013	-0.20505	-1.9359	-1.58094	0.934713
aPFF0645c_0	PFF0645c	0.319013	-0.20505	-1.9359	-1.58094	0.934713
intPFF0650w_2	PFF0650w	-0.22496	-1.1212	-0.55961	0.394273	0.944863
aPFF0650w_0	PFF0650w	-0.22496	-1.1212	-0.55961	0.394273	0.944863
intPFF0655c_2	PFF0655c	-0.85	-0.39735	0.060193	0.350253	0.433453
aPFF0655c_2	PFF0655c	-0.85	-0.39735	0.060193	0.350253	0.433453
aPFF0655c_1	PFF0655c	-0.85	-0.39735	0.060193	0.350253	0.433453
aPFF0655c_0	PFF0655c	-0.85	-0.39735	0.060193	0.350253	0.433453
intPFF0660w_2	PFF0660w	0.25047	-0.41035	-0.96005	-0.74845	0.28035
aPFF0660w_1	PFF0660w	0.25047	-0.41035	-0.96005	-0.74845	0.28035
aPFF0660w_0	PFF0660w	0.25047	-0.41035	-0.96005	-0.74845	0.28035
intPFF0665c_2	PFF0665c	-0.91564	-0.77531	0.05686	0.60314	0.58881

aPFF0665c_1	PFF0665c	-0.91564	-0.77531	0.05686	0.60314	0.58881
aPFF0665c_0	PFF0665c	-0.91564	-0.77531	0.05686	0.60314	0.58881
intPFF0670w_2	PFF0670w	-1.29357	-1.53027	-1.8388	0.002657	2.786137
aPFF0670w_6	PFF0670w	-1.29357	-1.53027	-1.8388	0.002657	2.786137
aPFF0670w_5	PFF0670w	-1.29357	-1.53027	-1.8388	0.002657	2.786137
aPFF0670w_4	PFF0670w	-1.29357	-1.53027	-1.8388	0.002657	2.786137
aPFF0670w_3	PFF0670w	-1.29357	-1.53027	-1.8388	0.002657	2.786137
aPFF0670w_2	PFF0670w	-1.29357	-1.53027	-1.8388	0.002657	2.786137
aPFF0670w_1	PFF0670w	-1.29357	-1.53027	-1.8388	0.002657	2.786137
aPFF0670w_0	PFF0670w	-1.29357	-1.53027	-1.8388	0.002657	2.786137
intPFF0675c_2	PFF0675c	1.119942	0.357522	-2.16316	-2.52451	0.044062
aPFF0675c_3	PFF0675c	1.119942	0.357522	-2.16316	-2.52451	0.044062
aPFF0675c_2	PFF0675c	1.119942	0.357522	-2.16316	-2.52451	0.044062
aPFF0675c_1	PFF0675c	1.119942	0.357522	-2.16316	-2.52451	0.044062
aPFF0675c_0	PFF0675c	1.119942	0.357522	-2.16316	-2.52451	0.044062
aPFF0680c_1	PFF0680c	-0.30159	-0.83959	-0.57509	0.082753	0.719253
aPFF0680c_0	PFF0680c	-0.30159	-0.83959	-0.57509	0.082753	0.719253
intPFF0680c_2	PFF0683c	-0.30159	-0.83959	-0.57509	0.082753	0.719253
aPFF0685c_2	PFF0683c	0.502887	0.102237	-0.22797	-0.36069	-0.22257
intPFF0685c_2	PFF0685c	0.502887	0.102237	-0.22797	-0.36069	-0.22257
aPFF0685c_1	PFF0685c	0.502887	0.102237	-0.22797	-0.36069	-0.22257
aPFF0685c_0	PFF0685c	0.502887	0.102237	-0.22797	-0.36069	-0.22257
intPFF0690c_2	PFF0690c	-1.1754	0.102473	0.226553	0.416613	0.669693
aPFF0690c_1	PFF0690c	-1.1754	0.102473	0.226553	0.416613	0.669693
aPFF0690c_0	PFF0690c	-1.1754	0.102473	0.226553	0.416613	0.669693
intPFF0695w_2	PFF0695w	0.105267	0.728647	0.763197	0.030137	-0.90331
aPFF0695w_0	PFF0695w	0.105267	0.728647	0.763197	0.030137	-0.90331
intPFF0700c_2	PFF0700c	-0.18111	0.39156	0.74339	0.53461	-0.23956
aPFF0700c_0	PFF0700c	-0.18111	0.39156	0.74339	0.53461	-0.23956
intPFF0710w_2	PFF0705c	0.19892	-0.5841	-1.68279	-1.14321	0.9146
intPFF0705c_2	PFF0705c	-0.02153	-0.65886	-1.55478	-0.96356	0.973023
aPFF0705c_1	PFF0705c	-0.02153	-0.65886	-1.55478	-0.96356	0.973023
aPFF0705c_0	PFF0705c	-0.02153	-0.65886	-1.55478	-0.96356	0.973023
aPFF0710w_1	PFF0710w	0.19892	-0.5841	-1.68279	-1.14321	0.9146
aPFF0710w_0	PFF0710w	0.19892	-0.5841	-1.68279	-1.14321	0.9146
intPFF0720w_2	PFF0715c	-0.74089	-1.11158	-1.1296	0.078263	1.694243
aPFF0715c_1	PFF0715c	-0.99539	-0.15901	0.376787	0.478547	0.252847
aPFF0715c_0	PFF0715c	-0.99539	-0.15901	0.376787	0.478547	0.252847
intPFF0725w_2	PFF0720w	1.069353	0.709233	0.302533	-0.42487	-1.07557
intPFF0715c_2	PFF0720w	-0.99539	-0.15901	0.376787	0.478547	0.252847
aPFF0720w_1	PFF0720w	-0.74089	-1.11158	-1.1296	0.078263	1.694243
aPFF0720w_0	PFF0720w	-0.74089	-1.11158	-1.1296	0.078263	1.694243
aPFF0725w_0	PFF0725w	1.069353	0.709233	0.302533	-0.42487	-1.07557
intPFF0735w_2	PFF0730c	0.589653	-0.12826	-0.6675	-0.41184	0.323923
aPFF0730c_1	PFF0730c	-1.11303	0.147643	0.011973	0.102693	0.601523
aPFF0730c_0	PFF0730c	-1.11303	0.147643	0.011973	0.102693	0.601523
intPFF0730c_2	PFF0735w	-1.11303	0.147643	0.011973	0.102693	0.601523

aPFF0735w_0	PFF0735w	0.589653	-0.12826	-0.6675	-0.41184	0.323923
intPFF0740c_2	PFF0740c	-0.72032	-0.28791	-0.10145	0.283613	0.659573
aPFF0740c_1	PFF0740c	-0.72032	-0.28791	-0.10145	0.283613	0.659573
aPFF0740c_0	PFF0740c	-0.72032	-0.28791	-0.10145	0.283613	0.659573
intPFF0745c_2	PFF0745c	0.212882	0.024182	-0.44318	-0.20564	0.449202
aPFF0745c_4	PFF0745c	0.212882	0.024182	-0.44318	-0.20564	0.449202
aPFF0745c_3	PFF0745c	0.212882	0.024182	-0.44318	-0.20564	0.449202
aPFF0745c_2	PFF0745c	0.212882	0.024182	-0.44318	-0.20564	0.449202
aPFF0745c_1	PFF0745c	0.212882	0.024182	-0.44318	-0.20564	0.449202
aPFF0745c_0	PFF0745c	0.212882	0.024182	-0.44318	-0.20564	0.449202
intPFF0750w_2	PFF0750w	-0.87641	-0.56995	-0.55036	0.23586	1.27495
aPFF0750w_1	PFF0750w	-0.87641	-0.56995	-0.55036	0.23586	1.27495
aPFF0750w_0	PFF0750w	-0.87641	-0.56995	-0.55036	0.23586	1.27495
intPFF0760w_2	PFF0755c	-0.24683	-0.16783	0.35867	0.47933	0.03183
aPFF0755c_3	PFF0755c	0.50257	0.5838	0.36035	-0.12696	-0.62941
aPFF0755c_2	PFF0755c	0.50257	0.5838	0.36035	-0.12696	-0.62941
aPFF0755c_1	PFF0755c	0.50257	0.5838	0.36035	-0.12696	-0.62941
aPFF0755c_0	PFF0755c	0.50257	0.5838	0.36035	-0.12696	-0.62941
intPFF0755c_2	PFF0760w	0.50257	0.5838	0.36035	-0.12696	-0.62941
aPFF0760w_0	PFF0760w	-0.24683	-0.16783	0.35867	0.47933	0.03183
aPFF0765c_1	PFF0765c	-1.31947	-0.69464	-0.21522	0.56322	1.21614
aPFF0765c_0	PFF0765c	-1.31947	-0.69464	-0.21522	0.56322	1.21614
intPFF0770c_2	PFF0770c	-0.5718	-0.81303	0.209715	0.615285	0.187025
intPFF0765c_2	PFF0770c	-1.31947	-0.69464	-0.21522	0.56322	1.21614
aPFF0770c_2	PFF0770c	-0.5718	-0.81303	0.209715	0.615285	0.187025
aPFF0770c_1	PFF0770c	-0.5718	-0.81303	0.209715	0.615285	0.187025
aPFF0770c_0	PFF0770c	-0.5718	-0.81303	0.209715	0.615285	0.187025
intPFF0780w_2	PFF0775w	0.557913	0.890133	-0.00011	-0.37579	-0.15603
intPFF0775w_2	PFF0775w	-0.8286	0.473993	1.620753	0.783913	-1.20333
aPFF0775w_1	PFF0775w	-0.8286	0.473993	1.620753	0.783913	-1.20333
aPFF0775w_0	PFF0775w	-0.8286	0.473993	1.620753	0.783913	-1.20333
aPFF0780w_0	PFF0780w	0.557913	0.890133	-0.00011	-0.37579	-0.15603
intPFF0785w_2	PFF0785w	-0.88012	-1.33988	-0.50523	0.592567	1.215217
aPFF0785w_1	PFF0785w	-0.88012	-1.33988	-0.50523	0.592567	1.215217
aPFF0785w_0	PFF0785w	-0.88012	-1.33988	-0.50523	0.592567	1.215217
intPFF0795w_2	PFF0790c	0.574568	0.136668	-0.04342	-0.05424	-0.10217
aPFF0790c_0	PFF0790c	1.019842	-0.00423	0.174482	0.137402	-0.45654
intPFF0790c_2	PFF0795w	1.019842	-0.00423	0.174482	0.137402	-0.45654
aPFF0795w_2	PFF0795w	0.574568	0.136668	-0.04342	-0.05424	-0.10217
aPFF0795w_1	PFF0795w	0.574568	0.136668	-0.04342	-0.05424	-0.10217
aPFF0795w_0	PFF0795w	0.574568	0.136668	-0.04342	-0.05424	-0.10217
intPFF0800w_2	PFF0800w	0.043367	-0.11968	-0.11056	0.087617	0.239887
aPFF0800w_2	PFF0800w	0.043367	-0.11968	-0.11056	0.087617	0.239887
aPFF0800w_1	PFF0800w	0.043367	-0.11968	-0.11056	0.087617	0.239887
aPFF0800w_0	PFF0800w	0.043367	-0.11968	-0.11056	0.087617	0.239887
aPFF0805c_1	PFF0805c	-0.90413	-1.10886	-0.33454	0.615703	1.099023
aPFF0805c_0	PFF0805c	-0.90413	-1.10886	-0.33454	0.615703	1.099023

intPFF0810c_2	PFF0810c	-0.94224	-0.28926	0.408267	0.591567	0.260097
intPFF0805c_2	PFF0810c	-0.90413	-1.10886	-0.33454	0.615703	1.099023
aPFF0810c_1	PFF0810c	-0.94224	-0.28926	0.408267	0.591567	0.260097
aPFF0810c_0	PFF0810c	-0.94224	-0.28926	0.408267	0.591567	0.260097
intPFF0815w_2	PFF0815w	-1.18239	0.148943	0.613113	0.559553	0.208723
aPFF0815w_1	PFF0815w	-1.18239	0.148943	0.613113	0.559553	0.208723
aPFF0815w_0	PFF0815w	-1.18239	0.148943	0.613113	0.559553	0.208723
intPFF0820w_2	PFF0820w	0.60164	0.38328	0.1767	-0.1102	-0.43428
aPFF0820w_1	PFF0820w	0.60164	0.38328	0.1767	-0.1102	-0.43428
aPFF0820w_0	PFF0820w	0.60164	0.38328	0.1767	-0.1102	-0.43428
intPFF0825c_2	PFF0825c	-0.24733	0.37464	1.34223	0.70827	-1.06614
aPFF0825c_1	PFF0825c	-0.24733	0.37464	1.34223	0.70827	-1.06614
aPFF0825c_0	PFF0825c	-0.24733	0.37464	1.34223	0.70827	-1.06614
intPFF0830w_2	PFF0830w	0.68658	0.25261	0.17577	-0.35927	-0.92161
aPFF0830w_1	PFF0830w	0.68658	0.25261	0.17577	-0.35927	-0.92161
aPFF0830w_0	PFF0830w	0.68658	0.25261	0.17577	-0.35927	-0.92161
intPFF0835w_2	PFF0835w	-0.25125	0.327253	0.235503	0.155163	0.102913
aPFF0835w_1	PFF0835w	-0.25125	0.327253	0.235503	0.155163	0.102913
aPFF0835w_0	PFF0835w	-0.25125	0.327253	0.235503	0.155163	0.102913
intPFF0840w_2	PFF0840w	-0.08085	-0.95075	-0.93749	-0.14517	0.894087
aPFF0840w_0	PFF0840w	-0.08085	-0.95075	-0.93749	-0.14517	0.894087
intPFF0860c_2	PFF0860c	-1.5049	-2.75947	-1.41204	1.015377	2.773807
aPFF0860c_0	PFF0860c	-1.5049	-2.75947	-1.41204	1.015377	2.773807
intPFF0865w_2	PFF0865w	-0.66273	-1.42139	-1.01573	0.358393	1.673053
aPFF0865w_0	PFF0865w	-0.66273	-1.42139	-1.01573	0.358393	1.673053
intPFF0875w_2	PFF0870w	-0.32225	-0.77477	-0.28921	-0.04013	0.060933
intPFF0870w_2	PFF0870w	0.351423	-0.3506	-2.36532	-1.82552	1.247263
aPFF0870w_1	PFF0870w	0.351423	-0.3506	-2.36532	-1.82552	1.247263
aPFF0870w_0	PFF0870w	0.351423	-0.3506	-2.36532	-1.82552	1.247263
aPFF0875w_1	PFF0875w	-0.32225	-0.77477	-0.28921	-0.04013	0.060933
aPFF0875w_0	PFF0875w	-0.32225	-0.77477	-0.28921	-0.04013	0.060933
intPFF0880c_2	PFF0880c	-0.0121	0.2905	0.63201	0.43899	-0.3025
aPFF0880c_0	PFF0880c	-0.0121	0.2905	0.63201	0.43899	-0.3025
intPFF0885w_2	PFF0885w	-0.26556	0.44282	0.82637	0.52763	-0.33982
aPFF0885w_0	PFF0885w	-0.26556	0.44282	0.82637	0.52763	-0.33982
intPFF0890c_2	PFF0890c	-0.24826	-0.15471	-0.16758	0.01458	0.30871
aPFF0890c_1	PFF0890c	-0.24826	-0.15471	-0.16758	0.01458	0.30871
aPFF0890c_0	PFF0890c	-0.24826	-0.15471	-0.16758	0.01458	0.30871
intPFF0895w_2	PFF0895w					
aPFF0895w_0	PFF0895w					
intPFF0905w_2	PFF0900c	-0.61777	0.04885	0.2728	0.3652	0.28615
aPFF0900c_0	PFF0900c	-0.49864	-0.72281	0.39811	0.87689	0.26981
intPFF0900c_2	PFF0905w	-0.49864	-0.72281	0.39811	0.87689	0.26981
aPFF0905w_0	PFF0905w	-0.61777	0.04885	0.2728	0.3652	0.28615
aPFF0910c_1	PFF0910c	-0.52065	-0.84977	0.126783	0.645883	0.343433
aPFF0910c_0	PFF0910c	-0.52065	-0.84977	0.126783	0.645883	0.343433
intPFF0915w_2	PFF0915w	-0.6774	-1.098	-0.94073	0.223567	1.553837

intPFF0910c_2	PFF0915w	-0.52065	-0.84977	0.126783	0.645883	0.343433
aPFF0915w_1	PFF0915w	-0.6774	-1.098	-0.94073	0.223567	1.553837
aPFF0915w_0	PFF0915w	-0.6774	-1.098	-0.94073	0.223567	1.553837
intPFF0920c_2	PFF0920c	0.042552	0.265212	-0.11593	-0.12796	0.183232
intPFF0925w_2	PFF0920c-a	0.504705	0.667705	0.576705	-0.11364	-0.95085
aPFF0920c_2	PFF0920c-a	0.042552	0.265212	-0.11593	-0.12796	0.183232
aPFF0920c_1	PFF0920c-a	0.042552	0.265212	-0.11593	-0.12796	0.183232
aPFF0920c_0	PFF0920c-a	0.042552	0.265212	-0.11593	-0.12796	0.183232
aPFF0925w_0	PFF0925w	0.504705	0.667705	0.576705	-0.11364	-0.95085
intPFF0930w_2	PFF0930w	0.517007	0.617487	-0.22523	-0.62593	-0.28665
intPFF0935c_2	PFF0935c	-1.00259	-0.54576	0.129673	0.607793	0.644323
aPFF0935c_5	PFF0935c	-1.00259	-0.54576	0.129673	0.607793	0.644323
aPFF0935c_4	PFF0935c	-1.00259	-0.54576	0.129673	0.607793	0.644323
aPFF0935c_3	PFF0935c	-1.00259	-0.54576	0.129673	0.607793	0.644323
aPFF0935c_2	PFF0935c	-1.00259	-0.54576	0.129673	0.607793	0.644323
aPFF0935c_1	PFF0935c	-1.00259	-0.54576	0.129673	0.607793	0.644323
aPFF0935c_0	PFF0935c	-1.00259	-0.54576	0.129673	0.607793	0.644323
intPFF0940c_2	PFF0940c	-1.32603	-0.49589	0.105783	0.746383	1.037053
aPFF0940c_1	PFF0940c	-1.32603	-0.49589	0.105783	0.746383	1.037053
aPFF0940c_0	PFF0940c	-1.32603	-0.49589	0.105783	0.746383	1.037053
intPFF0950w_2	PFF0945c	-1.21837	0.122867	0.925517	0.815817	0.014967
aPFF0945c_2	PFF0945c	-0.21217	0.62584	0.54682	0.1784	-0.24695
aPFF0945c_1	PFF0945c	-0.21217	0.62584	0.54682	0.1784	-0.24695
aPFF0945c_0	PFF0945c	-0.21217	0.62584	0.54682	0.1784	-0.24695
intPFF0945c_2	PFF0950w	-0.21217	0.62584	0.54682	0.1784	-0.24695
aPFF0950w_1	PFF0950w	-1.21837	0.122867	0.925517	0.815817	0.014967
aPFF0950w_0	PFF0950w	-1.21837	0.122867	0.925517	0.815817	0.014967
intPFF0960c_2	PFF0960c	-0.25271	-0.18791	-0.50468	-0.12298	0.699747
aPFF0960c_1	PFF0960c	-0.25271	-0.18791	-0.50468	-0.12298	0.699747
aPFF0960c_0	PFF0960c	-0.25271	-0.18791	-0.50468	-0.12298	0.699747
aPFF0955c_0	PFF0960c					
intPFF0970w_2	PFF0965c	0.13542	0.25592	0.32367	0.01733	-0.44192
intPFF0965c_2	PFF0965c	-0.10505	0.517017	0.067727	-0.19914	-0.04318
aPFF0965c_3	PFF0965c	-0.10505	0.517017	0.067727	-0.19914	-0.04318
aPFF0965c_2	PFF0965c	-0.10505	0.517017	0.067727	-0.19914	-0.04318
aPFF0965c_1	PFF0965c	-0.10505	0.517017	0.067727	-0.19914	-0.04318
aPFF0965c_0	PFF0965c	-0.10505	0.517017	0.067727	-0.19914	-0.04318
aPFF0970w_0	PFF0970w	0.13542	0.25592	0.32367	0.01733	-0.44192
intPFF0975c_2	PFF0975c	-0.05682	-0.17381	0.01285	0.06431	-0.02403
aPFF0975c_1	PFF0975c	-0.05682	-0.17381	0.01285	0.06431	-0.02403
aPFF0975c_0	PFF0975c	-0.05682	-0.17381	0.01285	0.06431	-0.02403
intPFF0980w_2	PFF0980w	-1.03073	-0.93834	0.026458	0.834258	0.920908
aPFF0980w_0	PFF0980w	-1.03073	-0.93834	0.026458	0.834258	0.920908
intPFF0985c_2	PFF0985c					
aPFF0985c_0	PFF0985c					
aPFF0990c_1	PFF0990c	0.38198	-0.18089	-0.54722	-0.74928	-0.36861
aPFF0990c_0	PFF0990c	0.38198	-0.18089	-0.54722	-0.74928	-0.36861

intPFF0995c_2	PFF0995c	0.362647	-0.03324	-1.58876	-1.6234	0.334577
intPFF0990c_2	PFF0995c	0.38198	-0.18089	-0.54722	-0.74928	-0.36861
aPFF0995c_1	PFF0995c	0.362647	-0.03324	-1.58876	-1.6234	0.334577
aPFF0995c_0	PFF0995c	0.362647	-0.03324	-1.58876	-1.6234	0.334577
intPFF1005w_2	PFF1000w	0.89808	0.64162	-0.15975	-0.78525	-0.75662
intPFF1000w_2	PFF1000w	0.385763	0.045163	0.103433	-0.13227	-0.4865
aPFF1000w_1	PFF1000w	0.385763	0.045163	0.103433	-0.13227	-0.4865
aPFF1005w_0	PFF1005w	0.89808	0.64162	-0.15975	-0.78525	-0.75662
aPFF1010c_1	PFF1010c	-0.0457	-0.3335	-0.676	-0.3065	0.5725
aPFF1010c_0	PFF1010c	-0.0457	-0.3335	-0.676	-0.3065	0.5725
aPFF1015w_1	PFF1015w	-0.37654	0.106217	0.393067	0.332267	-0.02588
aPFF1015w_0	PFF1015w	-0.37654	0.106217	0.393067	0.332267	-0.02588
intPFF1020c_2	PFF1020c	-0.05109	-0.72787	-1.72117	-0.90111	1.335598
aPFF1020c_2	PFF1020c	-0.05109	-0.72787	-1.72117	-0.90111	1.335598
aPFF1020c_1	PFF1020c	-0.05109	-0.72787	-1.72117	-0.90111	1.335598
aPFF1020c_0	PFF1020c	-0.05109	-0.72787	-1.72117	-0.90111	1.335598
intPFF1025c_2	PFF1025c	-0.56054	0.543602	1.292332	0.615382	-0.89696
aPFF1025c_0	PFF1025c	-0.56054	0.543602	1.292332	0.615382	-0.89696
intPFF1030w_3	PFF1030w	0.83298	0.73609	0.86682	-0.15532	-1.60909
aPFF1030w_1	PFF1030w	0.83298	0.73609	0.86682	-0.15532	-1.60909
aPFF1030w_0	PFF1030w	0.83298	0.73609	0.86682	-0.15532	-1.60909
intPFF1035w_2	PFF1035w	0.56736	0.61719	-0.40139	-0.98861	-0.52469
aPFF1035w_1	PFF1035w	0.56736	0.61719	-0.40139	-0.98861	-0.52469
aPFF1035w_0	PFF1035w	0.56736	0.61719	-0.40139	-0.98861	-0.52469
intPFF1040w_2	PFF1040w	0.512257	0.049707	0.153077	0.013257	-0.39837
aPFF1040w_0	PFF1040w	0.512257	0.049707	0.153077	0.013257	-0.39837
intPFF1045w_2	PFF1045w	-0.16517	0.109878	0.574578	0.386198	-0.34577
aPFF1045w_2	PFF1045w	-0.16517	0.109878	0.574578	0.386198	-0.34577
aPFF1045w_1	PFF1045w	-0.16517	0.109878	0.574578	0.386198	-0.34577
aPFF1045w_0	PFF1045w	-0.16517	0.109878	0.574578	0.386198	-0.34577
intPFF1050w_1	PFF1050w	0.302337	0.362337	0.963837	0.403497	-1.021
aPFF1050w_0	PFF1050w	0.302337	0.362337	0.963837	0.403497	-1.021
aPFF1055c_1	PFF1055c	0.2473	0.37801	0.24198	-0.02498	-0.31151
aPFF1055c_0	PFF1055c	0.2473	0.37801	0.24198	-0.02498	-0.31151
intPFF1060w_3	PFF1060w	0.520505	-0.04027	0.313065	0.184045	-0.49824
intPFF1055c_2	PFF1060w	0.2473	0.37801	0.24198	-0.02498	-0.31151
aPFF1060w_0	PFF1060w	0.520505	-0.04027	0.313065	0.184045	-0.49824
intPFF1065c_2	PFF1065c	0.725343	0.827763	-0.54753	-0.90931	-0.0501
aPFF1065c_1	PFF1065c	0.725343	0.827763	-0.54753	-0.90931	-0.0501
aPFF1065c_0	PFF1065c	0.725343	0.827763	-0.54753	-0.90931	-0.0501
aPFF1070c_1	PFF1070c	0.54804	0.9553	0.7104	-0.2064	-1.1578
aPFF1070c_0	PFF1070c	0.54804	0.9553	0.7104	-0.2064	-1.1578
intPFF1080w_2	PFF1075w	-1.11831	-0.81643	0.60737	1.18163	0.48743
intPFF1075w_2	PFF1075w	0.066237	-0.15316	0.349817	0.465017	-0.068
intPFF1070c_2	PFF1075w	0.54804	0.9553	0.7104	-0.2064	-1.1578
aPFF1075w_1	PFF1075w	0.066237	-0.15316	0.349817	0.465017	-0.068
aPFF1075w_0	PFF1075w	0.066237	-0.15316	0.349817	0.465017	-0.068

aPFF1080w_0	PFF1080w	-1.11831	-0.81643	0.60737	1.18163	0.48743
intPFF1085c_2	PFF1085c	0.272167	1.094667	0.791667	-0.12233	-0.99583
aPFF1085c_1	PFF1085c	0.272167	1.094667	0.791667	-0.12233	-0.99583
aPFF1085c_0	PFF1085c	0.272167	1.094667	0.791667	-0.12233	-0.99583
intPFF1090c_2	PFF1090c	0.522497	0.239537	0.457417	0.212917	-0.5337
aPFF1090c_1	PFF1090c	0.522497	0.239537	0.457417	0.212917	-0.5337
aPFF1090c_0	PFF1090c	0.522497	0.239537	0.457417	0.212917	-0.5337
intPFF1095w_2	PFF1095w	0.229437	0.637167	0.430087	-0.02747	-0.48139
aPFF1095w_2	PFF1095w	0.229437	0.637167	0.430087	-0.02747	-0.48139
aPFF1095w_1	PFF1095w	0.229437	0.637167	0.430087	-0.02747	-0.48139
aPFF1095w_0	PFF1095w	0.229437	0.637167	0.430087	-0.02747	-0.48139
intPFF1100c_3	PFF1100c	1.380538	0.988648	-0.30663	-1.5387	-1.54698
aPFF1100c_2	PFF1100c	1.380538	0.988648	-0.30663	-1.5387	-1.54698
aPFF1100c_1	PFF1100c	1.380538	0.988648	-0.30663	-1.5387	-1.54698
aPFF1100c_0	PFF1100c	1.380538	0.988648	-0.30663	-1.5387	-1.54698
intPFF1105c_2	PFF1105c	-0.2821	-0.54999	0.214737	0.607097	0.244327
aPFF1105c_1	PFF1105c	-0.2821	-0.54999	0.214737	0.607097	0.244327
aPFF1105c_0	PFF1105c	-0.2821	-0.54999	0.214737	0.607097	0.244327
intPFF1110c_2	PFF1110c	0.227613	0.214423	0.394153	0.105513	-0.49726
aPFF1110c_1	PFF1110c	0.227613	0.214423	0.394153	0.105513	-0.49726
aPFF1110c_0	PFF1110c	0.227613	0.214423	0.394153	0.105513	-0.49726
intPFF1115w_2	PFF1115w	0.559667	0.443647	0.427957	-0.05162	-0.75281
aPFF1115w_1	PFF1115w	0.559667	0.443647	0.427957	-0.05162	-0.75281
aPFF1115w_0	PFF1115w	0.559667	0.443647	0.427957	-0.05162	-0.75281
intPFF1120c_2	PFF1120c	-0.14253	-0.74436	-0.38478	0.38178	0.82936
aPFF1120c_0	PFF1120c	-0.14253	-0.74436	-0.38478	0.38178	0.82936
intPFF1125c_2	PFF1125c	0.540787	-0.52093	-0.26064	0.221977	0.283267
aPFF1125c_1	PFF1125c	0.540787	-0.52093	-0.26064	0.221977	0.283267
aPFF1125c_0	PFF1125c	0.540787	-0.52093	-0.26064	0.221977	0.283267
intPFF1130c_3	PFF1130c	0.731643	0.402783	-0.15757	-0.31177	-0.15412
aPFF1130c_0	PFF1130c	0.731643	0.402783	-0.15757	-0.31177	-0.15412
intPFF1135w_3	PFF1135w	0.66696	0.35571	0.23258	-0.14808	-0.63371
aPFF1135w_1	PFF1135w	0.66696	0.35571	0.23258	-0.14808	-0.63371
aPFF1135w_0	PFF1135w	0.66696	0.35571	0.23258	-0.14808	-0.63371
intPFF1140c_2	PFF1140c	0.52925	-0.19226	0.08202	-0.01652	-0.47974
aPFF1140c_1	PFF1140c	0.52925	-0.19226	0.08202	-0.01652	-0.47974
aPFF1140c_0	PFF1140c	0.52925	-0.19226	0.08202	-0.01652	-0.47974
intPFF1145c_2	PFF1145c	1.723427	1.012137	0.124227	-1.59939	-2.52655
aPFF1145c_3	PFF1145c	1.723427	1.012137	0.124227	-1.59939	-2.52655
aPFF1145c_2	PFF1145c	1.723427	1.012137	0.124227	-1.59939	-2.52655
aPFF1145c_1	PFF1145c	1.723427	1.012137	0.124227	-1.59939	-2.52655
aPFF1145c_0	PFF1145c	1.723427	1.012137	0.124227	-1.59939	-2.52655
intPFF1150w_2	PFF1150w	-0.04801	-0.07991	0.268567	0.294267	-0.08825
aPFF1150w_0	PFF1150w	-0.04801	-0.07991	0.268567	0.294267	-0.08825
intPFF1155w_2	PFF1155w	-0.73328	-0.05159	0.59668	0.69432	0.15759
aPFF1155w_1	PFF1155w	-0.73328	-0.05159	0.59668	0.69432	0.15759
aPFF1155w_0	PFF1155w	-0.73328	-0.05159	0.59668	0.69432	0.15759

intPFF1160w_2	PFF1160w	-0.34125	-0.60271	-0.28308	0.383417	0.806047
aPFF1160w_0	PFF1160w	-0.34125	-0.60271	-0.28308	0.383417	0.806047
intPFF1170w_2	PFF1165c	-0.66882	-0.39577	0.267382	0.552522	0.297672
aPFF1165c_0	PFF1165c	-0.33072	-0.03741	-0.11225	0.083373	0.416153
intPFF1165c_2	PFF1170w	-0.33072	-0.03741	-0.11225	0.083373	0.416153
aPFF1170w_0	PFF1170w	-0.66882	-0.39577	0.267382	0.552522	0.297672
intPFF1175c_2	PFF1175c	-0.5834	-0.70849	0.033247	0.558087	0.498327
aPFF1175c_1	PFF1175c	-0.5834	-0.70849	0.033247	0.558087	0.498327
aPFF1175c_0	PFF1175c	-0.5834	-0.70849	0.033247	0.558087	0.498327
intPFF1180w_2	PFF1180w	-0.80255	-1.24224	-0.55152	0.49052	1.18624
aPFF1180w_0	PFF1180w	-0.80255	-1.24224	-0.55152	0.49052	1.18624
intPFF1185w_2	PFF1185w	-0.18011	-0.03744	-0.25171	-0.13401	0.295522
aPFF1185w_4	PFF1185w	-0.18011	-0.03744	-0.25171	-0.13401	0.295522
aPFF1185w_3	PFF1185w	-0.18011	-0.03744	-0.25171	-0.13401	0.295522
aPFF1185w_2	PFF1185w	-0.18011	-0.03744	-0.25171	-0.13401	0.295522
aPFF1185w_1	PFF1185w	-0.18011	-0.03744	-0.25171	-0.13401	0.295522
aPFF1185w_0	PFF1185w	-0.18011	-0.03744	-0.25171	-0.13401	0.295522
aPFF1190c_0	PFF1190c	-0.50614	-1.08131	-0.21539	0.72639	0.93231
intPFF1200w_2	PFF1195c	-0.40397	-0.73887	-0.06829	0.522607	0.551187
intPFF1195c_2	PFF1195c	-0.08642	-0.17836	-1.04551	-0.46265	1.132697
intPFF1190c_2	PFF1195c	-0.50614	-1.08131	-0.21539	0.72639	0.93231
aPFF1195c_1	PFF1195c	-0.08642	-0.17836	-1.04551	-0.46265	1.132697
aPFF1195c_0	PFF1195c	-0.08642	-0.17836	-1.04551	-0.46265	1.132697
aPFF1205w_0	PFF1200w	-1.26583	-0.97083	0.337667	1.097667	0.802167
aPFF1200w_0	PFF1200w	-0.40397	-0.73887	-0.06829	0.522607	0.551187
intPFF1215w_2	PFF1210w	-0.23187	-0.45715	-0.57044	0.018777	0.846487
intPFF1210w_2	PFF1210w	0.443857	-0.36228	-0.88293	-0.65773	0.242617
aPFF1210w_1	PFF1210w	0.443857	-0.36228	-0.88293	-0.65773	0.242617
aPFF1210w_0	PFF1210w	0.443857	-0.36228	-0.88293	-0.65773	0.242617
aPFF1215w_1	PFF1215w	-0.23187	-0.45715	-0.57044	0.018777	0.846487
aPFF1215w_0	PFF1215w	-0.23187	-0.45715	-0.57044	0.018777	0.846487
intPFF1220w_3	PFF1220w	-0.73445	-0.88473	-0.98852	0.093857	1.608067
aPFF1220w_0	PFF1220w	-0.73445	-0.88473	-0.98852	0.093857	1.608067
intPFF1225c_2	PFF1225c	-0.91517	-0.99484	-0.43915	0.540152	1.257172
aPFF1225c_2	PFF1225c	-0.91517	-0.99484	-0.43915	0.540152	1.257172
aPFF1225c_1	PFF1225c	-0.91517	-0.99484	-0.43915	0.540152	1.257172
aPFF1225c_0	PFF1225c	-0.91517	-0.99484	-0.43915	0.540152	1.257172
intPFF1230c_3	PFF1230c	0.766557	1.148697	0.759867	-1.08903	-2.51636
aPFF1230c_1	PFF1230c	0.766557	1.148697	0.759867	-1.08903	-2.51636
aPFF1230c_0	PFF1230c	0.766557	1.148697	0.759867	-1.08903	-2.51636
intPFF1235w_3	PFF1235w	-0.22918	-0.00113	0.081253	0.047413	-0.00221
aPFF1235w_0	PFF1235w	-0.22918	-0.00113	0.081253	0.047413	-0.00221
intPFF1240w_2	PFF1240w	0.88881	0.87445	0.37685	-0.64085	-1.36545
aPFF1240w_1	PFF1240w	0.88881	0.87445	0.37685	-0.64085	-1.36545
aPFF1240w_0	PFF1240w	0.88881	0.87445	0.37685	-0.64085	-1.36545
intPFF1245c_2	PFF1245c	-0.20671	0.051587	0.201317	0.349017	0.257747
aPFF1245c_0	PFF1245c	-0.20671	0.051587	0.201317	0.349017	0.257747

intPFF1250w_2	PFF1250w	1.082693	0.745543	-0.0336	-0.48074	-0.52338
aPFF1250w_1	PFF1250w	1.082693	0.745543	-0.0336	-0.48074	-0.52338
aPFF1250w_0	PFF1250w	1.082693	0.745543	-0.0336	-0.48074	-0.52338
intPFF1255w_2	PFF1255w	0.756158	0.907098	0.264248	-0.43195	-0.77969
aPFF1255w_0	PFF1255w	0.756158	0.907098	0.264248	-0.43195	-0.77969
intPFF1260c_2	PFF1260c	-0.30653	0.636972	0.799222	0.242672	-0.57442
aPFF1260c_2	PFF1260c	-0.30653	0.636972	0.799222	0.242672	-0.57442
aPFF1260c_1	PFF1260c	-0.30653	0.636972	0.799222	0.242672	-0.57442
aPFF1260c_0	PFF1260c	-0.30653	0.636972	0.799222	0.242672	-0.57442
intPFF1265w_2	PFF1265w	-0.09825	0.022753	0.194253	0.236413	0.046413
aPFF1265w_1	PFF1265w	-0.09825	0.022753	0.194253	0.236413	0.046413
aPFF1265w_0	PFF1265w	-0.09825	0.022753	0.194253	0.236413	0.046413
intPFF1270c_2	PFF1270c	0.720773	0.515613	0.396273	-0.08661	-0.75295
aPFF1270c_1	PFF1270c	0.720773	0.515613	0.396273	-0.08661	-0.75295
aPFF1270c_0	PFF1270c	0.720773	0.515613	0.396273	-0.08661	-0.75295
intPFF1275c_2	PFF1275c	0.17428	-0.32642	-0.14441	0.15791	0.22592
aPFF1275c_1	PFF1275c	0.17428	-0.32642	-0.14441	0.15791	0.22592
aPFF1275c_0	PFF1275c	0.17428	-0.32642	-0.14441	0.15791	0.22592
intPFF1280w_2	PFF1280w	-0.40702	-0.55188	-0.97774	-0.24826	1.18788
aPFF1280w_1	PFF1280w	-0.40702	-0.55188	-0.97774	-0.24826	1.18788
aPFF1280w_0	PFF1280w	-0.40702	-0.55188	-0.97774	-0.24826	1.18788
intPFF1285w_3	PFF1285w	0.464787	0.460557	-0.14412	-0.56204	-0.42672
aPFF1285w_1	PFF1285w	0.464787	0.460557	-0.14412	-0.56204	-0.42672
aPFF1285w_0	PFF1285w	0.464787	0.460557	-0.14412	-0.56204	-0.42672
intPFF1295w_2	PFF1290c	0.59702	0.69642	0.47166	-0.12566	-0.78792
aPFF1290c_0	PFF1290c	1.326793	0.414053	0.209893	-0.27823	-0.96839
intPFF1290c_2	PFF1295w	1.326793	0.414053	0.209893	-0.27823	-0.96839
aPFF1295w_1	PFF1295w	0.59702	0.69642	0.47166	-0.12566	-0.78792
aPFF1295w_0	PFF1295w	0.59702	0.69642	0.47166	-0.12566	-0.78792
intPFF1300w_3	PFF1300w	-0.62782	0.33961	0.71904	0.62696	0.05789
aPFF1300w_1	PFF1300w	-0.62782	0.33961	0.71904	0.62696	0.05789
aPFF1300w_0	PFF1300w	-0.62782	0.33961	0.71904	0.62696	0.05789
intPFF1305w_2	PFF1305w	-0.60792	-0.22939	0.176547	0.482787	0.450227
aPFF1305w_1	PFF1305w	-0.60792	-0.22939	0.176547	0.482787	0.450227
aPFF1305w_0	PFF1305w	-0.60792	-0.22939	0.176547	0.482787	0.450227
intPFF1310c_2	PFF1310c	0.6812	0.50701	0.03148	-0.43748	-0.60301
aPFF1310c_0	PFF1310c	0.6812	0.50701	0.03148	-0.43748	-0.60301
intPFF1315w_2	PFF1315w	0.906557	0.077227	-0.26987	-0.53234	-0.55445
aPFF1315w_2	PFF1315w	0.906557	0.077227	-0.26987	-0.53234	-0.55445
aPFF1315w_1	PFF1315w	0.906557	0.077227	-0.26987	-0.53234	-0.55445
aPFF1315w_0	PFF1315w	0.906557	0.077227	-0.26987	-0.53234	-0.55445
aPFF1320c_0	PFF1320c					
intPFF1325c_2	PFF1325c	-0.23089	-0.25408	-0.1481	0.160763	0.420243
intPFF1320c_2	PFF1325c					
aPFF1325c_1	PFF1325c	-0.23089	-0.25408	-0.1481	0.160763	0.420243
aPFF1325c_0	PFF1325c	-0.23089	-0.25408	-0.1481	0.160763	0.420243
intPFF1330c_2	PFF1330c	0.053007	0.214977	0.485037	0.351297	-0.23664

aPFF1330c_0	PFF1330c	0.053007	0.214977	0.485037	0.351297	-0.23664
intPFF1340w_2	PFF1335c	1.16771	0.84647	0.41056	-0.34856	-1.10047
aPFF1335c_0	PFF1335c	-0.31134	0.397727	0.831547	0.584787	-0.27839
intPFF1335c_2	PFF1340w	-0.31134	0.397727	0.831547	0.584787	-0.27839
aPFF1340w_1	PFF1340w	1.16771	0.84647	0.41056	-0.34856	-1.10047
aPFF1340w_0	PFF1340w	1.16771	0.84647	0.41056	-0.34856	-1.10047
intPFF1345w_2	PFF1345w	0.278623	0.140403	-0.03481	-0.08353	-0.06074
aPFF1345w_1	PFF1345w	0.278623	0.140403	-0.03481	-0.08353	-0.06074
aPFF1345w_0	PFF1345w	0.278623	0.140403	-0.03481	-0.08353	-0.06074
intPFF1350c_2	PFF1350c	0.008447	0.101757	0.259487	0.137847	-0.20392
aPFF1350c_1	PFF1350c	0.008447	0.101757	0.259487	0.137847	-0.20392
aPFF1350c_0	PFF1350c	0.008447	0.101757	0.259487	0.137847	-0.20392
intPFF1360w_2	PFF1355w	-0.24999	0.078977	0.872257	0.436477	-0.80844
intPFF1355w_2	PFF1355w	0.394908	0.113238	0.215918	-0.02866	-0.49498
aPFF1355w_0	PFF1355w	0.394908	0.113238	0.215918	-0.02866	-0.49498
aPFF1360w_0	PFF1360w	-0.24999	0.078977	0.872257	0.436477	-0.80844
intPFF1365c_2	PFF1365c	0.039535	-0.32874	-2.34254	-1.72985	1.467555
aPFF1365c_9	PFF1365c	0.039535	-0.32874	-2.34254	-1.72985	1.467555
aPFF1365c_8	PFF1365c	0.039535	-0.32874	-2.34254	-1.72985	1.467555
aPFF1365c_7	PFF1365c	0.039535	-0.32874	-2.34254	-1.72985	1.467555
aPFF1365c_6	PFF1365c	0.039535	-0.32874	-2.34254	-1.72985	1.467555
aPFF1365c_5	PFF1365c	0.039535	-0.32874	-2.34254	-1.72985	1.467555
aPFF1365c_4	PFF1365c	0.039535	-0.32874	-2.34254	-1.72985	1.467555
aPFF1365c_3	PFF1365c	0.039535	-0.32874	-2.34254	-1.72985	1.467555
aPFF1365c_2	PFF1365c	0.039535	-0.32874	-2.34254	-1.72985	1.467555
aPFF1365c_15	PFF1365c	0.039535	-0.32874	-2.34254	-1.72985	1.467555
aPFF1365c_14	PFF1365c	0.039535	-0.32874	-2.34254	-1.72985	1.467555
aPFF1365c_13	PFF1365c	0.039535	-0.32874	-2.34254	-1.72985	1.467555
aPFF1365c_12	PFF1365c	0.039535	-0.32874	-2.34254	-1.72985	1.467555
aPFF1365c_11	PFF1365c	0.039535	-0.32874	-2.34254	-1.72985	1.467555
aPFF1365c_10	PFF1365c	0.039535	-0.32874	-2.34254	-1.72985	1.467555
aPFF1365c_1	PFF1365c	0.039535	-0.32874	-2.34254	-1.72985	1.467555
aPFF1365c_0	PFF1365c	0.039535	-0.32874	-2.34254	-1.72985	1.467555
intPFF1370w_2	PFF1370w	0.224253	0.237343	-0.2051	-0.29184	0.021723
aPFF1370w_4	PFF1370w	0.224253	0.237343	-0.2051	-0.29184	0.021723
aPFF1370w_3	PFF1370w	0.224253	0.237343	-0.2051	-0.29184	0.021723
aPFF1370w_2	PFF1370w	0.224253	0.237343	-0.2051	-0.29184	0.021723
aPFF1370w_1	PFF1370w	0.224253	0.237343	-0.2051	-0.29184	0.021723
aPFF1370w_0	PFF1370w	0.224253	0.237343	-0.2051	-0.29184	0.021723
intPFF1375c_2	PFF1375c-a	0.79273	0.12783	-1.25466	-1.46634	-0.07833
aPFF1375c_1	PFF1375c-a	0.79273	0.12783	-1.25466	-1.46634	-0.07833
aPFF1375c_0	PFF1375c-a	0.79273	0.12783	-1.25466	-1.46634	-0.07833
intPFF1377w_2	PFF1377w	1.993023	0.446903	-0.8318	-1.49654	-1.08624
aPFF1377w_0	PFF1377w	1.993023	0.446903	-0.8318	-1.49654	-1.08624
aPFF1380c_0	PFF1380c	-0.15816	0.136817	0.632377	0.421957	-0.38248
intPFF1390w_2	PFF1385c	0.001337	0.109307	0.275897	0.127937	-0.24147
intPFF1380c_2	PFF1385c	-0.15816	0.136817	0.632377	0.421957	-0.38248

aPFF1385c_1	PFF1385c	0.380063	0.451703	0.515093	0.030573	-0.72404
aPFF1385c_0	PFF1385c	0.380063	0.451703	0.515093	0.030573	-0.72404
intPFF1385c_2	PFF1390w	0.380063	0.451703	0.515093	0.030573	-0.72404
aPFF1390w_1	PFF1390w	0.001337	0.109307	0.275897	0.127937	-0.24147
aPFF1390w_0	PFF1390w	0.001337	0.109307	0.275897	0.127937	-0.24147
intPFF1400w_2	PFF1395c	-0.9988	-0.37468	0.92786	1.05492	-0.03554
aPFF1395c_1	PFF1395c	-1.21948	-0.82808	0.54566	1.12634	0.54358
aPFF1395c_0	PFF1395c	-1.21948	-0.82808	0.54566	1.12634	0.54358
intPFF1395c_2	PFF1400w	-1.21948	-0.82808	0.54566	1.12634	0.54358
aPFF1400w_2	PFF1400w	-0.9988	-0.37468	0.92786	1.05492	-0.03554
aPFF1400w_1	PFF1400w	-0.9988	-0.37468	0.92786	1.05492	-0.03554
aPFF1400w_0	PFF1400w	-0.9988	-0.37468	0.92786	1.05492	-0.03554
intPFF1405c_2	PFF1405c	-0.56415	-0.73029	0.736573	1.058093	-0.06205
aPFF1405c_0	PFF1405c	-0.56415	-0.73029	0.736573	1.058093	-0.06205
intPFF1410c_2	PFF1410c	0.448183	-0.53042	-0.06918	0.261843	0.016583
aPFF1410c_1	PFF1410c	0.448183	-0.53042	-0.06918	0.261843	0.016583
aPFF1410c_0	PFF1410c	0.448183	-0.53042	-0.06918	0.261843	0.016583
intPFF1415c_3	PFF1415c	-0.77501	0.244053	0.614903	0.542263	0.107113
aPFF1415c_1	PFF1415c	-0.77501	0.244053	0.614903	0.542263	0.107113
aPFF1415c_0	PFF1415c	-0.77501	0.244053	0.614903	0.542263	0.107113
intPFF1420w_2	PFF1420w	1.270993	0.279633	-1.71147	-1.99833	-0.09705
aPFF1420w_1	PFF1420w	1.270993	0.279633	-1.71147	-1.99833	-0.09705
aPFF1420w_0	PFF1420w	1.270993	0.279633	-1.71147	-1.99833	-0.09705
intPFF1425w_2	PFF1425w	-0.8196	-0.54801	0.66003	0.95147	0.12501
aPFF1425w_1	PFF1425w	-0.8196	-0.54801	0.66003	0.95147	0.12501
aPFF1425w_0	PFF1425w	-0.8196	-0.54801	0.66003	0.95147	0.12501
intPFF1430c_3	PFF1430c	0.343423	0.312383	0.711973	-0.01481	-1.23372
aPFF1430c_1	PFF1430c	0.343423	0.312383	0.711973	-0.01481	-1.23372
aPFF1430c_0	PFF1430c	0.343423	0.312383	0.711973	-0.01481	-1.23372
intPFF1435w_3	PFF1435w	0.8008	0.58699	0.71202	-0.29202	-1.61899
aPFF1435w_0	PFF1435w	0.8008	0.58699	0.71202	-0.29202	-1.61899
intPFF1440w_3	PFF1440w	-0.09733	-0.4327	-0.53575	0.084153	0.852403
aPFF1440w_9	PFF1440w	-0.09733	-0.4327	-0.53575	0.084153	0.852403
aPFF1440w_8	PFF1440w	-0.09733	-0.4327	-0.53575	0.084153	0.852403
aPFF1440w_7	PFF1440w	-0.09733	-0.4327	-0.53575	0.084153	0.852403
aPFF1440w_6	PFF1440w	-0.09733	-0.4327	-0.53575	0.084153	0.852403
aPFF1440w_5	PFF1440w	-0.09733	-0.4327	-0.53575	0.084153	0.852403
aPFF1440w_4	PFF1440w	-0.09733	-0.4327	-0.53575	0.084153	0.852403
aPFF1440w_3	PFF1440w	-0.09733	-0.4327	-0.53575	0.084153	0.852403
aPFF1440w_2	PFF1440w	-0.09733	-0.4327	-0.53575	0.084153	0.852403
aPFF1440w_10	PFF1440w	-0.09733	-0.4327	-0.53575	0.084153	0.852403
aPFF1440w_1	PFF1440w	-0.09733	-0.4327	-0.53575	0.084153	0.852403
aPFF1440w_0	PFF1440w	-0.09733	-0.4327	-0.53575	0.084153	0.852403
intPFF1445c_2	PFF1445c	0.497613	0.247423	-0.16685	-0.30449	-0.15176
aPFF1445c_1	PFF1445c	0.497613	0.247423	-0.16685	-0.30449	-0.15176
aPFF1445c_0	PFF1445c	0.497613	0.247423	-0.16685	-0.30449	-0.15176
intPFF1450w_2	PFF1450w	0.933113	1.001423	-0.29735	-0.96699	-0.54876

aPFF1450w_0	PFF1450w	0.933113	1.001423	-0.29735	-0.96699	-0.54876
intPFF1455c_2	PFF1455c					
aPFF1455c_0	PFF1455c					
intPFF1460c_2	PFF1460c	0.975123	0.532863	0.271773	-0.82011	-1.6982
aPFF1460c_1	PFF1460c	0.975123	0.532863	0.271773	-0.82011	-1.6982
aPFF1460c_0	PFF1460c	0.975123	0.532863	0.271773	-0.82011	-1.6982
intPFF1465w_2	PFF1465w	1.864017	0.804917	-0.22985	-1.37025	-1.7187
aPFF1465w_0	PFF1465w	1.864017	0.804917	-0.22985	-1.37025	-1.7187
intPFF1470c_3	PFF1470c	-1.17197	-1.09749	-0.22598	0.836428	1.327938
aPFF1470c_4	PFF1470c	-1.17197	-1.09749	-0.22598	0.836428	1.327938
aPFF1470c_3	PFF1470c	-1.17197	-1.09749	-0.22598	0.836428	1.327938
aPFF1470c_2	PFF1470c	-1.17197	-1.09749	-0.22598	0.836428	1.327938
aPFF1470c_1	PFF1470c	-1.17197	-1.09749	-0.22598	0.836428	1.327938
aPFF1470c_0	PFF1470c	-1.17197	-1.09749	-0.22598	0.836428	1.327938
intPFF1475c_2	PFF1475c	-0.40532	-0.24294	-1.43564	-0.7857	1.426603
aPFF1475c_1	PFF1475c	-0.40532	-0.24294	-1.43564	-0.7857	1.426603
aPFF1475c_0	PFF1475c	-0.40532	-0.24294	-1.43564	-0.7857	1.426603
intPFF1480w_2	PFF1480w	0.607887	0.776557	0.908637	0.081697	-1.22822
aPFF1480w_1	PFF1480w	0.607887	0.776557	0.908637	0.081697	-1.22822
aPFF1480w_0	PFF1480w	0.607887	0.776557	0.908637	0.081697	-1.22822
intPFF1485w_2	PFF1485w	-0.66715	-0.16576	-0.19474	0.14124	0.68076
aPFF1485w_1	PFF1485w	-0.66715	-0.16576	-0.19474	0.14124	0.68076
aPFF1485w_0	PFF1485w	-0.66715	-0.16576	-0.19474	0.14124	0.68076
intPFF1490w_2	PFF1490w	0.75997	0.94465	0.22345	-0.47595	-0.74715
aPFF1490w_1	PFF1490w	0.75997	0.94465	0.22345	-0.47595	-0.74715
aPFF1490w_0	PFF1490w	0.75997	0.94465	0.22345	-0.47595	-0.74715
intPFF1495w_2	PFF1495w	-0.65778	-0.85711	-1.13728	-0.06006	1.642773
aPFF1495w_0	PFF1495w	-0.65778	-0.85711	-1.13728	-0.06006	1.642773
intPFF1500c_2	PFF1500c	0.8178	0.67751	0.78423	-0.18423	-1.53501
aPFF1500c_1	PFF1500c	0.8178	0.67751	0.78423	-0.18423	-1.53501
aPFF1500c_0	PFF1500c	0.8178	0.67751	0.78423	-0.18423	-1.53501
intPFF1505w_2	PFF1505w	-0.25326	-0.41469	-0.88312	-0.29088	0.990693
intPFF1510w_2	PFF1510w	0.37534	0.11932	-0.0969	-0.2546	-0.24982
intPFF1520w_2	PFF1520w	0.759423	0.851873	0.252743	-0.41308	-0.76821
intPFF1525c_2	PFF1525c					
intPFF1530c_2	PFF1530c	2.369367	1.858177	0.497647	-1.55831	-2.80084
intPFF1535w_2	PFF1535w	1.773967	1.661177	0.687147	-1.03481	-2.29584
intPFI0060c_2	PFI0060c	0.608327	0.554357	0.023797	-0.46546	-0.57502
intPFI0085c_2	PFI0085c	-0.89453	-0.86536	0.510223	1.115443	0.459023
aPFI0085c_0	PFI0085c	-0.89453	-0.86536	0.510223	1.115443	0.459023
intPFI0090c_2	PFI0090c	0.464707	0.190467	0.522067	0.303267	-0.53013
aPFI0090c_1	PFI0090c	0.464707	0.190467	0.522067	0.303267	-0.53013
aPFI0090c_0	PFI0090c	0.464707	0.190467	0.522067	0.303267	-0.53013
intPFI0095c_2	PFI0095c	0.8378	0.08448	0.82179	0.09471	-1.58748
aPFI0095c_1	PFI0095c	0.8378	0.08448	0.82179	0.09471	-1.58748
aPFI0095c_0	PFI0095c	0.8378	0.08448	0.82179	0.09471	-1.58748
aPFI0100c_1	PFI0100c	0.51909	-0.17827	-1.28361	-1.24949	0.18717

aPFI0100c_0	PFI0100c	0.51909	-0.17827	-1.28361	-1.24949	0.18717
intPFI0105c_2	PFI0105c	0.79446	0.83568	0.36614	-0.47614	-1.09318
aPFI0105c_1	PFI0105c	0.79446	0.83568	0.36614	-0.47614	-1.09318
aPFI0105c_0	PFI0105c	0.79446	0.83568	0.36614	-0.47614	-1.09318
intPFI0110c_2	PFI0110c	0.873807	1.521967	0.343577	-1.44474	-2.07363
aPFI0110c_1	PFI0110c	0.873807	1.521967	0.343577	-1.44474	-2.07363
aPFI0110c_0	PFI0110c	0.873807	1.521967	0.343577	-1.44474	-2.07363
intPFI0115c_2	PFI0115c	-0.1778	0.126007	0.395987	0.189347	-0.29867
aPFI0115c_1	PFI0115c	-0.1778	0.126007	0.395987	0.189347	-0.29867
aPFI0115c_0	PFI0115c	-0.1778	0.126007	0.395987	0.189347	-0.29867
intPFI0120c_2	PFI0120c	2.446277	1.561117	-0.52174	-2.48842	-2.55078
aPFI0120c_1	PFI0120c	2.446277	1.561117	-0.52174	-2.48842	-2.55078
aPFI0120c_0	PFI0120c	2.446277	1.561117	-0.52174	-2.48842	-2.55078
intPFI0125c_3	PFI0125c-a	1.954523	2.302383	0.794733	-1.21207	-2.39172
aPFI0125c_1	PFI0125c-a	1.954523	2.302383	0.794733	-1.21207	-2.39172
aPFI0125c_0	PFI0125c-a	1.954523	2.302383	0.794733	-1.21207	-2.39172
aPFI0130c_1	PFI0130c	-0.74223	0.052653	0.487953	0.478713	0.106013
aPFI0130c_0	PFI0130c	-0.74223	0.052653	0.487953	0.478713	0.106013
intPFI0135c_2	PFI0135c	-0.18426	-1.18667	-0.44091	0.685247	1.106507
intPFI0130c_2	PFI0135c	-0.74223	0.052653	0.487953	0.478713	0.106013
aPFI0135c_1	PFI0135c	-0.18426	-1.18667	-0.44091	0.685247	1.106507
aPFI0135c_0	PFI0135c	-0.18426	-1.18667	-0.44091	0.685247	1.106507
intPFI0140w_2	PFI0140w					
aPFI0140w_0	PFI0140w					
intPFI0145w_2	PFI0145w	-0.80546	-0.3457	0.5509	0.7391	0.1367
aPFI0145w_0	PFI0145w	-0.80546	-0.3457	0.5509	0.7391	0.1367
intPFI0150c_2	PFI0150c	0.273123	0.283363	0.639773	0.272893	-0.6657
aPFI0150c_0	PFI0150c	0.273123	0.283363	0.639773	0.272893	-0.6657
intPFI0155c_2	PFI0155c	-1.59492	-0.95639	0.440912	1.011392	0.602782
aPFI0155c_0	PFI0155c	-1.59492	-0.95639	0.440912	1.011392	0.602782
intPFI0160w_2	PFI0160w	-0.92758	-1.55967	-1.57557	0.216383	2.482533
aPFI0160w_4	PFI0160w	-0.92758	-1.55967	-1.57557	0.216383	2.482533
aPFI0160w_3	PFI0160w	-0.92758	-1.55967	-1.57557	0.216383	2.482533
aPFI0160w_2	PFI0160w	-0.92758	-1.55967	-1.57557	0.216383	2.482533
aPFI0160w_1	PFI0160w	-0.92758	-1.55967	-1.57557	0.216383	2.482533
aPFI0160w_0	PFI0160w	-0.92758	-1.55967	-1.57557	0.216383	2.482533
intPFI0165c_2	PFI0165c	0.74881	0.49043	0.24788	-0.29388	-0.81793
aPFI0165c_3	PFI0165c	0.74881	0.49043	0.24788	-0.29388	-0.81793
aPFI0165c_2	PFI0165c	0.74881	0.49043	0.24788	-0.29388	-0.81793
aPFI0165c_1	PFI0165c	0.74881	0.49043	0.24788	-0.29388	-0.81793
aPFI0165c_0	PFI0165c	0.74881	0.49043	0.24788	-0.29388	-0.81793
intPFI0170w_3	PFI0170w	0.94729	0.3999	0.0287	-0.40015	-0.70201
aPFI0170w_2	PFI0170w	0.94729	0.3999	0.0287	-0.40015	-0.70201
aPFI0170w_1	PFI0170w	0.94729	0.3999	0.0287	-0.40015	-0.70201
aPFI0170w_0	PFI0170w	0.94729	0.3999	0.0287	-0.40015	-0.70201
aPFI0175w_1	PFI0175w-a	1.473197	-0.36099	-2.01901	-2.04965	-0.10467
aPFI0175w_0	PFI0175w-a	1.473197	-0.36099	-2.01901	-2.04965	-0.10467

intPFI0175w_2	PFI0175w-b	1.473197	-0.36099	-2.01901	-2.04965	-0.10467
intPFI0180w_3	PFI0180w	-1.59625	-1.98375	-0.15975	1.525087	1.790087
aPFI0180w_1	PFI0180w	-1.59625	-1.98375	-0.15975	1.525087	1.790087
aPFI0180w_0	PFI0180w	-1.59625	-1.98375	-0.15975	1.525087	1.790087
intPFI0185w_2	PFI0185w	-0.49202	-0.27791	0.087992	0.268622	0.220352
aPFI0185w_2	PFI0185w	-0.49202	-0.27791	0.087992	0.268622	0.220352
aPFI0185w_1	PFI0185w	-0.49202	-0.27791	0.087992	0.268622	0.220352
aPFI0185w_0	PFI0185w	-0.49202	-0.27791	0.087992	0.268622	0.220352
intPFI0190w_2	PFI0190w	0.739743	0.405273	0.657963	0.312703	-0.71361
aPFI0190w_0	PFI0190w	0.739743	0.405273	0.657963	0.312703	-0.71361
intPFI0195c_3	PFI0195c	0.124943	0.181783	0.274923	0.142243	-0.21112
aPFI0195c_1	PFI0195c	0.124943	0.181783	0.274923	0.142243	-0.21112
aPFI0195c_0	PFI0195c	0.124943	0.181783	0.274923	0.142243	-0.21112
intPFI0200c_2	PFI0200c	0.182912	0.161432	-0.12203	-0.04964	0.195902
aPFI0200c_2	PFI0200c	0.182912	0.161432	-0.12203	-0.04964	0.195902
aPFI0200c_1	PFI0200c	0.182912	0.161432	-0.12203	-0.04964	0.195902
aPFI0200c_0	PFI0200c	0.182912	0.161432	-0.12203	-0.04964	0.195902
intPFI0205w_2	PFI0205w	-0.97535	0.037472	0.632282	0.599232	0.089772
aPFI0205w_0	PFI0205w	-0.97535	0.037472	0.632282	0.599232	0.089772
aPFI0210c_3	PFI0210c	-0.65414	-0.74329	-0.33949	0.355768	0.878568
aPFI0210c_2	PFI0210c	-0.65414	-0.74329	-0.33949	0.355768	0.878568
aPFI0210c_1	PFI0210c	-0.65414	-0.74329	-0.33949	0.355768	0.878568
aPFI0210c_0	PFI0210c	-0.65414	-0.74329	-0.33949	0.355768	0.878568
intPFI0220w_2	PFI0215c	0.29576	-0.33079	0.37458	0.58442	-0.12921
intPFI0215c_2	PFI0215c	-1.09114	-1.09128	-0.15493	0.868267	1.222617
intPFI0210c_2	PFI0215c	-0.65414	-0.74329	-0.33949	0.355768	0.878568
aPFI0215c_0	PFI0215c	-1.09114	-1.09128	-0.15493	0.868267	1.222617
aPFI0220w_0	PFI0220w	0.29576	-0.33079	0.37458	0.58442	-0.12921
intPFI0225w_3	PFI0225w	-0.07229	0.200775	-0.03139	-0.19462	-0.07645
aPFI0225w_2	PFI0225w	-0.07229	0.200775	-0.03139	-0.19462	-0.07645
aPFI0225w_1	PFI0225w	-0.07229	0.200775	-0.03139	-0.19462	-0.07645
aPFI0225w_0	PFI0225w	-0.07229	0.200775	-0.03139	-0.19462	-0.07645
intPFI0230c_2	PFI0230c	-0.45888	-2.09662	-0.83127	1.00827	1.79162
aPFI0230c_0	PFI0230c	-0.45888	-2.09662	-0.83127	1.00827	1.79162
intPFI0235w_2	PFI0235w	-1.3986	-1.25902	0.261273	1.285893	1.076683
aPFI0235w_1	PFI0235w	-1.3986	-1.25902	0.261273	1.285893	1.076683
aPFI0235w_0	PFI0235w	-1.3986	-1.25902	0.261273	1.285893	1.076683
intPFI0240c_2	PFI0240c	-1.0883	-1.05318	-0.61144	0.536743	1.586733
aPFI0240c_3	PFI0240c	-1.0883	-1.05318	-0.61144	0.536743	1.586733
aPFI0240c_2	PFI0240c	-1.0883	-1.05318	-0.61144	0.536743	1.586733
aPFI0240c_1	PFI0240c	-1.0883	-1.05318	-0.61144	0.536743	1.586733
aPFI0240c_0	PFI0240c	-1.0883	-1.05318	-0.61144	0.536743	1.586733
intPFI0245c_2	PFI0245c	-0.77166	-0.93471	-0.10359	0.824253	1.060373
aPFI0245c_0	PFI0245c	-0.77166	-0.93471	-0.10359	0.824253	1.060373
intPFI0250c_2	PFI0250c	0.102312	-0.05308	0.082792	0.105792	-0.06259
aPFI0250c_3	PFI0250c	0.102312	-0.05308	0.082792	0.105792	-0.06259
aPFI0250c_2	PFI0250c	0.102312	-0.05308	0.082792	0.105792	-0.06259

aPFI0250c_1	PFI0250c	0.102312	-0.05308	0.082792	0.105792	-0.06259
aPFI0250c_0	PFI0250c	0.102312	-0.05308	0.082792	0.105792	-0.06259
intPFI0255c_2	PFI0255c	0.641643	0.915803	0.363393	-0.43173	-0.91714
aPFI0255c_1	PFI0255c	0.641643	0.915803	0.363393	-0.43173	-0.91714
aPFI0255c_0	PFI0255c	0.641643	0.915803	0.363393	-0.43173	-0.91714
intPFI0260c_2	PFI0260c	0.359068	-0.02168	-0.18955	-0.21539	-0.10971
aPFI0260c_9	PFI0260c	0.359068	-0.02168	-0.18955	-0.21539	-0.10971
aPFI0260c_8	PFI0260c	0.359068	-0.02168	-0.18955	-0.21539	-0.10971
aPFI0260c_7	PFI0260c	0.359068	-0.02168	-0.18955	-0.21539	-0.10971
aPFI0260c_6	PFI0260c	0.359068	-0.02168	-0.18955	-0.21539	-0.10971
aPFI0260c_5	PFI0260c	0.359068	-0.02168	-0.18955	-0.21539	-0.10971
aPFI0260c_4	PFI0260c	0.359068	-0.02168	-0.18955	-0.21539	-0.10971
aPFI0260c_3	PFI0260c	0.359068	-0.02168	-0.18955	-0.21539	-0.10971
aPFI0260c_2	PFI0260c	0.359068	-0.02168	-0.18955	-0.21539	-0.10971
aPFI0260c_1	PFI0260c	0.359068	-0.02168	-0.18955	-0.21539	-0.10971
aPFI0260c_0	PFI0260c	0.359068	-0.02168	-0.18955	-0.21539	-0.10971
intPFI0265c_2	PFI0265c	-0.66924	-1.43829	-2.80917	-0.97149	2.957127
aPFI0265c_1	PFI0265c	-0.66924	-1.43829	-2.80917	-0.97149	2.957127
aPFI0265c_0	PFI0265c	-0.66924	-1.43829	-2.80917	-0.97149	2.957127
aPFI0270w_0	PFI0270w	-0.20893	-0.90938	-2.13271	-0.94531	1.955395
intPFI0275w_2	PFI0275w	-0.06924	-0.36547	0.035662	0.333042	0.213612
aPFI0275w_1	PFI0275w	-0.06924	-0.36547	0.035662	0.333042	0.213612
aPFI0275w_0	PFI0275w	-0.06924	-0.36547	0.035662	0.333042	0.213612
aPFI0280c_0	PFI0280c-a	-0.01524	-0.21081	0.335123	0.435543	-0.09653
intPFI0280c_2	PFI0280c-c	-0.01524	-0.21081	0.335123	0.435543	-0.09653
intPFI0285w_2	PFI0285w	0.552767	0.164177	0.109137	-0.1283	-0.46734
aPFI0285w_1	PFI0285w	0.552767	0.164177	0.109137	-0.1283	-0.46734
aPFI0285w_0	PFI0285w	0.552767	0.164177	0.109137	-0.1283	-0.46734
intPFI0290c_3	PFI0290c	0.858263	0.358693	-0.26413	-0.41321	-0.18253
aPFI0290c_1	PFI0290c	0.858263	0.358693	-0.26413	-0.41321	-0.18253
aPFI0290c_0	PFI0290c	0.858263	0.358693	-0.26413	-0.41321	-0.18253
intPFI0300w_2	PFI0295c	-0.78048	-0.8376	-0.0618	0.709463	0.879263
intPFI0295c_3	PFI0295c	-0.59405	-0.1421	0.61936	0.57798	-0.16524
aPFI0295c_2	PFI0295c	-0.59405	-0.1421	0.61936	0.57798	-0.16524
aPFI0295c_1	PFI0295c	-0.59405	-0.1421	0.61936	0.57798	-0.16524
aPFI0295c_0	PFI0295c	-0.59405	-0.1421	0.61936	0.57798	-0.16524
aPFI0300w_0	PFI0300w	-0.78048	-0.8376	-0.0618	0.709463	0.879263
intPFI0310w_2	PFI0305c	1.52143	1.05183	0.26285	-0.63735	-1.24383
intPFI0305c_2	PFI0305c	0.57364	0.3448	-0.12984	-0.25266	-0.1023
aPFI0305c_1	PFI0305c	0.57364	0.3448	-0.12984	-0.25266	-0.1023
aPFI0305c_0	PFI0305c	0.57364	0.3448	-0.12984	-0.25266	-0.1023
aPFI0310w_1	PFI0310w	1.52143	1.05183	0.26285	-0.63735	-1.24383
aPFI0310w_0	PFI0310w	1.52143	1.05183	0.26285	-0.63735	-1.24383
intPFI0315c_2	PFI0315c	1.49093	0.60683	-0.78865	-1.05185	-0.22433
aPFI0315c_1	PFI0315c	1.49093	0.60683	-0.78865	-1.05185	-0.22433
aPFI0315c_0	PFI0315c	1.49093	0.60683	-0.78865	-1.05185	-0.22433
intPFI0320w_2	PFI0320w	1.158343	0.413783	0.555183	-0.50102	-1.87962

aPFI0320w_1	PFI0320w	1.158343	0.413783	0.555183	-0.50102	-1.87962
aPFI0320w_0	PFI0320w	1.158343	0.413783	0.555183	-0.50102	-1.87962
intPFI0325c_1	PFI0325c	0.944617	1.404907	0.402687	-1.24535	-1.98457
aPFI0325c_1	PFI0325c	0.944617	1.404907	0.402687	-1.24535	-1.98457
aPFI0325c_0	PFI0325c	0.944617	1.404907	0.402687	-1.24535	-1.98457
aPFI0330c_1	PFI0328c	0.563727	0.631357	0.563037	-0.0592	-0.88802
intPFI0335w_2	PFI0330c	0.13189	0.37806	0.78589	0.37211	-0.67456
aPFI0330c_0	PFI0330c	0.563727	0.631357	0.563037	-0.0592	-0.88802
intPFI0330c_2	PFI0335w	0.563727	0.631357	0.563037	-0.0592	-0.88802
aPFI0335w_1	PFI0335w	0.13189	0.37806	0.78589	0.37211	-0.67456
aPFI0335w_0	PFI0335w	0.13189	0.37806	0.78589	0.37211	-0.67456
intPFI0340c_2	PFI0340c	-0.51436	-0.52923	0.323363	0.513913	-0.00333
aPFI0340c_2	PFI0340c	-0.51436	-0.52923	0.323363	0.513913	-0.00333
aPFI0340c_1	PFI0340c	-0.51436	-0.52923	0.323363	0.513913	-0.00333
aPFI0340c_0	PFI0340c	-0.51436	-0.52923	0.323363	0.513913	-0.00333
intPFI0345w_3	PFI0345w	0.658787	0.789067	-0.78114	-1.14552	0.020267
aPFI0345w_1	PFI0345w	0.658787	0.789067	-0.78114	-1.14552	0.020267
aPFI0345w_0	PFI0345w	0.658787	0.789067	-0.78114	-1.14552	0.020267
intPFI0350c_2	PFI0350c	0.307227	0.203887	0.715227	0.352107	-0.74955
aPFI0350c_0	PFI0350c	0.307227	0.203887	0.715227	0.352107	-0.74955
intPFI0355c_2	PFI0355c	-0.94657	-0.81012	-0.516	0.305337	1.179457
aPFI0355c_1	PFI0355c	-0.94657	-0.81012	-0.516	0.305337	1.179457
aPFI0355c_0	PFI0355c	-0.94657	-0.81012	-0.516	0.305337	1.179457
intPFI0360c_2	PFI0360c	0.08524	-0.11619	-1.35812	-1.14988	0.65919
aPFI0360c_0	PFI0360c	0.08524	-0.11619	-1.35812	-1.14988	0.65919
intPFI0365w_2	PFI0365w	0.961083	0.734093	0.017313	-0.79715	-1.05843
aPFI0365w_1	PFI0365w	0.961083	0.734093	0.017313	-0.79715	-1.05843
aPFI0365w_0	PFI0365w	0.961083	0.734093	0.017313	-0.79715	-1.05843
intPFI0370c_2	PFI0370c	0.02553	0.34636	0.49628	0.10672	-0.52736
aPFI0370c_0	PFI0370c	0.02553	0.34636	0.49628	0.10672	-0.52736
intPFI0375w_3	PFI0375w	0.00117	-0.39083	-0.58283	-0.08517	0.67983
aPFI0375w_0	PFI0375w	0.00117	-0.39083	-0.58283	-0.08517	0.67983
intPFI0380c_2	PFI0380c	-0.87127	-0.77268	0.357193	0.951953	0.548383
aPFI0380c_0	PFI0380c	-0.87127	-0.77268	0.357193	0.951953	0.548383
intPFI0385c_2	PFI0385c	-1.01749	-0.90154	-0.70542	0.15742	1.26754
aPFI0385c_0	PFI0385c	-1.01749	-0.90154	-0.70542	0.15742	1.26754
intPFI0390c_2	PFI0390c	-0.43344	-1.35978	-0.42144	0.53744	0.81678
aPFI0390c_0	PFI0390c	-0.43344	-1.35978	-0.42144	0.53744	0.81678
intPFI0395w_2	PFI0395w	-0.39467	-0.80433	-0.921	-0.115	1.11133
aPFI0395w_2	PFI0395w	-0.39467	-0.80433	-0.921	-0.115	1.11133
aPFI0395w_1	PFI0395w	-0.39467	-0.80433	-0.921	-0.115	1.11133
aPFI0395w_0	PFI0395w	-0.39467	-0.80433	-0.921	-0.115	1.11133
intPFI0400c_2	PFI0400c	-1.15427	-1.37067	-0.13266	1.07366	1.32467
aPFI0400c_0	PFI0400c	-1.15427	-1.37067	-0.13266	1.07366	1.32467
intPFI0405w_2	PFI0405w	-1.14877	-1.63415	-0.9337	0.686363	2.034813
aPFI0405w_0	PFI0405w	-1.14877	-1.63415	-0.9337	0.686363	2.034813
intPFI0410c_2	PFI0410c	0.470478	-0.0384	-1.68532	-1.54301	0.592818

aPFI0410c_3	PFI0410c	0.470478	-0.0384	-1.68532	-1.54301	0.592818
aPFI0410c_2	PFI0410c	0.470478	-0.0384	-1.68532	-1.54301	0.592818
aPFI0410c_1	PFI0410c	0.470478	-0.0384	-1.68532	-1.54301	0.592818
aPFI0410c_0	PFI0410c	0.470478	-0.0384	-1.68532	-1.54301	0.592818
aPFI0415c_1	PFI0415c	2.011533	0.445343	0.288313	-0.38165	-1.47468
aPFI0415c_0	PFI0415c	2.011533	0.445343	0.288313	-0.38165	-1.47468
intPFI0425w_2	PFI0420c	0.10443	0.38384	0.67783	0.20717	-0.71084
intPFI0415c_2	PFI0420c	2.011533	0.445343	0.288313	-0.38165	-1.47468
aPFI0420c_0	PFI0420c	0.200563	0.846163	0.702673	-0.12101	-0.9725
aPFI0425w_1	PFI0425w	0.10443	0.38384	0.67783	0.20717	-0.71084
aPFI0425w_0	PFI0425w	0.10443	0.38384	0.67783	0.20717	-0.71084
aPFI0430c_1	PFI0430c	0.549313	0.917433	0.427133	-0.17147	-0.61177
aPFI0430c_0	PFI0430c	0.549313	0.917433	0.427133	-0.17147	-0.61177
intPFI0430c_2	PFI0435c	0.549313	0.917433	0.427133	-0.17147	-0.61177
aPFI0435c_1	PFI0435c	-0.31072	-0.53239	-1.82572	-0.64828	2.12339
aPFI0435c_0	PFI0435c	-0.31072	-0.53239	-1.82572	-0.64828	2.12339
intPFI0440w_2	PFI0440w	-0.34995	-0.80878	-1.4253	-0.36618	1.641232
intPFI0435c_2	PFI0440w	-0.31072	-0.53239	-1.82572	-0.64828	2.12339
aPFI0440w_1	PFI0440w	-0.34995	-0.80878	-1.4253	-0.36618	1.641232
aPFI0440w_0	PFI0440w	-0.34995	-0.80878	-1.4253	-0.36618	1.641232
aPFI0445c_1	PFI0445c	0.86117	-0.05183	-0.30283	-0.37517	-0.32917
aPFI0445c_0	PFI0445c	0.86117	-0.05183	-0.30283	-0.37517	-0.32917
intPFI0450c_2	PFI0450c	0.998663	0.966663	0.184663	-0.47	-0.732
intPFI0445c_2	PFI0450c	0.86117	-0.05183	-0.30283	-0.37517	-0.32917
aPFI0450c_0	PFI0450c	0.998663	0.966663	0.184663	-0.47	-0.732
intPFI0455w_2	PFI0455w	0.098157	0.374697	0.264357	-0.03252	-0.31636
aPFI0455w_1	PFI0455w	0.098157	0.374697	0.264357	-0.03252	-0.31636
aPFI0455w_0	PFI0455w	0.098157	0.374697	0.264357	-0.03252	-0.31636
intPFI0460w_2	PFI0460w	-0.04873	-0.05135	-1.28605	-0.93495	0.93735
aPFI0460w_1	PFI0460w	-0.04873	-0.05135	-1.28605	-0.93495	0.93735
aPFI0460w_0	PFI0460w	-0.04873	-0.05135	-1.28605	-0.93495	0.93735
intPFI0465c_2	PFI0465c	-0.43411	-0.85091	-1.01417	0.021007	1.468247
aPFI0465c_1	PFI0465c	-0.43411	-0.85091	-1.01417	0.021007	1.468247
aPFI0465c_0	PFI0465c	-0.43411	-0.85091	-1.01417	0.021007	1.468247
intPFI0470w_2	PFI0470w	0.721587	0.746067	-0.26112	-1.10954	-0.92873
aPFI0470w_1	PFI0470w	0.721587	0.746067	-0.26112	-1.10954	-0.92873
aPFI0470w_0	PFI0470w	0.721587	0.746067	-0.26112	-1.10954	-0.92873
intPFI0475w_2	PFI0475w					
aPFI0475w_0	PFI0475w					
intPFI0480w_2	PFI0480w	0.717867	0.597677	0.135897	-0.32256	-0.57384
aPFI0480w_2	PFI0480w	0.717867	0.597677	0.135897	-0.32256	-0.57384
aPFI0480w_1	PFI0480w	0.717867	0.597677	0.135897	-0.32256	-0.57384
aPFI0480w_0	PFI0480w	0.717867	0.597677	0.135897	-0.32256	-0.57384
intPFI0485c_2	PFI0485c	-0.0122	0.129198	-0.38098	-0.33231	0.290198
aPFI0485c_1	PFI0485c	-0.0122	0.129198	-0.38098	-0.33231	0.290198
aPFI0485c_0	PFI0485c	-0.0122	0.129198	-0.38098	-0.33231	0.290198
intPFI0490c_2	PFI0490c	-0.38191	-0.07294	0.018647	0.305187	0.502277

aPFI0490c_1	PFI0490c	-0.38191	-0.07294	0.018647	0.305187	0.502277
aPFI0490c_0	PFI0490c	-0.38191	-0.07294	0.018647	0.305187	0.502277
intPFI0495w_2	PFI0495w	-0.06943	0.177547	-0.5177	-0.32976	0.595987
aPFI0495w_4	PFI0495w	-0.06943	0.177547	-0.5177	-0.32976	0.595987
aPFI0495w_3	PFI0495w	-0.06943	0.177547	-0.5177	-0.32976	0.595987
aPFI0495w_2	PFI0495w	-0.06943	0.177547	-0.5177	-0.32976	0.595987
aPFI0495w_1	PFI0495w	-0.06943	0.177547	-0.5177	-0.32976	0.595987
aPFI0495w_0	PFI0495w	-0.06943	0.177547	-0.5177	-0.32976	0.595987
intPFI0500w_2	PFI0500w	-0.40742	-0.07192	0.561833	0.427833	-0.30042
aPFI0500w_1	PFI0500w	-0.40742	-0.07192	0.561833	0.427833	-0.30042
aPFI0500w_0	PFI0500w	-0.40742	-0.07192	0.561833	0.427833	-0.30042
intPFI0505c_2	PFI0505c					
aPFI0505c_1	PFI0505c					
aPFI0505c_0	PFI0505c					
intPFI0510c_2	PFI0510c	0.282058	0.362378	-0.28992	-0.54208	-0.13404
aPFI0510c_2	PFI0510c	0.282058	0.362378	-0.28992	-0.54208	-0.13404
aPFI0510c_1	PFI0510c	0.282058	0.362378	-0.28992	-0.54208	-0.13404
aPFI0510c_0	PFI0510c	0.282058	0.362378	-0.28992	-0.54208	-0.13404
intPFI0520w_2	PFI0515w	0.294853	0.119733	-0.15147	-0.40787	-0.35257
intPFI0515w_2	PFI0515w	0.017257	-0.31529	0.107077	-0.01874	-0.44037
aPFI0515w_0	PFI0515w	0.017257	-0.31529	0.107077	-0.01874	-0.44037
aPFI0520w_1	PFI0520w	0.294853	0.119733	-0.15147	-0.40787	-0.35257
aPFI0520w_0	PFI0520w	0.294853	0.119733	-0.15147	-0.40787	-0.35257
intPFI0525w_2	PFI0525w	-0.09968	-0.31264	0.374023	0.526143	-0.0717
aPFI0525w_1	PFI0525w	-0.09968	-0.31264	0.374023	0.526143	-0.0717
aPFI0525w_0	PFI0525w	-0.09968	-0.31264	0.374023	0.526143	-0.0717
intPFI0530c_2	PFI0530c	-0.63865	-0.99276	-0.17774	0.77324	1.03676
aPFI0530c_1	PFI0530c	-0.63865	-0.99276	-0.17774	0.77324	1.03676
aPFI0530c_0	PFI0530c	-0.63865	-0.99276	-0.17774	0.77324	1.03676
intPFI0535w_2	PFI0535w	-0.38471	-1.26941	-0.62618	0.342517	0.943747
aPFI0535w_0	PFI0535w	-0.38471	-1.26941	-0.62618	0.342517	0.943747
intPFI0540w_2	PFI0540w	1.587673	0.080633	-2.10953	-2.55031	-0.43347
aPFI0540w_1	PFI0540w	1.587673	0.080633	-2.10953	-2.55031	-0.43347
aPFI0540w_0	PFI0540w	1.587673	0.080633	-2.10953	-2.55031	-0.43347
intPFI0545w_2	PFI0545w	-0.53411	-1.05542	-0.40915	0.525487	1.012757
aPFI0545w_0	PFI0545w	-0.53411	-1.05542	-0.40915	0.525487	1.012757
intPFI0550w_2	PFI0550w	0.033805	0.106285	0.068855	-0.03858	-0.12451
aPFI0550w_4	PFI0550w	0.033805	0.106285	0.068855	-0.03858	-0.12451
aPFI0550w_3	PFI0550w	0.033805	0.106285	0.068855	-0.03858	-0.12451
aPFI0550w_2	PFI0550w	0.033805	0.106285	0.068855	-0.03858	-0.12451
aPFI0550w_1	PFI0550w	0.033805	0.106285	0.068855	-0.03858	-0.12451
aPFI0550w_0	PFI0550w	0.033805	0.106285	0.068855	-0.03858	-0.12451
aPFI0555c_1	PFI0555c	-0.11544	0.3822	0.7956	0.3754	-0.6022
aPFI0555c_0	PFI0555c	-0.11544	0.3822	0.7956	0.3754	-0.6022
intPFI0560c_2	PFI0560c-a	-0.54816	-0.28425	0.117645	0.507545	0.538865
intPFI0555c_2	PFI0560c-a	-0.11544	0.3822	0.7956	0.3754	-0.6022
aPFI0560c_0	PFI0560c-a	-0.54816	-0.28425	0.117645	0.507545	0.538865

intPFI0570w_2	PFI0565w	-0.53223	-0.21384	0.217398	0.416218	0.260608
intPFI0565w_2	PFI0565w	-0.91071	-1.03342	-0.46991	0.571747	1.333757
aPFI0565w_0	PFI0565w	-0.91071	-1.03342	-0.46991	0.571747	1.333757
aPFI0570w_1	PFI0570w	-0.53223	-0.21384	0.217398	0.416218	0.260608
aPFI0570w_0	PFI0570w	-0.53223	-0.21384	0.217398	0.416218	0.260608
intPFI0575c_2	PFI0575c	0.12852	-0.89162	-1.38926	-0.40074	1.30862
aPFI0575c_0	PFI0575c	0.12852	-0.89162	-1.38926	-0.40074	1.30862
intPFI0580c_2	PFI0580c	0.624803	0.703993	-0.10999	-0.79135	-0.72433
aPFI0580c_1	PFI0580c	0.624803	0.703993	-0.10999	-0.79135	-0.72433
aPFI0580c_0	PFI0580c	0.624803	0.703993	-0.10999	-0.79135	-0.72433
intPFI0585c_2	PFI0585c	0.105782	-0.45759	0.011432	0.395622	0.232152
aPFI0585c_2	PFI0585c	0.105782	-0.45759	0.011432	0.395622	0.232152
aPFI0585c_1	PFI0585c	0.105782	-0.45759	0.011432	0.395622	0.232152
aPFI0585c_0	PFI0585c	0.105782	-0.45759	0.011432	0.395622	0.232152
intPFI0590c_2	PFI0590c	0.50662	0.13088	0.05498	-0.07798	-0.29938
aPFI0590c_1	PFI0590c	0.50662	0.13088	0.05498	-0.07798	-0.29938
aPFI0590c_0	PFI0590c	0.50662	0.13088	0.05498	-0.07798	-0.29938
intPFI0595c_2	PFI0595c					
aPFI0595c_1	PFI0595c					
aPFI0595c_0	PFI0595c					
intPFI0605c_2	PFI0605c	-0.94505	-1.19921	-0.05332	0.985157	1.091047
aPFI0605c_1	PFI0605c	-0.94505	-1.19921	-0.05332	0.985157	1.091047
aPFI0605c_0	PFI0605c	-0.94505	-1.19921	-0.05332	0.985157	1.091047
intPFI0610w_2	PFI0610w	0.568563	0.150203	0.187093	0.006573	-0.42254
aPFI0610w_0	PFI0610w	0.568563	0.150203	0.187093	0.006573	-0.42254
intPFI0620w_2	PFI0615w	0.430633	-0.33818	0.216353	0.288313	-0.33316
intPFI0615w_2	PFI0615w	-0.26823	0.191143	-0.17654	-0.1533	0.310523
aPFI0615w_1	PFI0615w	-0.26823	0.191143	-0.17654	-0.1533	0.310523
aPFI0615w_0	PFI0615w	-0.26823	0.191143	-0.17654	-0.1533	0.310523
aPFI0620w_0	PFI0620w	0.430633	-0.33818	0.216353	0.288313	-0.33316
intPFI0625c_2	PFI0625c	0.245913	0.464443	0.750353	0.151813	-0.92028
aPFI0625c_1	PFI0625c	0.245913	0.464443	0.750353	0.151813	-0.92028
aPFI0625c_0	PFI0625c	0.245913	0.464443	0.750353	0.151813	-0.92028
intPFI0630w_2	PFI0630w	-0.23089	-0.16555	0.214107	0.379227	0.142887
aPFI0630w_0	PFI0630w	-0.23089	-0.16555	0.214107	0.379227	0.142887
intPFI0635c_2	PFI0635c	0.827677	0.611627	0.698747	-0.05141	-1.22629
aPFI0635c_0	PFI0635c	0.827677	0.611627	0.698747	-0.05141	-1.22629
intPFI0640c_2	PFI0640c	-1.00939	-0.59855	0.933593	1.162573	-0.03029
aPFI0640c_1	PFI0640c	-1.00939	-0.59855	0.933593	1.162573	-0.03029
aPFI0640c_0	PFI0640c	-1.00939	-0.59855	0.933593	1.162573	-0.03029
intPFI0645w_2	PFI0645w	-0.08374	0.197707	0.706077	0.604257	-0.22837
aPFI0645w_0	PFI0645w	-0.08374	0.197707	0.706077	0.604257	-0.22837
intPFI0650c_2	PFI0650c	0.521447	1.197787	0.631177	-0.30734	-0.99345
aPFI0650c_1	PFI0650c	0.521447	1.197787	0.631177	-0.30734	-0.99345
aPFI0650c_0	PFI0650c	0.521447	1.197787	0.631177	-0.30734	-0.99345
intPFI0655c_2	PFI0655c	-0.00991	-0.26246	0.713913	0.697753	-0.43388
aPFI0655c_0	PFI0655c	-0.00991	-0.26246	0.713913	0.697753	-0.43388

intPFI0665w_2	PFI0660c	-0.7869	-1.23851	-0.52174	0.58424	1.26951
aPFI0660c_1	PFI0660c	-0.38492	-0.1539	-0.15971	0.194373	0.610063
aPFI0660c_0	PFI0660c	-0.38492	-0.1539	-0.15971	0.194373	0.610063
intPFI0660c_2	PFI0665w	-0.38492	-0.1539	-0.15971	0.194373	0.610063
aPFI0665w_1	PFI0665w	-0.7869	-1.23851	-0.52174	0.58424	1.26951
aPFI0665w_0	PFI0665w	-0.7869	-1.23851	-0.52174	0.58424	1.26951
intPFI0670w_2	PFI0670w	-1.41064	-0.89428	0.29805	1.07545	0.94078
aPFI0670w_1	PFI0670w	-1.41064	-0.89428	0.29805	1.07545	0.94078
aPFI0670w_0	PFI0670w	-1.41064	-0.89428	0.29805	1.07545	0.94078
intPFI0675w_2	PFI0675w	1.420077	0.166627	-1.78124	-2.39242	-0.68529
aPFI0675w_0	PFI0675w	1.420077	0.166627	-1.78124	-2.39242	-0.68529
intPFI0685w_2	PFI0680c	0.45754	0.4993	0.6359	-0.0159	-1.0093
aPFI0680c_1	PFI0680c	0.755393	0.419543	0.675163	0.112003	-1.04188
aPFI0680c_0	PFI0680c	0.755393	0.419543	0.675163	0.112003	-1.04188
intPFI0680c_2	PFI0685w	0.755393	0.419543	0.675163	0.112003	-1.04188
aPFI0685w_1	PFI0685w	0.45754	0.4993	0.6359	-0.0159	-1.0093
aPFI0685w_0	PFI0685w	0.45754	0.4993	0.6359	-0.0159	-1.0093
intPFI0690c_2	PFI0690c	0.307283	-0.23842	-1.11217	-1.16867	0.024583
aPFI0690c_1	PFI0690c	0.307283	-0.23842	-1.11217	-1.16867	0.024583
aPFI0690c_0	PFI0690c	0.307283	-0.23842	-1.11217	-1.16867	0.024583
intPFI0695c_3	PFI0695c	0.77863	0.19837	0.11627	-0.48527	-1.06837
aPFI0695c_1	PFI0695c	0.77863	0.19837	0.11627	-0.48527	-1.06837
aPFI0695c_0	PFI0695c	0.77863	0.19837	0.11627	-0.48527	-1.06837
intPFI0700c_2	PFI0700c	1.287043	1.063273	-0.24505	-1.18879	-1.05411
aPFI0700c_1	PFI0700c	1.287043	1.063273	-0.24505	-1.18879	-1.05411
aPFI0700c_0	PFI0700c	1.287043	1.063273	-0.24505	-1.18879	-1.05411
intPFI0705w_2	PFI0705w	-0.12608	-1.00362	-1.29875	-0.33025	1.25862
aPFI0705w_0	PFI0705w	-0.12608	-1.00362	-1.29875	-0.33025	1.25862
aPFI0710c_2	PFI0710c	0.127512	0.152442	-0.66488	-0.51212	0.509892
aPFI0710c_1	PFI0710c	0.127512	0.152442	-0.66488	-0.51212	0.509892
aPFI0710c_0	PFI0710c	0.127512	0.152442	-0.66488	-0.51212	0.509892
intPFI0715w_3	PFI0715w	0.10104	-0.17618	-0.13014	-0.01486	0.06518
aPFI0715w_0	PFI0715w	0.10104	-0.17618	-0.13014	-0.01486	0.06518
intPFI0720w_3	PFI0720w	0.762457	0.345237	-0.00872	-0.17794	-0.2749
aPFI0720w_1	PFI0720w	0.762457	0.345237	-0.00872	-0.17794	-0.2749
aPFI0720w_0	PFI0720w	0.762457	0.345237	-0.00872	-0.17794	-0.2749
intPFI0725c_2	PFI0725c	-0.7895	-1.12648	-0.46504	0.469377	1.068817
aPFI0725c_0	PFI0725c	-0.7895	-1.12648	-0.46504	0.469377	1.068817
intPFI0730w_2	PFI0730w	-0.07489	0.22294	-0.20639	-0.33261	0.05306
aPFI0730w_1	PFI0730w	-0.07489	0.22294	-0.20639	-0.33261	0.05306
aPFI0730w_0	PFI0730w	-0.07489	0.22294	-0.20639	-0.33261	0.05306
intPFI0735c_2	PFI0735c	-0.442	-0.8205	-0.929	-0.072	1.1955
aPFI0735c_1	PFI0735c	-0.442	-0.8205	-0.929	-0.072	1.1955
aPFI0735c_0	PFI0735c	-0.442	-0.8205	-0.929	-0.072	1.1955
intPFI0740c_2	PFI0740c	0.127777	-0.04887	0.290737	0.355597	-0.07479
aPFI0740c_0	PFI0740c	0.127777	-0.04887	0.290737	0.355597	-0.07479
intPFI0745w_2	PFI0745w	0.42148	0.51562	0.20426	-0.10226	-0.32462

aPFI0745w_0	PFI0745w	0.42148	0.51562	0.20426	-0.10226	-0.32462
intPFI0750c_2	PFI0750c	-0.50047	0.122313	1.089373	0.713793	-0.67815
aPFI0750c_1	PFI0750c	-0.50047	0.122313	1.089373	0.713793	-0.67815
aPFI0750c_0	PFI0750c	-0.50047	0.122313	1.089373	0.713793	-0.67815
intPFI0755c_2	PFI0755c	0.0548	0.60866	0.62643	0.19074	-0.47483
aPFI0755c_2	PFI0755c	0.0548	0.60866	0.62643	0.19074	-0.47483
aPFI0755c_1	PFI0755c	0.0548	0.60866	0.62643	0.19074	-0.47483
aPFI0755c_0	PFI0755c	0.0548	0.60866	0.62643	0.19074	-0.47483
intPFI0760w_2	PFI0760w	0.171597	0.030027	0.543457	0.365877	-0.48469
aPFI0760w_1	PFI0760w	0.171597	0.030027	0.543457	0.365877	-0.48469
aPFI0760w_0	PFI0760w	0.171597	0.030027	0.543457	0.365877	-0.48469
intPFI0765w_2	PFI0765w					
aPFI0765w_0	PFI0765w					
intPFI0775w_2	PFI0770c	0.69275	-0.24077	-0.08346	-0.11254	-0.40723
intPFI0770c_2	PFI0770c	-0.64207	-0.77662	-0.71525	0.173587	1.267957
aPFI0770c_0	PFI0770c	-0.64207	-0.77662	-0.71525	0.173587	1.267957
aPFI0775w_0	PFI0775w	0.69275	-0.24077	-0.08346	-0.11254	-0.40723
intPFI0780w_2	PFI0780w	0.122103	0.089463	0.147073	0.142593	-0.0368
aPFI0780w_0	PFI0780w	0.122103	0.089463	0.147073	0.142593	-0.0368
intPFI0785c_2	PFI0785c	0.49716	-0.07213	-2.04958	-1.59309	1.15413
aPFI0785c_2	PFI0785c	0.49716	-0.07213	-2.04958	-1.59309	1.15413
aPFI0785c_1	PFI0785c	0.49716	-0.07213	-2.04958	-1.59309	1.15413
aPFI0785c_0	PFI0785c	0.49716	-0.07213	-2.04958	-1.59309	1.15413
intPFI0795w_2	PFI0790w	-0.1141	-0.42203	0.457043	0.597623	-0.18481
intPFI0790w_2	PFI0790w					
aPFI0790w_0	PFI0790w					
aPFI0795w_1	PFI0795w	-0.1141	-0.42203	0.457043	0.597623	-0.18481
aPFI0795w_0	PFI0795w	-0.1141	-0.42203	0.457043	0.597623	-0.18481
aPFI0800c_0	PFI0800c	-0.26492	-0.55038	-1.12425	-0.28275	1.35838
intPFI0805w_2	PFI0805w	0.496438	0.025808	-0.468	-0.41708	0.084108
intPFI0800c_2	PFI0805w	-0.26492	-0.55038	-1.12425	-0.28275	1.35838
aPFI0805w_3	PFI0805w	0.496438	0.025808	-0.468	-0.41708	0.084108
aPFI0805w_2	PFI0805w	0.496438	0.025808	-0.468	-0.41708	0.084108
aPFI0805w_1	PFI0805w	0.496438	0.025808	-0.468	-0.41708	0.084108
aPFI0805w_0	PFI0805w	0.496438	0.025808	-0.468	-0.41708	0.084108
intPFI0810c_2	PFI0810c	-0.07736	-0.99717	0.278847	0.808487	0.062507
aPFI0810c_0	PFI0810c	-0.07736	-0.99717	0.278847	0.808487	0.062507
intPFI0815c_2	PFI0815c	1.911813	1.200913	0.532673	-0.68801	-1.84525
aPFI0815c_0	PFI0815c	1.911813	1.200913	0.532673	-0.68801	-1.84525
intPFI0820c_2	PFI0820c	1.885593	0.903043	-0.38284	-1.4935	-1.56488
aPFI0820c_1	PFI0820c	1.885593	0.903043	-0.38284	-1.4935	-1.56488
aPFI0820c_0	PFI0820c	1.885593	0.903043	-0.38284	-1.4935	-1.56488
intPFI0823w_2	PFI0823w					
intPFI0825w_2	PFI0825w	0.59396	0.1742	-0.4544	-0.4916	0.0138
aPFI0825w_0	PFI0825w	0.59396	0.1742	-0.4544	-0.4916	0.0138
intPFI0830c_2	PFI0830c	-0.49943	-0.24286	-0.26295	0.11051	0.65241
aPFI0830c_2	PFI0830c	-0.49943	-0.24286	-0.26295	0.11051	0.65241

aPFI0830c_1	PFI0830c	-0.49943	-0.24286	-0.26295	0.11051	0.65241
aPFI0830c_0	PFI0830c	-0.49943	-0.24286	-0.26295	0.11051	0.65241
intPFI0840w_2	PFI0835c	0.161013	0.131233	-0.14238	-0.2509	-0.08151
aPFI0835c_1	PFI0835c	0.504813	0.451413	0.214173	-0.15351	-0.49025
aPFI0835c_0	PFI0835c	0.504813	0.451413	0.214173	-0.15351	-0.49025
intPFI0835c_2	PFI0840w	0.504813	0.451413	0.214173	-0.15351	-0.49025
aPFI0840w_0	PFI0840w	0.161013	0.131233	-0.14238	-0.2509	-0.08151
intPFI0845w_2	PFI0845w	-0.16323	-0.25081	-1.57138	-1.11728	1.092147
aPFI0845w_0	PFI0845w	-0.16323	-0.25081	-1.57138	-1.11728	1.092147
intPFI0850w_2	PFI0850w	0.43598	-0.1674	-1.3612	-0.9978	0.7419
aPFI0850w_1	PFI0850w	0.43598	-0.1674	-1.3612	-0.9978	0.7419
aPFI0850w_0	PFI0850w	0.43598	-0.1674	-1.3612	-0.9978	0.7419
intPFI0855w_2	PFI0855w	-0.47203	-0.40033	-0.36283	0.212667	0.874667
aPFI0855w_1	PFI0855w	-0.47203	-0.40033	-0.36283	0.212667	0.874667
aPFI0855w_0	PFI0855w	-0.47203	-0.40033	-0.36283	0.212667	0.874667
intPFI0865w_2	PFI0860c	0.934663	0.901283	0.400233	-0.37797	-1.01142
aPFI0860c_1	PFI0860c	0.408203	0.703983	0.363773	-0.19011	-0.61882
aPFI0860c_0	PFI0860c	0.408203	0.703983	0.363773	-0.19011	-0.61882
intPFI0870w_2	PFI0865w	0.402437	0.564337	0.202827	-0.20949	-0.444
intPFI0860c_2	PFI0865w	0.408203	0.703983	0.363773	-0.19011	-0.61882
aPFI0865w_0	PFI0865w	0.934663	0.901283	0.400233	-0.37797	-1.01142
aPFI0870w_1	PFI0870w	0.402437	0.564337	0.202827	-0.20949	-0.444
aPFI0870w_0	PFI0870w	0.402437	0.564337	0.202827	-0.20949	-0.444
intPFI0875w_1	PFI0875w	-0.80427	-0.10914	-0.35897	0.22747	1.19464
aPFI0875w_1	PFI0875w	-0.80427	-0.10914	-0.35897	0.22747	1.19464
aPFI0875w_0	PFI0875w	-0.80427	-0.10914	-0.35897	0.22747	1.19464
intPFI0880c_2	PFI0880c	-1.32418	-2.05558	-1.63435	0.44585	2.73608
aPFI0880c_1	PFI0880c	-1.32418	-2.05558	-1.63435	0.44585	2.73608
aPFI0880c_0	PFI0880c	-1.32418	-2.05558	-1.63435	0.44585	2.73608
intPFI0885w_2	PFI0885w	0.606297	0.651537	-0.32656	-0.7381	-0.2762
aPFI0885w_0	PFI0885w	0.606297	0.651537	-0.32656	-0.7381	-0.2762
intPFI0890c_2	PFI0890c-a	0.17781	-0.51105	-0.3159	0.1879	0.46105
aPFI0890c_0	PFI0890c-a	0.17781	-0.51105	-0.3159	0.1879	0.46105
intPFI0895c_2	PFI0895c	0.390577	0.132107	0.809297	0.396037	-0.93177
aPFI0895c_0	PFI0895c	0.390577	0.132107	0.809297	0.396037	-0.93177
intPFI0900w_2	PFI0900w					
intPFI0905w_2	PFI0905w	-0.97941	-1.84698	-0.98205	0.788713	2.078643
aPFI0905w_0	PFI0905w	-0.97941	-1.84698	-0.98205	0.788713	2.078643
intPFI0910w_2	PFI0910w	-0.93548	0.16388	0.48974	0.42326	0.15812
aPFI0910w_1	PFI0910w	-0.93548	0.16388	0.48974	0.42326	0.15812
aPFI0910w_0	PFI0910w	-0.93548	0.16388	0.48974	0.42326	0.15812
intPFI0915w_2	PFI0915w	1.825567	0.980647	-0.83953	-2.26513	-1.81331
aPFI0915w_1	PFI0915w	1.825567	0.980647	-0.83953	-2.26513	-1.81331
aPFI0915w_0	PFI0915w	1.825567	0.980647	-0.83953	-2.26513	-1.81331
intPFI0920c_3	PFI0920c	0.818447	0.805757	0.703737	-0.3194	-1.50492
aPFI0920c_1	PFI0920c	0.818447	0.805757	0.703737	-0.3194	-1.50492
aPFI0920c_0	PFI0920c	0.818447	0.805757	0.703737	-0.3194	-1.50492

intPFI0925w_2	PFI0925w	0.29139	0.20055	0.45791	0.04659	-0.73305
aPFI0925w_1	PFI0925w	0.29139	0.20055	0.45791	0.04659	-0.73305
aPFI0925w_0	PFI0925w	0.29139	0.20055	0.45791	0.04659	-0.73305
intPFI0930c_3	PFI0930c	0.50261	0.18396	-0.17743	-0.13557	0.07704
aPFI0930c_0	PFI0930c	0.50261	0.18396	-0.17743	-0.13557	0.07704
intPFI0935w_2	PFI0935w	-0.35411	0.02803	0.0837	0.2448	0.35047
aPFI0935w_1	PFI0935w	-0.35411	0.02803	0.0837	0.2448	0.35047
aPFI0935w_0	PFI0935w	-0.35411	0.02803	0.0837	0.2448	0.35047
intPFI0940c_2	PFI0940c	1.132677	0.052627	-1.61075	-2.12591	-0.57829
aPFI0940c_0	PFI0940c	1.132677	0.052627	-1.61075	-2.12591	-0.57829
intPFI0945w_2	PFI0945w	1.423923	0.445373	-1.52926	-2.13108	-0.57071
aPFI0945w_0	PFI0945w	1.423923	0.445373	-1.52926	-2.13108	-0.57071
intPFI0950w_2	PFI0950w	-1.12674	-1.6693	-1.0709	0.705737	2.284637
aPFI0950w_1	PFI0950w	-1.12674	-1.6693	-1.0709	0.705737	2.284637
aPFI0950w_0	PFI0950w	-1.12674	-1.6693	-1.0709	0.705737	2.284637
intPFI0960w_2	PFI0955w	-0.6474	-1.081	0.55249	1.20951	0.2965
intPFI0955w_2	PFI0955w	0.111323	-0.82513	-0.62475	0.077413	0.674293
aPFI0955w_1	PFI0955w	0.111323	-0.82513	-0.62475	0.077413	0.674293
aPFI0955w_0	PFI0955w	0.111323	-0.82513	-0.62475	0.077413	0.674293
aPFI0960w_1	PFI0960w	-0.6474	-1.081	0.55249	1.20951	0.2965
aPFI0960w_0	PFI0960w	-0.6474	-1.081	0.55249	1.20951	0.2965
intPFI0965w_2	PFI0965w	-0.15994	0.32868	1.10913	0.63287	-0.81168
aPFI0965w_0	PFI0965w	-0.15994	0.32868	1.10913	0.63287	-0.81168
intPFI0970c_2	PFI0970c	0.287903	-0.21253	-1.33046	-0.67488	1.189193
aPFI0970c_1	PFI0970c	0.287903	-0.21253	-1.33046	-0.67488	1.189193
aPFI0970c_0	PFI0970c	0.287903	-0.21253	-1.33046	-0.67488	1.189193
intPFI0975c_2	PFI0975c	-0.97632	-1.09964	-1.84912	-0.42666	2.342423
aPFI0975c_5	PFI0975c	-0.97632	-1.09964	-1.84912	-0.42666	2.342423
aPFI0975c_4	PFI0975c	-0.97632	-1.09964	-1.84912	-0.42666	2.342423
aPFI0975c_3	PFI0975c	-0.97632	-1.09964	-1.84912	-0.42666	2.342423
aPFI0975c_2	PFI0975c	-0.97632	-1.09964	-1.84912	-0.42666	2.342423
aPFI0975c_1	PFI0975c	-0.97632	-1.09964	-1.84912	-0.42666	2.342423
aPFI0975c_0	PFI0975c	-0.97632	-1.09964	-1.84912	-0.42666	2.342423
intPFI0980w_2	PFI0980w	-0.49514	0.03019	0.17186	0.13214	0.06831
aPFI0980w_1	PFI0980w	-0.49514	0.03019	0.17186	0.13214	0.06831
aPFI0980w_0	PFI0980w	-0.49514	0.03019	0.17186	0.13214	0.06831
intPFI0985c_2	PFI0985c	0.916233	0.078853	0.002803	-0.07814	-0.37519
aPFI0985c_0	PFI0985c	0.916233	0.078853	0.002803	-0.07814	-0.37519
intPFI0995w_2	PFI0990c	-0.96419	-0.73703	-0.13694	0.65794	1.06803
aPFI0990c_1	PFI0990c	-0.90739	-0.99205	-0.47439	0.521727	1.293387
aPFI0990c_0	PFI0990c	-0.90739	-0.99205	-0.47439	0.521727	1.293387
intPFI0990c_2	PFI0995w	-0.90739	-0.99205	-0.47439	0.521727	1.293387
aPFI0995w_0	PFI0995w	-0.96419	-0.73703	-0.13694	0.65794	1.06803
intPFI1000w_2	PFI1000w	0.056528	0.360028	0.111108	0.019888	0.047308
aPFI1000w_2	PFI1000w	0.056528	0.360028	0.111108	0.019888	0.047308
aPFI1000w_1	PFI1000w	0.056528	0.360028	0.111108	0.019888	0.047308
aPFI1000w_0	PFI1000w	0.056528	0.360028	0.111108	0.019888	0.047308

intPFI1010w_2	PFI1005w	-0.04752	-1.0224	-0.89045	-0.07055	0.8609
intPFI1005w_2	PFI1005w	-0.12367	-0.45667	-1.05417	-0.79583	0.47567
aPFI1005w_0	PFI1005w	-0.12367	-0.45667	-1.05417	-0.79583	0.47567
aPFI1010w_1	PFI1010w	-0.04752	-1.0224	-0.89045	-0.07055	0.8609
aPFI1010w_0	PFI1010w	-0.04752	-1.0224	-0.89045	-0.07055	0.8609
intPFI1015w_3	PFI1015w	0.28761	-0.45305	-1.00941	-0.87509	0.14005
aPFI1015w_1	PFI1015w	0.28761	-0.45305	-1.00941	-0.87509	0.14005
aPFI1015w_0	PFI1015w	0.28761	-0.45305	-1.00941	-0.87509	0.14005
intPFI1025w_2	PFI1020c	0.65705	0.09326	0.27298	0.09902	-0.50876
intPFI1020c_2	PFI1020c	0.403303	0.941473	1.125303	-0.01064	-1.58581
aPFI1020c_1	PFI1020c	0.403303	0.941473	1.125303	-0.01064	-1.58581
aPFI1020c_0	PFI1020c	0.403303	0.941473	1.125303	-0.01064	-1.58581
aPFI1025w_1	PFI1025w	0.65705	0.09326	0.27298	0.09902	-0.50876
aPFI1025w_0	PFI1025w	0.65705	0.09326	0.27298	0.09902	-0.50876
intPFI1030c_2	PFI1030c-a	-0.26154	0.078703	0.670103	0.606563	-0.17904
aPFI1030c_0	PFI1030c-a	-0.26154	0.078703	0.670103	0.606563	-0.17904
intPFI1035w_2	PFI1035w	-0.50654	0.00668	0.71414	0.56686	-0.28568
aPFI1035w_0	PFI1035w	-0.50654	0.00668	0.71414	0.56686	-0.28568
aPFI1040c_3	PFI1040c	0.65388	0.78512	0.85551	-0.10576	-1.42162
aPFI1040c_2	PFI1040c	0.65388	0.78512	0.85551	-0.10576	-1.42162
aPFI1040c_1	PFI1040c	0.65388	0.78512	0.85551	-0.10576	-1.42162
aPFI1040c_0	PFI1040c	0.65388	0.78512	0.85551	-0.10576	-1.42162
intPFI1045w_3	PFI1045w	0.465043	0.525283	0.633683	0.143983	-0.75062
intPFI1040c_2	PFI1045w	0.65388	0.78512	0.85551	-0.10576	-1.42162
aPFI1045w_1	PFI1045w	0.465043	0.525283	0.633683	0.143983	-0.75062
aPFI1045w_0	PFI1045w	0.465043	0.525283	0.633683	0.143983	-0.75062
aPFI1050c_0	PFI1050c	-1.00322	-0.77691	0.430313	1.111353	0.694573
intPFI1055w_2	PFI1055w	-1.07677	-0.80964	1.048787	1.425047	0.033977
intPFI1050c_2	PFI1055w	-1.00322	-0.77691	0.430313	1.111353	0.694573
aPFI1055w_1	PFI1055w	-1.07677	-0.80964	1.048787	1.425047	0.033977
aPFI1055w_0	PFI1055w	-1.07677	-0.80964	1.048787	1.425047	0.033977
intPFI1060w_2	PFI1060w	0.541357	0.444217	0.213907	-0.15501	-0.50899
aPFI1060w_2	PFI1060w	0.541357	0.444217	0.213907	-0.15501	-0.50899
aPFI1060w_1	PFI1060w	0.541357	0.444217	0.213907	-0.15501	-0.50899
aPFI1060w_0	PFI1060w	0.541357	0.444217	0.213907	-0.15501	-0.50899
aPFI1065c_1	PFI1065c	-0.61757	-0.58314	-0.33821	0.345547	0.955477
intPFI1065c_2	PFI1070c	-0.61757	-0.58314	-0.33821	0.345547	0.955477
aPFI1070c_1	PFI1070c	0.80342	0.44439	0.90648	0.11902	-1.45489
aPFI1070c_0	PFI1070c	0.80342	0.44439	0.90648	0.11902	-1.45489
intPFI1080w_2	PFI1075w	-0.74183	-0.38583	-0.00233	0.48233	0.71783
intPFI1075w_2	PFI1075w	0.458563	0.268183	0.445633	0.139033	-0.58252
intPFI1070c_2	PFI1075w	0.80342	0.44439	0.90648	0.11902	-1.45489
aPFI1075w_0	PFI1075w	0.458563	0.268183	0.445633	0.139033	-0.58252
aPFI1080w_1	PFI1080w	-0.74183	-0.38583	-0.00233	0.48233	0.71783
aPFI1080w_0	PFI1080w	-0.74183	-0.38583	-0.00233	0.48233	0.71783
intPFI1085w_2	PFI1085w	0.598048	0.688748	0.001338	-0.48356	-0.46797
aPFI1085w_2	PFI1085w	0.598048	0.688748	0.001338	-0.48356	-0.46797

aPFI1085w_1	PFI1085w	0.598048	0.688748	0.001338	-0.48356	-0.46797
aPFI1085w_0	PFI1085w	0.598048	0.688748	0.001338	-0.48356	-0.46797
intPFI1090w_1	PFI1090w	-0.44436	-0.74323	-0.65055	0.365713	1.393893
aPFI1090w_1	PFI1090w	-0.44436	-0.74323	-0.65055	0.365713	1.393893
aPFI1090w_0	PFI1090w	-0.44436	-0.74323	-0.65055	0.365713	1.393893
intPFI1100w_2	PFI1095w	-0.20741	-0.41545	0.38789	0.48261	-0.19505
intPFI1095w_2	PFI1095w	0.236627	-0.00812	-0.04444	-0.25862	-0.39074
aPFI1095w_0	PFI1095w	0.236627	-0.00812	-0.04444	-0.25862	-0.39074
aPFI1100w_1	PFI1100w	-0.20741	-0.41545	0.38789	0.48261	-0.19505
aPFI1100w_0	PFI1100w	-0.20741	-0.41545	0.38789	0.48261	-0.19505
intPFI1105w_2	PFI1105w	-0.43782	0.259597	0.802057	0.724777	-0.06426
aPFI1105w_1	PFI1105w	-0.43782	0.259597	0.802057	0.724777	-0.06426
aPFI1105w_0	PFI1105w	-0.43782	0.259597	0.802057	0.724777	-0.06426
intPFI1110w_2	PFI1110w	1.0473	0.9195	0.23225	-0.46975	-0.8665
aPFI1110w_1	PFI1110w	1.0473	0.9195	0.23225	-0.46975	-0.8665
aPFI1110w_0	PFI1110w	1.0473	0.9195	0.23225	-0.46975	-0.8665
intPFI1115c_2	PFI1115c	0.16179	0.04303	0.35544	0.25456	-0.29503
aPFI1115c_0	PFI1115c	0.16179	0.04303	0.35544	0.25456	-0.29503
intPFI1120c_2	PFI1120c	0.49208	0.33546	-0.06535	-0.19846	-0.11827
aPFI1120c_6	PFI1120c	0.49208	0.33546	-0.06535	-0.19846	-0.11827
aPFI1120c_5	PFI1120c	0.49208	0.33546	-0.06535	-0.19846	-0.11827
aPFI1120c_4	PFI1120c	0.49208	0.33546	-0.06535	-0.19846	-0.11827
aPFI1120c_3	PFI1120c	0.49208	0.33546	-0.06535	-0.19846	-0.11827
aPFI1120c_2	PFI1120c	0.49208	0.33546	-0.06535	-0.19846	-0.11827
aPFI1120c_1	PFI1120c	0.49208	0.33546	-0.06535	-0.19846	-0.11827
aPFI1120c_0	PFI1120c	0.49208	0.33546	-0.06535	-0.19846	-0.11827
intPFI1125c_2	PFI1125c	-0.13771	0.238533	0.637433	0.347233	-0.44287
aPFI1125c_0	PFI1125c	-0.13771	0.238533	0.637433	0.347233	-0.44287
intPFI1130c_2	PFI1130c	0.859563	0.669383	0.361873	-0.32515	-0.99141
aPFI1130c_0	PFI1130c	0.859563	0.669383	0.361873	-0.32515	-0.99141
intPFI1140w_2	PFI1135c	0.8141	0.71498	0.96878	0.06722	-1.46998
intPFI1135c_2	PFI1135c	0.360133	0.393343	0.949313	0.230353	-1.25268
aPFI1135c_0	PFI1135c	0.360133	0.393343	0.949313	0.230353	-1.25268
intPFI1145w_2	PFI1140w	0.909393	0.940523	0.249453	-0.47729	-0.84936
aPFI1140w_1	PFI1140w	0.8141	0.71498	0.96878	0.06722	-1.46998
aPFI1140w_0	PFI1140w	0.8141	0.71498	0.96878	0.06722	-1.46998
aPFI1145w_1	PFI1145w	0.909393	0.940523	0.249453	-0.47729	-0.84936
aPFI1145w_0	PFI1145w	0.909393	0.940523	0.249453	-0.47729	-0.84936
intPFI1150w_2	PFI1150w	-0.78156	-0.03624	0.648213	0.660953	0.039403
aPFI1150w_1	PFI1150w	-0.78156	-0.03624	0.648213	0.660953	0.039403
aPFI1150w_0	PFI1150w	-0.78156	-0.03624	0.648213	0.660953	0.039403
intPFI1160w_2	PFI1155w	-0.00052	-0.29041	-0.05093	0.209093	0.213573
intPFI1155w_2	PFI1155w	-0.65588	-1.1626	-0.68404	0.441877	1.360437
aPFI1155w_1	PFI1155w	-0.65588	-1.1626	-0.68404	0.441877	1.360437
aPFI1155w_0	PFI1155w	-0.65588	-1.1626	-0.68404	0.441877	1.360437
aPFI1160w_1	PFI1160w	-0.00052	-0.29041	-0.05093	0.209093	0.213573
aPFI1160w_0	PFI1160w	-0.00052	-0.29041	-0.05093	0.209093	0.213573

aPFI1165c_0	PFI1165c					
intPFI1170c_2	PFI1170c	-0.25503	0.203653	-0.0333	-0.00454	0.275013
intPFI1165c_2	PFI1170c					
aPFI1170c_1	PFI1170c	-0.25503	0.203653	-0.0333	-0.00454	0.275013
aPFI1170c_0	PFI1170c	-0.25503	0.203653	-0.0333	-0.00454	0.275013
intPFI1175c_2	PFI1175c	0.20362	-0.1181	0.17619	0.19181	-0.1819
aPFI1175c_0	PFI1175c	0.20362	-0.1181	0.17619	0.19181	-0.1819
intPFI1180w_2	PFI1180w	0.461563	0.131173	-0.62585	-0.86899	-0.22251
aPFI1180w_1	PFI1180w	0.461563	0.131173	-0.62585	-0.86899	-0.22251
aPFI1180w_0	PFI1180w	0.461563	0.131173	-0.62585	-0.86899	-0.22251
intPFI1190w_2	PFI1185c	-0.0668	0.270463	0.443063	0.191603	-0.3223
aPFI1185c_1	PFI1185c	-0.28832	0.26012	0.31902	0.13548	-0.11212
aPFI1185c_0	PFI1185c	-0.28832	0.26012	0.31902	0.13548	-0.11212
intPFI1185c_2	PFI1190w	-0.28832	0.26012	0.31902	0.13548	-0.11212
aPFI1190w_1	PFI1190w	-0.0668	0.270463	0.443063	0.191603	-0.3223
aPFI1190w_0	PFI1190w	-0.0668	0.270463	0.443063	0.191603	-0.3223
intPFI1195c_2	PFI1195c	-0.32799	-1.04853	-0.34769	0.37819	0.61503
aPFI1195c_1	PFI1195c	-0.32799	-1.04853	-0.34769	0.37819	0.61503
aPFI1195c_0	PFI1195c	-0.32799	-1.04853	-0.34769	0.37819	0.61503
intPFI1200w_2	PFI1200w	-0.12851	-0.10844	-0.46362	-0.49338	0.08044
aPFI1200w_1	PFI1200w	-0.12851	-0.10844	-0.46362	-0.49338	0.08044
aPFI1200w_0	PFI1200w	-0.12851	-0.10844	-0.46362	-0.49338	0.08044
intPFI1205c_2	PFI1205c	-0.56724	-0.02181	-1.11238	-0.58096	1.342473
aPFI1205c_1	PFI1205c	-0.56724	-0.02181	-1.11238	-0.58096	1.342473
aPFI1205c_0	PFI1205c	-0.56724	-0.02181	-1.11238	-0.58096	1.342473
intPFI1210w_2	PFI1210w	-0.45846	-0.46971	-1.60697	-0.70076	1.734653
aPFI1210w_3	PFI1210w	-0.45846	-0.46971	-1.60697	-0.70076	1.734653
aPFI1210w_2	PFI1210w	-0.45846	-0.46971	-1.60697	-0.70076	1.734653
aPFI1210w_1	PFI1210w	-0.45846	-0.46971	-1.60697	-0.70076	1.734653
aPFI1210w_0	PFI1210w	-0.45846	-0.46971	-1.60697	-0.70076	1.734653
intPFI1215w_2	PFI1215w	0.25104	0.1268	0.2054	0.1146	-0.2038
aPFI1215w_1	PFI1215w	0.25104	0.1268	0.2054	0.1146	-0.2038
aPFI1215w_0	PFI1215w	0.25104	0.1268	0.2054	0.1146	-0.2038
intPFI1220w_2	PFI1216w	1.75137	0.70889	-0.31163	-0.92185	-0.92265
intPFI1225w_2	PFI1220w	0.092767	0.330167	0.289657	0.064677	-0.24483
aPFI1220w_0	PFI1220w	1.75137	0.70889	-0.31163	-0.92185	-0.92265
aPFI1225w_0	PFI1225w	0.092767	0.330167	0.289657	0.064677	-0.24483
intPFI1230c_2	PFI1230c	1.28989	1.08757	0.30296	-0.72576	-1.34897
aPFI1230c_0	PFI1230c	1.28989	1.08757	0.30296	-0.72576	-1.34897
intPFI1235w_2	PFI1235w	1.304323	1.176903	0.368193	-0.75603	-1.45924
aPFI1235w_1	PFI1235w	1.304323	1.176903	0.368193	-0.75603	-1.45924
aPFI1235w_0	PFI1235w	1.304323	1.176903	0.368193	-0.75603	-1.45924
intPFI1240c_2	PFI1240c	-0.67366	-1.26621	-0.47184	0.68384	1.27121
aPFI1240c_1	PFI1240c	-0.67366	-1.26621	-0.47184	0.68384	1.27121
aPFI1240c_0	PFI1240c	-0.67366	-1.26621	-0.47184	0.68384	1.27121
intPFI1245c_2	PFI1245c	-0.6868	-0.56403	0.535043	0.721123	-0.04631
aPFI1245c_1	PFI1245c	-0.6868	-0.56403	0.535043	0.721123	-0.04631

aPFI1245c_0	PFI1245c	-0.6868	-0.56403	0.535043	0.721123	-0.04631
intPFI1250w_2	PFI1250w	-0.80668	-0.74356	0.616137	1.028197	0.185897
aPFI1250w_0	PFI1250w	-0.80668	-0.74356	0.616137	1.028197	0.185897
intPFI1255w_2	PFI1255w	-0.58492	-0.7664	-0.68521	0.143873	1.156063
aPFI1255w_0	PFI1255w	-0.58492	-0.7664	-0.68521	0.143873	1.156063
intPFI1260c_2	PFI1260c	-0.65414	0.208683	0.576133	0.446033	-0.03002
aPFI1260c_1	PFI1260c	-0.65414	0.208683	0.576133	0.446033	-0.03002
aPFI1260c_0	PFI1260c	-0.65414	0.208683	0.576133	0.446033	-0.03002
intPFI1265w_2	PFI1265w	-1.01709	-0.43686	0.340897	0.711217	0.489637
aPFI1265w_2	PFI1265w	-1.01709	-0.43686	0.340897	0.711217	0.489637
aPFI1265w_1	PFI1265w	-1.01709	-0.43686	0.340897	0.711217	0.489637
aPFI1265w_0	PFI1265w	-1.01709	-0.43686	0.340897	0.711217	0.489637
intPFI1270w_2	PFI1270w	1.04379	-0.12395	0.3804	-0.1294	-1.32205
aPFI1270w_0	PFI1270w	1.04379	-0.12395	0.3804	-0.1294	-1.32205
intPFI1275w_2	PFI1275w	0.677153	0.370743	0.053253	-0.12359	-0.26158
aPFI1275w_0	PFI1275w	0.677153	0.370743	0.053253	-0.12359	-0.26158
intPFI1280c_2	PFI1280c	0.529823	0.564613	-0.23599	-0.5061	-0.1267
aPFI1280c_4	PFI1280c	0.529823	0.564613	-0.23599	-0.5061	-0.1267
aPFI1280c_3	PFI1280c	0.529823	0.564613	-0.23599	-0.5061	-0.1267
aPFI1280c_2	PFI1280c	0.529823	0.564613	-0.23599	-0.5061	-0.1267
aPFI1280c_1	PFI1280c	0.529823	0.564613	-0.23599	-0.5061	-0.1267
aPFI1280c_0	PFI1280c	0.529823	0.564613	-0.23599	-0.5061	-0.1267
intPFI1285w_2	PFI1285w	0.80318	-0.06742	-1.05715	-1.22435	-0.21108
aPFI1290w_0	PFI1285w	0.277495	-0.62458	-0.6588	-0.25047	0.323205
aPFI1285w_1	PFI1285w	0.80318	-0.06742	-1.05715	-1.22435	-0.21108
aPFI1285w_0	PFI1285w	0.80318	-0.06742	-1.05715	-1.22435	-0.21108
intPFI1295c_2	PFI1295c	0.15182	-0.07159	-0.59983	-0.14917	0.77759
aPFI1295c_1	PFI1295c	0.15182	-0.07159	-0.59983	-0.14917	0.77759
aPFI1295c_0	PFI1295c	0.15182	-0.07159	-0.59983	-0.14917	0.77759
intPFI1300c_2	PFI1300c	-0.12023	-0.45815	-1.36119	-0.55103	1.403598
aPFI1300c_2	PFI1300c	-0.12023	-0.45815	-1.36119	-0.55103	1.403598
aPFI1300c_1	PFI1300c	-0.12023	-0.45815	-1.36119	-0.55103	1.403598
aPFI1300c_0	PFI1300c	-0.12023	-0.45815	-1.36119	-0.55103	1.403598
intPFI1305w_2	PFI1305w	0.445063	0.167683	-0.34487	-0.47747	-0.12052
aPFI1305w_1	PFI1305w	0.445063	0.167683	-0.34487	-0.47747	-0.12052
aPFI1305w_0	PFI1305w	0.445063	0.167683	-0.34487	-0.47747	-0.12052
intPFI1310w_2	PFI1310w	-0.15349	0.552987	0.608767	0.267067	-0.29565
aPFI1310w_1	PFI1310w	-0.15349	0.552987	0.608767	0.267067	-0.29565
aPFI1310w_0	PFI1310w	-0.15349	0.552987	0.608767	0.267067	-0.29565
aPFI1315c_1	PFI1315c	-0.06396	-0.15122	-0.54282	-0.13452	0.713883
aPFI1315c_0	PFI1315c	-0.06396	-0.15122	-0.54282	-0.13452	0.713883
intPFI1325w_2	PFI1320c	-0.5514	-0.22102	-0.48197	-0.00603	0.91102
intPFI1315c_2	PFI1320c	-0.06396	-0.15122	-0.54282	-0.13452	0.713883
aPFI1320c_0	PFI1320c	0.568917	0.306417	-0.33383	-0.41583	0.002917
intPFI1320c_2	PFI1325w	0.568917	0.306417	-0.33383	-0.41583	0.002917
aPFI1325w_0	PFI1325w	-0.5514	-0.22102	-0.48197	-0.00603	0.91102
intPFI1330c_2	PFI1330c	-0.62138	-0.85612	-0.04827	0.701933	0.777783

aPFI1330c_0	PFI1330c	-0.62138	-0.85612	-0.04827	0.701933	0.777783
intPFI1335w_2	PFI1335w	0.586528	0.149688	-0.88162	-0.93544	0.117428
aPFI1335w_2	PFI1335w	0.586528	0.149688	-0.88162	-0.93544	0.117428
aPFI1335w_1	PFI1335w	0.586528	0.149688	-0.88162	-0.93544	0.117428
aPFI1335w_0	PFI1335w	0.586528	0.149688	-0.88162	-0.93544	0.117428
intPFI1340w_2	PFI1340w	-0.40338	-0.79512	-1.21252	-0.23248	1.47862
aPFI1340w_1	PFI1340w	-0.40338	-0.79512	-1.21252	-0.23248	1.47862
aPFI1340w_0	PFI1340w	-0.40338	-0.79512	-1.21252	-0.23248	1.47862
aPFI1345c_1	PFI1345c	0.147103	-0.41352	-0.99597	-0.68737	0.470183
aPFI1345c_0	PFI1345c	0.147103	-0.41352	-0.99597	-0.68737	0.470183
intPFI1350c_2	PFI1350c	0.157423	0.232983	-0.55988	-0.75732	-0.00668
intPFI1345c_2	PFI1350c	0.147103	-0.41352	-0.99597	-0.68737	0.470183
aPFI1350c_0	PFI1350c	0.157423	0.232983	-0.55988	-0.75732	-0.00668
intPFI1355w_2	PFI1355w	-1.39522	-1.8809	-0.50695	0.858787	1.428737
aPFI1355w_1	PFI1355w	-1.39522	-1.8809	-0.50695	0.858787	1.428737
aPFI1355w_0	PFI1355w	-1.39522	-1.8809	-0.50695	0.858787	1.428737
intPFI1365w_2	PFI1360c	-0.38627	-0.51363	-0.36873	0.163067	0.706967
aPFI1360c_0	PFI1360c	-0.53452	-0.99141	-0.55582	0.35052	1.06201
intPFI1360c_2	PFI1365w	-0.53452	-0.99141	-0.55582	0.35052	1.06201
aPFI1365w_0	PFI1365w	-0.38627	-0.51363	-0.36873	0.163067	0.706967
intPFI1370c_2	PFI1370c	-0.87099	-0.16827	-0.13487	0.011653	0.442873
aPFI1370c_0	PFI1370c	-0.87099	-0.16827	-0.13487	0.011653	0.442873
intPFI1375w_3	PFI1375w-a	-0.9296	-0.78396	-0.48707	0.267407	1.081297
aPFI1375w_0	PFI1375w-a	-0.9296	-0.78396	-0.48707	0.267407	1.081297
intPFI1380c_2	PFI1380c	0.054463	-0.08881	-0.21738	-0.07146	0.213973
aPFI1380c_1	PFI1380c	0.054463	-0.08881	-0.21738	-0.07146	0.213973
aPFI1380c_0	PFI1380c	0.054463	-0.08881	-0.21738	-0.07146	0.213973
intPFI1390w_2	PFI1385c	0.596243	0.588793	0.424923	0.096743	-0.44013
aPFI1385c_1	PFI1385c	0.431833	0.549333	0.409333	-0.00967	-0.54267
aPFI1385c_0	PFI1385c	0.431833	0.549333	0.409333	-0.00967	-0.54267
intPFI1395w_2	PFI1390w	0.11965	-0.20725	-0.14349	0.09349	0.22525
intPFI1385c_2	PFI1390w	0.431833	0.549333	0.409333	-0.00967	-0.54267
aPFI1390w_0	PFI1390w	0.596243	0.588793	0.424923	0.096743	-0.44013
aPFI1395w_0	PFI1395w	0.11965	-0.20725	-0.14349	0.09349	0.22525
intPFI1400c_2	PFI1400c	2.129273	0.179033	-0.23938	-0.3685	-0.70429
aPFI1400c_0	PFI1400c	2.129273	0.179033	-0.23938	-0.3685	-0.70429
intPFI1405c_2	PFI1405c	-0.36379	-0.74531	1.540012	1.510592	-0.93309
aPFI1405c_0	PFI1405c	-0.36379	-0.74531	1.540012	1.510592	-0.93309
intPFI1415w_2	PFI1410c	-1.36331	-0.97991	0.367327	0.935007	0.530247
aPFI1410c_2	PFI1410c	-1.23071	-0.66278	0.699892	1.046882	0.255562
aPFI1410c_1	PFI1410c	-1.23071	-0.66278	0.699892	1.046882	0.255562
aPFI1410c_0	PFI1410c	-1.23071	-0.66278	0.699892	1.046882	0.255562
intPFI1410c_2	PFI1415w	-1.23071	-0.66278	0.699892	1.046882	0.255562
aPFI1415w_1	PFI1415w	-1.36331	-0.97991	0.367327	0.935007	0.530247
aPFI1415w_0	PFI1415w	-1.36331	-0.97991	0.367327	0.935007	0.530247
intPFI1425w_2	PFI1420w	0.461027	0.287877	0.277737	-0.0084	-0.48354
intPFI1420w_2	PFI1420w	0.28736	0.30419	1.24311	0.54189	-1.35919

aPFI1420w_0	PFI1420w	0.28736	0.30419	1.24311	0.54189	-1.35919
aPFI1425w_1	PFI1425w	0.461027	0.287877	0.277737	-0.0084	-0.48354
aPFI1425w_0	PFI1425w	0.461027	0.287877	0.277737	-0.0084	-0.48354
intPFI1430w_2	PFI1430w	-0.16581	-0.63847	-0.37658	0.172743	0.579133
aPFI1430w_1	PFI1430w	-0.16581	-0.63847	-0.37658	0.172743	0.579133
aPFI1430w_0	PFI1430w	-0.16581	-0.63847	-0.37658	0.172743	0.579133
intPFI1435w_2	PFI1435w	0.187983	0.181063	0.351883	0.023783	-0.5514
aPFI1435w_0	PFI1435w	0.187983	0.181063	0.351883	0.023783	-0.5514
intPFI1440w_2	PFI1440w	-0.52155	-0.29975	0.122757	0.381077	0.320587
aPFI1440w_1	PFI1440w	-0.52155	-0.29975	0.122757	0.381077	0.320587
aPFI1440w_0	PFI1440w	-0.52155	-0.29975	0.122757	0.381077	0.320587
intPFI1445w_2	PFI1445w	-0.87096	-1.61487	-2.39425	-0.53919	2.794428
aPFI1445w_2	PFI1445w	-0.87096	-1.61487	-2.39425	-0.53919	2.794428
aPFI1445w_1	PFI1445w	-0.87096	-1.61487	-2.39425	-0.53919	2.794428
aPFI1445w_0	PFI1445w	-0.87096	-1.61487	-2.39425	-0.53919	2.794428
aPFI1450c_0	PFI1450c	-0.51206	-0.42384	-0.15876	0.168805	0.432385
intPFI1455c_2	PFI1455c	0.33629	-0.36845	-1.5161	-1.0129	0.90245
intPFI1450c_2	PFI1455c	-0.51206	-0.42384	-0.15876	0.168805	0.432385
aPFI1455c_0	PFI1455c	0.33629	-0.36845	-1.5161	-1.0129	0.90245
intPFI1463w_2	PFI1460c					
aPFI1460c_0	PFI1460c	-0.90391	-0.98246	-0.07459	0.76759	0.93446
intPFI1465w_2	PFI1463w	0.17634	-0.18366	-0.22484	-0.23999	-0.129
intPFI1460c_2	PFI1463w	-0.90391	-0.98246	-0.07459	0.76759	0.93446
aPFI1465w_1	PFI1465w	0.17634	-0.18366	-0.22484	-0.23999	-0.129
aPFI1465w_0	PFI1465w	0.17634	-0.18366	-0.22484	-0.23999	-0.129
aPFI1470c_2	PFI1468c	0.236453	-0.26061	-0.88012	-0.77233	0.202493
aPFI1470c_1	PFI1468c	0.236453	-0.26061	-0.88012	-0.77233	0.202493
intPFI1470c_2	PFI1470c	0.236453	-0.26061	-0.88012	-0.77233	0.202493
aPFI1470c_0	PFI1470c	0.236453	-0.26061	-0.88012	-0.77233	0.202493
intPFI1475w_2	PFI1475w	-0.37209	-1.57076	-3.17041	-1.34349	2.874035
aPFI1475w_2	PFI1475w	-0.37209	-1.57076	-3.17041	-1.34349	2.874035
aPFI1475w_1	PFI1475w	-0.37209	-1.57076	-3.17041	-1.34349	2.874035
aPFI1475w_0	PFI1475w	-0.37209	-1.57076	-3.17041	-1.34349	2.874035
intPFI1480w_3	PFI1480w	0.58225	0.40373	-0.01971	-0.50529	-0.64673
aPFI1480w_1	PFI1480w	0.58225	0.40373	-0.01971	-0.50529	-0.64673
aPFI1480w_0	PFI1480w	0.58225	0.40373	-0.01971	-0.50529	-0.64673
intPFI1485c_2	PFI1485c	0.18923	0.07437	-0.65974	-0.78426	0.01963
aPFI1485c_1	PFI1485c	0.18923	0.07437	-0.65974	-0.78426	0.01963
aPFI1485c_0	PFI1485c	0.18923	0.07437	-0.65974	-0.78426	0.01963
intPFI1495w_2	PFI1490c-a	0.56165	1.14975	0.80401	-0.11201	-1.06275
aPFI1490c_1	PFI1490c-a	0.043703	0.596963	0.127563	-0.1529	-0.0798
aPFI1490c_0	PFI1490c-a	0.043703	0.596963	0.127563	-0.1529	-0.0798
intPFI1490c_2	PFI1495w	0.043703	0.596963	0.127563	-0.1529	-0.0798
aPFI1495w_1	PFI1495w	0.56165	1.14975	0.80401	-0.11201	-1.06275
aPFI1495w_0	PFI1495w	0.56165	1.14975	0.80401	-0.11201	-1.06275
intPFI1500w_2	PFI1500w	-0.91624	-0.38322	-0.06541	0.348748	0.692978
aPFI1500w_6	PFI1500w	-0.91624	-0.38322	-0.06541	0.348748	0.692978

aPFI1500w_5	PFI1500w	-0.91624	-0.38322	-0.06541	0.348748	0.692978
aPFI1500w_4	PFI1500w	-0.91624	-0.38322	-0.06541	0.348748	0.692978
aPFI1500w_3	PFI1500w	-0.91624	-0.38322	-0.06541	0.348748	0.692978
aPFI1500w_2	PFI1500w	-0.91624	-0.38322	-0.06541	0.348748	0.692978
aPFI1500w_1	PFI1500w	-0.91624	-0.38322	-0.06541	0.348748	0.692978
aPFI1500w_0	PFI1500w	-0.91624	-0.38322	-0.06541	0.348748	0.692978
intPFI1835c_2	PFI1505c	-0.4117	-0.8725	0.467017	0.822317	-0.06716
intPFI1505c_2	PFI1505c	0.220413	0.011413	-0.27709	-0.16725	0.191253
aPFI1505c_1	PFI1505c	0.220413	0.011413	-0.27709	-0.16725	0.191253
aPFI1505c_0	PFI1505c	0.220413	0.011413	-0.27709	-0.16725	0.191253
intPFI1510w_2	PFI1510w	0.911563	0.324173	0.629403	-0.20024	-1.54201
aPFI1510w_1	PFI1510w	0.911563	0.324173	0.629403	-0.20024	-1.54201
aPFI1510w_0	PFI1510w	0.911563	0.324173	0.629403	-0.20024	-1.54201
intPFI1515w_2	PFI1515w	0.12323	0.45885	0.0338	-0.0878	0.07115
aPFI1515w_0	PFI1515w	0.12323	0.45885	0.0338	-0.0878	0.07115
intPFI1520w_2	PFI1520w	0.077393	0.842033	0.633433	-0.08977	-0.76037
aPFI1520w_0	PFI1520w	0.077393	0.842033	0.633433	-0.08977	-0.76037
intPFI1525w_2	PFI1525w	-0.22699	0.05396	-0.12792	0.02992	0.39104
aPFI1525w_0	PFI1525w	-0.22699	0.05396	-0.12792	0.02992	0.39104
intPFI1530c_2	PFI1530c	-0.15	-0.283	-0.56	-0.204	0.582
aPFI1530c_0	PFI1530c	-0.15	-0.283	-0.56	-0.204	0.582
intPFI1535w_2	PFI1535w	0.228798	-0.12716	-0.47225	-0.45198	0.026778
aPFI1535w_0	PFI1535w	0.228798	-0.12716	-0.47225	-0.45198	0.026778
intPFI1540w_2	PFI1540w	-0.25084	0.05021	-0.22641	-0.28959	0.09779
aPFI1540w_1	PFI1540w	-0.25084	0.05021	-0.22641	-0.28959	0.09779
aPFI1540w_0	PFI1540w	-0.25084	0.05021	-0.22641	-0.28959	0.09779
intPFI1545c_2	PFI1545c	-1.0887	-0.9115	0.136017	0.979317	0.974837
aPFI1545c_0	PFI1545c	-1.0887	-0.9115	0.136017	0.979317	0.974837
intPFI1555w_2	PFI1550c	0.808853	-0.37429	-1.38843	-1.25891	0.137953
intPFI1550c_2	PFI1550c	0.69523	-0.18815	-1.0672	-1.1528	-0.12985
aPFI1550c_0	PFI1550c	0.69523	-0.18815	-1.0672	-1.1528	-0.12985
aPFI1555w_0	PFI1555w	0.808853	-0.37429	-1.38843	-1.25891	0.137953
intPFI1560c_3	PFI1560c	0.092833	-0.58169	-1.78863	-1.02671	1.320353
aPFI1560c_1	PFI1560c	0.092833	-0.58169	-1.78863	-1.02671	1.320353
aPFI1560c_0	PFI1560c	0.092833	-0.58169	-1.78863	-1.02671	1.320353
intPFI1565w_2	PFI1565w	-0.70989	-2.08154	-1.50843	0.27343	2.02454
aPFI1565w_0	PFI1565w	-0.70989	-2.08154	-1.50843	0.27343	2.02454
intPFI1570c_2	PFI1570c	-0.8048	-0.05397	1.092447	0.842887	-0.50619
aPFI1570c_1	PFI1570c	-0.8048	-0.05397	1.092447	0.842887	-0.50619
aPFI1570c_0	PFI1570c	-0.8048	-0.05397	1.092447	0.842887	-0.50619
intPFI1575c_2	PFI1575c	-0.98556	-1.0982	-0.4636	0.5516	1.2737
aPFI1575c_1	PFI1575c	-0.98556	-1.0982	-0.4636	0.5516	1.2737
aPFI1575c_0	PFI1575c	-0.98556	-1.0982	-0.4636	0.5516	1.2737
aPFI1580c_1	PFI1580c	-0.62645	-0.91877	-1.23396	-0.08854	1.72577
aPFI1580c_0	PFI1580c	-0.62645	-0.91877	-1.23396	-0.08854	1.72577
intPFI1580c_2	PFI1585c	-0.62645	-0.91877	-1.23396	-0.08854	1.72577
aPFI1585c_0	PFI1585c	-0.56163	-0.57321	0.061073	0.632373	0.641653

intPFI1585c_2	PFI1587c	-0.56163	-0.57321	0.061073	0.632373	0.641653
aPFI1615c_1	PFI1587c	0.67494	0.0793	-0.1651	-0.4239	-0.5053
aPFI1615c_0	PFI1587c	0.67494	0.0793	-0.1651	-0.4239	-0.5053
intPFI1615c_2	PFI1590c	0.67494	0.0793	-0.1651	-0.4239	-0.5053
intPFI1590c_3	PFI1590c	-0.57649	-0.22209	0.133683	0.524483	0.585753
aPFI1590c_1	PFI1590c	-0.57649	-0.22209	0.133683	0.524483	0.585753
aPFI1590c_0	PFI1590c	-0.57649	-0.22209	0.133683	0.524483	0.585753
aPFI1595c_0	PFI1595c	0.932107	1.247487	0.535537	-0.7402	-1.57115
intPFI1600w_2	PFI1600w	0.43071	0.33193	-0.01886	-0.37914	-0.45643
intPFI1595c_2	PFI1600w	0.932107	1.247487	0.535537	-0.7402	-1.57115
aPFI1600w_1	PFI1600w	0.43071	0.33193	-0.01886	-0.37914	-0.45643
aPFI1600w_0	PFI1600w	0.43071	0.33193	-0.01886	-0.37914	-0.45643
intPFI1605w_2	PFI1605w	0.362083	0.136603	-0.54021	-0.47913	0.242063
aPFI1605w_1	PFI1605w	0.362083	0.136603	-0.54021	-0.47913	0.242063
aPFI1605w_0	PFI1605w	0.362083	0.136603	-0.54021	-0.47913	0.242063
intPFI1615w_2	PFI1610c	-1.11939	-0.79803	0.105817	0.742517	0.760867
intPFI1610c_2	PFI1610c	-0.74031	-1.12548	0.392943	1.114723	0.448143
aPFI1610c_0	PFI1610c	-0.74031	-1.12548	0.392943	1.114723	0.448143
aPFI1615w_0	PFI1615w	-1.11939	-0.79803	0.105817	0.742517	0.760867
aPFI1615w_1	PFI1617w	-1.11939	-0.79803	0.105817	0.742517	0.760867
intPFI1620c_2	PFI1620c	-0.71097	-0.55747	-0.42689	0.336558	1.152808
aPFI1620c_2	PFI1620c	-0.71097	-0.55747	-0.42689	0.336558	1.152808
aPFI1620c_1	PFI1620c	-0.71097	-0.55747	-0.42689	0.336558	1.152808
aPFI1620c_0	PFI1620c	-0.71097	-0.55747	-0.42689	0.336558	1.152808
intPFI1625c_2	PFI1625c	-0.54639	-0.11654	0.674337	0.729497	-0.03762
aPFI1625c_1	PFI1625c	-0.54639	-0.11654	0.674337	0.729497	-0.03762
aPFI1625c_0	PFI1625c	-0.54639	-0.11654	0.674337	0.729497	-0.03762
intPFI1635w_2	PFI1630c	0.19623	0.06587	-0.04999	0.02299	0.10463
aPFI1630c_0	PFI1630c	0.792107	-0.08953	-0.65493	-0.27073	0.470867
intPFI1630c_2	PFI1635w	0.792107	-0.08953	-0.65493	-0.27073	0.470867
aPFI1635w_1	PFI1635w	0.19623	0.06587	-0.04999	0.02299	0.10463
aPFI1635w_0	PFI1635w	0.19623	0.06587	-0.04999	0.02299	0.10463
aPFI1640c_0	PFI1640c	0.503227	0.289847	-0.0357	-0.15896	-0.14751
intPFI1650w_2	PFI1645c	0.276787	0.668047	0.268147	-0.14881	-0.36171
intPFI1645c_2	PFI1645c	-0.01664	0.25618	0.40163	0.19287	-0.27068
intPFI1640c_2	PFI1645c	0.503227	0.289847	-0.0357	-0.15896	-0.14751
aPFI1645c_1	PFI1645c	-0.01664	0.25618	0.40163	0.19287	-0.27068
aPFI1645c_0	PFI1645c	-0.01664	0.25618	0.40163	0.19287	-0.27068
aPFI1650w_1	PFI1650w	0.276787	0.668047	0.268147	-0.14881	-0.36171
aPFI1650w_0	PFI1650w	0.276787	0.668047	0.268147	-0.14881	-0.36171
intPFI1660w_2	PFI1655c	-1.26426	-1.65304	-0.11926	1.16082	1.27926
aPFI1655c_0	PFI1655c	-0.45206	-0.59966	-0.23117	0.417507	0.800997
intPFI1655c_2	PFI1660w	-0.45206	-0.59966	-0.23117	0.417507	0.800997
aPFI1660w_0	PFI1660w	-1.26426	-1.65304	-0.11926	1.16082	1.27926
intPFI1665w_2	PFI1665w	-0.33514	-1.05626	-0.15346	0.597797	0.585597
aPFI1665w_0	PFI1665w	-0.33514	-1.05626	-0.15346	0.597797	0.585597
intPFI1670c_2	PFI1670c					

aPFI1670c_0	PFI1670c					
intPFI1675w_2	PFI1675w	1.674123	0.728383	-1.21477	-2.16357	-1.09072
aPFI1675w_0	PFI1675w	1.674123	0.728383	-1.21477	-2.16357	-1.09072
intPFI1680w_2	PFI1680w	-0.5819	-0.41149	0.01347	0.30303	0.34949
aPFI1680w_1	PFI1680w	-0.5819	-0.41149	0.01347	0.30303	0.34949
aPFI1680w_0	PFI1680w	-0.5819	-0.41149	0.01347	0.30303	0.34949
intPFI1685w_2	PFI1685w	-0.54638	-1.50907	-2.03785	-0.54181	2.099407
aPFI1685w_0	PFI1685w	-0.54638	-1.50907	-2.03785	-0.54181	2.099407
aPFI1690c_1	PFI1690c	1.205627	0.811877	-0.526	-1.09816	-0.54354
aPFI1690c_0	PFI1690c	1.205627	0.811877	-0.526	-1.09816	-0.54354
intPFI1690c_2	PFI1695c	1.205627	0.811877	-0.526	-1.09816	-0.54354
aPFI1695c_0	PFI1695c	-0.09567	0.092333	0.150333	0.244333	0.184333
intPFI1695c_2	PFI1696c	-0.09567	0.092333	0.150333	0.244333	0.184333
aPFI1700c_1	PFI1700c	0.337247	0.313787	-0.02858	-0.16408	-0.09695
aPFI1700c_0	PFI1700c	0.337247	0.313787	-0.02858	-0.16408	-0.09695
intPFI1705w_3	PFI1705w	0.76496	0.30022	-0.23644	-0.28856	-0.05422
intPFI1700c_2	PFI1705w	0.337247	0.313787	-0.02858	-0.16408	-0.09695
aPFI1705w_0	PFI1705w	0.76496	0.30022	-0.23644	-0.28856	-0.05422
intPFI1710w_2	PFI1710w	0.523273	-0.04936	-0.09904	-0.0158	-0.04948
aPFI1710w_1	PFI1710w	0.523273	-0.04936	-0.09904	-0.0158	-0.04948
aPFI1710w_0	PFI1710w	0.523273	-0.04936	-0.09904	-0.0158	-0.04948
intPFI1715w_3	PFI1715w	-0.74404	1.963163	0.443183	-0.76852	-0.4085
aPFI1715w_0	PFI1715w	-0.74404	1.963163	0.443183	-0.76852	-0.4085
intPFI1720w_2	PFI1720w	0.70983	0.48536	0.41177	-0.08827	-0.80036
aPFI1720w_1	PFI1720w	0.70983	0.48536	0.41177	-0.08827	-0.80036
aPFI1720w_0	PFI1720w	0.70983	0.48536	0.41177	-0.08827	-0.80036
intPFI1725w_2	PFI1725w	1.07773	0.24737	-0.07124	-0.43376	-0.71437
aPFI1725w_0	PFI1725w	1.07773	0.24737	-0.07124	-0.43376	-0.71437
intPFI1730w_2	PFI1730w	0.477293	0.859513	0.468973	-0.38731	-1.02685
aPFI1730w_1	PFI1730w	0.477293	0.859513	0.468973	-0.38731	-1.02685
aPFI1730w_0	PFI1730w	0.477293	0.859513	0.468973	-0.38731	-1.02685
intPFI1735c_2	PFI1735c	0.484588	1.528038	0.131658	-0.92204	-0.76727
aPFI1735c_1	PFI1735c	0.484588	1.528038	0.131658	-0.92204	-0.76727
aPFI1735c_0	PFI1735c	0.484588	1.528038	0.131658	-0.92204	-0.76727
intPFI1740c_2	PFI1740c-a	0.645222	0.801102	0.273772	-0.51079	-0.94912
aPFI1740c_0	PFI1740c-a	0.645222	0.801102	0.273772	-0.51079	-0.94912
intPFI1745c_2	PFI1745c	0.256293	0.652533	0.211933	-0.32527	-0.52687
aPFI1745c_0	PFI1745c	0.256293	0.652533	0.211933	-0.32527	-0.52687
intPFI1750c_2	PFI1750c	0.09673	0.97933	0.27284	-0.41384	-0.50033
aPFI1750c_0	PFI1750c	0.09673	0.97933	0.27284	-0.41384	-0.50033
intPFI1755c_3	PFI1755c	0.27112	0.08338	-0.08127	-0.13373	-0.08638
intPFI1760w_2	PFI1760w	0.040563	0.259163	0.531673	0.127993	-0.6235
aPFI1760w_0	PFI1760w	0.040563	0.259163	0.531673	0.127993	-0.6235
intPFI1765c_2	PFI1765c	0.846363	0.344173	0.358153	0.074513	-0.59751
aPFI1765c_0	PFI1765c	0.846363	0.344173	0.358153	0.074513	-0.59751
intPFI1770w_2	PFI1770w	0.78334	0.51031	0.50487	-0.13987	-1.05631
aPFI1770w_0	PFI1770w	0.78334	0.51031	0.50487	-0.13987	-1.05631

intPFI1775w_2	PFI1775w	0.476287	0.046067	0.207357	0.072977	-0.39873
aPFI1775w_1	PFI1775w	0.476287	0.046067	0.207357	0.072977	-0.39873
aPFI1775w_0	PFI1775w	0.476287	0.046067	0.207357	0.072977	-0.39873
intPFI1780w_2	PFI1780w	0.54131	-0.07705	0.4346	0.0764	-0.91395
aPFI1780w_1	PFI1780w	0.54131	-0.07705	0.4346	0.0764	-0.91395
aPFI1780w_0	PFI1780w	0.54131	-0.07705	0.4346	0.0764	-0.91395
intPFI1785w_2	PFI1785w	1.813643	0.051763	-0.17954	-0.2988	-0.6891
aPFI1785w_0	PFI1785w	1.813643	0.051763	-0.17954	-0.2988	-0.6891
intPFI1790w_2	PFI1790w	0.10461	0.36396	0.26557	-0.01657	-0.30396
aPFI1790w_0	PFI1790w	0.10461	0.36396	0.26557	-0.01657	-0.30396
intPFI1795c_2	PFI1795c					
aPFI1795c_0	PFI1795c					
intPFI1800w_2	PFI1800w					
aPFI1800w_0	PFI1800w					
aPFI1835c_0	PFI1835c	-0.4117	-0.8725	0.467017	0.822317	-0.06716
intPFL0035c_3	PFL0035c	1.660727	0.813887	-0.12627	-1.11339	-1.44955
aPFL0035c_1	PFL0035c	1.660727	0.813887	-0.12627	-1.11339	-1.44955
aPFL0035c_0	PFL0035c	1.660727	0.813887	-0.12627	-1.11339	-1.44955
aPFL0040c_1	PFL0040c	1.58929	1.39304	0.25117	-1.17467	-1.82354
aPFL0040c_0	PFL0040c	1.58929	1.39304	0.25117	-1.17467	-1.82354
intPFL0045c_2	PFL0045c	0.75774	0.6928	0.79015	-0.17565	-1.5028
aPFL0045c_1	PFL0045c	0.75774	0.6928	0.79015	-0.17565	-1.5028
aPFL0045c_0	PFL0045c	0.75774	0.6928	0.79015	-0.17565	-1.5028
intPFL0050c_2	PFL0050c	-1.38538	-0.63306	1.755627	1.621207	-0.7471
aPFL0050c_1	PFL0050c	-1.38538	-0.63306	1.755627	1.621207	-0.7471
aPFL0050c_0	PFL0050c	-1.38538	-0.63306	1.755627	1.621207	-0.7471
intPFL0055c_2	PFL0055c	-0.59693	0.788177	2.052157	0.761677	-1.89534
aPFL0055c_1	PFL0055c	-0.59693	0.788177	2.052157	0.761677	-1.89534
aPFL0055c_0	PFL0055c	-0.59693	0.788177	2.052157	0.761677	-1.89534
intPFL0060w_2	PFL0060w	2.48391	1.59246	-0.44291	-2.42609	-2.59346
aPFL0060w_0	PFL0060w	2.48391	1.59246	-0.44291	-2.42609	-2.59346
intPFL0065w_2	PFL0065w	1.699183	1.097083	-1.07868	-1.65866	-0.34542
aPFL0065w_0	PFL0065w	1.699183	1.097083	-1.07868	-1.65866	-0.34542
intPFL0070c_2	PFL0070c	0.441863	-0.1943	-0.05341	0.088573	-0.04604
aPFL0070c_1	PFL0070c	0.441863	-0.1943	-0.05341	0.088573	-0.04604
aPFL0070c_0	PFL0070c	0.441863	-0.1943	-0.05341	0.088573	-0.04604
intPFL0075w_2	PFL0075w	0.89974	0.90529	0.64842	-0.37142	-1.44229
aPFL0075w_1	PFL0075w	0.89974	0.90529	0.64842	-0.37142	-1.44229
aPFL0075w_0	PFL0075w	0.89974	0.90529	0.64842	-0.37142	-1.44229
intPFL0080c_2	PFL0080c	-0.61745	-0.67475	-0.6562	0.097463	1.105213
aPFL0080c_0	PFL0080c	-0.61745	-0.67475	-0.6562	0.097463	1.105213
intPFL0085c_2	PFL0085c					
aPFL0085c_0	PFL0085c					
intPFL0090c_2	PFL0090c	-0.84811	-0.89349	0.074963	0.871703	0.857153
aPFL0090c_0	PFL0090c	-0.84811	-0.89349	0.074963	0.871703	0.857153
intPFL0095c_2	PFL0095c	0.120393	-0.09495	0.018893	0.121773	0.044613
aPFL0095c_0	PFL0095c	0.120393	-0.09495	0.018893	0.121773	0.044613

intPFL0105w_2	PFL0100c	-0.16112	0.206597	0.011307	0.085027	0.298237
aPFL0100c_1	PFL0100c	0.66874	1.01878	0.14469	-0.54019	-0.61928
aPFL0100c_0	PFL0100c	0.66874	1.01878	0.14469	-0.54019	-0.61928
intPFL0100c_2	PFL0105w	0.66874	1.01878	0.14469	-0.54019	-0.61928
aPFL0105w_1	PFL0105w	-0.16112	0.206597	0.011307	0.085027	0.298237
aPFL0105w_0	PFL0105w	-0.16112	0.206597	0.011307	0.085027	0.298237
intPFL0110c_2	PFL0110c	-1.13224	-1.09825	-0.40577	0.663738	1.384768
aPFL0110c_0	PFL0110c	-1.13224	-1.09825	-0.40577	0.663738	1.384768
intPFL0115w_2	PFL0115w	0.060118	0.049218	-0.53773	-0.35519	0.465818
aPFL0115w_8	PFL0115w	0.060118	0.049218	-0.53773	-0.35519	0.465818
aPFL0115w_7	PFL0115w	0.060118	0.049218	-0.53773	-0.35519	0.465818
aPFL0115w_6	PFL0115w	0.060118	0.049218	-0.53773	-0.35519	0.465818
aPFL0115w_5	PFL0115w	0.060118	0.049218	-0.53773	-0.35519	0.465818
aPFL0115w_4	PFL0115w	0.060118	0.049218	-0.53773	-0.35519	0.465818
aPFL0115w_3	PFL0115w	0.060118	0.049218	-0.53773	-0.35519	0.465818
aPFL0115w_2	PFL0115w	0.060118	0.049218	-0.53773	-0.35519	0.465818
aPFL0115w_1	PFL0115w	0.060118	0.049218	-0.53773	-0.35519	0.465818
aPFL0115w_0	PFL0115w	0.060118	0.049218	-0.53773	-0.35519	0.465818
aPFL0120c_0	PFL0120c	0.05426	0.66969	0.14312	-0.31912	-0.31269
intPFL0125c_2	PFL0125c	1.69997	1.09313	0.26249	-0.81299	-1.53863
intPFL0120c_2	PFL0125c	0.05426	0.66969	0.14312	-0.31912	-0.31269
aPFL0125c_1	PFL0125c	1.69997	1.09313	0.26249	-0.81299	-1.53863
aPFL0125c_0	PFL0125c	1.69997	1.09313	0.26249	-0.81299	-1.53863
intPFL0130c_2	PFL0130c	0.006877	-0.15836	-0.49652	-0.26514	0.404697
aPFL0130c_3	PFL0130c	0.006877	-0.15836	-0.49652	-0.26514	0.404697
aPFL0130c_2	PFL0130c	0.006877	-0.15836	-0.49652	-0.26514	0.404697
aPFL0130c_1	PFL0130c	0.006877	-0.15836	-0.49652	-0.26514	0.404697
aPFL0130c_0	PFL0130c	0.006877	-0.15836	-0.49652	-0.26514	0.404697
intPFL0140w_2	PFL0135w	0.905543	0.564283	0.298933	-0.16527	-0.72162
intPFL0135w_2	PFL0135w	0.289623	0.768883	0.477983	-0.37032	-1.01572
aPFL0135w_1	PFL0135w	0.289623	0.768883	0.477983	-0.37032	-1.01572
aPFL0135w_0	PFL0135w	0.289623	0.768883	0.477983	-0.37032	-1.01572
aPFL0140w_0	PFL0140w	0.905543	0.564283	0.298933	-0.16527	-0.72162
intPFL0145c_3	PFL0145c	-0.14401	-0.52096	-0.36808	0.192743	0.664623
aPFL0145c_0	PFL0145c	-0.14401	-0.52096	-0.36808	0.192743	0.664623
intPFL0150w_2	PFL0150w	-0.53836	-0.72171	-0.54582	0.28282	1.12321
aPFL0150w_1	PFL0150w	-0.53836	-0.72171	-0.54582	0.28282	1.12321
aPFL0150w_0	PFL0150w	-0.53836	-0.72171	-0.54582	0.28282	1.12321
intPFL0160w_2	PFL0155c	0.891933	0.909313	0.179863	-0.4372	-0.67665
aPFL0155c_0	PFL0155c	0.56203	0.88244	0.28593	-0.33973	-0.63284
intPFL0155c_2	PFL0160w	0.56203	0.88244	0.28593	-0.33973	-0.63284
aPFL0160w_0	PFL0160w	0.891933	0.909313	0.179863	-0.4372	-0.67665
aPFL0165c_1	PFL0165c	-0.42337	-0.37815	-0.14596	0.265123	0.554313
aPFL0165c_0	PFL0165c	-0.42337	-0.37815	-0.14596	0.265123	0.554313
intPFL0170w_2	PFL0170w	-1.11781	-1.53347	-0.49656	0.82856	1.50347
intPFL0165c_2	PFL0170w	-0.42337	-0.37815	-0.14596	0.265123	0.554313
aPFL0170w_1	PFL0170w	-1.11781	-1.53347	-0.49656	0.82856	1.50347

aPFL0170w_0	PFL0170w	-1.11781	-1.53347	-0.49656	0.82856	1.50347
aPFL0175c_1	PFL0175c	1.249597	0.931527	0.106707	-0.80237	-1.19469
aPFL0175c_0	PFL0175c	1.249597	0.931527	0.106707	-0.80237	-1.19469
intPFL0180w_2	PFL0180w					
intPFL0175c_2	PFL0180w	1.249597	0.931527	0.106707	-0.80237	-1.19469
aPFL0180w_0	PFL0180w					
intPFL0185c_2	PFL0185c	0.225873	-0.26338	-0.01925	0.088413	-0.08296
aPFL0185c_1	PFL0185c	0.225873	-0.26338	-0.01925	0.088413	-0.08296
aPFL0185c_0	PFL0185c	0.225873	-0.26338	-0.01925	0.088413	-0.08296
intPFL0190w_1	PFL0190w	0.565417	0.953897	0.650707	-0.24937	-1.11956
aPFL0190w_0	PFL0190w	0.565417	0.953897	0.650707	-0.24937	-1.11956
intPFL0195w_2	PFL0195w	1.314135	0.542585	-0.76753	-1.03639	-0.22377
intPFL0205w_2	PFL0200c	-0.12306	-0.30974	0.279713	0.453453	0.001903
intPFL0200c_2	PFL0200c	-0.74109	-1.06055	0.335737	1.008457	0.437167
aPFL0200c_1	PFL0200c	-0.74109	-1.06055	0.335737	1.008457	0.437167
aPFL0200c_0	PFL0200c	-0.74109	-1.06055	0.335737	1.008457	0.437167
aPFL0205w_1	PFL0205w	-0.12306	-0.30974	0.279713	0.453453	0.001903
aPFL0205w_0	PFL0205w	-0.12306	-0.30974	0.279713	0.453453	0.001903
intPFL0210c_2	PFL0210c	-0.72625	-0.05579	0.824083	0.931583	0.091453
aPFL0210c_0	PFL0210c	-0.72625	-0.05579	0.824083	0.931583	0.091453
aPFL0220c_0	PFL0220c	0.236497	0.012537	0.139397	-0.02494	-0.36344
aPFL0215c_0	PFL0220c	0.470823	0.542893	0.136713	-0.23105	-0.39223
intPFL0230w_2	PFL0225c	0.74143	0.98384	0.69483	-0.19183	-1.15284
intPFL0220c_2	PFL0225c	0.236497	0.012537	0.139397	-0.02494	-0.36344
aPFL0225c_0	PFL0225c	0.315813	0.298933	0.454133	0.267533	-0.33727
intPFL0235w_2	PFL0230w	-0.91872	-1.12141	0.010813	0.976853	1.004073
intPFL0225c_2	PFL0230w	0.315813	0.298933	0.454133	0.267533	-0.33727
aPFL0230w_0	PFL0230w	0.74143	0.98384	0.69483	-0.19183	-1.15284
aPFL0235w_0	PFL0235w	-0.91872	-1.12141	0.010813	0.976853	1.004073
intPFL0245w_2	PFL0245w	0.701517	0.126107	-0.91742	-1.40938	-0.58191
aPFL0245w_0	PFL0245w	0.701517	0.126107	-0.91742	-1.40938	-0.58191
aPFL0240w_0	PFL0245w	-0.5903	0.003832	-0.09999	0.090432	0.518282
intPFL0250w_2	PFL0250w	0.087473	0.053153	-1.15005	-0.85479	0.832513
aPFL0250w_1	PFL0250w	0.087473	0.053153	-1.15005	-0.85479	0.832513
aPFL0250w_0	PFL0250w	0.087473	0.053153	-1.15005	-0.85479	0.832513
intPFL0255c_2	PFL0255c	0.172823	0.536873	0.587253	0.043413	-0.71121
aPFL0255c_1	PFL0255c	0.172823	0.536873	0.587253	0.043413	-0.71121
aPFL0255c_0	PFL0255c	0.172823	0.536873	0.587253	0.043413	-0.71121
intPFL0260c_2	PFL0260c	0.16792	-0.18262	-0.64675	-0.50825	0.24562
aPFL0260c_0	PFL0260c	0.16792	-0.18262	-0.64675	-0.50825	0.24562
intPFL0265w_2	PFL0265w	0.348133	0.244343	-0.59969	-0.55865	0.308323
aPFL0265w_0	PFL0265w	0.348133	0.244343	-0.59969	-0.55865	0.308323
intPFL0270c_2	PFL0270c	0.574483	0.278563	-0.14112	-0.34722	-0.2679
aPFL0270c_1	PFL0270c	0.574483	0.278563	-0.14112	-0.34722	-0.2679
aPFL0270c_0	PFL0270c	0.574483	0.278563	-0.14112	-0.34722	-0.2679
intPFL0275w_2	PFL0275w	0.783627	0.115857	-1.08621	-1.15295	0.077977
aPFL0275w_2	PFL0275w	0.783627	0.115857	-1.08621	-1.15295	0.077977

aPFL0275w_1	PFL0275w	0.783627	0.115857	-1.08621	-1.15295	0.077977
aPFL0275w_0	PFL0275w	0.783627	0.115857	-1.08621	-1.15295	0.077977
aPFL0280c_0	PFL0280c	-0.84905	-1.29075	0.520983	1.392683	0.556413
intPFL0285w_2	PFL0285w	-0.04627	-0.55867	0.136843	0.562823	0.236333
aPFL0285w_0	PFL0285w	-0.04627	-0.55867	0.136843	0.562823	0.236333
intPFL0290w_3	PFL0290w	-0.03934	0.048192	-0.22276	-0.25266	0.074642
aPFL0290w_3	PFL0290w	-0.03934	0.048192	-0.22276	-0.25266	0.074642
aPFL0290w_2	PFL0290w	-0.03934	0.048192	-0.22276	-0.25266	0.074642
aPFL0290w_1	PFL0290w	-0.03934	0.048192	-0.22276	-0.25266	0.074642
aPFL0290w_0	PFL0290w	-0.03934	0.048192	-0.22276	-0.25266	0.074642
intPFL0295c_2	PFL0295c	0.031983	0.363103	0.152803	-0.16314	-0.29344
aPFL0295c_1	PFL0295c	0.031983	0.363103	0.152803	-0.16314	-0.29344
aPFL0295c_0	PFL0295c	0.031983	0.363103	0.152803	-0.16314	-0.29344
intPFL0300c_2	PFL0300c	0.1297	-0.33649	-1.97552	-1.41048	1.23849
aPFL0300c_0	PFL0300c	0.1297	-0.33649	-1.97552	-1.41048	1.23849
intPFL0305c_2	PFL0305c	-0.71113	-0.42735	-0.87831	-0.33269	1.06335
aPFL0305c_1	PFL0305c	-0.71113	-0.42735	-0.87831	-0.33269	1.06335
aPFL0305c_0	PFL0305c	-0.71113	-0.42735	-0.87831	-0.33269	1.06335
intPFL0310c_3	PFL0310c	-0.09769	0.32896	0.57783	0.29967	-0.35796
aPFL0310c_1	PFL0310c	-0.09769	0.32896	0.57783	0.29967	-0.35796
aPFL0310c_0	PFL0310c	-0.09769	0.32896	0.57783	0.29967	-0.35796
intPFL0320w_2	PFL0315c	0.294257	-0.74235	-1.61833	-0.85169	1.096407
intPFL0315c_2	PFL0315c	0.01583	-0.54755	-1.79407	-1.18199	1.14583
aPFL0315c_3	PFL0315c	0.01583	-0.54755	-1.79407	-1.18199	1.14583
aPFL0315c_2	PFL0315c	0.01583	-0.54755	-1.79407	-1.18199	1.14583
aPFL0315c_1	PFL0315c	0.01583	-0.54755	-1.79407	-1.18199	1.14583
aPFL0315c_0	PFL0315c	0.01583	-0.54755	-1.79407	-1.18199	1.14583
intPFL0325w_2	PFL0320w	-0.50176	0.430773	0.229963	-0.1443	-0.18361
aPFL0320w_0	PFL0320w	0.294257	-0.74235	-1.61833	-0.85169	1.096407
aPFL0325w_1	PFL0325w	-0.50176	0.430773	0.229963	-0.1443	-0.18361
aPFL0325w_0	PFL0325w	-0.50176	0.430773	0.229963	-0.1443	-0.18361
intPFL0330c_2	PFL0330c	0.74698	0.9201	0.7543	-0.2253	-1.35643
aPFL0330c_2	PFL0330c	0.74698	0.9201	0.7543	-0.2253	-1.35643
aPFL0330c_1	PFL0330c	0.74698	0.9201	0.7543	-0.2253	-1.35643
aPFL0330c_0	PFL0330c	0.74698	0.9201	0.7543	-0.2253	-1.35643
intPFL0335c_2	PFL0335c	-0.17693	-0.06233	0.395177	0.291157	-0.27033
aPFL0335c_1	PFL0335c	-0.17693	-0.06233	0.395177	0.291157	-0.27033
aPFL0335c_0	PFL0335c	-0.17693	-0.06233	0.395177	0.291157	-0.27033
intPFL0345w_2	PFL0340w	0.578043	0.008793	-0.49184	-0.442	0.051873
intPFL0340w_2	PFL0340w	-0.13643	-0.23086	0.333463	0.412203	-0.10148
aPFL0340w_1	PFL0340w	-0.13643	-0.23086	0.333463	0.412203	-0.10148
aPFL0340w_0	PFL0340w	-0.13643	-0.23086	0.333463	0.412203	-0.10148
aPFL0345w_1	PFL0345w	0.578043	0.008793	-0.49184	-0.442	0.051873
aPFL0345w_0	PFL0345w	0.578043	0.008793	-0.49184	-0.442	0.051873
intPFL0350c_2	PFL0350c	-0.96495	-0.72177	-0.77584	0.116672	1.437852
aPFL0350c_3	PFL0350c	-0.96495	-0.72177	-0.77584	0.116672	1.437852
aPFL0350c_2	PFL0350c	-0.96495	-0.72177	-0.77584	0.116672	1.437852

aPFL0350c_1	PFL0350c		-0.96495	-0.72177	-0.77584	0.116672	1.437852
aPFL0350c_0	PFL0350c		-0.96495	-0.72177	-0.77584	0.116672	1.437852
intPFL0355c_2	PFL0355c		0.329393	0.279053	0.212893	-0.16023	-0.55439
aPFL0355c_1	PFL0355c		0.329393	0.279053	0.212893	-0.16023	-0.55439
aPFL0355c_0	PFL0355c		0.329393	0.279053	0.212893	-0.16023	-0.55439
intPFL0360c_2	PFL0360c		-0.55551	-0.54967	-0.24056	0.36036	0.80027
aPFL0360c_4	PFL0360c		-0.55551	-0.54967	-0.24056	0.36036	0.80027
aPFL0360c_3	PFL0360c		-0.55551	-0.54967	-0.24056	0.36036	0.80027
aPFL0360c_2	PFL0360c		-0.55551	-0.54967	-0.24056	0.36036	0.80027
aPFL0360c_1	PFL0360c		-0.55551	-0.54967	-0.24056	0.36036	0.80027
aPFL0360c_0	PFL0360c		-0.55551	-0.54967	-0.24056	0.36036	0.80027
intPFL0365c_2	PFL0365c		-0.63566	-0.77231	0.10683	0.63497	0.45361
aPFL0365c_0	PFL0365c		-0.63566	-0.77231	0.10683	0.63497	0.45361
intPFL0370w_2	PFL0370w		1.62633	0.52048	-0.5609	-0.91886	-0.5451
aPFL0370w_1	PFL0370w		1.62633	0.52048	-0.5609	-0.91886	-0.5451
intPFL0375w_2	PFL0375w		-0.42334	-0.29288	-0.03002	0.27526	0.4138
aPFL0375w_0	PFL0375w		-0.42334	-0.29288	-0.03002	0.27526	0.4138
aPFL0380c_1	PFL0380c		0.633063	0.712193	0.569113	-0.07995	-0.89953
aPFL0380c_0	PFL0380c		0.633063	0.712193	0.569113	-0.07995	-0.89953
intPFL0380c_2	PFL0385c		0.633063	0.712193	0.569113	-0.07995	-0.89953
aPFL0385c_1	PFL0385c		0.912873	0.194653	0.215693	-0.06003	-0.65999
aPFL0385c_0	PFL0385c		0.912873	0.194653	0.215693	-0.06003	-0.65999
intPFL0385c_2	PFL0390c		0.912873	0.194653	0.215693	-0.06003	-0.65999
aPFL0390c_1	PFL0390c		0.261177	0.086607	-0.05821	-0.06345	-0.01777
aPFL0390c_0	PFL0390c		0.261177	0.086607	-0.05821	-0.06345	-0.01777
intPFL0400w_2	PFL0395c						
intPFL0390c_2	PFL0395c		0.261177	0.086607	-0.05821	-0.06345	-0.01777
aPFL0395c_1	PFL0395c		-0.65009	-0.33653	0.864817	0.839017	-0.33463
aPFL0395c_0	PFL0395c		-0.65009	-0.33653	0.864817	0.839017	-0.33463
intPFL0395c_2	PFL0400w		-0.65009	-0.33653	0.864817	0.839017	-0.33463
aPFL0400w_0	PFL0400w						
intPFL0410w_2	PFL0405w		-0.1157	-0.25897	-0.39114	-0.31763	0.106552
intPFL0405w_3	PFL0405w		0.780267	0.611457	0.542547	-0.27855	-1.26495
aPFL0405w_6	PFL0405w		0.780267	0.611457	0.542547	-0.27855	-1.26495
aPFL0405w_5	PFL0405w		0.780267	0.611457	0.542547	-0.27855	-1.26495
aPFL0405w_4	PFL0405w		0.780267	0.611457	0.542547	-0.27855	-1.26495
aPFL0405w_3	PFL0405w		0.780267	0.611457	0.542547	-0.27855	-1.26495
aPFL0405w_2	PFL0405w		0.780267	0.611457	0.542547	-0.27855	-1.26495
aPFL0405w_1	PFL0405w		0.780267	0.611457	0.542547	-0.27855	-1.26495
aPFL0405w_0	PFL0405w		0.780267	0.611457	0.542547	-0.27855	-1.26495
aPFL0410w_5	PFL0410w		-0.1157	-0.25897	-0.39114	-0.31763	0.106552
aPFL0410w_4	PFL0410w		-0.1157	-0.25897	-0.39114	-0.31763	0.106552
aPFL0410w_3	PFL0410w		-0.1157	-0.25897	-0.39114	-0.31763	0.106552
aPFL0410w_2	PFL0410w		-0.1157	-0.25897	-0.39114	-0.31763	0.106552
aPFL0410w_1	PFL0410w		-0.1157	-0.25897	-0.39114	-0.31763	0.106552
aPFL0410w_0	PFL0410w		-0.1157	-0.25897	-0.39114	-0.31763	0.106552
intPFL0415w_2	PFL0415w		-0.97002	-1.3059	1.051797	1.708537	0.087237

aPFL0415w_0	PFL0415w	-0.97002	-1.3059	1.051797	1.708537	0.087237
intPFL0420w_2	PFL0420w	-0.82807	-0.60766	-1.11052	-0.11415	1.73566
aPFL0420w_2	PFL0420w	-0.82807	-0.60766	-1.11052	-0.11415	1.73566
aPFL0420w_1	PFL0420w	-0.82807	-0.60766	-1.11052	-0.11415	1.73566
aPFL0420w_0	PFL0420w	-0.82807	-0.60766	-1.11052	-0.11415	1.73566
intPFL0430w_2	PFL0425c	-0.04551	0.49254	1.21592	0.58208	-1.01254
aPFL0425c_2	PFL0425c	0.539513	0.319783	0.785273	0.317613	-0.9299
aPFL0425c_1	PFL0425c	0.539513	0.319783	0.785273	0.317613	-0.9299
aPFL0425c_0	PFL0425c	0.539513	0.319783	0.785273	0.317613	-0.9299
intPFL0435w_2	PFL0430w	-0.44716	-0.11221	0.384413	0.461253	0.061373
intPFL0425c_2	PFL0430w	0.539513	0.319783	0.785273	0.317613	-0.9299
aPFL0430w_0	PFL0430w	-0.04551	0.49254	1.21592	0.58208	-1.01254
aPFL0435w_1	PFL0435w	-0.44716	-0.11221	0.384413	0.461253	0.061373
aPFL0435w_0	PFL0435w	-0.44716	-0.11221	0.384413	0.461253	0.061373
intPFL0440c_3	PFL0440c	0.62453	0.38085	-0.43145	-0.61105	-0.07985
aPFL0440c_1	PFL0440c	0.62453	0.38085	-0.43145	-0.61105	-0.07985
aPFL0440c_0	PFL0440c	0.62453	0.38085	-0.43145	-0.61105	-0.07985
intPFL0445w_2	PFL0445w	-1.45568	-1.12696	0.047753	0.971353	1.103403
aPFL0445w_2	PFL0445w	-1.45568	-1.12696	0.047753	0.971353	1.103403
aPFL0445w_1	PFL0445w	-1.45568	-1.12696	0.047753	0.971353	1.103403
aPFL0445w_0	PFL0445w	-1.45568	-1.12696	0.047753	0.971353	1.103403
intPFL0450c_2	PFL0450c	-0.22514	0.012737	0.625037	0.567297	-0.2114
aPFL0450c_0	PFL0450c	-0.22514	0.012737	0.625037	0.567297	-0.2114
intPFL0460w_2	PFL0455c	0.29996	0.3642	0.4806	0.0614	-0.6462
aPFL0455c_1	PFL0455c	0.96473	0.69536	0.48753	-0.21203	-1.06986
aPFL0455c_0	PFL0455c	0.96473	0.69536	0.48753	-0.21203	-1.06986
intPFL0455c_2	PFL0460w	0.96473	0.69536	0.48753	-0.21203	-1.06986
aPFL0460w_0	PFL0460w	0.29996	0.3642	0.4806	0.0614	-0.6462
intPFL0465c_2	PFL0465c	-0.74743	-0.6815	-0.44484	0.04928	0.68428
aPFL0465c_2	PFL0465c	-0.74743	-0.6815	-0.44484	0.04928	0.68428
aPFL0465c_1	PFL0465c	-0.74743	-0.6815	-0.44484	0.04928	0.68428
aPFL0465c_0	PFL0465c	-0.74743	-0.6815	-0.44484	0.04928	0.68428
intPFL0470w_2	PFL0470w	0.40819	-0.09146	-0.39608	-0.86342	-0.76604
aPFL0470w_1	PFL0470w	0.40819	-0.09146	-0.39608	-0.86342	-0.76604
aPFL0470w_0	PFL0470w	0.40819	-0.09146	-0.39608	-0.86342	-0.76604
intPFL0480w_2	PFL0475w	0.246207	-0.06902	-0.9812	-0.89696	0.325357
intPFL0475w_2	PFL0475w	0.375337	-0.23068	-1.31562	-1.00004	0.633017
aPFL0475w_1	PFL0475w	0.375337	-0.23068	-1.31562	-1.00004	0.633017
aPFL0475w_0	PFL0475w	0.375337	-0.23068	-1.31562	-1.00004	0.633017
intPFL0485w_2	PFL0480w	-0.47677	-0.24311	0.335227	0.454107	0.063447
aPFL0480w_1	PFL0480w	0.246207	-0.06902	-0.9812	-0.89696	0.325357
aPFL0480w_0	PFL0480w	0.246207	-0.06902	-0.9812	-0.89696	0.325357
aPFL0485w_0	PFL0485w	-0.47677	-0.24311	0.335227	0.454107	0.063447
aPFL0490c_0	PFL0490c	-1.38766	-0.51569	1.09487	1.23613	-0.03431
intPFL0490c_2	PFL0495c	-1.38766	-0.51569	1.09487	1.23613	-0.03431
aPFL0495c_1	PFL0495c	-1.1588	-0.89254	-0.00869	0.684353	0.833703
aPFL0495c_0	PFL0495c	-1.1588	-0.89254	-0.00869	0.684353	0.833703

intPFL0500w_2	PFL0500w	-0.75103	-0.39482	-0.13635	0.346687	0.757157
intPFL0495c_2	PFL0500w	-1.1588	-0.89254	-0.00869	0.684353	0.833703
aPFL0500w_0	PFL0500w	-0.75103	-0.39482	-0.13635	0.346687	0.757157
intPFL0505c_2	PFL0505c	-1.1448	-0.63601	0.15002	0.55098	0.50901
aPFL0505c_0	PFL0505c	-1.1448	-0.63601	0.15002	0.55098	0.50901
intPFL0510c_2	PFL0510c	0.405993	-0.10395	-1.2966	-0.79024	0.987113
aPFL0510c_1	PFL0510c	0.405993	-0.10395	-1.2966	-0.79024	0.987113
aPFL0510c_0	PFL0510c	0.405993	-0.10395	-1.2966	-0.79024	0.987113
intPFL0515w_2	PFL0515w	0.472353	0.289233	0.664033	0.279633	-0.76257
aPFL0515w_0	PFL0515w	0.472353	0.289233	0.664033	0.279633	-0.76257
intPFL0525w_2	PFL0520c	1.62545	0.89927	0.36096	-0.55046	-1.42977
aPFL0520c_1	PFL0520c	0.996843	0.822283	0.087683	-0.58552	-0.82312
aPFL0520c_0	PFL0520c	0.996843	0.822283	0.087683	-0.58552	-0.82312
intPFL0520c_2	PFL0525w	0.996843	0.822283	0.087683	-0.58552	-0.82312
aPFL0525w_1	PFL0525w	1.62545	0.89927	0.36096	-0.55046	-1.42977
aPFL0525w_0	PFL0525w	1.62545	0.89927	0.36096	-0.55046	-1.42977
intPFL0530c_2	PFL0530c	0.6003	0.00398	-0.05121	-0.17329	-0.36348
aPFL0530c_1	PFL0530c	0.6003	0.00398	-0.05121	-0.17329	-0.36348
aPFL0530c_0	PFL0530c	0.6003	0.00398	-0.05121	-0.17329	-0.36348
intPFL0535c_2	PFL0535c	-0.81765	-0.97477	-0.45097	0.570633	1.305433
aPFL0535c_1	PFL0535c	-0.81765	-0.97477	-0.45097	0.570633	1.305433
aPFL0535c_0	PFL0535c	-0.81765	-0.97477	-0.45097	0.570633	1.305433
intPFL0540w_2	PFL0540w	-0.41941	-0.57797	-0.22207	0.39257	0.75047
aPFL0540w_1	PFL0540w	-0.41941	-0.57797	-0.22207	0.39257	0.75047
aPFL0540w_0	PFL0540w	-0.41941	-0.57797	-0.22207	0.39257	0.75047
intPFL0545w_2	PFL0545w	-0.07435	-0.06991	-0.40508	-0.26086	0.329535
aPFL0545w_2	PFL0545w	-0.07435	-0.06991	-0.40508	-0.26086	0.329535
aPFL0545w_1	PFL0545w	-0.07435	-0.06991	-0.40508	-0.26086	0.329535
aPFL0545w_0	PFL0545w	-0.07435	-0.06991	-0.40508	-0.26086	0.329535
intPFL0550w_2	PFL0550w	0.055992	-0.08845	0.062322	0.109302	-0.02793
aPFL0550w_0	PFL0550w	0.055992	-0.08845	0.062322	0.109302	-0.02793
intPFL0555c_2	PFL0555c	0.508742	0.539792	-0.01416	-0.27768	-0.20029
aPFL0555c_2	PFL0555c	0.508742	0.539792	-0.01416	-0.27768	-0.20029
aPFL0555c_1	PFL0555c	0.508742	0.539792	-0.01416	-0.27768	-0.20029
aPFL0555c_0	PFL0555c	0.508742	0.539792	-0.01416	-0.27768	-0.20029
intPFL0560c_2	PFL0560c	-0.6515	-0.71696	-0.73233	0.127667	1.273297
aPFL0560c_1	PFL0560c	-0.6515	-0.71696	-0.73233	0.127667	1.273297
aPFL0560c_0	PFL0560c	-0.6515	-0.71696	-0.73233	0.127667	1.273297
intPFL0565w_3	PFL0565w	-0.10655	0.259023	0.329103	0.143043	-0.1806
aPFL0565w_0	PFL0565w	-0.10655	0.259023	0.329103	0.143043	-0.1806
intPFL0575w_2	PFL0570c	0.33962	0.32889	0.10247	-0.05522	-0.16464
intPFL0570c_2	PFL0570c	0.78041	0.14794	0.28963	0.05237	-0.61394
aPFL0570c_0	PFL0570c	0.78041	0.14794	0.28963	0.05237	-0.61394
aPFL0575w_4	PFL0575w	0.33962	0.32889	0.10247	-0.05522	-0.16464
aPFL0575w_3	PFL0575w	0.33962	0.32889	0.10247	-0.05522	-0.16464
aPFL0575w_2	PFL0575w	0.33962	0.32889	0.10247	-0.05522	-0.16464
aPFL0575w_1	PFL0575w	0.33962	0.32889	0.10247	-0.05522	-0.16464

aPFL0575w_0	PFL0575w	0.33962	0.32889	0.10247	-0.05522	-0.16464
intPFL0580w_2	PFL0580w	-1.35253	-1.71585	-0.63404	0.969877	1.924187
aPFL0580w_1	PFL0580w	-1.35253	-1.71585	-0.63404	0.969877	1.924187
aPFL0580w_0	PFL0580w	-1.35253	-1.71585	-0.63404	0.969877	1.924187
intPFL0585w_3	PFL0585w	-0.45579	-0.38509	-0.41132	0.086983	0.779753
intPFL0590c_2	PFL0590c	-0.14626	0.21632	0.54286	0.03064	-0.75632
aPFL0590c_1	PFL0590c	-0.14626	0.21632	0.54286	0.03064	-0.75632
aPFL0590c_0	PFL0590c	-0.14626	0.21632	0.54286	0.03064	-0.75632
intPFL0600w_2	PFL0595c	-0.59009	-0.18205	0.073107	0.398727	0.539387
aPFL0595c_0	PFL0595c	-1.02182	-0.71594	0.151863	0.999803	1.084603
intPFL0595c_2	PFL0600w	-1.02182	-0.71594	0.151863	0.999803	1.084603
aPFL0600w_1	PFL0600w	-0.59009	-0.18205	0.073107	0.398727	0.539387
aPFL0600w_0	PFL0600w	-0.59009	-0.18205	0.073107	0.398727	0.539387
intPFL0610w_2	PFL0605c	-0.75574	-0.36731	0.572873	0.787793	0.138473
intPFL0605c_2	PFL0605c	0.043883	-0.07993	0.806103	0.627563	-0.60441
aPFL0605c_1	PFL0605c	0.043883	-0.07993	0.806103	0.627563	-0.60441
aPFL0605c_0	PFL0605c	0.043883	-0.07993	0.806103	0.627563	-0.60441
aPFL0610w_1	PFL0610w	-0.75574	-0.36731	0.572873	0.787793	0.138473
aPFL0610w_0	PFL0610w	-0.75574	-0.36731	0.572873	0.787793	0.138473
intPFL0615w_2	PFL0615w	-0.50033	-0.62236	0.152203	0.651463	0.450023
aPFL0615w_0	PFL0615w	-0.50033	-0.62236	0.152203	0.651463	0.450023
intPFL0620c_3	PFL0620c	-0.01223	0.400177	-0.24586	-0.1933	0.431657
aPFL0620c_1	PFL0620c	-0.01223	0.400177	-0.24586	-0.1933	0.431657
aPFL0620c_0	PFL0620c	-0.01223	0.400177	-0.24586	-0.1933	0.431657
intPFL0623c_2	PFL0623c-b					
intPFL0625c_2	PFL0625c	0.75198	1.18878	0.27944	-0.57411	-0.83161
aPFL0625c_2	PFL0625c	0.75198	1.18878	0.27944	-0.57411	-0.83161
aPFL0625c_1	PFL0625c	0.75198	1.18878	0.27944	-0.57411	-0.83161
aPFL0625c_0	PFL0625c	0.75198	1.18878	0.27944	-0.57411	-0.83161
intPFL0630w_2	PFL0630w	0.326237	-0.52717	-0.89766	-0.15	0.960507
aPFL0630w_0	PFL0630w	0.326237	-0.52717	-0.89766	-0.15	0.960507
intPFL0635c_2	PFL0635c	1.27973	-0.10965	-0.4162	-0.5818	-0.60935
aPFL0635c_1	PFL0635c	1.27973	-0.10965	-0.4162	-0.5818	-0.60935
aPFL0635c_0	PFL0635c	1.27973	-0.10965	-0.4162	-0.5818	-0.60935
intPFL0640w_2	PFL0637c	0.126093	0.126523	0.483373	0.369313	-0.2899
aPFL0640w_0	PFL0640w	0.126093	0.126523	0.483373	0.369313	-0.2899
aPFL0645c_0	PFL0645c	0.052185	-0.07993	-0.01407	0.112325	0.123595
intPFL0655w_2	PFL0650c	1.145853	0.381353	-0.0925	-0.41424	-0.57949
intPFL0650c_2	PFL0650c	0.501197	0.421027	-0.6878	-0.82686	0.134307
intPFL0645c_2	PFL0650c	0.052185	-0.07993	-0.01407	0.112325	0.123595
aPFL0650c_1	PFL0650c	0.501197	0.421027	-0.6878	-0.82686	0.134307
aPFL0650c_0	PFL0650c	0.501197	0.421027	-0.6878	-0.82686	0.134307
aPFL0655w_1	PFL0655w	1.145853	0.381353	-0.0925	-0.41424	-0.57949
aPFL0655w_0	PFL0655w	1.145853	0.381353	-0.0925	-0.41424	-0.57949
intPFL0660w_2	PFL0660w	-1.02632	-1.32137	0.444747	1.400587	0.746707
aPFL0660w_0	PFL0660w	-1.02632	-1.32137	0.444747	1.400587	0.746707
intPFL0665c_2	PFL0665c	0.379917	0.450397	0.955707	0.209627	-1.26406

aPFL0665c_0	PFL0665c	0.379917	0.450397	0.955707	0.209627	-1.26406
intPFL0670c_2	PFL0670c	0.279593	0.257013	0.633973	0.316193	-0.60985
aPFL0670c_1	PFL0670c	0.279593	0.257013	0.633973	0.316193	-0.60985
aPFL0670c_0	PFL0670c	0.279593	0.257013	0.633973	0.316193	-0.60985
intPFL0675c_3	PFL0675c	0.716857	0.713217	0.348567	-0.51123	-1.16938
aPFL0675c_1	PFL0675c	0.716857	0.713217	0.348567	-0.51123	-1.16938
aPFL0675c_0	PFL0675c	0.716857	0.713217	0.348567	-0.51123	-1.16938
intPFL0685w_2	PFL0680c	-0.56142	-1.09488	-0.77375	0.42775	1.51738
aPFL0680c_0	PFL0680c	-0.70276	-1.19019	-0.00762	0.92562	0.84319
intPFL0680c_2	PFL0685w	-0.70276	-1.19019	-0.00762	0.92562	0.84319
aPFL0685w_1	PFL0685w	-0.56142	-1.09488	-0.77375	0.42775	1.51738
aPFL0685w_0	PFL0685w	-0.56142	-1.09488	-0.77375	0.42775	1.51738
aPFL0690c_0	PFL0690c	0.313883	0.307573	0.519853	0.285813	-0.42391
intPFL0690c_2	PFL0692c	0.313883	0.307573	0.519853	0.285813	-0.42391
aPFL0695c_1	PFL0692c	0.33283	0.07237	0.31025	0.27375	-0.22087
aPFL0695c_0	PFL0692c	0.33283	0.07237	0.31025	0.27375	-0.22087
intPFL0700w_2	PFL0695c	0.69133	1.25333	0.95083	-0.11083	-1.30433
intPFL0695c_2	PFL0700w	0.33283	0.07237	0.31025	0.27375	-0.22087
aPFL0700w_0	PFL0700w	0.69133	1.25333	0.95083	-0.11083	-1.30433
intPFL0710w_2	PFL0705c	0.237825	0.639845	0.283785	-0.08614	-0.3022
aPFL0705c_0	PFL0705c	0.190018	0.232928	0.673148	0.393408	-0.55204
intPFL0715w_2	PFL0710w	0.114417	0.246917	0.276167	0.090167	-0.24458
intPFL0705c_2	PFL0710w	0.190018	0.232928	0.673148	0.393408	-0.55204
aPFL0710w_0	PFL0710w	0.237825	0.639845	0.283785	-0.08614	-0.3022
aPFL0715w_0	PFL0715w	0.114417	0.246917	0.276167	0.090167	-0.24458
intPFL0720w_2	PFL0720w	-0.79022	-0.49268	-0.27527	0.336102	0.943762
aPFL0720w_0	PFL0720w	-0.79022	-0.49268	-0.27527	0.336102	0.943762
intPFL0725w_2	PFL0725w	-0.97657	-1.06912	-0.61325	0.443587	1.402457
aPFL0725w_0	PFL0725w	-0.97657	-1.06912	-0.61325	0.443587	1.402457
intPFL0730w_3	PFL0730w	-0.53284	0.296163	1.141663	0.593003	-0.8285
aPFL0730w_1	PFL0730w	-0.53284	0.296163	1.141663	0.593003	-0.8285
aPFL0730w_0	PFL0730w	-0.53284	0.296163	1.141663	0.593003	-0.8285
intPFL0735w_2	PFL0735w	-0.9235	-1.1495	-0.2085	0.8345	1.1755
aPFL0735w_0	PFL0735w	-0.9235	-1.1495	-0.2085	0.8345	1.1755
intPFL0740c_2	PFL0740c	-1.12196	-0.2182	1.640893	1.369773	-0.72514
aPFL0740c_0	PFL0740c	-1.12196	-0.2182	1.640893	1.369773	-0.72514
intPFL0745c_2	PFL0745c	0.005333	-0.54067	-0.64067	-0.01567	0.788833
aPFL0745c_1	PFL0745c	0.005333	-0.54067	-0.64067	-0.01567	0.788833
aPFL0745c_0	PFL0745c	0.005333	-0.54067	-0.64067	-0.01567	0.788833
intPFL0750w_2	PFL0746c	-0.25821	-0.73595	-0.24411	0.489773	0.777613
aPFL0750w_0	PFL0750w	-0.25821	-0.73595	-0.24411	0.489773	0.777613
intPFL0760w_2	PFL0755c					
aPFL0755c_1	PFL0755c	0.851963	0.523743	0.084523	-0.40572	-0.69836
aPFL0755c_0	PFL0755c	0.851963	0.523743	0.084523	-0.40572	-0.69836
intPFL0755c_2	PFL0760w	0.851963	0.523743	0.084523	-0.40572	-0.69836
aPFL0760w_1	PFL0760w					
aPFL0760w_0	PFL0760w					

intPFL0765w_2	PFL0765w	0.505993	0.570553	-0.18485	-0.32999	0.055613
aPFL0765w_1	PFL0765w	0.505993	0.570553	-0.18485	-0.32999	0.055613
aPFL0765w_0	PFL0765w	0.505993	0.570553	-0.18485	-0.32999	0.055613
intPFL0770w_2	PFL0770w	-0.5548	-0.24502	0.159273	0.435393	0.382683
aPFL0770w_1	PFL0770w	-0.5548	-0.24502	0.159273	0.435393	0.382683
aPFL0770w_0	PFL0770w	-0.5548	-0.24502	0.159273	0.435393	0.382683
intPFL0775w_2	PFL0775w	-0.54438	-1.0721	-0.04331	0.77931	0.7151
aPFL0775w_0	PFL0775w	-0.54438	-1.0721	-0.04331	0.77931	0.7151
intPFL0780w_2	PFL0780w	-0.31377	0.27785	1.1693	0.4617	-1.16135
aPFL0780w_1	PFL0780w	-0.31377	0.27785	1.1693	0.4617	-1.16135
aPFL0780w_0	PFL0780w	-0.31377	0.27785	1.1693	0.4617	-1.16135
aPFL0785c_0	PFL0785c	0.379577	0.488127	0.011757	-0.35342	-0.35279
intPFL0790w_2	PFL0790w	0.651453	0.386213	-0.43069	-0.58265	-0.04405
intPFL0785c_2	PFL0790w	0.379577	0.488127	0.011757	-0.35342	-0.35279
aPFL0790w_1	PFL0790w	0.651453	0.386213	-0.43069	-0.58265	-0.04405
aPFL0790w_0	PFL0790w	0.651453	0.386213	-0.43069	-0.58265	-0.04405
intPFL0795c_2	PFL0795c	-0.20214	-0.3532	-0.84005	-0.43991	0.712137
aPFL0795c_0	PFL0795c	-0.20214	-0.3532	-0.84005	-0.43991	0.712137
intPFL0800c_2	PFL0800c	0.068587	-0.36695	0.052417	0.203917	-0.05771
aPFL0800c_0	PFL0800c	0.068587	-0.36695	0.052417	0.203917	-0.05771
intPFL0805w_2	PFL0805w	0.269258	0.469698	0.277378	-0.13866	-0.49313
aPFL0805w_1	PFL0805w	0.269258	0.469698	0.277378	-0.13866	-0.49313
aPFL0805w_0	PFL0805w	0.269258	0.469698	0.277378	-0.13866	-0.49313
intPFL0815w_2	PFL0815w	0.04706	0.11521	0.39108	0.21042	-0.34021
aPFL0815w_1	PFL0815w	0.04706	0.11521	0.39108	0.21042	-0.34021
aPFL0815w_0	PFL0815w	0.04706	0.11521	0.39108	0.21042	-0.34021
aPFL0820c_0	PFL0820c	-0.11551	-0.54746	0.319923	0.658743	0.075123
intPFL0830w_1	PFL0825c-a	0.68019	0.82803	0.57894	-0.46794	-1.43803
intPFL0820c_2	PFL0825c-a	-0.11551	-0.54746	0.319923	0.658743	0.075123
aPFL0825c_0	PFL0825c-a	0.555497	0.039537	0.195417	-0.18008	-0.7872
intPFL0825c_2	PFL0830w	0.555497	0.039537	0.195417	-0.18008	-0.7872
aPFL0830w_1	PFL0830w	0.68019	0.82803	0.57894	-0.46794	-1.43803
aPFL0830w_0	PFL0830w	0.68019	0.82803	0.57894	-0.46794	-1.43803
intPFL0835w_2	PFL0835w	0.272257	-0.09977	-0.19496	-0.0657	0.101607
aPFL0835w_1	PFL0835w	0.272257	-0.09977	-0.19496	-0.0657	0.101607
aPFL0835w_0	PFL0835w	0.272257	-0.09977	-0.19496	-0.0657	0.101607
intPFL0840c_2	PFL0840c	0.392007	-0.08905	-1.0754	-1.03176	0.233887
aPFL0840c_1	PFL0840c	0.392007	-0.08905	-1.0754	-1.03176	0.233887
aPFL0840c_0	PFL0840c	0.392007	-0.08905	-1.0754	-1.03176	0.233887
intPFL0845w_1	PFL0845w	0.485825	0.140045	0.476825	-0.08261	-1.06666
aPFL0845w_0	PFL0845w	0.485825	0.140045	0.476825	-0.08261	-1.06666
intPFL0850w_2	PFL0850w	1.743558	0.569738	-0.72021	-1.23395	-0.7319
aPFL0850w_0	PFL0850w	1.743558	0.569738	-0.72021	-1.23395	-0.7319
aPFL0855c_1	PFL0855c	0.420447	0.696777	-0.07955	-0.48111	-0.25144
aPFL0855c_0	PFL0855c	0.420447	0.696777	-0.07955	-0.48111	-0.25144
intPFL0865w_2	PFL0860c	-0.13913	-0.13535	-0.46779	-0.23137	0.466687
intPFL0860c_2	PFL0860c	0.50584	0.37081	0.11337	-0.25037	-0.50481

intPFL0855c_2	PFL0860c	0.420447	0.696777	-0.07955	-0.48111	-0.25144
aPFL0860c_0	PFL0860c	0.50584	0.37081	0.11337	-0.25037	-0.50481
aPFL0865w_1	PFL0865w	-0.13913	-0.13535	-0.46779	-0.23137	0.466687
aPFL0865w_0	PFL0865w	-0.13913	-0.13535	-0.46779	-0.23137	0.466687
intPFL0870w_2	PFL0870w					
aPFL0870w_0	PFL0870w					
intPFL0875w_2	PFL0875w	0.960673	-0.05889	-1.08174	-1.3201	-0.35645
aPFL0875w_1	PFL0875w	0.960673	-0.05889	-1.08174	-1.3201	-0.35645
aPFL0875w_0	PFL0875w	0.960673	-0.05889	-1.08174	-1.3201	-0.35645
intPFL0880c_3	PFL0880c	0.489688	0.564558	0.007248	-0.54483	-0.61739
aPFL0880c_3	PFL0880c	0.489688	0.564558	0.007248	-0.54483	-0.61739
aPFL0880c_2	PFL0880c	0.489688	0.564558	0.007248	-0.54483	-0.61739
aPFL0880c_1	PFL0880c	0.489688	0.564558	0.007248	-0.54483	-0.61739
aPFL0880c_0	PFL0880c	0.489688	0.564558	0.007248	-0.54483	-0.61739
intPFL0885w_2	PFL0885w	0.254673	0.074123	-0.21901	-0.42733	-0.27496
aPFL0885w_1	PFL0885w	0.254673	0.074123	-0.21901	-0.42733	-0.27496
aPFL0885w_0	PFL0885w	0.254673	0.074123	-0.21901	-0.42733	-0.27496
aPFL0890c_1	PFL0890c	0.058417	-0.11009	-0.08581	0.021647	0.096927
aPFL0890c_0	PFL0890c	0.058417	-0.11009	-0.08581	0.021647	0.096927
intPFL0895c_2	PFL0895c	1.335317	0.818917	-0.20507	-1.29159	-1.46058
aPFL0895c_1	PFL0895c	1.335317	0.818917	-0.20507	-1.29159	-1.46058
aPFL0895c_0	PFL0895c	1.335317	0.818917	-0.20507	-1.29159	-1.46058
intPFL0900c_2	PFL0900c	0.146497	0.623537	0.798167	0.152167	-0.8682
aPFL0900c_1	PFL0900c	0.146497	0.623537	0.798167	0.152167	-0.8682
aPFL0900c_0	PFL0900c	0.146497	0.623537	0.798167	0.152167	-0.8682
aPFL0905c_1	PFL0905c	-0.62345	-0.58671	-0.43106	0.258397	0.994547
aPFL0905c_0	PFL0905c	-0.62345	-0.58671	-0.43106	0.258397	0.994547
intPFL0915c_2	PFL0915c	-0.38208	-0.32258	0.02617	0.14883	0.08758
intPFL0905c_2	PFL0915c	-0.62345	-0.58671	-0.43106	0.258397	0.994547
aPFL0915c_0	PFL0915c	-0.38208	-0.32258	0.02617	0.14883	0.08758
aPFL0910c_0	PFL0915c	-0.19875	-0.13025	0.14	0.085	-0.14975
intPFL0920c_2	PFL0920c	-0.34298	0.16038	0.44374	0.14126	-0.38038
aPFL0920c_1	PFL0920c	-0.34298	0.16038	0.44374	0.14126	-0.38038
aPFL0920c_0	PFL0920c	-0.34298	0.16038	0.44374	0.14126	-0.38038
intPFL0925w_2	PFL0925w	-1.05516	-0.62699	0.20193	0.42857	0.20639
aPFL0925w_4	PFL0925w	-1.05516	-0.62699	0.20193	0.42857	0.20639
aPFL0925w_3	PFL0925w	-1.05516	-0.62699	0.20193	0.42857	0.20639
aPFL0925w_2	PFL0925w	-1.05516	-0.62699	0.20193	0.42857	0.20639
aPFL0925w_1	PFL0925w	-1.05516	-0.62699	0.20193	0.42857	0.20639
aPFL0925w_0	PFL0925w	-1.05516	-0.62699	0.20193	0.42857	0.20639
intPFL0930w_2	PFL0930w	-0.47169	0.042787	-0.05857	0.141687	0.505657
aPFL0930w_2	PFL0930w	-0.47169	0.042787	-0.05857	0.141687	0.505657
aPFL0930w_1	PFL0930w	-0.47169	0.042787	-0.05857	0.141687	0.505657
aPFL0930w_0	PFL0930w	-0.47169	0.042787	-0.05857	0.141687	0.505657
intPFL0950c_2	PFL0950c	0.060963	0.416513	0.118233	-0.15674	-0.19451
aPFL0950c_2	PFL0950c	0.060963	0.416513	0.118233	-0.15674	-0.19451
aPFL0950c_1	PFL0950c	0.060963	0.416513	0.118233	-0.15674	-0.19451

aPFL0950c_0	PFL0950c	0.060963	0.416513	0.118233	-0.15674	-0.19451
intPFL0955c_2	PFL0955c	-0.01051	0.21856	0.40127	-0.01847	-0.61416
aPFL0955c_0	PFL0955c	-0.01051	0.21856	0.40127	-0.01847	-0.61416
intPFL0957w_2	PFL0957w					
intPFL0960w_2	PFL0960w	-0.02831	-0.06443	0.33437	0.36463	-0.09957
aPFL0960w_0	PFL0960w	-0.02831	-0.06443	0.33437	0.36463	-0.09957
intPFL0965c_2	PFL0965c	0.857807	0.070977	-0.25319	-0.33747	-0.28064
aPFL0965c_1	PFL0965c	0.857807	0.070977	-0.25319	-0.33747	-0.28064
aPFL0965c_0	PFL0965c	0.857807	0.070977	-0.25319	-0.33747	-0.28064
intPFL0970w_2	PFL0970w	0.541253	1.079223	0.197813	-0.43465	-0.47556
aPFL0970w_1	PFL0970w	0.541253	1.079223	0.197813	-0.43465	-0.47556
aPFL0970w_0	PFL0970w	0.541253	1.079223	0.197813	-0.43465	-0.47556
intPFL0975w_2	PFL0973c	-0.5751	-1.02448	-1.28883	-0.01343	1.851417
intPFL0980w_2	PFL0975w	0.841023	0.696893	0.073713	-0.51255	-0.71973
intPFL0973c_2	PFL0975w					
aPFL0975w_4	PFL0975w	-0.5751	-1.02448	-1.28883	-0.01343	1.851417
aPFL0975w_3	PFL0975w	-0.5751	-1.02448	-1.28883	-0.01343	1.851417
aPFL0975w_2	PFL0975w	-0.5751	-1.02448	-1.28883	-0.01343	1.851417
aPFL0975w_1	PFL0975w	-0.5751	-1.02448	-1.28883	-0.01343	1.851417
aPFL0975w_0	PFL0975w	-0.5751	-1.02448	-1.28883	-0.01343	1.851417
aPFL0980w_1	PFL0980w	0.841023	0.696893	0.073713	-0.51255	-0.71973
aPFL0980w_0	PFL0980w	0.841023	0.696893	0.073713	-0.51255	-0.71973
intPFL0990w_2	PFL0985c	0.767103	0.518503	-0.00351	-0.16883	-0.15684
aPFL0985c_0	PFL0985c	0.541603	0.839963	0.321073	-0.31941	-0.6883
intPFL0985c_2	PFL0990w	0.541603	0.839963	0.321073	-0.31941	-0.6883
aPFL0990w_0	PFL0990w	0.767103	0.518503	-0.00351	-0.16883	-0.15684
intPFL0995c_2	PFL0995c	0.543313	0.351243	0.258653	-0.02889	-0.46448
aPFL0995c_0	PFL0995c	0.543313	0.351243	0.258653	-0.02889	-0.46448
intPFL1000c_2	PFL1000c	-0.30828	1.020407	0.534187	-0.21185	-0.51407
aPFL1000c_1	PFL1000c	-0.30828	1.020407	0.534187	-0.21185	-0.51407
aPFL1000c_0	PFL1000c	-0.30828	1.020407	0.534187	-0.21185	-0.51407
intPFL1005c_3	PFL1005c	-1.26362	-0.7249	-0.47069	0.52269	1.5849
aPFL1005c_0	PFL1005c	-1.26362	-0.7249	-0.47069	0.52269	1.5849
intPFL1010c_2	PFL1010c	-1.11285	-1.15034	-0.47804	0.484872	1.207752
aPFL1010c_3	PFL1010c	-1.11285	-1.15034	-0.47804	0.484872	1.207752
aPFL1010c_2	PFL1010c	-1.11285	-1.15034	-0.47804	0.484872	1.207752
aPFL1010c_1	PFL1010c	-1.11285	-1.15034	-0.47804	0.484872	1.207752
aPFL1010c_0	PFL1010c	-1.11285	-1.15034	-0.47804	0.484872	1.207752
intPFL1020w_2	PFL1015w-a	0.420327	0.546377	0.421257	0.010077	-0.53304
intPFL1015w_2	PFL1015w-a	0.331117	0.470427	-0.49535	-0.74431	-0.00509
aPFL1015w_0	PFL1015w-a	0.331117	0.470427	-0.49535	-0.74431	-0.00509
aPFL1020w_0	PFL1020w	0.420327	0.546377	0.421257	0.010077	-0.53304
intPFL1025c_2	PFL1025c	-0.44116	-0.58668	-1.26362	-0.49004	1.337517
aPFL1025c_1	PFL1025c	-0.44116	-0.58668	-1.26362	-0.49004	1.337517
aPFL1025c_0	PFL1025c	-0.44116	-0.58668	-1.26362	-0.49004	1.337517
intPFL1030w_2	PFL1030w	0.514547	0.373277	-0.15179	-0.63737	-0.60044
aPFL1030w_1	PFL1030w	0.514547	0.373277	-0.15179	-0.63737	-0.60044

aPFL1030w_0	PFL1030w	0.514547	0.373277	-0.15179	-0.63737	-0.60044
intPFL1035w_2	PFL1035w	-1.17121	-0.55094	0.923877	1.170957	0.085777
aPFL1035w_1	PFL1035w	-1.17121	-0.55094	0.923877	1.170957	0.085777
aPFL1035w_0	PFL1035w	-1.17121	-0.55094	0.923877	1.170957	0.085777
intPFL1040w_2	PFL1040w	-1.21632	-0.75186	0.391727	0.954107	0.617197
aPFL1040w_1	PFL1040w	-1.21632	-0.75186	0.391727	0.954107	0.617197
aPFL1040w_0	PFL1040w	-1.21632	-0.75186	0.391727	0.954107	0.617197
intPFL1050w_2	PFL1045w	-0.27418	-0.4371	-0.48956	0.01506	0.7201
intPFL1045w_2	PFL1045w	-1.10828	0.241917	0.790667	0.596167	-0.02458
aPFL1045w_1	PFL1045w	-1.10828	0.241917	0.790667	0.596167	-0.02458
aPFL1045w_0	PFL1045w	-1.10828	0.241917	0.790667	0.596167	-0.02458
aPFL1050w_1	PFL1050w	-0.27418	-0.4371	-0.48956	0.01506	0.7201
aPFL1050w_0	PFL1050w	-0.27418	-0.4371	-0.48956	0.01506	0.7201
intPFL1055c_2	PFL1055c	1.15527	1.75164	0.74072	-1.27122	-2.48214
aPFL1055c_1	PFL1055c	1.15527	1.75164	0.74072	-1.27122	-2.48214
aPFL1055c_0	PFL1055c	1.15527	1.75164	0.74072	-1.27122	-2.48214
intPFL1060c_2	PFL1060c	-0.10156	-0.1417	0.436883	0.531283	-0.06464
aPFL1060c_1	PFL1060c	-0.10156	-0.1417	0.436883	0.531283	-0.06464
aPFL1060c_0	PFL1060c	-0.10156	-0.1417	0.436883	0.531283	-0.06464
intPFL1065c_2	PFL1065c	-0.72033	-0.82284	0.11969	0.84781	0.74384
aPFL1065c_1	PFL1065c	-0.72033	-0.82284	0.11969	0.84781	0.74384
aPFL1065c_0	PFL1065c	-0.72033	-0.82284	0.11969	0.84781	0.74384
intPFL1070c_2	PFL1070c	-1.41479	-0.33654	0.22483	0.78367	1.01054
aPFL1070c_1	PFL1070c	-1.41479	-0.33654	0.22483	0.78367	1.01054
aPFL1070c_0	PFL1070c	-1.41479	-0.33654	0.22483	0.78367	1.01054
intPFL1075w_2	PFL1075w	-1.4391	-1.20749	0.269108	1.115058	0.853908
aPFL1075w_3	PFL1075w	-1.4391	-1.20749	0.269108	1.115058	0.853908
aPFL1075w_2	PFL1075w	-1.4391	-1.20749	0.269108	1.115058	0.853908
aPFL1075w_1	PFL1075w	-1.4391	-1.20749	0.269108	1.115058	0.853908
aPFL1075w_0	PFL1075w	-1.4391	-1.20749	0.269108	1.115058	0.853908
intPFL1080c_2	PFL1080c	0.47232	0.83238	-0.41542	-0.84332	-0.1059
aPFL1080c_0	PFL1080c	0.47232	0.83238	-0.41542	-0.84332	-0.1059
intPFL1085w_2	PFL1085w	0.730202	1.081752	-0.00163	-0.81379	-0.73992
aPFL1085w_3	PFL1085w	0.730202	1.081752	-0.00163	-0.81379	-0.73992
aPFL1085w_2	PFL1085w	0.730202	1.081752	-0.00163	-0.81379	-0.73992
aPFL1085w_1	PFL1085w	0.730202	1.081752	-0.00163	-0.81379	-0.73992
aPFL1085w_0	PFL1085w	0.730202	1.081752	-0.00163	-0.81379	-0.73992
intPFL1090w_2	PFL1090w	-0.15819	-0.86278	-1.87498	-0.87038	1.609425
aPFL1090w_0	PFL1090w	-0.15819	-0.86278	-1.87498	-0.87038	1.609425
intPFL1100w_2	PFL1095c	-1.18427	-0.98569	0.735383	1.281283	0.311353
aPFL1095c_0	PFL1095c	-0.09487	-1.43563	0.27627	0.98773	0.04963
intPFL1095c_2	PFL1100w	-0.09487	-1.43563	0.27627	0.98773	0.04963
aPFL1100w_1	PFL1100w	-1.18427	-0.98569	0.735383	1.281283	0.311353
aPFL1100w_0	PFL1100w	-1.18427	-0.98569	0.735383	1.281283	0.311353
intPFL1105c_2	PFL1105c	0.0055	-0.72548	-1.13204	-0.53296	0.79048
aPFL1105c_0	PFL1105c	0.0055	-0.72548	-1.13204	-0.53296	0.79048
intPFL1110c_2	PFL1110c	-0.35127	-1.77067	-2.10466	-0.43634	2.14067

aPFL1110c_1	PFL1110c	-0.35127	-1.77067	-2.10466	-0.43634	2.14067
aPFL1110c_0	PFL1110c	-0.35127	-1.77067	-2.10466	-0.43634	2.14067
intPFL1115w_2	PFL1115w	-0.02182	0.032137	-0.04702	0.022857	0.149197
aPFL1115w_1	PFL1115w	-0.02182	0.032137	-0.04702	0.022857	0.149197
aPFL1115w_0	PFL1115w	-0.02182	0.032137	-0.04702	0.022857	0.149197
intPFL1120c_2	PFL1120c	-0.46797	-0.12418	-0.12416	0.05648	0.38554
aPFL1120c_1	PFL1120c	-0.46797	-0.12418	-0.12416	0.05648	0.38554
aPFL1120c_0	PFL1120c	-0.46797	-0.12418	-0.12416	0.05648	0.38554
intPFL1125w_2	PFL1125w	0.141215	0.354575	-0.19516	-0.53896	-0.26136
aPFL1125w_2	PFL1125w	0.141215	0.354575	-0.19516	-0.53896	-0.26136
aPFL1125w_1	PFL1125w	0.141215	0.354575	-0.19516	-0.53896	-0.26136
aPFL1125w_0	PFL1125w	0.141215	0.354575	-0.19516	-0.53896	-0.26136
intPFL1130c_2	PFL1130c	0.573237	0.593697	-0.07684	-0.38793	-0.23631
aPFL1130c_6	PFL1130c	0.573237	0.593697	-0.07684	-0.38793	-0.23631
aPFL1130c_5	PFL1130c	0.573237	0.593697	-0.07684	-0.38793	-0.23631
aPFL1130c_4	PFL1130c	0.573237	0.593697	-0.07684	-0.38793	-0.23631
aPFL1130c_3	PFL1130c	0.573237	0.593697	-0.07684	-0.38793	-0.23631
aPFL1130c_2	PFL1130c	0.573237	0.593697	-0.07684	-0.38793	-0.23631
aPFL1130c_1	PFL1130c	0.573237	0.593697	-0.07684	-0.38793	-0.23631
aPFL1130c_0	PFL1130c	0.573237	0.593697	-0.07684	-0.38793	-0.23631
intPFL1135c_2	PFL1135c	-0.03731	0.31254	0.07367	0.04283	0.14996
aPFL1135c_2	PFL1135c	-0.03731	0.31254	0.07367	0.04283	0.14996
aPFL1135c_1	PFL1135c	-0.03731	0.31254	0.07367	0.04283	0.14996
aPFL1135c_0	PFL1135c	-0.03731	0.31254	0.07367	0.04283	0.14996
intPFL1140w_2	PFL1140w					
aPFL1140w_0	PFL1140w					
intPFL1145w_2	PFL1145w	0.395887	0.516557	0.655387	0.113947	-0.81822
aPFL1145w_0	PFL1145w	0.395887	0.516557	0.655387	0.113947	-0.81822
intPFL1155w_2	PFL1150c	-1.65558	-0.7346	1.225683	1.516483	0.089763
aPFL1150c_0	PFL1150c	-1.51156	-0.71322	1.282783	1.510583	-0.05752
intPFL1150c_2	PFL1155w	-1.51156	-0.71322	1.282783	1.510583	-0.05752
aPFL1155w_1	PFL1155w	-1.65558	-0.7346	1.225683	1.516483	0.089763
aPFL1155w_0	PFL1155w	-1.65558	-0.7346	1.225683	1.516483	0.089763
intPFL1160c_2	PFL1160c	-0.38268	-0.5316	-1.00328	-0.32238	1.128937
aPFL1160c_0	PFL1160c	-0.38268	-0.5316	-1.00328	-0.32238	1.128937
intPFL1165w_2	PFL1165w	0.73972	0.01942	-0.93434	-1.24516	-0.38842
aPFL1165w_1	PFL1165w	0.73972	0.01942	-0.93434	-1.24516	-0.38842
aPFL1165w_0	PFL1165w	0.73972	0.01942	-0.93434	-1.24516	-0.38842
intPFL1170w_2	PFL1170w	0.71339	0.39453	-0.2028	-0.4717	-0.31053
aPFL1170w_1	PFL1170w	0.71339	0.39453	-0.2028	-0.4717	-0.31053
aPFL1170w_0	PFL1170w	0.71339	0.39453	-0.2028	-0.4717	-0.31053
intPFL1175w_1	PFL1175w	0.84022	0.72039	0.65372	-0.28372	-1.42389
aPFL1175w_1	PFL1175w	0.84022	0.72039	0.65372	-0.28372	-1.42389
aPFL1175w_0	PFL1175w	0.84022	0.72039	0.65372	-0.28372	-1.42389
intPFL1180w_2	PFL1180w	-1.50703	-2.07484	-0.60682	1.23282	2.08084
aPFL1180w_0	PFL1180w	-1.50703	-2.07484	-0.60682	1.23282	2.08084
intPFL1185c_2	PFL1185c	0.069947	0.911257	0.849487	-0.04915	-1.04692

aPFL1185c_0	PFL1185c	0.069947	0.911257	0.849487	-0.04915	-1.04692
aPFL1190c_2	PFL1190c	1.138242	-0.07854	-0.87893	-0.8113	-0.03735
aPFL1190c_1	PFL1190c	1.138242	-0.07854	-0.87893	-0.8113	-0.03735
aPFL1190c_0	PFL1190c	1.138242	-0.07854	-0.87893	-0.8113	-0.03735
intPFL1195w_2	PFL1195w	0.840683	0.074603	-0.57422	-0.56612	-0.02694
intPFL1190c_2	PFL1195w	1.138242	-0.07854	-0.87893	-0.8113	-0.03735
aPFL1195w_0	PFL1195w	0.840683	0.074603	-0.57422	-0.56612	-0.02694
intPFL1200c_2	PFL1200c	0.482143	0.271283	0.161933	-0.07327	-0.38662
aPFL1200c_0	PFL1200c	0.482143	0.271283	0.161933	-0.07327	-0.38662
intPFL1210w_2	PFL1205c	-0.85068	0.31875	0.76166	0.52045	-0.11964
aPFL1205c_2	PFL1205c	-1.24329	0.29593	1.38214	0.89286	-0.58293
aPFL1205c_1	PFL1205c	-1.24329	0.29593	1.38214	0.89286	-0.58293
aPFL1205c_0	PFL1205c	-1.24329	0.29593	1.38214	0.89286	-0.58293
intPFL1205c_2	PFL1210w	-1.24329	0.29593	1.38214	0.89286	-0.58293
aPFL1210w_2	PFL1210w	-0.85068	0.31875	0.76166	0.52045	-0.11964
aPFL1210w_1	PFL1210w	-0.85068	0.31875	0.76166	0.52045	-0.11964
aPFL1210w_0	PFL1210w	-0.85068	0.31875	0.76166	0.52045	-0.11964
intPFL1215c_3	PFL1215c	0.547533	0.164323	-0.1504	-0.22794	-0.13916
aPFL1215c_1	PFL1215c	0.547533	0.164323	-0.1504	-0.22794	-0.13916
aPFL1215c_0	PFL1215c	0.547533	0.164323	-0.1504	-0.22794	-0.13916
intPFL1220w_2	PFL1220w	0.7317	0.39951	0.41548	-0.16948	-0.99351
aPFL1220w_0	PFL1220w	0.7317	0.39951	0.41548	-0.16948	-0.99351
intPFL1230w_2	PFL1225c	1.411177	1.101607	0.533037	-0.6237	-1.64877
intPFL1225c_3	PFL1225c	0.730133	0.579323	0.587853	-0.27519	-1.34766
aPFL1225c_0	PFL1225c	0.730133	0.579323	0.587853	-0.27519	-1.34766
aPFL1230w_1	PFL1230w	1.411177	1.101607	0.533037	-0.6237	-1.64877
aPFL1230w_0	PFL1230w	1.411177	1.101607	0.533037	-0.6237	-1.64877
intPFL1235c_2	PFL1235c	0.602017	0.481907	0.265927	-0.17809	-0.63407
aPFL1235c_1	PFL1235c	0.602017	0.481907	0.265927	-0.17809	-0.63407
aPFL1235c_0	PFL1235c	0.602017	0.481907	0.265927	-0.17809	-0.63407
intPFL1240c_2	PFL1240c	0.465482	0.798742	0.311182	-0.24313	-0.55785
aPFL1240c_2	PFL1240c	0.465482	0.798742	0.311182	-0.24313	-0.55785
aPFL1240c_1	PFL1240c	0.465482	0.798742	0.311182	-0.24313	-0.55785
aPFL1240c_0	PFL1240c	0.465482	0.798742	0.311182	-0.24313	-0.55785
intPFL1245w_2	PFL1245w	0.058973	0.153643	-0.19778	-0.05256	0.366523
aPFL1245w_1	PFL1245w	0.058973	0.153643	-0.19778	-0.05256	0.366523
aPFL1245w_0	PFL1245w	0.058973	0.153643	-0.19778	-0.05256	0.366523
aPFL1250c_1	PFL1250c	-0.82057	-0.41214	0.02179	0.50821	0.72114
aPFL1250c_0	PFL1250c	-0.82057	-0.41214	0.02179	0.50821	0.72114
intPFL1255c_2	PFL1255c	-0.15793	-0.26738	0.733753	0.721913	-0.38796
intPFL1250c_2	PFL1255c	-0.82057	-0.41214	0.02179	0.50821	0.72114
aPFL1255c_1	PFL1255c	-0.15793	-0.26738	0.733753	0.721913	-0.38796
aPFL1255c_0	PFL1255c	-0.15793	-0.26738	0.733753	0.721913	-0.38796
intPFL1260w_2	PFL1260w					
aPFL1260w_0	PFL1260w					
intPFL1265c_2	PFL1265c	1.512737	0.421337	-0.75393	-1.15023	-0.5665
aPFL1265c_1	PFL1265c	1.512737	0.421337	-0.75393	-1.15023	-0.5665

aPFL1265c_0	PFL1265c	1.512737	0.421337	-0.75393	-1.15023	-0.5665
intPFL1270w_2	PFL1270w	0.605287	0.116587	0.023317	-0.20898	-0.48025
aPFL1270w_0	PFL1270w	0.605287	0.116587	0.023317	-0.20898	-0.48025
intPFL1280w_2	PFL1275c	-0.35821	0.200692	0.687782	0.388332	-0.42525
aPFL1275c_1	PFL1275c	-0.63057	-0.38616	0.35508	0.61492	0.22416
aPFL1275c_0	PFL1275c	-0.63057	-0.38616	0.35508	0.61492	0.22416
intPFL1275c_2	PFL1280w	-0.63057	-0.38616	0.35508	0.61492	0.22416
aPFL1280w_2	PFL1280w	-0.35821	0.200692	0.687782	0.388332	-0.42525
aPFL1280w_1	PFL1280w	-0.35821	0.200692	0.687782	0.388332	-0.42525
aPFL1280w_0	PFL1280w	-0.35821	0.200692	0.687782	0.388332	-0.42525
intPFL1285c_2	PFL1285c	0.03481	-0.06005	-0.1394	0.0634	0.29905
aPFL1285c_0	PFL1285c	0.03481	-0.06005	-0.1394	0.0634	0.29905
intPFL1290w_2	PFL1290w	0.718527	0.800847	0.543797	-0.30696	-1.16301
aPFL1290w_1	PFL1290w	0.718527	0.800847	0.543797	-0.30696	-1.16301
aPFL1290w_0	PFL1290w	0.718527	0.800847	0.543797	-0.30696	-1.16301
intPFL1295w_2	PFL1295w	-0.48915	-0.20972	-0.48804	-0.07162	0.810557
aPFL1295w_1	PFL1295w	-0.48915	-0.20972	-0.48804	-0.07162	0.810557
aPFL1295w_0	PFL1295w	-0.48915	-0.20972	-0.48804	-0.07162	0.810557
intPFL1300c_2	PFL1300c	-1.65002	-1.53588	0.319767	1.468567	1.142717
aPFL1300c_1	PFL1300c	-1.65002	-1.53588	0.319767	1.468567	1.142717
aPFL1300c_0	PFL1300c	-1.65002	-1.53588	0.319767	1.468567	1.142717
intPFL1310c_2	PFL1310c	-0.17212	0.4101	0.87506	0.39894	-0.6751
intPFL1305c_2	PFL1310c	-0.74707	-0.77964	-1.17221	-0.23379	1.52764
aPFL1310c_1	PFL1310c	-0.17212	0.4101	0.87506	0.39894	-0.6751
aPFL1310c_0	PFL1310c	-0.17212	0.4101	0.87506	0.39894	-0.6751
intPFL1315w_2	PFL1315w	-0.29372	0.119927	0.223317	0.151907	-0.0037
aPFL1315w_2	PFL1315w	-0.29372	0.119927	0.223317	0.151907	-0.0037
aPFL1315w_1	PFL1315w	-0.29372	0.119927	0.223317	0.151907	-0.0037
aPFL1315w_0	PFL1315w	-0.29372	0.119927	0.223317	0.151907	-0.0037
intPFL1320w_2	PFL1320w	-0.43468	-0.8376	-1.48003	-0.41963	1.670437
aPFL1320w_1	PFL1320w	-0.43468	-0.8376	-1.48003	-0.41963	1.670437
aPFL1320w_0	PFL1320w	-0.43468	-0.8376	-1.48003	-0.41963	1.670437
intPFL1325c_2	PFL1325c	-0.07344	0.11518	0.00013	-0.00413	0.09482
aPFL1325c_1	PFL1325c	-0.07344	0.11518	0.00013	-0.00413	0.09482
aPFL1325c_0	PFL1325c	-0.07344	0.11518	0.00013	-0.00413	0.09482
intPFL1330c_2	PFL1330c	-0.99464	-1.75624	-1.33645	0.432833	2.260123
aPFL1330c_3	PFL1330c	-0.99464	-1.75624	-1.33645	0.432833	2.260123
aPFL1330c_2	PFL1330c	-0.99464	-1.75624	-1.33645	0.432833	2.260123
aPFL1330c_1	PFL1330c	-0.99464	-1.75624	-1.33645	0.432833	2.260123
aPFL1330c_0	PFL1330c	-0.99464	-1.75624	-1.33645	0.432833	2.260123
intPFL1335w_2	PFL1335w	-1.23183	-1.69085	-0.98429	0.56329	1.93285
aPFL1335w_1	PFL1335w	-1.23183	-1.69085	-0.98429	0.56329	1.93285
aPFL1335w_0	PFL1335w	-1.23183	-1.69085	-0.98429	0.56329	1.93285
intPFL1340c_2	PFL1340c	0.703187	0.384567	-0.61238	-1.00228	-0.35873
aPFL1340c_1	PFL1340c	0.703187	0.384567	-0.61238	-1.00228	-0.35873
aPFL1340c_0	PFL1340c	0.703187	0.384567	-0.61238	-1.00228	-0.35873
intPFL1350w_2	PFL1345c	0.78659	0.78306	0.60612	-0.19312	-1.14106

intPFL1345c_2	PFL1345c	0.839253	0.985753	0.803253	-0.40759	-1.70659
aPFL1345c_1	PFL1345c	0.839253	0.985753	0.803253	-0.40759	-1.70659
aPFL1345c_0	PFL1345c	0.839253	0.985753	0.803253	-0.40759	-1.70659
intPFL1355w_2	PFL1350w	0.318077	-0.17237	0.036257	0.240077	0.072707
aPFL1350w_1	PFL1350w	0.78659	0.78306	0.60612	-0.19312	-1.14106
aPFL1350w_0	PFL1350w	0.78659	0.78306	0.60612	-0.19312	-1.14106
aPFL1355w_0	PFL1355w	0.318077	-0.17237	0.036257	0.240077	0.072707
intPFL1365w_2	PFL1360c	-0.42669	-0.56151	0.608037	0.701797	-0.29415
aPFL1360c_1	PFL1360c	-0.47468	-0.89912	-0.06602	0.69952	0.72912
aPFL1360c_0	PFL1360c	-0.47468	-0.89912	-0.06602	0.69952	0.72912
intPFL1360c_2	PFL1365w	-0.47468	-0.89912	-0.06602	0.69952	0.72912
aPFL1365w_1	PFL1365w	-0.42669	-0.56151	0.608037	0.701797	-0.29415
aPFL1365w_0	PFL1365w	-0.42669	-0.56151	0.608037	0.701797	-0.29415
intPFL1370w_2	PFL1370w	-1.1411	-1.26648	-0.57653	0.595867	1.486817
aPFL1370w_0	PFL1370w	-1.1411	-1.26648	-0.57653	0.595867	1.486817
intPFL1375w_2	PFL1375w	0.827327	0.594367	0.282527	-0.28069	-0.81853
aPFL1375w_1	PFL1375w	0.827327	0.594367	0.282527	-0.28069	-0.81853
aPFL1375w_0	PFL1375w	0.827327	0.594367	0.282527	-0.28069	-0.81853
intPFL1380w_2	PFL1380w	-0.74056	-0.94166	-0.03117	0.526507	0.465997
aPFL1380w_1	PFL1380w	-0.74056	-0.94166	-0.03117	0.526507	0.465997
aPFL1380w_0	PFL1380w	-0.74056	-0.94166	-0.03117	0.526507	0.465997
intPFL1385c_2	PFL1385c	-1.1198	-1.73748	-2.84028	-0.68188	3.399317
aPFL1385c_1	PFL1385c	-1.1198	-1.73748	-2.84028	-0.68188	3.399317
aPFL1385c_0	PFL1385c	-1.1198	-1.73748	-2.84028	-0.68188	3.399317
intPFL1390w_2	PFL1390w	-0.41452	-0.67037	-0.78125	0.046087	1.192707
aPFL1390w_1	PFL1390w	-0.41452	-0.67037	-0.78125	0.046087	1.192707
aPFL1390w_0	PFL1390w	-0.41452	-0.67037	-0.78125	0.046087	1.192707
intPFL1395c_2	PFL1395c	-0.58108	-0.6604	-0.45434	0.25764	0.97285
aPFL1395c_4	PFL1395c	-0.58108	-0.6604	-0.45434	0.25764	0.97285
aPFL1395c_3	PFL1395c	-0.58108	-0.6604	-0.45434	0.25764	0.97285
aPFL1395c_2	PFL1395c	-0.58108	-0.6604	-0.45434	0.25764	0.97285
aPFL1395c_1	PFL1395c	-0.58108	-0.6604	-0.45434	0.25764	0.97285
aPFL1395c_0	PFL1395c	-0.58108	-0.6604	-0.45434	0.25764	0.97285
intPFL1405w_2	PFL1400c	0.947353	0.837713	0.346573	-0.37791	-0.95955
intPFL1400c_2	PFL1400c	0.894267	0.822657	0.462427	-0.22659	-0.93732
aPFL1400c_1	PFL1400c	0.894267	0.822657	0.462427	-0.22659	-0.93732
aPFL1400c_0	PFL1400c	0.894267	0.822657	0.462427	-0.22659	-0.93732
aPFL1405w_2	PFL1405w	0.947353	0.837713	0.346573	-0.37791	-0.95955
aPFL1405w_1	PFL1405w	0.947353	0.837713	0.346573	-0.37791	-0.95955
aPFL1405w_0	PFL1405w	0.947353	0.837713	0.346573	-0.37791	-0.95955
intPFL1410c_2	PFL1410c	1.383625	1.336605	0.492925	-0.92368	-1.85936
aPFL1410c_3	PFL1410c	1.383625	1.336605	0.492925	-0.92368	-1.85936
aPFL1410c_2	PFL1410c	1.383625	1.336605	0.492925	-0.92368	-1.85936
aPFL1410c_1	PFL1410c	1.383625	1.336605	0.492925	-0.92368	-1.85936
aPFL1410c_0	PFL1410c	1.383625	1.336605	0.492925	-0.92368	-1.85936
intPFL1415w_2	PFL1415w	-0.92411	-0.31044	-0.48086	-0.17414	0.72144
aPFL1415w_1	PFL1415w	-0.92411	-0.31044	-0.48086	-0.17414	0.72144

aPFL1415w_0	PFL1415w	-0.92411	-0.31044	-0.48086	-0.17414	0.72144
intPFL1420w_2	PFL1420w	-0.40464	-0.12541	0.25253	0.38127	0.16021
aPFL1420w_0	PFL1420w	-0.40464	-0.12541	0.25253	0.38127	0.16021
intPFL1425w_2	PFL1425w	-0.30031	0.32805	1.09615	0.63285	-0.74155
aPFL1425w_1	PFL1425w	-0.30031	0.32805	1.09615	0.63285	-0.74155
aPFL1425w_0	PFL1425w	-0.30031	0.32805	1.09615	0.63285	-0.74155
aPFL1430c_1	PFL1430c	-0.68653	-0.95883	-0.45158	0.364417	0.964167
aPFL1430c_0	PFL1430c	-0.68653	-0.95883	-0.45158	0.364417	0.964167
intPFL1430c_2	PFL1432w	-0.68653	-0.95883	-0.45158	0.364417	0.964167
intPFL1435c_2	PFL1435c	0.573872	-0.02004	-1.34377	-1.38979	0.174232
aPFL1435c_3	PFL1435c	0.573872	-0.02004	-1.34377	-1.38979	0.174232
aPFL1435c_2	PFL1435c	0.573872	-0.02004	-1.34377	-1.38979	0.174232
aPFL1435c_1	PFL1435c	0.573872	-0.02004	-1.34377	-1.38979	0.174232
aPFL1435c_0	PFL1435c	0.573872	-0.02004	-1.34377	-1.38979	0.174232
intPFL1445w_2	PFL1440c	0.183997	0.178667	0.025837	0.034167	0.061667
aPFL1440c_0	PFL1440c	0.244393	0.014033	-0.20407	-0.13427	0.100633
intPFL1440c_2	PFL1445w	0.244393	0.014033	-0.20407	-0.13427	0.100633
aPFL1445w_2	PFL1445w	0.183997	0.178667	0.025837	0.034167	0.061667
aPFL1445w_1	PFL1445w	0.183997	0.178667	0.025837	0.034167	0.061667
aPFL1445w_0	PFL1445w	0.183997	0.178667	0.025837	0.034167	0.061667
intPFL1450c_2	PFL1450c	0.293423	0.180393	-0.39805	-0.57429	-0.10923
aPFL1450c_1	PFL1450c	0.293423	0.180393	-0.39805	-0.57429	-0.10923
aPFL1450c_0	PFL1450c	0.293423	0.180393	-0.39805	-0.57429	-0.10923
intPFL1455w_2	PFL1455w	-1.19448	-0.2066	0.62519	0.82913	0.35794
aPFL1455w_1	PFL1455w	-1.19448	-0.2066	0.62519	0.82913	0.35794
aPFL1455w_0	PFL1455w	-1.19448	-0.2066	0.62519	0.82913	0.35794
intPFL1460c_2	PFL1460c	-0.25458	-0.03308	-0.06383	-0.12117	-0.00192
aPFL1460c_0	PFL1460c	-0.25458	-0.03308	-0.06383	-0.12117	-0.00192
intPFL1465c_2	PFL1465c	-0.78512	-0.52943	0.226833	0.687393	0.523973
aPFL1465c_0	PFL1465c	-0.78512	-0.52943	0.226833	0.687393	0.523973
intPFL1475w_2	PFL1470c	0.38448	0.31312	0.55851	0.18549	-0.66512
intPFL1470c_3	PFL1470c	1.223307	1.026467	0.815077	-0.35924	-1.75663
aPFL1470c_1	PFL1470c	1.223307	1.026467	0.815077	-0.35924	-1.75663
aPFL1470c_0	PFL1470c	1.223307	1.026467	0.815077	-0.35924	-1.75663
aPFL1475w_1	PFL1475w	0.38448	0.31312	0.55851	0.18549	-0.66512
aPFL1475w_0	PFL1475w	0.38448	0.31312	0.55851	0.18549	-0.66512
intPFL1480w_3	PFL1480w	0.083383	0.228603	-0.07371	-0.07413	0.148563
aPFL1480w_1	PFL1480w	0.083383	0.228603	-0.07371	-0.07413	0.148563
aPFL1480w_0	PFL1480w	0.083383	0.228603	-0.07371	-0.07413	0.148563
intPFL1485w_2	PFL1485w	0.285523	1.229863	0.390273	-0.50761	-0.7522
aPFL1485w_1	PFL1485w	0.285523	1.229863	0.390273	-0.50761	-0.7522
aPFL1485w_0	PFL1485w	0.285523	1.229863	0.390273	-0.50761	-0.7522
intPFL1490w_2	PFL1490w	1.214513	0.739453	0.505853	-0.62469	-1.77629
aPFL1490w_1	PFL1490w	1.214513	0.739453	0.505853	-0.62469	-1.77629
aPFL1490w_0	PFL1490w	1.214513	0.739453	0.505853	-0.62469	-1.77629
intPFL1495w_2	PFL1495w	0.336007	-0.16951	-0.71523	-0.42293	0.451847
aPFL1495w_1	PFL1495w	0.336007	-0.16951	-0.71523	-0.42293	0.451847

aPFL1495w_0	PFL1495w	0.336007	-0.16951	-0.71523	-0.42293	0.451847
intPFL1500w_2	PFL1500w	-0.49017	-0.01215	0.151793	0.165873	0.125813
aPFL1500w_0	PFL1500w	-0.49017	-0.01215	0.151793	0.165873	0.125813
intPFL1505c_2	PFL1505c	1.170543	0.830263	-0.74028	-1.78406	-1.1556
aPFL1505c_1	PFL1505c	1.170543	0.830263	-0.74028	-1.78406	-1.1556
aPFL1505c_0	PFL1505c	1.170543	0.830263	-0.74028	-1.78406	-1.1556
intPFL1510c_2	PFL1510c	0.683513	0.305913	-0.88782	-1.19302	-0.18575
aPFL1510c_1	PFL1510c	0.683513	0.305913	-0.88782	-1.19302	-0.18575
aPFL1510c_0	PFL1510c	0.683513	0.305913	-0.88782	-1.19302	-0.18575
intPFL1515c_2	PFL1515c	0.411393	0.124023	-0.3643	-0.79454	-0.57836
aPFL1515c_2	PFL1515c	0.411393	0.124023	-0.3643	-0.79454	-0.57836
aPFL1515c_1	PFL1515c	0.411393	0.124023	-0.3643	-0.79454	-0.57836
aPFL1515c_0	PFL1515c	0.411393	0.124023	-0.3643	-0.79454	-0.57836
intPFL1520w_2	PFL1520w	0.33818	0.20111	0.35279	-0.17979	-0.89511
aPFL1520w_0	PFL1520w	0.33818	0.20111	0.35279	-0.17979	-0.89511
intPFL1525c_2	PFL1525c	-0.26929	0.799433	0.276893	-0.17123	-0.14177
aPFL1525c_1	PFL1525c	-0.26929	0.799433	0.276893	-0.17123	-0.14177
aPFL1525c_0	PFL1525c	-0.26929	0.799433	0.276893	-0.17123	-0.14177
intPFL1530w_2	PFL1530w	0.351352	0.934572	0.192032	-0.32348	-0.33135
aPFL1530w_2	PFL1530w	0.351352	0.934572	0.192032	-0.32348	-0.33135
aPFL1530w_1	PFL1530w	0.351352	0.934572	0.192032	-0.32348	-0.33135
aPFL1530w_0	PFL1530w	0.351352	0.934572	0.192032	-0.32348	-0.33135
intPFL1535w_2	PFL1535w	-0.13062	0.216563	0.499373	0.267793	-0.3259
aPFL1535w_1	PFL1535w	-0.13062	0.216563	0.499373	0.267793	-0.3259
aPFL1535w_0	PFL1535w	-0.13062	0.216563	0.499373	0.267793	-0.3259
intPFL1540c_3	PFL1540c	-1.18281	-0.87798	0.306693	0.986973	0.727143
aPFL1540c_1	PFL1540c	-1.18281	-0.87798	0.306693	0.986973	0.727143
aPFL1540c_0	PFL1540c	-1.18281	-0.87798	0.306693	0.986973	0.727143
intPFL1545c_3	PFL1545c	-1.84431	-0.91998	0.714693	1.352973	0.720643
aPFL1545c_1	PFL1545c	-1.84431	-0.91998	0.714693	1.352973	0.720643
aPFL1545c_0	PFL1545c	-1.84431	-0.91998	0.714693	1.352973	0.720643
intPFL1550w_2	PFL1550w	-0.72249	-0.81456	-0.39538	0.403043	1.027223
aPFL1550w_1	PFL1550w	-0.72249	-0.81456	-0.39538	0.403043	1.027223
aPFL1550w_0	PFL1550w	-0.72249	-0.81456	-0.39538	0.403043	1.027223
intPFL1555w_2	PFL1555w	-0.00386	-0.0246	0.409143	0.308123	-0.30304
aPFL1555w_0	PFL1555w	-0.00386	-0.0246	0.409143	0.308123	-0.30304
intPFL1560c_2	PFL1560c	0.312363	0.130193	-0.09489	-0.27345	-0.25353
aPFL1560c_0	PFL1560c	0.312363	0.130193	-0.09489	-0.27345	-0.25353
aPFL1565c_1	PFL1565c	-0.011	-0.84998	-1.85079	-0.96587	1.381317
aPFL1565c_0	PFL1565c	-0.011	-0.84998	-1.85079	-0.96587	1.381317
intPFL1575w_2	PFL1570w	0.30889	-0.58444	-1.56061	-0.86139	1.07644
intPFL1570w_2	PFL1570w	0.384663	-0.7278	-0.64167	-0.44667	-0.10704
intPFL1565c_2	PFL1570w	-0.011	-0.84998	-1.85079	-0.96587	1.381317
aPFL1570w_1	PFL1570w	0.384663	-0.7278	-0.64167	-0.44667	-0.10704
aPFL1570w_0	PFL1570w	0.384663	-0.7278	-0.64167	-0.44667	-0.10704
intPFL1580w_2	PFL1575w	0.20739	-0.41647	-1.34055	-0.77995	0.94097
aPFL1575w_0	PFL1575w	0.30889	-0.58444	-1.56061	-0.86139	1.07644

aPFL1580w_1	PFL1580w	0.20739	-0.41647	-1.34055	-0.77995	0.94097
aPFL1580w_0	PFL1580w	0.20739	-0.41647	-1.34055	-0.77995	0.94097
intPFL1585c_2	PFL1585c	-0.24611	0.613567	1.181117	0.445717	-1.00573
intPFL1583c_2	PFL1585c					
aPFL1585c_1	PFL1585c	-0.24611	0.613567	1.181117	0.445717	-1.00573
aPFL1585c_0	PFL1585c	-0.24611	0.613567	1.181117	0.445717	-1.00573
aPFL1590c_1	PFL1590c	-0.1873	-0.04299	-0.02547	0.195128	0.373178
aPFL1590c_0	PFL1590c	-0.1873	-0.04299	-0.02547	0.195128	0.373178
intPFL1595w_2	PFL1595w	-3.65344	1.074203	-0.52291	-0.27743	2.476463
intPFL1590c_2	PFL1595w	-0.1873	-0.04299	-0.02547	0.195128	0.373178
aPFL1595w_0	PFL1595w	-3.65344	1.074203	-0.52291	-0.27743	2.476463
intPFL1600c_2	PFL1600c	0.171442	0.075552	-0.67198	-0.58794	0.343282
aPFL1600c_4	PFL1600c	0.171442	0.075552	-0.67198	-0.58794	0.343282
aPFL1600c_3	PFL1600c	0.171442	0.075552	-0.67198	-0.58794	0.343282
aPFL1600c_2	PFL1600c	0.171442	0.075552	-0.67198	-0.58794	0.343282
aPFL1600c_1	PFL1600c	0.171442	0.075552	-0.67198	-0.58794	0.343282
aPFL1600c_0	PFL1600c	0.171442	0.075552	-0.67198	-0.58794	0.343282
intPFL1605w_2	PFL1605w	-0.82225	-0.69078	0.586055	0.926055	0.128225
aPFL1605w_2	PFL1605w	-0.82225	-0.69078	0.586055	0.926055	0.128225
aPFL1605w_1	PFL1605w	-0.82225	-0.69078	0.586055	0.926055	0.128225
intPFL1610c_2	PFL1610c	0.637843	0.434293	0.461413	-0.00275	-0.77313
aPFL1610c_1	PFL1610c	0.637843	0.434293	0.461413	-0.00275	-0.77313
aPFL1610c_0	PFL1610c	0.637843	0.434293	0.461413	-0.00275	-0.77313
intPFL1620w_3	PFL1620w	0.223477	0.158777	-0.07671	-0.05867	0.083987
aPFL1620w_8	PFL1620w	0.223477	0.158777	-0.07671	-0.05867	0.083987
aPFL1620w_7	PFL1620w	0.223477	0.158777	-0.07671	-0.05867	0.083987
aPFL1620w_6	PFL1620w	0.223477	0.158777	-0.07671	-0.05867	0.083987
aPFL1620w_5	PFL1620w	0.223477	0.158777	-0.07671	-0.05867	0.083987
aPFL1620w_4	PFL1620w	0.223477	0.158777	-0.07671	-0.05867	0.083987
aPFL1620w_3	PFL1620w	0.223477	0.158777	-0.07671	-0.05867	0.083987
aPFL1620w_2	PFL1620w	0.223477	0.158777	-0.07671	-0.05867	0.083987
aPFL1620w_1	PFL1620w	0.223477	0.158777	-0.07671	-0.05867	0.083987
aPFL1620w_0	PFL1620w	0.223477	0.158777	-0.07671	-0.05867	0.083987
aPFL1615c_0	PFL1620w	-0.24172	-0.28446	0.045447	0.205147	0.115337
intPFL1625w_2	PFL1625w	0.547687	-0.38845	-0.37359	-0.07307	0.133787
aPFL1625w_1	PFL1625w	0.547687	-0.38845	-0.37359	-0.07307	0.133787
aPFL1625w_0	PFL1625w	0.547687	-0.38845	-0.37359	-0.07307	0.133787
aPFL1630c_0	PFL1630c					
intPFL1635w_2	PFL1635w	0.136667	0.029667	0.273167	0.266167	-0.12733
intPFL1630c_2	PFL1635w					
aPFL1635w_1	PFL1635w	0.136667	0.029667	0.273167	0.266167	-0.12733
aPFL1635w_0	PFL1635w	0.136667	0.029667	0.273167	0.266167	-0.12733
intPFL1645w_2	PFL1640c	0.110868	0.377328	0.018848	-0.15173	-0.04755
intPFL1640c_3	PFL1640c	-0.17223	-0.69586	0.49422	0.84028	-0.05214
aPFL1640c_1	PFL1640c	-0.17223	-0.69586	0.49422	0.84028	-0.05214
aPFL1640c_0	PFL1640c	-0.17223	-0.69586	0.49422	0.84028	-0.05214
aPFL1645w_5	PFL1645w	0.110868	0.377328	0.018848	-0.15173	-0.04755

aPFL1645w_4	PFL1645w	0.110868	0.377328	0.018848	-0.15173	-0.04755
aPFL1645w_3	PFL1645w	0.110868	0.377328	0.018848	-0.15173	-0.04755
aPFL1645w_2	PFL1645w	0.110868	0.377328	0.018848	-0.15173	-0.04755
aPFL1645w_1	PFL1645w	0.110868	0.377328	0.018848	-0.15173	-0.04755
aPFL1645w_0	PFL1645w	0.110868	0.377328	0.018848	-0.15173	-0.04755
intPFL1650w_2	PFL1650w	-0.69762	-0.24794	0.015205	0.342905	0.553715
aPFL1650w_3	PFL1650w	-0.69762	-0.24794	0.015205	0.342905	0.553715
aPFL1650w_2	PFL1650w	-0.69762	-0.24794	0.015205	0.342905	0.553715
aPFL1650w_1	PFL1650w	-0.69762	-0.24794	0.015205	0.342905	0.553715
aPFL1650w_0	PFL1650w	-0.69762	-0.24794	0.015205	0.342905	0.553715
aPFL1655c_1	PFL1655c	-0.80839	-0.95301	0.212037	0.987297	0.726347
aPFL1655c_0	PFL1655c	-0.80839	-0.95301	0.212037	0.987297	0.726347
intPFL1655c_2	PFL1660c	-0.80839	-0.95301	0.212037	0.987297	0.726347
aPFL1660c_1	PFL1660c	1.520323	0.213383	-0.70277	-0.70507	-0.13372
aPFL1660c_0	PFL1660c	1.520323	0.213383	-0.70277	-0.70507	-0.13372
intPFL1660c_2	PFL1665c	1.520323	0.213383	-0.70277	-0.70507	-0.13372
aPFL1665c_1	PFL1665c	0.89197	0.69816	0.22493	-0.29393	-0.68516
aPFL1665c_0	PFL1665c	0.89197	0.69816	0.22493	-0.29393	-0.68516
intPFL1670c_2	PFL1670c	-1.25272	-1.60238	-0.44424	0.90574	1.52238
intPFL1665c_2	PFL1670c	0.89197	0.69816	0.22493	-0.29393	-0.68516
aPFL1670c_1	PFL1670c	-1.25272	-1.60238	-0.44424	0.90574	1.52238
aPFL1670c_0	PFL1670c	-1.25272	-1.60238	-0.44424	0.90574	1.52238
intPFL1675c_2	PFL1675c	-0.32228	0.28042	-0.04109	-0.10241	0.21608
aPFL1675c_1	PFL1675c	-0.32228	0.28042	-0.04109	-0.10241	0.21608
aPFL1675c_0	PFL1675c	-0.32228	0.28042	-0.04109	-0.10241	0.21608
intPFL1680w_2	PFL1680w	0.092373	0.409123	0.687253	0.304253	-0.56163
aPFL1680w_1	PFL1680w	0.092373	0.409123	0.687253	0.304253	-0.56163
aPFL1680w_0	PFL1680w	0.092373	0.409123	0.687253	0.304253	-0.56163
intPFL1685w_2	PFL1685w	-1.52125	-2.01373	-0.61504	1.04104	1.85373
aPFL1685w_0	PFL1685w	-1.52125	-2.01373	-0.61504	1.04104	1.85373
intPFL1690w_2	PFL1690w	-0.99682	-0.25387	0.657247	0.660087	-0.05179
aPFL1690w_1	PFL1690w	-0.99682	-0.25387	0.657247	0.660087	-0.05179
aPFL1690w_0	PFL1690w	-0.99682	-0.25387	0.657247	0.660087	-0.05179
intPFL1695c_2	PFL1695c	-0.32188	-0.86257	-0.59735	0.139687	0.836907
aPFL1695c_0	PFL1695c	-0.32188	-0.86257	-0.59735	0.139687	0.836907
intPFL1700c_2	PFL1700c	-0.05499	-0.28301	-1.64623	-0.97093	1.391347
aPFL1700c_1	PFL1700c	-0.05499	-0.28301	-1.64623	-0.97093	1.391347
aPFL1700c_0	PFL1700c	-0.05499	-0.28301	-1.64623	-0.97093	1.391347
intPFL1705w_3	PFL1705w	0.54615	0.48258	-0.31449	-0.56062	-0.12469
aPFL1705w_2	PFL1705w	0.54615	0.48258	-0.31449	-0.56062	-0.12469
aPFL1705w_1	PFL1705w	0.54615	0.48258	-0.31449	-0.56062	-0.12469
aPFL1705w_0	PFL1705w	0.54615	0.48258	-0.31449	-0.56062	-0.12469
intPFL1715w_2	PFL1710c	-0.21527	0.341313	0.565633	0.261033	-0.34615
aPFL1710c_1	PFL1710c	-0.00333	0.456657	0.137437	-0.1826	-0.22032
aPFL1710c_0	PFL1710c	-0.00333	0.456657	0.137437	-0.1826	-0.22032
intPFL1710c_2	PFL1715w	-0.00333	0.456657	0.137437	-0.1826	-0.22032
aPFL1715w_1	PFL1715w	-0.21527	0.341313	0.565633	0.261033	-0.34615

aPFL1715w_0	PFL1715w	-0.21527	0.341313	0.565633	0.261033	-0.34615
intPFL1720w_2	PFL1720w	-1.28505	-1.86775	-0.5575	1.187	1.98575
aPFL1720w_1	PFL1720w	-1.28505	-1.86775	-0.5575	1.187	1.98575
aPFL1720w_0	PFL1720w	-1.28505	-1.86775	-0.5575	1.187	1.98575
intPFL1725w_2	PFL1725w	-1.05534	-1.25178	0.209817	1.184017	0.908617
aPFL1725w_1	PFL1725w	-1.05534	-1.25178	0.209817	1.184017	0.908617
aPFL1725w_0	PFL1725w	-1.05534	-1.25178	0.209817	1.184017	0.908617
aPFL1730c_3	PFL1730c	0.22852	0.2624	0.1742	-0.0452	-0.2884
aPFL1730c_2	PFL1730c	0.22852	0.2624	0.1742	-0.0452	-0.2884
aPFL1730c_1	PFL1730c	0.22852	0.2624	0.1742	-0.0452	-0.2884
aPFL1730c_0	PFL1730c	0.22852	0.2624	0.1742	-0.0452	-0.2884
intPFL1740w_2	PFL1735c	-1.24165	-0.73875	0.05674	0.79126	1.00425
intPFL1735c_2	PFL1735c	0.110057	0.238707	0.346597	0.199737	-0.21337
intPFL1730c_2	PFL1735c	0.22852	0.2624	0.1742	-0.0452	-0.2884
aPFL1735c_1	PFL1735c	0.110057	0.238707	0.346597	0.199737	-0.21337
aPFL1735c_0	PFL1735c	0.110057	0.238707	0.346597	0.199737	-0.21337
aPFL1740w_1	PFL1740w	-1.24165	-0.73875	0.05674	0.79126	1.00425
aPFL1740w_0	PFL1740w	-1.24165	-0.73875	0.05674	0.79126	1.00425
aPFL1745c_1	PFL1745c	-0.94251	0.62357	0.75936	0.40214	-0.05907
aPFL1745c_0	PFL1745c	-0.94251	0.62357	0.75936	0.40214	-0.05907
intPFL1750c_2	PFL1750c	-0.51016	0.099782	-0.23965	-0.22374	0.340332
aPFL1750c_2	PFL1750c	-0.51016	0.099782	-0.23965	-0.22374	0.340332
aPFL1750c_1	PFL1750c	-0.51016	0.099782	-0.23965	-0.22374	0.340332
aPFL1750c_0	PFL1750c	-0.51016	0.099782	-0.23965	-0.22374	0.340332
intPFL1760w_2	PFL1755w	0.31703	0.28535	-0.27145	-0.28905	0.14865
intPFL1755w_3	PFL1755w	-0.31246	-0.35118	-0.14914	0.17914	0.41218
aPFL1755w_1	PFL1755w	-0.31246	-0.35118	-0.14914	0.17914	0.41218
aPFL1755w_0	PFL1755w	-0.31246	-0.35118	-0.14914	0.17914	0.41218
aPFL1760w_1	PFL1760w	0.31703	0.28535	-0.27145	-0.28905	0.14865
aPFL1760w_0	PFL1760w	0.31703	0.28535	-0.27145	-0.28905	0.14865
intPFL1765c_2	PFL1765c					
aPFL1765c_0	PFL1765c					
intPFL1770c_2	PFL1770c	-0.25064	-0.62995	-0.97867	-0.23305	1.108232
aPFL1770c_0	PFL1770c	-0.25064	-0.62995	-0.97867	-0.23305	1.108232
intPFL1780w_2	PFL1775c	-0.39403	-0.41585	-0.8558	-0.19104	1.136513
intPFL1775c_2	PFL1775c	-0.87077	-0.11865	0.214547	0.349787	0.342487
aPFL1775c_1	PFL1775c	-0.87077	-0.11865	0.214547	0.349787	0.342487
aPFL1775c_0	PFL1775c	-0.87077	-0.11865	0.214547	0.349787	0.342487
aPFL1780w_1	PFL1780w	-0.39403	-0.41585	-0.8558	-0.19104	1.136513
aPFL1780w_0	PFL1780w	-0.39403	-0.41585	-0.8558	-0.19104	1.136513
intPFL1785c_2	PFL1785c	-0.01659	-0.87003	-1.37493	-0.56223	1.102867
aPFL1785c_1	PFL1785c	-0.01659	-0.87003	-1.37493	-0.56223	1.102867
aPFL1785c_0	PFL1785c	-0.01659	-0.87003	-1.37493	-0.56223	1.102867
intPFL1790w_2	PFL1790w	0.195047	-0.04325	0.253517	0.372817	0.000587
aPFL1790w_1	PFL1790w	0.195047	-0.04325	0.253517	0.372817	0.000587
aPFL1790w_0	PFL1790w	0.195047	-0.04325	0.253517	0.372817	0.000587
intPFL1800w_2	PFL1795c	0.570625	0.530535	0.755095	-0.16121	-1.46265

aPFL1795c_4	PFL1795c	0.607302	0.632232	0.684972	-0.03537	-1.08977
aPFL1795c_3	PFL1795c	0.607302	0.632232	0.684972	-0.03537	-1.08977
aPFL1795c_2	PFL1795c	0.607302	0.632232	0.684972	-0.03537	-1.08977
aPFL1795c_1	PFL1795c	0.607302	0.632232	0.684972	-0.03537	-1.08977
aPFL1795c_0	PFL1795c	0.607302	0.632232	0.684972	-0.03537	-1.08977
intPFL1795c_2	PFL1800w	0.607302	0.632232	0.684972	-0.03537	-1.08977
aPFL1800w_2	PFL1800w	0.570625	0.530535	0.755095	-0.16121	-1.46265
aPFL1800w_1	PFL1800w	0.570625	0.530535	0.755095	-0.16121	-1.46265
aPFL1800w_0	PFL1800w	0.570625	0.530535	0.755095	-0.16121	-1.46265
intPFL1810w_2	PFL1805c	0.00979	0.14704	-0.11608	-0.14942	0.08346
intPFL1805c_2	PFL1805c	-0.16938	-0.49954	0.367398	0.446138	-0.28092
aPFL1805c_0	PFL1805c	-0.16938	-0.49954	0.367398	0.446138	-0.28092
aPFL1810w_1	PFL1810w	0.00979	0.14704	-0.11608	-0.14942	0.08346
aPFL1810w_0	PFL1810w	0.00979	0.14704	-0.11608	-0.14942	0.08346
intPFL1815c_2	PFL1815c	0.345562	0.276522	0.080202	-0.14359	-0.29325
aPFL1815c_2	PFL1815c	0.345562	0.276522	0.080202	-0.14359	-0.29325
aPFL1815c_1	PFL1815c	0.345562	0.276522	0.080202	-0.14359	-0.29325
aPFL1815c_0	PFL1815c	0.345562	0.276522	0.080202	-0.14359	-0.29325
aPFL1820w_1	PFL1820w	0.702737	0.667297	0.517147	-0.26331	-1.13246
aPFL1820w_0	PFL1820w	0.702737	0.667297	0.517147	-0.26331	-1.13246
intPFL1825w_2	PFL1825w	-1.10463	-1.00805	-0.317	0.723103	1.361993
aPFL1825w_0	PFL1825w	-1.10463	-1.00805	-0.317	0.723103	1.361993
intPFL1830w_2	PFL1830w	0.71272	0.47439	0.28322	-0.12522	-0.62839
aPFL1830w_0	PFL1830w	0.71272	0.47439	0.28322	-0.12522	-0.62839
aPFL1835w_1	PFL1835w	1.014317	0.207397	-0.26528	-1.03238	-1.26206
aPFL1835w_0	PFL1835w	1.014317	0.207397	-0.26528	-1.03238	-1.26206
intPFL1840w_2	PFL1840w	-0.03564	0.218217	-0.92093	-0.74573	0.727117
aPFL1840w_0	PFL1840w	-0.03564	0.218217	-0.92093	-0.74573	0.727117
intPFL1845c_2	PFL1845c	-1.05723	-0.28083	1.22516	1.30984	-0.11617
aPFL1845c_0	PFL1845c	-1.05723	-0.28083	1.22516	1.30984	-0.11617
intPFL1850c_2	PFL1850c					
aPFL1850c_0	PFL1850c					
intPFL1855w_2	PFL1855w	0.74027	0.94965	0.2727	-0.4857	-0.84215
aPFL1855w_1	PFL1855w	0.74027	0.94965	0.2727	-0.4857	-0.84215
aPFL1855w_0	PFL1855w	0.74027	0.94965	0.2727	-0.4857	-0.84215
intPFL1865w_2	PFL1860c	-1.19859	-0.35976	0.448515	0.688375	0.369975
aPFL1860c_0	PFL1860c	0.233387	-0.41692	0.125847	0.318487	-0.10874
intPFL1860c_2	PFL1865w	0.233387	-0.41692	0.125847	0.318487	-0.10874
aPFL1865w_2	PFL1865w	-1.19859	-0.35976	0.448515	0.688375	0.369975
aPFL1865w_1	PFL1865w	-1.19859	-0.35976	0.448515	0.688375	0.369975
aPFL1865w_0	PFL1865w	-1.19859	-0.35976	0.448515	0.688375	0.369975
intPFL1870c_2	PFL1870c	-0.43944	-0.75533	-1.3741	-0.42174	1.529493
aPFL1870c_1	PFL1870c	-0.43944	-0.75533	-1.3741	-0.42174	1.529493
aPFL1870c_0	PFL1870c	-0.43944	-0.75533	-1.3741	-0.42174	1.529493
intPFL1875w_2	PFL1875w	-1.1873	-0.72702	0.68904	0.96546	0.09602
aPFL1875w_1	PFL1875w	-1.1873	-0.72702	0.68904	0.96546	0.09602
aPFL1875w_0	PFL1875w	-1.1873	-0.72702	0.68904	0.96546	0.09602

intPFL1880w_3	PFL1880w	0.475937	-0.01369	-0.29761	-0.38155	-0.19447
aPFL1880w_1	PFL1880w	0.475937	-0.01369	-0.29761	-0.38155	-0.19447
aPFL1880w_0	PFL1880w	0.475937	-0.01369	-0.29761	-0.38155	-0.19447
intPFL1885c_2	PFL1885c	0.74396	-0.25729	-0.43442	-1.11108	-1.28971
aPFL1885c_1	PFL1885c	0.74396	-0.25729	-0.43442	-1.11108	-1.28971
aPFL1885c_0	PFL1885c	0.74396	-0.25729	-0.43442	-1.11108	-1.28971
intPFL1890c_2	PFL1890c	0.167633	0.185343	0.047813	-0.29215	-0.45818
aPFL1890c_1	PFL1890c	0.167633	0.185343	0.047813	-0.29215	-0.45818
aPFL1890c_0	PFL1890c	0.167633	0.185343	0.047813	-0.29215	-0.45818
intPFL1895w_2	PFL1895w	-1.00955	-1.20924	0.52848	1.26452	0.45824
aPFL1895w_0	PFL1895w	-1.00955	-1.20924	0.52848	1.26452	0.45824
intPFL1900w_2	PFL1900w	-1.48028	-1.60537	-0.46714	0.971222	1.736452
aPFL1900w_3	PFL1900w	-1.48028	-1.60537	-0.46714	0.971222	1.736452
aPFL1900w_2	PFL1900w	-1.48028	-1.60537	-0.46714	0.971222	1.736452
aPFL1900w_1	PFL1900w	-1.48028	-1.60537	-0.46714	0.971222	1.736452
aPFL1900w_0	PFL1900w	-1.48028	-1.60537	-0.46714	0.971222	1.736452
intPFL1905w_2	PFL1905w	0.66527	1.05167	0.29116	-0.59216	-0.94267
aPFL1905w_0	PFL1905w	0.66527	1.05167	0.29116	-0.59216	-0.94267
intPFL1915w_2	PFL1910c	-1.07165	-0.73425	-0.23523	0.481067	1.020587
intPFL1910c_2	PFL1910c	-0.61897	-0.60416	0.178795	0.645655	0.444255
aPFL1910c_0	PFL1910c	-0.61897	-0.60416	0.178795	0.645655	0.444255
aPFL1915w_1	PFL1915w	-1.07165	-0.73425	-0.23523	0.481067	1.020587
aPFL1915w_0	PFL1915w	-1.07165	-0.73425	-0.23523	0.481067	1.020587
intPFL1925w_2	PFL1920c	-1.60926	-1.2157	0.25365	1.25485	1.1432
intPFL1920c_2	PFL1920c	-0.85973	-0.76035	0.5632	1.0388	0.30535
aPFL1920c_0	PFL1920c	-0.85973	-0.76035	0.5632	1.0388	0.30535
aPFL1925w_1	PFL1925w	-1.60926	-1.2157	0.25365	1.25485	1.1432
aPFL1925w_0	PFL1925w	-1.60926	-1.2157	0.25365	1.25485	1.1432
intPFL1930w_2	PFL1930w	-1.15916	-0.69255	-0.40619	0.364663	1.216363
aPFL1930w_8	PFL1930w	-1.15916	-0.69255	-0.40619	0.364663	1.216363
aPFL1930w_7	PFL1930w	-1.15916	-0.69255	-0.40619	0.364663	1.216363
aPFL1930w_6	PFL1930w	-1.15916	-0.69255	-0.40619	0.364663	1.216363
aPFL1930w_5	PFL1930w	-1.15916	-0.69255	-0.40619	0.364663	1.216363
aPFL1930w_4	PFL1930w	-1.15916	-0.69255	-0.40619	0.364663	1.216363
aPFL1930w_2	PFL1930w	-1.15916	-0.69255	-0.40619	0.364663	1.216363
aPFL1930w_1	PFL1930w	-1.15916	-0.69255	-0.40619	0.364663	1.216363
aPFL1930w_0	PFL1930w	-1.15916	-0.69255	-0.40619	0.364663	1.216363
intPFL1940w_2	PFL1935c	1.356123	1.142863	-0.26923	-1.17511	-0.9592
intPFL1935c_2	PFL1935c	1.656833	1.211853	0.004293	-0.77463	-0.91419
aPFL1935c_1	PFL1935c	1.656833	1.211853	0.004293	-0.77463	-0.91419
aPFL1935c_0	PFL1935c	1.656833	1.211853	0.004293	-0.77463	-0.91419
aPFL1940w_1	PFL1940w	1.356123	1.142863	-0.26923	-1.17511	-0.9592
aPFL1940w_0	PFL1940w	1.356123	1.142863	-0.26923	-1.17511	-0.9592
intPFL1945c_3	PFL1945c	2.28734	1.11729	-0.76059	-2.41741	-2.24929
aPFL1945c_0	PFL1945c	2.28734	1.11729	-0.76059	-2.41741	-2.24929
intPFL1975c_2	PFL1975c	0.663763	0.777193	0.030123	-0.50546	-0.51653
aPFL1975c_1	PFL1975c	0.663763	0.777193	0.030123	-0.50546	-0.51653

aPFL1975c_0	PFL1975c	0.663763	0.777193	0.030123	-0.50546	-0.51653
intPFL1980c_2	PFL1980c	-0.10304	0.373675	0.229015	0.073485	-0.02618
aPFL1980c_3	PFL1980c	-0.10304	0.373675	0.229015	0.073485	-0.02618
aPFL1980c_2	PFL1980c	-0.10304	0.373675	0.229015	0.073485	-0.02618
aPFL1980c_1	PFL1980c	-0.10304	0.373675	0.229015	0.073485	-0.02618
aPFL1980c_0	PFL1980c	-0.10304	0.373675	0.229015	0.073485	-0.02618
intPFL1985c_2	PFL1985c	-1.36534	-1.04834	0.299163	1.087503	0.839003
aPFL1985c_1	PFL1985c	-1.36534	-1.04834	0.299163	1.087503	0.839003
aPFL1985c_0	PFL1985c	-1.36534	-1.04834	0.299163	1.087503	0.839003
intPFL1990c_2	PFL1990c	-0.28493	-0.18284	-0.93498	-0.50602	0.928178
aPFL1990c_2	PFL1990c	-0.28493	-0.18284	-0.93498	-0.50602	0.928178
aPFL1990c_1	PFL1990c	-0.28493	-0.18284	-0.93498	-0.50602	0.928178
aPFL1990c_0	PFL1990c	-0.28493	-0.18284	-0.93498	-0.50602	0.928178
intPFL1995c_2	PFL1995c	-1.18488	0.328937	0.908127	0.720707	0.030397
aPFL1995c_1	PFL1995c	-1.18488	0.328937	0.908127	0.720707	0.030397
aPFL1995c_0	PFL1995c	-1.18488	0.328937	0.908127	0.720707	0.030397
intPFL2000w_2	PFL2000w	-1.34519	-0.90103	0.33306	1.09394	0.87803
aPFL2000w_0	PFL2000w	-1.34519	-0.90103	0.33306	1.09394	0.87803
intPFL2005w_2	PFL2005w	-1.12639	-0.88867	0.552912	1.196472	0.564052
aPFL2005w_0	PFL2005w	-1.12639	-0.88867	0.552912	1.196472	0.564052
intPFL2015w_2	PFL2010c	0.480947	0.321747	-0.32674	-0.47142	-0.05391
aPFL2010c_1	PFL2010c	0.766717	0.455407	0.675187	-0.06785	-1.29157
aPFL2010c_0	PFL2010c	0.766717	0.455407	0.675187	-0.06785	-1.29157
intPFL2010c_2	PFL2015w	0.766717	0.455407	0.675187	-0.06785	-1.29157
aPFL2015w_1	PFL2015w	0.480947	0.321747	-0.32674	-0.47142	-0.05391
aPFL2015w_0	PFL2015w	0.480947	0.321747	-0.32674	-0.47142	-0.05391
intPFL2020c_2	PFL2020c	0.283467	-0.17182	-0.79085	-0.25181	0.863157
aPFL2020c_1	PFL2020c	0.283467	-0.17182	-0.79085	-0.25181	0.863157
aPFL2020c_0	PFL2020c	0.283467	-0.17182	-0.79085	-0.25181	0.863157
intPFL2030w_2	PFL2025w	-0.32552	0.00858	0.26559	0.34541	0.14542
intPFL2025w_2	PFL2025w	0.00553	0.37834	0.16882	-0.01282	-0.07834
aPFL2025w_0	PFL2025w	0.00553	0.37834	0.16882	-0.01282	-0.07834
aPFL2030w_1	PFL2030w	-0.32552	0.00858	0.26559	0.34541	0.14542
aPFL2030w_0	PFL2030w	-0.32552	0.00858	0.26559	0.34541	0.14542
intPFL2040w_2	PFL2035c	-0.47371	-1.10144	-0.10037	0.78287	0.78194
aPFL2035c_0	PFL2035c	-0.83781	-1.05893	0.238367	1.051967	0.714267
intPFL2045w_2	PFL2040w	-0.30361	-0.86795	-0.16111	0.551773	0.645613
intPFL2035c_2	PFL2040w	-0.83781	-1.05893	0.238367	1.051967	0.714267
aPFL2040w_1	PFL2040w	-0.47371	-1.10144	-0.10037	0.78287	0.78194
aPFL2040w_0	PFL2040w	-0.47371	-1.10144	-0.10037	0.78287	0.78194
intPFL2050w_2	PFL2045w	0.506953	0.544713	0.607563	0.106103	-0.76055
aPFL2045w_1	PFL2045w	-0.30361	-0.86795	-0.16111	0.551773	0.645613
aPFL2045w_0	PFL2045w	-0.30361	-0.86795	-0.16111	0.551773	0.645613
aPFL2050w_1	PFL2050w	0.506953	0.544713	0.607563	0.106103	-0.76055
aPFL2050w_0	PFL2050w	0.506953	0.544713	0.607563	0.106103	-0.76055
intPFL2055w_2	PFL2055w	0.501843	0.261793	0.615413	0.333253	-0.62113
aPFL2055w_0	PFL2055w	0.501843	0.261793	0.615413	0.333253	-0.62113

intPFL2060c_3	PFL2060c	-0.00821	0.52003	-0.09856	-0.19244	0.24147
aPFL2060c_1	PFL2060c	-0.00821	0.52003	-0.09856	-0.19244	0.24147
aPFL2060c_0	PFL2060c	-0.00821	0.52003	-0.09856	-0.19244	0.24147
intPFL2065c_2	PFL2065c	-0.84572	-0.58141	0.576813	0.782853	0.011073
aPFL2065c_0	PFL2065c	-0.84572	-0.58141	0.576813	0.782853	0.011073
intPFL2070w_2	PFL2070w	-0.65229	-0.52356	-0.42585	0.218613	0.977023
aPFL2070w_0	PFL2070w	-0.65229	-0.52356	-0.42585	0.218613	0.977023
aPFL2075c_0	PFL2075c	0.441627	0.321847	0.371807	0.142527	-0.40051
intPFL2075c_2	PFL2078w	0.441627	0.321847	0.371807	0.142527	-0.40051
intPFL2080c_2	PFL2080c	0.564635	0.315545	-0.03138	-0.43177	-0.56798
aPFL2080c_0	PFL2080c	0.564635	0.315545	-0.03138	-0.43177	-0.56798
intPFL2085w_2	PFL2085w					
aPFL2085w_0	PFL2085w					
intPFL2090c_2	PFL2090c	-0.06484	-0.39781	-1.05537	-0.41163	1.07381
aPFL2090c_0	PFL2090c	-0.06484	-0.39781	-1.05537	-0.41163	1.07381
aPFL2095w_0	PFL2095w	0.32258	-0.0459	-0.38971	-0.46029	-0.1141
intPFL2100w_2	PFL2100w	0.65858	-0.26541	-1.40194	-1.53656	-0.13109
aPFL2100w_1	PFL2100w	0.65858	-0.26541	-1.40194	-1.53656	-0.13109
aPFL2100w_0	PFL2100w	0.65858	-0.26541	-1.40194	-1.53656	-0.13109
intPFL2105c_2	PFL2105c	1.140503	0.319983	0.193793	-0.51513	-1.29482
aPFL2105c_1	PFL2105c	1.140503	0.319983	0.193793	-0.51513	-1.29482
aPFL2105c_0	PFL2105c	1.140503	0.319983	0.193793	-0.51513	-1.29482
intPFL2110c_2	PFL2110c	-0.1284	-0.34632	-1.55519	-0.87525	1.350208
aPFL2110c_2	PFL2110c	-0.1284	-0.34632	-1.55519	-0.87525	1.350208
aPFL2110c_1	PFL2110c	-0.1284	-0.34632	-1.55519	-0.87525	1.350208
aPFL2110c_0	PFL2110c	-0.1284	-0.34632	-1.55519	-0.87525	1.350208
intPFL2115c_2	PFL2115c	-0.82845	-0.83425	-0.00975	0.63225	0.68625
aPFL2115c_1	PFL2115c	-0.82845	-0.83425	-0.00975	0.63225	0.68625
aPFL2115c_0	PFL2115c	-0.82845	-0.83425	-0.00975	0.63225	0.68625
intPFL2120w_3	PFL2120w	-0.55462	-0.18641	0.749563	0.322103	-0.83043
aPFL2120w_1	PFL2120w	-0.55462	-0.18641	0.749563	0.322103	-0.83043
aPFL2120w_0	PFL2120w	-0.55462	-0.18641	0.749563	0.322103	-0.83043
intPFL2125c_2	PFL2125c	0.504087	0.430047	0.386667	-0.01233	-0.60871
aPFL2125c_1	PFL2125c	0.504087	0.430047	0.386667	-0.01233	-0.60871
aPFL2125c_0	PFL2125c	0.504087	0.430047	0.386667	-0.01233	-0.60871
intPFL2130w_2	PFL2130w	1.32	0.49902	-0.43404	-0.74896	-0.43502
aPFL2130w_1	PFL2130w	1.32	0.49902	-0.43404	-0.74896	-0.43502
aPFL2130w_0	PFL2130w	1.32	0.49902	-0.43404	-0.74896	-0.43502
intPFL2135c_2	PFL2135c					
aPFL2135c_1	PFL2135c					
aPFL2135c_0	PFL2135c					
intPFL2140c_2	PFL2140c	0.63895	0.77726	-0.13202	-0.56098	-0.29426
aPFL2140c_0	PFL2140c	0.63895	0.77726	-0.13202	-0.56098	-0.29426
intPFL2145w_2	PFL2145w	0.994203	0.303003	-0.26102	-0.47232	-0.35934
aPFL2145w_0	PFL2145w	0.994203	0.303003	-0.26102	-0.47232	-0.35934
aPFL2150c_0	PFL2150c					
intPFL2155w_2	PFL2155w	0.474023	0.238883	-0.30377	-0.38557	-0.02022

intPFL2150c_2	PFL2155w					
aPFL2155w_1	PFL2155w	0.474023	0.238883	-0.30377	-0.38557	-0.02022
aPFL2155w_0	PFL2155w	0.474023	0.238883	-0.30377	-0.38557	-0.02022
intPFL2160c_2	PFL2160c	0.346033	0.459353	0.020043	-0.15888	-0.08419
aPFL2160c_1	PFL2160c	0.346033	0.459353	0.020043	-0.15888	-0.08419
aPFL2160c_0	PFL2160c	0.346033	0.459353	0.020043	-0.15888	-0.08419
intPFL2165w_2	PFL2165w	0.330833	-0.08118	-0.5874	-0.13644	0.707843
aPFL2165w_1	PFL2165w	0.330833	-0.08118	-0.5874	-0.13644	0.707843
aPFL2165w_0	PFL2165w	0.330833	-0.08118	-0.5874	-0.13644	0.707843
intPFL2175w_2	PFL2170c	0.26266	-0.20431	0.57913	0.54187	-0.47269
aPFL2170c_2	PFL2170c	-0.28618	-0.25944	0.558543	0.644453	-0.1349
aPFL2170c_1	PFL2170c	-0.28618	-0.25944	0.558543	0.644453	-0.1349
aPFL2170c_0	PFL2170c	-0.28618	-0.25944	0.558543	0.644453	-0.1349
intPFL2170c_2	PFL2175w	-0.28618	-0.25944	0.558543	0.644453	-0.1349
aPFL2175w_0	PFL2175w	0.26266	-0.20431	0.57913	0.54187	-0.47269
intPFL2180w_2	PFL2180w	0.315907	0.006457	0.214087	0.194247	-0.20212
aPFL2180w_0	PFL2180w	0.315907	0.006457	0.214087	0.194247	-0.20212
intPFL2185w_2	PFL2185w	-0.29377	-0.56715	-1.1377	-0.2313	1.45865
aPFL2185w_1	PFL2185w	-0.29377	-0.56715	-1.1377	-0.2313	1.45865
aPFL2185w_0	PFL2185w	-0.29377	-0.56715	-1.1377	-0.2313	1.45865
intPFL2190c_2	PFL2190c	-0.16631	0.01757	-0.05288	-0.14912	-0.05957
aPFL2190c_2	PFL2190c	-0.16631	0.01757	-0.05288	-0.14912	-0.05957
aPFL2190c_1	PFL2190c	-0.16631	0.01757	-0.05288	-0.14912	-0.05957
aPFL2190c_0	PFL2190c	-0.16631	0.01757	-0.05288	-0.14912	-0.05957
intPFL2195w_2	PFL2195w	0.675187	0.186447	0.269897	-0.38716	-1.17631
aPFL2195w_0	PFL2195w	0.675187	0.186447	0.269897	-0.38716	-1.17631
intPFL2205w_2	PFL2200w	1.027133	0.978843	-0.16144	-0.8329	-0.64318
intPFL2200w_2	PFL2200w	0.234377	0.892117	0.225267	-0.29093	-0.33278
aPFL2200w_0	PFL2200w	0.234377	0.892117	0.225267	-0.29093	-0.33278
aPFL2205w_1	PFL2205w	1.027133	0.978843	-0.16144	-0.8329	-0.64318
aPFL2205w_0	PFL2205w	1.027133	0.978843	-0.16144	-0.8329	-0.64318
intPFL2210w_2	PFL2210w	0.5874	0.55402	-0.01153	-0.40047	-0.40602
aPFL2210w_1	PFL2210w	0.5874	0.55402	-0.01153	-0.40047	-0.40602
aPFL2210w_0	PFL2210w	0.5874	0.55402	-0.01153	-0.40047	-0.40602
intPFL2215w_2	PFL2215w	-0.75041	-1.77745	-1.34911	0.00761	1.54995
aPFL2215w_1	PFL2215w	-0.75041	-1.77745	-1.34911	0.00761	1.54995
aPFL2215w_0	PFL2215w	-0.75041	-1.77745	-1.34911	0.00761	1.54995
intPFL2220w_2	PFL2220w	0.817263	1.373693	-0.87138	-1.74396	-0.37503
aPFL2220w_1	PFL2220w	0.817263	1.373693	-0.87138	-1.74396	-0.37503
aPFL2220w_0	PFL2220w	0.817263	1.373693	-0.87138	-1.74396	-0.37503
intPFL2225w_2	PFL2225w	0.731377	-0.44488	-1.58673	-1.58093	-0.00278
aPFL2225w_0	PFL2225w	0.731377	-0.44488	-1.58673	-1.58093	-0.00278
intPFL2235w_2	PFL2230c	1.103823	0.706393	-0.03729	-0.50805	-0.59073
aPFL2230c_1	PFL2230c	0.767147	0.824267	0.303217	-0.39388	-0.85293
aPFL2230c_0	PFL2230c	0.767147	0.824267	0.303217	-0.39388	-0.85293
intPFL2230c_2	PFL2235w	0.767147	0.824267	0.303217	-0.39388	-0.85293
aPFL2235w_1	PFL2235w	1.103823	0.706393	-0.03729	-0.50805	-0.59073

aPFL2235w_0	PFL2235w	1.103823	0.706393	-0.03729	-0.50805	-0.59073
intPFL2240w_2	PFL2240w	-1.12241	-1.05445	0.629667	1.273667	0.427287
aPFL2240w_1	PFL2240w	-1.12241	-1.05445	0.629667	1.273667	0.427287
aPFL2240w_0	PFL2240w	-1.12241	-1.05445	0.629667	1.273667	0.427287
intPFL2245w_3	PFL2245w	-1.63261	-1.24395	0.582393	1.318273	0.624613
aPFL2245w_0	PFL2245w	-1.63261	-1.24395	0.582393	1.318273	0.624613
intPFL2255w_2	PFL2250c	-0.60136	-0.89421	-0.65007	0.28407	1.22221
intPFL2250c_2	PFL2250c	-0.79024	-0.78481	0.06462	0.68838	0.65431
aPFL2250c_1	PFL2250c	-0.79024	-0.78481	0.06462	0.68838	0.65431
aPFL2250c_0	PFL2250c	-0.79024	-0.78481	0.06462	0.68838	0.65431
aPFL2255w_0	PFL2255w	-0.60136	-0.89421	-0.65007	0.28407	1.22221
intPFL2260w_2	PFL2260w	0.360083	0.295603	0.415793	0.068873	-0.58194
aPFL2260w_0	PFL2260w	0.360083	0.295603	0.415793	0.068873	-0.58194
intPFL2270w_2	PFL2265c	0.692393	0.029033	-0.00407	-0.09027	-0.33937
aPFL2265c_0	PFL2265c	0.416543	0.028303	0.365893	0.248773	-0.41764
intPFL2265c_2	PFL2270w	0.416543	0.028303	0.365893	0.248773	-0.41764
aPFL2270w_1	PFL2270w	0.692393	0.029033	-0.00407	-0.09027	-0.33937
aPFL2270w_0	PFL2270w	0.692393	0.029033	-0.00407	-0.09027	-0.33937
intPFL2275c_2	PFL2275c	-1.25097	-0.31316	0.99032	1.03968	-0.04784
aPFL2275c_0	PFL2275c	-1.25097	-0.31316	0.99032	1.03968	-0.04784
intPFL2280w_2	PFL2280w	-0.63727	-1.00467	-0.78241	0.05941	1.06617
aPFL2280w_1	PFL2280w	-0.63727	-1.00467	-0.78241	0.05941	1.06617
aPFL2280w_0	PFL2280w	-0.63727	-1.00467	-0.78241	0.05941	1.06617
intPFL2285c_3	PFL2285c	-0.11352	-0.51189	-0.76721	-0.48895	0.369727
aPFL2285c_1	PFL2285c	-0.11352	-0.51189	-0.76721	-0.48895	0.369727
aPFL2285c_0	PFL2285c	-0.11352	-0.51189	-0.76721	-0.48895	0.369727
intPFL2290w_2	PFL2290w	0.143697	-0.06447	-1.72055	-1.41211	0.945307
aPFL2290w_1	PFL2290w	0.143697	-0.06447	-1.72055	-1.41211	0.945307
aPFL2290w_0	PFL2290w	0.143697	-0.06447	-1.72055	-1.41211	0.945307
intPFL2300w_2	PFL2295w	-0.16233	-0.29638	-0.71176	-0.13158	0.964043
intPFL2295w_2	PFL2295w	0.883263	1.724673	0.812663	-0.739	-1.74301
aPFL2295w_0	PFL2295w	0.883263	1.724673	0.812663	-0.739	-1.74301
intPFL2305w_2	PFL2300w	0.766777	0.412607	0.038777	-0.23544	-0.40477
aPFL2300w_0	PFL2300w	-0.16233	-0.29638	-0.71176	-0.13158	0.964043
aPFL2305w_1	PFL2305w	0.766777	0.412607	0.038777	-0.23544	-0.40477
aPFL2305w_0	PFL2305w	0.766777	0.412607	0.038777	-0.23544	-0.40477
intPFL2310w_2	PFL2310w	0.43278	0.65711	0.35428	-0.27028	-0.76111
aPFL2310w_1	PFL2310w	0.43278	0.65711	0.35428	-0.27028	-0.76111
aPFL2310w_0	PFL2310w	0.43278	0.65711	0.35428	-0.27028	-0.76111
intPFL2315c_2	PFL2315c	-0.97409	-0.87731	0.61773	0.97501	0.06983
aPFL2315c_0	PFL2315c	-0.97409	-0.87731	0.61773	0.97501	0.06983
intPFL2320w_2	PFL2320w	-0.11411	-0.24349	-0.05504	0.227703	0.318153
aPFL2320w_0	PFL2320w	-0.11411	-0.24349	-0.05504	0.227703	0.318153
intPFL2325c_3	PFL2325c	-0.23549	-0.48554	0.25508	0.39692	-0.11746
aPFL2325c_0	PFL2325c	-0.23549	-0.48554	0.25508	0.39692	-0.11746
intPFL2330w_2	PFL2330w	-0.39322	-0.67489	-0.60122	0.186557	1.063227
aPFL2330w_1	PFL2330w	-0.39322	-0.67489	-0.60122	0.186557	1.063227

aPFL2330w_0	PFL2330w	-0.39322	-0.67489	-0.60122	0.186557	1.063227
intPFL2335w_2	PFL2335w	0.2667	0.09917	-0.41226	-0.34257	0.21916
aPFL2335w_2	PFL2335w	0.2667	0.09917	-0.41226	-0.34257	0.21916
aPFL2335w_1	PFL2335w	0.2667	0.09917	-0.41226	-0.34257	0.21916
aPFL2335w_0	PFL2335w	0.2667	0.09917	-0.41226	-0.34257	0.21916
intPFL2340w_2	PFL2340w	0.651805	1.289675	0.358495	-0.60827	-0.92714
aPFL2340w_0	PFL2340w	0.651805	1.289675	0.358495	-0.60827	-0.92714
intPFL2345c_2	PFL2345c	-0.68471	-0.41846	-0.09808	0.37558	0.69246
aPFL2345c_1	PFL2345c	-0.68471	-0.41846	-0.09808	0.37558	0.69246
aPFL2345c_0	PFL2345c	-0.68471	-0.41846	-0.09808	0.37558	0.69246
aPFL2350c_1	PFL2350c	0.991003	1.071463	0.922583	-0.16992	-1.5623
aPFL2350c_0	PFL2350c	0.991003	1.071463	0.922583	-0.16992	-1.5623
intPFL2355w_3	PFL2355w	0.13809	0.28268	0.87674	0.416	-0.84094
intPFL2350c_2	PFL2355w	0.991003	1.071463	0.922583	-0.16992	-1.5623
aPFL2355w_1	PFL2355w	0.13809	0.28268	0.87674	0.416	-0.84094
aPFL2355w_0	PFL2355w	0.13809	0.28268	0.87674	0.416	-0.84094
intPFL2360w_2	PFL2360w	0.405407	-0.04404	-0.70541	-0.28825	0.696377
aPFL2360w_0	PFL2360w	0.405407	-0.04404	-0.70541	-0.28825	0.696377
intPFL2365w_2	PFL2365w	-0.06852	0.077607	-0.03971	0.117547	0.323727
aPFL2365w_1	PFL2365w	-0.06852	0.077607	-0.03971	0.117547	0.323727
aPFL2365w_0	PFL2365w	-0.06852	0.077607	-0.03971	0.117547	0.323727
aPFL2370c_0	PFL2370c	-0.51698	-0.42812	-0.30126	0.20726	0.75012
intPFL2375c_2	PFL2375c	-0.5248	-0.97997	-0.37505	0.587387	1.090307
intPFL2370c_2	PFL2375c	-0.51698	-0.42812	-0.30126	0.20726	0.75012
aPFL2375c_0	PFL2375c	-0.5248	-0.97997	-0.37505	0.587387	1.090307
intPFL2380c_2	PFL2380c	0.021997	-0.19349	-0.38277	-0.05139	0.488327
aPFL2380c_1	PFL2380c	0.021997	-0.19349	-0.38277	-0.05139	0.488327
aPFL2380c_0	PFL2380c	0.021997	-0.19349	-0.38277	-0.05139	0.488327
intPFL2385c_2	PFL2385c	-0.43576	-0.16468	-0.19064	-0.11786	0.20818
aPFL2385c_1	PFL2385c	-0.43576	-0.16468	-0.19064	-0.11786	0.20818
aPFL2385c_0	PFL2385c	-0.43576	-0.16468	-0.19064	-0.11786	0.20818
intPFL2390c_2	PFL2390c	-0.15513	-0.04292	0.127645	0.147855	0.010865
aPFL2390c_4	PFL2390c	-0.15513	-0.04292	0.127645	0.147855	0.010865
aPFL2390c_3	PFL2390c	-0.15513	-0.04292	0.127645	0.147855	0.010865
aPFL2390c_2	PFL2390c	-0.15513	-0.04292	0.127645	0.147855	0.010865
aPFL2390c_1	PFL2390c	-0.15513	-0.04292	0.127645	0.147855	0.010865
aPFL2390c_0	PFL2390c	-0.15513	-0.04292	0.127645	0.147855	0.010865
aPFL2395c_1	PFL2395c	0.14836	0.0292	0.36059	0.28391	-0.2652
aPFL2395c_0	PFL2395c	0.14836	0.0292	0.36059	0.28391	-0.2652
intPFL2400w_3	PFL2400w	0.947687	0.373587	0.575827	0.049507	-1.04825
intPFL2395c_2	PFL2400w	0.14836	0.0292	0.36059	0.28391	-0.2652
aPFL2400w_0	PFL2400w	0.947687	0.373587	0.575827	0.049507	-1.04825
intPFL2405c_2	PFL2405c	0.9373	0.16007	-0.53242	-0.69862	-0.27755
aPFL2405c_4	PFL2405c	0.9373	0.16007	-0.53242	-0.69862	-0.27755
aPFL2405c_3	PFL2405c	0.9373	0.16007	-0.53242	-0.69862	-0.27755
aPFL2405c_2	PFL2405c	0.9373	0.16007	-0.53242	-0.69862	-0.27755
aPFL2405c_1	PFL2405c	0.9373	0.16007	-0.53242	-0.69862	-0.27755

aPFL2405c_0	PFL2405c	0.9373	0.16007	-0.53242	-0.69862	-0.27755
intPFL2410w_2	PFL2410w	0.109197	0.000527	-0.0178	-0.46686	-0.70369
aPFL2410w_1	PFL2410w	0.109197	0.000527	-0.0178	-0.46686	-0.70369
aPFL2410w_0	PFL2410w	0.109197	0.000527	-0.0178	-0.46686	-0.70369
intPFL2415w_2	PFL2415w	-1.29229	-1.22955	0.194607	1.131727	0.951887
aPFL2415w_0	PFL2415w	-1.29229	-1.22955	0.194607	1.131727	0.951887
intPFL2425w_2	PFL2420w	-0.44158	-0.1991	0.32471	0.48729	0.1501
intPFL2420w_2	PFL2420w	-0.13878	0.87241	0.08693	-0.31193	0.00059
aPFL2420w_1	PFL2420w	-0.13878	0.87241	0.08693	-0.31193	0.00059
aPFL2420w_0	PFL2420w	-0.13878	0.87241	0.08693	-0.31193	0.00059
aPFL2425w_0	PFL2425w	-0.44158	-0.1991	0.32471	0.48729	0.1501
intPFL2430c_2	PFL2430c	0.98187	0.93413	-0.02427	-0.63073	-0.60613
aPFL2430c_1	PFL2430c	0.98187	0.93413	-0.02427	-0.63073	-0.60613
aPFL2430c_0	PFL2430c	0.98187	0.93413	-0.02427	-0.63073	-0.60613
intPFL2435w_2	PFL2435w	-0.37855	-0.02711	0.410652	0.419262	-0.01585
aPFL2435w_0	PFL2435w	-0.37855	-0.02711	0.410652	0.419262	-0.01585
intPFL2440w_2	PFL2440w	0.001792	0.118382	0.170562	0.169772	0.020282
aPFL2440w_2	PFL2440w	0.001792	0.118382	0.170562	0.169772	0.020282
aPFL2440w_1	PFL2440w	0.001792	0.118382	0.170562	0.169772	0.020282
aPFL2440w_0	PFL2440w	0.001792	0.118382	0.170562	0.169772	0.020282
intPFL2445c_2	PFL2445c	-0.09692	-0.02642	0.27233	0.33867	0.02342
aPFL2445c_0	PFL2445c	-0.09692	-0.02642	0.27233	0.33867	0.02342
intPFL2450c_2	PFL2450c	1.109623	0.369903	-0.15181	-0.63953	-0.80424
aPFL2450c_0	PFL2450c	1.109623	0.369903	-0.15181	-0.63953	-0.80424
intPFL2455w_2	PFL2455w	0.250082	0.414612	0.104182	-0.26172	-0.39054
aPFL2455w_0	PFL2455w	0.250082	0.414612	0.104182	-0.26172	-0.39054
intPFL2460w_2	PFL2460w	1.22466	-0.12631	-2.09662	-2.38238	-0.22219
aPFL2460w_1	PFL2460w	1.22466	-0.12631	-2.09662	-2.38238	-0.22219
aPFL2460w_0	PFL2460w	1.22466	-0.12631	-2.09662	-2.38238	-0.22219
aPFL2465c_0	PFL2465c	-0.97101	-1.73698	-0.08954	1.197203	1.125643
intPFL2475w_2	PFL2470c	1.05586	1.0382	0.69484	-0.49534	-1.6767
intPFL2465c_2	PFL2470c	-0.97101	-1.73698	-0.08954	1.197203	1.125643
aPFL2470c_1	PFL2470c	0.656977	0.230627	0.461497	0.116337	-0.73679
aPFL2470c_0	PFL2470c	0.656977	0.230627	0.461497	0.116337	-0.73679
intPFL2470c_2	PFL2475w	0.656977	0.230627	0.461497	0.116337	-0.73679
aPFL2475w_1	PFL2475w	1.05586	1.0382	0.69484	-0.49534	-1.6767
aPFL2475w_0	PFL2475w	1.05586	1.0382	0.69484	-0.49534	-1.6767
intPFL2480w_2	PFL2480w	1.31726	0.4397	0.25185	-0.35435	-1.1392
aPFL2480w_1	PFL2480w	1.31726	0.4397	0.25185	-0.35435	-1.1392
aPFL2480w_0	PFL2480w	1.31726	0.4397	0.25185	-0.35435	-1.1392
aPFL2485c_1	PFL2485c	-0.43936	0.34528	0.89945	0.45005	-0.59728
aPFL2485c_0	PFL2485c	-0.43936	0.34528	0.89945	0.45005	-0.59728
intPFL2490c_2	PFL2490c	0.471657	0.280697	0.056857	-0.16002	-0.31436
intPFL2485c_2	PFL2490c	-0.43936	0.34528	0.89945	0.45005	-0.59728
aPFL2490c_1	PFL2490c	0.471657	0.280697	0.056857	-0.16002	-0.31436
aPFL2490c_0	PFL2490c	0.471657	0.280697	0.056857	-0.16002	-0.31436
intPFL2505c_2	PFL2505c	-1.05548	-1.02907	-2.54085	-1.01206	2.805907

aPFL2505c_3	PFL2505c	-1.05548	-1.02907	-2.54085	-1.01206	2.805907
aPFL2505c_2	PFL2505c	-1.05548	-1.02907	-2.54085	-1.01206	2.805907
aPFL2505c_1	PFL2505c	-1.05548	-1.02907	-2.54085	-1.01206	2.805907
aPFL2505c_0	PFL2505c	-1.05548	-1.02907	-2.54085	-1.01206	2.805907
intPFL2510w_2	PFL2510w	0.91681	0.46293	-0.33812	-0.66488	-0.37443
aPFL2510w_1	PFL2510w	0.91681	0.46293	-0.33812	-0.66488	-0.37443
aPFL2510w_0	PFL2510w	0.91681	0.46293	-0.33812	-0.66488	-0.37443
intPFL2515c_3	PFL2515c	-1.19865	0.973233	2.059033	0.936633	-1.32157
aPFL2515c_0	PFL2515c	-1.19865	0.973233	2.059033	0.936633	-1.32157
intPFL2520w_2	PFL2520w	1.694758	0.391578	-1.68672	-2.56561	-1.05996
aPFL2520w_4	PFL2520w	1.694758	0.391578	-1.68672	-2.56561	-1.05996
aPFL2520w_3	PFL2520w	1.694758	0.391578	-1.68672	-2.56561	-1.05996
aPFL2520w_2	PFL2520w	1.694758	0.391578	-1.68672	-2.56561	-1.05996
aPFL2520w_1	PFL2520w	1.694758	0.391578	-1.68672	-2.56561	-1.05996
aPFL2520w_0	PFL2520w	1.694758	0.391578	-1.68672	-2.56561	-1.05996
intPFL2525c_2	PFL2525c	1.447927	0.874867	-0.50748	-1.03568	-0.52253
aPFL2525c_1	PFL2525c	1.447927	0.874867	-0.50748	-1.03568	-0.52253
aPFL2525c_0	PFL2525c	1.447927	0.874867	-0.50748	-1.03568	-0.52253
intPFL2530w_2	PFL2530w	1.88488	0.7616	-0.66419	-1.38981	-0.9876
aPFL2530w_1	PFL2530w	1.88488	0.7616	-0.66419	-1.38981	-0.9876
aPFL2530w_0	PFL2530w	1.88488	0.7616	-0.66419	-1.38981	-0.9876
intPFL2535w_2	PFL2535w	-0.41611	-0.59098	0.056203	0.548963	0.465143
aPFL2535w_1	PFL2535w	-0.41611	-0.59098	0.056203	0.548963	0.465143
aPFL2535w_0	PFL2535w	-0.41611	-0.59098	0.056203	0.548963	0.465143
intPFL2540w_3	PFL2540w	0.402697	-0.1845	0.317757	-0.08292	-0.96416
aPFL2540w_1	PFL2540w	0.402697	-0.1845	0.317757	-0.08292	-0.96416
aPFL2540w_0	PFL2540w	0.402697	-0.1845	0.317757	-0.08292	-0.96416
intPFL2545c_2	PFL2545c	2.62687	0.95363	-0.51277	-1.94123	-2.21163
aPFL2545c_0	PFL2545c	2.62687	0.95363	-0.51277	-1.94123	-2.21163
intPFL2550w_2	PFL2550w	1.84537	1.30113	-0.16952	-1.48848	-1.66963
aPFL2550w_1	PFL2550w	1.84537	1.30113	-0.16952	-1.48848	-1.66963
aPFL2550w_0	PFL2550w	1.84537	1.30113	-0.16952	-1.48848	-1.66963
intPFL2555w_2	PFL2555w	1.281227	1.426847	0.419297	-1.00396	-1.75051
aPFL2555w_0	PFL2555w	1.281227	1.426847	0.419297	-1.00396	-1.75051
intPFL2565w_2	PFL2560c	2.315483	1.017563	-0.18212	-1.72022	-2.3399
aPFL2560c_0	PFL2560c					
intPFL2560c_2	PFL2565w					
aPFL2565w_0	PFL2565w	2.315483	1.017563	-0.18212	-1.72022	-2.3399
intPFL2570w_2	PFL2570w	-0.42181	-0.11049	-0.79979	-0.23905	1.174653
intPFL2575c_2	PFL2575c	-2.30022	-0.3479	1.537128	1.415688	-0.15973
aPFL2580w_0	PFL2580w	0.669157	0.171207	0.195587	-0.02825	-0.50987
intPFL2590w_2	PFL2590w	0.37654	0.25732	-0.16564	-0.17536	0.08668
intPFL2595w_2	PFL2595w					
intPFL2650w_2	PFL2650w	0.212857	0.073737	0.328537	0.003797	-0.6184
aMAL8P1.330_0	RzID:4480	1.232123	0.171383	-0.89677	-1.40557	-0.76072
aPFC1125w_1	RzID:4493	1.644977	-0.26886	-0.77077	-0.88889	-0.6478
aPFC0002c_0	RzID:4493	1.707053	0.658743	-0.84849	-1.43785	-0.73108

aPFA0002w_1	RzID:4493		-0.43633	-0.39138	0.167243	0.521423	0.360043
aMAL8P1.335_0	RzID:4493		1.13827	-0.14305	-0.18324	-0.41132	-0.75583

RNA_TP6	H3K4me3_	H3K4me3_	H3K4me3_	H3K4me3_	H3K4me3_	H3K4me3_	H3K4me3_	H3K4me3_
-0.38492	5.192867	5.313377	3.784142	4.557723	3.19393	3.27617	0.001825	2.119447
-0.54927								
0.065012	0.294609	0.423981	0.575627	0.315372	0.143161	0.385763	0.052352	0.432466
-0.11706	3.58117	2.711618	1.519957	2.419633	2.230585	1.825003	0.031029	2.061213
-0.68991	0.260974	0.221797	0.128207	0.075465	-0.25081	-0.1152	0.014556	0.511788
-0.97728								
1.01179	-1.65209	-2.00595	-2.62835	-2.59526	-2.49353	-2.47355	0.00104	0.97626
1.01179	-1.97608	-2.21834	-2.99316	-3.00898	-2.888	-2.60112	0.008471	1.032907
1.36845	-1.60981	-2.11369	-2.73935	-2.23319	-2.67643	-2.53626	0.009692	1.129543
1.36845	-1.95862	-2.16052	-2.79199	-2.78778	-2.98865	-2.81915	0.001844	1.030025
1.048313	-1.59118	-2.16555	-2.62191	-2.68134	-2.62621	-2.61629	0.025512	1.090163
1.048313	-2.01328	-2.33989	-3.05191	-3.07222	-2.93233	-2.6288	0.000706	1.058943
0.436083	-0.22519	-0.83977	-1.29187	-0.52793	-1.25236	-1.03526	0.00669	1.066683
0.436083	2.815987	1.924707	0.963527	1.652717	3.26307	1.326132	0.04085	2.299543
0.436083	-0.73716	-1.28079	-1.56253	-0.81474	-1.26026	-1.1224	0.012108	0.825374
0.062673	-0.48478	-0.89252	-1.2889	-0.75553	-1.42213	-1.09331	0.002514	0.937351
-0.07745								
-1.17764	6.64051	6.625765	5.542615	5.840953	5.378955	5.313997	0.007166	1.326513
0.340533	4.090477	3.837087	2.75859	3.508323	2.68794	2.6245	0.001525	1.465977
0.336787	4.247217	3.639187	2.797967	3.278417	2.68535	2.772293	0.03348	1.561867
-0.18916	5.5136	5.46072	4.367387	4.571377	3.97751	4.063383	0.005538	1.53609
-0.12748								
-0.25982	4.809477	4.72264	3.577807	4.21493	3.50797	3.42117	0.004221	1.388307
2.262227	0.602266	0.62888	0.625022	0.323264	0.816533	0.910532	0.010504	0.587268
-0.12661	2.86138	2.84227	1.935857	2.905263	1.835	1.837433	0.052889	1.070263
-0.31366	0.787677	1.299327	0.906073	1.243713	1.443135	1.364807	0.124029	0.655458
-0.67436	5.4416	5.48932	4.588257	4.738173	4.4537	4.21104	0.003703	1.27828
-0.55794	5.321723	5.576527	3.856733	4.741367	3.73721	3.893513	0.003687	1.839317
0.341097	-0.12651	-0.09707	-0.26402	-0.18521	0.121106	0.103169	0.048718	0.385127
0.341097	0.455269	0.805034	0.876056	0.528961	1.250035	0.994307	0.009698	0.794766
2.262833								
2.262833	-0.77054	-1.02997	-1.04287	-0.63471	-0.57601	-0.57352	0.009063	0.469352
2.262833	-0.89734	-1.3557	-1.50054	-0.85855	-1.02594	-0.82675	0.001542	0.673792
0.902373								
0.902373	-1.10306	-1.66624	-2.01986	-1.25166	-2.45667	-1.73945	0.0179	1.353613
0.902373	0.96505	1.0371	1.155271	1.1627	1.35254	1.147293	0.004557	0.38749
0.09319	1.019584	0.8814	0.952305	0.840927	1.214181	1.000763	0.156594	0.373254
0.09319	2.928197	2.786347	2.317607	2.311627	1.69985	2.038413	0.089912	1.228347
0.09319	0.520554	0.747739	0.373433	0.419367	1.025868	0.853895	0.015753	0.652434
-0.3565	0.130007	0.931777	0.650994	0.497076	1.254165	0.889718	0.049933	1.124158
-0.3565	0.261503	0.474661	0.134278	0.445051	0.61755	0.354936	0.058253	0.483272
-0.4069	1.450047	1.239939	0.986463	0.939982	0.812518	0.753734	0.023681	0.696313
-0.4069	0.744469	0.811981	0.76655	1.108603	1.4256	1.037872	0.016392	0.681131
-0.4069	0.524265	0.488531	0.166267	0.869564	1.460175	0.951169	0.060374	1.293908
-0.4069	-1.5435	-2.05128	-2.05583	-1.22548	-1.46004	-1.37061	0.033085	0.83035

	7.423293	7.691033	6.45391	6.533425	6.80584	6.696043	0.02298	1.237123
-0.10836	0.6274	0.814069	1.148532	0.572645	1.002651	1.091909	0.017688	0.575887
0.133487	0.469917	0.230897	0.036247	0.238246	0.52749	0.597733	0.035364	0.561486
0.364757	-0.83712	-0.78857	-0.72036	-0.79655	-0.69073	-0.61379	0.083066	0.223329
0.364757	1.219263	1.194157	1.176093	0.651631	0.968829	1.399627	0.000646	0.747996
-0.19289	0.046169	0.138263	0.816827	0.497051	0.25043	0.230627	0.027148	0.770658
-0.62915	0.943178	1.478313	1.54254	0.765072	1.28011	1.113307	0.004271	0.777468
2.1446	0.447135	0.59307	0.675321	0.313282	0.300144	0.416459	0.029595	0.375177
2.1446	-1.40163	-1.67625	-1.91782	-1.53783	-1.6003	-1.40424	0.002249	0.516183
2.1446	-1.45433	-2.07904	-2.22233	-1.41426	-1.63091	-1.55603	0.001575	0.808065
2.1446	-1.26659	-2.03001	-2.17326	-1.42475	-1.81918	-1.54772	0.004125	0.906672
2.1446	-1.19872	-1.90872	-2.22406	-1.17405	-1.75055	-1.31607	5.83E-05	1.050013
2.1446	-1.08217	-1.55506	-1.74047	-1.14785	-1.27588	-1.0182	0.002672	0.722269
2.1446	-0.56094	-0.80438	-0.39272	-0.14399	-0.30516	-0.44172	0.006654	0.660391
0.480853	0.276166	0.476746	0.504878	0.13996	0.845331	0.939063	0.008532	0.799103
0.480853	0.149235	-0.08685	-0.08823	-0.15044	0.007191	-0.02749	0.047459	0.299678
-0.23087								
-0.23087	-0.25755	-0.15706	-0.82509	0.183813	-0.93287	-0.3556	0.24839	1.116687
-0.23087	-0.83272	-1.58299	-1.59601	-0.75695	-1.05191	-0.86292	0.000277	0.839058
-0.23087	-0.62834	-1.13866	-1.15561	-0.76359	-1.03373	-0.69582	0.03515	0.527263
-0.23087	0.803805	1.21079	1.447133	0.766131	0.93302	0.860147	0.001398	0.681002
0.20587								
-0.23087								
0.20587	0.999539	1.39335	1.369482	0.737881	1.64013	1.36895	0.004078	0.902249
1.73893								
1.73893	0.570279	0.656519	0.342408	0.440384	0.680685	0.539412	0.121499	0.338276
1.73893	0.523853	0.275255	-0.19976	0.090172	-0.22015	-0.14002	0.112579	0.744005
0.931247	0.348263	0.627124	0.609259	0.147958	0.099219	0.380487	0.015652	0.527905
0.931247	-0.80693	-0.96031	-0.87046	-0.83367	-1.17479	-0.89584	0.15041	0.367863
0.931247	-0.53476	-1.08429	-1.44541	-0.86209	-0.93174	-0.93055	0.014985	0.910649
-0.10396	2.195947	2.471837	1.16331	1.107695	1.084522	1.105708	0.079779	1.387315
-0.69202	-0.42105	-0.9635	-1.13472	-0.4192	-0.80988	-0.69984	0.001638	0.715521
-0.69202	0.587013	0.710092	1.26904	1.126452	0.796888	0.717754	0.00177	0.682027
0.65863	0.31646	0.637779	0.837033	0.288622	0.857074	0.769443	0.017969	0.568451
0.65863	-0.39597	-0.55602	-0.18834	-0.45689	-0.50892	-0.46807	0.064728	0.367671
0.65863	-0.5346	-0.61414	-0.24427	-0.11693	-0.49233	-0.5466	0.000647	0.49721
0.148823	0.72123	1.242209	1.55095	0.723461	1.46348	1.237037	0.005007	0.82972
0.148823	-0.92101	-1.21085	-1.50942	-0.95228	-1.20297	-0.98927	0.009843	0.588412
0.148823	-0.77931	-1.33956	-1.58951	-0.89194	-1.26937	-0.82619	0.029925	0.810204
0.148823	-0.34106	-0.75328	-1.19467	-0.50361	-0.64743	-0.55462	0.018545	0.853613
0.148823	-0.78293	-1.12559	-1.08774	-0.87991	-0.84945	-0.79754	0.015453	0.342659
0.904913								
0.904913	-1.10978	-1.51309	-1.80438	-1.19333	-1.49152	-1.23498	0.025598	0.6946
0.904913	0.378153	-1.05374	-0.84998	-0.47222	-1.40822	-0.50461	0.022424	1.786373
0.904913	-1.53529	-2.22635	-2.53593	-1.57383	-2.02455	-1.60493	0.000154	1.000633
0.86074	-1.12491	-1.44348	-1.69591	-0.91399	-1.45065	-1.11427	0.0001	0.781917

0.86074	-0.04804	-0.99267	-1.23315	-0.82282	-1.20069	-0.99114	0.056396	1.185117
0.86074	-0.98538	-1.90967	-1.69484	-0.99503	-1.30366	-1.10074	0.030684	0.924294
-0.18031								
-0.62979	0.163754	0.471088	0.905669	0.309863	0.642313	0.583959	0.010169	0.741914
-0.62979	0.946113	2.099467	1.514627	1.391375	1.338575	1.313997	0.102135	1.153354
1.524593	1.32033	1.57105	1.525497	1.279567	2.179975	1.865747	0.006224	0.900408
1.524593	-1.06925	-1.70744	-1.83042	-1.03793	-1.62402	-1.11887	0.015148	0.792496
1.524593	-0.6793	-0.86025	-1.0884	-0.80833	-0.99209	-0.82542	0.034643	0.409098
1.76037	0.758836	0.816679	0.555623	0.147515	0.877889	1.097019	0.002153	0.949504
0.042053	0.671899	0.832656	0.648989	0.391421	0.92585	0.684285	0.044025	0.534429
-0.12665	0.184709	0.27472	0.14683	-0.00872	0.556861	0.484503	0.028091	0.565579
-0.12665	1.091405	0.940523	0.601351	0.701875	1.081772	0.696019	0.23548	0.490054
0.042053	0.256539	0.265597	0.229112	0.343187	0.806877	0.627311	0.014098	0.577765
-0.06361	0.635081	0.22512	0.146744	-0.0293	0.185227	-0.15086	0.0896	0.785938
-0.06361	-0.21077	-0.32548	-0.85285	-0.6395	-0.19873	-0.36317	0.046949	0.654118
-0.52057								
-0.10396	0.12507	0.388861	0.493057	0.276385	0.53887	0.413354	0.004623	0.4138
-0.52057	-0.36511	-0.54337	-0.5083	-0.14418	-0.40095	-0.3245	0.00872	0.399194
-0.52057	-0.41578	-0.88708	-1.12706	-0.69815	-0.33292	-0.54699	0.110906	0.794145
-0.52057	0.25272	-0.14465	-0.54191	-0.41053	-0.36386	-0.6185	0.063486	0.871224
-0.52057	-1.17278	-1.64735	-1.8853	-1.09559	-1.53958	-1.2191	0.003687	0.789709
-0.52057	-1.221	-1.73813	-1.81013	-1.36111	-1.73297	-1.46023	0.007148	0.589133
2.408297								
2.408297	1.068952	0.947417	1.057547	0.751104	1.428025	1.285577	0.012338	0.676921
0.709847	0.665057	1.018874	1.244484	0.285104	0.895134	1.139002	0.016903	0.95938
0.709847	0.516169	0.546176	0.0871	-0.04169	-0.14086	0.137848	0.008623	0.687041
0.709847	0.571981	1.06658	1.44076	0.922312	1.28754	1.056776	0.003973	0.868779
-0.55275	1.550103	1.289835	1.155047	0.593209	1.98529	1.406788	0.003358	1.392081
-0.55275	0.488472	0.842271	1.617563	1.111794	0.990335	0.64345	0.000545	1.129091
-0.04402	0.274089	0.641197	0.979134	0.410275	0.755411	0.760557	0.000464	0.705045
-0.55275	0.260322	0.725259	0.860585	0.169446	1.09896	0.966338	0.020237	0.929514
-0.04402	-0.45088	-0.43132	-0.57283	-0.63974	-0.29891	-0.39642	0.073787	0.340834
-0.04402	-0.56402	-0.75947	-0.58469	-0.51188	-0.53683	-0.61696	0.074702	0.247589
-0.04402	0.316876	-0.12171	-0.51743	-0.15067	-0.50898	-0.30427	0.010959	0.834308
-0.04402	-0.65863	-0.74212	-0.13244	-0.73277	-0.27501	-0.49345	0.057569	0.609677
-0.04402	0.32052	0.466876	0.317229	0.374143	0.503827	0.311655	0.078587	0.192172
-0.04402	-0.50352	-0.61331	-0.6378	-0.42834	-0.53393	-0.6543	0.027874	0.225954
-0.04402	-0.6114	-1.27854	-1.26553	-1.17638	-1.08255	-0.91442	0.078651	0.667141
-0.04402	-0.39539	-0.55377	-0.59574	-0.93414	-0.80872	-0.56874	0.07916	0.538759
-0.04402	0.754111	0.954348	-0.30098	-0.01588	-0.56346	-0.11183	0.029657	1.517806
0.005905								
-0.02993	-1.03852	-1.45333	-1.74283	-0.99114	-1.30497	-1.07692	0.002861	0.751689
-0.02993	-0.1222	-0.38906	-0.54726	-0.65182	-0.44565	-0.46703	0.04115	0.529625
0.099803	1.388641	1.084805	1.2539	0.901258	1.26313	1.003845	0.104342	0.487383
0.099803	0.787816	1.084467	1.21187	0.789714	0.833678	0.669172	0.006391	0.542698
0.138867	-0.16946	-0.18962	0.098302	0.497131	0.841853	0.026211	0.039594	1.031469
1.54046								

1.54046	-0.13509	-0.11209	0.217464	0.623929	0.781609	0.163956	0.010664	0.916694
1.54046	-0.74677	-0.91633	-1.59746	-1.02077	-0.87238	-0.66319	0.016475	0.934271
1.54046	-1.15545	-1.69676	-1.83437	-1.02798	-1.3382	-1.24779	0.004219	0.806384
1.54046	-0.70796	-1.1721	-1.65724	-1.12235	-1.53166	-1.05592	0.016638	0.949275
1.54046	-1.05239	-1.6122	-1.7424	-1.06773	-1.37912	-1.10002	0.005661	0.690011
1.54046	-0.12207	-0.44344	-0.35836	-0.34249	-0.25705	-0.45388	0.041839	0.331816
0.973702	0.691077	1.05356	1.024953	0.531217	0.922732	1.025344	0.040589	0.522343
0.138867	0.181965	0.632603	0.538768	0.140642	0.533107	0.478121	0.00578	0.491961
0.138867	-0.29985	-0.5348	-0.40503	0.016186	0.048207	-0.10469	0.038138	0.58301
0.973702	0.708337	0.631162	0.565168	0.237812	0.820922	0.657576	0.003662	0.58311
-0.47436								
-0.44633								
-0.44633	-0.60354	-0.78113	-1.12208	-0.91221	-0.92485	-0.75296	0.012034	0.518545
-0.44633	0.047522	0.517756	-0.61571	0.506848	-0.47339	-0.23545	0.095017	1.133469
-0.44633	0.115305	0.132154	0.378351	0.221476	0.132077	0.06707	0.048872	0.311282
0.928962	0.633845	1.072582	0.879717	0.253061	1.36665	1.013686	0.01893	1.113589
0.928962	-0.81476	-1.1154	-1.42368	-0.88629	-0.82376	-0.71687	0.008651	0.70681
0.928962	-1.06506	-1.49108	-1.71616	-1.26943	-0.4787	-1.13356	0.213009	1.237457
0.928962	-1.22205	-1.55159	-1.72121	-1.16703	-1.35173	-1.10792	0.01031	0.613295
0.928962	-0.95039	-0.88154	-0.65476	-0.31887	-1.00132	-1.1864	0.106824	0.86753
0.928962	-0.34437	-0.77018	-0.83157	-0.07566	-0.11714	-0.04707	0.007146	0.7845
-0.34741								
1.27202	1.590307	1.222873	0.914087	0.344516	0.607763	0.843095	0.001523	1.245791
1.27202	1.098985	1.50425	1.800127	1.086412	1.519915	1.482113	0.013422	0.713715
-0.34741	0.40442	0.767212	0.947617	0.395959	0.865803	0.670096	0.03059	0.551658
-0.99576	3.308557	3.051267	2.111893	3.007377	2.963352	1.982943	0.096112	1.325613
-0.99576	-0.23138	-0.55823	-0.7575	-0.15848	-0.56639	-0.60011	0.053119	0.599018
-0.99576	-0.22991	-0.23527	-0.04623	0.296399	0.131012	-0.09189	0.030518	0.531672
0.067428	0.465048	0.346996	0.464447	0.135381	0.62827	0.496534	0.043527	0.492889
0.067428	-0.07592	0.081044	0.396313	-0.02417	0.536925	0.338428	0.01186	0.612842
0.067428	0.710362	0.599703	0.751215	0.475431	0.519042	0.509178	0.346137	0.275785
0.067428	-0.81949	-1.19696	-1.29126	-1.07111	-1.5231	-1.03411	0.01732	0.703605
0.067428	0.359896	0.735576	1.075911	0.908368	1.021132	0.667171	0.001006	0.716014
-0.71657								
-0.71657	0.105478	0.436652	0.48801	0.314589	0.193018	0.136248	0.057332	0.382532
-0.71657	-0.72689	-1.03346	-1.12397	-0.52252	-0.72533	-0.61999	0.001564	0.601444
0.375288	-0.22353	-0.09545	-1.106	-0.97955	-1.00717	-0.77067	0.004918	1.010553
0.375288	0.477414	0.762745	0.853262	0.319939	0.274223	0.297946	0.044981	0.57904
1.053908								
1.053908	-0.42659	-0.32189	-0.26871	-0.10028	-0.42751	-0.44848	0.012817	0.3482
1.053908	-1.30292	-1.83235	-2.19964	-1.3184	-1.59526	-1.35593	0.001802	0.896723
1.053908	-0.84189	-1.19498	-1.44852	-0.92005	-1.14577	-0.99266	0.008772	0.606625
1.053908	0.533636	0.740352	0.605252	0.402483	0.689822	0.760925	0.002638	0.358442
	-0.10334	0.061043	0.04242	-0.12948	0.324314	0.190741	0.02114	0.45379
-0.36598	0.051373	0.55458	1.016348	0.534076	0.839126	0.529219	0.00497	0.964975
-0.36992	0.431985	0.841364	1.156586	0.776176	1.05271	0.627338	0.002894	0.724601

-0.83914								
-0.83914	-0.1395	-0.11187	-0.11876	-0.64906	-0.14407	0.019321	0.033978	0.668385
-0.83914	2.534013	2.135397	2.117847	2.316403	1.053974	1.645148	0.057438	1.480039
0.461637								
0.461637	-1.16061	-1.59654	-1.3788	-0.96786	-1.65027	-1.28914	0.008603	0.682407
0.461637	-0.86906	-1.50215	-1.86424	-1.01491	-1.3387	-0.96709	0.008169	0.99518
0.461637	-0.07408	-0.81792	-1.35306	-0.58353	-1.10027	-1.06085	0.015385	1.278985
0.461637	0.132382	0.404192	0.437433	0.24515	0.706591	0.222893	0.017079	0.574209
1.332213	1.035326	1.05544	1.58413	0.52972	1.73179	1.37323	0.01394	1.20207
1.573767	0.951098	1.227679	1.35163	0.82839	1.381395	1.349997	0.009737	0.553005
1.573767								
1.573767	0.988285	1.282542	0.972512	0.574112	1.90468	1.558253	0.003817	1.330568
-0.6226	1.260743	1.53981	1.733453	1.343243	1.62953	1.48085	0.017568	0.47271
-0.94955	0.752643	0.884047	1.32998	0.668199	1.062595	0.621043	0.080892	0.708937
-0.43583	0.561475	0.73719	0.863578	0.336162	0.682252	0.696056	0.034368	0.527416
-0.6226	0.648669	0.504257	0.969936	0.742333	0.475129	0.720163	0.024698	0.494807
-0.43583	-0.81013	-0.92778	-1.03192	-0.68696	-0.91894	-0.76054	0.010763	0.344965
-0.94955	0.824841	0.855762	0.942172	0.598849	0.83779	0.777189	0.010994	0.343324
-0.94955	0.082579	0.663569	1.245363	0.872325	0.516778	0.271425	0.00013	1.162785
1.976913	0.360744	0.510845	0.572707	0.188021	0.535275	0.705253	0.004199	0.517232
1.976913	0.286077	0.646066	0.819636	0.117708	0.302508	0.409087	0.04317	0.701928
-0.17249	0.125765	0.245349	0.430042	0.595887	0.302042	0.109964	0.090442	0.485923
0.46939	0.306787	0.612348	0.845578	0.208567	1.06772	0.804645	0.042478	0.859153
0.46939	-1.2445	-1.62004	-1.62291	-1.13983	-1.20444	-1.21585	0.025313	0.483088
0.46939	0.172071	0.633579	0.894535	0.479202	0.846825	0.682476	0.007331	0.722465
-0.26621	1.203987	1.549937	1.803173	1.268827	1.719915	1.563253	0.005935	0.599187
-0.17249	1.259393	1.870423	1.915767	1.42735	1.52667	1.587943	0.028447	0.656373
-0.17249	-0.11165	0.308297	0.465624	0.63167	0.555342	0.016848	0.016263	0.743316
0.257027	1.00543	1.322543	1.168742	0.764717	1.48354	1.274287	0.00128	0.718823
-0.26621	1.811793	2.05824	1.512193	1.253433	1.74305	1.505497	0.012869	0.804807
-1.01047	0.670308	0.97913	1.049086	0.603024	1.413095	1.407363	0.066005	0.810071
-0.32075	0.757676	0.752409	1.030768	0.681179	1.328675	1.172247	0.03831	0.647496
-0.32075	-0.45565	-0.57386	-0.53311	-0.03755	-0.42606	-0.3531	0.039419	0.536305
0.235237	1.060383	1.375623	1.601756	1.591153	1.940725	1.585243	0.047894	0.880342
0.257027	0.545534	0.747314	0.985327	1.089629	1.18439	0.920139	0.016705	0.638856
0.235237	3.379227	1.4992	1.014159	2.131845	2.503235	1.006461	0.030477	2.372766
-0.45531								
-0.45531	0.362278	0.540709	0.990337	0.790979	1.014665	0.585701	0.005301	0.652387
0.050937	0.151588	0.148595	-0.4435	-0.54915	-0.25222	0.020696	0.000941	0.700734
0.050937								
0.32292	-0.19389	-0.09061	0.009079	0.314157	0.43753	0.214509	0.01025	0.631422
-0.10689	-0.17414	-0.08781	0.062037	0.059765	-0.15878	-0.26437	0.003746	0.326412
-0.10689	-0.32567	-0.39687	-0.32243	0.112277	0.003352	-0.1082	0.011623	0.509144
-0.10689	0.559131	0.964947	0.946962	0.847454	1.58201	1.235607	0.085335	1.022879
0.32292	0.419436	0.909475	1.318753	0.828576	0.964624	0.744642	0.000611	0.899317
0.32292	0.529601	0.896493	0.952333	0.711013	1.76925	1.24323	0.006637	1.239649
-0.32824	0.701532	0.604435	0.850441	0.25385	0.481436	0.887602	0.003337	0.633753

0.475707	0.791296	0.852788	1.182621	0.57512	1.170053	1.298157	0.003989	0.723036
0.475707	-0.82392	-1.07896	-0.90339	-1.04348	-1.09533	-0.91684	0.048039	0.271401
0.475707	0.072159	0.08263	0.07784	-0.37999	0.162632	0.350064	0.004389	0.730059
0.823398	0.582864	0.414893	0.527138	0.443915	0.543149	0.573172	0.31323	0.167971
0.526255	0.413632	0.355076	0.545444	0.545808	0.944661	0.841564	0.003985	0.589585
0.526255	0.659409	0.478214	0.426081	0.539596	0.516748	0.658516	0.243235	0.233328
0.76993	0.877972	1.168099	1.19532	0.935464	1.325535	0.961957	0.017313	0.447563
0.24101								
0.24101	-0.65336	-0.65963	-0.59799	-0.46169	-0.38553	-0.53993	0.037273	0.2741
0.094247	0.500776	0.641468	0.920424	0.437334	0.766205	0.801988	0.140025	0.48309
0.094247	0.649202	0.973043	0.649134	0.153904	1.603735	1.015022	0.013151	1.449831
-0.6038	3.85957	4.142647	2.90598	2.73589	2.848621	2.3045	0.061583	1.838147
-0.6038	-0.73681	-0.57219	-0.54407	-0.69672	-0.64751	-0.78754	0.269934	0.243475
-0.6038	0.29908	0.230514	0.367481	0.759503	0.901999	0.221848	0.039909	0.680151
-0.73137								
-0.64519								
-0.64519	0.318663	1.064333	0.705736	0.727789	0.465152	0.224233	0.041655	0.840101
-0.75781	-0.10959	0.839652	1.016909	0.580471	-0.0904	-0.20354	0.000835	1.220454
-0.73137	0.533586	1.364173	1.612143	0.795388	0.510476	0.30331	0.000767	1.308834
0.76546	0.707302	0.97295	1.031215	0.607819	1.33	1.139101	0.032965	0.722181
0.76546	-0.05359	-0.15896	0.330645	0.082784	0.210039	-0.18514	0.011951	0.515782
-0.06019	2.533997	2.217443	1.90823	1.59354	2.627295	2.37266	0.302292	1.033755
0.22067	1.064715	1.2856	1.617853	0.965009	1.343375	1.27987	0.016047	0.652844
0.22067	0.838339	1.107691	1.153617	0.69185	1.132525	0.842951	0.039158	0.461767
3.022477	0.532183	0.78997	0.845595	0.259154	0.383106	0.537554	0.038273	0.586441
3.022477	-0.00075	-0.18198	-0.14344	0.143727	-0.03809	0.083921	0.096221	0.325712
0.478793	-0.78068	-1.36295	-1.12207	-0.94827	-1.02312	-0.74389	0.002933	0.619065
0.478793	2.160573	3.629067	1.884502	2.421783	1.462072	2.035612	0.062033	2.166995
0.478793	-0.72341	-1.50492	-1.78746	-1.23736	-1.61955	-1.23594	0.030604	1.064052
-0.87729	-0.38605	-0.57257	-0.54097	-0.45702	-0.73232	-0.66324	0.002851	0.34627
-0.87729	-0.73446	-0.2721	0.355275	0.075086	-0.54262	-0.5499	0.007086	1.089735
-0.87729	0.326239	1.155766	1.27061	0.583504	0.790956	0.335501	0.016313	0.944371
0.303257	0.394834	0.675506	0.757971	0.706537	0.783911	0.676387	0.009427	0.389076
-0.28848								
-0.28848	0.363255	0.575644	0.591754	0.578954	1.004592	0.694684	0.038147	0.641337
-0.28848	0.408117	0.509556	0.397596	-0.02886	0.595457	0.647035	0.018328	0.675892
1.239727								
1.239727	-1.0538	-1.51782	-1.66177	-1.08518	-1.12566	-0.95688	0.015627	0.704889
1.239727	-1.02805	-1.7094	-1.58225	-1.04719	-1.17859	-0.96041	0.002829	0.748985
0.210548								
0.041077	0.426101	0.820441	1.154651	0.763983	1.090855	0.815355	0.003258	0.72855
0.041077								
0.210548	0.713174	0.873047	1.36737	0.910871	1.0994	0.962945	0.010997	0.654196
-0.19262	-0.57291	-0.84845	-0.77711	-0.19109	0.056837	-0.19002	0.025607	0.905285
0.29973	1.886433	1.847423	1.012346	2.015752	1.50216	1.407166	0.101367	1.003406
0.29973	0.412224	0.50416	0.882725	0.606576	0.807633	0.399546	0.134339	0.483179
-0.19262	0.361277	0.540565	0.443445	0.395252	0.619726	0.412188	0.044431	0.258449

-0.95489	0.987637	1.203317	1.249137	1.213433	0.990928	0.838536	0.074843	0.410601
-0.75011	-0.54354	-0.44945	-8.93E-05	-0.08642	-0.79721	-0.66299	0.016412	0.797125
-0.75011	0.552854	1.318083	-0.03479	0.132646	0.235268	0.891761	0.105052	1.352874
-0.75011	0.215306	0.48505	0.859199	0.768784	0.889361	0.243196	0.196129	0.674055
-0.94119	2.199363	2.300424	1.746012	1.809429	1.786679	1.506298	0.065199	0.794126
-0.84317	-0.15904	0.189929	0.624181	0.603928	0.082795	-0.04597	0.00779	0.783226
-0.84317	0.140447	-0.0204	0.439383	0.10609	-0.24409	-0.28148	0.027343	0.720863
-0.94119	-0.5216	-0.88084	-1.00263	-0.67662	-1.01855	-0.79224	0.006888	0.496951
-0.94119	-0.03605	-0.44749	-0.92086	-0.17283	-0.73183	-0.40573	0.004898	0.884808
2.262227	-0.19017	0.244056	0.384288	0.977593	0.156904	0.463671	0.064222	1.167762
2.262227	-1.00149	-1.17814	-1.11949	-0.99712	-1.1103	-1.04134	0.059798	0.181023
0.185723	0.276667	0.827887	0.914407	0.335787	1.055215	0.686543	0.052203	0.778548
0.185723	0.31186	0.249028	0.327154	0.224137	0.28023	0.192276	0.100655	0.134878
0.185723	-0.58954	-0.49153	-0.77631	-0.96253	-0.83708	-0.72886	0.030198	0.471006
0.437038	0.106156	0.303094	0.764614	0.064915	0.534803	0.563492	0.02031	0.699699
0.437038	-0.91367	-1.61277	-1.57781	-1.07154	-1.40314	-1.09041	0.001608	0.699102
0.437038	0.179666	0.331481	-0.74432	-0.50684	-0.95756	-0.61832	0.140923	1.289041
0.437038	-0.10254	-0.36093	-0.37268	-0.60834	-0.65827	-0.55438	0.131799	0.555732
0.437038	-1.10928	-1.85598	-2.11295	-1.21088	-1.63143	-1.16188	0.000716	1.003669
0.437038	-1.23634	-1.80617	-2.05287	-1.31261	-1.69149	-1.34646	0.001683	0.816537
0.437038	-0.75503	-1.27978	-1.54061	-0.79732	-1.3668	-1.02758	0.000815	0.785577
0.437038	-1.16186	-1.58578	-1.56062	-1.24127	-1.92134	-1.54449	0.006838	0.759477
0.437038	-1.21828	-1.73464	-1.89507	-1.34153	-1.67219	-1.28603	0.005454	0.676787
0.437038	-0.7753	-1.32436	-1.59082	-0.86351	-1.1505	-0.89021	0.01052	0.815513
0.437038	-0.92705	-1.21225	-1.56464	-0.98485	-1.57436	-0.94074	0.058653	0.647311
0.437038	-0.78048	-1.26298	-1.64571	-0.76831	-1.2384	-0.85428	0.022469	0.877399
0.437038	0.042875	-0.64796	-1.06982	-0.39926	0.785054	-0.5361	0.036558	1.854871
0.437038	-1.34589	-1.75815	-1.821	-1.27979	-1.6341	-1.28486	0.000497	0.54121
0.437038	1.119872	0.847103	0.181686	0.515022	1.003676	0.410384	0.111091	0.938186
0.036197	0.549462	1.223037	1.761473	1.018925	1.40194	0.962487	0.003666	1.212011
0.036197	-0.27762	-0.49429	-0.13237	0.200974	-0.21125	-0.08577	0.006354	0.695262
0.036197	0.952692	1.491697	1.6275	1.14843	1.621205	1.246633	0.011332	0.674808
-0.27771	2.128677	2.402423	1.957773	1.437627	1.472235	1.25963	0.000384	1.142793
0.121703	1.143795	1.287963	1.192165	0.629224	0.995382	1.097355	0.005693	0.658739
0.121703	0.306348	0.51095	0.177015	-0.28949	0.496066	0.397082	0.014794	0.800438
0.121703	0.071272	0.141148	-0.12307	-0.3861	-0.15854	0.1058	0.037687	0.527246
-0.27771	-0.89697	-1.19475	-1.4219	-1.15796	-1.69322	-1.12237	0.040588	0.796248
-0.27771	0.440386	0.539747	0.542634	0.806131	1.032557	0.747786	0.017316	0.592171
0.441337	2.472993	2.087303	1.930843	1.439997	1.882055	2.323133	0.071665	1.032997
0.441337	-0.48543	-0.87027	-1.00166	-0.5052	-0.85776	-0.55898	0.028908	0.516229
0.441337	-0.20078	-0.40793	-0.44393	-0.30318	-0.33168	-0.19351	0.004656	0.250424
0.48941	1.5923	1.559675	1.630428	0.952197	2.037678	1.670827	0.088152	1.08548
0.48941	0.230544	0.263817	0.343147	0.139194	0.106652	-0.03558	0.009603	0.378726
0.48941	0.02021	-0.11874	0.130578	0.404698	0.2919	0.259718	0.014426	0.523441
-0.40086	-0.59611	-0.95829	-1.19463	-0.69354	-0.82282	-0.69178	0.000768	0.598513
-0.40086	0.627518	1.109261	-0.11996	0.878192	-0.58092	0.221825	0.057561	1.690183
0.204597	0.313168	0.487789	0.454498	0.735775	0.873841	0.784756	0.115238	0.560672

-0.40086	0.609287	0.102282	-0.56413	-0.0345	0.217681	-0.39942	0.014649	1.17342
0.204597	-0.92065	-1.70547	-1.70202	-1.08279	-1.41213	-1.08642	0.046374	0.784812
0.204597	0.8126	0.712606	0.457028	0.827146	0.184876	0.13751	0.237347	0.689636
-0.18078	0.900135	1.042561	1.081034	0.797061	0.764138	0.823999	0.044144	0.316896
-0.18078	-0.01173	0.01135	-0.03433	0.088549	0.132055	0.006495	0.213985	0.166385
-0.19113	0.49536	0.723708	0.969676	0.827127	1.208045	0.945044	0.000854	0.712685
-0.19113	-0.15498	-0.34592	-0.31914	-0.47428	-0.52872	-0.32618	0.004938	0.373738
0.987057	1.58381	2.050367	1.96282	1.393653	2.307685	1.981317	0.004828	0.914032
-0.14461	1.444327	1.813367	1.83837	1.249967	2.079985	1.849937	0.005194	0.830018
-0.94521	1.287123	1.559433	1.88537	1.158013	1.773415	1.45682	0.003937	0.727357
-0.94521	0.665921	1.158591	1.604567	1.089524	1.40573	1.044769	0.000399	0.938645
-0.14461	-0.13495	-0.28617	0.435537	0.750892	0.328402	-0.1714	0.004987	1.037064
-0.14461	0.370664	0.651057	1.213568	1.2331	1.049318	0.701641	0.000752	0.862436
1.185985	5.55645	5.646057	4.82478	4.627153	5.20846	4.545413	0.050988	1.100643
1.185985	-0.83343	-1.48063	-1.78772	-1.31149	-1.26755	-1.30408	0.018941	0.954288
1.185985								
1.185985	-0.01681	-0.02043	0.425435	0.969882	0.746266	0.369166	0.004926	0.990316
-0.40806	-1.07667	-1.8213	-2.05964	-1.29882	-1.92195	-1.42815	0.007602	0.982964
-0.40806	0.10142	-0.92662	-0.53108	-0.68748	-0.5714	-0.65466	0.106343	1.028039
-0.40806	-0.89118	-1.49516	-1.52557	-1.08576	-1.15146	-0.78201	0.058728	0.743559
-0.71768	0.043814	0.385704	0.914746	0.471058	0.350371	0.231336	0.001999	0.870933
-0.71768	-0.44311	-0.30086	-0.10202	-0.1926	-0.2943	-0.24544	0.025056	0.341098
0.374707	0.701844	0.789004	0.897678	0.465131	0.839652	0.760482	0.039647	0.432547
0.065063								
0.065063	-0.18749	-0.51503	-0.73797	-0.40497	-0.62633	-0.48681	0.022405	0.550479
0.065063	-0.41881	-0.99808	-1.04534	-0.59414	-0.87639	-0.69535	0.002882	0.626523
-0.83535	-0.01578	0.307595	0.698281	0.400231	-0.33057	-0.3808	0.003369	1.079077
-0.83535	0.509558	0.924029	1.076038	0.4282	0.644283	0.561494	0.005624	0.647838
0.789467								
0.789467	-0.72816	-1.51014	-1.82478	-0.94815	-1.41533	-1.0952	0.001836	1.096616
0.789467	0.425881	0.475202	0.626035	0.534841	0.80947	0.700713	0.014366	0.383589
0.281003	-1.25817	-1.54693	-1.6536	-1.10164	-1.69592	-1.52584	0.133687	0.594271
0.057353	0.4779	0.637728	0.593012	0.745307	0.815591	0.68466	0.03297	0.337691
0.057353	-0.202	-0.47524	-0.70342	-0.4359	-1.22635	-0.72093	0.137208	1.024354
0.057353	-1.13562	-1.6514	-1.63134	-1.19847	-1.69932	-1.63115	0.031289	0.563701
0.281003	1.45062	1.242803	0.563982	0.74833	0.420478	0.390274	0.011701	1.060346
1.279947	1.085523	1.067437	1.334033	0.920023	0.994625	1.116957	0.002007	0.414011
1.279947	0.074308	0.04408	-0.11161	-0.10904	0.183646	-0.02418	0.127519	0.295259
-0.85048								
-0.39782								
-0.39782	0.750307	1.090711	1.25782	0.536091	0.577576	0.408798	0.02357	0.849022
-0.1409	0.395661	0.279196	1.210977	0.171467	1.07694	1.254703	0.000192	1.083236
-0.1409	1.266507	1.479347	1.025453	0.99734	0.965214	1.01378	0.146755	0.514133
1.537117	0.166493	0.316526	0.525496	0.039278	0.80547	0.770058	0.00472	0.766192
1.537117	0.404603	0.598776	0.722891	0.619512	0.663379	0.532548	0.008038	0.318288
-0.31262	0.223286	0.356978	0.790341	0.452494	0.488445	0.421451	0.037726	0.567054
-0.31262	0.095353	-0.16273	-0.35345	-0.53358	-0.49922	-0.28246	0.111864	0.628937

-0.31262	-0.89036	-1.3769	-1.63225	-0.92647	-1.22741	-0.9016	0.000395	0.741884
-0.31262	-0.7334	-1.08668	-1.36015	-0.74641	-0.98956	-0.86677	0.010291	0.626752
	0.214748	0.229914	0.218304	0.642625	0.704798	0.565097	0.006695	0.49005
-0.85217	-0.52219	-0.30933	-0.18512	-0.32276	-0.65341	-0.32893	0.06588	0.468293
-0.85217	-0.47862	-0.33505	-0.19241	0.152405	0.063608	-0.18074	0.019638	0.631027
-0.85217	1.063188	1.230277	1.912677	1.73145	1.49909	1.182699	0.021357	0.849489
0.607493	0.243629	0.587604	0.912742	0.815694	1.58302	1.140257	0.004238	1.339391
0.980293	0.534304	0.892493	1.169803	0.823682	1.712275	1.270617	0.002466	1.177971
1.139853								
1.139853	0.082021	0.246347	0.205124	0.005615	0.248195	0.150659	0.034258	0.24258
1.139853	-0.37895	-0.61123	-0.53137	-0.09166	-0.25541	-0.26837	0.017911	0.519569
0.980293	1.259192	1.297717	1.743745	1.215042	2.90244	1.142504	0.212416	1.759936
0.607493	0.045175	0.025554	-0.17949	0.399937	0.09784	0.06531	0.002392	0.579426
0.607493	0.037131	-0.19088	0.052829	0.343243	0.113972	-0.05845	0.009135	0.534124
-0.4997	0.10514	0.159765	0.784603	0.507754	0.020741	0.338248	0.031711	0.763863
-0.89942	0.095519	0.291695	0.723988	0.469248	0.037971	0.174817	0.027784	0.686017
-0.89942	-0.00282	0.480638	0.521746	-0.11329	-0.02986	-0.11545	0.030278	0.637201
-0.89942	-0.46542	-0.30556	0.318576	0.236198	-0.14346	-0.26942	0.002725	0.783997
-0.54481								
-0.54481	-0.72456	-1.09394	-1.36205	-0.95401	-1.33152	-1.03072	0.014387	0.637484
-0.54481	0.443672	0.069791	-0.32616	-0.0828	-0.10274	-0.26756	0.024831	0.769836
-0.54481	0.000241	0.025735	0.077365	-0.4039	0.238862	0.190666	0.004009	0.642762
-0.28069	-0.14052	-0.35335	0.194423	-0.05391	0.207165	-0.13506	0.069961	0.560515
-0.28069	-0.22959	-0.29527	-0.38216	-0.36493	-0.09516	-0.2436	0.029798	0.286999
-0.06448	0.235828	0.086232	-0.15564	-0.24924	0.578225	0.385461	0.012403	0.827462
0.258243	-0.01278	0.272707	0.425322	-0.32175	1.223875	1.127717	0.002529	1.545625
0.258243	0.444702	1.260976	1.57418	0.789254	1.12435	0.808452	0.004557	1.129478
0.558763	1.162588	1.48189	1.300857	0.564115	1.167825	1.289673	0.004291	0.917775
0.558763	-0.66398	-1.35279	-1.43096	-0.85545	-1.28624	-0.97992	0.035755	0.766976
0.558763	-0.41867	-0.38489	-0.55961	-0.38864	-0.26187	-0.1724	0.1232	0.387209
0.067987	0.528184	0.764278	0.705355	0.528775	0.622485	0.609254	0.180313	0.236094
0.558763								
0.067987	0.020398	0.064834	0.652741	0.467461	0.579505	0.281668	0.047735	0.632342
2.17784	0.716654	1.111465	1.36965	0.697954	1.28566	1.204585	0.018491	0.671696
2.17784	-0.94189	-1.28692	-1.51496	-1.07189	-1.2962	-1.04972	0.029857	0.573071
2.17784	-0.21274	-0.28722	-0.5262	-0.01886	-0.24102	-0.09564	0.106719	0.50734
0.08375								
0.08375	-0.23839	-0.3785	-0.69807	-0.39963	-0.59264	-0.57657	0.116903	0.459679
1.525737								
-0.0045	1.218377	0.998295	1.402323	1.067753	1.31956	1.094674	0.197265	0.404028
-0.0045	-0.97004	-1.21155	-1.09676	-0.71259	-0.77773	-0.75683	0.002643	0.498958
-0.0045	0.875144	0.861071	1.254308	0.800806	1.737365	0.854334	0.175848	0.936559
-0.44867								
-0.44867	-0.07459	0.234352	0.37972	0.588712	-0.2072	0.390443	0.129076	0.795909
-0.44867	-0.01691	0.186895	0.645826	0.641305	-0.04548	-0.1048	0.014693	0.750631
-0.84837								

-0.84837	0.192588	1.125593	1.385383	0.69526	0.5048	0.227056	0.003472	1.192795
-0.50916								
-0.50916	1.45316	2.001663	2.143297	1.274177	1.437335	1.105842	0.006747	1.037455
0.405747								
0.405747	0.091736	-0.51017	-0.26905	-0.09404	-0.66691	-0.53693	0.065271	0.758648
0.405747	-1.0678	-1.03092	-1.52471	-1.03594	-1.41585	-1.04697	0.090272	0.493785
0.405747	-0.82849	-1.40068	-1.67999	-0.85866	-1.45656	-1.1381	0.011119	0.851495
0.405747	-0.70652	-1.25224	-1.51174	-0.79133	-1.2342	-0.87699	0.007196	0.805218
0.405747	-0.8744	-1.64447	-1.85496	-0.99649	-1.3556	-0.9922	0.006548	0.980558
0.405747	-0.72505	-1.39645	-1.62953	-0.92651	-1.48036	-1.05869	0.004305	0.904483
0.405747	-0.36931	-1.26153	-1.07922	-0.81947	-0.68613	-0.90182	0.047869	0.892221
0.405747	-1.05074	-1.58687	-1.92155	-1.16526	-1.56367	-1.23687	0.002646	0.870812
0.405747	0.794826	1.028455	0.873049	0.227735	0.71324	0.543033	0.007702	0.80072
-0.17721	1.47372	1.291416	1.055541	1.076836	1.276635	1.20382	0.148196	0.418179
-0.17721	1.539453	1.557163	1.474472	1.413006	2.034725	1.719347	0.15701	0.621719
-0.97533	0.213928	0.376896	0.47075	0.546441	1.2805	1.054076	0.065756	1.066572
-0.97533	1.733713	2.059753	2.262233	1.314302	1.67101	1.569773	0.058783	0.947931
-0.46533	-0.05106	0.649721	1.218877	0.70245	0.261938	-0.08914	0.000308	1.308014
-0.46533	1.52893	1.780263	1.887493	1.376367	1.670685	1.077635	0.009118	0.809858
1.20907	-0.75414	-1.31487	-1.64195	-0.99938	-1.28154	-1.22601	0.041164	0.887805
0.058603	-0.93024	-1.34496	-1.12422	-0.6736	-0.83421	-0.9186	0.026269	0.671359
0.058603	-0.93744	-1.50977	-1.74452	-1.05074	-1.40376	-1.11013	0.000431	0.807075
0.058603	0.358715	0.723203	0.446387	0.754416	1.25213	0.933676	0.004705	0.893415
1.525737	1.764683	1.73929	1.347593	1.424313	1.531275	1.390673	0.041255	0.41709
1.525737	-0.30312	0.175551	0.420604	0.191154	0.219222	0.000388	0.021163	0.723721
0.219325								
0.219325	0.706884	1.36189	1.374872	0.705957	1.586495	1.386003	0.00566	0.880538
1.10856								
0.058603	0.924522	0.29159	0.045086	0.375169	0.658783	0.362328	0.025514	0.879437
0.839163	-0.46056	-0.58246	-0.85476	-0.47921	-0.21333	-0.41707	0.081275	0.641434
0.839163	-1.06604	-1.36884	-1.57606	-1.15881	-1.45872	-1.16687	0.034433	0.510021
-0.44891	0.987012	1.251723	1.7987	0.812807	1.23667	0.940331	0.007976	0.985893
0.839163								
1.10856	0.207682	0.238314	0.547647	0.147271	0.346997	0.367234	0.052863	0.400376
-0.44891	1.557277	1.72619	1.994273	1.81558	2.382745	2.01517	0.025785	0.825468
-1.15622								
-1.15622	-0.32085	-0.30484	-0.18281	-0.08351	0.127093	0.007673	0.006045	0.447946
-1.15622	-1.06611	-1.38478	-1.47176	-0.93504	-1.32981	-1.02251	0.01456	0.536721
-1.15622	0.508402	1.276787	1.640853	1.0402	0.683948	0.494251	0.010706	1.146602
0.326377								
0.326377	0.743499	0.942528	0.885296	0.817021	1.18769	1.035298	0.051603	0.444191
0.429833	-0.15149	-0.26919	-0.66145	-0.50719	-0.31309	-0.59913	0.035953	0.509962
0.429833	0.307571	0.352927	0.011347	-0.51094	0.471175	0.233338	0.018251	0.982113
0.543247	0.09632	0.059748	-0.10467	-0.62667	-0.18734	0.212853	0.052895	0.839524
0.429833	0.083439	0.799066	0.134909	-0.32226	0.131606	0.366748	0.078207	1.121331
0.543247	-1.28901	-1.43557	-1.49272	-1.14676	-1.14341	-1.17614	0.041932	0.349318
0.543247	-0.61745	-1.18984	-1.33364	-0.85639	-1.02262	-0.95435	0.010744	0.716197

0.543247	-0.98849	-1.11343	-0.89093	-0.52064	-0.86486	-0.82794	0.006832	0.592788
-0.89941	1.127872	1.630992	1.959803	1.265641	1.784905	1.277047	0.028578	0.831932
-0.24883	1.023285	0.890329	0.302765	0.592179	0.458148	0.270177	0.011282	0.753107
-0.24883	4.277503	4.445953	3.255473	3.53832	3.469865	3.63315	0.031522	1.19048
0.69081	0.599659	0.236961	0.695128	0.138395	0.780092	0.272866	0.077285	0.641697
-0.24883	-0.22829	0.107334	0.040958	-0.25648	0.581618	0.406373	0.008258	0.838097
0.69081	0.37657	0.63081	0.565329	0.059131	1.04927	0.666777	0.000796	0.990139
0.37916	0.338198	0.685098	0.818466	0.72025	0.924495	0.592786	0.014917	0.586296
0.37916	0.120004	-0.66694	-0.77942	-0.62054	-0.95368	-0.7171	0.079192	1.073688
0.37916	-0.83894	-1.48006	-1.71375	-1.06427	-1.37427	-1.08423	0.002054	0.874802
0.37916	-0.68084	-1.22507	-1.47298	-0.9096	-1.33811	-1.0038	0.000856	0.792144
0.37916	-1.18546	-1.7515	-1.76687	-1.25933	-1.5496	-1.33792	0.079245	0.581413
0.37916	-1.07936	-1.48134	-1.60645	-1.02436	-1.35771	-1.02383	0.02892	0.582623
0.37916	0.663934	1.009179	1.279522	1.118713	1.53885	1.1125	0.008019	0.874916
-0.40817	0.146136	0.425934	0.832514	0.319212	0.630719	0.355071	0.004098	0.686378
-0.06181	1.113388	0.755937	1.144867	0.394355	0.915796	0.739055	0.02447	0.750512
-0.17537	0.248357	0.49193	0.981044	0.631743	0.745775	0.489908	0.019932	0.732687
-0.17537	0.271367	0.30773	0.804639	0.091739	0.540257	0.188666	0.002703	0.7129
0.330027	1.476397	1.568637	1.393559	1.477827	2.05239	1.900157	0.057049	0.658831
0.435247								
0.435247	1.6857	1.336532	1.371293	1.416363	1.970375	1.885177	0.027439	0.633843
-0.3377	1.209465	0.69976	0.49662	0.229585	0.405072	0.433898	0.03087	0.97988
-0.3377	0.229707	0.551924	0.936217	0.885075	1.264865	0.747877	0.037266	1.035158
0.014362	0.167725	0.531315	0.192553	0.068151	0.849887	0.447624	0.010645	0.781735
-0.64125	0.626728	1.065427	0.816005	0.700562	1.015773	0.904931	0.035463	0.438698
0.906277	0.707632	0.907222	1.193509	0.578875	0.694479	0.77304	0.018724	0.614634
0.906277	0.525209	1.034063	0.846529	0.483744	0.042408	0.623252	0.059805	0.991655
-0.58975	-0.14344	-0.00336	0.519431	0.296788	0.075788	0.054961	0.001441	0.662868
-0.58975	-0.33952	-0.46688	-0.24082	0.334186	0.461943	0.099086	0.003725	0.928827
-0.58975	0.264131	0.157112	0.590354	1.08319	0.801716	0.544479	0.000996	0.926078
0.061767	0.989449	1.27827	1.778933	0.869425	0.920362	1.121268	0.00259	0.909509
0.061767	0.186544	-0.25654	-0.3408	-0.21886	-0.18542	-0.37651	0.132138	0.563052
0.061767	-0.51493	-1.19095	-1.55169	-0.94069	-1.23777	-1.29929	0.01652	1.036764
0.061767	0.886591	1.393594	0.622198	0.914843	-0.03233	0.66098	0.056069	1.425924
-0.31071	0.187927	0.323836	0.479207	0.363867	0.331172	0.310239	0.04478	0.29128
-1.1648								
-1.1648	-0.15283	0.434869	1.064457	0.157456	0.216576	-0.01207	0.015545	1.217292
-0.31071	1.397891	1.068698	1.365838	1.50944	0.374531	1.253617	0.131409	1.134909
-0.31071	-0.53632	-1.04899	-1.32107	-0.7922	-1.1234	-0.82686	0.000664	0.784742
-0.31071	-0.68186	-0.97694	-1.34639	-0.7321	-1.10902	-0.99046	0.003513	0.664526
0.51355	1.240247	1.80498	1.94328	0.966943	0.986656	1.250457	0.000267	0.976337
0.51355	-0.82534	-1.38589	-1.64635	-1.03339	-1.22678	-1.2555	0.002196	0.821014
0.51355	-0.51665	-0.80256	-0.35093	-0.81536	-0.97194	-0.9432	0.096308	0.621014
-0.86849								
-0.86849	-0.41917	-0.56411	-0.74451	-0.7038	-0.20445	-0.4412	0.019443	0.540056
-0.86849	-1.05146	-1.56308	-1.72858	-1.23682	-1.48103	-1.23261	0.011551	0.677119
-0.86849	1.636646	-0.29375	-1.23075	-0.16821	-0.65143	-0.23923	0.035342	2.867396

-0.86849	-1.2232	-1.08749	-1.90615	-1.28885	-1.85363	-1.64102	0.160322	0.818653
-0.86849	-0.5492	-1.03839	-0.82973	-0.86455	-1.03661	-0.86067	0.004983	0.489186
-0.86849	3.641247	4.32114	2.77809	2.54796	2.86207	2.499373	0.058393	1.821767
3.149357								
3.149357	-0.68708	-1.49944	-1.56933	-0.90186	-1.16558	-0.92361	0.001084	0.882245
3.149357	0.309043	0.397777	0.250421	-0.06874	0.630727	0.522445	0.028288	0.699465
-0.04492	1.6052	1.803903	2.058637	1.52498	2.09558	1.787473	0.01598	0.5706
-0.24793	0.605855	0.852832	1.147234	0.653431	0.863603	0.846483	0.005537	0.541379
-0.24793	1.61777	1.686677	2.011157	1.523357	1.84287	1.881093	0.039961	0.4878
-0.24793	0.948751	1.256897	1.220679	0.862773	1.429165	1.147046	0.029499	0.566392
-0.04492	-0.18737	0.450847	0.72582	0.463422	0.29701	0.107961	0.001861	0.913185
-0.04492	-0.26022	-0.19842	-1.20424	-0.76526	-0.81071	-0.76002	0.066326	1.005818
-0.04492	1.40971	1.757633	1.505443	1.112187	1.70159	1.469117	0.032801	0.645447
0.439173	1.790973	1.696757	0.864914	0.970973	1.258565	1.257447	0.005512	0.926059
-0.19104	2.661313	2.37322	1.62312	2.033011	1.681505	1.724653	0.023196	1.038193
-0.19104	-0.74046	-1.75184	-1.91488	-1.34676	-1.95939	-1.646	0.004748	1.218926
-0.19104	0.761973	1.10835	1.213133	0.954436	0.199812	0.299191	0.009224	1.013322
-0.34979								
-0.34979								
-0.34979	0.109165	0.783183	1.524843	0.505795	0.522898	0.37044	0.001348	1.415679
-0.38072								
-0.38072	0.665116	0.296219	1.408848	0.985775	0.596432	0.67405	0.047427	1.112629
-0.38072	-0.06282	-0.35782	-0.73409	-0.61977	-0.45476	-0.75631	0.014912	0.693485
-0.38072	1.41775	1.245	0.637077	0.929362	0.861043	0.901271	0.007541	0.780673
0.644243	0.854424	1.36237	1.826143	1.161611	0.761479	0.471027	0.00356	1.355116
0.644243	-1.0129	-1.04573	-0.85617	-0.82649	-0.73477	-0.76521	0.014883	0.310959
1.08129	4.270343	5.031013	3.490643	3.486797	4.011525	3.02925	0.02443	2.001763
1.08129	-0.99095	-1.35908	-1.6948	-0.8582	-1.40224	-0.96766	0.030864	0.836598
1.08129	0.645574	0.244499	0.096436	0.385762	-0.13674	0.67795	0.083928	0.814685
	-0.49776	-0.76758	-0.71328	-0.28324	-0.34366	-0.40007	0.055846	0.484343
	0.385069	0.146357	0.449724	0.337495	0.553113	0.364472	0.063043	0.406756
-0.45986								
-0.62352								
-0.62352	1.073916	0.989187	0.556243	0.543229	0.681903	0.362455	0.045199	0.711461
0.03739	0.346826	0.488384	0.959692	0.403054	0.850316	0.592137	0.011438	0.612866
0.03739	-0.17071	-0.2843	-0.02392	-0.22465	-0.35769	-0.3482	0.075568	0.33377
-0.88734								
-0.88734	1.165767	1.328347	1.64678	1.068614	1.47957	1.309953	0.03895	0.578166
-0.86634	0.801562	1.017499	1.477174	0.942496	1.248686	0.869005	0.056291	0.675611
-0.86634	-0.19486	-0.39717	-0.3694	-0.17701	-0.26781	-0.05601	0.12522	0.341165
-0.86634	-0.67842	-1.09911	-0.89154	-0.53853	-0.62274	-0.73096	0.033416	0.56058
0.263903								
0.263903	-1.23792	-1.39032	-1.48627	-0.94192	-1.3676	-1.12192	0.018024	0.544346
0.263903	-1.4345	-1.76722	-1.89833	-1.39514	-1.75533	-1.36117	0.000241	0.537167
	0.719236	0.763383	0.357478	0.583428	0.884955	0.837341	0.002758	0.527477

-0.30179								
-0.30179	0.379688	0.374793	0.112291	0.327772	0.582455	0.257919	0.18825	0.470164
2.664365								
2.664365	-0.95667	-1.54379	-1.50156	-1.03722	-1.29357	-1.04607	0.002843	0.587122
2.664365	-1.09023	-1.73464	-1.53122	-0.88882	-1.1237	-0.90767	0.008649	0.845826
2.664365	-0.61417	-1.0394	-1.04733	-0.67598	-0.42716	-0.51137	0.016747	0.620171
2.664365	-0.86793	-1.78516	-1.78688	-1.05678	-1.36717	-1.23064	0.004743	0.918958
2.664365	-0.40237	-0.88568	-1.43471	-0.60449	-0.84288	-0.76342	0.03503	1.03234
2.664365	0.157802	-0.04195	-0.11758	0.08434	0.789138	0.347468	0.013643	0.906719
2.664365	0.308949	-0.03098	-0.12271	-0.33953	0.745089	0.539557	0.007481	1.08462
-0.35473								
-0.35473	0.154021	0.097871	-0.35097	0.491251	1.07555	0.626715	0.001565	1.426515
-0.82783	0.419123	0.70156	1.413807	0.572579	0.703341	0.464152	0.005618	0.994684
-0.82783	1.697733	1.717673	1.80332	1.740163	1.39102	1.290533	0.05089	0.512787
	1.769234	1.35968	0.844636	0.952947	0.960334	0.699141	0.008265	1.070093
-0.08352	0.577697	0.663542	0.945661	0.662132	0.961318	1.013641	0.003036	0.435944
-0.08352	-0.66101	-0.96583	-0.84233	-0.49293	-0.63242	-0.69064	0.027743	0.472894
-0.08352	0.122258	-0.22727	-0.27926	0.107887	0.016899	-0.25934	0.131998	0.401518
-0.342	0.158031	0.516754	0.595945	0.471291	0.491701	0.597458	0.048865	0.439427
-1.11266								
-1.11266	-0.69313	-1.0091	-1.31833	-0.75801	-1.05914	-0.8841	0.083296	0.625197
-1.11266	0.377728	0.450282	0.03889	0.711402	-0.54433	0.055202	0.106737	1.255735
-1.19043	0.363171	0.625772	0.520748	0.281416	0.837097	0.517983	0.019967	0.555681
-1.19043	0.482225	0.776923	0.829938	0.680071	0.398365	0.612799	0.082203	0.431573
0.630132								
0.630132	-0.84528	-1.17366	-1.45331	-0.90649	-1.0982	-0.75689	0.014793	0.696423
0.630132	-1.45192	-2.05906	-2.2093	-1.44965	-1.83897	-1.59981	0.002426	0.759653
0.630132	-0.17577	0.093502	0.336751	0.34219	0.7113	0.357686	0.000123	0.887068
-0.40746								
-0.40746	-0.92174	-1.28595	-1.50351	-0.91974	-1.29591	-1.04881	0.000482	0.583765
-0.40746	-1.47729	-2.09228	-2.22892	-1.76203	-2.07135	-1.7775	0.002076	0.75163
-0.40746	-0.80309	-1.04006	-1.11203	-1.05081	-1.0025	-0.90196	0.05436	0.308939
-1.38646	0.437169	0.731635	0.947521	0.490763	0.033282	0.078534	0.001768	0.914239
1.404203	0.140897	0.52767	0.85806	0.453164	0.204543	0.071356	0.034084	0.786704
0.211923								
0.211923	4.84724	5.39958	3.777507	3.718713	4.226425	3.70973	0.072271	1.68985
-0.53898	0.778285	1.26243	1.62634	1.095082	1.000503	1.02252	0.002016	0.848055
-0.71462	0.014319	0.313619	0.637957	0.102132	0.698496	0.212595	0.196114	0.684177
-0.71462	0.152972	-0.62527	-0.6136	-0.10895	-0.80702	-0.51104	0.054709	0.959992
-0.71462	-0.38896	-0.04036	0.91556	0.532051	-0.03356	-0.33715	0.000468	1.304517
0.016933	0.821722	0.943743	1.043222	0.557493	1.28847	0.904323	0.040305	0.730977
1.859797	0.724211	0.574712	0.772102	0.365745	0.678716	0.828531	0.002736	0.462786
1.859797	-0.27621	0.023706	0.4494	0.400641	0.741644	0.074959	0.002812	1.017859
1.859797	-0.66816	-1.01735	-1.18001	-0.7779	-0.7263	-0.84539	0.012438	0.511858
-0.2394	1.004623	1.478137	1.727776	0.825486	1.56327	1.308293	0.005652	0.90229
-0.53147	0.879395	1.219073	1.14955	0.620459	0.944715	1.10544	0.000967	0.598615
-0.53898	1.29318	1.800793	1.735687	1.133663	1.672375	1.47938	0.018949	0.66713

-0.2394	0.19785	0.120215	0.010857	0.252176	0.124276	0.182793	0.203493	0.241319
-0.2394	0.581817	0.850225	0.811316	0.522354	0.732548	0.19943	0.07403	0.650795
-0.53878	-0.15782	0.467038	0.868423	0.237892	0.178467	0.144101	0.006516	1.02624
-0.53878	-1.06518	-1.59161	-1.58009	-1.18642	-1.62485	-1.24627	0.043183	0.559673
-0.53878	-0.78372	-0.85479	-1.22712	-0.67451	-1.1147	-1.19539	0.204309	0.552615
-0.53878	-1.12809	-1.69494	-1.71162	-1.23179	-1.45564	-1.22903	0.012858	0.58353
-0.53878	-0.86181	-1.10553	-1.06079	-1.06835	-1.20895	-1.0549	0.003772	0.347144
-1.20871	0.256631	0.787809	0.942433	0.697186	0.579045	0.4647	0.002582	0.685802
-1.20871	0.076181	-0.17478	-0.34178	-0.56675	-0.20387	-0.29532	0.012264	0.642927
-1.20871	-0.52073	-0.60895	-0.22105	-0.23906	-0.47401	-0.74002	0.029827	0.518971
0.544142	-0.26092	-0.15669	0.351521	-0.07179	-0.42119	-0.02726	0.115683	0.772712
0.544142	0.428654	-0.13987	-0.26695	0.417703	0.28801	0.557324	0.001664	0.824273
0.544142	-0.21282	-0.67086	-1.08064	-0.54616	-0.76482	-0.60303	0.00771	0.867822
0.544142	-1.22488	-2.15353	-1.92702	-1.47102	-1.55506	-1.46677	0.022881	0.928653
0.544142	-0.77465	-1.57089	-1.48841	-0.76229	-1.44374	-1.03877	0.018756	0.808603
1.680537	0.415585	0.662369	0.539749	0.212929	0.760014	0.761974	0.007774	0.549045
1.680537	0.292477	-0.45467	-0.24915	0.238849	0.087687	-0.29952	0.105829	0.747143
1.680537	0.834631	0.548211	0.644663	1.181503	1.124335	0.809492	0.013914	0.633292
1.680537	-0.03932	0.396837	-0.1721	-0.05939	-0.41731	-0.34934	0.025776	0.814144
1.680537	-0.29368	-0.29842	-0.67395	-0.29268	0.131639	-0.3284	0.030624	0.805588
1.680537	0.570589	-0.24881	-0.71689	-0.45151	-0.48584	-0.31541	0.074262	1.287479
1.680537	-0.85622	-1.23951	-1.28683	-0.96428	-1.18314	-0.98083	0.067344	0.430611
0.900233	0.300708	0.343313	0.585796	0.291625	-0.04326	0.207653	0.015897	0.629054
0.900233	1.054802	1.374883	1.70297	1.491844	0.819108	0.792703	0.007859	0.910267
0.204377								
0.204377	-0.87351	-1.23592	-1.61692	-1.05518	-1.36445	-1.12471	0.001116	0.743405
0.204377	0.212436	-0.73976	-1.31941	-0.49046	-1.23249	-0.79885	0.028222	1.531849
0.204377	-0.498	-1.1494	-1.42986	-0.74711	-0.6353	-0.99923	0.007912	0.931861
0.204377	0.259434	0.398577	-0.74386	-0.21017	-1.06374	-0.59407	0.143317	1.46232
-0.70468	0.336308	1.182613	1.66131	0.725034	0.838761	0.881885	0.001476	1.325002
-0.69354	0.595857	0.880529	1.310177	0.962458	1.016776	0.653202	0.002783	0.71432
-0.69354	0.292256	1.089164	1.493139	0.82016	0.781154	0.342477	4.38E-06	1.200883
0.728413	0.19807	0.253666	0.536987	0.392472	0.648391	0.30354	0.144793	0.45032
0.728413	1.123557	1.468123	1.387363	0.981215	1.657665	1.40206	0.005986	0.67645
-0.99838	0.928717	1.417733	1.760997	0.95023	1.55569	1.194687	8.99E-05	0.83228
-0.99423	1.096578	1.65265	2.009513	1.068831	1.624105	1.369993	0.002549	0.940682
-0.99423	1.06076	1.553743	1.724217	1.160663	1.65441	1.22918	0.000803	0.663457
-0.663								
-0.663	-0.76612	-1.62952	-1.80168	-1.14839	-1.23174	-1.30385	0.025731	1.035558
-0.663	-0.3223	-0.03545	0.212555	0.353581	0.621401	0.135591	0.006256	0.943704
-0.663	-0.56825	-0.87272	-1.14144	-0.5057	-1.37685	-0.6395	0.119638	0.87115
-0.663	-0.74071	-1.25104	-1.65969	-1.06124	-1.32162	-1.13983	0.001373	0.918981
-0.663	0.139158	-0.54697	-1.0622	-0.58439	-0.83566	-0.75497	0.006726	1.201363
0.074657	1.058391	1.076701	1.159589	1.361063	1.23158	0.894267	0.334255	0.466796
0.074657	-0.83918	-0.91155	-1.09025	-0.44887	-0.94007	-0.55643	0.064715	0.641381
0.074657	0.161943	-0.2303	-1.20683	-0.44826	0.143908	0.025451	0.006258	1.368769
0.45296	0.071884	-0.15387	-0.53401	-0.49488	-0.23255	-0.41989	0.006575	0.605895

0.45296	0.256007	0.042382	0.685776	0.32518	0.432296	0.24254	0.002263	0.643394	
0.45296									
0.259043	-0.10699	-0.18712	-0.4372	-0.32858	-0.05214	-0.05882	0.076864	0.38506	
0.10469									
0.10469	-0.89117	-0.82971	-1.17751	-0.99679	-1.31777	-0.904	0.094723	0.488053	
0.10469	-0.86705	-1.65572	-1.58191	-0.95051	-1.25074	-0.89832	0.010659	0.788676	
0.10469	0.581015	1.046763	1.23039	0.682196	0.750616	0.678328	0.002063	0.649375	
0.259043	0.108503	0.233414	0.226618	0.250031	0.621338	0.497874	0.055829	0.512835	
2.11042	-0.32771	-0.57119	-0.6091	-0.27335	0.143933	-0.04796	0.077898	0.75303	
2.60855	-0.2113	-0.15879	-0.23029	0.357632	0.30238	0.113267	0.001783	0.587922	
2.60855	1.027519	1.049379	0.893366	0.48183	1.017335	0.964359	0.001946	0.567548	
2.60855	0.715577	0.874326	1.079838	0.497811	0.531565	0.74796	0.047158	0.582027	
0.08862	1.117898	1.247923	1.44914	0.985562	1.23664	1.303227	0.053757	0.463578	
2.616293									
2.616293	-0.98799	-1.51982	-1.91035	-1.17921	-1.63202	-1.24354	0.000696	0.922367	
2.616293	0.83104	0.102565	-0.17354	-0.0885	-0.24852	-0.2178	0.039809	1.079557	
2.616293	-0.76534	-1.21504	-1.55177	-0.96468	-1.36638	-1.08603	0.000389	0.786424	
2.616293	-0.78183	-1.55291	-1.60037	-0.83199	-1.45562	-1.23657	0.000701	0.818545	
0.574027	-0.16413	-0.21997	-0.67974	-0.4249	-0.34907	-0.48842	0.117018	0.515607	
-0.32645	0.2464	0.565776	0.654526	0.644969	0.876084	0.662825	0.042188	0.629684	
-0.32645	0.342718	0.385604	0.39282	0.067957	0.510825	0.529518	0.058675	0.461561	
-0.32645	0.720985	0.589311	0.644119	0.90794	1.3425	0.661773	0.028373	0.753189	
0.02797									
0.02797	-0.81488	-1.23152	-1.36008	-0.7933	-1.2181	-0.97613	0.022002	0.566779	
0.02797	-0.49276	-0.6117	-0.82722	-0.17023	-1.25727	-0.53899	0.081651	1.08704	
0.02797	-0.78138	-1.15413	-1.57104	-0.79815	-1.26123	-0.95485	0.000139	0.789658	
0.02797	1.319867	2.255423	1.74398	1.495597	1.88819	1.973123	0.0659	0.935557	
0.697617	-1.02052	-1.08296	-1.61205	-1.23557	-1.02223	-1.36308	0.054828	0.59153	
0.121133	1.294443	1.441233	0.915809	0.910261	1.4088	1.16178	0.024326	0.530973	
0.121133	-1.13084	-1.814	-1.89341	-1.49858	-1.52494	-1.36716	0.002174	0.76257	
0.121133	1.085826	1.679145	0.672704	0.310877	-0.21368	0.870528	0.078259	1.892821	
0.697617	-0.56467	-0.67242	-0.87869	-0.74914	-0.45638	-0.70211	0.010675	0.422306	
0.698533									
0.698533	-0.88157	-1.33247	-1.36635	-0.94269	-0.93462	-0.70878	0.072161	0.657571	
0.698533	-0.60369	-0.99389	-1.27362	-0.67959	-0.94809	-0.80031	0.01256	0.669929	
0.698533	-0.70129	-1.20141	-1.19405	-0.67419	-0.96984	-0.81339	0.015428	0.527225	
1.081223	-0.37074	-0.36755	-0.98733	0.368914	-0.59617	-0.44113	0.037249	1.356246	
1.081223									
1.081223	-1.01653	-1.67335	-1.72039	-0.74505	-1.44256	-1.02285	0.039475	0.975341	
1.081223	-0.21016	-0.55236	-0.38896	-0.13734	0.096396	-0.30356	0.009117	0.648756	
1.304837	-0.8699	-1.13744	-1.48234	-1.08098	-1.39214	-1.19055	0.029905	0.612444	
1.304837	-0.23346	-0.02631	0.10155	-0.21063	0.318785	0.174266	0.005627	0.552241	
-0.84589	0.090889	0.603271	0.947426	0.899568	-0.02591	0.043975	0.012924	0.973334	
-0.08354	1.109396	1.302013	0.541353	0.749976	0.74626	0.636247	0.053004	0.76066	
-0.08354	0.283455	0.550968	1.020424	0.522158	0.396095	0.093979	0.029806	0.926445	
-0.08354	-0.15807	-0.01741	0.007167	-0.10167	-0.01869	-0.06217	0.066587	0.165233	
-0.08354	0.535973	0.62496	0.554511	0.548509	1.384995	0.818497	0.129089	0.849022	

-0.05071	0.568396	0.722521	0.69442	0.448405	1.251412	1.009442	0.103005	0.803007
-0.84589	-0.40021	-0.5195	-0.54162	-0.15735	-0.24571	-0.40175	0.064431	0.384269
-0.84589	-0.34182	-0.20462	-0.34099	-0.68939	-0.28408	-0.53041	0.020853	0.484767
-1.38427	0.411431	0.341953	0.648183	0.276396	0.558132	0.417182	0.115128	0.371787
-1.09583	0.46714	1.207729	1.577163	1.025684	0.999239	0.790585	0.007633	1.110023
-1.09583	0.139581	0.492258	0.524646	0.355355	0.641787	0.350235	0.006554	0.502206
-0.30176	0.122423	0.328865	0.185595	0.086245	0.202425	0.277565	0.058571	0.24262
0.079683	-1.0335	-1.23781	-1.30008	-0.79648	-1.22439	-0.96256	0.078444	0.503598
0.079683	0.475289	0.773997	0.541816	0.309467	0.407126	0.576861	0.162862	0.464529
0.390412	0.264803	0.242474	0.356022	0.631331	0.466256	0.460178	0.007268	0.388857
0.390412	-0.16579	-0.88468	-1.41323	-0.80969	-0.94895	-0.46516	0.013549	1.247439
0.390412	-0.42806	-0.59818	-1.214	-0.88702	-1.18957	-0.84821	0.166786	0.785941
0.390412	-1.0114	-1.48969	-1.55778	-0.96004	-0.97882	-0.80779	0.010571	0.749991
0.390412	-1.17606	-1.78785	-2.13599	-1.42259	-1.95326	-1.6102	0.009568	0.959925
0.390412	-1.91395	-2.11578	-2.44947	-1.68653	-1.91766	-1.66516	0.007608	0.784303
0.390412	-1.37136	-1.77788	-1.86139	-1.44219	-1.59177	-1.37518	0.042551	0.490033
0.390412	-0.96599	-1.32891	-1.29849	-1.03509	-1.18198	-0.99658	0.00193	0.362923
-0.69923	-0.45185	-0.4104	-0.20596	-0.21083	-0.40985	-0.55079	0.082448	0.344825
-0.69923	2.039418	1.673462	1.746634	1.923946	1.084263	0.534863	0.141938	1.504555
-0.69923	0.315227	0.641239	0.719284	0.425825	1.01401	0.730883	0.020752	0.698782
0.03436								
0.03436	-0.65246	-0.64633	-0.55365	-0.42981	-0.6058	-0.45455	0.184788	0.222655
0.03436	0.319667	-1.3313	-1.19748	-0.94477	-1.89199	-1.0303	0.064333	2.211657
-0.44892								
-0.44892	-0.57979	-0.62889	-0.58913	-0.63944	-0.76434	-0.70047	0.205372	0.184551
-0.44892	-0.50491	-0.62371	-1.04609	-0.75234	-1.07095	-0.85059	0.030467	0.566045
-0.5037	0.611486	0.647121	0.922687	0.782863	0.500487	0.787111	0.124401	0.4222
-0.5037	0.514648	0.67434	0.823797	0.660038	0.665382	0.585884	0.139973	0.309149
-0.5037	-1.10997	-1.80195	-2.04784	-1.23592	-1.41459	-1.08724	0.009734	0.960595
-0.5037	-0.51937	0.138322	-0.7803	-0.31926	-0.99773	-0.44508	0.115751	1.136049
-0.5037	-1.1498	-1.69851	-1.9945	-1.41206	-1.59849	-1.5008	0.007778	0.8447
-0.5037	-0.69533	-1.3078	-1.51505	-0.84716	-1.36355	-0.92339	0.006201	0.819724
0.041677	-0.46571	-0.816	-0.98047	-0.11937	-0.50535	-0.58577	0.006905	0.861109
0.436473								
0.436473	-0.38051	-0.53858	-0.70493	0.066795	-0.20069	-0.27318	0.001694	0.771727
0.436473	-0.88178	-1.25	-1.08585	-0.63109	-0.89004	-0.79961	0.032441	0.618916
0.041677	-0.32585	-0.4588	-0.37197	0.241284	0.194384	-0.00806	0.008705	0.700087
-0.97856	1.792763	1.977893	1.665687	1.24108	1.514275	1.297367	0.139467	0.736813
-1.23575	0.23221	0.626981	1.042463	0.746157	0.382047	0.328803	0.000104	0.810253
-1.18384	3.167463	2.810033	2.828843	2.61966	2.265825	1.81954	0.057677	1.347923
1.606143	-0.43927	-0.77389	-0.70857	-0.56187	-0.65602	-0.5084	0.013314	0.334623
1.606143	1.04765	1.171747	1.105229	0.527036	1.10464	1.049194	0.068991	0.644711
0.53262	0.794379	0.705277	0.632171	0.576116	1.010643	0.808618	0.156251	0.434527
0.53262	0.028418	0.277004	-0.77594	-0.33032	-0.50526	-0.30784	0.124977	1.052947
0.53262	-0.89981	-1.08945	-1.20575	-0.84519	-0.9348	-0.69149	0.03244	0.514261
-0.3178	0.92758	0.760703	0.700164	1.014712	1.285873	0.590256	0.233079	0.695617
-0.3178	-0.66645	-0.89237	-0.9208	-0.21045	-0.34747	-0.05074	0.014369	0.870052

0.504853								
0.504853	1.3452	1.543443	1.66663	1.027896	1.430515	1.16063	0.007023	0.638734
0.20239	1.493837	1.354253	1.143321	0.517162	1.144844	0.903119	0.014043	0.976675
	1.089965	1.368887	1.192257	0.986918	0.946883	0.770561	0.009744	0.598326
	0.873408	1.013753	0.79257	0.524894	0.794248	0.801086	0.009126	0.488859
0.20239	1.159397	1.230407	1.40979	1.474473	1.603245	1.397857	0.00099	0.443848
-0.34364								
-0.34364	0.312675	0.271409	0.357452	0.403045	0.73818	0.527987	0.01561	0.466771
-0.34364	0.134466	-0.00291	0.016585	0.021371	-0.04279	-0.00207	0.102635	0.177259
-0.34364	-0.95273	-1.43431	-1.94164	-1.05902	-1.35565	-1.25236	0.000951	0.988918
-0.34364	-0.90942	-1.50743	-1.61763	-0.82302	-1.07821	-0.70291	0.066078	0.914719
-0.34364	0.211137	-0.14916	-0.68796	-0.26775	-0.52426	-0.31343	0.002989	0.899095
-0.34364	-1.04268	-1.49618	-1.70602	-1.3823	-1.63011	-1.34457	0.028335	0.66334
-0.39409								
-0.39409	-0.45328	-0.61287	-0.28477	-0.16267	-0.2208	-0.22205	0.006165	0.450201
-0.39409	-0.37954	-0.38583	-0.21551	-0.12919	-0.22766	-0.25859	0.042199	0.256638
-0.50991								
-0.50991	2.310363	2.22297	1.786727	1.197	1.231363	1.527927	0.074262	1.113363
-0.19349								
-0.19349	-0.77073	-0.88716	-0.599	-0.25922	-0.51404	-0.48181	0.013534	0.62794
-0.19349	0.755374	1.363637	1.56517	0.839431	0.888006	0.634383	0.000576	0.930787
-0.64358	0.217037	0.524978	0.773047	0.418023	0.725497	0.58887	0.037378	0.55601
-0.47162	0.843809	0.980558	0.942095	0.612362	1.26113	1.042385	0.0147	0.648768
-0.47162								
-0.64358	-1.18552	-1.43751	-1.53922	-0.84778	-1.20108	-0.8987	0.005728	0.691436
-0.64358	0.384746	1.571413	0.88332	0.78485	0.504074	0.371647	0.0039	1.199767
	-0.33781	-0.53067	-0.85599	-0.55758	-0.54763	-0.39487	0.001471	0.51818
-0.36173								
-0.19673	0.285295	0.258255	0.236703	0.04376	0.343159	0.428262	0.008085	0.384503
-0.19673	2.981683	3.065933	2.661957	2.3968	2.429655	2.60026	0.06478	0.669133
-0.34721								
-0.08983	0.106777	0.378401	0.448375	0.101065	0.225691	0.087451	0.079735	0.360924
-0.76877	0.932133	1.5971	1.750713	1.029036	0.918701	0.864498	0.006988	0.886215
-0.95641	0.774315	1.41073	1.71774	0.817691	0.548527	0.675991	0.010576	1.169213
0.222298								
0.222298	0.099187	-0.0023	-0.07852	0.552091	0.415281	0.332611	0.009348	0.63061
0.222298	-0.60926	-0.66931	-0.66194	-0.71291	-0.30426	-0.30911	0.006577	0.408648
0.060117								
0.060117	0.389702	0.57351	0.999869	1.012302	0.999403	0.644358	0.022197	0.6226
-0.88767	1.197477	1.67042	1.877217	0.813308	1.537865	1.349823	0.009934	1.063909
-0.88767	0.153574	0.055668	-0.08103	-0.23484	0.059398	0.14607	0.007005	0.388415
-0.88767	-0.03882	0.427591	0.364126	0.479397	-0.48855	0.048202	0.058116	0.96795
-0.87754	0.362332	0.56368	0.915104	0.47407	0.963539	0.723708	0.059193	0.601207
-0.87754	0.810311	1.136988	1.511503	0.876999	1.66083	1.306753	0.012418	0.850519
0.263253	3.442837	3.26399	2.81227	1.606604	3.891505	2.851447	0.092606	2.284901
0.263253	-0.08627	-0.08174	-0.21011	0.155519	-0.33131	0.303678	0.251364	0.634989

0.263253	-1.14769	-1.62729	-1.86468	-1.05242	-1.33046	-1.11575	0.000292	0.812253
0.263253	0.555845	0.256532	0.264043	0.184205	0.203225	0.184233	0.013412	0.371639
-0.58265	3.40372	3.316747	2.5436	2.582387	2.60156	2.518627	0.013018	0.885093
-0.58265	0.111652	-0.27043	0.2176	0.426657	-0.08192	-0.22353	0.120933	0.697085
-0.58265	0.422873	0.998215	1.293507	1.020045	1.20145	0.623786	0.000382	0.870634
-0.01426								
-0.01426	-0.03702	0.287051	0.296831	0.389855	0.272427	0.247481	0.044448	0.426872
-0.01426	0.71433	1.574933	1.686177	0.733544	0.130971	-0.00703	0.003487	1.693209
-0.73964								
-0.71063								
-0.71063	-1.39512	-1.9341	-2.02646	-1.44951	-1.94811	-1.55835	0.014993	0.631337
-0.71063	0.275264	-0.05064	-0.54128	-0.32549	-1.00757	0.005878	0.026151	1.282839
-0.71063	-1.72738	-2.1514	-2.27382	-1.47534	-1.94076	-1.58367	0.000326	0.798484
-0.71063	-0.64896	-0.57881	-0.12246	-0.27536	-0.81662	-0.55093	0.020536	0.694158
-0.19673								
-0.36173	0.675165	1.094248	1.093247	1.438343	0.987737	1.244116	0.143908	0.763178
-0.36173	-0.18893	-0.08973	-0.01884	0.135975	-0.19415	-0.07698	0.089942	0.330121
0.3616	0.399788	0.480035	0.909751	0.639834	0.442486	0.374176	0.002875	0.535575
0.3616	-1.17413	-1.27108	-1.76634	-1.24373	-1.45202	-1.34876	0.001723	0.59221
0.3616	-0.10988	0.134088	0.187502	0.207902	0.623022	0.080014	0.01	0.732905
-0.55508								
-0.55508	-1.23223	-1.95568	-1.96455	-1.21893	-2.02978	-1.59228	0.058252	0.810849
-0.55508	-0.1258	-0.14042	-1.01818	-0.48804	-0.72109	-0.78147	0.007126	0.892382
-0.55508								
-0.55508	-1.20427	-1.63643	-1.92437	-1.34852	-1.54417	-1.49235	0.011878	0.720098
-0.55508	0.610809	0.521167	0.576125	0.09536	0.790008	0.592353	0.003638	0.694648
-0.8675	0.680092	1.065035	1.69727	1.094207	0.879396	0.676903	0.013916	1.020367
-0.8675	-0.93181	-1.09819	-0.97191	-0.7811	-1.0581	-0.79766	0.109141	0.317094
-0.8675	0.463869	-0.8545	-1.11435	-0.7311	-0.79984	-1.22749	0.070764	1.691361
-0.59944	1.185837	1.379713	1.168003	0.408484	0.637668	0.956797	0.002218	0.97123
0.47407								
0.47407	-0.7538	-0.86329	-0.71988	-0.46322	-0.39902	-0.5556	0.001795	0.464271
0.47407	0.354503	0.362897	0.648109	0.242711	0.789132	0.606971	0.004583	0.546421
-0.62182	-0.45615	-0.2536	0.192102	0.280269	-0.11953	-0.31089	0.070387	0.736416
-0.62182	0.894869	1.33707	1.599373	1.077176	1.422155	1.196543	0.00075	0.704505
1.666037	0.273089	0.543245	0.550142	0.21339	0.155532	0.399137	0.041225	0.39461
1.666037	-0.24406	-0.37278	-0.22293	-0.09385	0.085525	-0.13708	0.013861	0.458304
1.666037	0.383059	0.264532	0.017014	-0.17445	0.694322	0.589144	0.04513	0.868776
-0.19047	0.102436	0.296175	0.602953	0.113903	0.636691	0.708749	0.022789	0.606313
-0.62182	0.686809	1.043807	1.455373	1.071909	1.455105	1.024429	0.009382	0.768565
-0.19047	1.315447	1.627187	1.767517	1.28294	1.93367	1.50793	0.003661	0.65073
-0.19047	-0.68293	-1.06514	-0.71529	-0.66097	-0.72342	-0.80028	0.078623	0.404171
1.942683								
1.942683	1.355397	1.087327	0.626856	0.970527	1.044597	1.027006	0.001811	0.728541
1.944663								
1.944663	-1.09065	-1.75339	-1.72524	-1.13287	-1.33961	-1.18201	0.021493	0.66274
1.944663	-0.71946	-1.26143	-1.44819	-0.78755	-1.06541	-0.83958	0.008849	0.728732

1.944663	-0.71692	-0.8346	-1.16355	-0.48726	-1.26838	-0.86032	0.101748	0.781115
1.944663	-0.94925	-1.3648	-1.61255	-1.0918	-1.2803	-1.16798	0.01076	0.663299
1.944663	-1.14347	-1.6227	-1.94825	-1.41605	-1.78477	-1.23638	0.001945	0.804779
1.944663	-1.42632	-2.11753	-2.14558	-1.51921	-1.98187	-1.6683	0.048502	0.719266
1.944663	-1.12658	-1.88241	-1.912	-1.05338	-1.3471	-1.12552	0.003482	0.858627
1.944663	-1.05879	-1.8157	-1.91494	-1.06092	-1.19758	-1.34846	0.026861	0.85615
1.944663	-1.02264	-1.15318	-1.74164	-0.89642	-1.32008	-1.2037	0.030632	0.845214
1.944663	-1.00799	-0.99899	-0.80759	-0.9335	-1.29338	-1.12525	0.078387	0.485786
1.944663	-0.52215	-1.06838	-1.76374	-0.80068	-1.35997	-1.1217	0.019109	1.24159
1.944663	-0.4688	-0.37922	-0.05527	0.293162	0.170537	-0.12124	0.003579	0.76196
-0.38755								
-0.38755	-0.85042	-0.9253	-0.87612	-0.40014	-0.61334	-0.66869	0.065396	0.525159
-0.38755	0.964845	1.37604	1.524613	1.323437	1.38571	1.120408	0.043199	0.559769
0.095015								
0.095015	-0.05823	0.01087	0.087612	0.069069	0.272514	-0.02323	0.015844	0.330744
0.095015	-0.96903	-0.97618	-0.95712	-0.32329	-1.40471	-0.58335	0.183409	1.081415
0.095015	-1.04402	-1.52459	-1.74364	-1.00861	-1.31482	-0.99138	0.039437	0.752258
0.095015	-0.99092	-1.38478	-1.57073	-0.96479	-1.22644	-1.23908	0.002795	0.605944
0.095015	-0.78477	-1.36921	-1.73802	-0.9689	-1.21484	-1.26815	0.003897	0.953256
0.095015	-0.54923	-0.89575	-1.26134	-0.85169	-0.89504	-0.68507	0.016084	0.712102
0.095015								
0.095015	-0.67794	0.330304	-0.86825	-1.00421	-1.3685	-1.07106	0.007436	1.698799
0.095015	-1.19883	-1.56869	-1.78062	-1.10932	-1.4422	-1.23143	0.006599	0.671301
0.095015	0.318851	-0.24745	-0.25914	-0.17866	-0.27424	-0.39678	0.17496	0.715626
-0.29137	0.329497	0.622412	0.782004	0.558415	1.030804	0.714035	0.049664	0.701307
-0.29137	-0.19931	-0.05584	-0.26187	-0.14541	-0.42927	-0.29922	0.074232	0.373435
-0.29137	0.532479	0.344339	0.602869	0.877267	1.14302	0.657904	0.035335	0.798681
0.276133								
0.276133	-0.43063	-0.92507	-0.96172	-0.58904	-1.18269	-0.85491	0.128202	0.75206
0.276133	-0.19127	0.248336	0.688	0.29442	-0.51116	-0.14812	0.088163	1.199161
0.211807								
0.211807	0.210746	0.178556	0.62828	0.695532	0.734427	0.523868	0.006721	0.555871
0.211807	0.85767	0.928842	1.225944	1.087352	1.249264	0.876075	0.00951	0.391594
2.667297	0.969369	1.371805	1.674383	0.967334	2.03117	1.690463	0.019878	1.063836
2.667297	-0.35355	-0.36398	-0.22156	-0.36953	-0.54438	-0.36334	0.047243	0.32282
2.667297	-0.58927	-1.09452	-1.11627	-0.64739	-1.23402	-0.87839	0.07843	0.644749
2.667297	-1.05468	-1.70116	-1.88502	-0.97176	-1.65369	-1.13281	0.019656	0.913255
0.810237	-0.30423	-0.29657	-0.30929	-0.28869	-0.55443	-0.20014	0.259344	0.354289
-0.16174	0.575557	1.068273	1.389557	0.954148	1.23434	1.336757	0.004131	0.814
-0.16174	0.037659	-0.08973	-0.27336	-0.16345	0.013424	-0.01522	0.010744	0.311022
-0.16174	-1.05439	-1.29651	-1.22038	-1.21139	-1.4357	-1.13451	0.028592	0.381304
-0.16174	-0.50872	-0.46008	-1.16101	-0.85724	-0.73934	-0.52073	0.025307	0.700931
-0.22693	0.258659	0.452668	0.409189	0.560287	0.797369	0.32184	0.007891	0.53871
-1.5089								
-1.5089	1.016537	1.707593	1.518317	1.1081	0.596105	0.416122	0.000147	1.291471
0.65768								
0.65768	-1.20255	-1.42177	-1.50491	-1.01656	-1.60228	-1.13941	0.102548	0.585717

0.65768	0.372254	0.452163	0.488121	0.465841	0.575538	0.333718	0.161991	0.24182
-0.79203								
-0.79203	-0.36975	-0.53605	-0.3783	-0.31708	-0.63163	-0.28882	0.048013	0.342804
-0.79203	0.261771	0.937824	1.101343	0.494594	0.343798	0.035883	0.003061	1.065461
0.40724	0.306727	0.360742	0.600069	0.591225	0.425876	0.451257	0.024941	0.293342
0.40724	-0.54312	-0.82604	-0.93684	-0.73733	-1.13006	-1.04184	0.004416	0.586935
0.40724	-0.35148	-0.61137	-0.53521	-0.3364	-0.04071	-0.15993	0.002139	0.570665
-0.26064								
-0.26064	-0.51024	-0.71844	-1.04787	-0.59584	-0.33006	-0.39276	0.001328	0.717809
-0.26064	-0.72437	-0.92334	-0.70899	-0.54871	-0.75956	-0.61723	0.005882	0.374623
-0.60465	0.425414	0.728077	0.977509	0.785227	0.933489	0.692182	0.011168	0.552096
0.304167	1.220908	1.43871	1.556027	1.052332	1.555945	1.041693	0.004156	0.514334
-0.74007	0.341768	0.59935	1.241773	1.306014	0.6838	0.710985	0.006598	0.964246
-0.74007	0.223107	0.443961	0.811166	0.761923	0.851723	0.661102	0.004579	0.628616
-1.13415								
-1.13415	0.552667	0.689585	1.181113	1.19565	0.831148	0.721251	0.00258	0.642983
-0.18708	0.907065	1.256044	1.73144	1.19123	1.49885	1.258337	0.008341	0.824375
0.304167	0.983433	1.344773	1.856693	1.185677	1.84675	1.452883	0.013864	0.87326
-0.72087								
-0.72087	-0.28833	-0.45458	-0.0612	0.063359	-0.33992	-0.09282	0.011965	0.517942
-0.72087	0.133653	0.293438	0.757549	0.739514	0.681709	0.296166	0.008025	0.623896
0.112397								
0.112397	0.040754	-0.7824	-1.16504	-0.45493	-1.30651	-1.14011	0.061312	1.347264
0.112397	-0.22911	-0.72292	-1.25621	-0.5449	-1.10727	-0.81047	0.012653	1.027103
-0.18708	-0.2531	-0.43497	-0.49308	-0.402	-0.14804	-0.20401	0.079173	0.345047
-0.18708	-0.37287	-0.35769	-0.08485	-0.06412	0.290011	-0.03916	0.011839	0.66288
	0.974821	1.92089	1.584543	1.32677	0.200793	0.056037	0.000369	1.864853
0.35965								
0.35965	0.121976	-0.294	-0.44084	0.223705	0.530379	0.588608	6.61E-05	1.029444
1.792893								
1.792893	1.353943	1.686077	1.505381	1.00604	1.22407	1.19423	0.027734	0.680037
-0.63675								
-0.63675	1.13537	1.587113	1.76397	1.22478	1.4738	1.081491	0.092293	0.682479
0.863627								
0.863627	0.941473	1.076568	1.04978	0.65112	0.893971	1.042695	0.003367	0.425448
-0.66517	0.050733	0.481856	0.872376	0.051874	0.23117	0.38068	0.02176	0.821643
-0.66517	-1.11907	-1.38906	-1.64318	-1.14512	-1.40369	-1.20168	0.005196	0.524113
-0.66517	-0.29272	-0.27762	-0.2596	-0.24146	-0.34877	-0.22617	0.158969	0.1226
-0.66517	0.851796	1.48914	1.61878	0.912263	1.109975	0.951638	0.024714	0.766984
	-1.59751	-2.12914	-2.19656	-1.36291	-2.61639	-1.90527	0.012654	1.253478
-0.61721	0.380571	0.968794	1.217317	0.209693	0.762601	0.724231	0.005512	1.007624
-1.2464	0.630529	0.845211	1.043943	0.447161	0.68058	0.709903	0.036859	0.596782
-1.2464	1.270521	1.378937	1.63084	0.984916	1.005072	0.90837	0.006264	0.72247
-0.21932								
-0.64633	-0.58344	-0.46747	-0.96883	-0.70203	-0.79191	-0.77536	0.140113	0.501359
-0.64633	0.375541	0.620195	1.446277	0.972144	0.827619	0.496682	0.00084	1.070736

-0.40037	0.364551	0.363539	0.576397	0.030778	0.556783	0.638449	0.020037	0.607671
-0.40037	-0.33816	-0.4011	-0.24753	-0.16402	0.230791	0.08419	0.006611	0.63189
-0.40037	-0.81637	-1.00018	-0.71175	-0.34716	-0.75809	-0.8003	0.01817	0.653026
-1.44068								
-1.44068	1.199145	1.437493	1.98869	1.405907	1.017025	0.943429	0.01248	1.045261
	-1.61168	-2.17376	-2.40132	-1.69198	-2.44126	-2.08591	0.025573	0.829576
-0.782								
-0.782	-0.78512	-1.31361	-1.15513	-0.5759	-0.90352	-0.8682	0.001787	0.737708
-0.782	-0.06762	-0.46932	-0.46494	-0.21115	-0.63151	-0.4252	0.036297	0.563891
1.228033	0.647295	1.114777	1.365186	1.141803	1.38695	0.845305	0.061501	0.739655
-0.7041	-0.36057	-0.45174	-0.39889	-0.38067	-0.45078	-0.3734	0.323782	0.091168
-0.72514	-0.0786	0.283839	0.731468	0.039496	0.046525	0.030422	0.011908	0.810071
-0.72514	-0.08883	-0.68806	-0.81949	-0.58697	-0.58732	-0.80979	0.071302	0.730662
	-0.08675	0.379451	0.829425	0.360064	-0.20334	-0.26344	0.011624	1.09287
-1.18384	1.468737	1.72497	1.969113	1.359747	1.178515	1.014178	0.007276	0.954935
-0.37863								
-0.37863	1.094471	0.914141	1.058591	0.269782	0.939219	0.927768	0.060128	0.824689
3.18833								
3.18833	-0.34479	-0.54714	-0.35988	0.020035	-0.68692	-0.29285	0.019368	0.706952
3.18833	-0.30343	-0.88896	-1.2379	-0.57193	-0.95582	-0.34449	0.012049	0.934464
-1.20148	0.352841	0.331852	0.237029	0.234865	0.324217	0.387947	0.048958	0.153082
-1.20148	0.791689	1.186077	1.488303	1.040117	0.964849	0.886517	0.128102	0.696614
0.192997								
0.192997	-0.79137	-0.61082	-0.57193	-0.56056	-0.14953	-0.3192	0.009589	0.641838
0.43608								
0.43608	0.352844	-0.03092	-0.54672	0.027887	1.224236	-0.2061	0.120366	1.770955
0.43608	-0.72827	-0.85681	-1.36169	-0.68416	-0.96951	-0.82661	0.002621	0.677526
-0.16752	0.688276	1.249003	1.62461	0.932103	1.176687	1.119357	0.006816	0.936334
0.011737								
0.011737	1.069017	1.339977	1.528093	0.682146	1.83737	1.396783	0.002082	1.155224
	1.048788	1.731257	1.790543	1.29179	1.65976	1.11096	0.03914	0.741756
	0.917953	1.168971	1.439524	1.299505	1.575765	1.167707	0.061797	0.657812
-0.16752	-0.68149	-0.96563	-0.91994	-0.85431	-1.17128	-1.00582	0.17044	0.48979
-0.16752	-0.76798	-1.10451	-1.19886	-0.67742	-0.85006	-0.83871	0.003136	0.521447
-0.16752	-0.21836	-0.35748	-0.47918	-0.76292	0.027472	-0.1322	0.007535	0.790388
-0.16752	-1.00712	-1.41792	-1.53128	-1.05551	-1.03451	-1.03033	0.006279	0.524168
-0.16752	0.453362	0.962997	0.445048	0.730313	0.955399	0.490629	0.081556	0.517949
-0.02148	-0.24808	-0.12029	-0.33429	-0.35235	-0.33047	-0.22499	0.153902	0.23206
-0.57806	2.156823	1.972424	1.334843	1.76625	1.613875	1.683678	0.04798	0.82198
-0.57806	-0.31771	-0.42765	-0.3975	-0.01174	-0.3443	-0.44844	0.242928	0.436703
-0.57806	-0.8229	-0.91468	-0.35258	-0.25042	-0.41373	-0.62907	0.015381	0.664253
-0.02148	0.683213	0.912462	1.384127	1.20454	1.291295	1.050513	0.005693	0.700914
-0.70012								
-0.70012	0.945234	1.392597	1.508859	1.372874	1.891455	1.017381	0.096983	0.946221
0.164495								
0.164495	0.085604	0.269512	0.678224	0.502453	0.480489	0.217571	0.056873	0.59262
0.164495	-1.13355	-1.70592	-1.97407	-1.34142	-1.78151	-1.51627	0.005391	0.840518

0.164495	-1.00368	-1.55538	-1.99434	-1.3265	-1.00101	-0.86532	0.005094	1.129027
0.164495	0.316651	0.676015	-0.34411	0.501124	0.534325	0.218866	0.049565	1.020123
0.164495	0.044045	-0.79003	-1.24696	-0.69646	-1.09605	-1.00547	0.036444	1.291007
0.164495	0.481472	0.5833	0.06548	-0.48056	-0.39969	-0.67506	0.054262	1.258357
-1.20072	0.970727	1.059679	1.31119	0.752568	0.544838	0.544532	0.037976	0.766658
-1.20072	0.890368	1.311083	1.608433	1.050508	0.961269	0.893564	0.053522	0.718065
-0.10701	0.083205	0.056125	-0.37252	-0.26261	0.195922	0.272369	0.006883	0.644893
-1.20072								
-0.10701	-0.38467	-0.20321	0.262954	-0.1822	-0.1379	-0.15314	0.00163	0.647619
-0.10701	0.766598	0.82373	-0.00838	-0.0806	0.714351	0.628722	0.006781	0.904334
-1.33829	0.26298	0.197253	0.653689	0.246091	0.548444	0.572508	0.008827	0.456436
-1.33829	-0.87832	-1.11467	-1.11256	-0.73583	-1.18024	-0.99006	0.041848	0.444406
-1.33829	-1.07908	-0.95305	-0.01509	0.096638	-0.77567	-0.90163	0.026898	1.175717
-0.14596	0.294478	-0.53043	-0.86783	-0.4979	-0.71493	-0.68563	0.020485	1.162313
-0.14596								
-0.07909	0.667432	0.927697	1.035362	0.368269	1.180226	1.122065	0.043397	0.811957
-0.07909	-0.23118	-0.22864	0.130544	0.284923	0.319758	0.219255	0.005168	0.550935
-0.07909								
-0.91128	0.567115	0.850686	1.173339	0.654223	0.922942	0.70353	0.008113	0.606224
-0.91128	-0.906	-0.83777	-0.67907	-0.71487	-1.1841	-0.99103	0.087911	0.505023
-0.91128	-0.06788	0.114026	0.802791	0.51806	0.032196	-0.21081	0.000954	1.013603
0.105263								
0.105263	0.555766	1.114393	1.487933	0.874885	0.652278	0.667996	0.014316	0.932167
0.120843	-0.66211	-0.52421	-0.65313	-1.07036	-0.90825	-0.94685	0.313308	0.546157
0.120843	-0.75501	-0.58746	-0.36068	-0.18278	-0.2204	-0.24621	0.040124	0.572229
-0.32754								
0.006397	1.32945	1.78708	1.859887	1.274815	1.302725	1.440043	0.060604	0.585071
-0.32754	1.141777	1.460157	1.588307	0.997709	1.067769	1.163218	0.035376	0.590598
0.006397	0.616948	0.624069	0.839738	0.924457	-0.00433	0.915178	0.130088	0.928782
0.006397	1.163537	1.054547	1.01944	0.721536	0.997908	1.17212	0.038641	0.450584
0.11104								
0.11104	0.122645	0.045473	0.251092	0.042946	-0.17941	-0.04082	0.029894	0.4305
-0.8126								
0.456337								
0.456337	1.034286	1.533867	1.51716	0.723358	1.27954	0.905125	0.003798	0.810509
-0.54974	0.347036	0.592648	0.832551	0.331378	0.332582	0.320026	0.021625	0.512525
-0.54974	-1.27346	-1.94426	-1.99501	-1.26881	-1.74783	-1.55708	0.006476	0.726203
-0.54974	-1.01409	-1.40664	-1.54106	-1.08292	-1.49689	-1.20352	0.056308	0.526969
-0.02965								
-0.02965	0.375209	0.019184	-0.04663	-0.08077	0.021373	-0.1097	0.028422	0.484909
-0.36255	0.198599	0.353328	0.489231	0.295926	0.290912	0.308509	0.069157	0.290632
1.07867								
1.07867	-0.69069	-0.95859	-1.17519	-0.68563	-1.11002	-0.67908	0.018953	0.496113
1.07867	1.105344	0.978838	0.892704	0.52819	0.485871	0.454988	0.13758	0.650356
-0.56107	0.143683	0.288893	0.511031	0.102316	0.070185	0.20917	0.048954	0.440846
-0.56107	1.344501	1.270121	0.887719	0.845967	0.617889	0.584932	0.171152	0.759568
-0.14426								

-0.14426	-0.67719	-1.00688	-1.28484	-0.92191	-1.30037	-0.96834	0.002297	0.623182
-0.14426	-0.68362	-1.24947	-1.29506	-0.93882	-1.40109	-1.12854	0.07701	0.717461
-0.58427	0.587681	0.809106	0.979386	0.838485	1.09629	0.800985	0.077094	0.508609
	0.213722	0.88866	1.26398	0.801789	0.956954	0.62306	0.000457	1.050258
	0.902998	1.515292	1.525217	0.965965	1.871815	1.361407	0.012773	0.968817
-0.58427	0.105564	0.374432	0.096737	-0.14439	0.272882	0.170798	0.003589	0.518821
-0.58427	-0.33749	-0.26019	0.473817	-0.03478	-0.33224	-0.59607	0.005233	1.069883
-0.67803	0.444857	0.991249	1.413657	0.320354	0.617478	0.482471	0.003927	1.093303
-1.09014								
-0.67803								
-1.09014	1.250853	1.866793	2.085337	1.223363	1.1218	0.898223	0.000913	1.187114
-0.39547								
-0.39547	0.515365	-0.05599	-0.418	0.107791	0.353367	-0.18311	0.072126	0.933365
-0.39547	-0.23496	0.320039	1.058972	0.598013	0.054171	-0.37006	0.00015	1.429031
1.647065	0.830677	0.935047	0.68184	0.085764	0.278359	0.817469	0.005448	0.849283
1.647065	0.921458	1.740671	1.21265	1.331756	1.475551	1.248324	0.167911	0.819212
0.17272								
0.10488								
0.10488	1.180593	1.214677	1.053904	0.889694	1.21475	1.496607	0.033617	0.606913
0.781963								
0.781963	-0.33305	-0.61739	-1.11024	-1.00658	-1.01116	-0.83289	0.001118	0.777191
0.781963	0.240061	0.390957	0.561273	0.728176	0.759335	0.548414	0.007981	0.519274
0.797923	0.111638	0.417111	0.55461	0.561445	1.042335	0.64751	0.008592	0.930697
0.341097	0.300427	0.750193	0.632117	0.289352	1.10396	0.79169	0.039737	0.814608
0.797923	-0.26003	-0.07758	0.244374	0.008046	-0.15299	-0.20421	0.012721	0.504402
-0.40769	1.328957	1.985037	1.918868	2.074399	0.999791	1.6223	0.138249	1.074609
-0.47895	2.633837	2.775313	2.8727	2.424165	1.460185	2.018103	0.101369	1.412515
-0.43393	2.25843	2.419377	2.032023	1.860003	1.524875	1.514133	0.003908	0.905243
0.053483	5.586207	5.664103	4.846123	4.777377	5.04499	4.74526	0.069132	0.918843
-0.82685	6.942223	6.607903	5.57418	5.6728	5.11823	5.518167	0.003028	1.823993
0.414117	4.147813	5.03868	3.181957	3.517897	3.76701	3.506533	0.041451	1.856723
-0.50135	6.52747	6.325769	5.48158	5.480909	5.466725	5.239963	0.019038	1.287507
0.827622	5.121013	5.499893	4.32663	4.489997	3.55863	3.657892	0.00342	1.941263
0.10157	5.605747	5.564183	4.57239	4.842873	4.06416	3.951003	0.000744	1.654743
-0.84003								
-0.46675	6.35572	6.390643	5.31108	5.67933	5.001205	4.9687	0.005603	1.421943
0.7892	0.626696	0.344459	-0.01894	-0.15009	0.359642	0.864903	0.007582	1.014996
	3.855333	3.9672	2.45297	2.74902	2.54215	2.22089	0.013203	1.74631
-0.42912								
	2.82867	2.99219	1.557117	2.131323	1.634145	1.790487	1.95E-05	1.435073
-0.35364	3.12881	3.766827	2.098717	2.69231	1.957535	1.887333	0.024431	1.879493
-0.4639								
1.295323								
-0.13758								
-1.09207								
0.421363								
-0.96695	5.900043	6.16716	5.25145	5.08024	4.665165	5.140657	0.054567	1.501995

-0.28538	0.336468	0.528108	0.121734	-0.25035	-0.20334	-0.02368	0.008795	0.778458
-0.58742	0.721709	1.227656	1.02317	0.572309	0.499848	0.322627	0.024062	0.905029
-0.51564								
-0.66704	1.188391	1.685493	1.776683	1.042308	1.682465	0.996147	0.01995	0.780536
0.043007	0.669388	1.074016	1.390441	1.093173	1.48134	1.293033	0.035061	0.811952
0.137832	-0.8124	-0.70844	-0.41927	-0.39233	-0.51533	-0.50175	0.023256	0.420073
0.137832	-0.53687	-0.1231	-0.94124	-0.50599	-0.41048	-0.92306	0.034197	0.818134
0.654535								
0.654535	-0.46462	-1.00686	-1.07646	-0.87061	-0.80051	-0.61427	0.15492	0.611841
0.654535	-0.35431	-0.86001	-1.17614	-0.71544	-0.88441	-0.74096	0.018786	0.821825
0.654535	-0.36914	-0.7899	-0.2193	0.18573	-0.16318	-0.03077	0.00104	0.975625
-0.65801	1.016748	1.338823	1.64275	1.198343	1.291635	1.031983	0.00189	0.626002
0.870215								
0.870215	0.355435	0.805712	0.792211	0.442749	0.169349	0.187281	0.063519	0.636364
-0.54647								
-0.54647	0.356838	0.614644	0.732107	0.884027	1.013705	0.8114	0.000113	0.656867
-0.2857								
-0.2857	0.14861	0.53534	0.64299	0.134831	0.129905	-0.38202	0.050285	1.025007
0.622835	1.264173	1.47179	1.40532	0.980722	1.35436	1.447973	0.017293	0.491068
0.56877								
0.622835	-0.8909	-0.73979	-0.63524	-0.85956	-0.55422	-0.52627	0.008327	0.364624
0.622835	-1.23908	-1.61367	-1.81971	-1.14457	-1.54473	-1.12784	0.006686	0.691874
0.622835	-0.93548	-1.35445	-1.81674	-1.07011	-1.18453	-0.98751	0.0497	0.88126
0.622835	-0.54397	-1.08581	-1.20521	-0.74444	-0.94622	-0.66095	0.009534	0.661241
0.56877	1.66453	2.068073	1.726857	1.086178	2.068145	1.855527	0.025012	0.981967
-0.46175	0.775621	1.032013	1.409607	0.731638	0.946144	0.883718	0.001255	0.677969
0.096313	0.325855	0.20701	0.420989	0.569247	0.70176	0.681505	0.015493	0.49475
-0.10672	-0.12493	-0.01684	0.16606	-0.31973	0.16034	0.152778	0.085486	0.485785
-0.10672	0.562624	0.713137	0.856586	0.798316	1.005165	0.913663	0.026666	0.442541
0.096313	0.443276	0.284052	0.103048	0.406874	0.415282	0.519747	0.069916	0.416699
-0.37648	-0.62278	-0.63398	-0.66843	-0.80153	-0.83777	-0.69567	0.070678	0.214985
-0.37648	3.353957	2.842803	-0.90738	-0.0475	1.523725	1.267328	0.041218	4.261336
-0.77471	0.859339	0.434056	0.495533	0.25799	0.364395	0.308734	0.099419	0.601348
-0.37648								
-0.77471	-0.96951	-1.45181	-1.29148	-0.94028	-1.0385	-0.96517	0.01229	0.511538
-0.77471	0.128446	0.414712	1.193917	0.742501	0.387939	0.383238	0.002735	1.06547
0.463913	2.81967	2.773767	1.980363	2.141243	2.93166	2.11618	0.139816	0.951297
-0.17644	1.675863	1.925537	2.226077	1.711283	2.03487	1.74799	0.001579	0.550213
-0.17644	0.461441	0.523892	0.672399	0.654417	0.622417	0.524634	0.048984	0.210958
0.463913	0.528634	0.679194	0.758415	0.614692	1.552045	1.098926	0.004927	1.023411
0.463913	0.309953	0.880281	0.589278	0.642715	1.159944	0.866886	0.055561	0.849991
-0.38429	0.459469	0.577902	0.544177	0.198715	0.703618	0.492973	0.01624	0.504903
-0.38429	0.498993	0.948062	1.341877	0.864553	1.21474	0.901791	0.000353	0.842883
-0.49828	0.885312	1.339574	1.492687	0.983579	1.44865	1.058013	0.02733	0.607375
-0.49828	0.066611	0.145602	-0.22601	-0.39355	0.050725	0.526513	0.006907	0.920064
-0.49828	-0.1712	-0.35505	-0.06752	-0.0687	0.099437	-0.13256	0.08404	0.454487
3.25285								

3.25285	0.13051	-0.17596	-0.20273	0.096889	0.479065	0.187459	0.001729	0.681797
3.25285	0.679099	1.39145	1.069723	1.227998	-0.10744	1.111019	0.089259	1.498887
-0.33446	4.464937	4.085647	3.715643	3.327316	3.40775	2.914117	0.00537	1.55082
-0.33446	-0.42351	-0.30346	-0.0597	0.15881	-0.04777	-0.42141	0.051364	0.582323
-0.33446	-0.82742	-1.40525	-1.38826	-0.76502	-1.06334	-0.91474	0.009583	0.640229
-0.33446	-1.02272	-1.62291	-1.88307	-1.22246	-1.44448	-1.31181	0.000711	0.860354
-0.33446	1.019035	1.330846	1.605483	0.631868	0.861419	0.679884	0.005985	0.973615
-0.70519	1.266976	1.69603	1.904943	1.004885	2.37585	1.716283	0.035143	1.370965
-0.19867	4.377407	4.191862	3.391446	3.385516	3.409829	2.91943	0.008886	1.457977
-0.19867	-0.92031	-1.48605	-1.71666	-1.16355	-1.46185	-1.26859	0.004213	0.796347
-0.19867	0.869329	1.324003	1.32553	0.722592	1.128865	0.990842	0.027196	0.602938
-0.70519	-0.2087	-0.43034	-0.32278	-0.34338	-0.37168	-0.3547	0.005455	0.221635
-0.70519	0.094636	0.06845	0.012082	0.421362	0.023204	0.24041	0.113403	0.409279
0.421583	-0.01485	0.520845	0.378982	-0.13192	0.556492	0.527209	0.062638	0.688416
0.421583	1.105972	0.474868	-1.05193	-0.37315	0.168699	-0.11061	0.015744	2.157906
1.187147	-0.11388	-0.21494	-0.41555	-0.31873	-0.53005	-0.15727	0.159654	0.416169
0.302957	0.414662	0.579321	0.454926	0.370099	0.58745	0.58575	0.012176	0.217351
0.302957	1.625867	2.85819	1.405056	1.89698	0.797167	1.418103	0.038922	2.061023
1.252567								
1.252567	0.09358	-0.37224	-0.70517	-0.30318	-0.1938	-0.01856	0.000727	0.798753
1.252567	1.079419	1.353194	1.318388	0.66992	1.35398	1.221741	0.042562	0.68406
0.144127								
-0.67006								
-0.67006	-0.92641	-1.33623	-1.46567	-1.00192	-1.12986	-0.96116	0.003776	0.539257
-0.67006	-0.41031	-0.4626	-0.70341	-0.6703	-0.93908	-0.74942	0.013528	0.528778
0.051793	-1.01601	-1.11511	-1.15328	-0.97703	-1.1863	-1.10553	0.048997	0.209268
0.144127	-1.08065	-1.662	-1.70428	-1.13121	-1.49756	-1.32867	0.010013	0.623629
0.144127	-1.12984	-1.52331	-1.59023	-1.46925	-1.53557	-1.56644	0.030719	0.460383
0.144127	0.993928	1.528834	1.626763	1.07219	1.5092	1.49766	0.008103	0.632836
-0.31324								
-0.31324	0.149523	-0.67818	-0.77072	-0.04955	0.049811	-0.19814	0.044971	0.920244
-0.31324	-0.59033	-1.15849	-1.24504	-0.79404	-0.97613	-1.02674	0.020468	0.654708
1.290277	1.158591	0.674378	1.167542	0.595873	1.101357	0.744371	0.06151	0.571669
1.290277	0.414139	0.764253	0.714544	0.490442	0.98624	0.692852	0.020471	0.572101
-0.7329	1.06442	1.3477	0.864538	0.609341	1.712125	1.187483	0.069343	1.102784
-0.25475	0.303142	1.01703	1.398983	0.481105	0.430152	0.32706	0.004363	1.095841
-0.25475	-0.53215	-0.60812	-0.64239	-0.44156	-0.15944	-0.37857	0.047181	0.482947
0.349875	0.524087	0.847471	1.055517	0.463358	1.04795	1.00789	0.029785	0.592159
0.349875	0.096682	0.375528	0.206254	0.030741	0.314011	0.081226	0.029557	0.344787
0.349875	-0.74697	-1.14589	-1.40753	-0.9574	-1.17525	-0.87619	0.002538	0.660553
0.349875	-1.34414	-1.67197	-1.42774	-1.39341	-1.88087	-1.53816	0.092472	0.536725
0.349875	-0.50901	-0.56564	-0.47903	-0.51241	-0.51635	-0.56809	0.078475	0.08906
-0.34688	1.510187	1.968383	2.12471	1.616613	2.19345	1.432433	0.104277	0.761017
-0.34688	0.751771	1.200233	0.935383	0.586023	1.54507	1.08018	0.007836	0.959047
-0.34688	-1.27232	-1.69599	-1.81631	-1.22127	-1.43681	-1.26889	0.022489	0.59504
-0.34688	-1.11948	-1.56816	-1.61523	-1.12122	-1.35374	-1.00954	0.04082	0.605695
-0.34688	-0.67646	-0.96987	-1.08594	-0.83022	-0.9255	-0.88791	0.042948	0.409482

-0.34688	-0.50826	-0.50182	0.187524	0.185224	-0.03144	-0.20954	0.000254	0.695783
-0.52677	-0.07339	0.096861	0.037885	-0.08531	0.344322	0.261742	0.005987	0.429633
0.92929								
0.92929	0.489395	0.649423	0.772384	0.909442	0.599766	0.69227	0.004009	0.420047
0.92929	0.641791	0.714103	0.951816	1.045927	1.176945	0.798835	0.015776	0.535154
-0.54046	0.841563	1.4319	1.544133	0.935243	1.32329	1.065955	0.010859	0.70257
-0.25499								
-0.25499	1.169101	1.552747	1.837427	1.136673	1.6997	1.210117	0.011888	0.700753
-0.25499	-0.09478	-0.16763	-0.57859	-0.46942	-0.62459	-0.33785	0.00393	0.529809
-0.25499	0.780221	0.019377	-0.19201	-0.44506	-0.22384	-0.20482	0.095419	1.22528
-0.54046	1.07165	1.326246	1.765763	1.549807	1.245475	1.368291	0.046094	0.694114
-0.45177	-0.32607	-0.20137	0.661579	0.038699	-0.05562	-0.13753	0.030378	0.987646
-0.45177	1.002773	0.923829	1.100757	0.679448	0.920859	0.638658	0.063633	0.462099
3.44577	0.447628	1.153171	1.228382	0.387849	1.1802	0.730552	0.043655	0.840532
3.44577	0.425825	0.756115	0.898804	0.684922	1.08364	0.818133	0.037982	0.657815
-1.14055								
-1.14055	-0.03964	0.310413	0.703284	0.455228	-0.03286	-0.06943	0.012294	0.772713
-1.14055	-0.3918	-0.72116	-0.36219	-0.30773	-0.50143	-0.47583	0.030703	0.413432
0.209525								
0.209525	-0.51338	-0.90303	-1.35073	-1.02059	-1.27709	-0.85575	0.001565	0.837349
0.209525	-0.0882	-0.05088	-0.09037	-0.10713	-0.14614	-0.04957	0.30677	0.096573
-0.16479								
-0.16479	-1.14368	-1.70708	-1.7209	-1.56464	-1.79504	-1.44915	0.019572	0.651352
-0.16479	-0.36548	-0.63481	-1.11708	-0.65085	-0.95749	-0.82311	0.000311	0.7516
-0.16479	-0.11891	-0.65215	-0.90474	-0.63836	-0.83842	-0.6982	0.026794	0.785834
-0.16479	-1.14854	-1.66783	-1.94021	-1.43943	-1.7536	-1.55115	0.034924	0.791664
-0.16479	-1.3257	-1.81592	-1.93283	-1.44558	-1.63196	-1.49767	0.028243	0.60713
-0.16479	-0.5214	-0.96495	-1.37972	-0.6967	-0.84161	-0.83194	0.010106	0.858321
-0.16479	-0.48671	-0.6819	-1.35502	-0.95747	-2.14877	-0.97756	0.015612	1.662055
-0.16479	0.074355	0.490578	0.23972	0.630804	0.326685	0.105798	0.126553	0.556449
-0.29393								
-0.29393	-0.89999	-1.37625	-1.48256	-0.8564	-1.14568	-0.97221	0.079565	0.626156
-0.29393	-1.10643	-1.75694	-1.86853	-1.14451	-1.26238	-1.07762	0.018177	0.790911
-0.29393	-0.7538	-1.241	-1.45915	-0.66363	-1.21477	-0.88769	0.003205	0.795516
-0.29393	-0.05175	-0.46054	-1.15699	-0.57666	-0.6746	-0.15684	0.003837	1.105238
-0.29393	-0.80959	-1.10415	-1.12725	-0.85996	-0.86267	-1.21927	0.134143	0.409681
0.159573								
0.159573								
0.159573	0.885488	1.482283	1.51768	1.01557	1.860635	0.992555	0.017761	0.975147
-0.66457	0.318368	0.637887	0.912879	0.015077	0.995762	0.902874	0.032238	0.980685
-0.66457	1.257177	1.683332	1.059794	1.21042	0.267315	1.071749	0.085305	1.416017
-0.66457	1.261967	-0.37411	-0.9657	-0.46033	-0.5385	-0.58683	0.065748	2.227668
-0.66457	-0.41521	-1.08276	-1.25458	-0.53101	-0.60055	-0.76772	0.108861	0.839372
-0.66457	-0.18928	-0.43178	-0.91866	-0.55577	-0.55661	-0.37329	0.004278	0.72938
-0.66457	-0.07434	0.015073	-0.34916	-0.23416	-0.19361	-0.2253	0.205664	0.36423
-0.66457	-0.6167	-1.10655	-1.20675	-0.83475	-1.08609	-0.99861	0.071326	0.590054
-0.66457	-0.71293	-0.57928	-0.44613	-0.37719	-0.36411	-0.40912	0.00772	0.348822

-1.16829								
-1.16829	-0.45471	-0.7216	-0.74012	-0.32865	-0.58459	-0.58231	0.008659	0.411468
-1.16829	-0.0227	0.019443	0.232075	0.532097	0.063943	-0.07822	0.00227	0.610318
-0.54188	0.862643	0.829412	1.061895	0.525754	0.916321	0.774042	0.047917	0.53614
-0.80343	0.122563	0.161408	0.513572	0.557727	0.395768	0.155366	0.005465	0.435164
-0.80343	0.273788	0.456227	-0.07808	-0.09112	0.241409	0.273875	0.029712	0.547342
-0.80343	-0.65618	-0.78766	-0.8886	-0.88307	-0.79339	-0.60912	0.076129	0.279482
-0.03079	-0.42634	0.546947	1.20815	0.43587	-0.32561	-0.25469	8.57E-05	1.634487
-0.67409	0.904321	1.31668	1.696527	1.256294	1.71154	1.394153	0.093906	0.807219
-0.67409	-0.3598	-0.3146	-0.15087	-0.12268	-0.23921	-0.26862	0.045234	0.237122
-0.67409	-0.81831	-1.14152	-0.9623	-0.74939	-1.0575	-0.92859	0.146414	0.392127
0.428463								
0.24527								
0.24527	-0.1702	-0.39139	-0.71922	-0.42197	-0.50791	-0.52605	0.050548	0.549019
0.24527	-0.35477	-0.44465	-0.37001	-0.37191	-0.31928	-0.3092	0.02228	0.135458
0.180787	0.708884	1.155909	1.2557	0.86482	1.508525	1.102264	0.053184	0.799641
0.091425	1.073975	1.603303	1.384557	0.907348	1.742245	1.347563	0.035728	0.834897
0.877728	0.495588	0.410355	0.359817	0.138205	0.245783	0.295273	0.104197	0.357382
0.189987								
0.189987	0.512912	0.626777	1.186864	0.386762	1.031011	0.399567	0.090089	0.800102
0.877728	0.895379	0.831067	0.984796	1.755297	2.018875	1.298233	0.02069	1.187808
0.877728	0.644077	0.385449	-0.09233	0.778025	0.27334	0.357694	0.005495	0.87035
0.877728	-0.89184	-1.2756	-1.47703	-0.89851	-0.96312	-0.8079	0.042971	0.669133
0.877728	-0.15599	-0.42605	-0.46116	-0.11213	-0.58837	-0.2067	0.137517	0.476237
0.53946	1.074577	1.04086	1.40019	1.002581	0.807118	1.16093	0.055748	0.593073
0.53946	0.255982	0.335021	0.604021	0.880872	1.24125	0.807149	0.007021	0.985268
0.53946	1.721207	1.380433	0.724993	0.960807	0.869492	1.033296	0.005595	0.996214
-0.2041	0.556597	0.598649	0.686825	0.536193	0.430889	0.639895	0.111521	0.255936
-0.2041	0.471919	0.646053	0.564973	0.519353	1.046676	0.785748	0.004228	0.574757
-0.2041	0.525536	0.703562	1.074554	0.897568	1.297855	0.903862	0.006757	0.772319
-0.57875	-0.22361	-0.48799	-0.58451	-0.41819	-0.37643	-0.42283	0.015559	0.360895
-0.57875	0.239133	0.472036	1.003285	0.413089	0.46989	0.425186	0.001034	0.764152
-0.51737								
-0.57875								
-0.51737								
-0.22822	0.25904	0.276351	0.749151	0.304986	0.739148	0.4213	0.067901	0.490111
-0.22822	1.739767	1.783733	1.437913	1.795123	1.42982	1.42409	0.208538	0.371033
-0.55655	0.70546	1.38314	1.434043	0.667161	1.160205	1.024412	0.028998	0.766882
-0.55655	0.666342	1.022093	0.209786	0.438858	-0.22496	0.221457	0.078746	1.247057
-0.55655	0.995946	1.032118	0.524905	0.731196	1.147689	0.632685	0.348716	0.622784
-0.55655	-0.49629	-0.45321	-0.58989	-0.83197	-0.64828	-0.98265	0.226854	0.529443
-0.55655	-1.05158	-1.14348	-1.38138	-0.93927	-0.9899	-0.94363	0.002926	0.442111
-0.55655	0.422495	-0.83382	-1.45272	-1.03611	-0.68134	-0.7781	0.05489	1.875215
0.451805	0.410006	0.653263	0.749496	0.664358	0.912029	0.65565	0.009142	0.502023
0.451805	0.493805	0.479843	0.536754	0.267988	0.340008	0.591072	0.024701	0.323084
1.053967	0.782582	0.77378	1.130689	0.594093	1.096373	1.218327	0.002507	0.624233
1.796647	0.683427	0.898498	1.190302	0.457323	0.806077	1.017593	0.009403	0.732979

1.796647	0.56804	1.034142	1.315506	0.77447	1.025317	0.989127	0.045103	0.747466	
0.84687									
0.84687	2.616757	2.302937	2.255617	2.754243	1.77313	1.880383	0.140664	0.981113	
0.84687	-0.84266	-1.21915	-1.54686	-0.99199	-1.18977	-1.03534	0.020972	0.704202	
0.84687	-0.76544	-1.35606	-1.59575	-0.89243	-1.11774	-0.96484	0.024414	0.830313	
0.84687	0.248432	0.608919	-0.40461	-0.24837	-0.7505	-0.08485	0.001204	1.359418	
0.84687	3.022997	1.59143	0.82215	1.171125	1.137191	0.897228	0.05653	2.200847	
0.84687	-0.84642	-1.24156	-1.78605	-1.07933	-1.05317	-1.17293	0.006241	0.93963	
0.84687	-1.29133	-1.86538	-1.86771	-1.20099	-1.60532	-1.27401	0.008324	0.666717	
0.84687	0.949734	1.500181	-0.01555	0.385676	0.399568	1.112978	0.170793	1.515734	
-0.60946	0.727759	1.027611	1.142613	0.248667	0.477107	0.483172	0.002707	0.893946	
-0.60946	1.369757	1.73895	1.749587	1.223339	1.70684	1.328806	0.033629	0.526248	
-0.29053	0.883608	0.798636	1.22343	0.920814	0.981931	0.722159	0.064504	0.501271	
-0.29053	-0.52672	-0.57572	-0.63928	-0.68547	-0.48348	-0.43946	0.098571	0.246007	
-0.29053	-1.23086	-1.5626	-1.34314	-1.03051	-1.33474	-1.32647	0.013852	0.532088	
1.516285	1.228567	1.654527	1.69192	1.173791	1.81341	1.591677	0.073093	0.639619	
1.516285	-1.23975	-1.74105	-1.9252	-1.18763	-1.52916	-1.07604	0.003464	0.84916	
1.516285	-0.87368	-1.69955	-1.62598	-0.89734	-1.0825	-0.9687	0.000409	0.82587	
1.516285	-1.05815	-1.35545	-1.58327	-1.11978	-1.36769	-1.20565	0.01434	0.525119	
1.516285	-0.89541	-1.32772	-1.48508	-0.90227	-1.0965	-0.84003	0.059291	0.645053	
1.516285	-0.48408	-0.46959	-0.25891	-0.34809	-0.58971	-0.27367	0.077172	0.3308	
1.948493									
1.948493	-0.71887	-1.33923	-1.45668	-0.81583	-0.80965	-0.68441	0.0121	0.772266	
1.948493	-1.23856	-1.53623	-1.77446	-1.14269	-1.42475	-1.0861	0.0233	0.688356	
1.948493	-0.29132	-0.89536	-1.19639	-0.25514	-0.64509	-0.3314	0.041935	0.941251	
1.948493	-0.60636	-0.73028	-1.24898	-0.80211	-0.94416	-0.83555	0.00365	0.642616	
1.948493	-0.81043	-1.28212	-1.73643	-1.03808	-1.413	-1.14157	0.000333	0.925999	
1.948493	-0.56459	-0.98935	-1.26935	-0.8295	-1.21191	-0.82952	0.071513	0.704756	
0.911003									
0.911003	1.32318	1.521737	1.42923	0.694897	0.339351	0.360485	0.001324	1.182386	
-0.20216									
-0.20216	-0.55059	-0.95304	-0.32961	0.220233	-0.35565	-0.28933	0.000957	1.173275	
-0.20216	-0.83457	-1.46117	-1.4606	-0.5617	-1.23942	-0.91576	0.00102	0.899475	
-0.20216	-0.827	-1.54919	-1.69955	-0.91373	-1.29945	-0.98923	0.002535	0.872548	
-0.20216	0.258898	0.640297	1.119123	0.417328	-0.20825	-0.15716	0.00026	1.327369	
0.42245	-0.41946	-0.39158	0.372865	-0.09221	-0.2782	-0.16683	0.026199	0.792323	
0.42245	-0.96038	-1.45767	-1.55	-0.76861	-1.32795	-0.98344	0.003232	0.781395	
0.42245	-0.61322	-1.11814	-1.17616	-0.86542	-1.29004	-1.12968	0.010525	0.67681	
1.109687									
1.109687	-0.04171	0.647057	0.668137	0.153883	0.031405	-0.11003	5.73E-05	0.778166	
-1.18399	0.188713	0.376744	1.072429	0.681555	0.86601	0.569343	0.082048	0.883716	
-1.18399	-0.31885	-0.47646	0.924174	0.50751	-0.06664	-0.27369	0.000379	1.400637	
3.330797	0.308997	1.072837	1.425753	0.508112	0.387904	0.130089	0.00063	1.295665	
3.330797	-0.42761	-1.00797	-1.06971	-0.54977	-0.57707	-0.63935	0.000237	0.642092	
3.330797	-0.27064	-0.4883	-0.91062	-0.43114	-0.66868	-0.58242	0.012301	0.639982	
-0.40138	0.469218	0.862273	1.120337	0.342063	0.99522	0.856877	0.004948	0.778273	
-0.40138	0.342507	0.471701	0.57606	0.627977	0.62708	0.506737	0.018203	0.28547	

0.84529								
0.84529	-0.87031	-1.2393	-1.47191	-0.95112	-1.27656	-1.1591	0.036884	0.601603
0.84529	0.164533	0.388159	0.314405	0.239684	0.52517	0.342952	0.006793	0.360636
1.810037								
1.810037	-0.92133	-1.31397	-1.50674	-0.98351	-1.23301	-1.0089	0.015513	0.58541
1.810037	-0.46456	-1.02384	-0.90701	-0.79214	-0.64672	-0.66649	0.002785	0.559281
1.810037	-0.38989	-0.62248	-0.46841	-0.31093	0.150901	0.005181	0.00716	0.773385
0.117145	0.329018	0.28942	0.145833	0.039956	0.645613	0.673156	0.001643	0.6332
0.117145	0.765488	0.465399	0.86889	1.675325	0.229282	1.467671	0.150239	1.446043
0.117145	-0.45139	-0.7565	-1.11049	-0.54812	-0.67632	-0.68245	0.005216	0.6591
0.117145	-1.05623	-1.69901	-1.9238	-1.23437	-1.66505	-1.34647	0.006733	0.867575
0.117145	-0.46922	-1.12877	-1.29228	-0.62281	-0.87039	-0.74936	0.018971	0.823065
0.117145	0.030453	-0.53465	-0.78389	0.124587	-0.53114	-0.76752	0.200193	0.908478
0.117145	-0.98143	-1.31237	-1.61484	-0.84596	-1.38608	-0.992	0.013277	0.768878
-0.24111	0.703975	0.663276	1.166737	0.821718	1.17169	0.959046	0.004678	0.508414
-0.24111	0.62556	1.055618	0.945415	1.263915	1.421871	1.147519	0.18922	0.796311
-0.36311	0.431352	0.70846	1.02567	0.808715	0.495153	0.347657	0.005252	0.678014
-0.36311	0.697145	0.162304	0.221475	0.652114	-0.65078	0.368862	0.095379	1.34793
-0.36311	-0.5281	-1.04453	-1.26675	-0.66121	-0.53771	-0.68602	0.030646	0.738653
-0.11352								
-0.11352	-0.76354	-1.5756	-1.76153	-1.06463	-1.34853	-1.07271	0.024107	0.997998
-0.11352								
0.71323	0.716291	0.718459	0.46952	0.196695	0.769417	0.736541	0.010918	0.572722
0.71323	-0.8692	-1.23933	-1.05435	-0.93094	-1.38642	-1.18399	0.166638	0.517217
0.71323	-0.79199	-1.10493	-1.29799	-0.88164	-1.17887	-0.90239	0.008987	0.506001
-0.19623	0.56868	1.047889	1.23243	0.644397	0.731744	0.656406	0.033549	0.66375
-0.19623	-0.10476	0.181159	0.844823	0.287501	-0.0995	-0.10796	0.004463	0.952779
-0.19623	-0.32255	-0.75	-1.16343	-0.62144	-0.75952	-0.7975	0.029228	0.840884
-0.19623	0.197804	0.380873	0.629666	0.42341	0.032971	0.120023	0.024599	0.596695
-0.64473								
-0.503	0.097971	0.368216	0.507469	0.319166	0.283403	0.144058	0.213799	0.409498
-0.90514	-0.67449	-0.33216	-1.30396	-0.51856	-1.54553	-0.95627	0.08575	1.213369
-0.90514	-0.50117	-0.29105	0.302558	0.474564	0.355887	-0.39471	0.073262	0.97573
-0.503	-0.92037	-0.75304	-1.55494	-0.21428	-1.31282	-0.85451	0.11671	1.340659
-0.503	-0.47119	-0.65089	0.302935	0.223355	0.33556	-0.52011	0.035038	0.986447
-0.06003	0.627414	0.355616	-1.29135	-0.6673	-0.75412	-0.83736	0.050774	1.91876
-0.58378	-1.0863	-1.52858	-1.68697	-1.10636	-1.42426	-1.09572	0.000444	0.600666
-0.64473	-1.05925	-1.38613	-1.6686	-1.02796	-1.30998	-1.073	0.000799	0.640646
1.352723	0.175964	0.257533	0.366714	0.383377	0.298311	0.264969	0.141921	0.207413
1.128555	-1.25494	-1.36073	-1.44741	-1.41579	-1.33021	-1.23169	0.075977	0.215723
1.128555	-0.31515	-0.33955	-0.51084	-0.43828	-0.92242	-0.59692	0.018429	0.607263
1.128555	0.441593	0.68652	1.11019	0.922034	1.165248	0.984608	0.084062	0.723655
1.352723	-1.03492	-1.1322	-0.59426	-0.79851	-0.73852	-0.66654	0.03212	0.537939
1.352723	-0.2023	-0.56805	-0.86124	-0.37618	-0.83111	-0.50212	0.06921	0.658936
1.352723	1.162059	1.267573	1.531787	1.419137	1.78785	1.360983	0.019167	0.625791
1.37557	0.512968	0.729803	1.165891	0.10982	0.357287	0.556162	0.056157	1.056071

1.37557	-0.58872	-1.07174	-1.16332	-0.70211	-0.67414	-0.56113	0.022211	0.602185
1.37557	-1.56812	-1.64444	-1.98115	-1.82093	-2.03982	-1.59837	0.052704	0.471697
-0.68361	0.633869	0.990778	0.823435	0.524626	1.115185	1.006481	0.010613	0.590559
-0.68361	0.246034	0.423375	0.498029	0.188439	0.272112	0.116921	0.140786	0.381108
-0.68361	0.354538	0.722059	1.1579	0.98802	0.639266	0.460429	0.015561	0.803361
1.073323								
1.073323	-1.02119	-1.39561	-1.62239	-1.12496	-1.46449	-0.63108	0.000879	0.991312
1.073323	-0.40776	-0.76873	-0.78304	-0.2379	-0.20155	-0.20729	0.016478	0.581494
1.350173	0.052771	-0.23216	-0.00802	-0.57786	-0.72628	-0.46553	0.020688	0.779055
1.350173	-0.38675	-0.55477	-0.44119	-0.71184	-0.2453	-0.28112	0.003566	0.46654
1.350173	0.179566	-0.04061	-0.2344	-0.28007	0.517449	-0.30931	0.226222	0.826755
1.350173	-0.90685	-1.37326	-1.63415	-1.2752	-1.4413	-1.09454	0.016329	0.727298
0.22651	-0.772	-1.37431	-1.44383	-0.70001	-1.62415	-1.06383	0.017182	0.924137
	0.733903	0.451822	-0.03173	0.606711	0.150683	0.35918	0.03675	0.765629
	3.6616	2.907963	2.193623	2.399477	2.64685	2.080176	0.068229	1.581424
-0.88842								
-0.88842	0.366046	0.437554	1.008791	0.952936	0.415249	0.62437	0.002675	0.642745
-1.45373								
2.310652								
2.310652	0.286625	0.350542	0.548341	0.64082	0.682568	0.566693	0.026237	0.395943
2.310652	1.818363	1.710453	0.778732	2.170053	0.660012	0.541506	0.048238	1.628547
2.310652	-1.05248	-1.08322	-1.27758	-1.10212	-1.12352	-1.22178	0.141093	0.225096
0.251537	1.186737	1.330533	1.744387	1.270778	1.76776	1.405813	0.011331	0.581023
0.251537	-0.86978	-1.22143	-1.34069	-0.97741	-1.23186	-1.11453	0.019439	0.470918
0.251537	-0.94839	-1.11536	-1.22756	-0.87542	-1.24722	-1.04351	0.02597	0.371791
-0.62523								
-0.62523	1.396107	1.710373	2.154427	1.669607	1.611205	1.346453	0.000128	0.807973
-0.55911	1.482367	1.851929	1.675267	1.169646	1.730245	1.316188	0.082278	0.682283
-0.55911	-0.16515	0.245437	0.195742	-0.191	0.248205	0.250967	0.019558	0.441971
-0.55911	-0.48744	-0.52508	-0.27856	-0.40989	-0.46648	-0.59555	0.091097	0.316985
-0.31949	-0.14394	0.191949	0.375405	-0.03597	-0.64821	-0.43078	0.010898	1.02361
	0.571223	1.166877	1.454637	0.898107	0.614385	0.663305	3.82E-07	0.883414
-0.48892								
-0.48892	-0.75163	-0.42176	-0.00855	0.024934	-0.51969	-0.63237	0.014823	0.776565
-0.48892	1.179423	1.026971	1.277515	0.878035	-0.01809	0.744168	0.013246	1.295608
-0.82128	0.178613	0.53075	0.917675	0.382256	0.224882	0.043956	0.003702	0.87372
-0.82128	-1.29413	-1.8001	-1.94771	-1.38132	-1.673	-1.43069	0.01681	0.65358
-0.82128	-1.09717	-1.44917	-1.66023	-1.24336	-1.65247	-1.40028	0.002699	0.563057
0.688228								
0.688228	-0.68955	-0.90836	-1.13609	-0.79955	-0.85945	-0.75782	0.034554	0.446541
0.688228	-0.91364	-1.57547	-1.86074	-1.0565	-1.08363	-0.98303	0.023309	0.947101
0.688228	-0.03035	-1.2503	-1.78446	-1.07125	-1.46606	-1.21328	0.037906	1.75411
0.688228	-0.61729	-0.70615	-0.53175	-0.41091	-0.77648	-0.58229	0.126776	0.365572
0.160923	3.026203	3.034217	2.49706	2.204283	2.530175	2.125253	0.003859	0.908963
-0.91855								

-0.07272	-0.32089	-0.64356	-0.77504	-0.41542	-0.6904	-0.36414	0.010375	0.454147
-0.07272								
-0.07272								
-0.07272	-1.15013	-1.69442	-1.80381	-1.2403	-1.48626	-1.09253	0.001203	0.71128
-0.07272								
-1.0924	0.60854	0.573407	0.667856	0.674944	1.16804	1.04451	0.027979	0.594633
-1.0924	1.47287	1.760787	1.854367	1.137076	1.547455	1.322587	0.023119	0.717291
-0.90388	-0.08369	-0.01314	0.231553	0.393868	0.422421	0.068002	0.036191	0.506107
-1.0552	1.996477	2.73799	2.323387	2.027972	2.72049	2.23256	0.097757	0.741513
-1.0552	0.339805	0.579057	0.281204	0.357129	0.318741	0.240593	0.060802	0.338464
-0.90388	-0.39048	-0.06192	0.402847	-0.33711	0.194562	-0.06066	0.005576	0.793324
-0.90388	0.872887	1.009695	1.09577	0.600264	1.202145	1.053088	0.010623	0.601881
-0.98351	2.778633	2.886657	1.94049	2.159427	2.37287	1.945127	0.061909	0.946167
-0.98351	2.2531	2.40312	2.550953	1.836953	2.327655	1.979063	0.022499	0.714
-0.48379	0.310557	0.339083	0.188278	0.5087	0.900927	0.278806	0.048753	0.712649
0.095913	0.377838	0.556488	0.989757	0.319641	0.348932	0.546434	0.001091	0.670116
0.095913	-0.30027	-0.1347	0.112812	0.202793	-0.27628	-0.1909	0.034876	0.503062
	-0.34387	-0.41416	-0.48259	0.023865	-0.24105	-0.33581	0.02141	0.506452
-0.08852	0.139074	0.345296	0.635527	0.06438	0.671082	0.634188	0.135127	0.606701
-0.08852	-0.69591	-0.36092	-1.02186	-0.75195	-0.85853	-0.44663	0.131204	0.660946
-0.08852	0.556062	0.419049	1.487079	0.186743	0.468495	0.386556	0.038072	1.300335
-0.71196	-1.19878	-1.86501	-1.87172	-1.07354	-2.31509	-1.40952	0.020774	1.241544
-0.54862								
-0.54862	1.76467	1.756573	1.797283	1.44075	2.036045	1.962457	0.047713	0.595295
-0.14971								
-0.14971	-0.80693	-0.94151	-0.73445	-0.59208	-0.54145	-0.68996	0.012559	0.400057
-0.14971	1.029916	1.33852	2.032363	1.740353	2.05883	1.505967	0.001344	1.028914
-0.65753								
-0.65753	-0.63078	-1.10563	-1.13711	-0.55699	-0.2953	-0.56858	0.067679	0.841807
-0.65753	0.661856	0.317382	0.81431	0.664095	0.815891	0.43401	0.145938	0.498508
-0.85934								
0.006003								
0.006003								
-0.96025	-0.78037	-1.09276	-1.29793	-1.14272	-1.11148	-0.7857	0.037426	0.517559
-0.96025	-0.74097	-0.92085	-0.74833	-0.42343	-0.54732	-0.55416	0.0016	0.497413
-0.76873								
-0.76873	-1.4575	-1.88951	-2.39215	-2.26116	-2.47856	-2.68867	0.00773	1.23117
-0.92267								
-0.85684	0.476956	0.504088	0.548724	0.669569	0.833148	0.637319	0.003927	0.356192
0.563537	-0.08759	0.242163	0.545491	0.273804	0.193421	0.285758	0.064584	0.633082
-0.23602								
-0.23602	1.638777	1.58787	1.378797	1.135947	1.369145	1.237477	0.05616	0.50283
-0.24637	0.323262	0.614737	0.847891	1.030298	1.2459	0.948971	0.003369	0.922638
0.563537	0.051099	0.072853	0.181373	0.681596	0.450907	0.283283	0.002468	0.630497
0.563537	-0.38067	-0.11928	-0.03402	-0.29915	-0.16364	-0.19007	0.000337	0.346653
-0.24637	-1.2479	-1.00526	-1.79363	-1.37923	-2.02222	-1.552	0.120846	1.016956

-0.24637	-0.48086	-0.52978	-0.37867	-0.53718	-0.04622	-0.11318	0.02863	0.490962
-0.25893	1.370337	1.552873	1.984107	1.317639	2.148975	1.867657	0.054546	0.831336
-0.25893	0.311554	0.371149	0.763142	0.411596	0.771775	0.483838	0.012641	0.460221
-0.24295	0.060027	0.289727	0.579042	0.311871	0.417109	0.251669	0.040734	0.519015
-0.24295	0.936893	1.207741	1.454817	0.720324	1.022136	0.915202	0.001384	0.734493
-0.24295	-0.70237	-0.99626	-1.05359	-0.60933	-0.97042	-0.80338	0.023139	0.444269
-0.40307								
	0.529245	0.572971	0.760082	0.720234	0.992808	0.695449	0.007319	0.463563
	-0.77023	-0.91726	-1.02193	-0.75189	-0.83105	-0.71631	0.073412	0.305626
-0.36106								
-0.36106	-2.18444	-2.41038	-3.05907	-3.36397	-3.38054	-3.2899	0.046728	1.196097
-0.36106	-1.26181	-1.58951	-1.73632	-1.8138	-2.0137	-2.23179	0.001512	0.96998
0.655277	1.077305	1.139271	1.149977	0.865917	1.558907	1.12036	0.034652	0.69299
0.655277	-0.72369	-1.06426	-0.82794	-0.53597	-0.72492	-0.8988	0.072789	0.528294
0.655277	-0.22377	-0.29542	-0.2717	0.068077	0.303871	0.558132	0.052966	0.853551
0.093917	0.380069	0.581704	0.483767	0.143789	0.754314	0.518098	0.002424	0.610525
0.1545	0.368402	0.773469	0.701509	0.784861	1.130335	0.734551	0.008981	0.761933
0.093917	0.10699	0.338244	0.173795	0.436241	0.806897	0.380012	0.009664	0.699906
0.004793	0.30427	0.554706	0.576146	0.617457	1.046761	0.601778	0.011042	0.742491
-0.6022	-0.15881	-1.06151	-1.12619	-0.77676	-0.82934	-0.83325	0.08421	0.967381
-0.6022	0.481869	1.120141	1.122348	0.455812	0.222942	-0.01835	0.016257	1.140696
0.387957	0.38758	0.548612	0.707849	0.543356	0.476349	0.466363	0.006773	0.320269
0.387957	-0.12835	-0.32191	0.158133	0.241653	0.065441	0.367495	0.053082	0.689401
0.387957	-0.18346	0.155835	0.674627	0.58786	0.53163	0.103516	0.00431	0.85809
0.012617	0.802353	1.11573	1.302263	1.021828	1.195895	0.90432	0.00804	0.49991
-0.10269								
-0.10269	0.731587	0.898571	0.97721	1.057978	1.036485	0.821	0.051609	0.326391
-0.20502	0.345884	0.739755	0.992336	0.493725	0.969936	0.856177	0.006517	0.646452
0.012617	1.716573	1.947976	1.338161	1.204298	1.28246	1.810036	0.212289	0.743678
0.012617	0.262248	0.443532	0.26111	0.454693	0.841267	0.548984	0.027461	0.580156
-0.47094								
-0.47094	1.74258	1.77997	1.171627	1.121073	1.942365	1.654843	0.016998	0.821292
0.716597	0.161007	0.584375	0.561845	0.145135	0.449398	0.478743	0.115466	0.439239
0.716597	0.65453	1.028356	0.993522	0.557525	0.895834	0.756001	0.038488	0.470831
0.716597	2.015763	2.0045	1.419283	1.61898	2.021605	1.63586	0.033463	0.602322
-0.30268								
-0.30268	-0.92624	-1.64173	-1.52588	-1.19812	-1.24031	-1.07645	0.002491	0.715484
-0.30268	0.002787	-0.41738	-0.51764	-0.40598	-0.43032	0.262049	0.08882	0.77969
0.56972								
0.992147								
0.992147	-0.43425	-0.62299	-0.64257	-0.34608	-0.22516	-0.46038	0.083603	0.417418
0.992147	-0.18039	-0.06775	0.266816	-0.01913	-0.04413	-0.32357	0.057195	0.590382
0.56972	0.1788	0.455172	0.699541	0.290035	0.346897	0.227167	0.014488	0.520741
0.56972	0.539615	0.536966	0.822025	0.421051	0.65674	0.505257	0.077376	0.400974
1.274637	0.888279	0.475569	0.926336	0.974735	0.97409	0.844741	0.075605	0.499166
1.274637	-0.58784	-1.28926	-1.59526	-0.99984	-1.21757	-0.96374	0.024235	1.007412
1.274637	1.25863	1.060669	1.01352	2.443253	1.846165	1.660667	0.042403	1.429733

-0.8643	0.191747	0.03226	-0.49991	-0.27009	-0.37596	-0.10865	0.010569	0.691659
-0.8643	1.186161	1.35412	1.667847	1.117107	1.450755	0.89341	0.022371	0.774436
-0.8643	1.38625	1.64066	1.946797	1.349333	1.71557	1.67165	0.032371	0.597463
0.383615	-0.65991	-1.14083	-1.08054	-0.48077	-0.75673	-0.64791	0.001195	0.660061
0.383615	-0.8655	-1.21788	-1.30412	-0.73805	-0.94525	-0.88044	0.033277	0.566076
0.383615	-0.82689	-0.88388	-1.20723	-0.6553	-1.17419	-0.65325	0.167366	0.553983
0.383615								
0.383615	-0.18538	-0.52027	-1.00018	-0.62084	-0.84918	-0.79285	0.001323	0.814803
0.383615	-0.29835	-0.36373	-0.72573	-0.44472	-0.34362	-0.47957	0.08429	0.427384
0.383615	1.345664	0.876536	2.316379	1.153521	0.252681	1.061141	0.068505	2.063698
0.383615	-0.4341	-0.69116	-1.23621	-0.71906	-0.697	-0.77456	0.009122	0.80211
0.383615	0.424843	0.929774	0.783486	0.191871	1.1019	0.823584	0.014498	0.910029
0.321238	0.978369	0.840407	1.165477	1.110568	1.17578	0.612557	0.036377	0.563223
0.383615	-0.78527	-0.8869	-1.08227	-1.05699	-1.43459	-0.84422	0.081093	0.649317
0.321238	-1.0655	-1.25359	-1.40861	-1.04699	-1.15802	-1.21045	0.024328	0.36162
0.321238	-1.33837	-1.76988	-1.90242	-1.26207	-1.73796	-1.40186	0.005228	0.640357
0.321238	-1.09865	-1.53298	-1.89695	-1.1766	-1.47864	-1.18513	0.002721	0.798303
0.321238	-0.83559	-1.30028	-1.47275	-0.87983	-1.31984	-1.06225	0.003853	0.63716
0.321238	-0.87448	-1.00882	-1.24069	-1.00035	-1.17432	-1.15162	0.075204	0.366215
0.9977	0.650184	1.41118	1.41954	0.310737	0.616753	0.964088	0.004274	1.108803
0.9977	-0.83764	-1.0985	-1.32865	-0.8517	-1.39224	-1.04428	0.131559	0.554596
0.9977	-0.43727	-0.55641	-1.27014	-0.80495	-1.26469	-0.88838	0.001151	0.832872
0.9977	-1.0016	-1.57638	-1.8739	-1.15246	-1.81486	-1.46727	0.011168	0.872301
0.9977	-0.24093	-0.30682	-0.31759	-0.08837	0.06367	-0.20847	0.38343	0.381259
0.026002	0.407537	0.631347	0.90102	0.543489	0.880614	0.814234	0.126985	0.493482
0.026002	1.237879	1.4943	1.707523	1.183003	1.69001	1.216432	0.123319	0.52452
-0.3266	0.626199	0.773766	1.126572	0.680649	0.989331	0.998715	0.010564	0.500374
-0.3266	1.094164	1.382009	0.991292	0.61673	0.817765	0.89835	0.056782	0.765279
0.90368	0.118478	0.2798	0.508524	-0.09258	-0.01465	-0.01215	0.131321	0.601108
0.90368	-0.56183	-0.95989	-1.29829	-0.48476	-0.95269	-0.80971	0.07173	0.813529
0.90368	0.060334	-0.68952	-1.20239	-0.68203	-0.75914	-0.49007	0.02221	1.26272
	-0.24005	0.180069	0.404499	-0.00412	-0.27533	-0.27976	0.029357	0.68426
-1.34701	0.280779	0.575531	1.0588	0.594563	0.604007	0.492772	0.009652	0.778021
-1.34701	0.801348	1.361213	1.6798	1.450893	1.194635	0.886021	0.00097	0.878452
0.137832	0.213487	0.396546	0.239151	0.617445	1.050469	0.669969	0.039767	0.836981
0.043007	0.566619	1.156571	0.410448	0.862115	0.939031	0.80196	0.123492	0.746123
0.53534								
0.53534	1.28806	1.29019	1.65057	1.335523	1.735575	1.601293	0.007757	0.447515
0.53534	-0.22923	-0.44866	-0.69453	-0.40869	-0.64665	-0.61509	0.00363	0.465297
-0.93282	1.994293	1.94333	1.895542	1.64593	1.71991	1.17058	0.013912	0.823714
-0.78736								
-0.78736	0.496539	1.133018	1.585963	1.036612	1.05843	0.719339	0.00703	1.089424
-0.75881	0.606499	0.816716	1.006505	0.804114	0.732493	0.638314	0.002368	0.400006
-0.75881								
-0.75881	0.641909	1.040792	1.29061	1.22569	1.43438	0.93385	0.005755	0.792471
-0.54115	0.849482	1.159294	1.211781	1.036695	1.87546	1.214807	0.020811	1.025978
0.767737	-0.979	-1.29614	-1.51753	-0.86111	-1.03527	-0.93173	0.002306	0.656423

0.767737	-0.09228	-0.16761	0.005621	-0.07054	0.390316	0.108403	0.011304	0.55793
0.767737	0.840483	1.52525	1.732523	1.257498	1.383764	1.401417	0.026855	0.89204
-0.54115	0.831931	1.499697	1.728577	0.937763	1.780275	1.323453	0.022528	0.948344
-0.54115	1.003453	1.376187	1.990593	1.171127	1.49125	1.267277	0.001023	0.98714
-0.73581								
-0.73581	-0.59063	-1.1953	-1.33752	-0.86154	-1.2177	-0.93029	0.017158	0.74689
-0.73581	0.217933	-0.26634	-0.22462	0.068969	-0.23355	-0.19158	0.158051	0.484271
0.458245	0.571442	0.360721	0.781725	0.357668	0.507618	0.565062	0.02695	0.424058
0.458245	-0.97149	-0.65615	0.081144	-0.15946	-0.82285	-0.73505	0.001341	1.052631
0.458245	-0.43841	-1.13542	-1.68473	-0.77072	-1.06855	-0.9159	0.00125	1.246314
0.458245	0.517181	0.653652	0.983153	0.560268	0.576073	0.541948	0.031391	0.465972
0.026687								
-0.58595	0.577666	0.874578	0.741114	0.566987	0.610381	0.522614	0.111819	0.351964
-0.58595	0.087556	0.633168	1.280093	0.759691	-0.0146	-0.06798	0.00106	1.348069
0.963902	1.057122	0.56193	0.710244	0.515266	0.797724	0.813938	0.057978	0.541857
0.963902	0.432325	0.692445	0.621373	0.406634	1.03125	0.834267	0.034311	0.624616
-0.92452	-0.27192	-0.1484	0.00273	-0.18204	-0.04854	-0.17782	0.007427	0.274654
-1.01235	0.400675	0.79893	0.88238	0.50423	0.82263	0.62762	0.022733	0.481705
-0.49726								
-0.49726	0.508025	0.457462	0.287078	-0.25978	-0.04775	0.236245	0.005309	0.767801
2.393497	1.91193	1.708549	1.529405	1.190344	1.873726	1.705997	0.167804	0.721586
2.393497	-0.33898	-0.68457	-0.85639	-0.47288	-0.46013	-0.35082	0.005431	0.517409
2.393497	0.787889	0.823696	0.6823	0.381014	1.215725	1.190583	0.004223	0.834711
0.013958								
0.013958	-0.26931	-0.51882	-0.99372	-0.5991	-0.92286	-0.86617	0.077065	0.724404
0.013958	-0.43655	-1.30177	-1.65029	-0.98612	-1.48836	-1.17503	0.015088	1.213735
0.013958	-0.01669	-0.0586	0.073574	-0.24773	0.216127	0.174372	0.028515	0.463853
0.01339	1.216751	1.612367	1.760083	1.185304	1.604715	1.26	0.030963	0.57478
-0.05943	1.034466	1.655617	1.800697	1.190584	1.679545	1.164783	0.086888	0.766231
-0.05943	0.421742	0.641214	0.864323	0.685685	0.825279	0.61717	0.05719	0.442581
0.21698								
0.21698	0.868616	1.346453	1.111593	1.072904	1.2857	1.136053	0.113433	0.477837
0.21698	-0.88272	-1.61419	-1.41938	-0.9375	-0.91678	-0.92718	0.007494	0.731469
1.275097	0.904013	0.878724	1.19336	0.504317	1.526935	1.26016	0.012126	1.022618
1.275097	1.324673	1.50755	1.266953	0.16993	1.86583	1.753383	0.007339	1.6959
0.083047	0.007915	0.252213	0.270298	0.200746	0.771071	0.577502	0.013855	0.763155
0.083047	-0.57762	-0.46526	-0.9325	-0.54219	-0.3566	-0.70568	0.246093	0.575902
0.083047	1.364335	-0.03987	-0.39088	0.790852	-0.20203	-0.33849	0.103683	1.755216
0.083047	-0.86663	-1.39454	-1.57495	-1.0898	-1.27494	-1.02607	0.002112	0.708319
0.083047	-0.68106	-0.49043	-0.71339	-0.29047	-0.56587	-0.30754	0.319122	0.422925
0.083047	-1.05332	-1.34223	-1.67834	-1.33926	-1.18458	-1.17229	0.000457	0.625024
0.083047	-0.78458	-1.49941	-1.9125	-1.35019	-1.49831	-1.1649	0.076848	1.127921
0.083047	1.915917	1.920347	0.977557	1.186525	0.988969	1.159778	0.195155	0.942789
0.083047	2.584637	2.03294	1.092822	1.414207	2.71618	1.17505	0.013271	1.623358
0.083047	-0.60061	-1.65331	-1.69368	-1.31866	-1.66131	-1.36192	0.127805	1.093069
-0.86121	0.702001	0.889654	1.145446	0.756549	0.655523	0.768037	0.032562	0.489923
-0.86121	1.220757	1.726167	1.897463	1.296697	1.367215	1.20132	0.007864	0.696143

1.765183	-0.12273	0.153226	0.262573	-0.06496	0.539805	0.358548	0.034319	0.662533
0.801747	-0.9167	-1.3793	-1.6196	-1.16987	-1.12896	-0.97791	0.011681	0.7029
0.801747	0.217971	1.297619	0.444971	0.828633	0.414273	0.70516	0.098489	1.079647
0.036523	1.121607	1.724897	1.719403	1.18046	1.639875	1.396607	0.009584	0.60329
1.00803								
0.036523	1.21505	1.72159	1.647667	0.98817	1.563925	1.27921	0.011441	0.73342
1.00803	1.498207	1.955523	1.897867	1.345333	1.54555	1.637463	0.066592	0.61019
-0.23137	1.11444	1.492427	1.634806	0.92108	2.25112	1.612187	0.025164	1.33004
-0.23137	-0.40281	-0.59819	-0.41728	-0.00924	-0.75528	-0.30223	0.060219	0.746034
-0.23137	-1.53083	-1.03428	-1.90998	-1.24785	-1.77795	-1.45698	0.145409	0.875709
-0.23137	-0.17999	-0.61741	-1.06297	-0.68743	-0.7227	-0.66942	0.029466	0.882988
-0.45422	3.130517	2.680253	2.324893	2.05378	2.26417	2.083477	0.066182	1.076737
-0.45422	0.047987	0.202755	0.848825	0.9378	0.881057	0.477345	0.00223	0.889813
-0.53176	1.204317	1.626257	1.858267	1.024475	1.96564	1.526233	0.019685	0.941165
-0.45422	-0.25736	-0.25876	-0.23663	-0.2537	-0.0267	0.002714	0.140424	0.261469
-0.53176	1.45183	2.262239	1.037693	0.183835	0.23261	1.010108	0.090073	2.078404
-0.65497	-0.05682	0.232615	0.766783	0.288011	0.252231	0.052757	0.013996	0.823605
0.785607	-0.25566	-0.52818	-0.46152	-0.13145	-0.38669	-0.31805	0.024412	0.396723
0.785607	-0.28528	-0.72554	-0.93183	-1.05747	-1.33549	-0.66025	0.068964	1.05021
-0.65497	-0.43335	-0.43857	-0.25021	-0.06503	-0.08265	-0.08693	0.015109	0.373538
-0.65497	0.827771	1.359913	1.568997	1.08569	1.17786	0.936033	0.002798	0.741226
-0.36892								
-0.36892	0.84956	1.415407	1.85035	1.079993	1.057095	0.948878	0.009243	1.00079
0.801747								
1.765183	-0.29315	-0.39798	-0.28683	0.031916	0.105054	-0.03299	0.001516	0.503037
1.765183	1.31774	1.20541	0.245709	0.180088	0.714688	0.419806	0.075602	1.137651
-0.28133	0.24321	0.403937	0.57789	0.271663	0.158335	0.35467	0.001704	0.419555
-0.92558	0.130117	0.568683	0.906547	0.538907	0.206637	0.219408	0.001371	0.77643
-0.92558	-0.1922	0.397255	1.05807	0.359648	-0.01601	-0.11184	0.00199	1.250268
-0.49371								
-0.97265	1.427795	1.92285	2.207967	2.049133	1.753485	1.162873	0.023897	1.045094
-0.50499								
-0.54693								
-0.54693	-0.91829	-1.28769	-1.46682	-0.97053	-1.14219	-1.00185	0.004907	0.548527
-0.54693	0.628077	1.182709	0.675883	0.990858	-0.15061	0.768055	0.114326	1.33332
1.282342								
0.52814	2.48429	3.9875	2.66959	2.579117	2.10187	2.710907	0.05391	1.88563
0.52814	0.020891	0.448717	0.362653	0.273629	1.33944	0.892045	0.001192	1.318549
1.282342	-0.91294	-1.49039	-1.76622	-1.01982	-1.24478	-1.09022	0.008824	0.853279
1.282342	-1.18536	-1.87487	-2.21879	-1.10286	-1.72933	-1.3076	0.000542	1.115927
1.282342	0.401815	0.477539	0.280104	0.242631	1.093255	0.805544	0.007568	0.850625
1.55349	0.516111	0.74826	0.680649	0.339785	0.720765	0.834463	0.042195	0.494679
1.55349	-1.297	-2.10406	-2.12829	-1.4808	-1.75696	-1.41223	0.002959	0.831293
1.55349	-0.71731	-1.17409	-1.35971	-0.57823	-1.03288	-0.85092	0.00931	0.781482
-0.62178	-0.55162	-0.36325	-0.1634	-0.28563	-0.2632	-0.81083	0.139642	0.647428
-0.62178	0.798779	0.522124	0.438949	0.321559	1.352428	0.495642	0.097223	1.030869
1.464323								

1.464323	-0.31948	-0.71356	-1.11092	-0.47587	-0.33884	-0.36622	0.047239	0.791432
1.464323	-0.3729	-0.5822	-0.80483	-0.25853	-0.36605	-0.30506	0.001266	0.546293
0.427183	0.311239	0.551442	0.808295	0.539797	1.011815	0.854913	0.006573	0.700576
0.427183	0.762366	1.14725	1.213403	0.686499	0.757811	0.592855	0.02343	0.620548
0.20783	0.395713	0.829956	1.041935	0.783494	0.669835	0.606182	0.017364	0.646221
0.20783	-0.35462	-0.62037	-0.79983	-0.63238	-0.81699	-0.58186	0.030952	0.462372
0.20783	-0.71946	-1.12694	-1.3052	-0.78575	-1.0683	-0.91806	0.01309	0.585743
0.20783	-1.01497	-1.43149	-1.534	-1.12017	-1.23683	-1.02737	0.009854	0.519031
0.20783	-1.44735	-1.26501	-2.14813	-1.34706	-1.95326	-1.69687	0.172149	0.883115
0.20783	-0.9087	-1.6001	-1.69113	-0.9322	-1.60052	-1.23465	0.009818	0.782437
0.20783	-1.40502	-1.86182	-2.14288	-1.06962	-1.27039	-1.27886	0.002223	1.073264
0.20783	1.640557	0.974758	0.056042	0.514245	0.745235	1.371475	0.01077	1.584516
0.20783	-1.219	-1.82242	-2.0033	-1.27314	-1.67105	-1.27891	0.019487	0.784293
0.20783	-0.30307	-0.33626	-0.50677	-0.42121	-0.4615	-0.51999	0.049308	0.216914
0.108413	0.041232	-0.00824	-0.0103	0.066652	0.003106	0.06707	0.088135	0.077367
0.108413	0.029037	0.074926	0.148374	0.614722	0.353492	0.10377	0.02419	0.585686
0.300107	-0.66482	-1.00333	-0.89152	-0.63074	-0.72654	-0.46746	0.017261	0.535872
0.300107	-0.15412	-0.12798	0.225064	0.246627	0.704588	0.187331	0.003993	0.858708
-0.04983	0.719131	0.668412	1.088631	0.727306	1.021611	0.879118	0.071062	0.420219
0.300107	-0.08708	-0.40713	-0.19786	0.025398	-0.12075	-0.31346	0.061965	0.432531
-0.04983	0.048971	-0.27525	-0.22107	0.384581	0.124321	0.114044	0.002134	0.659833
-0.04983	-0.3677	-0.6324	-0.26376	0.095169	0.089601	-0.16713	0.014341	0.727567
-0.26956								
-0.26956	0.410302	0.790983	1.120111	0.684407	0.555354	0.364863	0.013263	0.755249
0.280422	0.422156	0.778258	0.53923	0.459467	0.546279	0.477703	0.09525	0.356103
0.280422	-0.89917	-1.19115	-1.60287	-1.06094	-1.09359	-1.01528	0.009933	0.703705
0.280422	-0.94116	-1.14682	-1.59774	-1.19439	-1.21878	-0.96685	0.035517	0.656578
0.280422	3.343887	3.201443	2.44327	2.411263	2.961875	2.499613	0.121408	0.932623
1.38499	0.429842	0.862184	1.142696	0.736497	0.992	0.915269	0.020132	0.712853
1.38499	-0.45169	-0.66638	-1.01435	-0.7683	-0.21929	-0.35712	0.014719	0.795055
1.38499	-0.158	0.044677	0.429903	1.460899	0.2093	0.475069	0.011471	1.618901
1.38499	-0.6217	-0.86685	-0.99727	-0.40028	-0.59659	-0.55585	0.003952	0.59699
0.863147	0.437446	0.758376	0.566629	0.160297	0.410108	0.437796	0.019959	0.598078
0.863147	-0.55337	-0.63548	-0.65985	-0.4171	-0.14579	-0.4643	0.000841	0.514062
0.863147	0.777066	0.551038	0.653193	0.154112	0.252506	-0.04413	0.018226	0.821192
1.925133	1.284562	1.370111	1.490558	0.88064	1.86179	1.407137	0.081447	0.98115
1.925133	1.020006	1.271626	1.230045	0.97497	1.648225	1.241858	0.042914	0.673255
1.925133	0.042124	-0.24002	-0.14476	-0.1874	0.457659	0.263239	0.039494	0.697678
-0.20016	1.23351	1.730077	1.670937	0.868156	1.55283	1.26329	0.024708	0.86192
-0.97157	0.762275	1.45897	1.582597	0.892314	1.405955	1.131977	0.00248	0.820321
1.123723	-0.03511	-0.05379	-0.08134	-0.29935	0.232242	0.144051	0.030914	0.531594
1.123723	-0.47711	-0.6089	-0.88402	-0.74499	0.012014	-0.56399	0.006808	0.896033
-0.41533	-0.02302	0.228225	0.547872	0.18235	0.346267	0.194608	0.001288	0.570895
-0.59749	-1.11528	-1.89124	-1.96329	-1.13171	-1.54485	-1.40356	0.001398	0.848009
-0.59749	1.9271	1.972013	1.066645	1.356098	1.385677	1.223671	0.03714	0.905368
1.123723	0.428668	0.561701	0.541795	0.736915	1.075914	0.819559	0.050223	0.647246
2.05502	-0.85527	-1.23934	-1.41474	-0.74413	-0.78516	-0.63398	0.011249	0.780762

2.05502	-0.41013	-1.23162	-1.11967	-0.90425	-1.06163	-0.87204	0.078744	0.821494
2.05502	-0.8938	-1.50015	-1.78968	-1.03739	-1.40491	-1.13756	0.001733	0.895887
2.05502	0.611429	0.615018	0.058089	-0.00915	1.27883	1.113588	0.003497	1.287975
0.286367	0.708701	1.034737	1.294723	0.276798	1.512555	1.367083	0.006475	1.235757
2.05502	0.157312	0.614953	0.934438	0.089278	0.916971	0.897858	0.012722	0.84516
0.286367	-0.25661	-0.33463	-0.93091	-0.69907	-0.57911	-0.56598	0.036968	0.6743
0.286367	0.895881	-0.66316	-0.93322	0.675211	-0.32454	-0.77328	0.11221	1.829104
0.286367	-0.43478	-0.34711	0.28402	-0.08106	-0.34792	-0.37609	0.011629	0.718802
-0.79496	0.321533	0.491442	0.902801	0.264646	0.99411	0.872378	0.111979	0.729463
-0.59045	0.73191	0.906732	1.30175	0.832546	1.064898	0.948614	0.061513	0.56984
-0.59045	-0.07559	-0.74376	-0.93872	-0.59539	-0.29492	-0.49692	0.065055	0.863124
-0.59045	0.332153	0.381551	0.716793	0.484908	0.607169	0.675195	0.091678	0.38464
1.824865	0.841176	1.202241	1.338837	1.150179	1.891335	1.466533	0.002439	1.050159
1.824865	-0.99489	-1.38783	-1.61925	-1.18004	-1.3789	-1.12982	0.005821	0.624353
1.824865	-1.00422	-1.34844	-1.808	-1.16781	-1.61159	-1.33171	0.00027	0.803776
1.824865	-0.2427	-0.82759	-0.9731	-0.79194	-1.0134	-0.73352	0.013662	0.770701
1.824865	0.440928	1.98092	-0.54561	-0.11233	-0.1086	-0.14857	0.083034	2.526528
0.193068								
0.428988	-0.65405	-0.97804	-1.18337	-0.79335	-0.95016	-0.79439	0.022494	0.529317
0.428988	-1.21629	-1.63315	-1.71354	-0.91642	-1.1423	-0.96642	0.000286	0.797119
0.428988	-1.10294	-1.61722	-1.7071	-1.09436	-1.45008	-1.14726	0.007369	0.612747
	2.63781	2.719473	2.184333	2.16445	2.12095	2.149887	0.162156	0.598523
0.193068	-0.61469	-1.10077	-1.16411	-0.71423	-0.86323	-0.70473	0.03115	0.549423
0.193068	-0.53438	-0.83978	-1.03351	-0.49806	-0.4149	-0.28196	0.014995	0.75155
0.193068	0.749586	0.376661	0.075433	1.131382	0.021339	0.302425	0.182712	1.110043
0.193068	-0.16169	-0.36401	-0.17525	0.394492	0.102231	0.170475	0.004526	0.758498
-0.60524								
-0.60524	-0.62673	-1.14189	-1.00316	-0.6208	-1.02484	-1.00584	0.008547	0.521094
-0.60524	0.487986	0.982619	1.076807	0.94912	0.843215	0.596768	0.001687	0.588821
-0.63658	0.627501	0.580024	0.851751	0.725906	0.641941	0.935087	0.110422	0.355064
-0.63658	-0.37293	-0.68289	-0.90907	-0.44276	-0.46003	-0.42625	0.029709	0.536133
-0.63658	1.55215	1.098618	0.404074	0.939353	0.839448	0.952571	0.005766	1.148076
-0.63658								
-0.24865								
-0.24865	-0.133	-0.34009	-0.66075	-0.68002	-0.57389	-0.5401	0.012425	0.547017
0.055418								
0.055418								
-0.20037								
-0.20037								
0.453977								
0.453977	-1.89956	-2.16344	-1.95401	-2.30439	-2.74429	-2.73164	0.170536	0.844727
0.593832	-0.11287	0.088903	0.213895	-0.10909	0.447981	0.242804	0.023407	0.560854
-0.52798	-0.20631	-0.43294	-0.27659	-0.02161	-0.2629	-0.25994	0.00338	0.411338
-0.52798	0.779351	1.165913	1.46242	1.212257	1.09618	0.95158	0.003249	0.683069
0.211537	0.674961	0.687342	0.344084	0.412163	0.378772	0.144312	0.057968	0.54303
0.211537	-0.98421	-1.70101	-1.72606	-1.09102	-1.35668	-1.47933	0.00027	0.741853
0.211537	-1.05896	-1.48092	-1.46283	-0.84777	-1.24203	-0.96427	0.001306	0.63315

0.211537	0.628715	1.045299	1.35625	1.09862	1.42837	0.948638	0.00898	0.799655
-0.30579	2.701767	2.942697	2.729757	2.296633	2.38541	2.06992	0.032407	0.872777
-0.7467	0.357436	0.657821	0.723104	0.495967	0.739484	0.673779	0.099586	0.382048
-0.7467	-0.02059	-1.02177	-1.14452	-0.61045	-0.48852	-0.48618	0.017817	1.123929
-0.7467	-0.1946	-0.61255	-0.97339	-0.4276	-0.83497	-0.63839	0.003677	0.778792
-1.41116								
-1.41116	1.054361	1.6155	1.638553	1.115522	1.64352	1.329847	0.003463	0.589159
-1.41116	0.621619	1.08023	1.431003	0.863872	1.104015	0.795773	0.003112	0.809384
0.273863	0.313427	0.550165	0.635795	0.195246	0.500943	0.607497	0.079844	0.440549
0.273863	-1.12252	-1.52397	-1.66904	-0.98759	-1.24881	-1.09703	0.000986	0.681443
0.273863	0.31563	-0.34382	-0.64613	-0.3435	-0.12268	0.398904	0.179493	1.045031
-0.39423	0.056645	0.082623	0.167253	-0.04058	-0.10685	0.001501	0.005265	0.274104
0.276487	0.729109	1.106741	1.47613	0.954998	1.448215	1.291217	0.017442	0.747021
-0.39423	-0.38445	-0.33398	0.608226	0.307244	-0.87614	-0.57296	0.00343	1.484367
0.276487	-0.52621	-0.43209	-0.50537	-0.40973	-0.50389	-0.37525	0.053583	0.150962
0.276487	0.127256	0.054079	0.092449	0.350978	-0.11845	-0.1621	0.003167	0.513079
0.582363	0.907637	1.424702	1.60282	0.936241	0.777559	0.998044	0.076086	0.825261
0.582363	0.409539	0.630687	0.63961	0.183091	0.032786	0.084261	0.01647	0.606824
1.330103	0.240574	0.248412	0.518175	0.707487	1.121367	0.832225	0.026238	0.880793
1.330103	-0.09409	-0.39299	-0.41881	-0.25833	0.035391	-0.18896	0.001981	0.454205
1.330103	-1.38104	-1.95246	-1.96266	-1.39166	-1.69918	-1.4252	0.002618	0.58162
1.330103	-0.60189	-0.38878	-1.16562	-0.58273	-0.55876	-0.44731	0.0698	0.776845
0.09569	0.355401	0.533235	1.127329	0.836372	0.72346	0.518808	0.011825	0.771928
0.09569	1.244988	0.703796	0.391433	1.12274	0.734081	1.285463	0.097375	0.89403
0.09569	0.316878	0.367351	0.485952	0.619642	0.254362	0.167118	0.068822	0.452524
-0.48783	2.53623	2.591253	2.034327	2.183897	1.77831	1.62613	0.020246	0.965123
-0.48783	-0.55004	-1.12867	-0.94624	-0.38877	-0.63106	-0.6166	0.002673	0.7399
-0.48783	-0.38418	-0.51368	-0.56901	0.046611	-0.38321	0.004081	0.150572	0.615621
-0.76064								
-0.76064	-0.49255	-0.42069	0.309829	-0.0927	-0.69321	-0.50577	0.004472	1.003043
0.345083	1.824087	1.396797	1.298929	1.587323	1.503835	1.361571	0.187171	0.525158
-0.12727								
-0.12727	0.898552	1.3252	1.548941	1.221633	1.439515	1.254127	0.067695	0.650389
-0.08568	0.261287	-0.19925	-0.60477	-0.051	-0.36836	-0.25292	0.002661	0.866052
	-1.50219	-2.058	-2.20178	-1.53364	-1.7816	-1.38957	0.079505	0.812211
0.559667								
0.559667	-0.15339	-0.07951	-0.19317	-0.49003	-0.40442	-0.22649	0.044046	0.410525
-0.42969								
-0.42969	-0.89129	-1.1066	-1.15638	-0.77685	-1.1204	-0.93201	0.043988	0.379533
-0.42969	-1.03288	-1.32327	-1.59213	-0.95682	-1.68126	-1.07994	0.011869	0.724438
-0.42969	-0.7817	-1.29752	-1.49707	-0.86165	-1.2197	-0.96013	0.007612	0.715366
-0.42969	-0.99711	-1.7279	-2.05677	-1.34834	-2.01146	-1.61012	0.006194	1.059663
-0.42969	-0.96932	-0.59887	-1.11736	-0.92392	-1.29683	-0.95508	0.050637	0.697957
-0.42969	-0.48656	-1.16738	-1.36919	-0.65097	-0.78415	-0.80675	0.027785	0.882634

-0.42969	-1.45279	-1.90826	-2.03067	-1.26463	-1.68323	-1.58835	0.006174	0.766047
-0.42969	-1.03204	-1.52413	-1.81174	-1.29903	-1.5781	-1.23281	0.014649	0.779704
-0.42969	-0.69951	-1.28376	-1.55785	-0.82444	-1.45018	-1.24203	0.018352	0.858343
-0.42969	-1.4211	-1.41822	-2.07161	-1.36493	-1.51725	-1.19478	0.008566	0.87683
-0.42969	-1.32025	-1.89279	-2.18604	-1.30932	-1.91198	-1.33823	0.002524	0.876727
-0.42969	-0.88752	-1.3528	-1.35306	-1.11371	-1.37717	-1.15635	0.075519	0.48965
-0.42969								
-0.42998								
-0.42998	-0.27573	-0.83938	-1.36453	-0.75361	-0.94906	-0.16871	0.024047	1.195818
-0.42998	-0.49974	-0.73996	-0.34226	-0.14161	-0.13585	-0.3464	0.017667	0.60411
-0.01548	-0.94444	-1.04255	-1.17942	-0.8223	-0.69766	-0.62882	0.003821	0.550602
-0.05194	0.690312	0.615745	0.427004	0.311209	0.828731	1.006142	0.107124	0.694932
-0.05194	0.183353	-0.00097	-0.01468	0.190134	0.551586	0.403689	0.000338	0.566262
-0.38895								
-0.25212	1.01692	1.514857	1.778457	0.966323	0.887698	0.620233	3.92E-05	1.158224
-0.25212	0.806854	1.50385	1.758403	1.043228	1.081596	0.979787	0.008748	0.951549
-0.38895	0.95197	1.49082	1.77965	0.976703	1.19847	1.054656	0.001972	0.82768
0.57273	-0.22055	-0.04001	0.042861	0.23064	0.119623	0.167688	0.023345	0.451187
0.57273	0.741687	1.106844	1.165157	0.818616	1.444935	1.191747	0.042323	0.703248
	0.743663	1.068857	1.3557	0.793935	1.55273	1.24204	0.026684	0.809067
0.409963	0.564182	0.602671	1.134819	0.719337	1.045522	0.905038	0.027311	0.570637
	-1.01275	-1.31648	-1.30802	-0.77066	-1.17121	-1.03604	0.000749	0.545815
0.409963	-0.84773	-1.38423	-1.66385	-0.96933	-1.32472	-1.00338	0.028212	0.81612
0.409963	-1.20961	-1.53304	-1.56097	-1.01983	-1.2266	-1.10115	0.012289	0.541147
0.409963	1.161743	1.629187	2.037967	1.375297	1.39838	1.21939	0.002087	0.876223
-0.93963								
-0.93963	0.582116	0.895752	1.451007	1.072629	0.561716	0.435434	0.000339	1.015573
0.48231	0.174947	0.679849	1.208666	0.425796	1.10039	0.915663	0.020436	1.033719
0.48231								
0.48231	0.320197	0.528382	0.603654	0.558621	1.617835	0.828933	0.01914	1.297638
0.609857	1.085099	1.362497	1.538223	0.919145	1.64593	1.679727	0.00341	0.760582
0.609857	0.216047	0.282004	0.253848	0.333091	0.485133	0.4959	0.11547	0.279853
	0.867716	1.318487	1.827077	0.906114	0.793538	0.716843	0.001007	1.110234
	0.033597	-0.41485	-0.98005	-0.37813	-0.7287	-0.66465	0.00435	1.01365
0.69339	0.111839	0.029339	-0.15428	-0.40954	-0.1303	0.174899	0.041107	0.584439
-0.47321								
-0.47321	0.596631	0.666667	0.251477	0.058241	0.371	0.419198	0.027493	0.608427
-0.47321	0.15525	0.626341	0.997013	0.656509	0.524007	0.419009	0.008721	0.841762
0.69339	-0.99491	-1.51968	-1.56908	-0.97936	-1.1055	-0.96257	0.014133	0.606516
0.69339	1.60404	1.19851	0.733478	1.360793	1.589995	1.180442	0.009069	0.870562
-0.32481								
-0.32481	-0.56667	-0.71742	-0.26758	-0.53723	-0.8808	-0.8783	0.09238	0.613218
-0.32481	0.721821	0.123085	-0.08583	0.639132	0.381276	0.868866	0.230821	0.954694
0.01453	0.478901	0.573609	0.508085	0.438098	0.602044	0.648668	0.13587	0.21057
-0.51355	0.325667	0.374335	0.352136	0.71225	0.577982	0.488197	0.01762	0.386583

-0.51355	0.664905	0.727755	0.87324	1.44782	1.26999	0.929623	0.004493	0.782915
-0.58005								
-0.62588								
-1.16866								
-1.16866	0.271772	0.513727	0.824058	0.422699	-0.02065	0.195611	0.081184	0.844704
-0.62588	-0.3376	-0.47251	-0.60491	-0.30237	-0.26711	-0.19185	0.005577	0.413061
-0.62588	-0.02514	-0.23056	-0.28372	-0.03663	0.017457	0.316971	0.081812	0.60069
-0.06972	0.193487	0.535943	0.485078	0.296742	0.64662	0.431894	0.020569	0.453133
-0.06972	0.867149	1.531844	1.668578	1.183047	0.410407	1.118049	0.17654	1.258172
0.536557								
0.536557	-0.02192	0.456337	1.187473	0.613436	0.387776	0.289	6.70E-05	1.209388
0.536557	-0.15872	-0.04546	-0.07704	-0.20707	-0.67652	-0.47214	0.004853	0.63106
-0.59227								
-0.59227	0.706651	1.075995	1.443963	0.918011	0.433635	0.368957	0.015511	1.075007
-0.54881	0.682036	0.973499	1.32447	0.943473	0.98746	0.923047	0.028674	0.642434
-0.98688	0.325797	0.520244	1.088371	0.546692	0.458492	0.579869	0.001369	0.762574
-0.98688	-0.0016	0.524103	1.518233	1.180623	0.62592	0.234538	1.49E-05	1.519832
-0.54881	-0.56504	-0.99485	-1.16667	-0.74005	-0.91232	-0.82944	1.34E-05	0.601629
-0.54881	-0.4275	-0.36178	-0.16396	-0.15363	-0.34289	-0.46872	0.01183	0.315083
-0.55451	0.220291	0.133551	0.513332	0.503259	0.444384	0.442954	0.036443	0.379781
-0.16645	0.576394	0.812868	0.807776	0.160843	0.139677	0.469631	0.095149	0.673191
-0.55451	1.064603	0.890325	1.362452	0.697924	1.011027	0.578026	0.027319	0.784426
-0.16645	0.402482	0.59925	0.999378	0.851089	0.519379	0.316996	0.031649	0.682381
-0.16645	-0.71159	-0.81703	-0.65843	-0.23867	-0.45257	-0.43731	0.015765	0.578361
	1.194533	1.438883	1.28611	0.392437	1.183359	0.798024	0.01494	1.046446
	0.841018	1.203143	1.346397	0.712392	0.870561	0.835333	0.051606	0.634004
-0.43809	0.35597	0.493479	0.923947	0.518335	0.464628	0.510973	0.001602	0.567977
-0.43809	-0.48637	-0.50421	-0.2824	-0.51487	-0.30169	-0.54217	0.181071	0.259773
-0.43809	0.810913	0.697631	0.705049	0.734827	0.552525	0.808423	0.109265	0.258388
-0.83382								
-0.83382	2.022333	2.001677	1.849237	1.345457	2.407245	1.440013	0.205107	1.061788
-1.29081	0.691799	0.543007	0.691455	0.503754	0.38687	0.505744	0.006006	0.304929
-1.29081								
-1.29081	-0.31674	0.013199	0.154229	-0.31964	-0.13953	-0.10663	0.074768	0.473868
-0.4275	2.808277	2.70866	2.164167	2.798467	2.41298	1.985967	0.113627	0.82231
-0.62062								
-0.62062	0.915055	1.8271	1.55951	1.193081	0.830338	0.782519	0.000469	1.044581
1.16458								
1.16458	1.645577	1.99848	1.983703	1.18674	1.31476	1.218353	0.011648	0.81174
1.029873	0.012529	0.269008	0.61473	-0.02371	0.560492	0.598786	0.005578	0.638443
1.029873	-0.60042	-0.95257	-1.07941	-0.62936	-0.12239	-0.76615	0.008543	0.957016
1.029873	1.125257	1.230667	1.126066	0.852598	1.16156	1.191647	0.010338	0.378068
-0.10762	0.835255	0.79322	0.610914	0.91825	1.225532	0.622	0.222409	0.614618
0.33935	0.342669	0.358232	1.01576	0.368574	0.615877	0.548837	0.022342	0.67309
0.33935	-0.41581	-0.63147	-0.60962	-0.20872	-0.02902	-0.35286	0.035323	0.602455
0.33935	-1.15888	-1.78503	-1.75645	-1.26015	-1.63471	-1.39608	0.057115	0.626157
0.33935	0.490794	0.811797	1.120672	0.967632	1.09402	0.823138	0.004904	0.629878

-0.10762	1.000964	1.23483	1.328737	0.990453	1.41878	1.426883	0.078254	0.436431
-0.10762	1.49181	1.179317	1.210722	1.118323	1.79616	1.43993	0.089702	0.677837
0.835717	0.662052	0.797486	0.909519	0.385111	1.098863	1.103823	0.009238	0.718713
0.835717	0.004378	0.63933	1.116911	0.747932	0.42993	0.286173	0.000443	1.112534
0.835717	-0.11312	-0.27085	-0.51766	-0.39284	0.705529	0.376885	0.006498	1.223194
-0.01691								
-0.01691	-0.59111	-0.80621	-0.79941	-0.62927	-0.7993	-0.59251	0.07367	0.215102
-0.01691	-0.56124	-0.57055	-0.608	-0.3615	-0.85439	-0.55304	0.125573	0.492888
-0.05008	0.466261	0.583454	0.646567	0.581754	0.715372	0.732697	0.097543	0.266436
-0.05008	0.254523	0.055884	0.291069	0.661885	0.533813	0.181314	0.055135	0.606002
-0.05008	-0.79637	-0.52914	-0.25106	-0.25664	-0.13471	-0.42547	0.005324	0.661666
-0.07572	0.819476	1.517377	1.794927	1.32252	1.89179	1.40649	0.000222	1.072314
-0.07572	0.778448	1.317533	1.447017	0.692011	1.573935	1.353103	0.001512	0.881924
-0.07572	0.471857	0.603825	0.97303	0.944461	1.49128	0.54797	0.003582	1.019423
0.820857	0.62227	0.634188	0.954942	0.815946	0.265405	0.580775	0.08027	0.689536
0.820857	0.44945	0.485936	0.45745	0.42416	0.722923	0.41654	0.001905	0.306383
0.593875	-0.8645	-0.75966	-0.73725	-0.49527	-0.4993	-0.68322	0.025774	0.369232
0.593875	0.891232	1.121603	0.789779	0.463627	1.179235	1.054837	0.011544	0.715608
0.593875	-0.56492	-0.46522	-0.52974	-0.16035	-0.86183	-0.33545	0.230334	0.701487
0.762017	1.364927	1.387473	1.089445	1.1812	1.56419	1.241547	0.261936	0.474745
0.762017								
0.762017	0.299028	0.624065	0.794067	0.546383	0.982982	0.654423	0.008318	0.683954
1.16798								
1.16798	0.709263	0.965273	1.208253	0.901115	1.003628	0.72348	0.008595	0.49899
-0.79112								
-0.79112	1.340309	0.96376	0.513213	0.460075	0.732835	0.467012	0.035295	0.880234
-0.79112	1.873816	0.938712	1.311603	0.608996	0.856356	0.600164	0.109644	1.273652
-0.5528								
-0.54186								
-0.54186	-0.45182	-1.00741	-0.81665	-0.74283	-0.93024	-0.74594	0.00568	0.55559
-0.54186	-0.78013	-1.11622	-1.51557	-1.06452	-1.18415	-0.92923	0.002541	0.735432
-0.13798	-0.54157	-0.37834	-0.33621	-0.18013	0.006404	-0.26998	0.006351	0.54797
-0.13798	0.020677	0.427568	0.523532	0.366598	0.161481	0.256664	0.043115	0.502855
-0.13798	3.412557	2.00778	1.244772	1.442559	1.685735	1.347606	0.056293	2.167785
1.26649	0.915982	1.609303	1.410096	0.785288	1.579485	1.164963	0.093502	0.824015
1.26649	-0.39405	-0.07144	-1.00696	-0.202	-0.54008	-0.30398	0.139508	0.935517
-0.41136								
-0.41136	0.889401	1.272423	1.550343	0.691645	0.197081	0.401723	0.000693	1.353262
0.367387	0.349752	0.385947	0.681258	0.440825	0.07056	0.426125	0.00209	0.610697
0.367387	-0.87675	-0.98282	-0.60731	-0.66823	-0.71995	-0.88904	0.029029	0.375519
0.367387	-0.34468	-0.34016	-0.4609	-0.4075	-0.39558	-0.29726	0.069362	0.163644
0.058557								
0.058557								
1.055963								
1.055963	1.49101	1.559987	1.687213	1.50364	2.28521	1.79139	0.029467	0.7942
1.055963	0.285198	0.168432	0.385001	0.371578	0.988084	0.620606	0.095346	0.819652
1.055963	-1.26168	-1.57362	-1.62543	-1.06797	-1.37237	-1.16967	0.002228	0.557457

1.055963	-0.40467	-0.14636	0.102789	-0.09248	0.028986	-0.16671	0.034936	0.507458
3.254057	1.860523	1.723923	1.884393	1.102963	1.66523	1.26811	0.0403	0.78143
3.254057	-0.2272	-0.27326	-0.55655	-0.41434	-0.3706	-0.29724	0.087976	0.329346
3.254057	-0.33656	-0.40906	-0.54367	-0.36377	-0.4687	-0.3659	0.108159	0.207108
0.416903								
0.416903	-0.01802	-0.40093	-0.00072	0.820774	0.551557	0.254149	0.008352	1.221703
0.416903	0.038425	-0.06552	0.434304	1.275383	0.556959	0.450815	0.001005	1.340906
0.101183	1.131773	1.75369	1.719567	1.003184	1.635025	1.365967	0.001587	0.750506
0.101183	-0.02761	0.007598	0.3739	-0.00358	-0.00985	0.074468	0.144022	0.401511
2.87805	-0.60298	-1.0288	-1.33843	-0.64603	-1.10519	-0.76573	0.021563	0.735452
2.87805	-1.03513	-1.87081	-1.99337	-1.3397	-1.68162	-1.38632	0.003687	0.95824
-0.14863	0.558194	1.102	1.58888	0.629108	0.869341	0.792328	0.00492	1.030686
-0.14863	-0.01297	0.180514	-0.02476	-0.32132	0.363901	0.259487	0.002202	0.68522
-0.69582	-0.40758	-0.68129	-0.78996	-0.60946	-0.49291	-0.63344	0.009209	0.382381
-0.69582	-0.05365	-0.05227	0.435783	0.72059	0.467002	0.035661	0.004713	0.774239
-0.00189	-0.27556	-0.15111	-0.4095	-0.05044	0.108788	-0.18499	0.041967	0.518292
0.018152	-0.388	-0.84373	-1.0812	-0.46823	-0.90367	-0.67591	0.000539	0.693201
-0.57702								
-0.57702	-0.10568	0.247855	0.334846	0.004423	0.354438	0.045631	0.049739	0.460121
	-0.23631	-0.12754	-0.17891	-0.54449	-0.4966	-0.24364	0.007436	0.416953
	-0.48678	-0.49168	-0.72971	-0.78158	-0.71085	-0.47459	0.003892	0.306989
-0.83029								
0.677757	-0.02709	0.122939	-0.06074	-0.4005	0.122204	0.130222	0.028098	0.530718
1.441653	1.87024	2.234773	2.260877	1.082552	2.210255	1.96476	0.031523	1.178325
1.441653	-0.50273	-0.31499	-0.57594	-0.01796	-0.38815	-0.21683	0.152065	0.557986
1.441653	0.0067	0.202664	0.334461	-0.09845	-0.20305	-0.11422	0.087489	0.537507
0.677757	0.514706	0.466107	0.784406	0.300914	1.124635	0.931936	0.028749	0.823721
-0.83029	-0.11355	0.321376	0.664001	0.592623	0.497905	0.258003	0.003915	0.777549
-0.83029	-0.59072	-0.87058	-0.40497	-0.63764	-0.90351	-1.1735	0.021302	0.768525
0.416173	0.786015	1.267667	1.50193	1.233047	0.757324	0.686224	0.002338	0.815706
0.416173	-0.13804	-0.21228	0.448008	-0.21887	-0.88384	-0.36041	0.009747	1.331851
1.314842								
1.314842	0.96688	0.067757	-0.51235	-0.17322	-0.34898	-0.23145	0.073818	1.479228
1.314842	-0.75354	-1.02462	-0.83648	-0.34911	-1.08181	-0.59214	0.093233	0.7327
1.314842	-0.76562	-1.40582	-1.48245	-0.82504	-1.12214	-0.86705	0.016183	0.716835
-0.78058								
-0.78058	-0.35094	0.024094	0.191258	0.075924	-0.37409	-0.29456	0.005409	0.565351
0.58018	-0.72665	-1.0044	-1.14642	-0.80651	-0.79061	-0.67694	0.010369	0.469472
0.58018	0.878511	1.483603	1.50883	0.886483	1.642545	1.234683	0.004081	0.764034
-0.0048	-0.49648	-0.66548	-0.63179	-0.3708	-1.12698	-0.50795	0.028565	0.756176
0.58018								
-0.0048	-0.79891	-0.49203	-0.38785	-0.33696	-0.72579	-0.63622	0.137648	0.461949
0.220082	0.988723	1.2431	1.459673	1.14075	0.91085	0.784362	0.000609	0.675311
-0.5326	1.527203	1.641167	1.77154	1.416943	1.30797	1.184427	0.009539	0.587113
	0.242559	0.615186	1.122121	0.474381	0.494044	0.444457	0.001849	0.879562
1.232117	-0.61547	-1.24374	-1.16148	-0.96259	-0.94216	-0.54477	0.140806	0.698968
1.232117	-0.246	-0.15707	0.286016	-0.01315	0.005961	-0.09841	0.011878	0.532014

-0.42581								
-0.42581	0.857431	0.808073	1.20462	0.872739	0.656453	0.585252	0.027542	0.619368
	-0.4804	0.009361	0.356	-0.39825	-0.11651	-0.41801	0.014695	0.836403
0.705783	1.078057	1.194922	1.230721	0.968281	1.45094	1.29073	0.104434	0.482659
1.232117								
0.705783	-0.01633	0.745005	1.288563	0.966759	0.751826	0.106022	0.000922	1.304895
0.705783	0.282683	0.070309	0.150894	0.871881	0.606166	0.349858	0.000162	0.801571
-0.2439	0.151427	0.390316	1.025983	0.383353	0.419482	0.253371	0.019208	0.874556
-0.2439	1.208263	0.052592	-0.11946	0.305715	0.338337	0.193153	0.126824	1.327722
-0.2439	0.248819	0.789045	0.962657	-0.10939	0.402383	0.306326	0.001282	1.072047
-0.17135								
-0.17135	0.266478	0.771333	1.247687	0.468185	0.650293	0.531821	0.001599	0.981209
	-0.27531	0.038644	0.950988	0.412619	-1.06482	-0.91957	0.000569	2.015803
0.4816								
0.4816	0.572524	0.581431	0.778232	0.586519	1.02186	0.268381	7.35E-05	0.753479
0.295253								
	0.890398	1.156055	1.069227	0.581198	1.07685	0.97715	0.006524	0.574858
0.295253	-0.1348	0.151617	-0.12532	-0.22823	0.028543	0.017609	0.014859	0.379847
0.295253	-0.20325	-0.57417	-0.65306	-0.53107	-0.48723	-0.61886	0.015566	0.449805
0.295253	-0.90207	-1.34729	-1.75326	-1.32463	-1.35919	-1.15533	0.007332	0.851191
-0.39211	0.334134	0.806078	1.172432	0.712011	1.117636	0.905696	0.007282	0.838298
-0.39211	-0.32208	-0.31732	-0.22449	-0.05673	-0.10811	-0.16286	0.042699	0.265351
-0.36523	0.539834	0.903442	1.127197	0.596044	0.990433	0.602001	0.017258	0.587364
-0.36523	-0.04448	-0.08848	-0.31095	0.114419	-0.07371	-0.06138	0.099073	0.425367
-0.53399								
-0.63417								
-0.63417	-1.08219	-1.1159	-1.88742	-1.73184	-1.87536	-2.20119	0.072345	1.119004
-0.63417	-1.72449	-2.02705	-2.13657	-2.31332	-2.59621	-2.41582	0.05957	0.871723
-0.8725								
-0.8725	-0.78967	-1.18351	-2.17338	-2.09009	-2.91661	-2.61131	0.038411	2.126938
-0.8725	1.793543	1.701337	0.912747	0.957475	0.785262	0.688019	0.035232	1.105525
-0.95141								
-0.95141	-1.40652	-2.13824	-2.75319	-2.803	-2.9929	-2.89666	0.031722	1.586377
-0.95141	-1.96682	-2.29674	-2.82614	-3.28047	-3.24004	-3.24511	0.000253	1.313653
0.041697								
0.041697	-1.50764	-1.74119	-2.15643	-2.19263	-2.51114	-2.63513	0.000369	1.127491
-1.5334								
-1.5334	0.367061	0.567026	0.493407	0.375669	0.218342	0.196811	0.171197	0.370214
0.121358								
0.121358	0.14108	0.427081	0.156936	-0.22725	-0.03356	0.128532	0.009029	0.654332
-0.83321	0.76045	1.161068	1.504297	0.683639	1.319565	0.983137	0.030292	0.820658
-0.66704	-0.36345	-0.42658	-0.49426	-0.37969	-0.03759	-0.14197	0.071946	0.456672
-0.51564	-0.58449	-0.69877	-1.03809	-0.62981	-0.89336	-0.77805	0.082339	0.453599
-0.51564	-1.33721	-1.79019	-1.80843	-1.29708	-1.48774	-1.30354	0.054063	0.511357
-0.51564	-1.25839	-1.66227	-1.58605	-1.07394	-1.24156	-0.99826	0.010824	0.66401
-0.83321	0.668603	1.269553	1.62233	0.905	0.819796	0.71075	0.003014	0.953727

0.523635	0.660059	0.648578	1.10404	0.101109	0.861374	1.1294	0.009568	1.028291
-0.46175	0.149111	0.126382	0.426325	-0.01571	0.456905	0.586145	0.042106	0.601859
0.523635	-0.17759	-0.76168	-0.948	-0.24683	-0.71355	-0.46946	0.005271	0.770411
0.523635	-0.82511	-1.30905	-1.51903	-1.01571	-1.12939	-1.0215	0.000996	0.693924
0.523635	1.496807	1.789487	1.011305	1.105867	1.41015	1.486037	0.144197	0.778182
-0.30889	0.371626	0.647682	0.978748	0.338483	0.759531	0.62159	0.001816	0.640265
-0.30889	0.178787	-0.31698	0.314063	0.219738	-0.05033	-0.14728	0.108496	0.631038
-0.30889	1.0755	1.84321	2.125743	1.670683	1.544	1.025788	0.000153	1.099956
0.03579	0.197765	0.350828	0.384291	0.429412	0.232318	0.338013	0.022853	0.231647
0.03579	-0.19606	-0.60676	-0.87787	-0.34377	0.121401	-0.08476	0.006319	0.999275
0.03579	0.845622	0.928691	0.938321	0.61556	1.381955	1.18279	0.001577	0.766395
-0.97795								
-0.97795	-0.52312	-0.35892	-0.94961	-0.80509	-0.99292	-0.85529	0.245723	0.634006
-0.97795	0.546887	1.053934	1.347357	0.701897	1.10461	0.682769	0.011211	0.80047
1.242743	0.213572	0.667394	0.901357	-0.0064	0.491488	0.537369	0.013242	0.907762
1.242743	0.220834	-0.12966	0.099462	0.236903	0.293399	0.266857	0.04782	0.423063
1.242743	-1.1219	-1.69978	-1.81307	-1.26752	-1.44156	-1.17649	0.000544	0.691177
1.242743	0.070816	0.242029	0.881587	0.939315	1.024006	0.507732	0.012426	0.953189
0.35348								
0.35348	-0.83322	-1.49873	-1.53224	-0.89259	-1.14699	-0.93749	0.004536	0.699021
0.35348	-1.07385	-1.54468	-1.568	-0.93049	-1.4065	-1.34403	0.06169	0.637513
0.066733	0.402883	0.585139	0.784358	0.472762	0.996984	0.795359	0.008382	0.594101
0.066733	-0.89894	-1.22368	-1.37575	-0.94306	-1.18127	-0.99638	0.009041	0.476811
0.066733	1.54991	2.405333	1.815307	1.719597	1.93942	1.566703	0.154599	0.855423
1.39113	1.92664	2.203967	2.080563	1.619672	2.66141	2.35685	0.039146	1.041738
1.39113	-0.75982	-0.69303	-0.42658	-0.40538	-0.33606	-0.454	0.063127	0.42376
1.39113	0.948288	-0.68527	-0.33126	0.842952	-1.12196	0.061444	0.063748	2.070253
-0.56773								
-0.56773	-0.95306	-1.63022	-1.65879	-0.92214	-1.28683	-0.96392	0.010113	0.73665
-0.56773	0.486954	0.997769	1.304673	1.021143	1.23957	0.970293	0.017293	0.817719
0.067198	0.141722	0.304101	0.403299	0.97598	0.945852	0.528554	0.022376	0.834258
1.44841								
1.44841	0.262814	0.397057	0.681812	1.015347	1.317755	0.838952	0.006359	1.054941
1.44841	-0.12224	-0.11531	-0.09135	-0.24227	0.220246	0.235953	0.042638	0.478227
0.067198	0.519298	0.32394	0.109254	-0.26398	0.64097	0.249448	0.042183	0.904945
0.067198	-0.38865	-1.58163	-1.69444	-1.01126	-1.27788	-1.05537	0.065172	1.305798
0.067198	-0.24136	-0.14068	0.21824	0.241776	-0.00927	-0.20058	0.003035	0.483134
0.567857								
0.567857	-0.70662	-1.33043	-1.57908	-0.78821	-1.28319	-0.919	0.000369	0.872465
0.567857	0.050706	0.42717	0.454594	0.04917	0.324291	0.362805	0.197828	0.405424
0.636572								
0.636572	-0.41182	-0.58246	-0.6944	-0.43044	-0.87087	-0.6878	0.118589	0.45905
0.636572	-1.12525	-1.74833	-1.86924	-1.11348	-1.49263	-1.14291	0.027481	0.755753
0.636572	-0.88152	-1.19038	-1.60588	-0.84659	-1.10076	-0.88072	0.012317	0.759296
0.636572	-0.83244	-1.12771	-1.12421	-0.78384	-1.15811	-0.95283	0.017279	0.374264
0.636572	-0.98704	-1.61302	-1.58531	-1.04705	-1.08621	-1.03481	0.000491	0.625977
0.015863								

0.015863	0.820759	1.026861	1.166703	0.555906	0.982475	0.893403	0.005075	0.610798
0.467823	0.743416	1.084328	1.346627	0.753486	1.51533	1.168039	0.051902	0.771914
0.467823	1.427786	0.677528	0.160832	0.872396	0.650531	0.402904	0.029933	1.266954
0.467823	0.157941	0.161463	0.234018	-0.13022	0.215697	0.229984	0.01357	0.364233
-0.66056								
-0.66056	0.871322	1.335447	1.51613	0.736077	0.836679	0.858996	0.014994	0.780053
	0.241633	0.591714	0.975721	0.737198	0.62086	0.450263	0.035446	0.734088
-0.31949	0.632663	0.91417	1.164117	0.624451	1.007889	0.829862	0.014305	0.539666
-0.31949	0.604486	1.141173	1.482353	0.953733	0.96594	0.645946	0.001206	0.877867
-0.78852	6.031493	5.670391	4.992487	4.965403	4.712708	4.376243	0.001677	1.65525
-0.78852	-0.84876	-1.0086	-1.19576	-0.73856	-1.19083	-0.94183	0.018152	0.457201
-0.78852	0.495193	0.736003	1.155	0.554445	0.155641	0.156115	0.000187	0.999359
-0.68683	0.877005	1.155871	1.563	0.914965	1.41199	1.1999	0.092298	0.685995
-0.68683	-0.62805	-1.1396	-1.28714	-0.74377	-0.9272	-0.81688	0.005753	0.659088
0.67064	-0.46476	-0.63765	-0.63833	-0.49718	-0.66527	-0.60276	0.212466	0.200508
0.67064	0.066607	0.348386	0.53099	0.047402	0.316778	0.291925	0.024058	0.483589
0.577643	0.62485	0.772441	1.104389	0.606431	0.909772	0.917537	0.00934	0.497957
0.577643	0.122239	-0.01345	0.328688	0.844124	0.447383	0.385012	0.000591	0.85757
-0.59296								
-0.59296	0.422061	0.742979	0.666804	0.486522	0.540864	0.444892	0.074909	0.320917
-0.59296	0.157294	0.351147	0.704963	0.390777	-0.00104	-0.10391	0.001184	0.80887
-0.17693								
-0.17693	0.478984	1.175343	1.287423	0.900681	0.80312	0.646874	0.000138	0.808439
0.364243	0.548739	0.588109	0.76669	0.405418	0.8137	0.710329	0.029355	0.408282
0.364243	-0.91853	-1.60324	-1.86597	-0.99654	-1.39242	-1.14164	0.002059	0.947435
0.364243	-0.15171	-0.44255	-0.46344	-0.06843	0.022115	-0.15482	0.002176	0.485554
	1.379263	1.396827	1.095018	0.885038	1.32013	1.298783	0.003838	0.511788
-0.71557	1.012357	1.201568	1.290217	0.526223	1.63249	1.448703	0.00486	1.106267
	-0.12787	0.157462	0.308674	-0.00873	0.456706	0.294661	0.06321	0.584572
-0.71557	0.237853	0.333601	0.281286	0.307997	0.432806	0.368085	0.023853	0.194953
-0.71557	-0.44498	-0.69702	-0.46805	-0.50707	-0.53388	-0.50699	0.157781	0.252044
-0.71557	-0.52474	-1.10723	-1.3198	-0.75321	-0.99385	-1.09965	0.03282	0.795061
-0.71557	-0.90578	-1.59511	-1.89515	-1.33588	-1.52658	-1.50464	0.003122	0.989376
-0.71557	0.171573	0.352736	0.266223	-0.4027	0.282436	-0.24159	0.011565	0.755434
0.240703	-0.63377	-0.7936	-0.84381	-0.49976	-0.33554	-0.31291	0.001282	0.5309
0.240703	0.502937	-0.73757	-0.81284	-0.56616	-0.41421	-0.57151	0.070761	1.315773
-0.50496								
0.240703								
-0.50496	0.485927	0.588197	1.172087	0.481097	1.014174	0.818688	0.03436	0.690989
	-0.62206	-0.18456	0.422769	0.110909	-0.28796	-0.36153	0.001065	1.044824
-0.16226								
-0.07272								
-0.16226	0.603528	0.891613	1.422493	0.863709	0.98371	0.818439	0.002915	0.818966
-0.16226								
0.437883								
0.437883	-0.67465	-0.77809	-0.80526	-0.56758	-0.63743	-0.63029	0.041249	0.237682
0.437883	0.24151	-0.36663	-0.69091	-0.01737	-0.07232	0.434575	0.089582	1.125487

0.437883	-0.95196	-1.25308	-1.8542	-0.88856	-1.29523	-0.99674	0.00408	0.965643
0.437883	-1.52967	-1.61326	-1.89783	-1.11125	-1.49264	-1.22193	0.049285	0.786585
0.437883	-0.35873	-0.06161	0.327487	0.185926	0.273578	0.083101	0.018381	0.686215
-1.29688	-0.1102	-0.35783	-0.43624	-0.34934	0.499707	0.462779	0.033484	0.935945
-0.48379	1.676973	1.976477	2.184477	1.385653	1.800835	1.53333	0.019927	0.798823
-1.29688	1.70278	2.163767	2.29595	1.389787	1.78144	1.572243	0.001927	0.906163
-1.10585	1.65292	2.04856	2.16202	1.68867	2.19512	1.799267	0.086529	0.5422
-0.93228								
-1.10585	0.990357	1.59925	2.051573	1.288073	1.259	1.191697	0.004003	1.061216
-0.80087	1.143473	1.59276	1.85606	1.235164	1.444615	1.089012	0.03815	0.767048
-0.80087	1.36459	2.30023	2.350053	1.834073	2.320045	1.70411	0.007451	0.985463
-0.80087	0.232513	-0.08322	-0.71912	-0.1461	0.491505	0.21057	0.01527	1.210625
-0.93228	-0.62333	-0.99748	-1.13449	-1.01484	-1.22677	-1.0017	0.049974	0.603439
-0.93228	-0.37802	-0.71307	-0.73482	-0.55183	-0.0484	-0.1433	0.005388	0.686428
0.071827	0.3966	0.68844	0.784391	0.331641	0.814039	0.681473	0.014756	0.482398
0.071827	-0.61948	-1.14726	-0.86725	-0.64613	-0.6973	-0.69067	0.005492	0.527786
0.071827	0.844781	0.990393	1.241867	1.302097	1.322505	1.39435	0.00442	0.549569
1.702483	0.877758	0.629375	0.522007	0.286083	1.070172	1.136693	0.000725	0.850611
1.702483	1.542003	1.19824	0.881956	0.664434	1.49734	1.71039	0.044199	1.045956
-0.46326								
-0.46326	-0.64201	-0.75827	-0.68452	-0.40229	-0.79199	-0.57059	0.061814	0.389693
-0.46326	-0.81513	-1.15441	-1.41572	-1.17634	-1.06376	-1.08678	0.01366	0.600585
-0.46326	-1.04812	-1.68684	-1.65772	-1.06549	-1.2722	-1.05692	0.021118	0.638725
-0.46326	-0.53365	-1.42973	-1.5715	-0.97144	-1.28214	-0.947	0.025	1.037847
-0.46326	-1.10629	-1.66792	-1.84636	-1.18124	-1.58154	-1.27609	0.006331	0.740071
-0.46326	-1.01146	-1.7646	-1.77959	-1.43385	-1.82215	-1.36759	0.021785	0.810686
1.16369								
1.16369	1.60194	1.881523	1.951047	1.230655	1.815235	1.76978	0.008624	0.720392
0.053417	0.812633	1.272858	1.450637	0.73459	1.018426	1.090523	0.01266	0.716047
0.053417	0.015465	-0.28868	-0.09192	0.060125	0.594125	0.264469	0.004226	0.8828
0.053417	-0.2686	-0.32401	-0.28046	-0.26657	-0.25331	-0.19093	0.115417	0.133077
0.3978								
0.3978	0.498169	0.679768	0.876891	0.734691	0.630171	0.574872	0.019654	0.378721
0.3978	-0.63492	-1.23103	-1.43319	-0.70755	-0.83614	-0.82364	0.002315	0.798263
-0.40307								
-0.40307	1.094238	1.312919	1.705133	1.57201	1.4699	1.168473	0.045088	0.610896
-0.34269								
-0.34269	0.340467	0.63891	0.598589	0.076751	0.553721	0.541212	0.02699	0.562159
-0.34269	0.434912	0.69907	1.295803	1.265323	0.900725	0.75701	0.001331	0.860891
-0.85929	3.529808	3.2998	2.68313	3.007197	2.560005	2.159727	0.004966	1.370081
-0.85929	2.041277	1.414634	0.528634	0.324949	0.147242	0.764817	0.06095	1.894036
0.722142	0.531241	0.486816	-0.23447	-0.00211	-0.12005	0.382394	0.007461	0.765712
1.147158								
0.722142	0.859568	1.01404	0.210556	0.652287	0.698597	0.029304	0.068736	0.984736
1.147158	-1.1113	-1.57845	-1.73837	-1.05522	-1.35776	-1.07606	0.009465	0.683154
1.147158	0.542296	0.007786	0.188604	-0.17251	-0.17629	-0.30962	0.071771	0.851918
1.147158	-0.70553	-1.14795	-1.28349	-0.63357	-0.82767	-0.7264	0.015131	0.649923

1.147158	0.596468	0.556509	0.486242	0.979316	1.257925	1.006497	0.025485	0.771683
0.004793	0.173786	0.522723	0.779913	0.528401	1.37523	0.753428	0.009326	1.201444
0.1545	0.015103	0.211452	0.388419	0.813696	0.603183	0.516798	0.01164	0.798593
0.1545	-0.11398	-0.01411	0.030502	0.201156	0.505778	0.292438	0.020506	0.619758
-0.2403								
-0.2403	1.64161	1.965757	1.930417	1.434657	1.769825	1.639737	0.059706	0.5311
-0.26258								
-0.26258	-0.05515	0.173774	0.026718	-0.24977	0.099639	-0.00477	0.004841	0.423544
-0.26258	1.226716	1.058802	1.924643	1.582489	0.841377	1.377599	0.081833	1.083267
0.301683	0.068552	0.134181	0.515236	0.410049	0.408322	0.07558	0.004997	0.446684
0.301683	-0.80896	-1.07375	-1.03341	-1.00394	-1.06874	-0.79658	0.037677	0.277169
0.301683	-0.12187	0.021439	-0.17717	-0.03944	-0.29648	-0.35682	0.069561	0.378261
-0.49875								
-0.49875	-0.79947	-1.2318	-1.45935	-0.94203	-1.1145	-0.91612	0.000425	0.659879
-0.49875	-0.13255	-0.32163	-0.34948	-0.0135	-0.01705	-0.11319	0.062221	0.335975
-0.61185								
-0.6022								
-0.61185	0.060167	-0.03315	-0.09857	-0.23384	0.277145	0.237308	0.039188	0.51099
-0.61185	0.752016	1.36586	1.734003	1.123477	0.724261	0.562834	0.000158	1.17117
0.07278	1.346857	1.450303	1.43239	1.323617	1.88609	1.493273	0.022621	0.562473
-0.20502	0.391671	0.431478	0.434206	0.519331	0.976521	0.820799	0.000267	0.58485
-0.20502	-0.42432	0.085714	0.198864	0.593661	0.247302	0.05034	0.087347	1.017976
0.07278	0.299009	0.43774	-0.20748	-0.23446	0.120727	-0.03628	0.107881	0.672196
0.07278	-0.38278	-0.28555	-0.12835	-0.03514	-0.3548	-0.30543	0.013093	0.347634
	0.535631	0.550689	1.077062	0.889858	1.091535	0.857246	0.082268	0.555904
	-0.44868	-0.22932	0.132033	-0.27005	-0.16629	-0.42441	5.70E-05	0.580709
0.07754								
0.07754	-0.26813	-0.88437	-1.01222	-0.71982	-0.96833	-0.78638	0.01299	0.744088
0.07754	-0.55922	-0.67888	-0.90133	-0.03414	-0.8607	-0.71082	0.083332	0.867193
1.863333	0.613128	1.175919	1.33409	0.45738	0.873382	0.863652	0.051515	0.87671
1.863333	-0.63014	-0.89026	-0.96886	-0.63419	-0.59533	-0.59851	0.032807	0.373534
1.863333	0.450206	0.166797	0.0948	0.175942	0.746313	0.656655	0.007722	0.651513
-0.75203	0.208467	0.40383	0.717359	0.551148	0.437166	0.206813	0.000511	0.510546
-0.75203	0.332631	0.439278	0.75636	0.398078	0.448173	-0.04045	0.000105	0.796812
0.05854	1.916143	1.713999	1.436699	1.655092	1.393255	0.908311	0.034923	1.007833
-0.75203	1.008625	1.144403	1.247283	0.881998	1.09709	1.00048	0.037563	0.365285
0.05854	0.626301	0.901091	0.934701	1.2587	0.881822	0.785597	0.044065	0.632399
0.05854	-0.25732	-0.32697	-0.6999	-0.26548	0.036692	-0.11531	0.013311	0.736594
0.05854	0.990382	0.94694	0.803088	0.685321	1.823665	1.21326	0.026873	1.138344
-0.62663	0.057591	0.055906	0.168883	0.555633	0.301541	0.086912	0.007629	0.499727
-0.62663	0.187619	0.479482	0.781617	0.966606	0.892196	0.599845	0.004956	0.778987
	0.278769	-0.02922	-0.48338	-0.27044	-0.07542	-0.61802	0.080515	0.896788
-0.62663	-0.6432	-1.25152	-1.01343	-0.3029	-0.88062	-0.84291	0.018182	0.948616
	-0.52387	-0.66278	-0.75555	-0.33648	-0.89136	-0.61451	0.051627	0.554871
-0.76789								
-0.76789	-0.56736	-0.98603	-1.18249	-0.5823	-0.64934	-0.56726	0.066535	0.615225

-0.76789	0.170422	1.065983	-0.00568	0.159908	-0.15526	0.252481	0.067062	1.221242
0.76764	1.507163	1.119988	0.927003	0.778798	1.356592	0.995153	0.139503	0.728365
0.76764	-0.26287	-0.4088	-0.34174	-0.24199	-0.25447	-0.11845	0.017077	0.29035
0.76764	-0.16284	-0.34862	-0.5207	-0.71065	-0.12465	-0.28529	0.048893	0.586006
-1.23932								
-1.23932	1.04453	1.224765	1.504987	1.268901	1.083655	0.921211	0.050215	0.583775
-1.27172	3.442353	2.85781	1.984953	1.950393	2.461395	2.07009	0.007996	1.49196
-1.27172	0.986081	1.26859	1.344487	0.624989	0.589114	0.741514	0.001947	0.755373
-0.21709	-0.37734	-0.15591	0.174743	0.092024	0.009489	0.129123	0.134574	0.552087
-0.128	-0.23506	-0.28715	-0.03058	-0.08653	-0.15685	-0.06706	0.206419	0.256563
-0.128	-0.42633	-0.4982	-0.53095	-0.214	-0.15624	-0.39569	0.134585	0.374715
-0.128	-0.26283	-0.55997	-0.40655	-0.13414	-0.07339	-0.43243	0.089325	0.486582
-0.65801	0.604506	0.984203	1.408017	0.818216	1.295525	1.108175	0.003856	0.803511
-0.21709	-0.81587	-1.49866	-1.56103	-1.09019	-1.48397	-1.30268	0.020507	0.745168
-0.21709	-0.69361	-1.0684	-1.2132	-0.71222	-1.06818	-0.81045	0.008758	0.519585
-0.21709	-0.33596	-0.66706	-1.25304	-0.60832	-0.62961	-0.56384	0.013388	0.917078
-0.21709	-1.0539	-1.50908	-1.91531	-1.26869	-1.57575	-1.39396	0.010907	0.861412
-0.21709	-0.71382	-1.28795	-1.47646	-0.70743	-0.94205	-0.73361	0.001925	0.769023
-0.21709	-0.40784	-1.09908	-1.12628	-0.90219	-1.07905	-1.00605	0.108912	0.718438
-0.82681	0.766439	1.027921	1.05101	0.734536	0.673943	0.731655	0.068874	0.377067
-0.3487	0.936644	1.068887	1.51725	0.843177	0.729566	0.866127	0.016012	0.787684
-0.3487	1.045397	1.44242	1.414777	1.005089	1.26583	1.101498	0.042703	0.437331
-0.82681	0.778385	1.398757	1.743937	1.092207	1.21657	1.076977	0.002987	0.965552
-1.05057	0.863969	0.763125	1.291877	0.630321	0.682161	0.646473	0.009823	0.661556
-1.05057	0.473551	0.459894	0.449137	0.077782	0.695998	0.598793	0.00084	0.618216
-1.05057	-0.35023	-0.37905	-0.22098	0.111295	-0.35913	-0.32979	0.000964	0.490344
0.069027	0.398669	0.617174	0.945138	0.374239	0.863999	0.864269	0.004141	0.570899
0.069027	3.10853	3.846763	3.043493	4.148333	3.0002	3.95476	0.251622	1.148133
0.069027	-1.07255	-1.39843	-1.65558	-1.1898	-1.34323	-1.15153	0.004275	0.583034
0.069027	-0.89633	-1.54025	-1.91329	-0.98433	-1.50076	-1.06413	4.88E-05	1.016959
0.069027	-0.6483	-1.05251	-1.37047	-0.85051	-1.12807	-0.89604	0.001515	0.722164
0.069027	-0.4909	-1.03873	-1.46789	-0.66549	-0.93355	-0.8534	0.014137	0.976992
0.069027	0.564222	0.677979	0.759457	0.619511	0.933826	0.770552	0.008936	0.369604
-0.11292	0.013608	0.499517	1.129839	0.10888	0.870509	0.600691	0.001104	1.116231
-0.11292	0.547653	0.921194	1.577949	1.238091	1.36897	0.588597	0.028028	1.030297
-1.20534								
-1.20534	1.489703	1.42223	1.73658	0.973703	0.767046	0.61395	0.026554	1.12263
-0.253								
-0.253	-0.62505	-1.10877	-1.06983	-0.79521	-1.11399	-0.86433	0.020315	0.488937
-0.253	-0.36795	-0.64433	-0.87624	-0.56775	-0.56396	-0.48171	0.000893	0.508298
-0.04418								
-0.04418	0.634806	1.16723	1.265057	0.605738	0.939575	0.685229	0.023038	0.659319
-0.03816	0.180495	0.439565	0.66364	0.278034	1.060774	0.776508	0.003969	0.880279
-0.03816	0.872996	1.41031	1.813077	1.277743	1.040905	0.815382	0.000822	0.997695
-0.22569								
-0.22569	0.106682	0.074162	0.13475	0.373737	0.74164	0.718853	0.004982	0.667478
-0.22569	1.583433	2.13374	2.368137	1.624017	1.45981	1.166199	0.015571	1.201938

0.353123								
0.353123	0.676578	0.554084	0.033413	0.209727	0.746076	0.813962	5.86E-05	0.780549
0.003357	0.567449	0.442088	-0.21394	0.100206	0.349329	0.629066	0.018588	0.843006
0.003357	-0.43099	-0.64238	-0.90929	-0.41855	-0.47313	-0.50435	0.004151	0.490746
0.003357	-0.53722	-0.81988	-0.98912	-0.28493	-0.74866	-0.62393	0.101782	0.704188
-0.90195	-0.20103	0.219131	0.350732	0.311368	0.39227	0.153624	0.016064	0.593303
-0.90195	0.047591	0.243085	0.901892	0.652122	-0.0111	-0.06566	0.007195	0.96755
0.302957	0.334027	0.291847	0.269964	-0.0217	0.299301	0.30614	0.044158	0.355728
1.187147	0.108192	0.485223	0.70809	0.49724	0.554958	0.331943	0.008701	0.599899
1.187147	1.413203	1.449413	1.133276	0.860838	1.330545	0.898618	0.032396	0.588575
-0.41518								
-0.41518	0.102254	-0.18676	-0.11972	0.170468	0.063237	-0.08823	0.051913	0.357227
-0.41518	-0.86261	-1.20178	-1.45121	-0.90013	-1.30735	-1.06588	0.006058	0.5886
-0.41518	0.081662	0.256042	0.676149	0.310807	0.322671	0.100069	0.009382	0.594487
-1.48168	-0.59088	-0.10022	0.057788	-0.18032	-0.19769	-0.14627	0.043419	0.64867
-0.95219	0.274385	0.870163	0.925801	0.542334	-0.02414	0.285668	0.01869	0.949944
-0.51089	0.733498	1.0692	1.470727	1.040414	0.913352	0.743664	0.020766	0.737229
-0.95219	0.454366	0.214263	0.54235	0.196583	0.284353	0.25308	0.022601	0.345767
-1.48168	1.460341	2.050587	2.03763	1.710893	1.89092	1.23498	0.015975	0.815607
-0.51089	0.17308	0.127504	-0.52655	-0.15266	-0.20034	0.289946	0.114155	0.816492
-0.51089	-0.73609	-0.97518	-0.6614	-0.48622	-0.81658	-0.79147	0.068296	0.488959
1.394895								
1.394895	1.379883	1.418713	1.068591	0.441827	1.47324	1.451123	0.002485	1.031413
1.394895	-0.41293	-0.76678	-1.13528	-0.70641	-0.93629	-0.75626	0.08277	0.722356
1.394895	-0.41811	-0.85537	-1.05993	-0.62416	-0.97	-0.66902	0.010589	0.641822
1.394895	-0.85433	-1.05715	-0.87964	-0.72673	-1.11457	-1.0515	0.04093	0.387831
-1.2107	0.916384	1.077828	1.046791	0.567325	1.34719	1.119631	0.020485	0.779865
-0.7329	0.429597	1.15691	1.807953	0.957616	1.208385	0.69102	0.002787	1.378356
-1.2107	-0.25371	-0.30565	0.399807	0.531947	0.341835	0.005162	0.017099	0.837594
-1.2107	0.350733	1.047467	1.6393	1.273553	0.845109	0.495478	0.000851	1.288567
-0.43059								
-0.43059	2.795117	2.756323	2.444213	2.17654	2.23002	2.061793	0.15098	0.733323
2.174405	1.798953	1.852216	1.320097	1.512327	1.186862	0.880957	0.056526	0.971259
2.174405	-0.3746	-0.48659	-1.42858	-0.87429	-0.94887	-0.54966	0.028896	1.05398
2.174405	-1.21492	-1.96767	-2.21458	-1.0143	-1.55535	-1.08677	0.000456	1.200286
2.174405	-1.30437	-0.69232	-1.79112	-1.10077	-1.58386	-0.79917	0.114124	1.098804
0.77172	3.45734	3.46499	2.721856	2.664692	3.320961	2.592243	0.182384	0.872747
0.77172	0.0226	-0.46569	-0.92166	-0.49752	-0.51003	-0.61933	0.004228	0.94426
0.77172	-1.04646	-1.31136	-1.73963	-1.09555	-1.23806	-1.18336	0.000202	0.693174
-0.48739	0.478251	1.018697	1.234463	0.187385	0.279597	0.541017	0.00042	1.047079
-0.48739	-0.21339	-0.33971	-0.67255	-0.37801	-0.44329	-0.54311	0.011287	0.459156
-0.48739	0.94812	0.976186	1.279593	1.243493	0.239665	0.055513	0.003879	1.22408
0.350727								
0.350727	-0.0747	0.156199	0.649066	-0.51009	-0.80715	-0.6062	0.000429	1.456211
0.350727	0.473001	1.200503	1.305173	0.595132	0.029873	0.026156	1.79E-05	1.279017
0.14501	1.461731	1.093112	1.341679	1.009725	1.15194	1.055841	0.134141	0.452006
0.14501	-0.0921	0.285559	-0.36598	0.41397	0.446753	0.170452	0.000664	0.812734

0.14501	-0.30254	-0.41361	-0.2119	0.121097	-0.06318	-0.139	0.021833	0.534705
0.051477	0.532145	0.757745	0.884771	0.60021	0.899304	0.639339	0.046555	0.367159
-0.67371	0.537528	0.898511	1.14129	0.776714	0.769858	0.754284	0.00263	0.603762
0.051477	0.65204	1.045929	1.254978	0.905225	1.022407	1.00485	0.041029	0.602938
-0.67371	0.973017	0.041934	-0.19831	0.061773	0.332509	0.605431	0.002106	1.171332
-0.67371	-0.54322	-0.22434	-0.20765	-0.10416	-0.46506	-0.28445	0.023634	0.439062
-0.78969								
-0.78969	1.457395	-0.21781	0.089329	-0.3653	-0.49921	-0.4743	0.088041	1.956604
-0.78969	-0.57894	-1.03747	-0.92891	-0.39527	-0.68857	-0.66102	0.011058	0.642199
-0.54188	0.294483	0.333671	0.587139	0.779649	0.545585	0.387147	0.058059	0.485166
-0.03079	-1.04424	-1.32203	-1.43117	-1.01665	-1.16683	-1.00715	0.046341	0.424018
-0.03079	0.056601	0.072225	0.440694	0.579977	1.19795	0.356946	0.039284	1.141349
-0.3837								
-0.3837	0.083364	-0.26176	-0.15303	-0.13183	-0.16816	-0.15896	0.029797	0.345126
-0.3837	-0.04227	-0.03568	0.081203	0.165477	0.488737	0.269049	0.013139	0.531003
-0.4437	3.141873	2.481067	2.350817	2.24916	2.718455	1.774449	0.058782	1.367424
-0.4437	0.576528	0.37306	0.234369	-0.06391	-0.16072	0.137277	0.016442	0.737245
-0.4437	-0.84399	-1.28143	-2.1638	-1.35694	-1.8349	-1.46948	0.063995	1.319814
-0.4437	0.302793	0.782906	1.051726	0.682559	0.860462	0.69528	0.004787	0.748932
-0.45722								
-0.45722	1.284287	1.572353	1.745733	1.050187	1.457275	1.421633	0.005302	0.695547
-0.45722	0.863826	0.187668	-1.13604	-0.62888	-0.67932	0.741376	0.071603	1.999866
-0.45722	-0.15599	-0.04668	0.429181	-0.31676	0.018411	-0.151	0.001328	0.745936
-0.70809								
-0.70809	0.218545	0.495366	0.578749	0.491817	0.181306	0.144727	0.000496	0.434022
0.428463	-0.36785	-0.39232	-0.02415	0.005076	0.00255	-0.17554	0.047556	0.397394
0.428463	-0.7724	-1.05756	-0.78545	-0.63779	-0.66949	-0.66085	0.005228	0.419771
0.428463	-1.15159	-1.54485	-1.83285	-1.36739	-1.48011	-1.16388	0.016897	0.68126
0.428463	-1.09348	-1.43788	-1.66159	-1.16436	-1.45256	-1.32545	0.014266	0.568108
0.428463	-1.29789	-1.5268	-2.21277	-1.30712	-1.87255	-1.46884	0.001899	0.914883
0.428463	-0.66075	-0.71458	-1.10767	-0.60958	-1.44318	-0.75266	0.070597	0.833593
0.428463	-1.69034	-2.12813	-2.19955	-1.7069	-2.13085	-1.77167	0.034431	0.509209
0.428463	-1.32563	-1.88961	-1.83775	-1.1422	-1.55063	-1.23309	0.00202	0.747401
0.428463	-0.47909	-0.57594	-0.58096	-0.31184	-0.39222	-0.50812	0.024629	0.269126
0.091425	0.085212	0.402952	0.633442	0.750367	0.776578	0.522155	0.012516	0.691367
0.180787	0.220806	0.490234	0.818174	0.882264	0.87656	0.370647	0.004557	0.661458
0.152278								
0.152278	-1.09588	-1.5214	-1.7344	-1.0549	-1.51222	-1.206	0.020706	0.679497
0.152278	-0.62335	-0.77284	-1.50491	-0.89384	-1.20314	-0.94683	0.020446	0.881561
0.152278	-0.92008	-1.29437	-1.77759	-0.89256	-1.33965	-0.80908	0.049924	0.96851
0.152278	0.62555	0.293901	0.621383	0.610971	0.257806	0.202786	0.01793	0.422764
-0.57342	-0.29302	-0.2373	-0.16632	-0.08653	0.061135	-0.13962	0.007699	0.354159
-0.57342	-1.05511	-1.34214	-1.32273	-1.15163	-1.4046	-1.37378	0.098305	0.34949
-0.92199								
-0.57342								
-0.92199	-0.12437	-0.21932	0.194495	-0.21047	-0.20629	-0.16696	0.075693	0.413819
-0.92199	0.12335	0.128432	0.406809	0.293918	-0.01043	0.031056	0.004309	0.417239

-0.26888								
-0.26888	-0.02102	-0.09141	0.033234	0.208333	0.551053	0.468048	0.032804	0.642459
-0.26888	1.71809	1.942927	2.38539	1.55515	1.72063	1.726167	0.004157	0.83024
1.053967	0.729896	0.486904	1.019512	0.826868	0.627988	0.468647	0.045793	0.550864
1.053967	0.830898	1.023674	1.133327	0.721033	1.17829	1.12484	0.02393	0.457257
-0.98298	1.577587	2.00727	2.220723	1.414293	2.151095	1.82133	0.019647	0.80643
-0.98298	-1.15187	-1.30469	-1.69262	-1.30491	-1.45014	-1.22774	0.024075	0.540753
-0.98298	-0.55215	-0.50388	-0.45506	-0.56966	-0.56127	-0.5484	0.21535	0.114595
0.80053								
0.80053	-0.47568	0.245134	0.205985	-0.00351	-0.41555	-0.03644	0.001795	0.720815
0.80053	3.468543	3.085067	2.270132	1.310174	2.11133	2.19271	0.148186	2.158369
1.62345	0.342444	0.328801	0.323698	-0.05145	0.251572	0.706152	0.004113	0.757602
1.62345	-0.41728	-0.46977	-0.76448	-0.71045	-0.77752	-0.85527	0.093372	0.437993
-0.08021								
-0.08021	-0.67802	-1.20993	-1.36984	-0.67679	-1.03358	-0.95889	0.022687	0.693053
-0.08021	-0.8559	-1.26587	-1.11441	-0.70624	-1.06181	-0.96017	0.053291	0.559632
0.439057								
0.439057	0.907048	1.069063	1.175548	0.891505	0.665283	0.866777	0.032031	0.510265
-0.33792								
-0.33792	0.175909	0.405371	1.496887	1.64211	0.853531	0.632463	0.00184	1.466201
-0.33792	-0.32205	-0.46092	0.155039	0.557908	-0.18225	-0.13428	0.000464	1.018829
-0.33792	-1.61638	-1.86957	-2.26526	-1.9688	-2.67673	-2.59655	0.062685	1.060352
-0.33792	-0.30449	-0.41991	0.016296	0.441752	-0.18582	-0.08836	0.0001	0.861659
1.838217								
1.838217	-0.36664	-0.61539	-0.51471	-0.0673	-0.33796	-0.40535	0.043992	0.548093
1.838217	-0.27471	-0.50104	-0.41931	0.104991	-0.33615	-0.28134	0.010171	0.60603
	1.190115	0.642843	0.897197	0.866367	0.572486	0.553861	0.132673	0.636254
-0.09803	2.006843	1.952077	1.94898	1.428929	1.8824	1.56175	0.186076	0.577914
-0.09803	-0.16791	0.338241	0.560858	0.651807	0.279384	0.124129	0.000461	0.819716
-0.09803	0.748303	1.418627	1.304924	1.085813	1.049125	0.735616	0.026414	0.68301
-0.93282	0.224353	0.518533	1.151422	0.851426	0.353003	0.292157	0.021732	0.927069
1.386973	1.318086	1.667312	1.87165	1.347763	1.852	1.629483	0.097444	0.553564
1.386973	0.196762	0.351113	0.302162	0.328627	0.115149	0.454754	0.047005	0.339605
3.786927								
3.786927	-0.23724	-0.77801	-0.85215	-0.45972	-0.43587	-0.11068	0.08639	0.741468
3.786927	-0.26444	-0.44619	-0.44395	-0.05252	-0.10895	-0.11782	0.003519	0.393671
0.48886	5.79432	6.514053	4.80995	4.793217	5.40455	4.76296	0.04696	1.751093
0.108413	0.453183	0.51556	0.802274	0.25738	0.239501	0.42317	0.114192	0.562773
0.48886	-0.25848	-0.32461	-0.26445	-0.68254	-0.59809	-0.34406	0.049807	0.424055
-0.2385	0.94233	1.49022	1.846937	1.021954	1.63047	1.406667	0.005683	0.904607
-0.2385	-0.48348	-0.85856	-0.87023	-0.45008	-0.79035	-0.53182	0.012287	0.420149
-0.2385	0.038834	0.056729	0.499563	0.64543	0.312684	0.181438	0.000613	0.606596
-0.59749	1.367067	1.618027	1.91377	1.663763	1.782605	1.514377	0.016138	0.546703
-0.41533	0.797071	0.588214	-0.14775	-0.15826	-0.22581	-0.31002	0.037368	1.107091
-0.41533	2.257244	0.773394	-0.08669	0.228145	0.022499	0.118019	0.028808	2.343931
-0.41533	1.383433	1.700623	2.06818	1.714643	1.814695	1.523737	0.003961	0.684747

0.121238								
0.121238	-0.70398	-1.17894	-1.44562	-0.89615	-1.34362	-1.13862	0.003649	0.74164
0.121238	-0.89804	-1.69931	-2.03962	-0.89763	-1.23455	-0.78733	0.005866	1.252283
0.121238	-1.35754	-1.87174	-1.9403	-1.23153	-1.78656	-1.27871	0.005643	0.70877
0.121238	-0.4703	-0.92506	-0.86977	-0.42867	-0.93976	-0.90282	0.09824	0.511086
-0.56365								
-0.56365	-0.09729	-0.02214	0.164268	0.111343	0.52449	0.041969	0.035808	0.621777
-0.56365	-0.34168	-0.48915	-0.40494	-0.33879	-0.36931	-0.38685	0.124301	0.150363
0.499223								
0.499223	1.136709	0.976614	0.850825	1.16033	0.922407	1.093313	0.314676	0.309506
0.018212	1.153673	1.589013	1.413147	0.960765	1.66759	1.446683	0.005597	0.706825
-0.6169								
0.018212	0.063075	0.136562	0.217732	-0.2577	0.504049	0.501248	0.001083	0.761745
-0.6169	0.126902	0.189966	-0.12518	-0.08118	0.360058	0.238725	0.014845	0.485235
-0.6169	-0.16003	0.140427	0.446992	0.18428	0.04196	-0.26704	0.001949	0.714031
0.32741								
0.32741	0.146275	0.452954	0.660024	0.265496	0.594704	0.311944	0.062294	0.51375
0.32741	-0.71472	-0.88649	-0.99506	-0.71891	-0.61381	-0.53546	0.004264	0.459604
-0.30579	-0.61454	-1.00577	-0.91365	-0.89124	-1.08841	-0.81706	0.006324	0.473874
-0.30579	-0.33971	-0.56811	-1.17384	-0.64586	-0.75704	-0.69128	0.005867	0.834129
-0.30579	-0.87143	-1.36659	-1.32595	-0.81534	-1.06785	-0.93096	0.002023	0.551242
-1.38077								
-1.38077	0.587191	0.780095	1.066078	0.88496	0.559315	0.192538	0.011728	0.87354
-0.02342								
-0.02342	1.379949	-0.53428	-0.94986	-0.47225	-0.2824	-0.61394	0.044058	2.329814
-0.02342	-0.13459	0.250986	0.177242	0.413929	0.377758	0.154011	0.020818	0.548514
0.284273	1.059246	1.049931	1.509623	1.08099	1.55769	1.36761	0.030172	0.507759
0.284273	-0.35134	-0.11584	-0.58198	-0.29735	-0.39152	-0.25109	0.124491	0.466146
0.284273	2.390713	1.9608	1.67119	1.452557	1.534835	1.926185	0.104623	0.938157
0.863117	0.729663	1.262147	1.126305	0.805257	1.55883	1.181099	0.008237	0.829167
0.863117	-0.38662	-0.35373	0.436471	0.429952	-0.19639	-0.44975	0.033518	0.886219
0.863117	0.272085	0.487737	0.513937	0.565537	0.880819	0.716043	0.004238	0.608734
-0.67583	1.348411	0.944094	0.509855	0.6671	0.849906	0.935152	0.068538	0.838556
-0.67583	0.662874	0.819102	1.120706	0.98279	1.07289	0.842898	0.112666	0.457831
-0.67583	-0.09006	0.28034	1.008897	0.883185	0.818141	0.271598	0.000224	1.098955
-0.30747	0.454418	0.853758	1.138226	0.914748	1.28823	0.813311	0.002054	0.833812
-0.05194	-0.11874	0.108293	0.324334	-0.14952	0.238521	0.178625	0.120778	0.473852
-0.01548	0.59597	1.149467	1.327723	0.993657	1.32811	0.979726	0.001942	0.73214
-0.30747	0.491433	0.956184	0.809927	0.873068	1.18159	0.931147	0.011858	0.690157
-0.31844								
-0.31844	0.070339	0.129466	0.159837	-0.29352	0.304274	0.151433	0.090737	0.597793
-0.31844	0.586365	0.636189	0.61333	0.478972	0.925867	0.666971	0.039533	0.446895
-0.65094	0.367808	0.5979	0.834402	0.199832	0.224435	0.463899	0.018263	0.63457
-0.65094	-0.00073	-0.11365	-0.28365	-0.46029	0.070332	0.030809	0.005502	0.530624
-0.65094	-0.04616	-0.10463	0.21945	0.032516	0.209559	-0.0115	0.030626	0.324082
-0.35921								
-0.35921	-0.87541	-1.43217	-1.6335	-0.94694	-1.34646	-1.06769	0.004029	0.758094

-0.35921	1.061905	1.149888	1.48675	1.20392	1.38477	1.288103	0.044825	0.424845
-0.71241								
-0.71241	0.394853	0.230452	0.51271	0.311272	0.417931	0.364429	0.256002	0.282257
-0.71241	0.085051	-0.14397	0.503624	0.735401	0.867356	0.408923	0.00438	1.011329
-0.51355	0.23695	0.241543	-0.18115	-0.20754	-0.05808	-0.01044	0.000981	0.449079
0.01453	0.49264	0.288807	0.256916	0.446719	0.993808	0.870483	0.04699	0.736892
0.01453	-0.52243	-0.92726	-1.15868	-0.61164	-0.48731	-0.45209	0.013831	0.706589
-0.42927								
-0.42927	0.634751	0.79806	0.628525	0.190187	0.320092	0.385153	0.003991	0.607874
-0.58005	0.250665	-0.02369	0.391972	0.411376	0.357228	-0.00786	0.073379	0.435068
-0.58005	1.182469	2.1235	2.19201	1.872321	1.094638	1.373554	0.055977	1.097372
-0.17848								
-0.17848	-0.58253	-0.90006	-0.61943	-0.41074	-0.64152	-0.66249	0.003542	0.489318
-0.17848	-0.19364	-0.35966	-0.27265	0.12533	0.085593	0.013415	0.001023	0.484992
-0.08836	0.780432	0.715162	0.202369	0.404354	0.25648	0.238894	0.002515	0.578063
-0.06972	-0.56214	-0.95121	-1.73188	-0.82633	-1.38689	-1.29871	0.007909	1.169735
-0.08836	-0.55493	-0.89958	-0.9272	-0.91358	-0.89859	-0.83457	0.198373	0.372271
-0.08836	1.926587	1.285979	0.042403	1.156106	1.18117	0.197692	0.058761	1.884183
-0.08836	1.873143	1.400337	1.011258	1.118121	0.976411	0.83427	0.040689	1.038874
3.714607	0.445742	0.624112	0.843343	0.279817	0.322127	0.249819	0.016848	0.593524
3.714607	0.982325	1.170197	1.080288	0.742155	1.067761	1.050341	0.026378	0.428041
3.714607	0.861473	0.877267	0.928837	0.234036	0.657626	1.12724	0.011679	0.893204
0.117387								
0.117387	0.270312	-0.55922	-1.05606	-0.4979	-1.10787	-0.66764	0.051979	1.378182
0.117387	1.01799	1.428405	0.054424	0.368311	1.051853	0.314954	0.058889	1.373981
-0.96909	0.172282	0.384131	0.588333	0.161551	0.302457	0.366613	0.052396	0.426782
-1.29081	0.772847	1.328023	1.862883	1.209493	0.933335	0.819286	0.001041	1.090036
-0.96909	1.994283	2.340153	2.292583	1.94706	2.01887	1.729867	0.08552	0.610287
-0.24043	0.71701	0.955206	0.820691	0.734538	1.092678	0.918103	0.105274	0.375667
-0.4275	0.296336	0.157653	-0.03994	0.077855	0.506021	0.432763	0.018985	0.545965
-0.24043	-0.49738	-0.53362	-0.71159	-0.52014	-0.68317	-0.46689	0.124341	0.244707
-0.24043	-0.40091	-0.65139	-0.67571	-0.47207	-0.29997	-0.34428	0.122388	0.375747
-1.08929								
-1.08929	1.035095	1.66806	1.820237	0.884116	0.907289	0.733264	0.004057	1.086973
-1.47372	0.058345	0.538396	0.862564	0.431864	0.495079	0.235577	0.003574	0.804219
-0.83104	1.232773	2.003437	2.200723	1.424213	1.57639	1.252964	0.004101	0.96795
-0.83104	-0.08398	0.594614	1.17059	0.320058	-0.11932	-0.23756	8.10E-05	1.408148
-0.83104	0.593115	1.035871	1.169565	0.567528	1.084155	0.629256	0.015793	0.602036
-1.47372	0.5378	0.511983	0.602293	0.495595	0.223734	0.083317	0.034418	0.518975
0.02018	0.666952	0.489872	0.61661	0.170498	0.381244	0.495529	0.012039	0.496454
0.02018	1.083606	0.733809	-0.22083	-0.23641	-0.37429	-0.46218	0.05433	1.545783
0.02018	0.146535	-0.01314	0.362712	0.040768	0.126462	0.114314	0.021101	0.375849
	0.494125	1.31352	1.59393	0.771832	1.26005	1.267707	0.000647	1.099805
	0.010935	0.353229	0.407411	0.185056	-0.03535	0.067985	0.019716	0.442762
0.429057								
0.429057	-0.1462	-0.34887	-0.05891	0.072913	-0.33074	-0.29987	0.092168	0.421784
0.429057	1.50482	1.42654	1.107632	1.054628	1.224047	1.5073	0.112022	0.452672

0.163213	0.896761	1.108176	1.450574	0.986794	1.290897	1.167997	0.091939	0.553813
0.163213	-0.89324	-1.23501	-1.35403	-0.9208	-1.35069	-1.01446	0.050781	0.46079
0.163213	-0.50165	-0.88038	-1.08107	-0.68046	-0.52019	-0.4579	0.002181	0.623163
-0.41021	0.416563	0.092638	0.352709	0.188171	0.329167	0.300913	0.06757	0.323926
-0.41021	0.33651	1.243077	1.174244	0.468945	1.17123	0.966816	0.046665	0.906567
-0.41021	0.178355	0.315555	0.095593	0.652847	0.81692	0.221224	0.067343	0.721327
0.37783								
0.37783	-0.94871	-1.01003	-0.82254	-0.94459	-1.04967	-0.91515	0.110168	0.227134
0.37783	0.714543	0.569489	0.428891	0.414981	0.672299	0.429589	0.11037	0.299561
-0.68479								
-0.68479	-0.31993	-0.76103	-1.42501	-0.98327	-0.87951	-0.83053	0.015297	1.105082
-0.68479	-1.49622	-2.25053	-2.19521	-1.8035	-2.07268	-1.73189	0.034758	0.754318
-0.68479	-0.85651	-1.29731	-1.46219	-1.06265	-1.37808	-1.092	0.002918	0.60568
-0.68479	-0.06464	-0.51752	-1.00479	-0.43679	-0.54603	-0.36808	0.000401	0.940154
-0.5524								
0.593875								
-0.5524	0.537503	0.49549	0.728415	0.444134	0.405921	0.316081	0.031094	0.412333
-0.37937								
-0.37937	0.820111	0.995076	1.03633	0.76612	1.009579	0.89855	0.039296	0.27021
-0.37937	-1.02256	-1.56295	-1.86511	-1.02997	-1.31223	-1.08592	0.029115	0.842545
-0.52753	0.28669	0.464179	0.812511	0.919	0.98647	0.665816	0.068469	0.69978
-0.52753	-0.94688	-1.05457	-1.22997	-0.91575	-0.88599	-0.91299	0.086575	0.343977
-0.52753	-0.17181	-0.14834	-0.12629	0.026966	-0.02503	-0.14521	0.252252	0.198772
-0.39181	-1.19542	-1.59421	-1.67423	-1.41663	-2.03313	-1.55337	0.010174	0.837713
-0.39181	0.068915	-0.06059	-0.10373	0.476069	0.761463	0.253656	0.04149	0.865196
-0.39181	-0.66287	-0.8041	-0.68992	-0.32721	-0.43558	-0.55226	0.025362	0.476889
-0.5528	-0.85324	-0.71082	-0.51117	-0.55965	-0.96751	-0.89819	0.039739	0.456338
-0.77189								
-0.77189	0.080777	0.237477	0.539799	0.442149	-0.05205	-0.11078	0.049776	0.65058
-0.77189	0.188721	0.726072	0.898573	0.472151	-0.36734	-0.3198	0.007041	1.265908
-0.09582								
-0.09582	0.49347	0.924823	1.357983	0.589152	0.390756	0.113949	0.007999	1.244035
-0.09582	1.492987	2.17914	2.650553	1.580003	1.394015	0.929712	0.000546	1.720841
0.809413								
0.809413	0.294971	0.426289	0.752378	0.335444	0.356739	0.238224	0.014885	0.514155
1.112353	1.068231	1.538103	1.880917	0.774361	1.98218	1.538517	0.033455	1.207819
2.87805	1.020644	1.249347	1.532297	0.827585	1.413985	1.314357	0.052047	0.704712
1.112353	0.733505	1.077372	0.934382	0.824365	1.47222	1.209277	0.072543	0.738715
2.878123								
2.878123	-0.27347	-0.47702	-0.46175	-0.4145	-0.28277	-0.15704	0.029924	0.31998
-0.42381	0.324915	0.54348	0.810285	0.406761	0.999361	0.850143	0.012933	0.674446
-0.14863	0.328278	0.328824	0.902603	0.362022	0.800132	0.56361	0.013417	0.574325
-0.42381	0.894256	1.115287	1.1811	1.332747	0.897938	0.694231	0.081184	0.638516
0.14114								
0.14114	0.193708	-0.75535	-0.11489	-0.30597	-0.73364	-0.60461	0.067295	0.949054
0.14114	-0.75966	-1.47809	-1.53122	-0.60899	-1.15888	-1.14658	0.007941	0.922229
0.14114	-0.83006	-1.22715	-1.58977	-0.86	-1.36861	-1.07379	0.00106	0.759709

0.14114	-0.46522	-0.84495	-1.19659	-0.41601	-0.83005	-0.62634	0.012223	0.780583
-0.08789	0.546784	0.296188	0.638111	0.113038	0.336116	0.289642	0.036742	0.525073
-0.08789	0.081345	0.342969	0.079199	-0.43147	0.052893	0.259009	0.011277	0.774437
-0.08789	0.117575	0.420276	0.733592	0.220118	0.426546	0.315309	0.000341	0.616017
0.018152	-0.33018	-0.5184	-0.18759	-0.15076	0.031583	-0.31543	0.053044	0.54998
-0.69582								
-0.00189	0.772812	1.086846	1.025789	1.010043	1.71001	1.36483	0.001552	0.937198
-0.30705								
-0.30705	-0.10505	0.129168	0.206925	-0.03251	-0.10822	0.028135	0.018153	0.315142
-0.30705	-0.37029	-1.18315	-1.49363	-0.54433	-1.11178	-0.83413	0.012525	1.123341
0.655167								
0.655167	0.330554	0.512076	0.72057	0.492583	0.846631	0.589533	0.01099	0.516077
-0.48441	0.317452	0.635482	1.434383	0.728644	0.228667	0.11151	0.003199	1.322874
-0.48441	0.095476	0.199722	-0.47988	-0.1192	-0.32525	-0.26682	0.150415	0.679602
-0.48441	0.299191	0.635506	1.146077	1.002934	0.424696	0.444554	0.001457	0.846886
0.344553	0.752149	0.862264	1.273577	1.048468	1.143117	1.193786	0.026295	0.521427
0.344553	1.879899	0.099625	-0.18076	0.109687	0.408292	0.111439	0.034859	2.060654
0.344553	0.335437	0.707356	0.985109	0.555025	0.989141	0.840745	0.047349	0.653704
-0.45808	0.139757	0.370788	0.659898	0.23291	0.478144	0.404689	0.002115	0.520141
-0.45808	-0.51734	-0.4664	0.635546	0.532379	0.240883	-0.24495	0.002217	1.152887
0.18422	0.900565	1.033952	1.300613	0.954472	1.42058	1.027243	0.024261	0.520015
0.18422	-0.40663	-0.68704	-1.08784	-0.73777	-0.92782	-0.77551	0.009856	0.681204
0.18422	-1.22291	-1.64711	-1.76321	-1.17287	-1.71417	-1.2874	0.030812	0.590341
-0.75203								
-0.75203	-0.6633	-0.81986	-0.77591	-0.5472	-0.83435	-1.13495	0.125563	0.587747
-0.75203	0.321321	0.198958	-0.50808	-0.11764	-0.43312	0.203125	0.003228	0.829401
-0.5326	0.875703	1.344774	1.303134	0.803633	0.902511	0.901594	0.118921	0.541141
0.220082	-0.48426	-0.52256	0.052518	-0.22524	-0.38753	-0.43692	0.015116	0.575076
0.220082	-0.68962	-0.5583	-0.38333	-0.3528	-0.49397	-0.33092	0.040235	0.358708
-0.16725								
-0.16725	0.662329	1.15341	1.239254	1.058244	0.615477	0.542535	0.040894	0.696719
-0.19893	0.233265	0.431603	0.400091	0.012437	0.665368	0.588018	0.019268	0.652931
-0.30053	-0.34539	-0.84588	-1.29364	-1.12772	-1.27869	-0.95819	0.001616	0.94825
-0.18255								
-0.30053								
-0.18255	-0.50818	-0.84469	-1.05486	-0.49068	-0.88027	-0.54714	0.020197	0.564179
-0.18255	-0.45317	-0.81575	-1.0344	-0.49513	-1.07026	-0.58271	0.00882	0.617089
-0.45775	2.264123	2.361827	1.726267	1.963793	1.47684	1.52639	0.089629	0.884988
-0.45775	0.877854	0.707949	0.141835	0.463828	0.478902	0.530887	0.066037	0.736019
-1.07969								
-1.07969	-0.07649	0.251207	0.778823	0.431335	0.118299	-0.05298	0.003847	0.855315
-1.14147	0.554476	0.732965	1.297383	0.841155	0.709596	0.548486	0.016819	0.748897
-1.14147	0.572803	1.245303	1.486197	0.689939	0.120948	-0.01512	0.003165	1.501317
0.44124	-0.18244	-0.24815	-0.15148	-0.32361	-0.31154	-0.51782	0.065276	0.366344
0.44124	-0.33387	-0.45875	-0.77552	-0.73433	-0.58093	-0.38929	0.016602	0.441647
0.069552	0.344453	0.370954	-0.04948	-0.31768	0.584422	0.504119	0.01159	0.902103
0.44124	1.164094	1.504687	1.599079	1.571047	1.75105	1.437487	0.021991	0.586956

0.069552								
0.069552	0.962333	0.720341	0.0813	0.601258	0.490895	0.449515	0.032998	0.881033
0.069552	0.031343	-0.02714	0.52273	0.813403	0.435527	0.129731	0.002675	0.840541
-0.85283	0.390012	0.636649	0.551012	0.371435	0.455132	0.656062	0.037034	0.284627
-0.85283	0.127174	0.44582	0.793527	0.774759	0.432468	0.294239	0.012292	0.666353
0.05393	0.884741	1.219479	1.178193	0.877254	1.677065	1.46007	0.036729	0.799811
0.05393	0.186313	0.363744	0.673483	0.603143	0.964054	0.720137	0.01047	0.77774
0.05393	0.303564	0.276771	0.5676	0.848298	0.905624	0.657323	0.008	0.628853
-0.21349								
-0.21349	-0.72814	-0.5004	-0.28096	-0.05455	-0.65195	-0.48896	0.002311	0.673582
-0.21349	1.076351	1.316397	1.30495	0.731288	0.949347	0.925565	0.018074	0.585108
0.055607	0.137205	0.08907	-0.32366	-0.48144	0.373622	0.173797	0.005084	0.855064
1.955097								
1.955097	-0.57397	-0.72858	-0.69122	-0.58639	-0.85997	-0.78309	0.073114	0.285997
1.955097	-0.07924	-0.23562	-0.07019	-0.25516	0.158408	0.140466	0.029762	0.413567
0.87345	0.275043	0.190956	0.438552	0.269439	-0.64178	0.037599	0.160672	1.080329
0.87345	-0.29915	-0.36899	-0.25219	-0.53083	-0.71135	-0.65977	0.010338	0.459159
0.87345	0.160794	0.134025	0.250898	0.123136	0.12646	0.078313	0.202484	0.172585
-0.45947								
-0.7002								
-0.7002	0.806621	1.226417	1.50171	1.051731	0.947873	0.686623	0.023267	0.815087
-0.45947	0.452669	0.499124	0.261233	0.358846	0.622161	0.331133	0.038617	0.360928
-0.35549								
-0.35549	-0.73673	-1.29045	-1.24174	-0.61086	-0.8367	-0.70147	0.005917	0.679585
-0.35549	0.351831	0.638152	0.783237	0.577596	0.501388	0.329954	0.08626	0.453283
-0.67391								
-0.67391	-0.00845	-0.07719	-0.02013	0.179889	-0.05606	-0.05636	0.065381	0.257083
-0.67391	1.333343	1.695317	1.982807	1.568817	1.718755	1.413123	0.000507	0.649463
0.223767								
0.223767	0.847131	0.468348	1.348775	2.968863	0.898445	1.613026	0.102232	2.500515
0.223767	0.506058	0.408527	0.774022	-0.05603	0.098542	0.237889	0.198787	0.830049
-0.21765								
-0.21765	-0.82894	-1.43247	-1.77001	-1.17993	-1.23945	-1.0898	0.000551	0.94107
-0.21765	-0.78312	-1.06379	-1.50285	-0.83601	-1.22946	-0.94577	0.005686	0.719729
-0.21765	-0.5934	-1.134	-1.63417	-0.81335	-1.08639	-1.03722	0.003311	1.04077
-0.21765	-0.09445	-0.57434	-0.89109	-0.72298	-0.25443	-0.29982	0.013755	0.796645
1.69614	1.265283	1.006409	0.744713	0.575236	0.507326	0.566441	0.031511	0.757957
1.69614	-0.5432	-0.20643	-0.2411	0.019712	0.42463	-0.02845	0.004482	0.967826
1.69614	-0.17646	-0.54587	-0.13451	-0.67503	-0.29459	-0.38493	0.021178	0.540516
1.678923								
1.678923	-0.69308	-0.82151	-0.80292	-0.40529	-0.94089	-0.63298	0.095001	0.535597
1.678923	0.134457	0.221506	0.335414	0.758235	1.14443	0.626863	0.001794	1.009973
-0.69455								
-0.69455	-0.25899	-0.94148	-0.70465	0.045249	-0.52381	-0.5271	0.114758	0.986724
-0.69455	0.042954	1.017476	1.519403	1.098853	0.323576	-0.20703	0.005557	1.726436
-0.58595	0.359189	0.490129	1.075549	0.361773	0.966298	0.926689	0.003251	0.71636
0.026687	-1.25673	-1.55377	-1.77653	-1.15402	-1.5566	-1.26094	0.041567	0.622511

0.026687	-0.77429	-0.73247	-1.11069	-0.85852	-0.17595	-0.53273	0.004728	0.934734	
1.233923									
1.233923	-0.86354	-1.27668	-1.43496	-1.04759	-1.12516	-1.00602	0.012992	0.57142	
1.233923	0.579573	1.150513	0.552632	0.752274	1.3451	0.787824	0.124305	0.792468	
-1.01235	-0.38583	-0.33484	0.595359	0.197337	-0.48152	-0.29639	0.011354	1.076883	
-0.92452	0.479662	-1.15775	-0.92445	-0.47746	-1.10418	-0.81273	0.035896	1.637412	
-0.92452	0.332121	0.731328	1.099978	0.566858	0.483913	0.310466	0.013331	0.789512	
-0.65949	-0.06608	0.406816	1.160328	0.561934	0.605793	0.358751	0.01027	1.226411	
-0.65949	-0.14732	-0.43832	-0.2269	0.223415	-0.54289	-0.58931	0.000674	0.812721	
-0.65949	-0.55412	-0.72054	-0.24522	-0.4423	-0.67248	-0.416	0.019139	0.475317	
-0.20285	1.211819	1.121417	1.226974	1.02277	1.114584	1.043124	0.087535	0.204204	
-0.20285	0.702528	1.07302	1.292853	0.526377	0.549452	0.643132	0.000628	0.766477	
-0.20285	2.382973	2.183987	2.50475	2.064867	2.640435	2.355057	0.086051	0.575568	
0.01339	-1.11077	-1.65866	-1.83816	-1.12665	-1.22592	-1.08237	0.017812	0.755798	
0.01339	-1.02754	-1.43195	-1.72783	-1.20264	-1.35184	-1.19205	0.001508	0.700297	
-0.88417									
-0.88417	0.784407	1.316556	1.466097	1.032921	1.435185	0.798114	0.031666	0.68169	
0.888423	0.253473	0.403056	0.616881	-0.15238	0.716843	0.765664	0.002829	0.91804	
0.888423	-0.39751	-0.78817	-0.73414	-0.10068	-0.23641	-0.36285	0.009297	0.68749	
0.888423	-0.1985	-0.45468	-0.83829	-0.1958	-0.31646	-0.32751	0.046881	0.642487	
0.391283	1.78609	2.02796	2.018827	1.59738	2.27328	1.939597	0.060076	0.6759	
0.391283	-0.41835	-0.57513	-0.43275	-0.3861	-0.25045	-0.17681	0.009828	0.398319	
0.391283	1.177463	1.248733	1.47962	1.24478	1.510915	1.416433	0.027215	0.333452	
-0.35144									
-0.35144	-0.6463	-0.72503	-0.55651	-0.58029	-0.31372	-0.23543	0.043337	0.489605	
-0.35144	-0.89355	-1.4036	-1.54192	-0.93393	-1.08289	-1.03591	0.003756	0.648365	
2.92325									
2.92325	-0.22273	-0.54796	-0.89925	-0.56924	-0.84891	-0.5274	0.022194	0.676521	
2.92325	0.401699	0.245321	0.457454	0.648117	0.527861	0.435855	0.042478	0.402795	
0.06473									
0.785607	0.065897	-0.07363	-0.13928	-0.05938	-0.40448	0.019567	0.072733	0.470377	
0.06473	0.007115	-0.20176	-0.23846	-0.15307	-0.09508	0.031003	0.054505	0.269459	
0.06473	0.821416	0.959071	0.883836	0.966562	0.866363	1.089038	0.287274	0.267622	
-0.45565									
-0.45565	1.959037	2.215367	2.067843	1.30279	1.67428	1.860167	0.000701	0.912577	
-0.92558	-0.30098	-0.28033	0.120688	-0.16858	0.08226	-0.07264	0.053177	0.421664	
-0.28133	1.512146	1.020076	0.403796	0.602831	-0.20231	0.526853	0.095466	1.714455	
-0.28133	-0.33905	-0.51057	-0.40143	-0.29573	-0.35833	-0.40917	0.088411	0.214837	
-0.97265									
-0.49371	-0.19886	-0.38895	-0.02822	0.025405	0.018281	0.148327	0.002072	0.53728	
-0.49371	0.031109	-0.20864	-0.38058	-0.13574	0.145035	0.266902	0.005332	0.647484	
0.92185	1.06283	1.15414	1.748507	0.319945	0.606813	0.909739	0.005041	1.428562	
0.92185	0.276357	-0.09034	-0.38906	-0.23457	-0.45941	-0.33678	0.068858	0.735764	
0.92185	0.190361	0.258973	0.313207	0.057184	-0.26094	-0.09604	0.001428	0.574143	
-0.50499	0.563818	0.802403	0.370037	0.360851	0.562009	0.758942	0.237106	0.441552	
-0.50499	-0.71844	-1.51475	-1.85138	-1.21978	-1.2166	-1.20954	0.032818	1.132939	
-0.50499	-0.81695	-1.24622	-1.43748	-0.88383	-0.98498	-0.82741	0.003829	0.620529	

-0.50499	0.141741	0.671433	0.947099	0.450591	0.688878	0.432843	0.0016	0.805359
0.415737	-0.43821	-0.82505	-0.88492	-0.48187	-0.59982	-0.55886	0.004155	0.446708
0.415737	1.138533	1.19993	1.720667	1.230497	1.276945	1.16064	0.013549	0.582133
-0.8731	1.1057	1.57454	1.870127	1.247155	1.95675	1.506533	0.004668	0.85105
-0.2841	1.611043	1.789323	1.294107	0.845934	1.446775	1.319357	0.005212	0.943389
-0.2841	0.207997	0.907554	1.359633	0.815653	0.738314	0.507973	1.31E-05	1.151636
-0.62178	-0.11602	0.942422	1.192077	0.596649	0.612369	0.253223	3.80E-05	1.308093
-0.8731	0.792396	0.505671	0.061355	0.047136	-0.54421	-0.17558	0.055459	1.336608
-0.8731	0.388817	0.953432	1.436623	0.844017	0.84087	0.584044	9.92E-06	1.047806
1.815643								
1.815643	0.585306	0.500989	0.577726	-0.07505	-0.05111	0.256935	0.001998	0.660358
1.815643	1.59958	1.460765	1.812063	1.218747	0.949974	0.628309	0.020829	1.183754
2.643987								
2.643987	1.014068	1.244007	1.037934	0.431803	0.616308	0.791494	0.016024	0.812203
0.33663								
0.33663	-0.68906	-0.852	-0.66316	-0.53856	-0.51621	-0.55578	0.019595	0.335784
0.33663	-0.75416	-0.59395	-0.78273	-0.63949	-0.62059	-0.46805	0.192844	0.314682
-0.24035								
-0.71838	1.574987	1.6669	1.85592	1.40753	1.513345	1.38295	0.007828	0.47297
-0.71838								
-0.24035	0.007386	0.416043	0.593377	0.310228	0.360761	0.231286	0.026346	0.585991
-0.24035	-0.55253	-0.87832	-0.94802	-0.64962	-0.8057	-0.71997	0.024776	0.395485
-0.7391	0.39489	0.675959	0.77046	0.348489	0.554019	0.372497	0.081324	0.421972
-0.7391	0.763231	0.772831	0.316747	-0.11687	0.192792	0.32912	0.005682	0.889698
-0.7391	1.168994	1.326821	1.684767	1.193247	1.417455	1.078589	0.001544	0.606178
-0.21697								
-0.21697	1.029709	0.827065	-0.09899	0.300464	0.047299	0.046613	0.08565	1.128703
-0.21697	0.773755	0.610194	0.649168	0.493472	0.625241	0.327648	0.271116	0.446108
-0.30242	0.60054	0.570805	1.043133	0.434089	1.071568	0.932441	0.039156	0.637479
-0.30242	-0.8192	-0.96878	-1.19892	-0.78918	-0.95147	-0.81265	0.024712	0.409741
-0.30242	2.161517	1.715013	1.09356	1.85807	1.75904	1.732037	0.026297	1.067957
0.665433	0.741609	1.081757	0.972071	0.536091	0.453397	1.050281	0.055697	0.628361
0.665433	-0.44184	-1.13936	-0.82531	-0.55483	-0.78451	-0.54392	0.000454	0.697522
0.665433	0.314075	-0.0113	-0.03048	0.602712	0.770943	0.535735	0.01309	0.801423
-0.3282								
-0.3282	-0.92824	-1.53969	-1.53599	-1.07828	-1.16665	-0.99832	0.00169	0.61145
-0.3282	0.300936	0.116316	0.136965	0.082351	0.109228	0.119367	0.027758	0.218585
-0.97157	0.383502	0.655785	0.817542	0.456751	0.594489	0.591973	0.027156	0.43404
-0.20016	0.063944	-0.37925	-0.2476	-0.33767	-0.12674	-0.08136	0.042137	0.443196
-0.20016	-0.39407	-0.05892	0.315247	0.104796	0.014642	-0.08995	0.004818	0.70932
2.137657	0.656947	0.872572	1.091654	0.892493	1.061898	0.742356	0.019265	0.434707
2.137657	-0.03865	0.062979	-0.59501	-0.03927	-0.24012	-0.21381	0.151082	0.657992
2.137657	-0.62685	-1.06019	-1.13952	-0.45996	-1.04692	-0.74807	0.016375	0.679551
-0.79496	-0.73741	-1.24345	-1.52201	-0.75558	-1.26613	-0.92918	0.005957	0.784598
-0.79496	0.000956	-0.59153	-0.47147	-0.02851	0.641367	-0.23397	0.044554	1.232897
0.103617	0.392742	0.437042	0.257213	0.029041	0.342198	0.396996	0.010853	0.408002
0.103617	2.40869	1.66654	1.584934	1.819757	1.99193	1.899493	0.093121	0.823756

-0.11809	0.293483	0.409698	0.981498	0.230392	0.528931	0.603416	0.002164	0.751106
-0.11809	-0.11538	-0.66858	-0.82208	-0.29758	-1.01026	-0.95795	0.053664	0.894885
-0.11809								
-0.11809	0.466315	0.098871	0.545613	0.041859	-0.26258	0.095346	0.006661	0.808188
-0.71955	1.364168	1.295737	1.399237	1.093229	1.516995	1.163487	0.213639	0.423766
-0.05768	0.791691	0.53207	1.158898	0.861127	1.22335	0.875641	0.040847	0.69128
-0.05768	1.111956	1.215423	1.232951	1.459487	1.44591	1.28266	0.252004	0.347531
-0.71955	0.090429	0.304488	0.453908	0.089612	0.584928	0.291085	0.005322	0.495315
-0.71955	0.972179	1.49724	1.82463	1.41152	1.346055	0.981375	3.80E-05	0.852451
	-0.41895	-0.19321	-0.58023	-0.76103	-0.61275	-0.64911	0.023745	0.56782
-0.27839	0.181357	0.463511	0.341456	0.093638	0.656243	0.578678	0.100769	0.562604
	2.260047	2.258753	1.250413	1.559593	1.342446	0.998958	0.022453	1.261089
	-0.96617	-0.9317	-1.75309	-1.28574	-2.57989	-1.50403	0.056154	1.648193
-0.22859								
-0.22859	-1.1534	-1.29679	-1.94525	-2.14071	-2.37516	-2.23549	0.000959	1.221762
-0.22859	-1.16027	-1.20672	-1.67788	-1.03349	-2.97274	-1.7751	0.017461	1.939245
-0.59951								
-0.47498								
-0.59951	-0.07817	0.284241	0.622903	0.085327	0.391041	0.222328	0.050672	0.701073
	-0.6026	-0.89303	-1.2445	-0.7234	-1.26405	-1.03049	0.000283	0.66145
-1.32342								
-1.32342								
1.41539								
1.41539	0.777948	1.132833	1.35056	0.810326	0.832915	0.801602	0.050531	0.572612
1.717953	-0.51386	-0.84446	-0.7249	0.012086	-0.77809	-0.5147	0.012344	0.856548
-0.37756								
-0.37756	0.657304	0.822896	1.27368	0.854866	0.94625	0.773291	0.022519	0.616376
-0.59334	0.479072	0.797112	1.032348	0.075743	0.725463	0.658586	0.004543	0.956605
0.000497								
-0.59334	0.08866	0.326051	0.37782	0.382821	0.283338	0.20175	0.013704	0.294161
0.000497	-0.45442	-1.08949	-1.30108	-0.79616	-1.09408	-0.91948	0.015344	0.846665
0.000497	0.104958	0.627922	0.587179	0.265644	-0.59111	0.147523	0.066428	1.219028
-0.56424								
-0.56424	1.79184	2.257893	2.135073	1.308803	1.23696	1.112737	0.000517	1.145157
1.327057								
1.327057	-0.75032	-1.34147	-1.35214	-0.68994	-1.11501	-0.85736	0.001449	0.662202
1.327057	-0.63907	-0.96607	-1.08997	-0.62278	-1.03372	-0.70466	0.014876	0.467197
0.239123								
0.239123	0.395569	0.821759	0.593793	0.226289	0.606488	0.474602	0.001066	0.59547
0.59779	0.486614	0.608309	0.528135	0.337848	0.137377	0.071573	0.001841	0.536737
0.59779	0.260846	0.482128	0.764866	0.810232	0.418825	0.382108	0.000406	0.549386
0.503113								
0.503113	0.934004	1.471707	1.69404	0.798815	0.715071	0.566011	0.001497	1.128029
1.325203								
1.325203	-0.33423	-0.36195	-0.2755	-0.01585	-0.09454	-0.54995	0.035226	0.534101
1.325203	1.56065	1.963103	1.025945	1.187746	0.519171	0.533455	0.050036	1.443932

1.629213	0.191185	0.251602	0.771798	0.131872	0.346299	0.43054	0.015271	0.639926
1.629213	-0.2701	-0.24901	-0.18888	-0.11	-0.53803	-0.38678	0.016423	0.428026
1.629213	1.214885	1.44788	1.290457	1.02778	0.717245	0.539935	0.002541	0.907945
-0.12383								
-0.12383	-0.64404	-1.48011	-1.23751	-0.9531	-1.05251	-0.93474	0.011164	0.836067
-0.12383	-0.74951	-1.2723	-1.29151	-0.61536	-0.71293	-0.79765	0.118371	0.676147
-1.49084	0.6075	1.331707	1.725137	1.159085	1.22259	0.668355	0.001268	1.117637
-1.49084	-0.83061	-1.03231	-1.13285	-0.90083	-0.70908	-0.74252	0.00202	0.423775
-1.49084	-0.52308	-0.34402	-0.13213	-0.14258	-0.50087	-0.49984	0.021556	0.390947
-0.06327								
-0.06327	-0.80797	-0.85633	-0.6162	-0.59914	-1.15857	-0.77141	0.056125	0.559422
-0.12615	1.248208	1.707997	1.90218	1.205595	2.042605	1.712563	0.03011	0.83701
-0.62278	0.947244	1.13758	1.24804	0.766274	1.027391	1.016803	0.007825	0.481766
-0.62278	1.087	1.303234	1.66586	1.041508	1.46163	1.319633	0.039154	0.624352
-0.12615	-0.52408	0.026075	0.628444	-0.04651	-0.15905	-0.24478	0.000257	1.152523
-0.12615	0.340632	0.534505	0.749658	0.466959	0.566005	0.576257	0.024926	0.409026
0.204163								
0.204163	-0.84592	-1.25045	-1.31221	-0.89252	-0.7052	-0.70318	0.010084	0.609031
0.204163	0.022877	0.24851	0.553935	0.661447	0.555242	0.367208	0.012768	0.63857
-0.12486	-0.08576	0.189437	0.553952	0.09382	0.900122	0.492666	0.028285	0.985881
-0.12486	-0.86625	-1.38363	-1.47089	-0.9035	-0.97826	-0.84341	0.045442	0.627475
-0.12486	-1.30729	-1.44575	-1.4106	-0.90933	-1.14229	-1.06276	0.034001	0.536416
-0.12486	-0.19767	-0.17091	0.184906	0.425215	0.483873	0.267928	0.002784	0.681545
0.15583								
0.15583	-0.0405	0.290547	0.857361	0.049487	0.564617	0.343375	0.019918	0.897858
2.299547	1.131292	1.449939	1.529087	0.513294	1.534005	1.44707	0.038077	1.020711
2.299547	-0.99141	-1.4723	-1.60938	-1.04994	-1.11932	-0.86559	0.045912	0.743786
2.299547	-1.22164	-1.44931	-1.56726	-1.2287	-1.17052	-1.03989	0.007393	0.527371
-0.02802								
-0.02802	0.469307	0.779992	0.820387	1.030348	1.022765	0.704499	0.003646	0.561041
0.494513	0.2756	0.472433	1.014126	0.153511	0.798742	0.526001	0.010324	0.860615
0.494513	1.4806	1.765107	1.85184	1.187307	1.350935	1.58352	0.010035	0.664533
1.565383	1.610733	1.84706	1.471193	0.576231	0.893465	1.245065	0.003569	1.270829
1.565383	0.284921	-0.01652	0.348734	0.392616	-0.10778	-0.16733	0.250471	0.559943
1.565383	-1.09888	-1.5224	-1.64445	-1.18632	-1.50253	-1.21314	0.018334	0.545574
1.565383	-0.51677	-0.71119	-0.62644	-0.39978	-0.53623	-0.41053	0.009853	0.311412
1.565383	0.598834	0.728055	0.545466	0.065471	0.455128	0.376533	0.02241	0.662584
-1.15437								
-1.15437	1.32455	1.610173	1.73955	1.084558	1.19196	1.328087	0.013923	0.654992
2.900943	1.540997	1.975967	2.08702	1.581118	1.59312	1.49159	0.008208	0.59543
2.900943	1.278155	1.437783	1.514993	1.371387	1.20205	1.414077	0.112695	0.312943
0.055633								
0.055633	-0.63402	-0.95976	-0.74489	-0.30757	-0.45937	-0.39032	0.000178	0.652186
0.055633	0.455555	-0.08234	-0.72617	0.539632	-0.59778	1.060573	0.003581	1.786741
-1.00456								
-1.00456	-0.00052	-0.1938	-0.50989	0.047978	-1.07086	-0.41277	0.046125	1.118834
-1.00456	0.034711	-0.7195	-1.0423	-0.63135	-0.71602	-0.20383	0.024956	1.077009

-0.74142	0.380464	0.59186	1.213126	0.561827	1.041108	0.787408	0.03549	0.832662
-0.74142	-0.09165	-0.48169	-0.21915	-0.07859	-0.45825	-0.62124	0.052418	0.542656
-1.19943								
-1.19943	1.73009	2.082497	2.308747	1.809047	1.293585	1.091833	0.005807	1.216914
-0.66762	3.776837	4.83187	2.8228	3.172043	3.205125	3.113877	0.030121	2.00907
-0.66762	-0.91153	-1.60396	-1.64815	-1.03106	-1.11261	-0.97766	0.013191	0.736617
-0.66762	-0.4777	-0.78151	-0.53332	-0.68727	-0.73711	-0.56342	0.082182	0.303802
0.394827	0.463579	0.490704	0.906731	0.49359	0.819248	0.638646	0.081139	0.443152
0.394827	-0.46517	-0.83769	-0.78325	-0.51611	-0.49623	-0.40531	0.051855	0.432378
0.394827	-0.69086	-1.35596	-1.51675	-0.82046	-1.13249	-0.99624	0.019129	0.82589
0.394827	-0.72864	-1.0016	-1.46904	-0.96958	-1.34688	-1.06386	0.013665	0.740404
0.394827	0.000333	-0.38986	-0.8521	-0.36526	-0.63755	-0.57512	0.004074	0.852431
0.394827	-0.96695	-1.07222	-0.95343	-0.74692	-1.10461	-0.94896	0.054946	0.357688
0.13263	-0.53658	-0.95741	-1.14098	-0.74928	-1.05665	-0.75652	0.003708	0.604401
0.13263	-0.60807	-1.23914	-1.23148	-0.71871	-1.24459	-0.85631	0.005246	0.636516
0.117313	1.131433	0.847777	0.736026	0.269278	0.63706	0.367234	0.01757	0.862155
0.117313	-0.1406	-0.67646	-1.15812	-0.54128	-0.67815	-0.67427	0.005605	1.017522
0.117313	-0.20224	-0.61845	-0.20123	0.110389	-0.39701	-0.45738	0.000816	0.728842
0.789205	1.035805	1.49869	1.672653	1.138394	1.73879	1.233771	0.006714	0.702985
1.45504	0.390893	0.835141	1.096931	0.602366	0.822943	0.727816	0.024766	0.706038
0.789205	1.94942	2.25351	2.080377	1.586983	1.98989	1.70531	0.096151	0.666527
1.45504	-0.65772	-1.48948	-1.55214	-0.55275	-1.21732	-0.92809	0.019628	0.999387
1.45504	-0.88439	-1.4381	-1.50451	-0.95737	-1.10725	-0.79022	0.01751	0.714289
-0.64546	0.744686	1.416587	1.572623	0.871137	1.57146	1.263017	0.005617	0.827937
0.255957	0.483446	0.697815	0.704119	0.485847	0.824897	0.678605	0.125136	0.341451
0.255957	0.458937	0.762295	1.343903	1.145331	1.1017	0.695183	0.012274	0.884966
-0.64546	0.697699	0.806229	1.005562	1.18072	0.881812	0.647172	0.013161	0.533548
-0.08923	-0.34074	-0.53674	-0.57949	-0.51216	-0.57994	-0.41736	0.048201	0.239193
-0.08923	0.11252	0.605477	0.675924	1.018911	0.965485	0.663921	0.000793	0.906392
-0.38738								
-0.35922								
-0.35922	0.084504	-0.20719	-0.15843	-0.06014	-0.09685	-0.11606	0.201008	0.291699
-0.35922	1.881837	2.466027	2.033865	1.767455	0.846641	1.454246	0.068298	1.619386
-0.38738	0.6961	1.026261	0.907549	0.54648	0.995499	0.959191	0.010551	0.479781
-0.38738	1.35594	1.755527	2.010787	1.434307	1.947925	1.442807	0.04461	0.654847
-0.37912	0.019819	-0.2665	-0.2444	-0.47677	-0.06985	-4.03E-05	0.016639	0.49659
-0.00777	-0.05692	0.134739	0.394396	-0.083	-0.03423	0.165521	0.057945	0.477398
-0.37912	-0.30425	-0.05681	0.131018	-0.33795	-0.07211	-0.18291	0.032707	0.468973
-0.00777	1.357797	1.857375	0.768364	0.609536	0.501316	1.161091	0.063228	1.356059
-0.09206	0.287083	0.37608	0.372864	0.389371	0.22815	0.182123	0.130835	0.207248
-0.09206	-0.48479	-0.60746	-0.68493	-0.60716	-0.31725	-0.31657	0.054191	0.36836
-0.09206	-0.85293	-1.32404	-1.40704	-0.73427	-1.31068	-0.96152	0.010176	0.672774
-0.09206	-0.66979	-0.83417	-0.97368	-0.63526	-0.98025	-0.70905	0.038734	0.344997
0.046947	-0.45026	-0.26282	0.379593	0.211891	-0.15788	-0.33118	0.003964	0.829848
0.046947	0.024373	0.550986	0.839078	0.485106	0.068529	-0.00239	0.00225	0.841466
0.046947								
0.046947	0.083765	0.236229	0.032398	0.332207	0.668645	0.403264	0.008283	0.636246

1.377837	0.583738	0.893121	0.950104	-0.02601	0.160566	0.669993	0.002012	0.976114
1.377837	0.373129	0.675442	0.635313	0.817186	1.2223	0.824585	0.002222	0.849171
0.25495								
0.25495	-0.72375	-1.14047	-1.28266	-0.76137	-1.02319	-0.75009	0.009875	0.558907
0.25495	-0.60339	-0.52964	-1.12097	-0.70181	-0.81267	-0.66504	0.045656	0.591327
0.13677								
0.13677	1.022176	-0.44276	-0.77166	-0.29031	-0.35246	-0.35385	0.113424	1.793833
0.13677	0.38618	0.365561	0.249181	0.136676	0.324384	0.408788	0.06554	0.272113
-0.17876	0.505908	0.881396	1.099026	0.322612	1.176948	1.272084	0.007193	0.949472
-0.17876								
-0.70209								
-0.70209	0.37082	0.713186	1.157263	0.338533	-0.06498	0.051872	0.025083	1.222246
-0.54463	0.153277	-0.19961	0.517369	0.587043	0.396563	0.621613	0.1075	0.821219
-0.37178	-0.70129	-0.4539	-1.42199	-0.95407	-0.61792	-1.04173	0.12761	0.968089
-0.37178	-1.22058	-1.74035	-1.48305	-0.81641	-1.6213	-0.99194	0.061963	0.923935
-0.37178	0.455635	0.796855	0.900096	0.996048	1.193735	0.873853	0.052237	0.7381
-0.54463	-0.24335	-0.43755	-0.50245	-0.38136	-0.16518	-0.34238	0.105655	0.337269
-0.2678	0.493821	0.551868	0.093675	0.253254	0.164779	0.126184	0.129722	0.458193
-0.66435	0.044722	0.159118	-0.10932	0.034378	-0.11781	-0.08569	0.112161	0.276926
-0.66435	-0.23456	-0.53515	-0.92234	-0.46275	-0.38173	-0.33168	0.006199	0.687778
-0.2678	0.687549	1.148067	1.350138	0.870806	0.731468	0.508617	0.021042	0.841521
-0.44265								
-0.44265	0.284904	0.55412	0.78316	0.455681	0.253036	0.241811	0.039896	0.541348
-0.48402								
-0.48402	0.280024	0.927188	1.38688	0.651355	0.957525	0.649196	0.000295	1.106856
-0.6189								
-0.6189	1.160584	0.830521	0.995007	1.157369	0.305928	-0.07441	0.071286	1.234993
1.743793	2.326363	2.297038	1.564447	1.93035	1.819142	1.506332	0.12611	0.820032
1.743793	-0.85086	-0.91754	-1.31234	-0.86962	-1.14173	-0.79877	0.004406	0.513578
1.743793	-0.98219	-1.24436	-1.48449	-1.27658	-1.07763	-1.03564	0.006926	0.502306
0.852047	0.650858	0.65268	0.905955	1.160452	1.367625	0.944279	0.055215	0.716767
2.648433								
2.648433	1.33901	1.313803	1.418018	1.448335	1.57	1.190103	0.020355	0.379897
2.648433	0.473261	0.445125	-0.68613	0.211722	-0.51201	0.26002	0.0633	1.159392
0.852047	1.126637	1.640193	1.71593	1.249754	1.29612	1.305157	0.042939	0.589293
-0.33332	0.655691	0.741051	0.811777	0.7655	0.590774	0.559133	0.06009	0.252643
-0.33332	0.6238	1.12975	1.338007	0.491369	0.751759	0.814985	0.002919	0.846638
0.269735								
0.269735	-0.65646	-1.45378	-1.69061	-0.45902	-1.04499	-0.67869	0.001127	1.231588
0.269735	-0.74705	-0.8853	-1.68276	-0.9129	-1.29966	-0.89665	0.000467	0.935714
0.269735	-0.86222	-1.30464	-1.82394	-1.13067	-1.39631	-1.25771	0.003886	0.961712
-0.2143	5.092457	5.369801	4.590955	4.265081	4.46069	3.803423	0.027072	1.566378
-0.2143	0.307012	0.128443	0.116167	-0.15134	0.235032	0.221676	0.243647	0.458348
-0.2143	-0.89766	-1.00187	-0.73129	-0.82899	-0.94008	-0.87082	0.007209	0.270578
-0.69967								
-0.69967	-0.52443	-0.8946	-0.72315	-0.31041	-0.22348	-0.27553	0.00536	0.67112
-0.69967	0.792186	1.26736	1.455243	1.279367	1.02786	0.809073	0.007373	0.663057

0.015352	0.90356	1.286699	1.5973	0.783002	1.28483	1.161769	0.049484	0.814298
0.015352	0.34036	-0.13048	-0.04445	0.320462	-0.18148	-0.11014	0.113006	0.521838
0.015352	0.807083	-0.54656	-0.24456	0.014708	0.166245	-0.85584	0.115204	1.662924
0.015352	-1.27982	-1.91359	-2.14401	-1.34202	-1.89122	-1.30486	0.002904	0.86419
0.015352	-0.99644	-1.47784	-1.57987	-1.22867	-1.38846	-1.18275	0.023137	0.583431
0.274193								
0.274193	-0.8902	-0.71118	-0.82964	-0.38093	-1.33302	-0.47532	0.15824	0.952087
0.274193	-0.74755	-1.35196	-1.58153	-0.83331	-1.31105	-0.85392	0.003135	0.833987
0.274193	-0.15018	0.135361	-0.48226	0.320151	-1.1848	-0.18667	0.11238	1.504949
0.274193	-1.11469	-1.75779	-1.89306	-1.15161	-1.42144	-1.13017	0.002153	0.778367
0.274193	-0.72427	-1.38549	-1.56984	-0.84999	-1.31093	-0.87195	0.003295	0.845564
0.274193	-1.16592	-1.65889	-1.99753	-1.15869	-1.47257	-1.56523	0.020125	0.838837
0.751437	0.030633	0.335538	0.737259	0.076553	0.897985	0.823272	0.017271	0.867352
0.751437	0.451567	0.179025	-0.0004	0.169017	0.029266	0.093888	0.079946	0.451967
0.751437	1.089414	1.62478	1.035709	0.878152	1.34773	1.465643	0.110438	0.746628
0.5019	0.142377	0.489858	1.022198	0.466789	0.466318	0.145487	0.019704	0.879821
0.258532	0.00213	0.141904	0.559512	0.254475	0.159236	-0.10262	0.019153	0.662128
0.258532	1.01304	0.167016	-0.60722	-0.16607	0.036026	-0.22608	0.04589	1.620261
0.258532	-0.05497	-0.19054	-0.0631	0.100924	-0.13392	-0.13299	0.050234	0.291466
0.5019	-1.12922	-1.05072	-1.13203	-0.76468	-1.33613	-1.1246	0.054327	0.571444
0.5019	0.956457	0.992873	-0.71275	-0.36977	0.559649	0.242603	0.118796	1.70562
0.856325								
0.856325	0.286842	-0.09771	-0.76916	-0.35811	-0.17996	0.146325	0.060007	1.056001
0.754107								
0.754107	0.321027	0.789706	0.279599	-0.03105	0.061323	0.377179	0.196252	0.820752
0.754107	0.265288	0.444076	0.172977	0.718936	0.357994	0.405489	0.005057	0.545958
-0.38568								
-0.38568	-0.26033	-0.57655	-0.43933	-0.47318	-0.76105	-0.63351	0.025346	0.500726
-0.38568	1.51587	1.305	1.26581	0.872288	0.658315	0.962135	0.030529	0.857556
-0.88323	0.646462	0.81908	0.934245	0.701045	0.659501	0.389079	0.109421	0.545166
-0.43054	0.214812	0.671661	1.051185	0.551254	0.12861	0.004753	0.000173	1.046432
-0.83722								
-0.88323	0.7187	0.588972	0.772315	0.476839	0.539322	0.248358	0.077354	0.523957
-0.83722	1.47119	1.65932	1.57213	1.249413	0.923225	0.926193	0.001094	0.736096
-0.43054	0.152124	0.463133	0.785769	0.534533	0.731747	0.158022	0.083445	0.633645
-0.43054	2.33391	1.901593	1.43645	1.447877	1.55833	1.332526	0.01945	1.001384
-1.00272	0.426586	1.028669	1.611607	1.005519	0.989136	0.708652	0.001298	1.185021
-1.00272	-0.04474	0.057596	-0.09892	-0.28553	-0.50419	-0.20326	0.02167	0.561786
-1.00272	-0.2096	-0.07246	0.159052	0.052407	-0.35534	-0.31117	0.015891	0.514394
-0.57845								
-0.51978								
-0.51978	0.811492	0.788129	0.477529	0.104306	0.227525	0.513111	0.001016	0.707186
-0.51978	0.247119	0.145009	0.293985	0.304713	0.64279	0.419783	0.017201	0.497781
-0.57845	0.613781	1.05482	1.377387	1.21146	0.797628	0.465127	0.022064	0.91226
-0.45628								
-0.45628	1.57223	1.98933	1.886593	1.754063	1.83144	1.427057	0.020089	0.562273
	0.258688	0.350562	0.521187	0.012786	0.231942	0.1034	0.020438	0.508402

2.464767	0.130097	-0.01865	-0.05008	-0.32095	-0.01411	0.379741	0.002203	0.700689
2.464767	-0.15632	-0.21698	-0.36994	-0.553	-0.11643	-0.09124	0.003159	0.461756
2.464767	-1.01328	-1.48652	-1.47817	-1.04802	-1.25158	-1.01922	0.026404	0.473241
-0.49918	3.09552	2.845677	2.785317	2.80928	2.35392	2.11979	0.038761	0.97573
-0.49918								
-0.78846	4.527347	4.402117	3.856023	3.67297	3.81455	3.40982	0.015173	1.117527
-0.49918	-0.04525	0.047994	0.449452	-0.03749	0.359308	0.375755	0.012221	0.494706
-0.78846	-0.97474	-1.41446	-1.48667	-1.10747	-1.19052	-1.0339	0.045317	0.511932
-0.78846	-0.52505	-0.45589	0.020029	-0.12831	-0.29803	-0.60396	0.004122	0.623989
0.165107								
-0.19065	0.021905	-0.30092	-0.66352	-0.42563	-0.36084	-0.21047	0.0076	0.685422
-0.19065	-0.15164	0.068547	0.412133	-0.01087	0.414285	0.070991	0.016001	0.565928
0.165107	1.170954	1.768503	1.863313	1.210513	1.62563	1.276673	0.007359	0.692359
-0.58845								
-0.58845	0.323721	-0.4122	0.519691	-0.40123	-0.2295	-0.42123	0.24497	0.940924
-0.58845	-0.68176	-1.07732	-1.56319	-0.91012	-1.2451	-1.09765	0.012355	0.881435
-0.58845	-0.76798	-1.09463	-1.24031	-0.87958	-1.14027	-0.96253	0.013937	0.47233
1.455233								
1.455233	1.559533	1.727837	1.52832	0.852939	1.318015	1.203087	0.000455	0.874898
-0.14567	0.267694	0.217197	-0.11233	-0.18406	0.278204	0.120662	0.045154	0.462259
	1.465593	1.85924	2.237214	2.265627	0.833528	1.571211	0.132246	1.432099
	-0.03601	-0.01428	-0.47905	-0.51326	-0.32093	-0.72458	0.063214	0.710308
-0.14567	-0.24237	-0.63009	-1.04724	-1.04015	-0.66981	-0.5552	0.024851	0.804873
-0.9737								
-0.9737	0.68914	1.100435	1.221137	0.773504	0.537737	0.537112	0.008137	0.684025
-0.58877								
-0.58877	-0.32739	-0.16969	0.089096	-0.23746	-0.08179	-0.17906	0.002511	0.416481
-0.96478	0.031172	0.384195	0.66069	0.314443	-0.12335	-0.17769	0.013624	0.838381
-0.78134	1.431338	1.818597	1.870847	1.35132	0.791128	0.973686	0.037803	1.079719
-0.78134	0.062722	0.203628	0.598685	0.295791	0.16768	0.18036	0.016898	0.535963
-0.96478	0.582344	1.008392	1.365623	0.862798	0.940702	0.615216	0.000704	0.783279
0.58666								
0.58666	0.528117	0.705191	0.877637	0.731257	0.75292	0.531079	0.053063	0.34952
-0.36481	-0.37604	-0.37481	-0.50235	-0.53228	0.038255	-0.08454	0.054607	0.570534
-0.63203	-0.65647	-0.83602	-0.96378	-1.03081	-1.04981	-1.03091	0.107731	0.393341
-0.63203	-0.44623	-0.59372	-0.54812	-0.74027	0.174257	-0.22696	0.005844	0.914526
0.057543	0.672314	0.925259	0.947601	0.472954	0.693886	0.675961	0.018679	0.474647
-0.63203								
-0.36481	1.05686	1.374757	1.67964	0.738955	1.32029	1.261643	0.001395	0.940685
0.057543	-0.58803	-0.28452	-0.17439	-0.25622	-0.06776	-0.35742	0.01736	0.520269
0.057543	0.655622	0.910216	1.334323	0.905221	1.4253	1.165897	0.001688	0.769678
-0.29294								
-0.29294	1.802513	1.307223	2.101333	2.240587	1.53783	2.256657	0.198498	0.949433
0.582203	1.40033	1.824997	1.869077	1.299167	1.65777	1.407747	0.033874	0.56991
0.582203	0.468795	0.439694	0.426179	0.304915	0.192097	0.117235	0.032096	0.35156
-0.75221								
-0.75221	-0.02061	0.34276	0.186327	-0.09118	0.431422	0.244848	0.026684	0.522601

-0.75221	-0.5503	-0.54201	-0.69453	-0.58832	-0.58218	-0.47997	0.133422	0.214558
-0.5292								
-0.5292	-0.76643	-0.78436	-0.42132	-0.27693	-0.4794	-0.55645	0.000375	0.507427
-0.5292	0.691497	1.27953	1.699687	1.04725	1.11455	0.926938	0.003095	1.00819
-0.29606	0.534188	0.985557	1.366227	0.509945	0.794342	0.696541	0.000325	0.856282
-0.29606	0.931223	1.398085	1.520182	1.111437	1.85318	1.498603	0.038246	0.921957
-0.29606	-0.15911	-0.67503	-0.56309	-0.05395	-0.21883	-0.16375	0.071149	0.62108
0.433877								
0.763733								
0.763733	0.288213	0.053555	-0.00541	0.15181	0.289608	0.131553	0.1215	0.295017
0.433877								
0.433877	-0.4612	-1.08438	-1.31039	-1.00021	-1.16082	-1.32397	0.006723	0.862774
0.6088	0.935681	1.446843	1.321217	0.667752	0.95058	1.020153	0.034756	0.779092
0.6088	0.227519	0.187483	0.135944	-0.02061	0.005186	0.149387	0.13137	0.248125
0.6088	0.478826	0.028407	0.078865	0.576915	0.770854	0.422203	0.04889	0.742447
2.535267	0.867286	1.039689	1.080716	0.460409	0.594388	0.755748	0.048372	0.620307
2.535267	-0.83447	-0.90303	-0.71228	-1.05299	-1.18153	-0.8498	0.01509	0.469249
0.847147								
0.847147	-0.27534	-0.40635	-0.60686	-0.60649	-0.53368	-0.32535	0.024124	0.331521
-1.31703								
-1.31703	0.632674	0.754146	1.074971	1.153025	0.242116	0.217325	0.071514	0.9357
-0.72411	0.346042	0.234377	0.611351	0.57105	0.787475	0.680519	0.054728	0.553097
-0.72411	-0.74619	-0.69301	0.017792	0.161072	-0.53034	-0.62648	0.000504	0.907266
-0.72411	-0.32267	-0.24291	0.546716	0.440915	-0.11175	-0.33715	0.00715	0.883867
-0.89619	0.041086	0.207855	0.220758	0.087927	0.087982	0.152188	0.068304	0.179672
-0.89619	-0.45211	-0.79728	-0.88817	-0.62705	-0.67169	-0.62418	0.033538	0.436065
-0.89619	0.19496	0.72641	0.867446	0.665763	0.196144	0.086242	0.027337	0.781204
-0.20592	0.140412	0.394011	0.84296	0.931344	0.565647	0.276106	0.003631	0.790932
0.149757	1.184642	1.57116	1.550033	0.904584	1.94238	1.727903	0.010007	1.037796
0.149757	-0.01005	-0.2807	-0.20685	0.298953	0.097043	-0.04665	0.019827	0.579654
0.149757	-0.47803	-0.93659	-1.1416	-0.01709	0.502876	-0.85576	0.014572	1.644476
0.149757	-0.73402	-0.98354	-1.02479	-0.7017	-0.85309	-0.7045	0.061407	0.323092
-0.20592	0.861721	1.663877	1.610717	1.505379	1.365405	1.002053	0.124962	0.802156
-0.09532								
-0.09532	-0.25287	-0.01806	0.020322	-0.06405	-0.23914	-0.33961	0.072078	0.359929
-0.09532	1.040673	0.489307	0.478762	0.246733	0.165465	0.23722	0.039006	0.875208
0.431027								
0.431027	1.867748	0.494792	1.451159	1.544704	-0.35029	0.921379	0.077979	2.218042
0.431027	-0.86202	-1.08294	-0.89978	-0.70958	-0.50911	-0.89793	0.241678	0.573834
-0.52499	1.019454	1.136698	1.615503	0.911132	1.916275	1.704473	0.061092	1.005143
-0.52499	-0.53541	-1.40054	-1.55062	-1.08511	-1.34356	-0.79257	0.04183	1.01521
-0.52499	0.291251	0.562872	1.30692	0.904566	0.653447	0.366315	0.000552	1.015669
0.886047								
0.886047	-0.16385	-0.41911	-0.46411	-0.56821	-0.39765	-0.35675	0.013911	0.404362
0.886047	-1.20852	-1.60056	-1.37205	-1.11608	-1.23504	-1.22268	0.02525	0.484488
-0.61719	-0.52654	-0.63813	-0.08468	-0.1641	-0.6089	-0.51952	0.007955	0.553448
-0.61719								

-0.61719	0.441338	0.617185	0.81008	0.811683	0.785226	0.455725	0.007016	0.370344
1.006723	0.645005	0.668437	0.854556	0.358807	0.522199	0.634313	0.120516	0.495749
1.006723	-0.27411	-0.091	-0.15321	-0.07395	-0.13166	-0.10576	0.117112	0.20016
1.006723	0.34521	0.734876	0.900027	0.684034	1.168816	0.758174	0.03097	0.823606
0.073817	0.64263	0.683738	0.787971	0.378815	0.677065	0.810542	0.037987	0.431727
0.073817	-1.23492	-1.37673	-1.14413	-0.72945	-1.07547	-0.95373	0.042354	0.647282
0.073817	-0.63483	-1.23732	-1.42027	-0.80518	-1.10737	-0.78078	0.000562	0.785436
0.073817	0.649846	0.964362	1.445257	0.990202	1.37635	1.04594	0.015397	0.795411
-0.54373								
0.519023								
0.519023	-0.07676	-0.08306	-0.18565	-0.51004	0.058944	-0.04529	0.009498	0.568984
0.519023	-1.5906	-2.03678	-2.26198	-1.54434	-1.96531	-1.55173	0.026455	0.717643
0.519023	-0.81223	-1.47675	-1.55736	-0.87485	-1.17467	-0.99566	0.005859	0.745127
0.519023	0.718022	0.931225	1.088947	0.982319	1.593755	1.088917	0.043996	0.875733
-0.54373	4.380873	3.765783	3.243579	3.227767	3.064005	3.600623	0.066798	1.316868
2.096603								
2.096603	-0.02828	-0.00601	0.42892	0.452743	0.586978	0.290987	0.003547	0.615262
2.096603	0.472745	0.585374	0.722658	0.738602	0.997041	0.638643	0.057125	0.524296
-0.29456								
-0.29456	-0.3807	-0.50721	-0.36337	-0.25819	-0.37427	-0.38344	0.029774	0.249023
-0.29456	-0.47387	-0.52737	-0.88068	-0.19327	-0.55085	-0.53488	0.163654	0.687405
	0.148461	0.334549	0.412951	0.23073	0.254394	0.058468	0.042326	0.354482
3.490152								
3.490152	-0.79049	-1.30523	-1.58708	-1.06754	-1.3968	-1.30036	0.017615	0.79659
3.490152	-1.07164	-1.25276	-1.50227	-1.09677	-1.45292	-1.24284	0.024547	0.430627
3.490152	-0.60579	-0.87017	-0.92645	-0.69608	-0.58248	-0.43303	0.004409	0.493414
-0.56192								
-0.56192	-0.92578	-1.48608	-1.41058	-0.91556	-1.32371	-1.22875	0.074241	0.570517
-0.56192	-0.72341	-1.45641	-1.82465	-0.989	-1.4585	-1.09616	0.012225	1.101237
0.582457	0.640757	0.480228	0.675034	0.597934	1.031175	0.732599	0.019279	0.550947
0.582457	-0.88135	-1.81243	-1.81807	-1.32575	-1.54308	-1.27365	0.014656	0.936714
0.582457	-0.56346	-1.16629	-1.21481	-0.45392	-0.57704	-0.49269	0.024756	0.760889
0.582457	-0.19262	-0.36844	-0.88054	-0.60426	-0.66596	-0.61794	0.031535	0.687923
0.582457	-1.25139	-1.76483	-1.47115	-0.93308	-1.58002	-1.2367	0.015865	0.831756
-0.93805	0.057933	0.064667	-0.04528	0.018251	0.345745	0.057051	0.005241	0.391024
-0.93805	2.137257	2.540503	2.38159	2.602413	2.806795	1.85983	0.197241	0.946965
-0.69815	0.522121	0.744785	1.430573	1.182096	0.926469	0.737996	0.007994	0.908452
-0.69815								
-0.69815	0.918271	1.33238	1.69015	1.27811	1.15581	0.981538	0.003417	0.771879
0.441657								
0.441657	-0.86786	-1.35204	-1.46841	-0.7633	-1.1341	-0.83823	0.001537	0.705108
0.441657	-0.70392	-1.20697	-1.36221	-0.7036	-1.18918	-0.82942	0.000633	0.658613
0.441657	0.595961	1.774955	-0.10007	1.614097	-0.36446	0.26755	0.087254	2.139419
0.441657	0.239749	0.669862	1.086957	0.057692	0.373747	0.305335	0.000941	1.029265
-1.15772								
-1.15772	-0.54592	-0.713	-0.40123	0.359843	-0.05442	-0.28224	0.000254	1.072846

0.109727	0.567238	0.723008	0.35401	0.110962	0.446148	0.582895	0.202331	0.612046
0.109727	-0.27991	-0.0362	0.079577	-0.18206	-0.13218	-0.21333	0.052424	0.359486
2.165077	0.589519	0.626413	1.045715	0.777808	0.650443	0.476382	0.072733	0.569333
0.109727	-1.25251	-1.50471	-1.84393	-1.30201	-1.6329	-1.34855	0.008152	0.59142
2.165077	-0.68624	-1.06295	-1.04272	-0.98546	-1.12729	-0.90976	0.050129	0.441046
2.165077	-1.29191	-1.80131	-1.90965	-1.12648	-1.55167	-1.21393	0.002079	0.783172
2.165077	-1.05422	-1.55096	-1.70881	-0.89299	-0.83207	-0.79962	0.00153	0.909188
2.165077	-0.65085	-1.05629	-1.17554	-0.64793	-0.7187	-0.61946	0.01906	0.556082
0.095713	0.737292	0.853962	1.051169	0.442571	0.781169	0.918722	0.007726	0.608597
0.095713	0.80351	0.73508	0.750097	0.761461	0.980739	0.962759	0.093489	0.245659
-0.2157	0.240939	0.371979	0.813098	0.641257	0.406172	0.192615	0.109317	0.620483
-0.2157	0.593665	0.942056	1.224616	0.705712	0.663521	0.499779	0.001258	0.724837
-0.71684	1.041322	1.138184	1.386187	0.797437	1.075955	0.875897	0.056112	0.58875
-1.17282	0.852536	0.887324	1.31497	0.855161	0.963018	0.856027	0.006546	0.462434
-1.17282	-0.47036	-1.14664	-0.7444	-0.27027	-0.54361	-0.50124	0.036265	0.876374
-1.17282	-0.21493	-0.22507	0.07628	0.495985	0.18529	0.080907	0.00802	0.721057
-0.71684	-0.12264	-0.18042	-0.26396	0.198947	0.275239	0.054617	0.021673	0.539201
-0.71684	-0.41537	-0.27856	0.010921	-0.10064	-0.54621	-0.42516	0.002086	0.557131
0.40063	0.06349	-0.0145	0.244429	0.066938	0.327207	0.476135	0.061426	0.490631
0.40063	-0.88212	-0.69276	-0.27711	-0.38244	-0.78281	-0.81209	0.021039	0.605012
0.40063	0.134291	0.096479	0.54984	0.486422	0.290898	0.229483	0.014995	0.453362
-0.09482	0.445046	0.523118	0.938354	1.120959	0.812955	0.711153	0.018599	0.675914
-0.09482	-0.39717	0.137612	0.898124	0.403388	-0.03113	-0.29185	0.008546	1.295297
-0.09482	0.645385	0.792747	1.049734	1.344523	1.02175	1.066522	0.024967	0.699138
-0.76141								
-0.76141	-0.44803	-0.73019	-0.36779	0.040014	-0.42023	-0.41681	0.013564	0.770209
-0.76141	0.481566	0.559701	1.056328	0.85432	0.58716	0.463941	0.005878	0.592387
0.362333	0.29872	0.377986	0.660944	0.148295	0.575553	0.568484	0.085218	0.512649
0.362333	-0.37276	-0.92993	-0.78597	-0.66366	-0.81829	-0.85824	0.014792	0.557169
-1.00025								
-1.00025	0.405843	0.099481	0.168443	0.173808	0.270598	0.216857	0.04773	0.306362
-1.00025	0.374725	0.684007	0.757153	0.633637	-0.15567	0.073962	0.036937	0.91282
2.085457								
2.085457	-0.96804	-1.08821	-1.01921	-0.69563	-0.69872	-0.66605	0.009359	0.422155
2.085457	0.154179	0.14692	0.409239	0.155639	0.331008	0.423485	0.055968	0.276565
	0.535626	0.603889	0.612757	0.25747	0.663634	0.746701	0.000445	0.489231
-0.37525								
-0.37525	-1.01831	-1.25878	-1.24522	-1.00941	-1.20965	-1.07263	0.19372	0.24937
-0.37525	0.770364	1.325397	1.58316	0.836156	1.049645	0.961493	0.002124	0.812796
-0.72997								
-0.72997	-0.54914	-0.67141	-0.78789	-0.3315	-0.53218	-0.38461	0.050329	0.456391
-0.91204								
-0.91204	-0.75877	-1.03198	-0.56309	-0.2538	-0.84627	-0.79482	0.000356	0.778181
-0.91204	0.369	0.355754	0.4178	-0.17207	-0.57476	-0.44556	0.023089	0.992557
-0.32655	-0.92387	-0.99423	-1.23143	-0.38101	-0.88662	-0.76547	0.00294	0.850421
-0.32655	-0.00512	-0.31401	-0.31508	0.172509	-0.42523	-0.37751	0.018375	0.59774

-0.32655								
0.494687								
0.494687	-0.27699	-0.26999	-0.42323	-0.2237	-0.34489	-0.29394	0.113463	0.199531
0.494687	-1.1348	-1.46604	-1.55645	-1.00449	-1.4495	-1.06907	0.012349	0.551953
2.219482								
2.219482	-0.01221	0.506097	1.144993	0.632363	0.123281	-0.02062	0.001482	1.165617
0.022068								
0.022068	1.11321	1.399427	1.68494	1.322493	1.38987	1.272027	0.001373	0.57173
-0.13535								
-0.13535	-0.67966	-1.06702	-0.89039	-0.62251	-1.01766	-0.8654	0.065458	0.444519
-0.13535	-1.00849	-1.51228	-1.72918	-1.02534	-1.41225	-1.13004	0.042019	0.720692
2.130047	0.57007	0.578809	0.68774	-0.10543	-0.30769	0.368884	0.004038	0.995428
2.130047	1.420606	1.367397	1.075198	0.513565	1.290265	1.364249	0.020748	0.907041
0.860967	1.385043	1.743517	1.709123	1.020758	1.572995	1.408733	0.032205	0.722758
0.860967	0.198186	-0.09408	-0.23195	-0.08804	0.779075	0.047289	0.10044	1.011028
0.860967	3.872637	4.536267	2.902473	3.181483	3.113535	3.157107	0.111467	1.633793
0.781427	-1.18504	-1.72411	-1.68544	-0.92871	-1.19693	-0.9827	0.005608	0.795397
0.781427	0.388189	0.532865	0.599647	0.621326	0.296659	0.494728	0.1005	0.324668
0.781427	0.598366	1.029831	1.042508	0.353362	0.839614	0.900695	0.04368	0.689146
-0.29984	0.735977	0.507468	0.571901	0.287809	0.437642	0.68906	0.051948	0.448168
-0.29984	1.54141	1.707883	1.817737	0.926066	1.388065	1.37347	0.000659	0.891671
2.55988	0.445492	0.776884	1.282397	0.55253	0.506329	0.412049	0.005464	0.870348
2.55988	0.883451	1.343217	1.786737	1.057197	0.797838	0.835477	0.000951	0.988899
-0.86727								
-0.86727	0.92105	1.035908	1.488483	1.059718	1.34392	1.239367	0.000394	0.567433
-0.86727	0.230682	0.77215	0.988188	0.64983	0.529469	0.153636	0.026275	0.834552
0.290537								
0.290537	-0.38473	-0.63714	-1.00504	-1.00313	-0.65005	-0.50194	0.034933	0.620312
-0.16059								
-0.3062								
-0.3062	-0.82747	-1.29638	-1.32895	-1.06063	-1.15066	-1.03438	0.028596	0.501478
-0.3062	-0.78177	-1.00379	-1.12246	-0.67011	-1.2464	-0.95819	0.007606	0.576289
-0.16059	-0.4416	-0.54993	-0.31872	-0.35114	-0.17033	-0.34687	0.020653	0.379596
-0.16059	0.219731	0.234666	0.445342	0.425376	0.493285	0.298099	0.109843	0.273554
0.00218	-0.91108	-1.42401	-1.02294	-0.64151	-0.86843	-0.78222	0.012004	0.782503
-0.88091	1.802715	1.91632	1.544523	1.659154	1.727475	1.119839	0.052788	0.796481
-0.88091								
-0.88091	-0.4512	-0.04601	-0.02851	0.234633	0.00093	-0.2469	0.006438	0.685834
0.00218	-0.89993	-1.2086	-1.33415	-1.17726	-1.05388	-0.94508	0.02777	0.434221
1.274177	-0.61474	-1.4321	-1.66586	-0.63969	-1.28226	-0.97462	0.001162	1.051121
1.274177	0.402428	0.177438	0.06933	0.237138	0.004252	0.049155	0.040259	0.398177
1.274177	-0.27879	-0.21622	-0.89355	-0.62359	-0.42312	-0.36571	0.054841	0.677329
1.274177								
1.274177	0.861518	0.541808	0.302577	1.22119	-0.0111	0.486579	0.157073	1.232292
1.274177	1.537384	0.000992	-0.35765	0.217648	-0.00807	-0.17492	0.107247	1.895036
0.133203	0.644662	0.8695	1.02907	0.517209	1.0432	0.764772	0.121465	0.525991
0.133203								

0.251112								
0.251112	1.680795	0.378808	0.364988	0.21349	0.234986	-0.00753	0.077042	1.688323
0.251112	-0.72934	-1.20323	-0.8699	-0.53781	-1.23123	-0.58542	0.120662	0.693418
0.251112	-0.22814	-0.50962	-0.16029	-0.45972	-0.08671	-0.36647	0.052014	0.422916
-0.24396	0.431435	0.703266	0.512604	0.252197	0.49902	0.530856	0.04584	0.451068
-0.24396	-0.55448	-0.4486	-1.052	-0.57082	-0.9459	-0.78831	0.121812	0.6034
-0.24396	-0.01853	0.478067	0.860489	0.26639	0.766852	0.289757	0.019315	0.87902
-0.21143								
-0.21143	0.526664	0.809966	0.703241	0.101469	0.869994	0.642705	0.007235	0.768525
-0.21143	-0.97496	-1.06804	-0.86967	-0.41711	-0.76291	-0.97156	0.055097	0.650927
0.125413	0.835657	0.876988	1.008137	0.442765	0.907177	0.851212	0.030117	0.565372
0.125413	-0.47527	-0.73587	-0.24857	-0.55077	-0.30661	-0.24428	0.123471	0.491592
0.125413	-0.63712	-0.83272	-0.58266	-0.39544	-0.33778	-0.41048	0.045256	0.494945
0.249403								
0.249403	-0.34614	-0.70514	-0.28661	-0.00104	-0.32426	-0.47738	2.43E-05	0.704098
0.249403	-0.88772	-1.63081	-1.73874	-0.85928	-1.4004	-1.28897	0.040013	0.879467
0.249403	-0.90799	-1.60316	-1.59238	-0.96882	-1.30633	-1.0821	0.01421	0.695169
0.249403	-0.37926	-0.61132	-1.50751	-0.4385	-1.33676	-0.76236	0.057762	1.12825
0.3386								
1.944942	-0.69189	-1.0579	-0.93104	-0.35838	-0.27468	-0.66019	0.004005	0.783222
1.944942	-0.88861	-1.25447	-1.59962	-1.11237	-1.18338	-0.94832	0.006305	0.711017
1.944942	0.836446	1.218375	1.166853	0.484503	1.19615	0.944135	0.022718	0.733872
1.944942	0.608261	0.405945	0.5119	0.144277	-0.12312	0.531067	0.106514	0.731385
0.3386	-0.21424	-0.7259	-0.84154	-0.63411	-0.52228	-0.17972	0.038534	0.661818
0.700735	-0.46036	-0.37046	-0.89042	-0.82538	-1.19189	-0.8467	0.022324	0.821423
0.700735	-0.98247	-1.3501	-1.32554	-1.11117	-1.25749	-1.11657	0.010011	0.367624
0.700735	-1.13924	-1.68855	-1.7449	-1.20065	-1.44224	-1.27599	0.019077	0.605663
0.700735	-1.00613	-1.77832	-1.86192	-1.14518	-1.61795	-1.27814	0.002511	0.855791
-1.08182								
-1.08182	1.022817	1.300077	1.370257	1.030309	0.998007	1.027658	0.085372	0.37225
0.789183	0.726238	0.91574	0.979047	0.712551	1.087121	1.094678	0.095032	0.382127
0.789183								
0.789183	-0.86923	-1.18101	-1.28148	-0.83364	-1.64636	-0.8918	0.19926	0.812724
1.624427	0.647167	1.003363	1.018934	0.383821	0.902335	0.943963	0.096051	0.635112
1.624427	-0.81268	-1.21326	-1.42734	-1.05359	-1.38439	-0.99294	0.02587	0.614663
1.624427	0.051212	-0.46277	-0.53871	-0.13845	-0.02563	-0.23098	0.145198	0.589926
0.89932	-0.21142	-0.27098	-0.50681	-0.07565	-0.34806	0.075218	0.190185	0.582032
0.89932	-0.08357	-0.60595	-0.53933	0.140149	0.005585	0.04247	0.011829	0.746102
	1.923677	2.145838	1.59399	1.825783	1.863685	1.753817	0.097154	0.551848
0.97362	-1.26575	-1.39596	-1.18304	-0.96754	-0.60669	-0.80049	0.011477	0.789263
0.89932	0.829595	1.419353	1.463597	0.898941	1.87571	1.447397	0.030764	1.046115
0.97362	0.220157	0.448699	0.582074	0.208929	0.917472	0.646121	0.014988	0.708543
0.97362	1.017323	1.417977	1.066361	0.794896	1.15193	0.927246	0.012879	0.62308
2.315647								
2.315647	1.226933	1.445947	1.389367	0.686466	1.380655	1.495853	0.003791	0.809388
0.304273	0.479407	0.950649	1.034809	0.525065	0.734359	0.679704	0.016053	0.555401
0.304273	0.744567	0.917836	0.696124	0.658488	0.858969	1.050164	0.001083	0.391677

1.048943	0.787792	0.810657	0.85493	0.618139	1.07577	1.005954	0.021268	0.457631
0.619273	0.012575	-0.36695	-0.86674	-0.5365	-0.30589	-0.36467	0.006294	0.879316
0.619273	0.849119	0.962209	1.356955	0.98949	1.65682	1.508157	0.001515	0.807701
0.619273	0.056802	0.078587	-0.18784	-0.23528	0.066093	0.206852	0.018294	0.442136
1.048943	-0.87968	-1.5167	-1.79184	-1.03952	-1.46644	-1.26817	0.001048	0.912157
1.048943	-0.5956	-1.1016	-1.24907	-0.70114	-0.69615	-0.6241	0.007827	0.653466
-0.56445	0.758824	1.352583	1.71262	1.063878	0.825559	0.828873	0.002793	0.953796
-1.08066	-0.87975	-0.67951	-0.12589	0.111424	-0.42293	-0.56706	0.001765	0.991172
-1.08066	0.079687	0.641578	1.42915	1.23066	0.576306	0.212501	0.009889	1.349463
-0.57991	-1.01606	-1.32651	-1.64508	-1.03971	-1.27587	-1.00409	0.027309	0.64099
-1.08066	2.273447	1.95386	1.005697	1.077483	2.02566	1.50129	0.016878	1.26775
-0.56445	-1.02559	-1.30665	-1.54453	-0.82221	-1.30276	-0.62158	0.052064	0.922957
-0.56445	0.358552	0.444617	0.104152	-0.12415	1.096465	0.830693	0.002039	1.220612
-0.57991	0.246616	0.916835	1.218647	0.428797	0.599103	0.345162	0.000786	0.972031
-0.57991	-0.54177	-0.60489	-1.0645	-0.78759	-0.98796	-0.76975	0.017981	0.522731
-0.57991	2.22175	1.617673	0.890283	1.31565	1.596268	1.18744	0.022604	1.331467
-0.57991	-0.68738	-0.08928	-0.78364	-0.78656	-0.35522	-0.94629	0.151056	0.857016
-0.12202	0.412083	0.714778	0.996727	0.366475	0.723262	0.677867	0.011197	0.630253
-0.63478								
-0.63478	0.71925	0.07391	-0.02716	0.249224	0.41806	-0.03382	0.129546	0.75307
-0.63478	-0.24703	-0.14962	0.098495	-0.12943	-0.47964	-0.43707	0.000372	0.578134
0.323337								
0.323337	0.07778	0.18545	0.437734	0.357187	0.63028	0.281655	0.01506	0.5525
-0.0175	0.631805	0.787566	0.343368	0.936079	1.333768	1.115523	0.020624	0.990401
-0.127	0.805247	0.882004	0.752387	0.894501	1.587755	1.23546	0.000117	0.835368
-0.0175	0.520214	0.57738	0.554337	0.304149	0.825629	0.593649	0.046113	0.52148
-0.127	0.507435	0.672917	1.173819	0.623317	1.07287	0.831649	0.024493	0.666385
-0.127	-0.9836	-1.07431	-1.48171	-1.09293	-1.19695	-1.0912	0.011469	0.498111
-0.127	1.470473	2.308723	2.255657	1.903547	1.771685	1.881057	0.071296	0.83825
0.707867	0.656458	0.73202	0.604513	0.648432	0.886595	0.653897	0.140802	0.282081
0.707867	0.194674	0.537951	0.566913	0.062223	-0.12657	0.052427	0.015618	0.693481
0.707867	0.14127	-0.19644	0.036835	0.527512	0.83088	0.449883	0.004441	1.027317
0.4165	-0.07407	0.054954	0.077218	-0.12149	-0.26615	-0.1313	0.239615	0.343369
-0.52647	0.41113	0.568485	0.656181	0.358697	0.749682	0.630507	0.029722	0.390985
0.4165	0.903216	1.56033	1.680427	1.150851	0.944742	0.709926	0.00348	0.970501
-0.52647	-0.00802	-0.43659	-0.80095	-0.52632	-0.66051	-0.55479	0.054727	0.792928
-0.52647	0.533635	1.229732	1.858813	1.064623	0.707395	0.562214	0.000776	1.325178
-0.71151	-0.8252	0.16283	-0.59342	-0.08127	-1.06386	-0.57019	0.036106	1.22669
-0.71151	1.153578	1.712213	2.039663	1.150294	1.617085	1.27346	0.013563	0.889369
-0.61836	2.369799	2.03915	1.43707	1.507984	1.524345	1.138799	0.033202	1.231
-0.71151								
-0.61836	-0.00518	0.589312	0.065789	-0.01317	-0.0278	-0.02335	0.238573	0.617116
1.794882	-1.26169	-1.56002	-1.41169	-1.37769	-1.95039	-1.85214	0.01407	0.688699
1.83065	-0.66681	-1.32364	-1.16369	-0.69553	-1.1618	-1.10486	0.087156	0.656826
1.83065	-0.96988	-1.49466	-1.65163	-1.09612	-0.94535	-0.98274	0.0305	0.706276
1.83065	1.675347	1.325203	0.736003	0.911907	0.976355	0.781913	0.038976	0.939343
1.794882	-0.25349	-0.18735	-0.28645	-0.31715	0.205962	-0.0903	0.129201	0.523115

1.794882	-0.2781	-0.80452	-0.896	-0.47671	-0.51887	-0.45908	0.064766	0.617905
1.794882	-1.28186	-1.76769	-1.8753	-0.98548	-1.27487	-1.22574	0.003679	0.889814
1.794882	-0.83945	-1.41462	-1.29463	-0.81791	-1.32649	-0.9291	0.111389	0.596703
1.794882	-0.80991	-1.03012	-1.65852	-1.00339	-1.06311	-0.9826	0.006207	0.84861
1.794882	-0.56564	0.291138	-0.85888	-0.26929	-1.05943	-0.51023	0.063877	1.350569
1.794882	-0.5821	-0.63291	-0.65467	-0.77883	-0.48878	-0.44635	0.052989	0.332478
1.83065	0.892043	1.202263	1.018387	0.483767	0.731435	0.79819	0.043309	0.718496
1.83065	-0.69158	-1.287	-1.26742	-0.61061	-0.87065	-0.60283	0.002152	0.684172
1.83065	-0.74459	-1.35407	-1.60422	-0.95269	-1.28295	-0.99675	0.002952	0.859634
1.83065	-0.32737	-0.68011	-0.65168	-0.11787	-0.40013	-0.29904	0.06507	0.562239
0.930063								
0.930063	-0.5948	-1.11097	-1.07132	-0.46563	-0.5824	-0.4482	0.041031	0.66277
0.930063	-1.1758	-1.63173	-1.87981	-0.96743	-1.31348	-0.95038	0.003858	0.929424
0.930063	-0.44583	-1.06813	-1.2573	-0.45691	-0.50637	-0.59314	0.008828	0.811479
0.930063	-0.78952	-1.27898	-1.31938	-0.68309	-0.95969	-0.75403	0.019681	0.636285
-0.59898	0.835608	1.331091	1.547733	0.523374	1.037648	1.003052	0.006591	1.024359
-0.59898	0.835489	0.889612	1.029463	0.309574	0.967861	0.81999	0.010564	0.719888
-0.59898	-0.71666	-1.04188	-1.40956	-0.92664	-0.94553	-0.90881	0.01694	0.692896
-0.59898	-0.63204	-0.87508	-0.73567	-0.34704	-0.37481	-0.34407	0.006928	0.531015
-0.42635								
-0.42635	0.382244	0.593122	0.449113	0.031359	0.576054	0.439183	0.02078	0.561763
-0.42635	0.917896	1.42404	1.72019	0.918704	0.851424	0.881926	0.015246	0.868766
-0.42523								
-0.42523	-0.22566	-0.19995	-0.14541	-0.20421	-0.26514	-0.27052	0.084243	0.125115
-0.65978								
-0.65978	-0.72141	-1.23399	-1.09173	-0.77135	-1.19094	-0.78936	0.048367	0.512584
1.283337	0.481976	0.674986	0.997394	0.632872	0.802286	0.954931	0.011478	0.515418
1.283337	-0.47746	-0.67777	-0.52921	-0.23347	-0.4705	-0.57565	0.017501	0.444295
1.283337	-0.01436	-0.50624	-0.32243	0.008336	-0.2598	-0.43738	0.005059	0.514578
-0.2844								
-0.2844	-0.82409	-1.34177	-1.63274	-0.99078	-1.18014	-1.04505	0.007827	0.808651
-0.2844	0.133172	0.252884	0.450611	-0.12348	-0.09812	-0.04287	0.002137	0.574093
1.732405								
1.732405	0.126723	0.207562	0.052078	-0.02678	0.41432	0.475114	0.01526	0.501895
0.500883	0.908876	1.088182	1.171933	0.838931	1.09178	1.238753	0.014694	0.399822
0.500883	1.802629	0.025746	-0.32142	-0.06818	0.049438	0.777206	0.075493	2.124048
0.500883	0.218982	0.26096	0.169616	0.308357	0.656563	0.51412	0.008122	0.486947
0.40666								
0.40666	0.498092	0.728192	0.550324	0.291966	0.861966	0.317445	0.011435	0.57
1.330797								
1.330797	2.632443	3.945527	2.702437	2.703563	2.058455	2.63604	0.039528	1.887072
-1.02994	-0.31818	-0.46349	-0.36554	-0.20526	-0.3826	-0.34664	0.039745	0.258225
0.113087	-0.26927	-0.09508	0.353766	0.128186	-0.03446	-0.13464	0.016847	0.623039
0.113087	1.301257	1.435883	1.392627	0.902431	0.975725	1.206657	0.003686	0.533452
0.113087	-1.12467	-1.61783	-1.37022	-1.04089	-1.47955	-1.36201	0.082509	0.576939
0.113087	-1.08347	-1.73432	-2.04126	-1.1348	-1.45341	-1.19569	0.007706	0.957785
0.113087	-0.92899	-1.57859	-1.75825	-0.95737	-1.21519	-0.91483	0.02808	0.843417

0.113087	1.936257	2.37844	1.236523	1.690061	1.042355	1.809823	0.111464	1.336085
0.113087	-0.77738	-1.37516	-1.79117	-0.85185	-1.25102	-0.96119	0.002036	1.013786
0.113087	-0.56979	-1.22116	-1.44841	-0.76353	-1.02921	-0.77857	0.058378	0.878613
0.113087	-1.28455	-1.56076	-1.9168	-1.32249	-1.55574	-1.26422	0.026106	0.65258
0.113087	-1.23798	-1.66143	-1.6835	-1.19855	-1.19929	-1.09536	0.111039	0.588139
-1.02994	0.655506	1.101733	1.486357	1.285813	1.31739	1.084089	0.000988	0.830851
0.18321	1.971197	2.25893	2.078467	1.300769	2.10457	1.642703	0.069965	0.958161
-0.61468	0.549001	0.365616	0.55236	0.447177	0.454166	0.591678	0.250631	0.226062
-0.61468	-0.20591	-0.19961	0.051302	0.162019	-0.0007	-0.0864	0.001031	0.367928
0.18321	0.473965	0.210736	0.243329	0.396719	0.233053	0.119808	0.161113	0.354157
-0.33983	-0.29636	-0.43343	-0.9729	-0.55282	-1.07011	-0.88577	0.06784	0.773747
-0.86457								
0.28327	-0.54702	-0.80978	-0.78099	-0.62681	-0.4533	-0.62139	0.021238	0.356484
-0.86457	-0.37314	-0.59881	-0.63177	-0.26477	0.156211	-0.14641	0.009807	0.78798
0.28327	1.318149	1.454857	1.56121	1.191172	2.11124	1.547787	0.02219	0.920068
-0.33983	-0.87174	-1.33546	-1.51119	-1.02906	-1.16489	-1.01133	0.000619	0.639452
-0.33983	-0.14863	-0.50458	-0.66148	-0.17058	-0.9125	-0.45315	0.05959	0.763874
1.005207	0.428667	0.646522	0.412168	-0.09623	0.667418	0.761911	0.017724	0.858139
1.005207	-0.10959	-0.14118	-0.32868	-0.49558	0.087838	0.058124	0.040379	0.583417
1.005207	1.161951	1.462953	1.34016	0.734003	1.177445	1.02609	0.02801	0.728951
0.79134								
0.79134	-1.12087	-1.72387	-1.82053	-0.92784	-1.46222	-1.09565	0.005351	0.892692
0.79134	-0.91857	-1.37757	-1.77971	-0.88286	-1.37712	-0.98166	0.006138	0.896858
0.79134	-0.13053	-1.14641	-1.38505	-0.86893	-1.33561	-1.0232	0.002337	1.254527
0.79134	-1.08517	-1.73922	-1.82721	-1.09414	-1.64385	-1.17589	0.008757	0.742045
0.79134	0.291278	0.587814	-0.91999	-0.28603	-0.35519	-0.4747	0.115675	1.507805
0.22555	1.141967	1.212687	1.154107	0.863787	1.057495	1.241767	0.103523	0.377979
0.22555	-0.73344	-0.54392	-0.50265	-0.31784	-0.3964	-0.40629	0.054961	0.415601
0.22555	0.93085	1.266303	1.449452	1.582553	1.729365	1.536598	0.168043	0.798515
0.17621	-0.51762	-0.75004	-0.82343	-0.26972	-0.51208	-0.50677	0.009598	0.553705
0.17621	0.558103	0.589738	0.521966	0.187725	0.338941	0.364351	0.023932	0.402013
-0.05308	0.939521	0.9056	1.030792	1.12442	1.122365	1.01344	0.004541	0.21882
0.17621	0.877265	0.987605	0.768362	0.918543	0.812141	0.779213	0.163438	0.219243
-0.28077	0.987789	1.038201	1.024291	1.439833	0.931308	1.092856	0.089179	0.508525
-0.28077	0.517196	0.225713	0.602	0.856437	1.185765	0.86741	0.005874	0.960052
-0.05308	0.474643	0.462085	0.604303	0.87162	1.146325	0.77307	0.003823	0.68424
0.473117	-0.7842	-1.32941	-0.92089	-0.80241	-0.45381	-0.74058	0.037964	0.875598
0.473117	0.375096	0.343058	0.129831	0.151846	0.737412	0.518939	0.006996	0.607581
0.05131	0.880901	1.009015	1.133272	0.718045	1.12245	0.971015	0.022037	0.415228
0.473117	0.401651	0.78524	0.746366	0.581373	1.13528	0.984009	0.007762	0.733629
0.05131	0.911063	1.018928	0.96963	1.071622	1.464925	0.991356	0.066947	0.553862
-0.0955	0.325254	0.536963	0.721994	0.8742	0.612921	0.580854	0.025189	0.548946
-0.42943	0.963821	1.129104	1.03349	1.088193	1.042131	0.860152	0.098619	0.268952
-0.53767	-0.00359	-0.05143	-0.12908	-0.5853	-0.13273	0.117268	0.003707	0.702572
-0.42943	0.378662	0.208258	0.146259	0.729174	0.970542	0.821706	0.012607	0.824283
-0.0955	0.635745	0.94174	0.896271	0.390376	0.684695	0.32263	0.043579	0.61911
-0.0955	0.712678	0.751843	0.243624	1.444622	0.980154	1.049106	0.069673	1.200998

-0.13116	-0.45997	-0.80581	-0.95761	-0.56662	-0.83793	-0.72464	0.016572	0.497646
-0.13116	-0.75847	-1.15822	-1.56474	-0.92192	-1.28314	-1.17168	0.030833	0.806266
-0.13116	0.331801	0.761509	1.28738	0.630234	0.29699	0.166683	0.001183	1.120697
-0.1025	-0.16559	-0.04735	0.284708	-0.05841	0.35447	0.07946	0.004699	0.520055
-0.1025	-0.63015	-0.82921	-1.36249	-1.05434	-0.63255	-0.57263	0.006153	0.789855
-0.1025	-0.90349	-1.18573	-1.6347	-0.61951	-1.45319	-0.91901	0.066327	1.015184
-0.1025	-0.5732	-0.58287	-0.37529	0.215833	0.371986	-0.17538	0.001361	0.954853
0.75924								
0.75924	0.005802	0.346108	0.231872	0.235303	0.559734	0.427293	0.00324	0.553931
0.75924	0.413083	0.791966	1.029113	0.612114	0.411422	0.33979	0.002888	0.689323
0.243797	-0.01045	0.018926	-0.01163	-0.08079	0.336298	0.335798	0.050517	0.417089
0.243797	-0.55611	-0.85993	-0.81788	-0.48471	-0.78743	-0.5858	0.034444	0.375217
0.243797	-0.35502	-1.20633	-1.35474	-0.63445	-0.62535	-0.50373	0.007178	0.999719
-0.34211	-0.25583	-0.34527	-0.24193	-0.29016	0.194415	-0.14061	0.097474	0.53968
-0.34211	-0.53986	-0.48465	-0.1566	-0.0456	-0.41445	-0.42679	0.132327	0.494258
2.01478	0.837292	1.326457	1.341167	0.798368	1.238605	1.315757	0.010959	0.542799
2.04177	-1.40562	-1.9284	-2.05585	-1.54149	-1.94451	-1.66095	0.009918	0.65023
-0.34211	0.247545	0.479281	0.83959	0.398898	0.868494	0.504817	0.005121	0.620949
2.01478	-1.0531	-1.39841	-1.44256	-1.24203	-1.16743	-1.13113	0.155138	0.389456
2.04177	-0.542	-1.23036	-1.21611	-0.53846	-0.72545	-0.6638	0.002918	0.691896
2.04177	-0.77671	-1.09994	-1.36608	-0.85427	-0.95015	-0.84859	0.013102	0.589362
-0.23161	0.09329	0.008782	-0.28513	-0.22527	0.214737	0.176756	0.010541	0.499871
0.092827	-0.30471	-0.52412	-0.66342	-0.43429	0.428875	0.251776	0.004105	1.092296
0.405767	-0.72893	-1.03523	-1.46835	-0.89915	-0.76466	-0.84261	0.009079	0.739416
0.405767	-1.25245	-1.86444	-2.13798	-1.40704	-2.12173	-1.47797	0.003467	0.885532
0.405767	0.199219	0.07612	-0.22286	0.067249	0.653007	0.266567	0.159948	0.875862
0.405767	0.100527	0.071691	-0.00014	-0.09401	0.269778	0.111882	0.036103	0.363792
0.405767	-0.43968	-0.59294	-1.01505	-0.53179	-0.70308	-0.49381	0.007425	0.575369
0.405767	-1.20714	-1.30082	-1.59039	-1.13884	-1.59804	-1.12407	0.001803	0.473973
0.405767	-0.71071	-0.99533	-1.0463	-0.69424	-0.80486	-0.69703	0.019833	0.352063
0.092827	-0.09171	-0.23024	0.125467	0.550043	-0.81902	0.928195	0.094941	1.747215
0.092827	-0.59145	-0.6827	-0.58773	-0.95093	-0.34579	-0.43574	0.013314	0.605144
0.344897	0.735584	1.29087	1.251483	1.057423	1.896995	1.345694	0.00918	1.161411
-0.06526	1.794263	2.169823	1.979026	1.73678	2.78025	2.203797	0.073089	1.04347
-0.23161	1.21866	1.247593	1.177887	1.0593	1.6597	1.540007	0.01652	0.6004
-0.06526	-0.03528	0.100441	0.021345	0.222147	0.384207	0.240682	0.010938	0.419491
1.6403	1.592357	0.922437	0.021541	0.463662	0.787029	0.165091	0.098517	1.570816
2.171913	-0.77628	-1.06284	-1.22247	-0.76364	-0.87354	-0.77988	0.040766	0.458831
2.171913	0.690064	0.816045	0.678186	0.700773	1.54665	1.119698	0.026322	0.868464
1.92735	-1.0184	-1.45776	-1.59295	-1.01891	-1.12231	-1.01476	0.044537	0.578187
1.6403	3.239297	3.12977	1.468554	1.887577	1.559995	2.07178	0.007737	1.770742
-0.90603								
-0.51614								
-0.51614	1.231076	1.445274	1.411208	0.941521	1.705505	1.325092	0.031022	0.763984
-0.90603	0.699966	1.257824	1.341973	0.646195	0.88866	0.432056	0.034882	0.909917
-0.33615	-0.87081	-0.85285	-0.0848	0.175909	-0.3579	-0.6246	0.001952	1.046716
-0.39293	-0.29627	-0.24888	-0.12655	-0.17411	-0.31617	-0.30385	0.073604	0.189621

-0.39293	-0.44217	-0.46727	-0.35085	-0.2218	-0.4199	-0.44603	0.036633	0.24547
-0.39293	-0.3517	-0.03556	0.114041	0.384367	0.265345	0.251486	0.023848	0.736068
-0.33615	-0.80091	-1.35654	-1.72297	-0.98111	-1.19132	-0.99735	0.001578	0.922058
-0.33615	-0.16066	-0.16017	-0.10019	0.237875	0.431906	0.121085	0.048853	0.592561
0.745792	0.924297	1.27802	1.132676	0.786196	1.357545	1.034017	0.165276	0.571349
0.745792	-1.12595	-1.45709	-1.80674	-1.24367	-1.59104	-1.19523	0.025535	0.68079
0.745792	0.397758	0.504106	0.569944	0.35428	1.15504	0.802111	0.005934	0.80076
3.305025	1.048017	0.915623	0.933197	0.813827	0.98741	0.680009	0.244346	0.368008
3.305025	-0.39667	-1.01219	-1.1327	-0.47728	-0.37967	-0.2594	0.021653	0.873306
3.305025	-0.80537	-1.39569	-1.56991	-0.67248	-0.94863	-0.90249	0.011114	0.897427
3.305025	1.224757	0.58345	-0.27341	-0.19415	-0.78213	0.431362	0.103392	2.00689
-1.27523								
-1.27523	0.069016	0.028016	0.279486	0.425794	-0.25092	-0.08974	0.000613	0.676712
-0.19527								
-0.07188	0.533684	0.521145	0.519682	0.367595	0.556869	0.633295	0.060028	0.265701
-0.07188	-1.09827	-1.56744	-1.91181	-1.21201	-1.61543	-1.32788	0.026624	0.81354
-0.07188	-0.62953	-0.44464	-1.56529	-0.97287	-1.11383	-0.69077	0.163275	1.120647
-0.07188	0.688838	0.266517	-0.24202	0.173327	-0.07264	0.082272	0.00777	0.930855
-0.14527								
-0.14527	1.073964	0.990271	1.193207	0.926836	1.654365	1.459917	0.015919	0.727529
-0.14527	0.432519	0.46626	0.665857	0.572678	0.738738	0.528555	0.027595	0.306219
-0.67007	-0.71214	-0.91019	-1.73154	-0.90763	-1.11751	-0.97989	0.019042	1.019404
-0.67007	-0.09138	-0.05379	0.011845	-0.02872	-0.36189	-0.29983	0.016949	0.373737
1.541593								
1.541593	0.703979	0.518257	0.346797	0.450541	0.67298	0.391706	0.184538	0.357182
-0.8565	1.201653	1.478747	1.748211	1.48585	1.201085	0.748397	0.011071	0.999814
-0.98185	1.217193	1.670297	1.81889	1.624653	1.314985	1.043459	0.040607	0.775431
-0.98185								
-0.8565	-0.32186	-0.51672	-0.49984	-0.00425	-0.38827	-0.33955	0.047836	0.512473
	-0.19094	0.054823	0.281477	-0.14351	0.175505	0.076565	0.015172	0.472413
0.467537								
0.467537	1.361503	1.248498	0.36911	0.936717	1.484916	1.217644	0.106603	1.115806
-1.32385								
-1.32385	0.775063	1.157067	1.320413	1.10577	0.418055	0.276254	0.002704	1.044159
-1.32385	1.369593	1.498957	1.560943	1.385527	0.820904	0.860219	0.017438	0.740039
-0.71534								
-0.71534	0.779613	1.117016	1.152783	0.516925	0.911351	0.806034	0.00045	0.635858
-0.95779	0.06254	0.236353	0.224385	0.556603	0.668571	0.421476	0.007342	0.60603
-1.20854	1.593943	1.663363	1.755433	1.097721	1.30025	1.06272	0.003223	0.692713
-1.20854	-0.85519	-0.90897	-1.04913	-0.67706	-0.58877	-0.65721	0.064896	0.460352
-1.20854	1.404268	1.980692	1.003691	0.664315	0.584902	0.225072	0.098588	1.75562
-0.95779	0.866233	0.86922	0.673671	0.330332	0.380601	0.693528	0.010851	0.538888
-0.95779	4.404493	4.41618	3.518243	4.121607	3.552705	3.41271	0.077443	1.00347
0.55677	0.497134	0.262357	0.803926	0.289764	0.751157	0.536393	0.044702	0.541569
0.55677	0.094118	0.091438	-0.15409	0.199052	0.047136	0.1475	0.094156	0.353138
-0.67898								
-0.90893	0.926032	0.993745	1.386943	0.867007	1.139328	1.199337	0.021003	0.519936

-0.90893								
-0.67898	0.173588	0.184345	0.266909	-0.05654	0.62341	0.509707	0.001783	0.679954
-0.67898	1.41619	1.571617	1.73837	1.219393	1.42093	1.049335	0.003502	0.689035
-0.1368								
-0.1368	-1.02586	-1.50437	-1.67414	-1.13493	-1.44255	-1.10117	0.034896	0.648285
-0.1368	-0.66502	-0.8012	-1.28334	-0.93192	-1.03483	-0.86015	0.031363	0.618323
0.969147	0.601731	0.735552	0.876425	0.26488	0.513738	0.719526	0.03222	0.611545
0.969147	0.438359	0.444528	0.797357	0.737424	0.870177	0.711173	0.037865	0.431818
2.786723	-0.04561	0.135821	0.187251	0.008665	0.501225	0.488938	0.025704	0.546831
2.786723	1.231953	1.781675	0.436202	0.503079	0.8364	0.981267	0.16263	1.345472
0.840967								
1.688605	0.228171	0.743343	0.892414	0.017283	0.791076	0.881718	0.01197	0.875131
0.840967	-0.01933	-0.18814	0.092091	0.286446	0.416432	0.283987	0.01935	0.604576
1.688605	-0.09442	-0.30228	-0.06446	0.181265	0.245277	0.206208	0.07734	0.547552
-0.60065	1.185795	2.093754	1.222836	0.914232	0.68787	0.779493	0.042144	1.405885
-0.60065	-0.18277	-0.11947	-0.16538	-0.35339	0.250983	0.028298	0.00158	0.604369
-0.60065	-0.53595	-0.79651	-0.80634	-0.71151	-0.75953	-0.7847	0.057971	0.270384
-0.60065	0.010402	0.310577	0.553928	0.780009	0.512281	0.336684	4.93E-05	0.769607
-0.79717	0.274742	0.604191	0.948818	0.547673	0.461268	0.260148	0.005675	0.68867
-0.79717	1.298461	0.223256	0.213526	0.193819	0.164154	0.197636	0.148933	1.134307
-0.79717	2.07328	2.0562	1.726957	1.484521	1.189076	1.811753	0.047596	0.884204
-0.39056	-0.0838	-0.14293	-0.2664	-0.15003	0.021995	0.129168	0.021186	0.39557
-0.39056	0.458091	0.797541	1.093544	1.047434	0.835447	0.73381	0.082131	0.635453
-0.24996								
-0.39056	0.006943	0.125436	0.319508	0.301827	0.454983	0.244881	0.043514	0.448039
-0.24996	1.174483	1.494568	1.248173	0.728295	0.246478	0.576651	0.103791	1.24809
-0.24996	0.934966	1.605497	1.889658	1.51783	1.518325	1.586387	0.002359	0.954692
-0.86386								
-0.82775								
-0.82775	2.769353	2.59225	2.392447	2.185933	3.176575	2.285397	0.035536	0.990642
-0.86386	-0.10597	-0.20809	-0.44582	-0.2348	-0.31369	-0.27162	0.036632	0.339847
-0.86386	-0.04313	0.779968	0.705555	0.164435	-0.17449	-0.27841	0.009909	1.058378
	-0.34942	-0.67275	-1.0867	-0.58785	-0.69793	-0.48345	0.003548	0.737285
	0.572056	1.227348	-0.34994	0.201167	-0.79258	-0.79946	0.067891	2.02681
0.417377	0.520233	0.63952	0.8621	0.533736	0.810101	0.720704	0.018434	0.341867
0.417377	-0.58091	-1.04624	-1.17387	-0.77307	-0.60636	-0.61431	0.00658	0.59296
0.417377	0.027305	0.17792	-0.09619	0.168706	0.589759	0.620416	0.031591	0.716608
-0.31421	0.697945	1.052967	1.022058	0.671719	0.84132	0.794439	0.009916	0.381248
2.134838	0.592029	0.903012	1.420122	0.39962	1.33773	0.68521	0.104141	1.020502
2.134838	-0.93693	-1.4497	-1.62376	-1.06824	-1.14323	-1.08192	0.004383	0.686835
2.134838	-0.2572	-0.65228	-1.20661	-0.62359	-0.89704	-0.71365	0.020674	0.949407
2.134838	-0.10925	-0.46594	-0.73421	-0.21338	-0.55962	-0.44199	0.014611	0.624964
-0.31421	-0.17372	-0.54043	-0.38767	-0.20587	-0.58658	-0.4909	0.019129	0.412859
-0.44498	1.106564	1.44597	1.767683	0.693128	1.50195	1.198804	0.003021	1.074555
-0.44498	-0.62545	-0.77589	-0.79413	-0.69017	-1.02002	-0.86125	0.025542	0.394566
-0.44498	-0.51577	-0.68802	-0.82492	-0.35243	-0.65741	-0.51447	0.063032	0.472493

2.721253	0.164457	0.218622	0.443189	0.04218	0.437051	0.451933	0.057343	0.409753
2.721253	-0.40479	-0.8219	-0.84812	-0.47442	-0.84828	-0.60803	0.024875	0.44349
2.721253	0.020165	-0.47826	-0.91082	-0.21102	-0.07057	0.238058	0.003025	1.148877
1.322613	-0.64131	-0.42513	-0.06506	-0.29671	-0.63204	-0.60601	0.088191	0.576252
1.322613	0.498071	1.061561	1.17935	0.747279	0.203099	0.461161	0.006385	0.976251
1.322613	-0.82666	-1.14637	-1.09869	-0.71002	-0.98853	-0.77145	0.00841	0.436358
-0.42486								
-0.28399	0.380139	0.918907	1.03356	0.28808	0.326888	0.305253	0.006754	0.74548
-0.28399								
-0.42486	-0.97705	-1.72468	-1.73038	-1.03852	-1.86693	-1.36573	0.022396	0.889879
-0.42486	1.057763	1.157687	1.388653	0.931387	1.16481	1.028413	0.017589	0.457266
-0.86228	0.970926	1.31663	1.45929	0.961246	1.111931	1.09478	0.004252	0.498044
-0.84108	3.53015	2.190893	1.290605	1.50476	1.50677	1.417744	0.036429	2.239545
-0.84108	0.108523	0.140827	0.356316	0.286559	0.278598	0.157022	0.105257	0.247794
1.566443	1.308733	1.5359	1.792095	1.413213	1.665845	1.620213	0.012913	0.483362
-0.84108	0.849605	0.789577	1.31139	1.018877	0.988719	1.001645	0.030849	0.521813
-0.86228	3.035297	2.632483	2.875393	2.08595	2.502135	2.30918	0.076154	0.949347
1.566443	1.161402	1.312407	1.210634	1.024581	1.484825	1.502457	0.099479	0.477876
	0.944165	0.637074	0.40592	0.546307	0.633365	0.405721	0.122087	0.538444
	-0.37348	-0.60562	-0.93003	-0.6408	-0.50341	-0.58078	0.011803	0.556556
-0.52562	-0.24412	-0.02507	0.488284	0.286439	0.253578	0.023842	2.25E-05	0.732405
-0.52562	0.005788	-0.03675	0.038397	0.17384	0.151347	0.037903	0.054106	0.210594
-0.88695	0.562943	0.489807	0.828534	0.135288	0.47569	0.722843	0.016268	0.693246
0.221037	0.299348	0.623296	0.941809	0.433294	0.948789	0.894379	0.013742	0.649441
-0.88695	0.007358	0.201584	0.461112	-0.00173	0.133376	0.048448	0.024246	0.462843
0.221037	0.912064	1.45786	1.780183	1.303673	1.548135	1.22701	0.003132	0.868119
2.55669	1.190991	1.46985	1.280744	0.587227	1.13676	1.226427	0.046214	0.882623
2.55669	1.133609	0.998554	1.376607	1.313007	0.942447	1.084949	0.01284	0.43416
2.55669	0.769594	0.845743	0.421096	0.968404	0.645053	0.807653	0.006533	0.547308
0.400547	0.728419	1.159295	0.655271	0.370839	0.811529	1.048875	0.001792	0.788456
0.400547	-0.31784	-0.49757	-0.38148	-0.07377	-0.2723	-0.08921	0.017385	0.423802
0.400547	2.178827	2.006263	1.93576	1.589431	1.67952	2.051383	0.324141	0.589395
1.779107	0.227245	0.626465	0.759343	0.038985	0.696766	0.810275	0.000271	0.77129
1.779107	-0.95705	-1.13147	-1.44995	-1.16268	-1.2327	-0.9722	0.003172	0.492904
1.779107	-0.73615	-0.84309	-0.80252	-0.80829	-0.64735	-0.64058	0.033258	0.202508
-0.15962								
-0.15962	-0.25947	-0.50028	-0.96088	-0.78238	0.054975	-0.86776	0.270288	1.015851
-0.15962	0.320895	0.884486	1.287767	0.669134	1.059222	0.551206	0.001143	0.966872
-0.05788								
-0.05788	-0.1315	-0.28191	-0.63131	-0.17812	-0.29881	-0.16728	0.074515	0.499805
-0.05788	0.469372	0.969767	1.348017	0.738024	1.00775	0.605375	0.000103	0.878644
0.416027								
0.416027	-0.05664	0.167896	0.138805	-0.45953	-0.33382	-0.15532	0.019012	0.627426
1.284537	0.128462	0.222079	0.107193	0.061187	0.417298	0.227406	0.025892	0.356111
1.284537	1.38555	1.811367	1.741313	1.057198	2.057755	1.702273	0.023485	1.000557
-0.18686								
-0.18686	0.695775	0.320612	0.041762	0.285663	0.191131	0.244498	0.016716	0.654013

1.284537	0.499699	0.87114	1.160272	0.520527	1.27699	1.140137	0.033992	0.777291
-0.18686	2.483507	3.089663	1.543647	1.680167	2.183555	1.61079	0.150223	1.546017
-0.0389	0.617465	0.792604	0.824728	0.293338	0.274612	0.072072	0.02272	0.752656
-0.0389	-0.7399	-1.36049	-1.55043	-0.83911	-1.26315	-0.85784	0.000357	0.810524
-0.0389	-0.41918	-1.04914	-1.13284	-0.5686	-0.80096	-0.49664	0.044376	0.713661
-0.46461	0.295081	0.572163	0.314742	-0.14012	0.233806	0.395938	0.00388	0.71228
-0.46461	0.553988	0.840365	1.266483	1.011387	1.17275	0.78881	0.010225	0.712496
-0.46461	1.043601	1.277607	1.70246	1.288083	1.36802	1.053737	0.006515	0.658859
0.314313	0.283804	0.574146	0.767225	0.044672	0.644466	0.821043	0.001341	0.776372
0.314313	-0.70111	-1.17338	-1.35695	-0.77035	-1.137	-1.09125	0.009868	0.655843
0.314313	0.200266	0.214414	0.185182	0.174562	0.356115	0.1614	0.026807	0.194715
-0.52412								
-0.23445	0.86968	1.42877	1.923497	1.532313	1.60713	1.286363	8.06E-05	1.053816
-0.23445								
-0.52412	-0.29925	-0.41028	-0.6799	-0.70906	-0.84807	-0.5755	0.055456	0.548813
-0.03395	0.546974	0.463057	0.532773	0.290531	0.468741	0.392737	0.049801	0.256443
-0.03395	1.187313	1.406227	1.510963	1.340217	1.79063	1.54274	0.004877	0.603317
-0.9827	0.782302	1.341593	1.74262	1.276497	1.02228	0.793544	0.003428	0.960318
-0.60997	-0.11209	-0.19261	-1.01882	-0.48708	-0.34795	-0.66034	0.032469	0.906722
-0.60997	-1.32633	-1.75732	-2.06769	-1.33912	-1.83105	-1.28898	0.00734	0.77871
-0.60997	0.224961	0.556792	1.049882	0.879626	0.735954	0.312824	0.025435	0.824921
-0.60997	1.099342	1.259057	1.182087	1.16279	1.522145	1.24909	0.011672	0.422803
-0.60997	-0.00478	0.351019	0.471139	-0.06598	0.089529	0.071886	0.004197	0.537117
-0.9827	-0.37329	-0.46631	-0.66502	-0.58687	-0.50736	-0.44053	0.019726	0.291724
-0.9827	0.432143	0.796953	0.710972	0.21558	0.340582	0.439673	0.00776	0.581373
-0.16887	-0.77305	-0.93217	-1.10717	-1.08114	-0.941	-0.64613	0.093221	0.461033
-0.16887	0.559365	0.499301	0.483497	0.049059	0.484641	0.519838	0.042027	0.510306
-0.16887	-0.68824	-1.17624	-1.44318	-0.75215	-1.19013	-0.96736	0.012342	0.754935
-0.16887	-0.94474	-1.34318	-1.71364	-1.01057	-1.3833	-1.15226	0.014501	0.768899
0.03598	0.942159	0.80565	0.537553	0.297971	0.341457	0.320437	0.058912	0.644188
0.03598	1.165277	1.9472	2.344207	1.430387	1.442585	1.272313	3.75E-05	1.17893
-0.8446	0.88695	1.48406	1.838217	1.21398	0.80767	0.733848	0.002505	1.104368
-0.8446	0.409088	0.598721	1.004112	0.633644	0.973654	0.63553	0.021221	0.595024
-1.18606	0.382097	0.286629	0.612633	0.371417	0.469445	0.534335	0.026839	0.326004
-1.18606	0.838213	1.16635	1.616763	1.21399	0.719718	0.632942	0.006194	0.983822
-0.4641	-0.34697	0.387712	0.627238	0.120702	0.0661	0.103794	0.026722	0.974203
-0.4641	-0.46733	-0.51057	-0.11324	0.196385	0.027351	-0.31816	0.002085	0.706955
-0.4641	1.137377	1.588083	1.907373	1.471033	1.4096	1.132198	0.041456	0.775175
-0.49757	0.279859	0.722776	1.288037	0.775891	0.346464	0.356902	0.016055	1.008178
-0.49757	-0.974	-1.56621	-1.73905	-0.95087	-1.33116	-1.0818	0.006113	0.788184
-0.49757	0.116247	-0.19382	-0.14732	0.491342	-0.23444	-0.22782	0.053337	0.725779
	-0.82681	-1.39486	-1.21592	-0.82312	-0.884	-0.88746	0.017224	0.571732
-0.71076	0.188038	0.42489	0.710795	0.617019	0.345025	0.289113	0.002033	0.522756
-0.71076	-0.78573	-1.00858	-1.25354	-0.79717	-1.0904	-0.92829	0.007478	0.467809
-0.71076	0.100371	0.286324	0.399167	0.42889	0.085892	0.03993	0.040442	0.38896
	1.67684	2.186352	1.47002	0.677053	1.159178	0.524772	0.168538	1.66158
	-1.01718	-1.1769	-1.32682	-1.05586	-1.12226	-0.87732	0.011878	0.449493

1.106453	2.30983	1.98466	1.768943	1.53592	1.87107	1.61321	0.09587	0.77391
1.106453	0.261845	0.366768	-0.34086	-0.02855	-0.1119	0.033875	0.026421	0.707629
-0.05355	-0.08195	0.061596	-0.0394	-0.17871	0.214045	0.170474	0.02446	0.392755
-0.05355	0.571001	0.942088	1.393337	1.008616	1.304105	1.026773	0.001274	0.822335
	0.814086	1.24978	1.298483	0.583614	1.145965	1.143944	0.010568	0.71487
-0.05355	0.269934	0.313437	0.439516	0.544554	0.927117	0.527454	0.000207	0.657183
	-0.59167	-0.74835	-0.5557	-0.4632	-0.31094	-0.42271	0.004083	0.437407
-0.37348								
-0.37348	0.014804	-0.34033	0.325996	-0.17393	0.0229	-0.0119	0.258607	0.666324
-0.37348	-1.02695	-1.46699	-1.55149	-1.17353	-1.05346	-0.95102	0.003161	0.600477
0.143725								
0.162037	-0.69632	-1.22483	-1.10204	-0.48444	-0.58545	-0.62265	0.004826	0.740387
0.162037	-0.38093	-0.81848	-1.12481	-0.25375	-0.37787	-0.33916	0.000927	0.871062
0.162037								
0.143725	1.087691	1.113552	0.757707	0.556895	0.685662	0.750531	0.01667	0.556657
0.143725	-0.65601	-1.09664	-1.40725	-0.37027	-1.12278	-0.91399	0.010886	1.036974
0.143725	-0.35274	-0.5104	-0.552	-0.37099	-0.49135	-0.41043	0.016387	0.199251
1.289857	5.93282	5.750427	5.317907	5.003377	5.24329	4.75389	0.028486	1.17893
0.821137	1.338283	1.250457	1.412143	0.914383	1.610155	1.735283	0.000986	0.820901
0.821137	0.332612	0.35912	0.222953	0.180876	0.60605	0.599944	0.12261	0.425174
1.289857	0.767794	0.602381	0.324524	0.331686	0.905726	0.930974	0.020282	0.60645
0.097247	-0.20811	0.446355	0.779268	0.677516	0.906469	0.518	0.007753	1.114577
0.179	0.379041	0.304195	1.106203	0.549136	0.699951	0.609661	0.033111	0.802008
0.097247	0.206278	0.46642	0.354453	0.119096	0.837106	0.327746	0.023917	0.718009
0.179	-0.03753	0.092031	0.278691	0.269277	0.206885	0.088635	0.088462	0.316217
-0.22055								
-0.22055	-1.15978	-1.771	-1.68901	-0.96711	-1.4187	-1.08918	0.000101	0.803889
-0.22055	0.655013	1.264133	1.492837	1.064414	0.607977	0.449867	0.016608	1.042969
-0.60761								
-0.60761	-1.1083	-1.48281	-1.644	-1.07629	-1.43574	-1.06773	0.0405	0.576273
-0.60761	0.663924	1.34628	1.705443	0.983398	1.215035	0.94686	0.003349	1.04152
1.791853								
1.791853	0.623248	0.639636	0.220743	0.457128	1.345095	1.15832	0.000312	1.124352
1.791853	-0.41753	-0.47355	-0.51923	-0.43664	-0.6496	-0.45475	0.066638	0.23207
1.14838								
1.14838								
1.14838	0.293266	0.140779	-0.08261	-0.13949	0.268216	0.148796	0.019899	0.432761
2.710443								
2.710443	0.223283	-0.11487	-0.04466	0.167494	0.005946	0.418095	0.02514	0.532967
2.710443	0.391756	0.586333	0.2668	0.045742	0.320528	0.241271	0.023916	0.540591
1.085783								
1.085783	0.855381	0.508083	0.300128	0.322413	0.372908	0.175603	0.041399	0.679779
3.310173								
3.310173	0.842139	0.640527	0.643297	0.575874	1.01359	1.068962	0.017034	0.493088
3.310173	1.181876	1.309007	1.693707	1.181958	0.55728	1.029868	0.001472	1.136427
1.862553	0.498105	0.1814	0.36972	0.30414	0.990383	0.8528	0.038753	0.808983
1.862553	0.871304	0.105418	-0.236	0.131066	0.271857	0.247579	0.098232	1.107302

1.862553	1.738043	1.61474	1.0561	1.169321	0.761983	1.195344	0.181113	0.97606
3.07915								
3.07915	-0.58184	-0.54201	-1.31701	-0.59444	-0.89992	-0.66594	0.045676	0.774999
3.07915	-0.09343	-0.2063	-0.35235	-0.30331	-0.03079	-0.0507	0.011979	0.321562
-1.21552	0.037335	0.434264	0.848329	0.424492	0.424659	0.216599	0.051964	0.810993
-1.21552	-0.46737	-0.5958	-0.03648	-0.01966	-0.7157	-0.55664	0.006299	0.696037
-1.21552	-0.60456	-0.57957	0.204367	0.086686	-0.76766	-0.56447	0.000626	0.972024
1.4066	0.718581	1.026852	0.935094	0.421737	1.63282	1.202507	0.004931	1.211083
2.94584	0.292351	0.44321	0.166969	-0.31765	0.546356	0.669005	0.011135	0.986659
2.94584	0.669965	0.813362	0.957942	0.9325	0.676961	0.47024	0.004419	0.487702
2.94584	1.54337	1.709233	1.7803	1.0412	1.53838	1.308719	0.022866	0.7391
2.587867	0.562206	0.930157	1.276764	0.594544	1.145033	0.976571	0.006241	0.714558
2.587867	0.642751	0.596414	0.877022	0.844827	0.686289	0.631172	0.126781	0.280608
2.587867	1.75655	1.862663	1.739637	1.661813	1.34732	1.501387	0.001479	0.515343
0.788083								
0.788083	-0.60009	-1.1575	-1.4208	-0.60634	-1.24822	-0.99045	0.001864	0.820708
0.788083	-0.74966	-0.47548	-1.33442	-1.09016	-2.24227	-1.77886	0.022222	1.766785
	-0.05854	-0.19642	-0.36834	-0.03148	-0.40758	-0.4864	0.034403	0.454929
-0.56385	-1.25827	-1.93177	-2.26278	-1.59062	-2.38869	-1.76955	0.010421	1.130419
-0.56385	-0.00807	-0.44363	-0.44238	-0.04329	0.072047	0.090268	0.007832	0.533895
-0.56385	-0.06162	-0.41072	-1.23035	-1.05379	-1.19164	-0.37035	0.146004	1.16873
0.006223	0.172844	0.195328	0.001692	0.641995	0.486066	0.412077	0.063942	0.640303
3.21331	0.246267	0.308991	0.68779	-0.0482	0.223539	0.290533	0.00879	0.735986
3.21331	-0.03931	-0.11885	-0.47682	0.154806	0.258053	0.149214	0.024721	0.734877
3.21331	-0.48623	-0.80885	-0.91856	-0.40627	-0.35099	-0.24252	0.063637	0.676036
0.006223	0.360689	0.638531	0.86456	0.445792	0.955116	0.713632	0.001762	0.594427
-0.91348	0.505769	0.642673	0.713357	0.630037	0.818137	0.264419	0.068685	0.553718
-0.91348	1.58364	1.37631	0.838575	0.834756	1.066617	0.772117	0.053809	0.811523
0.419027								
0.419027	-0.82687	-1.25726	-1.36915	-0.87794	-1.12489	-0.88367	0.002479	0.542282
0.419027	-0.0273	-0.18707	-0.53941	0.140452	0.405254	0.253491	0.000704	0.94466
0.200822								
0.200822	-0.14572	0.395092	0.676754	0.330701	-0.18193	-0.27191	0.050944	0.948662
0.200822	-0.20078	-0.80147	-0.85277	-0.08256	-0.37041	-0.10824	0.006159	0.770211
0.200822	1.130343	1.337773	1.808257	0.902179	1.11055	1.038917	0.00436	0.906078
-0.03691	1.067649	1.313213	1.643933	0.980379	1.45103	1.272247	0.098166	0.663554
-0.03691	-0.75326	-0.56053	-1.0946	-0.42086	-1.21185	-0.56009	0.084978	0.790993
-0.03691	-0.46163	-0.75922	-1.26665	-0.75774	-1.17529	-0.26987	0.167799	0.99678
-0.03691	-0.98234	-1.50814	-1.81308	-1.12014	-1.45989	-1.22537	0.017867	0.830732
-0.03691	0.228197	0.417301	0.244722	0.770527	0.765689	0.397524	0.037476	0.54233
-0.5672	-0.42283	-0.6402	-1.11131	-0.79744	-0.96458	-1.00844	0.010174	0.688479
-0.5672	0.145598	0.548026	0.814574	0.399114	0.345807	0.298575	0.006275	0.668977
0.71797	0.409572	0.252073	0.601631	0.606304	0.464197	0.575307	0.060734	0.354231
-0.5672	0.866943	1.033713	1.287783	0.832685	1.184148	1.086913	0.017383	0.455098
0.71797	-0.02712	-0.43938	-0.89958	-0.83205	-0.27052	-0.15174	0.005784	0.87246
0.71797	-0.55288	-0.5663	-0.54141	-0.66419	-0.50395	-0.62652	0.00875	0.16024
0.970842	1.217843	1.24608	1.335353	1.107578	1.429775	1.22276	0.03725	0.322197

-1.1612									
-1.1612	0.34869	0.829801	1.427477	0.595018	0.325268	0.152495	0.001009	1.274982	
0.931807									
0.931807	0.995222	1.701447	1.868057	0.916485	1.720585	1.272123	0.008492	0.951572	
0.931807	-0.45777	-0.61491	-0.26937	-0.47108	-0.43141	-0.49322	0.128029	0.345547	
1.895637	0.570216	0.721656	0.929759	0.4579	0.850719	0.966357	0.007733	0.508457	
1.895637	-0.51128	-0.77448	-0.41195	-0.57967	-0.64394	-0.68488	0.02491	0.36253	
1.895637	-0.21102	-0.52647	-0.50196	-0.12165	-0.52938	-0.41675	0.03409	0.407737	
-0.22447									
-0.22447	-0.67744	-1.05805	-1.10038	-0.64449	-1.0947	-0.8779	0.02495	0.455891	
-0.22447	-0.7798	-1.13339	-1.00384	-0.57378	-0.76675	-0.76867	0.004435	0.55961	
-1.71877	0.013454	0.268323	0.866848	0.263149	0.431271	0.270981	0.000301	0.853393	
-1.71877	0.276976	0.913355	1.248923	0.514924	0.676583	0.438999	0.003638	0.971947	
0.429097	-1.55896	-1.92753	-2.16802	-1.32615	-1.93442	-1.62809	0.013134	0.841871	
0.429097	-1.04391	-1.59361	-1.6631	-1.15652	-1.29743	-1.36262	0.034243	0.619191	
0.29904	1.182033	1.632133	1.899435	0.993065	1.68084	1.347063	0.006566	0.906369	
0.29904									
-0.70638	0.968868	0.795597	-0.75603	-0.31667	-0.04285	-0.18592	0.018096	1.724898	
-0.70638	-0.48363	-0.50334	-0.17788	-0.51317	-0.56007	-0.44153	0.005963	0.382191	
-0.70638									
0.309127									
0.309127	-0.60202	-1.49713	-1.5219	-0.78923	-1.39675	-0.90559	0.000169	0.91988	
0.309127	-0.80135	-1.61158	-1.74551	-0.82202	-1.45804	-0.98424	0.002003	0.94416	
0.309127	0.475868	-0.35991	-0.70123	-0.03289	-0.3115	0.047348	0.008738	1.177096	
0.309127	-1.03232	-2.00862	-2.10641	-1.0213	-1.55745	-1.16457	0.010083	1.085104	
0.309127	-0.58687	-1.20846	-1.54622	-0.6049	-0.78719	-0.6873	0.001944	0.959351	
0.309127									
0.309127	0.981921	0.61408	0.047032	0.645305	0.488047	0.53021	0.002198	0.934889	
0.56922	0.140515	0.620727	0.869226	0.542146	0.927121	0.696395	0.019144	0.786606	
0.56922	0.653613	0.872802	0.921688	0.452438	0.860171	0.791315	0.007594	0.46925	
0.56922	0.527276	1.182717	1.25505	0.546036	1.20895	0.868574	0.025811	0.727774	
-0.7401	0.549072	0.99525	1.208923	0.865802	1.268725	0.980963	0.029267	0.719653	
-0.7401	0.097954	-0.07978	0.126719	-0.22418	0.536291	0.326412	0.016863	0.760469	
-0.7401	-0.58376	-0.30205	0.237571	0.076106	-0.59992	-0.44236	0.080351	0.837489	
-0.64838									
-0.64838	-0.10586	0.322021	0.43732	0.204412	-0.01931	0.003563	0.049443	0.543177	
-0.18059									
-0.18059	-0.34069	-0.82232	-0.98844	-0.48755	-0.78173	-0.49782	0.000158	0.647748	
-0.18059	-0.47557	-0.84245	-0.65231	-0.33558	-0.74945	-0.60381	0.025603	0.506874	
-0.19134	0.267625	0.480796	0.784369	0.526062	0.665285	0.586061	0.002493	0.516744	
-0.19134	0.260251	0.561218	0.499873	0.389956	0.410689	0.325437	0.026701	0.300968	
-0.19134	-0.10517	-0.12495	-0.07569	0.298665	0.169476	0.356873	0.179001	0.481823	
-0.75927									
-0.75927	-0.14265	-0.01706	0.07931	0.080015	0.332794	0.139198	0.026686	0.475442	
-0.75927	-0.21548	-0.58531	-0.28536	-0.01229	-0.4066	-0.41942	0.000286	0.573029	
0.121917									
-0.06629	-1.06474	-1.60214	-1.7215	-1.07766	-1.20493	-1.03548	0.006109	0.686016	

-0.12696	-1.55507	-2.49249	-3.03129	-1.38104	-2.18852	-1.71509	0.007941	1.650245
	0.083059	-0.23011	-0.69656	0.076473	-0.47397	-0.50681	0.019312	0.779614
-0.71079								
-0.92325								
-0.96185								
-0.12202	-0.50563	-0.68066	-0.82448	-0.63783	-0.55182	-0.5375	0.013063	0.318852
-0.12202	-0.02007	-0.36234	-0.42882	-0.17609	-0.07453	0.003434	0.017378	0.43225
	0.747061	0.584368	0.478066	0.459863	0.883713	0.893063	0.053134	0.4332
0.429097	0.590903	0.70511	0.674347	0.557493	1.084652	0.798275	0.056024	0.527159
	0.309131	0.464763	0.44093	0.219189	0.499609	0.46974	0.125873	0.280419
	6.694787	6.676894	5.553288	5.722893	5.491275	5.253913	0.034692	1.440874
0.093877								
-0.92133								
-0.81888								
	-1.42182	-1.64237	-1.7268	-1.13072	-1.74204	-1.1203	0.118567	0.621735
-0.08009	-0.67195	-1.43704	-1.76059	-0.91149	-1.7845	-1.53427	0.008754	1.112545
-0.44775								
-0.94074	0.176831	0.344544	0.749217	0.150949	0.551034	0.362823	0.019893	0.598267
-0.94074	0.467191	0.495418	0.976021	1.070059	0.724625	0.463789	0.057285	0.60627
-0.04406	0.456573	1.02106	1.40111	0.427967	0.960759	0.977236	0.000827	0.973143
-0.04406	0.212503	0.131118	0.155887	0.036678	0.855601	0.562576	0.002415	0.818923
-0.04406	-0.25276	0.487684	0.011869	-0.11919	-0.50096	-0.57832	0.146227	1.066001
-0.37873								
-0.37873	-0.70887	-0.80902	-0.64707	-0.24679	-0.52056	-0.37237	0.004577	0.562233
-0.37873	-0.4513	-0.77315	-0.44722	0.008037	-0.49874	-0.38063	0.014481	0.781191
-0.17287	1.013364	1.134126	1.602293	0.827895	1.008686	0.870635	0.006258	0.774398
-0.17287	0.356235	0.851656	1.371417	0.809289	0.520848	0.55659	0.000659	1.015182
1.304957								
1.304957	1.009059	1.681377	1.371303	0.325167	-0.04466	0.264369	0.034149	1.726038
1.72694								
1.72694	1.78665	2.347957	1.866053	1.194277	1.191745	1.258197	0.014658	1.156212
0.953613								
0.953613	-0.23257	0.281094	0.04112	-0.35797	-0.46803	-0.42888	0.062849	0.749124
-0.11882	-1.14358	-1.31215	-0.94297	-1.16955	-1.71901	-1.3683	0.034282	0.776041
-0.11882	-0.81512	-0.90204	-1.0196	-0.9295	-1.06188	-0.83902	0.227548	0.246761
-1.20977								
-1.20977	1.120237	1.455743	1.673263	0.831237	1.388175	1.24335	0.008709	0.842026
0.065012	-0.7067	-1.23881	-1.24217	-0.51197	-1.24762	-0.7632	0.110477	0.735642
0.065012	-0.15854	-0.56176	-0.80938	-0.42512	-0.5209	-0.47648	0.004816	0.650841
0.065012	-0.83169	-0.88504	-0.60453	-0.56498	-1.06393	-0.7814	0.002911	0.498945
-0.14916	-0.05391	-0.01469	0.341109	0.211397	0.099123	0.037678	0.004405	0.395014

0.303602	-1.50447	-2.211	-2.18588	-1.54716	-2.24855	-1.82114	0.030171	0.744075
-0.14916	0.638811	1.077238	0.638838	0.008801	0.425077	0.09523	0.171394	1.068437
0.303602	-0.89334	-1.43461	-1.67783	-0.81614	-1.71263	-1.1965	0.059167	0.896486
-1.03927								
-1.03927	0.752024	1.089759	0.99519	0.865092	1.141355	0.803146	0.023849	0.389331
-1.03927	0.222709	0.296543	0.499621	0.332673	0.359567	0.078987	0.088507	0.420634
-0.53753								
-0.53753	0.560554	0.899836	1.125487	0.772202	0.643081	0.619055	0.001014	0.564933
0.648462	0.714026	0.775922	0.614523	0.327816	1.138305	1.007464	0.004226	0.810489
0.648462	-0.53806	-0.79585	-1.13564	-0.90569	-1.1345	-0.83665	0.004193	0.597584
0.648462	-0.8086	-1.50966	-1.61507	-0.82305	-1.12172	-0.83186	0.010544	0.806469
0.648462	-0.34775	-0.45322	-0.90001	0.035869	-0.52078	-0.29983	0.109898	0.935878
0.648462	-0.87292	-1.50128	-1.58728	-1.00921	-1.51645	-1.16562	0.006348	0.714363
0.648462	-0.78662	-1.55898	-1.50498	-1.13989	-1.42283	-1.15264	0.005849	0.772361
0.648462	-1.06508	-1.38721	-1.55546	-0.7615	-1.31306	-1.1218	0.055738	0.793959
1.812337	0.347515	0.350084	0.423702	0.228731	0.056962	0.557789	0.012399	0.500826
1.812337	0.72438	1.11872	1.030729	0.222023	0.833484	0.968978	0.000269	0.896697
-0.75277	1.216403	1.629923	1.770587	1.048742	1.498925	1.452987	0.001551	0.721845
-0.75277	1.090742	1.14169	0.900809	0.501598	0.826405	0.852406	0.055174	0.640092
-0.75277	1.121743	1.35329	1.46169	1.242147	0.89205	0.816459	0.025728	0.645231
-0.04729	0.570361	0.827452	1.30985	0.695115	1.11629	0.960491	0.034665	0.739489
-0.04729	0.582072	0.551112	0.458736	0.437686	0.424741	0.13206	0.012064	0.450012
-0.25701								
-0.25701	0.380111	0.936349	1.013802	0.013775	-0.1519	0.08934	0.004572	1.1657
-0.25701	-0.10863	-0.83924	-0.37496	-0.53457	-0.58555	-0.58023	0.063827	0.73061
0.143103	0.820679	1.40496	1.73316	0.631673	1.599095	1.254867	0.011753	1.101487
0.143103	-0.62787	-0.69519	-0.64564	-0.13546	-0.16857	-0.27413	0.000994	0.559738
0.143103	0.288197	-0.15054	-0.70926	-0.03391	-0.32789	-0.1262	0.010868	0.997453
-0.74014								
-0.74014	1.24947	1.558	1.555983	0.737895	1.185565	1.24171	0.020917	0.820105
-0.74014	0.423251	1.05444	1.558967	1.005075	1.232075	0.909882	0.000896	1.135715
0.421612	-0.47645	-0.62695	-0.78663	-0.81125	-0.71168	-0.55995	0.008314	0.334795
	-0.60069	-0.63191	-0.89615	-0.83076	-0.51934	-0.52691	0.074029	0.376803
	0.728056	0.725589	0.746096	1.045068	1.171405	1.19736	0.127755	0.471771
	0.49198	0.0537	-0.03373	0.532924	0.100982	0.181394	0.250237	0.56665
0.421612	0.6434	0.746159	0.542697	0.098796	0.766347	0.630299	0.003819	0.667551
0.421612	1.569881	1.164563	0.643875	1.083	1.330085	1.535237	0.046572	0.926005
	0.722292	0.942388	1.251843	0.678042	0.75508	0.709469	0.013188	0.573801
-0.62706	0.720808	1.415823	1.635277	0.735692	1.38039	1.144797	2.59E-05	0.914469
	0.688216	1.185077	1.511863	0.961989	1.17176	1.072733	0.001465	0.823647
-0.62706	0.359451	0.416464	0.311613	-0.23868	0.152073	0.386262	0.036749	0.655147
-0.62706	-0.29045	-0.72294	-0.7616	-0.46999	-0.60328	-0.79703	0.094499	0.506578
2.908908								
2.908908	0.434967	0.361757	0.07924	0.40093	0.855342	0.723034	0.01743	0.776102
2.908908	-0.85838	-0.89198	-1.0975	-0.39875	-1.04985	-0.74134	0.203866	0.698753
2.908908	-0.88196	-1.38423	-1.62965	-0.92665	-1.23913	-0.90536	0.011765	0.747691
1.55423								

1.55423	2.70956	2.384617	2.243887	1.94121	2.5643	2.258017	0.197066	0.76835
1.498917								
1.498917	1.5738	2.163743	2.36057	1.451137	1.75944	1.357137	0.003647	1.003433
-0.13862	1.821943	1.96563	1.909247	2.08931	1.145233	0.781093	0.15449	1.308217
-0.13862	0.850341	0.86076	0.071846	0.061993	0.227531	0.133245	0.07624	0.798768
1.17511								
1.17511	-0.70467	-0.99584	-1.11584	-1.04155	-1.00625	-0.86169	0.086786	0.411168
1.17511	0.633747	0.848177	1.48044	1.665207	1.214865	1.123367	0.023961	1.031459
-1.31367								
-1.31367	0.193888	0.225292	0.472258	0.408338	0.126425	0.220778	0.020703	0.345834
0.025557								
0.025557	0.797022	1.18548	1.176567	1.044455	1.33557	1.16048	0.108694	0.538548
0.482683	-0.12146	0.046844	0.309291	-0.1619	-0.48258	-0.3643	0.017234	0.79187
0.482683	-1.00948	-1.25657	-1.1836	-0.82754	-1.28988	-1.22788	0.012589	0.462336
0.482683	0.515791	0.823731	0.858153	0.166015	-0.10918	-0.08329	0.001005	0.967336
-0.39292	0.291448	0.539873	0.809543	0.436337	0.512692	0.454246	0.005708	0.518095
-0.39292	-0.30662	-0.28347	0.507061	0.732002	-0.40578	-0.34245	0.00097	1.137781
-0.39292	0.196079	0.357043	0.926474	0.925054	0.789316	-0.18965	0.000655	1.116126
0.199503								
0.199503	-0.02082	-0.101	-0.035	-0.29118	0.045376	-0.11308	0.077678	0.336556
-0.7356	0.859054	0.994407	0.496413	0.341659	0.338026	0.755123	0.152647	0.656381
-0.77855	-0.32957	-0.19597	-0.06873	-0.42498	-0.05672	-0.06771	0.123316	0.368257
-0.7356	-0.36264	-0.49709	-0.48657	-0.27137	-0.48563	-0.46912	0.109502	0.225725
-0.77855	-0.40924	-0.59914	-0.51237	-0.23658	-0.67763	-0.4926	0.034386	0.441048
-0.77855	1.037091	1.620843	2.127593	1.66931	1.175895	0.806019	0.001631	1.321575
-0.1129	2.40922	1.913467	1.480104	1.27876	1.798462	1.428037	0.030519	1.13046
-0.1129	0.424495	0.455588	0.249478	-0.0972	0.68957	0.511723	0.000836	0.786773
-0.0251	4.611447	4.362313	3.690333	3.689475	3.696225	3.211547	0.016401	1.3999
-0.0251	0.605599	1.041513	0.899707	0.331516	1.413945	0.917702	0.008729	1.082429
-0.0251	-0.1227	0.074274	0.051622	0.112022	0.394495	0.187963	0.005703	0.517192
0.12844	0.322823	0.12227	-0.52692	0.640198	0.06485	-0.16313	0.180555	1.167119
0.12844	0.641532	0.955353	1.226	1.031992	1.102383	1.02311	0.005333	0.584468
0.33892	1.020895	1.408793	1.73362	1.001877	1.562095	1.373203	0.011977	0.731743
0.12844	1.138337	1.342863	1.291815	0.960456	1.099095	1.19968	0.029752	0.382407
0.33892	0.970317	1.046439	0.847941	0.331538	1.085075	1.033314	0.031154	0.753537
0.47516	0.675661	0.868673	0.692486	0.524855	0.929353	0.538158	0.057244	0.404498
-0.85869	-0.51207	-0.79643	-0.52707	-0.37377	-0.11592	-0.17999	0.015992	0.68051
-0.85869	-0.63974	-0.67086	-0.7699	-0.85697	-1.06085	-0.99565	0.047256	0.42111
-0.85869	0.27243	0.371246	0.821535	1.274533	0.735646	0.608269	0.016866	1.002103
-0.85869								
0.47516	0.09778	-0.30832	0.18811	0.371392	-0.04314	-0.11919	3.05E-05	0.67971
0.47516	-0.53712	-0.68439	-0.9952	-0.97552	-1.18439	-0.9581	0.007089	0.647271
-0.45723	0.303646	0.63962	0.910724	0.423411	0.713223	0.634325	0.022211	0.607078
-0.61687	0.891438	0.928556	0.635469	0.622917	0.67292	0.418281	0.088339	0.510275
-0.61687	0.162355	0.394812	0.552132	0.465713	0.377492	0.348114	0.069615	0.389778
-0.73351	-0.05638	-0.59958	-0.61576	-0.94332	-0.42864	0.116417	0.056449	1.059737
-0.61687	0.754824	0.932367	0.958642	0.590663	0.743127	0.736159	0.05471	0.367978

-0.45723	-0.49611	-0.61828	-1.01772	-0.94151	-0.88144	-0.70657	0.032508	0.521611
-0.45723	-0.21256	-0.60362	-0.91754	-0.51605	-0.82237	-0.60569	0.006121	0.704982
-0.45723	0.121406	0.266328	0.318984	-0.10474	0.444982	0.033716	0.068524	0.549717
-0.73351	0.037722	0.131027	0.689848	0.295524	0.669256	0.354295	0.008904	0.652127
-0.66354	0.635798	1.015504	1.2199	0.553939	0.924805	0.855197	0.001468	0.665961
1.28819	0.395483	0.498253	0.537965	0.478852	0.502438	0.648108	0.116266	0.252625
1.28819	-0.30605	0.367137	-0.86949	-0.76359	0.223683	-0.63641	0.179287	1.236626
1.28819	-0.26155	-0.3003	-0.48224	-0.45031	-0.26448	-0.35151	0.040919	0.220682
-0.53923	1.035438	0.852817	1.022299	0.745859	0.741449	0.614412	0.232167	0.421026
-0.53923	-0.73883	-1.23705	-1.55502	-1.06958	-0.88801	-0.75958	0.002774	0.816192
-0.53923	-0.66256	-0.95492	-1.34415	-1.02479	-1.08903	-0.65573	0.004276	0.688411
-0.66354	0.449201	0.757981	0.990425	0.708724	0.774982	0.61027	0.004048	0.541224
0.313793								
0.313793	0.20319	0.416863	0.328072	0.053969	-0.51003	-0.23544	0.003078	0.926892
0.313793	-1.24388	-1.59484	-1.61693	-1.09857	-1.41052	-1.24449	0.008559	0.518357
0.313793	-0.58292	-0.75693	-1.28726	-0.23106	-1.23014	-0.57656	0.019239	1.056197
0.313793	-0.20841	-0.82354	-1.61916	-0.51125	-1.4086	-0.89871	0.046852	1.410755
0.313793	-0.85136	-1.32495	-1.60388	-1.00762	-1.37475	-1.02614	1.60E-05	0.752528
-0.36648	-0.13796	-0.15843	0.4231	-8.90E-05	0.071004	-0.06524	0.017557	0.581534
-0.36648	0.581081	1.032708	1.495463	0.974679	0.052191	0.328703	0.019089	1.443272
-0.36648	0.028444	-0.027	0.229958	0.866188	0.596633	0.353363	0.000443	0.893192
-1.27128	-0.20893	-0.09246	-0.05108	-0.23456	0.153107	0.323721	0.07181	0.558285
-1.27128	2.689353	2.639057	2.361457	2.94526	2.49067	2.250467	0.137645	0.694793
-0.34444	3.38999	3.56293	3.297553	2.688053	2.969645	2.700657	0.103899	0.874877
-0.07622	0.052185	0.264986	0.775277	0.178981	-0.04713	0.30103	0.043746	0.822405
-0.07622	-0.7001	-1.04944	-1.17316	-0.80764	-0.80061	-0.60928	0.057204	0.563879
-0.07622	-0.62483	-0.69516	-0.22291	0.16307	-0.06754	-0.3836	0.000241	0.858232
-0.34444	-0.52469	-0.86154	-0.78165	-0.38506	0.297428	0.278773	0.011894	1.15897
-0.34444	0.248434	0.464645	0.924721	0.458568	0.389094	0.259728	0.064134	0.676287
1.81768								
1.81768	1.159757	0.022996	-0.18063	0.131732	-0.01802	-0.13378	0.103603	1.340385
1.81768	0.937096	0.463095	0.647505	0.345984	-0.65896	0.008028	0.102867	1.596057
1.81768	-0.69946	-0.92404	-0.23238	-0.38383	-0.81599	-0.65433	0.018839	0.691657
0.853605	0.37282	0.508245	0.594647	0.572987	0.969791	0.764762	0.057756	0.596971
0.853605	-1.04475	-1.40695	-1.4977	-0.93702	-1.2945	-1.03665	0.015786	0.560682
0.853605	-0.40928	-0.82513	-0.97916	-0.29775	-0.30229	-0.22227	0.144028	0.756895
0.853605	-0.69953	-1.23717	-1.42608	-0.61792	-0.67572	-0.60742	0.062592	0.818663
-0.23653	0.087119	0.425745	0.809093	0.307979	0.446051	0.409758	0.001516	0.721974
-0.23653	0.011637	0.055834	0.163234	0.252746	-0.1471	0.234687	0.273745	0.399847
-0.23653	1.59659	1.383883	0.943779	1.060494	0.986292	1.069193	0.008506	0.652811
1.968583	3.38796	2.709863	2.020058	1.767791	2.51193	1.848884	0.079226	1.620169
1.968583	-0.79449	-0.38327	-0.76474	-0.38229	-1.27148	-0.35135	0.071904	0.920133
1.968583	0.782777	0.775261	0.200189	0.002922	0.53635	0.417123	0.039983	0.779854
-0.06	-0.00386	0.361703	1.125749	0.389321	0.459241	0.260128	0.014822	1.129612
-0.06	0.774065	0.922054	0.973233	1.0835	1.33675	1.182937	0.051019	0.562685
-0.52426								
-0.52426	0.715029	1.243217	1.518843	1.111367	1.179865	0.920026	0.000381	0.803815

0.64695									
0.64695	0.13274	-0.04345	-0.19507	0.053992	-0.24468	0.073603	0.106435	0.377418
0.64695	0.836611	0.779811	0.585484	0.683966	1.122875	0.975501	0.016853	0.537391
0.147467								
0.147467	0.466183	0.910368	1.304587	1.360986	0.939736	0.532079	0.071511	0.894803
-0.33725								
-0.33725	0.019053	-0.09381	-0.22329	-0.3234	0.043502	0.011148	0.022035	0.3669
-0.33725	0.635995	0.436106	0.332082	0.537128	0.027128	0.199773	0.068135	0.608867
-1.5369	0.313289	0.586148	0.962995	0.489827	0.153474	0.236912	0.023547	0.809521
-0.6901								
-0.6901	1.6892	1.831666	1.542998	1.401154	1.347765	1.342744	0.031529	0.488921
-1.5369	-0.49775	-0.57206	-0.24569	-0.44882	-0.41384	-0.49048	0.128274	0.326366
-1.5369	-0.9895	-0.43093	-0.09331	-0.50974	-0.65924	-0.80402	0.016605	0.896192
-0.53042	0.129105	0.419563	0.801957	0.119346	0.098174	0.057623	0.000692	0.744334
-0.53042	-0.48242	-0.98994	-0.31136	-0.15209	-0.62864	-0.45539	0.00817	0.837851
-0.53042	0.813218	1.225898	1.375273	1.008455	1.415115	0.808733	0.019205	0.606382
-0.86295	1.55544	2.007663	1.98079	1.279647	1.803705	1.55013	0.053626	0.728017
0.828447								
0.828447	-1.05447	-1.33781	-1.30296	-1.17417	-1.26382	-1.01346	0.01923	0.324351
0.828447	-0.28153	0.040071	0.193754	-0.31243	-0.39835	-0.3222	0.037636	0.592107
-0.07099								
-0.07099	-0.5367	-0.83635	-0.89873	-0.49913	-0.51424	-0.50386	0.01914	0.399602
-0.07099	0.012025	-0.27858	-0.37179	-0.12758	-0.42167	-0.34483	0.056065	0.433698
-0.12321								
-0.15152	0.349512	0.840381	1.003638	0.477547	0.221213	0.289066	0.0024	0.782425
-0.12321	1.593773	1.783836	0.972605	1.38735	0.745956	0.41323	0.110013	1.370605
-0.29062								
-0.29062	-0.14987	0.568488	1.012427	0.443087	0.369653	0.139193	0.005995	1.162296
-0.29062	-0.80466	-1.23189	-1.45295	-0.84195	-1.17744	-0.9071	0.024003	0.648293
-0.29062	-0.12699	-0.45507	-0.7495	-0.78248	-0.5612	-0.11969	0.027485	0.662791
-0.01429	0.701163	0.844612	1.219828	0.626469	1.23529	1.000583	0.040114	0.608821
-0.01429	-0.37869	-0.61015	-0.73276	-0.53273	-0.64288	-0.40599	0.015517	0.354071
-0.01429	-0.47313	-0.08831	-0.6892	-0.23007	-0.86061	-0.14301	0.046033	0.772298
0.46174	1.135364	0.963004	0.879839	1.420258	0.266321	0.905395	0.102914	1.153937
0.809847								
0.46174								
0.809847	0.017674	-0.18946	-0.50015	-0.3675	-0.59999	-0.24424	0.006087	0.617662
0.809847	-0.02727	-0.29538	-0.52829	-0.31444	-0.09923	-0.01184	0.005112	0.516458
-0.78303								
-0.37157								
-0.37157	0.120144	0.333612	0.274104	-0.12488	0.60348	0.378294	0.008695	0.728359
-0.37157	-0.06217	0.268128	0.484244	0.149787	-0.00772	-0.04994	0.015049	0.546417
-0.78303	-0.05424	-0.41397	-0.27347	-0.13357	0.207932	-0.03072	0.008653	0.6219
-0.78303	1.113157	1.57603	1.68671	1.078157	1.448445	1.251373	0.002062	0.608553
0.043997								
0.043997	0.450127	0.412931	1.166624	1.19788	0.424757	0.840783	0.174614	0.78495
-1.05665	1.114329	1.086136	1.239576	0.823446	1.119115	0.957524	0.057704	0.416131

-1.05665	0.156506	0.266599	0.205383	0.332281	0.665269	0.568421	0.013419	0.508763
-1.05665	0.547251	1.053835	1.50367	1.097893	0.77978	0.635024	0.000471	0.956419
-0.81514								
-0.81514	0.053576	0.059245	0.13766	0.016973	0.142467	0.059181	0.300426	0.125494
-0.43502	1.193619	1.534453	1.268975	0.941228	1.150814	0.621218	0.006718	0.913235
-0.43502	0.813573	0.643728	0.767709	0.557916	0.688663	0.718985	0.021317	0.255657
0.07029	1.357033	1.870873	1.773857	1.091758	1.88674	1.75599	0.038607	0.794982
0.07029	0.040125	-0.34834	-1.28828	-0.70237	-0.78152	-0.99751	0.061943	1.328408
0.07029	0.043124	0.046239	-0.03514	0.130504	0.614353	0.423718	0.004539	0.649492
0.18245	0.428009	0.940757	1.367943	0.710131	0.862947	0.700106	0.00013	0.939934
-0.81649	0.602257	0.455736	0.535688	0.340857	0.618631	0.13055	0.223204	0.48808
-0.81649	0.342459	0.560587	0.968159	0.403394	0.261949	0.266093	0.013085	0.706211
-0.81649	0.870515	0.975376	1.041832	1.112445	1.747045	1.274417	0.011226	0.87653
0.18245	0.235866	0.38435	0.401665	0.153495	0.726124	0.409722	0.023422	0.572628
0.324443	0.258838	0.602188	0.892869	0.197308	0.192049	0.299994	0.000426	0.700821
0.09904								
0.09904	0.412718	0.601087	1.019357	1.049663	1.172495	0.88924	0.001273	0.759777
0.324443	2.383683	2.160285	2.154856	1.483869	1.523905	1.539302	0.067578	0.899815
	0.50754	0.615024	0.737135	0.840379	1.055145	0.890179	0.000382	0.547605
1.415928								
1.415928	-0.60577	-1.02291	-1.18293	-0.72407	-0.7771	-0.73678	1.88E-05	0.577163
1.415928	-1.12615	-1.37561	-1.61155	-0.77213	-1.38556	-1.05089	0.067999	0.839421
1.415928	-1.05563	-1.38866	-1.66354	-0.91218	-1.26038	-0.89014	0.008237	0.773401
1.415928	0.947717	1.291007	1.682133	1.198593	1.75745	1.435077	0.000352	0.809733
	0.220124	0.445786	0.608253	0.384022	0.787333	0.251461	0.000272	0.567209
-0.00257								
0.107143	-1.07957	-1.45367	-1.60884	-1.05948	-1.33795	-1.37507	0.0303	0.549366
0.107143	0.668006	0.912999	1.062499	0.836812	0.965153	0.83351	0.036598	0.394493
0.107143	0.80514	0.864516	1.258988	0.981559	1.143066	1.066983	0.015348	0.453848
-0.00257	0.773615	1.152108	1.531787	0.654019	1.22697	0.847394	0.002349	0.877767
-0.1158	0.773386	1.098072	1.30556	1.274807	1.7781	1.498403	0.050386	1.004714
0.239143	1.046907	1.334614	1.037085	0.375029	1.379985	1.196385	0.013309	1.004956
0.239143	-0.254	-0.45086	-0.45882	0.183041	0.168427	0.041355	0.000745	0.641859
0.239143	0.745162	1.079828	1.217333	1.222347	1.829665	1.364307	0.000776	1.084503
-0.60342	0.77727	1.246293	1.31643	0.863905	1.545515	1.278963	0.001169	0.768245
-0.60342	0.830035	0.985577	1.075094	0.890392	1.472985	1.065934	0.011153	0.64295
1.99152								
-0.52953	-0.03007	-0.12167	-0.0777	-0.45708	0.016114	-0.00476	0.006425	0.47319
1.99152	0.173679	0.170413	-0.26206	-0.37413	-0.53764	0.047779	0.012473	0.711318
-0.52953	-0.49307	-0.58778	-0.42862	-0.16068	0.144179	-0.18222	0.001184	0.731956
0.720517								
0.720517	-0.55221	-0.56729	-1.51058	-0.90434	-1.44553	-1.25352	0.000978	0.958371
0.229357								
0.229357	3.39811	3.28462	3.35873	2.613417	2.79193	2.537463	0.020414	0.860647
0.600923	0.880271	0.869118	1.214258	0.918439	0.997575	0.792687	0.014928	0.421571
0.600923	-0.65502	-0.92196	-0.70359	-0.12535	-0.54339	-0.68162	0.008141	0.796603
-0.09161	-0.60324	-0.76682	-0.75776	-0.41241	-0.69929	-0.62693	0.065103	0.354409

-0.09161	1.053861	1.128064	1.258306	1.116789	0.882272	1.004025	0.108976	0.376035
-0.14643								
-0.09161								
-0.14643	-0.98595	-1.47308	-1.19841	-0.88762	-1.31283	-0.98814	0.006141	0.585468
-0.14643	-0.96447	-1.33187	-1.36309	-0.88314	-1.23214	-1.13794	0.013107	0.479955
-1.25642	0.593836	1.148466	1.351513	0.703325	1.10282	0.842367	0.025986	0.757677
-0.46127	1.077234	0.739862	0.929432	0.824105	0.182771	0.256705	0.112507	0.894463
-0.46127	0.589949	0.984736	1.305673	0.950335	1.13019	0.854802	0.004448	0.715724
-0.46127	-0.00985	0.439299	0.421726	0.412502	0.37632	0.255663	0.055829	0.449152
-1.25642	-0.12333	-0.23541	-0.28343	-0.35057	-0.24259	-0.0955	0.007527	0.255068
0.36255								
0.36255	-0.57021	-0.66472	-0.85041	-0.73537	-0.62808	-0.55456	0.010691	0.295848
0.36255	0.566823	0.933424	0.978363	0.806059	1.47176	1.091792	0.017251	0.904937
-0.00189	1.132465	0.98965	0.5142	0.814503	0.587852	0.238111	0.04875	0.894354
-0.1047	-0.67692	-1.04994	-1.33543	-0.77597	-0.995	-0.8503	3.11E-06	0.658517
-0.1047	-0.93164	-1.45528	-1.29322	-0.66189	-0.93257	-0.78162	0.00181	0.793392
-0.1047	-1.06347	-1.07242	-0.95342	-0.6396	-0.85641	-0.95087	0.050927	0.432818
-0.00189	1.071123	1.442827	1.406837	0.979688	0.613356	0.797071	0.000879	0.829471
0.938093	-0.48889	-0.55336	-0.64155	-0.17087	-0.11635	-0.25178	0.103435	0.525195
0.860947	0.68027	1.008031	1.020965	0.770991	1.25257	1.001249	0.028708	0.5723
0.860947								
0.938093	1.156517	1.456573	1.697103	1.221951	1.95955	1.558807	0.01161	0.803033
	0.200306	0.464677	0.91724	0.26238	0.20631	0.232445	0.018475	0.716935
0.717183	-0.69817	-0.70625	-0.71951	-0.54245	-0.83616	-0.75624	0.201559	0.293712
-0.01864	0.659456	0.700902	0.851154	0.75423	1.048194	0.844326	0.024029	0.388738
-0.07558	0.537809	1.118657	1.270646	0.473428	0.304916	0.221835	0.043047	1.048811
-0.07558	0.717398	0.777035	0.766657	0.71455	1.139055	0.925904	0.022472	0.424505
-0.07558	0.993463	1.076758	1.148113	1.323513	1.387825	0.982777	0.083928	0.405048
-0.01864	0.195775	0.616908	0.570159	0.167247	0.548701	0.457943	0.083736	0.449661
-0.01864	0.451147	0.579965	0.821632	1.284943	1.0877	0.829504	0.00259	0.833796
0.260417	0.221676	0.365871	0.313218	0.278388	1.062663	0.545445	0.060163	0.840987
0.260417	0.309818	0.400703	0.336729	0.145261	1.00499	0.832212	0.038169	0.859729
0.003357								
0.003357	3.472593	2.1509	1.36478	1.814187	1.866785	1.653385	0.004313	2.107814
0.003357	-0.41038	-0.96079	-1.17518	-0.74258	-0.89963	-0.8726	0.014952	0.764802
1.753382								
0.480323	0.952533	0.961283	0.127424	0.210883	-0.45605	-0.105	0.179908	1.417338
0.480323	-0.80931	-1.04195	-1.02964	-0.73349	-0.70749	-0.57344	0.001891	0.468507
0.103777	0.151055	0.35028	0.622341	0.498026	0.839936	0.342875	0.028152	0.688881
0.480323								
1.753382	-0.06443	-0.23331	-0.58561	0.022784	0.533287	0.177051	0.043364	1.118898
1.753382	-0.95482	-1.52323	-1.70059	-1.05215	-1.24881	-1.01892	0.038409	0.745767
1.753382	-0.67383	-1.29048	-1.48446	-0.78027	-0.80194	-0.75981	0.00054	0.810634
1.753382	-0.14041	0.147857	-0.96413	-0.49487	-0.88696	-0.58856	0.098246	1.111982
0.103777	0.562591	0.879609	1.222704	1.221135	1.350625	0.788545	0.017907	0.788034
0.28395	0.296615	0.472203	1.139946	0.69326	0.798423	0.549171	0.003124	0.84333

0.28395	-0.93107	-1.03339	-1.53877	-0.93437	-0.99503	-0.96141	0.026563	0.607697
0.28395	-0.54264	-0.74269	-0.91124	-0.64396	-0.61114	-0.56063	0.066498	0.368601
-0.97474								
-0.97474	0.34195	1.19951	1.92816	0.73153	0.127078	-0.11774	0.003069	2.0459
0.072013								
0.072013	0.278829	0.533152	0.882075	0.424809	-0.08718	-0.13665	0.009399	1.01873
0.072013	-0.17111	0.466953	0.568796	-0.0044	-0.36182	-0.35472	0.001488	0.930616
0.089977	0.361291	0.773898	1.221846	0.476308	1.171764	0.806101	0.051543	0.860555
0.089977	0.249365	0.39884	0.746283	0.900155	0.92086	0.591518	0.003943	0.671495
-0.30279	0.155584	0.319605	0.564403	0.16631	0.631382	0.528253	0.011641	0.475798
-0.30279	0.842621	1.198363	1.520447	1.303793	0.848572	0.648593	0.009432	0.871854
-0.57652								
-0.57652	-0.27236	0.117019	1.321443	0.41304	-0.48102	-0.73288	0.000577	2.054319
1.83884								
1.83884	0.902077	1.078806	1.18593	0.762028	0.311586	0.331347	0.002761	0.874344
1.38887	0.321896	0.534542	0.604424	0.269771	0.580717	0.675499	0.045926	0.405728
1.38887								
2.248743	-0.27533	-1.09793	-1.43885	-0.55789	-1.18814	-0.79742	0.008474	1.163513
2.248743	-0.21056	-0.36346	-0.31051	-0.3762	-0.03418	-0.02987	0.078673	0.346325
2.248743								
2.248743	-0.14918	-0.29686	-0.65229	-0.57974	-0.14086	-0.20837	0.05154	0.511439
2.248743	-0.52005	-1.41034	-1.8156	-1.1273	-1.42001	-1.14718	0.039622	1.295556
2.248743	-1.24386	-1.76957	-1.83415	-1.28814	-1.53903	-1.15104	0.02824	0.683117
-0.52874	0.258841	0.973135	1.6689	0.751657	0.937542	0.39621	0.021533	1.410059
-0.56689	0.167129	0.703016	1.098851	0.595206	0.803897	0.33522	0.029175	0.931722
-0.56689	1.30612	1.893851	1.388527	1.813268	0.702036	0.867107	0.083226	1.191816
-0.52874	-0.18536	0.103163	0.37049	0.221417	0.041449	-0.06119	0.008044	0.555853
-0.03303								
-0.03303	-0.09552	0.677353	0.843445	0.313269	-0.19807	-0.26658	0.004848	1.110021
-0.03303	0.633957	1.108174	1.384961	0.822152	0.389672	0.346442	0.011291	1.038519
0.997657								
0.997657	0.404851	0.118974	-0.28822	0.117856	-0.30037	-0.15976	0.012474	0.705216
0.997657	-0.8496	-1.10466	-1.39648	-0.85464	-1.18409	-0.87087	0.04847	0.546886
-0.07319								
-0.07319	-0.17735	-0.28775	-0.79518	-0.19125	-0.81811	-0.67239	0.023947	0.640756
-0.07319	-0.40162	-0.61227	-0.6849	-0.30662	-0.55767	-0.5446	0.010988	0.378286
-0.1527								
-0.1527	0.404331	1.103698	1.320599	1.542136	-0.0141	0.681054	0.12378	1.556233
-0.1527	0.545753	0.706457	0.940412	0.58374	0.108151	0.212331	0.018086	0.832262
0.76095								
0.76095	-0.05314	0.002292	-0.53525	-0.3778	-0.78978	-0.72974	0.172451	0.792074
0.76095	-0.06114	-0.40043	-0.45893	-0.07224	-0.37692	-0.3349	0.021613	0.397786
0.530187								
0.53518								
0.53518	-0.40459	-0.80507	-1.08671	-0.75753	-1.00365	-0.81174	0.002329	0.682118
0.53518	0.588443	0.584938	0.163852	0.295801	0.504124	0.237558	0.10519	0.42459
0.549805	-0.84445	-1.17218	-1.25064	-0.83771	-0.93275	-0.79978	0.005579	0.450856

0.530187	-0.74632	-1.10975	-1.04684	-0.49131	-0.41882	-0.53177	0.009699	0.690929
0.530187	0.506947	0.60893	0.617264	0.644586	1.128165	0.89419	0.001045	0.621218
0.549805	-0.14616	-0.21297	-0.26341	-0.29049	-0.43076	-0.40525	0.055219	0.284603
0.549805	-1.01358	-1.28515	-1.43927	-1.07391	-1.22424	-1.06304	0.014	0.425683
0.549805	-0.80006	-1.00122	-1.25373	-1.19367	-1.13907	-0.98822	0.00395	0.45367
0.440283	1.150905	1.60666	1.74116	0.906375	1.45979	1.174187	0.004542	0.834785
1.395833	2.54058	2.142053	1.643766	1.714163	1.612767	1.469023	0.058857	1.071557
1.395833	-0.61901	-0.97557	-1.02046	-0.56996	-1.16856	-0.83611	0.006111	0.598596
1.395833	0.594297	0.872712	0.596019	-0.03911	0.484371	0.819636	0.003188	0.911825
-0.45742	0.828622	1.212277	1.65466	1.31209	1.294445	1.17823	0.000247	0.826038
-0.18157	-0.58876	-0.19345	0.228948	-0.05938	-0.41523	-0.59203	0.002717	0.820976
-0.18157	0.380316	0.515937	1.113597	0.845614	0.715739	0.622803	0.000476	0.73328
-0.18157	0.00429	0.070206	0.05298	0.31459	0.391663	0.306984	0.005913	0.387373
-0.45742	2.220637	2.4432	2.001853	1.97191	2.335345	1.77172	0.031102	0.67148
-0.38936								
-0.38936	0.713311	0.882287	0.860719	0.635682	0.754698	0.613115	0.039309	0.269172
-0.21225	-0.30509	0.283597	-0.63138	0.07095	-0.71827	-0.37609	0.150759	1.001863
-0.21225	-0.66086	-0.66849	-0.45538	-0.66362	-1.17215	-0.93045	0.034992	0.71677
0.57628	-0.04981	0.332369	0.705518	0.158289	0.611531	0.406538	0.002426	0.75533
-0.21225								
0.57628	-0.9385	-1.40257	-1.56138	-0.87228	-1.06285	-1.14464	0.001025	0.689092
0.57628	-0.66378	-1.19827	-1.29858	-0.68177	-0.75394	-0.72079	0.032945	0.634802
0.57628								
-0.73505	0.401391	0.592458	1.124629	0.512964	0.836099	0.863321	0.025875	0.723238
-0.73505	0.12475	0.41268	0.542395	0.195526	0.24823	0.013154	0.067541	0.529241
-0.42365								
-0.42365	0.012035	0.278362	0.380098	0.552306	0.691023	0.132681	0.159792	0.678988
-0.87165								
-0.87165	-0.15333	-1.01119	-1.57269	-1.08501	-1.25124	-1.15735	0.034023	1.419359
-0.87165	-0.38406	-0.28247	-0.03487	-0.28806	-0.35748	-0.43717	0.024891	0.402296
-0.37283								
-0.37283	-0.67688	-1.04342	-0.99026	-0.72322	-0.7917	-0.68351	0.031834	0.366543
-0.37283	-0.9764	-1.397	-1.73111	-0.87668	-1.2969	-1.11205	0.004124	0.854429
-0.37283	-1.66361	-2.00961	-1.75159	-2.21495	-2.62501	-2.75254	0.007557	1.08893
0.952243								
0.952243	1.41147	1.14938	0.980412	0.576919	0.86743	0.559439	0.083591	0.852031
-0.30965								
-0.30965	-0.03828	-0.6421	-0.75859	-0.22958	-0.37401	-0.42863	0.019057	0.720301
-0.30965	1.118895	1.735203	1.654543	1.233304	0.441134	0.846144	0.02981	1.29407
-0.0557								
-0.0557	-0.96723	-1.28653	-1.37481	-0.96478	-1.28408	-1.01402	0.068385	0.410026
-0.0557	0.895753	1.355313	1.82253	1.18145	1.06139	0.657458	0.000145	1.165072
2.0229	-1.26835	-1.95393	-2.00169	-1.41469	-1.68867	-1.49241	0.000286	0.733339
2.522143	0.050714	0.369253	0.233747	-0.24429	0.11871	0.248616	0.002832	0.613538
2.0229	0.311952	0.793632	0.524938	0.278725	0.609125	0.357709	0.053029	0.514907
0.46976	0.39005	1.002795	0.546293	0.787853	0.785004	0.327942	0.011277	0.674853
-0.58631	0.400346	0.484779	0.146671	-0.03833	0.31698	0.520401	0.011424	0.558729

-0.58631	2.10242	1.277463	1.090481	0.760951	1.032295	0.715385	0.015156	1.387035
-0.58631	0.59127	1.098133	1.069112	0.733396	0.979535	0.905486	0.003443	0.506863
0.46976	0.665728	0.892415	0.813762	0.615127	1.13116	0.844891	0.007761	0.516033
-1.02754	0.575392	0.369024	0.594077	0.443659	0.644254	0.563048	0.082827	0.27523
-0.17468	0.82499	0.581902	1.007385	0.6015	0.905011	0.940704	0.071565	0.425483
-0.17468	0.455015	0.261806	-0.35631	-0.26482	-0.33855	-0.27488	0.114076	0.811327
-0.17468	-0.20422	-0.18599	-0.0955	-0.02982	0.214168	0.131251	0.013073	0.418392
-1.02754	0.281276	0.599332	0.569981	-0.12156	0.516302	0.307013	0.053912	0.720891
-1.00826								
-1.00826	0.69551	1.1075	1.43961	0.693406	0.852095	0.702844	0.002592	0.746204
-0.17798								
-0.17798	0.269534	0.070427	0.151675	0.485981	0.493711	0.373411	0.027695	0.423284
-0.16067	1.265467	1.488497	1.714157	1.030888	2.06574	1.752727	0.022694	1.034852
-0.16067	0.663879	0.961486	0.989696	0.646749	0.503053	0.685981	0.132443	0.486643
-0.16067	-1.06193	-1.7144	-1.80152	-1.35301	-1.51849	-1.2637	0.009342	0.739591
-0.16067	-1.42734	-1.84843	-2.06326	-1.24834	-1.57547	-1.27424	0.036876	0.814927
-0.16067	-0.70838	-1.19151	-1.26512	-0.9391	-0.75054	-0.75864	0.061287	0.556742
-0.5385								
-0.5385	-0.93247	-1.01569	-1.15436	-1.18825	-1.58092	-1.0811	0.02879	0.648454
-0.5385	0.662675	1.219833	1.34922	0.648567	1.20132	0.879498	0.01011	0.700653
-0.89357								
-0.89357	0.008548	0.171627	-0.31264	-0.19573	-0.47961	-0.41626	0.127524	0.651232
-0.89357	-0.19004	-0.01385	0.252245	-0.04207	-0.50181	-0.31344	0.021714	0.754055
1.367907	0.638728	0.973521	1.3889	0.522824	1.138291	1.2062	0.000631	0.866076
1.367907	0.85437	0.13453	0.308883	0.164064	0.132275	0.09809	0.070451	0.75628
-0.29718	6.81563	6.81567	5.61264	5.894603	5.95747	5.403473	0.02676	1.412197
-0.29718	0.319913	0.861204	0.835418	0.456966	0.45906	0.505407	0.033732	0.541291
-0.12609	1.023622	0.561149	0.772719	0.665079	0.728861	0.814391	0.152298	0.462473
-0.12609	0.151815	0.52364	0.677405	0.473984	0.377926	0.221169	0.026054	0.52559
-0.12609	1.875563	1.68678	0.886119	1.426406	0.949232	0.803429	0.046258	1.072135
-0.12609	0.236602	0.419499	0.965911	0.185084	0.303351	0.292842	0.063939	0.780827
-0.36145								
-0.36145	-0.10588	-0.32627	-0.03765	0.303119	0.574228	0.155884	0.023058	0.9005
-0.36145	1.416095	1.764193	1.864627	1.228937	1.69707	1.389299	0.069723	0.635689
-1.40414								
-1.40414	0.257054	0.29911	0.654075	0.976236	0.025908	0.149247	0.066538	0.950328
0.313937								
0.313937	1.402673	1.831173	1.88022	1.027765	1.664405	1.56087	0.001933	0.852455
0.33924	4.106047	4.387817	3.807081	3.614023	4.042535	3.18478	0.052808	1.203037
0.33924	-0.12428	-0.26489	-0.28938	-0.42588	-0.31382	-0.15254	0.122809	0.301594
0.33924	0.605388	0.543755	1.09176	1.142053	1.120325	0.925539	0.002287	0.598298
-0.05921								
-0.05921	-0.14844	-0.05645	-0.19069	0.015842	-0.15757	0.061349	0.224646	0.252038
-0.05921	-0.28783	-0.11728	-0.20181	-0.3862	-0.86738	-0.74128	0.117282	0.750102
-0.39021	0.141981	0.134146	0.010885	-0.34319	-0.07783	0.224375	0.03511	0.567564
-0.39021	-1.17359	-1.5199	-1.81782	-1.35578	-1.62606	-1.3297	0.001842	0.644225
-0.39021	-0.39161	-0.33513	-0.40326	-0.45379	-0.47201	-0.4067	0.127655	0.136874

-0.14089	2.309683	2.615323	1.217377	1.453997	1.430251	0.766287	0.067992	1.849036	
-0.14089									
-0.14089	-0.89422	-1.53208	-1.59425	-0.92023	-0.99804	-0.85062	0.024337	0.743632	
-0.14089	0.308359	0.077665	-0.89248	-0.30454	0.292807	-0.40126	0.014238	1.200834	
-0.14089	-0.82335	-1.18078	-1.16334	-0.57968	-0.81658	-0.60506	0.010131	0.601097	
-0.07119	0.217007	0.583647	0.85523	0.326525	0.846487	0.844775	0.010985	0.638223	
-0.29457	0.327962	0.003642	0.401203	0.147864	0.22132	0.428105	0.115418	0.424463	
-0.29457	0.1973	-0.60899	-0.95461	-0.14554	-0.22726	-0.35281	0.044382	1.151908	
-0.29457	-0.15796	0.099719	0.34919	0.018528	-0.06055	-0.00899	0.027048	0.507148	
-0.13279	-0.16503	0.11816	0.097656	0.305879	0.217936	0.057029	0.026501	0.470914	
-0.07119	0.176343	0.187977	0.232486	0.504979	0.561948	0.469236	0.095841	0.385605	
-0.13279	0.294762	0.410795	0.359977	0.165367	0.449127	0.506299	0.051144	0.340932	
0.50679	1.57396	1.333867	1.3647	1.180531	1.514015	1.27267	0.117405	0.393429	
0.50679	1.018324	1.230426	1.056476	1.341542	1.11479	1.279446	0.266124	0.323218	
0.514833	0.565548	0.336966	0.341394	0.289422	0.19558	0.269174	0.162259	0.369968	
0.514833	-0.68342	-0.88308	-0.66076	0.036598	0.057634	-0.2663	0.015271	0.940718	
0.514833	-0.58506	-0.81745	-0.67399	-0.06099	0.015652	-0.09519	0.030618	0.833103	
0.514833	0.942683	0.626963	0.122816	0.256421	0.57754	1.170566	0.044886	1.04775	
0.181378									
0.181378	0.772207	1.304703	1.28063	0.717616	0.969596	0.862476	0.040291	0.587088	
1.05954	0.003574	-0.07217	-0.04023	0.161688	0.478753	0.47451	0.018462	0.550922	
1.05954	-0.29073	0.378341	0.491833	0.356727	0.558052	0.366275	0.02036	0.848785	
1.05954	-0.8482	-1.51896	-1.4245	-0.65818	-0.68372	-0.72833	0.003653	0.860778	
1.05954	-1.40387	-1.80815	-1.97453	-1.32599	-1.53763	-1.23393	0.003826	0.740607	
0.961588	0.811916	0.895653	0.860098	0.628152	0.944058	0.716729	0.033156	0.315906	
	0.206839	0.637109	-0.54192	0.208826	-0.40713	-0.30799	0.096639	1.17903	
	1.946667	1.602087	0.848587	1.042447	1.264086	1.031274	0.021419	1.098079	
0.961588	-0.50961	-0.60528	-0.63187	-0.63906	-0.39986	-0.56547	0.12582	0.2392	
-0.28265	2.043757	1.86698	1.384622	1.407452	2.033861	1.617878	0.112309	0.659135	
-0.28265	-0.83864	-1.14379	-1.37733	-0.9792	-1.22292	-1.26574	0.002025	0.538689	
-0.28265	1.62284	1.50435	0.920058	1.400883	1.73711	1.38826	0.013156	0.817052	
-0.11157									
-0.11157	2.57045	3.439323	2.44858	2.65315	0.904829	2.494027	0.039379	2.534494	
-0.25111									
-0.25111	-0.54197	-0.61343	-0.13618	-0.33561	-0.34079	-0.42825	0.044599	0.477252	
-0.25111	0.465437	0.699717	1.516867	1.108841	0.817095	0.551131	0.016558	1.051429	
0.234483									
0.234483	-0.91252	-1.88391	-1.96943	-1.53614	-1.91671	-1.6746	0.004119	1.056914	
0.234483	-0.895	-1.18941	-1.45047	-0.97778	-1.31842	-1.13374	0.011909	0.555472	
0.234483	-0.85407	-1.33099	-1.67874	-0.93464	-1.3211	-1.01458	0.003899	0.824669	
0.234483	-0.84523	-1.00429	-0.8745	-0.27137	-1.80608	-0.9928	0.114881	1.534706	
0.885333	-0.39898	-0.84918	-1.17366	-0.80787	-1.1965	-0.8394	0.014139	0.797511	
0.885333	0.585917	0.716492	0.684797	0.187198	0.797007	0.686381	0.024077	0.609809	
0.885333	0.562643	0.649842	0.671021	0.311586	0.810627	0.666869	0.095055	0.499041	
-0.66799	0.027288	-0.01028	-0.21224	-0.19853	0.379812	0.183626	0.017685	0.59205	
-0.66799	-0.06333	-0.24586	-0.1575	0.071173	0.289725	0.162944	0.039797	0.535584	

-0.66799	-0.30569	0.125787	0.658656	0.424314	-0.13735	-0.08945	0.010237	0.964349
0.306053	0.048788	0.235686	0.506618	0.249332	0.105989	-0.01978	0.02378	0.526401
0.306053	0.007177	0.363368	0.257975	0.49996	0.276202	0.663489	0.023303	0.656312
-0.42123								
0.306053	0.645244	0.952822	0.884903	0.318222	1.16033	0.922375	0.027156	0.842108
-0.42123	0.073718	0.292054	0.159301	-0.19465	-0.007	-0.06819	0.016082	0.4867
0.2535								
0.2535	0.476545	0.841058	1.326819	0.950404	1.011118	0.555044	0.026176	0.850274
0.690463	1.032086	1.126815	1.212436	0.51192	1.348795	1.080297	0.052635	0.836875
-0.61052								
-0.61052	-0.41927	-0.54633	-0.82171	-0.86894	-0.54731	-0.3719	0.07004	0.497048
-0.61052	0.884936	1.101157	1.465788	1.072964	1.380145	1.14994	0.089119	0.580852
0.690463	-0.92799	-1.18793	-1.33509	-1.00938	-0.78508	-0.71628	0.024045	0.618807
0.690463	3.660367	3.186293	3.173103	3.888513	4.193525	3.9787	0.250334	1.020422
0.690463	0.329573	0.360699	0.370679	0.818555	0.782982	0.513072	0.00717	0.488983
0.81596	-1.42385	-1.90719	-2.09306	-1.36736	-1.78482	-1.42569	0.001408	0.725697
0.658537	0.332272	0.377408	0.456935	0.196892	0.705345	0.560796	0.066729	0.508454
0.311102	0.022421	0.476167	0.616223	0.378981	0.838143	0.314422	0.001613	0.815722
0.81596	-1.2427	-1.33873	-1.35186	-0.98771	-1.52677	-1.23167	0.047753	0.53906
0.81596	-1.18928	-1.93898	-2.09457	-1.6645	-1.68703	-1.68059	0.006742	0.905297
0.319923	-1.0992	-1.51934	-1.64591	-1.15103	-1.48822	-1.2019	0.018537	0.546707
0.658537	1.459883	0.873073	0.12872	1.326841	0.6247	0.694873	0.020297	1.331163
0.658537	0.066846	0.16454	-0.28558	-0.05049	-0.17892	-0.08827	0.008195	0.450117
1.170363								
1.170363	0.627346	0.756383	0.879101	0.774938	1.40747	1.011219	0.00278	0.780124
0.222832	0.44054	0.784899	1.195533	0.493742	1.072585	0.976419	0.009107	0.754992
0.222832	1.175539	0.635787	0.61107	0.476534	0.780197	1.385602	0.240688	0.909069
0.222832	-1.14582	-1.40395	-1.39534	-0.99417	-0.96313	-0.93891	0.014979	0.465046
0.222832	-0.90431	-1.28904	-1.34706	-0.87095	-1.08043	-0.72511	0.001004	0.621951
-0.23419								
-0.23419	-0.66462	-1.01127	-1.30825	-0.72738	-0.11484	-0.25274	0.012463	1.193414
-0.23419	-0.49625	-0.93709	-1.19605	-0.50057	-0.74279	-0.58657	0.030395	0.699801
-0.23419	-1.0354	-1.67082	-1.80089	-1.05702	-1.31598	-1.06101	0.011933	0.765495
-0.23419	-0.36879	-1.20108	-1.73618	-1.12359	-0.81476	-1.1795	0.060277	1.367385
-0.23419	-0.82997	-0.61269	-0.97592	-0.22512	-1.29255	-0.51873	0.184281	1.067433
-0.23419	3.067757	2.671963	1.850563	2.098063	2.059385	2.06611	0.005734	1.217193
-0.23419	-1.28276	-1.4435	-1.88106	-1.08925	-1.38435	-1.17275	0.042135	0.791804
-0.23419	0.311696	-0.80429	-1.52101	-0.9552	-0.85228	-0.5409	0.02797	1.832703
0.422392								
0.422392	-0.5891	-0.66746	-0.28274	0.008273	-0.33303	-0.31198	0.014364	0.675733
0.422392	-0.48489	-1.56968	-1.86028	-1.19047	-1.58717	-1.29802	0.03188	1.375382
0.422392	-0.97088	-1.44564	-1.67127	-1.15203	-1.4424	-1.24507	0.001026	0.700389
0.422392	-0.92128	-1.29083	-1.40933	-1.06652	-1.18094	-0.98169	0.015614	0.488051
0.608155	0.169017	0.700529	0.709276	0.049934	0.371994	0.64504	0.063274	0.659342
0.608155	-0.93122	-1.11875	-1.17067	-0.84917	-0.91639	-0.80652	0.107518	0.364159
0.608155	-0.42252	-1.03262	-1.25935	-0.44424	-0.68921	-0.50804	0.00514	0.836826
0.608155	-0.87027	-1.38824	-1.71519	-0.72268	-0.86927	-0.90406	0.000787	0.992509

0.608155	-0.26286	-0.70545	-0.61148	-0.19097	-0.24891	-0.21604	0.021725	0.514484
0.161417	0.405248	0.197498	0.072305	-0.00296	0.34813	0.678136	0.001029	0.681101
1.15722								
1.15722	-0.31012	-0.69283	-0.87349	-0.49616	-0.53144	-0.48816	0.023472	0.563364
1.15722	-0.58246	-0.97402	-1.41494	-0.97847	-0.64196	-0.68951	0.011595	0.832481
0.161417	-0.38602	-0.30647	0.180463	-0.0189	-0.08314	-0.07724	0.002381	0.566481
0.161417	0.417485	0.754156	1.480747	0.947853	0.807528	0.570576	0.009405	1.063262
-0.96219								
-0.96219	-0.05189	-0.73619	-0.8747	-0.40109	-0.65383	-0.44162	0.011004	0.822806
-0.96219	0.533042	0.400847	0.555355	0.767525	1.067587	0.440194	0.233997	0.666739
-0.25595								
-0.25595	-0.6851	-1.20462	-1.35157	-0.91393	-1.27755	-1.14011	0.013324	0.666475
-0.25595	-0.82546	-1.38891	-1.46047	-0.61148	-0.6817	-0.72785	0.005821	0.848997
-0.28957	-0.43176	-0.64578	-0.55289	-0.09109	-0.04878	-0.14474	0.0038	0.596999
-0.62404	1.622906	1.788528	1.387134	1.314781	1.885339	1.419061	0.159045	0.570558
-0.62404	-0.37246	-0.73227	-1.07209	-0.64275	-0.59473	-0.61526	0.007292	0.699627
-0.62404	-0.87401	-1.22527	-1.10007	-1.08277	-1.28698	-1.26036	0.170955	0.412969
-0.28957	0.56447	0.722413	0.942941	0.809507	0.882573	0.771108	0.067151	0.378471
0.634163	0.008307	0.337605	0.157808	-0.07957	0.417296	0.431924	0.070527	0.511489
0.634163	0.360392	1.094692	1.37726	0.748606	0.007282	0.465816	0.060285	1.369978
0.634163	1.730583	1.89229	1.607433	1.78193	2.17465	1.881813	0.021356	0.567217
-0.93342	0.179644	0.154574	0.685104	-0.07992	0.623356	0.438327	0.059432	0.765022
-0.93342	1.41034	1.57646	1.479637	1.19649	1.072565	1.153556	0.000885	0.503895
-0.25295	0.73869	1.017147	1.132392	0.590592	1.138821	1.072439	0.055798	0.548229
-0.25295	-0.42873	-0.28718	-0.46028	-0.32642	-0.05894	-0.38902	0.065342	0.401336
-0.25295	0.499136	0.606486	0.842818	0.70977	0.826609	0.512459	0.054258	0.343682
-0.37313								
-0.37313	-0.87198	-0.81944	-1.04456	-0.2737	-1.17199	-0.6198	0.227274	0.89829
-0.37313	-0.72693	-1.44393	-1.55448	-0.84927	-0.92833	-1.01595	0.010982	0.827555
-0.37313	2.532417	2.067167	1.438157	1.011123	2.36762	1.159997	0.031516	1.521294
0.818087								
0.818087	-0.47671	-0.06217	0.656947	0.381676	0.084395	-0.10904	0.000376	1.13366
0.818087	0.531859	1.070679	0.248798	1.149819	0.145155	0.156465	0.063702	1.004664
-0.42105								
-0.42105	-0.06049	0.019334	0.146495	0.49096	0.531296	0.03439	0.103627	0.591782
-0.42105	0.205765	0.172124	-0.04162	0.020346	0.415197	0.522915	0.043	0.564531
-0.5096	0.800932	0.925773	1.346195	0.893969	1.163762	0.897945	0.06798	0.545264
-0.10873	0.661721	0.452101	0.497138	0.533571	1.15628	1.084616	0.0527	0.704179
-0.5096	0.79928	0.511212	0.251706	0.451825	1.333315	1.024647	0.02005	1.081609
-0.10873	-0.60727	-0.96935	-1.02522	-0.76359	-0.47535	-0.43763	0.04202	0.587589
-0.10873	0.010425	-0.25261	-0.75483	-0.49761	0.454715	0.325898	0.001961	1.20954
1.0923								
1.0923	-0.56121	-0.24745	-0.65323	-0.39413	-0.50572	-0.58122	0.141378	0.405788
1.0923	0.747702	1.288113	1.471947	0.703444	0.999763	0.996871	9.45E-05	0.768503
-0.67841								
-0.67841	1.35193	1.68207	1.737163	1.333397	1.347955	1.397993	0.033295	0.403767
-0.71728								

-0.71728	0.654667	1.294727	1.658337	0.575176	1.099695	0.929987	0.001366	1.083161
-1.18854								
-1.18854	1.102197	1.60044	1.658667	1.035504	1.39415	0.9695	0.099931	0.689167
0.238493	0.842237	1.069369	1.231906	0.982485	1.51228	1.215087	0.062957	0.670043
	0.396272	0.59575	0.514047	0.510372	0.814384	0.598071	0.005915	0.418112
	3.30901	3.117567	2.565373	2.405597	3.11291	2.228297	0.024085	1.080713
0.238493	-0.55083	-0.52534	-0.45064	-0.467	-0.05515	-0.10931	0.024115	0.495679
0.238493	-0.50415	-0.76639	-0.60911	-0.27955	0.029385	-0.10547	0.011395	0.795775
-0.7484								
-1.14859	0.992895	1.280914	1.27703	1.068621	1.588155	1.25668	0.01489	0.59526
-1.14859	5.724873	5.82005	4.768607	4.638123	4.90442	4.39	0.016852	1.43005
-0.7484	-1.0173	-1.65141	-1.67066	-1.13298	-1.30397	-1.10542	0.006948	0.65336
-0.7484	-0.8411	-1.38426	-1.65186	-0.95005	-1.12894	-0.84781	0.013293	0.810764
-0.7484	0.607983	1.114074	0.944149	0.553013	1.046875	0.737243	0.017508	0.561061
-1.05841	-0.05466	0.011866	0.032329	-0.13029	-0.18133	-0.01829	0.104609	0.213658
-1.05841	0.49254	1.431152	0.451201	0.071476	0.335036	0.306939	0.094792	1.359676
-1.05841	0.441781	0.530118	0.598051	0.256409	0.563158	0.420688	0.160812	0.341642
-0.94575								
-0.94575	1.154067	1.76579	1.720063	1.18752	1.07727	1.254014	0.026525	0.68852
2.702797	0.86777	0.999314	1.260936	0.402718	0.699933	0.663797	0.032687	0.858219
2.702797	-0.47563	-1.14977	-1.20682	-0.67003	-0.60969	-0.77366	0.033458	0.731197
2.702797	0.57595	0.851861	0.096884	0.112037	0.314467	0.369699	0.044831	0.754977
3.363493	0.514986	1.10846	1.476717	0.584927	0.529889	0.520713	0.003456	0.96173
3.363493	0.976038	1.09855	0.962254	0.550781	1.2248	0.990442	0.012602	0.674019
3.363493	0.261421	0.582768	1.162087	1.04685	0.456727	0.27564	0.00435	0.900666
-0.00117	0.632761	0.787001	0.972219	0.365091	0.732317	0.953915	0.006951	0.607128
-0.00117	1.269072	1.666933	1.82331	1.11316	1.83176	1.470003	0.028409	0.7186
-0.84506								
-0.84506	0.202743	0.370041	-0.62101	0.12346	-0.35815	-0.38127	0.096631	0.991056
-0.84506	1.29448	1.90903	2.349823	1.676043	1.671495	1.195551	0.026746	1.154272
-0.0971								
-0.0971	-0.25545	-0.56997	-0.8479	-0.58149	-0.59002	-0.35729	0.001179	0.592452
-0.0971	-0.99172	-1.59593	-1.68012	-1.30652	-1.68674	-1.56058	0.024787	0.69502
-1.32756								
-1.32756	0.752091	1.192387	1.25245	0.795082	0.675923	0.552317	0.005687	0.700133
0.032353								
0.032353	-0.18893	-1.03237	-1.29039	-0.87532	-1.53476	-1.15212	0.011446	1.345826
0.032353	0.025269	-0.30845	0.521849	0.475261	0.326627	0.146357	0.002186	0.830296
-0.93423	-0.76446	-0.79359	-0.77398	-1.02985	-0.85194	-0.99984	0.143022	0.265387
-0.93423	0.623364	1.328982	1.671007	1.006239	0.928893	0.675137	0.00306	1.047643
-0.44727								
-0.44727	0.446114	1.029243	0.872705	0.459281	0.244002	0.313999	0.001096	0.785241
-0.44727	-1.05427	-1.17534	-1.20552	-0.7165	-0.92018	-0.97374	0.001752	0.489016
-0.44727	-0.195	-0.09044	0.015301	-0.18469	-0.45629	-0.38139	0.036843	0.47159
0.008043	0.47565	0.81113	1.105712	0.748266	0.942371	0.592351	0.066124	0.630062
0.008043	-0.10461	-0.23928	-0.82581	-0.39746	-0.40338	-0.35231	0.039844	0.721197
0.008043	-0.26579	-0.45541	0.106504	0.304962	-0.06783	-0.26345	0.005285	0.76037

3.37489	0.851328	1.211563	1.209131	0.462561	-0.0548	0.390856	0.023783	1.266367
3.45307								
3.37489	-0.47785	-0.3126	-0.71027	-0.14975	-1.05493	-0.30017	0.257359	0.905185
3.37489	-0.1185	-0.04551	-0.06622	0.010207	0.372471	0.222153	0.023363	0.490968
3.45307	0.183825	-0.16337	-0.36157	-0.02539	0.507295	0.320928	0.06689	0.868864
-0.28748								
-0.28748	-0.70369	-1.02661	-1.00666	-0.64788	-0.49114	-0.49051	0.023383	0.536097
-0.28748	-0.82818	-1.30957	-1.59589	-0.82271	-0.95609	-0.75958	0.028461	0.836307
-0.56989								
-0.56989	0.67175	1.046146	1.604267	1.345013	1.282735	0.849098	0.001577	0.932517
-0.47734								
-0.47734	0.512157	0.961993	1.104631	0.35569	0.05297	0.23467	0.004821	1.051661
0.30199								
0.30199	1.052576	1.090651	1.147751	1.28421	1.35243	0.950526	0.19212	0.401904
	-0.36018	-0.41435	-0.35802	-0.41065	-0.31741	-0.34061	0.198266	0.096943
-0.68118	0.147428	0.396419	0.255477	0.044601	-0.2201	0.049318	0.037213	0.616519
-0.91445	-0.31248	-0.07457	0.218613	-0.06365	-0.22787	-0.32074	0.037967	0.539352
0.62323	0.354939	0.637135	0.812524	0.578726	0.71091	0.893798	0.001592	0.538859
-0.91445	0.302125	0.519718	0.660038	0.489039	0.2101	0.250412	0.103755	0.449938
-0.68118								
0.62323	-0.57234	-0.53638	-0.37857	-0.78096	-0.83006	-0.77213	0.076533	0.451483
0.62323	-0.0059	0.063167	0.042335	-0.07236	0.67247	0.318692	0.027363	0.744831
1.46344	0.366974	0.281353	0.570758	0.147894	0.211416	0.321184	0.058211	0.422864
1.46344	1.060975	1.41038	1.45371	0.961292	0.984637	0.942819	0.003547	0.510891
-0.1166	0.630215	0.279634	-0.47562	-0.47786	-0.18866	-0.32567	0.053514	1.108072
-0.1166	-1.21162	-1.53002	-1.4225	-1.02312	-1.29693	-0.79882	0.000605	0.731208
-0.1166	-1.35968	-1.87012	-1.90572	-1.51752	-1.6593	-1.35134	0.034736	0.55438
-0.52897								
-0.52897	-0.84174	-1.36277	-1.27135	-1.21853	-1.3223	-1.19875	0.111365	0.521028
-0.52897	1.476683	1.895037	2.10414	1.547083	2.03798	1.640187	0.078912	0.627457
-0.52897	0.882121	1.24912	1.66641	1.092034	1.15322	0.906891	0.000299	0.784289
1.275817	0.187261	0.441892	0.765507	0.366448	0.764636	0.647102	0.022579	0.578246
1.275817	-0.15437	-0.07838	0.103803	0.139058	0.05251	-0.04964	0.006824	0.293429
1.275817	-0.19985	-0.38337	-0.40388	-0.16974	0.043911	0.115516	0.052468	0.519392
0.061318	0.62464	0.792113	0.931591	0.656385	0.397944	0.52774	0.072993	0.533647
0.061318	-0.35827	-0.05793	0.133549	-0.27236	-0.31125	-0.22294	0.016409	0.491818
0.061318	0.286582	-0.04754	-0.55155	-0.29408	-0.57844	-0.49467	0.022064	0.865027
0.061318	-1.05704	-1.61378	-2.01503	-1.23965	-1.62884	-1.69581	0.00013	0.957991
0.224733								
-0.6239								
-0.6239	-0.2774	-0.36892	0.007301	0.157826	0.113625	-0.08639	0.000277	0.526748
0.224733	1.292737	1.800903	1.75372	1.160843	1.51529	1.34581	0.011189	0.64006
-0.63608	-1.07921	-1.58956	-1.59987	-0.85557	-1.1478	-1.09542	0.006173	0.744292
-0.63608	-0.65368	-1.38566	-1.18199	-0.5898	-0.91601	-0.64563	0.001649	0.795863
-0.63608	-0.40128	-0.20194	0.004342	-0.12852	-0.40709	-0.09722	0.122608	0.411435
-0.94208								

-0.59607	2.214597	2.099623	1.314466	1.465462	1.573185	1.361785	0.058349	0.900131	
-0.63608									
-0.59607	-1.19924	-1.06445	-1.2193	-0.93047	-1.21153	-1.12017	0.073212	0.288833	
-0.94208	0.204845	0.433767	-0.82238	-0.24231	-0.52019	-0.71817	0.096764	1.256145	
-0.94208	0.33296	0.674497	0.8295	0.230922	0.39481	0.307632	0.018416	0.598579	
0.038618	0.244406	0.536759	0.565855	0.297018	0.637017	0.666543	0.018685	0.422137	
0.038618	1.160049	0.059202	-0.8446	-0.51373	0.428531	-0.08521	0.057248	2.004645	
0.038618	-0.43665	-0.60805	-0.51287	0.012849	-0.1268	-0.10549	0.008696	0.620897	
0.758177	0.297683	0.566522	0.440909	0.121647	0.687183	0.69958	0.032008	0.577933	
0.758177	0.233741	1.106036	0.066274	0.138854	-0.58537	-0.4066	0.057875	1.691402	
0.758177	0.587549	0.153456	-0.19166	0.411351	0.215603	0.382027	0.006367	0.779205	
1.63939									
1.63939	0.408044	1.211326	1.655253	1.11856	1.37102	0.838243	0.000299	1.247209	
1.63939	0.272652	0.19413	-0.01247	-0.15548	0.8189	0.645605	0.005258	0.974382	
	0.413554	0.86049	0.560515	0.070745	0.357515	0.243755	0.090237	0.789745	
-0.57836									
-0.57836	2.433357	2.158873	2.526197	2.354143	2.35596	2.424803	0.147393	0.367323	
0.154213									
0.154213	-0.15354	-0.26424	0.13239	0.176263	0.120183	0.046404	0.001203	0.440503	
0.154213	1.453763	1.67905	2.00441	1.463193	1.291285	1.0212	0.018133	0.98321	
0.008073									
0.008073	0.722846	0.888249	0.721728	0.355969	0.87589	0.707157	0.005665	0.53228	
0.008073	3.26485	2.672533	2.43649	2.560083	2.72715	2.391233	0.058743	0.873617	
2.136737	0.576003	0.702532	0.93227	0.727326	0.460741	0.801322	0.116018	0.471529	
2.136737	-0.44381	-1.14439	-1.16675	-0.71366	-0.83342	-0.63318	0.010181	0.722931	
2.136737	-0.86709	-1.77553	-1.89314	-1.21686	-1.34593	-1.29288	0.002401	1.02605	
2.136737	-0.82046	-1.06834	-0.9043	-1.03572	-0.84572	-0.67433	0.050947	0.394012	
-1.23252	-0.11124	-0.39643	-0.51145	-0.23568	0.049247	0.108409	0.027788	0.619856	
-1.23252	-0.95628	-1.00062	-0.70332	-0.51907	-1.0443	-0.81257	0.022846	0.525233	
0.72607	0.421789	0.857554	0.887402	0.232417	0.944182	0.8498	0.050607	0.711765	
-0.41234	0.248814	0.670065	1.17513	0.678523	0.667725	0.391518	0.012896	0.926316	
-1.23252	0.162906	0.377438	0.649777	0.202385	0.45589	0.34214	0.02052	0.486871	
-0.41234	-0.51377	-0.53405	-0.70664	-0.69957	-0.72822	-0.66161	0.18336	0.214455	
-0.41234	-0.07937	-0.20624	-0.12273	0.28705	-0.34463	-0.26283	0.185522	0.631675	
0.72607	-0.28235	-0.57046	-0.74783	-0.33687	-0.35864	-0.21609	0.107056	0.53174	
0.72607	-1.00555	-1.18971	-1.49332	-1.14693	-1.31423	-1.05413	0.0017	0.48777	
0.169877									
0.169877	-0.07889	-0.09941	-0.24703	-0.18362	0.129472	0.153611	0.031755	0.400646	
-0.68583	0.338848	0.506817	0.831602	0.26716	0.610805	0.534445	0.010992	0.564442	
-0.68583	0.45744	1.325443	1.639587	0.732156	0.827063	0.624702	0.001821	1.182147	
-0.48512									
-0.48512	3.17109	2.980867	2.039523	2.541607	2.389105	2.552123	0.013734	1.131567	
-0.48512	0.184691	0.284943	1.186023	0.778098	0.961543	0.556798	0.001178	1.001332	
-0.30251	-0.38913	-0.66217	-1.13886	-0.49064	-0.58445	-0.45946	0.004519	0.749728	
-0.30251	0.849199	1.15667	1.548947	1.063493	1.451215	1.090984	0.006132	0.699747	
-0.30251	1.72485	2.031157	2.286457	1.51472	2.36565	2.00619	0.013669	0.85093	
-0.84303	1.667847	1.971763	2.05749	1.45722	2.23067	2.018647	0.018362	0.77345	

-1.26679	0.504984	0.540372	-0.15224	-0.14884	0.45434	0.617192	0.001198	0.769429
-0.84303								
-1.26679	0.059981	0.34394	0.720612	0.4793	-0.01926	-0.09788	0.001783	0.818489
0.00089								
0.00089	-0.00338	-0.08211	-0.13003	0.080935	0.3319	0.166261	0.003557	0.461932
0.00089	0.707286	0.856961	1.587517	1.175148	1.4568	0.625163	0.046539	0.962353
0.502747	0.181673	0.270831	0.270698	0.351286	0.513199	0.338653	0.053108	0.331525
0.502747	0.895724	1.308143	1.421487	0.874495	1.38685	0.802327	0.067813	0.619159
0.502747	0.962585	1.38646	1.295961	0.732438	1.43924	1.28808	0.03156	0.706802
-1.06374	0.56014	0.707526	1.202142	0.723723	0.853584	0.695402	0.061544	0.642002
-1.06374	-0.45943	-0.62836	-0.90594	-0.20115	-0.81442	-0.46761	0.209449	0.704793
-1.06374	-0.14726	-0.3441	0.049522	-0.0662	-0.42351	-0.38635	0.007998	0.473035
0.095242								
0.095242	-1.26178	-1.73234	-1.90729	-1.23578	-1.65361	-1.3059	0.009793	0.671513
0.095242	0.316648	-0.05176	-0.43937	0.100973	-0.11562	-0.35345	0.07192	0.756019
0.095242	-0.02709	-0.05195	0.2016	0.358753	0.343771	0.244434	0.039523	0.410701
-0.45359	0.429256	0.803248	0.86808	0.308403	1.016541	0.739115	0.006851	0.708138
-0.22027								
-0.22027	0.16067	0.267452	-0.06009	0.140372	-0.07505	-0.07019	0.010433	0.342499
-0.22027	-0.52826	-0.46832	-0.46725	-0.38959	-0.53842	-0.38859	0.220775	0.149829
-0.45359	-1.17698	-1.63127	-1.60262	-1.38733	-1.58523	-1.47417	0.09114	0.454291
-0.45359	0.662954	1.2717	1.443537	1.014296	1.335005	1.114561	0.002811	0.780583
-0.31196	1.263106	1.016003	1.061582	0.870962	1.096504	0.797601	0.17725	0.465505
-0.31196	0.035136	-0.28478	-0.52539	-0.01081	-0.23491	-0.09758	0.001776	0.560527
-0.07919	2.06676	2.297317	1.779417	2.170203	2.532485	1.716183	0.014949	0.816302
0.36937	-0.82591	-1.01438	-1.05459	-0.66973	-0.69948	-0.63939	0.010185	0.415194
0.36937	0.85947	1.12407	0.956828	1.147292	1.746625	1.359023	0.007712	0.887155
0.36937	0.730444	0.67855	0.859927	0.691724	0.984477	0.74674	0.041488	0.305927
-0.07919	-0.14587	0.164666	0.265851	-0.01062	-0.13816	-0.09504	0.056233	0.411723
0.08693	0.456136	0.652765	0.494879	-0.00254	0.588838	0.551496	0.02172	0.655308
0.08693	1.35769	1.7024	1.324107	1.086056	1.98587	1.669293	0.00771	0.899814
-0.44291								
0.08693	0.104243	0.173992	0.50279	0.595634	0.507598	0.370257	0.011963	0.491391
-0.44291	-0.05716	0.250184	0.665078	0.490321	0.567814	0.458081	0.009165	0.722239
-0.44291	-0.95024	-1.23069	-1.81855	-1.07568	-1.1415	-1.00067	0.009462	0.868312
-0.44291	-0.76415	-0.89984	-0.74834	-1.01785	-0.41459	-0.48323	0.021765	0.603262
-0.4026	0.020154	-0.08757	-0.04416	0.1359	0.277501	0.164815	0.052662	0.365066
-0.4026	-0.16804	-0.09928	0.326958	0.147946	0.231341	0.047677	0.04352	0.494993
-0.26714								
-0.26714	-1.18801	-1.75493	-1.93097	-1.0851	-1.603	-1.41988	0.003305	0.845878
-0.26714	-1.15024	-1.89885	-1.76388	-1.18037	-1.62224	-1.26446	0.00727	0.74861
-0.26714	-0.56022	-0.93357	-1.31482	-0.65691	-0.82906	-0.76335	0.015123	0.754608
-0.26714	-1.25395	-1.63843	-1.52631	-1.07622	-1.28078	-1.15687	0.008207	0.562213
-0.26714	-0.55192	-0.55112	-0.39887	-0.08358	-0.30649	-0.29497	0.002432	0.468334
0.649768	0.419373	0.450477	0.580344	0.358565	0.649406	0.43681	0.030857	0.290841
0.649768	-0.34152	-0.72711	-0.62959	-0.60037	-0.83195	-0.76677	0.010278	0.490423
0.649768	-0.69998	-1.44549	-1.32825	-0.4668	-1.12274	-0.67714	0.046767	0.978689

0.649768	0.22763	0.368795	0.919224	0.203304	0.450812	0.219509	0.029005	0.71592
0.46482	0.285788	0.520301	0.517296	0.084674	-0.15151	0.005606	0.047354	0.671809
0.46482	-0.20904	-0.53067	-0.42992	-0.33386	-0.59782	-0.55579	0.00741	0.388785
0.46482	-0.60498	-0.932	-0.88107	-0.65907	-0.7593	-0.62948	0.03563	0.327019
0.252387								
0.252387	-0.23132	-0.40849	-0.06986	0.229804	-0.04905	-0.08505	0.021957	0.638298
0.252387	-1.13592	-1.19606	-1.14651	-0.81874	-1.01085	-0.9361	0.029514	0.377316
-0.32427	0.31469	-0.04101	-0.46729	-0.17849	-0.44486	-0.59373	0.041748	0.908419
-0.32427	0.175897	0.56521	0.840642	0.488951	0.123338	-0.01479	0.003968	0.855436
-0.32427	-0.6935	-0.72501	-1.11628	-0.78812	-1.16465	-0.91813	0.015575	0.47115
-1.10621	0.730806	0.753805	0.940663	0.752851	0.789686	0.838963	0.11811	0.209857
-1.10621	-0.57995	-0.83428	-0.8105	-0.38792	-0.86482	-0.6108	0.055624	0.476904
-1.10621	0.870217	1.074783	1.27424	1.13467	0.977499	0.795299	0.121769	0.478941
0.00064	0.479978	0.610439	0.840195	0.574861	0.786662	0.770701	0.007563	0.360216
0.00064	0.641278	0.947656	1.592797	1.21109	1.044401	0.749936	0.015721	0.951519
0.032862	-0.44132	-0.77989	-0.87675	-0.50937	-0.84223	-0.60653	0.041036	0.435423
0.032862	0.999384	0.96239	0.980321	0.491781	0.863549	0.808289	0.007276	0.507603
0.032862	1.658777	1.927653	2.273817	1.889657	2.346475	2.208453	0.032978	0.687698
1.024793								
0.032862								
1.675433	-0.42521	-0.52811	-0.29406	-0.386	-0.3855	-0.35145	0.152645	0.234058
1.024793	0.799137	0.443302	-0.39816	-0.03411	0.262737	-0.00692	0.015746	1.197301
1.024793	-0.66881	-0.88839	-1.16179	-0.8463	-0.49685	-0.67275	0.135857	0.664938
-0.36428	0.418759	0.921579	1.113711	0.685702	0.781372	0.544157	0.00787	0.694952
-0.20604	1.227496	0.998125	0.558748	0.538624	0.619743	0.451733	0.097075	0.775762
-0.20604	0.575363	0.75863	1.120154	0.944096	0.603487	0.394809	0.027385	0.725345
-0.20604	0.37559	0.761082	1.053173	0.910681	0.531586	0.387349	0.015398	0.677583
-0.36428	0.585399	0.820573	1.213333	1.165367	0.654027	0.570854	0.011882	0.642479
	-0.58424	-0.05761	0.919192	0.496784	-0.11412	-0.25439	0.000891	1.503433
1.344022								
1.344022	2.48303	2.248387	1.874243	1.89725	2.01831	2.263783	0.059441	0.608787
	0.361459	0.422669	0.852707	0.91886	0.560562	0.523248	0.000227	0.557401
1.495297								
-0.46238								
-0.46238	-1.08369	-1.13022	-1.01959	-0.85156	-1.68146	-1.13256	0.157544	0.829899
-0.46238	-0.21368	-0.48179	-0.46195	-0.15394	-0.20896	0.017181	0.013717	0.498972
0.92427								
0.92427	0.291892	1.066547	-0.93582	-0.43944	-0.18807	-0.61755	0.118484	2.002363
0.92427	-0.83371	-1.5056	-1.74469	-0.80476	-1.28968	-0.85783	0.006309	0.93993
0.92427	-0.63829	-0.89214	-1.17042	-0.6419	-0.70375	-0.48191	0.00042	0.688506
0.92427	0.961148	0.88021	0.828866	0.865244	1.517565	1.248187	0.001031	0.688699
1.592363	1.965103	1.9396	1.690537	1.242373	1.800785	1.367797	0.082426	0.72273
1.592363	0.971232	1.127583	0.904457	0.502657	0.774034	0.679051	0.004663	0.624927
2.973563	0.844221	1.124084	1.017249	0.841027	0.999512	0.733559	0.055932	0.390525
2.973563	0.188504	-0.00099	-0.05104	0.378472	0.119748	0.17123	0.010106	0.429508
2.973563	0.239787	0.055613	0.08425	0.090224	-0.14399	0.323366	0.002688	0.467358
0.527727								

0.527727	-0.45585	-0.57752	-0.71427	-0.6565	-0.43877	-0.50315	0.041702	0.275501
0.527727	0.371448	0.147133	0.321359	0.513774	1.083162	0.363168	0.039236	0.936029
-0.2601								
-0.55507								
-0.55507	2.416453	2.314037	2.262233	1.701575	2.193745	1.612502	0.104907	0.803951
-0.55507	0.445263	0.934229	1.442787	0.844174	0.898582	0.666379	0.002562	0.997524
-0.2601	0.979835	1.71365	2.217383	1.370693	1.045591	0.906303	0.000153	1.311081
-0.2601								
0.195517	2.067143	1.915377	1.466347	1.639047	2.153415	1.525027	0.132472	0.687068
0.195517	-1.0936	-1.49338	-1.67389	-1.08521	-1.27138	-0.96486	0.020401	0.70903
0.195517	0.177906	0.173538	0.189634	0.283746	0.59292	0.362576	0.011884	0.419382
-0.32945	0.82077	1.391021	1.716057	1.138462	1.32255	0.940063	0.000669	0.895287
-0.21229								
-0.32945	-0.83589	-0.75718	-1.46442	-1.0684	-1.24756	-1.03854	0.190307	0.707241
-0.32945	-0.945	-1.32201	-1.81575	-0.94929	-1.08594	-0.96249	0.019429	0.870748
-0.21229	0.70763	1.18534	1.238741	0.990298	1.16811	0.830145	0.149467	0.531111
-1.01353								
-1.01353	-0.7225	-1.15131	-1.48485	-0.62594	-1.178	-0.75024	0.029974	0.858911
-1.01353	-0.56378	-0.99222	-0.92291	-0.31614	-0.71023	-0.653	0.000777	0.676072
-0.04482								
-0.04482	0.118058	0.29635	-0.1141	1.093338	-0.09933	0.266842	0.070088	1.20744
-0.04482	1.57621	1.580593	1.88767	1.592587	1.754685	2.31721	0.146649	0.741
-0.6811								
-0.6811	0.669277	0.845422	0.78132	0.489151	1.272095	1.115987	0.003023	0.782944
-0.6811	-1.3455	-1.39503	-1.80615	-1.32472	-1.73657	-1.34037	0.08256	0.481427
-0.6811	1.14966	1.71452	2.054337	1.256223	0.928616	0.827328	0.002736	1.227008
1.271078								
1.271078	-0.56372	-0.86282	-1.27805	-0.51205	-0.50999	-0.51706	0.040679	0.768063
1.271078	-0.82208	-1.27067	-1.5794	-0.71115	-0.8567	-0.73327	0.006352	0.868255
1.271078	-0.82739	-1.4311	-1.28366	-1.00082	-1.14969	-1.03491	0.013463	0.603712
1.271078	-0.9869	-1.45998	-1.64927	-0.97194	-1.10818	-0.92611	0.032382	0.723164
-0.4817	0.442884	0.718736	0.906485	0.278406	0.562525	0.893548	0.083105	0.628079
-0.4817	0.743166	1.004662	1.038531	1.015157	1.115625	0.80702	0.050716	0.372459
-0.02754								
-0.02754	-0.25382	-0.15301	0.042844	-0.26095	-0.65991	-0.47953	0.019967	0.702754
-0.02754	-0.90991	-1.49434	-1.65454	-0.91895	-1.29503	-1.0168	0.0136	0.744633
-0.02754	-0.75247	-0.84997	-0.82532	-0.86736	-1.10977	-0.8822	0.100556	0.357296
-0.29717								
-0.29717	-1.10071	-1.52706	-1.68318	-1.34241	-1.54753	-1.14541	0.001503	0.582467
-0.29717	-0.85519	-1.46042	-1.72492	-0.84013	-1.04206	-0.77322	0.025478	0.951697
-0.29717	0.682758	0.77309	0.892526	0.680405	1.3721	1.039339	0.025363	0.691695
-0.45316								
-0.45316	-0.30836	-0.32073	-0.54704	-0.59275	-0.33207	-0.25716	0.015751	0.335588
-0.45316	-0.89634	-1.59171	-1.74173	-0.97234	-1.40369	-1.20134	0.018246	0.845387
-0.45316	1.051047	1.4589	1.54216	1.089393	0.70617	0.569466	0.003856	0.972694
0.982983	-0.51512	-0.37441	-0.60143	-0.40593	-0.11739	-0.09048	0.014713	0.510951
-0.39721								

-0.39721	-0.42081	-0.56067	-0.42229	-0.30364	-0.34379	-0.48022	0.015806	0.25703
-0.39721	0.91152	1.244057	1.549023	0.986198	1.20274	1.037647	0.001953	0.637504
-0.18142	0.38544	0.55214	0.737506	0.97716	0.971143	0.774035	0.005118	0.59172
0.982983	0.170903	0.505736	0.832486	1.192499	1.135235	0.551705	0.001957	1.021596
0.982983	0.013209	0.391028	0.471169	0.970643	1.037635	0.509803	0.018161	1.024426
-0.18142	0.123284	0.465356	0.124038	-0.35695	0.090755	0.516188	0.018638	0.873141
0.20836								
0.20836	-0.70961	-1.045	-1.48784	-0.93163	-1.18212	-1.07696	0.00153	0.778223
0.20836	0.115227	-0.88562	-0.93204	-0.62243	0.013067	-0.82618	0.067609	1.047267
-0.42524								
-0.42524	0.032906	0.05336	0.289933	0.287019	-0.00283	0.131142	0.2577	0.292766
-0.42524	0.936061	1.741573	1.91074	1.057406	1.269055	0.939797	0.002523	0.974679
-1.1749								
-1.1749	0.723373	1.24027	1.568577	1.089903	1.055435	0.768854	0.002546	0.845204
0.071007	-0.06653	0.598838	0.665951	0.048212	-0.10578	-0.06732	0.014958	0.771733
-0.13047	-1.50148	-2.09963	-2.23518	-1.59229	-2.09482	-1.73372	0.00556	0.733699
0.071007	-0.37781	-0.59495	-0.29468	0.001143	-0.31096	-0.39816	0.011179	0.596098
-0.13047	0.830284	1.236238	1.494837	1.072906	1.44414	1.285727	0.027509	0.664553
-0.13047	0.032914	-0.55293	-1.04854	0.004631	-0.55616	-0.65503	0.026448	1.081458
-0.30596	-0.75069	-1.1106	-0.69052	-0.50513	-0.98858	-0.72565	0.06746	0.605472
-0.09657	0.536261	0.203434	-0.28403	-0.03989	-0.17266	-0.35065	0.015356	0.886907
-0.61027	0.596159	0.977781	1.318037	0.632691	0.924696	1.005827	0.003471	0.721877
-0.61027	1.072703	1.736753	1.798013	1.155883	1.34528	1.2065	0.005095	0.72531
0.498567								
0.498567	-0.01542	-0.02226	-0.30144	-0.1955	-0.17695	-0.12177	0.005911	0.286024
	-0.27409	-0.58842	-0.44205	-0.05312	-0.77781	-0.84389	0.017718	0.790766
0.936168								
0.936168	0.067163	-0.09004	-0.03683	-0.1155	-0.53394	-0.43403	0.038111	0.601103
0.936168	-0.17142	-0.49622	-1.1365	-0.43266	-0.80815	-0.75569	3.14E-05	0.965079
0.936168	-1.40213	-1.93543	-2.09189	-1.40833	-1.80518	-1.38811	0.019829	0.703787
0.936168	-0.89346	-1.33753	-1.44827	-0.80497	-0.96195	-0.79707	0.039771	0.651204
0.936168	-1.11068	-1.96104	-2.27742	-1.37223	-1.7546	-1.37429	0.014463	1.166739
0.936168	-0.05626	0.301752	0.575728	0.519949	0.571813	0.185294	0.001069	0.631991
	-0.23914	-0.38946	-0.32973	-0.11231	-0.03541	-0.1631	0.048797	0.354051
	0.411963	0.539473	0.61165	0.601863	0.909232	0.643232	0.024161	0.497269
2.17905								
2.17905	-0.65726	-0.67089	-0.65984	-0.7383	-0.76441	-0.64209	0.143304	0.122314
2.17905	-0.5127	-0.48211	-1.07242	-0.78186	-0.77623	-0.57994	0.095842	0.590309
-0.56833	0.086183	-0.11118	0.730642	0.325458	0.41551	0.429807	0.00394	0.841825
-0.56833	-0.62101	-1.23064	-1.38433	-0.74563	-0.99036	-0.79535	0.010834	0.763325
-0.56833	1.032403	1.622312	1.467867	1.099055	0.775415	0.976002	0.131121	0.846898
0.115797								
0.115797	-1.26714	-1.17853	-1.76554	-1.50731	-1.98342	-1.75848	0.174338	0.804891
0.115797	0.093869	0.440981	0.870711	0.40661	0.382507	0.10265	0.000252	0.776842
0.666063								
0.666063	-0.59947	-0.83686	-0.82312	-0.71397	-0.80897	-0.64451	0.047615	0.237381
-0.06938								

-0.06938	-1.072	-1.79173	-1.78924	-1.0855	-1.40352	-1.22903	0.005086	0.719726
-0.06938	-0.49537	-0.57417	-0.48837	-0.75564	-0.5796	-0.43019	0.049608	0.325449
1.799313								
1.799313	-0.03784	0.452957	0.927806	0.823204	1.162115	0.404917	0.002144	1.199953
1.799313	0.020592	0.181649	-0.00708	-0.33504	0.107709	0.253246	0.048424	0.588287
-0.27405	0.45291	0.894841	1.083687	0.281884	0.631756	0.913101	0.017721	0.801803
-0.27405	-0.20809	-0.17792	-0.04211	-0.18858	-0.37907	-0.19611	0.04382	0.336968
1.5433								
1.5433	-0.51442	-0.89868	-0.87564	-0.46041	-0.24426	-0.06592	0.002508	0.832759
1.5433	-0.73805	-0.98443	-1.20854	-0.7941	-0.98564	-0.81962	0.00179	0.470498
-1.10006	0.729425	0.530893	0.341748	-0.38136	0.474507	0.713488	0.007019	1.110787
-1.10006	0.660295	0.918874	0.988822	0.797777	0.402889	0.383192	0.008027	0.60563
1.991773								
1.991773	0.489793	0.455915	0.399193	0.473501	0.776124	0.532371	0.039527	0.376931
1.991773	1.226223	1.128698	1.260812	1.131653	1.573805	1.618843	0.030836	0.490145
-0.62337								
-0.62337	0.476838	0.50201	0.406462	0.011542	0.54667	0.595418	0.024162	0.583876
-0.62337	0.773652	1.214963	1.25435	0.746392	0.987209	0.89518	0.001201	0.507958
-0.69006								
-0.69006	0.706121	1.883858	0.44556	1.44082	1.271318	1.287509	0.098077	1.438298
-0.69006	0.119906	0.182763	0.241883	0.974237	0.665702	0.301218	0.002275	0.854331
0.584603	0.766153	1.006077	1.067203	1.111844	1.409225	1.243217	0.010151	0.643072
0.07569	-0.19973	-0.36098	-0.48642	-0.23033	0.11374	-0.07953	0.013769	0.600159
0.584603	0.445131	0.585213	0.545248	0.22473	0.773073	0.766455	0.005244	0.548343
0.083017	-0.76513	-0.76242	-0.85839	-0.56786	-0.20736	-0.41889	0.00033	0.651022
	1.03628	1.324573	1.124333	0.615192	1.1691	1.134833	0.004864	0.709382
	-0.24405	-0.36208	-0.40433	-0.46244	0.176329	-0.13966	0.064734	0.638771
0.083017	0.437533	0.64196	0.541922	0.46179	0.778048	0.672588	0.047201	0.340515
0.07569	0.202491	0.894885	1.206753	0.932518	0.765961	0.469445	0.000941	1.004263
0.07569	-0.29835	-0.16334	-0.11214	-0.00743	0.306965	0.126074	0.075205	0.605311
-0.68596								
0.2027	0.190254	0.498331	0.166639	0.102538	1.129975	0.764133	0.000527	1.027437
-0.68596	-0.55861	-0.5664	-0.99959	-0.8036	-0.1599	-0.31164	0.000592	0.839688
-0.68596	0.051822	-0.1188	-0.20049	0.243021	-0.34534	0.190004	0.121009	0.588364
1.86243								
1.86243	-0.27661	-0.3182	-0.18367	-0.34012	-0.13565	-0.49074	0.032924	0.35509
1.86243	-1.11554	-1.68266	-1.85156	-1.16987	-1.46241	-1.07508	0.001901	0.776484
1.86243	-1.2651	-1.80876	-2.01556	-1.3776	-1.6556	-1.41192	0.013563	0.750463
1.86243	-1.17492	-2.00193	-2.02491	-1.23453	-1.68333	-1.37662	0.007208	0.849987
1.86243	-1.09569	-1.82816	-1.65532	-1.01182	-1.36469	-1.09337	0.011464	0.816344
0.40571	0.683508	0.6556	1.012849	0.266682	0.919687	0.597838	0.045961	0.746167
-0.77523								
-0.77523	0.793511	1.129493	1.26651	0.446398	1.05234	0.792701	0.001718	0.820112
0.40571	1.41109	1.338517	1.250443	0.884381	0.551501	1.259906	0.047128	0.85959
	0.070499	0.342726	0.753885	0.978581	0.53136	0.32036	0.00072	0.908083
1.350687								
1.350687	-0.96099	-1.55686	-1.45175	-0.6798	-1.19787	-0.99482	0.000397	0.877058

1.350687	-0.42867	-1.10425	-1.08491	-0.84142	-1.11905	-0.84788	0.033645	0.690389
1.350687	0.143887	-0.33855	-0.64606	-0.43999	-0.60008	-0.48866	0.027569	0.789942
1.350687	0.461045	-0.41569	-1.19034	-0.54527	-0.93582	-0.57051	0.006076	1.651388
1.350687	-1.01584	-1.5778	-1.86955	-1.23167	-1.55526	-1.20781	0.001649	0.853709
1.350687	-1.26629	-1.80589	-1.79307	-1.10776	-1.37446	-1.20324	0.005163	0.69813
1.350687	0.743281	0.510411	0.015934	0.563992	-0.13599	0.593277	0.1418	0.879272
0.037517								
0.037517	-0.74502	-1.26782	-1.29806	-0.83717	-1.14155	-0.79606	0.002312	0.553039
0.037517	-0.78942	-1.29201	-1.36057	-0.91007	-1.23293	-0.80582	0.000347	0.571146
-0.74173								
-0.74173	-0.26796	-0.45975	-0.22562	0.377692	0.179791	-0.2687	0.003458	0.837442
-0.74173	0.199103	0.963532	1.126543	0.754219	0.379052	0.539008	0.000305	0.927441
0.872533	-0.43264	-0.38479	-0.27003	-0.271	-0.13553	-0.14984	0.025521	0.29711
0.872533	-0.9068	-1.47292	-1.52006	-0.93049	-1.33371	-1.09731	0.000125	0.613252
0.872533	0.896977	0.987181	0.755286	0.562639	0.809499	0.753379	0.052915	0.424542
-0.35985								
-0.13097	-1.16633	-1.12893	-2.11937	-1.41569	-2.15073	-1.36874	0.124987	1.021794
-0.13097	-1.13574	-1.69619	-1.79173	-1.13972	-1.53619	-1.03227	0.015216	0.759465
-0.13097	-0.57676	-0.82752	-0.86354	-0.65031	-0.71944	-0.6085	0.101729	0.286786
-0.35985	-0.84252	-0.98359	-1.32204	-0.85491	-1.35984	-1.02542	0.018098	0.517319
-0.23379	-0.01282	0.436688	0.029986	-0.36472	-0.08536	0.132275	0.00703	0.80141
-0.13097								
-0.82791								
-0.82791	-1.019	-1.4908	-1.48981	-1.00845	-1.37891	-1.09578	0.01783	0.482346
-0.82791	-0.77579	-1.04409	-1.20218	-0.84738	-1.17763	-1.00221	0.020605	0.426384
-0.82791	-0.76915	-0.59353	-0.19684	-0.76066	-1.44716	-1.18936	0.001235	1.250312
-0.84358	0.471969	0.792982	1.031978	0.303187	1.008605	0.852323	0.011525	0.728792
-0.84358	0.747552	1.073388	1.26242	0.493451	0.989773	1.001323	0.019123	0.768969
-0.84358	0.693698	0.853327	1.71172	0.942371	1.151863	0.943965	0.013903	1.018022
-0.76332								
-0.76332	-0.71561	-1.48127	-1.49809	-1.08793	-1.27672	-1.13206	0.043737	0.782483
-0.76332	1.07552	0.411773	-0.8513	-0.44388	-0.3603	-0.47374	0.037186	1.926821
-0.76332	-1.11228	-1.59229	-1.69276	-1.08228	-1.38245	-1.20363	0.006109	0.610476
-0.76332	0.799554	1.160973	1.505153	0.724946	0.739017	0.697017	0.016969	0.808137
0.000498								
0.000498	-0.8961	-0.95812	-1.11631	-0.34837	-0.87989	-0.85765	0.02604	0.767938
0.000498	-0.35284	-0.56801	-0.72534	-0.49673	-0.63307	-0.52612	0.046722	0.3725
-0.92299	-0.39755	-0.08701	0.21315	-0.37528	-0.75965	-0.45366	0.00318	0.972796
-0.92299	0.997161	1.312617	1.40909	0.733991	1.202832	1.44123	0.010336	0.707239
-0.92299	0.186757	0.927052	1.134439	0.703272	0.646666	0.415082	0.000785	0.947682
0.270807	0.305552	0.602618	1.403683	0.89118	0.648381	0.775586	0.004815	1.098131
0.270807	0.66617	0.773606	0.783132	0.27586	0.68822	0.803285	0.010739	0.527424
0.270807	0.614407	0.68063	1.007509	1.119313	1.131525	0.878552	0.003188	0.517118
-0.27704	0.743429	0.953286	1.08876	0.892005	0.877373	0.818362	0.032461	0.345331
-0.27704	-0.56972	-0.51124	-0.46681	-0.52825	-0.6192	-0.62269	0.042781	0.155875
-0.27704	-0.42368	-0.22158	-0.12626	-0.45341	-0.32578	-0.32477	0.031785	0.327151
-0.27704	-1.09596	-1.48999	-1.25634	-0.95041	-1.04788	-1.00383	0.008874	0.539574

-0.27704	-1.21726	-1.47668	-1.54661	-1.21942	-1.42841	-1.09827	0.115245	0.448339
-0.91015								
-0.91015	3.53703	3.73711	3.011593	3.19073	2.528605	2.75476	0.037982	1.208505
-0.42942	-1.14643	-0.1192	-1.87801	-0.70501	-1.4636	-1.11141	0.135026	1.75881
-0.42942	-0.50716	-0.78285	-0.96232	-0.67303	-1.08437	-0.83458	0.039274	0.577202
-0.22147	-0.54751	-1.14773	-1.16178	-0.8818	-1.17138	-1.03877	0.07254	0.62387
-0.22147	-0.70383	-1.17769	-1.38299	-0.9194	-1.05048	-0.84908	0.006729	0.67916
-0.40959								
-0.40959	-0.52394	-0.44468	-0.20417	-0.25566	-0.28421	-0.47929	0.033184	0.319771
0.429747	0.539556	0.625691	0.51076	0.348333	0.988691	0.590856	0.069574	0.640358
0.429747	0.459731	1.057197	1.273703	0.975056	0.899805	1.012235	0.005334	0.813972
0.190737	-0.06247	-0.0515	-0.00269	0.283656	0.039696	0.064214	0.00469	0.346129
0.190737	1.12266	1.543483	1.60181	0.783484	1.72149	1.462357	0.006474	0.938006
0.190737	0.086325	0.462291	0.489395	0.64902	0.614751	0.548896	0.101056	0.562694
2.803257	1.37837	1.558243	1.756623	1.211692	0.898119	1.2139	0.0098	0.858504
2.803257	0.262596	0.243107	0.057386	-0.01851	0.452141	0.627455	0.013896	0.645967
0.908582	0.022776	0.235773	0.214729	0.468946	0.684874	0.255554	0.042849	0.662098
0.908582	0.569489	0.780001	0.701746	0.814107	0.66357	0.746645	0.216945	0.244618
0.908582	-0.81112	-1.20558	-1.39037	-0.7871	-0.98023	-0.84028	0.00198	0.603272
0.908582	0.01961	-0.45225	-0.13924	0.149232	0.417698	-0.20206	0.098459	0.869946
1.266465	0.470616	0.729545	0.731634	0.257166	0.390482	0.679288	0.011504	0.474469
1.266465	0.53595	0.817654	0.905492	0.463926	1.208385	0.879649	0.010063	0.744459
1.266465	-0.86372	-1.08904	-1.04204	-0.76545	-0.72706	-0.69506	0.026838	0.393976
1.266465	-0.6021	-0.79726	-0.38988	-0.75313	-0.68092	-0.63926	0.06802	0.407379
-0.5597								
-0.5597	-0.24942	-0.07231	0.351865	-0.11805	0.012899	-0.03793	0.022295	0.601284
-0.5597	-1.2667	-1.66311	-1.71289	-1.16493	-1.45278	-1.21201	0.003086	0.54796
-0.5597	-1.32365	-1.86431	-2.10336	-1.01803	-1.63455	-1.21398	0.019293	1.085328
-0.5597	-0.95285	-1.40362	-1.54451	-0.95544	-1.28444	-1.05261	0.014022	0.591661
-0.32549								
-0.32549	-0.87308	-1.32229	-1.44547	-1.0225	-1.35234	-1.05435	0.026033	0.572389
-0.32549	0.004582	-0.01546	0.077366	-0.50441	0.083146	-0.07411	0.054653	0.587559
0.736512	-0.38532	-0.35597	0.034905	0.136717	0.327011	-0.15063	0.006803	0.712334
0.736512	-0.42988	-0.95313	-0.91674	-0.45508	-0.57195	-0.57003	0.088528	0.52325
0.736512	-0.59599	-0.96495	-0.98506	-0.69569	-0.68538	-0.78662	0.089522	0.38907
-0.22272	0.156854	0.415446	0.739441	0.21401	0.752835	0.756014	0.038703	0.59916
0.736512	0.134281	0.289827	0.396918	0.42505	0.521882	0.379344	0.115118	0.387601
-0.22272	-0.79309	-1.00945	-0.93336	-0.72415	-0.65007	-0.56484	0.088683	0.44461
-0.22272	0.23993	0.546549	0.743554	0.441051	0.622927	0.506428	0.028841	0.503623
-1.24523								
-1.24523	-0.3096	-0.4228	-0.14584	0.069254	0.017397	0.053606	0.022103	0.492054
-1.24523	-1.14556	-1.63058	-1.85964	-1.03009	-1.26026	-0.90015	0.00726	0.95949
-1.24523	-1.03808	-1.09374	-0.66783	-0.51335	-0.91197	-0.83296	0.001185	0.580392
-1.24523	-0.21803	-0.87031	-1.14743	-0.43257	-0.75187	-0.67152	0.040774	0.929402
0.24286								
0.24286	1.426383	1.90949	2.023027	1.639817	1.94127	1.610003	0.058347	0.596643
0.73398	0.125996	0.128438	0.107155	0.305146	0.517293	0.465628	0.098444	0.410138

0.504867	-0.86213	-1.10522	-1.2208	-0.59	-0.98385	-0.87307	0.082787	0.6308
0.504867	-0.58476	-0.44484	-0.21213	-0.287	-0.33267	-0.43481	0.097222	0.372634
-0.0002	-0.78073	-0.5901	-0.23782	-0.19896	-0.42379	-0.48815	0.014813	0.581773
0.504867								
0.73398	-0.59225	-0.68682	-0.58134	-0.32642	-0.47851	-0.58398	0.015655	0.360401
-0.0002	-1.15925	-1.40184	-1.72746	-1.38362	-1.23479	-1.17714	0.00706	0.568203
0.091412	0.417722	0.377212	0.529692	0.705693	0.684217	0.592218	0.01952	0.328481
-1.02484	0.448027	0.635634	0.754275	0.875138	1.24579	0.853988	0.060152	0.797763
0.091412	0.621643	0.915987	1.61367	1.083617	0.853867	0.609167	0.01169	1.004503
-1.02484	1.022443	1.636063	2.11959	1.726777	1.793415	1.30202	0.0125	1.097147
1.870537	0.209463	0.419047	0.529774	0.207326	0.560674	0.406476	0.074891	0.353348
1.870537	-0.88991	-1.03981	-1.48345	-0.9686	-1.32029	-1.15059	0.013636	0.593539
-0.27352	-0.0471	-0.22744	-0.10017	0.46011	0.302892	0.108529	0.018719	0.68755
	0.028318	0.152996	0.204398	0.332367	0.765304	0.564097	0.006557	0.736986
	-0.90148	-1.19505	-1.24012	-0.91957	-0.83943	-0.94574	0.012228	0.40069
	0.12878	0.25151	0.512825	0.62036	0.491323	0.136058	0.017343	0.49158
-0.27352	-0.47419	-0.61563	-0.82925	-0.39849	-0.21959	-0.36692	0.008065	0.609668
-0.27352	-0.09292	0.118004	0.324282	0.594802	0.236835	0.195796	0.020845	0.687723
0.128408	0.444148	0.683707	0.786509	0.379853	0.553965	0.419794	0.031585	0.406656
0.128408	1.041314	1.101705	1.101304	0.815984	0.882881	0.767444	0.067701	0.334261
0.128408	-1.1441	-1.54701	-1.74289	-1.1022	-1.53599	-1.08956	0.022392	0.65333
0.128408	-0.97945	-1.54007	-1.69197	-0.99138	-1.47096	-1.11109	5.54E-05	0.71252
0.128408	-0.95763	-1.30181	-1.13734	-0.85582	-0.94074	-0.91549	0.010232	0.445984
0.700777								
-0.54171	0.595488	1.059405	1.392127	0.875673	0.974928	0.951351	0.004058	0.796639
-0.54171	0.373503	1.036076	1.165133	0.434424	0.743799	0.573379	0.001157	0.791631
0.700777	-0.63988	-1.11594	-1.3851	-0.98192	-0.31572	-0.48369	0.167915	1.069376
0.700777	-0.54094	-0.78685	-0.95335	-0.71459	-1.04104	-0.62489	0.05538	0.500098
0.700777	0.164217	0.868971	0.978655	0.448448	0.672561	0.749765	0.003871	0.814438
-0.2481	0.47396	0.933679	1.325973	0.738764	1.022873	0.758901	0.00178	0.852013
-0.2481	0.550484	0.37358	-0.03376	1.037796	0.072283	0.027887	0.229377	1.071555
-0.2481	5.654283	4.122267	2.06069	3.067573	3.12112	3.402677	0.008667	3.593593
-1.31761	0.313132	0.936546	1.230773	0.291116	0.367139	0.107376	0.003747	1.123397
-1.31761	0.877522	1.001279	1.268043	0.963971	1.011111	0.824298	0.01561	0.443745
-1.01324								
-1.01324	1.248964	1.186661	1.179327	0.695799	0.574013	0.578833	0.149798	0.674951
-0.58358	0.606098	1.17568	1.77247	0.861052	0.523837	0.499197	0.002515	1.273273
-0.92773	-1.01339	-0.85671	-0.56381	-0.72839	-1.27163	-1.0918	0.003138	0.707823
-0.92773	0.786364	0.734765	0.345648	0.372953	0.006313	-0.14006	0.029399	0.926422
-0.92773	1.384443	1.625977	1.641707	1.421097	2.038505	1.568173	0.043179	0.654062
-0.58358	1.380993	1.69158	1.70737	1.36955	1.74923	1.483293	0.021952	0.37968
0.99236								
0.99236	0.894653	1.2945	1.26401	0.569079	1.2936	1.224327	0.004129	0.725421
0.99236	0.735173	0.769069	0.526834	0.7705	1.028376	0.683163	0.01381	0.501541
2.941942	0.626043	0.668593	0.75913	0.351764	1.032215	0.952477	0.054584	0.680451
2.941942	-1.06387	-1.66615	-1.82676	-1.14107	-1.70383	-1.14083	0.009017	0.762891
2.941942	-0.51602	-0.91571	0.10206	-0.27053	-1.14865	-0.59836	0.184768	1.250705

2.941942	-0.11673	-0.19888	-0.13489	0.080316	-0.14438	0.177591	0.208731	0.376472
1.608303									
1.608303	0.591105	0.817622	1.322577	1.048756	1.23694	1.011789	0.000116	0.731472
-0.73508									
-0.73508	-0.03051	0.195828	0.483627	0.196207	-0.25204	-0.19975	0.004809	0.735663
-0.66573	5.56306	5.19022	4.610457	4.523389	4.710217	4.01064	0.037413	1.55242
-0.66573	0.939716	1.164785	1.016453	0.906147	1.392885	0.996729	0.111117	0.486738
0.65494	1.121512	1.017258	0.540848	0.386184	1.095688	0.503967	0.120541	0.735328
0.65494	-0.32481	-0.41023	-0.65334	-0.36214	-0.22853	-0.2701	0.006849	0.424815
0.65494	0.589621	0.114335	0.037035	0.637009	1.218225	0.483629	0.019152	1.18119
	0.205178	0.431393	0.503527	0.310728	0.103194	-0.07468	0.014145	0.578209
0.203917									
	0.424667	0.524237	0.64606	-0.07413	0.663701	0.450272	0.0157	0.737835
0.203917	0.359607	0.263928	0.083679	0.07442	0.5707	0.08385	0.058171	0.49628
0.203917	0.133885	0.016088	0.044942	-0.05088	0.567857	0.291292	0.037213	0.618735
0.070553	0.070479	0.081399	0.208562	0.00358	0.399644	0.237307	0.048853	0.396063
0.437293	0.581885	1.06645	1.430267	0.399943	0.3633	0.458327	0.005383	1.066967
0.437293	0.539304	0.857859	1.09361	1.05061	0.801892	0.554771	0.00043	0.554306
0.437293	0.027639	0.031653	0.27407	0.209458	-0.09934	-0.1217	0.127334	0.395774
-0.71686	0.579935	0.602187	0.589273	0.666063	1.072003	0.813789	0.067254	0.492067
-0.47778									
-0.47778	0.828785	0.820756	0.784422	1.080945	1.47239	0.6929	0.014112	0.77949
-0.47778	1.418454	0.713912	0.998242	1.000153	1.03077	0.445652	0.091503	0.972802
-0.71686	0.138541	0.551702	0.622319	0.273689	0.490131	0.387794	0.017215	0.483778
	1.089985	1.099976	1.311111	1.134079	1.832015	1.463233	0.017792	0.74203
-0.62164									
-0.62164	0.709703	0.739839	0.832678	0.430875	1.034514	1.026015	0.078232	0.603639
	-0.02072	0.193643	0.226406	0.26921	0.131078	0.130934	0.004064	0.289928
	3.99705	3.859277	3.067323	3.221887	3.518275	2.984237	0.100602	1.012813
	-0.74677	-1.10945	-1.254	-0.82782	-0.92105	-0.88091	0.027405	0.50723
	-0.90411	-1.18225	-1.34613	-0.95332	-0.96782	-0.9799	0.004142	0.442026
-1.12246									
-1.12246	0.409018	0.45189	0.682531	0.177633	0.35129	0.127858	0.027546	0.554673
-0.64779									
-0.64779	0.912057	1.578473	2.224083	1.679083	1.475475	1.150343	0.00604	1.312026
0.21215	2.653543	2.35509	1.842383	1.7346	2.149315	1.7511	0.033699	0.918943
0.139378	0.548609	0.778331	0.896373	0.251483	0.421715	0.556124	0.014131	0.644891
0.139378	-0.34002	0.242698	0.575488	0.393999	0.057389	0.130647	0.010451	0.915509
0.21215	-0.63278	-0.81076	-0.62487	-0.42361	-0.58921	-0.44879	4.37E-06	0.387152
0.21215	-0.03574	0.60512	0.926613	0.283642	0.432028	0.37575	0.004548	0.962349
0.331247									
0.331247	0.264034	0.273992	0.350133	0.530133	0.350036	0.334041	0.061508	0.266098
0.044597	0.724378	-0.51015	-0.80015	-0.29803	0.106549	-0.17603	0.069992	1.524531
0.044597	0.210119	0.136978	0.391243	0.4801	0.720968	0.666755	0.005506	0.583989
0.044597	0.245485	0.35778	0.193076	0.350408	0.608628	0.449353	0.068162	0.415552
-0.3054	7.109553	7.220017	6.205803	6.237483	6.338245	5.995497	0.011572	1.22452
-0.3054	-0.97236	-1.64628	-1.58777	-0.98503	-1.22219	-0.98738	0.019724	0.673917

-0.3054	-0.61638	-1.08325	-0.56298	-0.86456	-1.03977	-1.05068	0.122709	0.520264
3.034545	-1.0536	-1.52555	-1.50069	-1.05489	-1.21625	-0.95443	0.003603	0.571122
3.034545	0.420547	0.995117	0.579223	1.6078	-0.02295	0.592863	0.131608	1.630751
3.034545	-0.87174	-1.34744	-1.40025	-0.60411	-1.09594	-0.80488	0.003142	0.796136
3.034545	-0.74542	-1.26083	-1.60238	-0.78662	-1.26261	-0.95287	0.002662	0.856955
3.034545	-1.17964	-1.8202	-1.89712	-1.35261	-1.76138	-1.39967	0.003304	0.71748
0.516263	3.71783	3.769577	2.998127	2.851567	2.93014	2.718493	0.010625	1.051083
0.516263	-0.04038	-0.0285	0.017312	0.085974	0.547774	0.461411	0.000862	0.588154
0.516263	-0.53311	-0.72873	-0.32203	0.054344	-0.43273	-0.41709	0.012377	0.783069
-0.31508	0.30337	0.44695	0.799278	0.394607	0.163568	0.342433	0.079944	0.63571
-0.31508	-1.37352	-1.92739	-1.92475	-1.29238	-1.9035	-1.48617	0.08086	0.635011
-0.31508	0.671031	0.750813	0.699584	0.532579	0.866754	0.696002	0.029246	0.334175
0.632393								
	0.108238	0.352658	-0.31951	-0.04322	-0.23068	-0.24752	0.151755	0.672166
0.632393	0.010121	0.386102	-0.00059	-0.16045	0.339857	0.182028	0.10877	0.546553
0.632393	0.361142	0.739151	0.786743	0.585453	1.317865	0.937795	0.021289	0.956723
-0.64107	0.098791	0.283774	0.867128	0.247286	-0.05199	-0.03436	0.008578	0.919122
-0.64107	-0.58861	-0.96044	-0.90248	-0.54682	-0.60416	-0.59048	0.030505	0.413619
-0.64107	-0.80338	-1.00761	-1.03485	-0.64218	-0.81103	-0.85577	0.008308	0.392673
0.878853	0.143881	0.608415	0.844466	0.148227	-0.11327	0.213976	0.052054	0.957738
0.878853	0.294119	0.35156	0.284461	0.039819	0.645669	0.633479	0.002989	0.60585
-0.37105								
-0.37105	0.816227	1.020657	0.904242	0.709083	1.03664	0.960655	0.017434	0.327557
-0.37105	0.796539	0.30987	0.27317	0.466213	0.280652	-0.05177	0.094114	0.848304
-0.20043	0.245025	0.141228	0.438532	0.571052	0.464141	0.255238	0.132913	0.429825
-0.20043	1.044163	1.426553	1.799904	1.322106	1.51383	1.16293	0.026119	0.75574
0.32709								
0.32709	0.093605	0.342121	0.488835	0.144805	0.39793	0.480843	0.034641	0.39523
	0.485447	0.609642	0.318799	0.12876	0.452475	0.406014	0.103903	0.480881
1.062152								
0.396152	-0.05812	0.236058	0.224191	-0.14469	-0.08052	0.042953	0.060343	0.380748
1.062152	1.991523	1.75615	1.732873	2.18961	2.903287	2.593778	0.325283	1.170414
0.396152	0.66117	0.388738	0.390769	0.225201	0.950177	0.360706	0.167993	0.724976
1.682943	1.79896	1.940667	2.513757	1.72522	2.284005	2.180433	0.022863	0.788537
1.682943	-0.93251	-1.36579	-1.70595	-0.61493	-1.30864	-1.02271	0.02893	1.091016
1.682943	-1.3895	-1.54132	-1.79617	-1.28938	-1.38583	-1.38135	0.004524	0.506793
0.491403								
0.491403	-1.36141	-1.73848	-1.85057	-1.2342	-1.04659	-1.14196	0.030831	0.803979
0.491403	-1.50785	-2.08263	-2.09731	-1.37129	-1.92357	-1.3463	0.000926	0.751009
0.040677	0.073867	0.22891	0.528356	0.403191	0.303592	0.229642	0.000168	0.454489
0.040677	-0.35403	-0.33032	-0.32238	-0.14453	-0.02287	-0.02684	0.032424	0.331161
0.040677	-0.94806	-1.34001	-1.5321	-0.89396	-1.35721	-1.1085	0.010511	0.638145
0.040677	-0.47531	-1.1557	-1.42467	-0.75718	-0.96713	-0.67623	0.015054	0.949367
0.040677	-0.36591	-0.6613	-1.21604	-0.40655	-0.88039	-0.69599	0.015935	0.850133
0.040677	0.888252	0.8476	0.986798	0.810644	0.70944	0.462513	0.005072	0.524285
0.985917								

0.985917	-1.02805	-1.68658	-1.88419	-1.34318	-1.52972	-1.50675	0.000461	0.856146
0.985917	-1.02556	-1.71327	-2.03762	-1.44977	-1.56205	-1.3788	0.049949	1.012058
0.985917	-0.31342	-0.51193	-0.90104	-0.34458	-0.5568	-0.48293	0.003351	0.587623
0.985917	-0.78866	-1.33291	-1.2237	-0.77822	-0.95851	-0.835	0.029026	0.55469
0.985917	-0.19878	-0.28378	-0.3533	-0.28259	-0.13144	0.026448	0.103785	0.379752
1.37455	0.568331	0.746956	0.920498	0.434628	0.603177	0.826792	0.076367	0.48587
1.37455	-0.3155	-0.82766	0.253752	0.662964	-0.65756	0.463015	0.080234	1.490626
1.37455	-1.34381	-1.81599	-2.04135	-1.32474	-1.50989	-0.89685	0.014199	1.1445
1.37455	-1.27523	-1.65376	-1.29351	-0.32092	-1.51058	-1.28764	0.052828	1.332836
1.37455	-0.67744	-0.72311	-0.75984	-0.51857	-1.05697	-0.58644	0.235167	0.538402
1.37455								
-0.05634	-0.25164	-0.03632	0.2085	-0.21615	0.322509	0.197203	0.093719	0.574147
-0.05634	-0.89089	-1.65026	-1.55252	-0.95587	-0.99317	-1.0372	0.001676	0.759372
-0.05634	-0.98477	-1.44003	-1.66117	-0.9936	-1.30054	-1.02184	0.01699	0.6764
-0.05634	-0.38011	-0.62895	-0.6489	-0.21927	-0.42814	-0.28972	0.016438	0.429629
-0.32521	0.157115	-0.05	0.082346	-0.15904	0.073834	0.186921	0.01625	0.345961
-0.32521	-0.17302	0.149218	0.912614	1.035957	0.469381	0.099378	0.000313	1.208979
-0.32521	1.802705	1.128469	0.810251	1.323858	0.582928	0.5435	0.047376	1.259205
0.31955	-0.84063	-1.37823	-1.42006	-0.95771	-0.76994	-1.09244	0.186924	0.650126
0.31955	1.635862	1.718801	1.561534	1.158167	0.729549	1.867961	0.119285	1.138413
0.350747	0.452585	0.85226	1.03902	0.377007	0.931848	0.927213	0.078172	0.662012
0.31955	0.625851	0.700861	0.925642	0.436123	0.703537	0.850196	0.023627	0.489519
0.350747	0.826797	0.970084	0.981807	0.875041	0.999919	1.061701	0.078666	0.234904
-0.2052								
-0.2052	1.937833	2.164367	2.306227	2.29388	2.67082	2.122493	0.100677	0.732987
-0.2052	-0.181	-0.3175	0.491972	1.215998	0.41644	0.010148	0.002855	1.533501
-0.2052	-1.16331	-1.72573	-1.97237	-1.20109	-1.52323	-1.27789	0.001254	0.809057
-0.2052	-0.60599	-1.20532	-1.41088	-0.66015	-1.06629	-0.96971	0.043042	0.804886
	0.966183	1.049151	0.929097	0.600414	0.53801	0.536835	0.044611	0.512315
0.225497								
0.225497	0.919735	0.644309	-0.19995	0.080835	0.2752	0.707808	0.005355	1.119683
0.225497	-0.71089	-1.25213	-1.3017	-0.77644	-1.10738	-0.69321	0.015995	0.608498
0.225497	0.467158	0.21988	0.572466	0.930035	1.055785	0.654166	0.033833	0.835905
-1.22465								
-1.22465	-0.69596	-0.94529	-0.95818	-0.60062	-0.89906	-0.8301	0.003649	0.35756
1.056953	0.434976	0.4892	0.559161	0.350733	0.223078	0.196957	0.034861	0.362205
1.056953	0.776875	1.181757	0.943596	0.717076	0.444518	0.342151	0.015223	0.839606
0.2322								
0.2322	1.085756	2.066873	2.160447	1.006003	0.303667	0.250807	0.000885	1.90964
-0.26786	-0.5164	-0.66012	-1.05894	-0.85047	-1.1258	-0.91067	0.029594	0.609391
-0.19342	0.936468	0.898663	0.800774	0.553355	0.8758	0.861863	0.054508	0.383113
-0.26786	0.067749	-0.31598	-0.17284	0.316594	0.962033	0.805578	0.005817	1.278018
-0.26786	-0.60655	-1.20468	-1.00824	-0.77173	-1.16918	-0.97431	0.001471	0.598126
-0.26786	-0.77258	-1.39176	-1.54473	-0.72926	-1.03122	-0.81693	0.011254	0.815469
-0.26786	-0.96691	-1.51801	-1.72444	-0.78566	-1.36929	-0.95058	0.004987	0.938776
-0.26786	-0.88746	-1.54977	-1.67504	-0.88739	-1.33147	-1.02807	0.011726	0.78765

-0.26786	-0.68397	-1.17064	-1.20147	-0.47099	-0.71166	-0.74268	0.093112	0.730474
-0.26786	-0.57745	-1.34189	-1.44488	-0.6033	-1.19215	-0.88103	0.006106	0.867433
-0.26786	-0.88676	-1.47154	-1.65488	-0.79641	-1.39717	-1.01859	0.00192	0.858467
-0.26786	-0.56101	-1.05318	-1.5653	-0.6792	-1.03591	-0.80482	0.001369	1.004282
-0.19342	-1.04393	-1.6595	-1.76044	-1.00363	-1.64949	-1.27795	0.011723	0.756809
-0.19342	-0.08306	-0.53158	-0.90471	-0.32475	-0.46851	-0.31114	4.92E-05	0.821647
-0.95395	0.19293	0.571975	1.128569	0.584345	0.327878	0.299369	0.001783	0.935639
-0.95395	0.653366	0.062191	-0.38444	-0.31993	0.16423	0.399864	0.000433	1.037809
-0.95395	-0.24805	0.107095	0.604016	0.185059	-0.43393	-0.29748	0.013517	1.037945
2.802293								
2.802293	-0.8657	-1.54378	-1.68111	-1.00827	-1.45064	-1.24369	0.009769	0.81541
2.802293	-0.14518	-0.07537	-0.07698	-0.33764	-0.65302	-0.41942	0.004827	0.577655
0.208837								
0.208837	0.390531	0.739348	0.437511	0.359801	0.750863	0.73616	0.055374	0.391062
0.208837	0.725017	1.136657	1.46525	1.065247	0.956367	0.770708	0.00108	0.740233
-0.01491								
-0.01491	2.357667	2.335807	2.227033	1.813262	2.54675	1.496335	0.090519	1.050415
-0.55455								
-0.55455	-0.16425	0.206381	-0.14231	-0.66997	-0.8326	-0.81767	0.165537	1.03898
-0.55455	-0.58284	-1.09717	-1.28971	-0.65381	-1.35375	-0.95195	0.003688	0.77091
1.340437	0.504634	1.065882	1.317573	0.394457	0.763928	0.592857	2.05E-05	0.923117
1.340437	-0.19775	-1.04155	-0.85988	-0.24762	-1.12241	-0.81643	0.032407	0.924654
1.340437	0.139503	0.148092	0.802107	0.371538	0.023682	-0.05939	0.010811	0.8615
-0.14857								
-0.32959								
-0.32959	0.710023	0.905535	0.884997	0.6841	1.112015	1.013411	0.010087	0.427915
-1.10609	5.291113	5.97113	4.183173	4.467563	4.592095	3.828633	0.015157	2.142497
-1.10609	-0.3632	0.271823	0.493076	0.323019	0.404153	0.039242	0.022381	0.85628
-0.60342	-0.24165	-0.41442	0.003677	0.04109	-0.33328	-0.26585	0.146791	0.455511
-0.1158	-0.56774	-0.78466	-0.342	-0.21658	-0.63693	-0.46507	0.009332	0.568073
-0.1158	1.391167	1.3463	1.102329	0.55016	1.312135	1.296787	0.022836	0.841007
1.289605	0.058327	0.225856	0.285656	-0.31945	0.088306	0.418278	0.008512	0.737723
1.289605	0.209392	0.524657	-0.09833	0.149024	-0.24362	-0.13711	0.165522	0.768273
1.289605	-0.46226	-1.03214	-1.48028	-0.31736	-0.95784	-0.68886	0.026696	1.16292
1.289605	-0.971	-1.54614	-1.61817	-1.11147	-1.14898	-1.25548	0.008471	0.647173
1.289605	-0.46102	-1.60326	-1.79517	-1.26486	-1.37746	-1.22734	0.010101	1.334145
1.289605	-1.51521	-1.777	-1.91623	-1.21961	-1.46304	-1.26332	0.016006	0.696625
1.289605	-0.88812	-1.46573	-1.71666	-1.12845	-1.08394	-1.28673	0.054215	0.828543
1.289605	-1.56995	-1.9329	-2.04456	-1.21808	-1.74007	-1.59098	0.044745	0.826488
1.289605	-0.82188	-0.69669	-1.3281	-1.08527	-0.8222	-0.94755	0.058473	0.63141
1.289605	0.763129	1.139107	-0.01372	0.144092	0.175782	-0.0742	0.124674	1.213306
	-0.46121	-0.19267	-0.13273	-0.14274	-0.17258	-0.33148	0.028462	0.328481
	0.770479	0.534303	0.41828	0.539226	0.596431	0.585302	0.07013	0.352199
	1.849637	2.463283	2.351057	1.004921	1.529285	1.269476	0.105992	1.458362
	-0.89362	-1.31852	-1.47234	-0.9826	-0.76915	-0.76984	0.007784	0.703194
	-0.92395	-1.38233	-1.56867	-0.86224	-1.13397	-0.8311	0.017328	0.737571

	-0.74778	-0.42882	0.153604	-0.63157	-0.60682	-0.82842	0.013643	0.982026
-1.08845								
-1.08845	0.254986	-0.113	-0.61641	-0.01114	-0.47224	-0.16941	0.013263	0.871396
0.520978								
0.520978	-0.29678	-0.53994	-0.43488	-0.27614	-0.7813	-0.39007	0.063882	0.505168
1.239297	1.333417	1.359417	1.635008	1.078691	0.890979	1.146697	0.039591	0.744029
1.239297	1.16238	1.634287	1.563512	1.645726	0.831431	1.260045	0.176795	0.814296
-0.47436	0.149266	-0.1411	0.069812	0.529076	0.139136	0.467092	0.132726	0.670171
-0.47436	0.822872	0.805535	0.952075	1.0036	0.600646	0.389688	0.125776	0.613912
-1.27673								
-1.27673	1.388263	1.64806	2.053113	1.087037	1.59435	1.326167	0.01929	0.966077
-0.96148	0.086777	0.538459	1.533957	0.970294	0.367525	0.136321	0.000367	1.44718
-0.36992	0.308242	0.967915	1.630097	1.16497	1.15862	0.673877	0.00314	1.321855
-0.96148								
-0.96148	-0.3719	-0.79495	-0.19577	-0.23633	-0.48195	-0.54548	0.002501	0.599179
-0.36598	-0.7141	-0.81393	-1.00465	-0.7631	-1.25629	-0.63358	0.057771	0.622708
-1.01047	0.368403	1.083117	1.566837	0.694146	0.574393	0.34373	0.006091	1.223106
0.054223	0.134439	0.346895	0.742601	-0.05309	0.54346	0.347073	1.13E-07	0.795687
0.054223	-0.04066	0.287881	-0.13216	0.587504	-0.64612	0.570435	0.176753	1.233619
0.054223	-0.0223	-0.19108	-0.38932	-0.51159	-0.50892	-0.18135	0.015784	0.489287
1.10458								
1.10458	-0.35789	-0.1997	-0.49956	-0.34382	-0.35621	-0.38716	0.199119	0.299855
1.10458	-0.66389	-1.07572	-1.2557	-0.8974	-0.94593	-0.69392	0.050982	0.591814
1.10458	0.470296	0.190565	-0.31994	0.09836	0.094127	0.021457	0.001582	0.79024
-0.28654	0.114365	0.429071	0.777449	-0.21655	0.556624	0.403316	0.006469	0.993995
-0.28654	-0.89653	-0.90241	-0.92414	-0.68059	-0.53641	-0.58868	0.021158	0.387734
-0.28654	0.42551	0.912389	1.54817	1.089326	1.300815	0.834956	0.0002	1.12266
-0.17537	0.705122	1.013972	1.53076	1.407653	1.374445	0.916389	0.005967	0.825638
-0.06181	0.520328	1.297957	1.366491	1.10681	1.307045	0.926866	0.001484	0.846163
-0.40817	0.092552	0.126951	0.461909	0.48167	0.454209	0.224982	0.032881	0.389118
-0.71606	2.3028	2.153427	1.220135	1.4147	1.27742	1.463077	0.003306	1.082665
-0.71606	2.326727	2.35464	2.152563	1.627487	1.62892	1.351508	0.013749	1.003132
0.65622								
0.65622	-0.4393	-0.93438	-1.42222	-0.8076	-1.00451	-0.73664	0.008421	0.982922
0.65622	0.898124	0.928439	-0.50124	0.449096	-0.31782	-0.05688	0.132249	1.429683
0.65622	0.541135	0.849789	-0.86759	0.295951	-0.18009	-0.01346	0.026957	1.717377
-0.35463								
0.459583								
0.459583	-0.54579	-1.02185	-0.86964	-0.72105	-0.66628	-0.60314	0.004801	0.476065
0.459583	-0.36211	-0.27269	-0.22034	-0.21486	0.140817	-0.14253	0.00852	0.502929
-0.35463	1.591067	1.676283	1.022026	1.108855	1.390005	0.894102	0.037937	0.782182
-1.16329	1.177541	1.848037	1.987177	1.504993	1.786745	1.27246	0.016511	0.809636
-0.82783	2.454307	3.005843	2.322893	2.262337	2.087005	2.119343	0.148747	0.918838
-1.16329	-0.1256	-0.10605	-0.00605	-0.62801	-0.07414	-0.12611	0.013927	0.621962
-1.16329	0.697911	0.929886	1.048935	0.773328	0.928675	0.72058	0.029455	0.351023
0.037213	0.035193	0.121231	0.325371	0.294155	0.519008	0.478726	0.006887	0.483815

0.037213	2.303983	2.291963	1.64988	1.872317	1.89652	1.8585	0.02407	0.654103
0.035012	0.079489	0.036178	0.06199	0.48575	0.130744	0.150551	0.05052	0.449573
-0.95831	2.155807	1.97037	1.521461	1.792767	1.540064	1.219261	0.064941	0.936546
-0.95831	0.405391	0.328484	0.429986	0.557274	0.227347	0.227749	0.03124	0.329927
0.035012	-0.80716	-0.91402	-0.76329	-0.69035	-0.32223	-0.64131	0.049108	0.591794
0.016933	0.808986	0.831191	0.021598	0.525689	0.489957	0.309935	0.050823	0.809593
0.016933	0.733753	0.78745	1.349521	1.327214	1.019904	0.469643	0.058181	0.879878
0.514073								
0.514073	-0.06765	0.124643	0.083142	-0.12346	-0.12339	0.043785	0.098787	0.248102
0.514073	-0.58468	-0.76517	-0.51162	-0.35149	-0.17926	-0.31857	0.000283	0.585908
0.178337								
0.178337	-0.33073	-0.59185	-0.54849	0.022552	-0.07424	-0.01287	0.002694	0.614397
0.178337	-0.37073	-0.77887	-0.62095	-0.26179	-0.52637	0.011711	0.038754	0.790579
-0.48179	3.195303	2.961765	2.275567	2.706445	3.302705	2.285435	0.225716	1.027138
-0.48179	0.24322	0.684561	0.323732	0.323083	0.328372	0.363798	0.166394	0.441341
-0.48179	0.632578	1.30075	1.671383	0.901068	1.047835	0.735442	0.001517	1.038806
0.286617								
-0.59944								
0.286617	0.035859	-0.48366	-0.66235	-0.40178	-0.59605	-0.45876	0.004926	0.698212
0.286617	0.757932	1.334327	1.72547	0.758503	1.154325	0.687035	0.002597	1.038435
-1.37571	0.866158	1.541283	1.99714	1.306946	1.71392	1.241903	0.000486	1.130982
-0.5365	0.805541	1.44956	1.729417	1.432673	1.654425	1.18524	0.00027	0.923876
-0.5365	0.312625	0.455779	1.080725	0.102742	0.44942	0.385892	0.014789	0.977983
-1.37571	-0.81635	-1.22033	-1.14921	-0.73307	-1.08851	-0.97005	0.017968	0.487254
-1.37571	-0.49119	-0.79925	-0.55428	-0.69039	-1.00164	-0.72973	0.022272	0.510447
-0.03072	-0.17556	-0.23908	-0.26831	-0.40168	-0.2781	-0.28969	0.053636	0.22612
-0.03072	-0.22425	-0.26925	-0.52942	-0.33243	-0.34777	-0.31476	0.07704	0.305165
2.940687								
-0.03072	0.843551	0.93134	1.08701	0.568292	0.54567	1.039051	0.019553	0.541341
2.940687	0.831667	1.26896	1.020186	0.610703	1.11217	1.14676	0.007336	0.658257
0.627537	0.808211	0.71238	1.32814	1.10207	1.425005	1.34574	0.075369	0.712625
0.627537	-0.55335	-0.80453	-0.86571	-0.60093	-0.93396	-0.58006	0.030273	0.380612
0.627537	-0.57112	-0.93147	-0.724	0.058274	-0.16504	-0.31678	0.000244	0.989741
0.616453	1.068383	1.5835	1.722553	0.895679	0.881475	0.829109	0.00921	0.893444
0.616453	-0.96597	-1.28983	-1.34943	-0.86341	-1.14487	-1.0241	0.004713	0.48602
0.616453	1.099145	1.416043	1.703153	0.88941	0.702928	0.847595	0.008196	1.000225
-0.76932								
-0.76932	-0.82974	0.40822	-1.30752	-0.59489	-1.11872	-0.69786	0.18966	1.715743
-0.76932	-0.98479	-1.38284	-0.85663	-0.68422	-0.98656	-0.96046	0.014332	0.698618
-0.77541	-0.15112	-0.31955	-0.13209	0.09046	0.330663	0.384431	0.019693	0.703983
-0.77541	0.082069	-0.01285	-0.03104	0.300477	0.1533	0.213613	0.058424	0.331521
-0.67902								
-0.67902	-0.00603	0.170654	-0.13939	-0.52988	-0.67928	-0.64765	0.214318	0.849935
-0.67902	-0.45935	-1.13716	-1.30768	-0.76167	-0.88531	-0.84872	0.00258	0.848327
-0.67902	-0.79692	-1.24135	-1.53222	-0.84149	-1.26275	-0.746	0.058767	0.786212
-0.67902	-0.05061	0.063927	0.55942	0.069499	-0.09249	-0.1822	0.004358	0.741619
-1.05265	-0.04662	-0.5225	-1.23344	-1.82057	0.315443	0.771171	0.001013	2.591745

-1.05265	1.336437	1.877403	1.825413	1.143447	1.233075	1.253607	0.000123	0.733957
0.810237	0.778724	0.760306	1.098157	1.097102	1.37778	1.163817	0.025415	0.617474
0.810237	0.903055	1.324843	1.508257	0.949697	1.66808	1.46481	0.013359	0.765025
-0.62273								
-0.62273	-0.56499	-1.02197	-0.83717	-0.434	-1.27254	-0.76631	0.038546	0.838537
-0.62273	0.357034	-0.15722	-0.09469	-0.16577	0.083731	0.026336	0.079039	0.522803
-0.77624								
-0.77624	-0.0711	-0.12438	0.080817	-0.15354	-0.11919	-0.20656	0.03924	0.287381
0.414927	0.25479	0.36632	0.584338	0.234743	0.40604	0.499418	0.023099	0.349596
0.414927	-0.92099	-1.75682	-2.09258	-1.25016	-1.71952	-1.29025	0.009386	1.171597
0.414927	-0.09534	-0.47691	-0.88043	-0.47521	-0.769	-0.53298	0.012436	0.78509
-0.38841								
-0.82169	1.17529	1.890073	2.130093	1.258313	1.31232	0.94347	0.005291	1.186623
-0.64743	-0.02686	0.525598	0.781131	0.247328	-0.35465	-0.18529	0.016181	1.135776
-0.82169	0.227297	0.318549	0.626548	0.230728	0.272113	0.228283	0.042435	0.399251
-0.38841	0.125597	0.333372	0.689965	0.308676	0.041572	-0.05697	0.010888	0.746934
-0.64743	-0.58422	-0.74077	-0.9058	-0.71769	-0.90855	-0.85375	0.027911	0.32433
-0.64743	-0.0288	-0.36305	-0.97461	-0.31109	-0.34813	-0.03947	0.08956	0.945808
-0.64743	-1.14711	-1.41564	-1.54299	-1.10822	-1.24202	-0.95706	0.001609	0.585928
-0.60465	0.596773	0.652012	1.047258	1.25449	1.04449	0.665812	0.011106	0.657717
	0.4695	0.582468	0.891307	0.466381	0.179191	0.246045	0.022759	0.712116
-0.16014	0.346063	0.675543	1.18168	0.462723	0.32837	0.371689	0.020402	0.853311
-0.16014	-0.52264	-0.94992	-0.83076	-0.6961	-0.95492	-0.75663	0.023663	0.432278
-0.16014	0.133772	0.060101	-0.26272	0.539312	-0.00914	-0.17848	0.138444	0.802037
3.7366	0.401641	0.691584	0.96523	0.56248	0.53733	0.254667	0.018385	0.710563
3.7366	1.315463	1.431567	1.436013	1.16251	1.201765	1.499367	0.020431	0.336857
-0.76156	-0.08448	0.015092	0.271139	0.079256	-0.17837	-0.257	0.0196	0.528136
-0.76156	0.598627	0.147127	-0.03149	-0.18985	-0.30274	0.10285	0.071202	0.901367
-0.61721	-0.79993	-1.12047	-1.03829	-0.78139	-0.8199	-0.66884	0.06016	0.451628
-0.61721	0.100585	0.697013	0.163238	0.200993	0.306099	-0.10427	0.016901	0.801287
-0.64633	0.600131	0.746066	0.7499	0.264528	1.008843	0.786006	0.062384	0.744315
-0.21932	0.548394	0.198877	0.571462	0.549403	1.19347	0.641272	0.021403	0.994593
-0.22639								
-0.22639	-0.68751	-1.23899	-1.08433	-0.67	-0.88323	-0.82768	0.010547	0.568998
-0.22639	-1.03039	-1.40153	-1.04254	-0.53942	-0.90003	-0.82667	0.010315	0.862106
0.298263	1.060393	1.265757	1.661023	1.03566	1.28415	1.33504	0.043107	0.625363
0.298263	0.272172	0.075575	0.534276	0.594324	0.034282	0.067957	0.000314	0.560041
0.298263	0.43102	0.260877	0.613633	0.748758	0.247298	0.305368	0.010384	0.501461
0.352483	0.016006	-0.0338	0.025953	0.117406	-0.19268	-0.25957	0.055276	0.376977
0.352483	0.062694	0.267304	0.362425	-0.09629	0.220186	0.178685	0.020527	0.458718
-0.4342								
-0.4342	-0.19661	-0.44854	-0.49037	-0.05692	-0.03632	-0.02888	0.016956	0.461487
-0.4342	0.253074	0.624321	1.027071	0.428622	0.174252	0.032724	0.037838	0.994347
0.345488	0.182514	0.300376	0.308018	0.272849	0.818263	0.469533	0.058309	0.635749
0.345488	-0.62164	-0.67344	-1.04188	-0.39783	-0.52937	-0.42393	0.042066	0.64405
0.345488	-0.32058	-0.50161	-0.6622	-0.31682	-0.54773	-0.44372	0.075111	0.345385

-0.7041	0.453575	0.83648	0.563073	-0.09836	1.073743	1.00289	0.010536	1.172099
1.228033	0.796936	0.797573	0.934251	1.069221	1.523145	1.09611	0.023156	0.726209
1.228033	-1.1255	-1.55857	-1.69593	-1.14936	-1.29431	-1.08378	0.018745	0.612151
-0.57289	0.741752	0.813702	0.848648	1.045412	1.409465	1.146633	0.006078	0.667713
	0.347498	0.229309	0.099001	0.478208	0.824275	0.641376	0.002211	0.725274
-0.57289	-0.76212	-1.28582	-1.3462	-0.89386	-1.19728	-0.94578	0.011192	0.584073
-0.57289	1.327662	0.382049	0.717719	0.595362	0.790555	0.320071	0.165531	1.007591
0.797327								
0.797327	-0.87105	-1.23926	-1.47138	-0.85753	-1.04402	-0.86057	0.020985	0.61385
0.797327	0.200986	0.430218	0.217927	0.607357	0.581905	0.681842	0.207025	0.480856
-0.21371	5.49937	5.168507	4.837954	4.504317	4.78269	4.058383	0.024713	1.440987
-0.21371	-0.79664	-0.90599	-0.69638	-1.01859	-1.05197	-1.11765	0.17471	0.421263
-0.21371	-0.04801	0.285888	0.57086	0.30218	0.506246	0.259611	0.044347	0.618871
1.813557								
1.813557	1.865973	-0.61776	-1.2301	-0.55708	-0.92884	-0.06183	0.062278	3.096071
1.813557	-0.08615	-0.37231	-0.72136	-0.42303	-0.0956	-0.41065	0.11262	0.635213
1.813557	-1.19162	-1.82753	-1.98987	-1.04526	-1.5121	-1.21212	0.000473	0.944604
1.813557	0.374182	0.454622	0.441983	0.427812	0.954851	0.590925	0.052242	0.580668
-1.59536	0.268181	0.600013	0.881694	0.665232	0.866201	0.831087	0.081113	0.613513
-1.59536								
	-0.62361	-1.32914	-1.46598	-0.76078	-1.10988	-0.83694	0.017305	0.842363
-0.10609								
-0.10609	-0.73011	-1.31415	-1.60311	-0.57821	-1.41976	-0.81793	0.018473	1.024897
-0.10609	0.223848	-0.44455	-1.17361	-0.5924	-0.83582	-0.79919	0.000693	1.397461
-0.10609	-0.21643	-0.60294	-0.64401	-0.20219	-0.51485	-0.63193	0.051568	0.441825
-0.11449								
-0.11449	1.165423	1.078702	1.54474	0.852267	0.885017	0.700492	0.04451	0.844248
0.351487	0.413652	-0.00559	-0.08755	0.207542	0.210451	-0.00234	0.24747	0.501204
0.351487	-0.72626	-0.81773	-0.18746	-0.45917	-0.55755	-0.82527	0.094819	0.637815
2.215622	4.219163	3.778323	3.190007	3.198893	3.628005	3.002437	0.027232	1.216727
0.351487	0.963757	1.38994	1.56943	1.147976	1.41298	1.1115	0.006967	0.605673
2.215622	0.917842	1.52429	1.71113	1.266497	1.488385	1.148943	0.011337	0.793288
2.215622	-0.55631	-0.91237	-0.98339	-0.5486	-0.4314	-0.39224	0.01266	0.591151
2.215622	0.071228	0.290059	0.237413	-0.592	0.089183	0.217424	0.000202	0.88206
2.044368	0.162913	0.665286	0.571595	-0.08062	0.060061	0.332842	0.005537	0.745907
2.044368	0.020218	-0.16488	-0.3342	0.115834	0.517138	0.270807	0.041059	0.851337
2.044368	-1.23505	-1.60952	-1.8503	-1.35268	-1.79477	-1.63429	0.004103	0.615253
2.044368	-1.23402	-1.62591	-1.57983	-1.13651	-1.45052	-1.19404	0.014269	0.489401
2.044368	0.452961	0.355312	0.24249	0.12382	0.549598	0.612127	0.008037	0.488307
0.11367								
0.11367	-0.6135	-0.99509	-0.7473	-0.35326	-1.28252	-0.74743	0.154949	0.929261
0.11367	-0.5884	-0.67265	-0.82992	-0.52247	-1.12831	-0.83317	0.028567	0.605844
0.11367	-0.59542	-0.79432	-0.87723	-0.67934	-0.71972	-0.63224	0.10019	0.281814
0.11367	-0.91372	-1.0967	-1.1414	-0.97129	-1.31657	-1.27474	0.201307	0.402846
-0.14596	0.776467	1.151603	1.019168	0.593367	1.30516	1.185291	0.003096	0.711793
-0.32152	1.60982	1.54106	1.678163	1.000312	2.034645	1.50995	0.008469	1.034333
-1.40584	0.188859	0.158782	0.656254	0.461883	0.448891	0.4029	0.063818	0.497472

-0.32152	-0.11676	0.059047	0.372487	0.478407	0.388971	0.165964	0.00751	0.595171
-0.58757	0.370758	0.951546	1.402983	0.883567	0.91607	0.712096	5.97E-05	1.032225
-0.58757	0.446284	0.378565	0.431849	0.266735	0.241938	0.139121	0.146132	0.307163
-1.40584	-0.40945	-0.33582	-0.67026	-0.01851	-0.78338	-0.11531	0.263825	0.764878
-1.40584	0.251129	0.411375	0.080195	-0.05142	0.183641	0.017214	0.015394	0.462794
-0.49912	0.410473	0.839006	0.861348	0.431171	-0.05244	0.164124	0.009741	0.913788
-0.49912	0.155448	0.279901	0.278472	0.072172	0.568325	0.384935	0.005255	0.496152
0.30251								
0.30251	0.038956	0.242859	0.297104	0.560454	1.072095	0.623908	0.002242	1.033139
0.30251	0.871303	0.005925	-0.18823	-0.00218	-0.03309	-0.1347	0.051044	1.059535
1.262037	0.822425	1.079757	1.313201	0.881153	1.15899	0.868924	0.004384	0.490777
0.120843	0.67916	1.072398	1.074843	0.787358	0.717571	0.700122	0.048957	0.395683
1.262037	-0.90323	-1.14022	-1.2308	-0.97372	-0.74891	-0.63625	0.040385	0.594551
1.262037	0.159387	0.044886	0.072653	-0.05066	0.51756	0.396049	0.038918	0.568217
-0.8126	3.663647	3.467027	2.94418	2.901407	2.995325	2.660923	0.056818	1.002723
-0.34412	1.014669	1.337127	1.099639	0.881135	0.860512	0.81937	0.024307	0.517757
-0.34412	-0.03454	-0.21228	-0.26285	0.017336	0.078313	-0.09661	0.035012	0.341163
-0.34412	-0.1178	0.266813	0.664384	-0.06605	0.702963	0.376243	0.001785	0.820764
-0.09261	0.942134	1.568219	1.852573	1.016489	2.001455	1.391727	0.037615	1.059321
-0.09261	0.541643	1.284869	1.51581	0.81343	0.827123	0.923806	0.005146	0.974167
-0.09261	-0.93015	-1.33658	-1.31227	-1.10252	-1.13432	-0.90503	0.090473	0.431543
-0.09261	-0.61114	-1.16422	-1.2367	-0.80232	-0.77258	-0.75029	0.004226	0.625564
1.485417	1.478903	1.731543	1.88769	1.27995	1.700185	1.613133	0.083625	0.60774
1.485417	0.156381	0.699098	1.113657	0.699853	0.403441	0.41623	0.001905	0.957275
-0.36255								
-0.77376	-0.02374	0.551179	0.841691	0.186836	0.327548	0.387583	0.008544	0.865428
-0.77376	0.875888	0.337873	-0.13291	0.873851	1.395955	0.203183	0.23282	1.528868
-0.77376	-0.52447	-1.03775	-1.16029	-0.62962	-0.70523	-0.54979	0.019356	0.63582
-0.77739	-0.08582	0.113138	0.166054	-0.03809	0.179675	0.197689	0.148376	0.283504
-0.77739	-0.68926	-0.79881	-0.77589	-0.72974	-0.90646	-0.69852	0.041114	0.217194
-0.77739	-0.85891	-1.38717	-1.65775	-0.76712	-1.26534	-1.01429	0.0131	0.890634
0.860043								
0.860043	-0.64506	-0.9812	-1.04379	-0.60219	-0.9179	-0.68456	0.079589	0.441595
0.860043	-0.39079	-0.63139	-0.87378	-0.28671	-0.31244	-0.2788	0.020533	0.594979
-0.3171								
-0.3171	0.403473	0.474879	0.547365	0.724122	0.46529	0.762572	0.132647	0.3591
-0.3171	0.850656	1.207633	1.466407	0.988522	1.041218	0.776326	0.025925	0.69008
-0.44791								
-0.44791	-0.9426	-1.53434	-1.72979	-1.12648	-1.4258	-1.00412	0.007812	0.787198
-0.44791	-0.73936	-1.5141	-1.50225	-0.93732	-1.05055	-0.90398	0.014382	0.774736
-0.44791	-1.16234	-1.93047	-1.9553	-1.26255	-1.63319	-1.31864	0.0009	0.79296
-0.44791	-1.20757	-1.84973	-1.9433	-1.43855	-1.78128	-1.4737	0.005553	0.73573
-0.54695	-0.12377	-0.13705	0.203415	0.159732	0.187249	0.828037	0.131034	0.965086
-0.65975	0.638439	0.618699	0.754847	0.497125	0.774836	0.462475	0.066389	0.312361
-0.54695	0.360554	0.799936	1.541397	0.774621	0.941663	0.656711	0.000175	1.180843
-0.65975	0.684309	1.100236	1.499293	0.97196	0.976494	0.781497	0.004264	0.814984
0.17272	0.062613	0.21288	0.311571	0.70338	0.362814	0.228942	0.053618	0.640767

0.17272	-0.24405	-0.31122	-0.39231	-0.26363	0.220877	-0.14325	0.039208	0.613186
-0.36818	-0.67473	-1.33911	-1.40212	-0.78903	-0.96797	-0.71631	0.000474	0.727394
-0.36818	-1.63264	-1.93931	-2.09771	-1.37075	-2.01289	-1.40701	0.002778	0.726958
-0.36818	-0.57194	-0.56359	-0.23985	-0.23092	-0.4539	-0.43053	0.002969	0.341023
0.216603								
0.216603	-0.53556	-0.76601	-0.83341	-0.46269	-0.75904	-0.50787	0.026864	0.370725
0.216603	-0.91268	-1.06163	-1.07002	-0.41603	-0.85609	-0.74711	0.076164	0.653989
0.601233								
0.601233	0.806046	1.168737	1.377187	1.394163	1.51523	1.382939	0.001934	0.709184
0.601233	0.113933	-0.1116	-0.41993	-0.25376	0.128834	0.259762	0.029151	0.679692
0.1147	3.262007	3.038606	2.328028	2.480807	2.442635	2.10921	0.006998	1.152797
0.1147	-1.05004	-1.62534	-1.72656	-1.14232	-1.3412	-1.25218	0.009982	0.676523
0.1147	-0.86197	-1.50526	-1.60432	-0.64015	-0.88313	-0.67879	0.000172	0.964176
0.598883	0.961209	1.084771	1.468233	1.112089	1.405675	1.076427	0.06283	0.507024
0.598883	-0.99225	-1.41235	-1.46603	-0.97923	-1.15622	-1.02342	0.025691	0.486802
0.598883	-0.52029	-0.6253	-0.5642	-0.40674	-0.55017	-0.45103	0.12295	0.21856
-0.39277								
-0.39277	-1.02025	-1.71164	-1.79246	-1.25834	-1.42291	-1.14765	0.00136	0.772204
-0.39277	-0.59173	-0.93837	-1.22563	-0.60019	-0.70446	-0.5043	0.030081	0.721332
-0.41976								
-0.41976	-0.63686	-1.06445	-0.97535	-0.77604	-0.9134	-0.66243	0.014982	0.427588
-0.41976	0.244294	0.642745	1.006417	0.429755	0.733855	0.513551	0.000502	0.762123
-0.51569	0.530175	0.580529	0.375083	0.447595	0.693524	0.23421	0.031364	0.459314
-0.51569	0.433933	0.845396	1.249775	0.944845	1.164565	0.691261	0.022153	0.815842
0.992237	0.679261	0.74432	1.027008	0.85773	1.223805	1.06647	0.069187	0.544544
0.992237	-0.0029	0.182105	0.241883	0.211079	0.544529	0.495642	0.047127	0.547431
0.992237	-0.8551	-1.4663	-1.61367	-0.96622	-1.32288	-1.13917	0.006779	0.758566
0.992237	-0.18338	0.322112	0.287665	0.703534	0.249125	0.484285	0.11484	0.886917
-0.30424	0.204552	0.400882	0.8829	0.295812	0.176176	0.347418	0.033371	0.706724
-0.30424	-0.47498	-0.70856	-0.49564	-0.32253	-0.52043	-0.58968	0.027305	0.386034
-0.30424	-0.96473	-1.2189	-1.2414	-0.96463	-1.29923	-1.11187	0.032583	0.334599
0.133487	0.704787	0.430649	0.325781	0.7056	0.633605	0.582372	0.02698	0.379818
-0.10836	-0.82617	-1.25528	-1.28226	-0.74571	-1.01693	-0.82658	0.046361	0.536543
-0.10836	-0.20023	-0.26648	-0.22699	0.068614	0.214942	0.061398	0.00555	0.481419
-0.62915	0.398853	0.816426	0.585879	0.203725	0.777042	0.776659	0.000634	0.612701
0.364757	-0.16534	-0.18833	-0.56744	-0.32613	-0.16581	-0.15942	0.040832	0.408026
-0.19289	0.105998	0.201668	-0.09712	-0.13995	-0.00835	0.046167	0.011383	0.341618
-0.19289	1.02266	1.289425	1.045486	0.883037	1.424645	1.094198	0.023778	0.541608
0.16163								
0.16163	0.502266	0.917689	1.23461	0.862143	0.849047	0.619201	0.000153	0.732344
1.626458	0.33072	0.693847	1.122028	0.372688	0.584225	0.79702	0.015534	0.791308
1.626458	-1.04098	-1.3307	-1.62032	-0.9576	-1.25273	-0.99761	0.033942	0.662717
1.626458	-1.15357	-1.93772	-2.0154	-1.05734	-1.48648	-1.20753	0.003062	0.958063
1.626458	0.982749	1.045422	1.30073	1.02195	1.413225	1.197433	0.019248	0.430476
-0.01111	0.079406	0.05346	0.261133	0.45309	0.633609	0.377803	0.04026	0.58015
-0.01111	1.207057	0.619072	0.32664	0.286598	-0.42155	-0.0525	0.037719	1.628608
-0.01111	-0.2621	-0.28709	-0.15682	0.164975	0.123108	-0.13174	0.015167	0.452066

-1.25673	0.339133	0.578135	0.853938	0.286101	0.476588	0.407801	0.139952	0.567837
-0.6989	3.214347	2.75493	2.121687	2.050513	2.812165	2.136023	0.027735	1.163833
-1.25673	2.590523	2.585877	2.314677	2.005073	2.149655	1.82362	0.043278	0.766903
-0.6989	-1.23225	-1.3055	-1.5131	-1.02234	-1.22159	-1.24072	0.004728	0.490761
-0.6989	-0.73335	-0.43154	-0.19374	-0.28376	-0.31244	-0.5601	0.092012	0.53961
-0.26747	1.386133	1.75149	1.900783	1.398577	2.014115	1.593583	0.012784	0.627982
-0.26747	-0.01818	0.08764	0.264966	0.259577	0.318902	0.234884	0.043103	0.337078
0.9106								
0.9106	1.368674	0.766353	0.104122	0.121834	0.390617	0.538531	0.118372	1.264552
-0.18031	-1.27875	-1.83211	-2.05557	-1.4259	-1.80689	-1.55244	0.014791	0.776813
-0.18031	-1.36061	-1.65745	-1.85196	-1.35847	-1.57422	-1.35647	0.020266	0.49549
-0.18031	-0.71607	-1.15015	-0.93267	-0.77972	-0.77143	-0.68539	0.133043	0.464755
1.76037	-1.41528	-1.5739	-1.78179	-1.28476	-1.35075	-1.4704	0.060693	0.497033
1.76037	0.736851	0.974325	0.702377	0.118274	0.521003	1.025051	0.026327	0.906776
-0.42035	0.484912	0.656882	-0.77887	-0.46485	-0.2203	-0.35931	0.04432	1.435753
-1.06731								
-0.42035	1.013863	1.507387	1.71453	1.351817	0.992755	0.805672	0.002359	0.908858
-1.06731	0.783663	1.13587	1.452603	0.968145	0.640415	0.823842	0.031553	0.812189
0.27171								
-0.54783								
-0.54783	0.971186	1.522523	1.75253	0.806963	1.381625	1.098373	0.033893	0.945567
0.27171	0.048398	0.185247	-0.00239	0.510384	0.824151	0.649732	0.017872	0.826543
-1.08754	0.290929	0.284849	-0.10614	-0.70807	1.309685	1.213613	0.007207	2.017758
-1.08754	0.458137	0.568658	0.480715	0.209679	0.563988	0.694433	0.003311	0.484754
-0.26853	0.246346	0.735111	1.294909	0.449153	1.12783	0.703121	0.039662	1.048563
-0.26853	-0.60483	-0.70485	-0.62812	-0.71922	-0.51445	-0.57494	0.182114	0.204764
-0.26853	-0.44411	-0.59367	-0.51267	-0.55513	-0.46602	-0.33775	0.074166	0.255923
-0.09096								
-0.09096	-0.02654	0.000445	-0.11884	-0.04731	0.223226	0.156506	0.01172	0.342068
-0.09096	0.390749	0.456035	0.585575	0.834162	0.768023	0.551962	0.003745	0.443413
-0.60013	0.021113	0.532617	0.517278	-0.00058	0.534492	0.355531	0.071797	0.535071
-0.60013	-0.64891	-0.84127	-0.7223	-0.63473	-0.7067	-0.60908	0.130348	0.232189
-0.60013	0.171286	0.390065	0.426071	0.146457	0.380402	0.421839	0.021668	0.279614
-0.60366	0.840296	1.24103	1.40966	0.853708	1.48308	1.091963	0.00268	0.642784
-0.60366	1.235384	1.551118	1.441093	1.35879	0.82437	0.785587	0.094332	0.765531
-0.60366	0.337512	0.557056	1.026781	0.487796	0.373698	0.347097	0.001478	0.689269
-0.82044	0.261514	0.566839	1.026141	0.287141	0.545545	0.515204	0.014446	0.764626
-0.82044	-0.02174	-0.11159	-0.91344	-0.78056	-0.38582	-0.46142	0.015199	0.891696
-0.82044	-0.6421	-0.9234	-1.24157	-0.74603	-0.9073	-0.79652	0.01205	0.59947
-0.82044	-0.06105	0.33076	0.868496	0.506113	0.18635	-0.29468	0.001761	1.163176
-0.21654	1.041376	1.642807	1.78706	0.920241	1.67635	1.28164	0.012651	0.866819
-0.21654	0.943779	0.33783	0.168319	0.217601	0.011133	-0.0744	0.057393	1.018174
-0.02993	0.232705	0.633652	0.974006	0.791344	1.125937	0.927453	0.032251	0.893232
0.005905	0.98489	-0.58028	-1.1636	-0.90569	-1.0658	-0.69918	0.003544	2.148488
0.005905	-0.43654	-0.78804	-0.87472	-0.69938	-0.74511	-0.73971	0.170218	0.438181
0.005905	0.416807	1.297502	1.038435	1.480439	0.534935	1.393433	0.288999	1.063633
-0.17177	0.927134	1.453537	1.691287	0.942865	1.47858	1.264513	0.001983	0.764153

-0.17177	-0.83582	-0.77365	-0.47911	-0.37087	-0.90509	-0.77755	0.039191	0.534223
-0.17177	2.385073	2.382207	1.822627	1.977073	1.63547	2.178547	0.05145	0.749603
-1.26733								
-1.26733	0.03392	0.856053	1.3036	0.724751	0.005739	-0.21676	0.006001	1.520358
-0.8216								
-0.8216	1.013412	1.283187	1.526873	1.080927	1.045535	0.846887	0.037778	0.679987
-0.64144								
-0.64144	0.103254	0.141997	0.068782	0.350556	0.632205	0.307264	0.051488	0.563423
-0.64144	1.293603	1.559227	1.657533	1.526697	1.29167	1.061047	0.004387	0.596487
0.056157	-0.44641	-0.32757	-0.41685	-0.27169	-0.10852	-0.157	0.1135	0.337889
0.056157	0.303854	0.817184	0.276113	0.546444	0.916354	0.009038	0.164405	0.907316
-0.80597	0.645494	0.712773	0.910816	0.650509	0.418146	0.589372	0.040887	0.49267
-0.80597	-0.83129	-1.42234	-1.31219	-0.77968	-0.79952	-0.64496	0.010945	0.777378
-0.80597	-0.84401	-1.38734	-1.70047	-1.11471	-1.26576	-1.22027	0.000573	0.856455
-0.80597	0.343179	-0.12388	0.195714	0.299437	0.620339	0.456682	0.001119	0.744215
-0.52003								
-0.52003	-1.31157	-1.80507	-1.91303	-1.25418	-1.51304	-1.40348	0.002653	0.658847
-0.52003	2.644767	2.28223	1.441552	1.728743	2.059925	1.522133	0.145796	1.203214
0.48419								
0.48419	1.070311	0.978768	1.160012	0.849949	1.662695	1.46112	0.008143	0.812746
0.48419	-0.68556	-1.02388	-1.10274	-0.7541	-0.78414	-0.71488	0.030016	0.417171
0.48419	0.434051	0.376647	-0.14499	0.150045	-0.44809	0.107166	0.039965	0.882137
0.48419	-0.92236	-1.71682	-1.98414	-1.27659	-1.40753	-1.21985	0.000994	1.061788
0.48419	-0.71508	-1.2202	-1.48892	-0.74854	-1.16146	-0.82225	0.005774	0.773839
0.48419	-1.73958	-2.18224	-2.45768	-1.60615	-2.04299	-1.59001	0.003516	0.867667
0.48419	-0.36837	-0.97627	-1.50563	-0.98666	-0.99751	-0.96859	0.001027	1.137259
0.48419	2.042307	2.04794	1.752307	1.60576	1.85785	2.018017	0.067996	0.44218
0.13482								
0.13482	-0.59152	-0.90849	-1.47241	-0.96819	-1.2108	-1.15312	0.003616	0.88089
0.13482	0.961248	-0.18631	-0.51187	-0.23762	-0.4392	-0.43714	0.061981	1.47312
-0.1016	0.138502	-0.07505	0.235577	0.427354	0.467755	0.367563	0.008731	0.542804
-0.50864								
-0.50864	-1.01803	-1.38444	-1.46524	-0.93169	-1.07647	-0.89809	0.032322	0.567147
-0.50864	-0.45806	-0.62516	-0.597	-0.45926	-0.58033	-0.54144	0.120518	0.1671
-0.50864	-0.51494	-1.01658	-1.31413	-0.88041	-1.24201	-0.93626	0.015653	0.799182
-0.1016	-0.72617	-0.66727	-0.11685	-0.40603	-1.00503	-0.88591	0.01653	0.888177
-0.1016	1.7932	1.863657	2.156433	1.97806	2.15487	1.66216	0.018616	0.494273
0.44825								
0.44825	-0.02084	-0.34045	-0.77903	-0.58532	0.115782	0.356698	0.005347	1.13573
0.867353	1.112477	0.809893	0.702949	0.706145	1.099331	0.549946	0.114159	0.562531
0.867353	-0.93626	-1.06929	-1.42042	-1.14484	-1.13345	-0.89839	0.01371	0.522028
0.867353	-0.73786	-1.12126	-1.1351	-0.96675	-1.18329	-0.92204	0.002842	0.445433
0.747832	1.467282	1.666736	1.623989	1.163366	2.304522	1.887717	0.064953	1.141156
0.747832	-0.34408	-0.44796	-0.61724	-0.33813	-0.4633	-0.28523	0.017693	0.332012
0.903695	0.08374	0.109704	0.499271	0.05643	0.832314	0.775374	0.001248	0.775884
0.903695	-0.86926	-1.31718	-1.7012	-1.16889	-1.39123	-1.16069	0.007109	0.831941
0.903695	-0.91384	-1.60134	-1.73362	-1.00919	-1.37446	-1.07376	0.000388	0.81978

0.903695	-1.05279	-1.60205	-1.80833	-1.43372	-1.60532	-1.4489	0.002076	0.75554
0.903695	-0.9387	-1.14878	-1.07322	-0.83632	-0.96821	-0.79349	0.004397	0.35529
-0.23124								
-0.23124	0.356836	0.128352	0.474571	0.647988	0.645934	0.700611	0.02193	0.572259
-0.88757								
-0.88757	-0.38508	-0.27854	-0.53848	-0.17197	-0.41453	-0.4212	0.03506	0.366511
-0.88757	-0.108	0.066077	0.158712	-0.04565	-0.53062	-0.31718	0.005776	0.689336
	0.517398	0.940998	0.678958	0.547087	1.058843	0.682424	0.03793	0.541445
0.746147	0.674163	1.099518	1.319561	0.846094	1.47559	1.303247	0.038919	0.801427
0.746147	0.93228	1.220527	1.33528	0.941513	0.759192	0.931047	0.014423	0.576088
1.332213	1.63762	1.683333	1.74094	1.36322	1.70837	1.670413	0.009459	0.37772
2.564393								
2.564393	-0.78206	-1.33517	-1.50403	-0.83197	-1.00754	-0.85114	0.005047	0.721962
2.564393	-0.47044	-0.89609	-1.12933	-0.5549	-0.54919	-0.49943	0.008497	0.658893
2.564393	-0.37371	-0.91324	-0.92537	-0.28071	-0.42531	-0.2211	0.055226	0.70427
0.61739	0.304324	0.741428	1.024271	0.576805	0.738198	0.360221	0.003503	0.719948
0.61739	1.098549	0.754316	1.037427	0.524066	0.241711	0.844496	0.201549	0.856838
0.61739	-0.67748	-1.29745	-1.74411	-0.7974	-1.29343	-1.00418	0.009463	1.06663
0.61739	0.115454	-0.14062	-0.739	-0.34618	-0.34193	-0.43084	0.003527	0.854459
0.330293								
0.330293	0.163694	0.607874	0.879422	0.248864	0.169051	-0.34781	0.008639	1.227234
0.860857	0.142907	0.187867	0.27207	-0.78652	-0.14673	0.407446	0.08612	1.193968
0.860857	0.128554	-0.00816	0.543373	0.427098	0.246825	0.094462	0.078926	0.551537
0.860857	0.448669	0.750196	0.270439	0.773862	1.23685	1.023992	0.014612	0.966411
-0.21126								
-0.21126	0.019185	-0.26685	0.323568	0.378863	-0.2206	-0.13397	0.058625	0.645712
-0.21126	0.07673	-0.05752	0.049134	-0.05544	-0.41744	-0.29434	0.010028	0.494166
1.417487	0.237699	0.457918	0.546973	0.187291	0.240818	0.559642	0.049971	0.372351
1.417487	-0.38211	-0.57847	-1.00569	-0.86785	-0.45414	-0.4469	0.019209	0.62358
1.417487	0.941375	0.867743	0.428429	1.063146	1.267665	1.211363	0.001376	0.839236
-0.32824	0.186947	0.035235	0.38067	0.02562	0.825886	0.207122	0.019287	0.800266
-0.32824	1.363694	2.18074	2.175033	1.282007	1.563185	1.785697	0.022252	0.898733
0.823398	0.54107	0.644821	1.118168	1.003943	1.12433	0.838587	0.005737	0.58326
0.823398	1.37339	1.672899	-0.16173	-0.3196	-0.05256	0.223264	0.119218	1.9925
1.55977								
1.55977	1.086588	1.075573	0.906652	0.534352	0.644668	0.370318	0.068727	0.716271
1.55977	-0.60914	-1.01788	-1.0066	-0.92375	-0.81281	-0.69222	0.017312	0.408731
-0.67425								
0.76993	-0.33931	-0.89781	-1.16391	-0.35087	-0.52547	-0.41997	0.000285	0.824596
-0.67425	-0.4822	-0.96338	-1.17684	-0.49867	-0.67143	-0.52925	0.001635	0.694634
-0.67425	-0.7134	-1.45013	-1.50608	-0.94094	-1.31753	-0.93443	0.018954	0.792673
-1.2652	3.131953	3.491764	2.798285	2.877633	2.438401	2.162777	0.013966	1.328987
-1.2652	0.980305	0.972542	0.6645	0.759293	0.81405	0.797547	0.125497	0.315805
0.20602								
0.20602	1.21088	1.21379	0.949578	0.516241	0.534321	0.779789	0.00899	0.697549
0.20602	0.683847	0.659514	0.631134	0.300161	0.691901	0.40764	0.193512	0.39174

0.46772	0.471364	0.959802	1.464523	1.456337	1.40114	0.969291	0.000836	0.993159
2.918667	0.532557	0.84158	0.88831	0.199522	0.141149	0.509083	0.073676	0.747161
2.918667	-0.39095	-0.47475	-0.60917	-0.26086	-0.92176	-0.2945	0.200884	0.660898
2.918667	-0.60577	-1.1225	-1.31499	-0.96982	-1.25934	-1.04445	0.004918	0.709223
0.46772	0.864291	1.052677	1.331676	0.767321	1.459935	1.014106	0.016263	0.692614
-0.46306	0.44818	0.541107	0.823506	0.469817	0.817108	0.825767	0.051689	0.377587
-0.46306	-0.21345	0.612966	0.8202	-0.25126	-0.72741	-0.549	0.000225	1.547607
0.5667	0.104465	0.180494	0.453577	0.142858	0.336828	0.55103	0.022577	0.446566
0.5667	-0.65013	-0.83564	-0.60495	-0.54972	-0.77652	-0.72094	0.049231	0.285926
0.5667	0.108725	0.046605	0.370666	0.424695	0.539636	0.355946	0.030429	0.493031
-0.73597								
-0.73597	0.056145	0.099835	0.298606	0.162942	0.420408	0.326254	0.008024	0.364263
-0.73597	0.480802	0.104717	-0.56885	-0.31775	-0.28375	-0.38165	0.078316	1.04965
-1.0692								
-1.0692	0.431033	0.957517	1.482543	1.33453	0.456543	0.477784	0.000255	1.05151
-0.85165								
-1.11829								
-1.11829	-0.51157	-0.99938	-1.38928	-0.86404	-1.23327	-0.94752	0.066021	0.877711
-1.11829	-1.07652	-1.35554	-1.72642	-1.05669	-1.29631	-1.14692	0.020026	0.669732
-0.85165	1.249427	1.242902	1.392006	0.869072	1.172676	1.353593	0.185561	0.522934
-0.31095								
-0.31095	0.212714	0.26159	0.110024	0.149626	0.603729	0.367054	0.068795	0.493704
-0.31095	-0.28544	-0.32495	-0.61299	-0.82855	-0.71612	-0.4049	0.008552	0.543109
-0.03345	0.985254	0.955398	1.225812	1.2379	1.230424	0.889611	0.076077	0.348289
-0.03345	-0.94892	-1.00337	-1.18182	-1.05626	-1.06343	-1.00193	0.026349	0.232896
-0.03345	-0.07826	-0.87559	-1.22195	-0.82711	-1.14397	-0.98283	0.03741	1.14369
-0.03345	-0.52005	-0.92314	-0.7186	-0.56336	-0.42037	-0.39196	0.025091	0.531177
-0.75781	-0.81013	-0.70679	-0.21593	-0.50695	-0.50387	-0.57563	0.053439	0.594193
-0.75781	1.979343	2.205349	1.7066	3.130083	2.314765	1.60223	0.093896	1.527853
-0.5383								
-0.5383	-0.34362	-0.80005	-0.88157	-0.76795	-0.74357	-0.79086	0.078104	0.537952
-0.5383	1.193243	1.45189	1.878597	1.369588	1.048875	1.351063	0.027797	0.829722
1.221847								
1.221847	0.770085	0.628404	0.76369	0.679617	0.68803	0.903523	0.073991	0.275119
-0.107								
-0.107	0.471978	0.810839	1.066543	0.840787	0.877802	0.642705	0.003405	0.594565
0.149537								
0.149537	-0.82435	-1.68394	-1.87478	-1.37432	-1.57632	-1.39381	0.00988	1.050433
0.149537	-0.89178	-1.20696	-1.43639	-0.90571	-1.17984	-1.04318	0.015367	0.544609
0.63231	0.247363	0.353855	0.299211	0.378827	0.587531	0.369923	0.029406	0.340168
0.63231	0.334734	0.666478	0.490837	0.112652	1.135167	0.764512	0.088845	1.022515
0.63231	0.524336	0.876816	0.90459	0.724565	1.354085	0.834677	0.002019	0.829749
0.1991	0.745357	0.919654	1.405013	0.594257	1.114011	0.992733	0.017745	0.810756
0.1991	-0.94725	-1.29993	-1.45632	-0.96053	-1.16764	-0.93152	0.01685	0.524798
0.1991	-0.72661	-0.27351	-0.26376	0.054412	-0.67746	-0.41204	0.024386	0.781021
-1.18259	0.057686	-0.02115	-0.37366	-0.31114	0.113775	0.514676	0.018019	0.888334
-1.18259	0.203534	0.168688	0.42719	0.365198	1.19113	0.118627	0.016135	1.072503

-1.18259	0.938001	1.595173	1.9528	1.045325	1.315405	0.570668	0.012694	1.382132
-0.53074	-0.01156	0.358278	0.569269	0.357539	-0.10782	-0.08106	0.004587	0.677094
-0.53074	-0.2765	-0.40581	0.124233	0.499713	0.098531	0.013983	0.000524	0.90552
-0.53074	0.478144	0.708767	1.49159	1.296097	1.216565	0.804192	0.000538	1.013446
-0.53451	-0.5572	-0.5816	0.099262	0.075766	-0.36971	-0.41904	0.0031	0.680863
0.530788	-0.31977	-0.51627	-0.17694	0.051573	-0.06566	-0.27557	0.02094	0.567838
-0.53451	-0.65	-0.73479	-0.55917	-0.2568	-0.11883	-0.40771	0.023324	0.615957
-0.53451	0.234629	0.3605	0.401354	0.693747	0.717904	0.418328	0.004487	0.483275
0.530788	0.239189	0.369025	0.407326	0.560069	0.66715	0.626906	0.103207	0.427961
0.530788	1.16696	1.076415	1.092297	0.575992	1.66746	1.248191	0.02775	1.091468
-0.06019	0.728006	1.341227	1.261013	0.675945	0.949552	0.631028	0.014697	0.710199
-0.13727								
-0.13727	-0.02881	-0.35167	-0.5851	-0.25788	-0.47415	-0.03656	0.10509	0.556288
-0.13727	-0.19328	-0.39352	-0.56094	-0.33836	-0.30826	-0.1823	0.032437	0.378636
3.211163	1.880937	1.889387	1.603583	1.178965	1.073445	1.043081	0.042855	0.846306
3.211163	0.793495	0.641714	0.714355	0.368531	1.03541	1.245279	0.009147	0.876748
3.23345	0.565778	1.19452	1.38194	0.521608	0.788911	0.864631	0.001554	0.860332
3.23345	-0.96806	-1.52304	-1.41415	-0.96204	-1.14411	-0.97714	0.006937	0.561003
0.212933								
0.165002	0.296226	0.149543	0.554079	0.716174	0.892846	0.705191	0.020001	0.743304
0.165002	-0.80613	-0.89696	-1.1891	-0.78432	-0.47466	-0.78781	0.006323	0.71444
0.165002	0.242032	0.199169	-0.37092	-0.10824	-0.17057	0.188013	0.08257	0.612952
0.165002	-0.30004	-0.38198	-0.05871	-0.07052	-0.60142	-0.42873	0.023979	0.542709
0.212933	-0.05054	0.146665	0.504857	0.610577	0.622143	0.368483	0.056385	0.67268
0.212933	-0.546	-0.65574	-0.24789	-0.16136	-0.07815	-0.16375	0.021574	0.577595
0.226937	0.597875	0.678152	0.50974	0.262757	0.755072	0.673131	0.046115	0.492315
0.226937	-0.44499	-0.64489	-0.57454	-0.07682	-0.5208	-0.17361	0.069702	0.56807
0.226937	0.018044	-0.30507	-0.58658	-0.37624	-0.42373	-0.44913	0.153472	0.60462
0.303257	1.036516	1.41398	1.29262	1.106654	1.452775	1.44344	0.010832	0.416259
0.093093								
0.093093	1.987973	3.0232	1.809793	1.576707	1.51517	1.43105	0.073901	1.59215
-0.94622								
-0.94622	0.094624	0.351974	1.12173	0.781047	0.457701	0.211617	0.001154	1.027106
-0.94622	0.534126	1.496717	1.885277	1.031183	1.290625	1.01652	0.014727	1.351151
-0.95489	-0.52263	-0.70459	-0.66943	-0.17989	-0.46117	-0.49301	0.007527	0.524699
-0.95489	0.244114	0.909192	1.285373	0.721758	0.482841	0.105721	0.014756	1.179652
-0.78202								
-0.78202	-0.89644	-1.62149	-1.42983	-0.82064	-1.19477	-1.03045	0.007161	0.800848
-0.78202	0.455576	1.037003	1.34934	0.916249	0.862365	0.641086	0.00076	0.893764
-0.52178	0.185386	0.540427	0.386509	0.1372	0.291488	0.193081	0.015499	0.403227
-0.52178	2.139903	2.053047	1.309582	1.49011	1.75427	1.170597	0.085896	0.969306
-0.52178	0.939471	1.360337	1.775923	1.258425	1.536465	1.15137	0.012744	0.836452
-0.14924								
-0.14924	-1.2924	-1.84761	-1.86449	-1.25769	-1.67708	-1.3838	0.02327	0.606796
-0.14924	-1.28704	-1.87398	-2.09026	-1.38411	-1.66004	-1.52511	0.000651	0.803223
-0.14924	-1.15515	-1.75417	-1.90914	-1.28281	-1.63283	-1.32097	0.01261	0.753991
-0.14924	-0.20748	-0.36678	-0.18645	-0.03923	0.232371	0.071313	0.003864	0.599155

-0.14924	1.356713	1.70769	1.992153	1.57947	2.01427	1.563933	0.012222	0.657557
2.645743	5.76058	5.97816	5.062237	5.276147	5.27355	4.653097	0.089689	1.325063
2.645743	0.810155	0.518231	0.039051	0.707629	0.24847	0.116711	0.005649	0.771104
2.645743	-0.3696	-0.0897	0.049492	0.123321	-0.34145	-0.39025	0.005427	0.513575
0.038107								
0.038107	1.514723	1.984727	2.202393	1.458853	1.74568	1.510157	0.027384	0.74354
-0.77112	0.363614	0.454	0.3539	-0.15095	0.677779	0.692262	0.027738	0.843207
-0.77112	1.727783	1.98239	2.34832	1.555767	1.51861	1.569523	0.005208	0.82971
-0.79062								
-0.79062	1.503673	1.357807	0.491465	1.057486	0.504191	0.589179	0.007244	1.012208
-0.79062	0.48874	0.936879	1.143384	0.924603	0.200649	0.318851	0.014748	0.942735
0.224872								
0.224872	0.189193	0.110439	0.24167	0.43054	-0.37371	0.062362	0.020939	0.804248
0.987057	1.285603	1.495977	1.515843	1.067698	1.517125	1.46411	0.025572	0.449427
-0.00119	0.933449	1.379725	1.58913	1.121812	1.41582	1.262238	0.012835	0.655681
0.054477	0.719507	1.068205	1.250861	0.830659	1.316605	1.07484	0.003453	0.597098
0.054477	0.570565	0.727998	0.736049	0.456671	1.38497	0.989855	0.008235	0.928299
-0.00119	0.178349	-0.20211	-1.35805	-0.84241	-1.05607	-1.00967	0.051404	1.536396
-0.00119	0.488635	0.819045	0.872792	0.561505	1.32781	1.029465	0.002916	0.839175
-0.72493								
-0.72493	-1.04403	-1.69136	-1.71934	-1.15088	-1.6707	-1.2798	0.014789	0.675306
-0.72493	0.219638	0.318115	0.022441	-0.04234	-0.05895	-0.06354	0.065604	0.381657
0.100213								
0.100213	-1.28376	-1.73416	-1.99373	-1.43581	-1.80678	-1.42895	0.00504	0.709973
0.100213	-1.11161	-1.57529	-1.82724	-1.2142	-1.54143	-1.29472	0.00696	0.71563
-0.26395	-0.36225	-0.83745	-0.89405	-0.6068	-1.20781	-0.79887	0.018925	0.845552
-0.26395	-1.34495	-1.74346	-1.6661	-1.53038	-1.52561	-1.53729	0.058258	0.398513
-0.26395	0.041725	-0.19261	0.239709	0.230779	-0.07032	-0.06313	0.114892	0.432315
-0.66356								
-0.66356	-0.26985	-0.28021	-0.49633	-0.34903	-0.42795	-0.37479	0.007739	0.226486
-0.66356	1.13277	1.660717	1.58745	1.546957	1.383465	1.160402	0.074044	0.527947
0.263647								
0.374707	1.023416	1.581303	1.767033	0.981264	1.143006	1.075881	0.005767	0.785769
0.263647	4.00362	3.991933	3.311187	3.129363	2.867805	2.962617	0.042508	1.135815
-1.23118								
-1.23118	1.289017	1.857977	1.98655	1.368557	0.933214	0.851937	0.005046	1.134613
1.85751								
1.85751	-0.85026	-1.1421	-1.1387	-0.62361	-0.78133	-0.69583	0.006857	0.518488
1.85751	0.177609	0.211603	-0.36236	-0.66568	0.41982	0.519193	0.001542	1.184871
1.813897	0.149391	0.223452	0.366726	-0.0884	0.349995	0.005602	0.162063	0.455122
1.813897	-0.96002	-1.51772	-1.5582	-0.9986	-1.35307	-0.99605	0.00538	0.598176
1.813897	-0.31789	-0.46125	-0.74972	-0.57979	-0.39739	-0.18944	0.024015	0.560279
-0.60409	2.544253	2.108281	1.429997	1.52162	1.97836	1.463365	0.118589	1.114256
-0.60409	3.79396	2.9682	1.862823	2.222573	1.447955	2.969317	0.068918	2.346005
-0.48205								
-0.48205	1.17701	1.44109	1.508227	1.173783	0.641482	0.77046	0.020914	0.866745
0.98351	1.669227	1.707293	1.625347	1.195883	1.580685	1.413253	0.052355	0.51141

0.98351	-0.84707	-1.18673	-1.41452	-0.90861	-1.09135	-0.8996	0.002155	0.567455
0.98351	0.28688	0.211587	0.344299	0.455516	0.305282	0.267427	0.116459	0.243928
-0.0785	-0.18493	0.435508	0.687955	0.470975	0.716635	0.372931	0.002405	0.901564
-0.43368	-0.38267	0.034582	0.493842	0.23102	0.228107	-0.15703	0.094722	0.876513
-0.43368	-0.42128	-0.07776	0.567522	0.438975	0.25536	-0.06427	0.002673	0.988799
-0.43368	-0.51642	-0.45313	-0.34284	-0.40705	-0.17301	-0.08118	0.116529	0.435237
-0.0785	0.499382	0.876415	0.75801	0.737525	0.970045	0.724124	0.05623	0.470663
-0.68898								
-0.68898	-0.5537	-0.73244	-0.84799	-0.67225	-0.82177	-0.70902	0.054223	0.294287
-0.68898	0.324644	0.189473	0.876848	0.445623	0.543273	0.311681	0.051679	0.687375
3.10509	0.482824	0.946025	1.313605	0.856388	1.026485	0.830657	0.034977	0.83078
3.10509	0.285681	-0.34933	-0.54106	-0.29286	-0.14282	-0.18888	0.023335	0.826745
3.10509	-0.65101	-1.14361	-1.19389	-0.73718	-1.26411	-0.92575	0.00746	0.613097
-0.85048	0.182637	-0.0875	-0.79264	-0.10818	-0.67371	-0.5543	0.000392	0.975273
-0.85048								
-0.58187								
-0.58187	2.38899	1.68444	1.345581	1.16103	3.415805	1.120655	0.068571	2.29515
-0.58187	-1.16491	-1.87415	-1.98794	-1.21244	-1.66233	-1.19563	0.01167	0.823026
-0.58187	-0.98199	-1.88966	-2.08433	-1.17297	-1.54586	-1.33861	0.018761	1.102343
-0.58187	-1.46917	-1.76264	-1.92211	-1.37905	-1.57439	-1.34653	0.015535	0.57558
-0.58187	-0.99719	-1.31255	-1.4681	-1.17007	-1.40943	-1.15868	0.024037	0.470909
-0.58187	0.038846	0.962712	1.062546	0.825493	-0.27574	1.104299	0.117342	1.380041
0.007893	0.599513	0.897878	1.106342	0.548461	0.56776	0.707789	0.004405	0.557881
0.007893	0.258746	0.381375	0.125179	0.491867	0.203587	0.375905	0.084047	0.366688
0.007893	0.435326	0.95753	0.717864	0.687235	0.327597	0.330546	0.206692	0.629933
0.241								
0.241	-1.01299	-1.28898	-1.18128	-0.35499	-1.23663	-0.64192	0.110991	0.933996
0.241	-0.86978	-1.3399	-1.45958	-0.73503	-1.00035	-0.818	0.014961	0.72455
0.532187	1.067965	1.211291	1.707813	1.052926	0.976576	0.946676	0.020034	0.761137
0.532187	-0.09577	0.073229	-0.29185	-0.21877	-0.61315	-0.27183	0.174326	0.686374
0.532187	1.881467	2.824637	2.567063	2.330943	1.96359	2.154667	0.088143	0.94317
-0.4997	-0.85022	-1.00334	-0.83431	-0.76752	-0.56582	-0.61726	0.011439	0.437523
-0.4997	0.459351	0.618277	0.751894	0.242404	0.561196	0.516895	0.011255	0.509491
-0.07502	0.263994	0.578517	0.81595	0.503566	0.202977	0.42056	0.149295	0.612973
-0.07502	1.324487	1.727607	2.083727	1.381791	1.210935	1.085472	0.063878	0.998255
-0.07502	1.065588	1.46367	1.740877	1.253297	1.641435	1.406293	0.002649	0.675289
-0.07804								
-0.07804	-0.75138	-1.25733	-1.44183	-0.83317	-1.26794	-1.00339	0.013933	0.690445
-0.07804	-0.42973	-0.11269	-0.46593	-0.58358	-0.52501	-0.49771	0.168201	0.470891
-0.72079	0.111371	0.52695	0.943747	0.73542	0.472094	0.176731	0.001357	0.832375
-0.72079	-0.99136	-1.41272	-1.7166	-1.13947	-1.49409	-1.31018	0.001561	0.72524
-0.72079	0.408634	0.469414	0.582111	0.860221	0.203807	0.437593	0.018354	0.656415
-0.81697								
-0.81697	-0.79581	-1.17005	-1.06191	-0.82674	-0.85266	-0.83109	0.007495	0.374233
-0.81697	0.692432	1.33213	1.819823	1.054088	1.035655	0.351757	0.003873	1.468066
0.412853								
0.412853	-0.22404	0.189288	0.196896	-0.02358	0.130723	0.024473	0.059135	0.420937

-0.30049	-1.31553	-1.46319	-1.78647	-1.26978	-1.41718	-1.2064	0.083187	0.580071
-0.30049	-0.16227	-0.03354	-0.20587	0.03019	0.316666	0.106731	0.012006	0.522534
-0.30049								
0.856597								
0.401087	0.470778	0.847104	0.609614	0.166918	0.792968	0.614774	0.008351	0.680186
0.401087	-1.34833	-1.21051	-1.29865	-0.63454	-1.02182	-1.04707	0.008522	0.71379
0.401087	-0.86611	-1.12984	-1.18925	-0.50669	-0.96499	-0.84546	0.073475	0.682562
0.856597	0.919042	1.134043	1.078945	0.85183	1.28189	1.29566	0.078923	0.44383
0.67995	0.176588	0.388221	0.674121	0.224565	0.855057	0.795612	0.016173	0.678469
0.049193	0.544237	0.537679	0.770505	0.436643	0.773942	0.750077	0.056204	0.337298
0.049193	-0.04007	-0.14577	-0.20669	-0.18314	-0.22503	-0.19283	0.064724	0.18496
0.049193	1.068954	1.058749	1.005816	1.37161	1.54652	1.31409	0.007596	0.540704
0.67995	-0.91362	-1.30562	-1.50363	-0.78093	-0.9415	-0.83441	8.87E-05	0.722701
0.67995	0.563998	0.289622	0.613259	0.740718	-0.03564	-0.02154	0.186829	0.776362
-0.92746								
-0.92746	0.216166	0.397997	0.700328	0.455491	0.234769	0.310126	0.046573	0.484162
-0.92746	0.935949	1.470077	1.62932	1.212687	0.695344	0.53572	0.024956	1.0936
0.85851								
1.20907	-0.37592	-0.4076	-0.40625	-0.26358	-0.06573	-0.27	0.024438	0.341874
0.85851	-0.27289	-0.42201	-0.66185	-0.39008	-0.56729	-0.52605	0.003727	0.388955
0.85851	0.520327	-0.01791	0.521322	0.515909	-0.108	0.237388	0.077659	0.629317
0.096083								
0.096083	-0.32574	-0.91819	-1.08556	-0.43809	-0.43824	-0.32411	0.121579	0.76145
0.096083	-0.48931	-0.3956	-0.34255	0.131088	-1.05073	-0.26161	0.189265	1.181821
0.096083	-0.81243	-1.29401	-1.48617	-0.88591	-1.26551	-1.08344	0.031952	0.673737
0.096083	-1.0559	-1.16955	-0.97143	-0.85175	-1.13891	-0.90891	0.021124	0.3178
0.096083	-0.45163	-0.66674	-0.9911	-0.58985	-0.80106	-0.66723	0.015734	0.53947
0.096083	-1.20457	-1.87473	-2.19676	-1.0748	-1.54581	-1.09445	0.011671	1.121953
2.03468	0.290279	0.541374	0.709054	0.229005	0.321659	0.478559	0.008635	0.480049
2.03468	0.46462	1.270747	0.826156	1.161103	1.3822	1.192309	0.00899	0.91758
2.03468	0.659651	0.81916	0.578172	0.327109	0.778704	0.824034	0.000659	0.496925
-0.89941	0.884125	1.486433	1.707327	1.33425	1.48772	1.024893	0.024962	0.823202
-0.6133								
-0.6133	-0.15623	-0.18556	-0.18756	-0.11026	0.172517	-0.02474	0.162785	0.360076
-0.6133	-0.50796	-1.25688	-1.34542	-0.44023	-1.07289	-0.70655	0.039379	0.905182
0.356318								
0.356318	0.059347	0.208324	-0.14017	-0.1644	-0.21192	-0.1733	0.028976	0.420244
0.356318	-1.00221	-1.35266	-1.78073	-1.06185	-1.47512	-1.35657	0.018013	0.778516
0.356318	0.159368	0.344043	1.004139	0.796166	0.738099	0.600481	0.002803	0.844772
-0.72477	0.213344	0.37485	0.402224	0.478667	0.441722	-0.01047	0.150784	0.489141
-0.72477	-0.71276	-0.65324	-0.3268	-0.08398	-0.72814	-0.78233	0.042311	0.698345
0.330027	0.587105	1.014257	1.181933	0.787426	0.47332	0.396338	0.002067	0.785595
-0.5742	0.403329	0.742096	1.12422	0.437131	0.322939	0.380299	0.008402	0.801282
-0.5742	1.633049	0.752367	0.621862	0.318877	1.434551	0.36777	0.123776	1.314172
-0.5742	1.91576	2.534263	2.296863	1.879523	1.973345	1.747447	0.014697	0.786817
0.014362	-0.51149	-0.69854	-0.89247	-0.52647	-0.67022	-0.55475	0.005191	0.380979
-1.09042	-0.00331	-0.38941	-0.68469	-0.2361	-0.47423	-0.38371	0.001111	0.681374

-1.09042	0.70168	0.523972	0.559737	0.45498	0.320107	0.631716	0.130693	0.381574
0.266557	2.016407	2.23092	2.397323	1.46855	2.49324	1.99268	0.011793	1.02469
-0.59995	2.358743	2.243148	1.56623	1.7703	1.598335	1.49777	0.034887	0.860973
-0.64125	1.037642	1.50103	1.62223	1.030307	1.824035	1.595393	0.015374	0.793728
-0.59995	1.601977	2.219213	2.2403	1.516523	2.463885	2.06358	0.014046	0.947362
0.266557	-1.18256	-1.78754	-1.66752	-1.11126	-1.51158	-1.2783	0.025562	0.67628
0.266557	0.792319	0.680312	0.991264	0.825027	1.19926	0.313796	0.012825	0.885464
-0.26442	0.374231	0.488705	0.743996	0.47652	0.752053	0.673371	0.019816	0.377822
-0.26442	-1.15365	-1.46945	-1.67183	-0.8983	-1.38347	-0.97182	0.004403	0.773532
-0.26442	-0.49151	-0.61919	-0.67339	-0.60892	-0.88074	-0.47072	0.079124	0.410023
0.760537	0.992664	0.845299	0.883583	0.537948	1.095385	0.951501	0.050822	0.557437
0.760537	1.015491	1.177108	1.327133	0.800052	1.43838	1.279557	0.00628	0.638328
-0.70985	0.455843	0.547147	0.655271	0.339526	0.258643	0.315392	0.095195	0.396628
-0.70985	0.266689	0.441022	0.177435	0.116208	0.776663	0.587049	0.009238	0.660454
-0.70985	-0.10728	-0.29702	-0.38876	-0.1342	-0.30368	-0.24962	0.075676	0.281473
-1.18042	0.997129	1.203407	0.892868	0.817226	0.57932	0.689456	0.071442	0.624087
-1.18042	2.064847	2.0025	2.29083	2.078783	1.704695	1.30549	0.011974	0.98534
0.083153	0.105972	0.065686	0.212957	-0.02888	-0.29363	0.083214	0.047446	0.506584
0.083153	-0.3308	-0.4129	-0.56531	-0.2804	-0.62147	-0.45458	0.026549	0.341065
0.083153	0.214036	0.208268	0.086459	0.088246	0.463898	0.365052	0.02132	0.377438
-0.245								
-0.245	1.45587	1.801523	1.625033	1.178307	1.057425	0.846495	0.010613	0.955029
0.439173	-0.62536	-0.64615	-1.15454	-0.70744	-0.77419	-0.81889	0.084	0.529176
0.439173	-0.24135	-0.13439	0.113534	-0.1861	-0.43531	-0.33687	0.015643	0.548848
0.124057	-0.10441	-0.39143	-0.75504	-0.25293	-1.05239	-0.5321	0.008242	0.947987
0.124057	0.617099	1.145247	1.440467	0.80582	1.294665	0.719296	0.002086	0.823367
-0.73721	1.7128	1.451869	0.898592	1.257539	1.31927	1.517921	0.067489	0.814208
-0.73721	-0.81079	-0.83525	-0.85492	-0.9207	-0.96925	-0.69296	0.022455	0.276286
-0.73721	-0.89684	-1.35275	-1.50514	-1.0923	-1.33368	-1.09317	0.002156	0.608301
-0.73721	-0.91729	-1.29349	-1.55343	-0.95478	-1.37058	-1.23131	0.002618	0.63614
-0.73721	1.039125	1.365467	1.726367	1.145466	1.3161	1.009342	0.013694	0.717025
-0.33321								
-0.33321	-0.55754	-0.38914	-1.04689	-0.60389	-1.0445	-0.90422	0.001864	0.657747
-0.33321	0.512893	0.890813	1.732853	1.26443	1.046565	0.785177	0.000202	1.21996
-1.0822	1.128209	2.013927	1.885447	0.622072	-0.13765	-0.16115	4.80E-05	2.175073
-0.78188								
-0.78188	0.758555	1.566413	1.66181	1.161397	0.113581	0.001831	0.00126	1.659979
-0.01278	0.05356	0.005391	0.055794	-0.1783	-0.31248	-0.29168	0.165199	0.368279
-0.01278	-1.12299	-1.64659	-1.72991	-0.97123	-1.61311	-1.26342	0.000859	0.75868
-0.01278	-0.59305	-0.70815	-1.20003	-0.54239	-0.6396	-0.71651	0.1201	0.657639
-0.01278	1.294998	0.294278	-0.03982	0.201383	0.372119	0.315857	0.034281	1.334815
-0.71891	0.090614	0.695401	1.238487	0.522976	0.454314	0.391698	0.00054	1.147873
-0.83871								
-0.01278								
-0.83871	-0.98482	-1.44527	-1.3169	-1.05595	-1.31396	-1.25046	0.00379	0.460448
-0.83871	-0.89994	-1.02295	-1.65682	-1.10033	-1.74709	-1.48177	0.074238	0.847149
-0.71891	-0.4708	-0.5244	-0.33639	0.038489	-0.22959	-0.26437	0.044707	0.562889

0.19243								
0.19243	-0.00644	0.096443	0.149844	-0.21284	-0.45659	-0.33304	0.070431	0.606437
-1.81298								
-1.81298	0.156831	0.324335	0.925011	1.14349	0.888275	0.522629	0.001954	0.986659
-0.45986	0.226258	0.434823	0.660208	0.627885	0.765823	0.39576	0.010604	0.539564
0.320127	-0.90152	-1.18032	-1.32266	-0.89086	-0.95161	-1.00442	0.021494	0.431798
0.320127	-0.04454	-0.73688	-0.33812	-0.61188	-0.43997	-0.56818	0.140514	0.692335
-0.15019								
0.320127	-1.25003	-1.59515	-0.73134	-0.01415	-0.40789	-0.73613	0.002847	1.580997
-0.15019	0.250857	0.413191	0.929909	0.728594	0.746307	0.18359	0.007977	0.746319
0.827503								
0.827503	1.584372	0.540354	0.763184	0.253947	0.30684	0.139919	0.084908	1.444452
0.827503	0.380789	0.912295	0.61816	0.445321	0.416909	0.343415	0.277646	0.56888
-0.53684								
-0.53684	-0.25399	-0.59997	-0.57091	-0.40679	-0.70679	-0.42761	0.027225	0.452803
-0.53684	1.250644	1.688997	1.491223	1.180407	0.571383	0.342792	0.006289	1.346205
-1.25027	0.069724	0.415905	0.802599	0.15435	0.633953	0.662299	0.039698	0.732875
-1.25027	-0.45836	-0.8478	0.049374	0.396641	-0.20264	-0.39513	0.000956	1.244438
0.157938								
0.157938	1.114979	1.436969	1.819163	1.534853	1.895665	1.746097	0.014561	0.780686
0.157938	0.129198	0.436734	0.539458	0.954701	0.696964	0.460301	0.001147	0.825502
-0.47633								
-0.47633	-0.33063	-0.76685	-0.93208	-0.37353	-0.70264	-0.65905	0.01325	0.601448
-0.47633	0.082023	0.064271	0.082017	0.387961	0.761552	0.131637	0.041304	0.69728
-0.17839								
-0.17839	-0.44654	-0.30622	0.390822	0.140761	-0.10699	-0.21392	0.018825	0.837363
-0.17839	0.823274	1.096968	1.724947	1.320137	1.207975	0.927847	0.033722	0.901673
0.475347	0.599175	0.847069	1.405753	0.218458	1.005336	0.841197	0.010286	1.187296
0.475347	-0.66466	-1.24028	-1.59417	-0.77638	-1.12228	-0.90966	0.0006	0.929509
0.475347	-0.62704	-0.91447	-0.92271	-0.56677	-0.85068	-0.69512	0.005923	0.355938
0.321038	0.347531	0.575919	0.600407	0.191311	0.458699	0.469482	0.030189	0.409096
-0.3639	0.415049	0.44287	0.815879	0.402828	0.614314	0.712125	0.085752	0.413052
-0.53909								
-0.53909	0.04254	0.117276	-0.36317	0.107795	-0.83323	-0.49534	0.024361	0.950504
-0.53909	-0.59157	-0.37048	0.290935	0.016012	-0.413	-0.49135	0.006305	0.882505
-0.3639	0.198216	0.261338	0.069964	0.105582	0.55453	0.412642	0.088716	0.484566
-0.3639	0.188462	0.750997	1.164654	0.44485	-0.13927	-0.06837	0.001513	1.303922
-0.57428	1.540933	2.02352	1.591043	1.218516	1.472265	1.399117	0.01302	0.805004
-0.57428	0.206097	-0.21436	-0.65497	0.402972	-0.17127	-0.31491	0.188442	1.057941
-0.57428	-0.00126	0.251402	0.788642	0.654514	0.194378	0.134522	0.007443	0.789904
-0.19346	0.2964	0.609317	0.368762	0.062281	0.267376	0.447864	0.115704	0.547036
-0.19346	0.102373	0.23711	0.234415	-0.13429	-0.30404	-3.51E-05	0.080301	0.541147
-0.82841	1.553645	1.114766	0.982746	0.97266	1.246544	0.942071	0.123679	0.611574
-0.82841	-0.43445	-0.42269	-0.64982	-0.51666	-0.59601	-0.34555	0.106085	0.304272
-0.82841	0.551681	1.200693	1.30216	0.77694	1.25852	0.784171	0.003329	0.750479
-0.07501								
-0.07501	-0.24293	-0.32213	-0.22723	-0.36009	-0.16525	-0.21744	0.083635	0.194839

-0.07501	-0.95171	-1.06665	-1.06991	-0.82965	-0.69987	-0.80697	0.005087	0.370041
-0.07501	-0.16217	-0.55045	-0.64562	-0.51845	-0.02324	-0.38715	0.037729	0.622387
1.63651								
1.63651	-0.87379	-1.26671	-1.55849	-0.88162	-1.18804	-0.9095	0.000603	0.684693
1.63651	-0.12559	-0.06809	0.016184	0.201048	0.529236	0.301917	0.002714	0.654827
-0.51491	0.459389	0.684906	1.176046	0.646096	0.570807	0.375444	0.048224	0.800601
-0.51491	1.031994	1.31483	1.6498	1.433087	1.290485	1.059564	0.004662	0.617806
	0.7039	0.511725	0.36887	0.309031	-0.18538	0.378394	0.000363	0.889279
	0.912836	0.991939	1.01547	0.772761	0.872782	0.9243	0.155655	0.242709
-0.15927								
-0.15927	-0.56782	-0.69462	-1.00727	-0.6213	-0.72078	-0.73781	0.007397	0.439448
-0.15927	0.859553	1.035383	1.161786	1.527643	2.02877	1.467843	0.001907	1.169217
-0.04834	1.165443	1.667457	1.730297	0.977691	1.38217	1.168937	0.005999	0.752606
-0.04834	0.328477	0.59321	0.336327	0.574267	0.313237	0.601073	0.296682	0.287836
-0.04834	0.542915	0.420349	0.435442	0.371783	0.129583	0.2631	0.087384	0.413333
-1.41368	5.560267	5.128267	4.160293	4.562813	4.708635	4.103027	0.040531	1.45724
-1.389								
-1.41368	1.655957	1.997497	2.041937	1.41527	1.06979	0.877786	0.002924	1.16415
-1.389	1.56762	2.0319	1.947307	1.404907	1.090205	0.833913	0.023844	1.197987
-1.41368	-0.30545	-0.15904	0.100636	-0.0224	-0.47263	-0.34795	0.006193	0.573271
-1.389	-0.3809	-0.12657	0.024944	-0.16647	-0.60747	-0.7552	0.010452	0.780148
	1.32766	1.274257	1.362792	0.810297	1.573125	1.180315	0.038571	0.762828
0.223543								
0.223543	0.553372	0.836781	0.793952	1.493673	0.46817	0.342392	0.120522	1.151281
0.223543	0.063874	0.100585	0.274112	0.562481	0.072984	0.315005	0.049613	0.498607
0.536963	0.541714	0.718014	0.86188	0.478932	0.735098	0.753294	0.055074	0.382948
-0.342	0.060549	0.225653	0.31135	0.391206	0.435169	0.33758	0.049872	0.374619
0.536963	-0.00141	0.002757	0.061109	0.298295	0.313457	0.189056	0.037348	0.314869
0.536963	-0.78903	-1.03364	-1.05074	-0.49081	-0.80506	-0.73574	0.004281	0.559926
-0.87411								
-0.87411	-0.53962	-0.91516	-1.15355	-0.71406	-0.62887	-0.94494	0.032987	0.613931
-0.87411	0.830049	0.475839	-0.84299	-0.01216	0.171391	-0.3111	0.005157	1.673039
-0.53602	0.295353	0.591947	1.059669	0.406349	0.35987	0.527355	0.002128	0.764316
-0.53602	0.286358	0.261214	-0.03314	0.203926	0.900196	0.682947	0.028726	0.933335
-0.53602	-0.24985	-0.47448	-0.21466	-0.00245	-0.37464	-0.36155	0.003558	0.472034
0.52303	0.666238	0.688831	0.962185	0.437667	0.73252	0.804319	0.072027	0.524518
1.404203	0.271876	0.572444	1.009285	0.527369	0.83322	0.551391	0.037709	0.737409
0.52303	-0.47602	-0.63166	-0.20545	-0.11456	-0.60255	-0.76802	0.003127	0.653457
-1.38646	0.241005	0.175629	-0.09648	-0.39293	-0.35973	-0.09173	0.008397	0.633939
-1.38646	0.876977	0.601362	1.043463	0.637499	0.42308	0.572607	0.203104	0.620383
1.27812	4.248367	4.08789	3.187795	3.259863	3.69404	3.011937	0.073174	1.23643
1.27812	-0.56322	-1.10771	-1.20382	-0.72132	-0.39239	-0.46751	0.022462	0.811424
1.27812	-0.4679	-0.82663	-1.37883	-1.06548	-1.28	-0.86257	0.025201	0.910929
-0.66958								
-0.22693								
-0.66958	-0.47543	-0.9164	-1.14499	-0.62922	-1.17559	-0.74932	0.014624	0.700152
-0.66958	0.156743	0.370738	0.476558	0.417917	0.039208	0.209207	0.025884	0.43735

-1.35067								
-1.35067	1.40613	1.653423	1.696143	1.202543	1.103455	0.981546	0.001822	0.714598
0.144002								
0.144002	0.692857	0.723588	0.745223	0.716919	0.824451	0.750348	0.149322	0.131593
-0.70468	0.185611	0.67486	1.035376	0.697764	0.449264	0.230932	0.007256	0.849765
-0.99423	0.337879	0.650156	1.00337	0.594169	0.622999	0.403795	0.002082	0.665491
-0.99838	0.65427	1.158916	1.340693	0.804261	0.711626	0.592154	0.001574	0.748539
0.25767								
0.25767	-1.10568	-1.40512	-1.58989	-1.15243	-1.44319	-1.13693	0.002301	0.484217
0.25767	-0.95252	-1.22399	-1.83412	-1.22988	-1.64379	-1.25663	0.01811	0.881599
-0.42277	0.643924	0.883907	0.903464	0.298879	0.661921	0.699505	0.009184	0.604584
-0.42277	0.163968	-0.13122	-0.01184	0.312603	0.213185	0.125798	8.61E-05	0.443824
-0.42277	0.70535	1.403847	1.465353	0.876865	0.683741	0.579322	0.019532	0.886031
-0.30488	0.220949	0.607018	0.927312	0.277773	-0.11954	0.318012	0.029721	1.046856
-0.30488	0.166726	0.250875	0.156604	0.352286	-0.04101	0.107967	0.122566	0.393292
-0.30488	-0.46211	-0.11662	0.489387	0.397915	-0.21618	-0.59774	0.009467	1.087127
0.193747	-0.70588	-1.11787	-1.30309	-0.74969	-1.14608	-0.90751	0.002283	0.597209
0.193747	-0.652	-0.73997	-0.71027	-0.59509	-0.96742	-0.67836	0.021384	0.372326
2.11042	-0.45373	-0.20828	-0.21654	-0.13058	-0.46124	-0.48872	0.198355	0.358146
0.842								
0.08862	2.195333	2.223123	1.766228	1.545573	1.69976	1.504283	0.007781	0.71884
0.842	0.783961	0.928658	1.017977	0.496447	1.16263	0.942917	0.016846	0.666183
0.4601								
0.4601	0.626426	0.86823	1.285743	1.264447	1.198425	0.976901	0.007286	0.659317
0.93691	0.656683	0.786935	1.071785	0.571277	0.936917	0.931168	0.02676	0.500508
0.93691	0.583752	0.431595	0.367219	0.741298	0.796078	0.580341	0.020964	0.428859
0.853083								
0.574027	-0.3346	-0.93277	-1.08465	-0.70313	-1.0521	-0.78529	0.022554	0.750058
-1.2005	-0.20366	-0.32331	-0.52963	-0.25957	-0.59404	-0.35794	0.086506	0.390382
-1.2005	0.462542	0.620587	0.924293	0.81787	0.554419	0.465306	0.001017	0.461751
-1.2005								
0.853083	0.883398	1.355367	1.68811	1.143582	1.066935	0.875406	0.005627	0.812704
-0.05071	-0.4857	-0.50023	-0.73189	-0.86681	-0.99035	-0.89103	0.013818	0.50465
-0.05071	0.439488	0.685113	1.028492	0.778607	1.62576	1.141892	0.002806	1.186272
-1.38427	-1.08444	-0.80228	-0.64286	-0.77493	-0.79753	-0.70299	0.068562	0.441581
-1.38427	0.845205	1.541523	1.783347	1.287403	1.208225	0.934568	0.002274	0.938142
0.617183	-0.42755	-0.48362	-0.03792	-0.19382	-0.22051	-0.27952	0.014156	0.445701
-0.46143	-0.14412	-0.20237	0.195834	-0.1974	-0.37284	-0.34655	0.020443	0.568671
-0.46143	-0.51837	-0.69486	-1.2694	-0.99935	-0.81094	-0.72124	0.003259	0.751035
-0.46143	0.1731	0.487424	0.755478	0.363766	0.379843	0.192138	0.034411	0.582377
0.617183	-1.00272	-1.53236	-0.98478	-0.58529	-0.56325	-0.90257	0.009429	0.96911
0.617183	0.915655	0.563101	0.419885	0.469422	0.334504	0.331419	0.03593	0.584236
0.079683	1.626437	1.833287	1.717557	1.575877	2.10433	1.52891	0.168051	0.57542
-0.30176	0.891352	0.836163	1.16268	1.189497	1.461085	1.484947	0.048602	0.648784
1.276127								
1.276127	-1.00069	-1.56193	-1.55749	-1.17432	-1.48667	-1.17736	0.008145	0.561239
1.276127	0.796047	0.89059	0.873019	1.132763	1.41985	1.246407	0.015885	0.623803

0.16685	0.592885	1.072853	1.39514	0.602252	0.736729	0.670954	0.003831	0.802255
0.16685	-1.19992	-1.52229	-1.85172	-1.19394	-1.81432	-1.41845	0.010547	0.657777
0.16685	-1.31481	-1.83852	-1.89228	-1.25043	-1.603	-1.36502	0.004166	0.64185
0.16685	-0.05609	-0.23375	-0.06929	-0.00618	-0.0649	-0.24803	0.024176	0.241855
-0.77764	0.346678	0.477542	1.165501	0.708673	0.730606	0.748722	0.002731	0.818823
-0.77764	-0.09635	-0.11304	0.048978	0.299585	0.648788	0.284399	0.013572	0.761827
-0.77764	0.069266	0.238687	0.386686	-0.04136	-0.10081	-0.10217	0.003492	0.488854
-0.71231								
-0.71231	2.627967	2.658447	2.944427	1.957293	2.092115	1.87506	0.145876	1.069367
-1.23575	1.023211	1.789477	2.363163	1.430477	1.735265	1.324233	0.004597	1.339952
-0.97856	0.167871	0.829978	1.55973	1.357474	0.331337	0.195619	0.003281	1.391858
-0.40858								
-0.40858	-0.45368	-0.03014	0.37189	-0.19105	-0.23849	-0.31124	0.001618	0.825571
-0.40858	-0.51235	-0.3388	-0.20073	-0.25306	-0.66665	-0.57374	0.023738	0.465913
-1.04991	0.337466	0.817377	0.8729	0.214576	0.309226	0.265208	4.13E-05	0.658325
-1.04991	0.347669	0.571112	0.790324	0.707922	-0.00462	0.360664	0.066261	0.794941
-0.51788	-0.03001	0.131312	0.535939	0.014348	0.081034	0.167247	0.009551	0.56595
-1.04991	-0.10312	-0.09612	0.418578	0.074826	-0.15006	0.110213	0.070936	0.568634
1.606143	-0.74362	-0.61168	-0.82705	-1.12725	-0.82109	-0.49586	0.029179	0.631393
-0.51788	-0.53904	-0.62062	-0.83881	-0.72386	-0.6824	-0.40375	0.001244	0.43506
-0.51788	0.421578	0.761378	0.978283	0.479812	0.69978	0.573628	0.004337	0.556705
-0.64485								
-0.64485								
-0.20237								
-0.20237	1.009427	1.366047	1.232487	0.405726	0.887236	1.093326	0.006306	0.960321
-0.31826								
-0.31826	2.49354	1.717833	0.742443	1.929374	1.351769	1.516043	0.099133	1.751097
-0.31826	0.277893	0.576042	0.196694	0.361641	-0.09945	0.208463	0.087358	0.675496
-0.10304	0.498714	0.4578	0.642833	0.207556	0.642526	0.601791	0.026539	0.435277
-0.10304								
0.349333								
0.537783								
0.537783	-0.39482	-0.55344	-0.67374	-0.21996	-0.35143	-0.21246	0.009147	0.461279
0.537783	0.5914	0.781618	0.926704	0.603637	0.83702	0.749908	0.159248	0.335304
0.349333	0.172922	0.671155	1.20106	0.614547	0.716644	0.370948	0.000348	1.028138
0.349333	1.71462	2.454783	2.1974	1.285963	1.56791	1.696793	0.048515	1.16882
-0.08983								
-0.34721	1.142516	1.685902	1.362316	2.676173	0.483664	1.296886	0.100224	2.192509
-0.34721	-0.87091	-1.28855	-1.27939	-1.06988	-1.1531	-1.04511	0.007004	0.417639
0.456283	5.967627	5.82857	5.00553	4.985893	5.126315	4.680433	0.011597	1.287193
0.456283	0.897997	1.304697	0.909137	1.419937	0.723636	1.123625	0.176898	0.696302
0.456283	0.807509	0.772211	0.670486	0.760915	1.591945	1.268083	0.000284	0.921459
-0.95641	0.846944	0.903969	1.121811	1.066626	1.356082	0.905608	0.056106	0.509138
-0.76877	-0.68791	-1.02724	-1.40922	-0.8262	-1.20512	-0.89026	0.01521	0.72131
-0.76877	0.778171	-0.84377	-1.3745	-0.72956	0.541015	-0.7927	0.093165	2.152668
-0.76877	-0.48118	-0.61993	-1.16676	-0.71742	-0.70412	-0.59778	6.68E-05	0.685577
0.041497	-1.20784	-1.43602	-1.2819	-1.16136	-1.60631	-1.21813	0.116315	0.444944

0.041497	1.210637	0.666331	0.517048	0.918862	1.383205	1.264977	0.013037	0.866157
0.041497	0.255557	-0.11704	-0.6802	-0.23041	-0.29456	-0.23314	0.002269	0.935758
-0.0835	0.897242	0.544108	0.14419	0.968393	0.642695	0.185394	0.208116	0.824203
-0.57925								
-0.0835								
-0.57925	-0.93872	-1.20762	-1.09997	-0.61471	-1.37304	-0.99993	0.082047	0.758332
-0.57925	-0.81537	-0.96544	-0.97672	-0.89452	-0.8755	-0.7707	0.123259	0.206022
-1.10566								
-1.10566	1.083744	2.008263	2.42876	1.789473	1.714985	0.993232	0.0088	1.435528
0.04631	0.386477	0.706245	0.850512	0.362321	0.963263	0.749351	0.010372	0.600942
0.04631	-0.48841	-0.56182	-0.66111	-0.23447	-0.40568	-0.52174	0.147655	0.426643
0.04631	1.19703	1.317033	1.386617	1.500497	1.781655	1.546093	0.010347	0.584625
-0.73964	-0.97174	-1.32159	-1.41911	-1.02864	-1.20971	-1.01443	0.003991	0.44737
-0.73964	-0.5945	-1.01168	-1.26983	-0.8274	-0.5088	-0.44959	0.015936	0.820234
-0.00271								
-0.00271	1.338226	0.223242	-0.3245	-0.52698	-0.13387	0.408719	0.038206	1.865201
-0.00271	-0.23135	0.003372	0.538598	0.320695	0.179572	-0.16381	0.033654	0.769949
-0.87961	7.897353	7.950213	6.84883	6.922407	6.6242	6.665287	0.002611	1.326013
-1.11792								
-1.11792								
1.369183								
1.369183	0.363849	-0.59999	-0.2193	-0.41027	-0.59618	-0.76226	0.085843	1.126106
1.369183	0.713351	1.164359	1.032559	0.587359	1.055568	0.980746	0.029729	0.577
-0.24248								
-0.24248	0.551179	0.797373	1.006752	0.685774	0.887243	0.857381	0.014648	0.455573
-0.47594								
-0.47594	0.534132	0.349906	0.110403	-0.06811	0.36181	0.352627	0.092228	0.602237
-0.47594	-0.09519	0.286692	0.83887	0.093086	0.238455	0.162747	0.006612	0.934064
1.066258								
1.066258	1.89942	2.10055	1.967963	1.31964	1.47494	1.42027	0.01309	0.78091
-0.86732								
-0.86732								
-0.86732	-0.21489	-0.1581	0.067799	0.123602	-0.30622	-0.13644	0.077795	0.429824
1.382787								
1.382787	-0.37208	-0.54146	-0.46398	-0.15013	-0.21738	-0.34598	0.042892	0.391329
1.382787	-0.64088	-0.84306	-0.74474	-0.71449	-1.00955	-0.5944	0.037556	0.415152
-0.46021								
-0.46021	-0.02857	-0.02419	0.032793	-0.32905	-0.28305	-0.1141	0.158243	0.36184
1.44464								
1.44464	0.480451	-0.64206	-0.91615	-0.43151	-0.48017	-0.48587	0.101879	1.396601
1.44464	-0.10116	-0.0357	0.199574	0.14175	0.379277	0.196022	0.002012	0.480437
1.112903	1.019965	1.42166	1.514377	0.533857	1.308725	1.313913	0.002351	0.98052
1.112903	0.418903	0.681596	0.389884	-0.10379	0.168849	0.19771	0.003749	0.785381
-1.2947	0.020097	0.526754	1.105643	0.703612	0.427963	0.067386	0.003073	1.085545
1.112903								
-1.33376	0.085516	0.072628	0.125727	-0.28244	0.356815	-0.1049	0.003322	0.63925
-1.33376	0.05621	0.493962	0.95943	0.491654	0.550424	-0.00854	0.017501	0.967971

-1.33376	0.164132	0.953018	1.64162	1.164177	0.312349	-0.02716	0.003968	1.668779
-1.2947	0.440785	0.486778	0.474577	-0.05164	0.846757	0.828827	0.026397	0.898401
-1.2947	-0.75329	-0.88808	-0.68444	-0.39544	-0.73215	-0.43043	0.031897	0.492643
1.412463	0.334217	0.464656	0.788857	0.506576	0.987474	0.749051	0.064191	0.653257
1.412463	1.415413	1.396063	1.39317	0.97172	1.121955	1.263593	0.029758	0.443693
-0.06044								
-0.06044	-1.07945	-1.29781	-1.08975	-0.85151	-0.99731	-1.08243	0.03201	0.446297
-0.06044	-0.27092	0.070359	0.063435	0.172929	-0.03662	-0.05579	0.001508	0.443846
-0.4416	1.435207	1.455166	1.539734	1.228386	1.541889	1.186578	0.029305	0.355311
-0.4416	0.777442	0.824941	0.991237	0.861977	1.24351	1.118238	0.027876	0.466068
-0.4416	1.073367	1.403487	1.779287	1.367483	1.477665	1.173513	0.003767	0.70592
-1.34809	1.349039	0.954582	0.656915	0.649728	0.785958	0.647204	0.112529	0.701836
-1.34809	0.652963	1.050374	1.126783	0.613743	0.9334	0.813647	0.023815	0.513041
-1.02876	0.522393	0.620026	0.333263	0.256701	0.093329	0.569344	0.30506	0.526698
-1.02876	1.955083	1.857123	1.95169	1.376803	1.22282	0.968011	0.057786	0.987072
-1.02876	-0.3663	-0.44258	-0.42121	-0.32814	-0.30048	-0.26013	0.255858	0.182456
0.785858	1.110877	1.429728	1.698197	0.879429	1.966415	1.757927	0.046082	1.086986
0.785858	-0.66482	-1.24143	-1.06919	-0.39906	-0.94472	-0.52817	0.095072	0.842366
0.785858	-0.9702	-1.424	-1.5503	-0.97275	-1.18152	-1.0999	0.053969	0.580101
-0.10196	-1.3194	-1.83614	-1.84509	-1.28782	-1.82581	-1.49796	0.036532	0.557264
0.785858	-1.00977	-1.44856	-1.72573	-1.21991	-1.36504	-1.23529	0.02043	0.715957
0.785858	0.030452	0.069844	0.391277	0.513624	0.17963	-0.05263	0.003187	0.566256
-0.10196	0.687495	-0.0575	-0.26909	-0.08653	0.236797	-0.19799	0.194723	0.956587
-0.10196	-0.2428	-0.47558	-0.31382	0.005688	0.253474	-0.10156	0.01513	0.72905
0.206433								
0.537105	0.336457	0.476377	0.451846	0.708616	0.890524	0.604058	0.001033	0.554066
0.537105	-0.05476	0.235719	0.107917	-0.14656	0.283005	0.106057	0.004049	0.429561
0.537105	-0.86141	-1.30004	-1.62938	-0.94107	-1.27416	-1.06398	0.00071	0.76797
0.537105	-0.60772	-0.93096	-1.48029	-0.48799	-1.16241	-1.04092	0.057117	0.992299
0.537105	-0.3922	-0.33114	-0.48983	-0.25772	-0.4092	-0.36621	0.055987	0.23211
0.537105	-0.29108	-0.78273	-0.04578	-0.29508	-0.03204	-0.19027	0.022022	0.750688
0.537105	0.064843	-0.31154	-0.07133	-0.05525	-0.03896	0.064025	0.019513	0.37638
0.206433	0.027164	0.217981	0.111729	0.163887	1.09137	0.534899	0.004366	1.064206
0.206433	0.332261	0.217266	0.25073	0.459078	0.576394	0.320937	0.016518	0.359128
0.84468	2.0378	2.397213	1.414683	1.722513	1.790915	1.756473	0.10105	0.982531
	-0.11349	-0.419	-0.44879	-0.37984	0.286638	0.015608	0.038853	0.735427
0.119062	-0.14132	-0.09389	-0.03916	-0.30262	0.033757	0.211227	0.012126	0.513847
0.119062	-0.50477	-0.52223	-0.83639	0.124677	-0.26287	-0.39217	0.012248	0.96107
0.119062	-0.28196	-0.19746	-0.18469	-0.2565	-0.32064	-0.27941	0.201224	0.135954
0.119062	-0.66096	-0.82208	-0.85147	-0.76954	-0.67972	-0.53287	0.019224	0.318605
-0.12781	0.887789	1.50454	1.716007	1.449	1.466235	1.058473	0.022256	0.828218
-0.90763	0.286701	0.477452	1.141303	0.277847	0.832169	0.715913	0.003229	0.863455
-0.90763	0.243871	0.458431	0.555888	0.267091	0.11393	0.354738	0.126543	0.441958
-0.12781	-0.35191	0.162179	0.21058	-0.16964	0.364236	0.106003	0.013738	0.71615
-0.94991								
-0.94991	0.261779	0.71352	0.866488	0.903569	0.447727	0.186752	0.048617	0.716816

0.271733	0.461682	0.809913	0.995714	0.353451	0.546426	0.756621	0.080228	0.642264
0.271733	-0.00553	0.129886	0.193307	-0.13068	0.325903	0.28116	0.064017	0.456579
-0.16975								
-0.16975	0.177119	0.177578	0.128585	0.19082	0.194914	0.256569	0.12442	0.127984
-0.41561	1.35566	1.8031	1.960497	1.074194	1.36919	1.130396	0.016198	0.886303
-0.37108	0.374683	0.849794	1.116451	0.767314	1.234105	0.9935	0.008851	0.859422
-0.37108	0.839592	1.209893	1.112967	0.58016	0.571947	0.840012	0.075265	0.637946
-0.41561	0.333763	0.479928	0.813081	0.803281	1.11024	0.659217	0.002587	0.776477
-0.41561	0.918609	1.192553	1.585417	1.338227	0.973268	0.837267	0.004623	0.74815
2.139823	1.380562	1.727223	1.693243	1.088019	1.546235	1.454595	0.110826	0.639204
2.139823	0.035811	-0.51565	0.003661	0.056893	-0.22506	-0.09952	0.047292	0.572538
0.948497	0.611202	1.062206	1.541007	0.435938	0.607186	0.77448	0.001616	1.105069
0.948497	0.350477	1.076684	1.207253	0.223811	-0.1754	-0.06421	0.002961	1.382653
0.948497	-0.56365	-0.56756	-0.5567	-0.44201	-1.00643	-0.85519	0.003741	0.564422
0.588443								
0.588443	1.652227	1.735367	1.440137	1.07586	1.27588	1.32737	0.126535	0.659506
-0.25051	0.488269	0.562056	0.801947	0.539101	1.033545	0.813235	0.03499	0.545276
-0.25051	0.553596	0.417918	1.047586	0.345406	0.747888	0.207423	0.236822	0.840163
-0.25051	0.312118	0.277556	0.520291	0.269717	0.228494	0.378954	0.10301	0.291797
0.161147	-0.99365	-1.43301	-1.5818	-0.79331	-1.27486	-0.99492	0.008171	0.788487
0.161147	-0.57139	-0.63599	-0.34951	-0.22171	-0.38689	-0.42437	0.000885	0.414272
-0.03228	1.001022	1.409527	1.294897	0.867105	1.49789	1.248243	0.079128	0.630785
-0.30248	0.671829	0.854152	0.914053	0.575052	1.03916	0.759418	0.000822	0.464108
0.161147								
-0.30248	1.067683	1.60506	1.472443	0.893926	1.640615	1.39856	0.031901	0.746689
-0.03228	-0.86721	-1.02135	-1.23492	-0.95296	-0.93466	-1.03783	0.061001	0.367712
-0.03228	-0.81918	-1.05071	-1.26891	-0.33714	-0.8251	-0.76561	0.082184	0.931779
-1.0138	0.77694	1.381793	1.667443	1.212107	1.40315	1.063114	0.00853	0.890504
-0.65224	0.188919	0.722182	1.29062	0.89648	0.528651	0.364905	0.000786	1.101701
-0.65224	-0.66952	-1.22485	-1.85363	-1.18205	-1.56029	-1.38843	0.009858	1.184112
-0.65224	0.470373	1.027264	-0.43402	-0.41337	-1.22641	-0.79513	0.025694	2.253674
-0.65224								
-1.0138	-0.07002	0.060536	0.183446	-0.04189	0.164901	0.000894	0.039968	0.25347
-1.0138	-0.08387	0.444334	1.000534	0.779205	0.431606	0.082875	0.003469	1.084404
0.06963	0.588575	0.803451	0.84303	0.262053	0.758481	0.88567	0.016253	0.623618
0.06963	0.44915	0.64049	0.893019	0.99216	1.05787	0.858372	0.006087	0.60872
1.106257								
1.106257	-0.71652	-0.85391	-0.93326	-0.67464	-0.70342	-0.58579	0.078758	0.347473
1.106257	0.000467	-0.00314	0.194449	0.168428	0.190166	0.185878	0.178029	0.197594
-0.37209	-0.37611	-0.67393	-0.84706	-0.56934	-0.90647	-0.66787	0.008792	0.530352
-0.37209	-0.80558	-0.74357	-0.49219	-0.66569	-0.78269	-0.86482	0.057248	0.372625
-0.37209								
-0.44898	-0.57631	-0.87096	-0.99033	-0.60658	-0.55833	-0.71037	0.046453	0.431998
-0.44898	0.249133	0.038304	-0.64695	-0.1505	0.104232	-0.13568	0.007732	0.896085
-1.11763	-0.19932	0.009014	0.684206	0.241728	0.203051	0.062527	0.020977	0.883529
-1.11763	0.511769	1.218013	1.561313	0.842667	0.748476	0.630103	0.002625	1.049545
-0.39358								

-0.39358	0.141822	0.497685	0.590754	0.281571	0.692556	0.660526	0.097799	0.550734
-0.39358	-1.19209	-1.37414	-1.28945	-1.0361	-1.13385	-1.13458	0.051593	0.338035
-0.39358	-1.42694	-2.12814	-2.15938	-1.40835	-1.77362	-1.3767	0.027643	0.782679
-0.44009								
-0.44009	0.199398	0.661912	1.046922	1.006268	0.622327	0.405728	0.014401	0.847524
-0.44009	-0.68239	-0.83234	-1.59157	-1.00483	-1.42861	-0.96467	0.00094	0.909185
-0.44009	-0.41493	-0.89557	-1.03707	-0.47069	-0.71022	-0.60645	0.003688	0.622144
-0.44009	-0.80878	-1.20941	-1.24027	-0.62809	-1.09109	-0.7354	0.003846	0.612187
-0.44009	-0.21055	-0.41372	-0.54539	-0.29028	-0.41862	-0.41842	0.134299	0.334839
0.451443	0.562691	0.71962	0.928585	0.618825	0.562785	0.665005	0.018022	0.365894
0.451443	-0.22947	-0.35533	-0.12464	-0.36171	-0.06791	-0.07418	0.033375	0.293805
0.451443	0.842552	1.213376	0.852916	0.432475	0.547724	0.775944	0.03698	0.7809
0.257503	0.961954	0.562854	0.487842	0.384079	0.520888	0.522174	0.083094	0.577875
0.257503	-0.02024	0.188516	0.666545	0.443608	0.229956	0.055917	0.00304	0.68678
0.257503								
-0.13279								
-0.13279	0.576987	0.451393	0.495786	0.605983	0.879648	0.649577	0.110445	0.428254
-0.13279	0.193013	0.492173	0.814715	0.940611	0.800706	0.438374	0.002203	0.747598
-0.13279	-0.39283	-0.95597	-0.98412	-0.44125	-0.48388	-0.59821	0.131218	0.591288
0.54282	0.229463	0.464314	0.586904	0.230453	0.465706	0.471151	0.015815	0.357441
0.54282	-0.34162	-0.25266	-0.37322	-0.47914	-0.33611	-0.10715	0.06781	0.37199
0.54282	-0.78804	-1.34031	-1.47834	-0.84833	-1.07233	-0.90457	0.002343	0.690297
0.246957	0.246864	0.273499	0.830866	0.591939	0.501975	0.651204	0.060626	0.584002
0.246957	0.677232	0.695139	0.640091	0.715839	0.967465	0.812697	0.0368	0.327373
1.61556								
1.61556	0.12226	-0.1143	-0.46356	-0.3919	-0.44504	-0.29063	0.069883	0.585817
0.41907	0.924632	1.030679	0.655951	1.224251	0.679354	1.445543	0.186139	0.789592
0.667627								
0.667627	0.258512	0.424798	0.936375	1.138323	0.777424	0.482602	0.001834	0.879811
0.41907	0.95777	0.933901	0.197464	0.531228	0.984824	0.528968	0.167209	0.787359
0.41907	-1.1042	-1.5202	-1.72217	-1.24786	-1.65491	-1.2997	0.014806	0.61797
-0.46763	0.1071	0.358936	0.645157	0.189069	0.542149	0.693548	0.011191	0.586448
-0.46763	0.343232	0.322907	0.24614	0.204686	-0.18884	0.074047	0.006626	0.532069
0.369897	0.672942	0.379302	0.463588	0.393856	0.534919	0.54186	0.173275	0.29364
0.369897	0.013966	0.102403	0.301046	-0.35911	0.540429	0.463606	0.012819	0.899542
0.218683	0.576258	0.81103	1.047402	0.509509	0.989948	0.942229	0.007898	0.537894
0.218683	-0.55744	-0.82965	-1.22846	-0.62806	-0.89474	-0.58943	0.016384	0.671022
0.218683	0.903974	0.555425	-0.02304	0.204778	0.203127	-0.01257	0.05549	0.92701
-0.27604	1.332837	1.61993	1.574323	0.973822	1.805715	1.533023	0.000204	0.831893
-0.79887								
-0.27604	3.130057	2.393177	2.053132	2.066888	2.415045	1.94049	0.001107	1.189567
-0.79887	0.431085	0.796608	0.872295	0.338275	0.421864	0.342088	0.039466	0.53402
-0.33313	-0.16067	-0.49281	-0.64767	-0.40761	-1.43329	-0.61371	0.000793	1.272622
-0.33313	0.26024	0.399512	0.489748	0.44247	0.966384	0.721778	0.011923	0.706143
-0.33313	-0.22792	-0.40396	-0.53211	-0.00477	0.078539	-0.079	0.023404	0.610646
-0.33313	-0.00981	-0.21951	-0.00709	0.242794	0.290741	0.210152	0.054125	0.510247
-0.33313	0.155409	-0.22123	-0.10527	0.018444	0.514914	0.349719	0.013853	0.736146

-0.36635	-0.264	-0.14085	-0.07627	-0.46444	-0.38065	-0.36002	0.023189	0.388174
-0.36635	0.084559	0.452688	0.43517	-0.22462	-0.46516	-0.57172	0.00076	1.024409
-0.36635	-0.61118	-0.88622	-1.19044	-0.86565	-1.16729	-0.85208	0.059356	0.579266
0.393023								
0.393023	0.216948	0.204113	0.401338	0.281176	-0.18061	-0.04205	0.032427	0.581949
0.393023	1.12378	1.613623	1.956367	0.982126	0.560084	0.584546	0.013333	1.396283
0.53131								
0.53131	-0.66802	-0.93334	-0.76101	-0.53894	-0.78457	-0.70103	0.093313	0.394399
0.53131	0.920947	1.134233	1.22697	0.879336	1.262631	0.46077	0.120141	0.801861
-0.44716								
-0.44716	-0.18084	-0.09687	0.286331	0.262735	0.095027	-0.06088	0.003473	0.467168
-0.44716	1.062001	1.354873	1.745437	1.017268	0.855005	0.840362	0.003905	0.905075
-1.11665								
-1.11665	-0.11504	0.501223	0.794807	0.43397	-0.25933	-0.15943	0.003295	1.054141
-1.11665	1.115307	1.570537	1.756697	1.15807	0.93295	0.658298	0.001396	1.098399
-0.64975	0.706536	0.451053	0.525551	0.204857	0.478383	0.213433	0.0111	0.501679
-0.64975	-0.39994	0.090707	0.604583	0.602362	-0.09751	-0.04145	0.003252	1.00452
-0.64975	-0.74353	-1.0273	-1.20476	-0.8326	-1.15172	-0.86651	0.143094	0.461229
-0.64975	-0.97082	-1.49703	-1.85705	-1.03726	-1.52919	-1.20006	8.42E-05	0.886229
0.579612								
0.579612	-0.10034	0.174111	0.424595	-0.03913	0.157771	0.00736	0.075239	0.524934
0.058003								
0.058003	-0.91605	-1.14859	-1.53159	-0.9533	-1.36651	-0.87674	0.008557	0.654851
0.058003	-1.15135	-1.51738	-1.58969	-1.01119	-1.43235	-1.14662	0.043507	0.578501
-0.5305								
-0.5305	1.363993	1.8306	2.054157	1.216175	1.71837	1.42456	0.064094	0.837981
-1.32885	0.544184	1.317377	1.458013	0.730645	0.622207	0.486801	0.006363	0.971213
-1.32885	0.127829	0.326765	0.782798	0.453273	0.106287	0.255608	0.022852	0.676512
-0.03919	0.330355	0.560492	0.871151	0.472344	0.705951	0.50182	0.023402	0.540796
-0.03919	-0.88458	-1.3939	-1.6566	-1.01677	-1.33373	-1.15339	0.000185	0.77202
-0.03919	-0.71951	-1.22071	-1.42841	-0.77705	-1.2027	-0.96431	0.000943	0.708892
-0.03919	-0.61203	-1.16227	-1.5727	-0.79728	-1.18371	-1.08255	0.002333	0.960668
-0.03919	-1.33202	-1.92823	-2.16538	-1.15781	-1.88778	-1.47143	0.02377	1.007564
-0.03919	-1.0647	-1.6584	-1.81717	-1.06744	-1.44924	-1.14841	0.000996	0.752466
-0.03919	0.292703	-0.26284	-0.66495	-0.28935	-0.33813	-0.43768	0.026398	0.957656
-0.03919	-0.66289	-1.15301	-1.42382	-0.96244	-1.28057	-1.05576	0.000944	0.760931
-0.03919	-0.79839	-1.42973	-1.73923	-0.82003	-1.25971	-0.99024	0.017366	0.940837
-0.03919	-0.91736	-1.22128	-1.42069	-1.02625	-1.22218	-0.83949	0.001864	0.581199
-0.03919	-1.18228	-1.6132	-2.09248	-1.25263	-1.61909	-1.34207	0.003244	0.910203
-0.03919	-0.41545	-0.5111	-0.47162	-0.44196	-0.70704	-0.40015	0.167311	0.306884
0.067562								
0.067562	0.535654	0.422309	0.432232	-0.07439	0.990744	0.816302	0.001042	1.06513
-1.30048	0.544291	0.840251	1.026365	0.36358	1.117715	0.847355	0.002521	0.754135
-1.30048	1.41267	2.185737	1.83956	1.18647	1.207599	0.867281	0.015844	1.318456
-1.09648	1.528731	1.84076	1.433948	1.315768	1.149315	1.280563	0.187691	0.691445
-1.09648	0.732629	1.441303	1.848763	1.172553	1.13804	0.882912	0.001515	1.116135
0.095797	0.882868	0.812808	0.493019	0.465133	1.47268	1.135935	0.054251	1.007547

-1.09648	1.286023	1.288427	0.869734	0.996365	2.009805	1.80247	0.009081	1.140071
0.095797	-0.24062	-0.71392	-0.53461	0.228142	0.163657	-0.02522	0.007176	0.942061
0.095797	-0.14875	-0.63832	-1.03028	-0.10558	0.024766	0.192112	0.03711	1.222391
0.972937	1.969227	1.787657	1.483527	1.507027	1.65263	1.718223	0.047253	0.4857
1.105073	0.363228	0.395692	0.724503	-0.00776	0.501073	0.534323	0.001974	0.732265
0.972937								
1.105073	0.167503	0.123554	0.140616	-0.10733	0.067816	0.164904	0.082027	0.274837
1.105073	0.700046	1.396423	1.524903	0.481585	1.183125	0.782311	0.000814	1.043319
1.95058								
2.134173	1.24566	1.426347	1.570687	0.862277	1.50052	1.509697	0.002403	0.708409
1.95058	1.144056	1.40503	1.442393	0.720034	1.33061	1.46919	0.002755	0.749156
2.134173	1.495727	2.25265	2.120563	1.772777	1.732925	1.890057	0.108918	0.756923
1.367397	2.48347	2.635707	2.350733	1.811723	1.796145	1.300533	0.014318	1.335174
1.367397	0.029483	-0.13106	-0.41894	0.158908	0.546953	0.370006	0.005732	0.965894
1.367397	0.529743	0.269095	0.064114	0.135042	0.822618	0.612987	0.003597	0.758504
0.00772								
0.00772	-0.32354	-0.38763	-0.17325	-0.20762	-0.57705	-0.2852	0.119234	0.403801
0.00772	-0.50932	-0.20815	0.050864	-0.40097	-0.33922	-0.4823	0.016863	0.560182
-0.40479	-0.29404	-0.09455	0.185987	0.154293	0.098761	0.026073	0.100917	0.48003
-0.40479	-0.43923	-0.44207	-0.43163	-0.3276	-0.12615	-0.68687	0.019618	0.560719
-0.40479	0.085199	0.364612	0.88042	0.838298	0.61028	0.526076	0.001256	0.795221
-0.63336								
-0.63336	0.471402	0.071882	0.101121	0.598098	0.083639	-0.03039	0.042602	0.628488
-0.63336	-0.1976	-0.45885	-0.10589	0.515434	0.163884	-0.02768	7.14E-05	0.974285
-0.44194								
-0.44194	-0.80333	-1.04039	-0.88466	-0.57305	-1.21567	-0.99591	0.011918	0.642622
-0.44194	0.313126	0.474885	0.647716	0.412096	0.571934	0.521899	0.006799	0.33459
-0.70654								
-0.70654	0.973485	1.412523	1.729427	1.255533	1.09601	0.975685	0.001568	0.755941
-0.03086	0.571802	0.906344	0.876381	0.656962	0.597384	0.778796	0.104841	0.334542
-0.03086	-0.97446	-1.09434	-0.67633	-0.50026	-0.89234	-1.08726	0.01483	0.594081
-0.82787								
-0.82787	-0.71091	-0.74043	-0.67274	-0.73109	-0.87441	-0.45699	0.130226	0.417421
-0.82787	0.566289	0.956874	1.198419	0.517149	-0.09	-0.10225	0.010963	1.300665
-0.46203	0.20802	0.406849	0.97202	0.401602	0.532706	0.368243	0.01105	0.764
-0.46203	2.332327	2.540907	2.062487	1.767557	2.15412	1.728723	0.022809	0.812183
-0.46203	-1.42538	-1.71123	-1.88369	-1.65872	-1.96148	-1.63087	0.020146	0.536092
-0.46203	-0.92006	-1.48617	-1.79127	-1.04015	-1.3878	-1.07745	0.036257	0.871215
-0.46203	-1.31959	-1.6984	-1.7773	-1.2432	-1.63744	-1.32322	0.019955	0.5341
-0.46203	-1.13081	-1.63647	-1.72097	-1.1695	-1.61398	-1.29289	0.014307	0.590163
-0.46203	-1.26997	-2.26875	-2.24613	-1.32701	-2.34789	-1.57686	0.002246	1.077917
-0.46203	-0.00116	0.250217	-0.37572	0.16647	-0.73045	0.116765	0.034482	0.980663
3.271353								
3.271353	-0.31461	-0.30772	-0.29852	-0.21961	-0.53295	-0.34008	0.0492	0.313344
3.271353	-0.24655	-0.36763	-0.4754	-0.41026	-0.53913	-0.62281	0.152793	0.376257
-0.29092	0.862117	1.028524	1.406201	0.897862	1.23309	1.242363	0.032161	0.544084
-0.29092	2.99639	3.71385	2.278793	2.376147	2.17929	2.583177	0.067498	1.53456

-0.72455								
-0.72455	-0.65617	-1.03924	-1.19652	-0.68001	-1.03341	-0.66502	0.010124	0.540355
-0.72455	-0.05533	0.233139	0.383238	0.067789	-0.50008	-0.34134	0.006759	0.883317
1.094577								
1.094577	2.581293	2.30639	1.898787	2.498083	2.649415	2.006853	0.205082	0.750628
1.608092								
1.608092	-0.06082	-0.38407	-0.29783	-0.5524	-0.34697	-0.26549	0.053701	0.491577
0.424837	0.241937	0.21227	0.497394	-0.10349	0.4624	0.412077	0.008298	0.600887
0.424837	0.069014	-0.15037	-0.29663	0.058156	0.277513	0.467081	0.004923	0.763713
0.424837	0.012615	0.541307	1.10479	0.398233	0.092923	-0.0743	0.001252	1.179086
3.113277	0.551046	0.669717	0.876338	0.089941	-0.16335	0.277828	0.033099	1.03969
3.113277	0.158811	0.196169	0.083241	0.00232	0.766581	0.486187	0.015605	0.76426
3.113277	-0.27139	-0.432	-0.74081	-0.55096	-0.47214	-0.32479	0.097082	0.469423
3.113277	0.742233	0.993433	1.138713	0.539604	0.546491	0.499796	0.033479	0.638917
0.93984	0.76179	0.59487	1.289135	0.538062	0.819084	0.630013	0.102098	0.751073
0.93984	-0.41436	-1.15766	-0.9663	-0.671	-0.69551	-0.73334	0.004807	0.743304
0.93984	-0.41455	-0.45997	-0.18276	-0.45437	-0.52469	-0.42883	0.12257	0.341935
0.12176								
0.12176	-0.39893	-0.44679	-0.33534	-0.11124	-0.53196	-0.0792	0.05368	0.45276
0.12176	-0.30679	-0.23772	0.113604	0.059439	-0.23362	-0.25965	0.016194	0.420398
-0.02382	-0.5995	-0.77308	-0.94059	-0.65758	-0.92641	-0.70807	0.027555	0.341085
-0.02382	-1.22228	-1.63652	-1.81064	-0.9705	-1.43207	-1.1139	0.002238	0.840139
-0.02382	-0.96706	-1.53021	-1.70946	-0.96535	-1.31249	-1.09259	0.00645	0.744107
-0.74203								
-0.02382								
-0.74203	-0.35916	-0.15737	0.175944	-0.07731	0.131507	-0.08762	0.005273	0.535109
-0.74203	-0.00503	-0.01159	0.18103	-0.08656	-0.51177	-0.37081	0.0855	0.692802
-0.65371	1.164933	1.605647	1.83624	1.068767	1.608825	1.377963	0.005178	0.767473
-0.1061								
-0.1061	0.580303	0.951105	1.246283	0.792812	1.196015	0.844485	0.010915	0.66598
-0.1061	0.783056	0.456426	0.394403	0.872245	0.607392	0.671865	0.030354	0.477842
-0.65371	-0.87475	-1.29937	-1.29668	-1.00337	-1.16432	-1.00755	0.014514	0.424625
-0.65371	-0.11082	0.026789	-0.04531	-0.30121	-0.24107	-0.20074	0.024182	0.327996
-0.76528	1.103565	1.922447	2.14574	1.107619	1.54423	1.148999	0.007796	1.042175
-0.76528	-0.41516	-0.28835	0.515518	-0.36291	-0.7226	-0.71385	0.010221	1.238118
-0.76528	0.07475	0.383622	0.70125	0.122583	-0.11126	0.061928	0.024223	0.812506
-0.26951								
-0.26951								
2.103643	0.458306	0.804617	0.777098	-0.04147	0.143769	0.556081	0.000312	0.84609
2.103643	-0.17381	-0.14914	-0.18867	0.103651	-0.22596	-0.07611	0.075014	0.32961
0.00289	0.41259	0.349829	0.318801	0.109789	-0.42384	0.128279	0.020029	0.836431
0.795733	-0.31485	-0.31285	0.46623	0.609996	0.008638	-0.2195	0.018584	0.924844
0.795733	-1.07338	-1.81404	-2.14866	-1.23018	-1.5327	-1.38172	0.000184	1.075282
0.795733	0.265618	0.378872	0.169391	0.514797	0.979535	0.419646	0.110912	0.810144
-0.35274	0.141603	0.741594	1.15135	0.325233	0.805151	0.588536	0.000194	1.009747
-0.35274	-0.77977	-1.08229	-1.20308	-0.69135	-0.76235	-0.80015	0.003546	0.511729
-0.35274	0.446411	-0.14967	0.338299	0.17137	0.166892	0.235185	0.161388	0.596083

-0.59921								
-0.59921	0.505892	0.987004	1.205576	0.591857	0.871373	0.630501	0.038489	0.699684
-0.37064								
-0.37064	-1.14938	-1.35762	-1.53661	-1.17962	-1.58949	-1.23124	0.082489	0.440113
-0.37064	-0.79193	-1.6269	-1.73147	-0.88895	-1.326	-0.93957	0.007829	0.939534
-0.37064	-1.25627	-1.93552	-2.27993	-1.42079	-1.87642	-1.52696	0.008611	1.023663
-0.37064	0.047496	-0.97389	-1.34127	-0.28622	-1.02012	-1.04295	0.064025	1.388766
-0.37064	0.177486	0.499391	0.9842	0.504874	0.657536	0.27007	0.002802	0.806714
-1.31399	1.484767	0.552815	0.684513	0.36975	0.391672	0.488983	0.001267	1.115016
-1.31399	-0.56653	-0.99911	-1.26175	-0.62052	-1.11672	-0.85353	0.020201	0.695217
-1.31399								
-0.73244								
-1.19162	-0.30192	-0.64492	-0.88605	-0.75834	-0.89705	-0.80584	0.035412	0.595127
-1.19162	0.437571	1.230637	1.217627	0.895849	0.484646	0.211346	0.041354	1.019291
-1.19162	-0.18349	-0.06487	0.280309	0.108861	0.038006	-0.19589	0.038314	0.476195
-0.73244	-0.96534	-1.75286	-1.77416	-1.09971	-1.3204	-1.1708	0.004002	0.808814
-0.73244	-0.39403	-0.74794	-0.50908	-0.44022	-0.38325	-0.51353	0.025808	0.36469
-0.57838								
-0.57838	-0.03339	-0.33767	-0.20193	0.296477	0.314938	-0.08892	0.003718	0.652611
-0.57838	0.665382	0.606739	0.776347	1.283072	0.808127	0.684606	0.095696	0.676332
-0.58527								
-0.58527	1.310081	1.030076	0.75053	0.647019	1.045309	1.107808	0.062437	0.663062
-0.66193								
-0.66193	-1.245	-1.2427	-1.05705	-0.61023	-0.80601	-0.72805	0.014552	0.634769
-0.66193	-0.49136	-0.28915	-1.0065	-0.74329	-0.75426	-0.60652	0.153706	0.717351
-0.12024	-0.11194	-0.13921	-0.76295	-0.55946	-0.74464	-0.71884	0.015941	0.651019
-0.12024	0.971708	1.532487	1.312954	0.857457	1.72462	1.47912	0.016699	0.867163
-0.12024	-0.05816	-0.04009	-0.16014	-0.01402	0.272612	0.052091	0.025106	0.432755
-0.19863								
-0.19863	0.888619	1.281946	1.351719	1.332244	1.78213	1.198585	0.050515	0.893511
0.304917								
0.304917	3.086523	3.190677	1.955415	3.134516	1.497913	1.98228	0.145576	1.692764
0.304917	0.494478	0.151685	0.457214	0.837814	0.781031	0.577843	0.017258	0.686129
	-0.01696	0.026907	0.060229	0.181615	0.312615	0.363538	0.105418	0.380501
0.188773	0.960369	0.549991	0.716888	1.106861	1.123255	0.854071	0.034989	0.573263
0.188773	4.111123	3.842957	3.186583	3.24226	3.579025	3.136983	0.05494	0.97414
	2.33605	2.162123	1.74174	1.559193	2.407305	1.66593	0.127952	0.848112
2.085193	-1.06021	-1.16821	-1.27861	-1.18158	-1.37188	-1.18435	0.128284	0.311674
2.085193	-0.68433	-1.14243	-1.11132	-1.12216	-0.74086	-0.70643	0.004416	0.458103
2.085193	-0.04101	-0.2139	0.111153	0.377586	0.866982	0.325273	0.00283	1.080885
-0.23513	0.344733	0.515972	0.497352	0.097246	0.282086	0.609793	0.068657	0.512547
-0.23513	-0.09442	-0.2552	-0.52139	-0.22344	-0.00147	0.124993	0.024801	0.646387
-0.23513	0.250413	0.236281	0.514404	0.558177	-0.3538	-0.04421	0.052306	0.911976
-0.54553								
-0.54553	-0.1866	-0.61605	-0.33319	-0.14178	-0.53828	-0.59688	0.020778	0.474276
-0.54553	-0.44485	-0.61435	-0.42442	-0.26556	-0.44202	-0.37468	0.135879	0.348789
0.397877	0.480276	0.662772	1.027289	0.692187	0.992234	1.042552	0.024211	0.562276

0.397877	1.192403	1.579823	1.350367	1.30358	1.69128	1.71091	0.110902	0.518507
0.465413	0.314056	0.488066	0.967253	-0.09935	0.540067	0.374295	0.041234	1.066603
0.465413								
0.741627								
0.741627	-0.98427	-0.89491	-0.38126	-0.54836	-0.68057	-0.65335	0.000705	0.60301
0.741627	0.182986	0.603139	1.37097	0.555305	0.630983	0.487069	0.000258	1.187984
-0.94917								
-0.94917	1.317733	1.60631	1.603537	1.179233	0.935379	0.928136	0.000588	0.678174
1.258328	1.423947	1.69546	2.107503	1.34835	2.11407	1.989987	0.006611	0.76572
1.258328	0.075698	0.10085	0.079105	-0.0278	-0.19356	-0.19095	0.015376	0.294411
0.869678								
0.869678	-0.25938	-0.53922	-0.46297	-0.18779	-0.38572	-0.26481	0.046154	0.351432
0.869678	-1.09676	-1.60131	-2.01004	-1.52668	-1.757	-1.44185	0.001773	0.913283
0.869678	-1.03448	-1.54519	-1.91296	-1.14124	-1.20669	-1.17115	0.003034	0.878487
0.869678	-1.16601	-1.64203	-1.94191	-1.16188	-1.59054	-1.14994	0.007093	0.791963
0.869678	-0.99268	-1.26163	-1.63201	-1.02914	-1.26535	-1.09814	0.020334	0.639334
0.11861								
0.11861	-0.20129	-0.5371	-0.31039	-0.00522	-0.17374	-0.1796	0.131212	0.531877
0.11861	0.480851	1.142896	1.338997	0.91038	0.860864	0.617987	0.00082	0.858146
0.017992	0.587763	0.480976	0.774752	0.502164	0.551382	0.427597	0.055183	0.347156
0.017992	0.007147	0.090886	0.156316	-0.09153	0.054361	-0.07533	0.030033	0.247845
0.017992	-0.14333	-0.5166	-0.99363	-0.47983	-0.56142	-0.33786	0.002748	0.850298
0.017992	0.835604	1.357396	1.369383	0.756191	0.998661	0.808387	0.025077	0.613192
-0.99102								
-0.99102	0.701538	0.912562	1.16822	0.649089	0.502262	0.328732	0.009593	0.839487
-0.46965	-0.78036	-0.12723	-0.51846	-0.67284	-0.95712	-0.85526	0.154758	0.82989
-0.46965	-0.37583	-0.4672	-0.20987	0.541555	-0.0109	-0.16357	0.001271	1.008757
1.351413								
0.297837								
-0.46965	3.23042	3.51205	2.685417	2.928423	3.014235	2.277753	0.03642	1.234297
0.297837	0.787086	1.081128	1.378763	1.334323	1.43187	1.08072	0.012243	0.644784
-0.43768	-0.00906	0.816919	0.948618	0.08873	0.594986	0.367466	0.007842	0.957675
1.351413	0.033109	0.42802	0.371057	-0.13299	0.113077	0.039464	0.024231	0.561011
1.351413	1.244606	1.565203	2.278777	1.31891	1.506705	1.362525	0.060424	1.03417
-0.43768	1.9991	2.216727	2.301703	1.395311	1.079495	1.260076	0.024159	1.222208
0.463707								
0.463707	0.268016	0.082615	0.704997	1.060801	0.290023	0.283999	0.00455	0.978187
-0.32946	0.663691	0.953799	1.142631	0.899883	0.973801	0.724729	0.032439	0.478941
-0.32946	1.192354	0.684403	0.348317	0.813826	0.460238	0.410496	0.048914	0.844038
-0.32946	-0.88669	-1.52915	-1.60004	-0.98462	-1.30082	-0.92077	0.018243	0.713344
-0.32946	1.345163	1.647593	1.9607	1.439373	1.577015	1.062965	0.000999	0.897735
0.161373	-0.30137	-0.40019	-0.40286	-0.23944	-0.30307	-0.18145	0.022548	0.221417
0.161373	0.27845	-0.19426	-0.54141	-0.16798	-0.21125	-0.23682	0.021312	0.819861
0.161373	0.876049	1.156372	1.283495	1.26572	1.88029	1.009455	0.00017	1.004241
-0.37606								
-0.37606	-0.11521	-0.47857	-0.76261	-0.92707	-0.75429	-0.61157	0.08303	0.811861
-0.37606	0.381395	0.456155	1.193156	0.700043	0.709038	0.594068	0.005974	0.811761

0.720788								
0.720788	1.341478	1.175118	1.230344	1.413957	-0.70626	0.235534	0.028676	2.120217
0.720788	-0.37804	-0.86519	-1.08834	-0.29549	-0.92939	-0.71601	0.035827	0.792842
-0.82253	0.73259	0.919911	1.247037	0.776675	0.943801	0.735546	0.005346	0.514447
-0.82253	-0.04337	0.216131	0.701616	0.649283	0.015273	0.213572	0.056	0.744988
-0.82253	0.588556	1.2915	1.602247	0.932797	0.932847	0.663068	0.000689	1.01369
	-0.09621	0.20218	0.466472	0.06981	-0.03696	-0.06485	0.004267	0.562679
-0.60143	0.782708	1.014597	1.564257	0.989872	1.32589	0.943667	0.004983	0.781549
-0.60143	0.581381	0.982494	1.199917	0.710408	0.71599	0.697934	0.013281	0.618536
	-0.69814	-0.53299	-0.19818	-0.08274	-0.52423	-0.48647	0.005997	0.615405
0.326333								
0.326333	-1.30242	-1.64213	-1.89134	-1.2332	-1.56357	-1.32575	0.009588	0.65814
0.326333	3.005187	2.547423	2.12954	1.81193	1.641975	1.844273	0.095688	1.363212
1.010827	0.672487	0.875273	1.165082	0.320083	0.430343	0.873131	0.013849	0.844998
1.010827	-0.91281	-1.17575	-1.14795	-0.68407	-1.22403	-0.98248	0.007293	0.539962
1.010827	-0.21149	-0.74296	-1.4142	-0.84354	-1.15479	-1.01876	0.036396	1.202718
1.010827	-0.65356	-1.0179	-0.99863	-0.73695	-0.87411	-0.76566	0.016236	0.364333
1.010827	-0.50854	-1.05017	-1.05553	-0.65437	-0.82365	-0.58223	0.021328	0.54699
0.352663	0.407147	0.746935	0.745729	0.459661	0.677985	0.685851	0.049335	0.339788
0.352663	-0.22626	-0.48433	-0.65169	-0.27638	-0.70594	-0.49065	0.007219	0.47968
0.352663	-0.62964	-0.40065	-0.2257	0.223965	0.032567	-0.45384	0.006409	0.853601
	0.573418	0.673149	0.509396	0.25133	1.11058	0.68514	0.017235	0.85925
0.445773								
	-0.75612	-0.89369	-1.08703	-0.71254	-0.88016	-0.70144	0.027202	0.385584
1.482087								
1.482087	-0.25561	-0.56556	-0.89214	-0.43939	-0.71791	-0.59342	0.001822	0.636529
1.482087	0.421258	0.134366	-0.48366	-0.34343	-0.63679	-0.22648	0.003015	1.058044
0.958143	-0.44768	-0.60796	-0.48157	-0.23738	-0.15718	-0.23474	0.109122	0.450789
0.958143	-1.30446	-1.62761	-1.82869	-1.34482	-1.21195	-1.14609	0.012554	0.682603
0.958143	-1.16237	-1.50772	-1.81951	-1.09586	-1.35063	-1.10164	0.002683	0.723643
0.958143	-0.78683	-1.02081	-0.864	-0.80438	-0.49971	-0.71112	0.03994	0.521101
0.958143	-0.59706	-1.08933	-1.26224	-0.56978	-0.26241	-0.31785	0.043117	0.99983
0.958143	-1.34805	-1.76499	-1.86274	-1.12839	-1.31053	-1.23416	0.016506	0.734353
-0.26745	-0.06109	-0.54892	-0.58678	-0.28367	-0.02042	-0.31018	0.167187	0.566361
-0.26745	-0.61281	-0.43636	-0.16178	0.145911	-0.62003	-0.21093	0.070444	0.765939
-0.87323	-0.03452	0.112846	0.304949	0.199979	0.153755	0.164016	0.053325	0.339473
-0.87323	0.180191	0.705145	1.086672	0.688603	1.105961	0.536574	0.024203	0.92577
0.131697	0.022343	0.121852	0.237831	0.22526	0.424621	0.287711	0.016609	0.402278
	-0.26495	-0.2626	-0.42036	-0.75393	-0.09889	-0.07827	0.022652	0.675667
	-0.16461	-0.20357	-0.12796	0.134888	0.236086	0.082092	0.002242	0.43966
	-0.33223	-0.89229	-0.99369	-0.43562	-0.72001	-0.57821	0.021942	0.661452
0.131697	0.536333	0.518268	0.841863	0.817627	1.18128	0.926486	0.029577	0.663012
0.061435								
0.061435	-0.76448	-0.59744	-0.18066	-0.36753	-0.5229	-0.27422	0.006348	0.583824
0.061435	-0.17252	-0.95359	-0.66595	-0.66331	-0.92746	-0.60959	0.001272	0.78107
0.061435	-0.94997	-1.61019	-2.16251	-1.26055	-1.66489	-1.34935	0.004807	1.21254
0.061435	-1.23088	-1.54031	-1.81411	-1.09477	-1.54595	-1.30917	0.019734	0.719333

0.061435	1.109821	0.604338	0.001353	0.299685	0.188307	0.335957	0.005344	1.108468
0.061435	-0.50523	-1.17403	-1.33102	-0.63914	-0.93265	-0.6083	0.007427	0.825798
0.061435	0.493607	1.043819	1.360383	0.587617	0.995271	0.669098	0.001555	0.866776
1.520623								
1.520623	-1.00034	-1.34471	-1.29629	-1.00304	-1.12101	-1.08136	0.029791	0.344377
1.520623	-0.14176	0.084526	0.059383	-0.08982	0.293018	0.006159	0.053422	0.434777
2.152927	-1.54477	-2.18895	-2.30447	-1.6475	-2.20996	-1.85304	0.024802	0.759696
1.451182								
2.152927	-0.93429	-1.50833	-1.795	-1.13417	-1.08803	-1.12168	0.005253	0.860717
2.152927	-0.82129	-1.32154	-1.38981	-0.82263	-1.18389	-0.96824	0.04844	0.568525
1.451182	-0.42775	-0.46798	-0.13422	-0.04935	-0.21751	-0.22962	0.014572	0.418624
1.451182	-0.92296	-1.33644	-1.47817	-1.03216	-1.26903	-1.06406	0.00054	0.555208
1.451182	-0.06279	-0.30705	-0.52676	-0.2463	-0.0577	-0.03148	0.004524	0.495272
1.451182	2.610827	1.996937	1.288242	1.544722	1.805505	2.156456	0.022147	1.322585
-0.76099								
-0.76099	-0.4792	-0.88328	-1.10552	-0.64602	-0.90161	-0.74533	0.005727	0.626319
-0.76099	0.329059	0.336998	0.772955	0.518593	0.279635	0.11872	0.002002	0.654235
0.200082	0.404095	0.435272	0.728747	0.437922	0.748463	0.599022	0.134815	0.344368
0.200082	-0.71022	-1.0122	-0.92563	-0.20791	-0.61749	-0.46446	0.005385	0.804296
0.200082	-1.03946	-1.11569	-1.5948	-1.08284	-1.33037	-0.83959	0.054463	0.755214
0.200082	-1.37369	-2.07748	-2.12425	-1.32751	-1.91374	-1.40661	0.002475	0.79674
0.200082	-0.41051	-0.47285	-1.0456	-0.09665	-0.93344	-0.42036	0.068633	0.948946
0.200082	-1.66034	-2.26888	-2.27393	-1.45941	-1.95369	-1.40006	0.017795	0.87387
0.200082	-0.69683	-1.55916	-1.31792	-0.64882	-0.84525	-0.77575	0.000873	0.910336
0.200082	-0.87887	-1.61956	-1.71238	-1.24768	-1.40911	-1.14948	0.000643	0.833509
-0.4939	0.113986	0.318246	0.593618	0.068057	0.418756	0.323884	0.018333	0.525561
-0.4939	0.666204	0.76161	0.414412	0.515034	0.546239	0.554302	0.037564	0.347198
0.576887								
0.576887	-0.22479	-0.53503	-0.53075	-0.37193	-0.65805	-0.42793	0.093062	0.433258
0.576887	0.898082	1.014113	1.266867	1.434293	1.62801	1.304703	0.000708	0.729928
-0.3382	0.321769	0.467753	0.480674	-0.03179	1.077756	0.701397	0.018863	1.10955
-0.3382	0.561798	0.851889	0.667589	0.684871	1.33662	1.004399	0.006289	0.774822
0.374613	0.684292	0.503818	0.625631	0.292024	0.560776	0.570788	0.243582	0.392268
0.374613	-1.35431	-1.52238	-1.56573	-1.27497	-1.3732	-1.02893	0.029694	0.536793
0.374613	-0.7906	-1.28515	-1.65869	-1.06019	-1.32444	-1.09209	0.000407	0.868091
0.374613	0.683207	-0.40068	-0.20687	-0.57541	-0.61705	0.07516	0.14853	1.300259
0.374613	-0.95901	-1.47272	-1.89126	-1.1108	-1.43041	-1.21341	0.001097	0.932246
0.374613	-0.89212	-1.58407	-1.61148	-0.88601	-1.10444	-0.9189	0.010017	0.725476
0.374613	-1.21848	-1.63661	-1.72125	-1.20881	-1.51405	-1.42613	0.03559	0.512443
-0.73095	0.456068	0.997303	1.30997	0.742304	1.159945	0.809037	0.008548	0.853902
-0.50033	0.243661	0.660654	0.944492	0.431717	0.657508	0.807138	0.009095	0.700832
-0.50033	1.143266	1.549737	2.01193	1.38201	1.800445	1.227083	0.002974	0.868664
-0.73095	-0.1866	0.168417	0.004858	0.376153	0.109316	0.01102	0.015232	0.562749
-0.41697								
-0.41697	0.143577	0.038774	0.107873	0.177412	0.145814	0.258667	0.101953	0.219893
-0.41697	-0.85787	-1.05408	-1.11249	-0.81194	-0.9666	-0.99905	0.043344	0.300556
0.772697	1.179284	1.532347	1.5721	1.32839	2.116705	1.46013	0.037228	0.937421

0.772697	-0.43455	-0.86431	-0.9868	-0.46621	-0.67785	-0.39685	0.081013	0.589952
0.772697	-1.33665	-1.72795	-1.81734	-1.39252	-1.35996	-1.20077	0.024612	0.616577
-0.47607								
-0.47607	-0.97476	-1.07394	-1.45032	-1.05721	-1.25945	-0.72028	0.072973	0.730037
-0.47607	-0.5673	-0.82245	-0.8865	-0.56357	-0.52357	-0.49183	0.144189	0.394678
-0.39708								
-0.39708	-1.26777	-1.36253	-1.17021	-1.00125	-1.45971	-1.10065	0.036116	0.45846
-0.39708	-0.84521	-0.44574	-0.14276	-0.6499	-0.67952	-0.81026	0.063771	0.702444
-1.23528								
-1.23528	0.828277	0.89726	1.048486	1.105141	1.015039	0.874107	0.025245	0.276864
-0.17878								
-0.17878	0.189624	0.325496	0.283677	0.450917	0.861983	0.508484	0.00603	0.672359
-0.17878	-1.19129	-1.71665	-1.51137	-1.06584	-1.30707	-1.00885	0.009726	0.707806
-0.17878								
-0.17878	-1.09178	-1.29375	-1.22893	-1.13289	-1.44436	-1.12081	0.033606	0.352572
0.11157								
0.60122	1.23279	1.265945	1.5323	1.302573	1.68156	1.50428	0.075216	0.44877
0.11157	-0.87769	-1.61405	-1.67717	-0.98806	-1.46495	-1.52452	0.028884	0.799472
0.11157	0.321288	0.281087	0.939824	0.209586	0.387063	0.143741	0.007822	0.796083
-0.54033								
-0.54033								
-0.54033	0.872837	1.443297	1.71177	0.901386	0.710879	0.836763	0.046248	1.000891
-0.35787								
-0.35787	0.304577	-0.21788	-0.50116	-0.17362	-0.32702	-0.47838	0.001409	0.805732
-0.35787	-0.09439	-0.11833	-0.07808	0.398373	-0.1765	-0.10543	0.036381	0.574873
-0.47502	-0.22954	-0.53863	-0.87539	-0.50024	-0.35317	-0.15593	0.013856	0.719458
-0.47502	0.157426	0.322277	0.437051	0.524092	0.636708	0.33297	0.023224	0.479282
-0.47502	0.840628	1.361863	1.379044	0.937556	1.158465	0.786045	0.045288	0.592999
-0.80318								
-0.80318	-0.74043	-0.97041	-1.09339	-0.70603	-0.97703	-0.81876	0.031707	0.387358
-0.80318	0.350239	0.634715	0.730929	0.895404	0.195188	0.357099	0.034108	0.700217
0.797443								
0.797443	-1.02408	-1.24029	-0.85833	-0.47971	-0.78915	-0.68181	0.0199	0.760581
0.797443	-0.85297	-1.43236	-1.67202	-0.96946	-1.41951	-1.03354	0.000504	0.819042
0.797443	-0.13466	-0.48513	-0.75897	-0.20051	-1.01793	-0.40951	0.060852	0.883271
0.23804								
0.99368	-0.81079	-0.87664	-1.0788	-0.7568	-1.15674	-0.82445	0.053792	0.399939
0.99368	0.948336	0.776716	0.326409	0.172798	1.347	1.415513	0.004759	1.242715
0.99368								
0.23804	0.791342	1.285613	1.61549	1.08336	1.020192	0.873636	0.011143	0.824148
-0.88517	1.80756	1.46278	1.233508	1.294697	1.589295	1.13072	0.032637	0.67684
-0.88517	-0.30623	-0.9599	-0.96832	-0.493	-0.41671	-0.85166	0.02285	0.662096
-0.88517	1.151934	1.337834	1.859313	1.319077	1.424955	1.133082	0.040363	0.726231
0.12364	2.849393	2.721513	2.011127	2.20402	2.36048	1.63028	0.022297	1.219113
0.12364	-0.68287	-1.19748	-1.53078	-0.84402	-1.14809	-1.04109	0.011766	0.847909
0.12364	0.26095	0.437022	0.401673	0.101824	0.555629	0.452503	0.077765	0.453805
-0.54978	0.83374	1.222232	1.155459	0.63843	0.891361	1.021761	0.018449	0.583802

-0.54978	0.727696	0.289227	0.189802	0.326082	0.061826	0.130745	0.131345	0.66587
-0.54978	0.529821	1.160117	1.54252	0.996482	1.23411	0.848624	0.003934	1.012699
0.154873								
0.154873	-0.35195	0.072906	0.479901	0.279209	-0.18168	-0.1657	0.052399	0.831852
0.154873	0.8065	1.31108	1.405717	0.601969	-0.01252	0.058519	0.05569	1.418234
-0.03167								
-0.03167	-0.55032	-1.01454	-1.3715	-0.98625	-1.0611	-0.84007	0.006723	0.821183
-0.03167	-0.80197	-1.08173	-1.16163	-1.01011	-1.05423	-0.8234	0.033844	0.359667
-1.36126	0.008891	0.128114	0.291656	-0.29386	0.413036	0.090237	0.01484	0.706892
-1.36126	0.983303	1.55501	1.39884	0.809725	0.898116	0.932782	0.003435	0.745285
0.879343	0.265484	0.356459	0.346685	-0.27337	0.871366	0.788392	0.009723	1.144732
0.879343	-0.59082	-1.13824	-0.52819	0.089641	-0.20018	-0.3702	0.004492	1.227877
0.879343	-0.68113	-0.87719	-1.07173	-0.71883	-0.62051	-0.6872	0.056256	0.451223
-0.7916	-0.09704	0.669793	0.536168	0.350517	0.559449	-0.28713	0.030045	0.956926
-1.15879	-0.29577	-0.08834	0.248149	0.082136	-0.38826	-0.62404	0.002399	0.872188
-0.99285	0.530628	0.227852	0.821384	0.395174	0.754385	0.562221	0.071051	0.593532
-1.15879	0.305844	0.596614	0.903465	0.738575	0.564795	0.688638	0.085663	0.597621
-0.7916	0.748049	0.990522	1.297763	0.990814	1.300445	1.029985	0.035512	0.552396
-0.99285	0.907809	1.233967	1.537567	1.065132	1.13021	0.999126	0.001673	0.629757
-0.99285	0.18045	0.08162	0.23337	0.012052	0.023853	-0.20739	0.062952	0.440758
0.213393								
0.213393	0.397796	0.651534	0.872639	0.720449	1.12765	0.50636	0.014748	0.729854
0.213393	0.056611	0.168143	0.349557	0.645452	0.54594	0.380924	0.002735	0.588841
0.45785	1.138647	1.385447	1.130317	1.411177	1.89189	1.651983	0.001719	0.761573
	0.020501	-0.09177	0.233903	0.115153	0.705153	0.450677	0.012362	0.796927
0.903847								
0.903847	-0.01406	-0.17023	-0.0119	0.344361	0.624727	0.310953	0.008546	0.794958
0.903847	0.388193	0.342622	0.097284	0.697115	1.03335	0.616939	0.000954	0.936066
-0.47229								
0.70351	0.77451	1.166182	1.351507	0.631711	1.334765	1.10654	0.046069	0.719795
0.70351								
-0.47229	-1.0424	-1.57706	-1.68361	-1.1239	-1.22633	-1.30084	0.008106	0.641208
-0.47229	-1.04621	-1.38653	-1.40531	-0.92605	-1.13847	-0.83766	0.011806	0.567652
-0.47229	0.074707	0.549408	0.763192	0.143295	-0.15961	-0.14697	0.002152	0.922807
0.984063								
0.984063	-0.31119	-0.6318	-0.78115	-0.28324	-0.44268	-0.25473	0.037806	0.526422
0.984063	-0.15352	-0.7051	-0.68283	0.132674	0.255315	-0.16838	0.005303	0.960416
0.213163	0.396235	0.387247	0.483961	0.204409	0.168472	0.397835	0.069019	0.315489
0.213163	-0.67897	-0.92485	-0.96133	-0.48428	-0.61213	-0.46732	0.022303	0.494008
0.213163	0.496297	-0.22533	-0.37153	0.709445	0.130195	0.503609	0.044783	1.080977
-0.57636	1.212399	0.603772	0.612272	0.685811	0.750465	0.584665	0.080408	0.627735
-0.57636	-0.53101	-0.88847	-0.91986	-0.62421	-0.81894	-0.67169	0.039588	0.38885
-0.57636	0.439203	1.221847	1.46976	0.881059	1.1235	0.665575	0.004873	1.030557
-0.97407								
-0.97407	-0.75468	-1.04102	-1.16774	-0.96694	-1.03641	-0.83143	0.013856	0.413053
-0.97407	1.129433	0.912765	0.815747	0.529471	0.515514	0.553576	0.036352	0.613919
-0.37902								

-0.37902	-0.24036	-0.6426	-0.65603	-0.0856	0.187613	-0.2275	0.205653	0.843644	
-0.37902	0.737203	0.852958	0.922197	0.752717	1.243625	1.032158	0.018111	0.506422	
0.068247	0.795812	0.854524	1.044771	0.792019	1.219065	1.127547	0.010795	0.427046	
-0.67789									
-0.67789	-0.31544	-0.48242	-0.27204	-0.30528	-0.3503	-0.49574	0.191091	0.223697	
-0.67789	0.318167	0.52308	0.925629	0.429051	0.450566	0.236761	0.009127	0.688868	
2.012767	0.253687	0.348753	0.607365	0.249553	-0.00226	0.329557	0.071916	0.60963	
2.012767	-0.54118	-0.71333	-1.09926	-0.71542	-0.66077	-0.47646	0.004861	0.622792	
2.012767	-0.24903	-0.49832	-0.37577	-0.13048	-0.25471	-0.25431	0.050957	0.367835	
-0.92659	0.328372	0.335086	0.301642	0.127007	0.33071	0.530134	0.119802	0.403128	
-0.92659									
-0.92659	0.624064	0.999743	1.48787	1.091154	0.966742	0.63685	7.55E-05	0.863806	
1.877653	-0.49811	-0.54393	-0.5007	-0.23405	-0.3591	-0.47549	0.046857	0.30988	
2.7563	0.173103	0.328665	0.485178	-0.22125	-0.50759	0.262822	0.083877	0.99277	
2.7563	0.553053	0.741384	0.766205	0.480389	1.314165	0.940501	0.015099	0.833776	
2.7563									
1.877653	-0.35494	-0.31093	-0.21141	-0.18117	0.152833	-0.05314	0.006812	0.507768	
1.877653	-0.47326	-0.73041	-0.71502	-0.28844	-0.24296	-0.32294	0.023667	0.487452	
1.877653	-0.83177	-1.18613	-1.39815	-1.00184	-0.79587	-0.84717	0.009014	0.602284	
1.122262	0.294697	0.744287	0.942907	0.359847	0.141426	0.288039	0.066193	0.801481	
1.122262	0.506068	0.358207	0.306537	0.602085	0.748863	0.570479	0.038151	0.442325	
1.122262	-0.42955	-0.38664	-0.3625	-0.33108	0.032477	-0.30495	0.069224	0.462027	
1.122262	0.982766	1.79139	1.739813	0.828922	1.522545	1.274215	0.028068	0.962468	
-0.33644									
-0.33644	0.47797	0.537545	0.614549	0.31932	-0.07946	0.405369	0.032106	0.694011	
-0.47796	0.012154	0.119954	0.206247	-0.18901	0.402681	0.190561	0.01147	0.591692	
-0.47796	-0.92534	-1.35776	-1.3935	-0.65982	-0.99533	-0.7048	0.015461	0.73368	
-0.47796	1.945899	-0.01328	-0.61018	-0.24426	-0.17273	-0.48631	0.035021	2.556076	
-0.47796	0.530431	0.658873	0.769204	0.183214	0.224963	0.196026	0.022873	0.58599	
-0.54631									
-0.54631	1.338795	0.233339	-0.40905	-0.06431	0.430315	0.502388	0.025698	1.747849	
-0.54631	0.623533	1.16761	1.567067	1.057181	0.775546	0.5112	0.008541	1.055867	
-1.18281									
-1.18281	1.170873	1.41849	1.68088	1.153557	0.639599	0.513864	0.000341	1.167016	
-1.28403									
-1.28403	0.540565	1.25081	1.554807	0.752244	0.540359	0.114953	0.006307	1.439854	
-0.68899	1.923677	1.88309	1.66195	1.751857	1.634535	1.238189	0.082394	0.685487	
-0.68899	3.70687	3.73383	2.275623	2.409173	1.724865	2.07228	0.032529	2.008965	
-0.68899	0.403746	0.618777	0.758006	0.780008	0.987864	0.635309	0.006055	0.584118	
1.231767	-0.10101	0.424868	0.592609	-0.06819	0.833431	0.608255	0.008671	0.934445	
1.222563	1.283476	0.900387	0.827367	0.556412	1.010616	0.85891	0.121956	0.727064	
1.222563	0.374415	0.464567	0.61562	0.633772	0.920795	0.684589	0.032719	0.54638	
	-0.7853	-1.09403	-1.18332	-0.90105	-0.98677	-0.90037	0.121632	0.398016	
1.231767	-0.1272	0.021685	-1.13288	-0.6416	-0.63103	-0.3912	0.145091	1.154566	
1.231767	0.685225	0.927734	0.785023	-0.03097	0.34252	0.472135	0.135865	0.958701	
	-0.29571	-0.02541	-0.13935	-0.13447	0.50728	0.214949	0.043272	0.802991	
0.068923									

0.068923	-0.90164	-1.44968	-1.75843	-1.00442	-1.5053	-1.18897	0.002288	0.856787
0.068923	-1.10655	-1.60234	-1.48691	-1.03358	-1.49545	-1.17595	0.008369	0.568759
0.068923	-1.3164	-1.77902	-1.75607	-1.34009	-1.55346	-1.22918	0.066448	0.549839
-0.46527								
-0.46527	-0.02987	0.050072	0.145636	-0.25137	0.164129	-0.07943	0.010251	0.415497
-0.46527	0.922377	1.043805	1.386403	0.961709	1.424985	0.987858	0.016142	0.502608
0.642123	-0.1689	-0.18721	-0.22916	-0.08758	0.399859	-0.01484	0.003323	0.62902
0.642123	-0.49629	-0.67656	-0.68325	-0.65228	-0.61145	-0.53527	0.282856	0.186957
0.642123	1.709663	2.24709	1.799397	1.86441	1.937195	2.013703	0.165307	0.537427
-0.87116								
-0.87116	0.707105	1.108662	1.300973	0.868734	0.838702	0.661952	0.000551	0.639021
-1.22608								
-1.22608	1.58495	1.718087	1.791427	1.31585	1.428965	1.428	0.008305	0.475577
-1.16409								
-1.16409	0.823308	1.54136	1.968433	1.237942	0.923265	0.608063	0.003514	1.36037
-0.94621								
-0.94621	-0.18006	-0.04893	0.189207	0.04187	-0.12939	-0.15562	0.020637	0.369264
-0.94621	0.659745	1.205918	1.512553	0.768436	1.12797	0.79883	0.011086	0.852808
0.4338	0.910488	1.39678	1.444063	1.09794	0.983556	0.985278	0.004033	0.533576
0.4338	1.075803	0.874787	0.677894	0.373004	1.011882	1.012179	0.044873	0.7028
-0.43468	2.838217	2.700793	2.296473	2.189167	2.229175	1.574777	0.022979	1.26344
0.4338	-0.36707	-0.32038	-0.52981	-0.05311	-0.13121	-0.14164	0.023955	0.476707
-0.43468	-0.03563	-0.09271	-0.07833	0.227125	-0.07276	-0.05655	0.031491	0.319833
-0.43468	-0.85241	-1.52515	-1.79462	-1.1085	-1.32183	-0.8782	0.007331	0.942205
0.222795	-0.81419	-1.25758	-1.76213	-0.89096	-1.18188	-0.81897	5.48E-05	0.947941
0.222795	-0.99628	-1.26216	-1.31832	-0.82656	-1.14709	-0.99756	0.070703	0.491759
0.222795	-0.54639	-0.45239	-0.56111	-0.60208	-0.25957	-0.30487	0.079718	0.342516
0.71497								
0.222795	-0.25925	0.053361	0.059497	0.390102	0.583023	0.265771	8.63E-05	0.842274
0.71497	-0.36575	-0.9686	-1.54842	-0.87118	-1.11707	-0.91578	0.004575	1.182674
0.71497	-0.59305	-1.16141	-1.31854	-0.81348	-0.99663	-0.85823	0.004764	0.725494
-0.27717								
-0.27717	-0.48618	-0.44357	-0.41862	-0.29326	-0.31909	-0.31226	0.079049	0.192917
-0.27717	-1.08588	-1.36582	-1.56286	-1.01032	-1.39993	-1.19618	0.051395	0.552541
-0.27717	-0.80578	-1.19679	-1.21487	-0.90649	-0.95265	-0.81803	0.027709	0.409087
-0.27717	0.578812	0.999382	0.855746	0.248023	1.002545	0.733239	0.035062	0.754521
0.47505	0.836819	0.987701	0.737563	0.666776	1.414945	1.37906	0.000519	0.748169
0.748253								
0.748253	2.99095	3.04302	2.199177	3.357123	2.861175	2.05301	0.185147	1.304113
0.47505	-0.63392	-0.69797	-0.40262	-0.73036	-0.65412	-0.54957	0.077258	0.327734
0.47505	2.906993	1.213558	1.791111	1.708838	1.287652	0.756357	0.053531	2.150636
0.752033								
0.752033								
0.752033	-0.53759	-1.12028	-1.23084	-0.72498	-0.63661	-0.72234	0.084571	0.693255
0.585873	0.809443	0.824867	1.079193	0.63092	1.105054	1.136473	0.004874	0.505553
0.585873	2.165853	2.49112	2.406317	1.22331	1.894885	1.826907	0.04451	1.26781
0.300595	4.670877	4.485933	3.48958	3.505683	3.84213	3.242627	0.037315	1.42825

0.300595	-1.12306	-1.3231	-1.71219	-1.31101	-1.33723	-1.36786	0.021606	0.589134
0.300595	-1.40013	-1.64718	-1.35292	-1.15722	-1.98431	-1.33477	0.041218	0.827085
0.300595	-0.69014	-1.22465	-1.07079	-1.02986	-1.60106	-1.20128	0.007564	0.910918
-0.14655	0.463087	1.009902	1.65103	0.887565	0.766115	0.624346	0.000486	1.187943
-0.14655	-0.87121	-1.081	-1.20094	-1.13211	-1.32154	-1.04084	0.042504	0.450326
-0.14655	-0.50804	-0.59262	-0.45189	-0.43797	-1.03156	-0.65397	0.04177	0.593583
0.029627								
0.029627	-0.24054	-0.48467	0.700518	0.357807	-0.06791	-0.26106	0.004601	1.185193
0.029627	-1.06376	-1.23904	-1.30888	-1.05327	-1.00332	-1.06643	0.017801	0.305562
-0.05035								
-0.05035	-0.41867	-0.62492	-0.5937	-0.31344	-0.09694	-0.11716	0.003873	0.527978
-0.05035	-0.61567	-0.73936	-0.73851	-0.53229	-0.43135	-0.47467	0.002858	0.308013
-1.11321								
-1.11321	0.429127	1.30394	1.739523	0.891937	0.733841	0.444034	1.71E-05	1.310396
-0.34689								
-0.34689	0.43947	0.855145	1.272803	1.088315	1.29766	0.9724	0.034027	0.85819
0.41124	6.568913	5.414984	4.629023	4.638707	4.67994	4.051273	0.010456	2.51764
0.41124	-0.21557	-0.10227	0.093926	-0.10002	0.0081	-0.11084	0.0414	0.309498
0.41124	1.154305	1.345956	1.327907	1.050894	1.944095	1.597043	0.012711	0.893201
0.520287								
0.520287	-0.63867	-0.73742	-0.77767	-0.51131	-0.38095	-0.4027	0.021246	0.396717
0.520287	0.752556	1.443974	1.649047	0.838872	0.967269	1.034527	0.023887	0.896491
0.558283								
0.558283	0.630341	0.260874	0.37601	0.333445	0.159204	0.160333	0.104371	0.471137
0.558283	0.862527	0.840846	1.091959	0.668713	1.04013	1.012141	0.001418	0.423246
-1.0732	0.803325	0.799877	1.099083	0.771396	0.697271	0.950959	0.223357	0.401812
-1.0732	0.405357	0.497263	0.620655	0.538423	0.491909	0.444592	0.03941	0.215297
-1.0732	1.063379	1.43995	1.813207	1.204	1.184365	1.09708	0.003416	0.749827
-0.32353	1.622787	1.137905	0.587775	0.824299	1.213534	0.746399	0.069157	1.035012
0.523123	-0.65995	-1.1401	-1.33788	-1.0273	-1.09222	-0.87848	0.007306	0.677935
0.523123	-0.24718	-0.63932	-0.75747	-0.29495	0.063941	0.109894	0.065044	0.867364
0.523123	-0.65256	-1.31922	-1.47949	-0.72167	-1.11747	-0.83643	0.000455	0.826932
-0.32353	0.483101	0.871301	-0.24056	-0.10223	-0.28383	-0.06294	0.098814	1.15513
0.07677	0.781352	1.303807	1.33263	0.750965	1.34661	1.265867	0.007632	0.595645
0.185795								
0.185795	0.321322	0.509079	0.483599	0.342648	0.509776	0.503622	0.143542	0.188453
0.185795	-0.39854	-0.46806	-0.33915	-0.46753	-0.46137	-0.54647	0.216253	0.207319
0.185795	-0.65861	-1.06861	-1.38005	-0.67092	-0.80619	-0.72059	0.001246	0.721448
0.185795	1.057053	1.646997	1.602103	0.882653	1.41205	1.081027	0.004677	0.764343
0.07677	0.050079	0.131156	-0.42863	-0.31129	-0.12696	-0.32833	0.038404	0.559786
-0.60091	0.487836	0.742917	0.929941	0.357339	0.946485	0.718398	0.016903	0.589146
-0.60091	-0.00824	-0.70202	-0.82698	-0.35676	-0.59404	-0.62543	0.160047	0.818737
-0.60091	-0.87299	-0.4608	0.107064	-0.53923	-0.45887	-0.57538	9.55E-05	0.980057
-0.51216	0.38816	0.920443	1.216201	0.447743	1.194695	0.943364	0.022169	0.828041
-0.51216	-0.84212	-0.85456	-1.21936	-1.10391	-0.96222	-0.99356	0.043988	0.377241
-0.51216	0.427262	0.832196	1.05784	0.489587	1.07952	0.780282	0.0083	0.652258
0.37736	0.191599	0.318925	0.720378	0.269129	0.732383	0.51613	0.036882	0.540784

0.37736	-0.85342	-1.05875	-1.71854	-1.17661	-1.53241	-1.01201	0.01265	0.865119
0.37736	-0.21492	-0.77092	-1.39583	-0.79846	-1.17258	-0.88823	0.046301	1.180913
0.37736	0.231173	0.066767	0.061455	0.229081	0.504017	0.213257	0.017882	0.442561
0.096383	0.678735	1.156655	1.54234	0.997168	1.043065	0.707795	0.00156	0.863605
-1.1714	0.464273	0.999291	1.39874	0.947737	0.922798	0.747095	0.000128	0.934467
-1.1714	0.832072	1.351803	1.892123	1.376627	1.883495	1.27879	0.01925	1.060051
0.096383	0.431005	0.594681	0.394113	0.4808	0.734949	0.64458	0.094888	0.340836
0.096383	0.9078	1.400041	1.753103	1.617193	1.957645	1.34819	0.030614	1.049845
-0.43534	0.611176	1.085703	1.286533	0.559782	0.725817	0.748109	0.019689	0.726751
-0.43534	-0.2752	-0.75399	-0.77573	-0.34817	-0.50369	-0.25736	0.057919	0.518371
-0.43534	-0.63888	-0.96447	-0.90901	-0.45218	-0.51595	-0.50224	0.010144	0.512281
-0.72322	-0.14786	0.427953	0.788506	-0.03409	0.174672	0.307648	0.004869	0.936362
-0.72322	-0.53037	-0.56663	-0.65468	-0.64454	-0.70677	-0.49197	0.025733	0.2148
-0.96514	-0.10533	0.834059	1.352743	1.022062	0.289753	0.034611	0.005459	1.458077
-1.30265	0.323198	0.500405	0.351359	0.265055	0.857812	0.862457	0.062169	0.597402
-1.30265	-0.30384	-0.7064	0.038595	0.021568	-0.28712	-0.31043	0.004303	0.744992
-1.30265	-1.26579	-1.53207	-1.55218	-1.33268	-1.52565	-1.26621	0.037177	0.286398
-1.30265	-0.93994	-1.30785	-1.71883	-1.3438	-1.61273	-1.45619	0.0052	0.778891
-1.30265	0.004932	-0.56742	-1.27953	0.144056	-0.62168	-0.87909	0.144563	1.423586
-1.30265	-0.3365	-0.49922	-0.31452	-0.19467	-0.04874	-0.28049	0.000148	0.450474
-0.96514	0.378128	0.616943	0.609649	0.153078	0.538726	0.575344	0.006441	0.463865
-0.96514	-0.33252	0.077914	0.573514	0.044574	-0.60325	-0.7377	0.00143	1.311209
-0.16875	0.407723	0.620492	0.666156	0.100995	0.393655	0.384091	0.046317	0.565161
-0.16875	0.433171	0.751068	0.636297	0.970426	1.348915	1.022399	0.012077	0.915744
-0.62681								
-0.62681	-0.26309	-0.38249	-0.24404	-0.11136	0.070406	-0.06428	0.007446	0.452895
-0.62681	0.297534	0.539456	0.780834	0.593708	0.55208	0.494467	0.009071	0.4833
-0.06603								
-0.06603	-0.36351	-0.32552	-0.37653	-0.26482	-0.34309	-0.3316	0.293772	0.111713
-0.06603	-1.11731	-1.49385	-1.68052	-0.80973	-1.50965	-1.00427	0.003787	0.870784
-0.06603	-0.43264	-0.59297	-0.82573	-0.46891	-0.54305	-0.54852	0.121814	0.393092
0.102572								
0.102572	-1.24191	-1.71979	-1.87551	-1.30479	-1.5101	-1.20165	0.001793	0.673867
0.102572	-0.69268	-0.99955	-1.23271	-0.62942	-0.78933	-0.74012	0.018502	0.603293
0.102572	-0.54501	-0.66953	-0.85106	-0.55168	-0.45107	-0.47573	0.00468	0.399996
0.010823	0.991682	1.035999	1.246517	0.755855	1.082767	1.009041	0.022947	0.490662
0.010823	1.023721	1.30218	1.33593	0.46624	1.069711	1.147543	0.004709	0.86969
0.008202	0.99995	1.212194	1.443217	1.104414	1.268068	1.17134	0.039618	0.443266
0.008202	-0.56334	-0.55777	-0.06062	0.2494	-0.13333	-0.39368	0.000218	0.81274
1.891867	0.723164	1.231061	1.580433	0.306276	0.667541	1.089392	0.003388	1.274157
1.891867	-0.15328	0.043348	-0.33701	-0.23564	-0.14394	-0.13785	0.012611	0.380357
1.891867	0.153498	0.495766	0.6274	-0.28675	0.609609	-0.04144	0.039234	0.914151
-0.80777								
-0.80777	-1.07048	-1.50405	-1.73801	-1.1228	-1.46341	-1.22641	0.015056	0.667526
-0.80777	-0.25965	-0.86419	-1.03707	-0.55397	-0.68076	-0.59932	0.016757	0.777421
-0.80777	-0.8817	-1.37953	-1.58649	-0.86735	-1.3977	-1.04687	0.015018	0.719141
-0.80777	-0.28213	-1.22628	-1.42274	-0.63323	-0.83662	-0.8045	0.006828	1.14061

0.573795	0.365462	0.113814	0.154485	0.144432	0.456863	0.473747	0.016449	0.359932
-0.81729								
-0.81729	-0.73375	-1.53543	-1.57708	-1.05253	-1.29463	-1.13463	0.017074	0.843329
-0.81729	-0.96952	-1.38096	-1.48063	-1.07451	-1.26669	-0.95537	0.033288	0.525255
0.573795	0.559757	0.817035	1.62456	1.54751	1.473025	1.208094	0.01148	1.064803
0.573795	-0.20313	-0.32347	-0.29078	0.212779	0.339535	-0.08542	0.004312	0.663
0.244037	0.530578	0.854556	1.353837	0.98978	1.134719	1.040624	0.003336	0.823259
0.244037	-0.75618	-0.85192	-0.90988	-0.78417	-0.7394	-0.75726	0.179505	0.17048
0.244037	1.501253	1.646167	1.540243	1.158733	1.744095	1.29747	0.005679	0.585362
-0.32081	0.147682	0.148398	0.092015	0.122929	0.671284	0.506343	0.014854	0.579269
-0.32081	-0.23931	-0.8053	-0.87011	-0.36513	-0.42457	-0.56145	0.004343	0.630803
-0.32081	-0.51741	-0.56786	-0.50949	-0.33346	-0.40933	-0.48239	0.128267	0.2344
0.792357								
0.792357	-0.36633	-0.4945	-0.72782	-0.39036	-0.42814	-0.40052	0.056756	0.361491
0.516523								
0.516523	0.450676	0.457501	0.27194	0.097251	0.652908	0.605837	0.007614	0.555656
-0.06849	0.25567	0.170355	0.517754	0.316901	0.834915	0.375841	0.053962	0.66456
-0.06849	0.038696	-0.18575	-0.20267	0.135305	0.197411	0.086072	0.098083	0.400081
0.494697	-0.55553	-0.9064	-1.2128	-0.53667	-0.38152	-0.25759	0.006339	0.955205
0.106627								
0.106627	-0.33044	0.018207	0.123834	0.227003	-0.21266	-0.33454	0.022973	0.561543
0.106627	-0.61132	-0.91484	-0.90914	-0.68969	-0.74708	-0.54515	0.028062	0.369685
-0.19999								
-0.19999	-0.24119	-0.49168	-0.88395	-0.83125	-0.51951	-0.52817	0.017779	0.642763
-0.19999	1.029021	1.542363	1.53657	1.198021	1.23632	0.631485	0.017692	0.910878
2.787627	0.430977	0.697242	0.47517	0.169131	0.716661	0.683346	0.001511	0.54753
2.787627	-0.2842	-0.15317	-0.23992	-0.36924	-0.26122	-0.21475	0.016802	0.216065
2.787627	-0.10527	-0.61023	-0.67591	-0.27043	-0.07652	-0.08961	0.057667	0.599392
0.3908	0.217929	0.472332	0.449711	0.28892	0.382463	0.230833	0.037735	0.254403
0.3908	0.210474	0.157524	0.081628	0.130111	0.869847	0.644296	0.006576	0.788218
0.3908	0.552482	-0.8641	-0.99134	0.296348	-0.28626	0.04479	0.111603	1.54382
0.359823	0.711782	0.49884	0.8037	0.775305	1.083445	0.809325	0.00303	0.584605
0.359823	1.578347	1.330456	0.564067	0.854152	2.08404	0.658719	0.124503	1.519973
0.359823	0.309591	0.206064	0.385197	0.889932	0.666364	0.636751	0.007809	0.683867
-1.23363								
-1.23363	1.501033	1.572573	1.297709	0.846186	1.758855	0.967597	0.02056	0.912669
-0.5167	0.470416	0.5506	0.969593	0.896876	1.26467	0.997867	0.052476	0.794254
-0.5167	-0.85494	-1.26177	-1.32934	-0.88428	-0.97812	-0.91765	0.014542	0.474393
-0.5167	-0.36696	-0.44722	-0.48673	-0.3786	-0.2225	-0.16353	0.082023	0.323198
1.568813	-0.17121	0.226065	0.394339	-0.1792	0.543931	0.348108	0.069477	0.723129
	0.382121	0.371493	-0.01816	0.045071	0.213803	-0.14625	0.080341	0.528373
1.568813	-0.40057	-0.41846	-0.35936	-0.45926	-0.07013	-0.1987	0.087007	0.389129
-0.19689								
-0.19689	-1.26617	-1.22731	-1.02577	-0.96794	-1.12976	-1.04215	0.045142	0.298232
-0.19689	0.69313	1.009321	1.316487	0.966842	1.26152	0.982961	6.40E-06	0.623357
-0.39038	0.010298	0.347695	0.484095	-0.00425	0.869972	0.764026	0.006011	0.874222
-0.39038	0.229266	0.989383	1.574793	0.698187	0.796276	0.410756	0.000508	1.345527

1.550347	-0.83999	-1.14963	-1.63858	-0.91759	-0.78284	-0.72932	0.015401	0.909259
1.922063	5.382803	5.393967	5.049523	4.46212	4.726005	4.256527	0.051995	1.13744
1.550347	-1.45326	-1.83855	-1.88683	-1.2886	-1.72099	-1.45181	0.011402	0.598227
1.550347	-0.90671	-1.29184	-1.15308	-0.84579	-0.80624	-0.92387	0.015465	0.485603
1.922063	-0.63084	-1.19029	-1.19399	-0.85872	-0.71934	-0.59846	0.032278	0.595522
1.922063	0.023582	-0.06097	-0.49531	-0.42397	0.087663	0.097439	0.006598	0.592748
0.49311	1.134944	1.263048	1.290551	1.175567	1.432088	1.308297	0.2067	0.297144
0.49311	-0.30737	-0.18349	0.158839	0.474782	0.460461	0.039974	0.006352	0.782152
0.49311	0.754042	0.53957	0.507771	0.036348	0.136682	0.774901	0.238778	0.738553
0.49311	-0.42165	-0.33433	-0.25707	-0.40638	-0.25432	-0.18793	0.113232	0.233718
0.076887	0.212307	0.570286	0.675432	0.577381	1.362295	1.036436	0.003142	1.149988
-0.037	-0.20801	0.096521	0.058243	0.186308	0.685853	0.515987	0.009429	0.893864
0.076887	-0.37778	-0.61607	-0.59523	-0.52954	-0.30417	-0.38222	0.158492	0.311905
0.076887	-0.18948	-0.53682	-0.60372	-0.79264	-0.27613	-0.20673	0.003731	0.603157
-0.85171	1.706317	1.841763	1.391154	1.370362	1.76644	1.505877	0.06021	0.471401
-0.85171	0.411956	0.818264	1.386873	0.705798	0.495348	0.252132	0.00203	1.134741
-0.85171	0.7379	1.177819	1.872983	1.025108	1.219755	0.854082	0.001976	1.135083
-0.67917								
-0.67917	0.817915	0.443896	0.520029	-0.08559	0.221616	-0.24855	0.042185	1.066464
-0.67917	0.753296	1.073321	1.55376	1.19349	1.34026	0.962088	0.032911	0.800464
0.301567	0.943837	1.114212	1.070916	0.777543	1.38237	1.28973	0.058201	0.604827
0.301567	1.539653	1.494787	1.325383	1.99993	1.824465	1.459527	0.085574	0.674547
0.27943	0.689189	0.611739	1.203305	0.970523	1.069364	0.859204	0.05187	0.591566
0.27943	-0.48314	-0.71762	-0.71731	-0.26967	-0.17773	-0.06599	0.016537	0.65163
0.27943	0.797988	0.682962	1.070806	0.368023	0.703394	1.151274	0.18848	0.783251
0.970817								
0.970817	0.300045	0.556397	1.273607	1.151853	1.005759	0.633604	9.27E-05	0.973562
-0.37152	-0.81369	-1.52065	-1.65671	-0.99481	-1.18609	-1.11497	0.025083	0.843022
-0.37152	-0.4308	-1.04943	-1.19844	-1.0056	-1.41505	-0.9712	0.00738	0.984241
0.403253	0.822064	0.688076	0.776785	0.611044	0.613465	0.842173	0.103726	0.231128
0.403253	-0.30985	-0.16203	-0.26291	0.299692	0.574785	0.147281	0.032519	0.884635
0.403253	-1.24109	-1.83359	-1.56326	-1.27343	-1.40187	-1.44057	0.012665	0.592502
0.763473								
0.763473	-0.49192	-0.44186	-0.6095	-0.8347	-0.61362	-0.56069	0.028769	0.39284
0.763473	-0.13093	-0.20818	-0.18565	-0.3222	0.072034	0.155612	0.003476	0.477808
0.585432	0.096365	0.457466	0.839541	0.237261	0.460764	0.192025	0.043879	0.743176
0.585432	-0.52586	-0.85399	-0.78229	-0.53001	-0.33676	-0.24935	0.001337	0.604638
0.585432	-1.08185	-1.56556	-1.87021	-1.23444	-1.50086	-1.21649	0.00131	0.788357
0.585432	-0.1985	-0.22261	-0.2454	-0.49092	-0.22125	-0.37779	0.087562	0.292417
0.585432								
0.585432	-0.85789	-1.26083	-1.54774	-0.61406	-1.21391	-0.905	0.045155	0.933678
0.585432	-1.01095	-1.64066	-1.93873	-1.07062	-1.41859	-1.12	0.000995	0.927784
0.585432								
0.585432	-0.71897	-0.89909	-0.98204	-0.84202	-1.03181	-0.89748	0.048691	0.312833
0.585432	0.602747	1.50476	1.98572	0.972264	1.28571	0.857775	0.001401	1.382973
0.769593	0.027403	0.230932	0.39402	0.229554	-0.09773	0.01384	0.045014	0.491746
0.769593	-0.46067	-0.32352	0.191818	-0.10131	-0.18145	-0.55554	0.036252	0.747355

0.769593	-0.85446	-1.32415	-1.56804	-0.95575	-1.39752	-1.03737	0.000991	0.713587
0.769593	-0.31849	-0.36095	-0.39286	-0.13514	-0.46072	-0.36311	0.032113	0.325577
0.089833	0.403406	0.562181	0.869203	0.210513	0.42055	0.536976	0.012062	0.658691
0.089833	1.10397	1.202006	1.243883	0.716569	1.154625	0.920085	0.022069	0.527315
0.048387								
0.048387	0.317121	-0.11356	0.20502	-0.09581	-0.79932	-0.47219	0.058944	1.116439
0.048387	0.009604	0.414657	0.813381	0.313603	0.542828	0.033306	0.061834	0.803778
0.048387	-0.56035	-0.50053	-0.60089	-0.56732	-0.30111	-0.32958	0.146182	0.299789
0.627827	0.692579	1.090173	0.952051	1.101239	0.995235	0.825942	0.147528	0.408659
0.627827	-1.29756	-1.74238	-1.76435	-1.19839	-1.46475	-1.28575	0.022573	0.565963
0.627827	-1.31878	-1.72385	-1.42019	-1.39017	-1.66362	-1.42164	0.00366	0.405077
0.627827	-1.17471	-1.67729	-1.92541	-1.0682	-1.44107	-1.30781	0.001493	0.857211
0.627827	-0.79273	-0.76348	-0.50763	-0.52757	-0.83278	-0.81156	0.099276	0.325155
0.265632								
0.627827	0.03526	0.018246	0.387897	0.030303	0.04593	0.224457	0.027147	0.369651
0.265632	-0.1865	-0.27867	-0.12665	-0.33192	-0.18728	-0.14866	0.065345	0.205273
0.265632								
0.265632	0.137646	0.477573	0.729048	0.108678	0.716459	0.310944	0.009214	0.62037
-1.05409	0.282803	0.717283	0.960514	0.819644	0.658426	0.412676	0.011351	0.677711
-1.12964	1.43622	1.289381	1.7786	1.769737	1.431895	0.922562	0.022046	0.856038
-1.12964	0.538483	1.025761	1.644727	0.84383	0.025828	-0.03271	0.007771	1.677434
-1.05409	-0.69577	-0.6447	-0.05307	-0.09074	-0.45354	-0.75126	0.047348	0.698184
-1.05409	-0.07791	0.795925	1.105253	0.41186	-0.21264	-0.31124	0.04074	1.416496
0.119837								
0.119837	-0.03435	0.297892	0.014038	0.130004	-0.13168	0.107662	0.113921	0.429574
-0.83558								
-0.83558	0.373876	-0.08424	-0.78677	-0.3766	-0.01893	0.480962	0.050539	1.267729
-0.83558	1.330053	1.61836	1.989353	1.509767	1.63353	1.413483	0.008597	0.6593
1.055543	0.014112	0.004534	-0.03859	-0.11108	0.160068	0.110372	0.039118	0.271151
1.055543	1.029532	1.255597	1.272488	0.612572	1.29026	1.402363	0.02199	0.789792
-0.44725	0.005545	0.069548	-0.26058	-0.01448	-0.53823	-0.07074	0.115144	0.607781
-0.44725	-0.86874	-1.24704	-1.3663	-1.02442	-1.22838	-1.14244	0.002429	0.497558
-0.44725	-1.10286	-1.61777	-1.39197	-1.16236	-1.36125	-1.30844	0.00902	0.514913
-0.44725	-0.03595	0.07308	-0.84487	-0.59269	-0.21438	-0.54714	0.021084	0.91795
-0.44725	-0.25157	-0.27562	-0.52962	-0.37402	-0.80508	-0.60426	0.099792	0.553508
-0.44725	-1.19877	-1.42946	-1.78809	-1.12682	-1.60099	-1.2191	0.061355	0.661265
-0.44725	-1.31735	-1.6075	-1.90859	-1.11827	-1.624	-1.40427	0.005303	0.790324
-0.44725	0.309074	-0.18507	-0.90885	-0.35256	0.223366	-0.59978	0.036113	1.217929
-0.44725	-1.16165	-1.66592	-1.80853	-1.10566	-1.36841	-0.88602	0.028515	0.922513
-0.44725	-0.84086	-1.41148	-1.76064	-0.80491	-1.0886	-0.71828	0.02131	1.042367
-0.44725	-0.43532	-1.01532	-1.29329	-0.84694	-0.92308	-0.70761	0.01119	0.857974
-0.44725	-0.07698	-1.51797	-0.29499	-0.15484	-1.06069	-1.07194	0.109014	1.440986
-0.44725	1.177033	1.613533	1.470546	0.837321	1.64413	1.28929	0.015386	0.806809
-0.54572								
-0.44725								
-0.54572	-0.77919	-1.16152	-1.10759	-0.62247	-0.99822	-0.7316	0.000478	0.539049
-0.54572								

-0.84167								
0.166623	-0.40219	-0.59687	-0.37305	-0.06999	-0.49821	-0.36358	0.014376	0.526885
-0.76572								
-0.76572	0.925657	0.601676	0.809761	1.056188	0.716604	0.790121	0.044024	0.454512
0.166623	0.13561	0.152555	0.171813	0.601173	0.533356	0.42708	0.020298	0.465562
0.188123	0.975338	1.153106	1.375307	1.156867	1.62012	1.511917	0.017232	0.644782
0.188123	0.818343	0.170303	-0.21115	0.114427	-0.05426	0.139032	0.039168	1.029497
0.188123	0.109835	-0.16091	-0.49073	-0.16261	0.007459	0.606403	0.116775	1.097129
0.07028	0.577329	0.910508	1.089585	0.676664	1.391545	1.175267	0.006342	0.814216
0.07028	0.352818	0.653764	0.821457	0.618811	1.31344	0.811926	0.017929	0.960622
0.983197	0.801012	1.113693	1.162377	0.183278	0.481144	0.749833	0.002409	0.979098
0.983197	-0.60227	-0.48205	-0.62715	-0.40734	-0.09903	-0.28147	0.000147	0.528114
0.983197	0.0033	0.084781	0.342466	0.321493	0.272303	0.180756	0.089902	0.339165
1.384928	0.376403	0.402483	0.839327	0.058426	0.30144	0.528583	0.006925	0.780901
1.384928								
1.384928	0.783273	-1.12103	-1.87682	-0.6085	-1.01437	-0.96076	0.053485	2.660093
1.384928	-0.19086	-0.63403	-1.3034	-0.65556	-0.95446	-0.77151	0.020289	1.112536
1.384928	-0.1228	-0.29136	-0.70972	-0.21157	-0.52358	-0.0453	0.163529	0.664417
1.384928	-0.65996	-1.05447	-1.29763	-0.74686	-1.04072	-0.89147	0.012755	0.637665
-0.73444	0.163296	1.028528	1.603557	0.718207	1.11031	0.517046	0.003212	1.44026
-0.73444	0.858042	1.009557	0.913579	0.752559	1.18359	1.143673	0.037047	0.431031
-0.25772								
-0.25772	0.720125	0.943296	1.241731	1.264017	1.52842	1.220317	0.010232	0.808295
2.761595	1.355447	1.253131	1.263113	1.103232	1.55967	1.512743	0.124019	0.456438
2.761595	-0.46654	-0.71786	-0.41625	-0.22748	-0.26264	-0.22301	0.012751	0.494847
2.761595	-1.25541	-1.59051	-1.96837	-1.35983	-1.68484	-1.25165	0.012618	0.71672
2.761595	-0.8048	-1.20437	-1.39202	-0.87935	-1.07928	-0.70854	0.005147	0.683478
0.18546	-0.84962	-0.95723	-1.45077	-0.80188	-1.31065	-1.06341	0.026495	0.648889
0.18546	0.085878	0.036275	0.088625	0.500346	0.498336	0.283339	0.002391	0.464071
0.18546	0.264282	0.345922	0.55476	0.441836	0.102093	0.31653	0.096234	0.452667
-0.24696	0.186985	0.219542	0.618409	0.54289	0.471118	0.462576	0.017424	0.431424
-0.24696	-1.03137	-1.42298	-1.39218	-0.94781	-1.09053	-1.06533	0.028545	0.475166
-0.24696	-0.61079	-1.07659	-0.9554	-0.31954	-0.55918	-0.48939	0.002543	0.757052
-0.23834	0.185172	0.46786	0.807753	0.476055	0.389715	0.409078	0.012082	0.622582
-0.23834	0.314079	0.366489	0.558434	0.619607	0.567121	0.153909	0.033626	0.465698
-1.29481								
-1.29481	-0.49716	-0.65781	-0.25351	-0.18881	-0.52439	-0.4964	0.030745	0.469
-1.29481	-0.2297	0.147663	1.11202	0.670576	0.237777	0.232418	0.012721	1.34172
-0.30876	0.422633	1.026694	1.70315	1.029033	0.914059	0.504308	0.000444	1.280517
-0.30876	-0.74728	-1.1428	-1.08942	-0.68531	-0.66397	-0.46341	0.050187	0.679395
-0.30876	-0.85539	-1.28522	-1.52784	-0.55118	-1.05309	-0.83856	0.010973	0.976652
-0.30876	-0.34141	-0.31519	-0.25782	-0.27414	-0.40311	-0.32492	0.128193	0.145284
1.582163								
1.582163	0.088616	0.124119	0.899539	0.658965	0.665317	0.212373	0.013847	0.810923
1.582163	-0.80043	-1.03497	-1.16383	-0.79132	-0.86573	-0.64914	0.006332	0.514687
1.582163	-0.87919	-1.56246	-1.64548	-0.96553	-0.5604	-0.67397	0.000398	1.085073
1.582163	-0.26963	0.074003	0.590505	0.420812	0.024207	-0.08951	0.001094	0.860136

0.962937								
0.962937	-0.79113	-0.47594	-0.41877	-0.20325	-0.8795	-0.38657	0.058581	0.676249
0.962937	0.397379	0.500603	0.30278	0.082871	-0.55648	-0.17242	0.011624	1.057086
-0.55651								
-0.55651	-0.2062	-0.29887	-0.12466	0.165482	0.150394	0.070298	0.005032	0.464354
-0.55651	-0.38169	-0.65803	-0.58307	-0.81771	-0.94991	-0.85367	0.103591	0.568218
-0.55651	0.452108	-0.484	-0.61472	-0.45782	-0.83622	-0.56307	0.059565	1.288325
1.843893								
1.843893	-0.80103	-1.51862	-1.44007	-1.07944	-1.20418	-1.0854	0.027378	0.717591
1.843893	0.841742	0.415652	0.011591	0.483671	0.038967	0.450827	0.138913	0.830151
0.083843	-0.36055	-0.29513	-0.24159	-0.58691	-0.52355	-0.28505	0.021443	0.345315
0.083843	-1.36745	-1.76294	-1.99754	-1.41603	-2.00503	-1.56282	0.072584	0.637583
0.083843	-0.30833	0.320235	0.706363	0.926076	0.654813	0.139146	0.003335	1.234405
-0.01413	0.184033	0.215095	0.915728	1.1313	0.786648	0.303147	0.004637	0.947267
-0.4882								
-0.01413	1.078217	0.798013	0.884877	0.60643	1.141245	0.8305	0.120561	0.534815
-0.4882	0.656047	1.324113	1.61099	0.91608	1.08047	0.711455	0.009196	0.954943
-0.05447	0.267213	0.444656	0.898656	0.512702	0.758012	0.754745	0.000759	0.631443
-0.05447	-0.20409	-0.09572	0.100937	-0.01408	0.181459	-0.10355	0.002141	0.385546
-0.05447	0.337624	0.508528	0.888726	0.286259	0.511636	0.462347	0.004247	0.602467
0.27301								
0.27301	0.53173	0.532769	0.635624	0.592691	1.264035	0.94068	0.006716	0.732305
0.92192								
0.92192	-0.68111	-1.18007	-0.91111	-0.31456	-0.9539	-0.62667	0.071712	0.86551
0.92192	-0.30466	-0.40829	-0.26248	-0.22298	0.297779	0.196396	0.00196	0.706071
1.996062								
1.996062	0.818521	0.932984	0.680564	0.159894	0.33286	0.502965	0.001382	0.773091
-0.65651	0.130562	0.348729	0.58205	0.204552	0.486551	0.223108	0.015868	0.451488
-0.65651	-0.24206	-0.23693	-0.27448	-0.29491	-0.09254	-0.11912	0.020582	0.202367
-0.65651	0.907135	0.772773	0.430902	0.481952	1.30568	0.699215	0.10824	0.874778
-0.10112	0.815527	1.15665	1.655877	1.229757	1.37857	1.087323	0.008455	0.84035
-0.10112	0.890458	1.041586	1.30157	1.226317	1.324845	1.033888	0.013593	0.434387
-0.70536								
-0.70536	-0.66819	-0.8523	-0.51841	-0.49362	-0.79355	-0.66422	0.013608	0.358678
-0.70536	0.038163	0.25127	0.111918	0.386609	0.319873	0.195624	0.005448	0.348446
0.610507								
0.610507	-1.05823	-1.50008	-1.73127	-0.92368	-1.24885	-1.08802	0.002217	0.807587
0.610507	0.644595	1.407362	0.321506	0.56842	0.232589	0.349432	0.194742	1.174773
0.02041								
0.02041	0.45193	0.626815	1.164377	0.961391	0.718052	0.341836	0.074157	0.822541
	-0.07754	0.024923	0.594264	0.55864	0.164481	0.174068	0.000301	0.671802
-0.54793								
-0.54793	-0.86008	-1.17559	-0.96854	-0.78031	-0.9655	-0.88987	0.040006	0.395278
-0.54793	-0.79909	-0.76055	-0.69976	-0.73693	-0.92128	-0.83053	0.108551	0.22152
0.155273								
0.155273	0.309533	0.634195	0.86613	0.719701	0.558553	0.456962	0.000337	0.556597

1.376433								
1.14796								
1.376433	-1.08308	-1.67253	-1.74101	-1.11296	-1.26042	-1.13591	0.008489	0.657934
1.376433	-1.1788	-1.63771	-1.8281	-1.25975	-1.49857	-1.22089	0.001215	0.6493
1.376433	-0.71677	-1.4824	-1.60161	-1.21015	-1.16588	-0.87726	0.026093	0.884832
1.14796	0.441463	0.8038	0.897248	0.38801	0.862604	0.785963	0.070006	0.509238
-0.76608								
-0.76608	-0.76297	-0.60821	-0.95419	-0.96576	-0.80785	-0.68272	0.127901	0.357546
-0.76608	-0.32956	-0.55874	-0.39684	-0.21181	-0.58133	-0.40378	0.063593	0.36952
-0.50071								
-0.50071	3.156647	2.74974	1.913627	1.92031	2.035775	2.033043	0.003566	1.24302
-0.09649	2.041165	1.772356	1.473566	1.531395	1.873503	1.09584	0.075787	0.945325
-0.09649	-0.38514	-0.23923	-0.55605	-0.09723	-0.24268	-0.2938	0.100648	0.458819
-0.09649	-0.25105	-0.11104	-0.07818	0.215865	0.040704	-0.0909	0.004617	0.466917
-0.02713								
-0.02713	0.251439	0.33078	0.150118	0.200109	-0.11532	0.048027	0.015603	0.446095
-0.02713	-0.79769	-1.38509	-1.54152	-0.94561	-1.33192	-1.01749	0.006189	0.743834
-1.03282								
-1.03282	0.736116	1.557323	1.835297	1.01945	0.575078	0.609528	0.004663	1.260219
0.91731	0.158248	0.432706	0.257655	-0.21398	0.437091	0.549001	0.013572	0.762979
0.91731	1.226346	1.310883	1.254867	1.835203	1.23243	0.92779	0.115346	0.907413
-0.40101	-0.96579	-1.39889	-1.56403	-1.33574	-1.43676	-1.2763	0.031991	0.598239
-0.87425	0.13251	0.587966	1.039732	0.220048	0.872038	0.699851	0.002159	0.907222
-0.87425	-1.04193	-1.44606	-1.74525	-1.09381	-1.38207	-1.20669	0.008835	0.703312
-0.87425	-0.97075	-1.3309	-1.7525	-1.17713	-1.28104	-1.26889	0.003234	0.781755
-0.87425	-0.51378	-0.77917	-1.36556	-0.78872	-0.9772	-0.43864	0.068116	0.926919
-0.87425	-0.74288	-1.02245	-1.17862	-0.85724	-1.10893	-0.91708	0.023678	0.435741
-0.40101	-0.76494	-0.6126	-0.07253	-0.4027	-0.29307	-0.31969	0.009093	0.692414
-0.40101	0.193778	-0.16031	-0.85652	-0.26936	-0.70284	-0.81989	0.063776	1.050296
0.295667	-0.99613	-1.35942	-1.45341	-1.16613	-1.48893	-1.15942	0.037684	0.492792
0.295667	0.245102	0.735925	0.872577	0.422095	0.788805	0.549135	0.001358	0.627475
0.859207	0.145089	0.466284	0.724988	0.353887	0.33665	0.348234	0.072069	0.579898
0.859207	0.154072	0.091045	0.508487	0.419852	0.001866	0.233072	0.019232	0.506622
2.223447	0.448931	0.650261	0.819506	1.051499	1.24443	1.043423	0.053856	0.795499
2.223447	-0.94489	-1.38381	-1.35507	-0.69449	-0.93422	-0.91176	0.034455	0.689319
2.223447	-0.51364	-0.8089	-0.83111	-0.52711	-0.39604	-0.41313	0.001795	0.435073
0.75457	1.156277	1.338117	1.157843	0.912783	1.289425	1.329283	0.026809	0.425333
0.245367	0.566297	0.684254	1.312841	0.749054	1.627365	1.168263	0.023005	1.061068
0.75457	0.512862	0.418798	0.647229	0.683381	0.417357	0.816334	0.177104	0.398978
0.245367	0.414924	0.480374	0.997621	0.486682	0.928124	0.811987	0.004448	0.582696
-0.16609	0.467304	0.615914	1.456697	0.543493	1.094865	0.688014	0.015854	0.989393
-0.16609	0.054247	0.422653	0.807597	0.443206	0.380151	0.386583	0.014226	0.75335
-1.18156								
-1.18156	-0.20006	-0.1249	0.401933	0.403313	-0.0035	-0.19229	0.007184	0.603377
0.823255								
0.823255	-1.19915	-1.3887	-1.52602	-1.40132	-1.61364	-1.663	0.056202	0.463847
0.823255	-1.48728	-1.99043	-2.13989	-1.52035	-1.76776	-1.37463	0.024902	0.765253

0.823255	0.620305	1.155127	0.434798	0.942693	-0.13698	0.819122	0.203031	1.292107
0.823255	-1.14862	-1.45303	-1.30599	-1.0118	-1.1969	-1.15218	0.002907	0.441227
1.24285								
1.24285	-0.88749	-1.46397	-1.4666	-0.95098	-1.25206	-1.01747	0.009521	0.579109
1.24285	0.928345	1.013919	1.44737	1.570277	1.478325	1.200106	0.02163	0.641932
-0.74629	-0.70104	-0.8668	-0.74342	-0.63016	-0.79102	-0.75709	0.082107	0.236645
-0.11777	-0.69173	-0.84112	-1.04783	-0.67804	-1.19334	-0.77145	0.097035	0.515304
-0.74629								
-0.74629	0.452072	0.456109	-0.08291	0.223075	-0.13914	0.149189	0.096273	0.595252
1.641267	1.38229	1.426908	1.482931	1.171572	1.494426	1.176548	0.103136	0.322854
1.641267	-1.10852	-1.71439	-1.70551	-1.29911	-1.54643	-1.43918	0.027545	0.605868
1.641267	2.566803	2.33253	1.966517	1.727663	2.19689	1.90517	0.036417	0.83914
-0.36477	-0.75853	-1.01483	-1.24579	-0.85136	-1.12954	-0.88455	0.03312	0.487255
-0.93977	0.781679	0.995786	1.327492	0.958823	1.19728	1.028372	0.002936	0.545813
-0.93977	0.000579	-0.7941	-1.07751	-0.71328	-0.50251	-0.44617	0.064999	1.078093
-0.93977	0.119134	-0.00314	-0.40841	-0.27066	0.215909	0.294573	0.000226	0.702984
-0.36477	0.018976	0.201447	0.118017	-0.16909	0.2487	0.092591	0.027561	0.417793
-0.36477	-0.08942	-0.11027	0.141088	-0.04967	0.471211	0.293059	0.019169	0.581476
-0.24223								
-0.24223	-0.93447	-1.17618	-1.13579	-0.95722	-1.01628	-0.97418	0.048263	0.24171
-0.24223	-0.1162	0.559237	1.099031	-0.19935	-0.05994	-0.12189	0.000473	1.298379
-0.17745	1.124006	1.003259	1.727977	1.144139	1.56308	1.37197	0.064315	0.724717
-0.17745	0.17195	-0.06064	0.203385	-0.24776	0.452452	-0.09156	0.227823	0.700209
-0.17745	-0.87065	-1.2233	-1.16971	-1.11339	-0.93522	-0.65685	0.067898	0.56645
1.722067	0.338092	0.622157	0.542167	-0.45185	-0.00898	0.221135	0.006123	1.074002
1.722067	-0.41198	-0.3495	-0.57683	-0.75737	-0.26658	-0.29113	0.132063	0.490791
-1.30164	0.24421	0.737173	1.216553	0.558374	0.601549	0.598062	4.78E-05	0.972343
-1.30164								
-1.30164	0.693559	0.833046	0.883387	0.189167	0.822512	0.598199	0.01319	0.69422
0.078477								
0.078477	1.133659	1.72583	1.772157	0.978253	1.46771	1.093457	0.008174	0.793904
-1.16412	-0.33221	-0.59058	-1.00665	-0.29915	0.064058	0.453813	0.003728	1.460467
-1.16412	0.841245	1.24056	1.669777	0.775093	1.034904	0.531905	0.007086	1.137872
1.397227								
1.397227	-0.43193	-0.5518	-0.6311	-0.21361	0.401341	0.099719	0.009414	1.032442
1.397227	-1.24819	-1.65087	-1.78145	-1.14469	-1.27633	-0.89828	0.011306	0.883172
1.397227	-0.93594	-1.00543	-0.74666	-0.53697	-1.45176	-0.61498	0.095174	0.914787
1.397227	-0.95761	-1.32195	-1.60536	-0.98103	-1.36586	-1.031	0.000781	0.647751
-0.65323	0.372605	0.480308	0.726513	0.556409	0.645298	0.491241	0.001714	0.353908
-0.65323	-0.36617	-0.55417	-0.61367	-0.3444	-0.59051	-0.39985	0.082692	0.269268
-0.65323	1.101939	2.062837	1.724053	1.46576	1.405965	1.199823	0.061252	0.960897
-0.13436	0.579509	0.739134	0.86228	0.336293	0.772833	0.678537	0.03409	0.525987
-0.13436	-0.81476	-1.18489	-1.34553	-0.82318	-1.02673	-1.2483	0.010724	0.53077
-0.13436	1.129137	1.47882	1.513527	1.285472	1.523265	0.865957	0.092218	0.657308
0.449857								
0.449857	0.116675	0.156289	-0.40776	-0.26546	-0.22233	-0.37713	0.243696	0.564046
0.449857	0.265955	0.693956	0.729055	0.599544	0.084644	0.310294	0.020069	0.64441

0.036347	0.602538	0.655337	0.788624	0.321512	0.408305	0.641506	0.075359	0.467111
0.036347	-0.31108	-0.23554	-0.20581	0.409299	0.072938	-0.09852	0.003749	0.720383
0.036347	0.398538	0.53134	1.233487	0.867346	1.426395	0.626901	0.010653	1.027857
-0.80063								
-0.11372	1.555387	1.07978	1.212237	0.765596	1.26139	0.795002	0.218655	0.789791
-0.11372	-0.14786	0.20428	0.266743	0.294656	0.266523	0.096613	0.033673	0.442514
-0.80063	1.49819	2.201843	2.002493	2.08015	1.80519	2.028463	0.08362	0.703653
-1.07254	0.175782	0.544298	0.965368	0.693037	0.487403	0.400721	0.00073	0.789587
-0.13415	-0.55367	-0.96396	-0.62081	-0.20919	-0.40776	-0.4196	0.000686	0.754766
-0.13415	-0.08375	0.196079	0.073165	0.369212	0.197665	0.100927	0.108575	0.452962
-0.13415	0.494596	0.719063	0.905175	1.077338	0.985402	0.777712	0.003382	0.582741
-0.57607								
-0.57607	-0.23915	-0.33034	-0.3049	-0.05901	-0.19554	-0.11044	0.022021	0.271332
-0.57607	0.851732	0.925437	1.07504	0.586258	0.630361	0.393498	0.010608	0.681542
0.535833								
0.535833	-1.29083	-1.45197	-1.41415	-1.2347	-1.38835	-1.31174	0.076509	0.21727
0.535833	-1.14026	-1.69635	-1.92593	-1.03048	-1.25304	-1.06634	0.001359	0.895455
0.535833	-1.07581	-1.25588	-1.14174	-0.90927	-1.11017	-0.89886	0.066617	0.357019
0.535833	1.3365	1.517697	1.73496	1.250367	1.614645	1.409023	0.00466	0.484593
-1.03095	-0.03917	0.220369	0.60902	0.290192	-0.14346	-0.06181	0.061431	0.752484
-1.03095	-1.13707	-1.32678	-1.48065	-1.07837	-1.63275	-1.12506	0.195798	0.55438
-1.03095	0.76383	1.060455	1.411067	0.890863	0.803429	0.722403	0.028549	0.688663
-0.81154								
-0.81154	-0.75263	-1.33781	-1.39851	-0.76609	-0.74268	-0.68421	0.009011	0.714303
-0.81154	-0.82218	-1.26194	-1.32887	-0.51043	-0.77898	-0.58422	0.01303	0.818435
-0.81154	-0.73664	-1.34223	-1.36543	-0.82666	-0.84523	-0.63787	0.017244	0.727564
-0.81154	-0.82075	-1.47722	-1.75173	-0.73811	-0.63326	-0.55522	0.085133	1.196509
-0.81154	-0.69531	-1.34402	-1.56545	-0.75593	-1.08747	-0.79878	0.000279	0.870137
-0.81154	-0.56801	-0.52741	-0.31833	-0.06818	-0.60689	-0.61367	0.007474	0.545491
0.97838	0.541102	0.600046	0.081825	0.181253	0.512664	0.185878	0.183324	0.518221
0.97838	-0.09922	-0.13218	0.088168	0.420566	0.695328	0.287711	0.018631	0.827512
0.97838	3.221927	2.15678	2.704363	2.605912	2.23619	1.553827	0.067962	1.6681
0.97838	-0.90814	-1.59722	-1.64568	-0.6652	-1.39532	-1.18861	0.059775	0.980484
0.97838	-0.79251	-1.01051	-0.8938	-0.70673	-0.61063	-0.5337	0.057654	0.476802
-0.87444								
-0.87444	0.531538	0.487251	0.836951	0.911599	1.065544	0.767102	0.012556	0.578293
-0.87444	0.487692	0.976141	1.430902	0.821371	1.023108	0.599148	8.12E-05	0.943209
-0.00515	-0.212	0.105194	0.585842	-0.22672	-0.01679	0.290042	0.03638	0.812564
-0.00515	-0.91453	-1.47473	-1.6097	-0.70276	-0.96203	-0.79225	0.004561	0.906941
-0.00515	1.539824	0.235602	0.629139	0.865868	1.082833	0.637522	0.089372	1.304222
-0.00515	-0.79143	-1.12197	-1.62493	-0.81162	-1.28457	-0.94988	0.007479	0.833494
-0.00515	-0.63696	-0.73594	-1.24421	-0.73366	0.174405	-0.78148	0.24713	1.418611
-0.00515	-0.72162	-1.07031	-1.04725	-0.77657	-1.12718	-0.83731	0.010533	0.405559
-0.00515	2.031673	2.156463	1.552223	2.04087	0.949218	0.855802	0.012145	1.300661
0.721913								
0.721913	-1.04106	-1.75113	-1.70592	-0.92625	-1.6047	-1.41332	0.048175	0.824875
0.721913	-0.79348	-1.06232	-0.6941	-0.3957	-0.58317	-0.67165	0.00254	0.666611

0.57281								
0.57281	-0.36807	-0.56064	-0.50396	-0.49963	-0.42405	-0.44141	0.06528	0.192564
0.57281	0.473997	0.414283	0.58865	0.438504	0.496069	0.524875	0.242341	0.174367
-1.12368	-0.42322	0.130174	0.781202	-0.10879	-0.42329	-0.45965	0.001297	1.240853
-0.57109								
-0.57109	-0.32054	-0.28457	-0.3606	-0.30168	-0.3555	-0.56554	0.154582	0.28097
-0.57109	-0.17589	0.160347	0.667508	0.159071	-0.00978	0.031208	0.006386	0.843397
-1.12368	1.323203	1.484733	1.831687	1.262273	1.475245	1.32426	0.000216	0.569413
-0.038								
-0.038	-0.51146	-0.50224	-0.51666	-0.38825	-0.77126	-0.57253	0.136523	0.383013
-0.038	-0.84667	-1.22257	-0.88375	-0.36769	-0.87612	-0.83754	0.030379	0.854875
-0.15691	0.339531	0.368201	0.654673	0.599443	0.751513	0.382234	0.079102	0.411981
-0.15691	-0.87865	-1.39468	-1.45239	-0.66214	-1.06616	-1.14696	0.053599	0.790254
-0.15691	0.012266	0.067	0.834196	1.20197	0.807657	0.281408	0.00538	1.189704
0.363553								
0.363553	-1.33653	-1.67786	-1.89965	-1.46201	-1.53043	-1.36241	0.006174	0.563117
0.363553	-1.28274	-1.86755	-2.02689	-1.30925	-1.92714	-1.47848	0.021325	0.744151
0.363553	-0.08053	0.061188	0.215483	0.31559	0.329919	0.106454	0.009319	0.410447
	0.392387	0.790616	0.837131	0.64111	0.888903	0.619293	0.001039	0.496515
-0.24607								
-0.55215	0.541935	1.29645	1.753713	1.092843	1.03567	0.66942	3.65E-06	1.211778
-0.55215	0.076789	0.039059	0.162333	0.336836	0.545919	0.428195	0.029909	0.506859
-0.55215	0.047681	0.10537	0.213147	0.627772	0.274649	0.207615	0.011553	0.580091
-0.19845	-1.10096	-1.5127	-1.71128	-1.13601	-1.46464	-1.18913	0.017848	0.610316
-0.24607	-0.39189	-0.69626	-1.44303	-0.34585	-1.27672	-0.99042	0.074289	1.097182
-0.24607	1.182877	1.57259	1.77236	1.079455	1.20876	1.063327	0.012227	0.709033
-0.19845								
0.792815	0.588864	0.481069	0.714796	0.378017	0.949304	0.821566	0.027068	0.571287
0.694098	-0.07529	0.203213	-0.67614	-0.18576	0.328812	-0.01223	0.018341	1.004955
0.694098	-0.87328	-1.39236	-1.59175	-1.10987	-1.44511	-1.16508	0.017056	0.71847
0.694098	-1.16292	-1.75028	-1.85799	-1.23348	-1.34152	-1.20508	0.012857	0.695073
0.694098	-0.87689	-0.77906	-0.6636	-0.70988	-0.56304	-0.51316	0.060914	0.363732
0.694098								
0.694098	-0.81601	-1.16889	-1.23108	-0.4333	-1.26148	-0.71035	0.09793	0.828176
0.694098	-0.83724	-1.38359	-1.65447	-0.94871	-1.1247	-0.99822	0.04659	0.817227
0.694098	-0.3059	-0.41283	-0.50343	-0.51797	-0.46461	-0.25262	0.221024	0.265351
0.694098	-0.03349	0.306298	-0.35344	-0.18049	-0.00666	-0.11947	0.09156	0.659737
0.694098								
0.694098	-0.33621	-0.28871	-0.35121	-0.41415	-0.68956	-0.27259	0.067455	0.416971
0.694098	0.289963	0.487356	0.499929	0.189578	0.879599	0.633569	0.001863	0.690021
0.792815	0.872914	1.175153	0.841138	0.636352	1.34941	1.246833	0.002339	0.713058
0.37511								
0.37511	0.493911	0.854976	0.796294	0.446507	0.545626	0.404135	0.008427	0.450841
-0.66159								
-0.66159	-0.01007	-0.12253	0.288071	0.158848	0.198336	0.159668	0.054716	0.410601
-0.66159	0.439923	1.508253	1.75509	1.50724	1.026153	0.711502	0.033463	1.315167

0.158142	0.137657	0.383544	0.780227	0.216754	0.325118	0.116516	0.021336	0.663711
0.158142	1.324467	1.90154	1.727983	1.085915	1.48269	1.425657	0.01395	0.815625
0.158142	-0.91138	-1.39001	-1.05993	-0.25175	-0.98566	-0.99806	0.015816	1.138267
0.158142	-0.02769	-0.03362	0.293934	0.455305	0.33999	-0.02822	0.038335	0.488924
-0.15066	-0.37931	-0.47318	-0.54561	0.234053	0.507829	0.170955	0.012266	1.053442
0.285153								
0.285153	0.630395	1.134238	1.57896	1.247983	0.285341	0.466933	0.004063	1.293619
0.285153	1.10423	1.719617	2.115563	1.44919	0.732491	0.732083	0.002428	1.38348
-0.26986								
-0.26986	0.753809	1.479287	1.789377	1.275667	1.742255	1.147706	0.007348	1.035568
0.430633	1.000557	1.277253	1.525583	1.257845	1.419425	1.268133	0.097266	0.525026
0.430633	0.456642	0.903853	1.553433	0.98466	0.9213	0.930169	0.000407	1.096792
0.430633	0.944898	0.955682	0.955852	0.557958	1.083495	1.008091	0.026707	0.525537
-0.69697	-0.26768	-0.43616	-0.23148	-0.36423	0.239358	0.12256	0.064698	0.675513
-0.88685	-0.10882	-0.12807	-0.09477	0.079957	0.085429	0.177445	0.094473	0.305517
-0.88685	-0.78415	-1.33253	-1.46548	-0.72688	-1.25588	-0.97478	0.006663	0.738596
-0.88685	-0.8565	-1.03259	-1.09695	-0.82962	-1.18155	-0.90116	0.056244	0.351933
-0.69697	1.498289	1.435926	1.036031	1.356079	0.288509	0.941206	0.094265	1.20978
-0.69697	0.399403	1.102795	1.783573	0.823275	-0.03996	-0.15071	9.93E-05	1.934282
-0.42337	-0.05275	0.106311	0.472745	0.459414	0.422776	0.221724	0.016567	0.5255
-0.42337	0.031657	0.113252	-0.47365	-0.46379	0.199677	0.050009	0.024034	0.673325
-0.42337	-0.73872	-1.09565	-1.29968	-0.63046	-0.78938	-0.65879	0.003898	0.669224
-0.22303								
-0.22303	-0.17555	-0.19048	0.076349	0.081078	-0.06204	-0.05458	0.035101	0.271561
-0.22303	-0.02591	-0.01317	0.209623	-0.19858	-0.62819	-0.25965	0.006044	0.837811
-0.08125								
-0.08125	-0.3498	0.781749	1.6069	0.352222	-0.31076	-0.47154	0.000743	2.078438
0.35867								
0.35867	0.464534	0.592868	0.580399	0.642045	0.899189	0.689944	0.011162	0.434654
0.35867	-0.78231	-1.27105	-1.23369	-0.88343	-1.07045	-0.90614	0.015736	0.488747
-0.73771								
-0.73771	0.205805	-0.04047	0.095735	0.104055	0.550469	0.562226	0.010102	0.602693
0.12466								
0.12466	-0.61928	-0.70559	-1.21186	-0.80706	-0.8239	-0.72871	0.00079	0.592587
0.12466	-0.0355	-0.27628	-0.56074	-0.48975	-0.35545	-0.62612	0.057195	0.59062
-0.30383	0.652776	0.806594	0.708852	0.449697	0.746038	0.67149	0.020448	0.356898
-0.30383	0.647204	1.016549	1.340833	0.809078	0.647408	0.504409	0.00626	0.836425
-0.3401	0.477269	0.663103	0.970487	0.70368	0.343766	0.209225	0.004938	0.761262
-0.29774								
-0.29774	0.12027	0.017611	0.198814	0.404329	0.52485	0.411334	0.014578	0.507239
-0.29774	-0.42667	-0.49526	-0.56867	-0.35099	-0.18617	-0.38732	0.13824	0.382499
-1.32872								
-1.32872	0.139971	-0.14126	0.113603	0.038583	-0.33861	-0.14938	0.16048	0.478582
-1.32872	1.321243	1.385133	1.602773	1.082123	1.473918	0.868066	0.010294	0.734707
0.821247								
0.821247	-1.15111	-1.6934	-1.7159	-1.10606	-1.54858	-1.16662	0.01458	0.609845
0.821247	-0.70943	-1.12855	-0.88528	-0.62497	-0.81148	-0.63147	0.005598	0.50358

-0.35205	-0.56289	-0.62711	-0.92746	-0.43009	-0.10376	-0.32747	0.003249	0.823704
-0.35205	-0.19895	-0.1671	-0.36948	-0.19983	-0.28833	-0.23036	0.118082	0.202389
-0.35205	0.005542	-0.50967	-0.78209	-0.40276	-0.42033	-0.30867	0.014372	0.787635
-0.59235								
-0.59235	0.543681	0.590747	0.789471	0.877519	0.463108	0.565285	0.022999	0.414411
-0.59235	-0.33085	-1.04101	-1.2582	-0.67284	-1.09941	-0.90363	0.060422	0.927355
-0.59235	-1.04274	-1.56007	-1.43891	-0.65945	-1.46512	-0.79	0.012261	0.90062
-0.59235	-0.94122	-1.40112	-1.63004	-1.21795	-1.61858	-1.19206	0.001221	0.688825
-0.59235	1.43979	-0.07721	-0.7268	-0.36109	-0.4854	-0.43696	0.031338	2.166593
0.411407								
0.411407	0.379684	0.122703	0.13563	0.637421	0.757112	0.71528	0.008946	0.634409
0.195837	-0.18115	0.013749	0.109179	0.023897	0.102364	-0.08812	0.017457	0.29033
0.63198	0.527413	0.854705	1.12534	0.718291	1.046054	0.798883	0.005639	0.597927
0.195837	-0.6598	-0.52597	-0.67341	-0.97328	-0.83281	-0.50818	0.025478	0.465103
-0.18932								
-0.18932	1.51087	2.560693	0.982366	1.629957	0.724892	0.57954	0.004738	1.981153
0.09985								
0.09985	0.19514	-0.2112	-0.71779	-0.27642	-0.43742	-0.28274	0.030119	0.912928
0.09985	0.418532	0.854691	1.009262	0.744702	0.310268	0.172131	0.014189	0.837131
-0.90197	1.338153	1.57871	1.858767	1.561997	1.026711	0.777417	0.000365	1.08135
-0.47896	1.43758	1.852983	2.20073	1.544473	1.76812	1.33703	0.002508	0.8637
-0.73365	0.546553	0.746412	0.764038	0.435663	0.994238	0.894651	0.066852	0.558574
-0.73365	0.203318	0.655587	1.01416	0.685009	0.629721	0.459982	0.001699	0.810842
-0.47896	-0.38308	-0.46521	-0.4465	-0.09897	-0.27072	-0.40173	0.036716	0.366235
-0.47896	0.762658	1.35451	1.61121	1.030144	0.98362	0.721993	0.010143	0.889217
-0.73406								
-0.73406	0.535145	0.600648	0.522715	0.741531	0.858782	0.67217	0.00716	0.336066
-0.03571	1.27934	1.57298	1.946987	0.995823	1.555785	1.537203	0.011191	0.951164
-0.03571	-0.15241	-0.11854	-0.04192	-0.30553	-0.02129	0.087006	0.03562	0.392538
-0.40421	0.949948	1.16097	1.16706	0.888004	1.06977	1.163427	0.038505	0.279056
-0.40421	-0.35648	-0.27624	-0.0218	-0.05608	-0.34336	-0.54811	0.0041	0.526311
-0.3208								
-0.3208	0.877503	0.950359	0.812935	0.702572	1.046447	1.15652	0.010654	0.453948
-0.3208	-0.61704	-1.45547	-1.94178	-1.03478	-1.09836	-0.91513	0.022224	1.324734
-0.3208	3.36778	4.020927	3.29934	3.50173	2.431685	3.137677	0.031913	1.589242
-0.3585	1.199577	1.432807	1.509	0.905289	1.61617	1.635393	0.001285	0.730104
-0.3585	-0.71955	-0.85291	-0.77493	-0.3652	-0.57959	-0.53808	0.001646	0.487715
-0.3585	0.434557	0.13525	0.603199	0.839046	0.373935	0.739202	0.232353	0.703797
-1.31597								
-0.9807								
-0.9807	-1.05912	-1.33829	-0.69834	-0.60896	-1.00888	-1.13271	0.013685	0.729325
-0.9807	0.640673	-0.16234	-0.82725	2.043919	-0.21853	-0.27916	0.074861	2.871171
-1.31597	0.898508	1.28295	1.68842	0.777236	1.40715	1.34761	0.008917	0.911184
0.444123	-1.80595	-2.08805	-2.51997	-1.90197	-2.50685	-1.81929	0.030023	0.714013
	-1.19707	-1.75391	-2.01067	-1.30002	-1.72972	-1.63529	0.032768	0.8136
	-0.80858	-0.96746	-1.46748	-0.64858	-0.86662	-0.59253	0.053713	0.874946
0.444123	-0.25203	-0.11074	-0.12015	-0.0286	0.184306	-0.01247	0.020436	0.43634

0.444123	-0.96714	-1.69066	-2.23114	-1.43074	-1.66475	-1.20024	0.004413	1.263999
0.096658								
0.096658	0.402313	-0.69152	-1.3156	-0.72219	-1.06836	-0.82877	0.080395	1.71791
0.096658	-0.15159	-0.31161	-0.64764	-0.47306	-0.80781	-0.61266	0.07421	0.65622
0.096658	0.298523	0.083889	0.201533	0.223923	0.106487	0.068257	0.09863	0.230266
0.21104								
0.21104								
0.21104	1.262337	1.679843	2.060087	1.537263	2.000355	1.682967	0.002992	0.79775
0.87847								
0.87847	0.064735	0.227327	0.403876	-0.07725	0.509763	0.461723	0.028592	0.587011
0.87847	-1.20361	-1.82979	-1.95135	-1.26462	-1.57714	-1.28196	0.009598	0.74774
0.87847	-0.77459	-1.64078	-1.86016	-1.28778	-1.51795	-1.34469	0.044195	1.085566
0.87847	-0.39153	-0.84515	-1.02813	-0.45359	-0.76129	-0.51337	0.012643	0.636601
0.87847	-0.40006	-0.98404	-1.27968	-0.87694	-1.21254	-0.9267	0.001325	0.87962
0.87847	-1.15173	-1.59042	-1.56741	-1.01278	-1.73853	-1.3399	0.04163	0.725745
-0.32568	0.572088	1.038003	0.737659	0.73285	0.810885	0.800802	0.126602	0.465915
-0.37249								
-0.32568								
-0.37249	0.111252	0.482959	0.526514	0.066014	-0.06958	-0.09527	0.026482	0.621779
-1.01263	2.54401	2.466347	2.248103	1.931617	2.293725	1.801237	0.104611	0.742773
1.19889	0.639654	0.446463	0.635691	0.265398	0.272572	0.249798	0.04513	0.389856
1.19889	-0.45076	-0.60816	-0.29168	-0.37517	-0.50492	-0.40215	0.014053	0.316481
1.470333	0.731418	0.81094	0.941603	0.480501	0.942024	0.710568	0.029852	0.461523
1.470333	-0.79643	-1.49071	-1.711	-1.01988	-1.34019	-1.17525	0.004272	0.91457
1.470333	-0.46233	-0.59266	-0.81683	-0.45694	-0.56991	-0.53007	0.07868	0.359882
0.02117	0.502996	1.024028	1.241437	0.725017	0.814326	0.68771	0.023999	0.738441
0.02117	-0.76348	-0.88554	-0.82698	-0.41166	-0.81352	-0.58206	0.010678	0.473875
0.02117	1.441013	1.589427	1.988367	1.551227	1.682465	1.44076	0.217731	0.547607
0.786313								
0.786313	-0.00347	-0.47173	-1.23574	-0.74159	-0.85526	-0.79458	0.008872	1.232267
0.786313	-1.0245	-1.42225	-1.54164	-0.85983	-1.06823	-0.8713	0.078067	0.681815
0.346037								
0.346037	-0.27149	-0.47273	-0.70202	-0.77649	-0.4376	-0.22943	0.007984	0.547056
0.346037	0.683763	1.175574	1.254258	0.806304	1.002513	0.750737	0.002443	0.570495
1.216507	0.668415	0.877429	1.035034	0.364498	0.948719	0.82852	0.008257	0.670536
1.216507	0.337784	0.455291	1.106793	0.974128	1.036007	0.69872	0.000161	0.769009
1.216507	-0.12683	-0.19118	-0.43164	0.053414	0.211458	0.328601	0.008829	0.760243
-0.60024	-0.94433	-1.19133	-1.33764	-0.96089	-1.11746	-0.97034	0.024494	0.39331
-0.60024	0.214879	-0.03397	0.215721	0.285569	0.546448	0.118982	0.022834	0.580415
-0.60024								
	1.205441	0.986184	0.468041	0.49226	1.710672	1.188319	0.181524	1.242631
0.125043								
0.125043	0.993549	1.466247	1.75435	1.042561	1.285565	1.314973	0.009505	0.760801
0.456167	0.750119	1.081424	2.05571	0.727591	0.390344	0.172155	0.011315	1.883555
0.456167	1.061599	1.520823	1.337376	0.522954	0.935021	1.255184	0.133818	0.997869
0.456167	0.088077	0.324879	0.179957	-0.12688	0.00792	0.034283	0.017099	0.451755

-0.11226									
-0.11226	-0.73046	-0.82142	-1.21712	-0.80948	-1.07578	-0.54442	0.0718	0.672704
-0.11226	0.058019	0.162006	0.199466	0.183681	-0.14051	-0.0209	0.03772	0.339978
-0.34855	-0.1246	0.382846	0.874084	0.361509	0.512918	0.146704	0.010971	0.998688
-0.34855	-1.53451	-1.80759	-1.88032	-1.52009	-1.68508	-1.35422	0.06174	0.526101
-0.34855	0.475645	0.556702	-0.06179	-0.11018	-0.03325	-0.25039	0.100087	0.807089
-0.28787								
-0.28787	-1.27041	-1.87802	-1.66055	-1.28876	-1.50724	-1.47542	0.085728	0.607612
-0.28787	-0.85424	-1.25305	-1.61157	-1.19018	-1.30309	-1.0702	0.001839	0.757329
-0.28787	-0.76218	-1.17965	-1.18744	-0.86558	-1.10369	-0.83295	0.0883	0.425254
1.110545	0.230211	0.381659	0.711927	0.360541	0.810957	0.657145	0.010823	0.580747
1.110545	-0.84673	-1.0864	-1.18779	-1.05257	-1.06348	-0.86521	0.114017	0.341059
1.110545	2.03549	1.645627	1.207693	1.516011	0.710153	1.433603	0.037214	1.325338
1.110545	-0.97882	-1.7465	-1.93168	-1.10237	-1.4782	-1.18516	0.003533	0.952862
-0.33114	0.058136	0.323697	1.086184	0.595639	0.221553	0.059254	0.003273	1.028048
-0.94201	1.588883	1.57054	1.262417	0.694189	1.33342	1.492433	0.001287	0.894694
-0.94201	0.442108	1.064764	1.487963	1.062273	0.407678	0.301281	0.003377	1.186682
-0.94201								
-0.33114	-0.48731	-0.69205	-0.28614	-0.28239	-0.53543	-0.49496	0.00049	0.409665
-0.33114	1.020223	1.364293	1.877547	1.340573	1.331	1.06413	0.00272	0.857323
-0.48758	0.678518	0.789442	0.600264	0.894634	0.83576	0.413529	0.255774	0.481105
-0.48758	-0.19738	-0.24565	-0.24821	0.171824	-0.19449	-0.40423	0.014226	0.576059
-0.55711	-0.01754	-0.24047	0.217468	0.371824	0.323159	0.149187	0.040141	0.612295
-0.48758	-0.16324	-0.21396	-0.37268	0.140638	-0.19691	-0.31872	0.004805	0.513315
-0.55711	-0.9655	-1.57152	-1.5741	-1.1508	-1.70155	-1.19368	0.105686	0.736047
-0.55711	-0.15177	-0.37054	-0.36001	0.037463	0.213226	-0.17713	0.001817	0.58377
-0.47275								
-0.47275	-0.72326	-1.10719	-1.12631	-0.55831	-0.6441	-0.58434	0.034751	0.568005
-0.47275	-0.80181	-1.14358	-0.98241	-0.74393	-0.9838	-0.89935	0.031594	0.399651
-1.39499								
-1.39499	-0.33404	-0.56695	-0.37202	-0.02856	-0.66443	-0.64813	0.02204	0.635869
-1.39499	1.38472	1.833053	2.30132	1.56172	1.064275	0.875031	0.002232	1.426289
0.561328								
0.561328	-0.07439	-0.11521	0.445279	0.358669	-0.19048	-0.19818	0.010408	0.643454
0.561328	0.634425	0.20698	0.089475	0.18765	-0.61446	-0.09549	0.081347	1.24888
0.561328	-0.29853	-0.93355	-0.94643	-0.67959	-0.85969	-0.79565	0.124598	0.647905
0.318813	0.776295	1.149702	1.316939	0.660847	1.051888	1.054377	0.014056	0.656091
0.318813	0.189828	0.058448	0.579521	0.686818	0.417081	0.141466	0.012929	0.62837
-1.06199								
-1.06199	-0.49841	-0.4908	-0.50875	-0.15865	-0.07986	-0.25679	0.008712	0.428885
-1.06199	0.389258	0.401945	0.813583	1.073003	1.093291	0.535491	0.019351	0.704033
-0.95049	0.956454	1.447973	1.553477	1.23709	1.49773	1.132734	0.005166	0.597022
0.886417	-0.584	-0.49925	0.002054	-0.33551	-0.69254	-0.61103	0.044468	0.694594
0.886417	-0.5608	-1.00725	-1.27893	-0.6771	-1.15956	-0.88907	0.009822	0.718123
1.1398								
0.886417	-1.25006	-1.71524	-2.13956	-1.37873	-1.87669	-1.71101	0.003608	0.889497
1.1398	1.577144	1.606959	1.511851	1.028487	0.216657	0.87688	0.132613	1.390302

0.358917								
0.358917	1.391906	1.10218	1.422181	1.555004	-0.39167	0.546308	0.07976	1.946678
0.358917	1.454913	1.520103	1.567497	1.33261	1.653555	1.804193	0.204118	0.471583
-0.29856								
-0.29856	-0.4881	-0.80939	-0.87278	-0.59633	-1.02722	-0.71761	0.018011	0.539122
-0.29856	-1.05521	-1.60116	-1.7816	-1.16868	-1.45118	-1.01584	0.01116	0.765766
2.85274								
2.85274	-0.72372	-1.14082	-1.29464	-0.64142	-0.94955	-0.79393	0.002559	0.653216
2.85274	-1.28369	-1.72217	-2.02275	-1.21972	-1.76315	-1.30246	0.015226	0.803027
2.85274	-0.88723	-1.5591	-1.81424	-0.9199	-1.51627	-1.1612	0.014525	0.927009
2.85274	-0.68189	-0.79079	-0.75768	-0.45471	-0.75576	-0.64939	0.128214	0.336079
-0.45543	0.791855	1.371487	1.484937	1.020095	1.258705	0.894979	0.040992	0.693082
-0.65639	-0.11141	0.361808	0.652413	0.60356	0.419849	0.197364	0.009085	0.763827
-0.65639	0.652626	1.274087	1.570773	0.680776	0.910119	0.612748	0.011082	0.958026
-0.45543	-0.53595	-0.58993	-0.54861	-0.3306	-0.49794	-0.58651	0.032274	0.259328
-0.45543	0.915757	1.244752	1.513753	0.976384	0.626066	0.513909	0.031755	0.999844
0.06629	0.784968	1.043015	1.535353	0.827793	0.904482	0.859372	0.011195	0.750386
0.06629	-0.48051	-0.44729	-0.41909	-0.19688	-0.26063	-0.37255	0.040425	0.283633
-0.64596	0.504403	0.704852	1.503653	0.834217	0.837962	0.592012	0.018961	0.99925
-0.64596	0.124402	-0.4561	-0.87106	-0.27578	-0.73346	-0.63247	0.096036	0.995464
-0.64596	-0.57546	-0.72627	-0.60239	-0.40272	-0.57197	-0.53618	0.005001	0.323551
0.867797								
0.867797	-0.25037	-0.40461	-0.81291	-0.36889	-0.22824	-0.57031	0.123757	0.584666
0.088935	6.251287	5.474158	4.168043	4.871357	5.92734	4.617483	0.038474	2.083243
0.088935	-1.09089	-1.70363	-1.71084	-1.19791	-1.38024	-1.35765	0.004557	0.619953
0.088935	-1.14596	-1.56152	-1.79377	-1.24963	-1.5327	-1.35592	0.015198	0.64781
0.088935	-1.09611	-1.54003	-1.67856	-1.20243	-1.51531	-1.18362	0.004112	0.58245
0.088935	-0.64399	-1.14851	-1.4402	-1.02075	-1.32147	-1.07436	0.000133	0.796212
0.088935	-0.367	-0.58347	-0.89666	-0.60568	-0.68716	-0.24363	0.151546	0.653029
-0.11244	-0.53172	-0.3267	-0.28107	-0.3927	-0.29554	-0.32477	0.058834	0.250647
-0.30579	0.406582	0.778862	1.515427	1.070006	0.805133	0.882232	0.007531	1.108845
-0.30579	-0.31196	-0.29707	-0.30885	-0.0026	-0.26058	-0.2478	0.107999	0.309355
-0.11244	0.225053	-0.15795	-0.61977	-0.47079	-0.30397	-0.56425	0.011766	0.844827
-0.60748	0.712706	0.811213	0.02443	0.284322	0.491015	0.227846	0.04089	0.786783
-0.60748	0.872441	1.322503	1.58173	0.884209	1.48151	1.12475	0.00167	0.709289
-0.60748	-0.59248	-0.70577	-0.58619	-0.39516	-0.26666	-0.50929	0.044176	0.439111
1.084677	-0.42988	-0.39694	-0.25778	-0.27087	-0.33699	-0.5262	0.049353	0.268419
1.084677	0.556629	0.661889	0.420873	0.449665	1.13038	0.627543	0.04188	0.709507
1.151537	0.41577	0.609574	0.645436	0.658635	0.985318	0.714969	0.009197	0.569548
1.084677	0.430434	0.516793	0.539534	0.152631	0.838436	0.635638	0.01392	0.685805
1.151537	-0.31451	-0.19144	0.163648	0.17037	0.174204	-0.31879	0.007886	0.492993
1.151537	-0.63922	-0.96496	-1.35141	-0.75979	-1.00809	-0.85288	0.035787	0.71219
-0.40138								
-0.40138	-1.07496	-1.42332	-1.37001	-0.86925	-1.33899	-1.1682	0.017963	0.554075
-0.40138	-1.43962	-2.19827	-2.30299	-1.61135	-2.40071	-1.86695	0.007563	0.961082
-0.40138	-0.12903	-0.09699	-0.19044	-0.61761	-0.1599	-0.0571	0.003792	0.560515
0.025248	-1.21974	-1.99707	-1.87648	-1.49578	-2.36918	-1.7468	0.000664	1.149436

0.025248	-0.93373	-1.44732	-1.54696	-0.90821	-1.32653	-1.04784	0.001097	0.638757
0.025248	-0.63739	-0.92422	-1.37082	-0.7804	-1.28928	-1.05739	0.002261	0.733436
-0.80666	2.090973	1.773523	1.479798	1.452917	1.804495	1.3072	0.011386	0.783773
-1.12031								
-1.12031	-0.8267	-1.04113	-1.24688	-0.83624	-1.23351	-0.91055	0.070277	0.420174
-1.12031	-1.01361	-1.44215	-1.48857	-1.03949	-1.35698	-1.10104	0.108744	0.474968
-1.12031	0.714959	1.331017	1.705007	1.205287	0.837287	0.650441	0.005814	1.054566
-0.80666	-0.07346	0.015815	-0.4787	-0.02491	-0.27612	-0.09668	0.198886	0.494513
-0.16022	1.386029	1.073971	1.241981	1.009489	1.384908	0.955244	0.246689	0.430785
-0.16022	0.504166	0.725518	1.047084	1.097638	0.762572	0.608391	0.049024	0.593472
-0.16022	2.807437	2.51791	1.99835	2.046027	1.97347	2.01498	0.076123	0.833967
-0.18104								
-0.18104	-0.03176	0.251293	0.406854	0.051562	-0.1514	0.145315	0.0849	0.558255
-0.18104	0.169494	0.497932	0.703526	0.37755	-0.25193	-0.23067	0.001739	0.955455
-0.07272	-0.10783	-0.19357	-0.35638	-0.10856	0.054709	-0.03399	0.000147	0.411085
-1.07485								
-1.07485	-0.44463	-0.34117	-0.7591	-0.22162	-0.12074	-0.19961	0.00226	0.638357
-1.07485	-0.05606	0.261523	0.248786	0.408764	0.419807	0.252137	0.147826	0.475869
-0.07272	0.869527	0.72188	1.111812	1.066882	1.28181	0.984737	0.00441	0.55993
-0.45165	0.505887	0.52446	0.824623	0.319005	0.249786	0.384526	0.005072	0.574837
-0.45165	-0.73472	-0.71792	-0.2663	-0.21692	-0.52989	-0.787	0.001374	0.570081
-0.45165	-0.19551	-0.18444	-0.33663	-0.12602	-1.17246	0.017418	0.050904	1.189879
-0.45165	0.903868	1.661143	1.64794	0.702038	0.927249	0.721796	0.000427	0.959105
-0.33834								
-0.33834	-0.07532	0.076746	0.028506	-0.21944	0.150724	0.15183	0.023902	0.371268
-0.33834	-0.39004	-0.01065	-0.06941	0.10238	-0.03929	-0.11884	0.002401	0.49242
-0.72794								
-0.72794	-0.16083	-0.00915	0.159761	-0.11089	-0.44381	-0.22127	0.0652	0.603566
-0.72794	0.200954	0.442721	0.995187	0.548879	0.258898	0.471007	0.026549	0.794233
-0.06598								
-0.06598	0.997921	1.3825	1.338827	0.805478	0.895464	0.923277	0.003945	0.577022
-0.69975	0.158014	0.62314	0.761999	0.729635	0.559913	0.334579	0.034786	0.603985
-0.79247								
-0.69975								
-0.79247	2.67891	2.0998	1.515488	1.842413	1.52351	1.66137	0.022111	1.163422
-0.79247	0.691254	1.027021	1.418163	0.939121	0.830327	0.66281	0.000175	0.755354
	-0.46413	-0.21542	-0.62827	-0.80187	-0.3858	-0.30317	0.014983	0.58645
-0.56061								
-0.56061	-0.21698	-0.30936	-0.56352	-0.45497	-0.40421	-0.34576	0.030777	0.346542
0.25829	-0.70419	-1.14705	-1.33277	-0.80907	-1.53168	-0.91132	0.11578	0.82748
0.25829	0.294128	-0.05693	-0.38069	-0.28767	-0.12299	-0.13662	0.058101	0.674821
0.233637	0.743867	0.952303	1.288673	0.371817	1.35712	1.32286	0.043879	0.985303
0.033977	0.358109	0.835575	0.975229	0.520127	0.44781	0.419583	0.017209	0.617119
0.233637	1.23986	1.83492	2.03075	1.38575	1.422745	1.13329	0.00386	0.89746
-0.05563	-0.4625	-0.81829	-0.59958	-0.39029	-0.52476	-0.57131	0.037431	0.428003
-0.05563								

3.354547								
3.354547	-0.9014	-1.38216	-1.47527	-0.91349	-1.39327	-1.01027	0.008367	0.573867
3.354547	0.802351	0.482856	0.298519	0.20458	-0.0795	0.682354	0.046977	0.88185
-0.44261								
-0.44261	0.164327	0.314737	0.485604	0.414102	0.348148	0.337991	0.027757	0.321277
-0.27611	1.257929	1.056799	1.417869	1.054536	1.039377	0.827821	0.008668	0.590048
-0.27611	-0.45844	-1.13197	-1.36845	-0.8918	-1.17423	-1.15385	0.050091	0.910016
-0.27611	0.281476	0.829972	1.318947	1.040583	0.518442	0.310062	0.002277	1.037471
1.554115	1.419417	1.956657	1.677777	1.026374	1.304035	1.213729	0.01375	0.930282
1.554115	0.577148	0.467824	-0.31681	0.263327	0.125825	-0.02773	0.017683	0.893963
1.554115	-0.73207	-1.50821	-1.5967	-0.98213	-1.30369	-1.059	0.005931	0.864631
1.554115	0.044201	-0.27493	-0.37311	-0.17581	0.127266	0.006877	0.007789	0.500375
0.615127	0.584463	0.81863	1.286063	0.824316	1.178125	0.724875	0.072976	0.7016
0.615127	-0.48618	-0.4374	-0.24376	-0.35659	-0.18652	-0.16507	0.019277	0.321109
0.615127	0.378437	0.672295	0.868487	0.917412	1.241365	0.967863	0.045119	0.862928
-0.24349	-0.37222	-0.80664	-0.86104	-0.3483	-0.72744	-0.63672	0.039359	0.512746
-0.24349	-0.97731	-1.72314	-1.72987	-1.06116	-1.50807	-1.18114	0.001871	0.752553
-0.24349	-0.25583	-0.54592	-1.06517	-0.26954	-1.20041	-0.7042	0.080401	0.94458
0.715								
0.715	-0.94725	-1.33493	-1.46899	-0.85512	-0.97002	-0.87665	0.003446	0.613862
0.715	-0.27116	-1.20536	-1.69486	-0.96202	-1.1335	-1.04299	0.017032	1.423699
0.715	-0.37088	-0.63974	-0.35497	-0.26127	-0.16723	-0.24045	0.007526	0.472508
-0.1792	-0.73755	-0.8991	-0.49734	0.120842	-0.36865	-0.63417	0.010434	1.019943
-0.5584	-0.80591	-0.92577	-1.08613	-0.78157	-0.86782	-0.87082	0.023805	0.304565
-0.5584	-0.85179	-0.98573	-0.38376	0.162739	-0.0813	-0.41305	0.004808	1.148464
-0.5584	-0.29776	-0.16633	-0.31787	0.374054	0.515122	0.104814	0.046763	0.832989
-0.1792	0.025676	0.524588	-0.73685	-0.5721	0.762232	-0.23944	0.078568	1.499084
-0.1792	-0.80261	-1.28732	-1.22292	-0.74811	-0.82478	-0.92242	0.049541	0.539209
-1.10742								
-1.10742	-0.41421	-0.37186	-0.28209	-0.59893	-0.57057	-0.39913	0.016057	0.316842
-1.10742	0.271049	0.439009	0.893076	0.642083	0.400044	-0.05437	0.030967	0.947448
-0.26531	0.541979	0.69105	0.695134	0.64976	0.807008	0.668139	0.16542	0.265029
-0.26531	1.166083	1.37993	1.469361	1.070367	0.797902	0.969825	0.084941	0.67146
-0.26531	-1.25193	-1.50773	-1.51786	-1.17954	-1.20715	-1.09453	0.031034	0.423327
0.345217	0.334579	0.452574	0.634563	0.500418	0.808944	0.652969	0.03981	0.474365
0.345217	-1.27444	-1.67308	-1.80591	-1.1778	-1.74373	-1.35664	0.024195	0.628109
0.345217	0.16902	0.407946	-0.84762	-0.64881	-1.14471	-0.67461	0.044448	1.552651
0.345217	0.197416	-0.26348	-1.00313	-0.59336	-0.955	-0.69508	0.00403	1.200547
0.345217	1.925358	1.346811	1.189601	1.307537	0.793572	1.102045	0.158237	1.131787
-0.18292								
-0.18292	1.15328	1.210122	1.198707	1.214343	1.30671	1.175227	0.176063	0.15343
-0.39902								
-0.39902	0.142861	-0.30507	-0.53722	-0.31078	-0.27308	-0.52006	0.08229	0.680085
-0.39902	0.039356	-0.18856	-0.69363	-0.32314	-0.30149	-0.61799	0.026652	0.732983
0.06								
0.06	-0.19264	-0.17969	-0.01869	0.004722	-0.30777	-0.31129	0.038158	0.316008
0.06	1.806923	1.937267	2.169217	1.618097	1.58279	1.492717	0.000251	0.6765

-0.48465									
-0.48465	0.744937	1.167287	1.52764	0.894694	0.887259	0.691278	0.0012	0.836362	
-0.27557									
-0.27557	1.077354	1.459052	1.194146	1.276361	0.536022	0.953178	0.156179	0.92303	
-0.27557	0.632204	1.07281	1.33705	0.646772	0.633564	0.564162	0.004275	0.772888	
-0.22543									
-0.22543	-0.53431	-0.29585	-0.17216	-0.42845	-0.36619	-0.38749	0.119258	0.362149	
-0.22543	-0.73936	-1.08721	-0.81354	-0.7477	-1.05001	-0.78002	0.020545	0.34785	
-0.4814	0.706889	0.70065	0.814711	0.480698	0.695192	0.812863	0.014886	0.334013	
-0.4814	2.069853	2.799847	2.41885	2.019533	2.00715	1.881177	0.080233	0.91867	
-0.69751	-0.68568	-0.44796	-0.0098	-0.69337	-0.48821	-0.37169	0.070396	0.683564	
-0.69751	-0.60443	-0.60471	-0.65679	-0.34196	-0.35418	-0.47357	0.054568	0.314831	
-0.69751	-0.61325	-0.50201	-0.52274	-0.49026	-0.76204	-0.64968	1.67E-05	0.271779	
0.034328	0.243487	0.452598	0.950421	0.32472	0.35354	0.55949	0.014257	0.706934	
0.034328	-0.18448	-0.40169	-0.22724	-0.13226	-0.01599	-0.14801	0.044443	0.385699	
0.034328	-0.81337	-1.31446	-1.57305	-0.88982	-1.08218	-0.89668	0.030915	0.759684	
0.034328	-1.24704	-1.5823	-1.64826	-1.19148	-1.22649	-1.02054	0.020617	0.627716	
0.034328	-0.87433	-0.9238	-0.83975	-0.66154	-0.95584	-0.8649	0.120506	0.294308	
0.034328	0.140734	0.441244	0.926357	0.607424	0.897853	0.472113	0.000338	0.785623	
-0.36672									
0.838573									
0.838573	-0.10336	0.027437	0.120578	0.117559	-0.17328	-0.10953	0.01963	0.293855	
0.838573	1.402743	1.817653	1.885183	1.311177	1.620855	1.37115	0.008968	0.574007	
-0.36672	-0.37473	0.090211	0.304757	-0.25316	-0.30976	-0.39343	0.065314	0.698185	
-0.36672	0.362666	0.636193	1.153897	0.830525	0.718886	0.766836	0.00325	0.791231	
-0.4386									
-0.4386	0.318289	0.632512	0.902014	0.477767	0.632926	0.353277	0.013246	0.583725	
-0.4386	-0.72717	-1.16051	-1.34299	-0.88794	-0.99547	-0.93747	0.0653	0.615819	
-0.4386	1.143569	1.162784	1.04627	0.53676	1.097	1.059729	0.044304	0.626024	
0.214058	0.98129	1.561963	1.659077	1.023518	1.464655	1.33513	0.010106	0.677787	
0.34284	0.916472	1.501757	1.712767	0.831861	1.303605	1.322497	0.000739	0.880905	
0.34284	-0.42496	-0.8273	-0.92186	-0.5325	-0.16244	-0.23853	0.012177	0.759413	
0.34284	-0.28035	-0.14499	-0.16085	-0.27362	-0.0159	-0.14307	0.082913	0.264457	
0.214058	-0.77359	-0.74487	-0.71427	-0.76176	-0.65579	-0.80062	0.1916	0.144829	
0.214058	-1.04805	-1.4602	-1.8077	-1.11224	-1.35192	-1.08666	0.00145	0.75965	
0.214058	0.331662	-0.98526	-0.99332	-0.88304	-1.19765	-1.0446	0.060509	1.529314	
0.214058	-0.33683	0.577395	-0.46054	-0.21645	-1.01873	-0.29001	0.066552	1.596129	
0.214058	0.112871	-0.78577	-1.06756	-1.08703	-1.91459	-1.10666	0.114626	2.027456	
0.214058	-0.91752	-1.29539	-1.22321	-0.93908	-1.16871	-0.9641	0.000715	0.377871	
-0.06556									
-0.06556	0.340628	0.877694	1.064805	0.152077	0.195805	0.433269	0.00373	0.912728	
-0.06556	0.394836	0.574247	1.05909	0.566358	0.600514	0.478213	0.000776	0.664253	
0.03172									
0.03172	1.287135	1.174787	1.34701	1.115924	1.41925	0.636512	0.068076	0.782739	
-0.15549	0.253732	0.465365	0.437407	0.095872	0.049855	0.38005	0.194533	0.415511	
1.430827	0.019408	-0.02877	0.202412	0.281609	0.386884	0.243557	0.054084	0.415654	
1.430827	-1.11246	-1.54738	-1.79403	-1.04662	-1.34983	-1.22671	0.001286	0.747416	

1.430827	-0.10145	-1.20084	-1.63963	-0.9964	-1.21623	-0.85591	0.05336	1.538183
0.628347	3.992853	3.602775	2.85818	3.379796	2.91879	2.521974	0.034634	1.470879
-1.32956	-0.07351	0.090223	-0.05771	-0.0862	-0.34733	-0.25815	0.021151	0.437554
-1.32956	3.33253	2.556147	2.25615	1.97824	2.55783	2.487533	0.116173	1.35429
0.628347	-0.90421	-1.03617	-0.7738	-0.74478	-1.20917	-0.91634	0.058468	0.464389
0.628347	-0.45334	-0.9235	-1.04446	-0.5668	-0.77395	-0.61844	0.018078	0.591114
0.628347	0.43002	0.32443	0.507458	0.407139	1.062656	0.690544	0.009173	0.738226
-0.30003								
-0.30003	-0.77655	-1.32338	-1.66493	-0.76771	-0.82798	-1.14562	0.013411	0.897226
-0.30003	0.671682	0.903641	1.045884	0.641041	0.92662	0.740664	0.056639	0.404843
0.752128								
0.752128	-0.43021	-0.9158	-1.0184	-0.79563	-0.81313	-0.70222	0.029553	0.588191
-0.63132	0.396183	0.995606	1.567897	0.719064	1.065295	0.458031	0.02713	1.171713
-0.80638	0.516456	1.171257	1.206866	0.586381	0.941436	0.754665	0.010652	0.69041
-0.80638	-0.66156	-0.9923	-0.92263	-0.58799	-1.00319	-0.62019	0.179355	0.415201
-0.80638	0.34513	0.9408	1.326461	0.549112	0.877693	0.052381	0.007558	1.274081
-0.63132	1.876967	1.64152	1.386094	1.333622	1.473795	1.369017	0.165325	0.543345
-0.75331	-1.03905	-1.37539	-1.25349	-0.65436	-0.97696	-0.98841	0.022541	0.721034
-0.05868								
-0.05868	-0.70417	-0.61854	-1.0245	-0.71402	-0.51743	-0.76748	0.101363	0.507078
-0.05868	0.751168	1.22824	1.627323	1.184777	1.136825	0.93888	0.006917	0.876156
-0.75331	-0.61355	-0.58754	-0.94834	-0.87305	-1.91863	-0.78964	0.006901	1.331086
-0.75331	-0.8975	-1.24243	-0.97827	0.022148	-0.57362	-0.73486	0.000521	1.264578
0.218373								
0.218373	-0.90397	-1.2953	-1.20924	-0.95495	-1.07708	-0.93907	0.051404	0.391327
0.218373	0.797471	0.527515	0.688886	0.60962	0.301486	0.145976	0.069822	0.651495
0.344873	0.867107	0.736118	0.741019	0.274195	0.924998	0.965217	0.004793	0.691022
0.344873	-0.25973	-0.31286	-0.47914	-0.65944	-0.19772	-0.25732	0.028463	0.461713
-0.26035	-0.26342	0.1647	0.459496	-0.20638	0.081727	-0.16184	0.000287	0.72292
-0.26035	-0.68999	-0.10404	-0.1703	-0.76731	-0.51743	-0.42281	0.086865	0.663272
-0.69018	0.514524	0.727132	1.062883	0.562099	0.75762	0.75306	0.021086	0.548359
-0.69018	-0.47824	-0.32622	0.103251	-0.20062	-0.69851	-0.62057	0.019283	0.801765
-0.69018	-0.3583	-0.10894	0.157158	0.549508	0.075682	-0.04743	0.004424	0.907804
-0.09585								
-0.09585	0.14304	-0.0908	-0.4472	-0.1436	-0.2805	-0.28174	0.01156	0.590241
-0.09585	-1.00051	-1.37494	-1.33056	-0.60056	-1.18466	-1.04786	0.048795	0.774381
-0.28937	0.143002	0.394687	0.760422	0.451096	0.655788	0.448456	0.029173	0.617419
-0.28937	-0.80283	-0.88873	-0.99511	-0.75151	-0.86087	-0.75144	0.061985	0.243671
-0.20313	0.142258	0.276128	0.399907	0.210858	0.58111	0.358302	0.064906	0.438852
-0.20313	1.117963	1.61095	1.77699	1.062697	1.225705	1.104251	0.002873	0.714293
1.915377	0.194395	0.354673	0.278888	0.151097	0.318679	0.261248	0.049524	0.203577
0.427977	1.340557	1.66682	1.564567	0.515306	0.735648	1.226182	0.000371	1.151514
1.915377	1.520283	1.88962	1.720527	0.721765	0.890108	1.353993	0.004719	1.167855
0.427977	0.562959	0.678952	0.890507	0.234756	0.216804	0.128507	0.013328	0.762
0.47								
0.47	0.443717	0.798926	1.333883	0.853517	0.494906	0.397652	0.004852	0.936231
-0.46736	0.280101	0.420916	0.36263	0.277157	0.406076	0.401199	0.061919	0.143759

-0.46736	0.792139	0.613457	0.517389	0.952183	1.219675	1.000307	0.040006	0.702286	
-0.92454									
-0.92454	0.70322	0.920664	0.714737	0.2629	1.68783	0.788286	0.031588	1.42493	
0.627727	0.300791	0.382459	0.742431	0.786463	1.120202	0.781348	0.023864	0.819411	
0.627727	-1.00157	-1.49932	-1.67066	-1.11964	-1.40594	-1.08541	0.009748	0.669091	
0.627727	-0.91765	-1.43857	-1.49631	-0.84267	-1.16303	-0.94389	0.002	0.653634	
0.627727	-0.31058	-0.82491	-0.76038	-0.24229	-0.51641	0.081658	0.03181	0.906564	
1.941773									
1.941773	1.84083	1.796297	1.445633	1.481333	1.896355	1.682817	0.052483	0.450722	
-1.52563	0.003403	-0.36935	-0.51022	-0.14545	0.519441	0.310616	0.009029	1.029656	
-1.52563	1.065205	1.6991	2.014643	1.27268	1.57232	1.051074	0.001794	0.96357	
0.8059	1.144846	1.586553	1.60371	1.419158	2.127935	1.740383	0.019217	0.983089	
0.2163	0.08091	0.630443	0.856298	0.161794	-0.06677	0.043308	0.002176	0.923071	
0.2163	1.027626	0.977348	0.877162	0.990765	1.376145	1.387677	0.018067	0.510515	
-0.27604	1.188719	1.763777	1.742557	1.091969	1.719405	1.764507	0.038929	0.672538	
0.2163	0.647064	1.118278	0.818263	0.470292	1.41565	1.199893	0.009202	0.945358	
0.8059	0.475532	0.773552	0.484592	0.52858	0.529995	0.570455	0.032724	0.29802	
-0.27604	0.723554	1.164973	1.337223	0.635432	1.513215	1.253423	0.005946	0.877783	
-0.27604	0.168407	0.592408	0.911946	0.15891	0.76996	0.587694	0.005822	0.753036	
1.013193	0.647524	0.669491	0.647505	0.24739	0.642582	0.854103	0.001997	0.606713	
1.013193	-0.95387	-1.35912	-1.16581	-0.96081	-0.94271	-0.91145	0.121235	0.447678	
1.013193	0.015754	-0.15665	0.165067	0.389176	0.614194	0.477032	0.017105	0.770839	
-0.83448	0.32048	0.416575	0.928187	0.274498	0.598158	0.528907	0.003066	0.653689	
-0.83448	1.292047	1.812773	1.954083	1.046644	1.37427	1.075683	0.00303	0.90744	
-0.58835									
-0.58835	1.867353	2.261747	2.112793	1.716983	1.981975	2.012853	0.086745	0.544763	
2.38118									
2.38118	0.818978	0.930307	0.526978	0.117944	0.958508	0.96813	0.013566	0.850185	
-0.5719	0.168101	0.67726	0.988516	0.345721	0.766413	0.328759	0.031334	0.820415	
-0.5719	-0.61234	-0.95391	-1.10871	-0.60918	-0.85827	-0.774	0.016748	0.499529	
-0.5719	1.12478	1.52525	1.675803	1.03421	1.498865	0.937418	0.007783	0.738385	
0.444988									
0.444988	-1.17307	-1.48952	-1.51174	-1.08345	-1.43819	-1.26546	0.069053	0.428291	
0.444988	-0.3028	-0.69627	-1.0745	0.022496	-0.65522	-0.71482	0.100276	1.096999	
0.444988	-1.10721	-1.74098	-1.35851	-0.94962	-1.21687	-0.88778	0.035367	0.853199	
0.444988	1.397322	0.129246	0.121995	0.535585	-0.80375	-0.38967	0.073711	2.201072	
0.444988	-1.25716	-1.49138	-1.75572	-1.04464	-1.47067	-1.21689	0.001157	0.71108	
-1.12597	0.149519	0.406586	0.477912	0.395138	0.218232	0.193761	0.037089	0.328393	
-1.12597	-0.89978	-1.58356	-1.62505	-0.99421	-1.31254	-1.05277	0.003221	0.725272	
-1.12597	0.320389	0.683183	0.926246	0.250273	0.326731	-0.1207	0.012067	1.046947	
-0.18394	-0.67123	-1.02474	-1.48684	-1.21052	-1.27291	-1.31188	0.055875	0.815609	
0.207293	0.990264	1.142972	1.336207	1.048091	1.7412	1.52413	0.009585	0.750936	
0.31229	0.912424	1.232493	1.27976	1.109656	1.132051	1.199936	0.033082	0.367336	
-0.18394	-1.42364	-1.85213	-2.25488	-1.52372	-1.82778	-1.67889	0.012515	0.831235	
0.31229	-1.2436	-1.6123	-1.64432	-1.35128	-1.42694	-1.40334	0.035492	0.400718	
0.31229	1.220767	1.718057	1.332336	1.010633	1.907425	1.271456	0.022998	0.896792	

0.207293	1.148687	1.588277	1.786653	1.350983	1.227134	1.631483	0.02926	0.637966
-0.49404									
-0.49404	0.262924	0.486327	0.422112	0.370451	0.685833	0.5525	0.02473	0.422909
-0.49404	-0.53094	-1.71561	-2.0311	-1.40869	-1.48003	-1.25691	0.025927	1.500165
-0.49404	-0.39623	-0.36387	-0.06207	-0.16981	-0.17315	-0.48635	0.008105	0.42428
0.370023									
0.370023	-0.89188	-1.4698	-1.5044	-1.05435	-1.32167	-1.27493	0.024729	0.612514
0.370023	-0.90861	-1.57125	-1.77618	-1.04193	-1.39606	-1.00651	0.003103	0.867575
0.370023	-1.20394	-1.72106	-1.80041	-1.46378	-1.75317	-1.29381	0.014976	0.59647
0.370023	-1.13662	-1.57422	-1.66302	-1.07548	-1.34541	-1.10403	0.022968	0.587543
0.370023	0.067946	0.295028	0.784766	-0.00571	0.135642	0.129903	0.000845	0.79048
-0.42486	-0.92777	-1.35434	-1.58797	-1.26445	-1.49307	-1.39604	0.024228	0.660201
-0.42486	-1.13111	-1.61487	-1.87538	-0.88504	-1.43734	-1.17667	0.022491	0.990346
	-1.03773	-1.75919	-1.74175	-1.04743	-1.74856	-1.24609	0.056651	0.721462
	-0.97913	-1.37504	-1.49073	-0.8702	-1.17259	-1.07438	0.010205	0.620533
0.389983	0.593731	0.827122	1.01371	0.5935	0.920735	0.876162	0.085893	0.42021
0.389983	1.349625	2.35344	1.523277	1.80329	0.590888	1.733971	0.049205	1.762552
0.389983	0.213865	0.341097	0.238742	0.494968	0.93946	0.642891	0.00482	0.725594
-1.34223									
-1.34223	0.355064	0.951209	1.155097	0.541654	-0.33149	-0.42273	0.004908	1.57783
-1.1948	0.776265	1.021542	1.204079	0.685349	1.052497	1.06543	0.021779	0.51873
-1.1948	-0.22369	0.066104	0.609766	0.735363	0.15451	0.009592	0.002411	0.959049
-1.1948	0.926362	1.274257	1.813357	1.24246	1.390945	1.087954	0.000173	0.886994
-0.27508	1.043446	1.146156	1.252309	0.936505	1.46711	1.143525	0.145481	0.530605
-0.27508	0.808162	1.31678	1.55831	0.879977	1.605285	1.295443	0.000249	0.797123
-0.21595									
-0.21595	-0.1895	-0.34263	-0.3317	0.034322	-0.24262	-0.1002	0.019833	0.376953
-1.04093	0.402776	0.570216	0.550883	-0.00347	0.144344	-0.10014	0.024724	0.670355
0.231923	0.176833	0.102115	0.542874	0.346245	0.465417	0.363035	0.003037	0.440759
0.231923	0.888573	1.2311	1.348363	0.737607	0.555979	0.351865	0.005187	0.996499
0.231923	0.531522	0.081098	0.304932	0.396441	0.391836	0.317428	0.219778	0.450425
-1.04093	0.847417	1.657107	1.92398	1.465725	1.442005	0.931456	0.040938	1.076563
0.242835									
0.242835	-0.86821	-1.03081	-0.84068	-0.72967	-1.37582	-1.2416	0.092309	0.646146
-0.7639									
-0.7639	0.157131	0.152826	0.539244	0.24684	0.631919	-0.03345	0.060502	0.665372
0.221797									
0.221797	0.240307	0.577772	1.001323	0.750091	0.873503	0.308664	0.009021	0.761015
2.751913	0.660245	0.949107	1.086321	0.302124	0.878838	1.001858	0.044419	0.784197
2.751913	-0.65323	-0.34928	0.047395	0.00909	-0.41247	-0.53598	0.002807	0.700622
2.751913	0.237162	0.192699	0.708511	0.42779	-0.14932	-0.26419	0.05171	0.972702
0.00904	-0.01344	0.107617	0.093503	-0.09065	0.487954	0.340229	0.0055	0.578606
0.00904	0.822951	1.125564	1.201017	0.785662	0.918954	0.793089	0.034408	0.415355
-0.54693	0.173632	0.291281	0.430684	-0.06602	0.451229	0.348727	0.023307	0.517247
0.00904	0.42466	0.541308	0.385616	-0.10764	0.444636	0.428165	0.079729	0.64895
-0.54693	0.433101	0.381423	0.631667	0.480416	0.75158	0.624937	0.155136	0.370157

-0.54693	-0.3893	-0.55733	-0.23672	-0.36864	-0.72038	-0.76051	0.090242	0.523787
-0.54879									
-0.84768									
-0.84768	-0.14374	0.276252	-0.02114	-0.31685	-0.19411	-0.06869	0.052362	0.593102
-0.54879	-0.58188	-0.87666	-0.93639	-1.07148	-1.07852	-0.83183	0.075705	0.496643
-0.54879	0.906381	1.520325	1.669053	1.099922	1.258325	0.767845	0.034919	0.901209
-0.45961									
0.470985									
0.470985	-0.91827	-1.60157	-1.63519	-1.09752	-1.12321	-0.9039	0.067799	0.731293
0.470985	0.434142	0.119214	-0.32206	0.029246	-0.74349	-0.06846	0.076211	1.177633
0.470985	-0.72518	-1.07038	-1.34292	-0.91413	-1.16546	-0.92628	0.00228	0.617739
0.470985	-0.44362	-0.78667	-1.89776	-1.18196	-1.58079	-1.18541	0.047933	1.454144
0.470985	-0.0443	0.145522	0.295506	0.080368	0.549558	0.287113	0.018281	0.593862
0.657277	0.335152	0.55541	0.978764	0.419992	0.719855	0.567508	0.021949	0.643612
0.657277	-0.97066	-1.49674	-1.48259	-0.69268	-1.13366	-0.93773	0.000568	0.80406
0.657277	-0.72137	-1.42138	-1.37745	-0.76655	-1.14438	-0.84626	0.029873	0.700011
0.657277	-0.05448	-0.72904	-0.66578	-0.22169	-0.4275	-0.47866	0.10521	0.674556
0.287007									
0.287007	-0.51848	-0.57094	-0.44057	-0.32034	-0.57103	-0.5787	0.119724	0.258358
0.287007	-0.07067	0.215607	0.998668	0.478693	-0.18896	-0.6375	0.009849	1.636165
-0.19451									
-0.19451	0.067684	-0.26063	-0.37855	-0.1083	-0.26101	0.046948	0.034943	0.446238
-0.19451	0.01294	-0.48427	-0.2772	-0.10051	-0.10357	-7.13E-05	0.003909	0.497207
0.868103									
0.868103	-0.06602	0.036091	0.110771	0.414504	0.745808	0.262551	0.012093	0.811825
0.575807	-0.00351	-0.03513	-0.04516	0.510677	0.192973	-0.04235	0.012044	0.555834
0.575807	-0.71673	0.063281	0.762591	0.155781	-0.33868	-0.64934	7.93E-05	1.479325
0.575807	0.320669	0.947503	0.978751	0.868326	1.1275	0.66741	0.001055	0.806831
-1.08536	0.843822	1.028642	1.431245	0.962336	1.22577	0.982728	0.035885	0.587423
-0.97995	0.252815	0.711498	0.961572	0.570061	0.429268	0.27908	0.006358	0.708758
-0.97995	0.485834	1.106817	1.344927	0.902286	0.885423	0.575153	0.001526	0.859093
-0.97995	0.045607	0.27281	0.780727	0.575061	0.445613	-0.05684	0.01894	0.837571
-1.08536	0.181317	0.253373	0.096936	0.040743	0.316975	0.233853	0.299283	0.276232
-1.08536	0.909538	0.164122	0.265965	-0.02947	-0.40385	0.104246	0.071018	1.313388
0.220973	0.62295	0.756712	1.029404	0.621902	0.658696	0.800734	0.093993	0.407502
0.220973	0.747505	1.3211	1.499917	0.926448	0.71895	0.61982	0.003191	0.880096
0.403722									
0.403722	0.221848	0.528902	0.722714	0.457681	0.646554	0.528648	0.005726	0.500866
0.403722	-0.10032	-0.18828	-0.02443	0.449127	0.469844	0.298422	0.017603	0.658125
0.403722	-0.67515	-0.8843	-1.77629	0.597388	-1.33085	-1.28774	0.051982	2.373678
-1.92898	-0.12585	-0.21509	0.210907	-0.16193	-0.22188	-0.46514	0.001865	0.676049
-0.77867	0.05677	0.211871	0.214706	-0.22965	0.279326	0.222196	0.035777	0.50898
-0.13452									
-1.92898	-0.66662	-0.79507	-0.4553	-0.42847	-0.86987	-0.76992	0.078038	0.441398
	0.054791	0.338864	0.469102	0.949354	0.633946	0.350154	0.058771	0.894563
-0.13452	-1.05801	-1.47433	-1.73506	-1.08114	-1.45194	-1.01027	0.022426	0.724798

-0.13452	-0.46624	-1.19783	-1.34385	-0.69282	-1.16257	-0.7127	0.00174	0.877604
-0.13452	-0.6789	-1.2605	-1.56158	-0.42466	-1.05366	-0.72752	0.021689	1.136916
-0.13452	-1.15578	-1.59032	-1.71703	-1.1783	-1.36007	-1.17966	0.02675	0.561243
-0.13452	-0.69147	-1.27801	-1.58454	-1.01579	-1.19547	-1.08845	0.00179	0.893066
-0.13452	-0.13796	-0.59007	-0.38055	-0.5706	-0.86831	-0.77987	0.098733	0.730346
-0.13452	-0.55534	-0.32689	-1.35088	-0.75691	-1.00354	-0.9676	0.069025	1.023994
-0.13452	-1.20985	-1.58473	-1.47902	-1.28234	-1.56442	-1.22398	0.015989	0.374877
-0.13452	-1.13067	-1.21406	-1.7458	-0.70275	-2.05505	-1.21986	0.036475	1.352295
-0.13452								
-1.08836	1.005457	1.210383	1.260877	0.82639	0.337889	0.318219	0.008372	0.942658
-1.08836	0.365051	0.733137	0.587452	0.492663	-0.04271	-0.04766	0.049535	0.780795
-0.23752	-1.41303	-1.85543	-2.04308	-1.53605	-1.93303	-1.64998	0.012994	0.630055
0.52155	-1.03681	-1.42089	-1.60039	-1.03701	-1.38304	-1.10325	0.020044	0.563573
0.55957								
-0.23752								
0.55957	-0.7395	-0.97617	-1.51852	-0.73271	-1.10656	-0.83184	0.032014	0.785803
0.55957	0.148131	0.04013	-0.32881	0.228453	-0.04695	0.102706	0.038122	0.557264
0.077968								
0.077968	-1.01748	-1.46638	-1.71155	-1.16605	-1.31631	-1.12775	0.000988	0.694072
0.077968	-1.01907	-1.81122	-1.83661	-1.07726	-1.73455	-1.27997	0.01071	0.817533
0.077968								
0.077968	-0.78575	-1.38189	-1.60758	-0.90577	-1.30124	-1.13218	0.003817	0.821836
0.077968	-1.07709	-1.863	-1.86016	-1.24214	-1.72488	-1.34145	0.055766	0.785914
0.027463	0.493605	0.995349	1.305329	0.003165	0.781934	0.836862	0.00264	1.302164
0.027463	-0.70729	-1.32054	-1.5012	-0.48231	-1.24252	-1.06917	0.071277	1.018896
0.027463	0.369142	0.763373	1.256187	1.051866	0.650344	0.604837	0.001199	0.887045
-0.04121								
-0.04121	0.192294	0.751293	1.098974	0.351888	0.270397	0.14957	0.001011	0.949404
-0.04121	-0.11468	-0.51574	-0.52138	0.241619	0.242588	0.191929	0.018742	0.763969
-1.08862	0.268332	0.253962	0.556195	0.481872	0.290314	0.374539	0.02162	0.302233
-0.6781								
-0.6781	0.009784	0.136801	0.408501	0.770781	0.746547	0.313357	0.008724	0.760996
-1.08862	1.3928	1.40935	1.43173	0.826986	0.999376	0.778367	0.00093	0.653363
-0.79004								
-0.79004	-0.26947	-0.53803	-0.47683	-0.65507	-0.89361	-0.10846	0.082474	0.78515
-0.79004	-0.13053	-0.05589	0.854319	0.847548	-0.03244	-0.02439	0.002042	0.984853
-0.2955	1.500087	1.683083	1.786291	1.060792	2.159285	1.846663	0.033093	1.098493
-0.2955	-0.17935	0.260757	0.475819	-0.07025	-0.00092	-0.08515	0.051097	0.655172
-0.2955	-1.138	-1.69672	-1.81178	-1.05164	-1.166	-0.99673	0.047916	0.815045
-0.2955	-0.90772	-0.84281	-0.58142	-1.03495	-1.2148	-0.82523	0.002951	0.63337
	0.120953	0.2785	-0.51327	-0.28797	0.139423	0.23361	0.215258	0.791774
	-0.5142	-0.67775	-1.25883	-0.83809	-0.72027	-0.57051	0.021735	0.744631
-1.18102	0.394859	0.368808	0.623802	0.602083	0.78023	0.65303	0.158105	0.411422
-1.23319	0.766869	0.211575	-0.12171	-0.05995	0.182931	0.335073	0.05395	0.888583
-1.23319	-0.08115	-0.12477	-0.55058	-0.33816	0.059195	-0.02948	0.009676	0.609772
-1.23319								

-1.18102	1.88201	1.729733	1.427298	1.495973	1.86611	1.354067	0.039186	0.527943
-1.18102	-0.48871	-0.27609	0.626402	0.18114	-0.37173	-0.64055	0.002676	1.266956
-0.12431	0.587162	0.683578	1.00149	1.071252	1.61082	1.224173	0.078684	1.023658
-0.12431	-0.04489	0.054735	0.138172	0.613315	0.279172	0.022688	0.01282	0.65821
-0.12431	-0.20428	-0.17549	-0.17773	0.013116	0.123081	0.096578	0.02497	0.327357
-0.61841								
-0.22927	0.890081	1.400865	0.537611	0.618525	0.389295	0.268084	0.097336	1.132782
-0.22927	-0.39413	-0.03321	0.591537	0.124551	0.281128	-0.00843	0.002163	0.985663
-0.61841	-0.24682	-0.34239	-0.27701	-0.23706	-0.31923	-0.18482	0.033734	0.15757
-0.61841	0.460555	0.752017	1.056904	0.831781	0.727257	0.492943	0.04357	0.596349
-0.79012								
-0.79012	-0.35299	-1.04839	-1.856	-1.02148	-0.93913	-0.82095	0.004797	1.503006
-0.79012	-0.92883	-1.46682	-1.72048	-0.9453	-1.41035	-1.07963	0.001438	0.791646
-0.79012	-0.42293	-1.26697	-1.14654	-0.39433	-0.50071	-0.54197	0.020802	0.872645
-0.79012	1.201466	-0.09477	-0.60404	-0.05051	-0.12772	0.150917	0.056291	1.805505
	0.754882	0.852053	1.355609	1.035964	1.20163	0.786439	0.052102	0.600727
-0.13344	1.001516	0.949663	0.974578	0.697719	0.709653	0.386437	0.025328	0.615079
-0.13344	0.405075	1.096527	1.081117	0.675637	0.843686	0.675558	4.00E-05	0.691451
1.681908								
1.681908	-0.79988	-1.3686	-1.59716	-0.83405	-1.46027	-1.22668	0.000414	0.797274
1.681908	-0.9367	-1.59914	-1.78889	-1.00232	-1.29202	-0.86409	0.033769	0.924796
1.681908	0.086462	-0.66144	-0.8795	-0.28494	-0.55933	-0.65748	0.073555	0.965966
0.42021	-0.73075	-0.33576	0.074019	-0.64379	-0.96507	-1.10917	0.002505	1.183186
0.42021	1.60062	2.342173	2.075997	2.095173	2.266525	1.569027	0.033428	0.773147
0.42021	-0.26108	-0.66028	-0.94637	-0.65908	-0.71563	-0.49422	0.076848	0.685298
0.42021	-0.07369	0.003293	-0.52542	-0.00609	0.175583	-0.14065	0.003249	0.701007
0.13942								
0.13942	-0.00056	-0.29446	-0.35989	-0.36376	-0.46804	0.011945	0.150533	0.479989
0.13942	-0.54085	-1.1795	-1.50323	-0.69952	-1.34657	-1.10846	0.002749	0.962388
0.13942	-0.47282	-1.28493	-1.69429	-1.01307	-1.43372	-1.25943	0.014528	1.22147
0.13942	0.156784	0.291235	-0.11579	-0.06102	-0.02237	-0.06393	0.2199	0.407027
-0.48415	0.80949	1.427073	1.674433	0.989074	1.646635	1.17105	0.004159	0.864943
0.230397	0.080125	0.224798	0.353118	-0.17067	0.075454	0.214842	0.049565	0.52379
0.230397	0.666971	1.13017	1.520877	0.917707	1.47843	0.746428	0.034948	0.853905
-0.48415	-0.09488	-0.16792	-0.27388	-0.207	0.019407	0.118392	0.03596	0.392269
2.516003								
2.516003	-0.30295	-0.59668	-0.78413	-0.67785	-0.91015	-0.52635	0.002467	0.607199
2.516003	0.469795	0.198066	-0.21773	-0.1069	-0.19471	0.290561	0.002678	0.687523
0.492077								
0.492077	0.816214	0.90014	0.772663	0.635638	1.302215	1.039173	0.006703	0.666577
0.491652								
0.491652	-0.25217	-0.16279	-0.96479	-0.60154	-0.51829	-0.52015	0.108664	0.801993
0.491652	-1.07535	-1.5037	-1.76199	-1.16012	-1.48121	-1.19736	0.000312	0.686638
0.491652	-0.57343	-0.3118	-0.40248	-0.7106	-0.30179	-0.05764	0.043728	0.652961
-0.84777								
-0.84777	-0.24348	-0.08812	0.289089	0.237353	-0.30106	-0.39904	0.015601	0.688129

-0.84777	1.146987	1.713383	1.897013	1.22527	0.756764	0.410908	0.00545	1.486106	
-0.56039									
-0.56039	-0.12426	-0.35671	-0.38583	0.477515	0.01153	0.078608	0.029845	0.86334	
-0.56039	0.795472	0.763668	1.176854	1.255615	1.47776	1.033412	0.024661	0.714092	
-1.28927									
-1.28927	-0.27858	-0.55889	0.132729	0.06833	0.252358	-0.17395	0.011369	0.811246	
-1.28927	0.104176	0.549397	1.339207	0.681654	0.407883	0.206124	0.002902	1.235031	
0.038432	1.209963	1.69423	1.673352	1.21665	2.276665	1.553737	0.056754	1.066702	
-0.55654	0.316346	0.379544	1.057914	0.361636	0.687008	0.554201	0.062508	0.741567	
0.038432	0.356525	0.682395	0.918953	0.366853	1.020163	0.782356	0.040319	0.663637	
-0.55654	0.873128	1.58234	1.666633	0.918819	1.434785	1.027792	0.023711	0.793506	
1.931153	0.389506	0.706027	0.723716	0.209627	0.384071	0.445158	0.002855	0.514089	
1.931153	0.38715	0.409416	0.70397	0.567603	0.993747	0.816512	0.009592	0.606597	
1.931153	1.710217	1.532127	1.318737	1.212457	1.24784	1.318523	0.017218	0.49776	
-0.71717	1.026482	1.046032	1.199565	0.644317	1.38243	1.107935	0.033167	0.738113	
-0.71717	0.992798	1.376057	1.518203	0.80252	1.15928	1.230967	0.000352	0.715683	
-0.61373	0.658152	1.385453	1.824467	0.893123	0.802093	0.806645	0.008967	1.166314	
-0.61373	-0.14056	-0.26742	-0.19432	-0.00684	-0.23125	-0.27716	0.193187	0.270318	
-0.61373	1.105569	0.762693	1.330482	1.036838	0.626543	0.965191	0.174549	0.703939	
-0.95331	0.277379	0.400587	0.892242	0.361721	0.395166	0.390241	0.001691	0.614863	
-0.95331	0.152184	0.088608	0.03497	-0.12571	0.053451	-0.03051	0.00133	0.277895	
-0.95331	-0.6298	-0.56538	-0.33214	-0.35835	-0.32524	-0.38912	0.015229	0.30456	
0.083592									
0.083592	-0.09535	-0.36429	-0.23348	-0.10236	-0.39166	-0.33988	0.119561	0.296314	
0.083592	1.002692	1.748434	-0.14784	0.266176	0.212675	0.080972	0.11835	1.896271	
0.083592	-1.42037	-1.8564	-1.90605	-1.32196	-1.59099	-1.29193	0.055666	0.614117	
0.083592	0.093554	-0.38894	-0.98377	-0.3979	-0.28181	0.237872	0.154826	1.221647	
0.083592	-0.66944	-0.95152	-1.29881	-0.39729	-1.17992	-0.81609	0.020904	0.901521	
-0.47082	0.256973	0.388328	0.851339	0.360215	0.652937	0.581919	0.016503	0.594366	
-0.47082	0.381254	0.508865	0.530309	0.442777	0.746369	0.717526	0.009235	0.365114	
0.298013	0.144125	0.448727	0.284749	-0.17818	0.551585	0.735896	0.002126	0.914072	
0.123077	-0.23607	0.023574	-0.33818	-0.48943	-0.4469	0.085244	0.000136	0.574676	
0.123077	0.328859	0.351636	0.474156	0.18051	0.643804	0.57649	0.123812	0.463294	
0.298013	-0.20508	-0.352	-0.18228	-0.31809	0.023174	-0.06122	0.002276	0.375177	
0.298013	-0.0295	0.070467	-0.11211	-0.07105	0.323197	0.184518	0.177029	0.435306	
-0.86438	0.072193	0.274305	0.373299	-0.40925	0.417729	0.324279	0.090289	0.826975	
-0.86438	-0.77754	-1.27985	-1.59253	-0.94203	-1.27346	-0.99245	0.01857	0.814984	
-0.86438	-0.98972	-1.73845	-1.76104	-1.05446	-1.32196	-1.01843	0.000705	0.771322	
-0.86438	-0.49483	-1.21794	-1.09719	-0.70882	-1.56313	-0.9783	0.016262	1.068295	
-0.86438	1.070808	1.631007	1.80656	1.259617	1.693955	1.049498	0.010284	0.757062	
-0.50294	0.393316	0.611575	1.059012	0.140513	0.107172	-0.12571	0.016333	1.184718	
-0.78456	0.025567	0.473016	0.778197	0.250841	-0.45332	-0.35351	0.004162	1.231516	
-0.78456	0.666836	1.043456	1.81537	0.895469	0.810419	0.221645	0.009823	1.593725	
-0.50294	0.516711	1.060747	1.37387	0.55929	0.583374	0.546134	0.000641	0.857159	
0.233818	1.145191	1.550948	1.529513	0.459769	1.895625	1.666497	0.001309	1.435856	
0.449453									
0.233818									

0.449453	-0.50018	-0.85971	-0.78335	-0.68857	-0.68823	-0.59849	0.011235	0.359533
0.449453	-0.48274	-0.7543	-1.29707	-0.75016	-0.69997	-0.72092	0.012868	0.814329
-0.86328	0.437753	0.43269	0.263452	0.456099	0.812021	0.681117	0.089683	0.548568
-0.86328	-0.31949	-0.15291	0.335091	0.054339	-0.19928	-0.20912	0.047784	0.654577
-0.83596	0.248055	0.717043	1.098498	0.76136	0.604279	0.552395	0.002332	0.850443
-0.86328	3.05208	3.414563	2.77558	2.565807	2.505985	2.137557	0.002621	1.277007
-0.83596	-1.07702	-1.40201	-1.45663	-1.1167	-1.34941	-1.23452	0.125705	0.379615
-0.83596	-0.30317	-0.21647	0.153283	-0.11353	-0.10023	-0.06847	0.005656	0.456452
-0.40541	0.523996	0.97004	1.245187	0.553968	1.049314	0.99543	0.001226	0.72119
0.41007	0.550962	0.881655	1.25617	0.336983	0.883923	0.733203	1.91E-05	0.919187
0.41007	-0.79432	-1.30635	-1.38674	-1.31413	-1.21832	-1.15946	0.017715	0.592424
0.41007	0.70245	1.2557	1.55772	1.456217	1.56131	1.161983	0.000855	0.85886
-0.40541	0.707028	0.898473	1.13353	0.95215	1.179505	1.016222	0.01457	0.472477
-1.02881	-0.25507	0.058411	0.548133	-0.11188	-0.64326	-0.45727	0.000782	1.191394
-1.02881	-0.19138	0.405574	0.638126	0.778007	0.108794	0.182947	0.002494	0.969386
-1.02881	0.693156	1.404537	1.7374	0.919283	0.719956	0.463319	0.004176	1.274081
-0.47365								
-0.47365	0.043931	0.326789	0.709692	0.371606	-0.14592	-0.12813	0.001609	0.855615
0.028397								
0.028397	0.87098	1.135539	0.693053	1.254874	0.398759	0.210053	0.261909	1.044821
0.028397	0.194545	0.719779	0.948816	0.460817	0.358387	0.346918	0.049199	0.754271
0.349353	5.768917	5.569797	4.666147	4.556121	5.1386	4.380283	0.047713	1.388633
0.349353	-1.09713	-1.45787	-1.28484	-1.17253	-1.25511	-1.19338	0.155945	0.360748
0.349353	0.261728	0.796697	0.884626	0.664388	1.183335	0.836543	0.001142	0.921607
1.08949								
1.08949	0.32247	0.267389	0.399439	0.818061	0.951494	0.508251	0.031583	0.684105
1.08949	0.516869	0.57428	0.796958	0.703235	1.02347	0.743376	0.035015	0.506601
1.00777	0.157945	0.45101	0.454996	0.109319	0.419847	0.444705	0.124344	0.345677
1.00777	0.489796	0.826002	0.747217	0.811169	1.298495	1.057505	9.45E-05	0.808699
0.652307	-0.73237	-1.30528	-1.73529	-1.13488	-1.37624	-1.18347	0.000462	1.002919
	2.21385	1.793207	0.860274	2.221297	1.250435	2.214897	0.084827	1.361023
	-0.27181	-0.72442	-1.06341	-0.7451	-0.81865	-0.65498	0.044712	0.791596
-0.44199	0.224559	0.294622	0.536043	0.854597	0.9881	0.662009	0.001048	0.763541
0.652307	0.034835	0.190037	-0.22861	0.487888	-0.13378	0.032619	0.120302	0.716501
0.652307	-0.54687	-0.96524	-1.358	-0.55313	-0.86654	-0.81255	0.002943	0.811131
-0.44199	-0.00366	-0.24134	-0.13812	-0.21715	-0.04843	0.073221	0.028455	0.314564
-0.56558	1.153733	1.395363	1.316933	0.48027	1.42889	1.354043	0.013299	0.94862
-0.97954	0.657004	1.106532	0.991804	0.395203	1.39263	0.878425	0.016698	0.997427
-0.91132	0.657017	0.801403	0.842542	0.262525	1.17508	0.880041	0.003397	0.912555
-0.91132	0.953055	1.074587	1.079874	0.301658	1.332745	1.226767	0.002252	1.031087
-0.97954	-0.70618	-0.82934	-0.69439	-0.03024	-0.19422	-0.36897	0.001237	0.799094
-0.97954	-0.32108	-1.31412	-1.48565	-0.63531	-0.97803	-0.7836	0.027387	1.164565
-0.56558	0.423073	1.022803	1.182497	0.80109	0.818735	0.501467	0.0032	0.759424
-0.15425	0.81302	0.946795	1.474327	1.001861	1.150391	1.078353	0.032856	0.661307
-0.15425	-0.31227	0.267094	0.676458	0.111812	-0.05736	-0.04416	0.002831	0.988723
-0.14656								

-0.14656	-0.54628	-0.70215	-0.64448	-0.68777	-0.53982	-0.57219	0.218009	0.162334
-0.14656	0.014302	-0.56965	-0.92755	-0.47724	-0.57885	-0.15261	0.056041	0.941854
-0.14656	-1.00177	-1.26447	-1.52045	-0.97413	-1.18698	-0.94071	0.024755	0.579744
-0.14656	-0.32135	-0.63424	-0.7417	-0.69338	-0.82337	-0.58009	0.025542	0.502025
0.055913	0.693702	1.350403	1.90563	1.054215	1.24147	0.927424	6.17E-05	1.211928
0.562703	0.297873	0.422546	0.698899	0.150874	0.391043	0.37095	0.028105	0.548024
0.562703	1.016394	1.2	1.34416	1.103137	1.44442	1.170594	0.022611	0.428026
0.562703	1.097003	1.359431	1.66216	1.36275	1.49196	1.35275	0.032649	0.565157
0.055913	-0.7673	-1.16679	-1.31436	-0.91242	-1.24402	-1.07437	0.016636	0.547056
0.055913	0.107158	-0.08657	0.042475	0.404081	0.570114	0.165195	0.010277	0.656685
1.780888	-0.36379	-0.0336	-1.34271	-0.47298	-0.95552	-1.00778	0.120663	1.309115
1.599557								
1.780888	-0.80841	-1.05365	-1.42482	-1.01835	-1.18505	-1.24595	0.014797	0.61641
1.599557	0.062912	-0.21674	-0.68119	-0.3903	-0.5732	-0.38377	0.00548	0.744099
1.599557	0.750312	0.691384	0.720299	0.173012	0.962675	0.849171	0.03662	0.789662
1.037677								
1.037677	1.034433	1.565278	0.288974	0.777753	0.921519	1.022286	0.095165	1.276305
1.037677	0.824373	0.930077	0.974351	1.253312	0.901814	1.538753	0.073584	0.71438
-0.70309	0.053887	0.086384	-0.12426	0.178617	0.112144	0.02688	0.103592	0.30288
0.367697	1.616743	1.427243	1.008093	0.736447	1.52432	1.19235	0.001979	0.880296
0.367697	0.341893	0.347547	0.079796	-0.09936	0.680824	0.503614	0.017088	0.780186
0.367697								
-0.70309	0.202253	0.426874	1.395913	1.077422	0.432685	0.247757	0.003806	1.19366
-0.70309	0.2803	0.498582	1.651243	1.112258	0.63602	0.135165	0.031053	1.516078
-0.7105	0.658893	0.764224	0.711622	0.558272	1.072302	0.620261	0.058692	0.51403
-0.6747	0.410816	0.752775	0.935211	0.501335	0.616095	0.550455	0.022276	0.524394
-0.7105	-0.92374	-0.82608	-1.0815	-0.70579	-1.22611	-1.04386	0.066138	0.520321
-0.6747	-0.00314	0.037985	0.646963	0.665395	-0.00866	-0.33578	0.006935	1.001173
-0.6747	1.055287	1.67616	2.19734	1.746817	1.693955	1.18806	0.006383	1.142053
-0.28067	1.35294	1.65766	1.589129	1.261403	1.358744	1.066819	0.007438	0.590841
-0.28067	-0.4312	-0.6026	-0.33074	-0.31289	-0.17534	-0.16684	0.068764	0.435766
-0.28067	0.505245	0.48059	0.367856	0.388663	0.36379	0.309991	0.195207	0.195254
0.108								
0.012813	0.152182	0.294369	0.195296	-0.07165	0.186908	0.159674	0.021897	0.366023
0.012813	-0.32971	-0.49558	-0.07942	0.089362	-0.22366	0.138924	0.159246	0.634506
0.108	-0.71004	-0.65001	-0.56951	-0.70368	-0.43192	-0.51397	0.084538	0.278123
0.108	-0.46789	-0.3737	-0.26921	-0.22136	-0.07189	0.106242	0.058483	0.574127
1.26729								
1.26729	-0.08041	0.044709	0.04743	-0.03624	0.088424	0.175202	0.109598	0.255613
1.26729	-0.34801	-0.66255	-0.72735	-0.35909	-0.5574	-0.46532	0.090701	0.379343
-0.79386	0.205013	0.549977	0.924744	0.577042	0.845214	0.780537	0.090092	0.719732
-0.79386	-0.30626	-0.053	-0.30788	-0.27558	-0.73225	-0.55678	0.068031	0.67925
-0.79386	-0.30718	-0.18891	-0.07089	-0.08076	-0.57482	-0.41628	0.005691	0.50393
-0.82365	0.446472	0.60522	0.76346	0.733318	0.732033	0.514134	0.009683	0.316988
0.046507	-1.22899	-1.85598	-1.91892	-1.1605	-2.21701	-1.47152	0.011452	1.056508
-0.82365	-0.20845	-0.40027	-0.1544	0.617368	0.4954	0.019506	2.15E-05	1.017637
0.046507	-0.86542	-1.47438	-1.66257	-0.74533	-1.25382	-0.83164	0.013454	0.917239

0.046507	-0.70311	-1.1686	-1.41315	-0.65214	-1.13247	-0.85691	0.003888	0.761015
-0.35681								
-0.35681	-0.01645	-0.63112	-1.12813	-0.81211	-0.25961	-0.40126	0.123422	1.111677
-0.35681	0.056713	0.634509	0.688801	0.314942	-0.14997	-0.24241	0.004884	0.931207
0.213538								
0.213538	-0.8241	-1.27107	-1.37892	-0.87547	-1.29334	-1.02156	0.017395	0.554812
0.213538								
0.213538	0.379737	0.227396	0.064166	-0.07419	0.510574	0.448606	0.062219	0.584763
0.213538	-0.59439	-0.89436	-1.12386	-0.86946	-0.93799	-0.71362	0.101785	0.529466
-0.83475								
-0.83475	-0.02254	0.046048	0.006196	0.150046	0.30771	0.055333	0.046194	0.330252
-0.08419	0.04254	0.127699	0.277999	0.121427	0.329196	0.106653	0.017109	0.286655
-0.08419	-0.13165	-0.14635	-0.03059	-0.0372	0.064486	0.093064	0.068674	0.239409
-0.08419	-0.8059	-0.95299	-1.28944	-1.11046	-1.0693	-0.92582	0.017602	0.483542
-0.08419	1.148218	1.0892	0.214674	1.318252	1.336	0.433772	0.089803	1.121326
-0.08419	-1.45311	-1.85805	-2.33677	-1.62887	-1.75428	-1.55742	0.049168	0.88366
-0.08419	1.12932	0.537834	0.787318	0.155818	0.481518	1.03654	0.027168	0.973501
-0.08419	-0.45083	-0.41856	-0.31738	-0.19128	-0.09726	-0.21969	0.030321	0.353571
-0.08419	-0.47704	-0.59227	-0.44932	-0.26031	-0.38176	-0.40293	0.02472	0.331959
0.338097	-0.76968	-0.69522	-0.22185	0.096938	-0.17032	-0.33289	0.011994	0.866615
-0.49893	0.160386	0.508804	0.411662	0.495493	0.68919	0.594704	0.020405	0.528804
-0.49893	-0.22823	-0.04063	0.525365	0.502519	0.187889	-0.03223	0.0017	0.753594
-0.49893	0.276649	0.809118	0.986122	0.887246	1.03455	0.671799	0.00571	0.757901
0.338097	-0.34373	-0.74548	-0.68436	-0.37072	-0.52224	-0.33177	0.013528	0.41371
0.338097	-0.50801	-0.73615	-0.54764	-0.49022	-0.47084	-0.55461	0.059985	0.265309
0.338097	0.284074	-0.25163	-0.34073	-0.43491	-0.01097	0.013042	0.101095	0.718984
0.338097	-1.03928	-1.39575	-1.42705	-1.08837	-1.23044	-1.03312	0.069723	0.393933
0.338097	-0.1513	0.009956	0.040567	-0.22293	0.246003	0.047023	0.033114	0.468931
-0.16976	0.039099	0.428799	0.700911	-0.004	0.285441	0.243525	0.020888	0.704915
-0.16976	-0.92609	-0.5426	-0.58331	-0.28376	-1.26247	-0.36032	0.173586	0.978713
-0.16976	-0.69546	-0.22775	-0.81522	-0.50991	-0.7627	-0.79391	0.134572	0.587473
0.236173								
0.236173	0.764565	1.059635	1.187933	0.81696	0.499086	0.597469	0.113697	0.688848
0.236173	3.202107	2.9994	3.155787	2.816693	2.231885	2.48063	0.011325	0.970222
-0.61215								
-0.71639								
-0.71639	-0.29941	-0.20798	-0.06587	-0.31583	-0.53927	-0.45784	0.093052	0.473401
-0.71639	0.281557	0.699925	0.64739	0.305197	0.721128	0.582949	0.058053	0.439571
-0.61215	0.505718	0.306044	0.579932	0.711585	0.667114	0.476193	0.112621	0.405541
0.059413								
0.059413	-0.17428	-0.15317	-0.26657	-0.10188	0.051249	0.089125	0.047259	0.355691
-0.67566	0.996843	1.146084	1.448427	1.178383	1.332725	1.353	0.003629	0.451583
-0.67566	-0.01505	0.515115	1.051969	0.555527	0.468091	0.289493	0.01897	1.067014
0.22254	1.079862	1.403183	1.523687	1.404517	1.376175	1.064737	0.001924	0.45895
-0.48811	1.366373	1.895913	2.235347	1.502638	2.441005	1.870233	0.006853	1.074632
-0.48811	-0.73108	-0.67611	-0.48813	-0.47693	-0.35961	-0.46745	0.038593	0.371474
-0.48811	-0.75918	-0.27721	-1.43344	-0.69206	-1.11482	-1.02242	0.163888	1.156227

-0.48811	-0.91033	-1.08239	-0.85825	-0.74355	-0.68245	-0.71411	0.045065	0.39994
-0.27285	0.937105	1.323063	1.665783	1.075137	1.601955	1.132225	0.011827	0.728679
0.22254	1.073195	1.35588	1.658937	1.137441	1.56865	1.22607	0.008518	0.585741
0.22254	0.11574	-0.10999	-0.23538	-0.09425	0.302093	0.183264	0.011601	0.537474
-0.27285	4.304895	4.049429	3.60297	3.805153	2.962855	3.66828	0.039046	1.34204
-0.31946	0.341766	-0.28191	0.049841	-0.39373	-0.65501	-0.76852	0.038902	1.110284
-0.31946	-0.90134	-1.49515	-1.63584	-0.97815	-1.10621	-0.92686	0.065822	0.734499
-0.31946	-1.12579	-1.13139	-1.94758	-1.39323	-1.99901	-1.60565	0.016644	0.873218
-0.31946	-0.65226	-0.97195	-1.4643	-0.97027	-1.55599	-1.10394	0.001567	0.903728
-0.31946	-0.73544	-1.4541	-1.60062	-0.77838	-1.31886	-0.84738	0.004674	0.865179
-0.31946	-0.79441	-1.34454	-1.86918	-1.10876	-1.44696	-1.16404	0.001095	1.074771
-0.31946	-0.85072	-1.45292	-1.59076	-0.84649	-1.53925	-1.11802	0.000471	0.744265
-0.31946	0.348647	-0.45054	-1.22006	-0.24845	-0.58624	-0.32927	0.010088	1.56871
-0.31946	-0.87344	-1.20879	-1.16556	-1.01038	-1.2252	-0.9155	0.221447	0.351767
-0.31946	-1.13836	-1.62911	-1.84268	-1.11316	-1.44559	-1.24528	0.019517	0.729516
	1.432434	1.130583	0.854935	0.987641	1.016123	0.462114	0.136255	0.97032
	1.368399	1.326109	0.590064	0.34852	0.52937	0.557399	0.047544	1.019879
	-0.31889	-0.00624	-1.23004	-0.55702	-0.9409	-0.67559	0.162847	1.223801
	-0.47634	-0.75734	-0.97478	-0.47126	-0.77672	-0.62057	0.000923	0.503522
-0.10399								
-0.10399	-1.24765	-1.6427	-1.72228	-1.17802	-1.39941	-1.39601	0.110348	0.544268
-0.10399	0.053125	0.722755	0.961025	0.512415	0.792939	0.536437	0.031723	0.9079
0.0547								
0.0547	0.195	0.196932	0.310604	0.627218	0.387748	0.258805	0.025601	0.432218
0.0547	0.898973	1.136258	0.832502	0.370222	0.753865	0.802667	0.016347	0.766036
0.317342	-0.33603	-0.21021	-0.01081	-0.32347	-0.3091	-0.04196	0.009183	0.325224
0.317342	0.062626	0.030018	-0.10961	0.103335	-0.0564	0.012838	0.014661	0.212942
-0.37069	0.769132	0.763541	0.741082	0.319827	0.574112	0.44692	0.059299	0.449305
-0.37069	0.22385	0.290207	0.549717	0.808753	0.640923	0.473171	0.013977	0.584903
-0.37069	0.059593	-0.55189	-0.72141	-0.47203	-0.68358	-0.50401	0.093226	0.780999
-1.02203	0.364226	0.168686	1.144404	0.403769	0.822563	0.822667	0.004482	0.975718
-1.02203	1.301057	1.638687	1.668997	1.082488	1.552965	1.438757	0.001564	0.586509
	0.832308	1.082597	1.372293	1.036428	0.795279	0.758852	0.017877	0.613442
-1.08246	-0.00338	0.124431	0.349397	0.155635	-0.0989	-0.0323	0.035215	0.4483
-1.05718	1.688433	1.981077	1.893307	1.808787	1.775085	1.491547	0.077642	0.48953
-1.08246	0.426925	0.224652	0.282812	0.183316	0.296256	0.238625	0.185449	0.243609
-1.05718	-0.22271	0.055485	0.738318	0.270959	-0.41251	-0.13101	0.103942	1.150828
0.701947								
0.701947	0.677883	0.895397	1.019306	0.99134	0.358518	0.479519	0.094944	0.660788
0.701947	0.6196	0.818818	0.862655	0.448087	0.845273	0.835187	0.010908	0.414568
0.948958	0.082396	-0.08467	-0.18888	-0.63617	-0.7133	-0.51558	0.004002	0.795695
1.051263								
1.051263	0.875272	1.055357	0.754448	0.131434	0.093611	0.307113	0.00033	0.961746
0.948958	-0.02742	-0.1145	-0.10159	0.039429	0.201904	0.033411	0.064976	0.316402
-0.46299	-0.11926	0.26672	0.205256	0.174348	0.412135	0.29553	0.028287	0.531391
-0.46299	-0.6967	-1.11816	-1.34583	-0.76278	-0.84671	-0.7138	0.006135	0.649128

-0.79382									
-0.79382	-0.54282	-1.19843	-1.55222	-0.61244	-1.23392	-0.82988	0.005111	1.009407	
-0.79382	0.050866	-0.41623	-1.01375	-0.56613	-1.00671	-0.7053	0.003742	1.064615	
-0.6111									
-0.6111	0.9919	1.423923	1.35874	0.680802	1.49827	1.16111	0.005906	0.817468	
-0.06328									
-0.06328	0.575419	0.670091	0.631323	0.582289	0.555878	0.482898	0.181852	0.187193	
-0.80832	0.986872	1.64255	1.706647	1.263	1.3604	1.163713	0.008803	0.719775	
-1.03998	-0.69369	-1.17543	-1.67628	-0.77706	-1.27656	-1.00403	0.017319	0.982585	
-0.59582									
-0.59582	-0.72971	-1.3537	-1.4796	-0.66352	-1.32036	-0.85712	0.001736	0.816082	
-0.59582	-0.29455	-0.47055	-0.67124	-0.35683	-0.59327	-0.28093	0.020344	0.390308	
-0.70272									
-0.70272	-0.97578	-1.36605	-1.62662	-1.0053	-1.57391	-1.26489	0.018471	0.650841	
-0.71501									
-0.71501	0.798466	0.510011	-0.0721	0.383212	-0.1523	-0.0432	0.115435	0.950762	
-0.69137	2.17421	2.00718	0.860752	0.912124	1.011812	0.781448	0.012675	1.392762	
-0.69137	-0.39644	-0.7842	-1.34393	-0.7416	-1.45131	-1.22942	0.069751	1.054866	
-0.43813									
-0.43813	-1.15804	-1.78574	-2.05097	-1.18222	-1.91322	-1.37446	0.010989	0.89293	
-1.09101									
-1.09101	0.708036	0.828343	1.020021	0.907083	0.960642	0.903096	0.100359	0.311985	
-1.09101	0.258445	0.484783	0.934968	0.279599	0.667979	0.436945	0.002237	0.676523	
0.038323	0.356089	-0.02123	-0.86077	-0.26457	-0.23903	-0.36329	0.075204	1.216858	
0.038323	0.04236	0.251802	0.222646	0.558929	0.601614	0.311725	0.036539	0.559254	
-0.4318									
-0.4318	-0.25038	-0.50974	-0.5329	-0.24086	-0.7463	-0.62675	0.046551	0.505434	
1.872987									
1.872987	0.44427	0.827536	1.010855	0.356343	0.477613	0.552621	0.008928	0.654512	
-0.20569	0.554318	-0.14011	0.110541	-0.17144	-0.6504	-0.16934	0.022067	1.204718	
-0.20569	-0.77181	-1.2871	-1.64555	-0.9952	-1.57025	-1.21248	1.43E-06	0.873736	
-0.20569	-0.68201	-1.35722	-1.33571	-0.60804	-1.35861	-0.89586	0.00962	0.750574	
-0.25471									
-0.25471	-0.81621	-1.17613	-1.42235	-0.89393	-1.30981	-0.98211	0.037234	0.606144	
0.76219									
0.76219	-0.71876	-0.71787	-0.56188	-0.49401	-0.59215	-0.55867	0.137864	0.224749	
-0.20171	-0.34235	-0.21974	-0.22858	-0.35101	-0.43701	-0.39227	0.121206	0.217273	
-0.00158	-0.43879	-0.39986	-0.34979	-0.51642	-0.5396	-0.49951	0.141931	0.189809	
-0.00158	-0.64263	-1.20682	-1.42339	-0.93584	-1.30989	-0.94234	0.001172	0.780756	
-0.20171	-0.12317	-0.39306	-0.97034	-0.50948	-1.02001	-0.69887	0.003989	0.896836	
-0.72528									
-0.72528	-0.482	-0.8933	-1.28149	-0.76805	-1.40469	-1.01581	0.013087	0.922686	
-0.72528									
-0.65105									
-0.65105	1.015848	1.554975	1.28408	1.401474	0.430964	1.342669	0.144144	1.124011	
-1.00002	0.460375	0.235845	0.362553	0.266534	0.793884	0.671584	0.026157	0.558039	
-1.00002	-0.6941	-1.14044	-0.98433	-0.48402	-0.81166	-0.57943	0.004511	0.656421	

-0.24202	-0.16808	-0.08001	0.021326	-0.30605	-0.00236	0.009239	0.068749	0.327372
-0.24202	0.379872	0.493112	0.002389	0.700179	0.089846	0.11098	0.117028	0.697791
0.626703	0.450896	0.787098	0.970401	0.338551	1.206185	0.993567	0.023143	0.867634
0.626703	0.651904	0.851402	0.778795	0.171193	0.515207	0.689049	0.028698	0.680209
0.81774								
0.81774	0.185777	0.009111	-0.65113	-0.24399	-0.47136	-0.44306	0.005616	0.836904
0.81774	-0.40693	-0.59966	-0.86651	-0.09852	-0.8482	-0.59568	0.047857	0.767986
0.107543								
	-1.00395	-1.11659	-1.02793	-0.90608	-0.99964	-1.14546	0.087155	0.239383
	6.545807	6.74038	5.896603	5.81095	5.76916	5.647345	0.033097	1.093035
0.574087								
-0.47516	0.401176	0.201253	-0.10456	0.443347	-0.09615	-0.27261	0.056036	0.715953
0.574087	0.125707	-0.12059	-0.35525	0.018525	-0.3185	-0.23168	0.00284	0.48096
0.574087	0.838348	1.267337	1.565997	1.044682	1.32911	1.128437	0.003517	0.727649
-0.32736	0.50367	0.438393	0.63851	0.400675	0.688793	0.554843	0.320758	0.288118
-0.32736	0.7724	1.139477	1.516837	1.10684	1.4442	1.231533	0.000356	0.744437
	0.049066	0.069128	0.118371	-0.14614	-0.3003	0.223245	0.09589	0.523548
1.38704	1.3635	1.164947	0.66971	0.754089	0.845691	0.717121	0.014549	0.69379
1.38704	-0.65736	-0.96137	-0.81776	-0.5493	-0.60701	-0.66731	0.006645	0.412067
-0.15066	0.165574	0.692317	0.816986	0.524167	1.33975	0.967936	0.007015	1.174176
	-0.06144	0.37009	0.984064	0.361724	0.58524	0.545064	0.012161	1.045499
-1.07254	0.322146	0.534652	0.996159	0.673448	0.62601	0.302281	0.053107	0.693878
	0.907447	1.033549	1.185193	0.713825	1.436055	1.181311	0.004529	0.72223
-0.37152	0.652049	0.942338	1.27766	0.676508	1.173905	1.108385	0.001783	0.625611
	0.866941	1.230366	1.783043	0.883925	1.318035	1.065498	0.031409	0.916102
0.45785								
-0.41662								
-0.41662	0.212821	0.349393	0.337435	0.244569	0.173805	0.099631	0.00862	0.249762
-0.41662								
	0.271287	0.569362	0.434694	0.321246	0.554073	0.597806	0.066123	0.326518
	-0.06174	-0.02818	0.335057	0.211311	0.249208	0.203428	0.031317	0.396793
-0.26745	0.525167	0.35937	0.467443	0.437086	0.120998	0.28236	0.015027	0.40417
	0.870846	1.25098	1.37256	1.030216	1.42355	1.47749	0.004019	0.606644
0.84468	0.666804	1.268484	1.299687	0.495864	1.080422	1.042683	0.000433	0.803823
	0.52982	0.40024	0.633505	0.351564	0.205835	0.191814	0.053896	0.441691
-1.01263	0.664167	0.941879	1.19722	0.673127	0.850373	0.860364	0.047303	0.533053
-1.23315								
-1.23315	5.576313	5.4346	4.52319	4.567157	4.18679	4.559497	0.007607	1.389523
-1.23315	4.179713	3.929821	3.406402	3.282975	3.21328	2.769071	0.014187	1.410641
-0.99697								
-0.92492	0.362609	0.27616	0.333233	0.285631	-0.17363	0.200721	0.023125	0.536238
-0.76369								

-1.19433	-0.68645	-1.1556	-1.45472	-0.85342	-1.1789	-1.15833	0.028098	0.768273
-1.19433								
2.570763	-0.36778	0.26998	0.908937	-0.29771	0.638865	0.526329	0.017176	1.276719
2.570763	-0.8126	-1.20184	-1.20495	-0.71782	-1.1058	-1.01891	0.00341	0.487124
2.570763	4.49283	3.59755	2.79496	3.118793	3.177015	2.684137	0.053243	1.808693
-0.42621								
-0.42621	0.685424	1.194607	1.25145	0.476723	0.945682	0.973762	0.00637	0.774727
0.368663	1.510303	1.782503	1.873773	1.56574	2.043365	1.706577	0.05018	0.533062
0.368663	-0.40784	-0.34879	-0.22808	-0.22615	-0.2168	-0.2559	0.090773	0.191046
3.706968	0.228654	0.517343	0.675743	-0.42205	-0.04073	0.138961	0.00379	1.097797
3.706968	-0.23768	-0.80662	-1.18773	-0.55348	-1.09446	-0.78408	0.005883	0.950046
3.706968	-0.57226	-1.20233	-1.27734	-0.59281	-0.99176	-0.69714	0.035221	0.705084
3.706968	-0.66792	-0.81299	-0.7256	-0.17123	-0.90294	-0.53611	0.001762	0.731717
-0.79861								
-0.79861								
-0.79861	0.62992	0.710704	0.73526	0.978853	0.921566	0.741312	0.049851	0.348933
1.451027								
1.451027	0.197568	0.312308	0.46659	0.01896	0.180751	0.183631	0.032594	0.44763
-0.07951	-0.02261	0.406285	0.629227	0.378967	0.057577	0.122613	0.003762	0.651838
-0.07951	0.407408	0.451963	0.649088	0.963917	0.865769	0.827146	0.020749	0.556509
-1.00155								
-0.07951	1.886273	1.89577	1.4545	1.378483	1.58045	1.236353	0.009632	0.659417
-1.00155	-0.61349	-0.65091	-0.44526	-0.42712	-0.17114	-0.26227	0.061455	0.479768
-1.00155	1.491643	1.056185	1.470183	0.953182	0.923439	0.685821	0.193574	0.805822
0.60339	5.333287	5.572207	4.7185	4.58289	4.435335	4.134487	0.028026	1.43772
0.60339	0.51234	0.856192	1.235917	0.973264	0.794708	0.635906	0.016051	0.723577
0.77877	0.506308	0.848474	1.077893	0.615382	0.954382	0.927356	0.008117	0.571585
0.77877	0.469009	0.385559	0.16832	0.215022	0.418331	0.535136	0.014536	0.366816
0.77877	-0.97554	-1.3248	-1.58569	-1.04891	-1.21126	-1.16076	0.002825	0.610146
0.77877	1.29868	1.120947	1.534853	1.163661	1.745355	1.396663	0.00416	0.624408
-0.89422	0.698142	0.901412	0.760694	0.30005	1.286809	1.171133	0.026649	0.986759
-0.89422	0.780949	0.658001	0.388001	0.298774	1.11652	1.082889	0.010496	0.817746
-0.89422	0.152187	0.325539	0.865957	1.01295	0.56659	0.341444	0.001239	0.860763
-0.18434								
-0.18434	-0.37297	-0.07615	0.229974	-0.29763	-0.36751	-0.25511	0.010333	0.602944
-0.18434	-0.5623	-0.63044	-0.90761	-0.60828	-1.13322	-0.82531	0.004738	0.570912
0.343943	0.179983	0.259503	0.748412	0.196925	0.764503	0.422164	0.049455	0.58452
0.652542	0.025432	0.160628	0.44393	0.241244	0.552435	0.471988	0.051032	0.527003
0.652542	0.057593	-0.40764	-0.62563	-0.30845	-0.6649	-0.46481	0.014528	0.722493
0.652542	-0.58034	-0.44099	-0.71934	-0.09461	-1.11102	-0.18261	0.158891	1.016411
0.652542	0.664377	1.293965	1.317536	0.510708	1.059335	0.875818	0.011987	0.806829
-0.14784	1.104449	1.173784	0.792509	0.653603	0.623921	0.475118	0.044469	0.698666
0.343943	1.338756	1.742714	0.781109	1.072746	0.199016	0.9493	0.087582	1.543698
0.343943	-1.03877	-1.43532	-1.4828	-1.13439	-1.2811	-1.18944	0.018622	0.444027
0.343943	-0.71966	-1.1577	-1.11523	-1.07342	-1.19228	-1.15499	0.035767	0.472621
-0.75685	3.200827	3.286067	2.674737	2.459797	2.64408	2.177453	0.030405	1.108613

-0.14784	-0.17434	-0.40952	-0.638	-0.48657	-0.67779	-0.46429	0.115802	0.503451
-0.14784	-0.04508	-0.07921	0.13967	-0.16766	0.295915	-0.12923	0.243048	0.463572
-0.14784	1.260274	1.355802	1.571994	1.109855	1.92939	1.57639	0.039122	0.819535
-0.75685	0.479164	1.21656	1.156325	0.643661	0.676487	0.458006	0.000345	0.758554
-0.75685	0.955497	1.406731	1.826807	0.895195	1.52606	1.084038	0.035841	0.931612
0.874387	-0.65813	-0.96018	-1.1407	-0.7599	-1.08885	-0.93017	0.013235	0.482575
0.874387	-0.22358	-0.26547	-0.59083	-0.58022	-0.42919	-0.43645	0.027534	0.367249
-0.29615	0.481722	0.983871	1.304316	0.802255	1.205595	0.940583	0.041784	0.822594
0.874387	1.009658	1.16602	1.34578	0.805322	1.380555	1.113267	0.06223	0.575233
-0.29615	0.010146	0.157967	0.13264	-0.04108	0.342907	0.264763	0.06013	0.383984
-0.29615	0.629042	1.151877	1.370543	1.38291	1.427655	0.980198	0.001077	0.798613
0.717323								
0.717323								
1.149717								
1.149717	-0.87785	-1.09584	-1.66448	-0.8716	-1.25683	-1.00556	0.003476	0.792876
1.149717	-0.22931	-0.56635	-0.658	-0.31098	-0.39731	-0.63988	0.037705	0.428695
1.599867								
1.599867	-0.60509	-0.65741	-0.68963	-0.56129	-0.75997	-0.58514	0.141449	0.198675
1.599867	0.90243	1.19636	1.42751	0.460014	0.406976	0.62201	0.012088	1.020534
1.416053	1.787214	0.163567	-0.54757	-0.21111	-0.10539	-0.56443	0.049796	2.351647
1.603692	0.544448	0.518714	0.269842	0.109051	0.172679	0.256016	0.01697	0.435397
1.416053	-1.06734	-1.57406	-1.78465	-0.9451	-1.28078	-0.90756	0.00231	0.877094
1.416053	-0.82207	-1.27673	-1.41642	-0.82405	-1.02823	-0.85461	0.015211	0.594353
1.603692	0.853787	1.494666	-0.05897	0.238749	0.449228	0.110948	0.104142	1.553631
1.603692	-0.85801	-1.34351	-1.65308	-0.73278	-1.04708	-0.80124	0.004469	0.920305
1.603692	-0.71436	-1.35926	-1.50751	-0.68922	-0.92219	-0.65831	0.060909	0.849203
1.603692	0.748567	0.7185	0.616829	0.226704	0.365053	0.737983	0.010041	0.521863
0.26892	0.290416	0.217865	0.860487	0.184821	0.747845	0.584331	0.020841	0.675666
0.26892	-0.18611	-0.4826	-0.55616	0.022492	-0.36039	-0.34167	0.027587	0.578652
0.26892	0.217483	0.342175	0.595286	0.755597	0.6451	0.409786	0.00374	0.538113
-1.11962	0.988123	1.782457	2.289697	1.359863	1.5977	1.193297	0.001511	1.301574
-1.27126	0.641842	0.803022	0.939685	0.867176	0.757619	0.687568	0.135687	0.297843
-1.27126	1.149984	1.538417	1.811643	1.176422	1.64847	1.27067	0.053707	0.66166
-1.11962	-0.47268	-1.02582	-1.01474	-0.32002	-1.05992	-0.59214	0.176952	0.739899
-1.11962	-0.79701	-1.26258	-0.76162	-0.67502	-1.21387	-0.987	0.01451	0.587562
-1.11962	0.463339	1.16157	1.381233	0.484198	1.062929	0.732971	0.001713	0.917894
	0.264725	0.433519	0.327042	-0.03458	0.268644	0.354132	0.035088	0.468101
	-0.0557	-0.10719	-0.28089	0.235981	0.363667	0.140061	0.015588	0.644555
0.790407								
0.790407	0.36558	0.134886	0.173808	0.286108	0.238689	0.251668	0.06546	0.230695
0.790407	-0.16376	-0.35916	-0.6285	-0.23875	-0.38057	-0.22732	0.119537	0.464736
0.111483	0.973679	1.130021	0.821676	1.020773	2.01086	1.606583	0.005206	1.189184
0.111483	0.446943	0.533606	0.110612	0.414545	1.365905	0.965973	0.006571	1.255293
0.37917	-0.07089	-0.19861	0.168123	-0.04671	-0.39769	-0.17956	0.030349	0.565812
0.37917	-0.64895	-0.79812	-1.17836	-0.74199	-0.578	-0.47954	0.002028	0.698818
0.37917	1.85283	1.980337	1.747987	2.467433	1.79755	1.329367	0.187466	1.138067

2.252457	1.004233	1.276567	1.157783	0.623234	0.921058	0.913729	0.010866	0.653332
2.252457	0.570433	0.746779	0.935722	0.86486	0.903738	0.960779	0.067885	0.390347
2.0379								
2.0379	2.180027	3.16106	2.395147	2.449497	2.12721	2.40505	0.127992	1.03385
-0.21855	0.629332	0.441047	0.479626	0.745116	1.14432	0.984893	0.001297	0.703273
-0.21855	0.535162	0.890574	1.253603	1.40535	1.38458	0.831596	0.004882	0.870188
-0.60887								
-0.21855	0.323064	0.498576	0.758113	0.854654	0.617172	0.448015	0.035291	0.53159
-0.60887	1.24146	1.388563	1.16963	1.407397	1.372655	1.218749	0.077174	0.237767
-0.60887	-0.45927	-0.53976	-0.42141	-0.30356	-0.32688	-0.36574	0.115637	0.236202
0.53459								
0.53459	-1.39617	-1.87634	-1.91772	-1.38262	-1.73045	-1.50039	0.017481	0.535103
0.53459	1.67676	1.25689	0.4839	0.705003	0.352285	0.354943	0.005666	1.324476
0.53459	-0.79442	-1.5489	-1.53512	-1.00457	-1.50037	-1.12249	0.025849	0.75448
0.53459	-0.96082	-1.50607	-1.76675	-1.01315	-1.37401	-1.21877	0.00415	0.805931
0.53459	-0.39863	-0.56923	-0.30931	0.0567	0.065325	-0.12499	0.001656	0.634558
0.76024								
1.05293	-0.41524	-0.28231	-0.16717	-0.25584	-0.19679	-0.30408	0.069175	0.248073
1.05293	-0.86454	-1.29414	-0.72534	-0.86275	-0.66768	-0.72758	0.105334	0.626461
1.05293	2.998697	3.01685	2.652847	2.133773	2.53861	1.966217	0.044047	1.050633
0.76024	0.178198	0.767754	0.846271	0.708779	0.414697	0.547165	0.137045	0.668073
0.76024	0.643542	0.975263	1.051853	0.90999	1.27055	1.091043	0.009459	0.627008
1.51464	0.372647	0.592233	0.856486	0.163099	0.504079	0.701283	0.015735	0.693387
1.51464	-1.3903	-1.68653	-1.93361	-1.20004	-1.90043	-1.45232	0.108661	0.733567
1.51464	-0.564	-0.34866	-0.17725	-0.08405	-0.13824	-0.15937	0.011493	0.479958
-0.22127								
-0.22127	-0.19119	-0.47414	-0.10003	-0.00748	-0.14581	-0.21947	0.02493	0.466651
-0.22127	-0.15942	-0.05485	0.328257	0.212023	0.118887	-0.06377	0.00355	0.487678
0.980945								
0.980945	2.959073	2.928403	3.034677	2.772137	2.06278	2.879307	0.194035	0.971897
-0.12822								
-0.12822	0.037467	0.144756	0.048063	-0.03455	-0.10463	0.086791	0.165132	0.249387
-0.12822	-0.69061	-1.48453	-1.7331	-1.04841	-1.25045	-1.16771	0.00618	1.042492
0.378458	1.064967	1.492287	1.876043	1.168716	2.006755	1.277082	0.032729	0.941788
-0.92524	0.391076	0.697259	1.03741	0.614467	0.663193	0.45798	0.009164	0.646334
-0.92524	1.248877	1.826737	1.89673	1.421137	2.01242	1.662717	0.004895	0.763543
0.71426	-1.11658	-1.49074	-1.67564	-1.41989	-2.03766	-1.56758	0.009059	0.92107
0.378458	-0.75731	-1.00666	-1.29968	-0.84741	-1.3103	-0.81377	0.039897	0.552986
0.378458	-0.89436	-1.25552	-1.23433	-0.66416	-0.86307	-0.72917	0.010223	0.591366
0.378458	0.110091	0.536887	0.829501	0.810479	0.97177	0.651699	0.001465	0.861679
0.71426	0.931649	1.162406	1.553953	0.909456	1.212685	1.21223	0.001653	0.644497
-0.4307								
-0.4307	-1.05615	-1.83015	-2.00917	-1.07691	-1.44637	-1.11486	0.001751	0.953017
-0.4307	-0.74608	-1.14889	-1.34751	-0.77934	-1.0612	-0.78966	0.061884	0.601427
-0.4307	0.095829	-0.17018	-0.71803	-0.15771	-0.5829	-0.25663	0.012397	0.813862
	0.865059	1.035737	1.39404	0.588313	1.32698	1.138162	0.043845	0.805727
	-0.08401	-0.09347	0.370257	0.271607	0.27064	0.225604	0.030906	0.463726

1.58331									
1.58331	0.523868	1.167563	0.82181	0.314458	0.244486	0.512788	0.096194	0.923077	
1.58331	0.80549	0.825793	1.036847	1.122596	1.48279	1.31393	0.022411	0.6773	
0.515248	0.643123	1.033359	0.779193	0.760554	1.533315	1.099345	0.003247	0.890192	
0.515248									
-0.47949	-0.01074	0.192745	0.305919	0.327556	0.495496	0.224392	0.001559	0.506234	
-0.47949	0.876832	1.066467	1.192037	1.071392	1.45172	1.069615	0.007609	0.574888	
-0.47949	-0.08075	-0.03038	0.281524	0.254543	0.020116	-0.10373	0.126905	0.385257	
-0.57754	-1.15103	-1.47157	-1.52815	-1.1133	-1.35461	-1.14336	0.12643	0.414853	
-0.57754	-0.77773	-0.97944	-0.80735	-0.70384	-0.77824	-0.89713	0.167639	0.275602	
-0.01052	0.134788	-0.20796	-0.54186	0.617903	-0.06466	-0.24192	0.164466	1.15976	
-0.01052	0.410609	0.380117	0.372419	0.314279	0.564995	0.500004	0.018372	0.250716	
	0.552506	0.82295	1.107864	0.503889	1.127414	1.002959	0.058313	0.623524	
-0.01052	0.557888	0.628328	0.575756	0.489646	0.836576	0.783135	0.039667	0.34693	
	0.596861	0.696321	0.748414	0.478027	0.79679	0.731528	0.083083	0.318763	
-0.0158	0.330517	0.588512	0.776811	0.420186	0.979027	0.864806	0.031808	0.64851	
-0.0158	0.909426	1.063173	1.091258	0.756677	1.41493	1.337707	0.066702	0.658253	
0.4397	0.126297	0.150518	0.702626	0.161191	-0.94358	-0.58229	0.028195	1.646203	
0.4397	-0.05236	0.333385	0.499524	0.382474	0.19947	0.195416	0.007429	0.551886	
0.4397	0.874186	0.670115	-0.13198	0.155378	-0.12006	-0.05886	0.011831	1.006164	
0.4397	0.144649	0.385742	0.760722	0.062905	-0.47886	-0.29605	0.000257	1.239585	
-0.45503	0.444356	0.427437	0.731507	0.622968	1.0284	0.775073	0.004626	0.600962	
0.353373	1.82047	2.350353	1.66364	1.261876	1.263605	0.98486	0.002039	1.365493	
0.353373	0.905147	1.115487	1.032393	0.819911	1.460435	1.153785	0.01094	0.640524	
0.353373	-1.05494	-1.3476	-1.47119	-1.12868	-0.96428	-0.97563	0.047678	0.506915	
0.353373	-0.84703	-1.09128	-1.084	-0.76542	-0.76058	-0.87178	0.016836	0.330704	
-0.45503	-0.33852	-0.29445	0.240141	0.35141	-0.00243	-0.40017	0.088892	0.75158	
-0.45503	0.130252	0.363649	0.613511	0.701677	0.823595	0.541331	0.00287	0.693343	
-0.10435	0.237183	0.537441	0.616547	0.547032	1.040926	0.696613	0.019936	0.803742	
-0.10435	-0.51718	-1.08806	-1.07643	-0.82695	-0.86071	-0.84685	0.145553	0.570888	
-0.10435	1.143978	1.34837	1.688793	1.32794	1.86052	1.54373	0.006619	0.716542	
-0.67975	0.602094	0.675835	0.923245	0.612625	1.295315	1.028526	0.101891	0.693221	
-0.67975	-0.04696	0.391509	0.748852	0.446983	0.562214	0.129879	0.034517	0.795813	
-0.31097									
-0.31097	0.590109	0.841028	1.23415	1.25158	0.897573	0.599565	0.021962	0.661471	
-0.03938	-0.20019	-0.34893	-0.24846	-0.19011	0.737443	0.439462	0.023962	1.086374	
-0.03938	-0.02441	-0.06043	0.494211	0.522654	0.609092	0.485407	0.004307	0.669522	
-0.03938	0.007877	-0.03342	-0.48495	-0.24416	0.784251	0.530356	0.013416	1.269201	
0.079508									
0.079508	-0.89485	-1.31761	-1.39807	-1.00293	-1.29968	-1.12525	0.005383	0.503224	
0.079508	-0.97977	-1.47128	-1.76904	-0.81751	-1.29199	-1.07619	0.002263	0.951538	
0.079508	-0.97452	-0.77815	-1.67126	-1.10765	-1.39007	-1.15378	0.170195	0.893111	
0.079508	-0.07259	0.021478	0.458679	0.005173	-0.42885	-0.31171	0.012481	0.887533	
1.927503									
-0.30159									
-0.30159	-0.15936	-0.28046	-0.3066	-0.53941	-1.08314	-0.90874	0.061159	0.923783	
1.927503	1.736953	2.015617	2.198337	1.597323	1.24938	1.135953	0.001333	1.062383	

0.10014	-0.46588	-0.44524	-0.02277	0.073331	-0.085	-0.338	0.012841	0.53921
-0.23724								
0.10014	0.283697	0.155968	0.25281	0.407762	0.436561	0.408424	0.164448	0.280593
-0.23724	-0.88656	-1.40376	-1.26056	-0.73859	-1.04597	-0.84242	0.008502	0.665174
-0.23724	-0.14752	-0.14833	-0.63539	-0.44698	-0.46786	-0.12689	0.026523	0.508498
-0.24604	1.115508	1.236254	1.387527	1.129143	1.196937	1.067819	0.268965	0.319708
-0.24604	0.224436	-0.00385	0.388305	0.037582	0.230739	0.166257	0.039925	0.392157
-0.24604	0.667604	0.185896	0.478289	0.511322	0.379036	0.235438	0.159485	0.481708
3.61775	0.151034	0.526588	0.586073	-0.01212	0.214398	0.718236	0.008163	0.730359
3.61775	1.048686	1.061578	0.803988	0.555431	0.591781	0.893943	0.012198	0.506147
2.43036	0.041034	-0.05913	-0.11363	-0.3706	0.265644	0.28223	0.005381	0.652833
2.43036	0.31929	0.422934	0.243632	0.615299	0.909224	0.677305	0.000142	0.665591
2.43036	-0.65607	-1.18061	-1.32513	-0.98788	-1.11855	-0.83519	0.001202	0.669054
-0.61254	0.390819	0.433513	0.477043	-0.05304	0.810088	0.552421	0.022754	0.863123
-0.61254	1.054651	1.304871	0.636481	1.339148	1.053243	0.803012	0.178965	0.702667
-0.14281	1.67344	1.459677	1.516367	1.074562	1.691725	1.194193	0.07619	0.617163
-0.14281	1.77581	0.887742	1.092922	0.769287	0.887682	0.775885	0.047958	1.006523
-0.14752	1.392537	1.788857	1.761733	0.996736	1.887845	1.666087	0.020836	0.891109
-0.14752	2.445763	2.365453	2.14237	1.933457	2.417845	2.01908	0.163693	0.512307
-0.81873	1.263137	1.592487	1.51536	0.907322	1.585605	1.46518	0.00222	0.685165
-0.81873	1.417017	2.095173	1.997863	1.254013	1.87519	1.684713	0.016273	0.84116
-0.4916	1.377983	1.949617	1.942907	1.161345	1.86103	1.575053	0.022125	0.788271
-0.4916	1.759517	2.335113	2.388493	1.46886	2.39357	1.922607	0.04795	0.92471
-0.58605	1.82082	2.356747	2.447787	1.454593	2.41938	2.025127	0.031156	0.993193
-0.68377								
-0.58605	0.845456	1.261747	1.420047	0.779452	0.945993	0.853126	0.010054	0.640595
-0.68377	-0.23408	-0.31012	-0.28981	0.153249	-0.06649	0.2164	0.053096	0.526524
-0.68377	-0.00844	0.765802	1.219333	0.498287	0.345801	0.192458	0.001168	1.227769
0.962257	-0.00377	-0.08946	-0.06817	0.136269	0.224902	0.089611	0.108705	0.314363
-0.03117	0.302926	0.381575	0.304752	0.020794	0.676011	0.595721	0.01507	0.655217
0.962257	0.834919	1.386367	1.645487	1.232367	1.22925	1.027525	0.005212	0.810568
0.962257	0.861619	1.06075	0.84838	0.483492	1.031544	0.862003	0.100503	0.577258
-0.03117	0.592044	0.701978	0.419936	0.608853	1.09317	0.808332	0.022117	0.673234
-0.74753	0.515601	1.396382	1.78495	1.417326	1.256745	0.728047	0.004817	1.269349
-0.94178	-0.22733	-0.36196	-0.33489	-0.52132	-0.36613	-0.58145	0.204461	0.354118
-0.94178	-0.84008	-1.01829	-1.04324	-0.63287	-0.81543	-0.76461	0.039588	0.410371
-0.94178	0.643009	1.137077	1.375657	0.819566	1.23306	1.040452	0.001026	0.732648
-0.74753	1.251237	1.344127	1.550923	1.29025	1.72872	1.451733	0.036643	0.477483
-0.85823	0.786558	1.136853	1.632633	0.878279	1.244865	0.986199	0.016011	0.846076
-0.16985								
-0.85823								
-0.16985	-0.36305	-0.52823	-0.43468	-0.2413	-0.29676	-0.26742	0.016891	0.286929
-0.16985	-0.92165	-1.46491	-1.36797	-1.06975	-1.19275	-0.97464	0.018522	0.543268
-0.16985	-1.23147	-1.72474	-1.82799	-1.32517	-1.54821	-1.27037	0.00958	0.596523
-0.16985	0.294936	-0.41836	-0.54499	-0.1049	0.27107	0.460302	0.000267	1.005296
0.039722	0.302851	0.551256	1.028327	0.504008	0.565147	0.655786	0.007882	0.725476
0.039722	-0.76531	-1.50343	-1.28987	-0.82799	-1.19464	-0.90435	0.032412	0.738121

0.039722	-0.58958	-1.23945	-1.4305	-0.63101	-0.96675	-0.63505	0.002933	0.840921
0.039722	0.599158	0.863791	1.046826	0.709055	0.774144	0.821918	0.02683	0.447668
-0.1472	-0.15028	-0.07404	0.1011	0.05309	-0.03164	0.066354	0.195695	0.251376
-0.1472	0.665989	0.454472	0.034491	0.106137	0.729081	0.715257	0.001393	0.69459
-0.1472	-0.03147	0.199511	0.484496	0.540729	0.246189	0.16868	0.012093	0.572196
-0.21217	3.527877	3.043452	2.667813	2.52102	2.87898	2.131937	0.005586	1.39594
-0.21217	-0.27925	-0.35928	-0.09825	-0.09709	-0.33607	-0.31653	0.075554	0.262192
-0.21217	0.446786	0.262151	0.277425	0.469659	0.245342	0.422779	0.106448	0.224318
-1.01339	2.031907	2.1351	2.219423	1.72289	1.840595	1.448623	0.037887	0.7708
0.322723								
-1.01339	-0.533	-0.99848	-0.78791	-0.45632	-0.43597	-0.43028	0.02093	0.568207
0.322723	0.425277	-0.10255	-0.35524	-0.32581	0.000615	-0.06077	0.010593	0.780513
0.322723	-0.51265	-1.06996	-1.0295	-0.85827	-1.41302	-0.94095	0.041587	0.900375
0.322723	-0.63543	-1.23518	-1.32724	-0.82317	-0.93817	-0.67571	0.006818	0.691813
-0.02738	0.929404	1.574137	1.745247	1.068599	2.008805	1.560437	0.00592	1.079401
0.27325	-0.69691	-0.97022	-0.79775	-0.4684	-0.7853	-0.80644	0.052436	0.501815
0.24052	-0.01638	-0.17237	-0.21007	0.068479	0.479352	0.26338	0.013952	0.689426
-0.02738	-0.25068	-0.33853	-0.50188	-0.44422	0.450811	0.188106	0.008197	0.952692
0.24052	-0.14081	-0.18389	-0.32774	-0.15822	0.450803	0.412059	0.007201	0.778542
0.24052	0.955979	0.398066	-0.27317	0.213442	-0.1867	0.015606	0.139211	1.229152
0.27325	-0.16044	-0.29186	-0.06368	0.098396	0.488726	0.373271	0.00623	0.780589
0.27325	0.654418	1.015368	1.152143	0.98013	1.689965	1.103517	0.00581	1.035547
1.130933								
1.130933	-0.12536	-0.23959	-0.0178	0.150623	0.1303	0.086334	0.024953	0.39021
1.130933	1.261109	1.578957	1.339441	1.418398	1.89475	1.847415	0.1044	0.633641
-1.14505	1.575417	1.75065	1.90478	0.965346	1.891375	1.805133	0.020277	0.939434
-0.88495	-0.12079	-0.2	-0.1637	0.036586	0.591427	0.549211	0.015487	0.79143
-1.14505								
-0.88495	1.194081	1.727437	1.663073	0.533774	1.901095	1.70956	0.005217	1.367321
-0.28864								
-0.28864	0.209929	0.055805	0.176382	0.291706	1.003092	0.786679	0.014973	0.947286
-0.62872								
-0.62872	1.365137	1.459623	1.37521	1.23481	1.196965	1.076951	0.031915	0.382672
-1.06414	-0.2954	0.253888	0.554961	-0.34053	-0.49672	-0.43726	0.023998	1.051681
-1.06414	2.537903	2.626433	2.021087	1.989867	2.4591	2.031953	0.110442	0.636567
-1.06414	0.80572	0.966295	1.08688	0.885414	1.086595	0.98541	0.012723	0.28116
-0.93483								
-0.93483	0.428225	1.059106	1.008712	-0.00063	-0.30386	0.001904	0.004685	1.362963
-0.28775								
-0.28775	-0.80806	-1.55835	-1.6001	-0.66019	-1.38281	-1.16267	0.014644	0.939909
-0.28775	-1.13493	-1.72127	-1.99991	-1.18587	-1.83775	-1.37296	0.021004	0.864974
-0.28775	-0.47557	-0.53867	-0.05705	0.001583	-0.66623	-0.49619	0.012856	0.667808
0.219983								
0.219983	-0.28248	-0.24282	-0.40194	-0.48318	-0.47289	-0.51982	0.063987	0.277005
0.409147								
0.409147	-0.09126	0.073543	0.648344	0.289865	-0.09173	0.098748	0.028921	0.740076
-1.30665								

-1.30665	-0.07357	-0.6467	-0.22614	0.103542	-0.2033	-0.121	0.007006	0.750244
-1.30665	0.036933	0.365959	0.89339	0.476955	0.558307	0.319904	0.062478	0.856457
0.49216								
0.49216	-0.41412	-1.02526	-1.50649	-0.73323	-0.98733	-0.69003	0.026621	1.092371
0.49216	-0.56651	-1.42256	-1.34389	-0.53095	-0.56121	-0.66021	0.016481	0.891613
0.49216	-0.62918	-0.88867	-0.97706	-0.70502	-0.66258	-0.59351	0.037104	0.383551
-0.09171	0.455791	0.465233	0.334312	0.185774	0.909381	0.898011	0.017355	0.723607
-0.09171	-0.5507	-0.82239	-0.94415	-0.65508	-0.72859	-0.60842	0.046488	0.393447
-0.09171	0.499897	0.535842	0.71656	0.471838	0.521644	0.3605	0.097142	0.356061
0.226953								
0.226953	-0.11215	-0.31252	-0.37584	-0.36079	-0.00976	-0.03954	0.006317	0.366077
0.226953	-0.58511	-0.77358	-0.63429	-0.49368	-0.73406	-0.5853	0.242483	0.279895
-0.23783	0.495394	0.882759	1.079346	0.441594	0.415556	0.565231	0.035896	0.66379
-0.23783	0.651463	0.95297	0.843419	0.518552	1.179985	0.698074	0.030865	0.661433
-0.44439								
-0.23783								
-0.12467								
-0.12467	-0.10712	-0.43556	-0.3676	0.07954	-0.29364	-0.28463	0.003291	0.515096
-0.12467	1.961083	2.066003	2.031267	2.012017	2.05016	1.70744	0.080224	0.358563
0.046502	-0.52124	-1.20532	-2.00772	-1.66891	-2.36346	-1.62382	0.049582	1.84222
0.046502	1.053001	1.126078	1.218297	1.546237	2.064845	1.619083	0.001328	1.011844
0.046502	-0.43576	-0.88925	-1.16171	-0.43331	-1.01433	-0.57844	0.014088	0.728395
0.046502	-0.43271	-1.3389	-1.79179	-0.29599	-1.14182	-0.91465	8.06E-05	1.495802
0.046502	-0.57668	-1.13255	-1.53806	-0.9297	-1.34	-1.19384	0.000125	0.961382
0.046502	-0.64147	-1.17729	-1.40082	-1.08929	-1.50048	-1.01747	0.002934	0.859015
-0.49136								
-0.49136	1.093008	1.150763	0.870027	0.755248	1.06246	0.95579	0.006433	0.395515
-0.26997								
-0.26997	-0.26082	-0.75991	-0.98883	-0.42485	-0.90729	-0.61127	0.002454	0.728005
-0.26997	-0.49898	-0.9673	-1.17042	-0.56819	-0.92503	-0.68464	0.001971	0.671438
-0.77996								
	-1.04262	-1.29355	-1.62589	-0.64129	-1.90049	-1.35376	0.004116	1.259199
-0.55766	-0.27154	-0.63056	-1.05885	-0.68916	-0.97715	-0.68425	0.000609	0.787315
-0.55766	-0.82498	-1.4141	-1.49447	-0.89238	-1.56684	-1.31386	0.055877	0.741852
-1.4181								
-1.4181	-0.68196	-0.89542	-1.061	-1.06944	-0.93365	-0.70258	0.070202	0.387485
-1.0384	-0.1674	-0.29068	-0.10039	-0.13552	-0.16485	0.002676	0.086544	0.293352
-1.0384	0.003517	0.425401	-0.0534	0.224854	-0.19733	-0.30836	0.005829	0.733761
-0.69837								
-0.69837	1.043814	0.804349	0.945917	0.808814	1.2905	1.017741	0.01372	0.486151
0.221955								
0.221955	0.107616	0.384664	0.25053	0.388956	0.330108	0.240708	0.147156	0.28134
	-0.11691	0.052026	0.074592	-0.11045	0.348742	0.285565	0.001441	0.465652
	-0.74468	-1.61755	-1.80908	-0.97974	-2.08628	-1.5459	0.04532	1.341599

	-0.95037	-1.55098	-1.79599	-1.01761	-1.43114	-1.0434	0.000956	0.845622	
-0.43337									
-0.43337	2.374683	2.510887	2.498653	2.017703	2.58721	2.389497	0.014674	0.569507	
-1.59841									
-1.59841	0.669375	0.907025	1.63297	1.251773	0.943406	0.749181	0.00042	0.963595	
-1.59841	0.289153	0.433851	1.032955	0.782784	0.413663	-0.10285	0.024713	1.135805	
1.024493									
1.024493	1.811077	1.861753	2.139907	1.674677	1.5635	1.55787	0.035105	0.582037	
1.024493	-0.3586	-0.65641	-0.95695	-0.27654	-0.64967	-0.48345	0.002459	0.680417	
-1.23208									
-1.23208	-0.20814	-0.18279	-0.15308	-0.07691	-0.46541	-0.20183	0.029313	0.388492	
-1.23208	0.371922	0.769492	1.23326	0.685708	0.601577	0.356236	0.000738	0.877024	
-1.70524									
-1.70524	0.513071	0.536454	0.666276	1.17448	0.44938	0.342558	0.026818	0.831922	
-1.70524	0.669992	0.787368	0.633759	0.295549	0.408538	0.458387	0.042768	0.49182	
1.655683									
1.655683	1.877987	3.149717	2.261819	2.304368	1.437369	2.141484	0.115793	1.712348	
1.327057									
1.327057	0.705689	1.018904	1.446863	0.895555	0.570236	0.41176	2.85E-05	1.035103	
-0.741	0.058815	0.272235	0.449963	0.119251	-0.31578	-0.00304	0.002643	0.765747	
-0.741	-0.11032	0.11614	0.608358	0.664377	0.302959	0.164434	0.000597	0.774695	
-0.10968	0.470183	0.690696	0.803692	0.126813	0.767252	0.740556	0.00208	0.676879	
-0.10968	-1.04309	-1.47567	-1.5649	-0.82458	-1.20668	-0.83177	0.011956	0.740324	
-0.10968	0.048115	-0.02158	0.397556	0.456908	0.47867	0.278865	0.030915	0.500248	
1.626813									
1.626813	1.652223	2.15123	2.03946	1.084283	0.832362	1.064546	0.003191	1.318869	
-0.29336									
-0.29336	1.754713	2.49847	2.842272	2.184719	1.442675	2.453172	0.188411	1.399597	
-0.29336	-0.2351	-0.70271	-0.41121	-0.62696	-0.61248	-0.17627	0.209098	0.526447	
-0.22051	1.861677	1.632727	1.482844	1.156555	1.827885	1.39067	0.138678	0.705121	
-0.22051	0.098847	0.010886	-0.33919	0.007907	-0.5392	-0.32601	0.067662	0.638049	
-0.22051	-0.43562	-0.47728	-0.44271	-0.35081	-0.03691	-0.16958	0.009203	0.440376	
0.62343	0.526439	0.845198	1.014976	0.663342	1.310845	1.029054	0.004149	0.784406	
0.62343	1.014205	1.030421	0.897837	0.756991	1.028867	0.862445	0.049618	0.27343	
1.64005	0.404412	0.654362	0.625048	0.213934	0.495693	0.805394	0.022026	0.59146	
1.64005	-0.43936	-0.44584	-0.7512	-0.24749	-0.61049	-0.28062	0.221294	0.503709	
1.64005	-0.91857	-1.05971	-1.63168	-0.9693	-1.103	-0.99091	0.036088	0.713113	
1.64005	-0.6341	-1.19284	-1.22858	-0.64862	-0.81065	-0.6381	0.009249	0.594483	
2.073397	0.299505	0.494976	0.669806	0.507757	0.901667	0.660559	0.026202	0.602162	
2.073397	-1.08854	-1.63389	-1.87408	-1.1018	-1.47323	-1.14592	0.010849	0.785535	
2.073397	1.234733	1.089291	0.397916	0.53553	0.544481	0.622985	0.038154	0.836817	
2.073397	-0.20362	0.06434	0.111154	0.28965	0.463888	0.155286	0.067491	0.667504	
2.073397	1.062267	1.216483	1.46533	1.23309	1.414535	1.34175	0.0162	0.403063	
-0.32952									
2.073397	1.010234	1.049337	1.167293	0.73023	1.49965	1.34407	0.069658	0.76942	
-0.32952	1.043672	1.262047	0.784209	0.759851	1.611145	1.180191	0.037781	0.851294	

0.409853	0.352406	0.544526	0.665066	0.468674	0.825006	0.580342	0.006594	0.472599
0.389063	0.346296	0.290483	0.363229	0.207493	0.583324	0.374986	0.079728	0.37583
0.409853								
0.389063	-1.40107	-1.91804	-1.89786	-1.32377	-1.62171	-1.38467	0.017678	0.594277
0.389063	-0.56966	-0.52856	-0.5317	-0.41978	-0.21347	-0.22496	0.017884	0.356194
-0.24432								
-0.24432	-0.42213	-0.96523	-0.88693	-0.49324	-0.73451	-0.48461	0.002209	0.5431
-0.24432	0.876821	1.306787	1.66763	0.96628	0.601028	0.491775	0.001513	1.175855
-0.24459	-0.52402	-0.58769	-0.58098	-0.56813	-0.17401	-0.32248	0.008777	0.413682
-0.14735	0.809885	1.373587	1.367227	0.716479	0.86934	0.817374	0.022106	0.657108
-0.26838	-1.23864	-1.77253	-1.96759	-1.23804	-1.72286	-1.43932	0.016742	0.729552
-0.43937	0.996418	1.109384	1.299813	1.00857	1.416525	1.209	0.099929	0.420107
-0.24459	0.868217	1.058054	1.152067	0.857682	1.27653	1.085583	0.041151	0.418848
-0.43937	-0.54707	-1.05475	-1.2186	-0.29794	-0.85594	-0.6784	0.022281	0.920664
-0.43937	-0.19818	-0.3664	-0.2824	0.001498	-0.34779	-0.3475	0.000457	0.367899
-0.26838	-0.1526	-0.07921	-0.1106	-0.36431	-0.13151	0.050862	0.004249	0.415176
-0.26838	-0.45989	-0.84928	-1.14712	-0.57666	-0.85751	-0.77283	0.005072	0.687233
1.537807								
1.537807	-0.95107	-1.48242	-1.37961	-0.9337	-1.16922	-0.92913	0.041622	0.553288
1.537807	0.262413	-0.35627	-0.53687	-0.17927	-0.90932	-0.29474	0.057283	1.171728
1.537807	0.294811	0.2692	-0.22631	0.463571	-0.81032	0.133007	0.118656	1.273893
1.537807	-0.23314	-0.03288	0.502601	0.119856	-0.27527	-0.20632	0.006833	0.777871
0.848023								
0.848023	0.849537	1.032842	1.21953	0.966335	1.746385	1.473547	0.004847	0.896848
-0.57842	0.063706	-0.03329	-0.13373	0.183827	0.76433	0.460611	0.011926	0.898063
-0.57842	-0.83998	-1.3108	-1.55635	-0.94218	-1.02268	-0.99323	0.000348	0.716366
-0.57842								
-0.70126	-0.77436	-0.93537	-0.76647	-0.59858	-1.11058	-0.679	0.233237	0.512006
-0.70126	0.488107	0.667895	0.732552	0.37958	0.537127	0.496826	0.010439	0.352972
-0.57293	0.573429	-0.00329	0.60258	0.169576	0.511666	0.292108	0.004551	0.605873
-0.70126	0.771813	1.27624	1.628457	1.091961	0.9863	0.780451	0.006623	0.856644
-0.57293	0.732013	1.256257	1.477963	0.961486	0.98368	0.795172	0.002752	0.745951
-0.57293	0.430468	0.55882	1.241729	1.155857	0.978972	0.744856	0.035138	0.811261
-0.3349								
-0.3349	-0.06705	-0.15569	-0.18374	-0.11997	-0.13276	-0.17556	0.23725	0.11669
-0.3349	3.30048	3.682423	1.898047	2.893957	2.00867	2.49269	0.101557	1.784377
-0.03959	0.463385	0.541949	0.576799	0.645757	0.864709	0.832154	0.023338	0.401323
-0.17031	0.299785	0.218239	0.769409	0.563993	0.873943	0.535626	0.051538	0.655704
-0.17031	0.849946	0.862832	0.597851	0.916046	1.119575	0.929243	0.010713	0.521724
-0.17031	1.024063	1.330367	1.34055	1.270507	1.41939	1.235584	0.023106	0.395327
-0.03959	0.591853	0.605922	0.720355	0.958074	1.28035	0.864697	0.000581	0.688497
-0.01232	1.668867	1.711636	2.04447	1.458793	2.552815	1.928523	0.024439	1.094022
-0.01232	0.152485	0.558013	0.773377	0.820931	1.086985	0.626064	0.002853	0.9345
-0.01232	0.258647	0.189881	0.175553	0.475324	0.563644	0.364197	0.145568	0.388091
-0.17233	0.225995	0.114661	0.089171	0.399713	0.466681	0.316122	0.016398	0.37751
-0.17233	0.733544	0.169679	-0.93963	-0.00255	-0.4455	-0.16407	0.010282	1.673172
-0.29074	0.740522	0.767083	0.969801	0.532391	0.898469	0.551142	0.131937	0.43741

-0.17233								
-0.85611	1.049188	1.52087	1.657903	0.651601	1.261275	1.054468	0.008609	1.006302
-0.29074	0.820864	1.398767	1.235152	0.812179	1.270095	0.828813	0.004214	0.586588
-0.29074	0.33779	0.464479	0.658523	0.608923	0.975937	0.682485	0.017893	0.638147
-0.85611	-0.04356	0.170362	0.158311	0.439999	-0.02337	-0.07646	0.059824	0.516458
-0.85611	-0.51398	-0.18595	-0.17729	-0.17684	-0.38703	-0.66006	0.024726	0.48322
-0.14701	0.653785	1.155477	1.407977	0.872902	1.135209	1.157346	0.00356	0.754192
-0.82995	1.26347	1.69022	1.908917	1.190254	1.680275	1.60066	0.011776	0.718663
-0.82995	0.771957	1.51481	1.854277	1.3402	1.26928	0.878306	0.000524	1.08232
-0.14701	-0.31889	-0.71723	-0.79832	-0.30852	-0.36465	-0.35514	0.003686	0.489807
-0.14701	0.239893	0.568813	0.423686	0.249745	-0.10906	0.136181	0.087788	0.677868
-0.25517	0.197416	0.176407	0.21308	0.305123	0.661586	0.428954	0.063109	0.485179
-0.93865	0.115186	0.121624	0.621297	0.204032	0.259215	0.094957	0.001619	0.52634
-0.25517	0.887364	1.210972	1.53972	0.825738	1.57243	1.364113	0.01503	0.746692
	0.93727	0.568299	0.990916	0.775147	1.431865	1.120492	0.030916	0.863566
0.023547	2.37329	2.274147	1.452027	1.66237	1.41212	1.335606	0.027673	1.037684
0.023547	-0.58735	-1.00795	-1.29561	-0.64894	-1.06188	-0.76616	0.010385	0.708263
0.023547	-1.18009	-1.52315	-1.32629	-0.93552	-1.27848	-1.178	0.008299	0.58763
0.06346	4.264853	3.962087	3.35344	3.278467	3.42832	3.232637	0.023934	1.032217
0.353367	5.16787	5.199917	4.323177	4.35951	4.385625	4.169523	0.013722	1.030393
0.353367	-0.58112	-0.9266	-0.98253	-0.68245	-0.83964	-0.55311	0.007577	0.429424
0.353367	-0.716	-1.28243	-1.37449	-0.9394	-1.11972	-1.00595	0.004266	0.658485
0.353367	-0.58369	-0.81182	-1.18046	-0.74087	-0.73508	-0.60721	0.002764	0.596773
0.06346	1.138239	1.30307	1.60628	1.079623	1.499455	1.4245	0.030389	0.526657
2.074267	1.032566	1.124477	1.326953	0.603294	1.71304	1.504663	0.045762	1.109746
2.074267	-0.35279	-0.6806	-0.74747	-0.2971	-0.40528	-0.2497	0.009733	0.497769
2.074267	0.809941	0.052171	-0.59507	-0.50341	-0.67347	-0.5393	0.142543	1.483411
-0.34554	-0.76218	-0.90636	-0.89963	-0.53637	-0.67668	-0.6189	0.045073	0.369989
-0.28629	0.684817	0.706673	1.152587	0.696723	1.25352	0.856626	0.020631	0.568703
-0.28629	-0.53528	-0.65309	-0.76967	-0.37328	-0.19265	-0.64679	0.024453	0.577021
-0.28629	1.057415	1.759197	2.004397	1.278893	1.385475	1.080773	0.003455	0.946982
-0.34554	0.664084	0.257762	-0.42013	-0.32834	0.318257	-0.0927	0.0782	1.084217
-0.34554	-1.17981	-1.82554	-2.15077	-1.28158	-1.61623	-1.24124	0.006222	0.970964
-0.34554	-0.67661	-1.30064	-1.53657	-0.78562	-1.03121	-0.68431	0.00544	0.859956
-0.34554	-0.73322	-0.66904	-1.3419	-0.53208	-1.16677	-0.64603	0.030559	0.80982
0.529965								
0.529965	-0.33385	-0.27963	-0.20341	-0.28943	-0.55095	-0.51532	0.037762	0.347541
0.529965	-1.54504	-1.90572	-2.07851	-1.29038	-1.67437	-1.37938	0.008823	0.788127
0.529965	-0.41387	-0.64818	-0.11924	-0.31796	-0.52405	-0.44196	0.029649	0.528933
-0.54003	0.625418	0.996782	1.428093	0.3681	0.818172	0.660602	0.001684	1.059993
0.70526	1.144629	1.176203	1.419177	0.8708	1.036136	0.88588	0.071058	0.548376
-0.54003	0.70604	0.956199	1.077986	0.601671	0.697923	0.331768	0.00842	0.746218
0.70526	-0.46318	-0.75802	-0.89322	-0.55748	-0.86782	-0.76316	0.033664	0.430047
0.70526	0.647731	1.140893	1.386677	0.814546	0.817229	0.643562	0.021618	0.743115
2.04928								
2.04928	1.946677	1.842767	1.540023	1.16201	0.733077	1.508237	0.001191	1.2136
1.83189	1.495167	1.989328	1.96358	1.298037	2.120815	1.811483	0.0366	0.822778

1.83189	1.3114	1.546473	1.562073	1.412243	1.38269	1.463187	0.168348	0.250673
0.19156	0.813224	0.598302	1.124681	0.721843	0.819243	0.496315	0.109121	0.628366
0.19156	1.192053	1.006189	0.846381	0.930539	0.717842	1.162297	0.052124	0.474211
2.144408	0.636385	0.817747	1.136041	0.476498	0.870986	0.754899	0.008057	0.659543
2.144408	-0.31667	-0.70056	-0.94324	-0.65286	-0.79211	-0.49763	0.000895	0.626569
2.144408	-0.10313	-0.53246	-0.51046	0.010748	0.171423	0.074615	0.003627	0.703887
2.144408	1.478253	1.62321	1.531227	1.161621	1.30249	1.56827	0.102133	0.461589
1.1355	1.339117	1.409037	1.105207	1.173551	1.402285	0.991728	0.092869	0.417308
1.1355	5.018683	5.018427	4.240757	4.30217	3.764135	4.859803	0.089939	1.254548
	0.22019	-0.19055	-0.32364	-0.28464	0.053497	-0.12284	0.038729	0.543828
	-0.58318	-1.15779	-0.89634	-0.93106	-1.27736	-1.069	0.033537	0.694174
	-0.90616	-1.34231	-1.64687	-0.8908	-1.48305	-1.08571	0.024986	0.756071
1.324277	0.322696	0.42154	0.621944	-0.14235	0.579592	0.387482	0.007679	0.764292
1.324277	0.614869	0.316712	-0.28611	0.140721	0.062748	-0.06702	0.02599	0.900984
1.324277	0.197976	0.029566	-0.1481	-0.0788	-0.08875	-0.33858	0.063706	0.536554
1.142153	0.385769	0.606471	0.676857	0.21583	0.883423	0.595807	0.039778	0.667593
1.142153	0.225315	0.106267	0.045717	0.082174	0.388775	0.259898	0.04888	0.343058
1.142153	-0.32798	-0.44781	-0.55635	-0.41592	-0.34891	-0.26758	0.01703	0.288765
2.247093								
2.247093	-0.79412	-0.95784	-1.59473	-0.82029	-1.11897	-0.93765	0.002785	0.800611
2.247093	-0.88371	-1.5752	-1.85358	-0.8713	-1.41344	-0.9257	0.019256	0.982283
1.397327								
1.397327								
1.397327	-0.55002	-0.53152	-0.83199	-0.54696	-0.58342	-0.6097	0.033351	0.30047
1.7439	0.589031	-0.0886	-0.41726	0.109029	0.366896	0.169413	0.028203	1.006292
1.7439	-0.62692	-0.76551	-0.83711	-0.6041	-0.66809	-0.61984	0.001016	0.233011
0.577177	0.523625	0.605149	0.50334	-0.24601	0.648055	0.771765	0.002876	1.017779
1.7439	-0.18713	-0.73492	-0.92078	-0.43953	0.093231	-0.27554	0.012509	1.014009
0.577177	-0.15142	-0.55729	-0.65423	-0.2355	0.075942	0.036847	0.0197	0.730175
0.577177	0.008267	-0.06761	-0.08033	-0.0412	-0.20393	-0.07034	0.047361	0.212198
1.409463								
1.409463	0.102923	0.020083	-0.14677	0.307438	0.69277	0.450311	0.003349	0.839541
1.409463	-0.76029	-1.04693	-1.45603	-0.89965	-0.49498	-1.04529	0.122986	0.961043
0.829142	0.23428	0.288793	0.707617	0.154019	0.807919	0.681895	0.012981	0.653901
0.829142	0.562124	1.118675	-0.22066	0.024542	0.040413	0.631567	0.169895	1.339331
0.829142	-0.52427	-0.90932	-1.10854	-0.44079	-0.70599	-0.44547	0.055267	0.667749
0.829142	-0.91103	-1.28773	-1.46261	-1.19694	-1.14426	-1.07403	0.009587	0.551586
-0.68273	0.189336	0.380127	0.359483	0.065305	0.482673	0.319275	0.05825	0.417368
-0.49384	1.15192	1.381263	1.80097	1.08929	1.616435	1.281262	1.51E-05	0.71168
-0.49384	0.829136	1.327586	1.516075	0.867526	1.144325	1.010622	0.045698	0.686939
-0.68273	0.714726	0.840564	0.801092	0.2039	0.396199	0.780772	0.007611	0.636663
-0.68273	2.048419	0.713126	1.326221	1.380362	0.038002	0.186511	0.078467	2.010418
-0.68273	0.369503	0.366396	0.460373	0.386439	-0.17423	0.553654	0.062416	0.727881
0.116912	0.979474	1.531756	1.896043	1.016694	2.32687	1.68977	0.00616	1.347396
0.116912	-0.52604	-0.67889	-0.69791	-0.44494	-0.65691	-0.56994	0.071953	0.252966
0.116912	-0.68477	-1.17144	-1.14401	-0.61799	-0.88325	-0.64995	0.120598	0.553458
0.116912	2.51938	2.0583	1.354477	1.657627	1.683395	1.695757	0.004244	1.164903

0.380677	0.900828	1.088883	1.392884	0.425754	1.171065	0.957052	0.036238	0.967129
0.380677	0.141086	0.028021	0.327004	0.393798	0.484065	0.008943	0.205048	0.475122
0.617813								
0.617813	0.12755	-0.07958	0.05161	0.491429	0.519825	0.320621	0.000446	0.599402
0.617813	0.67065	0.707322	0.762849	1.003216	1.22292	0.814237	0.004498	0.55227
-1.00367	0.931928	1.3611	1.764287	0.756766	1.64382	1.48816	0.00836	1.007521
-0.80683								
-1.00367	1.894363	1.687705	1.527001	1.410161	2.222269	1.349936	0.05643	0.872332
-0.80683	0.777163	0.840174	0.773101	0.547774	0.704301	0.609337	0.043623	0.2924
-0.01979	1.053992	0.93126	1.062849	0.49581	1.014475	0.664262	0.000631	0.567039
-0.01979	-0.666	-0.73579	-1.02023	-0.39316	-0.48268	-0.48853	0.007903	0.627071
-0.01979	-0.29914	-0.59446	-1.33049	-0.74972	-1.61164	-0.80853	0.069374	1.312491
-0.01979	-0.8823	-1.30094	-1.69153	-1.02205	-1.39711	-1.11159	0.003563	0.809232
-0.01979	-0.54852	-0.81587	-1.38835	-1.24903	-1.63576	-1.09423	0.039307	1.087245
-0.2248	3.216813	2.703553	2.13231	3.034717	2.83571	2.07322	0.057963	1.143593
-0.2248	-0.2856	-0.38272	-0.41986	-0.0697	0.264127	0.13132	0.021633	0.683991
-0.2248	-1.14331	-2.10895	-1.80488	-1.24734	-1.47744	-1.31903	0.007551	0.965639
-0.2248	-0.715	-0.8879	-0.94836	-0.59397	-0.89857	-0.84024	0.009206	0.35439
-0.2248	-0.78121	-1.43341	-1.63566	-0.77704	-1.21773	-0.87323	0.000586	0.858625
-0.2248	-0.132	-0.1227	-0.33704	-0.09282	0.182407	-0.10381	0.004206	0.519452
-0.31709	0.651387	0.738084	0.673726	0.552101	0.834951	0.899043	0.090691	0.346942
-0.31709	-0.58157	-0.78136	-1.23446	-0.78061	-1.07794	-0.89208	0.006967	0.652895
-0.31709	-0.21529	0.101273	0.536701	0.361636	0.116207	-0.15159	0.001923	0.751986
-0.62254	0.777944	1.38851	1.653463	0.763308	1.108584	0.999986	0.016698	0.890156
-1.00406	0.808475	1.49187	1.61532	0.849185	0.942288	0.749497	0.00762	0.865823
-1.00406	0.497976	1.057706	1.52204	1.245257	1.207215	1.003321	0.002435	1.024064
-0.62254	0.521744	0.932733	1.283137	1.294217	1.4329	1.024626	0.00529	0.911156
0.291063	0.195142	0.208008	0.414071	0.604176	0.857357	0.562131	0.015288	0.662215
0.291063	0.249364	0.450562	-0.12442	-0.11312	0.211161	0.120083	0.000528	0.574981
0.291063	0.57938	0.633254	0.282917	-0.18451	0.885565	0.859139	0.001252	1.070071
1.288113	0.427368	0.393695	0.543923	0.196035	-0.02621	0.482164	0.143008	0.570132
1.288113	-0.97905	-1.78871	-1.83487	-1.10325	-1.4925	-1.37741	0.029884	0.855823
1.288113	0.750564	0.664902	0.131036	0.148557	1.019397	0.819706	0.0242	0.88836
-0.06466								
-0.06466	-0.41918	-0.53075	-0.3354	-0.20829	-0.2103	-0.21038	0.071438	0.322459
-0.06466	0.278794	0.485757	0.313997	0.388782	0.101235	-0.07775	0.123536	0.563504
-0.78748								
-0.78748	-0.42853	-0.3116	-0.28138	-0.30009	-0.5037	-0.43174	0.20675	0.222326
-0.78748	-0.31204	-0.46127	-0.3686	-0.27275	-0.56646	-0.48463	0.081472	0.29371
-0.39342	0.635311	0.502097	0.324794	-0.06156	0.407144	0.543816	0.080658	0.696869
-0.39342	1.534377	2.048743	2.118707	1.46271	1.821765	1.463423	0.017586	0.655997
-0.9194								
-0.9194	2.62746	3.376923	2.52299	2.518793	2.527975	2.651227	0.138532	0.85813
0.50599								
0.50599	-0.60063	-0.74127	-0.66847	-0.35899	-0.93638	-0.16558	0.144129	0.770806
0.50599	-0.1192	-1.54531	-1.85067	-1.24733	-1.44006	-0.73847	0.099672	1.73147
0.50599	-1.22292	-1.82571	-1.76003	-1.18034	-1.49763	-1.27336	0.00697	0.645373

0.50599	-1.11641	-1.76791	-1.99643	-1.10085	-1.3464	-0.94314	0.01684	1.053287
-0.12963	0.420655	0.663776	0.632505	0.49669	0.708052	0.624756	0.030846	0.287397
-0.12963	-0.1558	-0.06936	-0.24627	0.044198	-0.26377	0.098543	0.207835	0.362308
-0.12963								
-0.34989	-0.27693	0.027275	0.427422	0.100155	-0.48107	-0.51656	0.039648	0.943978
-0.34989	0.676363	0.829345	0.733994	0.280279	1.023163	1.013253	0.007201	0.742884
-0.34989	0.528399	0.975039	0.478901	0.276688	1.405848	0.229432	0.235241	1.176416
3.140667	0.105821	0.417017	0.567124	0.031603	0.339715	0.408524	0.011836	0.535521
3.140667	-0.01708	-0.14581	-0.2756	-0.40865	-0.22982	-0.18972	0.043217	0.391566
3.140667	0.832174	1.067524	1.015117	0.310313	0.97207	0.924724	0.004577	0.757212
1.62501	1.207593	1.43766	1.56326	0.953019	1.457275	1.439413	0.002293	0.610241
1.62501	-0.0326	-0.13422	-0.03084	0.193674	0.442187	0.223489	0.009618	0.576406
0.731283								
0.731283	0.529751	1.050122	1.396887	0.666384	0.68863	0.578537	0.004561	0.867136
0.731283	1.318297	1.798923	2.069777	1.35976	1.324105	1.17222	0.000151	0.897557
-0.45421								
-0.45421	0.648749	0.980752	1.157106	0.786393	0.437879	0.444252	0.012805	0.719227
-0.75584								
-0.75584	-0.67463	-1.46117	-1.56417	-1.43373	-1.44313	-1.19347	0.108243	0.889538
-0.75584								
0.241518	0.02569	0.625726	0.754461	0.291867	0.718308	0.446441	0.040976	0.728771
0.241518	0.503868	0.773635	0.935696	0.69284	0.913697	0.811682	0.033954	0.431829
0.183935	1.107432	1.417425	0.927032	0.82012	1.255025	1.093994	0.177495	0.597305
0.183935	-0.02824	0.094203	0.049438	0.231074	0.412572	0.342037	0.015076	0.440814
-0.12436	0.342498	0.339674	0.107644	-0.14988	-0.04151	-0.03575	0.064947	0.49238
-0.12436	-0.78	-0.63293	-0.52051	-0.48937	-0.502	-0.48031	0.071225	0.299687
0.930897	0.76897	0.942272	0.851185	0.577962	0.955281	1.037461	0.067258	0.4595
-0.12436	0.610777	0.85356	0.904424	0.307772	0.696784	1.022208	0.040017	0.714435
0.930897	0.556894	0.84379	1.043644	0.543915	1.11673	0.839407	0.004418	0.572815
0.930897	-0.40805	-0.55659	-0.67874	-0.53058	-0.60645	-0.41624	0.025608	0.270687
1.35009								
1.35009	1.206337	1.721877	1.385483	1.153788	1.745225	1.30812	0.149384	0.591437
0.253003	1.39747	1.602453	1.688393	1.070362	1.79508	1.720463	0.028596	0.724718
0.253003	0.269057	0.802513	-0.34388	-0.26814	-0.439	-0.38294	0.066779	1.24151
0.253003	-0.393	-0.31257	-0.24398	-0.45752	0.01131	-0.16372	0.183407	0.468826
-0.91526	0.11785	0.401769	0.698434	0.210801	0.606384	0.383728	0.037845	0.580583
-0.91526	-0.5516	-0.84631	-1.09114	-0.67519	-0.81794	-0.76045	0.002671	0.539538
-0.91526	1.265887	1.221631	1.30065	1.770613	1.16619	1.118939	0.106459	0.651674
0.52504	0.159344	0.20158	0.107075	0.35256	0.652706	0.397989	0.046993	0.54563
0.38022	-0.69734	-0.92547	-0.94834	-0.54377	-0.50251	-0.45131	0.027258	0.497023
0.38022	0.429875	0.631153	0.362562	0.435906	0.95177	0.656348	0.004542	0.589207
0.38022								
0.52504	0.679137	0.538191	0.593752	0.414687	0.588083	0.442314	0.065649	0.264449
0.05638	1.235746	1.78093	1.973923	1.471801	2.28607	1.66665	0.029596	1.050324
0.05638	-0.75622	-0.87578	-1.4374	-0.64672	-0.85941	-0.86392	0.036221	0.790686
0.05638	-0.82803	-1.10452	-1.39824	-1.00924	-1.38464	-1.08121	0.006619	0.570209

0.05638	-0.0013	-0.11412	0.140485	0.1521	0.261603	0.147817	0.013421	0.375722
-0.51765								
-0.51765	0.526119	1.084802	1.300837	0.649773	0.861214	0.734424	0.01093	0.774718
-0.6878								
-0.6878	0.826914	0.594175	0.459117	0.532851	0.987374	1.098535	0.016847	0.639418
-0.6878	0.00017	0.154189	0.170618	0.421822	-0.17297	-0.00841	0.109938	0.594795
-0.43211								
-0.43211	0.36569	0.816272	1.016245	0.658387	0.583575	0.31616	0.064342	0.700085
-0.43211	-0.91726	-1.33963	-1.72715	-1.20871	-1.38738	-1.17308	0.005863	0.809889
-0.43211	-1.85334	-2.08317	-2.6403	-2.08833	-2.66192	-2.15218	0.010599	0.808574
-0.43211	-1.16688	-1.47465	-1.71406	-1.44669	-1.68937	-1.35796	0.006422	0.54718
-0.43211								
-0.43211	-0.63244	-1.63278	-1.45368	-1.1177	-1.3769	-1.03571	0.009177	1.00034
-0.43211	-0.96712	-1.22416	-1.34374	-0.95727	-1.2538	-1.00534	0.050057	0.386464
-0.24774								
-0.24774	-0.63656	-0.75125	-0.67182	-0.54261	-0.50551	-0.45864	0.00871	0.292614
-0.24774	0.283516	0.271838	-0.41355	0.18784	-1.13353	-0.08735	0.089256	1.417041
-0.24774	0.138972	-1.32141	-1.98996	-0.83737	-1.63271	-1.01279	0.001497	2.128932
-0.24774	-1.12336	-1.50466	-1.6334	-1.14843	-1.35721	-1.18736	0.022495	0.510037
-0.24774	-0.36587	-1.02114	-1.47481	-0.99703	-1.16331	-1.18861	0.000319	1.10894
-0.24774	-0.79306	-1.07376	-0.90504	-0.86356	-0.85911	-0.69856	0.197739	0.3752
2.937803	0.258859	0.003895	-0.05934	0.182318	0.074828	0.10888	0.002489	0.318203
-0.81063	2.367253	2.760733	1.987757	1.79131	2.10643	1.494093	0.078477	1.26664
-0.81063	-0.07158	0.45853	1.152057	0.509256	0.408792	0.260543	0.015118	1.223632
-0.81063	1.597787	1.433613	1.570727	1.326333	1.297775	0.928527	0.054259	0.66926
-0.35048	4.47052	3.693289	2.958767	3.265723	2.9452	2.45497	0.013946	2.01555
-0.35048	1.378705	-0.27956	-0.83187	-0.24322	-0.30868	0.087494	0.111924	2.210574
-0.35048	-0.27294	-0.34239	-0.57786	-0.30564	-0.26302	-0.19315	0.038845	0.384704
1.230887	-0.21807	0.313912	0.48934	-0.08665	0.172777	0.53694	0.001924	0.755013
1.230887	-0.51766	-0.37625	-0.34601	0.041365	-0.05888	-0.16797	0.006799	0.559025
1.230887	0.30447	0.389373	0.55503	0.47111	0.904791	0.664386	0.000954	0.600321
0.38653	-0.22475	0.277452	0.360118	0.499719	0.735448	0.523345	0.001666	0.960195
0.38653	0.556699	0.540769	0.454879	0.671126	0.912345	0.790299	0.006859	0.457466
-0.98224	0.422227	0.262426	0.702427	0.367507	0.52612	0.165744	0.006776	0.536683
-0.98224	-0.09837	-0.14411	-0.5247	-0.25917	-0.31647	-0.15469	0.006632	0.426336
-0.35022	-0.06274	0.222192	-0.05792	-0.11793	0.346286	0.006478	0.090071	0.464216
-0.25502	0.57916	0.825902	0.837249	0.910391	1.382755	1.08072	0.014409	0.803595
-0.35022	0.22827	0.735792	1.180689	0.515528	1.309765	0.773326	0.005428	1.081495
-0.25502	4.362843	3.88916	2.982733	3.08676	2.82314	2.98393	0.044043	1.539703
-0.67806								
-0.67806	0.570738	0.704576	0.083563	0.185406	-0.2097	0.220937	0.210446	0.914275
-0.67806	0.390267	1.097351	1.417647	0.560199	0.841828	0.589362	0.000238	1.027379
-0.12528	0.214476	0.717304	1.201737	0.574283	0.925672	0.738496	0.001247	0.987261
-0.12528	-0.62167	-0.78989	-1.00394	-0.81279	-0.65786	-0.63451	0.021316	0.382268
-0.12528	-1.51209	-1.94418	-2.12072	-1.59168	-2.13558	-1.76476	0.024149	0.623489
-0.12528	-0.96564	-1.41795	-1.42942	-1.0474	-1.26155	-1.00713	0.016662	0.463781
-0.12528	-0.10044	-0.14506	-0.68916	-0.34879	-0.352	-0.34681	0.001413	0.588721

-0.45305	-0.07107	0.380719	0.518797	-0.22294	-0.25559	-0.02315	0.020805	0.774388	
-1.23673									
-1.23673	0.310776	0.745248	0.891241	0.03545	0.357273	0.324323	0.005304	0.855791	
-0.45305	-0.39409	-0.57243	-0.4687	-0.19219	0.410641	0.126222	0.002932	0.983074	
-0.45305	-0.20632	-0.13481	-0.10353	-0.0321	0.569283	0.013907	0.016486	0.775605	
-0.48468	-0.41922	-0.48171	-0.6101	0.08693	-0.80156	-0.37246	0.133996	0.888492	
-0.48468	0.1416	0.385824	0.610575	0.351124	0.080806	0.016311	0.028445	0.594263	
-1.004	-0.39174	-0.48126	-0.49668	-0.46899	-0.35225	-0.08993	0.142912	0.406755	
-0.48468	-0.30186	-0.28714	-0.346	-0.43692	-0.15653	0.015279	0.159918	0.452197	
-1.004	0.605018	-0.08188	-0.43937	0.488191	-0.07681	0.210112	0.003805	1.044392	
-1.004	-0.17796	0.17979	0.652934	0.430742	-0.2509	-0.32346	0.00989	0.976392	
-0.0083	0.588695	0.766443	1.386147	0.520228	0.906205	0.61409	0.022772	0.865919	
-0.0083	-0.40672	-0.6451	-0.84797	-0.7165	-0.60994	-0.63068	0.040016	0.441249	
-0.0083	-0.93604	-1.79456	-1.83763	-1.07641	-1.32215	-0.83445	0.060575	1.003179	
-0.0083	-0.51979	-0.69271	-0.34436	-0.51678	-0.55562	-0.61677	0.102366	0.348343	
-0.059	0.04549	-0.00266	0.031627	-0.20418	0.077143	0.113465	0.07227	0.317642	
-0.059	0.515802	0.49344	0.452987	0.49793	0.629397	0.551404	0.193968	0.17641	
0.857258	0.47731	0.791965	0.9825	0.17042	0.584967	0.702227	0.037552	0.81208	
0.857258	0.944734	1.446403	0.952116	1.734532	0.579458	1.52337	0.145337	1.155074	
0.857258	-0.96983	-1.48372	-1.73518	-0.90793	-1.35174	-1.08696	0.005677	0.827257	
0.857258	-0.75452	-1.24551	-1.68981	-0.94136	-1.2303	-1.09411	0.011195	0.935298	
0.857258	0.867009	0.689586	0.118749	0.472927	0.40349	0.304582	0.064215	0.74826	
-0.87923									
-0.44226	0.008794	-0.13749	-0.05167	-0.08366	0.537234	0.341384	0.058076	0.674724	
-0.44226	1.315271	1.83732	1.991387	1.343833	1.4098	1.18642	0.013388	0.804967	
-0.44226									
-0.87923	-0.29905	0.10396	0.322214	0.101538	0.020419	-0.13118	0.034713	0.621262	
3.034377	0.0902	0.409471	0.210477	-0.07064	0.644734	0.677558	0.004201	0.7482	
3.034377	-0.44341	-0.25992	-0.93099	-0.35801	-0.24703	-0.48777	0.200939	0.683963	
3.034377	-0.43374	-1.1188	-1.42758	-0.59215	-0.44191	-0.69952	0.020671	0.993837	
3.034377	-0.33408	-0.72686	-1.04692	-0.83139	-0.59364	-0.51996	0.002925	0.712848	
1.557603	0.852035	1.131631	1.449984	0.953085	1.346339	1.262223	0.024665	0.597948	
1.557603	-0.19123	-0.19049	-0.39381	-0.01507	-0.02416	-0.07792	0.052413	0.378741	
0.495278									
0.495278	-0.72758	-1.05394	-1.37276	-0.77153	-1.21069	-0.69218	0.01595	0.680583	
0.495278									
0.495278	-0.26651	-0.18133	-0.19766	-0.2903	-0.13918	-0.3472	0.050018	0.208019	
2.603787	1.916503	1.928553	1.976673	1.176131	1.64753	1.41689	0.040601	0.800542	
2.603787	0.428417	0.662979	0.374452	0.397738	1.041505	0.602734	0.013772	0.667053	
2.603787	0.234775	0.309858	0.290454	0.398008	0.659894	0.610347	0.00714	0.425119	
0.039033	-0.13812	0.140762	0.370585	0.184197	0.678591	0.369812	0.045848	0.816708	
0.039033	-1.04304	-1.45665	-0.6744	-0.48373	-1.32423	-0.69712	0.129845	0.972927	
0.039033	0.426618	-0.21905	-0.44555	0.046994	-0.09983	-0.14227	0.062406	0.872165	
0.155163									
0.155163	-0.0303	0.294245	0.508405	0.11452	0.113326	0.113237	0.003185	0.538705	
0.051917	0.200451	0.419428	0.803145	0.244756	0.714173	0.57581	0.029164	0.602694	
0.051917	0.431807	0.913543	1.378967	1.052199	1.018339	0.817389	0.000742	0.947159	

0.47157									
0.47157	0.310907	-0.09487	-0.30588	0.051592	-0.13088	-0.41494	0.019479	0.72585	
0.47157	0.280451	0.178218	0.062125	-0.12136	-0.5574	-0.3226	0.015619	0.837848	
0.095897									
0.095897	0.683353	0.499188	-0.05454	0.164085	-0.5253	0.38971	0.063667	1.208651	
0.095897	-0.75165	-1.19967	-1.18459	-0.73235	-1.08735	-0.89886	0.007311	0.467325	
-0.09239	-0.32368	-0.01895	0.454172	-0.15134	0.287961	0.293521	0.007147	0.777847	
-0.09239	-0.46577	-1.00088	-1.31901	-0.70377	-0.97189	-0.76255	0.00324	0.853239	
-0.09239	-0.11466	-0.11735	0.02136	-0.06955	0.103761	0.028927	0.063112	0.221112	
-0.81388	0.531379	0.705109	0.788585	0.431524	0.88465	0.665184	0.15063	0.453126	
-0.81388	1.048579	0.542011	0.877813	0.351854	0.248085	0.079661	0.037558	0.968918	
-0.83108									
-0.83108	-0.46835	-0.87059	-1.14029	-0.64775	-0.9995	-0.82898	0.034208	0.67194	
-0.83108	-0.94548	-1.50965	-1.23121	-0.93524	-1.48206	-0.91278	0.092627	0.596866	
-0.83108	0.392191	0.641605	0.904851	0.668378	0.188312	-0.00116	0.005577	0.906015	
0.043203	0.691492	1.087663	1.083309	0.497846	1.504665	1.39176	0.057854	1.006819	
0.043203	-0.39849	-0.72106	-1.20884	-0.73029	-0.81397	-0.67449	0.007351	0.810356	
0.043203	0.12513	-0.32184	-0.46353	-0.18586	-0.06918	-0.0324	0.076801	0.588663	
0.74406									
0.74406	0.962789	1.174641	1.047306	0.844687	1.736835	1.178017	0.011885	0.892148	
0.63014	0.271102	0.28851	0.431387	0.482704	0.655015	0.521572	0.051308	0.383913	
0.63014	-0.78405	-1.41474	-1.6029	-0.9364	-1.43389	-1.0864	0.003461	0.81885	
0.63014	-0.96625	-1.45812	-1.81106	-1.19288	-1.51671	-1.22395	0.037805	0.844809	
0.63014	0.035058	-0.0781	0.203297	0.215113	0.691126	0.310771	0.009031	0.769222	
-0.85878	-1.28976	-1.62321	-1.64319	-1.36136	-1.50203	-1.32343	0.168891	0.353427	
-0.85878	-0.51072	-0.47925	-0.61088	-0.65499	-0.52055	-0.48426	0.054029	0.175736	
[truncated: 502,075 more chars]
